# Prognostic value of neuro-related genes in colorectal cancer and their potential implications for immunotherapy

**DOI:** 10.7150/jca.136690

**Published:** 2026-09-18

**Authors:** Huantong Wu, Weihua Liu, Shengtao Zhu

**Affiliations:** 1 Department of Gastroenterology, Beijing Friendship Hospital, Capital Medical University, State Key Laboratory of Digestive Health, National Clinical Research Center for Digestive Disease, Beijing Key Laboratory of Early Gastrointestinal Cancer Medicine and Medical Devices, Beijing, China.; 2 Center for Cancer Immunotherapy, Institute of Biomedicine and Biotechnology, Chinese Academy of Sciences, Shenzhen.

**Keywords:** colorectal cancer, neural-related genes (NRGs), prognostic signature, single-cell RNA sequencing (scRNA-seq), tumor microenvironment (TME)

## Abstract

Colorectal cancer (CRC) remains a leading cause of cancer mortality. This study investigates the prognostic and immunological role of neural-related genes (NRGs) in CRC. Using The Cancer Genome Atlas (TCGA) data, a four-gene signature (POU4F1, KLC3, ATP2A1, CALB2) was constructed via Cox and least absolute shrinkage and selection operator (LASSO) regression. This signature independently predicted patient survival. High-risk patients showed enrichment in cytokine signaling and oxidative phosphorylation. Importantly, CALB2 expression correlated with increased infiltration of NKT and central memory CD4+ T cells, while high-risk scores were associated with greater potential for immune evasion. Single-cell analysis revealed epithelial cells as the primary source of these NRGs. This NRG-based signature provides a novel biomarker for prognosis and immunotherapy optimization in CRC.

## 1. Introduction

Colorectal cancer (CRC) is the third most commonly diagnosed cancer and the second leading cause of cancer-related mortality worldwide 1. Most CRC cases evolve from non-malignant adenomas over 10-15 years through a process driven by oncogenic mutations, aberrant signaling pathways, and tumor microenvironment remodeling 2, 3. Epidemiologically, CRC incidence varies by demographics and genetics, with a lifetime risk of 4%-5% 1, 4. GLOBOCAN 2022 estimates exceed 1.9 million new cases and 904,000 deaths annually 5. While CRC has been historically concentrated in Western nations, its incidence is now rapidly rising in developing countries and among younger populations 6. Despite advancements in surgery, chemotherapy, and targeted therapies, the 5-year survival rate remains suboptimal at 64% 7, 8. Limitations in early diagnosis, combined with distant metastasis and therapeutic resistance, continue to drive poor clinical outcomes and heavy economic burdens 8, 9. Therefore, elucidating the molecular pathogenesis of CRC is essential to identify novel therapeutic targets and develop strategies to halt disease progression.

Emerging evidence suggests that the nervous system, beyond its traditional role in orchestrating diverse systemic activities and information transmission, may represent an underexplored dimension in cancer biology. The nervous system fundamentally orchestrates diverse systemic activities and information transmission to facilitate adaptive responses to environmental fluctuations, thereby maintaining physiological homeostasis 10. Recent evidence suggests that the intricate interplay between the nervous system and cancer represents a potential novel hallmark of malignancy, modulating key processes such as tumorigenesis, growth, invasion, and therapeutic resistance 11. Mechanistically, neural-derived signals can stimulate pro-tumorigenic inflammation and impair anti-cancer immunity. Specifically, neurosecretory neurotrophic factors, including nerve growth factor (NGF) and brain-derived neurotrophic factor (BDNF), have been shown to directly facilitate tumor cell proliferation and survival 12, underscoring the pivotal role of neural-related genes (NRGs) in oncogenic progression. These molecular insights are paralleled by clinical observations of neural involvement in cancer. Clinically, perineural invasion (PNI) is recognized as a critical histopathological feature across multiple malignancies, including pancreatic ductal adenocarcinoma (PDAC), head and neck squamous cell carcinoma (HNSCC), and CRC, where it consistently correlates with poor prognosis and diminished survival 13, 14. Molecular studies reveal that the transcription factor OCT1 promotes tumor progression in androgen receptor-negative prostate cancer by targeting the neural gene PFN2 15. In HNSCC, the loss of TP53 triggers the transdifferentiation of tumor-infiltrating sensory neurons into an adrenergic phenotype, which subsequently accelerates tumor expansion 16. Similarly, the neuropeptide Substance P (SP) promotes aggressive phenotypes in breast cancer 17, while NGF signaling, in conjunction with exosomal miR-21-5p, has been identified as a targetable axis in CRC 18.

Despite these advancements and sporadic findings in CRC, comprehensive studies specifically elucidating the systematic role of NRGs in CRC pathogenesis remain remarkably sparse. Identifying and validating NRGs within the CRC landscape is therefore essential for deciphering the molecular mechanisms governing disease progression. These research initiatives are essential for identifying new prognostic biomarkers and therapeutic targets, thereby enabling more effective therapies and better clinical outcomes in CRC patients.

To address this knowledge gap and advance the identification of NRG-based prognostic markers, in the present study, we integrated bulk transcriptomic data with single-cell RNA sequencing (scRNA-seq) to comprehensively characterize the expression profiles of neural-related genes (NRGs) across diverse cellular clusters in CRC. By mapping the NRG landscape at single-cell resolution, we aimed to elucidate the specific molecular mechanisms through which neural signaling modulates the tumor microenvironment. This study aims to elucidate the contribution of neural components to CRC progression, thereby establishing a solid scientific basis for the discovery of new prognostic biomarkers and therapeutic targets. Ultimately, this research aims to offer critical insights for refining individualized treatment strategies and developing targeted interventions to improve clinical outcomes for CRC patients.

## 2. Materials and Methods

### 2.1 Data collection

For the training cohort, the TCGA-COADREAD dataset (a combination of TCGA-COAD and TCGA-READ datasets) 19 was used. This dataset, obtained from The Cancer Genome Atlas (TCGA) (http://cancergenome.nih.gov/) (access date: August 5, 2025), contains gene expression profiles, somatic mutations, and survival data from 618 CRC patients and 51 normal tissue samples. Survival information was available for 524 of these CRC tumor tissue samples. Prognostic analysis was conducted after randomly dividing these 524 samples into training (n=367) and validation (n=157) sets at a 7:3 ratio. Secondly, the gene expression data of the CRC-related dataset GSE231559 were retrieved from the Gene Expression Omnibus (GEO) (http://www.ncbi.nlm.nih.gov/geo/). The single-cell dataset GSE231559 (platform: GPL20301) included 6 CRC and 3 normal tissue samples 20. Furthermore, 1,889 NRGs were sourced from prior research 11 **(Supplementary Table 1)**.

### 2.2 Differential expression analysis

Differential expression analysis was performed on the TCGA-COADREAD dataset using DESeq2 (v 1.40.2) 21, with cutoffs of |log₂FC| > 1 and adjusted p < 0.05. The top 10 DEGs with the highest |log₂FC| values were visualized via a volcano plot and an expression heatmap.

### 2.3 Enrichment analysis and construction of the protein-protein interaction (PPI) network

The ggvenn package (v 0.1.10) 22 was employed to identify overlaps between DEGs and NRGs (candidate genes). Functional annotation of these candidate genes was performed via Gene Ontology (GO) enrichment analysis—covering biological process (BP), cellular component (CC), molecular function and (MF)—and Kyoto Encyclopedia of Genes and Genomes (KEGG) pathway analysis using the clusterProfiler package (v 4.10.1) 23 (p < 0.05). Subsequently, candidate genes were submitted to the STRING database (https://string-db.org/) for constructing a PPI network (confidence > 0.99).

### 2.4 Confirmation of prognostic genes

Prognostic relevance of candidate genes was evaluated in the training set (n = 367) via univariate Cox regression analysis using the survival package (v 3.8-3) 24(criteria: p < 0.05, HR ≠ 1). After a PH assumption test (p > 0.05), qualifying genes were recorded as candidate prognostic genes. Least Absolute Shrinkage and Selection Operator (LASSO) regression was then applied using the glmnet package (v 4.1-8) 25. The lambda.min value was determined by five-fold cross-validation, and genes with non-zero regression coefficients at this lambda were selected as prognostic genes.

### 2.5 Establishment of the prognostic model

A risk model was constructed by calculating a risk score for each training-set CRC patient based on LASSO coefficients and expression levels of prognostic genes. The calculation formula was: 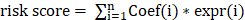. The survminer package (v 0.5.0) 26, identified the optimal cutoff to classify patients into HRG and LRG. Risk-survival status plots were generated, and group survival differences were assessed using KM curves (p < 0.05).

Receiver operating characteristic (ROC) curves from the survivalROC package (v 1.0.3.1) 27 were employed to assess the model's predictive performance. AUC values at 1, 2, and 3 years were derived, with AUC > 0.6 representing acceptable predictive accuracy.

Additionally, to verify the reliability of the prognostic model, the same method described above was applied for validation in CRC tumor tissue samples from the 30% validation set and the TCGA-COADREAD dataset, respectively. When the validation results were basically consistent with those of the training set, this consistency indicated that the risk model had good validity.

### 2.6 Clinical characteristics and independent prognostic analysis

Using the 70% training set, the Wilcoxon test (p < 0.05) assessed risk score differences across clinical subgroups (age, gender, tumor_stage, pathologic_T, pathologic_M), investigating how clinical characteristics correlate with risk scores.

In the training set (n = 367), prognostic factors such as risk score, age, gender, clinical stage, pathologic_T, and M stage were evaluated. Univariate Cox and PH tests (p < 0.05, HR ≠ 1; p > 0.05) selected prognosis-related variables. Subsequent multivariate Cox regression (survival package v 3.8-3; p < 0.05, HR ≠ 1) and a repeated PH test (p > 0.05) established independent prognostic factors, supporting the model's clinical relevance.

### 2.7 Construction and validation of a nomogram model

To facilitate clinical application, a nomogram was built with the regplot package (v 1.1) 28 based on the independent prognostic factors derived from the model. Applied to the 367 training-set samples, the nomogram assigned individual scores to each factor; their sum gave a total score, from which 1-, 2-, and 3-year OS probabilities were derived. Higher total scores indicated increased mortality risk (lower OS probability). Additionally, to assess predictive consistency, a calibration curve was created with the calibrate function in the rms package (v 6.8-1) 29 offering a visual comparison between predicted and actual outcomes and illustrating the nomogram's reliability. At the same time, the survivalROC package (v 1.0.3.1) was used to generate ROC curves for evaluating the nomogram. AUCs greater than 0.7 at 1, 2, and 3 years reflected favorable predictive performance of the nomogram model.

### 2.8 Gene Set Enrichment Analysis (GSEA)

Differential expression analysis between the HRG and LRG was performed using the DESeq2 package (v 1.40.2) on the 367-sample training set to investigate underlying biological pathways. Subsequently, the obtained log_2_FC values were sorted in descending order to generate a table of genes related to HRG and LRG. Using c2.cp.kegg_legacy.v2025.1.Hs.symbols from MSigDB (https://www.gsea-msigdb.org/gsea/msigdb/) as the background gene set, GSEA was conducted with the clusterProfiler package (v 4.10.1) (|NES| > 1, p < 0.05).

### 2.9 Immune infiltration and immune checkpoint analyses

Using ssGSEA (GSVA v 1.46.0) 30, infiltration scores for 28 immune cell types 31 were computed in 367 CRC samples to contrast HRG and LRG. Significant differences were tested with Wilcoxon (p < 0.05) and displayed in boxplots. Spearman correlations (psych package v 2.2.9) 32 were then calculated among differential immune cells, among prognostic genes, and between genes and immune cells, with thresholds of |cor| > 0.3 and p < 0.05. In parallel, differential expression of 38 immune checkpoints 33 was assessed between groups using the Wilcoxon test (p < 0.05).

### 2.10 Immunotherapy analysis

Tumor Immune Dysfunction and Exclusion (TIDE) is a computational tool designed to predict immunotherapy response and evaluate the capacity of tumor immune evasion. Using the 367-sample training set, expression profiles were converted to transcripts per million (TPM) format and submitted to the TIDE database (http://tide.dfci.harvard.edu/) under Homo sapiens to obtain TIDE scores, where higher values correspond to poorer predicted immunotherapy response. Using the psych package (v 2.2.9), Spearman correlation (|cor| > 0.3, p < 0.05) assessed the link between TIDE and risk scores, and Wilcoxon testing (p < 0.05) evaluated TIDE score differences between HRG and LRG.

### 2.11 Tumor variation analysis

Somatic mutation data of the training set were analyzed with the maftools package (v 2.18.0) 34 to compare mutation profiles between the HRG and LRG. Waterfall plots depicted somatic mutation patterns in tumors from the HRG and LRG, showcasing the 20 genes with the greatest mutation frequencies in each cohort.

### 2.12 Prediction of chemotherapeutic drug sensitivity

The pRRophetic package (v 0.5) 35 was employed to compute IC₅₀ values of COADREAD drugs (GDSC database: https://www.cancerrxgene.org/) in the 367-sample training set. Wilcoxon testing (p < 0.05) compared IC₅₀ levels between HRG and LRG.

### 2.13 scRNA-seq analysis

The Seurat package (v 5.3.0) 36 was employed to analyze scRNA-seq data from the GSE231559 dataset. Quality control (QC) was conducted with PercentageFeatureSet, retaining cells expressing 200-3000 genes, genes present in ≥3 cells, cells with total expression < 10,000, and cells exhibiting < 20% mitochondrial gene content. After filtering, NormalizeData was used for normalization, FindVariableFeatures identified the top 2000 HVGs, and ScaleData performed data scaling. Subsequently, PCA dimensionality reduction was performed on the HVGs using the runPCA function. Additionally, permutation testing was performed on the scaled HVGs using the JackStraw function, and p-values were calculated (p < 0.05). To determine the appropriate number of principal components (PCs), the ElbowPlot function was used to create a scree plot.

Following PCA reduction in the GSE231559 dataset, JackStraw and ScoreJackStraw functions were used to define the optimal cluster number. Cells were then clustered with FindNeighbors and FindClusters based on chosen PCs (resolution = 0.1) and visualized using t-SNE (RunTSNE). The FindAllMarkers function pinpointed cluster-specific marker genes, which were compared with published markers 20. to guide cell-type annotation via the SingleR package (v 2.2.0) 37.

Using the Wilcoxon test (p < 0.05), prognostic gene expression was compared between CRC and normal samples for each annotated cell type in the GSE231559 dataset. The cell type showing the largest number of prognostic genes with significant expression differences were selected as key cells for further analyses.

### 2.14 Functional enrichment analysis

Functional enrichment analysis on each cell type in the GSE231559 dataset was conducted using the analyze_sc_clusters function (ReactomeGSA package v 1.14.0) 38 to define their biological pathways and roles in CRC. The top 10 pathways exhibiting the greatest differences were visualized.

### 2.15 Metabolic pathway analysis

To investigate the metabolic activity of cells, the VISION algorithm implemented in the scMetabolism package (v 0.2.1) 38, which covers 85 KEGG metabolic pathways, was utilized to quantify metabolic activity at single-cell resolution. scMetabolism was further applied to evaluate the metabolic activity of distinct cell types, and the results were visualized using the DotPlot.metabolism function.

### 2.16 Cellular communication and pseudo-time analyses

To analyze the intercellular interactions and communication patterns among distinct cell types, separate analyses were performed on CRC and normal samples from the GSE231559 dataset. First, the CellChat package (v 1.6.1) 39 was used to characterize the intercellular communication networks across all annotated cell types, followed by a further analysis of the communication networks between key cells and other cell subsets, and the construction of cell-cell communication profiles. Subsequently, the CellChat package (v 1.6.1) was applied to detect ligand-receptor pairs involved in cell-cell communication, inferring possible interactions between key cells and other cell populations. Bubble plots were generated to display the results.

To obtain the differentiation trajectories of cell subsets within the key cells, in this study, the Seurat package (v 5.3.0) was used to perform secondary clustering on the key cells (resolution = 0.1). The same dimensionality reduction and clustering methods as those used in the primary clustering were adopted. Additionally, pseudotime analysis of key cells was conducted using the Monocle3 package (v 2.26.0) 40 Single-cell trajectories were plotted to visualize developmental progression, with parallel assessment of prognostic gene expression across distinct states.

To obtain the differentiation trajectories of cell subsets within the key cells, in this study, the Seurat package (v 5.3.0) was used to perform secondary clustering on the key cells (resolution = 0.1). The same dimensionality reduction and clustering methods as those used in the primary clustering were adopted. Additionally, the Monocle3 package (v 2.26.0) 40 was applied to conduct pseudotime analysis of key cells, generating single-cell trajectory plots to visualize developmental progression and simultaneously track prognostic gene expression patterns across distinct states.

### 2.17 Statistical analysis

R (v 4.2.1) was used for all bioinformatics analyses. The Wilcoxon test was applied to evaluate differences between groups, with statistical significance defined as p < 0.05.

## 3. Results

### 3.1 Identification of DEGs

Comparative analysis of CRC and control samples in the training set revealed 5,361 DEGs (|log₂FC| > 1, adjusted p < 0.05), with 2,656 genes upregulated and 2,705 genes downregulated in CRC** (Figures 1A**-**B) (Supplementary Table 2)**.

### 3.2 Acquisition of candidate genes

Next, From the intersection of 5,361 DEGs and 1,889 NRGs, 674 candidate genes were identified (**Figure 2A; Supplementary Table 3**). Among them, 31 genes showed enrichment across 2,682 GO signaling pathways (p < 0.05) according to GO analysis. A total of 2,238 BP pathways (e.g., modulation of chemical synaptic transmission, regulation of trans-synaptic signaling) and 180 CC pathways (e.g., synaptic membrane, neuronal cell body) were identified. And 264 MF pathways, including receptor ligand activity and channel activity **(Figure 2B) (Supplementary Table 4)**. These genes exhibited significant enrichment in 145 KEGG pathways (p < 0.05), such as neuroactive ligand-receptor interaction and hormone signaling **(Figure 2C) (Supplementary Table 5)**.

Subsequently, a PPI network containing 242 key nodes and 252 interactions was constructed (interaction score > 0.99), with 432 isolated nodes removed; this network suggested that there might be relatively strong interactions among these 242 proteins. Interactions such as EDNRA-EDN3 and ADCYAP1-VIPR1 were identified **(Figure 2D)**.

### 3.3 Identification of 4 prognostic genes

Using the 674 candidate genes, univariate Cox regression and PH assumption testing (p < 0.05, HR ≠ 1, p(PH) > 0.05) identified 114 candidate prognostic genes. Among these genes, POU4F1, KLC3, ATP2A1, and CALB2 were risk factors (HR > 1) **(Figures 3A**-**E)**. Subsequently, using the LASSO algorithm, the lambda. min was 0.07525804, and a total of 4 prognostic genes were screened, namely POU4F1, KLC3, ATP2A1, and CALB2 **(Figures 3F**-**G; Supplementary Table 6)**.

### 3.4 Establishment of the prognostic model of prognostic genes

A risk model was developed using the 4 prognostic genes and their LASSO regression coefficients. CRC risk score was calculated using: risk score = POU4F1 expression* (0.14736474) + ATP2A1 expression* (0.17807761) + KLC3 expression* (0.17564069) + CALB2 expression* (0.07693267) **(Table 1)**. Additionally, Applying the cutoff 0.4168864, the 367 training-set patients were divided into HRG (n = 37) and LRG (n = 330), with higher risk scores predicting shorter survival **(Figure 4A)**. KM survival analysis demonstrated significantly lower survival probability in the HRG compared to the LRG (p < 0.0001), confirming that higher risk scores are associated with worse clinical outcomes **(Figure 4B)**. ROC analysis of the training set yielded AUCs of 0.63, 0.65, and 0.63 for 1-, 2-, and 3-year survival, respectively **(Figure 4C)**, indicating reliable predictive performance of the prognostic model.

In addition, the stability of the prognostic model was evaluated using the 30% validation set and the TCGA-COADREAD dataset, and the results were comparable to those of the 70% training set, indicating that the prognostic model had good validity **(Figures 4D**-**I)**.

### 3.5 Distribution of clinical characteristics, independent prognostic analysis

Risk score variations among clinical subgroups were examined via the Wilcoxon test. Significant effects were observed for tumor_stage (p < 0.05) and pathologic_T (p < 0.01); the higher the grade, the higher the risk score, while age, gender, and pathologic_M were not significant **(Figures 5A**-**E)**. This indicated that the risk score could effectively reflect the degree of local progression of CRC.

Univariate Cox and PH tests identified risk score, age, M stage, pathological T4, and clinical stage III/IV as factors satisfying p < 0.05, HR ≠ 1, and p(PH) > 0.05 **(Figures 5F-K)**. Risk score, age, and M stage further passed multivariate Cox regression (p < 0.05) and the PH assumption (p > 0.05) **(Figures 5L**-**M)**. These results indicated that these three factors could significantly affect the OS of CRC patients independently of other clinical factors, with risk stratification providing additional prognostic value beyond that of conventional clinical factors. Therefore, independent prognostic factors correlated with CRC patient outcomes included risk score, age, and M stage.

### 3.6 Construction and validation of a nomogram model of independent prognostic factors

A nomogram based on risk score, age, and M stage—the three independent prognostic factors—was constructed to predict 1-, 2-, and 3-year mortality in CRC patients, reflecting the model's clinical diagnostic utility. When the total score reached 108, the predicted 1-, 2-, and 3-year mortality probabilities were 5.23%, 9.52%, and 12.9%, respectively **(Figure 6A)**. Additionally, Calibration curves of 1-, 2-, and 3-year OS closely followed the reference line slope, demonstrating strong agreement **(Figure 6B)**. ROC analysis produced AUC values of 0.85, 0.85, and 0.83 at 1, 2, and 3 years, respectively **(Figure 6C)**, which supported that the nomogram's predictive performance was favorable.

### 3.7 Biological pathways, immune microenvironment-related analyses

GSEA was conducted for the HRG and LRG, and a total of 58 pathways were enriched (|NES| > 1 and p < 0.05). Significantly enriched pathways included cytokine-cytokine receptor interaction, oxidative phosphorylation, and ribosome, etc. **(Figure 7A) (Supplementary Table 7)**. These results suggested that these pathways might contribute to the occurrence and development of CRC.

Infiltration of 28 immune cell types was assessed in the 367-sample training set between HRG and LRG **(Figure 7B)**. Central memory CD4 T cells and natural killer T cells were among the five cell types with significantly elevated infiltration in the HRG (p < 0.05) **(Figure 7C)**. These two cell types were also highly positively correlated (cor = 0.75, p = 2.20e-83) **(Figure 7D) (Supplementary Tables 8-9)**. CALB2 showed a highly significant positive association with natural killer T cells (cor = 0.51, p = 2.45e-25) and with central memory CD4 T cells (cor = 0.46, p = 8.8e-21) **(Figure 7E) (Supplementary Table 10)**. Together, the data implied that CALB2 could cooperate with the level of natural killer T-cell infiltration to affect CRC progression.

Significant expression differences were observed for seven immune checkpoint genes (p < 0.05), such as CD40, CD28, and CD276, between the HRG and LRG **(Figure 7F)**.

In addition, While TIDE and risk scores were not correlated, TIDE scores were significantly higher in the HRG than in the LRG (p < 0.01) **(Figure 7G)**. These findings suggested that tumors in the HRG possess stronger immune escape capabilities and may derive limited benefits from immune checkpoint inhibitor therapy.

### 3.8 Tumor variation analysis

In tumor somatic mutation profiles, TP53 (76% HRG, 60% LRG) and APC (59% HRG, 76% LRG) exhibited relatively high mutation rates across both groups. Missense mutations predominated in all samples **(Figures 8A**-**B)**.

A-B Somatic tumor mutations in high/low risk groups (Note: The figure lists the TOP20 genes, including APC, TP53, TTN, etc., and displays different types of mutations such as Missense Mutation, Frame_Shift_Ins, Nonsense_Mutation, Frame_Shift_Del, Multi_Hit, and Splice_Site. On the right, a bar chart illustrates the mutation frequency percentage of individual genes across the samples).

### 3.9 Prediction of chemotherapeutic drug sensitivity

A total of 33 drugs displayed significantly different sensitivities between the HRG and LRG (p < 0.05). Sepantronium bromide and luminespib were more effective in the LRG, whereas drugs such as gefitinib and carmustine exhibited greater sensitivity in the HRG. These 31 drugs demonstrated better efficacy in HRG patients **(Figure 9)**. Collectively, these findings suggested that different risk stratifications might affect the therapeutic efficacy of chemotherapeutic drugs.

### 3.10 Epithelial cells as key drivers in CRC progression

Before QC, the scRNA-seq data included 45,270 cells and 24,193 genes **(Figure 10A);** post-QC, 17,286 cells and 24,193 genes were kept **(Figure 10B)**. Using the top 2,000 HVGs and 30 PCs (p < 0.05; **Figures 10C-E**), t-SNE clustering (resolution = 0.1) identified 13 cell clusters **(Figures 10F-G)**. Marker-gene annotation categorized these clusters into eight cell types: T cells, myeloid cells, mast cells, B cells, plasma cells, epithelial cells, fibroblasts, and endothelial cells **(Figures 10H-K)**.

Moreover, the expression levels of 2 prognostic genes (ATP2A1 and KLC3) were significantly different in epithelial cells from the CRC group (p < 0.01) **(Figure 10L)**. Therefore, epithelial cells were identified as the key cell types for subsequent downstream analyses.

### 3.11 Functional enrichment and metabolic pathway analyses

Functional enrichment analysis showed that epithelial cells had high positive enrichment scores in the pathways of sensory perception of salty taste and hepoxilin (HX) and trioxilin (TrX) synthesis **(Figure 11A)**.

Among the top 10 metabolically active pathways, epithelial cells displayed high activity in nine (e.g., starch and sucrose metabolism, pyruvate metabolism) but not in the pentose phosphate pathway **(Figure 11B)**.

### 3.12 Pseudo-time analysis

The key cell type, epithelial cells, was subjected to reclustering and ultimately divided into 8 clusters (0-7) **(Figures 12A**-**D)**. Cells were ordered along a pseudotime trajectory from light to dark blue and grouped into five developmental stages (1-5), each marked by a unique color. The pseudotemporal distribution trajectory of each cell subset showed that cluster 0 and cluster 1 corresponded to the early and late developmental stages of cells, respectively; cluster 2 corresponded to the early and middle developmental stages, while the distribution characteristics of other clusters were not obvious. Developmental trajectories of key cells differed between disease and control groups upon stratification, indicating that disease status affects cellular developmental paths **(Figure 12E)**.

Furthermore, during cell differentiation, POU4F1 was not expressed, whereas the other 3 prognostic genes exhibited dynamic pseudotemporal expression patterns in the key cells at different stages. Specifically, the expression levels of ATP2A1, KLC3, and CALB2 were high in the early stage and then decreased, and ATP2A1 maintained a longer period of high expression compared with KLC3 and CALB2. These findings indicated that all 3 prognostic genes are closely associated with CRC progression **(Figure 12F)**.

### 3.13 Cellular communication analysis

Cell communication analysis revealed that T cells were the most abundant cell type in both the CRC group and the control group **(Figures 13A**-**D)**. In the CRC group, epithelial cells possessed the largest number of ligand-receptor pairs (6 pairs in total) with endothelial cells and exhibited the strongest interaction with myeloid cells. Within the control group, epithelial cells had the greatest number of ligand-receptor interactions with fibroblasts and endothelial cells (2 pairs per cell type), and the interaction with endothelial cells was the most robust **(Figures 13E**-**H)**.

Analysis of ligand-receptor interactions between the key cell type and other cell subsets showed that in the CRC group, the communication probability from epithelial cells to myeloid cells was the highest, with the corresponding ligand-receptor pair being MIF-(CD74+CD44) **(Figure 13I)**. The highest communication probability in the control group was from epithelial to endothelial cells, with PRSS3-F2RL3 identified as the corresponding ligand-receptor pair **(Figure 13J)**.

## 4. Discussion

CRC is a serious worldwide health concern with substantial morbidity and mortality 41. While treatment options have advanced, the molecular drivers of CRC progression, including the influence of neural factors, remain partially unclear. Neural components actively remodel the tumor microenvironment; neural signaling and innervation confer survival benefits to tumor cells and facilitate aggressive behaviors like invasion and metastasis 11, 16. Here, by integrating bulk and single-cell transcriptomic data, we systematically uncovered NRGs and mapped their expression profiles among different cell populations in CRC. Through Cox and LASSO regression analyses, we identified four key prognostic NRGs—POU4F1, ATP2A1, KLC3, and CALB2—which were further validated for their associations with immune infiltration and chemotherapeutic sensitivity. These results elucidate the critical role of neural elements in CRC progression and establish a solid scientific basis for the development of new prognostic biomarkers and targeted therapies.

### 4.1 Identification and functional characterization of a four-gene prognostic signature in CRC

In this study, through an in-depth analysis of gene expression profiles from patients with CRC, we identified a four-gene signature—comprising POU4F1, ATP2A1, KLC3, and CALB2—possessing robust prognostic stratification capabilities. These genes exhibit significant differential expression in CRC and correlate closely with patient outcomes, thereby serving as novel molecular biomarkers and potential therapeutic targets for personalized treatment. All four genes were characterized as independent risk factors associated with poor prognosis (Hazard Ratio > 1). Importantly, each of these genes retains intrinsic neural properties that are co-opted in the tumor microenvironment to drive CRC progression.

POU4F1 (Pou domain, class 4, transcription factor 1), primarily expressed in the developing nervous system 42, is a transcription factor that governs neuronal differentiation and survival 43. As a member of the class IV POU family that controls neural-specific gene expression programs 44, POU4F1's regulatory functions are aberrantly reactivated in cancer, conferring distinct oncogenic properties across various malignancies, including breast and esophageal squamous cell carcinomas 45. Previous research utilizing endoplasmic reticulum-related gene signatures, including POU4F1, successfully stratified risk in CRC patients 46. Mechanistically, POU4F1 drives CRC progression by facilitating cell proliferation, metastasis, and chemoresistance through transcriptional programs normally reserved for neural development 47. *In vitro* assays further demonstrate that POU4F1 knockdown significantly attenuates the proliferation and migration of colon adenocarcinoma (COAD) cells while inducing cellular senescence 48. Its consistent upregulation in COAD and its correlation with adverse clinical outcomes underscore its role as a pivotal mediator of tumor malignancy by exploiting neural developmental pathways 49. ATP2A1 encodes the sarco/endoplasmic reticulum Ca^2+^-ATPase 1 (SERCA1), an ATP-dependent calcium pump predominantly expressed in type II skeletal muscle cells 50.

Beyond its canonical role in muscle physiology, ATP2A1 is functionally important in neurons where precise calcium homeostasis is essential for synaptic transmission and plasticity 51, 52. This protein is essential for regulating intracellular calcium homeostasis, and its elevated expression has been implicated in driving malignant progression 53. Dysregulation of intracellular calcium levels is also a hallmark of neurodegeneration, leading to the aberrant activation of calcium-dependent processes and subsequent cell death 54, positioning ATP2A1 as a bridge between neural calcium regulation and oncogenic transformation. Clinical data show that elevated expression of ATP2A1 and ATP2A3 correlates with decreased survival in breast cancer patients 55. In CRC, Cox regression analysis identified ATP2A1 as an independent risk factor for poor prognosis. It appears to modulate CRC cell dynamics through a "calcium signaling-autophagy inhibition-immune microenvironment remodeling" axis, highlighting its role as a key driver of tumorigenesis 56. The neural origin of this calcium signaling dysregulation is particularly relevant in CRC, where perineural invasion is a common feature. KLC3 (kinesin light chain 3) serves as a core regulatory subunit of the kinesin-1 complex, mediating cargo recognition and recruitment through interaction with the heavy chain tail domain 57. In the nervous system, kinesin-mediated transport is essential for neuronal function, enabling long-distance trafficking of organelles and signaling molecules along axons 58, 59. It plays a fundamental role in intracellular transport, organelle positioning, and signal transduction. KLC3 has been integrated into prognostic signatures associated with 5-fluorouracil (5-FU) resistance in CRC 60. Functionally, KLC3 activates the PI3K/AKT signaling pathway to promote proliferation and migration in ovarian cancer 61 and interacts with the fructose transporter SLC2A5 to regulate MAPK signaling in gastric cancer progression 62. Consequently, KLC3 likely functions as a central oncogene in CRC, participating in tumor development, chemoresistance, and prognostic outcomes through multi-pathway regulatory mechanisms analogous to its neuronal transport functions. CALB2 (calbindin 2), a 29 kDa member of the EF-hand calcium-binding protein family, is an integral component of calcium signaling 63, 64. In the nervous system, CALB2 is highly expressed in specific neuronal populations where it protects against calcium-mediated excitotoxicity and regulates neurotransmitter release 65, 66. It has been linked to neural plasticity 67 and identified as a prognostic biomarker in CRC that may serve as a target for gemcitabine (GEM) therapy 68. Studies have validated its role in promoting the invasion and migration of colon cancer cells in vitro 68, suggesting that CALB2 drives CRC via a multifaceted "calcium signaling-invasion-metabolic/immune remodeling" pathway. The neural calcium buffering function of CALB2 is particularly relevant in the tumor microenvironment, where calcium dynamics influence both cancer cell behavior and immune cell function 66, 69. Our study further revealed a strong positive association between CALB2 expression and the infiltration of both NKT cells and central memory CD4+ T cells. This suggests that CALB2 may act as an "immune regulatory hub," where its high expression potentially activates immune-related pathways and promotes the secretion of chemokines (e.g., CXCL9, CXCL10) to recruit anti-tumor immune cells 68. Thus, CALB2 exhibits a dual functional role: directly promoting tumor cell invasion through neural calcium regulatory mechanisms while simultaneously modulating the immune landscape to influence tumor immune evasion 70.

Collectively, these four genes represent critical molecular determinants of CRC progression, each contributing distinct mechanisms rooted in their neural origins. The convergence of these neural-derived functions underscores that cancer cells can co-opt neural signaling and cellular machinery to drive malignant progression. The risk model derived from these prognostic genes demonstrates high predictive performance for CRC outcomes. Furthermore, a nomogram combining clinical staging and risk scores offers a practical and effective tool for individualized prognosis assessment. By identifying high-risk patients, these models assist clinicians in formulating more aggressive therapeutic strategies, such as intensified postoperative adjuvant chemotherapy or targeted therapy, ultimately improving long-term survival rates. These models also facilitate the selection of patient cohorts for clinical trials, significantly enhancing the success rates of novel therapeutic interventions and advancing the goals of precision medicine in oncology.

### 4.2 Functional enrichment reveals immune-metabolic reprogramming in high-recurrence-risk CRC

To further understand the biological basis underlying the prognostic performance of our gene signature, we conducted functional enrichment analysis to identify key pathways associated with high-risk CRC. Our analysis revealed significant enrichment of the "cytokine-cytokine receptor interaction" and "OXPHOS" pathways in the HRG, offering important clues to the molecular mechanisms of CRC and potential therapeutic targeting. Cytokines are central to coordinating robust immune responses and possess substantial potential in the treatment of infections, autoimmune diseases, and malignancies 71. The cytokine-cytokine receptor interaction pathway represents a complex network of signaling molecules and their cognate receptors; their binding triggers intracellular signal transduction that modulates diverse cellular functions 72. Disruption of these signaling axes is frequently associated with various pathological conditions 73. In CRC, cytokines play a pivotal role in mediating intercellular communication and shaping the TME 74. Patients in high-risk groups exhibit enhanced chemokine signaling and cytokine-receptor interactions, alongside increased focal adhesions 75. Furthermore, Src-related kinase lacking C-terminal regulatory tyrosine and N-terminal myristoylation sites (SRMS) has been found to regulate CRC progression by modulating these interactions, as well as IL-17 and IgA signaling pathways 76. Notably, the C-X-C motif chemokine ligand 12 (CXCL12) has been identified as a potential biomarker that promotes chemo-sensitivity and radio-resistance in CRC 77, underscoring the intimate link between aberrant cytokine activation and CRC pathogenesis.

Additionally, OXPHOS serves as a fundamental energy-transfer process, generating the majority of cellular ATP via proton and electrochemical gradients across the inner mitochondrial membrane 78, 79. While traditionally viewed through the lens of the Warburg effect, cancer is increasingly recognized as a metabolic disease in which OXPHOS plays a vital role in progression. OXPHOS not only provides the requisite energy for tumor survival but also orchestrates the conditions necessary for proliferation, invasion, and metastasis. Metabolic shifts toward OXPHOS can further impair the functionality of immune cells within the TME, facilitating immune evasion 80. In CRC specifically, the E3 ubiquitin ligase ARIH1 has been shown to promote progression by facilitating the oxidative phosphorylation of K63-linked ubiquitin-associated PHB1 through mitochondrial translocation 81. OXPHOS levels also serve as a predictive biomarker for oxaliplatin response 82. Mechanistically, elevated PHB2 interacts directly with NDUFS1 to stabilize mitochondrial complex I, thereby upregulating OXPHOS and driving CRC tumorigenesis 83. Moreover, upregulated OXPHOS has been identified as a signature metabolic alteration during CRC liver metastasis, a process that can be induced by TGFβ signaling 84. These findings highlight that the aberrant activation of the OXPHOS pathway is a key metabolic driver of CRC development, therapeutic response, and distant metastasis, representing a significant opportunity for prognostic stratification and targeted intervention.

### 4.3 Characterization of the immune landscape and synergistic cellular modules in high-risk CRC

Beyond metabolic reprogramming, alterations in the tumor immune microenvironment represent another critical dimension distinguishing high-risk from low-risk CRC. Immune cell infiltration analysis revealed distinct landscapes of the TME between the HRG and LRG, providing critical insights into the immune regulatory mechanisms of CRC. Notably, central memory CD4+ Tcm cells and NKT cells exhibited significantly higher infiltration levels within the HRG. T cells are recognized as the cornerstone of cancer immunotherapy 85, with CD4+ T cells serving as the central orchestrators of both innate and antigen-specific adaptive immune responses 86. These cells are typically activated through T-cell receptor (TCR) recognition of cognate antigens presented by major histocompatibility complex II (MHC-II) on antigen-presenting cells, supplemented by costimulatory signaling. Upon activation, CD4+ T cells proliferate and secrete a diverse repertoire of cytokines and chemokines 87. The Tcm subset 88, along with memory stem T cells (Tscm), represents a promising immunological indicator for the early detection and auxiliary diagnosis of CRC 89.

Furthermore, tumor recurrence after immunotherapy is largely driven by cancer stem cells (CSCs), which are characterized by high expression of surface markers such as CD44, CD133, and Lgr5. Notably, CD44 is also a functional molecule highly expressed on Tcm cells, where it mediates cell homing, activation, and the maintenance of immune memory in anti-tumor immunity. This shared expression of CD44 suggests that Tcm cells may interact with CSCs within the tumor microenvironment, potentially influencing both immune surveillance and tumor recurrence mechanisms. Thus, Tcm cells constitute a core memory immune subpopulation intricately linked to CRC immune regulation and the risk of recurrence.

NKT cells form a unique lymphocyte population linking innate and adaptive immune responses. These cells recognize lipid antigens in the context of CD1d and swiftly release cytokines upon activation 90. Alongside NK cells, NKT cells are quintessential innate effectors possessing potent anti-tumor and antimicrobial capabilities 91. Interleukin-17A (IL-17A), a pro-inflammatory cytokine primarily secreted by Th17, γδ T, and NKT cells, has been shown to induce CRC cell pyroptosis through mitochondrial dysfunction, subsequently promoting the infiltration of CD8+ T cells 92. Alongside NK cells, NKT cells are quintessential innate effectors possessing potent anti-tumor and antimicrobial capabilities. Interleukin-17A (IL-17A), a pro-inflammatory cytokine primarily secreted by Th17, γδ T, and NKT cells, has been shown to induce CRC cell pyroptosis through mitochondrial dysfunction, subsequently promoting the infiltration of CD8+ T cells. These observations suggest that NKT cells can contribute to anti-tumor immunity in CRC. However, accumulating evidence indicates that this protective function may be compromised in the tumor microenvironment. Specifically, CRC tumor cells express ligands for NK cell receptors that alter the phenotypes of circulating NK and NKT cells, facilitating immune evasion during metastasis 93. This indicates a dual role for NKT cells in HRG patients: while they may exert anti-tumor effects through IL-17A-mediated pyroptosis and CD8+ T cell recruitment, their functional phenotype can be subverted by tumor-derived ligands to weaken immunosurveillance and promote metastasis. The significant positive correlation observed between Tcm and NKT cells in this study suggests a tight synergistic regulatory nexus within the HRG immune microenvironment, collectively forming a central module of the anti-tumor immune response characteristic of high-risk CRC.

In conclusion, by integrating bulk and single-cell transcriptomics, we identified a neural-associated four-gene signature—POU4F1, ATP2A1, KLC3, and CALB2—possessing robust prognostic value in CRC. This multi-omic framework elucidates the molecular drivers and immune landscapes of CRC, offering a theoretical basis for personalized medicine. Although our study provides important computational biology insights, several limitations should be acknowledged. First, our analyses are primarily based on bioinformatics approaches and lack integration with metabolomics data as well as experimental validation. Second, although the prognostic model was validated using multiple internal strategies within the TCGA cohort, independent external validation using GEO cohorts could not be completed due to platform heterogeneity and data availability constraints; this limitation warrants confirmation in future prospective cohort studies. Furthermore, our findings remain contingent upon data quality and bioinformatics assumptions, necessitating rigorous biological experiments to exclude potential false-positive results. In future work, we will employ gain-of-function and loss-of-function assays to further elucidate the regulatory mechanisms of these targets on malignant phenotypes, including proliferation, invasion, and metastasis, and will combine these with in vivo animal models to evaluate their therapeutic potential. In summary, this study establishes a foundation for the development of novel targeted intervention strategies.

## Supplementary Material

Supplementary tables.

## Figures and Tables

**Figure 1:**
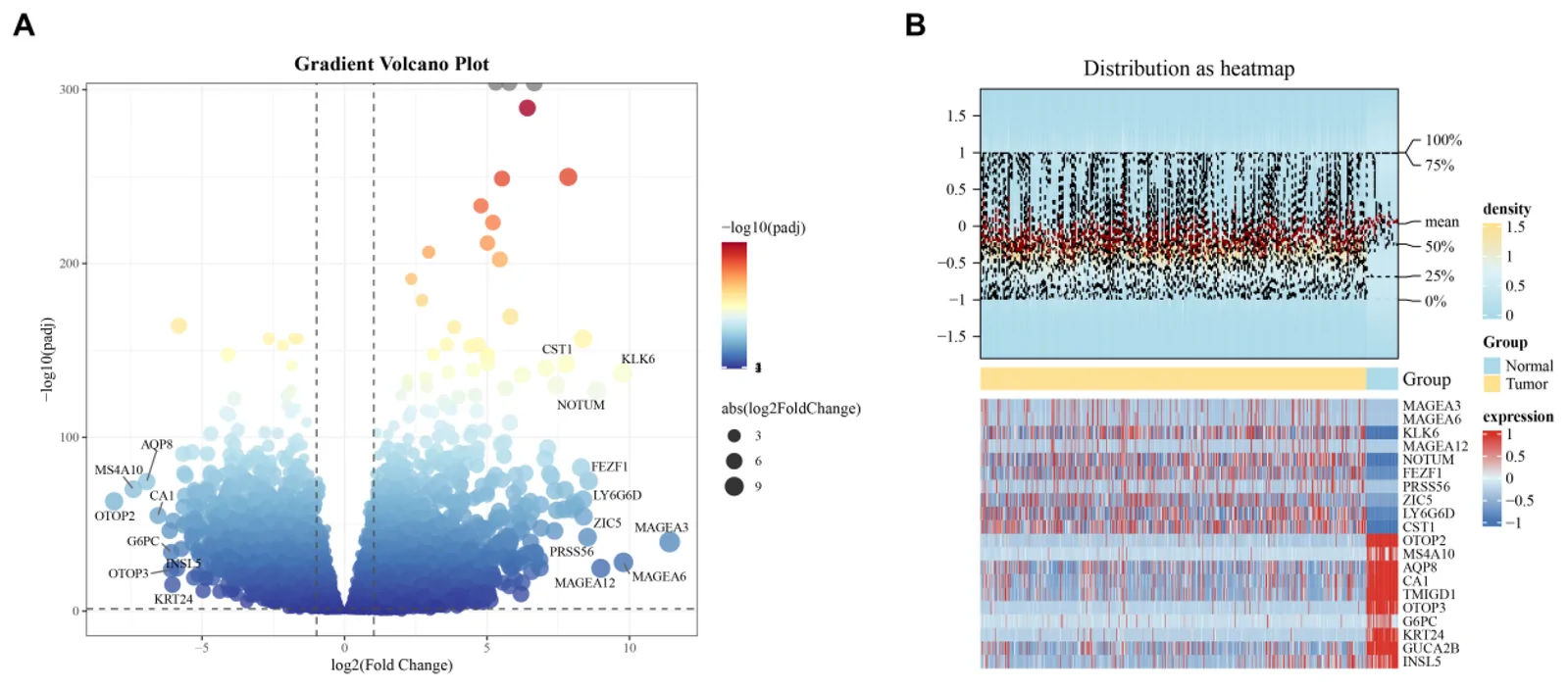
** Differential expression analysis.** (A) Volcano plot of DEGs (|log₂FoldChange (FC)| > 1 and p-adjust < 0.05). (B) Heatmap of DEGs (|log_2_FC| > 1 and p-adjust < 0.05).

**Figure 2:**
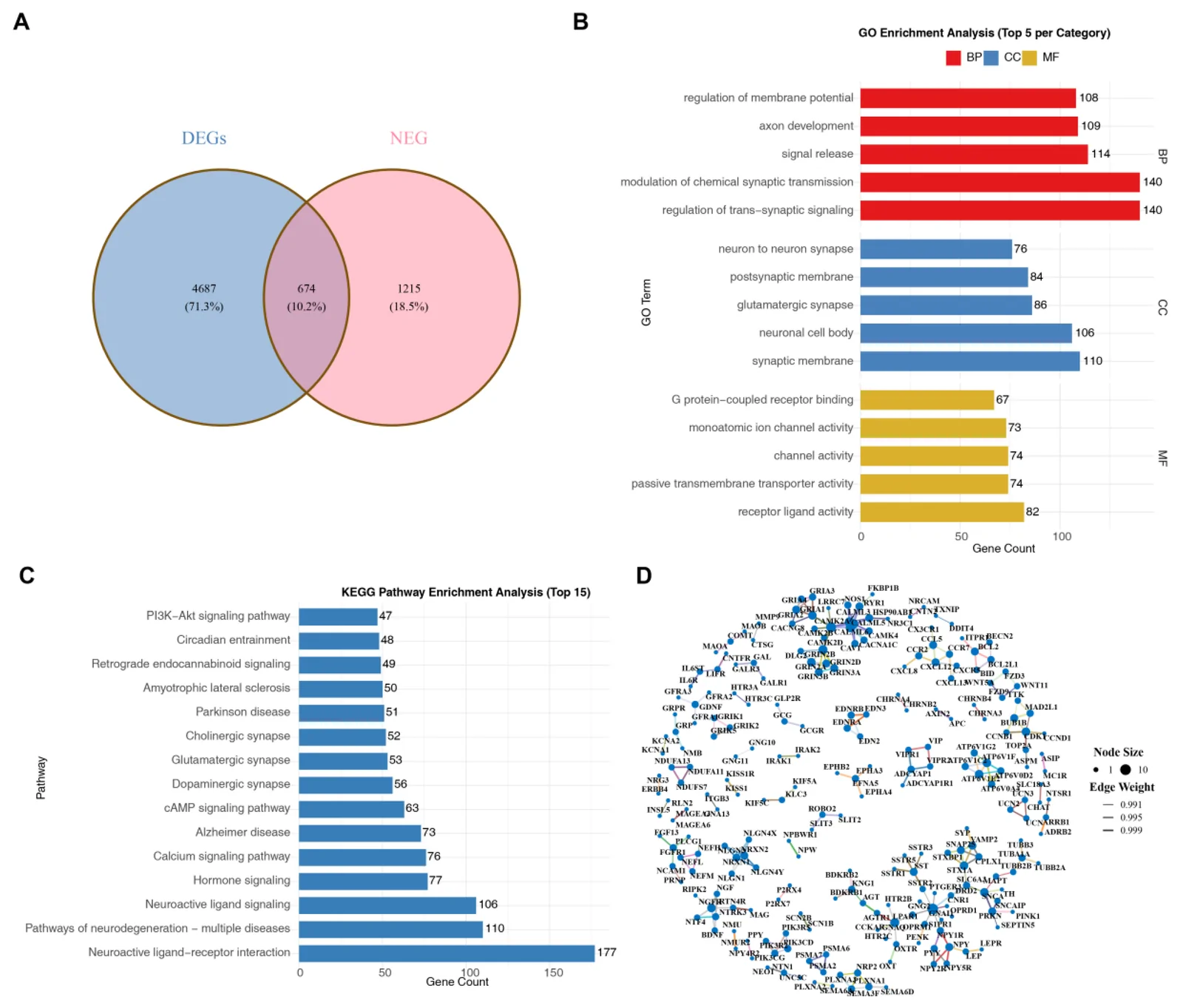
** Acquisition of candidate genes.** (A) Candidate genes displayed by a Venn diagram. (B) GO enrichment plot (p < 0.05). (C) KEGG enrichment plot (p < 0.05). (D) Protein-protein interaction (PPI) network (interaction score > 0.99).

**Figures 3:**
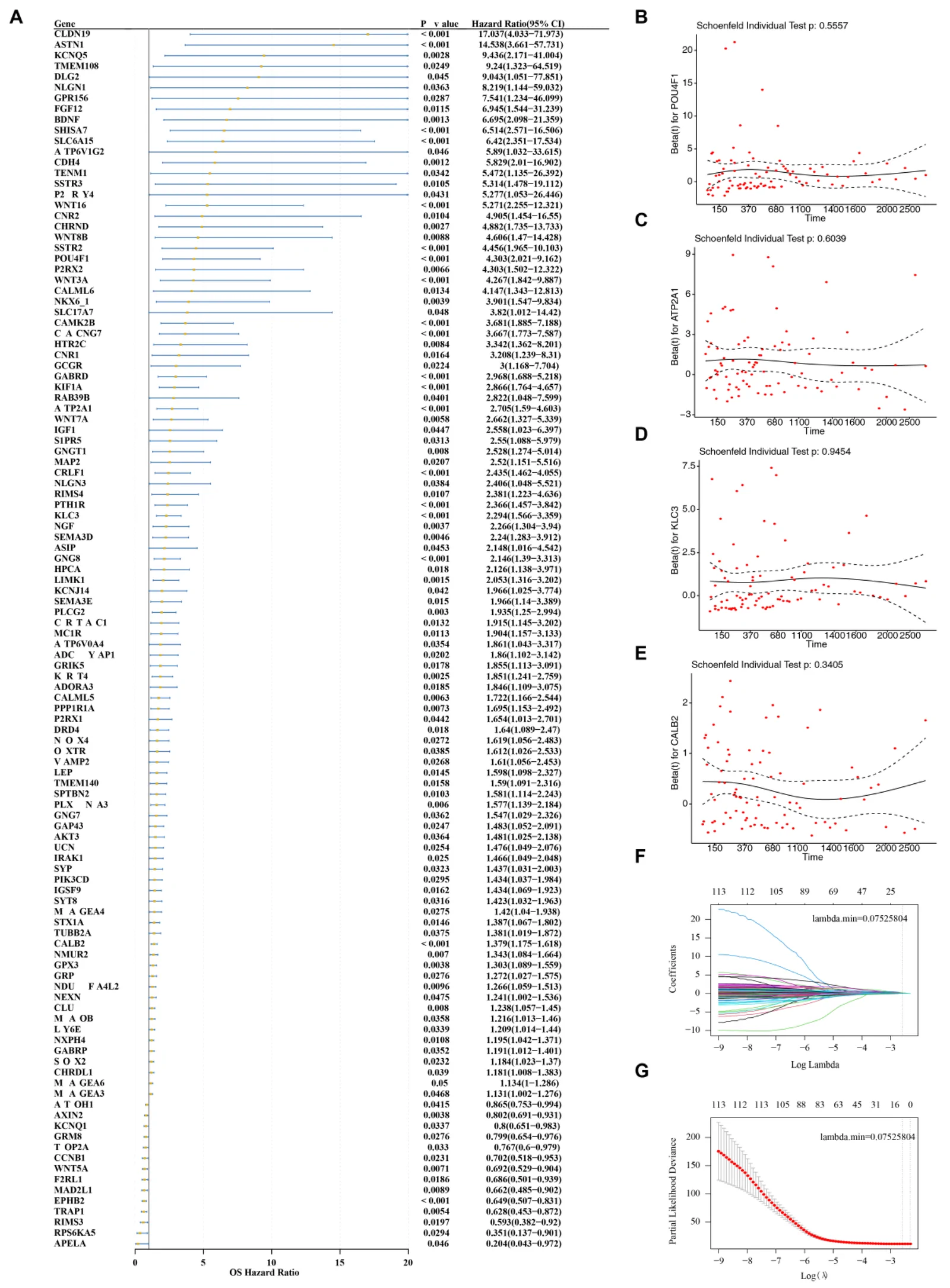
** Identification of prognostic genes.** (A) Univariate Cox regression forest plot (p < 0.05, HR ≠ 1, p (PH) > 0.05) (Note: On the left side of the figure are the gene information screened by the univariate test: genes with a hazard ratio (HR) greater than 1 are defined as risk factors, while those with an HR less than 1 are protective factors. The middle panel presents a visualization of these values, where the gray dashed line represents the critical value of 1; the yellow dots denote the HR values, and the horizontal segments extending to the left and right of each dot indicate the corresponding 95% confidence interval (95%CI). (B-E) 4 candidate prognostic genes PH hypothesis testing (p > 0.05). (F-G) Cross-validation plot and regression coefficient path plot of LASSO regression.

**Figure 4:**
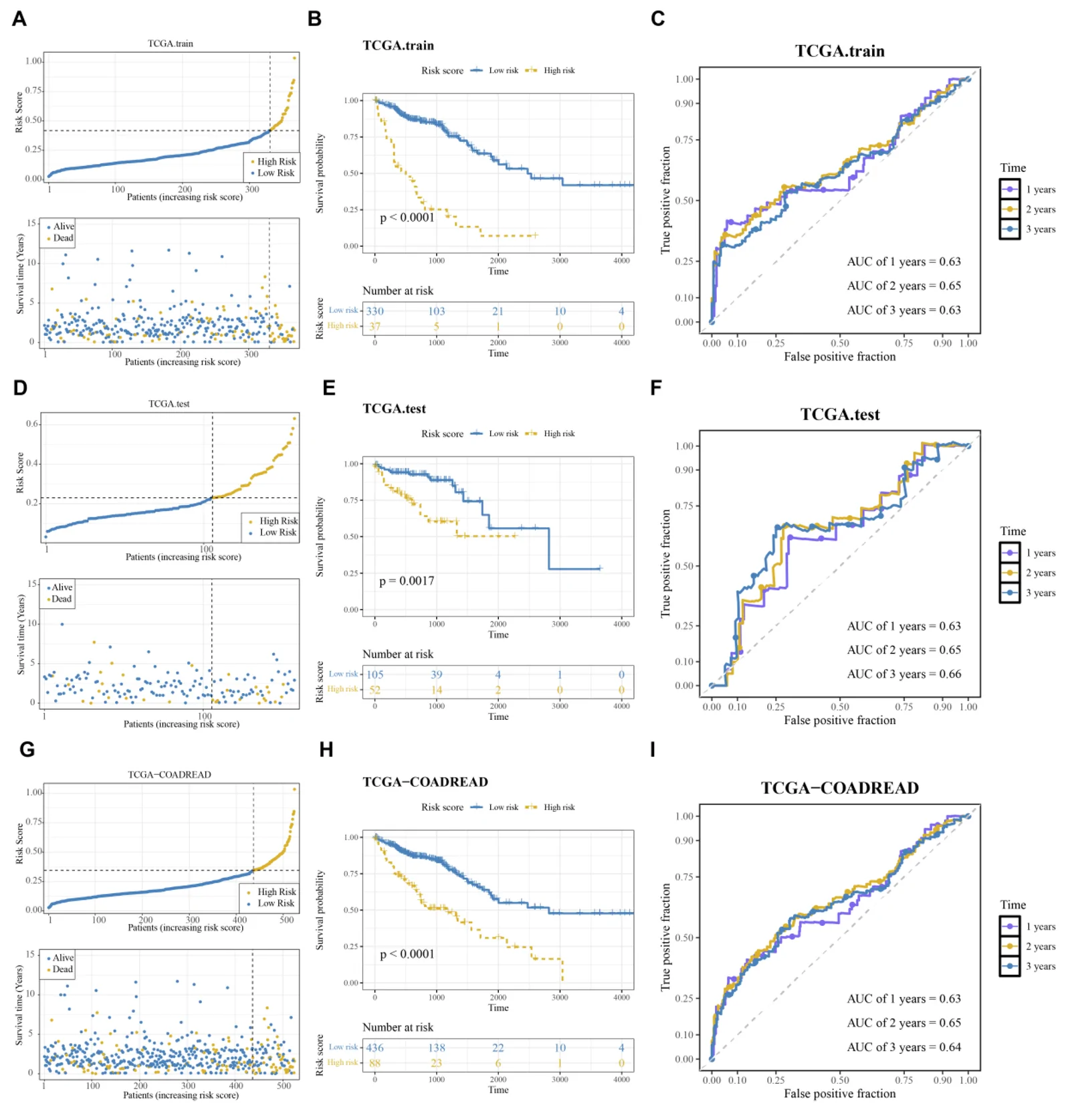
** Establishment of the risk model** (Based on the 70% training set). (A) Risk curve-survival status plots. (B) Kaplan-Meier (KM) survival curve (p < 0.05). (C) Receiver operating characteristic (ROC) curves (AUC > 0.6). (D-F) (Based on the 30% validation set) Risk curve-survival status plots; KM survival curve; ROC curves. (D-I) (Based on the TCGA-COADREAD dataset) Risk curve-survival status plots; KM survival curve; ROC curves.

**Figure 5:**
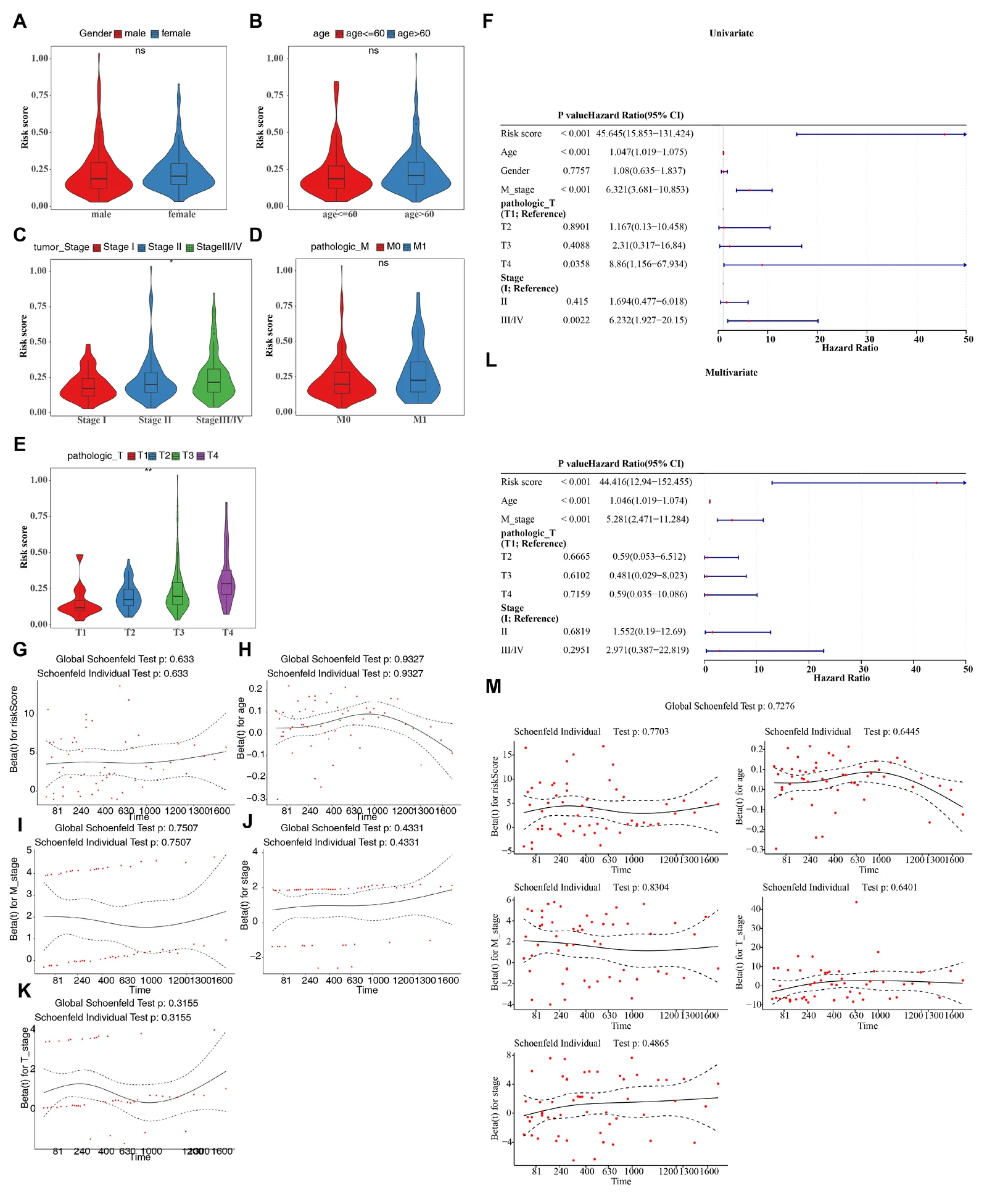
** Distribution of clinical characteristics, independent prognostic analysis.** (A-E) Box plot of risk score differences among subgroups stratified by clinical characteristics (p < 0.05). (F) Univariate Cox regression forest plot (p < 0.05, HR ≠ 1). (G-K) PH hypothesis testing (p > 0.05). (L) Multivariate Cox regression forest plot (p < 0.05, HR ≠ 1). (M) PH hypothesis testing (p > 0.05).

**Figure 6:**
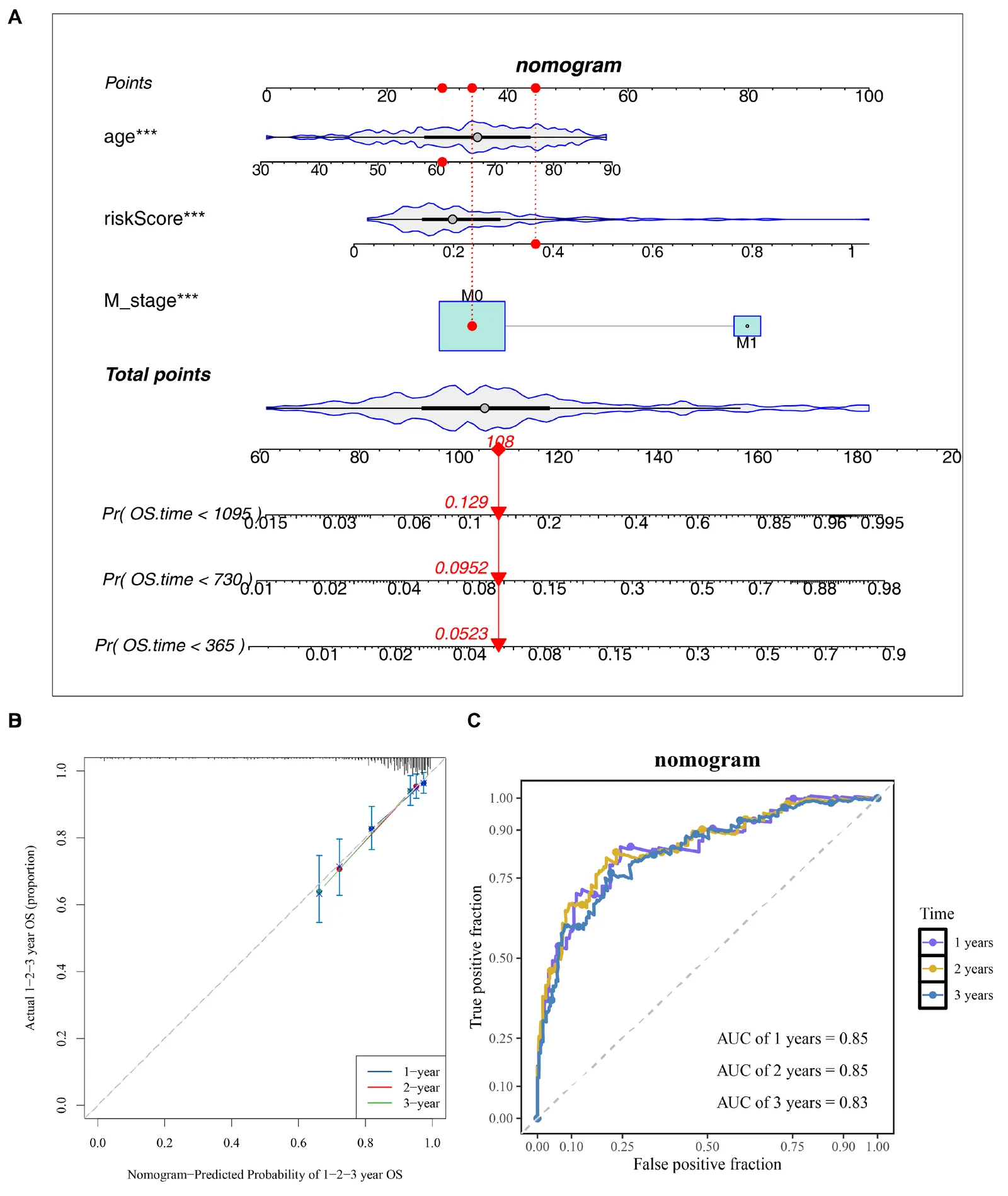
** Construction of the nomogram.** (A) Nomogram (Note: variable names (left side): A scale is marked on the line corresponding to each variable, representing the range of possible values of that variable, while the length of the line segment reflects the magnitude of the contribution of this factor to the disease; scores: The individual score refers to the score assigned to each variable corresponding to its different values; the total score refers to the sum of the individual scores corresponding to the values of all variables; predicted probability: Represents the probability of death.). (B) Calibration curves for 1-year, 2-year, and 3-year survival of the nomogram. (C) Receiver operating characteristic (ROC) curves (AUC > 0.7).

**Figure 7:**
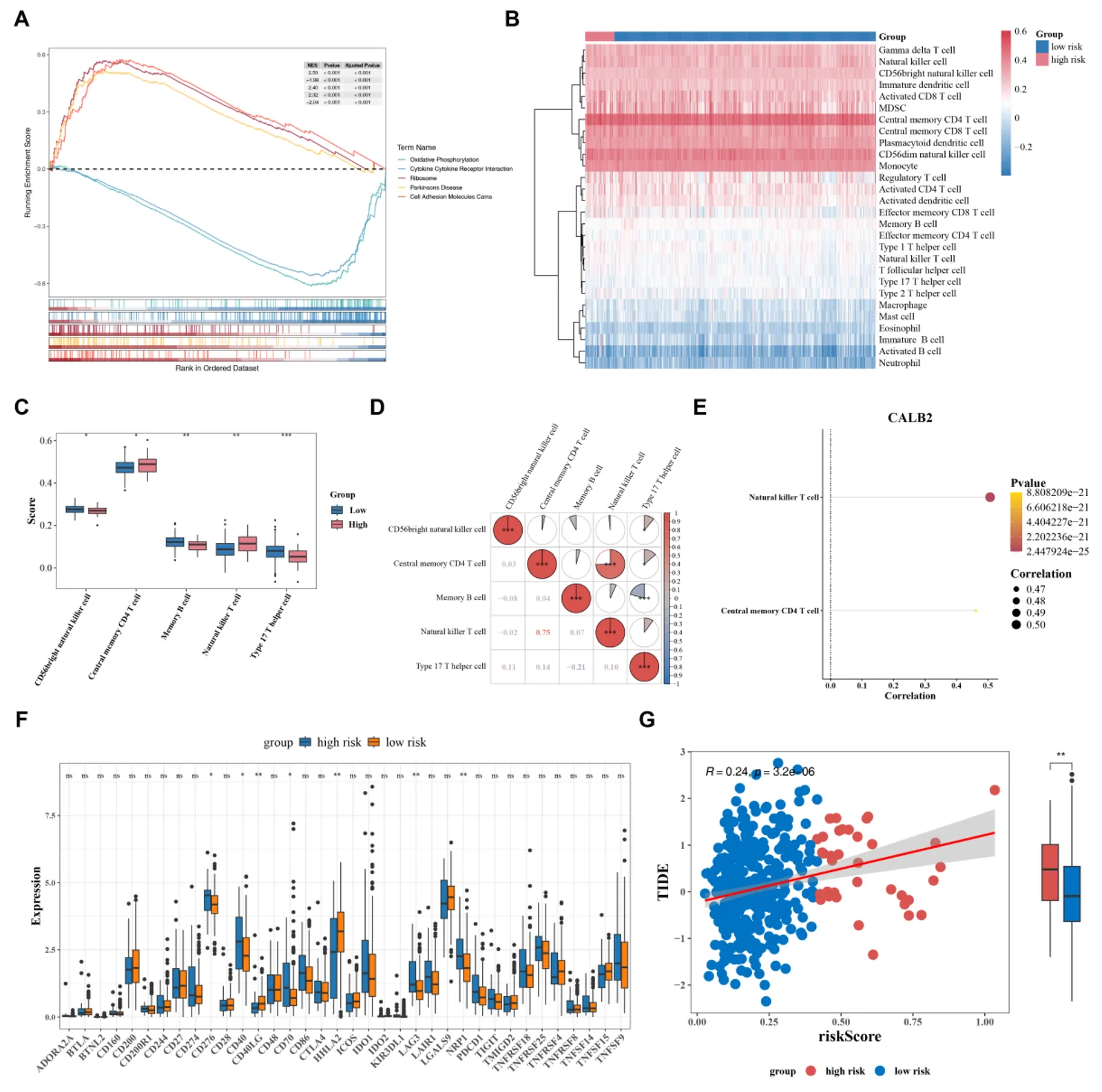
** Biological pathways, immune microenvironment**-**related analyses.** (A) GSEA enrichment plot (high-risk group vs. low-risk group) (|NES| > 1 and p < 0.05). (B) Abundance of immune cells (high-risk group vs. low-risk group). (C) Box plot of differential immune cells between high-risk and low-risk groups (p < 0.05). (D) Correlations between differential immune cells (|correlation coefficient (cor)| > 0.3 and p < 0.05). (E) Correlations between differential immune cells and prognostic genes (|cor| > 0.3 and p < 0.05). (F) Expression of immune checkpoints in high-risk and low-risk groups (p < 0.05) (Note: The x-axis represents immune checkpoints, and the y-axis represents expression values. ***indicates p < 0.001, **indicates p < 0.01, * indicates p < 0.05, and ns indicates no significance.). (G) Differential analysis of TIDE scores between high- and low-risk groups (p < 0.05).

**Figures 8:**
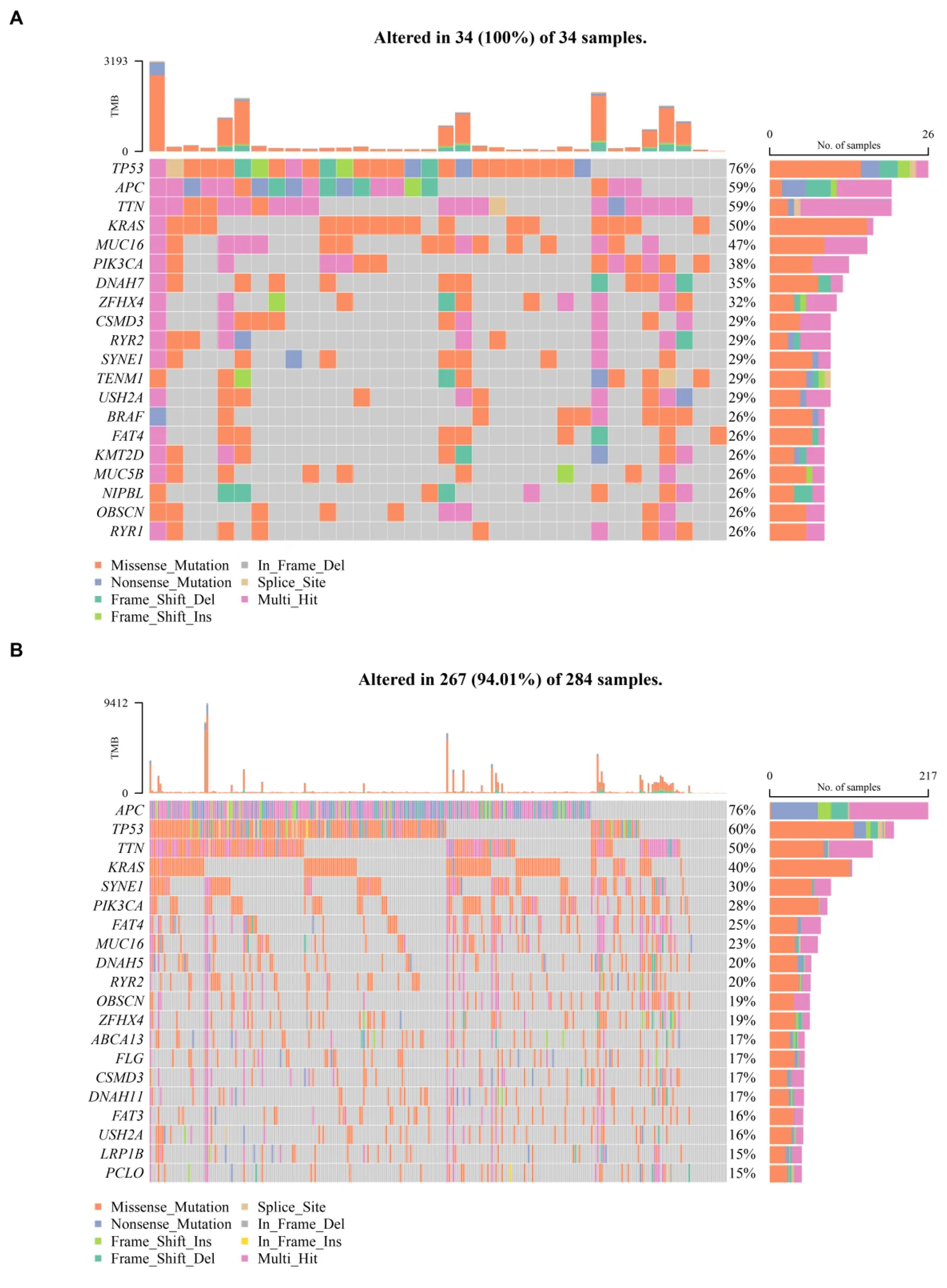
**Distribution and frequency of somatic mutations in high- versus low-risk groups.** A-B Somatic tumor mutations in high/low risk groups (Note: The figure lists the TOP20 genes, including APC, TP53, TTN, etc., and displays different types of mutations such as Missense Mutation, Frame_Shift_Ins, Nonsense_Mutation, Frame_Shift_Del, Multi_Hit, and Splice_Site. The bar chart on the right shows the mutation frequency percentage of each gene in the samples).

**Figure 9:**
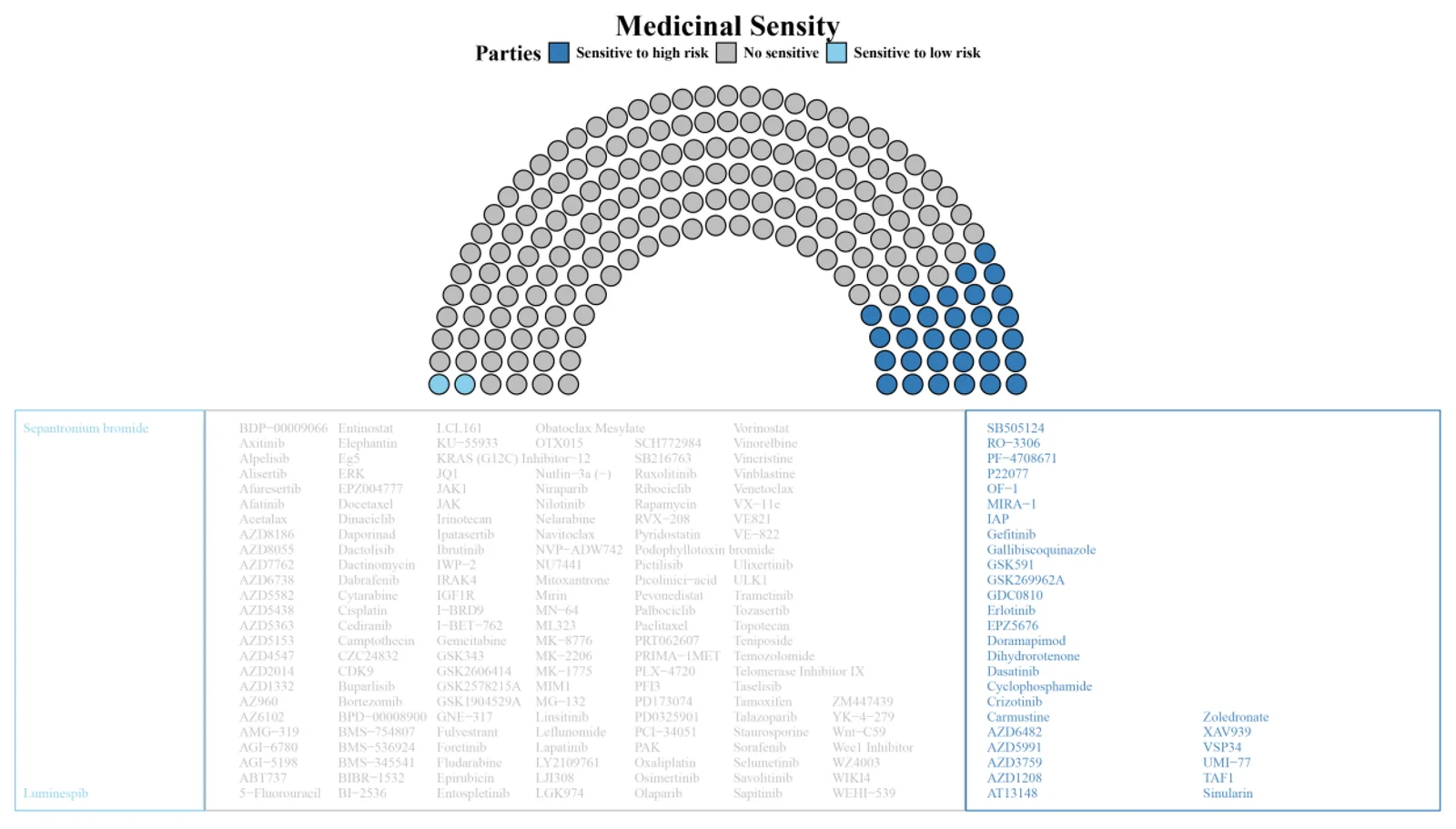
** Boxplot of drug sensitivity analysis (high-risk group vs. low-risk group) (p < 0.05)**.

**Figure 10:**
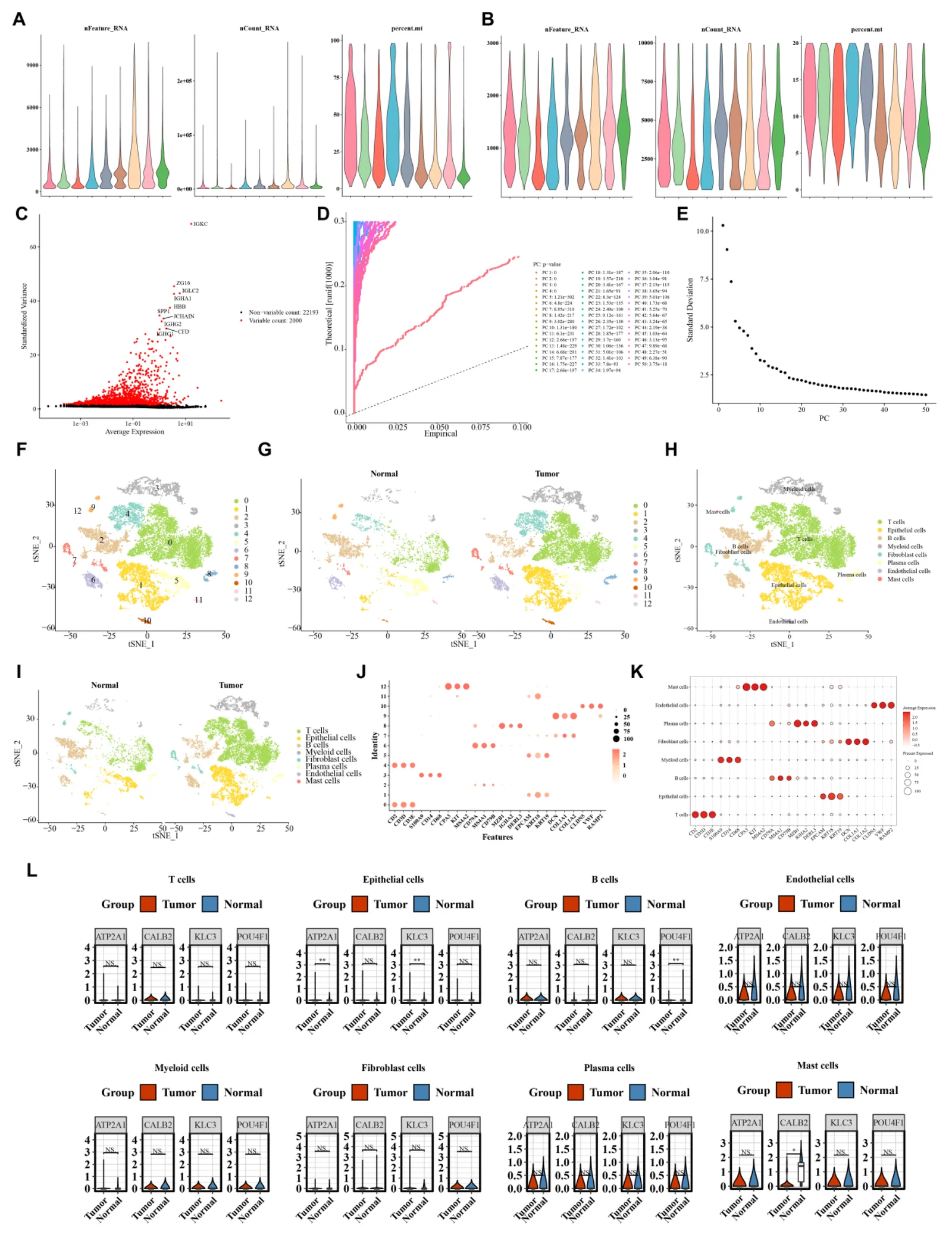
** Single-cell RNA sequencing (scRNA-seq) analysis.** (A-B) Raw scRNA-seq data quality control. (C-E) screening of highly variable genes (Note: The x-axis represents the gene expression level, the y-axis represents the differential gene expression, and the red dots in the figure represent highly variable genes), PCA plot, scree plot. (F-G) TSNE plot. (H-K) Final cell annotation TSNE plot. Cell type marker expression plot. (L) Analysis of expression levels of prognostic cells in annotated cell types (CRC vs. control groups) (Note: Differences in the expression of prognostic genes between CRC and control groups across distinct cell types: The x-axis represents the grouping, and the y-axis represents the expression levels of each prognostic gene in different cell types and different groups. * indicates p < 0.05, ** indicates p < 0.01, *** indicates p < 0.001, and ns indicates no significance.).

**Figure 11:**
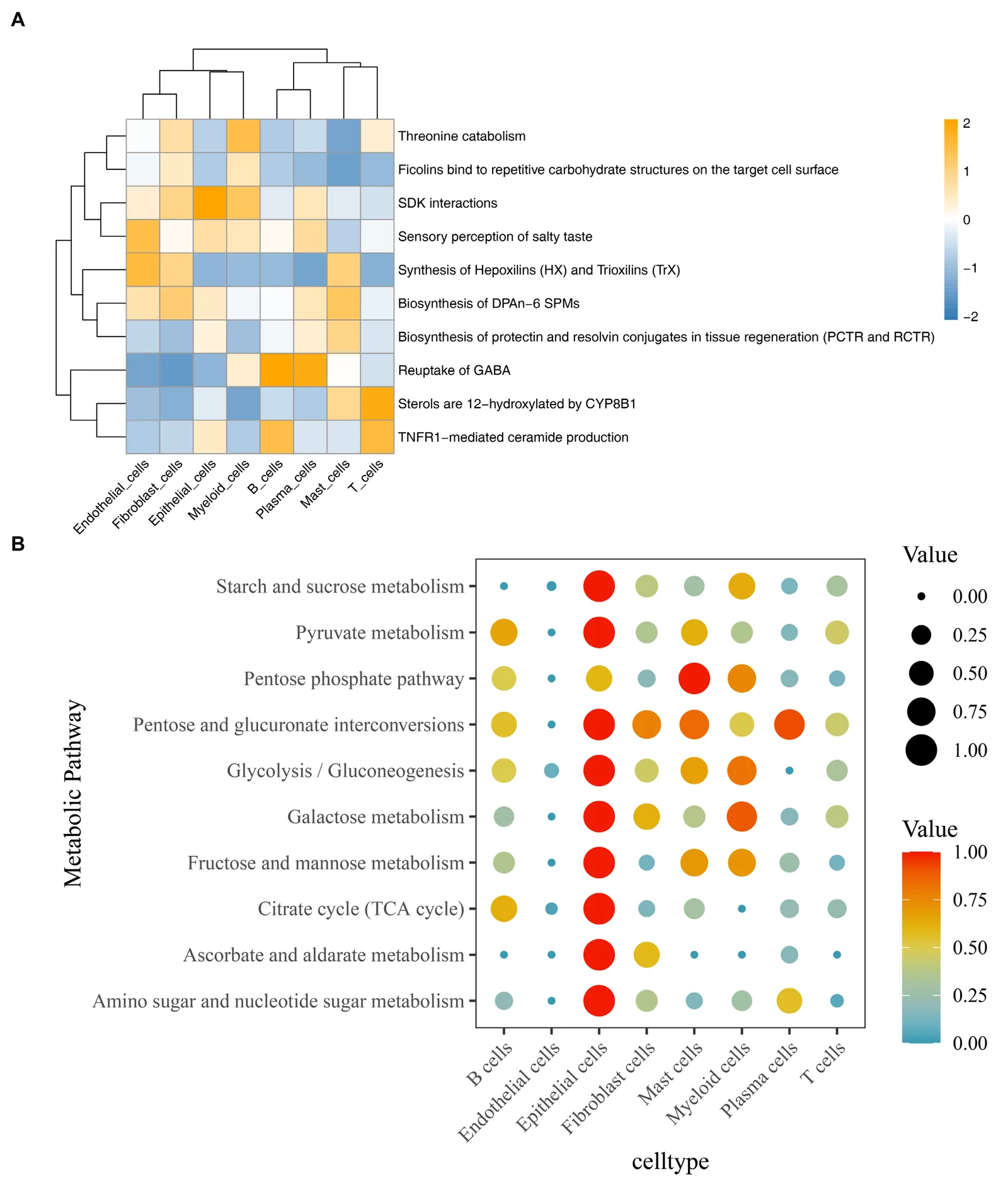
** Functional enrichment and metabolic pathway analyses.** (A) Functional enrichment analysis. (B) Heatmap of metabolic activity in different cell types.

**Figure 12:**
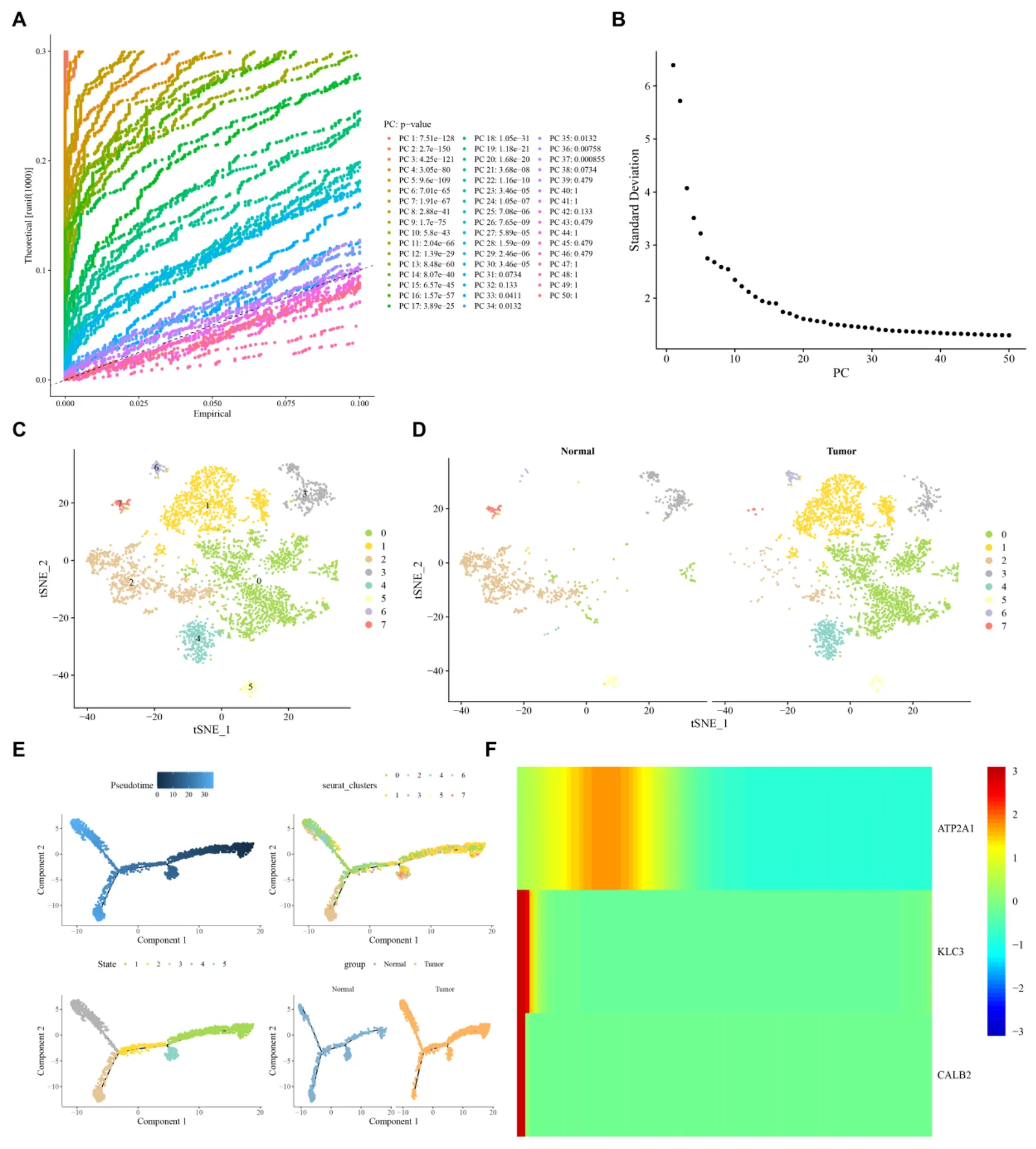
** Pseudo-time analysis.** (A-B) Key cells were subjected to secondary clustering. PCA plot, scree plot. (C-D) TSNE plot, Final cell annotation TSNE plot (E) Pseudotime trajectory plot of pseudotemporal analysis. (F) Gene expression distribution plot of prognostic genes along the pseudotime of key cells.

**Figure 13:**
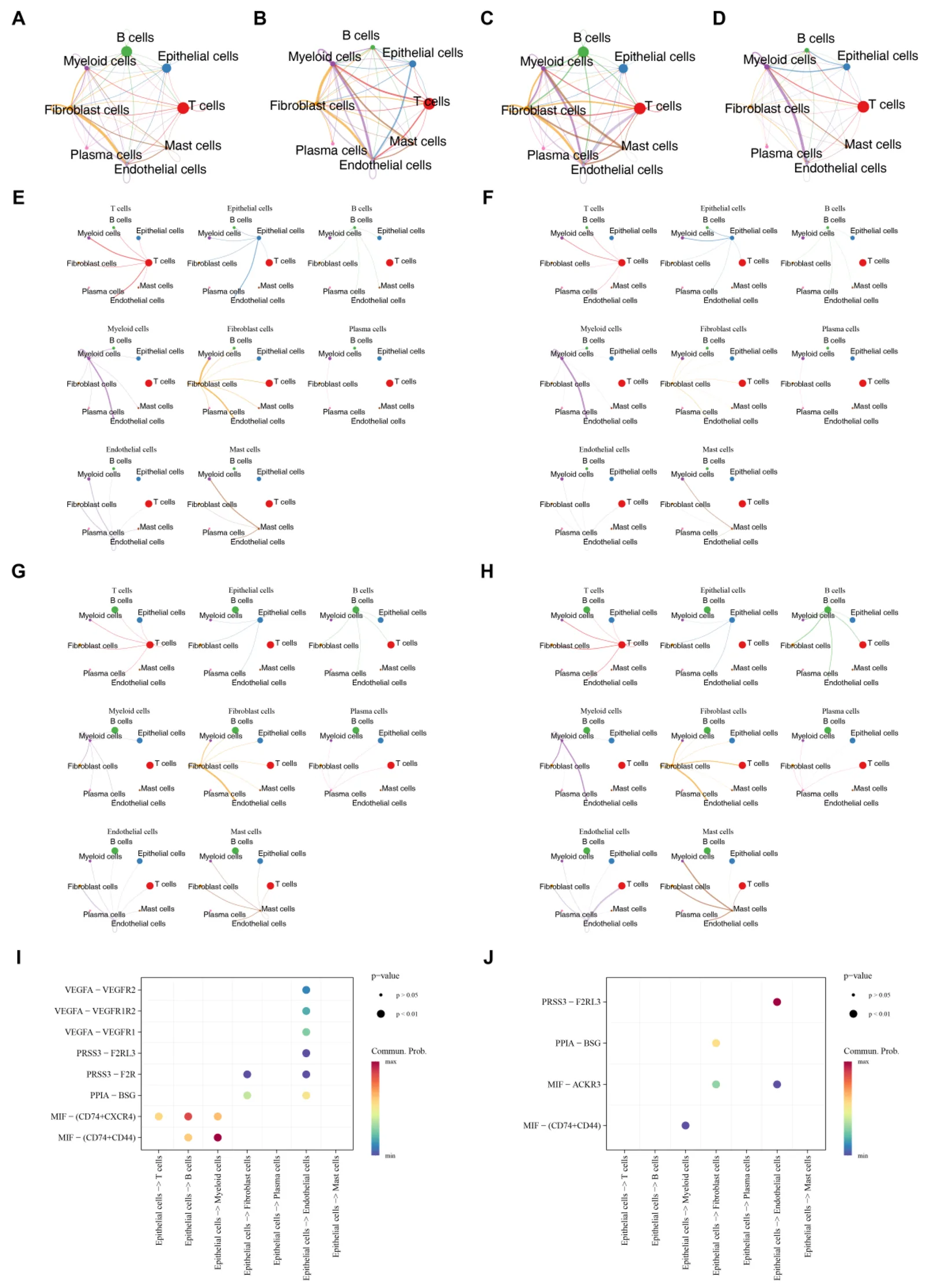
** Cellular communication analysis.** (A) The number of interactions between key cells and other cells in the control group. (B) The number of interactions between key cells and other cells in the CRC group. (C) The weight/strength of interactions between key cells and other cells in the control group. (D) The weight/strength of interactions between key cells and other cells in the CRC group. (E-F) cell-cell interaction network between key cells and other cells (number and weight) (in the CRC group). (G-H) cell-cell interaction network between key cells and other cells (number and weight) (in the control group). (I-J) Bubble plot of ligand-receptor pairs involved in interactions between key cells and other cells (CRC vs. control groups).

**Table 1:** Linear partial coefficients (coef) of prognostic genes obtained by LASSO regression.

Genes	Coef
POU4F1	0.14736474
ATP2A1	0.17807761
KLC3	0.17564069
CALB2	0.07693267

## Data Availability

The datasets analysed in this study are available in the The Cancer Genome Atlas (TCGA) database (http://cancergenome.nih.gov/), including TCGA-COAD and TCGA-READ dataset; and the Gene Expression Omnibus (GEO) database (http://www.ncbi.nlm.nih.gov/geo/), including GSE231559 dataset.
