# Supplementary material for: Prognostic value of neuro-related genes in colorectal cancer and their potential implications for immunotherapy
Source: J Cancer. 2026 Sep 18;17(10):1744–66. doi: 10.7150/jca.136690 (PMC13617419; doi:10.7150/jca.136690)
Supplement: Supplementary file 1 [file jcav17p1744s1.pdf]

Supplementary Table 1: List of 1,889 neural-related genes (NRGs).

Symbol
CHRM1
CHRM2
CHRM3
CHRM4
CHRM5
ADRA1A
ADRA1B
ADRA1D
ADRA2A
ADRA2B
ADRA2C
ADRB1
ADRB2
ADRB3
DRD1
DRD2
DRD3
DRD4
DRD5
HRH1
HRH2
HRH3
HRH4
HTR1A
HTR1B
HTR1D
HTR1E
HTR1F
HTR2A
HTR2B
HTR2C
HTR4
HTR5A
HTR6
HTR7
TAAR9
TAAR6
TAAR1
TAAR8
TAAR5
TAAR2
AGT
AGTR1
AGTR2
APLN
APELA
APLNR
NMB
GRP
NMBR
GRPR
BRS3
KNG1
BDKRB1
BDKRB2
C3
C3AR1
C5

Symbol
C5AR1
VGF
FPR1
FPR3
FPR2
CCK
CCKAR
CCKBR
EDN1
EDN2
EDN3
EDNRA
EDNRB
GAL
GALP
SPX
GALR1
GALR2
GALR3
GHRL
GHSR
KISS1
KISS1R
POMC
MC1R
MC2R
MC3R
MC4R
MC5R
MLN
MLNR
NMU
NMS
NMUR1
NMUR2
NPFF
NPVF
NPFFR1
NPFFR2
NPS
NPSR1
NPY
PYY
PPY
NPY1R
NPY2R
NPY4R
NPY4R2
NPY5R
GPR83
NPW
NPB
NPBWR1
NPBWR2
NTS
NTSR1
NTSR2
PENK
PDYN

Symbol
PNOC
OPRD1
OPRK1
OPRM1
OPRL1
QRFP
QRFPR
HCRT
HCRTR1
HCRTR2
OXT
OXTR
SST
CORT
SSTR1
SSTR2
SSTR3
SSTR4
SSTR5
TAC1
TAC4
TACR1
TACR2
TAC3
TACR3
UTS2
UTS2B
UTS2R
AVP
AVPR1A
AVPR1B
AVPR2
F2
CTSG
GZMA
PLG
PRSS3
PRSS2
PRSS1
F2R
F2RL1
F2RL2
F2RL3
PARD3
PRLH
PRLHR
PMCH
MCHR1
MCHR2
FSHB
CGA
FSHR
LHB
LHCGR
TSHB
GPHA2
GPHB5
TSHR
PTGDR

Symbol
PTGER1
PTGER2
PTGER3
PTGER4
PTGFR
PTGIR
TBXA2R
ADORA1
ADORA2A
ADORA2B
ADORA3
P2RY2
P2RY1
P2RY4
P2RY6
LPAR6
P2RY10
P2RY14
P2RY8
P2RY11
P2RY13
GPR35
CNR1
CNR2
PTAFR
GNRH1
GNRH2
GNRHR
TRH
TRHR
MTNR1A
MTNR1B
GPR50
LPAR1
LPAR2
LPAR3
LPAR4
S1PR1
S1PR2
S1PR3
S1PR4
S1PR5
LTB4R
LTB4R2
MAS1
RLN1
RLN2
RLN3
INSL3
INSL5
RXFP1
RXFP2
RXFP3
RXFP4
CYSLTR1
CYSLTR2
CALCA
CALCB
ADM

Symbol
ADM2
IAPP
CALCR
CALCRL
CRH
UCN
UCN3
UCN2
CRHR1
CRHR2
GIP
GIPR
GCG
GCGR
GLP1R
GLP2R
GHRH
GHRHR
PTH
PTH2
PTH1R
PTH2R
ADCYAP1
ADCYAP1R1
SCT
SCTR
VIP
VIPR1
VIPR2
GRM1
GRM2
GRM3
GRM4
GRM5
GRM6
GRM7
GRM8
GABBR1
GABBR2
GPR156
GRIN1
GRIN2A
GRIN2B
GRIN2C
GRIN2D
GRIN3A
GRIN3B
GABRA1
GABRA2
GABRA3
GABRA4
GABRA5
GABRA6
GABRB1
GABRB3
GABRB2
GABRD
GABRE
GABRG1

Symbol
GABRG2
GABRG3
GABRP
GABRR1
GABRR2
GABRR3
GABRQ
SLURP1
SLURP2
LYPD6
LYPD6B
LYNX1
PATE4
PATE1
PATE3
PATE2
CHRNA1
CHRNA2
CHRNA3
CHRNA4
CHRNA5
CHRNA6
CHRNA7
CHRNA9
CHRNA10
CHRNB1
CHRNB2
CHRNB3
CHRNB4
CHRND
CHRNE
CHRNG
P2RX1
P2RX2
P2RX3
P2RX4
P2RX5
P2RX7
P2RX6
GRIA1
GRIA2
GRIA3
GRIA4
GRIK1
GRIK2
GRIK3
GRIK4
GRIK5
GRID1
GRID2
GLRA1
GLRA2
GLRA3
GLRB
TRPV1
TSPO
NR3C1
GH1
GH2

Symbol
CSH1
CSH2
GHR
THRA
THRB
LEP
LEPR
PRL
PRLR
APP
MAPT
SNCA
TARDBP
FUS
SOD1
ATXN2L
ATXN2
HTT
ATXN1L
ATXN1
ATXN3
ATXN3L
PRNP
SPTBN2
GNAQ
PRKN
PLCB1
PLCB2
PLCB3
PLCB4
PLCG1
PRKCA
PRKCB
PRKCG
TRPC3
SIGMAR1
ITPR1
ITPR2
ITPR3
RYR1
RYR2
RYR3
DLG4
CACNA1B
CACNA1C
CACNA1D
CACNA1F
CACNA1S
STX1A
ATP2A1
ATP2A3
ATP2A2
PSEN1
PSEN2
DERL1
VCP
UBA52
RPS27A
UBB

Symbol
UBC
PSMA6
PSMA2
PSMA4
PSMA7
PSMA8
PSMA5
PSMA1
PSMA3
PSMB6
PSMB7
PSMB3
PSMB2
PSMB5
PSMB1
PSMB4
PSMC2
PSMC1
PSMC4
PSMC6
PSMC3
PSMC5
PSMD2
PSMD1
PSMD3
PSMD9
PSMD12
PSMD11
PSMD6
PSMD7
PSMD13
PSMD4
PSMD14
PSMD8
ADRM1
SEM1
UCHL1
UBA1
UBA7
UBE2L3
UBE2L6
UBE2J2
UBE2J1
UBE2G2
UBE2G1
GPR37
SEPTIN5
SNCAIP
HSPA5
VAPB
ATF6
DDIT3
XBP1
ERN1
TRAF2
MAP3K5
MAP2K7
MAPK8
MAPK10

Symbol
MAPK9
CASP12
MAP3K10
EIF2AK3
EIF2S1
ATF4
SLC6A3
PINK1
ND1
ND2
ND3
ND4
ND4L
ND5
ND6
NDUFV1
NDUFV2
NDUFV3
NDUFA1
NDUFA2
NDUFA3
NDUFA4
NDUFA4L2
NDUFA5
NDUFA6
NDUFA7
NDUFA8
NDUFA9
NDUFA10
NDUFAB1
NDUFA11
NDUFA12
NDUFA13
NDUFB1
NDUFB2
NDUFB3
NDUFB4
NDUFB5
NDUFB6
NDUFB7
NDUFB8
NDUFB9
NDUFB10
NDUFB11
NDUFS1
NDUFS2
NDUFS3
NDUFS4
NDUFS5
NDUFS6
NDUFS7
NDUFS8
NDUFC1
NDUFC2
NDUFC2-KCTD14
SDHA
SDHB
SDHC
SDHD

Symbol
UQCRFS1
CYTB
CYC1
UQCRC1
UQCRC2
UQCRH
UQCRHL
UQCRB
UQCRQ
UQCR10
UQCR11
COX3
COX1
COX2
COX4I2
COX4I1
COX5A
COX5B
COX6A1
COX6A2
COX6B1
COX6B2
COX6C
COX7A1
COX7A2
COX7A2L
COX7B
COX7B2
COX7C
COX8C
COX8A
ATP5F1A
ATP5F1B
ATP5F1C
ATP5F1D
ATP5F1E
ATP6
ATP5PB
ATP5MC1
ATP5MC2
ATP5MC3
ATP5PD
ATP5PO
ATP5PF
ATP8
BCL2
VDAC1
TOMM40
TOMM40L
MCU
CALML3
CALM2
CALM3
CALM1
CALML6
CALML5
CALML4
PPP3CA
PPP3CB

Symbol
PPP3CC
PPP3R1
PPP3R2
BAD
FASLG
FAS
FADD
CASP8
BID
BAX
BAK1
LRRK2
HTRA2
TRAP1
VDAC2
VDAC3
SLC25A4
SLC25A5
SLC25A6
SLC25A31
PPIF
CYCS
APAF1
CASP9
CASP3
HIP1
IFT57
HSD17B10
PPID
PARK7
BCL2L1
MFN1
MFN2
OPTN
SQSTM1
MAP1LC3C
MAP1LC3B
MAP1LC3A
MAP1LC3B2
TBK1
TANK
WDR41
SMCR8
C9orf72
RAB8A
RAB39B
RAB1A
MTOR
ULK1
ULK2
ATG101
ATG13
RB1CC1
AMBRA1
BECN1
BECN2
PIK3R4
ATG14
NRBF2

Symbol
PIK3C3
ATG2A
ATG2B
WIPI2
WIPI1
ZFYVE1
ALS2
RAB5A
RAC1
CHMP2B
FIG4
SPG11
TNF
TNFRSF1A
TNFRSF1B
DAXX
MAP2K3
MAP2K6
MAPK11
MAPK12
MAPK13
MAPK14
NOS1
CCS
GPX6
GPX7
GPX2
GPX3
GPX1
GPX5
GPX8
CAT
PRPH
NEFL
NEFM
NEFH
KIF5A
KIF5B
KIF5C
KLC3
KLC1
KLC2
KLC4
TUBA1B
TUBA4A
TUBA3C
TUBA1A
TUBA1C
TUBA8
TUBA3E
TUBA3D
TUBAL3
TUBB6
TUBB
TUBB1
TUBB2A
TUBB3
TUBB4A
TUBB8

Symbol
TUBB2B
TUBB4B
GSK3B
CSNK2A1
CSNK2A2
CSNK2A3
CSNK2B
BDNF
DCTN1
DCTN2
DCTN3
DCTN4
DCTN5
DCTN6
ACTR1A
ACTR1B
ACTR10
HAP1
DNAH1
DNAH3
DNAH2
DNAH7
DNAH5
DNAH6
DNAH9
DNAH11
DNAH12
DNAH14
DNAH8
DNAH10
DNAH17
DNALI1
DNAI1
DNAI2
DNAL1
DNAL4
CAMK2A
CAMK2D
CAMK2B
CAMK2G
CAPN1
CAPN2
CDK5R1
CDK5
CASP7
DKK1
DKK2
DKK4
WNT1
WNT2
WNT2B
WNT3
WNT3A
WNT4
WNT5A
WNT5B
WNT6
WNT7A
WNT7B

Symbol
WNT8A
WNT8B
WNT9A
WNT9B
WNT10B
WNT10A
WNT11
WNT16
FZD1
FZD7
FZD2
FZD3
FZD4
FZD5
FZD8
FZD6
FZD10
FZD9
LRP5
LRP6
CSNK1E
TPTEP2-CSNK1E
DVL3
DVL2
DVL1
FRAT1
FRAT2
AXIN1
AXIN2
APC
APC2
CTNNB1
CSNK1A1L
CSNK1A1
AGER
HRAS
KRAS
NRAS
ARAF
BRAF
RAF1
MAP2K1
MAP2K2
MAPK1
MAPK3
NOX1
CYBB
NOX4
RELA
NFKB1
NOS2
PTGS2
IL6
IL1A
IL1B
CSF1
SLC38A1
SLC38A2
GLS2

Symbol
GLS
SLC17A6
SLC17A8
SLC17A7
DLGAP1
SHANK3
SHANK1
SHANK2
TRPC1
HOMER1
HOMER2
HOMER3
PLA2G4E
PLA2G4A
JMJD7-PLA2G4B
PLA2G4B
PLA2G4C
PLA2G4D
PLA2G4F
PLD1
PLD2
GNAS
ADCY1
ADCY2
ADCY3
ADCY4
ADCY5
ADCY6
ADCY7
ADCY8
ADCY9
PRKACA
PRKACB
PRKACG
GNAI1
GNAI3
GNAI2
GNAO1
GNB1
GNB2
GNB3
GNB4
GNB5
GNG2
GNG3
GNG4
GNG5
GNG7
GNG8
GNG10
GNG11
GNG12
GNG13
GNGT1
GNGT2
GRK2
GRK3
KCNJ3
CACNA1A

Symbol
SLC1A2
SLC1A7
SLC1A1
SLC1A6
SLC1A3
GLUL
SLC38A3
GAD1
GAD2
SLC32A1
ABAT
SRC
GABARAP
GABARAPL1
GABARAPL2
NSF
TRAK2
PLCL1
GPHN
SLC12A5
KCNJ6
SLC6A1
SLC6A13
SLC6A11
SLC6A12
SLC38A5
CHAT
ACHE
SLC18A3
GNA11
KCNQ1
KCNQ2
KCNQ3
KCNQ4
KCNQ5
KCNJ2
KCNJ12
KCNJ18
KCNJ4
KCNJ14
PIK3CG
PIK3R5
PIK3R6
FOS
CREB1
CREB3
CREB3L1
CREB3L2
CREB3L3
CREB3L4
CREB5
CAMK4
JAK2
FYN
PIK3CA
PIK3CD
PIK3CB
PIK3R1
PIK3R2

Symbol
PIK3R3
AKT1
AKT2
AKT3
SLC5A7
TH
DDC
SLC18A1
SLC18A2
CALY
GNAL
ATF2
ATF6B
PPP1R1B
PPP1CA
PPP1CB
PPP1CC
SCN1A
KCNJ9
KCNJ5
ARRB1
ARRB2
PPP2CA
PPP2CB
PPP2R1B
PPP2R1A
PPP2R2A
PPP2R2B
PPP2R2C
PPP2R2D
PPP2R3B
PPP2R3C
PPP2R3A
PPP2R5B
PPP2R5C
PPP2R5D
PPP2R5E
PPP2R5A
GSK3A
CLOCK
BMAL1
MAOB
MAOA
COMT
LRTOMT
TPH2
TPH1
CYP2C8
CYP2C9
CYP2C18
CYP2C19
CYP2D6
CYP2D7
CYP2J2
CYP4X1
ALOX5
ALOX12
ALOX12B
ALOX15

Symbol
ALOX15B
PTGS1
HTR3A
HTR3E
HTR3D
HTR3C
HTR3B
KCNN2
KCND2
RAPGEF3
DUSP1
SLC6A4
PPP1R1A
RAP1A
RAP1B
CREBBP
EP300
RPS6KA3
RPS6KA1
RPS6KA2
RPS6KA6
GUCY1A2
GUCY1A1
GUCY1B1
PRKG1
PRKG2
PPP1R17
GNAZ
GNA12
GNA13
LYN
IGF1
IGF1R
NAPEPLD
DAGLA
DAGLB
ABHD6
FAAH
MGLL
RIMS1
SYT1
VAMP2
RAB3A
STX2
STX3
STX1B
STXBP1
UNC13A
UNC13B
UNC13C
SNAP25
CPLX1
CPLX2
CPLX3
CPLX4
NAPA
DNM1
DNM3
DNM2

Symbol
CLTA
CLTB
CLTC
CLTCL1
AP2A2
AP2A1
AP2B1
AP2M1
AP2S1
ATP6V1A
ATP6V1B1
ATP6V1B2
ATP6V1C2
ATP6V1C1
ATP6V1D
ATP6V1E2
ATP6V1E1
ATP6V1F
ATP6V1G1
ATP6V1G3
ATP6V1G2
ATP6V0E1
ATP6V0E2
TCIRG1
ATP6V0A2
ATP6V0A4
ATP6V0A1
ATP6V0D1
ATP6V0D2
ATP6V1H
ATP6V0C
ATP6V0B
SLC6A2
SLC6A5
SLC6A9
SLC6A7
NGF
NTF4
NTF3
NTRK1
NTRK2
NTRK3
SH2B2
SH2B1
SH2B3
GRB2
SOS1
SOS2
RPS6KA5
KIDINS220
FRS2
CRK
CRKL
RAPGEF1
MAP3K3
MAP2K5
MAPK7
MAPKAPK2
SHC1

Symbol
SHC2
SHC3
SHC4
GAB1
NFKBIB
NFKBIA
NFKBIE
FOXO3
PDPK1
IRS1
PLCG2
PRKCD
ABL1
PTPN11
MATK
NGFR
ARHGDIB
ARHGDIA
ARHGDIG
RHOA
CDC42
MAP3K1
JUN
TP53
TP73
TRAF6
ZNF274
PRDM4
MAGED1
BEX3
YWHAE
RIPK2
IRAK1
IRAK2
IRAK3
IRAK4
IKBKB
SORT1
CLN8
ITGB1
KCNJ10
ATP1A2
PER2
KIF17
KIF3B
KIF1C
MAP2
KIF1A
TRIM46
FSTL4
NFATC4
SLC2A4
RAPGEF2
SYT2
RIMS4
RAB3GAP1
SYT11
SYT4
SYT5

Symbol
DOC2B
SYT8
SYT7
RIMS2
RIMS3
EEF2K
SH3GL2
TMEM108
UBE3A
WASF1
EIF4A3
CNTF
CNTFR
IL6R
IL6ST
LIFR
SULF1
GATA3
GFRA1
GFRA2
GFRA3
GFRAL
SULF2
RET
GFRA4
GDF15
NF1
DTNBP1
KMO
FAT3
CCR4
ARX
EVX1
LHX6
FEZF1
PAFAH1B1
FEZF2
RELN
SOX1
CNTN2
NR2F2
NKX2-1
DAB1
OLIG3
CELSR2
LHX1
ASCL1
TBX20
NEUROD4
VEGFA
NRP1
PHOX2B
NTN1
CNTN4
NLGN4Y
NLGN1
ASTN2
ASTN1
NCAM2

Symbol
NINJ2
NLGN3
NLGN4X
NLGN2
TNR
NRXN3
NRXN1
NRXN2
SEMA3A
NRG3
CHL1
SDCCAG8
TUBGCP2
VAX1
ACAP3
COL3A1
UNC5D
CTNNA2
DAB2IP
DNAAF4
DCC
DCX
FBXO45
MARK2
EMX2
PHACTR1
FGF13
FGFR1
NTNG1
FOXG1
ZNF609
ADGRL3
SATB2
SRGAP2
PLXNB2
FLRT2
NIPBL
ASPM
NSMF
AUTS2
GAS6
GATA2
MDGA1
DISC1
GPM6A
WDR62
IGSF10
LRP12
TLX3
HSP90AA1
NEXMIF
BARHL2
SEPTIN14
ITGA3
CEP85L
MAP1B
MARK1
MDK
MEF2C

Symbol
NDN
ATOH1
NEO1
NKX6-1
NRCAM
NR4A2
PAX6
PCM1
DCDC2
PEX7
PEX13
PLXNA1
SEPTIN4
GPR173
DDIT4
POU4F1
NDE1
ULK4
ELP3
PLXNA3
AXL
BARHL1
ZMIZ1
TBC1D24
SEMA6A
SH3RF1
SHTN1
BBS1
PEX5
BBS4
CX3CL1
CXCL12
TNN
DRGX
SRGAP2C
SPOCK1
SRF
STAT3
CDKL5
TOP2B
TWIST1
TYRO3
CXCR4
NDNF
FBXO31
KIAA0319L
NDEL1
USP9X
EOMES
SCRT1
SOX14
KIRREL3
NTNG2
POMGNT2
UNK
SCRT2
UNC5C
NRP2
ALKBH1

Symbol
CDK5R2
NAV1
ARHGEF2
DCLK1
DNER
ADGRG1
PLAA
KIF20B
CELSR1
SEMA3E
KIAA0319
LRIG2
KIF1B
TMOD2
PTEN
DLGAP2
HDAC6
CDH4
OLFM1
SLC9A6
SEMA6C
SEMA6B
SEMA4F
SEMA4D
SEMA4B
SEMA3C
TWF2
RNF157
ZFYVE27
NRN1L
PRICKLE1
TTL
CPNE9
DSCAM
MEGF8
EMX1
CTTN
ALCAM
SEMA3D
RUFY3
RAB21
CYFIP1
NEDD4L
FN1
ADNP
MACF1
FLRT3
FLRT1
SIN3A
GDI1
CYFIP2
GOLGA4
ANAPC2
HSP90AB1
OSTN
IFRD1
APOE
ITGA4
DRAXIN

Symbol
L1CAM
LAMB2
ARHGAP4
LIMK1
SYT14P1
MAG
EIF2AK4
MT3
ITSN2
CDKL3
NRN1
TRPV2
SYT17
BCL11A
SEMA5B
POU4F2
POU4F3
SEMA4C
IMPACT
PRKCZ
SEMA3G
PAK6
RTN4
SMURF1
DIP2B
ISLR2
CPNE5
SEMA4G
PTPRS
CACNG7
RNF6
RYK
SEMA3F
SEMA4A
RAPH1
RTN4R
SLIT1
SLIT3
BMPR2
AURKA
STK11
TRPC5
VCL
DDR1
SEMA3B
MUL1
SEMA6D
ST8SIA2
TSC22D4
SYT3
LLPH
RASAL1
SEMA7A
SSNA1
IQGAP1
CPNE1
SEMA5A
SPAG9
PLXNA4

Symbol
MAP3K13
SLIT2
LHX2
CPNE6
SPAG6
SLC23A2
CDH1
KCNMB2
FGF12
FMR1
GBA1
KCNMB3
KCNMB4
GPER1
ANK3
HCN1
KCNA1
KCNA2
NPR2
GPR88
GJD2
MYH14
CLCN1
CLDN19
FKBP1B
SCN1B
CACNB4
CNTNAP1
CLU
MACO1
TENM1
PROKR1
NXPH4
NXPH3
NXPH2
PTGDR2
SORCS1
GPR139
PROKR2
CPE
AGRP
SORCS3
CRCP
PCSK1N
CMKLR2
GPR171
GPR149
NPPA
NPPB
NPY6R
GPR143
GPR84
SORCS2
PROK2
PYY3
SCG5
SORL1
ECEL1
CARTPT

Symbol
MOXD2P
DBH
ALDH9A1
MOXD1
PAH
SLC44A4
PRIMA1
HNMT
BCHE
ALDH5A1
COLQ
SLITRK3
SLC7A11
FRRS1L
LHFPL4
SHISA6
ZDHHC2
SHISA7
CHRDL1
CLN3
AGTPBP1
GCHFR
CACNG3
CACNG2
GSG1L
EFNB2
ARC
SCRIB
CACNG5
CACNG4
HPCA
ITGB3
OPHN1
VAC14
RALA
CACNG8
ATAD1
NUMB
GPC6
ADAM10
DAG1
C1QL2
DLG1
ERBB2
ERBB4
GPC4
IQSEC2
C1QL3
NPTX1
RAPSN
NETO2
NETO1
OGT
LGI1
TAMALIN
CLSTN1
GRIP1
NSG1
VPS35

Symbol
GRIPAP1
LRRC7
GRIP2
RAB11A
AP3D1
ARHGAP44
SLC18B1
TOR1A
SLC29A4
GDNF
GPM6B
RAB3B
SLC22A1
SLC22A3
SLC22A2
CSPG5
PRRT2
SNAP47
TPRG1L
STXBP5
SYT9
CTBP2
RPH3A
SV2C
PIP5K1C
SNAPIN
PNKD
ERC2
BLOC1S6
MYOF
PCLO
GIT1
NR4A1
SYT10
ASIC1
STX19
PDZD11
PFN2
LIN7C
PPT1
MCTP2
VPS18
PTPRN2
NAPB
LIN7B
RAP1BL
SNCG
STXBP2
STXBP3
SYN1
SYN2
SYP
SLC30A1
MCTP1
FER1L4
DNAJC5
SYN3
DYSF
BRSK1

Symbol
DOC2A
FBXL20
PPFIA3
CASK
CADPS
STX11
SNAP23
LIN7A
SYNJ1
BAIAP3
FER1L5
SYT12
SNAP29
CADPS2
OTOF
SLC4A8
RPH3AL
PREPL
SNPH
SV2B
SV2A
FLOT1
SLC29A1
GFAP
SLC6A16
SLC29A2
ICA1
SLC6A18
SLC6A17
SLC6A15
SLC6A6
SLC6A8
SYNGR3
SLC28A2
FURIN
PCSK6
SPRY1
SPRY2
CORO1A
ZDHHC17
DOK5
AKT1S1
BCAR1
MAGI2
OLFM3
CPT1C
CNIH3
DLG3
EPS8
PTK2B
CNIH2
ABHD12
VWC2
VWC2L
SHISA8
PORCN
SHISA9
OLFM2
CLCF1

Symbol
OSMR
CRLF1
SNTG2
MRGPRX2
ASIP
GPR151
MRAP2
PMCHL2
MRAP
NIPSNAP1
CHRFAM7A
ZACN
ANXA9
SPDYE11
LYPD1
FNTA
GPIHBP1
LY6E
LY6H
LY6G6D
TMEM35A
A2M
EFNA5
EMB
EFNB3
EPHA3
EPHA4
EPHB2
EPHB3
EXT1
PALLD
PLXND1
GAP43
CNTNAP2
TNFRSF21
CNTN6
ARHGAP35
OPCML
NTM
PCDH12
ROBO4
CRTAC1
DSCAML1
AMIGO1
IGSF9
ROBO1
ROBO2
ROBO3
YWHAZ
MYPN
NEXN
BACE1
CALB1
CALB2
DLG2
DLX1
DLX2
EGR1
EGR2

Symbol
EGR3
ENO2
FABP7
FOSB
INHBA
JUNB
MCM2
MT-CO2
NCAM1
NES
NEUROD1
NPTX2
NRGN
PLAT
PLK2
PPP1R9B
PROX1
RBFOX3
RHEB
RGS2
SHARPIN
SOX10
SOX2
SOX9
ARTN
NRTN
PTN
SDC3
CXCL16
CXCR6
CCR7
PLXNB1
PLXNA2
ICAM5
CLDN1
CLDN4
LAMA3
ITGA6
ITGB4
BSG
SCN2B
BYSL
VCAM1
MMP13
MMP1
MMP11
SNAI2
MIF
HMGA2
AMACR
NANOG
PDPN
EPAS1
SDC2
SDC1
SHH
MUC1
MKI67
KIF14

Symbol
MALT1
CAV1
PIM2
DAD1
CORO1C
CCND1
CLDN3
PKM
LGALS1
KLF14
SCG2
APOC1
ADAM30
MCAM
COL4A3
COL4A6
NLRC4
CCNB3
SOX5
SOX11
NOTCH4
MAGEA2
MAGEA4
MAGEA6
MAGEA9
MMP7
KRT4
KRT6B
GAL3ST4
TMEM140
TXNIP
SCPEP1
MYOD1
MYF5
MYF6
MYOG
CXCR2
CCNB2
CDK1
CDKN3
RRM2
TOP2A
MAD2L1
TTK
BUB1B
KIF11
NUSAP1
PCLAF
CX3CR1
CCR2
CCL2
MMP2
MMP9
CCL5
CCR5
CXCL13
CXCR5
CD74
MAGEA3

Symbol
CXCL8
CCNB1

Supplementary Table 2: Summary table of DEGs.

GeneSymbol	log2FoldChange	pvalue	padj	change
A1CF	-1.30057547521537	1.29900127007762e-17	5.07505252767818e-17	DOWN
A2M	-1.3397581559308	5.36014731830962e-23	2.82238419273437e-22	DOWN
A2ML1	2.09632431863196	1.35774735201108e-08	3.04083017885131e-08	UP
AADAC	2.55571499325222	2.84274755068153e-18	1.15252067753242e-17	UP
AADACL2	-5.20207977528081	1.98186655628348e-46	3.37673604154248e-45	DOWN
AADAT	1.0411602271502	1.44935919940486e-15	5.01245118952073e-15	UP
AANAT	1.41923852320242	1.43506873477247e-10	3.68512783934421e-10	UP
AARD	-2.53380756742574	1.99817689357563e-29	1.46749279830588e-28	DOWN
AARSD1	1.49303793771693	1.00703252398309e-28	7.11666288154125e-28	UP
ABCA10	-1.7170365290761	1.00913150434486e-21	4.94429006335316e-21	DOWN
ABCA12	3.47986148941056	1.27653988618032e-22	6.58228872906658e-22	UP
ABCA5	-1.3354832929957	1.09897493259588e-24	6.35129455734883e-24	DOWN
ABCA6	-2.75318884037288	1.81516688969409e-40	2.3378329179174e-39	DOWN
ABCA8	-4.64131174834857	5.45490874548359e-75	3.51801207352244e-73	DOWN
ABCA9	-3.1507192820103	1.79379629941867e-46	3.06529685591534e-45	DOWN
ABCB1	-1.75503473888943	5.8601572087271e-16	2.07488648791308e-15	DOWN
ABCB11	-5.35307051611933	1.78585194209934e-94	2.90626540820334e-92	DOWN
ABCB5	-3.62451353894544	1.02347767792426e-24	5.9228370906265e-24	DOWN
ABCB6	2.93083903097324	6.91796915150914e-48	1.25220994631215e-46	UP
ABCC1	1.0239746678434	4.83615071499565e-35	4.74166060812045e-34	UP
ABCC2	3.45052153405618	4.72575340392002e-39	5.6324123218658e-38	UP
ABCC8	-2.06893138409995	1.45281107872747e-15	5.02339144437679e-15	DOWN
ABCC9	-1.3429297065245	7.71526745601641e-12	2.15194541424978e-11	DOWN
ABCD2	-2.14265526843642	1.26121103161294e-27	8.47605854630492e-27	DOWN
ABCD3	-1.11522732843796	9.00743939044039e-49	1.71792488615267e-47	DOWN
ABCG2	-4.69606556091325	4.1954818857528e-105	9.3661443687966e-103	DOWN
ABCG5	1.35434545902677	4.13200388211007e-08	8.97139446373849e-08	UP
ABHD1	1.15983792871845	3.39565987740121e-12	9.65838377085713e-12	UP
ABHD3	-1.95478505186857	2.4583624304994e-70	1.28166062881096e-68	DOWN
ABHD5	-1.20732928173621	7.51377462350879e-62	2.65390177523648e-60	DOWN
ABI3BP	-3.28432061570518	2.78456855235359e-59	8.76811793890291e-58	DOWN
ABLIM2	1.13856242002313	3.42450650607412e-17	1.30769587259361e-16	UP
AC004233.4	1.83604451684727	6.87169165695773e-06	1.26406895016485e-05	UP
AC004687.2	2.71242681886736	3.19487913207389e-45	5.09454490172186e-44	UP
AC004805.1	2.47832209257897	2.29701470970696e-09	5.41513011403927e-09	UP
AC005324.2	2.22206086596213	5.82521001314353e-12	1.63366696664307e-11	UP
AC008397.1	1.46227734250835	2.46287766086997e-06	4.69315919333868e-06	UP
AC008676.3	-1.88437288096115	1.19544444944068e-14	3.92531049907485e-14	DOWN
AC008687.4	2.22215877328709	4.73312996985482e-14	1.50096507312114e-13	UP
AC010422.8	1.28619735282404	8.70166601560913e-20	3.84573884085791e-19	UP
AC010980.1	-1.77818635532714	6.07032202708585e-14	1.91351407417896e-13	DOWN
AC012651.1	1.42458321180568	1.36583235713326e-07	2.85924967958181e-07	UP
AC013394.1	1.82169322037969	4.62982343583032e-17	1.75182780287078e-16	UP
AC013477.1	1.0458236553282	7.23462373588157e-05	0.000121598941228674	UP
AC022415.2	-1.1549178138021	1.0537097497629e-10	2.72998778048227e-10	DOWN
AC055839.2	-1.19823943443244	2.87083550749961e-13	8.72880368292137e-13	DOWN
AC068831.6	1.46279504813812	8.70983825803242e-29	6.17274780574353e-28	UP
AC068946.2	1.87901838317284	3.05678584124505e-08	6.69616453058247e-08	UP
AC069503.2	1.56536413309606	8.95646458830126e-07	1.76723986261858e-06	UP
AC092042.3	-1.37326857942201	6.47958814218688e-07	1.29198520920531e-06	DOWN
AC092718.8	1.43584347215109	1.69881519535256e-15	5.85308052961499e-15	UP
AC093512.2	1.17158994953976	5.82674433343285e-22	2.8939275264708e-21	UP
AC104304.2	3.14737395513855	2.99454557462886e-20	1.35968766860528e-19	UP
AC108488.2	1.62137977902645	1.29596263025468e-09	3.11650286985564e-09	UP
AC119396.1	-2.06723522079655	2.06650209319994e-29	1.51639279177794e-28	DOWN
AC136428.1	-2.56180686896369	9.70398345499465e-17	3.59522263620898e-16	DOWN
AC138969.1	1.10014870120636	2.57052119428611e-05	4.502161089932e-05	UP
ACAA2	-1.54771358554682	2.33623145706705e-44	3.56224153782037e-43	DOWN

GeneSymbol	log2FoldChange	pvalue	padj	change
ACACB	-1.8581521952199	3.92551624315436e-59	1.22062525610798e-57	DOWN
ACADL	-1.83189161053521	1.89461183543894e-06	3.64741579773336e-06	DOWN
ACADM	-1.36395462948058	4.00192945374294e-39	4.78938814969249e-38	DOWN
ACADS	-2.10347731637977	5.31569412113142e-70	2.75482683723992e-68	DOWN
ACAN	3.21626565859251	1.06827781442393e-88	1.29180010990027e-86	UP
ACAT1	-1.2261937537287	6.11655886961046e-39	7.22575573924878e-38	DOWN
ACBD7	2.00687290947728	2.95028695403854e-38	3.38205047601535e-37	UP
ACD	1.04599796815283	2.30246297259573e-37	2.52951342219618e-36	UP
ACKR1	-2.21751291146675	2.0113671120919e-20	9.20955443672265e-20	DOWN
ACKR2	-2.39123390803799	1.94016363637861e-48	3.64445193098822e-47	DOWN
ACKR4	-1.64516746781796	1.52608622212382e-12	4.43709123156487e-12	DOWN
ACO2	-1.00904546434597	6.27629770350243e-33	5.54485905180557e-32	DOWN
ACOD1	1.32358735258609	0.000925161931589259	0.00140869576029764	UP
ACOX1	-1.53505676521259	4.32900165222888e-79	3.39553629595773e-77	DOWN
ACP4	1.32205664812805	5.34039561190217e-10	1.3179182084758e-09	UP
ACP7	2.84286472024068	3.03258366057377e-08	6.6448193383127e-08	UP
ACRV1	1.0294735832896	9.215279775499e-14	2.87675989119333e-13	UP
ACSBG1	-1.21959408532883	2.0785385976854e-21	1.00287039627309e-20	DOWN
ACSF2	-1.35899811384096	1.25785396035245e-26	8.04075294112233e-26	DOWN
ACSL6	4.29618052778862	5.72615030052669e-50	1.15806568156877e-48	UP
ACSM1	-1.12900265018113	3.42706418987644e-10	8.56298877002705e-10	DOWN
ACSM5	-2.68204650918158	1.24536242236184e-28	8.75120898328764e-28	DOWN
ACTA2	-1.39667144164838	1.22101964553985e-15	4.24007459983402e-15	DOWN
ACTBL2	3.14407920730828	5.09454541779882e-10	1.25956722078845e-09	UP
ACTG2	-4.14186948558895	1.13846838934538e-52	2.63619016804136e-51	DOWN
ACTL10	1.2277142809067	6.12370010340755e-16	2.1637984963603e-15	UP
ACTL6B	-3.03556449605635	2.87268240376691e-20	1.30639902577157e-19	DOWN
ACTL8	1.52559408025923	8.2840667632591e-08	1.75636739983722e-07	UP
ACTR3B	1.26022856566768	6.1555499212467e-60	1.99602589904411e-58	UP
ACTR5	1.08220942143455	9.53909105667278e-46	1.56702068462116e-44	UP
ACVR1C	-1.3545236567448	1.04660674000708e-15	3.64783089746662e-15	DOWN
ACVRL1	-1.542886806612	1.28354294903503e-35	1.29492082106298e-34	DOWN
ACY1	2.63981978690399	9.29798969133921e-33	8.14415968286166e-32	UP
ADA	1.18505586933681	4.12306645099906e-17	1.56484211227652e-16	UP
ADA2	-1.45023256931061	6.42656556552467e-18	2.55465265063209e-17	DOWN
ADAM11	-1.38363665920938	1.37108093287703e-11	3.75919261284644e-11	DOWN
ADAM12	3.94889893562116	1.56158254983268e-65	6.64837089003335e-64	UP
ADAM2	2.53519343894021	0.000255539208303593	0.000409431747717195	UP
ADAM23	-1.07239636594326	7.03951009224768e-09	1.61033879711388e-08	DOWN
ADAM28	-1.47483461405555	9.77573724928468e-18	3.8503712445554e-17	DOWN
ADAM33	-2.14648205757421	5.02960658736506e-25	2.95480902516153e-24	DOWN
ADAM8	1.16333842527251	1.06759605566896e-11	2.95173866582465e-11	UP
ADAMDEC1	-3.66964177198425	1.76377726853119e-82	1.62500812576368e-80	DOWN
ADAMTS1	-2.06233550898697	3.08003083416886e-38	3.52613917918358e-37	DOWN
ADAMTS12	3.2942032024843	4.66539518198013e-78	3.53210983929652e-76	UP
ADAMTS14	1.28483782954814	1.00688675366876e-19	4.4331021597052e-19	UP
ADAMTS18	1.44551134790795	6.04179906660665e-09	1.38757382150912e-08	UP
ADAMTS2	2.3554895591567	1.08448179843627e-37	1.21051804847249e-36	UP
ADAMTS20	3.89709255895277	3.52343332599413e-06	6.63066513622995e-06	UP
ADAMTS6	1.96220338337892	3.30384875863699e-33	2.94874005300594e-32	UP
ADAMTS7	1.00112081150908	1.62877481560615e-12	4.72934064768214e-12	UP
ADAMTS8	-2.12984338416425	7.31344135683122e-26	4.51912542038687e-25	DOWN
ADAMTSL1	-2.14820307125297	2.72983152782971e-35	2.7069792935136e-34	DOWN
ADAMTSL2	1.42950053758685	1.19157637182737e-17	4.66687345088394e-17	UP
ADAMTSL3	-3.37937856255551	4.94186240655821e-51	1.0584581806322e-49	DOWN
ADAMTSL4	-1.35935815468581	7.09703252765177e-20	3.150156191792e-19	DOWN
ADAMTSL5	1.31172151484429	1.64937522649841e-22	8.45966739882677e-22	UP
ADAT2	1.44592160197204	1.99334624472554e-56	5.56252214093042e-55	UP
ADCY2	-2.27907349182711	7.46533094039133e-34	6.86707911595532e-33	DOWN

GeneSymbol	log2FoldChange	pvalue	padj	change
ADCY3	1.12421626907788	5.87225508954516e-30	4.42656181273809e-29	UP
ADCY5	-2.8996540439204	1.10832007902582e-44	1.72160370526999e-43	DOWN
ADCY9	-1.47262375172565	2.53777783520722e-54	6.395126692397e-53	DOWN
ADCYAP1	-1.10035411987369	2.85604997291726e-05	4.98720398901006e-05	DOWN
ADCYAP1R1	-4.41259577988799	5.34748335897462e-46	8.85972671073502e-45	DOWN
ADD2	-1.58657538505591	1.48888300710202e-09	3.56615127959664e-09	DOWN
ADGRA1	-1.48347427950194	0.000269555963655473	0.000430977687552361	DOWN
ADGRB3	-3.89024074679684	5.52506546011478e-50	1.11869726577882e-48	DOWN
ADGRD1	-1.47167822464419	4.07033703979144e-13	1.22666630103649e-12	DOWN
ADGRD2	1.8656746485468	1.63136329821933e-08	3.63678518907862e-08	UP
ADGRF2	1.43275864790061	2.3162412481483e-06	4.42438666674049e-06	UP
ADGRG1	1.38383917494457	2.59588999411653e-45	4.16610437488951e-44	UP
ADGRG4	-4.06710517366754	6.63968046857847e-13	1.97130018754232e-12	DOWN
ADGRG7	-1.47746774038948	1.6754944255062e-08	3.73229940275546e-08	DOWN
ADGRL2	-1.19994773442533	5.64145806671693e-16	1.99907833365368e-15	DOWN
ADGRL3	-2.55246864711544	8.22694280299329e-44	1.22545556055194e-42	DOWN
ADH1A	-2.8860993844218	1.17519420609523e-19	5.15457347877489e-19	DOWN
ADH1B	-4.6129583672991	4.53308281126432e-44	6.798843324078e-43	DOWN
ADH1C	-3.97102293406729	5.99955933962294e-45	9.4117411514283e-44	DOWN
ADH6	-1.39300196231235	5.23598351814789e-11	1.38247431389703e-10	DOWN
ADHFE1	-2.55095884365691	3.35503706486409e-58	9.98654024110743e-57	DOWN
ADIPOQ	-1.97940135998511	0.0134490521607058	0.0181106136628545	DOWN
ADORA3	-2.17169465253994	1.28705122274288e-38	1.50109999608987e-37	DOWN
ADRA1A	-3.06860875698294	5.11181263998508e-36	5.26075611702483e-35	DOWN
ADRA1B	-1.44981199386384	1.22400367070125e-11	3.369087790784e-11	DOWN
ADRA1D	-1.25931743280522	2.73538786576415e-07	5.59446898127215e-07	DOWN
ADRA2A	-1.68706969316044	2.24631828844643e-15	7.67716199346767e-15	DOWN
ADRA2C	1.97783347924228	1.26314261490836e-15	4.38236747427761e-15	UP
ADRB1	-1.10932579161559	4.18151289141639e-06	7.82847908592986e-06	DOWN
ADRB2	-2.0465293603402	3.01982917875063e-32	2.57010193008723e-31	DOWN
ADRB3	-2.69817720091764	1.40341996663392e-15	4.85646897436335e-15	DOWN
ADRM1	1.37582119430549	4.04489665321225e-34	3.76449948810181e-33	UP
ADTRP	-3.03602964366093	2.08672660336751e-33	1.88661320583793e-32	DOWN
AEN	1.32105816971232	3.40267254114081e-49	6.61280546416126e-48	UP
AFF2	-1.9091827889881	4.15185099906862e-13	1.25079898696855e-12	DOWN
AFF3	-3.18932640770727	1.8599369651926e-58	5.58397971981014e-57	DOWN
AFP	4.06716412361829	6.06127678231805e-23	3.18194189359374e-22	UP
AGAP3	1.11644646592067	1.31223073176265e-40	1.70394285847749e-39	UP
AGFG2	-1.06656667210264	3.14764230134638e-27	2.07928282979304e-26	DOWN
AGR3	-1.41560973140947	1.34522659979327e-09	3.23184751410047e-09	DOWN
AGRN	1.12688033482264	5.97791232949414e-22	2.96562357246386e-21	UP
AGT	2.16004708039496	5.26309476031902e-36	5.41005130232793e-35	UP
AGTR1	-3.74283040031277	1.33500391653942e-30	1.03225680278423e-29	DOWN
AGTRAP	1.08797608795032	1.35440705380448e-29	1.00103098590396e-28	UP
AHCY	1.7489177819412	1.07847482033193e-47	1.94404576050103e-46	UP
AHCYL2	-2.69487889415869	6.55323718578602e-85	6.75216089444331e-83	DOWN
AHNAK	-1.78942548092057	9.06055282040739e-40	1.12533100044047e-38	DOWN
AHNAK2	-1.21826786855733	6.02366492315883e-08	1.29222714435093e-07	DOWN
AHSG	5.12865182897874	3.42589446331158e-28	2.35604661491487e-27	UP
AICDA	-3.33100779051063	3.0793530738896e-15	1.04625902586614e-14	DOWN
AIF1	-1.17815895423998	3.30387452890145e-16	1.18619313756208e-15	DOWN
AIRE	1.65086569764602	1.16988365959459e-07	2.4590999715742e-07	UP
AJUBA	2.95436056487998	1.71050862829565e-210	2.97850867445122e-207	UP
AK5	-1.46388492877102	2.51601849669517e-10	6.34396612843223e-10	DOWN
AKAP12	-1.8597900734727	5.71725702021988e-18	2.28126939718352e-17	DOWN
AKAP14	1.72222890264297	2.7764214642763e-07	5.67572516523164e-07	UP
AKAP4	4.74491650227818	4.13922200348535e-22	2.07115726283593e-21	UP
AKAP5	-1.52727617050854	3.47042939205248e-21	1.65563252065232e-20	DOWN
AKAP6	-2.38552515352803	2.01633435900507e-52	4.62588013087685e-51	DOWN

GeneSymbol	log2FoldChange	pvalue	padj	change
AKAP7	-1.25398534299391	2.26440728667545e-14	7.30187483016288e-14	DOWN
AKAP9	-1.21721741083847	1.79655067505287e-29	1.32164498963648e-28	DOWN
AKNAD1	1.62538026856602	2.07544366463401e-14	6.70495371656252e-14	UP
AKR1B10	-3.09232753842225	2.43632170991323e-34	2.29193246540891e-33	DOWN
AKR1B15	-1.0770046928897	0.00121666585078616	0.00183252335089867	DOWN
AKR1C1	-1.04048345748467	3.23408162396804e-07	6.58270757664002e-07	DOWN
AKR1C2	-1.65401141553476	9.66213272656397e-17	3.58047919062904e-16	DOWN
AKR1C4	6.12945477422024	1.21342219276516e-55	3.2606976300339e-54	UP
AKR1C8P	3.61685793077896	1.29288213849068e-16	4.76666455167019e-16	UP
AKR1E2	1.42204050550205	1.2270510558604e-14	4.02612399391316e-14	UP
AKT3	-1.24106755635252	5.653553892605e-15	1.89500161562908e-14	DOWN
AL022238.3	1.4671562358588	2.77607819341492e-05	4.85291131231142e-05	UP
AL031708.1	1.35143067338891	1.01555475376729e-10	2.6350551225376e-10	UP
AL049839.2	-2.1117231363666	2.83950754715707e-08	6.23431407371656e-08	DOWN
AL117339.4	1.05845647324007	4.61260571604673e-09	1.06821789245274e-08	UP
AL121845.3	3.66944519121747	7.2353064414618e-16	2.54316493873989e-15	UP
AL135905.2	1.27489579137005	8.00068918830215e-06	1.46248163800027e-05	UP
AL136531.2	-1.86206985945466	2.36547341677722e-19	1.02183052856219e-18	DOWN
AL139260.3	-1.51516574524465	1.95468874557969e-12	5.64835631045125e-12	DOWN
AL159163.1	1.61862500478787	1.0555854164275e-05	1.90792078640773e-05	UP
AL353588.1	1.18033231793145	5.90297265510793e-07	1.18012012449362e-06	UP
AL365205.1	1.1388295043016	2.5078356409146e-13	7.6585306936594e-13	UP
AL391987.2	1.00916444335127	3.11337357924161e-06	5.88569907016982e-06	UP
AL441992.2	1.67410005908596	3.42041367799103e-07	6.94654343070421e-07	UP
ALAS2	-1.4705023672794	4.84451321078356e-08	1.04648937525585e-07	DOWN
ALB	3.99814844916751	6.15544161835144e-14	1.93929265243991e-13	UP
ALDH1A1	-1.09069517355692	1.00966533224742e-06	1.98367397387164e-06	DOWN
ALDH3B2	3.76981736958883	3.31217837537631e-43	4.79027924006875e-42	UP
ALDH4A1	1.86816078315778	8.90483407809395e-39	1.0448778692847e-37	UP
ALDOB	1.30874998703995	2.3543346871956e-06	4.49419314932437e-06	UP
ALG1L	1.61338212390241	1.08712376288387e-23	5.96035456016905e-23	UP
ALG3	1.07422545534534	1.24049318091476e-30	9.6216961065785e-30	UP
ALK	-2.37162861392215	3.85751686936811e-11	1.02676461703312e-10	DOWN
ALKAL1	3.57843259925911	5.69428932928742e-53	1.32915093955606e-51	UP
ALKAL2	-2.10779638276343	3.2837070865894e-25	1.94818369672167e-24	DOWN
ALKBH2	1.22080519306084	9.97484492764484e-52	2.19031493978663e-50	UP
ALKBH6	1.24768701303045	4.84221985290007e-27	3.16864240129835e-26	UP
ALOXE3	2.93343443411018	4.15027337240759e-22	2.0760904979527e-21	UP
ALPI	-4.30286945682158	2.54054284031574e-59	8.01421602869889e-58	DOWN
ALPK3	1.54281479057405	3.21626076724431e-10	8.04665930172776e-10	UP
ALPP	2.78921488955471	3.87470429638986e-11	1.03118181129507e-10	UP
ALX1	2.91310198279111	1.56964357766911e-06	3.04062783601649e-06	UP
AMBP	1.88670213569877	4.76944196877208e-15	1.60638864607791e-14	UP
AMELX	5.47371980716057	5.52587385100327e-41	7.28403038361241e-40	UP
AMER2	-3.489155877391	5.37619500853598e-24	2.99570187787639e-23	DOWN
AMER3	-1.09438050673629	0.00870369245546803	0.0119656874093688	DOWN
AMH	4.26243453561759	3.95738839327554e-47	6.98176333253364e-46	UP
AMHR2	-1.58774346366811	3.71368977116945e-10	9.26321157217785e-10	DOWN
AMN	-1.39789171564805	6.91797458372387e-14	2.17363210801847e-13	DOWN
AMOTL1	-2.04481529431156	8.95223390073973e-32	7.43018345631939e-31	DOWN
AMPD1	-4.04164225963397	4.09993025826009e-55	1.07034611075087e-53	DOWN
AMPD2	1.17059849135287	1.13252082565755e-55	3.05272215745742e-54	UP
AMPH	-1.1425102687525	1.63290479549393e-11	4.44903320355746e-11	DOWN
ANAPC11	1.00068852119282	4.43760688350221e-12	1.25400922853658e-11	UP
ANGPT1	-1.65293622871179	4.94326876169631e-22	2.46427537782473e-21	DOWN
ANGPT2	1.36906715881858	4.93280745705685e-30	3.73456418477091e-29	UP
ANGPTL1	-4.42612578444648	5.34409855439313e-52	1.19610267516256e-50	DOWN
ANGPTL5	-3.81210306444823	3.48184674361906e-52	7.87394770735567e-51	DOWN
ANGPTL7	-5.06363027438573	6.43341621633404e-50	1.29959485585875e-48	DOWN

GeneSymbol	log2FoldChange	pvalue	padj	change
ANK2	-3.31007086628517	1.19084591887211e-61	4.16389557938153e-60	DOWN
ANK3	-1.72247888359468	1.75701657029093e-73	1.06602541945909e-71	DOWN
ANKK1	1.14624257192122	2.91023817359305e-07	5.94160831478201e-07	UP
ANKLE1	1.00577002852331	2.55739220491947e-06	4.86687108899046e-06	UP
ANKRD1	2.22084932192535	2.59493376747507e-10	6.53442974592097e-10	UP
ANKRD12	-1.03535066358545	2.69825835232983e-20	1.22836007030377e-19	DOWN
ANKRD13B	2.40619313292116	5.66081260334137e-76	3.86555803380327e-74	UP
ANKRD18A	1.3231873474834	1.3361948848185e-14	4.37188303820829e-14	UP
ANKRD18B	3.07237915117843	4.26926740269733e-14	1.35683068594942e-13	UP
ANKRD20A1	-1.63419713523875	1.58477083230458e-09	3.78592598476054e-09	DOWN
ANKRD20A4P	-1.70498347722558	3.34506034231058e-08	7.30743140643008e-08	DOWN
ANKRD31	1.05117041267714	2.62147592849153e-07	5.36906143764092e-07	UP
ANKRD33	2.92067005112422	7.18972944027678e-14	2.25616793554766e-13	UP
ANKRD33B	-1.93068940942036	9.47390382386507e-30	7.07414610998982e-29	DOWN
ANKRD35	-1.93335655112385	8.60467628994495e-36	8.75194090168291e-35	DOWN
ANKRD36C	1.02340966878916	1.48489415925047e-07	3.098437626726e-07	UP
ANKRD39	1.04834236316987	2.94064880277654e-24	1.66305675877713e-23	UP
ANKRD40CL	-1.57330988366272	4.94193643547894e-23	2.6061156617503e-22	DOWN
ANKRD44	-1.07629556009694	1.30181719113408e-20	6.02406132054684e-20	DOWN
ANKRD53	-1.61829933469972	4.07178230219961e-26	2.54402386897029e-25	DOWN
ANKRD55	-1.0736698501796	4.23967394384079e-07	8.55649540844919e-07	DOWN
ANKRD65	-1.61729314643758	1.10782659318535e-18	4.58971793174791e-18	DOWN
ANKS1B	-3.43556926465957	6.12096096970526e-54	1.5096925405875e-52	DOWN
ANKS6	1.05906449200647	3.06099630379543e-45	4.88552966434371e-44	UP
ANKZF1	1.00098728254625	2.66895956035615e-50	5.5326896219621e-49	UP
ANLN	2.00642966495462	4.67202055103892e-83	4.44556797023173e-81	UP
ANO1	1.04657720882122	6.56972948664249e-08	1.40469915951505e-07	UP
ANO10	-1.07155650055843	1.14587186770817e-46	1.97750910132829e-45	DOWN
ANO3	-1.98737728933313	7.53474811559538e-08	1.60237626938034e-07	DOWN
ANO4	-1.17697482143014	7.18491928988073e-09	1.64230768698731e-08	DOWN
ANO5	-3.21272575837733	1.20355930037583e-32	1.04787890487221e-31	DOWN
ANO9	1.26545842293432	1.88634400282265e-16	6.88758819902513e-16	UP
ANOS1	2.76539217238506	6.86389174779612e-43	9.82902524707021e-42	UP
ANP32D	1.60487108067541	1.90124820678494e-05	3.36926877923327e-05	UP
ANPEP	-3.88229711212076	2.14312722072193e-40	2.7500570592801e-39	DOWN
ANTKMT	1.03079086894683	1.73591165661419e-10	4.4250372824803e-10	UP
ANTXR2	-1.28996909623752	3.98751551002328e-27	2.62215285407989e-26	DOWN
ANXA10	2.93349976830879	3.19760699574133e-10	8.00228953964412e-10	UP
ANXA3	1.59557151677644	3.49553294374364e-39	4.19777345857986e-38	UP
ANXA8L1	2.43895740030479	6.42142152707443e-05	0.000108392400428605	UP
AOC1	-1.78024312515159	2.13442519061619e-35	2.12867960161511e-34	DOWN
AOC3	-2.777815436196	1.72802363582087e-42	2.42271139859492e-41	DOWN
AOX1	-1.88386667425058	8.98158387035702e-19	3.73974940063431e-18	DOWN
AP001453.3	1.52365345889775	2.24134183752847e-13	6.86637674470147e-13	UP
AP001931.2	2.37221721256221	1.78467191083311e-35	1.79012050595259e-34	UP
AP006621.6	1.94600928525838	7.37640826517198e-38	8.27612094854638e-37	UP
AP1S3	1.56859215276654	2.72153296428322e-42	3.78212717534428e-41	UP
AP3B2	-1.0127744454346	0.000775618109614732	0.0011898368551424	DOWN
AP3S2	-1.13653640848821	6.01844981399022e-62	2.14752595514368e-60	DOWN
APBA1	-1.31003180637982	1.68033185408538e-19	7.31307637470352e-19	DOWN
APBB1	-1.58613252784681	6.03276430139512e-25	3.53579686234241e-24	DOWN
APBB1IP	-1.28680582288701	7.87332494225476e-16	2.76240594840786e-15	DOWN
APC	-1.03652439331949	1.14206794786297e-22	5.91166146734181e-22	DOWN
APCDD1	2.51846199825161	1.51321558250648e-24	8.68191859577771e-24	UP
APCDD1L	-1.35047073722238	8.47484065918888e-07	1.67562621095102e-06	DOWN
APELA	2.89911920754249	2.87910566402478e-35	2.85013456098144e-34	UP
APLN	3.24567164572268	2.5511121978718e-90	3.29055679270679e-88	UP
APLP1	-1.26863748616963	1.83452086848128e-09	4.36400435558259e-09	DOWN
APOA2	5.19205702419173	7.59898821781138e-24	4.19534501701806e-23	UP

GeneSymbol	log2FoldChange	pvalue	padj	change
APOA5	2.31992611711299	8.71491214199905e-06	1.58886781623526e-05	UP
APOBEC2	-1.21786366993934	6.83733027578473e-10	1.67546344064508e-09	DOWN
APOBEC3A	-2.62353816312701	1.05477416315866e-31	8.72116927971592e-31	DOWN
APOBEC3B	-1.51272598945615	4.63040582185924e-25	2.72764738078603e-24	DOWN
APOBR	-1.75034133881537	1.45602937942816e-25	8.81259631003914e-25	DOWN
APOC1	1.24040445913926	1.86486500627448e-09	4.4307401220163e-09	UP
APOLD1	1.11486504703914	3.68750181365202e-07	7.47154632081947e-07	UP
APPL2	-1.85319875785846	1.01063605129225e-144	5.67684050359739e-142	DOWN
AQP11	-1.20730244470382	3.04652532532699e-30	2.32569686496795e-29	DOWN
AQP12A	-1.23680150494356	1.22250347161907e-06	2.38916419206542e-06	DOWN
AQP12B	-1.05748502096189	3.66585768964092e-05	6.33144018545103e-05	DOWN
AQP2	3.18615888682092	1.67000213393073e-12	4.84553376793396e-12	UP
AQP4	-2.64699580110684	2.11860308858573e-09	5.01035387498892e-09	DOWN
AQP5	4.88754907537289	7.09986926907358e-33	6.25025397281993e-32	UP
AQP6	1.8975686185146	1.18974325973543e-16	4.39105540096928e-16	UP
AQP7	-1.9558754770701	3.39527195515264e-28	2.33590954385906e-27	DOWN
AQP8	-6.93994537933758	3.87659133630612e-77	2.82439685937651e-75	DOWN
AQP9	2.12117684664046	2.78756251407726e-16	1.00579830206439e-15	UP
AR	-1.94972029695257	1.19938096065873e-18	4.95958695510579e-18	DOWN
AREG	1.76940638698604	4.1592745531955e-19	1.77252686722451e-18	UP
ARFGAP1	1.28014914343006	4.04821970932634e-34	3.76557958325318e-33	UP
ARG2	1.05263516837765	6.4232636547133e-15	2.14721232519721e-14	UP
ARHGAP10	-1.35703827105765	9.36989355043093e-18	3.69470009949397e-17	DOWN
ARHGAP11B	1.47072079641496	6.76168534179135e-20	3.00897589717896e-19	UP
ARHGAP15	-1.42338596357252	6.37262932784256e-27	4.14209012638009e-26	DOWN
ARHGAP20	-2.69648748117533	6.72824216229028e-47	1.17866077235373e-45	DOWN
ARHGAP25	-1.15528166268689	3.88613448510224e-18	1.56569319271368e-17	DOWN
ARHGAP28	-1.31454323419529	8.73080634852067e-18	3.44738165412223e-17	DOWN
ARHGAP30	-1.05162367536634	1.98013097654234e-12	5.71997689026739e-12	DOWN
ARHGAP33	1.05402530532741	3.96896151182308e-16	1.41825419259954e-15	UP
ARHGAP36	-2.61203051327876	3.37193663400575e-12	9.59560918580522e-12	DOWN
ARHGAP39	1.20330242052127	4.58710903231139e-29	3.29111370332255e-28	UP
ARHGAP40	4.68824331978516	2.41134968299266e-24	1.37084009239148e-23	UP
ARHGAP42	-1.18826624967852	2.27432268352111e-35	2.26172363724461e-34	DOWN
ARHGAP44	-2.15972176490418	4.76674058845685e-46	7.9353015169024e-45	DOWN
ARHGAP6	-1.00946000533519	1.52618381103976e-06	2.95940297345605e-06	DOWN
ARHGEF10	1.38651883268362	2.45921536051827e-12	7.07222412431125e-12	UP
ARHGEF19	1.43932784093456	1.05199981030713e-35	1.06379051665959e-34	UP
ARHGEF25	-2.39325675046424	4.00217290995014e-45	6.35276544037938e-44	DOWN
ARHGEF26	-2.00646330526428	2.68605771966947e-47	4.7678209044449e-46	DOWN
ARHGEF37	-1.60958955160852	6.83726990414226e-45	1.07162358992645e-43	DOWN
ARHGEF39	1.3053084833675	5.53630756736516e-41	7.29226351516864e-40	UP
ARHGEF6	-1.3521870073687	4.34497941808686e-23	2.30106832746796e-22	DOWN
ARID3A	2.24749973185907	8.19760430254603e-44	1.22213085376913e-42	UP
ARID3C	1.05562435821211	1.01718036798777e-06	1.99776243489409e-06	UP
ARL14	-2.51688252584213	3.58692953600617e-59	1.11733817550046e-57	DOWN
ARL2BP	1.32200793611162	1.32860427889968e-08	2.9774757153771e-08	UP
ARL4D	-2.35480182177866	2.26813175875241e-48	4.23765861750597e-47	DOWN
ARL6IP4	1.39014419612977	1.49926418609981e-17	5.83389659721921e-17	UP
ARMC2	1.29809622913973	6.22097221617158e-20	2.77758433846656e-19	UP
ARMC3	2.1717398383805	3.10388762054695e-11	8.31379712914691e-11	UP
ARMC4	-1.06892561345293	4.31918516773061e-05	7.41350136280859e-05	DOWN
ARMCX1	-2.01813278084754	4.61620966312669e-41	6.09416670690107e-40	DOWN
ARNTL2	2.17029934927825	7.5268778739536e-69	3.71290437448029e-67	UP
ARPC1B	1.28330474155757	2.49215781668537e-18	1.01439794441193e-17	UP
ARPP21	-3.72292679542118	7.20086205298864e-29	5.12208377976679e-28	DOWN
ARRB1	-1.1142124300029	2.43543114405692e-38	2.80106753708475e-37	DOWN
ARRDC4	-1.75557459642227	4.31283130793244e-54	1.07284759378611e-52	DOWN
ARSH	1.81601378709323	2.98560271149603e-14	9.57250966954159e-14	UP

GeneSymbol	log2FoldChange	pvalue	padj	change
ART1	-1.86156096504285	1.70174578832938e-06	3.28811578031286e-06	DOWN
ART4	-1.08006672877616	0.000736739865071925	0.00113309055559949	DOWN
ART5	1.0159181398486	4.69563114312837e-05	8.02956153346699e-05	UP
ARVCF	1.05061351226727	2.33485976210924e-21	1.12280897645977e-20	UP
ASAP3	-1.8584841272491	4.27766206786221e-55	1.11507379622282e-53	DOWN
ASB11	-3.23251107897272	1.26546433130903e-30	9.80228220688794e-30	DOWN
ASB2	-2.99736055001447	1.86777471985874e-56	5.22047531250406e-55	DOWN
ASB4	1.96949935455752	1.94362077163533e-09	4.60967971894389e-09	UP
ASB5	-4.85460462538619	2.70147338374381e-20	1.22950224859203e-19	DOWN
ASB9	2.11705890563869	1.53155098073345e-37	1.69757461664618e-36	UP
ASCL1	-1.68895395226976	1.41966784717452e-05	2.54327944679526e-05	DOWN
ASCL2	4.119598922434	1.72476308739671e-93	2.73029996734899e-91	UP
ASCL5	4.10608246715882	3.14513919661762e-65	1.3228577012247e-63	UP
ASF1B	1.05723763272076	1.28258719770603e-30	9.92608483273563e-30	UP
ASGR1	2.6566261251675	3.34432167301308e-37	3.63966708076104e-36	UP
ASIC1	1.72167140394034	1.02722681468705e-35	1.04176473640918e-34	UP
ASIC3	1.15004698083151	1.08194424597503e-09	2.61701557926978e-09	UP
ASIC4	1.79603934588623	2.02069183605226e-10	5.12397072101033e-10	UP
ASIP	1.3753254759484	4.05661611559028e-12	1.14858303123209e-11	UP
ASNS	1.64625302935745	9.37851694358648e-28	6.34452663320402e-27	UP
ASPA	-4.03768596503788	9.70943391699288e-93	1.45750321376377e-90	DOWN
ASPG	-2.84072289247939	2.93588365992735e-29	2.1327718886239e-28	DOWN
ASPHD1	2.44383410161413	8.17829886940688e-48	1.4788028890237e-46	UP
ASPM	1.30535331896232	5.74555507232855e-28	3.92188751369883e-27	UP
ASTN1	-3.61409839346693	2.07940897935249e-25	1.24514266016041e-24	DOWN
ASXL3	-2.25050254902633	9.88195965237886e-23	5.13348936237688e-22	DOWN
ATAD2	1.5503002597519	1.56458777931492e-49	3.08540962641117e-48	UP
ATAD3A	1.21274806829331	5.10807410887389e-29	3.65735585764067e-28	UP
ATAD3B	1.35509205072708	7.01336093126215e-33	6.1772207332356e-32	UP
ATAD3C	2.06010063741829	1.68533879176235e-20	7.74730844270269e-20	UP
ATAD5	1.054284581363	8.06311321632156e-25	4.69889526224254e-24	UP
ATAT1	1.03350953055569	6.55311368114308e-31	5.15865138018736e-30	UP
ATCAY	-3.24532852219802	2.68754004090523e-28	1.85706883858265e-27	DOWN
ATG9B	3.59913925738534	1.13719426519807e-89	1.43492490868797e-87	UP
ATIC	1.04538075018905	1.20798780487636e-60	4.01425413097558e-59	UP
ATL1	-1.24604949047123	9.78710890871682e-31	7.64914395994102e-30	DOWN
ATOH1	-1.26567140653515	1.01668890205286e-05	1.8414399679058e-05	DOWN
ATP11A	2.17031401363031	1.36521868580119e-70	7.18204017397465e-69	UP
ATP13A4	-2.60558816530036	3.73483948216842e-27	2.4569233057423e-26	DOWN
ATP1A2	-5.02330440920612	8.25375615567874e-61	2.76389722959296e-59	DOWN
ATP1B2	-2.46323778697318	1.97395520780887e-45	3.20041732156199e-44	DOWN
ATP2A1	1.24969008997943	1.49605743114099e-14	4.87752257039095e-14	UP
ATP2A3	-1.60883359324821	5.16918258411667e-23	2.72347886043037e-22	DOWN
ATP2B1	-1.12825561965812	1.91928141302161e-35	1.91740948049027e-34	DOWN
ATP2B2	-2.26524237823173	2.08414843388317e-18	8.52508261198205e-18	DOWN
ATP2B3	-3.6510985746814	1.49806071660923e-35	1.50697465386e-34	DOWN
ATP2B4	-2.34001262504754	1.70282968160436e-61	5.87155905856963e-60	DOWN
ATP5MF	1.00746840395174	6.98990878289759e-13	2.07351416757403e-12	UP
ATP5MF-PTCD1	1.36883124126581	2.77550002793016e-06	5.27099814443755e-06	UP
ATP6V0A4	4.64268716437763	2.26237939465451e-24	1.28741216990585e-23	UP
ATP6V0D2	-2.14715257064594	2.29485939486082e-31	1.85345021533912e-30	DOWN
ATP6V1C2	2.69371517831246	3.96811807964496e-50	8.12904001421856e-49	UP
ATP6V1E2	1.13455982195175	1.45944008079523e-37	1.61867707814569e-36	UP
ATP6V1F	1.2364502830862	2.90095453566284e-33	2.59713734341887e-32	UP
ATP6V1FNB	1.70192941653988	1.10170481035041e-26	7.0659248112824e-26	UP
ATP6V1G2	-2.41925164929942	3.31294728439475e-65	1.39008074851002e-63	DOWN
ATP8A1	-1.41069339182628	8.98023466453907e-22	4.41232579609534e-21	DOWN
ATP8A2	-1.8725766292261	1.14546602690966e-29	8.49127285082075e-29	DOWN
ATP8B1	-1.66440077918994	5.69598133897089e-64	2.20900051348552e-62	DOWN

GeneSymbol	log2FoldChange	pvalue	padj	change
ATP8B2	-1.09893806350921	3.5654777504881e-14	1.13876859994955e-13	DOWN
ATP8B4	-1.20849726959898	5.51019871776102e-20	2.46782639589436e-19	DOWN
ATRNL1	-3.20628166619333	5.77777249030768e-35	5.64898104288196e-34	DOWN
ATXN7L2	1.04104431789882	2.27129355470185e-34	2.14247208385825e-33	UP
AUNIP	2.1753384113355	1.86943767303368e-96	3.25525182005355e-94	UP
AURKA	1.54079307581303	1.3436180288205e-61	4.66994425865296e-60	UP
AURKB	1.22856299584403	1.51376364548013e-30	1.16788508457002e-29	UP
AVPR1B	-4.00873968096916	1.30463887940116e-43	1.92685978006891e-42	DOWN
AXDND1	-1.21414627430639	9.56216045702961e-06	1.73751330520981e-05	DOWN
AXIN2	2.52615556400771	1.26490987547334e-53	3.06767070496063e-52	UP
AXL	-1.12888035087839	1.01720448875618e-14	3.35974616136405e-14	DOWN
AZGP1	3.21656207117314	1.33079608764126e-69	6.75602107116537e-68	UP
B2M	-1.09278887028177	5.5115581051247e-24	3.06916409608367e-23	DOWN
B3GALT1	-3.31713745483928	2.1204747307199e-31	1.71898633547606e-30	DOWN
B3GALT4	-1.4660150649006	1.57204408954034e-43	2.3061502722128e-42	DOWN
B3GALT5	-2.69512667254021	2.85240944457989e-27	1.88640355710101e-26	DOWN
B3GNT4	1.42245434583788	3.48641210241368e-14	1.11412908679261e-13	UP
B3GNT5	-1.05221901137494	8.58433187302579e-28	5.82536909216672e-27	DOWN
B3GNT6	-1.77365022865494	1.19623624223858e-06	2.33861700753344e-06	DOWN
B3GNT7	-3.15049488702504	2.38331274912495e-35	2.3674058699665e-34	DOWN
B3GNT8	-1.26801743097514	1.06681757909083e-18	4.42508206400871e-18	DOWN
B3GNTL1	1.55616656264461	1.02143581984407e-62	3.74447619598838e-61	UP
B4GALNT2	-4.16813974182426	1.77879346532509e-27	1.18629378060919e-26	DOWN
B4GALNT4	2.76268130704552	4.58251255087262e-23	2.42170837779499e-22	UP
B9D1	1.12700041906205	4.41587774582161e-22	2.20578540413057e-21	UP
BAALC	-2.26531912967823	2.90206335857344e-35	2.87122893538859e-34	DOWN
BAAT	2.65606854837676	1.2503183466565e-22	6.45090173935691e-22	UP
BACE2	1.19157236384375	6.30878439898145e-25	3.68764225375844e-24	UP
BACH2	-1.6760831197707	1.25003192984919e-16	4.6096581945074e-16	DOWN
BAG2	-1.1789654571617	2.93249993641882e-10	7.35575070482007e-10	DOWN
BAMBI	1.72457702850267	7.40060385447639e-18	2.93278823208915e-17	UP
BANF2	2.26354392407678	3.89929291257365e-06	7.31900263950037e-06	UP
BANK1	-1.57620364361981	4.8217366307485e-18	1.93324660260704e-17	DOWN
BARX1	1.26202227368943	0.000254160556106703	0.000407335275056237	UP
BARX2	-1.87752293687939	8.87122033187593e-16	3.10314503091514e-15	DOWN
BATF	1.12679747446388	1.74593770282799e-09	4.15839327306028e-09	UP
BCAR3	-1.43067782329873	5.55508002191951e-52	1.24172796433484e-50	DOWN
BCAS1	-2.49872976642676	3.66551495259076e-38	4.182674434434e-37	DOWN
BCHE	-4.45355318078933	7.94878658022127e-52	1.75650026296184e-50	DOWN
BCKDHA	2.35415873343198	1.25836331232238e-20	5.83383396098764e-20	UP
BCL2	-1.63418770827637	1.01911865274286e-31	8.43436934420691e-31	DOWN
BCL2L1	1.05692026201848	3.9335732336841e-29	2.83977241783339e-28	UP
BCL2L11	-1.03003106158255	9.45518012422301e-43	1.34512296979653e-41	DOWN
BCL2L12	1.18346834696831	3.48325328836482e-36	3.61680915386384e-35	UP
BCL2L15	-1.25439542458327	2.72915301700112e-19	1.17427085458464e-18	DOWN
BDKRB1	-1.48860809256736	2.34051293678935e-24	1.33100430334138e-23	DOWN
BDKRB2	-1.2053900458173	1.88207091185503e-27	1.25325050815035e-26	DOWN
BDNF	1.12324208139416	4.47889893073582e-11	1.18780181359889e-10	UP
BEAN1	2.10948859883768	1.16595160787549e-17	4.56959607200898e-17	UP
BECN2	2.08823424303472	7.93416743553259e-07	1.57193830418624e-06	UP
BEND4	-2.76812461398199	1.87289141108664e-32	1.61071190949699e-31	DOWN
BEND5	-2.58148105019472	1.3740788857898e-48	2.59228988496833e-47	DOWN
BEND6	-1.18074510701049	4.97681310244673e-09	1.15026873576991e-08	DOWN
BEST2	-3.61589906379468	2.47064568535144e-26	1.55874468547191e-25	DOWN
BEST3	3.93048969613467	1.755169062751e-24	1.00436276338098e-23	UP
BEST4	-5.81060234046928	4.69980159483161e-168	5.45584301138686e-165	DOWN
BEX1	-2.63324087336587	2.06481672770398e-16	7.52439338234604e-16	DOWN
BEX4	-1.85776150615935	2.13885088121784e-24	1.21791400898124e-23	DOWN
BFSP1	1.83463107289854	1.34415655343704e-41	1.81863232828276e-40	UP

GeneSymbol	log2FoldChange	pvalue	padj	change
BFSP2	-1.84340998135949	1.85785969531894e-16	6.78642980377359e-16	DOWN
BGLAP	1.26805902593427	4.72283264510425e-09	1.09287288836147e-08	UP
BGN	2.51903260996499	8.48554147305171e-42	1.15798380619318e-40	UP
BHLHA15	-1.04571028572315	5.25383947616718e-10	1.29710912800934e-09	DOWN
BHLHA9	5.8448958791441	2.39730886008739e-27	1.5896549573763e-26	UP
BHLHE40	1.1775690312993	6.75823605902026e-26	4.18338411533544e-25	UP
BHLHE41	-1.173908521274	8.5470507948911e-12	2.37785262009009e-11	DOWN
BHMG1	3.11451410364673	3.89269427613752e-13	1.17475711317821e-12	UP
BHMT2	-2.19946362458913	2.28062857427744e-25	1.36141876461752e-24	DOWN
BICD1	1.21506357976932	9.73395516453616e-32	8.06746126987474e-31	UP
BICDL1	1.07032833523527	1.26528958056429e-27	8.50018806572761e-27	UP
BID	1.04294912366396	1.72255951214855e-26	1.09430604834158e-25	UP
BIN2	-1.21576650035204	2.66412774711612e-29	1.93940035370121e-28	DOWN
BIRC5	1.4267838740318	1.24401868971571e-42	1.75971547067585e-41	UP
BIRC7	2.3933269224585	1.77131463283423e-20	8.12965253071754e-20	UP
BLACAT1	5.00562506651655	7.77907747984733e-151	5.01692874654006e-148	UP
BLK	-2.30579514974797	1.93507295499847e-16	7.05811172295524e-16	DOWN
BLM	1.05396723692613	2.59438693297751e-25	1.54342533870644e-24	UP
BMERB1	-1.41975337044708	1.63153607454779e-21	7.91804282778727e-21	DOWN
BMF	-1.13237348435668	3.80432857647676e-23	2.02150666775068e-22	DOWN
BMP2	-1.94346628553228	7.490435219005e-42	1.02459503903012e-40	DOWN
BMP3	-5.24009639137964	3.25631155027374e-66	1.42467721168132e-64	DOWN
BMP4	1.93472498648148	2.23599489124922e-31	1.81010595264169e-30	UP
BMP5	-2.61665155856247	3.87364462813511e-28	2.65767430692343e-27	DOWN
BMP6	-1.934303114562	2.44797829778027e-26	1.5450034831188e-25	DOWN
BMP7	3.17978861671847	5.99281071676149e-39	7.09400496335607e-38	UP
BMX	-2.13421890592327	2.12160460228146e-23	1.14164094374311e-22	DOWN
BNC2	-1.92209873368734	7.47106513660692e-22	3.68850743475294e-21	DOWN
BNIPL	1.24107298726262	1.47764315942838e-15	5.10621161641724e-15	UP
BOC	-1.58682151636261	4.27935835720825e-15	1.44523791842644e-14	DOWN
BOP1	2.01097956748571	3.64696906910364e-56	1.00165098423189e-54	UP
BPI	1.18051661391593	2.93973732877026e-06	5.57014647506817e-06	UP
BPIFA1	2.41554952480673	0.017496769266582	0.0232130471039232	UP
BPIFB1	1.9966831806996	0.000836159882831034	0.00127854338248479	UP
BPIFB2	4.59910331982147	6.6070914061737e-12	1.84966692372512e-11	UP
BPIFB4	2.04030716368923	0.00801079250107851	0.0110593776120891	UP
BRAT1	1.10833272492506	6.07513097712344e-39	7.18168742054654e-38	UP
BRCA1	1.17205381338784	1.29742399277265e-37	1.44081913176978e-36	UP
BRCA2	1.89423120887549	3.70829193616064e-57	1.06731384271678e-55	UP
BRDT	1.73975629205654	5.65638268748719e-05	9.59612156442074e-05	UP
BRICD5	1.19747387508299	4.82227728605894e-19	2.04805644834498e-18	UP
BRINP2	-1.15109059409447	0.000848619567941657	0.00129691175500861	DOWN
BRINP3	-3.29112154636271	3.19942144201505e-14	1.02410892591559e-13	DOWN
BRIP1	1.00578222759493	7.84810951080378e-21	3.67659754941152e-20	UP
BRIX1	1.19146938515675	1.91523753755152e-35	1.91446792430452e-34	UP
BRSK2	2.74098924892794	9.25412902939706e-27	5.95939899367201e-26	UP
BST1	-1.14690659189688	3.06754229871353e-12	8.75514080437611e-12	DOWN
BTBD11	1.37916862116593	5.07639976464829e-10	1.25543742510752e-09	UP
BTBD16	3.49068364463431	1.34443340886177e-69	6.80541248503196e-68	UP
BTBD19	1.08901184449582	2.68994023319204e-15	9.15915707480896e-15	UP
BTBD3	-1.16089646310665	1.89949451195205e-40	2.44282850344321e-39	DOWN
BTC	-1.25219352371918	7.50911705536953e-27	4.85721602099367e-26	DOWN
BTD	-1.14830045390991	4.27854401300151e-64	1.68176719860937e-62	DOWN
BTK	-1.62152470941571	4.32192370495641e-23	2.29025129258691e-22	DOWN
BTLA	-1.89739016900149	1.47868019009157e-24	8.49216957455954e-24	DOWN
BTN1A1	1.61521785130071	2.29722426649102e-09	5.41513011403927e-09	UP
BTNL3	-2.67115083236166	9.2769251952917e-35	8.96443387489536e-34	DOWN
BTNL8	-3.20386257100738	1.21696015648185e-41	1.65167008611212e-40	DOWN
BTNL9	1.06482701965665	4.84372134245775e-09	1.12025129148913e-08	UP

GeneSymbol	log2FoldChange	pvalue	padj	change
BUB1	1.34786416557142	3.32126476497208e-55	8.69671930112162e-54	UP
BUB1B	1.20064175277143	9.24257492724369e-35	8.93619973393084e-34	UP
BVES	-2.95305502153321	1.15988329489263e-56	3.27342752252274e-55	DOWN
BX276092.9	4.33454745454396	5.62205920777212e-10	1.3854644351109e-09	UP
BYSL	1.55675035383187	6.68526760964005e-93	1.01226578162315e-90	UP
C10orf105	-2.26742059668462	1.33390665425047e-20	6.16436214715058e-20	DOWN
C10orf67	1.23892496876795	3.58575656041291e-05	6.19924334655182e-05	UP
C10orf95	2.84707368838009	1.33947742203575e-12	3.90888559576143e-12	UP
C10orf99	-1.86891655442174	1.14683645797303e-15	3.98838890407118e-15	DOWN
C11orf86	-3.69734765004129	2.15055244419946e-34	2.03077926848401e-33	DOWN
C11orf87	1.32254272457272	0.000225528471712188	0.00036288368859031	UP
C11orf98	2.28906412118784	2.98126755292221e-10	7.47377078880426e-10	UP
C13orf42	-1.25460223620454	0.00102480382647189	0.00155538298878716	DOWN
C14orf132	-2.04828923803652	1.86959081666591e-28	1.30064661968052e-27	DOWN
C14orf180	-2.87617243417709	1.45010714548615e-07	3.02766375591731e-07	DOWN
C15orf48	-2.09167300618972	4.15657431008296e-30	3.15374415954137e-29	DOWN
C16orf54	-1.38026444135137	6.83678417590599e-10	1.67546344064508e-09	DOWN
C16orf89	-3.57669685651974	3.62049399273348e-46	6.05606742511701e-45	DOWN
C16orf96	-1.54270155521555	5.15812050237692e-14	1.63306095105253e-13	DOWN
C17orf78	4.08690429930946	7.74045497353863e-22	3.81934095931505e-21	UP
C17orf99	2.05968319470772	2.62317421657368e-13	8.00093407482879e-13	UP
C19orf81	3.25036494193164	4.08409361027061e-13	1.2305991008071e-12	UP
C1orf105	4.21864225978964	7.46474180618381e-41	9.77319917827659e-40	UP
C1orf112	1.20180707795576	2.67923717727317e-49	5.23021939101544e-48	UP
C1orf115	-2.37080694132706	5.67712158241632e-68	2.69361629740096e-66	DOWN
C1orf21	-1.09071759695007	7.69718013486812e-19	3.21725870591595e-18	DOWN
C1orf210	-1.58242256629005	1.15227978983357e-60	3.83645276871355e-59	DOWN
C1orf87	1.80009156789881	0.000114310131241962	0.000188760769589026	UP
C1orf94	-1.05979920279844	0.0180692687855478	0.0239288293682214	DOWN
C1QA	-1.37847871739916	2.62546950777994e-17	1.00832158224464e-16	DOWN
C1QB	-1.18358419032201	1.72389687519752e-11	4.69254592587376e-11	DOWN
C1QC	-1.1757200347174	1.73808053050796e-11	4.73041517313773e-11	DOWN
C1QL4	1.7946495124114	1.85376156194981e-07	3.84006068025602e-07	UP
C1QTNF12	-1.27228528804042	2.69492122340504e-10	6.77641346760316e-10	DOWN
C1QTNF2	-1.70202983393521	1.36812604220267e-39	1.67768864597712e-38	DOWN
C1QTNF3	-1.26795396984598	2.16117065564896e-16	7.86237166982221e-16	DOWN
C1QTNF5	3.64583842791164	1.10077421579868e-15	3.83355628394048e-15	UP
C1QTNF7	-2.98304224687241	6.06584486313886e-42	8.31689422061709e-41	DOWN
C1QTNF8	2.87256184737283	2.38557334147096e-12	6.86724889982374e-12	UP
C1QTNF9	-2.72997603366183	4.22389524199499e-46	7.05183967870171e-45	DOWN
C1QTNF9B	1.36275240436521	5.95531672026273e-05	0.000100777385859995	UP
C2	1.93167643562482	4.15485606550856e-39	4.96558055378864e-38	UP
C20orf144	2.08637011072947	6.66822352627491e-37	7.15867917774506e-36	UP
C20orf194	-2.02477901677524	5.47094515774309e-38	6.17004974299096e-37	DOWN
C20orf204	2.39815831281093	3.05454985315357e-22	1.54036711824394e-21	UP
C20orf27	1.08262528731157	1.60244431486488e-17	6.22842920864781e-17	UP
C20orf85	2.66120496542508	2.91885545912294e-07	5.95850294369376e-07	UP
C22orf23	-1.12966848188143	9.15234426515867e-18	3.6113702852755e-17	DOWN
C22orf31	-1.31027757220742	1.59039746534336e-10	4.06660661733098e-10	DOWN
C2CD4A	4.28968793350312	2.02851541498981e-94	3.27060545566829e-92	UP
C2CD4B	2.56262648352472	3.14239422086963e-26	1.9746846108987e-25	UP
C2CD4D	1.16366448343753	1.23111875525262e-15	4.27381795957215e-15	UP
C2orf15	1.27064237422232	5.21123984680907e-49	1.00602349725595e-47	UP
C2orf66	1.59221902561299	1.3343190499095e-09	3.20608494771273e-09	UP
C2orf72	-1.1456711974639	1.65735913536141e-14	5.38525744057628e-14	DOWN
C2orf74	-1.51122479036817	2.81613837711257e-16	1.01526744432011e-15	DOWN
C2orf81	1.4949319746496	1.15397265961874e-23	6.32089522552412e-23	UP
C2orf88	-3.74485148986942	2.14371426174627e-107	5.25753470982928e-105	DOWN
C3orf18	-1.18184809484762	3.27103216035586e-20	1.48406677978834e-19	DOWN

GeneSymbol	log2FoldChange	pvalue	padj	change
C3orf70	-1.48409237096121	2.54675956955197e-22	1.291025455156e-21	DOWN
C3orf80	-1.20949705427041	4.60754770538355e-11	1.22117546718179e-10	DOWN
C3orf85	-1.88677729467602	4.50912530144063e-16	1.60567277860911e-15	DOWN
C4BPB	1.16004791104581	8.30380458833499e-10	2.02399425107331e-09	UP
C4orf19	-1.52043278571961	8.38785275840179e-44	1.24729018003459e-42	DOWN
C4orf36	2.1692802517897	1.44556080136958e-09	3.46572356247397e-09	UP
C4orf48	2.08323112418448	2.12319613586515e-19	9.1991078163274e-19	UP
C4orf50	-1.69997615634374	1.17284356514452e-12	3.4329677256449e-12	DOWN
C4orf54	1.47461613445596	2.40768475977592e-05	4.2267380504061e-05	UP
C5orf34	1.50024430425668	6.2335592824207e-59	1.91437332953777e-57	UP
C5orf38	2.89891832017428	1.69070525138644e-14	5.49053534919659e-14	UP
C5orf46	6.09810862535799	5.74945150784378e-47	1.00922579744036e-45	UP
C6	-1.64855621864795	1.81630948466158e-08	4.0361660357851e-08	DOWN
C6orf141	1.08417936591097	6.87898691720564e-10	1.68519694976508e-09	UP
C6orf15	8.11204682955365	5.53616090072337e-63	2.05546204188264e-61	UP
C6orf201	-1.0659413297281	2.85104391635332e-08	6.25885372106156e-08	DOWN
C7	-3.92010340390504	3.91273037422981e-45	6.22213461246243e-44	DOWN
C7orf31	-1.71288496057364	3.95221038873551e-45	6.27918243604484e-44	DOWN
C8orf74	5.79997633225755	1.96888107332068e-39	2.39080377473731e-38	UP
C8orf88	-3.16259421887124	1.83605121589799e-48	3.45260905209845e-47	DOWN
C9	1.01853422849202	0.000350551515160565	0.000554923048499175	UP
C9orf116	1.85557198973226	1.05160154000882e-35	1.06379051665959e-34	UP
C9orf16	1.25535765337271	2.67642401246179e-28	1.85012192651834e-27	UP
C9orf43	1.4791969978042	2.33906742477371e-32	2.00345209383102e-31	UP
C9orf50	2.33045065796427	2.90581717448492e-31	2.33605699258107e-30	UP
C9orf57	3.09601428380212	9.0450003343343e-14	2.82410957184442e-13	UP
CA1	-6.54567626200426	3.5688816889508e-57	1.02888968294206e-55	DOWN
CA10	-2.33154632163894	1.08786835807918e-13	3.38450093250542e-13	DOWN
CA12	-2.30207180319349	2.80049762186257e-41	3.72821598543523e-40	DOWN
CA14	-2.24940422804055	3.90368291586662e-49	7.56116024627201e-48	DOWN
CA2	-4.80785130046702	1.97697013672939e-93	3.07365901704186e-91	DOWN
CA4	-4.72107988180594	1.18434714262899e-38	1.3844790174581e-37	DOWN
CA5A	3.09119459766089	2.40878231459537e-13	7.36507927024569e-13	UP
CA6	3.24766691405294	2.16312487826894e-09	5.11078609298467e-09	UP
CA7	-5.66554145448724	2.44063422259766e-93	3.76095254142416e-91	DOWN
CA9	5.52664300012826	4.64697920586335e-78	3.53210983929652e-76	UP
CABCOCO1	-1.75781141887522	1.01422730484056e-15	3.53851734305523e-15	DOWN
CACNA1A	-1.30276608849066	6.15169402220413e-16	2.17236763351532e-15	DOWN
CACNA1C	-1.21021543709037	1.53649939853954e-11	4.19687278851278e-11	DOWN
CACNA1D	1.35407862553721	1.9405730913665e-23	1.04746432857919e-22	UP
CACNA1H	-1.9268174994793	6.22269476284653e-28	4.24425318861914e-27	DOWN
CACNA2D1	-2.21985820750217	3.50006781832121e-32	2.96721912952421e-31	DOWN
CACNA2D3	-1.29295022502373	1.00876694413434e-13	3.14121223143979e-13	DOWN
CACNB1	-1.09779817666261	5.94208223428452e-19	2.50652805100766e-18	DOWN
CACNB2	-1.44548985065432	4.67581867617495e-20	2.10007816889952e-19	DOWN
CACNG1	2.35066387248613	1.11241808412408e-05	2.00835003616928e-05	UP
CACNG4	3.55521000335046	2.95067833591377e-24	1.66818707348268e-23	UP
CACNG5	-2.70820088438805	3.86109573902197e-16	1.38141072742119e-15	DOWN
CACNG6	-1.61789891518939	9.29615608512303e-06	1.69041317784302e-05	DOWN
CACNG7	-2.23475756849874	8.51767037841104e-12	2.37043622022169e-11	DOWN
CACNG8	3.38285512830676	3.68612003513621e-42	5.09011960125509e-41	UP
CAD	1.43969024758078	1.28867195974083e-64	5.19436223031647e-63	UP
CADM1	-1.08018232106743	2.5462625039011e-09	5.98516049951807e-09	DOWN
CADM2	-4.70874137991455	4.04537481979298e-39	4.83805712479775e-38	DOWN
CADM3	-4.61644813596275	1.28143164279901e-58	3.86716970468964e-57	DOWN
CADPS	2.19900577803625	4.56467390466992e-26	2.84279923826957e-25	UP
CAGE1	1.00676008072955	5.20813867288082e-08	1.12197598306166e-07	UP
CALB1	3.31366078144673	1.15310544161876e-15	4.00938998700228e-15	UP
CALB2	-2.02828882607128	3.29892171866544e-12	9.39704300460026e-12	DOWN

GeneSymbol	log2FoldChange	pvalue	padj	change
CALCA	3.85501137076027	1.38136543712295e-23	7.52854972038243e-23	UP
CALCB	1.0787876266292	0.0208792469828247	0.027437199283973	UP
CALD1	-2.04600779454973	1.208695562731e-31	9.96072684989819e-31	DOWN
CALHM1	-1.00222595353341	0.000203180480112429	0.000328107363460793	DOWN
CALHM3	2.81502846028979	1.2317845936163e-12	3.600044499604e-12	UP
CALML3	3.57965789783119	1.26794101414223e-13	3.93418689936898e-13	UP
CALML5	2.47923469510464	0.000110746448287983	0.000183188743615336	UP
CALML6	1.6781630106147	5.25306195807853e-12	1.47820891848774e-11	UP
CALN1	-2.22208144159948	7.75237400265779e-14	2.42747866405827e-13	DOWN
CALY	-2.68543862239442	1.39780067586386e-21	6.80265600022847e-21	DOWN
CAMK1D	-1.04585478882175	2.88904189128954e-12	8.25921629502952e-12	DOWN
CAMK2A	-2.00611292604993	8.80396438474149e-26	5.40943655015891e-25	DOWN
CAMK2B	-1.06991672052247	0.000248851939845215	0.000399084438066378	DOWN
CAMK2D	-1.02799526369984	8.02168028460006e-31	6.29479579971793e-30	DOWN
CAMK2N1	-1.16389167002846	6.62436714707731e-26	4.10498594776004e-25	DOWN
CAMK2N2	1.45501022770077	5.44761903797888e-11	1.43769915593098e-10	UP
CAMK4	-1.58376925321546	1.17453743729255e-26	7.51643527952047e-26	DOWN
CAMKV	4.35897951869738	6.48415787366377e-37	6.96966920087082e-36	UP
CAMP	-1.63508515340043	1.08080106475028e-10	2.79684781401346e-10	DOWN
CAND2	-2.30883773519645	2.52693771937e-53	6.01936614328177e-52	DOWN
CAP2	-2.47671037368096	7.74453616536781e-59	2.35761552880332e-57	DOWN
CAPN12	2.07365220641551	9.97817443118901e-28	6.73971882739698e-27	UP
CAPN13	-1.7988482186673	1.09605112905595e-17	4.30630377036354e-17	DOWN
CAPN14	1.64004897339133	2.71243583292736e-08	5.96208598318153e-08	UP
CAPN2	-1.18079988756161	6.62384474034246e-40	8.28599198732639e-39	DOWN
CAPN5	-1.38283884804844	9.36190303105321e-39	1.09776981467831e-37	DOWN
CAPN9	-1.49960303649167	4.42904464764468e-09	1.02652674629891e-08	DOWN
CAPNS2	1.07721075357334	0.000340032766038713	0.000538859715601758	UP
CAPS	1.40462343373792	4.31468214202672e-11	1.14512361132619e-10	UP
CAPSL	1.58416878493866	0.00185147876501811	0.00274008156852459	UP
CARD14	2.42746483004076	2.17032468212662e-55	5.76975018165967e-54	UP
CARMIL3	1.77688281060557	1.88125183951864e-30	1.4469186520114e-29	UP
CARS2	1.00979955222363	4.41272276003903e-31	3.50701695210222e-30	UP
CARTPT	-2.48207735687196	8.48608347302887e-09	1.93110522106445e-08	DOWN
CASD1	-1.41880523584697	5.98741506297814e-49	1.15076002750153e-47	DOWN
CASP14	2.21188234456855	0.00105462109334581	0.00159813045848321	UP
CASP5	-2.12611318317174	1.04134980294204e-29	7.74584541590335e-29	DOWN
CASP7	-1.71371367537093	7.55770758231397e-86	8.07376454790388e-84	DOWN
CASQ1	-2.56461925681554	4.46648256720774e-32	3.76633709166045e-31	DOWN
CASQ2	-4.30344574729849	1.30670824403653e-32	1.13428268461655e-31	DOWN
CASR	-3.54224682075964	1.2954421867487e-42	1.82800119917789e-41	DOWN
CATIP	1.23856246824551	8.19942987619738e-06	1.4977097706307e-05	UP
CATSPER1	1.20581006895082	4.67610935982925e-10	1.15924106325038e-09	UP
CATSPERG	-1.17413271702653	4.8028629035436e-19	2.04030865429141e-18	DOWN
CAV1	-2.15565737314922	3.99375292848284e-41	5.30459342056993e-40	DOWN
CAV3	-2.13060469095898	5.57224287021297e-12	1.56423448491082e-11	DOWN
CAVIN1	-1.38823638806656	1.63973752369305e-21	7.95119729882125e-21	DOWN
CAVIN2	-3.16303339886945	9.34180125765416e-70	4.7984892418741e-68	DOWN
CBARP	2.52601352665838	2.15066232851402e-33	1.9424005770962e-32	UP
CBFB	1.24422014555705	3.15701611611353e-66	1.38471339118098e-64	UP
CBLIF	3.61496169535194	4.46995540759984e-28	3.05956499656195e-27	UP
CBLN1	1.41844455473768	1.81408274479855e-06	3.49779900732778e-06	UP
CBLN2	-2.97350480146495	3.065748962596e-46	5.14791578454043e-45	DOWN
CBS	1.34264722782276	1.06335457970027e-05	1.92156426902458e-05	UP
CBSL	1.02912295848051	0.0132791722840847	0.0178943063753883	UP
CBX2	3.64037182344257	9.01324887621005e-121	2.90643893854529e-118	UP
CBX3	1.04974754883187	1.02341503309592e-31	8.46590307425145e-31	UP
CBX4	1.59026798532503	7.06197621621086e-66	3.05136952488535e-64	UP
CBX6	-1.2326368782569	3.89532230332645e-16	1.39279768517091e-15	DOWN

GeneSymbol	log2FoldChange	pvalue	padj	change
CBX7	-1.82431337843424	2.91824177024496e-69	1.45602704714256e-67	DOWN
CBX8	1.73200846236062	9.66156423908552e-110	2.43821475500284e-107	UP
CBY2	7.25996753356832	2.95960972006171e-80	2.39700856071789e-78	UP
CCBE1	-3.93005088334939	2.49349270855968e-45	4.00915868274697e-44	DOWN
CCDC113	1.77448149100579	9.25978337093171e-51	1.95680349318002e-49	UP
CCDC114	1.55501131406421	5.34304429484893e-16	1.89564853924622e-15	UP
CCDC13	-1.50257900156697	2.20194572196918e-19	9.53081801060136e-19	DOWN
CCDC136	-1.56329397520779	2.17445514408373e-16	7.90805919463867e-16	DOWN
CCDC141	-1.14137950732515	2.33345003239399e-06	4.45579179888985e-06	DOWN
CCDC148	2.12729105619591	5.10648233719938e-27	3.33405237861465e-26	UP
CCDC150	1.95560061156714	3.30492919335141e-48	6.11569947330798e-47	UP
CCDC152	-1.95668667733456	6.92007529490081e-24	3.83145536121169e-23	DOWN
CCDC154	1.18205459603491	2.10791550422722e-08	4.66333790815761e-08	UP
CCDC158	-2.24070648817185	5.00733436139203e-48	9.15889844904616e-47	DOWN
CCDC160	-1.19753563339398	0.00331180543539678	0.00477269453335795	DOWN
CCDC168	1.13701662412204	1.5022551102265e-05	2.68598092559544e-05	UP
CCDC169	-1.58624598687851	4.90878071113879e-05	8.37677366944922e-05	DOWN
CCDC175	1.1795142889833	3.16224400081378e-07	6.43949886401243e-07	UP
CCDC178	-1.8099099084643	1.34545325271028e-15	4.66514884297971e-15	DOWN
CCDC187	2.55084081345644	1.58079843077318e-21	7.68036916156625e-21	UP
CCDC189	1.71767534399107	6.10341080110498e-22	3.02443631985319e-21	UP
CCDC190	1.67940487754869	0.00168879622771508	0.00250699136514942	UP
CCDC192	1.61076390669673	2.34662439509493e-26	1.48318586540065e-25	UP
CCDC194	1.53843491289482	7.49728576050147e-06	1.37493667138085e-05	UP
CCDC198	6.31023049735834	3.89317361834925e-34	3.62523166932168e-33	UP
CCDC33	3.30335901460574	1.52637835365216e-31	1.25017997517145e-30	UP
CCDC68	-2.23021289305453	1.30476602966018e-47	2.34225679118275e-46	DOWN
CCDC69	-3.04341452614627	1.27599366990914e-94	2.09612054472905e-92	DOWN
CCDC73	1.18300495982923	3.40575287577301e-06	6.42030689897536e-06	UP
CCDC74A	1.21308029964677	1.04002389509835e-24	6.01659006157727e-24	UP
CCDC78	3.64198878466432	4.03463595693701e-76	2.76594944559623e-74	UP
CCDC80	-1.85910057530149	3.83742730246962e-17	1.45929507791884e-16	DOWN
CCDC83	1.67112247345886	0.000365583190856301	0.000577563064995533	UP
CCDC85A	-1.4439622380909	2.25191235676452e-10	5.69205252842803e-10	DOWN
CCDC85B	1.95995277470895	3.94512453819664e-29	2.84575201257739e-28	UP
CCDC86	1.43151427826747	8.31227862992437e-73	4.8899225602322e-71	UP
CCDC88B	1.30413291893813	3.75222521827511e-11	9.99349919330444e-11	UP
CCER2	1.03547825897053	2.17808590740664e-06	4.16917774053774e-06	UP
CCKAR	-2.47802818069322	1.50780795181651e-05	2.69535569910491e-05	DOWN
CCL11	-1.35223235849516	6.07484997798655e-10	1.49450922106075e-09	DOWN
CCL13	-2.4188889549053	1.96132234034838e-18	8.03399339272789e-18	DOWN
CCL14	-1.59914274747828	3.03440759713735e-23	1.62179679217166e-22	DOWN
CCL15	-1.51058535246122	1.88116578066148e-23	1.01697422349141e-22	DOWN
CCL16	-1.35405825158158	1.12972243015676e-10	2.91953942955174e-10	DOWN
CCL19	-2.34667073913047	6.26323110613748e-17	2.34793634555806e-16	DOWN
CCL20	1.61586970758018	1.57527939417216e-15	5.43713381381959e-15	UP
CCL21	-2.21621142860027	4.31092570790666e-19	1.83580702743406e-18	DOWN
CCL23	-3.30920677078715	8.270826909864e-55	2.12732509573799e-53	DOWN
CCL24	1.86255896049471	4.32822417494583e-19	1.8427229231866e-18	UP
CCL25	4.18742922245424	1.48419563059611e-19	6.47726779838846e-19	UP
CCL26	1.94794895283938	1.61012565409072e-15	5.55520467895419e-15	UP
CCL28	-2.15878781594742	1.71927761043693e-29	1.26586811968449e-28	DOWN
CCL3	1.35874034981883	3.50192363329832e-09	8.16427851474409e-09	UP
CCL5	-1.3409726242672	2.52949354395095e-12	7.26593056430516e-12	DOWN
CCL8	-2.42016150636307	8.04947814042334e-22	3.96507957168859e-21	DOWN
CCN4	2.54914433139548	2.909848757939e-51	6.29431011453314e-50	UP
CCN5	-3.66668951305418	2.41504995900327e-33	2.17667002774968e-32	DOWN
CCN6	5.62678646934447	2.2090610473498e-39	2.67499165629361e-38	UP
CCNA1	-1.72774212833561	4.64393312139322e-10	1.15175626609913e-09	DOWN

GeneSymbol	log2FoldChange	pvalue	padj	change
CCNA2	1.07666053647322	3.80390892649265e-28	2.61085794785245e-27	UP
CCNB1	1.44899303533892	4.77624399044939e-46	7.94352785154682e-45	UP
CCNB1IP1	1.10470190450729	4.23824895715041e-36	4.37985929322612e-35	UP
CCND1	1.65467580338422	4.7847582050183e-73	2.84358343426565e-71	UP
CCNE1	1.63649498821514	5.09403020962131e-46	8.45585990468406e-45	UP
CCNF	1.52432323093843	1.02674103631654e-61	3.59731220631385e-60	UP
CCNG2	-1.07474848386932	3.43402957415064e-31	2.74675043521751e-30	DOWN
CCNI2	1.41335991666559	2.74214505013621e-29	1.99536029076564e-28	UP
CCNJL	-1.56501596586678	4.35908826383411e-23	2.30783836844461e-22	DOWN
CCNL2	1.01416384006487	2.12885520611385e-25	1.27387476646256e-24	UP
CCNO	3.07020454154805	1.83371241921348e-91	2.49456518404409e-89	UP
CCNP	4.90890218593497	9.9109376388651e-107	2.36409804254189e-104	UP
CCNYL1	-1.42310893967829	1.3302417645649e-90	1.76820609514264e-88	DOWN
CCR10	-1.69612583217937	3.52029755636742e-19	1.50500715318011e-18	DOWN
CCR2	-2.16901285139931	1.65157098939157e-26	1.04997464907906e-25	DOWN
CCR7	-1.35490819444711	1.93617772808966e-12	5.59672356892849e-12	DOWN
CCR8	2.00665552801957	1.34992054474446e-21	6.57699117113464e-21	UP
CCR9	-1.65955121039201	4.0353418242709e-13	1.21633039961968e-12	DOWN
CCT6A	1.12677395684038	3.0247711022356e-76	2.09841988857484e-74	UP
CD101	-1.3321985112819	7.82012127145487e-30	5.85937055507073e-29	DOWN
CD14	-1.01483166282918	4.59550315283372e-11	1.21816861623221e-10	DOWN
CD160	-2.35869472901647	3.00991022255802e-59	9.46057160747342e-58	DOWN
CD163	-1.22865293464566	1.67901058049474e-08	3.73965352240406e-08	DOWN
CD163L1	-2.8910920884883	5.53385042339514e-63	2.05546204188264e-61	DOWN
CD164L2	1.04097859063005	1.11182880066434e-05	2.00749428722191e-05	UP
CD177	-5.1512786957868	2.23347984138061e-48	4.17739897722456e-47	DOWN
CD180	-1.73393181680929	3.72098771616995e-24	2.09416803819222e-23	DOWN
CD19	-2.08875147153031	5.98969961937866e-22	2.97062487816123e-21	DOWN
CD1C	-1.81597494169033	1.45366981151667e-22	7.46468664934822e-22	DOWN
CD1D	-1.7081027297135	2.70938645276296e-36	2.83014674876794e-35	DOWN
CD200R1	-1.74881912646964	1.39744553987962e-25	8.46979435639535e-25	DOWN
CD200R1L	3.20573729696097	2.54092561490067e-10	6.40398577685127e-10	UP
CD207	-1.45315204012022	1.02690813987836e-12	3.01696498054696e-12	DOWN
CD209	-2.31310520279577	4.2519940444083e-38	4.823450963862e-37	DOWN
CD22	-2.6000698918057	4.50825091651084e-26	2.808664515535e-25	DOWN
CD226	-1.00959060811855	5.68647570472388e-09	1.30890418303182e-08	DOWN
CD244	-1.04645192655724	6.592697771774e-08	1.40926401055611e-07	DOWN
CD247	-1.02340356142532	5.43913600536293e-12	1.52933433330187e-11	DOWN
CD27	-1.71749935272861	1.64796617896691e-20	7.5775112422368e-20	DOWN
CD276	1.04125975041142	2.03907095509699e-31	1.65453599911947e-30	UP
CD28	-1.07498905901927	3.0004498331838e-11	8.04168584657988e-11	DOWN
CD300LD	2.41746803397197	6.3441307564587e-08	1.35846469333762e-07	UP
CD300LG	-2.84388112861725	1.49693980899986e-11	4.09010087778355e-11	DOWN
CD302	-1.35341023803872	4.03601787602147e-27	2.65304565025148e-26	DOWN
CD33	-1.31565486542416	9.35455662706801e-17	3.47093318873078e-16	DOWN
CD36	-2.54131756704251	1.60058516669588e-31	1.30911176644788e-30	DOWN
CD37	-1.41879814393615	5.22893080737741e-16	1.85667561478105e-15	DOWN
CD38	-1.08800121987382	8.79498518047822e-08	1.86106546296837e-07	DOWN
CD3E	-1.06580479642559	5.74272950244171e-11	1.51306020314749e-10	DOWN
CD3G	-1.11544885351328	8.66041466288428e-12	2.40862163432046e-11	DOWN
CD4	-1.05227209304138	6.34166773689063e-13	1.88668136515422e-12	DOWN
CD40LG	-1.29428355397819	6.27232513235068e-14	1.97575972376307e-13	DOWN
CD44	1.29773895454476	4.56893007188118e-56	1.24700281099792e-54	UP
CD46	1.0086526094095	3.02948074017353e-27	2.00198664249826e-26	UP
CD48	-1.51020586647588	6.17554531250482e-16	2.18034814530913e-15	DOWN
CD52	-1.23193489906306	9.60661375173605e-14	2.99731168713456e-13	DOWN
CD55	1.05799068350368	4.80547712373666e-11	1.27189197682971e-10	UP
CD69	-1.20130754759172	7.75869558688593e-10	1.89563864535491e-09	DOWN
CD70	1.34676186193574	7.48061251827613e-10	1.83000710565808e-09	UP

GeneSymbol	log2FoldChange	pvalue	padj	change
CD79A	-2.48412510111428	1.12768640106191e-24	6.51290325097545e-24	DOWN
CD79B	-2.06526314429633	1.05261323501896e-26	6.75604653939743e-26	DOWN
CD8A	-1.10268166926329	3.03240725918159e-08	6.6448193383127e-08	DOWN
CD96	-1.3872543472872	1.78448287139017e-16	6.52648517333992e-16	DOWN
CDC14A	-1.96529288711025	1.98909639213079e-82	1.81341023435463e-80	DOWN
CDC20	1.21603398837872	3.27225303216197e-28	2.254837437635e-27	UP
CDC20B	1.88236727409344	8.27131217356809e-06	1.51036450166046e-05	UP
CDC25A	1.18962640003468	7.40567864574971e-34	6.81580773036151e-33	UP
CDC25B	2.21437330487719	5.84759361684313e-86	6.28544121296848e-84	UP
CDC25C	1.19551451344377	3.29789749363113e-39	3.96590394037285e-38	UP
CDC42EP2	3.23346595214151	2.07516035060964e-14	6.70495371656252e-14	UP
CDC42EP3	-1.05460136937753	2.18314602673664e-22	1.11090361670266e-21	DOWN
CDC45	1.66976571806279	7.98840697099599e-54	1.95368160935328e-52	UP
CDC6	1.64356948412122	5.28198355111913e-45	8.31601985313178e-44	UP
CDCA2	1.04168267696633	2.2860500246179e-16	8.30870154011094e-16	UP
CDCA3	1.54639615757556	3.05006917203732e-56	8.43029436391838e-55	UP
CDCA4	1.46455999526891	2.26810114126356e-46	3.84561296716868e-45	UP
CDCA5	1.49371758242241	4.9137994818395e-64	1.91847512056662e-62	UP
CDCA7	1.66008214656636	2.08531398374647e-53	5.00159399434948e-52	UP
CDCA8	1.06001827384353	1.43225061590657e-36	1.5151749680912e-35	UP
CDH10	-4.23407313587943	1.03370656383483e-35	1.04711648609982e-34	DOWN
CDH11	1.47628768175086	8.26304288224199e-20	3.65745718628571e-19	UP
CDH12	-1.21826843993959	0.0132476749112219	0.0178532438069118	DOWN
CDH15	1.57730627959241	2.8913721575792e-12	8.26452123767671e-12	UP
CDH16	1.59505005845023	1.18272473657726e-05	2.12998095335814e-05	UP
CDH19	-4.44567651768769	1.65465536988855e-35	1.66162133540192e-34	DOWN
CDH22	-1.40202592154867	0.000432593801763274	0.0006796062676023	DOWN
CDH23	-1.2411564064928	1.75108091324245e-12	5.07600664929095e-12	DOWN
CDH24	1.11313581151438	8.38584619689444e-22	4.1284348268737e-21	UP
CDH3	5.77843785587618	0	0	UP
CDH4	1.13132168948855	2.02061225112129e-07	4.17327969739949e-07	UP
CDH7	1.86027387453508	2.17411258803639e-06	4.16202973784935e-06	UP
CDHR2	-2.14879864301968	1.0993878354118e-23	6.02569731760329e-23	DOWN
CDHR5	-2.3163512984206	1.91201740595547e-42	2.67421358151829e-41	DOWN
CDIP1	-1.10653893433655	1.49010641903757e-12	4.33537561816226e-12	DOWN
CDK1	1.43865916531664	2.25716444336531e-40	2.89212689126712e-39	UP
CDK15	-1.82573087383712	2.11954746284103e-15	7.25957513187467e-15	DOWN
CDK2	1.08770754647343	8.09624623977868e-69	3.9824840613917e-67	UP
CDK4	1.33460775916752	5.48593234207739e-51	1.16923549415659e-49	UP
CDK5R2	-1.04610000320554	0.000421973103483857	0.000663280163473949	DOWN
CDK5RAP1	1.08114850358117	2.33558335184285e-47	4.15418926513171e-46	UP
CDK5RAP3	2.04998466554458	3.5004336365449e-40	4.45237771462062e-39	UP
CDKL1	-1.60637728462369	1.25038555799054e-87	1.45153091475262e-85	DOWN
CDKL2	-1.9233153169318	2.76810167218435e-23	1.48219417028739e-22	DOWN
CDKL4	1.31794504809007	5.52283487479069e-05	9.38143826697203e-05	UP
CDKN1A	-1.14079167153907	3.97327920202556e-18	1.59932294833266e-17	DOWN
CDKN2A	3.1425218942864	8.54124503822263e-41	1.11658183070999e-39	UP
CDKN2B	-3.22741657578018	5.23950922393956e-92	7.41752635093167e-90	DOWN
CDKN3	1.19039044742186	1.08702377037135e-29	8.07523247161957e-29	UP
CDO1	-1.95430742417504	3.21476137912403e-15	1.0912015574013e-14	DOWN
CDON	-1.68348021124274	1.2821964475491e-31	1.0541495156361e-30	DOWN
CDR2L	1.13480719021237	1.21522560481288e-16	4.48415415482237e-16	UP
CDRT1	1.79071007783572	1.1972835207229e-14	3.92993363738884e-14	UP
CDSN	1.26651063888339	2.72885879957994e-05	4.77324141407186e-05	UP
CDT1	1.59621510292954	1.60719658876462e-37	1.77915538462545e-36	UP
CEACAM1	-2.21219141158368	1.22826196598683e-55	3.29548930874093e-54	DOWN
CEACAM16	-1.0100020226589	3.2162095336858e-05	5.5897651073032e-05	DOWN
CEACAM18	1.99237381598045	2.08980933518026e-06	4.00548706147428e-06	UP
CEACAM3	-1.31332727732458	3.47058153073572e-12	9.86665080729815e-12	DOWN

GeneSymbol	log2FoldChange	pvalue	padj	change
CEACAM7	-4.17796204916124	7.39390599265451e-50	1.47818696957627e-48	DOWN
CEBPB	1.56282669458843	2.17958207949612e-39	2.64113171539776e-38	UP
CEBPE	-1.20413041965025	5.22235292827336e-05	8.89444752934507e-05	DOWN
CECR2	1.75456427153849	2.93850761835698e-09	6.8792999675249e-09	UP
CEL	6.11856497120331	1.61065903847315e-73	9.84084415330982e-72	UP
CELA3B	-3.21250880552379	1.77582749922676e-17	6.8846598647666e-17	DOWN
CELF4	-2.64761028463145	3.26248970990519e-23	1.7378321602502e-22	DOWN
CELSR1	2.26408514421697	2.94056891395091e-61	1.00400248036524e-59	UP
CELSR3	2.75067671782188	2.52997897288018e-72	1.44916196890667e-70	UP
CEMIP	5.20802259048111	1.22462274966701e-227	2.66554449249396e-224	UP
CEND1	-2.02731711223942	2.79364627254239e-19	1.20142658789283e-18	DOWN
CENPA	1.41976164110404	5.00507835392137e-53	1.17299366590623e-51	UP
CENPE	1.24146347518996	2.66382915129853e-24	1.50944539575533e-23	UP
CENPF	1.38404992158302	2.4673359354983e-32	2.11227731783835e-31	UP
CENPH	1.28844324561134	2.19890045791351e-43	3.20950994749773e-42	UP
CENPI	1.51695258846409	1.63080792690464e-52	3.76122628227689e-51	UP
CENPJ	1.0350451736628	8.5587329399489e-24	4.7162410342826e-23	UP
CENPN	1.24007209297671	1.63069162343433e-47	2.91532168776817e-46	UP
CENPP	1.03137513971116	1.90846553489455e-42	2.67139150796774e-41	UP
CENPW	1.11963024558752	1.28987844739838e-27	8.66203370788582e-27	UP
CEP250	1.02651171115355	2.56711240695239e-24	1.45653725455399e-23	UP
CEP295NL	-1.06351479345683	1.07190056805524e-05	1.93680653642689e-05	DOWN
CEP55	1.52855685434252	4.98200711433429e-51	1.06574557594475e-49	UP
CEP72	2.02025920316095	2.74155215804307e-92	3.97822064400033e-90	UP
CEP85L	-1.1062496201149	9.58885837714577e-13	2.82283670196516e-12	DOWN
CER1	2.02571971911436	2.92456463699355e-05	5.10326125102402e-05	UP
CERKL	1.69653316045572	6.37270209629659e-08	1.36407942965965e-07	UP
CERS1	-1.24511425287008	2.36033322346905e-06	4.50465611795994e-06	DOWN
CES2	-1.96182339786374	2.17703353816704e-36	2.2795360793808e-35	DOWN
CES3	-2.15483158880277	9.45864578600814e-31	7.39575209123304e-30	DOWN
CFAP157	1.95093928652654	1.13539656579659e-73	6.98609908134844e-72	UP
CFAP161	1.45772861526161	8.09107125131037e-10	1.9743529105811e-09	UP
CFAP20DC	1.82832577461803	1.09253529578414e-18	4.52959931083076e-18	UP
CFAP251	2.54132185739358	7.28034615840464e-68	3.43557364922222e-66	UP
CFAP43	1.03821943399488	1.37694107919299e-09	3.30530397187586e-09	UP
CFAP45	1.97557760286378	3.04437426735284e-35	3.00860891699291e-34	UP
CFAP46	1.27319469769075	3.54311030932253e-05	6.12916548939333e-05	UP
CFAP47	2.09574349208482	1.08840900862093e-07	2.29448741732642e-07	UP
CFAP54	-1.60169974034408	1.5295329570466e-19	6.66844200827554e-19	DOWN
CFAP73	1.71319873355649	3.77756913478747e-12	1.07218926396176e-11	UP
CFAP74	1.9546875373794	1.07296181998778e-14	3.53585998702636e-14	UP
CFAP92	2.7659161053372	2.08477669657049e-86	2.26888853858637e-84	UP
CFAP99	-1.11409422311124	4.22749405892005e-10	1.05131896669487e-09	DOWN
CFB	1.58617592576741	1.25361979177134e-18	5.18018069153166e-18	UP
CFD	-2.30448741665916	4.50548962268511e-23	2.38172710381954e-22	DOWN
CFH	-1.38052670561607	4.24151423788382e-17	1.60804457705793e-16	DOWN
CFI	1.19492706615438	2.03931429298658e-11	5.52006525474511e-11	UP
CFL2	-2.81387758553019	3.47771520523318e-77	2.5444308768372e-75	DOWN
CFP	-1.25044200018559	1.59531501740842e-15	5.50519627390662e-15	DOWN
CGA	1.49815569757091	0.000890527385047191	0.00135845408285823	UP
CGAS	1.14260302519541	3.6964997855197e-15	1.25130541923123e-14	UP
CGB5	4.16274128262405	2.02785364277921e-09	4.80291287836158e-09	UP
CGB7	2.45477666488578	5.10707242871848e-07	1.02512336831441e-06	UP
CGB8	2.66736620940039	0.000420134519383971	0.000660569064201634	UP
CGN	-1.37455240914486	2.86366997718745e-44	4.33232713403694e-43	DOWN
CGREF1	3.53464446261145	1.52502343059373e-121	5.10677557633242e-119	UP
CGRRF1	-1.14513966402439	3.3286536418654e-34	3.11288108838895e-33	DOWN
CHAC1	1.70519925865441	2.64606153892996e-30	2.02531294845659e-29	UP
CHAD	-2.62817877342786	3.173563758997e-39	3.82430904743355e-38	DOWN

GeneSymbol	log2FoldChange	pvalue	padj	change
CHAF1B	1.331708800662	1.37870596802815e-53	3.33436208628809e-52	UP
CHAT	-4.71199006433616	4.8569235356202e-39	5.78478861325271e-38	DOWN
CHCHD6	1.20140635686521	5.07889934653975e-38	5.74278404683745e-37	UP
CHD1L	1.22614141793903	8.45817834137681e-19	3.52687402917611e-18	UP
CHD5	-1.20133228147904	4.44316711953543e-09	1.02952586896168e-08	DOWN
CHD7	1.04692110893115	1.20446063677333e-27	8.11031441149807e-27	UP
CHEK1	1.19919650422641	5.57984558307038e-43	8.00344737545341e-42	UP
CHGA	-4.51199217231949	1.5317139494977e-35	1.53904991359512e-34	DOWN
CHGB	-2.58479721445272	7.09978149421566e-16	2.49754535674297e-15	DOWN
CHI3L1	2.88235801136623	5.70848251287509e-42	7.83308163882537e-41	UP
CHI3L2	-1.40964608419448	1.1291949756155e-09	2.72827419320005e-09	DOWN
CHKB	1.75723969536643	6.20953531321186e-25	3.63328758094618e-24	UP
CHKB-CPT1B	1.92439291681277	3.07384933392606e-15	1.04459286595735e-14	UP
CHL1	-2.78835917074858	3.86431691209202e-43	5.57492546729564e-42	DOWN
CHN2	1.74531999319583	2.53809383156586e-13	7.74957529178614e-13	UP
CHODL	-3.13870700856364	4.61774874162248e-39	5.51123090047103e-38	DOWN
CHP1	-1.43961580089162	3.56649637486087e-69	1.77438289644149e-67	DOWN
CHP2	-3.86323567429404	1.4643335741161e-47	2.62060025961805e-46	DOWN
CHPF	1.77438997137648	9.99111898862058e-56	2.70148066690761e-54	UP
CHRDL1	-3.18663916369773	6.63829804028337e-27	4.31155105466074e-26	DOWN
CHRFAM7A	-1.18454440370941	4.30394040302328e-05	7.38879170243955e-05	DOWN
CHRM2	-3.86947036152877	2.38377008856571e-18	9.71415599162056e-18	DOWN
CHRM4	-2.10929369802252	4.04322276547965e-18	1.62672453824624e-17	DOWN
CHRM5	-1.23638358155849	9.02097467163636e-12	2.50569838821509e-11	DOWN
CHRNA1	-1.74172228316935	1.73169416508182e-21	8.3900919578658e-21	DOWN
CHRNA3	-2.11699651371012	7.8476977820385e-20	3.47538050556044e-19	DOWN
CHRNA4	-2.17043185270105	1.34334834025921e-12	3.91952490766315e-12	DOWN
CHRNA6	1.89675417435851	4.81119132419614e-10	1.19137193583941e-09	UP
CHRNA7	-1.12040962366092	0.000110935947399677	0.000183484769383603	DOWN
CHRNB2	-1.05028881813502	2.37088679641725e-05	4.16549811179635e-05	DOWN
CHRNB4	-1.79892424602709	3.05971024114193e-19	1.31228409923656e-18	DOWN
CHRND	3.33185930860115	1.16618234945603e-09	2.81490618950345e-09	UP
CHRNG	1.39980472876877	0.000130330114212223	0.000214320358747515	UP
CHST15	-1.18087360891263	2.01557472018309e-14	6.51758637001821e-14	DOWN
CHST4	5.10182710748465	4.29436609621786e-37	4.65036049959214e-36	UP
CHST5	-2.33514509247495	3.83292200388266e-17	1.45790019339469e-16	DOWN
CHST8	-2.46761998266381	4.44978659438434e-23	2.35442521932587e-22	DOWN
CHST9	-2.37728243949881	6.63947814767212e-06	1.22316158469546e-05	DOWN
CHSY3	1.36747116489404	5.62871848477627e-19	2.37661476101336e-18	UP
CHTF18	1.86602646714591	1.45822394749542e-50	3.04461074313403e-49	UP
CHTF8	1.01124927471553	1.5398861854591e-07	3.20665368899777e-07	UP
CIART	-1.16039493797429	1.96789110926569e-10	4.9973585949604e-10	DOWN
CIBAR1	1.31571220856322	2.60326603170032e-25	1.54817866837424e-24	UP
CIDEA	-1.8381124780887	0.0166829559278484	0.0221874521936625	DOWN
CIDEC	-2.24664495931919	1.66453186392559e-18	6.84080560456369e-18	DOWN
CILP	-2.87882263583904	5.46034918879865e-22	2.7150502691191e-21	DOWN
CILP2	2.70672121915429	1.15488756341503e-26	7.39884368717659e-26	UP
CIP2A	1.23513338378557	4.30608513210386e-34	3.99903255495064e-33	UP
CIPC	-1.32997314414328	1.00142126785877e-111	2.60264903540669e-109	DOWN
CITED1	3.73972783763989	5.2363847284258e-19	2.21851988506274e-18	UP
CITED2	-1.77345608512138	2.73335518281446e-82	2.46610952323048e-80	DOWN
CITED4	1.37563860173466	2.65561619193771e-13	8.0942140294436e-13	UP
CKAP2	1.47314939402155	4.37423926952715e-52	9.85363886161401e-51	UP
CKAP2L	1.46667621623244	8.77693511900965e-50	1.74865870969468e-48	UP
CKB	-1.40613126959055	2.97361430443296e-09	6.95868107553973e-09	DOWN
CKLF-CMTM1	2.99564628748535	6.71516206874796e-12	1.87871332106537e-11	UP
CKM	-1.38703432321869	1.30376475893697e-08	2.92293752380192e-08	DOWN
CKMT1A	-1.05581119334978	1.93127713973955e-16	7.04574247523252e-16	DOWN
CKMT1B	-1.14013395566956	2.82581283268829e-18	1.14592172462974e-17	DOWN

GeneSymbol	log2FoldChange	pvalue	padj	change
CKMT2	3.42180844615944	1.40189066913125e-26	8.93525703571829e-26	UP
CKS2	1.43600116534583	1.0971613420159e-39	1.35399507076703e-38	UP
CLBA1	1.24916338269177	2.12407116617723e-44	3.26447053986267e-43	UP
CLC	-1.41006302277004	0.000140365975371615	0.000230171647909024	DOWN
CLCA1	-2.50902596845942	9.31950320946409e-10	2.26427388567599e-09	DOWN
CLCA2	-1.44884282203397	9.23705534288397e-05	0.000153844901660104	DOWN
CLCA4	-5.32786203099238	6.971005706339e-41	9.14052126238562e-40	DOWN
CLCN1	2.19884421747122	1.62547825483231e-20	7.4780588775152e-20	UP
CLCN2	-1.32350963664898	7.04998338682799e-27	4.56871457814796e-26	DOWN
CLCN4	1.7209845233782	9.63211865283483e-25	5.5908027367271e-24	UP
CLCNKB	-3.3240464637487	8.51078263527271e-40	1.05931563994284e-38	DOWN
CLDN1	4.78686741979768	2.52497941273567e-237	6.28106664485233e-234	UP
CLDN10	1.92118127364703	2.11567670345263e-05	3.73406430541461e-05	UP
CLDN11	-2.17533541136679	4.48144868193895e-23	2.36973780439122e-22	DOWN
CLDN14	3.25377259487448	1.2141452601279e-35	1.2256180530207e-34	UP
CLDN16	2.86627794906078	1.99948352344653e-38	2.31035212964662e-37	UP
CLDN18	5.62968347363872	4.17196591257402e-31	3.32022131789997e-30	UP
CLDN19	-1.6312737624581	2.58979365293257e-05	4.53500370861975e-05	DOWN
CLDN2	5.59713796163978	4.34337154271795e-92	6.19927284207768e-90	UP
CLDN23	-2.94385182579769	3.5736816587598e-90	4.57562637676356e-88	DOWN
CLDN5	-1.77647291763468	4.02255881393382e-22	2.01509829191685e-21	DOWN
CLDN6	2.80031147802065	1.74607847225392e-14	5.66613202336145e-14	UP
CLDN8	-5.14176404448033	2.20560875781487e-22	1.12167830899037e-21	DOWN
CLDN9	2.80510090376886	9.86799902217592e-30	7.35894933503851e-29	UP
CLEC10A	-2.80964507075394	3.94608349274059e-42	5.44047124775075e-41	DOWN
CLEC17A	-2.35762008846938	3.85194214484793e-19	1.64477362845113e-18	DOWN
CLEC18B	1.11889977432551	2.92794038075435e-06	5.54900150740918e-06	UP
CLEC2A	2.61899631543981	0.00746688618077232	0.0103536302807603	UP
CLEC3A	1.6621634003242	0.00478678192735564	0.00677825759949937	UP
CLEC3B	-4.08198129166401	3.00776453867562e-151	2.01439245815225e-148	DOWN
CLEC4F	-1.88873970967599	4.72852941445795e-15	1.59353363061653e-14	DOWN
CLEC4M	-3.49348904053248	9.3943809478723e-21	4.38563955617427e-20	DOWN
CLEC5A	3.39693032667881	9.25619708469229e-49	1.76343719732765e-47	UP
CLEC6A	1.49152096960922	2.97473497921484e-06	5.63461984042945e-06	UP
CLEC9A	-2.96515298491929	3.37020154254035e-54	8.4560979049359e-53	DOWN
CLECL1	-2.1083465782553	8.39762014634555e-29	5.95632422029797e-28	DOWN
CLIC2	-1.49878377887749	4.67120133663534e-23	2.466331985289e-22	DOWN
CLIC3	2.01416562933886	1.10358781698576e-19	4.84904735230207e-19	UP
CLIC5	-2.13374686791459	1.17813905443616e-50	2.47466047706837e-49	DOWN
CLIC6	-1.01354839613889	2.65507763410176e-06	5.04808798790238e-06	DOWN
CLIP3	-2.0120535994709	1.12215940973344e-39	1.38386415026121e-38	DOWN
CLIP4	-2.19105167517428	1.67122460544619e-32	1.44064525022943e-31	DOWN
CLMN	-1.37782724901963	2.67512290321234e-53	6.35496795547565e-52	DOWN
CLMP	-2.07147771941737	3.22163899619402e-30	2.45078199391553e-29	DOWN
CLPB	1.05487176128682	2.20106857858295e-44	3.37684644571498e-43	UP
CLPSL1	2.35945563570109	0.00332642350568772	0.00479257095023501	UP
CLPSL2	3.76548440735183	1.74422136713453e-18	7.1598601286925e-18	UP
CLSPN	1.2174193101535	5.64205941503185e-30	4.26041546374456e-29	UP
CLU	-2.36509561308208	5.40559804980898e-31	4.27658695326323e-30	DOWN
CLVS2	-3.21625062943019	3.15674766947201e-16	1.13500820087789e-15	DOWN
CMA1	-3.94847856865404	1.7007880620409e-28	1.18653135113455e-27	DOWN
CMBL	-1.76306674313676	1.22776716963902e-31	1.01131077222915e-30	DOWN
CMKLR1	-1.44922111524198	6.83455115459052e-19	2.86771178927433e-18	DOWN
CMSS1	1.39326258310214	2.307166229076e-64	9.17230263627861e-63	UP
CMTM5	-4.82062761034909	4.37123831999069e-74	2.72818540738344e-72	DOWN
CMTM7	1.43843675666662	1.50017522471465e-35	1.50823043810371e-34	UP
CMTM8	1.32962707017585	4.86307902003111e-59	1.50946158602142e-57	UP
CMYA5	-1.13340864858709	3.92318214067415e-15	1.32675025472051e-14	DOWN
CNDP1	1.55981045263106	7.00645712031951e-06	1.28804305147934e-05	UP

GeneSymbol	log2FoldChange	pvalue	padj	change
CNGA1	-1.61051628459657	2.95741100290293e-19	1.2699728185832e-18	DOWN
CNGA3	-3.78477944792764	1.01145113229037e-37	1.13044920196227e-36	DOWN
CNGB1	-3.75573744269077	5.92123562990454e-66	2.57123381604807e-64	DOWN
CNIH3	1.36410755531306	7.65106492023265e-21	3.58814956789688e-20	UP
CNKSR2	-2.21678543098103	2.90955325490198e-24	1.64707577462965e-23	DOWN
CNN1	-4.13369269506301	1.30196949293684e-55	3.48251839946377e-54	DOWN
CNN2	1.16184031260956	1.88364545729187e-58	5.64542484472001e-57	UP
CNNM1	-1.04786212849387	1.97217301440274e-05	3.48892092855785e-05	DOWN
CNNM2	-1.69246852887148	9.4406608590661e-120	2.98891322798033e-117	DOWN
CNNM4	-1.61803826521829	1.40228686691079e-79	1.1200927162164e-77	DOWN
CNPY2	1.78615866208782	1.69309513686199e-39	2.06022820532339e-38	UP
CNR1	-3.93332033683123	1.41427889095711e-43	2.08525303372025e-42	DOWN
CNR2	-2.4060683069397	6.96958168350187e-23	3.64667445477218e-22	DOWN
CNRIP1	-1.50575539964127	1.25756668024928e-30	9.74544219100168e-30	DOWN
CNST	-1.0111731808401	1.87452040102074e-60	6.20551782185819e-59	DOWN
CNTFR	-3.71093552969754	1.4047195709935e-27	9.41508155877978e-27	DOWN
CNTN1	-2.93718424380527	1.99004688301692e-23	1.07283858742953e-22	DOWN
CNTN2	-4.02147845210323	2.13981914301187e-49	4.20549331120379e-48	DOWN
CNTN3	-2.61424395486924	3.76998900726945e-22	1.89238450803064e-21	DOWN
CNTN4	-2.50972305816459	6.33782718446348e-53	1.47343904890604e-51	DOWN
CNTN6	-1.73811476285412	4.94813397255135e-08	1.0679456725835e-07	DOWN
CNTNAP1	-1.11896384036009	1.88274240220861e-11	5.1089595526973e-11	DOWN
CNTNAP3	-2.81944096866052	6.10773212606944e-32	5.11071309520649e-31	DOWN
CNTNAP3B	-2.06924771819059	2.71366907257132e-14	8.71989657883085e-14	DOWN
CNTNAP5	-1.50424811903327	0.00229237645622844	0.00335974675804274	DOWN
COCH	1.47049679871198	3.60178150639669e-11	9.6059913795904e-11	UP
COL10A1	7.77037626448865	1.09541276776946e-145	6.35814084172318e-143	UP
COL11A1	6.22971212917602	2.27429310066993e-139	1.10006293783237e-136	UP
COL12A1	1.71355672943137	4.05067951507319e-22	2.02860173701379e-21	UP
COL14A1	-1.58637921731605	3.06842229669965e-17	1.17558718267175e-16	DOWN
COL17A1	-1.02741936599872	1.72176511098896e-07	3.57215487640304e-07	DOWN
COL19A1	-3.01966220058618	2.7228466366553e-31	2.19301241832002e-30	DOWN
COL1A1	2.64851654005106	1.91449696404453e-40	2.46030521290829e-39	UP
COL1A2	1.51735235150571	2.61318570094399e-16	9.45034322129546e-16	UP
COL21A1	-2.2749298867448	3.1933883297511e-33	2.85307701313268e-32	DOWN
COL22A1	1.73309707425301	5.98036827870795e-16	2.11486906655446e-15	UP
COL23A1	-1.10375216405679	3.38706571122514e-12	9.637087455811e-12	DOWN
COL25A1	-1.41252123693087	7.05426781610949e-09	1.61350276476966e-08	DOWN
COL26A1	1.33810804783021	1.36886777329866e-07	2.86457090931974e-07	UP
COL27A1	1.96927861614768	5.82945445454479e-30	4.3962014039406e-29	UP
COL28A1	-1.55065577767747	3.23508814292816e-13	9.80208627680668e-13	DOWN
COL3A1	1.28053877802292	6.89676996209378e-12	1.92828284120005e-11	UP
COL4A1	1.0580769646793	1.26610887874617e-14	4.14880577824747e-14	UP
COL4A5	-1.19588409920002	5.2587617905714e-09	1.21366228044029e-08	DOWN
COL4A6	-2.16523379999246	5.99577550266621e-22	2.9727915383806e-21	DOWN
COL5A1	1.52015411538434	8.47578243586749e-20	3.74781106032911e-19	UP
COL5A2	1.6890018040417	3.55696775143689e-22	1.78803347158691e-21	UP
COL5A3	1.24440783486979	5.89932385475012e-16	2.08790500574723e-15	UP
COL6A5	-2.7675653623988	1.76205338089729e-18	7.22965021714525e-18	DOWN
COL6A6	1.33985364303613	3.49788030299964e-06	6.58400061789349e-06	UP
COL7A1	3.24040286036553	3.63596888372761e-68	1.74898138597649e-66	UP
COL8A1	1.62481543322875	8.56806337445895e-15	2.84453169760636e-14	UP
COL9A1	1.51127033284241	1.57512564268668e-09	3.76546716311135e-09	UP
COL9A2	1.05125575840604	8.33145246797727e-08	1.76579099329373e-07	UP
COL9A3	3.12037990275328	2.18855102249291e-32	1.87822764685407e-31	UP
COLEC11	-1.02951602854979	3.37518674433437e-08	7.37139430315996e-08	DOWN
COLEC12	-1.83576461856486	4.47347902487761e-18	1.79692480415672e-17	DOWN
COMP	6.00597355483325	4.77113225866949e-81	3.99421759712557e-79	UP
COMT	1.18068739810624	6.39932428685791e-24	3.5532982719087e-23	UP

GeneSymbol	log2FoldChange	pvalue	padj	change
COQ8A	1.49075100564214	1.43528451398876e-17	5.59619553114338e-17	UP
CORIN	3.34129491954466	1.79917907883411e-54	4.55364902612475e-53	UP
CORO2B	-2.02173555804635	1.07019154457258e-20	4.97736254424206e-20	DOWN
CORO6	-1.78801032911957	2.12072242721303e-26	1.34235331243404e-25	DOWN
COX20	1.08021701344292	2.92515860757265e-14	9.38562499238299e-14	UP
COX7A1	-1.60105170354132	1.50857369688388e-24	8.65813901906361e-24	DOWN
CP	-2.14038804390725	4.46146439081744e-14	1.4161042551459e-13	DOWN
CPA3	-1.95355470744863	1.25723145402693e-19	5.50746448024427e-19	DOWN
CPA4	3.68687511043453	9.04502994768413e-35	8.75492531845601e-34	UP
CPA5	1.22303435465805	9.24314764757693e-05	0.000153916926448558	UP
CPAMD8	-1.51884507592578	1.15380920164298e-25	7.02738007282586e-25	DOWN
CPB1	-3.1317056590394	9.60990495353431e-11	2.4975712679984e-10	DOWN
CPB2	2.5639281212282	2.66591656741714e-11	7.17046728273628e-11	UP
CPEB1	-3.14273390829122	2.6168638363113e-42	3.64248201292476e-41	DOWN
CPEB2	-1.12544449728836	1.8362633089784e-33	1.66535692704379e-32	DOWN
CPEB3	-1.45876316303448	8.91615547997264e-68	4.18482521220387e-66	DOWN
CPED1	-2.55120208058203	4.18492859242702e-50	8.53303999765009e-49	DOWN
CPLX1	1.6907299736119	2.21792271262807e-20	1.0141987446164e-19	UP
CPM	-2.62853874572369	1.57031465767825e-61	5.42537482820464e-60	DOWN
CPN1	3.49842829310773	3.88199285222826e-17	1.47559793791423e-16	UP
CPNE1	1.92017639793337	2.62203795452417e-44	3.98060565842454e-43	UP
CPNE4	-1.65785701758464	2.71239253043636e-06	5.15452265988086e-06	DOWN
CPNE5	-1.97333626716656	2.47279702826366e-41	3.30205633843214e-40	DOWN
CPNE7	5.82033689323545	2.76715364281487e-173	3.44174617016681e-170	UP
CPNE8	-2.2029800382335	1.30092538132191e-47	2.33777230804525e-46	DOWN
CPNE9	1.80604568087583	9.8229008999484e-18	3.8680726678155e-17	UP
CPQ	-1.29376999438539	2.58829129870907e-25	1.54085184220243e-24	DOWN
CPS1	2.51570578739002	6.5373492251186e-14	2.05849660139223e-13	UP
CPSF4L	1.08914341948894	1.95007109622285e-08	4.32567999981255e-08	UP
CPT1A	-1.21899001952733	6.230703042754e-51	1.32472810846734e-49	DOWN
CPT1B	1.4692067504603	1.64602631768232e-13	5.08196033152522e-13	UP
CPT2	-1.18199402072178	1.13231371374028e-50	2.38127762045404e-49	DOWN
CPXM1	1.70326490552053	7.42237559637293e-24	4.09913816237367e-23	UP
CPXM2	-2.46183275245055	2.181542624551e-28	1.51162760530468e-27	DOWN
CPZ	1.90239284741385	7.20445492640188e-17	2.69093036536757e-16	UP
CR1	-1.76462414863789	4.27047204510061e-14	1.35696587082732e-13	DOWN
CR2	-2.91571730115638	4.83460420703343e-19	2.05279110112346e-18	DOWN
CR589904.2	-1.79792878361332	2.6240515049592e-09	6.16218595493656e-09	DOWN
CRABP1	-1.13241127009372	0.00068369856085637	0.00105514872287441	DOWN
CRABP2	1.21765638977013	2.93252002051245e-07	5.9849942706497e-07	UP
CRACD	-2.1206760732977	3.4773412087803e-43	5.02080783320824e-42	DOWN
CRB1	-2.16300196449231	5.60168776905703e-19	2.3658061877902e-18	DOWN
CRCT1	1.95933309932278	0.0131167105493735	0.017690409116373	UP
CREB3L3	-4.20631519738451	1.29210796337506e-49	2.55966734542092e-48	DOWN
CREG2	2.4624946389403	1.49434967032194e-18	6.15447275527812e-18	UP
CRHBP	-2.67709079663904	1.39020290953399e-40	1.79982180399371e-39	DOWN
CRLF1	-1.04523158047393	5.56955191002452e-07	1.11551193247363e-06	DOWN
CRLS1	2.36870927257394	3.949553953032e-25	2.33209843961161e-24	UP
CROT	-1.27499753181114	1.2523629685338e-36	1.32809965719118e-35	DOWN
CRP	2.25952057269403	0.000554739714209781	0.000862240707269028	UP
CRTAC1	-2.04877183300395	8.39646852286036e-12	2.33707970569961e-11	DOWN
CRYAB	-2.46162286749915	6.75264643292005e-39	7.97178524314826e-38	DOWN
CRYBA2	-1.78302869563037	1.05768534689336e-08	2.38722941613144e-08	DOWN
CRYBG3	-1.03818882816727	6.95153716528639e-14	2.18338955012864e-13	DOWN
CRYGN	-1.16110634623103	1.68321224184085e-06	3.25374941909133e-06	DOWN
CSAG1	6.23083748409891	9.44258837731754e-18	3.72252187942563e-17	UP
CSAG3	4.52828683382745	9.83330377649077e-07	1.93389788412055e-06	UP
CSE1L	1.54784278013705	6.55168642503627e-78	4.91743602237744e-76	UP
CSF1	-1.01757509876804	7.41383111849368e-17	2.76827575658298e-16	DOWN

GeneSymbol	log2FoldChange	pvalue	padj	change
CSF1R	-1.29711135846076	7.56289204988567e-15	2.52236428394291e-14	DOWN
CSF2	3.67872089330774	8.85270182879147e-36	8.99370460587782e-35	UP
CSF2RB	-1.3196470444134	3.19159246437795e-14	1.02179076268088e-13	DOWN
CSF3	2.30529915623353	1.54587053215883e-15	5.3377441158996e-15	UP
CSMD1	-1.69635058930383	3.44633155494089e-08	7.51922959105197e-08	DOWN
CSMD2	1.71978218474545	4.30110728584048e-16	1.53316645175722e-15	UP
CSMD3	-1.02213146901544	0.00460209897517991	0.00653214455940721	DOWN
CSN1S1	-1.48250729752024	0.000170288150424817	0.000277149973207527	DOWN
CSNK1A1L	1.1093147380807	0.00420302266329034	0.00599305876481123	UP
CSNK1E	1.06861661032205	1.83220981631184e-50	3.82087060256743e-49	UP
CSPG4	-1.35715483321721	9.27405944058101e-18	3.65856812503029e-17	DOWN
CSRNP3	-1.34523808861604	1.64009516567416e-10	4.18937613611326e-10	DOWN
CSRP1	-2.34255204578448	2.08929411651436e-105	4.72478940920319e-103	DOWN
CST1	8.36374209086429	1.66074638583869e-160	1.70109275391818e-157	UP
CST2	6.33747317856882	4.43230490929913e-82	3.9579346351603e-80	UP
CST4	6.4722672720376	2.44426465929834e-53	5.83840610594816e-52	UP
CST5	3.68426038404444	1.44999055414348e-17	5.64973943148366e-17	UP
CST6	2.96122760561315	2.43334353348819e-21	1.16952279736765e-20	UP
CSTL1	3.42546913668773	6.2243292117741e-23	3.26556928486358e-22	UP
CT83	4.8678914479479	5.50689366959786e-06	1.0209392651618e-05	UP
CTAG2	3.37403190839801	0.000772076804085931	0.00118471742946319	UP
CTHRC1	3.74138761237603	9.39581672993779e-83	8.79620197410789e-81	UP
CTLA4	1.02208374375703	3.93943934272061e-09	9.16098521297997e-09	UP
CTNNA3	-4.11271542276875	1.05062591293439e-45	1.71779803022784e-44	DOWN
CTNND2	-3.24892426889637	7.30977729114598e-32	6.09603218250599e-31	DOWN
CTPS1	1.10618414490006	2.95619941390586e-48	5.47620216961092e-47	UP
CTSF	-1.10059052516879	1.28989377659477e-12	3.76671479655288e-12	DOWN
CTSG	-3.34361493369169	1.77983534325746e-30	1.36952155687769e-29	DOWN
CTSH	1.07193058171753	6.77047354841464e-21	3.18374982172682e-20	UP
CTSS	-1.20178183664495	2.29636204809375e-37	2.52440355703639e-36	DOWN
CTSV	1.46106919036079	6.69435946759722e-19	2.81159868329162e-18	UP
CTSW	-1.39397382043467	1.05671233816002e-11	2.92211083760211e-11	DOWN
CTTNBP2	1.27151189190775	8.23826175204596e-11	2.14557062351744e-10	UP
CTU1	1.90411954190419	3.33058423398547e-29	2.41346081008694e-28	UP
CTU2	1.29637765106873	2.30519314914792e-41	3.08771756200867e-40	UP
CUX2	-2.57398501721003	3.31604423119583e-22	1.6707835126682e-21	DOWN
CUZD1	1.26121623772942	7.99508839439584e-05	0.000133928306119879	UP
CWH43	-3.84300966500158	3.91967223330598e-30	2.97659191445953e-29	DOWN
CX3CR1	-1.66163700051121	1.72689352713017e-22	8.8390349758723e-22	DOWN
CXCL1	2.86964713167652	3.28759228754612e-50	6.77477449740125e-49	UP
CXCL10	1.74283976716824	8.69621264204668e-13	2.56612694011115e-12	UP
CXCL11	2.50900659058006	2.63567102733894e-21	1.26502038586144e-20	UP
CXCL12	-2.79895823180581	4.59504163071537e-64	1.7980552790033e-62	DOWN
CXCL13	-1.96081973943918	6.83834798065872e-13	2.02890021106168e-12	DOWN
CXCL17	4.62100523932397	4.250407110124e-31	3.37955885883969e-30	UP
CXCL2	2.31750801593383	6.23694186579277e-34	5.75842357948301e-33	UP
CXCL3	2.89545545397581	2.31301005329799e-55	6.13970183812163e-54	UP
CXCL5	5.05431724643042	7.34609648167095e-49	1.40568767071798e-47	UP
CXCL6	2.18483502934683	2.69170726836366e-19	1.15930493851141e-18	UP
CXCL8	3.31711496198015	2.14739023778923e-35	2.13915939420045e-34	UP
CXCR5	-2.74949689837947	3.46567567577558e-18	1.39920729288848e-17	DOWN
CXorf21	-1.52841056317726	4.04803170407685e-20	1.82470556725576e-19	DOWN
CXXC5	1.05012863975779	3.18208712059031e-30	2.42175188071849e-29	UP
CYBRD1	-2.16334938005272	1.37338807108404e-39	1.68295612116724e-38	DOWN
CYP11A1	-1.83828087781333	1.02263971402117e-15	3.56643808136405e-15	DOWN
CYP17A1	1.60919183893961	0.000824105302955273	0.00126144036922997	UP
CYP19A1	1.38179321720209	2.50516640094354e-12	7.19729786431352e-12	UP
CYP1A1	1.81674197608901	0.000257229085179659	0.000411987680301085	UP
CYP24A1	2.56396227843976	9.13337188552807e-15	3.02528827549363e-14	UP

GeneSymbol	log2FoldChange	pvalue	padj	change
CYP26A1	2.95768146993619	1.21117395955219e-13	3.76207138025015e-13	UP
CYP26B1	-1.33745271908669	2.55245224239994e-13	7.79204959623249e-13	DOWN
CYP26C1	1.01403049423378	0.0033632859659	0.0048408744027291	UP
CYP27B1	1.92993281786787	4.17077959182748e-67	1.91624762618712e-65	UP
CYP27C1	-1.05280832432854	1.56452643263123e-07	3.25523942781786e-07	DOWN
CYP2C18	-2.04553655010344	2.72751681660977e-20	1.24103084211199e-19	DOWN
CYP2C8	-1.74229155446556	3.60385991857357e-17	1.37377435994137e-16	DOWN
CYP2F1	1.84284149364657	5.91649118307243e-15	1.98122809559308e-14	UP
CYP2S1	1.34413406860918	2.00796020795581e-25	1.20443028250549e-24	UP
CYP2W1	4.0383497448886	1.04688336407187e-34	1.00937873857052e-33	UP
CYP39A1	1.19839532947418	1.77531383233616e-14	5.75563950148382e-14	UP
CYP3A4	-2.64335248145499	7.34505433083621e-23	3.83967070137649e-22	DOWN
CYP4B1	-2.80752383726738	5.1693755500376e-32	4.34641894991814e-31	DOWN
CYP4F12	-1.17528143428337	2.11663455290542e-14	6.83548914498185e-14	DOWN
CYP4F3	1.27525118120527	3.95049133203506e-14	1.25850540733126e-13	UP
CYP4F8	2.70376474640331	8.2535295462783e-18	3.26336762010318e-17	UP
CYP4X1	2.89476507898352	1.29317011239957e-28	9.07616733865926e-28	UP
CYP4Z1	2.37092766967192	9.75081327884745e-10	2.36444661780491e-09	UP
CYP8B1	-1.8064797432974	7.46677716649269e-14	2.34015462203271e-13	DOWN
CYRIA	-1.63328122431464	7.68325421213181e-26	4.74259147805215e-25	DOWN
CYS1	-2.21491190036474	5.88868160106307e-35	5.75418702128571e-34	DOWN
CYSLTR1	-1.88403010264369	2.78734324027096e-34	2.61508662946327e-33	DOWN
CYSLTR2	-1.44731304877591	2.50324361386636e-15	8.53681571646198e-15	DOWN
CYSRT1	2.67389768473272	2.98909734293244e-49	5.82205280005398e-48	UP
CYTL1	-1.18830258257412	4.01471426491916e-08	8.72435036753244e-08	DOWN
CYYR1	-1.04552522359638	2.75780465604993e-17	1.0572798871818e-16	DOWN
DAAM2	-1.89410840708405	9.31968213573702e-44	1.38467256851185e-42	DOWN
DACH1	2.1499018787846	5.50368773356093e-34	5.08952280958558e-33	UP
DACH2	-1.43535661139517	3.83846773063612e-05	6.62036832345155e-05	DOWN
DACT3	-2.61544224241464	2.32700214027922e-46	3.94164282769281e-45	DOWN
DAND5	-3.15239679740856	1.49465715113873e-46	2.57178507636154e-45	DOWN
DAO	-5.62814728843752	4.90628508795846e-82	4.33670772774724e-80	DOWN
DAPL1	1.71774631576746	6.59387481072574e-09	1.51157375038398e-08	UP
DAW1	1.7933756278167	7.02864511593463e-07	1.39794171791856e-06	UP
DBF4	1.0530435730622	1.1077611829263e-30	8.61905517350116e-30	UP
DBF4B	1.17404748282254	2.15284234549841e-43	3.14491977870501e-42	UP
DBNDD1	2.47062290511593	8.35032548936058e-77	5.9348660304586e-75	UP
DBX1	1.74361349876298	4.33961389966455e-05	7.44783134583667e-05	UP
DCAF12L2	-2.54200799152744	2.18912426588313e-14	7.0643478209457e-14	DOWN
DCAF13	1.27684428682128	2.86969971689839e-47	5.08860297050424e-46	UP
DCAF4L1	1.34948205462122	1.47661241280936e-15	5.10366255344372e-15	UP
DCDC1	1.46677306223066	2.40290672254711e-05	4.21877543453446e-05	UP
DCDC2	1.46992621780687	4.42389427917655e-07	8.91588785686356e-07	UP
DCHS2	-1.36516470387092	3.9761535074786e-05	6.84698981662628e-05	DOWN
DCLK1	-2.9823381283131	2.74649948580398e-43	3.98872356516302e-42	DOWN
DCLK2	-1.38552144070999	3.02430396862067e-21	1.44795724513587e-20	DOWN
DCLK3	1.15676589227177	4.06843528858618e-15	1.37560512000294e-14	UP
DCN	-1.888729611092	1.90235917084786e-24	1.08573517672808e-23	DOWN
DCSTAMP	2.92142285108565	1.95352785875185e-21	9.43861836971311e-21	UP
DCUN1D5	1.25550173722494	4.40241037770607e-58	1.30594841408852e-56	UP
DCX	-1.58983645257036	1.89381735242457e-07	3.92163652726472e-07	DOWN
DDIAS	1.8549416532075	2.862994618815e-64	1.13046089109809e-62	UP
DDIT4	1.78104304847486	2.1335880357003e-34	2.01585287388222e-33	UP
DDN	4.57699907865786	1.33513936190576e-127	5.05408298018806e-125	UP
DDR2	-2.14749197182772	5.28208950125056e-29	3.78039558098134e-28	DOWN
DDX11	1.2292311424406	9.22942692989556e-36	9.37096274812078e-35	UP
DDX21	1.09402156925481	6.00473542695596e-39	7.10329198298805e-38	UP
DDX25	-1.88554499143473	1.42651870121002e-12	4.15662151006863e-12	DOWN
DDX27	1.20314593787313	1.10055864446896e-34	1.05878605945513e-33	UP

GeneSymbol	log2FoldChange	pvalue	padj	change
DDX31	1.12791326425493	1.27020560955618e-105	2.91027503673708e-103	UP
DDX39A	1.16166206848191	8.52494751044927e-36	8.67591531265068e-35	UP
DDX53	3.05428509647281	0.00642417266923498	0.00897106226183539	UP
DDX55	1.07324908684315	1.29065460494437e-62	4.71156575176023e-61	UP
DDX56	1.0858357691813	1.05975949326239e-75	7.20843439694453e-74	UP
DDX60	-1.55189470476951	1.41286182388195e-24	8.12220631867163e-24	DOWN
DEFA5	2.05966988303028	3.84619819216387e-06	7.2224575779305e-06	UP
DEFA6	3.35520076809064	7.08242916141152e-16	2.49194461482438e-15	UP
DEFB1	-1.94010591524065	8.07279335039441e-15	2.68419993527626e-14	DOWN
DEFB126	2.23491218481295	0.0185387133772137	0.0245076386302325	UP
DEFB4A	3.9033942648201	1.57212069831183e-09	3.75879963197912e-09	UP
DENND1C	-1.08107580031501	1.19012699595535e-38	1.38898668770579e-37	DOWN
DENND2A	-1.38591987226247	1.67500704160742e-27	1.11836263863152e-26	DOWN
DENND5B	-1.20569912878606	9.04544435385615e-21	4.22614227350945e-20	DOWN
DEPDC1B	1.10151233774447	1.31101652875962e-27	8.80059013696658e-27	UP
DERL3	-1.18305300549087	5.25049371619475e-10	1.29646691832245e-09	DOWN
DES	-3.71401229349583	8.08660686513676e-28	5.49403376288047e-27	DOWN
DGAT2	2.03666968609993	5.38420969727695e-59	1.6593848399767e-57	UP
DGCR6	1.20711896330251	0.00130245222314038	0.00195614978105428	UP
DHCR7	1.34947814039558	9.12088900556273e-37	9.7317426626142e-36	UP
DHDDS	-1.23211514986351	3.66452290248949e-81	3.08262499038886e-79	DOWN
DHDH	1.25984185640216	4.49437788282313e-11	1.19172532470838e-10	UP
DHH	-1.06994758313814	5.84970302044971e-10	1.44013684002673e-09	DOWN
DHPS	1.57255862810234	1.56762362480808e-29	1.15714413644693e-28	UP
DHRS11	-2.2245696402261	1.31908921686946e-61	4.59386010666957e-60	DOWN
DHRS2	3.88968796674083	1.19222522199328e-56	3.35925854216328e-55	UP
DHRS7C	-5.26339265653419	2.88208134819381e-72	1.64543221364258e-70	DOWN
DHRS9	-3.31281279418833	3.09888099606606e-33	2.77148509422179e-32	DOWN
DHX34	1.40159303660834	1.71832601597965e-51	3.74483240503799e-50	UP
DIAPH3	1.57107456197264	1.18148962152529e-57	3.47521601007091e-56	UP
DIO2	1.97825519485936	2.00350370404312e-31	1.62643403256424e-30	UP
DIP2C	-1.21122067472841	7.56706471763192e-23	3.95335427327107e-22	DOWN
DIRAS1	-1.81651550512737	8.9389985583491e-15	2.96315975436004e-14	DOWN
DIRAS2	-2.28112355683025	8.04467794227129e-15	2.67536243332257e-14	DOWN
DISP2	-2.2485267394218	7.69727380251635e-43	1.10043209132362e-41	DOWN
DIXDC1	-1.83379514919456	2.28596686233601e-61	7.83573641217657e-60	DOWN
DKC1	1.46210258285833	2.71178043688936e-98	5.02342901569728e-96	UP
DKK1	3.00057844809954	6.73411296109275e-18	2.67475157371141e-17	UP
DKK4	7.73418000650511	3.33899635300548e-72	1.90006351290472e-70	UP
DLC1	-1.00481788959652	1.17492788355769e-14	3.86019230875284e-14	DOWN
DLG2	-2.03984081939061	3.85103058792004e-34	3.58790773822642e-33	DOWN
DLGAP2	-2.16739597748496	1.2093578972591e-21	5.90536990044103e-21	DOWN
DLGAP5	1.1122133661035	1.17157196603096e-25	7.12808617906954e-25	UP
DLK1	1.77060781802826	0.0147123044010927	0.019706565887402	UP
DLL3	2.71557878452453	3.74073423166583e-21	1.78117049975382e-20	UP
DLX3	3.22137543862168	5.97244249761202e-19	2.51811479929584e-18	UP
DLX4	2.4509618331933	2.2982557997005e-42	3.20156225921478e-41	UP
DLX5	2.24901545268323	3.31368742076233e-12	9.43755954493531e-12	UP
DLX6	4.05399516277689	7.2395703740822e-27	4.68808623740771e-26	UP
DMBX1	6.34031676071634	4.93641515192842e-63	1.84063805225974e-61	UP
DMD	-2.35923860496721	7.03178181865318e-38	7.8945465382468e-37	DOWN
DMGDH	-1.2618498713888	3.3871756134742e-14	1.08261543607611e-13	DOWN
DMKN	1.70393014119438	2.39922567672279e-14	7.7280275080973e-14	UP
DMPK	-1.23684071266233	2.23785009716517e-23	1.20382093734745e-22	DOWN
DMRT1	4.80124364444969	2.15031792881869e-15	7.36206962141561e-15	UP
DMRT3	3.92440925434482	3.83984320761722e-21	1.82686310858576e-20	UP
DMRTA1	-3.44652219976532	9.40301987000585e-37	1.00143599386185e-35	DOWN
DMRTA2	5.18650735665786	1.1357952177682e-36	1.206687134045e-35	UP
DNA2	1.30243781231592	8.82242984133635e-36	8.96818276866257e-35	UP

GeneSymbol	log2FoldChange	pvalue	padj	change
DNAAF3	2.08252035563201	1.76161816099378e-20	8.08728105388471e-20	UP
DNAAF4	1.59779394317258	1.48914439971953e-16	5.47402816810559e-16	UP
DNAAF5	1.02022091834385	1.04469150679755e-40	1.36059934239834e-39	UP
DNAH10	1.26577979183597	1.86707495065329e-11	5.06802433604455e-11	UP
DNAH12	1.8722475453039	3.42658106824358e-17	1.30820118704945e-16	UP
DNAH14	1.69906057384057	2.43315135140768e-25	1.44948561348142e-24	UP
DNAH2	2.82006884108038	1.12602149918932e-46	1.94711145634396e-45	UP
DNAH5	2.29033938062518	9.53227282644966e-34	8.745282756953e-33	UP
DNAI4	-2.32181566018962	1.51518287015651e-58	4.5646850031203e-57	DOWN
DNAJB4	-1.19454416014446	3.04991681850431e-23	1.6290859374422e-22	DOWN
DNAJB5	-2.56089256902377	3.13471483131032e-54	7.87659298089561e-53	DOWN
DNAJC2	1.11696976329608	5.72472130341992e-37	6.1685997559685e-36	UP
DNAJC28	-1.58989167887146	4.16066862440859e-45	6.59833540590408e-44	DOWN
DNASE1	1.13472182604485	9.24103640162977e-16	3.2292628308565e-15	UP
DNASE1L2	2.56274666776547	1.52883331043097e-40	1.97489424588535e-39	UP
DNASE1L3	-3.26983655545801	5.60661054223489e-36	5.75297049923018e-35	DOWN
DNER	-2.62877729912862	4.65301304542404e-21	2.20051374687585e-20	DOWN
DNMT3B	1.69633255144403	1.64187073112315e-41	2.21284017345568e-40	UP
DNTTIP1	1.29966806611605	2.70179756904685e-33	2.42257472038171e-32	UP
DOCK10	-1.37473532420375	6.98607146070696e-21	3.28424574366334e-20	DOWN
DOCK2	-1.34729073889161	7.31546843407096e-16	2.57030370949309e-15	DOWN
DOCK3	-1.78894058080914	2.97410782803692e-21	1.42470810480349e-20	DOWN
DOK7	2.10097684931839	9.05955781068812e-28	6.1359035456053e-27	UP
DPCD	1.06789403667553	9.69040709738201e-29	6.85373918711263e-28	UP
DPEP1	5.47538820220026	5.38856183148288e-99	1.05428120417541e-96	UP
DPEP2	-1.31388910179068	7.937218520576e-15	2.64214846298585e-14	DOWN
DPEP3	-2.43992855692179	2.50535020413558e-21	1.20346656840311e-20	DOWN
DPF1	1.55238706746246	5.09326707712477e-17	1.9234235440029e-16	UP
DPF3	-2.34731446136179	2.88300677955068e-39	3.48140062776116e-38	DOWN
DPP10	-1.84833710772422	2.0424940781527e-06	3.91868106906929e-06	DOWN
DPP6	-5.25114588043206	1.36470813034454e-70	7.18204017397465e-69	DOWN
DPP7	1.06142569913105	1.93743646829464e-20	8.8733774914294e-20	UP
DPT	-4.01594780023551	1.14739732354696e-55	3.08804166845799e-54	DOWN
DPY19L2	-1.4970941638862	4.50099823997976e-12	1.27151009657313e-11	DOWN
DPYD	-1.78789071728256	6.46671850349729e-23	3.38967397054179e-22	DOWN
DPYSL3	-1.08314515220496	1.6470110302567e-09	3.93137807674569e-09	DOWN
DPYSL5	-2.90714251177143	4.64954273509056e-20	2.08881547074644e-19	DOWN
DQX1	-1.00355296158094	2.23682869067663e-08	4.93911970463508e-08	DOWN
DRC1	1.18237473053552	2.01554345850609e-05	3.56311251197629e-05	UP
DRC7	1.01606911984259	0.00199463219278529	0.00293969787329415	UP
DRD2	3.23612663628555	3.95661598533881e-22	1.98320535845437e-21	UP
DRD4	1.05905083360967	4.96882461271276e-09	1.14857484376964e-08	UP
DRD5	-2.63782981462438	7.54014719896815e-07	1.497281140103e-06	DOWN
DSC2	-1.64098528236691	1.71521946467092e-47	3.06329400392972e-46	DOWN
DSC3	4.30126675505708	4.8656145072004e-38	5.50519463378041e-37	UP
DSCAML1	-2.35961053643265	2.20046442135364e-19	9.5267744826034e-19	DOWN
DSCC1	1.84193507495424	3.25513105083035e-71	1.78244015685877e-69	UP
DSEL	-1.6060618403363	3.93009914291696e-20	1.77246351659189e-19	DOWN
DSG1	2.31099964767005	6.36565471220833e-30	4.79019643490422e-29	UP
DSG3	5.34243024849503	2.49686749092003e-53	5.95588405745075e-52	UP
DSG4	2.18200128553652	7.55062151748023e-39	8.9017584620097e-38	UP
DSN1	1.21244497018618	2.60255946540119e-45	4.1729620599476e-44	UP
DST	-1.19448142559085	3.13804572168633e-21	1.50035118483592e-20	DOWN
DTD1	1.19925099836825	1.46979482367657e-30	1.1349683931122e-29	UP
DTL	1.23526289271776	5.61485354460211e-39	6.6647201617012e-38	UP
DTNA	-1.81905123752553	1.37076929958586e-15	4.74820087799654e-15	DOWN
DTNB	1.38863542151617	3.97587495924853e-39	4.76147941302576e-38	UP
DTYMK	1.05834459225675	2.32943609833952e-32	1.99618458564892e-31	UP
DUOXA2	2.52622756104392	2.29266555636396e-15	7.82941465639647e-15	UP

GeneSymbol	log2FoldChange	pvalue	padj	change
DUS3L	1.11939388069907	6.79714499657238e-25	3.96644389495023e-24	UP
DUS4L	1.07085627061098	3.76668145622241e-32	3.18239806876277e-31	UP
DUSP1	-1.16263387940427	3.99968681744575e-13	1.20620967357435e-12	DOWN
DUSP14	1.61966651032228	1.36782609674014e-64	5.5006826379991e-63	UP
DUSP15	2.45913812252153	7.80285761981464e-26	4.81130169029152e-25	UP
DUSP2	1.39300964880438	1.69199324967219e-22	8.67059401310823e-22	UP
DUSP26	-2.69447996731839	1.57732348228899e-32	1.36104726447464e-31	DOWN
DUSP4	3.1615313598017	1.08708931313122e-40	1.41475980639417e-39	UP
DUSP9	4.43483935664035	4.06232782503966e-41	5.39156359888839e-40	UP
DXO	1.06921460140598	5.3088107669871e-35	5.19630814421283e-34	UP
DYDC2	1.45636010642231	0.0018282166352893	0.00270703539713372	UP
DYNC1I1	-2.2009308091356	2.23678331700047e-19	9.67199103524441e-19	DOWN
DYNLRB1	1.16616945460923	2.29362622962386e-16	8.33390954608394e-16	UP
DYRK2	-1.03232804472333	1.92198879446173e-49	3.78164868677538e-48	DOWN
DZIP1	-1.4737548446755	1.93554913859093e-24	1.10395405012394e-23	DOWN
E2F1	1.89675399305655	5.03841352395808e-64	1.96272695061929e-62	UP
E2F5	1.41880548578642	3.22456953723479e-44	4.8656351258119e-43	UP
E2F7	1.81290137744431	2.16451477192755e-57	6.32394223549906e-56	UP
EAF2	-1.32224050539721	1.89950018450187e-46	3.24274477575794e-45	DOWN
EBF1	-1.5744858381357	9.5022900607605e-26	5.82207518747441e-25	DOWN
EBI3	-1.29580118492696	1.36559704300039e-15	4.73122588733898e-15	DOWN
EBPL	1.12015477512511	3.98078118529365e-37	4.31883755635628e-36	UP
ECE1	-1.06841513240702	6.29899353170495e-35	6.14822726275663e-34	DOWN
ECEL1	1.91153283956529	8.44469703034611e-11	2.19801957233807e-10	UP
ECI2	-1.1502109913121	1.32217069942556e-19	5.78466291183347e-19	DOWN
ECM2	-1.02009491378156	3.83407249858461e-08	8.34533805223173e-08	DOWN
ECRG4	-4.26447130153924	4.31579894964305e-48	7.94408108986623e-47	DOWN
ECT2	1.73142855721747	5.13804555039082e-59	1.5891436442088e-57	UP
EDAR	2.86729493511441	5.81598439627758e-32	4.87596226732698e-31	UP
EDIL3	-2.18287150790351	2.82501574760333e-51	6.11840786231552e-50	DOWN
EDN2	-2.73988110725161	2.31425755656248e-35	2.30012367764968e-34	DOWN
EDN3	-3.21999341003455	2.1975972260654e-44	3.3744938710297e-43	DOWN
EDNRA	1.76485145524742	1.16275374379081e-25	7.07938144777253e-25	UP
EDNRB	-1.43155986616889	4.09009913702852e-26	2.55455151625099e-25	DOWN
EEF1A2	-1.26581985972382	7.39107628521627e-06	1.3563158536671e-05	DOWN
EEF1AKMT1	1.02517159866794	1.21128629114146e-20	5.61857437071022e-20	UP
EEF1AKMT4	1.83871995867318	6.61737432523233e-48	1.19904619277077e-46	UP
EEF1E1	1.17604426116241	1.19192571591202e-36	1.26554893238878e-35	UP
EEPD1	1.02585811449563	7.33053918531707e-21	3.44246706671861e-20	UP
EFCAB1	-1.59268389796216	9.24438611397816e-13	2.72373088667854e-12	DOWN
EFCAB5	-1.03849500616774	1.02333327016499e-13	3.1854312179805e-13	DOWN
EFCAB8	1.04402269864725	7.32238730626905e-06	1.34413588619084e-05	UP
EFEMP1	-1.47137515851073	1.0089188905887e-14	3.33301169452116e-14	DOWN
EFHC2	-2.48001925479492	7.89442128848493e-28	5.36862616094398e-27	DOWN
EFHD1	-1.16381732792275	1.41559256789242e-13	4.38217126839301e-13	DOWN
EFNA3	1.91302444695368	2.39799840204453e-37	2.6311497274607e-36	UP
EFNA5	-1.42172638664114	2.30640180960303e-10	5.82555478831123e-10	DOWN
EFS	-1.17986349239547	9.19764507305878e-15	3.04484018359644e-14	DOWN
EGFL6	3.44955149522809	2.81207374179479e-86	3.04140621527159e-84	UP
EGLN3	-1.00202989386149	3.95822981929965e-10	9.8604657859034e-10	DOWN
EGR4	2.20898379142212	5.0782960787972e-14	1.60866599272504e-13	UP
EHD2	-1.29455600239525	1.01336834548441e-16	3.75282496808168e-16	DOWN
EHHADH	-1.17838329959735	2.61394444745573e-33	2.35106480700137e-32	DOWN
EID3	-1.17879308528565	3.45096251621851e-21	1.64679666470027e-20	DOWN
EIF2S2	1.11457496568028	1.09159727956794e-48	2.07510736125727e-47	UP
EIF2S3B	1.05177969799077	6.56709678460005e-07	1.30928390554432e-06	UP
EIF3B	1.17571001362903	7.72013902264071e-59	2.35430439231598e-57	UP
EIF4A1	1.0644729936309	3.39396689462195e-15	1.15090838434376e-14	UP
EIF4E3	-1.94290323440578	3.58092521565075e-55	9.36256017719617e-54	DOWN

GeneSymbol	log2FoldChange	pvalue	padj	change
EIF4EBP1	1.52284422086751	2.79029482206599e-36	2.9094253734512e-35	UP
EIF4EBP3	1.4630190413946	6.42764702786347e-12	1.80000993400107e-11	UP
EIF5A2	1.10114017032557	3.29224939938799e-28	2.26771909776674e-27	UP
EIF6	1.08774971418397	2.66976573819798e-20	1.21634303504033e-19	UP
ELANE	-3.58825644072955	3.12951829753766e-59	9.81879317387807e-58	DOWN
ELAVL3	-3.53784938170313	1.76712378175446e-37	1.95246994998036e-36	DOWN
ELAVL4	-3.40663203664218	2.86251696644189e-55	7.54084840191416e-54	DOWN
ELF5	7.36642280733541	4.20826893706051e-48	7.75434783079733e-47	UP
ELFN1	1.39603168202167	2.12043093430981e-21	1.02279955288467e-20	UP
ELFN2	1.77396343540828	1.24527603459163e-09	2.99958384151945e-09	UP
ELMOD1	-1.4240627900351	2.15041827466547e-07	4.43243766770239e-07	DOWN
ELOVL4	-1.6159233511414	7.02107518099811e-22	3.4732381286e-21	DOWN
EMCN	-1.20845479137379	3.32184756442084e-17	1.26905071608732e-16	DOWN
EME1	1.82469533871893	5.67447537470643e-68	2.69361629740096e-66	UP
EME2	1.17539711490994	6.30664452638226e-20	2.81295084881901e-19	UP
EMILIN3	-2.62785102148319	2.77822697852584e-51	6.02456617398138e-50	DOWN
EML1	-2.41101665581132	4.54370226219287e-53	1.06630037050626e-51	DOWN
EML5	-1.73786046715982	3.35741144420608e-13	1.01603415846299e-12	DOWN
EMP1	-1.83306766571792	9.54160274717737e-35	9.21508200979476e-34	DOWN
EMX1	1.01901770955952	0.00583540750068357	0.00818527072735646	UP
EN1	2.70223716671022	1.20617746694448e-07	2.53355467212354e-07	UP
EN2	6.22117896607932	1.35387496263464e-48	2.55694411327083e-47	UP
ENAM	-1.59701095301383	8.1712551689859e-07	1.61762239947194e-06	DOWN
ENC1	1.98484397049654	1.31115401578117e-127	5.05408298018806e-125	UP
ENDOD1	-1.73507082388132	2.67389401890781e-98	5.0065071560475e-96	DOWN
ENDOG	1.05426892371734	1.37980050550568e-06	2.68722359941509e-06	UP
ENDOU	-1.1836541403031	1.28577110186887e-08	2.88296834880797e-08	DOWN
ENGASE	1.4904030732541	1.13975035130323e-27	7.68349704500314e-27	UP
ENHO	-2.18958042787407	1.20595001727555e-24	6.95568322319283e-24	DOWN
ENOX1	-1.49951923178469	1.23221457618829e-20	5.71412847274746e-20	DOWN
ENPP1	-1.26369937177723	9.47572040617774e-17	3.51439232018686e-16	DOWN
ENPP2	-1.47391868696589	1.19012434103895e-18	4.92247865807868e-18	DOWN
ENPP6	-3.48774216578027	1.4771462803604e-86	1.62794608733643e-84	DOWN
ENTPD3	-2.24899223737196	2.98855516130923e-21	1.4312351766743e-20	DOWN
ENTPD5	-2.56171181661388	2.99047959639841e-85	3.09959650071938e-83	DOWN
ENTPD8	-2.16231338870276	1.85882602117824e-19	8.07578281107204e-19	DOWN
ENTR1	1.21925050187231	7.24975835880871e-67	3.27896213771263e-65	UP
EPAS1	-1.22996015359406	5.65727074943081e-41	7.44596035977617e-40	DOWN
EPB41L3	-2.77978254547319	1.45144751800369e-49	2.86553918718801e-48	DOWN
EPB41L4A	-1.75081277428581	3.89642039272846e-22	1.9541580731158e-21	DOWN
EPB41L4B	-1.33630855936284	2.70073525055928e-40	3.44779346906076e-39	DOWN
EPDR1	1.03039442161019	4.26536981568117e-11	1.13255389753669e-10	UP
EPGN	1.94811403712576	1.24867291121031e-09	3.00651844619817e-09	UP
EPHA1	1.20930058419023	1.80621705910191e-26	1.14661529894792e-25	UP
EPHA10	-1.27637353690138	3.9315441016296e-24	2.20909898166106e-23	DOWN
EPHA3	-1.37620965418672	1.31691611629117e-13	4.0846919011361e-13	DOWN
EPHA4	-1.11571601174413	1.407422873418e-07	2.94277791724635e-07	DOWN
EPHA5	-2.99097947329862	1.06312209021943e-25	6.48411382031208e-25	DOWN
EPHA6	-4.56915292368821	2.25666581650219e-38	2.60061693333902e-37	DOWN
EPHA7	-3.58413444008487	8.85847000640403e-35	8.58389194332295e-34	DOWN
EPHA8	4.56062069866085	1.0687233743715e-16	3.95445816360623e-16	UP
EPHB2	1.07421858780446	8.65819688476342e-19	3.60682254436329e-18	UP
EPHB3	1.96741951297702	1.12416369115744e-29	8.34757456465861e-29	UP
EPHB4	1.23335457026296	5.42517393401072e-51	1.15770286412903e-49	UP
EPHX2	-1.50118818590337	2.23374866573134e-28	1.54657119349423e-27	DOWN
EPHX3	1.97293569300037	6.73649611330579e-17	2.52046856082926e-16	UP
EPHX4	4.41596707730574	4.77426708943916e-156	3.4639297011835e-153	UP
EPM2A	-1.78365948822338	9.5066448941376e-88	1.11850815906499e-85	DOWN
EPO	1.18430398709816	2.19567982000287e-05	3.87094995501772e-05	UP

GeneSymbol	log2FoldChange	pvalue	padj	change
EPOP	3.72051446070194	1.07203971445701e-119	3.33346920497142e-117	UP
EPPIN	2.04022045268651	0.000282092517412973	0.000450194941408862	UP
EPYC	6.89416952618178	8.40253795079274e-50	1.67598388702353e-48	UP
ERBB4	-2.09456550130861	4.37464069724242e-08	9.47811602103797e-08	DOWN
ERCC6L	1.65754362801712	1.32440982214919e-46	2.28336121119641e-45	UP
EREG	2.85147594242362	2.70024706235254e-27	1.78645144744471e-26	UP
ERFE	3.14616363779148	4.17552137644626e-41	5.52915237475732e-40	UP
ERICH2	2.32785982959389	1.00063498170455e-25	6.11370418821803e-25	UP
ERICH3	-3.00153077207507	1.97458263633323e-18	8.08450680612992e-18	DOWN
ERICH4	2.88797721374534	1.44106229825247e-14	4.70527241692674e-14	UP
ERP27	2.68421449020096	5.984835803577e-25	3.50888706557866e-24	UP
ERV3-1	1.06953865634595	8.51904828814787e-12	2.37044084118758e-11	UP
ERVFRD-1	-2.2658019040605	2.21732380869711e-15	7.58104446904433e-15	DOWN
ERVMER34-1	2.55161339437196	2.62038656457779e-22	1.32641835026143e-21	UP
ERVV-1	2.74884934027978	2.34833722582706e-08	5.1787735705834e-08	UP
ERVV-2	3.79245295637558	7.18507382623271e-11	1.87828916994173e-10	UP
ESM1	5.52650938552437	4.87108941289518e-253	1.41367133244573e-249	UP
ESPL1	1.103428571082	2.61439209311107e-32	2.23268315435719e-31	UP
ESR1	-1.44479616248267	3.41261556707572e-15	1.15700690945268e-14	DOWN
ESR2	-1.34244672867586	2.12726311194201e-22	1.08310036749258e-21	DOWN
ETFDH	-1.88389254288308	4.53082386901517e-117	1.25230533382795e-114	DOWN
ETHE1	-1.25088989279818	1.12894218865798e-20	5.24640254366196e-20	DOWN
ETNK1	-1.15308223301187	2.28940073498173e-29	1.67360768254562e-28	DOWN
ETV4	5.30783304482609	0	0	UP
ETV5	1.26968814366703	6.96335030150713e-16	2.45153293166486e-15	UP
EVA1A	3.79064579614776	1.44003388189821e-102	3.05796463237726e-100	UP
EVC2	-1.0393844442845	3.74591517374455e-10	9.3422545002025e-10	DOWN
EVI2A	-1.35884933613763	5.59662618330711e-14	1.7686760749533e-13	DOWN
EVI2B	-1.57893798520283	5.64518629454757e-22	2.80535470739032e-21	DOWN
EVPL	1.76868569256487	7.55866145235265e-40	9.42154415675137e-39	UP
EVX1	3.44173091571399	3.373133254471e-32	2.86379177767448e-31	UP
EVX2	-3.07309182666442	5.58564196478889e-18	2.23233379694443e-17	DOWN
EXO1	1.50369393346458	1.01821821583014e-51	2.2330269259761e-50	UP
EXOC1L	1.00458195860516	0.0109708201674088	0.0149269332376223	UP
EXOSC2	1.09973377011125	7.17408899672868e-65	2.96024672274968e-63	UP
EXOSC3	1.00859346688425	9.03673178337145e-47	1.5672969177674e-45	UP
EXOSC4	1.70855516065922	1.11916917030724e-31	9.24921346111057e-31	UP
EXOSC5	1.47148596766902	3.47223654131455e-50	7.13837720117003e-49	UP
EXOSC7	1.14100545030778	7.29325020325539e-45	1.14001226022699e-43	UP
EXOSC8	1.09794157631909	1.02992199747844e-37	1.15035482630481e-36	UP
EXTL1	-1.72584552153465	1.99772530765859e-14	6.4610681244909e-14	DOWN
EYA1	2.78083350836929	1.68489973976747e-16	6.17017017214951e-16	UP
EYA2	-2.74233480976506	2.63739175629328e-27	1.74553031745857e-26	DOWN
EZH2	1.25325477775988	3.97652626928082e-65	1.66450124824488e-63	UP
F12	1.8922034553485	2.6939382080714e-33	2.41702100999088e-32	UP
F13A1	-2.65337537453056	7.88331107294562e-31	6.19179502540379e-30	DOWN
F2	3.6870858551575	8.74097756328082e-28	5.92704993416701e-27	UP
F2RL1	-1.3245268983721	5.43229873486118e-49	1.04753729645778e-47	DOWN
F2RL2	1.48829173561662	1.72388472473035e-18	7.08138823112281e-18	UP
F5	2.15352617800875	1.81063442833718e-14	5.865781823374e-14	UP
F7	3.51109578552049	2.0827407974215e-27	1.38317183468728e-26	UP
F8	-1.0918874932456	7.59827403830794e-17	2.8331637222496e-16	DOWN
FABP1	-2.66616481341941	3.42978099660696e-21	1.63713751353939e-20	DOWN
FABP2	-3.17102660743711	2.11917835088401e-32	1.81958839368556e-31	DOWN
FABP4	-1.88888275648718	1.22911135163384e-06	2.40100022055195e-06	DOWN
FABP6	4.65531549201606	1.74272156643915e-86	1.90855412807578e-84	UP
FABP7	1.18053371457844	0.0128631910056078	0.0173673524835735	UP
FADS1	1.42086114173169	1.74708292435452e-16	6.39519759549825e-16	UP
FADS2	1.7975031791896	2.06507069282902e-16	7.52439338234604e-16	UP

GeneSymbol	log2FoldChange	pvalue	padj	change
FADS3	1.01672179928178	2.17835224955295e-15	7.45511944211194e-15	UP
FAM107A	-3.5087468705585	6.45886053046292e-89	7.92029143781344e-87	DOWN
FAM107B	-1.57568879379777	2.0769155870314e-36	2.17863440463722e-35	DOWN
FAM110B	-1.47028722522549	2.63642458403943e-18	1.07061710079008e-17	DOWN
FAM110D	-1.25036590483241	2.22317638817583e-20	1.01580085141185e-19	DOWN
FAM111B	1.08639921790793	1.68037553666601e-22	8.61359411832945e-22	UP
FAM124A	-1.82890742132408	3.34847187861849e-25	1.98457933364138e-24	DOWN
FAM126B	-1.06543336655298	1.42997110156826e-31	1.17342539074496e-30	DOWN
FAM131C	2.64552119752743	7.31044930242558e-29	5.19367008172732e-28	UP
FAM133A	-1.04109415496677	0.0155696439853517	0.0207861849817472	DOWN
FAM135B	-4.56254861448171	1.40556527494505e-61	4.87551954833032e-60	DOWN
FAM13B	-1.1097165631698	5.25886802407089e-45	8.28711935775081e-44	DOWN
FAM13C	-1.89964087747591	9.9683041822204e-33	8.71813564666016e-32	DOWN
FAM151A	-2.27521833517592	2.41144316366491e-67	1.12574959273183e-65	DOWN
FAM153A	-1.3697906128383	1.89142813805797e-06	3.64168931534757e-06	DOWN
FAM155B	1.62086268843084	7.69469742909642e-07	1.52675212320939e-06	UP
FAM161B	-1.36907449618702	9.93996664794271e-51	2.09545568087925e-49	DOWN
FAM162A	-1.12365301571606	2.61370442402735e-41	3.4848725218674e-40	DOWN
FAM163A	-2.14183344543543	4.90179592781274e-31	3.88683845587446e-30	DOWN
FAM163B	-2.88810475145487	9.82818126076331e-31	7.67779812892201e-30	DOWN
FAM166C	3.3389151660704	6.31248809645958e-87	7.09157130475166e-85	UP
FAM169A	1.15820897766181	1.02393652792945e-08	2.3146575049767e-08	UP
FAM174C	1.03723419864246	6.34971839660501e-20	2.83143780896499e-19	UP
FAM177B	-1.06260923159229	0.000255015751023552	0.000408630649910104	DOWN
FAM180A	-1.39673171646434	6.95890878256504e-10	1.70429646456828e-09	DOWN
FAM180B	-4.74767141730113	5.93083855706e-70	3.06450124018058e-68	DOWN
FAM181B	-2.16213242265851	1.6754028979917e-13	5.16990796787694e-13	DOWN
FAM183A	1.50753081591826	3.33414092243347e-07	6.77844668795493e-07	UP
FAM184A	-1.13417833122088	7.77516086709553e-06	1.4231985302085e-05	DOWN
FAM189A1	-1.95730767561123	3.39285460482793e-12	9.65198125042784e-12	DOWN
FAM189A2	-2.29959242055611	5.7549935119513e-29	4.10872087017663e-28	DOWN
FAM214A	-1.34977744546098	8.38742188263533e-55	2.15413240770397e-53	DOWN
FAM216B	-1.73546019112615	1.79366040702791e-09	4.26855387010756e-09	DOWN
FAM221B	1.14798332879848	3.48647761375974e-07	7.07328844091789e-07	UP
FAM222A	1.47551169382825	6.90281577558957e-20	3.06942622830289e-19	UP
FAM227A	2.21113799448158	6.22803339649869e-59	1.91437332953777e-57	UP
FAM237B	1.29241772210635	3.97562390603225e-07	8.03849733810261e-07	UP
FAM240A	2.29867970797675	6.40024990188908e-12	1.79262588936134e-11	UP
FAM241B	1.3476250448191	1.62215296519737e-37	1.79457112979554e-36	UP
FAM24B	1.16828104979024	3.35030629265685e-13	1.0140602029208e-12	UP
FAM43B	-1.51971139692873	2.14346740479521e-09	5.06709176210955e-09	DOWN
FAM53A	1.57817197424293	2.2453884553296e-27	1.49005141664079e-26	UP
FAM71E1	1.56092778611119	5.00976179199434e-33	4.43943929180649e-32	UP
FAM72A	1.57147465023072	4.66237316965794e-36	4.80389964516294e-35	UP
FAM72D	1.70167234041771	4.24669870027992e-27	2.78732621439782e-26	UP
FAM81B	1.30175556314742	0.00796833675972769	0.0110060004756991	UP
FAM83A	2.03566721604752	1.62066611019197e-12	4.70658088338438e-12	UP
FAM83C	1.58068661172509	3.8527080196396e-05	6.64361306654628e-05	UP
FAM83H	1.09482145763626	5.9636035685325e-27	3.88202725005071e-26	UP
FAM89A	1.82061705522564	4.00446741510405e-77	2.90540796246695e-75	UP
FAM95C	1.1997503442789	1.77781513424477e-08	3.95314709904279e-08	UP
FAM9A	3.21089870614947	3.94040585931246e-06	7.39139149285875e-06	UP
FANCA	1.40741720749793	1.47912767639943e-54	3.76550441946539e-53	UP
FANCB	1.87075195656932	3.53670342278926e-50	7.26233687512139e-49	UP
FANCD2	1.13551458628679	1.44582617227728e-41	1.95467167219444e-40	UP
FANCD2OS	1.46946448756152	4.53802695202237e-07	9.14062039509144e-07	UP
FANCG	2.38293709862057	3.86119020064743e-46	6.45248608098596e-45	UP
FANCI	1.29063664989606	3.53168541409684e-49	6.85587938859178e-48	UP
FAP	2.97750469034418	1.08301496691517e-41	1.4744753415867e-40	UP

GeneSymbol	log2FoldChange	pvalue	padj	change
FAS	-1.40366638552374	3.428193044459e-25	2.0302873653435e-24	DOWN
FASN	1.4361798109778	1.00530531977238e-30	7.84291287329594e-30	UP
FAT4	-1.27129441442696	1.07101293649477e-14	3.53010567162282e-14	DOWN
FAXC	-2.00607894017249	3.3528774273523e-20	1.51961620620733e-19	DOWN
FAXDC2	-1.84876971113014	6.31836595068615e-30	4.75666693900985e-29	DOWN
FBF1	1.14497842178756	2.1942809891226e-28	1.5198494376926e-27	UP
FBL	1.15188341738332	1.23579985952694e-32	1.07487427342371e-31	UP
FBLIM1	-1.2871794931146	1.76769549980564e-75	1.18845103235968e-73	DOWN
FBLN1	-1.61004176148673	3.18000489112315e-17	1.21701551177474e-16	DOWN
FBLN2	-1.30381330275518	9.37615880268934e-10	2.27613346202746e-09	DOWN
FBLN5	-1.38530918236455	7.08763768798279e-20	3.14759079471677e-19	DOWN
FBN3	1.32067517118194	0.000301900054320746	0.000480528852457692	UP
FBXL16	1.22009890272009	8.85514418471634e-09	2.0111468069449e-08	UP
FBXL22	-2.60379183023083	9.22171376491868e-71	4.92569637388126e-69	DOWN
FBXL6	1.38525024904392	6.5315081511061e-26	4.05032590581234e-25	UP
FBXL7	-1.46092140925882	2.49247999837385e-23	1.33831496181572e-22	DOWN
FBXO16	1.00830069403701	3.05314968681618e-09	7.1390486768538e-09	UP
FBXO2	2.44512906385762	7.25779920186784e-19	3.04090609966614e-18	UP
FBXO27	-1.02284259508252	3.74844003744016e-06	7.04344300981392e-06	DOWN
FBXO32	-2.26351927455359	4.25891328320776e-52	9.61535213343419e-51	DOWN
FBXO39	1.56642289735661	5.15507392803595e-09	1.19036337765402e-08	UP
FBXO41	2.02487056735407	6.54856143965159e-64	2.53400222997007e-62	UP
FBXO47	1.54222789045317	0.000233584723652297	0.00037529163987428	UP
FBXW10	1.28407267392177	0.000978257456951306	0.00148651458623279	UP
FBXW12	1.41770567425958	8.27657894612528e-10	2.01820570212687e-09	UP
FCAR	1.30460684674933	4.09364627649152e-06	7.66892550968767e-06	UP
FCER1A	-2.66040610973816	2.41763538014553e-33	2.17787298885019e-32	DOWN
FCER2	-2.99425626834028	1.48329948415362e-20	6.84385106453816e-20	DOWN
FCGBP	-2.27871065819722	1.89162200235834e-13	5.81855041990211e-13	DOWN
FCGR2B	-1.27410178395116	6.39538073478179e-10	1.57092346924468e-09	DOWN
FCHO1	1.54977745941883	1.69708633947679e-24	9.71764696787548e-24	UP
FCMR	-1.64180768396358	4.53547545953124e-24	2.54106287570198e-23	DOWN
FCN1	-1.76127293432098	1.09205502113644e-13	3.3969192717129e-13	DOWN
FCN3	1.11575979126902	2.71903488731939e-14	8.73552665920526e-14	UP
FCRL1	-2.35902135013813	1.77757159522635e-12	5.15022532240873e-12	DOWN
FCRL2	-2.41002882031189	1.93480038672281e-21	9.3507297069121e-21	DOWN
FCRL3	-2.1307434554802	3.60318986899687e-18	1.45371513412517e-17	DOWN
FCRL4	-3.36867866540749	3.6759589032799e-33	3.27246791323174e-32	DOWN
FCRL5	-1.61743637360819	9.06572422857417e-11	2.35860534875485e-10	DOWN
FCRLA	-2.91969810526287	7.86790498732337e-28	5.35380342103407e-27	DOWN
FCRLB	1.03843236479687	8.9956712905937e-10	2.1874266748095e-09	UP
FDCSP	-2.81423421215221	3.11600023842986e-14	9.98507768711432e-14	DOWN
FDX2	2.19593241963028	8.12618709633741e-11	2.1176488462814e-10	UP
FER1L6	-2.42873886469504	7.98986503940288e-15	2.65814902428587e-14	DOWN
FERMT1	1.42538172124425	6.87110203489934e-32	5.74119480488014e-31	UP
FERMT2	-1.9721505863405	1.90629160109488e-33	1.72706845212618e-32	DOWN
FETUB	-2.24334690215677	8.09305739253772e-07	1.60250634951398e-06	DOWN
FEV	-3.94782135732858	6.5185066217442e-27	4.23532670912059e-26	DOWN
FEZ1	-1.64214181302639	2.05448253399446e-33	1.85842620075041e-32	DOWN
FEZF1	8.56515073375124	1.63731234422805e-77	1.20807287500182e-75	UP
FEZF2	3.76278345319898	5.65938137089366e-10	1.39426722992885e-09	UP
FFAR4	-1.24920197577213	1.43037716199587e-07	2.98897846175857e-07	DOWN
FGA	4.76714202543142	2.71750722628667e-37	2.97059782706444e-36	UP
FGB	5.71778248620673	2.13208322400383e-37	2.34825839213022e-36	UP
FGD2	-1.03722054913471	8.04805749440318e-14	2.51780138609491e-13	DOWN
FGF10	-2.25445036816176	3.39329598414684e-26	2.12919191655636e-25	DOWN
FGF12	-1.13079216075661	1.08877717850141e-10	2.81706939216124e-10	DOWN
FGF13	-1.36957363481226	5.38346689045557e-11	1.42119934753643e-10	DOWN
FGF14	-1.38828227524171	3.73892132311122e-08	8.14230077530461e-08	DOWN

GeneSymbol	log2FoldChange	pvalue	padj	change
FGF17	1.77362062989438	1.81042639978464e-11	4.91885706029802e-11	UP
FGF18	2.05132229259372	4.61723221389529e-31	3.66286398818035e-30	UP
FGF19	6.56480638261472	4.89347387013543e-59	1.51619324734285e-57	UP
FGF2	-1.78800222616512	6.32513939344603e-19	2.65973562564781e-18	DOWN
FGF20	6.14270688556145	5.87833940852539e-32	4.92349803370142e-31	UP
FGF21	2.47723904616738	3.48902184546144e-08	7.60951119677105e-08	UP
FGF23	3.77239504891333	1.34245359258776e-10	3.45443245274578e-10	UP
FGF3	5.26003291602644	3.27590774584196e-19	1.40224635148343e-18	UP
FGF4	1.60518005854754	3.93947309049458e-06	7.39123423389527e-06	UP
FGF7	-1.58712103294901	5.47184615457399e-13	1.63685375518978e-12	DOWN
FGF8	2.68104206891923	2.58283685909722e-14	8.30715519531952e-14	UP
FGF9	-1.42893104957749	1.40447721270637e-08	3.143465514763e-08	DOWN
FGFBP1	-1.1152187934597	1.09557095108949e-07	2.30902650342789e-07	DOWN
FGFBP2	-3.12966232435563	6.25172311646852e-46	1.03480280063751e-44	DOWN
FGFR1	-1.22761047773795	6.75379644734673e-16	2.38112689891979e-15	DOWN
FGFR2	-2.30204306949352	1.34359107921587e-32	1.16571756165351e-31	DOWN
FGFR4	1.41655672647698	3.36963237865316e-23	1.79325820933642e-22	UP
FGFRL1	2.47910091109919	4.27837962630852e-45	6.77267494844638e-44	UP
FGG	5.03057437409561	2.2558388944391e-15	7.70818733690505e-15	UP
FGGY	1.92863564113042	2.62889202117131e-18	1.06805638741615e-17	UP
FGL1	2.28192559626223	4.10632251937845e-05	7.06345885902765e-05	UP
FGL2	-2.85099454844746	1.57654222157511e-63	5.98089971770968e-62	DOWN
FHL1	-3.43855047502709	1.38490196653218e-75	9.34701470667628e-74	DOWN
FHL5	-1.84378597861135	1.07675193938062e-30	8.38152951293462e-30	DOWN
FIBCD1	1.78604594971124	5.64159492107097e-09	1.29908876435611e-08	UP
FIGN	-1.96081345331428	2.61731619175073e-29	1.90771564867959e-28	DOWN
FIGNL1	1.11051507275849	3.38721468439942e-36	3.52128771936997e-35	UP
FILIP1	-2.79733007236445	1.94682264905635e-52	4.47229852084673e-51	DOWN
FJX1	2.93233849253799	1.06849566422121e-84	1.08172761634209e-82	UP
FKBP10	1.90006065702238	2.29317326119822e-30	1.75907603512091e-29	UP
FKBP1B	-1.0449273355721	9.2858904616419e-08	1.96208240029815e-07	DOWN
FKBP2	1.92063678167588	2.82842573989988e-22	1.4284042171948e-21	UP
FKBP5	-1.18427845623464	1.09826546849936e-28	7.73941586522836e-28	DOWN
FLI1	-1.35352612159396	3.74549079604525e-25	2.21460887034078e-24	DOWN
FLNA	-2.22335004199618	1.16405627437972e-37	1.2968465710668e-36	DOWN
FLNB	-1.02046111587184	4.65814709470886e-26	2.89997552235128e-25	DOWN
FLNC	-3.3819833139471	4.14504823486439e-50	8.46163246350452e-49	DOWN
FLRT1	-1.24556172207753	1.7143427187965e-10	4.37261604839657e-10	DOWN
FLRT2	-1.3414890093856	3.53304841448392e-12	1.00411179245601e-11	DOWN
FLT3	-1.85464223051552	1.43107925042598e-18	5.90226977443571e-18	DOWN
FLVCR2	-1.86184040852889	3.4646717353422e-43	5.00666630103849e-42	DOWN
FMC1	1.4374652133301	1.62613148535912e-11	4.43127191777126e-11	UP
FMN2	-3.38577539457336	1.48545781959156e-23	8.08068635193622e-23	DOWN
FMO1	1.03337183801919	1.92665397724264e-07	3.98773632541615e-07	UP
FMO2	-1.89462396363209	3.49227718994726e-10	8.72217766904067e-10	DOWN
FMO3	1.0070633403551	1.47441221116254e-09	3.53246282787196e-09	UP
FMO4	-1.77731704696936	5.34687842697798e-61	1.80786784561102e-59	DOWN
FMO5	-2.07154386841378	8.29949468939684e-46	1.36725734178304e-44	DOWN
FNBP1	-1.98012359836651	7.07991814313266e-68	3.35007104962959e-66	DOWN
FNDC10	1.57773122763105	2.49582540614379e-18	1.01565337221738e-17	UP
FNDC11	2.63141716205259	1.54943004252684e-36	1.63615678171739e-35	UP
FNDC5	-1.02788972827788	3.98056307196376e-07	8.04754960781434e-07	DOWN
FNIP2	-1.21192435670632	8.91539700290511e-40	1.10809284804844e-38	DOWN
FO393400.1	4.00252113181257	4.40999122791177e-15	1.48820110952767e-14	UP
FO681492.1	-1.80158196206163	7.51885425427436e-17	2.80595390333647e-16	DOWN
FOLR1	4.31463151806407	2.17366274169794e-38	2.50662180935009e-37	UP
FOLR2	-2.36610997593186	3.63184941891795e-32	3.07295402971906e-31	DOWN
FOSB	-1.70903847778337	3.35238321612688e-11	8.96009960743167e-11	DOWN
FOSL1	2.29495137363639	1.20900593977459e-28	8.50602845628075e-28	UP

GeneSymbol	log2FoldChange	pvalue	padj	change
FOXA2	1.43584251982684	3.88720862146405e-28	2.66593004039203e-27	UP
FOXB1	1.26856235152137	0.000458141186329521	0.000718186215120269	UP
FOXC1	1.28965905190404	6.80559008577504e-10	1.66838997836971e-09	UP
FOXC2	1.45478268864559	9.28255314521053e-09	2.10437570521483e-08	UP
FOXD1	3.957066886407	5.58482059395308e-29	3.99213797218822e-28	UP
FOXD2	-1.1132956693915	6.6099260193358e-12	1.85016302483032e-11	DOWN
FOXD3	-3.3375971941177	4.81935891596256e-24	2.69145275188121e-23	DOWN
FOXD4	1.45358425980393	4.00737021531667e-12	1.13537809240659e-11	UP
FOXF1	-1.49003293932358	1.79019152499437e-27	1.19343817093136e-26	DOWN
FOXF2	-2.15232981509869	2.94714994663706e-42	4.08914119687579e-41	DOWN
FOXG1	3.74931968830234	3.30774331471121e-08	7.23135396598447e-08	UP
FOXI2	-1.93828336846815	4.91979495389377e-13	1.4750067068208e-12	DOWN
FOXI3	3.66300322306861	1.00149640138034e-12	2.94479176582843e-12	UP
FOXJ1	1.50187498867029	1.0398722716674e-05	1.88069130313091e-05	UP
FOXL2	2.96117900772029	8.44404363950444e-09	1.92229221982862e-08	UP
FOXL2NB	3.57260080798777	4.19824271945464e-10	1.04449207706621e-09	UP
FOXL3	-2.90565088141004	6.60640952952343e-19	2.77532953287313e-18	DOWN
FOXM1	1.38732679826424	5.39368656192644e-38	6.08686092694913e-37	UP
FOXN3	-1.3441620686017	1.14016824954573e-48	2.16507630636203e-47	DOWN
FOXO6	1.02969628780471	1.02844656379989e-08	2.32424919084328e-08	UP
FOXP2	-2.45982008680617	3.35247300514828e-25	1.98627466616696e-24	DOWN
FOXP3	1.3980167891348	1.06221095085845e-19	4.671957385021e-19	UP
FOXP4	1.04568259073601	6.15570924530102e-25	3.60300386851854e-24	UP
FOXQ1	6.41571165840054	7.16574838306104e-294	3.11942941485605e-290	UP
FOXRED2	1.00162072727562	5.26203583380114e-19	2.22884529248308e-18	UP
FOXS1	1.69416887457205	4.82345939508577e-30	3.65336661359846e-29	UP
FPGS	1.06602945102626	2.88594399764677e-30	2.20504356432748e-29	UP
FPGT-TNNI3K	-1.14463492381017	6.2284143827715e-08	1.33467117459021e-07	DOWN
FREM1	1.27511191676687	2.50249119783693e-06	4.76760166607597e-06	UP
FRMD1	-1.37800015401886	1.08594335716865e-09	2.62595912767361e-09	DOWN
FRMD3	-2.02688675583039	9.48888228299561e-29	6.71393365273476e-28	DOWN
FRMD4B	-1.16903765419761	6.84638322080169e-33	6.03931464153089e-32	DOWN
FRMD5	1.64967828552491	2.1591487001511e-21	1.04003475285563e-20	UP
FRMPD2	2.05011026402022	7.88488944256604e-06	1.44237398742938e-05	UP
FRMPD4	-4.75024428056182	1.0510049426256e-35	1.06379051665959e-34	DOWN
FRRS1L	-3.60987120086793	3.0882148974561e-39	3.72403642724398e-38	DOWN
FSBP	1.12353590854582	3.31982030374843e-17	1.26855455231888e-16	UP
FSCN1	1.55485008339689	9.46744397999747e-16	3.30771673402279e-15	UP
FSIP1	-1.23053662818504	3.59859370163434e-07	7.29564700507146e-07	DOWN
FSIP2	-2.8045028408187	1.94124579451619e-36	2.03877641857119e-35	DOWN
FSTL3	1.23941993162445	4.62065921664016e-14	1.4660994704693e-13	UP
FSTL4	1.30982758056941	8.31202607823555e-06	1.51748070979572e-05	UP
FSTL5	-1.08253576767205	0.018838323045903	0.0248716141631896	DOWN
FTSJ1	1.20686518399914	4.92869051575115e-62	1.7622851735272e-60	UP
FUCA1	-1.79984594886593	2.32264318085078e-100	4.6487569779488e-98	DOWN
FUT1	2.86797424319194	8.48618071284732e-119	2.54775628884156e-116	UP
FUT7	1.07762064956635	4.6705151393532e-08	1.01015625539135e-07	UP
FUT9	-3.43047126130246	1.50747926923207e-06	2.92573969183438e-06	DOWN
FXYD1	-1.78104692754605	8.85319331137073e-12	2.4606648863671e-11	DOWN
FXYD4	2.53234575979928	8.50139100016204e-19	3.5431958230211e-18	UP
FXYD5	2.11442817668346	7.01034835508582e-71	3.76762950330584e-69	UP
FXYD6	-2.28645820687587	3.68882322409889e-34	3.44230861742947e-33	DOWN
FYB1	-1.11975023434334	8.97959414399147e-11	2.33654621681595e-10	DOWN
FYCO1	-1.06732477795239	1.96274413034522e-34	1.85948115025578e-33	DOWN
FZD10	3.29991669424358	6.26762332927978e-20	2.79697911411453e-19	UP
FZD3	1.2131989040809	1.49036747200089e-17	5.80187095684138e-17	UP
FZD9	1.45442823216392	6.870009602629e-08	1.46548422406687e-07	UP
G6PC	-6.08049666518005	1.21262782459353e-35	1.22479630566399e-34	DOWN
G6PC2	-1.54556043348315	1.68265775858579e-06	3.25303869770782e-06	DOWN

GeneSymbol	log2FoldChange	pvalue	padj	change
GAB1	-1.2911540752314	4.34036630590348e-61	1.4704046397801e-59	DOWN
GABARAP	1.0874481474441	5.9835794890206e-09	1.37510980127116e-08	UP
GABRA3	1.97813901249237	3.85339702216882e-05	6.64414321685569e-05	UP
GABRA4	1.24725123304305	0.000530603520333305	0.000826422101928786	UP
GABRB1	1.71774853160442	3.69186332797219e-08	8.04183339129094e-08	UP
GABRB3	-1.04279319875743	0.00307684280349063	0.00444770577263675	DOWN
GABRD	2.92915245504267	6.70661521925586e-77	4.78615945954518e-75	UP
GABRE	2.344825482925	1.14995535245845e-42	1.62798150832186e-41	UP
GABRG2	-2.96878097609274	7.67615871056388e-12	2.14206653248476e-11	DOWN
GABRP	3.05157670918466	4.43383315815154e-21	2.10067867639888e-20	UP
GABRR1	2.00508455005135	9.85618632233004e-06	1.78888651689319e-05	UP
GAD1	5.29711469145019	2.47356287601622e-81	2.09088108544031e-79	UP
GADL1	1.08955916054536	0.00200622317883047	0.00295653048518746	UP
GAL	1.39988203461979	1.00299848483227e-07	2.11750880412032e-07	UP
GALK1	1.40467979299178	5.53637596798189e-29	3.96076067093133e-28	UP
GALNT12	-1.40566408285932	4.66470977889998e-38	5.28131283354911e-37	DOWN
GALNT14	1.27690207095512	8.92018459150794e-06	1.62493120924707e-05	UP
GALNT15	-2.10276392685028	1.25925316717097e-23	6.87809767877921e-23	DOWN
GALNT16	-2.67269818816554	3.62462385525689e-34	3.38421314700205e-33	DOWN
GALNT17	-2.00123801523903	1.14062982741389e-24	6.58547320449537e-24	DOWN
GALNT6	1.89256014272864	4.51110783904608e-57	1.29410083692437e-55	UP
GALNT9	1.45686769676026	6.85968290558979e-06	1.26212656841753e-05	UP
GALR1	-4.12244250230783	5.53320308903599e-38	6.23622429704749e-37	DOWN
GALR3	1.08984359467863	0.00378064152075407	0.00541384134875745	UP
GAP43	-3.22093847164594	3.54074188411274e-32	2.99878105194821e-31	DOWN
GAPT	-1.93912583586906	2.30154414276051e-22	1.16876022624348e-21	DOWN
GAREM2	-1.10231203954485	7.41471207069603e-17	2.76827575658298e-16	DOWN
GARS1	1.21873195152348	8.73835506032824e-68	4.11245882879718e-66	UP
GAS1	-1.08304152907485	3.34433625315735e-05	5.80029155141723e-05	DOWN
GAS2	1.50767655494852	6.96560856725213e-20	3.09655710956245e-19	UP
GAS7	-1.67415525779547	5.06003228554203e-27	3.30496407307365e-26	DOWN
GASK1A	-1.33770158336223	1.27854569806013e-10	3.29485218888873e-10	DOWN
GAST	3.97937216787728	1.1451059699017e-17	4.48991899434772e-17	UP
GATA1	-1.08998034848392	2.71334423042112e-06	5.1557685600527e-06	DOWN
GATA4	4.58439101446062	8.18328280284373e-16	2.86768974533946e-15	UP
GATA5	-1.02026751193418	0.013467270799823	0.0181323425684155	DOWN
GBP3	-1.32152325122598	5.77081810925143e-17	2.17128901764035e-16	DOWN
GBP6	1.14139956569765	0.000105963597888141	0.00017554410903113	UP
GBP7	2.42439540212005	1.38512669400387e-10	3.56108240405868e-10	UP
GBX2	5.82467011996384	5.97245672723978e-43	8.55953818859476e-42	UP
GCG	-4.62382031725527	3.09303423740028e-20	1.4036748807884e-19	DOWN
GCGR	-1.13214863092806	1.58819137504824e-05	2.83178132436156e-05	DOWN
GCHFR	1.71731924028539	8.63584688272552e-14	2.69732738598923e-13	UP
GCKR	1.85589463755609	2.93559545233168e-13	8.91325607871867e-13	UP
GCM1	2.35334176031372	1.80242955458616e-10	4.58721219438889e-10	UP
GCNT2	-3.78289432666749	5.0420519092734e-93	7.70151314878752e-91	DOWN
GCNT3	-2.11739790238891	6.63219068516473e-22	3.28272701537162e-21	DOWN
GCNT4	-2.20826490271466	6.85045205900256e-29	4.87482311824322e-28	DOWN
GCOM1	-1.33230729065679	1.47395997959149e-23	8.02064535144583e-23	DOWN
GCSAM	-1.11759178257674	2.13439424258285e-16	7.76885596699314e-16	DOWN
GCSAML	-2.63709305465244	4.34843688855689e-34	4.03621170258215e-33	DOWN
GCSH	1.10235699805385	1.57501134719746e-25	9.51619451379229e-25	UP
GDF15	2.99414651032325	8.59021086481561e-73	5.03640881444559e-71	UP
GDF5	-1.52881272515704	3.4957050363037e-06	6.58101573042482e-06	DOWN
GDF6	-1.32330669874105	1.37417780152408e-06	2.67687191609116e-06	DOWN
GDF7	-1.08173837229527	2.78677167712123e-07	5.69415818322312e-07	DOWN
GDNF	-2.14222936644067	1.73879593771353e-32	1.49815208626451e-31	DOWN
GDPD2	-3.16997443467547	5.43583370735598e-48	9.89071811349945e-47	DOWN
GDPD3	-1.90532086627382	9.98764701801759e-30	7.44180134894053e-29	DOWN

GeneSymbol	log2FoldChange	pvalue	padj	change
GDPD5	2.84004962617239	1.11016773150106e-59	3.54703682727117e-58	UP
GET4	1.95268095110041	7.23534775167604e-27	4.68709488094996e-26	UP
GFI1B	-1.95688392461156	3.86356351679252e-16	1.38172584756435e-15	DOWN
GFRA1	-3.11884690191517	3.23079281009661e-42	4.47556047750296e-41	DOWN
GFRA2	-3.67187309641334	5.35189122483241e-82	4.68303929135712e-80	DOWN
GFRA3	-2.88885533355548	8.94150088578483e-31	7.00712668425613e-30	DOWN
GFUS	1.26851205695642	8.54257662546971e-26	5.25625041622982e-25	UP
GFY	3.18613530327916	5.45890627284222e-09	1.25868557903869e-08	UP
GGCT	1.25080289236691	7.62433635440112e-46	1.25960691593156e-44	UP
GGH	1.20081262853144	4.2907919903242e-15	1.44853743558579e-14	UP
GGN	1.34320998179011	2.64796404104722e-16	9.57213988929941e-16	UP
GGT6	-1.76371069467423	2.17522709443527e-24	1.23822260200724e-23	DOWN
GGTA1	-2.46026536832935	2.51095917451783e-48	4.67629220383733e-47	DOWN
GHR	-2.43622641826808	1.64522180236977e-31	1.34246706863471e-30	DOWN
GHRHR	1.05342824068592	0.00187671002157143	0.00277624259668876	UP
GIMAP1	-1.65613024031018	4.69204862375731e-36	4.83161695360651e-35	DOWN
GIMAP4	-1.36213828777338	2.70229894031562e-22	1.36708691016025e-21	DOWN
GIMAP5	-1.44912665160771	2.50609993165642e-14	8.06332559311405e-14	DOWN
GIMAP6	-1.53574345395034	8.31519177278591e-28	5.6471308244743e-27	DOWN
GIMAP7	-1.80227376356691	2.95501813847238e-37	3.22606462979433e-36	DOWN
GIMAP8	-1.60865709650738	5.88441805266866e-32	4.92621978611151e-31	DOWN
GINM1	-1.03137344255542	2.19505285195627e-47	3.90822651442889e-46	DOWN
GINS1	1.6403969730925	1.44395640681679e-49	2.85398557456308e-48	UP
GINS2	1.10201788629665	2.08059313112751e-25	1.24542345109396e-24	UP
GINS4	1.31908288050811	1.14445843589684e-33	1.04721254567902e-32	UP
GJA3	3.05426665959314	6.25386761293307e-25	3.65799787517647e-24	UP
GJB3	1.19572216812617	3.27616287924497e-13	9.91963557925452e-13	UP
GJB4	3.88895828935721	3.86295562558467e-61	1.3112211756005e-59	UP
GJB5	4.30784747380529	1.19097602974821e-34	1.14198599152013e-33	UP
GJB6	2.42342218363102	2.91809770472987e-11	7.83181802288244e-11	UP
GJB7	2.54421673280369	1.50072791877368e-07	3.12960182630013e-07	UP
GJC1	-1.51740915976421	1.35754370756662e-22	6.98549307915412e-22	DOWN
GJC3	2.71832410050536	1.12542938101861e-35	1.13738257757847e-34	UP
GLA	1.04091560239694	4.55940912605316e-35	4.4728445696881e-34	UP
GLB1L3	-1.39171005284954	3.47366061448431e-05	6.01380515808464e-05	DOWN
GLDN	-2.91313279054604	4.44299296747294e-54	1.10364959404574e-52	DOWN
GLI3	-1.89748972601539	4.77227550078984e-22	2.37971458462925e-21	DOWN
GLIPR2	-2.03916918543333	3.0768496586648e-60	1.01089024728925e-58	DOWN
GLOD5	-1.29659161930914	3.21014648066353e-17	1.22826369298603e-16	DOWN
GLP2R	-3.97666538651635	1.63848301934054e-101	3.35657703715022e-99	DOWN
GLRA3	-1.12295884648758	0.000136861249054276	0.000224657327468148	DOWN
GLRA4	-2.41925066324962	3.07511467164148e-24	1.73684631129722e-23	DOWN
GLRB	-1.03942089770615	1.14732576647768e-07	2.41313969944146e-07	DOWN
GLS2	2.33342088638772	2.66213639548473e-34	2.5003118152414e-33	UP
GLTP	-1.61518118281607	2.61382306942107e-160	2.27572505539146e-157	DOWN
GLYAT	-1.51590939007708	0.00510181614464662	0.00720443796340375	DOWN
GLYATL1	3.06324906504008	4.573059282597e-37	4.94907901105416e-36	UP
GLYATL3	-2.72170106512431	8.98697082636634e-07	1.77285740341585e-06	DOWN
GMPR	1.03251332310647	1.06179213201458e-07	2.23973184673167e-07	UP
GNA11	-1.55324504074499	2.63808277861064e-66	1.16296039047967e-64	DOWN
GNA13	-1.12389208427172	1.23225443011812e-56	3.46643721997524e-55	DOWN
GNA15	1.1410909739436	7.79705882217943e-14	2.44103173805484e-13	UP
GNAI1	-1.54202043708711	1.63646594913148e-29	1.20744837170451e-28	DOWN
GNAL	-1.30390424731845	4.56166319204312e-19	1.93973726893887e-18	DOWN
GNAO1	-3.61082055836563	7.18163898321526e-62	2.54692219174597e-60	DOWN
GNAQ	-1.06549939917276	2.07185008426689e-34	1.96071334333366e-33	DOWN
GNAZ	-1.12349623333203	1.44873685047821e-12	4.21994894235148e-12	DOWN
GNB1L	1.64327309195886	9.53639163870927e-41	1.24480650378444e-39	UP
GNG10	2.94494291604896	3.44795861555457e-20	1.56189654975681e-19	UP

GeneSymbol	log2FoldChange	pvalue	padj	change
GNG11	-1.20768748531512	9.66709118199175e-19	4.01845449396091e-18	DOWN
GNG12	-1.16615880071835	1.34431379940941e-40	1.74300343924914e-39	DOWN
GNG13	-1.90798834052023	8.09224977011535e-09	1.84389355204159e-08	DOWN
GNG2	-2.13490677339111	1.04365465370783e-64	4.21651008933051e-63	DOWN
GNG4	2.67478512138155	2.40353098521488e-22	1.21877358897923e-21	UP
GNG7	-2.66905134409993	1.54641860056135e-61	5.35343679752979e-60	DOWN
GNG8	-2.51780858145477	3.94454074004326e-29	2.84575201257739e-28	DOWN
GNGT1	3.5038238896122	2.11338279884236e-19	9.1588687596421e-19	UP
GNL3	1.08265113504163	9.64024664259972e-53	2.23820819716785e-51	UP
GNMT	1.20731403733315	1.10466528489193e-12	3.23939653179911e-12	UP
GOLGA6L2	5.22396055614134	2.99888708249799e-22	1.51273524819054e-21	UP
GOLGA7B	2.55140310146591	4.36159930758427e-45	6.89814066693596e-44	UP
GOLGA8A	1.06939681489985	4.82367021323116e-08	1.04224555680598e-07	UP
GOLGA8B	1.33592875778781	1.45819043803534e-13	4.51163292421985e-13	UP
GOLGA8N	-1.36311607587859	2.34695301603364e-14	7.56105325961032e-14	DOWN
GOLGA8R	-1.42676347988051	5.17025146452109e-16	1.83658891782345e-15	DOWN
GOLGA8S	-1.75920270185054	1.0460212227098e-08	2.362434183015e-08	DOWN
GOLM1	-1.27690069362237	1.25715898271047e-33	1.14912910057414e-32	DOWN
GOLT1A	1.23183185474025	2.96648540721896e-18	1.20017217462602e-17	UP
GP1BA	-1.31353021564289	2.32209275656176e-18	9.47167982431714e-18	DOWN
GP2	-1.03923316606095	0.0166524173925611	0.0221502210722379	DOWN
GP9	-1.10162182773253	0.000480988907726382	0.000752376917915872	DOWN
GPA33	-1.81200546720447	1.61706266223391e-31	1.32072758618569e-30	DOWN
GPAT3	-2.53566468382135	1.29891195013956e-66	5.79947533020005e-65	DOWN
GPC2	1.43633084729981	3.60428189177302e-23	1.91696275447292e-22	UP
GPC5	-1.25336971258553	0.000232356063864558	0.000373420963550859	DOWN
GPC6	-1.0398906718434	1.91389052479459e-09	4.54164291472446e-09	DOWN
GPCPD1	1.1259595520973	2.16144106032498e-16	7.86237166982221e-16	UP
GPD1L	-1.47864527604123	7.40746633629748e-82	6.41722444348e-80	DOWN
GPER1	-2.10087249326713	9.89163521707575e-25	5.73378974816711e-24	DOWN
GPIHBP1	-2.08126860139659	2.05264148205381e-18	8.40015185123455e-18	DOWN
GPLD1	-1.18779427639665	1.28310217431769e-21	6.26195576272251e-21	DOWN
GPM6A	-4.59864615917478	1.81258782879294e-35	1.81602945125267e-34	DOWN
GPM6B	-2.93706867590547	1.05723778751769e-78	8.18208708466472e-77	DOWN
GPR119	-3.95135300208205	1.93012955871117e-21	9.33074569845575e-21	DOWN
GPR12	-3.2288832217114	2.30710338440151e-13	7.06286765692395e-13	DOWN
GPR142	-2.00549070535977	1.48276259166886e-16	5.45171980758655e-16	DOWN
GPR143	2.30880342537063	2.36128796025321e-23	1.26904652012003e-22	UP
GPR146	-1.06287776596798	2.91093676216164e-14	9.34343628378262e-14	DOWN
GPR15	-3.57279978076914	1.59623328294275e-34	1.52219113668577e-33	DOWN
GPR156	-1.55311935222603	1.20318965304907e-14	3.94857546712091e-14	DOWN
GPR157	1.30040622027741	6.2669845628879e-34	5.78309497581171e-33	UP
GPR162	-1.28694038553139	3.80984985374393e-23	2.0238229256633e-22	DOWN
GPR17	-1.71803737714655	2.7230256864565e-14	8.74673423321656e-14	DOWN
GPR171	-1.18111381038943	2.64821129231434e-10	6.66280930979188e-10	DOWN
GPR174	-1.400254066675	2.43345139476232e-12	7.0016009809974e-12	DOWN
GPR18	-1.7468880140663	7.99766934569019e-23	4.17331184646399e-22	DOWN
GPR182	-1.04501597877662	1.17918526821472e-08	2.65114952555492e-08	DOWN
GPR183	-1.38982121823457	5.31544447398758e-17	2.00471809888555e-16	DOWN
GPR19	1.52936589580251	3.11564781828077e-34	2.91681588493135e-33	UP
GPR26	-2.59767192350172	1.05536435316568e-09	2.55343330299765e-09	DOWN
GPR27	-1.64109609956563	1.78574251382657e-09	4.25029174320149e-09	DOWN
GPR3	2.16317622524992	4.36763907973996e-38	4.95141271455156e-37	UP
GPR31	-1.63010165766702	4.02060528196762e-14	1.27990493189949e-13	DOWN
GPR34	-1.8091293397594	2.83752626353992e-26	1.78697449645644e-25	DOWN
GPR37L1	-1.62471076782601	2.72942299917499e-31	2.19729277321471e-30	DOWN
GPR4	1.0970180782206	1.17369613946943e-19	5.14929979253747e-19	UP
GPR78	1.87811256596003	8.12506827430152e-14	2.54143728867276e-13	UP
GPR82	-1.617892018815	1.95749063061314e-22	9.98997196684248e-22	DOWN

GeneSymbol	log2FoldChange	pvalue	padj	change
GPR84	1.43919727393409	1.73430065379099e-13	5.34880929586655e-13	UP
GPR88	-2.54430731188562	9.47022159712862e-27	6.09630198413311e-26	DOWN
GPRASP1	-1.9997255101747	6.14053004159189e-37	6.61255718084351e-36	DOWN
GPRC6A	3.16390948657514	8.48832778267886e-10	2.06839143128725e-09	UP
GPRIN2	-1.37076296381724	8.55455059582889e-19	3.56449843324165e-18	DOWN
GPS2	1.22899505045026	1.54050952264654e-10	3.94483710556533e-10	UP
GPSM2	1.30399475192452	9.55796719434466e-34	8.7642381650934e-33	UP
GPT	-2.71255492625938	3.65657700101566e-35	3.59932025543729e-34	DOWN
GPT2	1.67885540057938	2.12206443138805e-52	4.85565150377924e-51	UP
GPX2	1.10180569880332	6.47507441241524e-17	2.42526286821653e-16	UP
GPX3	-2.34029156202538	2.26573060924397e-37	2.49230367016837e-36	DOWN
GRAMD1A	1.61458797044552	4.03758424617113e-67	1.85995911318989e-65	UP
GRAMD1C	-1.3751212203274	2.53364825279568e-21	1.21672413198927e-20	DOWN
GRAMD2B	-1.6212636609222	9.05032751206325e-87	1.0037793182647e-84	DOWN
GRAP2	-1.04577293259182	4.0888778872893e-12	1.15752935541162e-11	DOWN
GRB7	1.69760234305574	4.21611852080796e-34	3.91966213576236e-33	UP
GREB1L	1.84664192750682	8.27017916312262e-08	1.75363650471815e-07	UP
GREM2	-3.77902165949473	1.11697800799682e-49	2.22031256315623e-48	DOWN
GRHL1	2.31984940273032	1.51870274522333e-57	4.45204897349728e-56	UP
GRHL3	3.94377430560959	3.9919637603846e-87	4.51377045192059e-85	UP
GRIA1	-2.58167038763602	1.27814885664061e-20	5.91925692571355e-20	DOWN
GRIA2	-2.5135294049981	2.44779049333134e-16	8.86878399092355e-16	DOWN
GRIA3	-2.24878890048221	6.26309585978259e-25	3.66216548711868e-24	DOWN
GRIA4	-2.86998731302826	1.55667685052497e-38	1.80950694246937e-37	DOWN
GRID2	-1.9027556445513	3.38674650605601e-05	5.87033813557171e-05	DOWN
GRIK1	-2.43310701414679	8.22333875237709e-63	3.02733610349138e-61	DOWN
GRIK2	-1.4685604760574	2.14976516690838e-09	5.08128964997634e-09	DOWN
GRIK3	-3.75009810812717	7.31567938218754e-75	4.6833795986041e-73	DOWN
GRIK5	-2.92904435197878	1.3038396693102e-41	1.76545568909009e-40	DOWN
GRIN2A	-2.85904518818205	1.72086571641818e-22	8.81336315293815e-22	DOWN
GRIN2B	3.10026774837063	2.17441261301651e-66	9.63436306118487e-65	UP
GRIN2D	5.01636117434215	8.5847962539018e-216	1.66096730187991e-212	UP
GRIN3A	-1.97317467862451	3.30032817189753e-42	4.56825234159394e-41	DOWN
GRIN3B	1.71964231671813	3.12990405549903e-14	1.00260918710707e-13	UP
GRINA	1.46265675906537	1.29505672524794e-39	1.59032600541202e-38	UP
GRK1	3.49160654428634	5.21893101717274e-27	3.4061936207657e-26	UP
GRM2	1.42988043082469	1.3970001280096e-15	4.83809928978345e-15	UP
GRM4	1.70040542125808	1.67833510863024e-07	3.48578831662433e-07	UP
GRM8	2.15628226572353	5.70182032450778e-18	2.27563138461274e-17	UP
GRP	1.92834810643287	1.58013781867734e-12	4.59117968240089e-12	UP
GRPR	2.66632274267648	5.87136332263489e-16	2.07843158237531e-15	UP
GSC	2.30399551738033	2.48589278591779e-15	8.48096612092212e-15	UP
GSDMA	2.29375837553807	2.02189906158217e-27	1.3432784570519e-26	UP
GSDMC	3.00047950307068	1.49093706674513e-35	1.5006755574123e-34	UP
GSG1	-2.56536317984504	1.9949094507079e-15	6.84345119487326e-15	DOWN
GSG1L	-3.0087112001841	1.22701463165602e-25	7.45759364084687e-25	DOWN
GSKIP	-1.00955485721926	1.72473287563855e-17	6.69328583986942e-17	DOWN
GSN	-1.88811056149342	1.28271881018101e-49	2.54396157650135e-48	DOWN
GSTA1	-2.24122456568338	8.42026019968687e-14	2.63046269926709e-13	DOWN
GSTA2	-1.02210164152691	0.000443390418753542	0.000695751767302463	DOWN
GSTM1	-1.3488651015159	0.00296430813655422	0.00429215845516536	DOWN
GSTM2	-1.39703054387546	5.96422483952008e-22	2.95967646425087e-21	DOWN
GSTM5	-3.07234785048937	4.71692875918013e-75	3.07625020537841e-73	DOWN
GSTP1	1.24371702524779	7.35875177804366e-26	4.54551063182243e-25	UP
GSX1	3.03355423168301	1.47570568350819e-06	2.86598963494625e-06	UP
GTF2F2	1.01895595672712	6.76049095535171e-26	4.18338411533544e-25	UP
GTF2H4	1.65499693167033	2.9828075117042e-14	9.56707076833768e-14	UP
GTF2IRD1	2.21254783013229	2.80514351014031e-136	1.28542010373877e-133	UP
GTF3A	1.83123515531162	1.31210714915222e-49	2.59633202138496e-48	UP

GeneSymbol	log2FoldChange	pvalue	padj	change
GTPBP3	1.07877591823469	1.43934933472927e-43	2.12042216291377e-42	UP
GTPBP4	1.07217063374305	1.29593618205625e-81	1.10078715795832e-79	UP
GTSE1	1.33178792406905	5.9728007679679e-56	1.62506843394727e-54	UP
GUCA2A	-5.08737722256931	1.30256266678243e-70	6.89408015704633e-69	DOWN
GUCA2B	-6.00405108741921	4.83031333326584e-54	1.19815165344955e-52	DOWN
GUCY1A1	-1.53290225755477	7.16359343851829e-16	2.51897521294263e-15	DOWN
GUCY2D	1.36616635223715	3.30492121910544e-07	6.72061113958694e-07	UP
GULP1	-1.02280940587792	4.7615392876828e-08	1.02958752783336e-07	DOWN
GVQW3	1.29287115833153	1.66850247184341e-22	8.55525133751747e-22	UP
GYPC	-1.7277648884989	2.4545737740979e-31	1.97968935286553e-30	DOWN
GYPE	-1.53445558538647	6.12000192958245e-16	2.16293065962694e-15	DOWN
GZMA	-1.55460199464408	1.24744128448966e-14	4.08994447125183e-14	DOWN
GZMB	2.23773082494194	2.59388636295399e-23	1.39062017358737e-22	UP
GZMM	-1.47254450698	1.42840540391027e-14	4.66569561025879e-14	DOWN
H1-2	-1.165971835498	3.14262361531511e-07	6.40029298403298e-07	DOWN
H1-4	1.99327241489767	2.95181826517159e-19	1.2678838542534e-18	UP
H1-5	2.39830708060618	6.82461518337124e-17	2.55234158479475e-16	UP
H2AC12	2.1657395637283	6.5563909313539e-15	2.19087383012215e-14	UP
H2AC13	-1.68129758398362	4.69735639930923e-22	2.34302683990752e-21	DOWN
H2AC14	1.63368452848831	1.51563308465982e-07	3.15803744204636e-07	UP
H2AC16	2.24511168849457	8.71375188668337e-12	2.42306869375308e-11	UP
H2AC21	2.33059002129284	8.93440578883258e-08	1.88941957737359e-07	UP
H2AC4	2.23621918591025	3.86760604207102e-10	9.63746766035814e-10	UP
H2AP	1.32615788288802	0.016450849414048	0.0219038569235982	UP
H2AW	1.96721749409971	2.13711278610708e-35	2.13013995102934e-34	UP
H2AX	1.14682183277435	1.46790580940551e-23	7.99269664139404e-23	UP
H2BC10	2.77763446883906	1.48987045759588e-10	3.82078266246202e-10	UP
H2BC14	1.16927724305582	0.00547684603608092	0.00770653091121431	UP
H2BC17	1.40794756417746	5.5162395824886e-09	1.27156843857392e-08	UP
H2BC18	1.63461830948583	5.52019160184138e-15	1.85065645673593e-14	UP
H2BC3	3.26205077110395	2.44427668476595e-05	4.28708601045824e-05	UP
H2BU1	1.99245591224891	2.19632337418128e-19	9.51121087157886e-19	UP
H3-5	1.08185086956077	4.16979459821221e-08	9.05006024413177e-08	UP
H3C11	3.05407462131616	5.11402019401394e-12	1.44001348056864e-11	UP
H3C12	2.38709805155505	2.19831054578728e-15	7.52046788483183e-15	UP
H3C13	1.32507467097793	9.9848969654408e-07	1.96260312517463e-06	UP
H3C2	1.28372526373049	6.25088388910964e-07	1.2478120045978e-06	UP
H3C6	-1.15954263058359	7.52109086035338e-17	2.80618716844511e-16	DOWN
H4C1	2.16601516804912	2.35505298020778e-07	4.83534224081571e-07	UP
H4C13	2.58599217007642	9.51878058595393e-06	1.73017250880184e-05	UP
H4C2	2.51384260211322	5.30994327394535e-13	1.58924101459626e-12	UP
H4C3	1.65761210197092	4.19703929540512e-10	1.04434188698041e-09	UP
H4C4	1.44491410166775	1.13760555861355e-08	2.56130405897824e-08	UP
H4C5	1.35344100034945	5.93212687169257e-12	1.66311584634975e-11	UP
HABP2	6.53188918391741	1.21461152148776e-41	1.64976836378053e-40	UP
HACD4	-1.59519216957106	5.37224613037782e-32	4.51045910647391e-31	DOWN
HADHB	-1.01277974945315	1.4663467734678e-72	8.51116545546491e-71	DOWN
HAGHL	3.70439922076245	4.7041524651634e-82	4.17925545285154e-80	UP
HAMP	1.53076247931673	7.04807777314445e-10	1.72540669568065e-09	UP
HAND1	-4.12903948402519	1.08401019710859e-16	4.00761561831251e-16	DOWN
HAND2	-4.0529874417888	2.58307301145258e-49	5.04815379892522e-48	DOWN
HAPLN1	-2.54230843382006	4.46242823067198e-48	8.20530757979843e-47	DOWN
HAPLN3	1.68161712248043	6.2728370492691e-24	3.48417580666421e-23	UP
HASPIN	1.22567631229577	2.58290553540149e-32	2.20687605927115e-31	UP
HAUS5	1.20222780429229	9.11401032160914e-59	2.76003933443791e-57	UP
HAUS7	1.72090443335531	1.49065546907247e-30	1.15056665261342e-29	UP
HBA2	-1.98338448235417	1.43868882725888e-17	5.60821324133847e-17	DOWN
HBB	-2.59436269632803	1.63261577172161e-25	9.8437459948021e-25	DOWN
HBG2	3.55374738527456	0.00131154239474971	0.00196844403721571	UP

GeneSymbol	log2FoldChange	pvalue	padj	change
HBQ1	3.18625249377277	2.87949625289899e-13	8.75208033718451e-13	UP
HBZ	4.04900067298062	6.86712390558002e-05	0.000115645288750353	UP
HCAR1	3.79606577593111	4.16549421753728e-30	3.1591354882394e-29	UP
HCAR2	2.08103110988829	3.25482885003398e-12	9.27751428476701e-12	UP
HCAR3	2.61535709293337	7.97014978235755e-14	2.49432455356204e-13	UP
HCFC2	-1.30494573394174	8.63028789525506e-43	1.23078790434133e-41	DOWN
HCLS1	-1.19802407012566	9.10942319377398e-18	3.59524900437866e-17	DOWN
HCN1	2.18135008665815	1.77000110118367e-11	4.81277782244084e-11	UP
HCN4	-2.03514066163819	4.65453533976486e-17	1.76079565221215e-16	DOWN
HCRT	3.47316033466693	5.30129714786776e-19	2.24492916429527e-18	UP
HDAC10	2.22335757332612	1.46704676137956e-32	1.26966626520389e-31	UP
HDAC9	-1.88532991090846	5.28275581297839e-26	3.28530810612117e-25	DOWN
HDC	-1.47125224595007	4.55727467669595e-13	1.36891191901512e-12	DOWN
HEATR1	1.0553077133452	1.12396778827944e-39	1.38511331191153e-38	UP
HECTD2	-1.21802882764202	2.97762025289751e-19	1.27833583490395e-18	DOWN
HECW1	1.37462655291025	1.98144347113247e-12	5.72281890244314e-12	UP
HECW2	1.28993127726556	1.23186460888146e-18	5.09149262626462e-18	UP
HELLS	1.39369911666227	1.85011591770093e-41	2.48580775269493e-40	UP
HELZ2	1.15493884663879	6.46509151530657e-23	3.38967397054179e-22	UP
HEMGN	-1.62866556293289	0.000789236243817853	0.00120976854873242	DOWN
HENMT1	1.16316572534486	1.60874772045652e-18	6.6146691986563e-18	UP
HEPACAM	-2.81411490529758	1.19826503575683e-11	3.30200808160052e-11	DOWN
HEPACAM2	-2.39537521982486	6.41510969790237e-14	2.02037086579081e-13	DOWN
HEPHL1	2.42642014054552	2.40310295595912e-16	8.71231142455053e-16	UP
HES4	1.96057024086055	4.19611710215911e-23	2.22493870584338e-22	UP
HES6	1.86733828047418	2.4284014814024e-37	2.66283091912217e-36	UP
HES7	2.99357690178837	3.68339255967915e-16	1.31945926026935e-15	UP
HFM1	-1.23973687299204	4.08282490749218e-06	7.65029916218242e-06	DOWN
HGD	-1.12604417853954	2.57334007206693e-10	6.48192834874894e-10	DOWN
HGFAC	1.65029126965749	1.05219035988621e-07	2.22001584111216e-07	UP
HGH1	1.38787323450552	4.90204838367807e-42	6.7371245860289e-41	UP
HHIP	-2.12830377608994	2.29158041657966e-25	1.36748765571973e-24	DOWN
HHLA2	-2.8050967421009	3.84719475072557e-51	8.28074192761239e-50	DOWN
HIF3A	-2.29542502889215	4.95795486054371e-24	2.76707910213614e-23	DOWN
HIGD1A	-1.85876374681018	3.10237266273727e-76	2.14371488794619e-74	DOWN
HILPDA	2.4355732132692	1.14327150251409e-93	1.8264024470897e-91	UP
HINT2	1.17322852776073	1.81441491130381e-11	4.92892462566822e-11	UP
HIPK4	-2.07656327054391	2.93118974735631e-26	1.84395979301718e-25	DOWN
HJURP	1.6776962744987	4.22937765868361e-88	5.00994239256174e-86	UP
HK2	-1.27472072184625	2.63157101316853e-29	1.91730318210475e-28	DOWN
HLF	-2.76401395617287	9.10839713292951e-65	3.69707504139165e-63	DOWN
HM13	1.29249593201528	1.57901206589308e-46	2.70890020723115e-45	UP
HMCN2	-2.28468586095294	3.18253779570444e-31	2.55027752584452e-30	DOWN
HMGA1	1.45136082851648	5.91401058299066e-44	8.85474344639865e-43	UP
HMGA2	1.52118464156344	1.26495329064471e-14	4.14579929418338e-14	UP
HMGCLL1	-3.04548732245539	3.69385015668042e-61	1.25626978082571e-59	DOWN
HMGCS2	-2.24248770674843	8.81743372357189e-16	3.08557020555782e-15	DOWN
HMMR	1.22457686341032	9.02219199435615e-23	4.69526088457034e-22	UP
HMOX1	-1.58998164375421	9.2359718665639e-37	9.84849835348911e-36	DOWN
HMX2	-1.86894429850094	8.93948180782254e-07	1.76408881141901e-06	DOWN
HNRNPA1P48	1.15026340371759	1.09137346392423e-22	5.65766184201031e-22	UP
HOGA1	2.83073688098067	1.0406449881149e-27	7.02354696823443e-27	UP
HOMER1	1.84213240246746	6.83955404772677e-47	1.19695632797052e-45	UP
HORMAD1	1.19468724978994	0.000744205678578462	0.00114426962305402	UP
HOXA9	1.0485261105858	2.35305268056534e-09	5.54073107865913e-09	UP
HOXB13	-1.53240778926125	5.57281746709552e-09	1.28427038849304e-08	DOWN
HOXB6	1.32729815056825	2.89654726372797e-12	8.27795462059662e-12	UP
HOXB8	2.87818490940859	2.26138054844136e-41	3.03136408699072e-40	UP
HOXC11	5.00563093873011	6.88126589757203e-24	3.81117948709993e-23	UP

GeneSymbol	log2FoldChange	pvalue	padj	change
HOXC12	5.06366787795506	5.54824384768156e-12	1.55799984066568e-11	UP
HOXC13	4.59879362359361	1.00376335140734e-10	2.60601330521186e-10	UP
HOXC6	1.87712861070672	1.48568077851022e-07	3.09970757203432e-07	UP
HOXC8	1.63861571978413	1.89048589165048e-05	3.35053748919183e-05	UP
HOXC9	1.04408065370991	0.00144566712916164	0.00216080701460014	UP
HOXD1	-2.05662558849778	2.58737241369386e-17	9.94128769630433e-17	DOWN
HOXD12	-2.49126028016006	4.3102832049432e-14	1.3691164072907e-13	DOWN
HOXD3	-1.06065419784958	1.34667509238793e-09	3.23488113998496e-09	DOWN
HPCA	-1.43340542763609	7.87010152430918e-19	3.28795772175614e-18	DOWN
HPCAL4	-1.91128425463932	5.14397421791631e-14	1.62887839710087e-13	DOWN
HPDL	1.43174132690331	2.55185992521124e-16	9.23431772188347e-16	UP
HPGD	-2.89411179744795	5.46223079109585e-48	9.92837419262548e-47	DOWN
HPGDS	-3.09231967698874	2.01034528304915e-69	1.00882254794625e-67	DOWN
HPN	2.98378598065979	7.97127326225402e-18	3.15534851820025e-17	UP
HPSE	-1.03690822536483	2.42435204370978e-14	7.80751657797639e-14	DOWN
HPSE2	-3.40164679089291	1.63319452660602e-63	6.18235136778056e-62	DOWN
HRC	-1.06737138726138	2.6626635023576e-16	9.6219724884444e-16	DOWN
HRCT1	-2.2740455693856	2.30383414136559e-30	1.76647573331568e-29	DOWN
HRG	2.4491762142506	1.40479031928911e-06	2.73375210435643e-06	UP
HRK	-2.57168499566667	1.69800641233505e-13	5.23780082515326e-13	DOWN
HROB	1.45717025296234	2.00033312566899e-59	6.32156092872488e-58	UP
HS3ST4	2.87277985163441	1.95449513545585e-09	4.63420803291023e-09	UP
HS3ST6	-4.23889993767794	1.81805375911266e-28	1.26580448250415e-27	DOWN
HS6ST2	3.37532724893867	1.66149227566653e-37	1.8369247616623e-36	UP
HS6ST3	-1.96720205566236	1.14623148735954e-07	2.41142067045932e-07	DOWN
HSD11B2	-2.23668041798436	1.70652364085861e-49	3.36150409030215e-48	DOWN
HSD17B11	-1.28952756300985	5.41259238016431e-43	7.77635900295389e-42	DOWN
HSD17B2	-3.26419603981425	2.98909203281563e-46	5.03375817866717e-45	DOWN
HSD17B6	-1.60501710748548	7.84134888530536e-25	4.57271962959887e-24	DOWN
HSD3B2	-5.72618008296993	1.25984731225631e-37	1.40087619721067e-36	DOWN
HSF2BP	1.20942705395692	1.45910731325926e-17	5.6839900773565e-17	UP
HSF4	2.07274029143215	1.75205203190439e-40	2.25988755789268e-39	UP
HSP90AB1	1.075240695797	5.99587557725944e-71	3.23238951785816e-69	UP
HSPA2	-1.62586720724566	8.91901350795222e-15	2.95766105911202e-14	DOWN
HSPA4L	1.01304303793319	4.43951768894138e-07	8.94633971965471e-07	UP
HSPA6	1.47325287846345	4.61002036916323e-13	1.38427805980754e-12	UP
HSPB2	1.68607059027158	0.0004253731640652	0.000668564212101032	UP
HSPB3	-2.17234374983664	2.18595579640663e-07	4.50248974246849e-07	DOWN
HSPB6	-2.87453124212583	1.10755535548878e-25	6.74802708366903e-25	DOWN
HSPB7	-2.67900145990513	3.28838838148728e-24	1.85429750281211e-23	DOWN
HSPB8	-3.78526915978311	2.5623989426093e-65	1.08036447427738e-63	DOWN
HSPD1	1.26469456114293	2.44386748318247e-52	5.57003461841052e-51	UP
HSPE1	1.35698030998928	1.96938732476136e-35	1.9663383879627e-34	UP
HSPH1	1.58164246467392	1.69397133189097e-36	1.78445993963808e-35	UP
HTN1	2.08274377851661	0.0373106031000345	0.047617233346592	UP
HTR1D	4.13765901550488	1.26506879405533e-81	1.07983543680811e-79	UP
HTR2B	-1.03643963322772	0.000247632973424735	0.000397312537201227	DOWN
HTR2C	5.13285897721942	2.080565873857e-07	4.29303158685531e-07	UP
HTR3A	-2.60945102792636	4.20799319864148e-17	1.59603105136014e-16	DOWN
HTR3C	-2.36641406174517	4.25796594459115e-06	7.96219512383651e-06	DOWN
HTR3E	-3.23905330343192	7.55109947426485e-20	3.34743623078854e-19	DOWN
HTR4	-2.84147304620609	1.74078827085149e-23	9.44604118427453e-23	DOWN
HTR7	-2.36526003785194	1.67951133180913e-29	1.23711213285924e-28	DOWN
HTRA3	1.15956769168876	6.70694791383758e-11	1.75726879361501e-10	UP
HTRA4	1.54838044016853	3.85946828106768e-12	1.09525621216153e-11	UP
HUNK	1.03853603266265	1.10242419390856e-07	2.32262704035449e-07	UP
HVCN1	-1.3056214696922	1.43869757409998e-24	8.26800028310329e-24	DOWN
HYAL3	1.43043495317929	9.76261670184345e-35	9.41808557502493e-34	UP
HYDIN	2.09208024021064	5.72914089399096e-09	1.31785376997443e-08	UP

GeneSymbol	log2FoldChange	pvalue	padj	change
IARS1	1.08494843160795	3.51269805277584e-58	1.04379882581887e-56	UP
IBSP	5.86569452269465	2.38826252929367e-52	5.45043452458594e-51	UP
ICAM3	-1.44091500324942	1.77287016736435e-14	5.74985811590901e-14	DOWN
ICAM5	1.47532859870957	1.5520578170467e-11	4.23538360260684e-11	UP
IER3	1.21259896693431	8.05783490227061e-17	3.00002307362066e-16	UP
IER5L	2.18218169901885	7.48772370652538e-50	1.49522629474457e-48	UP
IFI30	1.23034068663434	2.55723493046254e-11	6.88559329583179e-11	UP
IFI6	1.62170894498534	1.34037776892302e-16	4.93863692134077e-16	UP
IFITM1	2.1837223857424	1.17120651301981e-43	1.7327288879536e-42	UP
IFITM2	1.45376122255465	5.79494146672447e-20	2.59201941330781e-19	UP
IFITM3	1.5647420526726	6.154030921229e-31	4.85987031434742e-30	UP
IFITM5	4.10854824163514	2.47707265948377e-19	1.0689780971398e-18	UP
IFNE	4.08046565401301	5.003075598074e-14	1.58512655366198e-13	UP
IFRD2	1.26013800421994	2.13269888241723e-38	2.46101296484633e-37	UP
IGF1	-1.81219614880172	2.47666471816075e-14	7.9745123404832e-14	DOWN
IGF2	5.67423529720253	4.09693394708608e-37	4.43932239082824e-36	UP
IGF2BP1	4.88281803838762	3.98986368878737e-33	3.54828888727551e-32	UP
IGF2BP3	3.889091587427	4.35404351656969e-27	2.85562936926659e-26	UP
IGFBP1	3.46032901750263	1.38916699408431e-18	5.73348302156672e-18	UP
IGFBP6	-1.86513516676601	5.40841580569426e-22	2.69076412641583e-21	DOWN
IGFL1	6.67914623178069	1.00854643686064e-35	1.02341603176307e-34	UP
IGFL2	3.34795082328035	7.714090372234e-27	4.98794859456779e-26	UP
IGFL3	3.08210715208772	1.63924448316366e-13	5.06461394345791e-13	UP
IGFL4	5.60422932011277	2.41727544085819e-100	4.78318377859814e-98	UP
IGFN1	1.77960594199165	7.87442791118094e-10	1.92337513280115e-09	UP
IGLL1	-1.77468996822423	5.48881776174632e-08	1.180689112851e-07	DOWN
IGLL5	-2.33586802178244	4.17303870772663e-18	1.67817836068461e-17	DOWN
IGSF10	-2.73392344557333	4.00594523117718e-29	2.88842750768067e-28	DOWN
IGSF11	-3.39822782417747	8.28128453638357e-33	7.26824635242173e-32	DOWN
IGSF23	2.90240903430884	3.99148092315409e-32	3.36742525750398e-31	UP
IGSF3	-1.24116872394212	1.47600780011694e-39	1.80236492450465e-38	DOWN
IGSF5	-1.63183979897254	1.36674576881455e-12	3.98512124453579e-12	DOWN
IGSF9	-1.58019624041756	4.38666659547488e-29	3.15379956346012e-28	DOWN
IGSF9B	-1.25645698018347	1.46099837396385e-09	3.50225284771924e-09	DOWN
IKZF1	-1.62998080920195	8.43130219881802e-23	4.39432101730075e-22	DOWN
IKZF3	-1.13142875858491	1.58067919590049e-09	3.77666943444226e-09	DOWN
IL10	-1.23355614355901	2.8265678409502e-10	7.09821543329474e-10	DOWN
IL10RA	-1.43073104976914	1.35226231295751e-19	5.91334597075066e-19	DOWN
IL11	4.69229634047691	1.15331031041911e-68	5.62537603230476e-67	UP
IL11RA	-1.08837597432783	5.15514561518184e-21	2.43269784816155e-20	DOWN
IL13	1.54914200406782	2.3849367618767e-06	4.54911861480545e-06	UP
IL13RA2	1.41084846863089	5.51078448068672e-11	1.45392863882118e-10	UP
IL16	-1.82058311367264	8.69710438130057e-33	7.62797581935465e-32	DOWN
IL17A	2.63198729805995	2.2704317813023e-15	7.75652905784126e-15	UP
IL17B	-1.23319272797518	9.5150010469374e-14	2.96926009373335e-13	DOWN
IL17C	2.90422865577064	2.23550626032697e-19	9.66886997294423e-19	UP
IL17F	2.38594207395523	4.55679422426406e-10	1.13095008305459e-09	UP
IL1A	2.47609051830832	1.27967958580615e-23	6.98747589452573e-23	UP
IL1R2	-2.08602483013301	3.91826921885808e-21	1.86315734319978e-20	DOWN
IL1RAPL1	-2.22994142949457	1.73468388167464e-14	5.63125474116341e-14	DOWN
IL1RL1	-1.00999106462063	1.66907849746568e-07	3.46780382727239e-07	DOWN
IL1RL2	1.04647900734713	1.04362415768599e-07	2.20247575539766e-07	UP
IL1RN	1.77095485869419	2.39239987622298e-17	9.21044860594091e-17	UP
IL2	-2.16399147907063	2.19289762015398e-15	7.50342429941858e-15	DOWN
IL20	1.91122067556001	2.2153974312078e-06	4.23873370724331e-06	UP
IL20RA	1.33733893976347	1.22577509112555e-32	1.06668773921885e-31	UP
IL22	1.03078120361946	0.0116418247973924	0.015801628747135	UP
IL22RA2	-1.0863472371678	2.39834454957477e-05	4.21119024319304e-05	DOWN
IL23A	2.77385692397345	1.13055343405369e-47	2.03581457571634e-46	UP

GeneSymbol	log2FoldChange	pvalue	padj	change
IL24	3.40457566858932	2.63359523900499e-27	1.74368037630395e-26	UP
IL36B	3.69266168644881	4.79935377148069e-10	1.18860968884644e-09	UP
IL36G	1.64499972985106	6.7209202463597e-09	1.5396840448607e-08	UP
IL36RN	3.61434738793344	8.16086164510338e-16	2.86040828957699e-15	UP
IL37	1.42334038235239	1.55940254036267e-06	3.0217979563026e-06	UP
IL5RA	-1.9211618360785	1.10561991102928e-16	4.08490547650176e-16	DOWN
IL6R	-2.79537542370444	7.43257411443301e-106	1.72564550739496e-103	DOWN
IL6ST	-1.3473319307896	2.87732452526925e-41	3.82756699453884e-40	DOWN
IMPA1	-1.1483439535448	2.18425286868847e-29	1.59808383203389e-28	DOWN
IMPDH1	1.64697860393292	3.14577153598894e-59	9.85203592736966e-58	UP
INA	-2.76774101741876	8.27718794844434e-20	3.66278713459368e-19	DOWN
INAFM2	-1.62278969654432	7.4117513737702e-81	6.08777484299342e-79	DOWN
INHA	2.40723711190432	1.4835598478451e-11	4.05545174733542e-11	UP
INHBA	5.0170094417002	9.9141104115899e-151	6.16551444989339e-148	UP
INHBB	2.36061314094934	1.22989489871309e-23	6.72406903337239e-23	UP
INHBE	2.99264334379651	1.94892058707981e-31	1.5836002885124e-30	UP
INO80B	1.53227853070573	7.21928447000949e-20	3.2027872732809e-19	UP
INPP5D	1.04927923098671	5.46676064224122e-17	2.06134047343756e-16	UP
INSL4	3.34333023636493	1.61462640815309e-06	3.12602731211583e-06	UP
INSL5	-5.87797564284929	4.73967521856573e-26	2.94967707580004e-25	DOWN
INSM1	-1.14251371978942	0.000701183054258125	0.00108069574471559	DOWN
INSYN1	-1.53719287461466	6.84236674657439e-17	2.55788175521897e-16	DOWN
INSYN2B	1.31644654323131	2.21901595048371e-06	4.24472423879741e-06	UP
INTS13	1.12855387392804	1.57063381061271e-42	2.2073806734624e-41	UP
IP6K3	-1.74100433435648	4.08826048333081e-22	2.04624546698015e-21	DOWN
IPCEF1	-1.05186455635957	3.21064536863111e-11	8.59049904793694e-11	DOWN
IPO4	3.14612679735781	2.42304829324721e-41	3.23810743901103e-40	UP
IQANK1	2.87660303634635	1.50130416567316e-132	6.37614864313821e-130	UP
IQCM	-2.59220619232937	2.87422136097146e-18	1.16372304338753e-17	DOWN
IQCN	-2.14412257400826	7.87689427391414e-39	9.26759189132884e-38	DOWN
IQGAP2	-1.69885612752092	9.54693758448768e-28	6.45595433625957e-27	DOWN
IQGAP3	1.917049011596	1.71312930078197e-74	1.0808232070477e-72	UP
IRAG1	-1.78596195662008	1.93754986236911e-30	1.48956096041648e-29	DOWN
IRAG2	-2.47647650592594	2.38912314771682e-40	3.05670840346753e-39	DOWN
IRAK1	1.1158916717991	9.71659570504301e-33	8.50226537748311e-32	UP
IRAK2	1.60483255643859	5.20258721806623e-32	4.37223220213259e-31	UP
IRF4	-2.47714564608634	1.0916066539556e-32	9.52311957180804e-32	DOWN
IRX2	2.78964867295796	6.90276326328457e-14	2.16963568056993e-13	UP
IRX3	3.21216188327319	1.17357476801283e-24	6.77119199317677e-24	UP
IRX4	1.89203578384882	0.00107904024052814	0.00163371252137349	UP
IRX5	4.21206456720965	2.27973588236866e-57	6.63830115713804e-56	UP
IRX6	-2.17982274304754	4.79260982668686e-06	8.92076054645626e-06	DOWN
ISM1	1.27411640031982	5.78704143701598e-07	1.15774072314751e-06	UP
ISM2	3.83929997098727	7.24417222771496e-29	5.15078689265825e-28	UP
ISX	-2.25728925276605	1.53654381392308e-26	9.78275591657865e-26	DOWN
ITGA11	1.86138849109314	1.05958378990035e-22	5.49777489080298e-22	UP
ITGA2	1.2396979852414	5.47332615546175e-31	4.32820292211878e-30	UP
ITGA4	-1.00690972781354	2.79180187101009e-13	8.49888915732495e-13	DOWN
ITGA7	-1.94167240425455	9.09194648388364e-35	8.79544800688144e-34	DOWN
ITGA8	-1.89907082821733	6.70892433465977e-44	1.00276823553159e-42	DOWN
ITGAL	-1.14080475707005	1.71935079766767e-12	4.98568783343666e-12	DOWN
ITGAX	1.03905787819739	2.64651020359259e-09	6.21241334256644e-09	UP
ITGB1BP2	-1.3696570575921	5.17144052930697e-23	2.72384434170666e-22	DOWN
ITGB3	-1.46798218450059	5.0609380234804e-23	2.66725526037724e-22	DOWN
ITGB7	-1.25370736429884	1.94783784402232e-12	5.62949383866568e-12	DOWN
ITGB8	1.00313660421092	3.62368215859167e-12	1.02918247312929e-11	UP
ITGBL1	2.33227575824529	1.64377019819465e-19	7.15574261529087e-19	UP
ITIH2	1.38135953526786	1.87405686928445e-06	3.60904139182151e-06	UP
ITIH5	-2.09437851262725	5.85843914466639e-39	6.93965992014122e-38	DOWN

GeneSymbol	log2FoldChange	pvalue	padj	change
ITIH6	2.09816562141284	2.79151783288557e-08	6.13049565191531e-08	UP
ITLN1	-2.13255250633155	2.22907295974882e-08	4.92261857300014e-08	DOWN
ITLN2	2.42535736295888	6.76812064920487e-08	1.44445746861876e-07	UP
ITM2A	-2.33094072258285	8.95508546124689e-45	1.39476657546236e-43	DOWN
ITM2C	-2.23100478802249	1.66651181730958e-52	3.83341747355504e-51	DOWN
ITPKA	-1.34928489627375	7.16323493561878e-16	2.51897521294263e-15	DOWN
ITPKB	-1.05402811563389	2.29300501629449e-34	2.16178106923313e-33	DOWN
ITPR1	-1.52358031806463	3.63295543091069e-28	2.49548926699991e-27	DOWN
ITPRID1	-2.02727972350457	1.5465702581785e-11	4.22173191811604e-11	DOWN
IVL	4.59235579857062	4.29907348107752e-10	1.06850937091069e-09	UP
IZUMO1	2.32179338829703	3.40403505872744e-19	1.45673291908629e-18	UP
IZUMO2	2.88707327345157	5.92732992970326e-14	1.8701321990564e-13	UP
JADE3	1.31841373205396	6.27844929693593e-49	1.20536535399719e-47	UP
JAG2	2.06839723606976	2.18216696524485e-45	3.52814051678817e-44	UP
JAKMIP2	-1.16202024194878	7.93974794835253e-09	1.81104049023661e-08	DOWN
JAKMIP3	-1.23091029318714	8.1791478862964e-12	2.27804705924631e-11	DOWN
JAM2	-2.53828437544716	1.07252476642473e-53	2.61201031576977e-52	DOWN
JAM3	-1.85675663933645	7.00131578116067e-33	6.16973237334771e-32	DOWN
JAML	-1.63437705390288	3.90044708727067e-29	2.81936426445181e-28	DOWN
JAZF1	-1.86325034194762	1.73238875578145e-46	2.96618342226376e-45	DOWN
JCAD	-1.81698648214547	1.73902781314582e-25	1.04672282441438e-24	DOWN
JCHAIN	-3.77831501814853	1.18656969948306e-38	1.38576379457402e-37	DOWN
JMJD7	1.3189514488149	2.80522316650008e-10	7.04562974156439e-10	UP
JPH1	1.59391583856626	7.52548277960239e-49	1.43843283909129e-47	UP
JPH2	-3.21833853026106	5.08475630106316e-45	8.02000556797218e-44	DOWN
JPH3	-1.71391890076424	6.50837152052422e-13	1.93396370796738e-12	DOWN
JPH4	-2.62146606981202	1.57981985139411e-39	1.92642878657742e-38	DOWN
JSRP1	1.20534330374658	1.33115036848236e-06	2.59479697373595e-06	UP
KAAG1	1.78691897634059	2.28141650038914e-05	4.01356895547344e-05	UP
KANK2	-1.95950056434763	2.41220036676347e-61	8.25218958476469e-60	DOWN
KANK4	-1.0653925043797	4.57006465221817e-06	8.52657621226562e-06	DOWN
KASH5	1.49830732078527	0.000505495613651388	0.000788727161336167	UP
KAT2A	1.62890116869549	1.92594243388868e-90	2.50271907472415e-88	UP
KAT2B	-1.84512794650598	1.43591287810777e-54	3.66084201266334e-53	DOWN
KATNAL1	-1.07915169021609	5.71735387698306e-17	2.15210296281682e-16	DOWN
KBTBD11	-1.16781218871775	3.54593143632203e-22	1.78351542751807e-21	DOWN
KCNA1	-4.2360615712669	9.37733332810116e-26	5.74956004374034e-25	DOWN
KCNA10	2.18657623294819	6.76051357361009e-07	1.34599614517805e-06	UP
KCNA2	-1.28583186275162	3.89053347005452e-07	7.87101885837798e-07	DOWN
KCNA3	-2.29334413454259	6.71400702917343e-26	4.15757483637969e-25	DOWN
KCNA5	-2.67875998717434	3.98096359339893e-56	1.09166171735206e-54	DOWN
KCNA6	-2.40587748662014	1.48913915149915e-17	5.79838551991383e-17	DOWN
KCNAB1	-1.91345871581945	1.29503170376506e-39	1.59032600541202e-38	DOWN
KCNB1	-3.50108363415593	6.84613335003172e-57	1.95109198075454e-55	DOWN
KCNB2	-1.79920644273334	2.60518181232748e-09	6.11952393067022e-09	DOWN
KCNC1	-1.64257635063573	8.14318095405795e-18	3.2211996808953e-17	DOWN
KCND3	-1.29503851381784	5.49287973819057e-10	1.35458879593701e-09	DOWN
KCNE5	1.04246464411245	4.62049644417623e-06	8.61697596470394e-06	UP
KCNG1	-1.11543392655756	6.571937522142e-05	0.00011081354514676	DOWN
KCNG3	-2.21542531748463	1.10360619650832e-18	4.57332096615881e-18	DOWN
KCNH1	-1.25509146392441	3.99852400045971e-16	1.42764606151333e-15	DOWN
KCNH4	2.15013948508279	1.72726246501863e-23	9.37849120778592e-23	UP
KCNH5	-1.63535199514139	0.00515828548988314	0.00727886752312279	DOWN
KCNH8	2.52004687316682	1.65917866360904e-44	2.55901488657433e-43	UP
KCNIP1	-2.13956164073907	6.43229724132056e-17	2.41027742335087e-16	DOWN
KCNIP3	-1.65244906172084	1.21441538896606e-19	5.32392124070141e-19	DOWN
KCNIP4	-3.65213570640953	2.14110619881351e-83	2.09455518201909e-81	DOWN
KCNJ11	1.45516875260156	3.07602324230613e-23	1.64151985039156e-22	UP
KCNJ13	1.12973670816119	0.0213158266097359	0.0279918920629963	UP

GeneSymbol	log2FoldChange	pvalue	padj	change
KCNJ14	2.03439398152132	7.50126689336806e-55	1.93797567380145e-53	UP
KCNJ15	1.97547671422405	2.48490626665064e-16	8.99951597778445e-16	UP
KCNJ16	-1.90039465920434	6.79964122674282e-09	1.55648945288909e-08	DOWN
KCNJ5	-1.20368516931147	6.61914075882112e-08	1.41474282598935e-07	DOWN
KCNJ9	-1.18841798848605	4.00628737947797e-07	8.09767639452698e-07	DOWN
KCNK10	-1.7694167735447	2.34466554359765e-18	9.56151314067116e-18	DOWN
KCNK15	1.63327820092311	6.39864149945052e-09	1.46836510845983e-08	UP
KCNK2	-2.01933768708589	9.56610985677937e-11	2.48693148605702e-10	DOWN
KCNK3	-2.94705799875637	1.53846706458975e-26	9.79142068556332e-26	DOWN
KCNK9	2.31097066851465	5.8653503691051e-18	2.3398246501083e-17	UP
KCNMA1	-3.14534837326893	2.85817375455848e-44	4.32777213809799e-43	DOWN
KCNMB1	-3.33405928778725	7.09729819082523e-49	1.36107107265242e-47	DOWN
KCNMB2	-2.08825324884199	6.64844161145164e-18	2.6413258904907e-17	DOWN
KCNN3	-2.55007824475424	1.01473299581408e-68	4.96335552137938e-67	DOWN
KCNN4	1.63417530687024	1.16575932808839e-25	7.09519999301051e-25	UP
KCNQ1	1.08322598967339	5.30269795737161e-15	1.78082699193273e-14	UP
KCNQ5	-2.23263928477864	1.36248142798599e-14	4.4561616544726e-14	DOWN
KCNS1	1.40870289613422	1.83114888069577e-11	4.97360715326086e-11	UP
KCNS2	-2.492440215107	4.32317669522476e-25	2.55011774369745e-24	DOWN
KCNS3	-1.03017548210951	1.0411400433492e-10	2.69862631361114e-10	DOWN
KCNT1	1.92511337779222	1.76713574026444e-11	4.80573709905117e-11	UP
KCNT2	-1.97744914953494	4.28142688672957e-23	2.269482081541e-22	DOWN
KCNV2	1.75135284161788	6.56243900809446e-11	1.72096009710766e-10	UP
KCP	1.98150661369866	4.65799986661004e-40	5.8775182374841e-39	UP
KCTD12	-1.55594942240236	4.21416130623579e-20	1.89762582946687e-19	DOWN
KCTD14	1.68117926547596	2.33260589917447e-40	2.98659312664154e-39	UP
KCTD16	1.78271505436149	8.67824171919631e-09	1.97251302775572e-08	UP
KCTD19	1.50230909580059	1.12302470125384e-11	3.10105124055394e-11	UP
KCTD4	-3.01453602584141	2.62199670430018e-20	1.19489213849723e-19	DOWN
KCTD8	-2.84411621433667	6.42127954832659e-11	1.68470304015385e-10	DOWN
KCTD9	-1.42638586028068	1.87803106592776e-40	2.41701071330377e-39	DOWN
KERA	-1.17669832163399	0.000200138121820116	0.000323374326366678	DOWN
KHDC4	1.00700683832464	6.24011520224977e-31	4.92115606960033e-30	UP
KHDRBS2	-4.4835256159361	5.01321792977008e-48	9.16003817535009e-47	DOWN
KIAA0513	-1.96484974235683	7.8562772267322e-52	1.73826372743441e-50	DOWN
KIAA0895	1.3194672528981	4.68183634541149e-25	2.75701103424586e-24	UP
KIAA1210	1.21652231909896	0.000725573315145653	0.00111611379298863	UP
KIAA1549	2.27155281223953	4.50581608893923e-116	1.20707347010306e-113	UP
KIAA1549L	1.98731902446464	1.00285273524875e-12	2.94778438198625e-12	UP
KIAA2012	1.69838415065108	2.87438856578978e-18	1.16372304338753e-17	UP
KIF12	1.60741256724509	1.27641416681863e-20	5.91436931527748e-20	UP
KIF13B	-1.30825695762502	1.90985880556056e-35	1.91018790242539e-34	DOWN
KIF14	1.71097284680968	8.91729095508104e-45	1.39022352715436e-43	UP
KIF18A	1.31216418431278	5.03690259320474e-40	6.32810857543104e-39	UP
KIF18B	1.81513867512369	2.27571308006909e-73	1.37593721747372e-71	UP
KIF1A	-2.6529178209424	1.78224288676209e-15	6.1332401950965e-15	DOWN
KIF20A	1.52488858278602	6.94344739511195e-66	3.00761814654439e-64	UP
KIF21B	1.16296636841271	1.15046072357061e-21	5.62407989318784e-21	UP
KIF23	1.31699954874639	5.19889327520319e-51	1.11077703805047e-49	UP
KIF24	1.05914817007292	2.31445821132869e-36	2.42197480972755e-35	UP
KIF25	1.73929300958138	4.72122257147571e-12	1.33220950635402e-11	UP
KIF26B	3.0776463561606	4.26293679837547e-52	9.61535213343419e-51	UP
KIF2C	1.45814101066082	6.8770379199205e-65	2.84441475770963e-63	UP
KIF4A	1.30276965663561	2.48353799137602e-49	4.86454972371548e-48	UP
KIF4B	1.8106123501788	1.97597015598746e-11	5.3535970633592e-11	UP
KIF5A	-2.46731594835156	8.83924179429557e-30	6.60591061648363e-29	DOWN
KIF5C	-2.03362105876189	9.46984621209303e-22	4.64764464744013e-21	DOWN
KIF9	1.00620738907941	2.12197729081779e-29	1.55448004059782e-28	UP
KIFC1	1.21838897688829	1.49950738285096e-42	2.10912132936863e-41	UP

GeneSymbol	log2FoldChange	pvalue	padj	change
KIFC2	1.70880396471836	1.21266661379577e-28	8.52833753878259e-28	UP
KIFC3	1.14200085350363	3.25855498235417e-16	1.17068340635264e-15	UP
KIR2DL4	-2.09508142670727	2.94084639380248e-15	1.00017496592349e-14	DOWN
KIR3DL2	-1.34597613868901	1.32815523154152e-05	2.38498164863694e-05	DOWN
KIRREL3	-2.08274472633744	1.38277766113038e-23	7.53388842717878e-23	DOWN
KISS1	3.44721284005237	1.50396098145597e-43	2.2099976852399e-42	UP
KISS1R	3.14027131303351	9.15143271009758e-22	4.49390574678311e-21	UP
KIT	-1.77317597680278	6.24406764184761e-20	2.7871814880157e-19	DOWN
KITLG	-1.29779854197627	6.89588889831041e-23	3.61137183116629e-22	DOWN
KL	-1.51337617671945	3.6354395960867e-11	9.69135175852076e-11	DOWN
KLB	-2.33295983120843	6.2360653940813e-52	1.39216162445048e-50	DOWN
KLC3	6.03217036123157	2.2494860946116e-64	8.96345569003932e-63	UP
KLF1	1.25314915058083	2.17829728086261e-09	5.14593549744411e-09	UP
KLF15	-1.05987589335767	2.65874025833506e-07	5.44218221680832e-07	DOWN
KLF4	-2.49042856699781	1.03572124370871e-66	4.6602103402325e-65	DOWN
KLF8	-1.27064051790072	1.06956952964668e-20	4.97579861601328e-20	DOWN
KLF9	-1.98224088912573	1.96995064432901e-46	3.35972091769844e-45	DOWN
KLHDC1	-1.45123035053165	2.44169625911661e-33	2.19841039089956e-32	DOWN
KLHDC8B	-1.49744898074132	8.92263572902944e-35	8.64126006393713e-34	DOWN
KLHL10	-1.79518940695622	7.84608032607835e-20	3.47538050556044e-19	DOWN
KLHL17	1.73135917193024	1.54976398564013e-37	1.71666922913178e-36	UP
KLHL29	1.00956427590929	9.66255111987442e-15	3.19570755271364e-14	UP
KLHL31	1.69332064585471	1.20777054092372e-31	9.95781649105336e-31	UP
KLHL32	1.15283161646727	2.06278854033133e-07	4.25735887789374e-07	UP
KLHL33	-1.30246206613132	4.22384380278388e-13	1.27160774788859e-12	DOWN
KLHL34	-1.9094176615248	1.37032964426509e-09	3.29034060887866e-09	DOWN
KLHL35	3.89855849606659	1.90165173916339e-82	1.74281377547643e-80	UP
KLHL38	-1.2612780982411	6.90435143160301e-08	1.47262948895766e-07	DOWN
KLHL4	-1.36236255048761	6.66434526205098e-12	1.86479582272367e-11	DOWN
KLHL6	-1.60673583510185	2.6004629676461e-20	1.18538904857648e-19	DOWN
KLK1	-1.18650797586948	2.02971730371399e-09	4.80667311431683e-09	DOWN
KLK10	5.18759917383697	3.41262049148461e-78	2.6063140622027e-76	UP
KLK11	3.24691541480647	1.39796562147952e-28	9.79194503894727e-28	UP
KLK12	3.29641296500481	1.2213491070133e-22	6.30704389099127e-22	UP
KLK13	-1.05464208111849	0.00119560335796586	0.00180251439586662	DOWN
KLK15	-1.93033512488159	5.19411499647349e-15	1.74537098482425e-14	DOWN
KLK2	1.09706932916161	0.000488599614551195	0.000763801516130708	UP
KLK5	4.09744523915371	2.07508340714891e-11	5.61426777014977e-11	UP
KLK6	9.75532179511027	2.10159826099036e-140	1.04557515767501e-137	UP
KLK7	8.30062295416215	2.34439478569125e-85	2.45921363874951e-83	UP
KLK8	8.06077465680425	8.41986891539555e-65	3.43361071250077e-63	UP
KLRB1	-1.87794148339364	1.79870718926213e-28	1.25333686621134e-27	DOWN
KLRC1	-1.43312644792889	8.23887147432194e-10	2.00957373557036e-09	DOWN
KLRC4-KLRK1	-1.18019995675417	2.78961499169521e-05	4.87510697013133e-05	DOWN
KLRF1	-2.20347357457225	1.4126116440168e-28	9.89055350111159e-28	DOWN
KLRG1	-1.02640693122381	1.40547618199511e-11	3.84864864869961e-11	DOWN
KLRG2	3.30419541997437	3.97931198076741e-19	1.69749533368699e-18	UP
KNDC1	1.10027474926304	4.22523629513544e-06	7.9060863536636e-06	UP
KNG1	-1.3470646892366	1.92269969974361e-06	3.6994441847111e-06	DOWN
KNSTRN	1.03466752049523	8.81789300398609e-32	7.32999497288883e-31	UP
KNTC1	1.1342679419804	6.44609081540295e-34	5.9452213648629e-33	UP
KPNA2	1.1821967908068	2.90308556577606e-50	6.01087145741481e-49	UP
KRCC1	-1.12073242281329	4.46135486496223e-23	2.35982904810411e-22	DOWN
KREMEN2	2.50667163326428	9.94403124406115e-38	1.11282401062234e-36	UP
KRT1	-2.46650615223167	3.38462246157409e-16	1.2141827549112e-15	DOWN
KRT12	-1.30083080732741	8.49608008996397e-09	1.9331274350783e-08	DOWN
KRT13	2.33514251505103	1.23357618109582e-10	3.18178966692659e-10	UP
KRT15	1.65967963025241	1.32262370480148e-24	7.61098697016132e-24	UP
KRT16	4.88268373200861	7.59823440485824e-43	1.08716561784549e-41	UP

GeneSymbol	log2FoldChange	pvalue	padj	change
KRT17	5.00125422084376	2.44858042146517e-71	1.34501990154489e-69	UP
KRT20	-2.72616428547019	1.80780085099425e-40	2.33006929817638e-39	DOWN
KRT222	-2.95671459271951	1.062337229899e-22	5.51041947698283e-22	DOWN
KRT23	7.43325524393048	2.85519646277836e-133	1.27480861554768e-130	UP
KRT24	-6.03856207953795	1.6423675615027e-16	6.01821261541382e-16	DOWN
KRT3	2.46204469987713	4.73865216827885e-06	8.82629649858576e-06	UP
KRT31	5.55420916876485	1.31643386064969e-17	5.13970018284599e-17	UP
KRT32	4.4352772417929	1.46337801413952e-13	4.52687890570464e-13	UP
KRT34	3.68053206657788	9.61576731778129e-11	2.49872192664566e-10	UP
KRT35	4.4404365717445	5.06932932261857e-12	1.42789115973402e-11	UP
KRT36	1.34243955685986	2.8627153848664e-06	5.43011579484516e-06	UP
KRT37	4.47925747415804	1.09326058038011e-15	3.80815092741727e-15	UP
KRT38	3.88320845741679	6.53053456597287e-10	1.60299123762737e-09	UP
KRT39	1.67559999105449	3.10556743179488e-05	5.40448187985651e-05	UP
KRT4	2.72787113630461	1.49537114266744e-10	3.83432450408895e-10	UP
KRT40	2.13448810427199	5.46990278008727e-08	1.17705656339174e-07	UP
KRT5	2.85587137525896	2.22815783162802e-14	7.1889776398256e-14	UP
KRT6A	5.62950149152561	3.71443418798987e-45	5.91219767051806e-44	UP
KRT6B	5.93291457694893	1.54115886079988e-70	8.08319254310492e-69	UP
KRT6C	4.60149754202572	3.46368185500074e-19	1.4815301434814e-18	UP
KRT7	3.26006473007921	1.87231958086303e-37	2.06476889560278e-36	UP
KRT74	3.33963698202744	4.37638159895211e-08	9.48070823370901e-08	UP
KRT75	7.45294744041699	3.09868480681109e-60	1.01614686517894e-58	UP
KRT78	2.49339116899846	8.22613215631388e-07	1.62793089257749e-06	UP
KRT80	6.65957203271537	0	0	UP
KRT83	3.77111825152236	1.91293585176235e-23	1.03318709636283e-22	UP
KRT84	2.06961524271302	0.000410461213665504	0.000645651410438791	UP
KRT9	-3.13751335979502	7.44403133326596e-23	3.89024362563506e-22	DOWN
KRTAP13-2	-5.28488771288395	1.49326535492848e-20	6.8843605044664e-20	DOWN
KRTAP3-1	4.79941114137329	2.25413834684889e-17	8.68968586089878e-17	UP
KRTAP3-2	3.41624394561543	2.79748014056083e-13	8.51319847738305e-13	UP
KRTAP3-3	2.14643503707109	1.39469482589912e-05	2.50214516828574e-05	UP
KRTAP4-1	1.17052172186627	3.23203567563388e-08	7.06939294307409e-08	UP
KRTAP5-1	2.21240362477908	7.1499472537011e-37	7.65695150852997e-36	UP
KRTAP5-4	4.148546898695	7.49616807120625e-29	5.32344105317759e-28	UP
KRTCAP2	1.39684613792084	7.08740991667898e-31	5.57421268650095e-30	UP
KY	-2.90518140737803	5.14284788772709e-39	6.11696791454862e-38	DOWN
KYAT1	1.99058942506647	7.03206946091136e-18	2.78991627985531e-17	UP
L1CAM	-1.50271914810784	2.78707346210364e-07	5.69415818322312e-07	DOWN
L1TD1	1.02390061526743	0.00591533334044827	0.00829070343345345	UP
L3MBTL1	1.33848451636372	2.02670754863601e-14	6.54993662665162e-14	UP
LACTB2	1.09202012906069	3.4710437751572e-19	1.48431447094333e-18	UP
LAGE3	1.26216485802776	1.11635040810339e-27	7.52866369337887e-27	UP
LAIR2	2.5575182736088	1.6957574083908e-24	9.71323149747006e-24	UP
LAMA1	-2.65202816118675	2.7975366764789e-41	3.72712365321554e-40	DOWN
LAMA2	-1.02267817506524	6.67397518595354e-08	1.42611277350606e-07	DOWN
LANCL3	-1.75071820691471	4.08550674661235e-28	2.80082397554176e-27	DOWN
LAPTM4B	1.48600386378578	9.90910383406907e-25	5.74200416181846e-24	UP
LARGE1	-1.26003135774111	1.32063336628484e-43	1.94882955992524e-42	DOWN
LARGE2	4.51693087690745	3.45246146911867e-142	1.82174883520495e-139	UP
LAS1L	1.05048627132091	3.09421431607765e-36	3.22246135680982e-35	UP
LAX1	-1.44109549641213	1.69885088526085e-12	4.92706370170672e-12	DOWN
LBP	2.6991045000567	1.60492706581923e-17	6.23668712276508e-17	UP
LBX2	1.78291252771861	2.56651151874636e-37	2.81073365257424e-36	UP
LCE1E	3.94595764488795	5.26043195243598e-09	1.21388684849944e-08	UP
LCN1	2.55364013990484	2.3461744602283e-10	5.92343567869442e-10	UP
LCN10	-2.51138995097365	5.64717228164168e-16	2.00069605168315e-15	DOWN
LCN12	2.18319405435802	1.31294620074273e-29	9.70799668515212e-29	UP
LCN15	3.38247801373882	4.2622642914024e-14	1.35485228379317e-13	UP

GeneSymbol	log2FoldChange	pvalue	padj	change
LCN2	2.31065948930912	1.73861019431527e-20	7.98797343367066e-20	UP
LDB3	-4.19857637544682	8.41531160755079e-65	3.43361071250077e-63	DOWN
LDHB	1.45073334537735	1.04219576303374e-18	4.32501306523034e-18	UP
LDHD	-2.91907824036869	1.2580780160315e-56	3.53337298276719e-55	DOWN
LDLRAD1	1.6197588540395	1.43982071668671e-08	3.22008709731129e-08	UP
LDLRAD2	-1.99861482967267	4.88093225452389e-40	6.15435723012487e-39	DOWN
LDLRAD3	1.88675428313379	1.96859335569906e-45	3.1946986116298e-44	UP
LDOC1	-1.10529746768701	2.75506584176356e-11	7.40567482288188e-11	DOWN
LEAP2	-1.69603210252504	4.27355676617562e-40	5.4080991256843e-39	DOWN
LEF1	2.06428909397838	4.48717304536126e-40	5.67018463271957e-39	UP
LEMD1	7.12506251720995	8.653498458289e-97	1.52205422883016e-94	UP
LEP	-2.20562426586788	9.52776228662971e-07	1.8750782628513e-06	DOWN
LEPR	-1.52805303034149	4.28835887960815e-26	2.67549957615968e-25	DOWN
LEXM	-2.6075672513225	5.78850695426136e-54	1.42972016446175e-52	DOWN
LGALS12	-1.68925989164107	9.16639048842217e-09	2.07885331564073e-08	DOWN
LGALS14	1.53476339785942	0.00974005935620958	0.0133241930685582	UP
LGALS2	-2.67050019087161	2.29173212988451e-28	1.58545616121092e-27	DOWN
LGALS3	-1.17727125388594	9.98223634799787e-30	7.44095383252084e-29	DOWN
LGALS4	-1.85861789073064	5.42890853593728e-50	1.10050738458994e-48	DOWN
LGALS7	2.38625490230168	4.15691109245443e-05	7.14483198627074e-05	UP
LGALS7B	2.82465811105637	1.23734907546067e-15	4.29459028323633e-15	UP
LGALS9B	-2.011895920445	1.4559004482852e-14	4.75193898893913e-14	DOWN
LGALS9C	-2.65902252657532	3.91933945468509e-24	2.20295216024634e-23	DOWN
LGI1	-5.26495909056785	2.63518568357333e-63	9.88932937673759e-62	DOWN
LGI3	-1.91145300254365	4.64865541134535e-10	1.15276326798286e-09	DOWN
LGI4	-2.09166568518423	3.30567332642594e-27	2.1787164887606e-26	DOWN
LGR4	-1.31641353883062	1.02118282057201e-42	1.44921405498129e-41	DOWN
LGR5	2.87732461417795	2.06610312883396e-42	2.88277674538347e-41	UP
LGR6	2.09232612138281	5.50939133998224e-19	2.32739037853253e-18	UP
LHB	2.13750509495962	3.19683278923138e-12	9.11817352315904e-12	UP
LHFPL1	-1.07287959533115	0.000271210422668804	0.000433503496413794	DOWN
LHFPL4	-1.25209724772803	0.00332694613844569	0.00479292736897119	DOWN
LHFPL5	1.40379572982039	1.01072384301745e-24	5.85292127650912e-24	UP
LHX1	3.89155324801646	4.025432246916e-10	1.00207078935737e-09	UP
LHX3	2.53575470195564	3.03583392240666e-07	6.18859471913688e-07	UP
LHX5	3.73856430580622	2.76121886908061e-15	9.39634632935326e-15	UP
LIF	1.85049164546564	2.20947631625073e-44	3.38378285794846e-43	UP
LIFR	-3.48471432453037	2.59295225031635e-79	2.04303518256826e-77	DOWN
LILRA4	-1.99449675301968	6.11469699485258e-17	2.29472454248638e-16	DOWN
LILRB5	-2.68093727021561	4.15962463049909e-47	7.32371523669167e-46	DOWN
LIMA1	-1.47010398299039	8.46580478134805e-42	1.15619653849109e-40	DOWN
LIME1	2.30498014219846	5.91096518777415e-45	9.28112144406775e-44	UP
LIMK1	1.0482718494246	6.3032591824853e-31	4.96870313013203e-30	UP
LIMS2	-2.41169963190971	1.433471846637e-38	1.67075269514659e-37	DOWN
LIN28A	3.31441016791632	4.54315920643707e-07	9.14989952136118e-07	UP
LIN28B	3.40795786228639	0.00047479824895447	0.000743160621037679	UP
LINC00514	3.06625674089486	3.29864181963259e-25	1.95571161066606e-24	UP
LINC00634	1.3681713375499	2.39317256488414e-11	6.45381971075228e-11	UP
LINGO4	-1.14451934941134	1.30465459904883e-07	2.7338395026205e-07	DOWN
LIPC	-2.17293716504281	1.54125072954907e-14	5.01734884158496e-14	DOWN
LIPF	1.86880025220631	0.0216045430834235	0.0283517905427427	UP
LIPG	1.25522331138844	2.19026940287902e-25	1.30972393929713e-24	UP
LIPH	-1.30942571125396	8.62585707692766e-27	5.56716268645446e-26	DOWN
LIPI	-2.02693357775027	4.62241386064091e-13	1.38776021647138e-12	DOWN
LIPJ	-1.23892353502301	1.71965572625683e-08	3.82675593115784e-08	DOWN
LITAF	-1.29672652919768	1.30743561511462e-71	7.2045494829085e-70	DOWN
LITAFD	2.23621871651375	2.76105581302986e-08	6.06512739652946e-08	UP
LIX1	-2.62194558959679	2.46480771373077e-13	7.53241430663283e-13	DOWN
LKAAEAR1	1.45377580217967	0.00157293299806521	0.00234138162893738	UP

GeneSymbol	log2FoldChange	pvalue	padj	change
LMNB2	1.06815398750699	3.03279907202588e-35	2.99887167752338e-34	UP
LMO1	-1.79250679219233	0.000111544215137668	0.000184403248665357	DOWN
LMO2	-1.26846452277536	7.01324982106594e-28	4.77784503655013e-27	DOWN
LMO3	-3.95539821106959	4.73235232023476e-52	1.0621861956806e-50	DOWN
LMOD1	-3.56695316602912	7.63707293844813e-55	1.970138534477e-53	DOWN
LMTK3	2.24247877857456	1.18002418891273e-54	3.02172958846139e-53	UP
LMX1A	-1.92458525301947	6.84060209656754e-06	1.25874885667897e-05	DOWN
LONRF2	-3.86599128670061	3.16408257794977e-31	2.53782450160476e-30	DOWN
LOX	1.06590131774839	1.83725943279338e-09	4.36932511652979e-09	UP
LOXL2	1.75550156723507	3.75623953852112e-32	3.17511646040137e-31	UP
LOXL4	-1.82538563097501	7.74678864072884e-26	4.77842120442831e-25	DOWN
LPAR1	-2.27166386624845	1.33057274112356e-54	3.4022412835807e-53	DOWN
LPCAT1	1.10174551940027	2.75722629001103e-21	1.32226883470014e-20	UP
LPCAT3	-1.12122043345771	1.78863734770074e-36	1.88190586921529e-35	DOWN
LPO	5.45860222776384	3.40767815624023e-42	4.71309767550525e-41	UP
LPP	-1.61145162311058	2.09147911824831e-52	4.79196393237603e-51	DOWN
LRAT	-3.5644549272317	4.19367146048682e-31	3.33597081505057e-30	DOWN
LRCH2	-2.44273537481927	2.09261057719334e-39	2.53927721119635e-38	DOWN
LRFN4	1.3245431016943	4.02120125596434e-26	2.51332295298302e-25	UP
LRFN5	-2.28892365852558	8.53977036136461e-26	5.25625041622982e-25	DOWN
LRP11	1.05990541178352	8.50463598699517e-32	7.0755483249664e-31	UP
LRP1B	-2.84230873755383	1.47260859721865e-24	8.46009023535744e-24	DOWN
LRP2	2.54389142588218	1.74542175726997e-10	4.44797732465124e-10	UP
LRP5L	1.15667549721838	7.19938320745145e-20	3.1947721659366e-19	UP
LRP8	2.8492433734898	9.06175255989503e-138	4.26465668447168e-135	UP
LRRC1	-1.18249061404736	3.46199627343465e-75	2.27485815506859e-73	DOWN
LRRC10B	1.36247733218378	2.71956014552548e-11	7.31136341115257e-11	UP
LRRC15	1.68874908058424	1.34857576899061e-15	4.67504476715778e-15	UP
LRRC18	-1.46044012144854	6.74914423641663e-14	2.12288382566334e-13	DOWN
LRRC19	-2.85271878640192	1.36335252250311e-50	2.85680595359166e-49	DOWN
LRRC31	-1.55330394957504	8.78807631523309e-22	4.32034931894844e-21	DOWN
LRRC36	3.19115487261042	1.48139552257688e-53	3.56784788860735e-52	UP
LRRC37A3	1.20797990806597	2.71638746683239e-16	9.80726828943654e-16	UP
LRRC38	1.02796888075367	0.00130935744242506	0.00196567300154733	UP
LRRC3B	-3.47624539748315	1.51595733735677e-41	2.04789488870392e-40	DOWN
LRRC43	1.98142989135974	6.19672041055103e-24	3.44409487739946e-23	UP
LRRC46	1.29681076071344	4.36372678305768e-22	2.18098663815681e-21	UP
LRRC4B	-1.10653435240386	8.33219892962385e-12	2.31956163008058e-11	DOWN
LRRC4C	-1.98007734805319	6.48887255936737e-13	1.92849868367066e-12	DOWN
LRRC53	-1.29155480406116	0.00781356185272048	0.0108059369820842	DOWN
LRRC55	-2.00369226986148	1.33521242579604e-23	7.28438994205134e-23	DOWN
LRRC6	1.30769869194136	2.18548626901801e-17	8.43063190128723e-17	UP
LRRC61	1.21295532757676	1.38807069270865e-22	7.1362488845987e-22	UP
LRRC66	-1.83541566293825	5.17531138677483e-34	4.79094615512547e-33	DOWN
LRRC69	1.08667002118059	1.16479612303613e-13	3.61930672562957e-13	UP
LRRC7	-1.81853353709579	2.04203110283808e-46	3.47584433956202e-45	DOWN
LRRC73	1.2313008349619	4.71291505167785e-11	1.24853171755464e-10	UP
LRRC74A	1.44205517524889	3.05302466334407e-07	6.22218146802555e-07	UP
LRRC75A	-1.2332083826404	4.12895564679132e-33	3.66450074809262e-32	DOWN
LRRC8E	2.53658490036998	5.11188691229239e-67	2.33630674025584e-65	UP
LRRC9	2.19939212497814	2.73711301313033e-16	9.8800474497592e-16	UP
LRRK2	-1.64395496627455	5.41705517599493e-15	1.81747941771868e-14	DOWN
LRRN1	1.15056251422047	4.57683675276092e-05	7.83719720482111e-05	UP
LRRN2	-3.51244482666139	1.19510837876762e-59	3.81143263726751e-58	DOWN
LRRN3	-1.46002044841543	1.15756978641355e-10	2.98928706670904e-10	DOWN
LRRN4	2.98631511010205	3.07054019644868e-30	2.3430024733024e-29	UP
LRRN4CL	-1.88084659600732	3.80644071079155e-34	3.54826296022555e-33	DOWN
LRRTM1	-1.18211635444858	0.00124082980485822	0.00186698085129147	DOWN
LRRTM2	-1.11175438098632	2.65799500364964e-08	5.84537345270918e-08	DOWN

GeneSymbol	log2FoldChange	pvalue	padj	change
LRRTM4	-1.59276265039427	1.47333776055255e-06	2.86171003061925e-06	DOWN
LRTM2	-1.27994636888749	4.41815172378672e-05	7.57590112912832e-05	DOWN
LSAMP	-1.14228085392549	6.2142739120448e-11	1.63112981052813e-10	DOWN
LSM7	1.30107995991294	3.70178469419909e-24	2.0840341700643e-23	UP
LSMEM2	-1.09277974947855	2.23988561580322e-07	4.60757569143314e-07	DOWN
LSP1	-1.43445275246216	1.77088634102659e-23	9.60038725289414e-23	DOWN
LTB	-1.04127317895576	7.03797924996159e-11	1.84067787142657e-10	DOWN
LTB4R	1.54643466556788	1.04867337577795e-25	6.39823037576083e-25	UP
LTB4R2	1.03708509304276	4.94816759174769e-13	1.48325774272857e-12	UP
LTBP4	-1.31174202592085	8.78504171429163e-21	4.10888883617943e-20	DOWN
LTF	-1.27009521049449	3.73369695027547e-09	8.68950347435802e-09	DOWN
LTK	-1.67888849463714	1.16783946907051e-12	3.41947009835627e-12	DOWN
LURAP1	-1.27069077950374	2.00826693522177e-17	7.76764818814232e-17	DOWN
LUZP2	-2.36921807691945	3.21432113706535e-24	1.81311868998118e-23	DOWN
LVRN	-3.41527027872099	4.10298578784192e-54	1.02504005055511e-52	DOWN
LXN	-1.35208451474419	1.60172903403261e-31	1.30943228495821e-30	DOWN
LY6D	4.34889380675961	4.67649733536136e-22	2.33329077652285e-21	UP
LY6E	1.94989199217788	9.6574016839441e-28	6.52811861500461e-27	UP
LY6G6C	3.25333577327708	1.86287528562582e-37	2.05565572551346e-36	UP
LY6G6D	8.37128144926104	1.52723762980563e-66	6.8014805237354e-65	UP
LY6G6E	5.30370643555855	4.03071526120728e-40	5.11193334620557e-39	UP
LY6G6F	5.53287246575359	2.64248879176579e-42	3.6752122468864e-41	UP
LY6G6F-LY6G6D	7.9966406083193	6.5096704680254e-61	2.18406342696968e-59	UP
LY6H	-2.14535583101083	6.94376361011924e-19	2.91283439515795e-18	DOWN
LY86	-1.3016984467418	3.06319031817888e-17	1.17384095533558e-16	DOWN
LY9	-2.17108233231339	3.23023925342416e-29	2.34172173688072e-28	DOWN
LY96	-1.17928055774751	7.76222121007867e-12	2.16365548152873e-11	DOWN
LYAR	1.33598515612692	7.13448419896706e-54	1.75470018865273e-52	UP
LYG1	1.10348361456659	1.97145542315586e-10	5.0042205952497e-10	UP
LYNX1	-2.63831049184379	2.29835675419596e-45	3.70567464451984e-44	DOWN
LYPD3	1.81766463852731	3.08412930110496e-18	1.24689908335595e-17	UP
LYPD6B	1.16491701506947	3.00205152451519e-09	7.02239699037923e-09	UP
LYPD8	-3.55664108554443	1.24721500556598e-34	1.19393924639474e-33	DOWN
LYVE1	-4.25667731488818	5.13051308938898e-81	4.27452748447513e-79	DOWN
LYZ	1.42352386257041	4.14603387433489e-08	8.99961204859057e-08	UP
LYZL4	2.93731626110619	1.1434128922451e-10	2.95360461247055e-10	UP
LZTS1	1.62586838997121	1.53735746496946e-27	1.02882419437023e-26	UP
LZTS3	2.25256899505645	2.49585725323845e-51	5.41899779933181e-50	UP
MAB21L1	-3.64896261850989	5.90332285466391e-45	9.277487442984e-44	DOWN
MAB21L2	-3.50139884683954	1.24192907904141e-55	3.32703246974585e-54	DOWN
MAB21L3	-1.1294276926227	2.16192843757411e-10	5.4717528900404e-10	DOWN
MACC1	2.22540786000732	1.65552831070721e-68	8.00769846509574e-67	UP
MACIR	-1.43849686181886	1.88894535922199e-62	6.86684875576879e-61	DOWN
MACROD1	1.25439905233869	8.90715616136633e-20	3.93256364700486e-19	UP
MAD2L1	1.37519094840148	1.31462663286032e-40	1.70578193427696e-39	UP
MAD2L2	1.29330433978751	6.03033120145738e-38	6.78772832650145e-37	UP
MADCAM1	-2.3619791825503	2.27432719280274e-31	1.83942681877725e-30	DOWN
MAF	-1.79065222863314	3.4150582279943e-38	3.9045573817508e-37	DOWN
MAFA	-2.04136483454895	5.35626405596302e-18	2.14213656422793e-17	DOWN
MAG	-1.00747777765125	0.00940067427476991	0.0128832001532007	DOWN
MAGEA1	4.79116631901528	4.08861738611184e-07	8.25737584601779e-07	UP
MAGEA10	3.33991629282685	5.82742578170286e-06	1.07858168725332e-05	UP
MAGEA11	6.56317219392201	2.92213918098989e-24	1.65366296907953e-23	UP
MAGEA12	8.99254863042642	3.54238387346608e-26	2.21963045659104e-25	UP
MAGEA3	11.4102372411176	1.2281968714919e-41	1.66562243950845e-40	UP
MAGEA4	6.19185321103472	4.76125873291127e-11	1.26057166361843e-10	UP
MAGEA6	9.78741832972892	1.13371596539248e-29	8.41133195798005e-29	UP
MAGEA9B	3.9447239403071	0.00136812854981095	0.00204983844758717	UP
MAGEB17	5.45199554755465	4.42235406644332e-36	4.56469776875978e-35	UP

GeneSymbol	log2FoldChange	pvalue	padj	change
MAGEB2	3.35289299675891	0.00015651376105683	0.000255376135802341	UP
MAGEB5	2.88730318628201	1.3878382780831e-09	3.33100316144191e-09	UP
MAGEB6	2.75623642206124	0.00958603287236561	0.0131217349584547	UP
MAGEC1	4.30208018864603	2.97938426339125e-06	5.64281250581161e-06	UP
MAGEC2	4.59364253341791	7.93975486685576e-06	1.4521053617956e-05	UP
MAGEE2	-3.50839232369514	5.08994965795395e-37	5.50163211632229e-36	DOWN
MAGEH1	-1.0461382142409	1.61699359363442e-14	5.25704059857286e-14	DOWN
MAGI2	-1.38823046927423	1.35588136041798e-27	9.09474658280363e-27	DOWN
MAGI3	-1.17468210557864	3.17824318847396e-44	4.80405804174454e-43	DOWN
MAL	-3.66871812186997	1.9739897832549e-65	8.34298157665474e-64	DOWN
MALL	-2.30612109698539	1.55427722086733e-46	2.67173042911775e-45	DOWN
MALRD1	2.95582770611771	2.96017291271021e-16	1.0658703666051e-15	UP
MAMDC2	-4.81996924797991	1.28952890184017e-72	7.5098885510846e-71	DOWN
MAN1A1	-1.40569295712434	1.96209741209416e-31	1.59356353716397e-30	DOWN
MAN1C1	-1.15046378688456	4.22225264507576e-16	1.50598290267727e-15	DOWN
MANEAL	1.31867608149085	4.46712130628469e-19	1.9004637993241e-18	UP
MAOA	-2.01710451556053	8.36531428654243e-43	1.19397719402921e-41	DOWN
MAOB	-2.43815127748604	2.61872992729676e-24	1.48534020273676e-23	DOWN
MAP1A	-1.73213813392277	5.70247510534889e-22	2.83301566360743e-21	DOWN
MAP1B	-1.49387395705929	7.01286173263174e-17	2.62049273283941e-16	DOWN
MAP1LC3C	-1.77750334912251	3.74942290434299e-17	1.42769956337906e-16	DOWN
MAP2	-1.53337859842875	2.79166432340041e-12	7.98870186743984e-12	DOWN
MAP2K6	-1.16476784368256	7.04704894420097e-24	3.89803885849338e-23	DOWN
MAP3K19	-1.21697145003196	3.28203694437379e-07	6.67719468540494e-07	DOWN
MAP4K1	-1.33425140242067	2.64205539724239e-17	1.01446770964017e-16	DOWN
MAP6	-2.24770050781964	4.06611536557396e-33	3.61241157452752e-32	DOWN
MAP6D1	1.01152650735562	9.25780582080214e-06	1.68379123415111e-05	UP
MAP7D2	2.34968983252779	1.17591013341092e-14	3.86269065328889e-14	UP
MAP9	-1.14586864184351	2.56277042756175e-07	5.25253312795818e-07	DOWN
MAPK10	-2.06053507231458	1.00685250706587e-40	1.31229960370794e-39	DOWN
MAPK15	4.31555406081271	1.09506368208413e-87	1.27975462390141e-85	UP
MAPK4	-3.84080262242433	4.89292488527847e-35	4.79462583158999e-34	DOWN
MAPT	-2.41078979698625	3.83970080733266e-24	2.15958366143681e-23	DOWN
MARCHF8	-1.4159446061857	2.7333962608602e-83	2.65902955812059e-81	DOWN
MARCHF9	1.01926970661637	2.0647149065009e-40	2.65139237956491e-39	UP
MARCKS	-1.23265809672293	1.79186819494996e-43	2.62420528836532e-42	DOWN
MARVELD3	-1.20064992090012	5.14735918746056e-53	1.20471727864584e-51	DOWN
MAS1L	-4.18172764101495	4.76547844058935e-24	2.66478086339057e-23	DOWN
MASP1	-3.402854837461	3.11980106961485e-33	2.78876262963056e-32	DOWN
MAT1A	3.16703365266821	3.17785624064934e-29	2.30470681875997e-28	UP
MATK	-1.04608409839267	6.27375791543785e-08	1.3440569215246e-07	DOWN
MATN2	-1.82211171485034	8.81833580518566e-26	5.41635560408105e-25	DOWN
MATN3	2.60187526945826	2.52592568177592e-29	1.84264532453976e-28	UP
MB	-2.13511008668711	1.5856565409482e-18	6.52434719932207e-18	DOWN
MBNL1	-1.04584702108821	8.89441261862434e-54	2.17220767080092e-52	DOWN
MBOAT4	-1.0025353368625	9.97972955180718e-11	2.59175288121728e-10	DOWN
MC1R	1.70033912956097	4.63552058286044e-23	2.44823536273427e-22	UP
MC3R	2.62224076904938	5.28797363656246e-06	9.81866975191535e-06	UP
MCEMP1	3.08247370157141	4.344689712285e-26	2.70967342263678e-25	UP
MCF2L2	-1.25665672920146	4.28323182232853e-36	4.42371979372519e-35	DOWN
MCIDAS	3.95747841523999	8.14309649182927e-86	8.6460816592819e-84	UP
MCM10	1.67606370183842	5.74777315819787e-63	2.1249676009278e-61	UP
MCM2	1.28427685091193	4.50262849234042e-32	3.79497918379108e-31	UP
MCM3	1.05058502854643	2.16686489398465e-50	4.51335148312856e-49	UP
MCM4	1.19091107628351	5.70869994276142e-40	7.15663010102986e-39	UP
MCM7	1.29439643129761	2.30865423707344e-50	4.80293861770129e-49	UP
MCM8	1.05111034110325	2.6399416934143e-23	1.41487549114876e-22	UP
MCMDC2	1.20530738424748	4.3347102206645e-12	1.22572765625903e-11	UP
MCOLN2	-1.764243616565	5.46571053796854e-19	2.30949812175798e-18	DOWN

GeneSymbol	log2FoldChange	pvalue	padj	change
MDFI	3.64093797575835	6.56166786548993e-141	3.36053889828754e-138	UP
MDFIC	-1.06516243624389	3.18400945961295e-12	9.08308596334213e-12	DOWN
MDGA1	-1.16945783079747	2.12924306461962e-10	5.39137843306986e-10	DOWN
MDP1	1.7757204956143	5.46214877400053e-06	1.01280371208254e-05	UP
MEF2C	-1.68503189690115	1.19388109776699e-36	1.26685262373044e-35	DOWN
MEGF10	-1.92241197670982	2.3780364679651e-16	8.6232296994328e-16	DOWN
MEGF11	1.67492765664776	1.20739761869393e-11	3.32612161593378e-11	UP
MEGF6	1.69002296197512	2.21959095426013e-21	1.06826250100972e-20	UP
MEI1	-1.49519549372168	6.50921562349223e-20	2.89958996295396e-19	DOWN
MEIG1	1.20992172305888	1.34587194938188e-08	3.0153973564831e-08	UP
MEIOB	-1.12965684382173	5.55534294200997e-10	1.36960479469375e-09	DOWN
MEIS1	-2.06217734606709	9.39162111907817e-37	1.00083414043151e-35	DOWN
MEIS2	-2.09599927920581	9.05819715050988e-27	5.83754207926827e-26	DOWN
MELK	1.4149467460027	6.98595799686404e-50	1.39984449481465e-48	UP
MELTF	1.82352254203785	1.86919017192098e-26	1.1861592005707e-25	UP
MEOX1	-1.4594642582365	8.99848000180645e-16	3.14639623035052e-15	DOWN
MEOX2	-1.43802052270489	2.4711669423427e-07	5.06836630942442e-07	DOWN
MEP1A	-2.33215293411643	6.97474547700383e-27	4.52500905331847e-26	DOWN
MEP1B	-5.57196160188906	1.36912078666594e-80	1.11404206814084e-78	DOWN
MESP2	-1.33226485186311	2.35307157503367e-12	6.77704851737698e-12	DOWN
MEST	1.11448306665442	4.26820972310656e-41	5.64330568780976e-40	UP
MET	1.76234155824171	8.44504536267013e-74	5.23322330605605e-72	UP
METTL1	1.42799484698419	4.77202428390955e-75	3.10056936028795e-73	UP
METTL11B	3.66570609348114	2.88025396085025e-21	1.38088827699024e-20	UP
METTL24	-3.53839015407808	7.80625737780418e-54	1.91181940534042e-52	DOWN
METTL26	1.47949794125213	9.43986756525524e-34	8.66507189846017e-33	UP
METTL27	1.20618164406391	1.14190648838786e-09	2.75707399920935e-09	UP
METTL7A	-2.52844502012783	1.16469409259813e-77	8.70421383451124e-76	DOWN
MEX3A	2.86867843273895	1.64433095353051e-70	8.59841888703508e-69	UP
MEX3D	1.3355551006975	9.71420642440414e-29	6.86778223581605e-28	UP
MFAP2	2.51300421566103	4.45554678750918e-50	9.07420306560203e-49	UP
MFAP4	-2.18079672265051	3.90356539505268e-29	2.82044747817645e-28	DOWN
MFAP5	-2.72777526060337	1.17385384166282e-27	7.90727928234994e-27	DOWN
MFSD12	1.70005942625959	5.22303167506519e-59	1.61256472620408e-57	UP
MFSD3	1.00329713739931	4.2547026034801e-13	1.2804551751538e-12	UP
MFSD4A	-2.27472880197675	6.55856651949287e-35	6.38726615234505e-34	DOWN
MFSD6L	-1.21374344921523	5.87374237092272e-11	1.54570766064496e-10	DOWN
MGAM	-1.81381096555781	6.18962692519326e-18	2.46297928812592e-17	DOWN
MGAT4A	-1.3233680515101	2.00973439589435e-42	2.80637570454759e-41	DOWN
MGAT4C	-3.86452285829722	3.06405402221629e-31	2.45986042825506e-30	DOWN
MGLL	-1.42135726926976	4.61609097926277e-46	7.6918652843926e-45	DOWN
MGP	-1.76909412266781	6.82754701134499e-17	2.55288975968543e-16	DOWN
MIA	2.46237668738299	6.14063986886521e-08	1.31634817230764e-07	UP
MICU3	-2.17011321743808	6.14060408076504e-33	5.43048953064305e-32	DOWN
MIDEAS	-1.14591629312453	1.75744156132011e-54	4.46098103604477e-53	DOWN
MIER1	-1.36106428877177	7.73648054121827e-51	1.63688135679506e-49	DOWN
MIER3	-1.99104687328573	1.2489318726928e-92	1.84302124569489e-90	DOWN
MIF	5.77998056712878	4.92486653833637e-27	3.22151393809358e-26	UP
MINAR1	1.18249282341243	1.06411642920065e-16	3.93824854020637e-16	UP
MINDY4	1.55662451924645	2.83948651188828e-29	2.06360511817657e-28	UP
MIOX	3.29937391410978	1.16107649210925e-24	6.70130094699977e-24	UP
MIPOL1	-1.46706392002671	4.77122006467458e-18	1.91387364630681e-17	DOWN
MITF	-1.32552039891052	4.74070229109952e-15	1.59732679943723e-14	DOWN
MIXL1	-2.00567590158199	1.14517007850704e-20	5.31897748120647e-20	DOWN
MKRN3	1.33277206826193	0.00399141545126314	0.00570487706253346	UP
MLIP	-1.27062923937534	7.58341585067589e-07	1.50535818750364e-06	DOWN
MLLT11	-1.22523275890016	7.95741593114656e-15	2.64785942306621e-14	DOWN
MLXIPL	3.25565095089101	1.01140275333417e-73	6.24523267510918e-72	UP
MME	2.6779412175114	9.29088319889357e-26	5.69856108285783e-25	UP

GeneSymbol	log2FoldChange	pvalue	padj	change
MMP1	4.54123017574133	5.66501753369653e-67	2.57558617008506e-65	UP
MMP10	3.43625040201422	2.92645321113553e-42	4.06366266072591e-41	UP
MMP11	4.47793454791831	1.32788883652053e-106	3.1246659878827e-104	UP
MMP12	1.72457089717081	8.51571788170158e-15	2.82823184196204e-14	UP
MMP13	5.48389635443621	6.86562706590777e-56	1.86216766508804e-54	UP
MMP14	1.15923033145168	5.07269342237311e-18	2.03152738187173e-17	UP
MMP20	3.71307628037078	3.3287677270505e-20	1.50947480289402e-19	UP
MMP27	-4.15348917863604	1.74176413920682e-23	9.44839219813345e-23	DOWN
MMP28	-2.68748731712517	7.62312011595268e-39	8.97507711826125e-38	DOWN
MMP3	4.82943467403208	5.83284648968358e-75	3.74787291235646e-73	UP
MMP7	7.06710558366155	2.344841707022e-143	1.27596027013669e-140	UP
MMP8	4.43519397002856	7.45970138145267e-30	5.59433449796592e-29	UP
MMP9	1.40700044847406	1.3724452715172e-10	3.52900022340947e-10	UP
MMRN1	-3.27823926923088	5.367322853457e-37	5.79784074734781e-36	DOWN
MMS22L	1.16600451615933	1.03271466582095e-39	1.27717759062075e-38	UP
MND1	1.71186500709781	1.85346038660674e-09	4.4060485613629e-09	UP
MOB3B	-1.54882505781905	6.80310722102118e-57	1.94200829573183e-55	DOWN
MOBP	-3.01295534951721	7.44677545094592e-54	1.82892384946857e-52	DOWN
MOCS1	-1.6431126986381	1.39437117841232e-34	1.33261170854521e-33	DOWN
MOCS3	1.04027757226725	2.22063832545755e-25	1.32697238027427e-24	UP
MOGAT1	1.81848750170109	2.03844759996559e-07	4.20861845603519e-07	UP
MOGAT2	-2.80556966036424	1.64387937179065e-34	1.5667691024078e-33	DOWN
MOK	1.01835756608057	4.57064883925002e-12	1.29076724355921e-11	UP
MORC4	1.32835449705597	2.39883882399647e-33	2.16317868680738e-32	UP
MORN3	1.43722718016593	5.05382193265697e-11	1.33518739665234e-10	UP
MORN5	-4.8964229890597	2.87762895169618e-26	1.81157458191922e-25	DOWN
MPC1	-1.25306463459924	3.87400790712153e-40	4.92394888224141e-39	DOWN
MPDZ	-1.74994865222126	1.35099582601178e-23	7.36533823367034e-23	DOWN
MPEG1	-2.13943555262165	9.72025433472429e-39	1.13825681728685e-37	DOWN
MPG	1.02026329003832	3.60581044355947e-18	1.45409859318437e-17	UP
MPIG6B	1.01456150712364	2.48679472753491e-08	5.47994894843906e-08	UP
MPP2	-2.06633279648521	4.00516361337583e-35	3.93799627327574e-34	DOWN
MPP3	1.34597860799935	5.24541950269592e-28	3.58471310048839e-27	UP
MPP4	2.19448443798697	7.35570669431141e-11	1.92171798341488e-10	UP
MPP5	-1.00735275329483	1.74532030329803e-46	2.98538923785153e-45	DOWN
MPP6	1.49527880745023	1.61482927539312e-24	9.25576766702447e-24	UP
MPP7	-1.02237543586966	5.16343980793864e-18	2.06691902012955e-17	DOWN
MPPE1	-1.02588744604343	4.00653552312336e-38	4.55093301135989e-37	DOWN
MPPED1	-2.39540756886587	1.80551116225067e-12	5.2285657522486e-12	DOWN
MPPED2	-2.12140705429707	4.40252490220436e-25	2.59604355306754e-24	DOWN
MPZ	-2.92870503822387	7.78292776080118e-83	7.34906063001605e-81	DOWN
MRC1	-1.3857755821801	1.09578633119242e-10	2.83436235666274e-10	DOWN
MRGBP	1.66817228482916	1.57426348716901e-91	2.15847638599008e-89	UP
MRGPRE	-2.24965606423133	3.13091674772833e-15	1.06357107546222e-14	DOWN
MRGPRF	-2.49795672527307	4.65866199332163e-38	5.27789728625306e-37	DOWN
MRLN	2.14430419889609	1.02675889546662e-05	1.85871219947606e-05	UP
MRM2	1.03525062473546	4.76682159010321e-08	1.03047379700145e-07	UP
MROH2B	-3.13811195636006	8.21697128922238e-10	2.0045127635084e-09	DOWN
MROH6	2.34912153504986	2.00389356311808e-45	3.24291808685643e-44	UP
MRPL12	1.07656879614616	5.57281599320339e-10	1.37371807601431e-09	UP
MRPL17	1.19202822726661	2.99454742327432e-55	7.86486489916678e-54	UP
MRPL2	1.80802652655144	6.45080411080603e-19	2.7112684523646e-18	UP
MRPL38	1.74706002922412	1.59193531189766e-27	1.06453032204585e-26	UP
MRPL4	1.06624621886621	1.03151201777793e-20	4.80516820908696e-20	UP
MRPS2	1.03602063118428	2.63310658552035e-18	1.06951912698078e-17	UP
MRPS24	2.06857286415857	9.32405544226691e-22	4.57738306783743e-21	UP
MS4A1	-2.79323253862716	1.33404091735005e-18	5.50726754239364e-18	DOWN
MS4A10	-7.40966511902833	1.19856102048055e-72	7.0035379361167e-71	DOWN
MS4A12	-5.36725085753931	1.14255805502859e-44	1.77320529520614e-43	DOWN

GeneSymbol	log2FoldChange	pvalue	padj	change
MS4A15	2.65927139597261	4.53687031615477e-16	1.61489212623064e-15	UP
MS4A2	-2.26227389949604	5.44201679104601e-25	3.19493723474323e-24	DOWN
MS4A4A	-1.64344022874633	4.01595625215185e-19	1.7127074753544e-18	DOWN
MS4A4E	-1.90771342916879	4.06309379674003e-25	2.3983271960215e-24	DOWN
MS4A6A	-1.4909963810805	3.67152896955401e-20	1.65928715148829e-19	DOWN
MS4A7	-1.51930191626161	6.22835899785086e-19	2.62157155498132e-18	DOWN
MS4A8	-2.02951878561516	7.96193794651457e-17	2.96495349577969e-16	DOWN
MSH4	1.20696239624374	2.37208933070468e-05	4.16677005099975e-05	UP
MSH5	2.03672187874814	1.76943590243272e-45	2.87686156573865e-44	UP
MSH5-SAPCD1	1.97392984461721	2.06205875155512e-28	1.43111315427777e-27	UP
MSLN	2.98735038988037	2.28545207344515e-27	1.51606007447239e-26	UP
MSLNL	5.80965664408275	8.84554211368239e-34	8.12380932624216e-33	UP
MSMB	2.69536149908204	9.61321910606038e-06	1.74642654453656e-05	UP
MSRB3	-2.60236089926155	1.30073113473918e-42	1.8339782388027e-41	DOWN
MST1	1.79187833389252	7.89585459035062e-28	5.36862616094398e-27	UP
MSTN	-2.27144957819419	9.06336563698446e-24	4.98327710252006e-23	DOWN
MSX1	3.3967928345139	3.69682414157473e-68	1.76363832266413e-66	UP
MSX2	4.64868896091398	1.11429766562343e-92	1.65839873944451e-90	UP
MT-ND5	-1.06673633022069	3.10113607548387e-13	9.40604119184821e-13	DOWN
MT-ND6	-1.3893118860863	4.49755894479894e-17	1.70363267143319e-16	DOWN
MT1E	-2.43848904758153	9.14433361429346e-27	5.89087240938557e-26	DOWN
MT1F	-2.94717455208803	5.45894858721006e-65	2.26865565033625e-63	DOWN
MT1G	-3.42031639428146	4.00760870265305e-54	1.00409338617694e-52	DOWN
MT1H	-3.91478253367626	6.26951316752459e-52	1.3978365273509e-50	DOWN
MT1M	-4.35496954255758	1.56706257094621e-58	4.71282565593893e-57	DOWN
MT1X	-1.680659936249	1.17383244922349e-23	6.42361547401904e-23	DOWN
MT2A	-2.19778687302808	5.34997866773349e-36	5.49611672809695e-35	DOWN
MT4	5.08173208590701	4.78675681537893e-10	1.18565855513789e-09	UP
MTARC2	-1.11293050966496	5.628218286814e-24	3.13312548044412e-23	DOWN
MTBP	1.65615427779943	4.4895342772522e-52	1.01002920374409e-50	UP
MTERF3	1.08938165534852	6.1548807583666e-36	6.30440815561398e-35	UP
MTFP1	1.18617737045644	1.86558435445463e-09	4.43184452443636e-09	UP
MTFR2	1.36063882000544	4.6326222924138e-44	6.94215593612749e-43	UP
MTG1	1.2779594846565	1.14363877647637e-20	5.31328228782898e-20	UP
MTG2	1.08876697511427	7.79491925428009e-33	6.85173795935282e-32	UP
MTHFD1L	2.33874291283824	5.2269877485079e-195	7.58479480539733e-192	UP
MTHFD2	1.41353153504216	2.17235881356009e-54	5.48221507543794e-53	UP
MTHFS	1.1741451350907	1.94147907752109e-10	4.93460446312579e-10	UP
MTM1	-1.43476181246149	2.78290250697965e-67	1.29223150277431e-65	DOWN
MTMR9	-1.00114694567518	5.03841496910941e-27	3.29331530995128e-26	DOWN
MTNR1A	-1.09051524150623	8.75984544362019e-06	1.59689267912226e-05	DOWN
MTRNR2L10	-1.42175139397796	1.42024184965327e-09	3.40736722623483e-09	DOWN
MTRNR2L3	1.03217923278222	0.000659525738614913	0.00101856511631942	UP
MTTP	1.83237394604663	4.1664457762085e-06	7.80194862900511e-06	UP
MTUS2	-1.50668467139105	6.31708247643173e-11	1.65761538821739e-10	DOWN
MUC12	-1.38769329312364	1.15750892693083e-07	2.43367579626256e-07	DOWN
MUC15	3.25198413524009	1.02952516794031e-06	2.02109602585622e-06	UP
MUC16	3.26148470086558	2.32451597971755e-17	8.9550435298278e-17	UP
MUC17	1.62227000642646	4.11271859040256e-06	7.70382624942768e-06	UP
MUC19	2.650693565415	1.70708285654274e-08	3.79974866176387e-08	UP
MUC21	3.43836842127716	5.15304424202825e-10	1.27330721422503e-09	UP
MUC4	-1.3215839123579	1.08767699434184e-07	2.29322187946174e-07	DOWN
MUC5AC	4.12778286492121	6.67259111098614e-27	4.33059370166238e-26	UP
MUC6	4.86765871533281	1.67989478893327e-33	1.5275199978953e-32	UP
MUC7	2.12110627812052	0.00252980037586041	0.00369015479972261	UP
MUCL1	2.93980100824934	5.46322175011154e-12	1.53536282012092e-11	UP
MUCL3	3.04939419513313	1.98376319974129e-11	5.37387501510503e-11	UP
MUSK	-4.22577541990223	4.1744137158914e-66	1.8217811036295e-64	DOWN
MXD1	-1.84239032664011	2.5556587105185e-55	6.77346805574714e-54	DOWN

GeneSymbol	log2FoldChange	pvalue	padj	change
MXI1	-1.68935552449656	2.21789972877478e-97	4.022946664287e-95	DOWN
MXRA7	-1.19918803737768	2.57051626224221e-17	9.88088293033633e-17	DOWN
MYADML2	3.36674058101018	8.11751887131097e-38	9.10176150071719e-37	UP
MYBBP1A	1.04602627447572	5.83680304231616e-34	5.39470548704094e-33	UP
MYBL2	2.15248973317437	2.74292841892409e-60	9.02884925495749e-59	UP
MYBPC2	-1.49301296538459	1.77055322022279e-11	4.81352743540038e-11	DOWN
MYBPC3	1.31780825524137	7.20397974765585e-14	2.26023242064741e-13	UP
MYBPH	1.71265818347763	4.48989647239396e-08	9.71936440499701e-08	UP
MYBPHL	5.74206397676294	5.05173310369759e-30	3.8229390932067e-29	UP
MYC	1.99064802071641	5.86313865657492e-73	3.47261338186867e-71	UP
MYCN	2.00202043149888	1.76427675621336e-21	8.53845223900593e-21	UP
MYCT1	-1.30021055612472	3.9681586902325e-22	1.98841862656168e-21	DOWN
MYEOV	3.07101901671315	7.28021239770273e-49	1.39461318461163e-47	UP
MYG1	1.48169096218404	1.64190295181618e-10	4.19337871809551e-10	UP
MYH11	-4.53946458906698	3.03730869404711e-56	8.42176055564369e-55	DOWN
MYH15	-1.84014487105295	1.32123480810978e-30	1.02206404769505e-29	DOWN
MYH2	-3.02535616167341	3.34144785604359e-11	8.9322430944561e-11	DOWN
MYH4	2.58942618733007	2.31038707315035e-16	8.39015017826217e-16	UP
MYH6	2.32568866464263	6.66443704085911e-06	1.22737009193527e-05	UP
MYH7B	1.75757782001149	4.45913073502097e-14	1.41562157682626e-13	UP
MYL2	-1.35504339838576	3.1488235957066e-06	5.95013187976549e-06	DOWN
MYL9	-3.30116131168179	1.94170800880108e-66	8.62524529521766e-65	DOWN
MYLK	-3.53093867898905	1.65926585633153e-72	9.59893566654515e-71	DOWN
MYLPF	1.41111255228182	1.81116609090236e-12	5.24406969419402e-12	UP
MYO15B	-1.12737295413875	5.18922990328362e-16	1.84295452388084e-15	DOWN
MYO18B	2.41990532103269	1.01661414866883e-13	3.16508173981232e-13	UP
MYO1A	-1.93745845496207	3.19338690975632e-39	3.84553570259937e-38	DOWN
MYO1D	-1.10831558252556	2.96284732481665e-33	2.65118501886086e-32	DOWN
MYO3A	-1.32018481601117	6.73242638533368e-05	0.000113398859206631	DOWN
MYO3B	1.54563437134385	1.00053739922486e-10	2.59802531057298e-10	UP
MYO5A	-1.09160519498156	1.35539095743803e-13	4.20178435853097e-13	DOWN
MYO7A	1.11376268684953	3.34264844328535e-14	1.06918883894935e-13	UP
MYOC	-4.85138104615711	1.63461653775302e-20	7.51811351608381e-20	DOWN
MYOCD	-3.17007142631108	6.70350721630364e-50	1.34945862609821e-48	DOWN
MYOM1	-3.40416082235495	6.40225703608431e-77	4.58775727445827e-75	DOWN
MYOM3	2.03751186497919	8.68158107875568e-25	5.05255251752582e-24	UP
MYORG	-1.24167642629013	3.2151758605634e-40	4.09552723189396e-39	DOWN
MYOT	-2.12504211540877	4.38065412387957e-13	1.31676730984145e-12	DOWN
MYOZ2	-1.57647479974957	6.07238986648846e-12	1.70216556254288e-11	DOWN
MYOZ3	-1.04624789621193	1.51540436414507e-07	3.15793874974368e-07	DOWN
MYPN	-2.62168147234359	1.65186468199548e-27	1.10375747151141e-26	DOWN
MYRFL	1.15664629785239	0.000202867673548577	0.000327662999675482	UP
MYT1	1.35039264169436	6.86336892122689e-07	1.36569355531167e-06	UP
MYT1L	-2.86619336737374	5.4609944103792e-15	1.83151571009116e-14	DOWN
MYZAP	-1.25359537970519	5.02005064040855e-26	3.12304901041208e-25	DOWN
MZB1	-1.97634994224842	2.57125869748301e-17	9.88155544013941e-17	DOWN
MZT1	1.25116230306049	3.23462428148779e-26	2.03117607694002e-25	UP
MZT2A	1.06333229872544	4.2475490992353e-21	2.01587823562236e-20	UP
NAA10	1.74833304815139	1.8510603983743e-35	1.85350860936697e-34	UP
NAA11	1.53483758012433	0.00653842704800443	0.00912069455955308	UP
NAAA	-2.10068138626953	5.55477796787265e-89	6.8599538123806e-87	DOWN
NAALAD2	-1.49822245829851	3.46470225286463e-25	2.05067506217307e-24	DOWN
NAALADL1	-3.23433206937083	4.56250092586073e-77	3.28292680256251e-75	DOWN
NACA2	2.76862604324882	7.84547820156291e-26	4.83586944863062e-25	UP
NACAD	-2.12782763269627	7.5601318021936e-39	8.90694012663038e-38	DOWN
NAIP	-1.11446783655955	1.05117657184485e-10	2.72383000677596e-10	DOWN
NANOS3	3.89311598596968	1.20755813307859e-48	2.28308466572178e-47	UP
NANP	1.22198585753512	1.24248107688794e-39	1.5289980912968e-38	UP
NAP1L2	-3.77774200069831	5.37787752320789e-61	1.81482521921742e-59	DOWN

GeneSymbol	log2FoldChange	pvalue	padj	change
NAP1L3	-2.12885527142535	2.08429390934152e-28	1.44596851965593e-27	DOWN
NAP1L5	-1.11003846252095	2.83956283730634e-24	1.60797748572408e-23	DOWN
NAT1	-1.47182513392544	1.48377586731857e-31	1.21643075224191e-30	DOWN
NAT10	1.07695527527193	1.20330327351402e-66	5.38640614439578e-65	UP
NAT16	3.45723989462546	9.73442291492508e-22	4.77345835588821e-21	UP
NAT2	-1.73919162924024	2.21882555629423e-20	1.01434521952616e-19	DOWN
NAT9	1.16626363488211	4.18480617943273e-51	8.97414162591898e-50	UP
NBEA	-2.52876386640041	2.13472545558316e-29	1.56316124298022e-28	DOWN
NBEAL1	-1.68541489671632	1.39772739681381e-30	1.07979712336819e-29	DOWN
NBEAL2	1.34480611878087	7.157317064366e-35	6.958702514897e-34	UP
NBPF1	-1.61912905371245	4.58912695163271e-75	3.00415291762332e-73	DOWN
NBPF6	1.18869371583281	0.00188168076076676	0.00278241398498909	UP
NCAM1	-3.05926509756688	1.05056941223646e-58	3.17596617626276e-57	DOWN
NCAM2	-1.43912359938525	8.58042284119755e-11	2.23301304638728e-10	DOWN
NCAN	-2.21646782520663	1.757596117038e-17	6.81778150723605e-17	DOWN
NCAPD3	1.0242380423223	1.95504771495989e-45	3.17567591983177e-44	UP
NCAPG	1.18890977265451	4.54080011709758e-34	4.2102743577753e-33	UP
NCAPG2	1.26180511698698	6.83180268600784e-62	2.4277995953358e-60	UP
NCAPH	1.18444482924422	6.75618404243056e-50	1.35849229481343e-48	UP
NCF1	-1.4911015147938	9.17157074920899e-17	3.40521452997817e-16	DOWN
NCKAP1L	-1.31850793731656	3.25482101228986e-17	1.2445366334432e-16	DOWN
NCOA4	-1.05808210048155	2.76734408481353e-55	7.31225531849134e-54	DOWN
NCR2	-2.82362339201409	5.09961273422174e-19	2.16162503751225e-18	DOWN
NCR3	-1.33516679957537	3.33793053027398e-12	9.50349645579803e-12	DOWN
NCS1	-1.3259826878979	3.69488811074301e-24	2.0808242778903e-23	DOWN
NDN	-1.80590493219005	9.56271840283812e-35	9.23035562908094e-34	DOWN
NDNF	-1.20143823685822	9.8008515602043e-10	2.37591853289486e-09	DOWN
NDP	1.95210277482934	1.00112509448842e-11	2.77147714949553e-11	UP
NDRG1	-1.19024994522523	8.93444284735847e-21	4.17764375137092e-20	DOWN
NDRG2	-1.40641104384293	3.00106553723308e-43	4.34755026620962e-42	DOWN
NDRG4	-1.21745698141459	5.98732392931366e-19	2.52377805812488e-18	DOWN
NDST4	-1.12040437451789	0.0236983427149452	0.0309525383809887	DOWN
NDUFA11	1.43861775677985	7.42400403152488e-16	2.60738568376246e-15	UP
NDUFA13	2.43298083138356	1.59410500716417e-30	1.22823674733406e-29	UP
NDUFA4L2	2.28223212982248	8.6079556356467e-26	5.29460725833684e-25	UP
NDUFAF8	1.07092859998586	2.05194127607579e-21	9.90311902447555e-21	UP
NDUFS7	1.10214159249301	6.31315153044696e-08	1.35216368511283e-07	UP
NEB	2.56570054828648	2.60340659843772e-27	1.72434838716607e-26	UP
NEBL	2.2379180858456	4.12502300160993e-102	8.55107446750402e-100	UP
NECAB1	-3.18294028385028	1.57674050798613e-48	2.96819269897973e-47	DOWN
NECAB2	-1.54843239579797	4.44234943301656e-10	1.10317499539529e-09	DOWN
NECTIN3	-1.09536776212262	4.19977254589931e-16	1.49827165215621e-15	DOWN
NECTIN4	1.99098397502406	1.20934284486302e-41	1.64389437608116e-40	UP
NEDD4L	-1.31889511928916	6.34049117223896e-56	1.72241767210916e-54	DOWN
NEDD9	-1.05271969758506	1.51857781986007e-19	6.62399688808202e-19	DOWN
NEFH	-1.82049884798128	3.15511246490456e-24	1.78145179479193e-23	DOWN
NEFL	-2.60929615967739	1.75503848177795e-12	5.0866320045272e-12	DOWN
NEFM	-3.72052909227269	1.08956519182455e-44	1.69398202546794e-43	DOWN
NEGR1	-3.21458932533	1.42724639784074e-74	9.03732419112756e-73	DOWN
NEIL3	1.23629590336399	2.54016340418163e-32	2.17142196156185e-31	UP
NEK2	1.38666143475041	2.14682484376264e-52	4.90586102420458e-51	UP
NEK5	2.062371051307	1.92687209493616e-34	1.82748495583461e-33	UP
NELFCD	1.46040271601592	5.98191603167682e-57	1.71039579408191e-55	UP
NEO1	-1.1047898042136	1.99773712322256e-27	1.32823965355763e-26	DOWN
NEU4	-2.08587409164596	1.81097869239299e-22	9.2612546169278e-22	DOWN
NEURL1	-1.27106840468994	1.49732819734466e-07	3.12326017014404e-07	DOWN
NEURL1B	-1.41929541284674	9.88617014787621e-40	1.22525182053358e-38	DOWN
NEURL2	1.15720526973798	5.85017882892931e-14	1.84712899271344e-13	UP
NEUROD1	-2.4268408865215	2.82064589688128e-06	5.35264897584935e-06	DOWN

GeneSymbol	log2FoldChange	pvalue	padj	change
NEUROG2	5.91754425615893	3.16129908055503e-27	2.08704939047635e-26	UP
NEXMIF	-3.77419490607917	4.098624477789e-41	5.43559390950037e-40	DOWN
NEXN	-2.27338776326154	4.07433817225754e-29	2.93531032658339e-28	DOWN
NFASC	-1.39363719281017	6.91294474713014e-18	2.74390487535394e-17	DOWN
NFE2	1.80780152206644	4.01553894328975e-12	1.13750739579477e-11	UP
NFE2L3	2.71436318471874	1.05109716360242e-182	1.40790422383146e-179	UP
NGB	-5.09657217014246	1.42576906148777e-33	1.29915838135461e-32	DOWN
NGF	-1.11480808135941	3.04093252473945e-08	6.66227454117867e-08	DOWN
NGFR	-1.82579635357275	5.9378549950274e-14	1.87311356935529e-13	DOWN
NHEJ1	-1.1833647455072	6.96927065889275e-50	1.3997221451361e-48	DOWN
NHLH2	1.54757797206772	0.00637744739488245	0.00890898447549844	UP
NHSL1	-1.06768669398613	1.42294869988792e-31	1.16821337629177e-30	DOWN
NHSL2	-1.61803001946566	5.44348188350893e-21	2.56528687517026e-20	DOWN
NIBAN1	-3.36771373946121	5.60274232478958e-78	4.2234005238771e-76	DOWN
NIBAN3	-2.15954458509914	4.00923271999504e-16	1.43117608350294e-15	DOWN
NIFK	1.00097289111651	6.36674900925285e-52	1.41770077363325e-50	UP
NIM1K	-1.47378428027039	4.31618887091345e-33	3.82872118233397e-32	DOWN
NIPAL1	-1.38567809265559	2.28118757911414e-38	2.6271375208409e-37	DOWN
NIPAL4	-1.46885066658179	3.00115329580384e-10	7.52145687101787e-10	DOWN
NKAIN3	-2.22462581711665	3.1641286185556e-24	1.78596342414615e-23	DOWN
NKAPL	-2.20564261077751	4.03107285045717e-29	2.90534236527362e-28	DOWN
NKD1	3.14996651010493	1.60906314683789e-38	1.8666633295062e-37	UP
NKD2	4.41037439776084	4.71801031197516e-104	1.02693391953029e-101	UP
NKPD1	3.27915723594537	6.63518075609472e-55	1.71932146586127e-53	UP
NKRF	1.42840367936453	7.12224146633705e-85	7.295270038431e-83	UP
NKX2-1	5.78621331356876	1.30888428134421e-15	4.54015975917267e-15	UP
NKX2-2	-1.6815805566577	1.83588559003094e-05	3.25675181124783e-05	DOWN
NKX2-3	-2.82689690081669	1.92716114562475e-72	1.11118069631668e-70	DOWN
NKX2-5	2.93787631125937	0.000614701236357801	0.000951955943498612	UP
NKX2-8	3.77777254930693	2.51658824920523e-07	5.15848748480409e-07	UP
NKX3-2	-1.9312101260132	1.33531112933026e-23	7.28438994205134e-23	DOWN
NKX6-1	2.74849480149352	2.51264772423423e-06	4.78485726400816e-06	UP
NKX6-3	2.47868110805857	2.41310783078178e-05	4.23555944234845e-05	UP
NLE1	1.31557872055259	1.25563964813741e-51	2.74334418983898e-50	UP
NLGN1	-3.87843404011352	1.50006558402725e-33	1.36471483880181e-32	DOWN
NLGN3	-1.44746294704727	4.67178930567737e-24	2.61491054901189e-23	DOWN
NLGN4X	-2.22021202516099	2.62552735751819e-32	2.24109352335609e-31	DOWN
NLGN4Y	-1.04910223992611	0.0205096482228908	0.0269800184713453	DOWN
NLRP4	-1.42486550260922	0.00496229286034451	0.00701673735615616	DOWN
NLRP6	1.16604244980837	3.11833076727769e-05	5.42560887795828e-05	UP
NLRP7	-2.1182349973294	1.28180192770689e-17	5.00898046839321e-17	DOWN
NMB	1.91593707701749	7.85397983739285e-57	2.2310171436953e-55	UP
NME1	2.18315318179958	4.13439367210627e-60	1.34816848337803e-58	UP
NME2	3.79870288588756	3.08748726515854e-52	7.0003145505476e-51	UP
NMRK2	-2.48294565723919	4.33008597156072e-07	8.73289170984328e-07	DOWN
NMU	2.85696577052971	9.83441386361615e-31	7.67922191063444e-30	UP
NMUR2	3.59606133895862	1.36570256495401e-13	4.23224395151168e-13	UP
NNAT	-2.46804111250633	2.69421568965273e-33	2.41702100999088e-32	DOWN
NOB1	1.31617823406827	1.91124337468295e-62	6.93343351736548e-61	UP
NOC3L	1.05039860217884	5.71106359334809e-35	5.58689608713317e-34	UP
NOC4L	1.07123336093339	3.07824874500956e-30	2.34682773191119e-29	UP
NOD2	1.46224975592426	5.54623660294441e-22	2.75696882577993e-21	UP
NODAL	1.47703344450702	3.65627921790325e-09	8.51274101101073e-09	UP
NOL4	-1.05344898087934	0.00423924164965459	0.00604222925803678	DOWN
NOL6	1.10555709697426	7.51602722834631e-52	1.66509646472257e-50	UP
NOLC1	1.02609299219297	8.51330028482772e-49	1.62546159933887e-47	UP
NOP16	1.29658593550189	4.03916924678352e-54	1.01054675422761e-52	UP
NOP2	1.61306422811079	5.05460560906769e-27	3.30265844167714e-26	UP
NOP56	1.38095000588192	6.52423595434526e-58	1.9320836849152e-56	UP

GeneSymbol	log2FoldChange	pvalue	padj	change
NOP58	1.11046424857153	1.09098903774404e-49	2.17113052734137e-48	UP
NOS1	-3.42345044102884	1.2388123340168e-24	7.13813341238732e-24	DOWN
NOTCH2NLC	-1.10129515117823	1.15390401355945e-09	2.78565514877452e-09	DOWN
NOTCH3	1.36208285267111	1.27764497047761e-20	5.91849743839496e-20	UP
NOTUM	8.85359483242493	3.43798185947308e-130	1.36058132088647e-127	UP
NOVA1	-3.21458494020926	6.26994674270236e-41	8.23989302873028e-40	DOWN
NOX4	2.78007959617256	2.18665398918577e-40	2.80384432354137e-39	UP
NPAS3	-1.91421090293776	4.46406442226755e-20	2.00756078990044e-19	DOWN
NPAS4	-2.9002973602005	8.40363689988625e-36	8.56246514556578e-35	DOWN
NPBWR1	1.50565220622686	8.8622868192901e-06	1.61539830822044e-05	UP
NPC1L1	3.9616679507211	2.89063988199566e-32	2.46376467279444e-31	UP
NPEPL1	1.71226753922845	3.28397436283011e-37	3.5762254896786e-36	UP
NPFFR1	3.3276282109006	4.69851443306911e-65	1.95730219672326e-63	UP
NPIPA3	1.30779006189528	0.0083636808523508	0.0115109685964262	UP
NPIPA5	1.01180198603603	9.24266277725356e-07	1.82185291985868e-06	UP
NPIPB11	1.03851518895602	1.05924213415696e-08	2.39043329213e-08	UP
NPIPB13	2.41629270836276	5.8436509860909e-17	2.19631976302182e-16	UP
NPIPB15	1.10741077105513	8.72441176556598e-05	0.000145668982715313	UP
NPIPB4	1.20074454881365	4.55285400239641e-15	1.53551901498603e-14	UP
NPIPB5	1.99121093979186	6.6149072683847e-22	3.27510322048288e-21	UP
NPM2	1.6502070176367	9.5295723865582e-22	4.6756394468058e-21	UP
NPM3	1.51901188759415	3.73373524797872e-38	4.25494318540926e-37	UP
NPPC	-1.73720320176596	3.00389090278229e-08	6.5844350818414e-08	DOWN
NPR1	-1.75323758915028	6.54918031915163e-31	5.15788678866518e-30	DOWN
NPR3	-1.26188569992502	7.13145431161594e-06	1.31019217058629e-05	DOWN
NPRL3	1.12964214351543	1.6947739704874e-34	1.61351006823932e-33	UP
NPSR1	5.64058728312781	9.70474449173276e-33	8.49616469756373e-32	UP
NPTX1	-5.10570687125978	1.13383576658903e-63	4.35838459240945e-62	DOWN
NPTX2	2.65733186745575	1.87030497575804e-15	6.42866572105701e-15	UP
NPW	3.00686499617192	2.54519479660882e-26	1.6051965589768e-25	UP
NPY	-2.94039551675471	3.94536516723737e-17	1.49870514086179e-16	DOWN
NPY1R	-2.03703990925003	3.16761940030878e-13	9.60267350584555e-13	DOWN
NPY2R	-4.64942079613664	2.98404419269404e-28	2.05705310876411e-27	DOWN
NPY4R	-1.47254095315948	4.95381558008378e-11	1.30995885643126e-10	DOWN
NPY4R2	-1.83236374696856	1.10681636271634e-15	3.85382789921609e-15	DOWN
NPY5R	-1.78686467000388	2.51120746522376e-06	4.78263760165606e-06	DOWN
NQO1	1.07108600514165	3.7941630289017e-14	1.21069746788098e-13	UP
NR0B2	2.52448608050475	2.64193258025219e-26	1.6643984088253e-25	UP
NR1H4	-3.88003198877348	2.48550466991575e-31	2.00370800079828e-30	DOWN
NR2C2AP	1.2150173414158	2.57926492652183e-56	7.16311645383167e-55	UP
NR3C1	-1.95206894509728	3.19347827858884e-40	4.07086656406058e-39	DOWN
NR3C2	-2.79071729609334	5.19972392459645e-72	2.93018746598699e-70	DOWN
NR4A3	-1.87935793201965	2.87963860769061e-18	1.16557757033279e-17	DOWN
NR5A1	2.59424834780761	2.7815796873799e-08	6.10944085473589e-08	UP
NR5A2	-2.31206235983122	6.13279490061957e-62	2.18385189375232e-60	DOWN
NRAP	-2.20369663630599	1.97875239141899e-12	5.71694298851484e-12	DOWN
NRCAM	1.19815038075632	1.6289640392898e-06	3.15238395378454e-06	UP
NRG1	-1.4719648195327	3.05033898974242e-12	8.70889536454906e-12	DOWN
NRG2	-2.87358328713455	4.65355018372391e-36	4.7976476820121e-35	DOWN
NRG3	-2.1894919335768	4.45898095985435e-20	2.00579270095437e-19	DOWN
NRG4	-1.61260019879801	1.85893069849293e-15	6.39083124439436e-15	DOWN
NRK	-1.08345550652909	0.000256441887434551	0.000410802445804768	DOWN
NRP2	-1.14883628702047	1.4798556166281e-11	4.04596103820773e-11	DOWN
NRSN1	-3.56329604541281	3.42909041605422e-25	2.0302873653435e-24	DOWN
NRXN1	-5.01878439995617	2.16056349943735e-49	4.24147601078946e-48	DOWN
NRXN2	-1.08245622967918	2.39424452225315e-06	4.56587228846721e-06	DOWN
NSDHL	1.04135907430367	1.70908979224142e-28	1.19184543661594e-27	UP
NSG1	-1.12817239906953	7.5615324306655e-10	1.84876388957004e-09	DOWN
NSG2	-4.09029065931228	2.23408836747869e-44	3.41507936792105e-43	DOWN

GeneSymbol	log2FoldChange	pvalue	padj	change
NSMF	1.16677466439562	6.56978221236879e-20	2.92507332303702e-19	UP
NSUN5	1.05214398138435	7.96538052392006e-40	9.92139993297711e-39	UP
NT5DC2	1.74076851803566	4.19300967541466e-40	5.31002745294513e-39	UP
NT5DC4	2.27831198288986	1.76560542757305e-17	6.8473245680021e-17	UP
NTF4	2.00933201315116	9.35168863447866e-05	0.000155620177935949	UP
NTHL1	1.19006909060101	6.76493524646856e-25	3.95020298620534e-24	UP
NTMT1	1.33695797941169	1.51800113217397e-65	6.47866512611404e-64	UP
NTN1	-2.80057271885995	3.52756180498487e-59	1.10279055135012e-57	DOWN
NTN4	-1.04468237558297	3.93187020836514e-17	1.49423081489005e-16	DOWN
NTNG1	-2.72759804588196	1.96272770177354e-13	6.03087656096394e-13	DOWN
NTRK3	-2.20433813296151	8.27329908219791e-16	2.89865104463405e-15	DOWN
NTSR1	-1.42794340491377	3.10484360677098e-05	5.40376229132464e-05	DOWN
NUDCD1	1.34742704084596	2.44288530411233e-45	3.93141975975121e-44	UP
NUDT1	1.15608049604999	1.06055996427773e-30	8.25918186850097e-30	UP
NUDT10	-2.0078662350558	1.82409198821975e-17	7.06942216578467e-17	DOWN
NUDT11	-1.52498519044926	1.2589560600345e-15	4.36871300784791e-15	DOWN
NUDT12	-1.01784582051678	1.56765404835676e-11	4.27593357519365e-11	DOWN
NUDT3	1.95168527200543	3.68251998813472e-20	1.66382253641385e-19	UP
NUF2	1.61500408542777	1.96299113828942e-47	3.50220949703214e-46	UP
NUFIP1	1.34487417438348	1.36551303796593e-47	2.44626322326139e-46	UP
NUGGC	-2.6093890079549	1.11738832698828e-32	9.74315620322828e-32	DOWN
NUP210	1.32718831749287	4.48238507405756e-25	2.64172409047511e-24	UP
NUP62CL	1.48170978226018	2.71760313473477e-37	2.97059782706444e-36	UP
NUPR2	-2.38966106129592	9.61605413917734e-08	2.03085931747113e-07	DOWN
NUTF2	1.0471992334083	8.51055752657574e-36	8.66633556785166e-35	UP
NUTM2A	-1.41480267420474	7.7816332826862e-22	3.83857168134319e-21	DOWN
NUTM2B	-1.43962450636769	1.38289838064641e-16	5.09314921789254e-16	DOWN
NUTM2D	-1.30826959258916	6.51491526037936e-28	4.44008686610512e-27	DOWN
NUTM2F	1.55230031439312	3.09098713873992e-05	5.38125965275727e-05	UP
NWD2	-2.62429941010892	3.80885770121596e-25	2.25054764680263e-24	DOWN
NXF3	2.40975385642014	4.76297902474244e-10	1.17993674431413e-09	UP
NXN	-1.04942197122186	9.78439416495954e-10	2.37225919791758e-09	DOWN
NXPE1	-2.3318663822934	3.97888542356976e-19	1.69749533368699e-18	DOWN
NXPE2	-1.59195775241317	7.36510595370091e-11	1.92363266794351e-10	DOWN
NXPE3	-1.05209978843614	1.04930655566729e-20	4.88675449420553e-20	DOWN
NXPE4	-2.88323978948365	3.06099193514933e-23	1.63399915900537e-22	DOWN
NXPH1	4.36360292860869	1.88568278681777e-23	1.01878356707595e-22	UP
NXPH2	-2.65105704083569	2.14262315014263e-09	5.06578369496723e-09	DOWN
NXPH3	-1.69156768406326	8.36350072192121e-13	2.46920376518844e-12	DOWN
NXPH4	5.30779204399033	3.54275819027085e-56	9.74566324916055e-55	UP
NXT1	1.42591279792694	3.39889198625636e-37	3.69443858655943e-36	UP
NYAP1	-1.10899052467284	1.15949931502124e-08	2.60910779587492e-08	DOWN
OASL	-1.40121317782367	3.23671339061116e-16	1.16327946895175e-15	DOWN
OBP2A	2.70091560210385	1.3493467758474e-10	3.47114424698343e-10	UP
OBP2B	5.87598181885336	1.04137433935846e-30	8.11703284299416e-30	UP
OBSCN	1.02509449172861	4.07736555588194e-09	9.47413483114121e-09	UP
OCA2	1.7182697212039	2.01165572292668e-08	4.45853195896558e-08	UP
OCEL1	-1.0663838408394	2.85928403637625e-31	2.29970960394548e-30	DOWN
ODAM	2.2040539401196	4.28899739399449e-14	1.36260375153487e-13	UP
ODC1	1.01105428251327	8.3677638392162e-28	5.68061878098525e-27	UP
ODF3L2	1.31346902731836	3.41123421008115e-09	7.96030840259221e-09	UP
OGN	-4.25621097064844	5.31880076231413e-40	6.67264248373025e-39	DOWN
OLAH	1.58188630715729	1.96996675282557e-07	4.07399418847406e-07	UP
OLFM1	-1.78040424270326	3.97039867715622e-21	1.88742976154303e-20	DOWN
OLFM3	-3.4868879416095	7.32989564799881e-21	3.44246706671861e-20	DOWN
OLFM4	1.16951085012605	0.000462227219574937	0.000724135184386719	UP
OLFML1	-1.41601086090311	1.21508515362681e-22	6.27655822607641e-22	DOWN
OLFML2A	-1.50702078122523	3.25552841438939e-32	2.76664305904161e-31	DOWN
OLFML2B	1.0565849791646	6.9000665894718e-10	1.69012321736492e-09	UP

GeneSymbol	log2FoldChange	pvalue	padj	change
OLFML3	-1.14169649434766	7.23970408406903e-12	2.02254078639329e-11	DOWN
OLR1	3.470924168259	1.12103511182503e-46	1.9404159445536e-45	UP
OMD	-2.74385056006009	2.34322657035869e-16	8.50763224971973e-16	DOWN
OMG	-1.30708734626464	2.91024211069154e-10	7.30202390107662e-10	DOWN
ONECUT1	1.58911585318652	7.57429214516824e-05	0.000127099498047427	UP
ONECUT2	2.56324501217802	4.57174758276408e-31	3.62843394068692e-30	UP
ONECUT3	6.46622161373655	6.24532202606614e-35	6.09925925069488e-34	UP
OOEP	1.11936906421909	0.0230873295869227	0.0302088721142986	UP
OPN3	-1.58966365563853	2.53605057239856e-41	3.38392709710162e-40	DOWN
OPN4	-1.53453713559269	5.06099676662486e-09	1.16957049365944e-08	DOWN
OPRD1	1.67192963107941	5.16706627906686e-08	1.11340335499803e-07	UP
OPRM1	-3.33382558397264	2.14604303520655e-28	1.48762131258167e-27	DOWN
OPTC	-1.19117015969001	0.00162799716664316	0.00241962398965153	DOWN
OR10A2	1.16035509535359	0.000317167304068676	0.000503954217150092	UP
OR10G4	-1.75920000552011	0.0043519413229516	0.00619677440972739	DOWN
OR10H1	2.83291647411516	1.02772486634995e-10	2.66544133121116e-10	UP
OR10Q1	1.13106848606409	0.00824249266487367	0.0113585410551951	UP
OR13J1	1.0226866274393	0.00844330048252825	0.0116187127629417	UP
OR2AG1	1.44018294126325	0.000915448048734583	0.00139463664677299	UP
OR2B6	1.27576941929091	1.30969888213478e-05	2.35280992825884e-05	UP
OR2C1	-1.25110416947524	1.42569545103327e-07	2.97991056161832e-07	DOWN
OR2I1P	1.4970897341711	5.15777782743764e-10	1.27429604581685e-09	UP
OR2T10	2.75104277232257	0.00139394711241981	0.00208690577496055	UP
OR2W3	-1.92267322208245	1.67014731678834e-09	3.98496509005693e-09	DOWN
OR4C6	1.66923278734481	0.00298879953351554	0.00432582214920672	UP
OR51B5	2.08319618914009	0.000387693205130985	0.000611051935277502	UP
OR51E1	1.39637355895761	1.44727698568417e-11	3.95999908103685e-11	UP
OR51E2	-1.87123042060392	1.89246850534205e-19	8.21989375992044e-19	DOWN
OR52K1	2.76991032934207	3.06015890964903e-07	6.23599146796005e-07	UP
OR5M11	2.34756672200988	3.02058898615767e-08	6.620203400373e-08	UP
OR6A2	1.96979518610454	2.84838724256624e-06	5.40352620708202e-06	UP
OR6T1	-1.21639796859712	0.00597792923042348	0.00837169709581503	DOWN
ORC6	1.96612034825819	3.52305358745257e-96	6.07395367508035e-94	UP
ORM1	4.21330932166907	3.77814079390938e-20	1.7056978388474e-19	UP
ORM2	3.17822350393217	8.3508305910259e-24	4.60459192785098e-23	UP
OSBPL1A	-1.66248202346576	2.74036103149668e-35	2.71587402626361e-34	DOWN
OSBPL3	1.77512064104528	5.8949766806705e-92	8.27816362423512e-90	UP
OSM	2.20470772288907	7.6051227077583e-21	3.56852604985705e-20	UP
OSR1	-2.34229265864915	1.66981304824421e-21	8.09478134996558e-21	DOWN
OTC	-1.29990899644388	7.44025586286749e-05	0.000124970748857058	DOWN
OTOA	-1.4336759443372	5.42632229908172e-18	2.16915863622383e-17	DOWN
OTOG	1.94726094010561	5.8937224165967e-08	1.26528650524224e-07	UP
OTOP2	-8.08593861009138	1.43575682740461e-65	6.14271096697701e-64	DOWN
OTOP3	-6.08204272156581	1.96173294618728e-25	1.17791916523997e-24	DOWN
OTUB2	1.93420487213427	2.05438233229158e-46	3.49345308126888e-45	UP
OTUD6B	1.00331411626102	7.11930498949354e-19	2.98431530529733e-18	UP
OTUD7A	-1.61196041497652	3.00992398182869e-27	1.98981800666602e-26	DOWN
OTX1	5.64576993711022	1.32307442379051e-131	5.48540355749144e-129	UP
OVGP1	1.62366824021788	1.2848753588822e-39	1.58005188024123e-38	UP
OVOL3	1.12455359638019	1.40625346780074e-06	2.73629362328911e-06	UP
OXGR1	1.29384150415293	9.33056396149501e-08	1.97128258021733e-07	UP
OXT	1.42424379915045	3.82713772606541e-06	7.18821585848097e-06	UP
OXTR	2.74356074286362	2.41771195400092e-64	9.58989026310206e-63	UP
P2RX1	-2.19444214507904	1.46100330435962e-39	1.78529477465361e-38	DOWN
P2RX2	-3.88770646726901	1.40600343213687e-36	1.48831232606683e-35	DOWN
P2RX4	-1.28245699489342	1.37970312143606e-39	1.68950565777539e-38	DOWN
P2RX7	-1.16772584804606	2.72230321496191e-10	6.8442774880352e-10	DOWN
P2RY1	-2.61883570032488	8.83892209578974e-60	2.83970757295179e-58	DOWN
P2RY10	-1.59081753469208	1.30872942115081e-18	5.40533809546943e-18	DOWN

GeneSymbol	log2FoldChange	pvalue	padj	change
P2RY12	-2.96194191033122	1.30259836710845e-39	1.598459856692e-38	DOWN
P2RY13	-1.47053489550644	7.55672356929383e-15	2.52078979908263e-14	DOWN
P2RY14	-2.7651260681271	1.87619529828358e-59	5.94003431436582e-58	DOWN
P2RY2	-1.07016584333001	1.9867591331706e-15	6.81683483466002e-15	DOWN
P2RY4	-3.57237072820917	3.27357152942537e-39	3.9393711846499e-38	DOWN
P2RY8	-1.71613946157622	2.75415209336494e-30	2.10619457188247e-29	DOWN
P3H1	1.20554592160047	6.33925359840169e-38	7.13084127318918e-37	UP
P3H2	-1.8589798744758	1.51115417890695e-23	8.21790372183221e-23	DOWN
P4HA3	1.75099354924227	5.66384939955496e-20	2.53598893274494e-19	UP
PABPC1	1.00860383179808	1.57012824872943e-34	1.49893877166259e-33	UP
PABPC1L	3.13450456528769	7.88050284693024e-75	5.02649069866653e-73	UP
PABPC3	1.97630678455667	7.10470507845016e-24	3.9286830590998e-23	UP
PABPC5	-1.81429232121578	8.64216657944244e-25	5.03129544125146e-24	DOWN
PACC1	1.53029744653508	2.21539951983553e-98	4.19312519988001e-96	UP
PACSIN1	-1.74283290017231	5.47262488787684e-21	2.57832297544912e-20	DOWN
PACSIN2	-1.16636334327518	1.74248050707875e-76	1.21764661233189e-74	DOWN
PADI1	2.07229285798903	1.31199199863997e-17	5.12350676661086e-17	UP
PADI2	-3.27681401195963	7.73496464432467e-55	1.99243993123706e-53	DOWN
PADI3	4.55548948821592	9.0602065730011e-33	7.93987806017454e-32	UP
PAEP	5.08185565700742	1.90284709021942e-35	1.90426875758567e-34	UP
PAFAH1B3	1.86531966985494	5.46104305630477e-53	1.27641802334812e-51	UP
PAFAH2	-1.1962014485282	3.82832434712736e-51	8.25032324957038e-50	DOWN
PAG1	-1.84882505093085	3.94911900567766e-53	9.28016319107492e-52	DOWN
PAGE1	3.07207848944056	0.000687683093441425	0.00106101592292181	UP
PAGE2	1.40661060514117	0.00878405065604968	0.0120713972120427	UP
PAGE2B	1.65851510555077	5.86830905298105e-05	9.93726203827279e-05	UP
PAH	5.55500508861044	8.92184064613922e-57	2.5261140027841e-55	UP
PAICS	1.21227757416062	1.28314257830658e-53	3.10756073936752e-52	UP
PAK3	-2.05322031481126	1.95244731941168e-25	1.17315269747811e-24	DOWN
PAK6	1.15662537664264	3.41495644598743e-21	1.63050826964571e-20	UP
PALD1	1.94085456761073	2.65621992198601e-43	3.86405659996178e-42	UP
PALLD	-1.70621029117709	9.82069337880635e-40	1.21887194444159e-38	DOWN
PALM	-1.88972752761532	3.23442566721653e-25	1.91959966405049e-24	DOWN
PALM3	2.93002388829149	8.60959344443024e-31	6.7500608126008e-30	UP
PALMD	-1.02979313979999	8.09000769781087e-09	1.84362392411963e-08	DOWN
PAM16	1.22975753587959	1.01950511227679e-25	6.22681252896377e-25	UP
PAMR1	-1.59983770377885	8.5707324670875e-27	5.53363605670725e-26	DOWN
PANK3	-1.083511399246	7.44343919098789e-30	5.58675028589104e-29	DOWN
PANX2	1.78582514390958	2.82372294744504e-13	8.59005724735507e-13	UP
PAPPA2	-2.47704061442226	2.15147093822177e-18	8.79219982334093e-18	DOWN
PAPSS2	-2.01000971470917	3.32682241593601e-53	7.83896599847006e-52	DOWN
PAQR4	2.12806378018233	2.1989065660979e-72	1.2636818493552e-70	UP
PAQR5	-1.83085613533532	4.27589262338424e-39	5.1067296468443e-38	DOWN
PAQR8	-1.45334404319282	6.78280149940642e-33	5.98626064415428e-32	DOWN
PARM1	-1.58988735631459	2.128666698843e-33	1.92353260129492e-32	DOWN
PARPBP	1.29727109928559	7.98812157605918e-30	5.98010150489761e-29	UP
PASK	1.03583719537825	1.43168444274637e-35	1.44186935809963e-34	UP
PAX2	2.41151786192981	2.15523445910073e-09	5.09283452793066e-09	UP
PAX3	3.1392365864684	2.01976858612509e-07	4.17252703644516e-07	UP
PAX5	-1.96361943904079	1.94259765021172e-10	4.93672692398375e-10	DOWN
PAX7	4.40303333114685	1.25544678095024e-08	2.81642550846258e-08	UP
PAX9	2.7686370334172	3.54383433022654e-32	2.99994103997252e-31	UP
PAXX	1.04495491409693	9.8349214329038e-27	6.32172340019025e-26	UP
PBLD	-2.30160566160347	6.48304304989658e-59	1.98399347324867e-57	DOWN
PBOV1	1.79658435706864	0.000190936329492728	0.000309223800730736	UP
PBX1	-1.49086066570237	2.0524027853977e-29	1.50668169064629e-28	DOWN
PBX4	2.02917256345811	2.30919988092978e-56	6.42333826303997e-55	UP
PBXIP1	-1.09761471327877	1.74124833583404e-31	1.41816451224875e-30	DOWN
PC	-1.03563220698706	5.63874647350439e-19	2.37972594142346e-18	DOWN

GeneSymbol	log2FoldChange	pvalue	padj	change
PCBP1	1.73612461682028	1.52194082372369e-14	4.95634104423053e-14	UP
PCDH10	-2.57014088741755	4.70012195977999e-13	1.41036056670083e-12	DOWN
PCDH11X	-3.02321753101627	6.0453008497248e-14	1.90666226582608e-13	DOWN
PCDH11Y	-3.92530058323969	2.63234928595766e-13	8.02610718199629e-13	DOWN
PCDH15	-2.16326650190665	2.56394460293714e-10	6.46013129372658e-10	DOWN
PCDH20	-1.67432423556855	1.90175146495498e-08	4.22280008406797e-08	DOWN
PCDH7	-1.66818178642422	3.86720456459918e-20	1.74500215297656e-19	DOWN
PCDH9	-2.21724746004745	2.99119431566626e-19	1.28384684788505e-18	DOWN
PCDHA3	-1.48266555981446	3.05483602372544e-09	7.14203271765993e-09	DOWN
PCDHB4	-1.1962254661294	2.86490613262142e-10	7.19138107068426e-10	DOWN
PCDHB5	-1.00853212545603	9.74371648550918e-07	1.91671187485508e-06	DOWN
PCDHB8	1.32622058361799	6.82255984740392e-05	0.000114905923805827	UP
PCDHB9	1.5808358065606	4.27791055295262e-15	1.44502922325051e-14	UP
PCDHGA1	1.40203299362838	2.06937240457367e-17	7.99334110045282e-17	UP
PCDHGA4	-1.01738142120552	1.51410144167915e-06	2.93728257619865e-06	DOWN
PCDHGB7	-1.0210772732891	4.02230684091641e-07	8.12816862259225e-07	DOWN
PCDHGC3	-1.08633745646645	6.72468938737305e-10	1.6497184601624e-09	DOWN
PCDHGC4	-1.24701024228779	1.45164993693462e-07	3.0305215623837e-07	DOWN
PCED1A	1.11837014785608	1.31707076639991e-20	6.08979109275669e-20	UP
PCID2	1.06725038233124	3.22708043908507e-40	4.10768652673891e-39	UP
PCK1	-2.78370730378255	2.20443597504101e-31	1.78538807597159e-30	DOWN
PCNA	1.08362046517739	4.65422328832339e-27	3.04905907146633e-26	UP
PCNX2	1.28912638544415	4.15476105279336e-40	5.26541879274314e-39	UP
PCOLCE2	-3.25331158998748	8.92076046673706e-27	5.75109966706007e-26	DOWN
PCP2	1.82281941054146	9.55244644038648e-17	3.54209433276086e-16	UP
PCP4	-1.58904261998076	7.12562790740892e-06	1.30925987919924e-05	DOWN
PCP4L1	-3.86755172385916	3.12533901852656e-37	3.40773502376976e-36	DOWN
PCSK1	2.42910673922137	2.1832781284054e-11	5.90057768895284e-11	UP
PCSK2	-4.92189755213567	7.86206711671006e-35	7.63536947592149e-34	DOWN
PCSK4	1.3914712517385	8.77591015821306e-16	3.07165675547666e-15	UP
PCSK5	-2.5451578361467	1.03091739505814e-57	3.03745593911125e-56	DOWN
PCSK9	2.71001373452365	1.13312551144378e-46	1.95745183837009e-45	UP
PCYT1B	-1.00351188648509	0.000142183835666238	0.000232998977080389	DOWN
PDCD2L	1.79491429718833	7.80782298600453e-83	7.34906063001605e-81	UP
PDCD4	-2.04283511973541	2.71544043928608e-127	1.00604179509125e-124	DOWN
PDCD5	1.04487158522334	6.50994761361627e-37	6.99307327550279e-36	UP
PDE1A	-1.76008028683805	1.17106334633862e-26	7.49695810654204e-26	DOWN
PDE1B	-1.23942852316855	5.56329731061975e-12	1.56197510593069e-11	DOWN
PDE1C	-1.66065004287754	3.45961185964808e-32	2.93578076569454e-31	DOWN
PDE2A	-2.70173639065237	3.66297044156698e-71	1.99322825934393e-69	DOWN
PDE3A	-2.07350500002694	3.49001476248375e-28	2.39919569913658e-27	DOWN
PDE4D	-1.55515696003321	6.78721643811669e-37	7.28193467880011e-36	DOWN
PDE5A	-2.10063403994568	1.66280150073406e-52	3.82994213389976e-51	DOWN
PDE6A	-3.48889991119693	2.23579537094699e-44	3.41507936792105e-43	DOWN
PDE6B	-1.04078177016881	1.58056763120906e-09	3.77666943444226e-09	DOWN
PDE6G	-1.31634417062242	1.3995802889898e-15	4.84510766842534e-15	DOWN
PDE7B	-2.29385175757515	9.613076586142e-52	2.11354169942539e-50	DOWN
PDE8A	-1.21201242553117	7.025073003541e-72	3.9333632222077e-70	DOWN
PDE9A	-2.59111289711529	2.81971966144207e-55	7.43936037343799e-54	DOWN
PDF	2.76514927168751	1.31589902769619e-27	8.8299613754427e-27	UP
PDGFD	-1.70151672429588	1.80580789668429e-34	1.71546824358775e-33	DOWN
PDGFRA	-1.23977580024501	3.90677581715329e-18	1.57328139001134e-17	DOWN
PDIA2	1.83894041221959	4.07551075363381e-14	1.29690915118833e-13	UP
PDK4	-3.38011266767171	1.08132790987293e-88	1.29856295824947e-86	DOWN
PDLIM2	-1.02177754148537	2.94973410451231e-16	1.06233133323418e-15	DOWN
PDLIM3	-2.24538104689524	6.32485350426931e-35	6.17000975181185e-34	DOWN
PDLIM7	-1.02880514688058	1.43551217758034e-13	4.44304542271712e-13	DOWN
PDPN	1.712025039298	8.90604190657534e-29	6.30666562501816e-28	UP
PDRG1	1.3984185526155	3.30580820158268e-43	4.78504058305563e-42	UP

GeneSymbol	log2FoldChange	pvalue	padj	change
PDX1	4.68390571091757	6.57363966839473e-157	5.20303579753443e-154	UP
PDXP	1.37963605827809	1.69472723917485e-07	3.51899420650509e-07	UP
PDZD3	-1.20250837843867	5.73291066409753e-11	1.51070177654253e-10	DOWN
PDZD4	-3.25104779640432	1.85164516727935e-55	4.93007603942436e-54	DOWN
PDZD7	2.164693692048	4.25695308434199e-46	7.10022261088574e-45	UP
PDZK1	-2.43320098253336	1.02096267279448e-28	7.20925507760355e-28	DOWN
PDZK1IP1	1.11204481746586	2.31891329495962e-11	6.2584062624197e-11	UP
PDZRN3	-1.54883260014698	1.93404351926271e-23	1.0442635597185e-22	DOWN
PDZRN4	-4.35697111575541	4.35541826709954e-49	8.41741379411812e-48	DOWN
PEBP4	-1.43869656938294	7.75397980130566e-09	1.76982632429067e-08	DOWN
PEG3	-3.03412441803985	7.14112827437092e-40	8.91989733371216e-39	DOWN
PELI2	-1.29729864581893	4.73870743902641e-20	2.1272264149463e-19	DOWN
PEMT	1.24131434728818	2.40270841995469e-22	1.21871138120218e-21	UP
PENK	-3.24737508705565	3.01796215344633e-16	1.08578047475126e-15	DOWN
PER1	-1.61603205648833	8.69991384439116e-33	7.62797581935465e-32	DOWN
PER3	-1.77583156176776	7.81753443225773e-33	6.86814970075196e-32	DOWN
PERM1	1.0384646115837	0.000434779899862649	0.000682906653247796	UP
PERP	1.23432376641419	3.64358663108125e-36	3.78103539970308e-35	UP
PET100	1.25849915866988	4.97067459200107e-15	1.67254795498579e-14	UP
PEX11A	-1.26022452325966	6.69990052383035e-50	1.34945862609821e-48	DOWN
PEX26	-1.77793077305173	1.88029472177577e-160	1.8189762216823e-157	DOWN
PF4	1.8794189989463	3.25950862636893e-12	9.28933284958463e-12	UP
PFDN2	1.06334420988402	1.09940967578557e-34	1.05826537780288e-33	UP
PFDN4	1.09867089521878	5.87258076129417e-18	2.34217244151203e-17	UP
PFDN5	1.06131734503909	7.33952897233311e-22	3.62459495165163e-21	UP
PGAM5	1.04830866246938	7.76452479193215e-37	8.29979559250549e-36	UP
PGC	4.41241801959752	1.23565721812041e-44	1.91427928284081e-43	UP
PGF	1.4649509807437	2.04119488375594e-23	1.0993914788383e-22	UP
PGGHG	3.17833018986676	3.0652242632991e-57	8.85153401274083e-56	UP
PGLYRP3	4.48436292488167	5.81568526664393e-17	2.18722521702097e-16	UP
PGLYRP4	2.50892802018259	5.5541120978701e-13	1.66060703915199e-12	UP
PGM1	-1.38338549772994	4.68047640561936e-39	5.58226956514041e-38	DOWN
PGM5	-3.61433506521909	1.2839914268331e-52	2.96920885995283e-51	DOWN
PGP	1.08568787930348	1.30657865327944e-17	5.10351146019626e-17	UP
PGPEP1L	-2.11503782890402	1.23900404672492e-06	2.42005355755705e-06	DOWN
PGR	-2.78062307472891	2.99353409155519e-34	2.80551179420078e-33	DOWN
PHACTR3	1.61956617569852	2.96256355383126e-10	7.42824288681254e-10	UP
PHF21B	-1.30245198846033	1.6855417612213e-05	2.9992171150773e-05	DOWN
PHF24	-2.46232999557235	3.91229264280194e-23	2.07760755684996e-22	DOWN
PHGDH	1.6697268465802	1.11107713437539e-17	4.36238695397491e-17	UP
PHGR1	-1.33235785868601	3.43552957670233e-09	8.01485483911008e-09	DOWN
PHKA1	1.25409442893419	1.09824374296426e-24	6.34917606116754e-24	UP
PHKG1	-1.00945321301754	7.62412768405372e-13	2.25627014552052e-12	DOWN
PHLDA1	2.47099266272628	1.03794080804812e-59	3.32236457546726e-58	UP
PHLDA2	1.45490637021492	1.01121945885588e-22	5.24839476514381e-22	UP
PHLDB2	-1.36153971186674	2.68894911938821e-14	8.64205814247083e-14	DOWN
PHLPP2	-2.65253421431107	2.15025312314093e-160	1.97065040175016e-157	DOWN
PHOX2A	-3.60545089621767	1.07642716077527e-29	7.99992580050352e-29	DOWN
PHOX2B	-4.65806185961878	1.38210342237172e-35	1.39354759083722e-34	DOWN
PHYH	-1.02823424923531	1.02776019550174e-23	5.64021061590666e-23	DOWN
PHYHD1	-1.37645192737555	5.21529235488532e-14	1.65026141696562e-13	DOWN
PHYHIP	-1.16793877382393	2.58304215269528e-08	5.6859388899231e-08	DOWN
PHYHIPL	1.70019769609527	2.96023637465722e-06	5.60776718797934e-06	UP
PI15	-1.74303750447529	2.01775773291914e-11	5.46341399522952e-11	DOWN
PI16	-4.32349135461672	4.97446196427823e-40	6.25417373169508e-39	DOWN
PIANP	-2.34057054803361	2.25364535312642e-32	1.9331392381276e-31	DOWN
PID1	-1.64196426204751	2.13204581440908e-21	1.02783260704057e-20	DOWN
PIF1	1.64446976066953	1.30571575896775e-44	2.01922100452091e-43	UP
PIGR	-1.66542151032554	2.9381037491415e-09	6.87927935777881e-09	DOWN

GeneSymbol	log2FoldChange	pvalue	padj	change
PIGZ	-1.68670800595542	3.1617965267769e-27	2.08704939047635e-26	DOWN
PIK3C2G	-3.37522411425575	1.20530124657533e-17	4.71956613596047e-17	DOWN
PIK3CD	-1.05020791379192	1.50224686187926e-12	4.36923744878963e-12	DOWN
PIK3CG	-1.82367900620073	9.20582879179429e-31	7.20454367422534e-30	DOWN
PIK3R2	1.2331340210567	1.15524584242231e-10	2.98372824890236e-10	UP
PIK3R5	-1.0080282092863	2.40487125334824e-10	6.06898886008013e-10	DOWN
PILRB	1.79771032361528	5.43837479068737e-22	2.70489632191486e-21	UP
PIMREG	1.23625865949597	1.14844463925136e-31	9.473172194829e-31	UP
PINK1	-1.40075643496801	3.68792601766577e-51	7.95760294245527e-50	DOWN
PINLYP	-1.14957019734879	1.12733199151874e-19	4.95087817612e-19	DOWN
PIP5K1B	-1.1022419958427	1.16388622425141e-30	9.04361928732251e-30	DOWN
PIP5KL1	1.10779661371003	1.54167146409742e-08	3.44168271850363e-08	UP
PIPOX	1.25483474690834	5.16303091552458e-06	9.58974478208314e-06	UP
PIRT	-4.18740861260014	1.18297354941971e-44	1.83429371469683e-43	DOWN
PITPNM3	-1.47691045281765	7.37805985562259e-24	4.07595673432602e-23	DOWN
PITX1	1.77036100689523	1.59268710113892e-36	1.67878090146078e-35	UP
PIWIL1	4.43332665799195	2.28331626802398e-39	2.76298722551087e-38	UP
PJA2	-1.49119609375112	1.40736650895557e-68	6.82631560458031e-67	DOWN
PKD1L3	1.53012635487528	2.7672485236819e-10	6.95326097299755e-10	UP
PKHD1L1	-3.37691052805661	1.11772238584328e-55	3.01750386119209e-54	DOWN
PKIB	-3.97791716297614	7.38815236803466e-89	8.99649630661452e-87	DOWN
PKMYT1	2.01710828531649	8.33697824804163e-52	1.83761774978669e-50	UP
PKNOX2	-2.77573322455881	7.26831986474059e-52	1.61227074910481e-50	DOWN
PKP1	4.0369450780477	3.61806296046804e-61	1.23290274619628e-59	UP
PLA2G10	-1.4081133844342	9.86127044394557e-15	3.2601918025522e-14	DOWN
PLA2G2A	-1.00014771114898	0.00142107909157694	0.00212533283703764	DOWN
PLA2G2C	-3.16350961126719	4.10370524929435e-50	8.38706801713174e-49	DOWN
PLA2G2D	-1.63260493419638	2.14386015207759e-08	4.74044912103201e-08	DOWN
PLA2G2F	2.50113380841329	6.62410988998377e-11	1.73660983911905e-10	UP
PLA2G3	4.32867112661153	1.1284878614337e-26	7.23237362206294e-26	UP
PLA2G4B	1.3669399951267	2.21908109765424e-13	6.79937694060414e-13	UP
PLA2G4D	1.30380964910906	1.65489668772657e-08	3.68824156482568e-08	UP
PLA2G4E	1.74455361670862	7.55498483346482e-20	3.34830620781682e-19	UP
PLA2G5	-2.52249689947818	3.75407682022367e-31	2.99586341294935e-30	DOWN
PLAAT2	-3.19748106884038	2.25279632993054e-45	3.63558317822804e-44	DOWN
PLAAT3	1.1027496796764	1.48549639809634e-11	4.06010811176449e-11	UP
PLAAT5	-1.98027322213161	1.79580290042983e-10	4.57235208439605e-10	DOWN
PLAC1	2.78442368799193	9.93017003441187e-21	4.62955959328551e-20	UP
PLAC8	-3.00908060237489	9.46455455747186e-43	1.34535745721843e-41	DOWN
PLAC9	-2.63743217935139	3.31664684342162e-84	3.31912479795981e-82	DOWN
PLAGL2	1.2196386987666	1.47471152766416e-21	7.17495161531602e-21	UP
PLAU	2.16639387119069	2.32904124143786e-48	4.34679476282502e-47	UP
PLCB4	1.43744324279769	2.42864057815653e-13	7.42449409891848e-13	UP
PLCD1	-2.17760730512939	3.50652242596295e-92	5.04620454572669e-90	DOWN
PLCD3	-1.57117348725152	2.7227245500474e-37	2.97432889523057e-36	DOWN
PLCD4	-4.24667648852117	5.24425661515052e-64	2.03835358124143e-62	DOWN
PLCE1	-2.07447763718024	1.01332390070777e-89	1.28795686737404e-87	DOWN
PLCG1	1.0384266149473	3.29719370214136e-25	1.95551886700911e-24	UP
PLCG2	-1.87709294332016	2.16408637587132e-36	2.26734272340838e-35	DOWN
PLCL1	-1.34735626888226	9.42553994804449e-20	4.15616427235499e-19	DOWN
PLCL2	-2.46786675096133	3.69312320605581e-50	7.5746000455889e-49	DOWN
PLCXD3	-2.84570555286983	5.20393455050518e-14	1.64696678167842e-13	DOWN
PLD1	-1.21310113023259	2.36960873447452e-34	2.23037821045431e-33	DOWN
PLD4	-1.19237996818207	3.12213405764966e-10	7.81678222082724e-10	DOWN
PLD5	-3.53868772664165	1.14541351952186e-22	5.92721712197151e-22	DOWN
PLEKHB1	1.21224662604702	6.10112228282101e-12	1.70994434750945e-11	UP
PLEKHD1	1.04704911670662	1.12792787675391e-05	2.03508528835518e-05	UP
PLEKHG4	2.11173983379223	3.06152276992e-56	8.44854136174597e-55	UP
PLEKHG4B	-1.7505201110821	2.2236579356267e-10	5.62390060030032e-10	DOWN

GeneSymbol	log2FoldChange	pvalue	padj	change
PLEKHH2	-1.10422163933858	5.85417923404529e-11	1.54102529104204e-10	DOWN
PLEKHN1	3.47894614870419	5.06170636315186e-124	1.72822535101105e-121	UP
PLEKHO1	-1.63913880900593	1.5849174246467e-34	1.51222833508893e-33	DOWN
PLEKHS1	1.72692028771622	1.99986936581222e-27	1.32914981934688e-26	UP
PLIN1	-1.88799256939328	7.57341766844143e-09	1.72952028669601e-08	DOWN
PLIN4	-2.97175093940881	5.19519628326942e-17	1.96064050456373e-16	DOWN
PLK1	1.54542670733456	4.0292906126722e-43	5.8081156819918e-42	UP
PLK5	2.39628558570571	4.87266617728847e-16	1.73158645194131e-15	UP
PLN	-3.75218095146612	3.22408733107086e-49	6.2727410833449e-48	DOWN
PLOD2	-1.02693823024695	1.3299695234264e-14	4.35233213896334e-14	DOWN
PLOD3	1.28856397746742	6.78402745390083e-38	7.62130774546936e-37	UP
PLP1	-5.6013662703393	6.85021223267078e-69	3.39836882072639e-67	DOWN
PLPP1	-1.88315324393629	1.50550750946372e-95	2.5451846856594e-93	DOWN
PLPP2	1.03816444027187	4.49561007064014e-21	2.12722984130589e-20	UP
PLPP3	-1.52412037764167	3.65828968990338e-68	1.75487047852032e-66	DOWN
PLPP4	2.69252968358478	3.32071273049434e-28	2.28551663146632e-27	UP
PLPP7	-1.31718284561952	7.73832319039056e-17	2.88353138699488e-16	DOWN
PLPPR1	1.2165001110566	0.000352086800275762	0.000557201440807219	UP
PLPPR3	1.12365455636488	0.000479227235744822	0.000749823331478532	UP
PLPPR5	-1.48245989872596	1.99054809838149e-08	4.4126561473096e-08	DOWN
PLS1	-1.46553666506669	3.32270928547807e-47	5.87394282111977e-46	DOWN
PLSCR3	1.99003060789897	2.58657519740412e-13	7.8920683217799e-13	UP
PLSCR4	-1.69879663547085	2.51001793408743e-49	4.9108923917151e-48	DOWN
PLXNA1	1.35966156776273	6.40164971450838e-51	1.35775793518556e-49	UP
PLXNA2	-1.13112459261472	3.80090093764249e-32	3.20975208667161e-31	DOWN
PLXNA3	1.2063059139967	7.71319146140095e-26	4.75938351939669e-25	UP
PM20D1	-1.34966977831938	1.49868824718049e-09	3.58864940156132e-09	DOWN
PMAIP1	1.91962421915759	2.84955759806703e-43	4.13494553792843e-42	UP
PMEPA1	1.86186012902528	5.70336279753473e-32	4.7838466470844e-31	UP
PMFBP1	1.50895786278905	4.6579311680787e-18	1.86972234738945e-17	UP
PMP2	-4.76053555437537	1.56179896268266e-46	2.68201236066994e-45	DOWN
PMP22	-1.37402898058293	1.36721341830245e-22	7.03109487681648e-22	DOWN
PNCK	-3.36956545036136	3.43185091546025e-35	3.38385164161435e-34	DOWN
PNMA1	-1.2135222011808	2.05609126333279e-22	1.0483958175231e-21	DOWN
PNMA5	2.88491091225944	1.60954098446627e-09	3.8435185357256e-09	UP
PNMA8A	-1.43184236575978	4.28454545770549e-13	1.28899084407439e-12	DOWN
PNMA8B	-1.1710093707435	4.8356449347853e-11	1.27948769563009e-10	DOWN
PNMA8C	-2.5037560293171	2.54600890415571e-20	1.1614789899938e-19	DOWN
PNMT	1.25168606829623	1.93822270375422e-06	3.72766423022667e-06	UP
PNO1	1.04674908434879	6.62016422126926e-47	1.16089546409831e-45	UP
PNOC	-1.90331661583067	1.92089944818602e-16	7.01015955915633e-16	DOWN
PNPLA1	1.23568775115774	1.19852941491744e-08	2.69290228412353e-08	UP
PNPLA3	3.59658493032819	2.68244992527103e-33	2.40894793959487e-32	UP
PODN	-1.01687353111375	7.70088867775339e-08	1.636709075378e-07	DOWN
PODNL1	1.98148102558956	2.55791841988846e-34	2.40502340418562e-33	UP
PODXL2	1.57796585641424	6.7979782005637e-35	6.6167241143888e-34	UP
POGLUT2	1.06424687373499	1.07548547173444e-23	5.89840268324783e-23	UP
POLA2	1.14720955553515	2.34213480120821e-25	1.39622024284281e-24	UP
POLD1	1.07306309905211	1.1599776146996e-22	5.99901698983193e-22	UP
POLD2	1.31584422873829	2.77421991072943e-48	5.14456776416737e-47	UP
POLE2	1.03043090549441	4.28882858398405e-24	2.40752327959105e-23	UP
POLQ	1.33723765572285	7.21527539362963e-34	6.64408198991394e-33	UP
POLR1B	1.19515934507107	1.98940834962964e-92	2.91105610017655e-90	UP
POLR1C	1.42873156781651	4.77788790382839e-67	2.18940426498326e-65	UP
POLR1D	1.10226435424396	9.36560952646484e-31	7.3262964368523e-30	UP
POLR1G	1.87823382017116	7.00578044688838e-97	1.24481280532314e-94	UP
POLR1H	1.01404361992028	7.87614386263484e-25	4.59147281821428e-24	UP
POLR2I	2.1203766342396	1.76579035058641e-23	9.5757419416883e-23	UP
POLR3G	1.42703918169963	3.06690918786828e-26	1.92864173666849e-25	UP

GeneSymbol	log2FoldChange	pvalue	padj	change
POLR3K	1.01095489023406	3.01464677074709e-23	1.61222494530157e-22	UP
POP1	1.44444550229373	4.44349519779141e-56	1.21467161505717e-54	UP
POPDC2	-3.44203042844174	1.47058368231353e-77	1.0943279341934e-75	DOWN
POPDC3	-1.29722143268374	9.61625091371591e-06	1.74679508825929e-05	DOWN
POSTN	1.18125902882478	1.36827311979123e-07	2.86367065323613e-07	UP
POTEF	1.62006229799823	2.79237442638626e-09	6.54510915152295e-09	UP
POU2AF1	-2.03091201760112	2.86905425789449e-18	1.16210378675778e-17	DOWN
POU2F2	-1.5229834897674	1.92706942307105e-20	8.82821885396902e-20	DOWN
POU3F2	2.15942812673328	5.47526951703231e-12	1.53850037276236e-11	UP
POU3F3	-2.32666948775711	3.50748262554546e-13	1.0603436624761e-12	DOWN
POU3F4	-4.38109614560416	1.67650898496343e-12	4.86307695405102e-12	DOWN
POU4F1	2.72556596138352	1.34433678791957e-19	5.88016490531109e-19	UP
POU5F1B	4.66942617836565	6.60094234977225e-97	1.18497122821221e-94	UP
POU5F2	1.54567805811945	3.13743121807704e-05	5.45727125757597e-05	UP
POU6F2	3.53831186746543	5.11603990619436e-15	1.72013135521457e-14	UP
PPA1	1.15823709658559	4.94751515594364e-48	9.06853488531017e-47	UP
PPAN	1.02047690576094	3.24288796692146e-27	2.13976537203499e-26	UP
PPARD	-1.01841560260167	5.70584570027048e-30	4.30671396527134e-29	DOWN
PPARGC1A	-1.9131664528645	8.83153280658676e-27	5.6977947669913e-26	DOWN
PPARGC1B	-1.39890649664946	5.81168724524774e-49	1.11856264680075e-47	DOWN
PPAT	1.50674335275032	9.94870351359052e-70	5.0951992435927e-68	UP
PPBP	5.75747281263147	2.70510406654885e-45	4.3294096609205e-44	UP
PPEF1	2.74968456910017	2.49961665768906e-37	2.73919602645309e-36	UP
PPFIA4	1.21537922149966	1.90035956796031e-10	4.83150257802495e-10	UP
PPIL1	1.06391135234749	2.71513492489698e-31	2.18781325530917e-30	UP
PPM1A	-1.00627235072072	1.95457682756401e-60	6.44603149590381e-59	DOWN
PPM1H	2.2578480957207	2.77251138960632e-114	7.31480921624467e-112	UP
PPM1J	1.31688060158262	3.14772851704845e-21	1.50456757253266e-20	UP
PPM1L	-1.06956941095941	2.3422893754966e-20	1.06994451457299e-19	DOWN
PPM1N	1.59750575461343	5.14725376362937e-22	2.56229644900167e-21	UP
PPP1R12B	-2.90203759716931	7.26997407002656e-69	3.59636529776626e-67	DOWN
PPP1R13L	1.26228889103244	2.07755664183305e-26	1.31646629564188e-25	UP
PPP1R14A	-2.51051945073628	2.27315989454152e-44	3.46910896088093e-43	DOWN
PPP1R14D	-1.36299287372617	2.35278750198135e-18	9.5923879119647e-18	DOWN
PPP1R16B	-1.78155862679161	7.63239765125065e-30	5.72117693935547e-29	DOWN
PPP1R1A	-3.55554229196972	1.87012401011676e-31	1.52099343242238e-30	DOWN
PPP1R27	1.86811239880842	2.64887413857715e-08	5.82752310486973e-08	UP
PPP1R35	1.67253471402941	1.86711034393986e-32	1.60711776663494e-31	UP
PPP1R36	-1.21373556301465	1.34947205387733e-15	4.67722071539928e-15	DOWN
PPP1R3C	-2.40324161355797	1.27100396843025e-31	1.04544128966821e-30	DOWN
PPP2R2B	-2.23909845988397	3.69049522229093e-32	3.12105844127013e-31	DOWN
PPP2R2C	1.96576562647462	2.10075137005265e-11	5.68106594296115e-11	UP
PPP2R3A	-2.14880496011208	1.11865762944441e-71	6.18386834968747e-70	DOWN
PPP2R3B	1.67396179061288	2.20365789055462e-43	3.21376003754001e-42	UP
PPP4R4	1.04484056990192	0.000139356666471054	0.000228581163645485	UP
PPRC1	1.03178010347997	5.43934252137645e-47	9.56719912370991e-46	UP
PPY	-4.43724994124025	2.87456882988055e-25	1.70835723667952e-24	DOWN
PRAME	2.79625678192477	1.43950277170575e-15	4.97935275401515e-15	UP
PRB1	3.15205386118679	0.000109766898866727	0.000181620202391326	UP
PRB2	2.96607659879324	1.15276804449923e-06	2.25465011331742e-06	UP
PRC1	2.36588004651328	3.25549538032311e-33	2.90707390038801e-32	UP
PRDM12	2.77943591872514	1.96936886216249e-48	3.69532543069347e-47	UP
PRDM13	4.18403236794193	4.62400266274049e-24	2.58982818804439e-23	UP
PRDM6	-2.40724396093917	4.94381044887372e-34	4.57907294394883e-33	DOWN
PRDX4	1.24002772672397	2.75100650142321e-36	2.87017832290487e-35	UP
PRDX6	-1.47302988273832	1.71078321869485e-98	3.27361188869599e-96	DOWN
PRELID3A	1.90903589663766	6.28906252139776e-59	1.92801840994893e-57	UP
PRELP	-2.56717525117693	3.80179259596327e-22	1.90779868799736e-21	DOWN
PREX2	-1.53821049515431	4.50004259168592e-24	2.52202258284605e-23	DOWN

GeneSymbol	log2FoldChange	pvalue	padj	change
PRH2	1.0164331350346	3.94025790609003e-06	7.39139149285875e-06	UP
PRICKLE2	-1.81228166588937	1.30728545482847e-33	1.19369489380851e-32	DOWN
PRIMA1	-4.76604612696818	2.79385879307318e-53	6.60998140812273e-52	DOWN
PRKAA2	-2.11198205392333	4.65680561885467e-12	1.31445868440779e-11	DOWN
PRKACB	-2.06592153772781	1.64117849810353e-41	2.21284017345568e-40	DOWN
PRKAR2B	-2.06456297505522	7.87033959079387e-32	6.55096669667752e-31	DOWN
PRKCB	-2.55988208776347	5.63168991939682e-47	9.89552134878474e-46	DOWN
PRKCG	3.66881958618562	1.5348951604765e-32	1.32575046772705e-31	UP
PRKDC	1.12346287985809	2.56022873584337e-28	1.77050289822242e-27	UP
PRKG1	-1.41647810742945	6.95443691004625e-24	3.84925651349763e-23	DOWN
PRKG2	-4.05721265810222	4.26237392381681e-116	1.15969870524097e-113	DOWN
PRKN	-1.46390513477027	1.86755985355803e-22	9.54780379624369e-22	DOWN
PRL	1.63153673823775	9.8274827683338e-05	0.000163272547891419	UP
PRMT1	1.28669451792839	2.01497952203991e-28	1.39899674710052e-27	UP
PRMT3	1.19699055223689	2.12254294867589e-46	3.60583808441886e-45	UP
PRND	-1.87630297653231	2.02047566114121e-10	5.12397072101033e-10	DOWN
PRNP	-1.15365645484773	1.16184511930128e-19	5.09859099354669e-19	DOWN
PROCR	1.17339176143638	9.77520218305323e-18	3.8503712445554e-17	UP
PRODH	1.23683494873052	1.61562611722602e-06	3.12761507273559e-06	UP
PROKR1	-2.94995901184215	9.81251583152501e-30	7.32070857645008e-29	DOWN
PROM2	-1.17491937309367	2.65827208894345e-07	5.44186361212936e-07	DOWN
PROSER1	1.21955124738867	3.14172823812888e-29	2.2804049108186e-28	UP
PROX1	1.80586372045286	2.1665173448587e-29	1.58577412887871e-28	UP
PROZ	1.60204800061558	2.99212548251822e-14	9.59165703738765e-14	UP
PRPH	-3.40010748777447	3.90327909292723e-39	4.67775628665807e-38	DOWN
PRPH2	-1.22738598400747	5.12430214373197e-15	1.72257670325878e-14	DOWN
PRR11	1.41272820954298	2.58470651836073e-55	6.84004477267712e-54	UP
PRR19	1.64069056115008	9.84793428262306e-40	1.2213823337843e-38	UP
PRR20G	2.78381333612948	1.62997875444905e-05	2.90480197024064e-05	UP
PRR22	1.27958220353105	1.2758278193922e-17	4.98787378066376e-17	UP
PRR33	-1.59785610465635	2.8574865696304e-18	1.15822657441746e-17	DOWN
PRR36	4.12181489283168	5.50956439420338e-82	4.79690223981317e-80	UP
PRR4	1.05379346331263	6.99672277784512e-10	1.71307555864197e-09	UP
PRR5-ARHGAP8	1.72226545575031	3.21310535129042e-24	1.81302020356514e-23	UP
PRR5L	-1.08508981687487	1.13055663122122e-31	9.3344630722879e-31	DOWN
PRR7	3.68350962108175	1.47312373783608e-91	2.03583362277299e-89	UP
PRR9	6.86430763164261	2.03065903801599e-26	1.28721754018829e-25	UP
PRRG3	-1.89995104317285	3.38419169934293e-22	1.70413331580851e-21	DOWN
PRRT1	-1.66765744936161	3.79972511814657e-36	3.93836985013608e-35	DOWN
PRRT4	-1.42351786835611	1.32355817908561e-06	2.58028645011393e-06	DOWN
PRRX1	1.25831014901519	4.40761307654425e-10	1.09486114838609e-09	UP
PRSS1	4.12957420189798	1.59220047612537e-25	9.61337964312453e-25	UP
PRSS2	5.46099927707189	2.4766072344782e-36	2.59009980624438e-35	UP
PRSS21	1.22552904754397	0.0027794384624734	0.00403757086402346	UP
PRSS22	3.64782407739302	4.91902377586942e-107	1.18965223623909e-104	UP
PRSS33	4.71093203475487	2.44576603675463e-46	4.13476932019499e-45	UP
PRSS36	-1.16211835988348	6.43069307591826e-20	2.86607777146057e-19	DOWN
PRSS41	3.97388030757055	7.73312072450804e-29	5.48947538425844e-28	UP
PRSS48	1.32484146822223	0.000835915115230057	0.00127850592020211	UP
PRSS51	2.82805702412735	4.18112319944833e-21	1.98543491333498e-20	UP
PRSS53	1.13574595313902	6.67319904675634e-13	1.98057635931768e-12	UP
PRSS56	8.52656161064633	1.84836684941938e-44	2.84576586639608e-43	UP
PRUNE2	-1.6520422224912	2.33244091222187e-17	8.98358628721951e-17	DOWN
PRX	-1.13079116328048	2.43200852268382e-19	1.05005118783767e-18	DOWN
PSAPL1	2.0988258555618	6.05528977031547e-05	0.000102389552117405	UP
PSAT1	2.72500198777033	2.05555885903944e-79	1.62697483692972e-77	UP
PSD	-3.64895891387416	1.32916918119493e-55	3.54981947118823e-54	DOWN
PSMA2	1.18753970144089	1.12030157333672e-18	4.64029764427028e-18	UP
PSMA6	1.14908606990384	4.03533114606830e-11	1.07245453672905e-10	UP

GeneSymbol	log2FoldChange	pvalue	padj	change
PSMA7	1.28615109720996	1.1844287690709e-34	1.13633378269045e-33	UP
PSMG1	1.07953831656145	6.05300802767049e-25	3.54646799413951e-24	UP
PSMG3	1.00066193945289	3.41308895699382e-27	2.24866129429184e-26	UP
PSMG4	1.13022408791144	1.2675381078708e-26	8.09968479719422e-26	UP
PSORS1C2	3.01696812019145	2.30891723830741e-21	1.11064021742119e-20	UP
PSPH	1.40714880125916	1.01766772558319e-45	1.66704121407151e-44	UP
PSRC1	1.33197150140458	4.99293248728043e-48	9.14215913785637e-47	UP
PTBP2	-1.1033017865834	6.0684250713912e-26	3.76852659658113e-25	DOWN
PTCD1	1.03529799619373	3.19274146128296e-19	1.36765577036458e-18	UP
PTCHD1	-3.31928250936879	9.79185801791276e-25	5.67784294591791e-24	DOWN
PTCHD4	1.26768769185496	2.74582346552868e-05	4.80146856851285e-05	UP
PTCRA	-1.21409123908821	2.07181009611717e-08	4.58603208387446e-08	DOWN
PTF1A	5.97890208443667	1.07665642818748e-19	4.73190771934089e-19	UP
PTGDR	-1.75438287096888	5.36617636812664e-08	1.15516416242044e-07	DOWN
PTGDR2	-2.42381072357089	1.49893230450107e-39	1.82907555839363e-38	DOWN
PTGER3	-1.29550096343359	1.29971402547331e-10	3.34791720792408e-10	DOWN
PTGER4	-1.58021452628252	2.73100809895454e-38	3.13274334829351e-37	DOWN
PTGES	1.01557076217618	1.83603900724705e-08	4.07896749594193e-08	UP
PTGFR	-1.21426055832447	2.53460714755765e-10	6.38898585124803e-10	DOWN
PTGIS	-1.57643228021599	1.87891877623925e-10	4.77838654164657e-10	DOWN
PTGR1	-1.0850309284676	3.55094923967459e-29	2.57100536841803e-28	DOWN
PTGS1	-2.96221070953571	2.89684341264723e-88	3.45498180441275e-86	DOWN
PTH1R	-2.0300852176712	2.3256074252802e-24	1.32295988554081e-23	DOWN
PTK2B	-1.28108025778201	8.73910991936524e-39	1.02612354029607e-37	DOWN
PTK7	2.08879885620398	1.60199968856061e-38	1.86094867090766e-37	UP
PTN	-2.76135256814583	4.81844353954232e-62	1.72641064514507e-60	DOWN
PTP4A1	-1.20839659847339	7.78641937140152e-15	2.59393381507968e-14	DOWN
PTP4A3	2.36145741776336	4.50347819484013e-41	5.94985324785669e-40	UP
PTPMT1	1.38283403618076	8.9663806556798e-21	4.19032706273088e-20	UP
PTPN13	1.08115049372168	7.36593033406725e-08	1.56743180871457e-07	UP
PTPN21	-1.52742393390534	2.55526546721678e-87	2.90815931899645e-85	DOWN
PTPRC	-1.24825353900688	9.53411768159025e-12	2.64359221639381e-11	DOWN
PTPRH	-2.05854529815172	3.0497527757048e-57	8.82148589424379e-56	DOWN
PTPRN	-1.57472857314421	4.47724496173059e-11	1.18754404445719e-10	DOWN
PTPRO	1.4015910853516	2.15184945989163e-09	5.08552587474116e-09	UP
PTPRR	-1.7627209938161	3.98934096683171e-18	1.6054170153788e-17	DOWN
PTPRT	-1.84246491787605	1.02987404879346e-14	3.39772580743472e-14	DOWN
PTPRU	1.69260720502807	2.81352363657525e-14	9.03575932934062e-14	UP
PTPRZ1	-3.05913547560451	1.9650119213354e-18	8.04721368443401e-18	DOWN
PTRH1	2.45223345234696	3.46388159107852e-17	1.32215191024661e-16	UP
PTTG1	1.31307429008163	1.4869086049734e-38	1.73071788358301e-37	UP
PTTG2	-1.08149732748003	1.06206973087441e-06	2.08216845572125e-06	DOWN
PTX3	-1.84993322710529	5.36834099711574e-23	2.82584406840316e-22	DOWN
PUM3	1.11642573990138	2.14958263392212e-53	5.14864957420714e-52	UP
PURG	-1.07518709130542	1.27484374417524e-06	2.48838180891419e-06	DOWN
PUS1	1.60838190675003	5.61919373213462e-63	2.08185149909915e-61	UP
PUS7	1.64901935765337	7.7542540584555e-91	1.03865250707604e-88	UP
PUSL1	1.19851433893003	6.21995949165424e-24	3.45590793325384e-23	UP
PVALB	-1.24553031479148	4.36935052526284e-06	8.16434174229013e-06	DOWN
PYCR1	1.89974517068447	1.30516574210175e-69	6.64527808982976e-68	UP
PYCR3	1.75079590243237	1.45174953316097e-40	1.87810658402169e-39	UP
PYDC1	2.25124524635492	4.16375442158451e-05	7.15588785462407e-05	UP
PYGM	-4.0049822821512	9.65771197239491e-82	8.28422357513855e-80	DOWN
PYGO1	-1.29095719939412	3.60558587823605e-11	9.61323945762125e-11	DOWN
PYHIN1	-1.00581866521034	6.28565230147033e-08	1.34643945781157e-07	DOWN
PYY	-5.64740268768761	2.26228367939769e-51	4.91799571902022e-50	DOWN
QKI	-1.05368977071299	1.81343687843336e-13	5.58595017940211e-13	DOWN
QPCT	1.20590860492566	1.67052809032446e-10	4.2614863224172e-10	UP
QPRT	1.0900647710206	7.24066115699947e-08	1.54171720135524e-07	UP

GeneSymbol	log2FoldChange	pvalue	padj	change
QSOX2	1.26915572722319	6.20028217602608e-73	3.6598479163099e-71	UP
R3HDML	1.97499271511101	1.49608968001673e-18	6.16018198111407e-18	UP
RAB15	1.17839364779365	1.84141328955605e-29	1.35350483795017e-28	UP
RAB23	-1.11507330760992	2.81358793132096e-19	1.20940524927405e-18	DOWN
RAB27A	-1.7190207440213	4.53678052215426e-65	1.89445945401132e-63	DOWN
RAB30	-1.20591906434407	9.56566887857512e-26	5.8588460141621e-25	DOWN
RAB36	1.81624592094283	2.50265771028807e-44	3.80600687416997e-43	UP
RAB37	-1.55658127064056	2.03320976046264e-25	1.21853360416687e-24	DOWN
RAB39B	-1.58260992981798	1.17080649291106e-15	4.07012446816934e-15	DOWN
RAB3B	-1.15898863205229	4.69612696506155e-07	9.44814082525901e-07	DOWN
RAB3C	-2.18294819740328	5.9201375827213e-18	2.36005851025472e-17	DOWN
RAB42	-1.03863081425983	1.09572919522065e-12	3.21373294195337e-12	DOWN
RAB44	-1.46414578706472	7.80638807331157e-15	2.59959142322766e-14	DOWN
RAB5IF	3.7944222801928	2.34915450223092e-37	2.57918205216564e-36	UP
RAB6B	-1.57180598936917	9.60596094263996e-24	5.27494789953294e-23	DOWN
RAB9B	-2.41715908239526	2.05550768538899e-43	3.005252336329e-42	DOWN
RAC3	1.29603052411203	4.81398226470664e-16	1.71143064874105e-15	UP
RACGAP1	1.04950680676695	4.1782876962566e-45	6.62024783029264e-44	UP
RAD51AP1	1.1375930812853	1.72656971217615e-22	8.8390349758723e-22	UP
RAD51AP2	2.09452406330944	3.72844472550786e-22	1.87315083685137e-21	UP
RAD54B	1.98749137574534	1.81598203986054e-64	7.25268239910358e-63	UP
RAD54L	1.53371464343189	2.46934850344526e-57	7.16646091508205e-56	UP
RADIL	-1.45167571070292	2.27116792701144e-10	5.73905777289947e-10	DOWN
RAE1	1.35886645352513	1.9524797536556e-63	7.35898916675432e-62	UP
RAET1E	1.00925889276169	2.79283177313204e-06	5.30217833248454e-06	UP
RAET1G	1.25849312864904	4.26739985728854e-10	1.06109144245274e-09	UP
RAET1L	3.24437966025259	5.55047871553293e-39	6.5928025834635e-38	UP
RAI2	-1.16509692698568	3.27429522202094e-10	8.1883243861914e-10	DOWN
RALYL	-3.52813326304476	4.37583631746786e-17	1.65824674202541e-16	DOWN
RAMACL	1.61220756214087	3.63912758494832e-10	9.08113050110421e-10	UP
RAN	1.00020104901118	3.1787573725069e-46	5.33253392364765e-45	UP
RANBP1	1.06444442856039	1.0069824526147e-44	1.56698708198211e-43	UP
RANBP17	1.36503189799382	2.72695013695045e-22	1.37915720983788e-21	UP
RANBP3L	-1.41126917602288	2.88962345459079e-09	6.7694084777061e-09	DOWN
RAP1A	-1.33707031464905	2.87178006112683e-75	1.90863764138937e-73	DOWN
RARRES2	-1.09809437327153	1.19409368363595e-12	3.49281930340212e-12	DOWN
RASAL1	1.06659138825645	1.24684704950331e-07	2.61740176889705e-07	UP
RASD2	-1.97022437067612	1.02952809923442e-25	6.28582496212095e-25	DOWN
RASGEF1A	1.23254008703536	9.80060237136593e-06	1.77916898553581e-05	UP
RASGEF1C	-3.10797029596662	4.45899577759515e-26	2.778972565328e-25	DOWN
RASGRF1	1.02088438286882	3.98245461381312e-06	7.46865720951297e-06	UP
RASGRP2	-1.67982761293191	7.39845372697357e-27	4.78919236980635e-26	DOWN
RASGRP4	-1.43087738589277	2.28476585313622e-17	8.80580517942916e-17	DOWN
RASL10B	2.27114801622599	4.41692409108788e-21	2.09341042999764e-20	UP
RASL11B	1.04134848086936	0.000632709720382591	0.000978800138683552	UP
RASL12	-1.70406358585629	1.09697809793777e-33	1.0048227048601e-32	DOWN
RASSF10	2.3802253519381	6.86069771744242e-26	4.24237675262162e-25	UP
RASSF2	-1.30854457004105	6.27225179413471e-19	2.63940842173194e-18	DOWN
RASSF6	-1.15177726867112	7.63572438848853e-19	3.19310443748201e-18	DOWN
RASSF8	-1.34261845112095	3.70741818196731e-13	1.11942557313329e-12	DOWN
RAVER2	-1.15975655244816	1.18269903185621e-48	2.24095084240611e-47	DOWN
RBBP8NL	1.39892001939276	3.87947996526075e-25	2.29149880037603e-24	UP
RBFOX1	-1.12957474539499	0.000569936829635839	0.00088467730562033	DOWN
RBFOX3	-4.89185805425786	3.36294829544285e-71	1.8357059143745e-69	DOWN
RBKS	-1.27257316704182	7.78169517249617e-46	1.28316911021473e-44	DOWN
RBL1	1.25752999137702	2.30977467720381e-34	2.1752356113656e-33	UP
RBM14-RBM4	1.16590796903893	6.82133446767173e-16	2.40299205109382e-15	UP
RBM20	-2.95742038373911	3.37812605972546e-44	5.09292719290039e-43	DOWN
RBM24	-2.31750669463908	1.04116510590444e-26	6.68503244436358e-26	DOWN

GeneSymbol	log2FoldChange	pvalue	padj	change
RBM28	1.08449409898128	7.84057567270775e-72	4.37589564707885e-70	UP
RBM47	-1.30486036418723	6.22749200944806e-70	3.20826385682009e-68	DOWN
RBMS3	-1.89185799442348	1.09098981584149e-36	1.15979277553406e-35	DOWN
RBP1	1.51233523898831	8.68301228662737e-10	2.11405610943851e-09	UP
RBP2	2.06269396656105	8.93097906721636e-12	2.48109665758517e-11	UP
RBP3	2.4807186526532	1.22902505356242e-08	2.75928484498226e-08	UP
RBP4	1.37814577661445	5.02576576111724e-08	1.08362629022207e-07	UP
RBP5	-1.19362905022606	2.4529552064668e-20	1.11961491507749e-19	DOWN
RBPMS2	-3.48575123643083	2.13017273809925e-58	6.36238385737945e-57	DOWN
RCAN1	-1.31416393633854	1.29171492894074e-44	1.99934507179068e-43	DOWN
RCAN2	-1.97296525117677	3.92557012394326e-51	8.4390064899042e-50	DOWN
RCC1	1.05017546171091	2.15797754073684e-37	2.3752757848831e-36	UP
RCL1	1.10466190698936	4.33300855818407e-64	1.69933959512746e-62	UP
RCN1	1.43292665385224	3.06668864951158e-55	8.04220624306402e-54	UP
RCOR2	1.48964183115744	7.91652367663846e-22	3.90179526694893e-21	UP
RCSD1	-1.78129264228801	1.67582609994782e-42	2.35331934503156e-41	DOWN
RD3	-1.25948359220762	0.000142762779752548	0.000233881671260807	DOWN
RDH12	1.48053336265549	7.22532067313285e-13	2.14152355542574e-12	UP
RDH16	2.26207459543081	4.62072103959664e-27	3.02825048786211e-26	UP
RDH5	-1.99746695838835	6.07685617111684e-53	1.41465637042323e-51	DOWN
RDH8	1.49612126360029	0.00041779640624343	0.000656952214368507	UP
RDM1	1.17814158282687	3.51223821569004e-17	1.33972845673189e-16	UP
RECK	-1.49093283830365	3.78571530353297e-34	3.53083345369146e-33	DOWN
RECQL4	2.35281295229511	8.39592459733502e-66	3.6098329632937e-64	UP
REEP1	-1.46340174539339	3.83995308844265e-09	8.93321351089538e-09	DOWN
REEP2	-2.38470621413255	5.26873484414213e-35	5.15998199330972e-34	DOWN
REEP6	1.94234625854305	9.68803504151877e-30	7.23093674144733e-29	UP
REG1A	2.75626564969933	2.23524477393551e-10	5.65156341636982e-10	UP
REG1B	2.65288910009625	2.63270271712652e-08	5.7934098841557e-08	UP
REG3A	5.40916485868399	1.47132779341701e-33	1.33926977871251e-32	UP
REG3G	2.34563147558416	5.98221701743709e-05	0.000101212927443288	UP
REG4	2.34350783696291	5.14858767552871e-10	1.27238656250329e-09	UP
RELL1	-1.48225637436894	3.95766483811043e-40	5.02294590568636e-39	DOWN
RELL2	2.18828527396622	1.59010556553864e-41	2.14639598548251e-40	UP
RELN	-2.8025907845556	1.06674319416359e-28	7.52032357893548e-28	DOWN
RELT	1.18728054633953	5.44572664892604e-27	3.55022231889738e-26	UP
REM2	1.71011456548523	1.20946503646748e-29	8.95425794218037e-29	UP
REN	2.39163255989726	7.03544936391182e-14	2.20934679483853e-13	UP
REP15	-2.08622916965571	3.2248218397161e-13	9.77268059432239e-13	DOWN
RERG	-2.40535889049851	1.77509623258377e-33	1.61072176643987e-32	DOWN
RERGL	-5.31922637619006	4.50971800486993e-55	1.1738074681435e-53	DOWN
RETN	1.91685670299903	1.5425290076227e-09	3.68956835298545e-09	UP
RETNLB	-1.06797777229635	0.00124581891343786	0.00187416369241413	DOWN
RETREG1	-1.46109266716145	1.04051536670576e-24	6.01743410177598e-24	DOWN
RETSAT	-1.86511931254468	1.28416324334044e-119	3.92300606250651e-117	DOWN
REX1BD	1.27204426673272	3.59300850819037e-20	1.62548862439904e-19	UP
RFC3	1.76091383640961	3.71467156429001e-73	2.23046813617179e-71	UP
RFC4	1.1656970585801	4.63010467058172e-50	9.40770275715746e-49	UP
RFPL2	1.58334545309209	7.43895364951735e-06	1.36452649214206e-05	UP
RFX6	-1.21619719087923	0.00364606100003323	0.00522800232160562	DOWN
RFX8	2.71604585756767	7.81287868310673e-32	6.50936155545156e-31	UP
RGL1	-1.34485224221075	2.5158522371758e-29	1.83606601030772e-28	DOWN
RGL3	1.28784104699566	3.51703150363619e-08	7.6696392702338e-08	UP
RGL4	-1.04981076812612	3.87996397438896e-13	1.17111826462186e-12	DOWN
RGMA	-2.43796674503942	3.17573755264004e-31	2.5459999081087e-30	DOWN
RGR	3.56072599269773	1.38723624706561e-14	4.53377341782159e-14	UP
RGS10	-1.03093204304002	7.16514648074029e-23	3.74674761769161e-22	DOWN
RGS13	-2.67459907609409	8.93209120032032e-22	4.38990979596889e-21	DOWN
RGS16	2.0466864043952	3.32937110956808e-48	6.15438844277166e-47	UP

GeneSymbol	log2FoldChange	pvalue	padj	change
RGS18	-1.53989032912343	2.13665852009263e-17	8.24592970088053e-17	DOWN
RGS2	-1.97729692003043	7.86781906431955e-32	6.55096669667752e-31	DOWN
RGS20	1.73450692902417	2.49357609615263e-10	6.29011162716294e-10	UP
RGS22	-2.05731803157696	5.28456698502584e-16	1.87528357265651e-15	DOWN
RGS5	-1.39184589835771	4.40887082716474e-19	1.87614046220478e-18	DOWN
RGS9	-1.81053207314283	7.3749322950206e-65	3.03592662064288e-63	DOWN
RHBDF2	1.5434502768489	1.56305958282032e-59	4.95766056751371e-58	UP
RHBDL1	1.72880808874698	4.5529185927746e-22	2.27358679254328e-21	UP
RHBDL2	-1.73683539295704	8.54407270160319e-21	3.99725787084944e-20	DOWN
RHBG	3.32779443402279	9.35590590817538e-19	3.8928169552941e-18	UP
RHCG	2.50908184353788	9.0699327390004e-21	4.23644685579973e-20	UP
RHEBL1	1.69919691518603	2.9165150208202e-50	6.02784178202901e-49	UP
RHEX	-2.86421020123215	3.35649304096177e-37	3.65063168783681e-36	DOWN
RHOD	1.11768505760711	1.22690109923582e-11	3.37605098836737e-11	UP
RHOH	-1.20273450056691	7.20260917923626e-13	2.13515549264625e-12	DOWN
RHOJ	-1.10100657668842	6.32737309496987e-16	2.23259468495867e-15	DOWN
RHOU	-1.51026598521882	4.08128810816768e-47	7.19306374772507e-46	DOWN
RHPN1	3.37553208097603	2.09255186741893e-109	5.20537223819512e-107	UP
RIBC2	1.16148850326568	3.59741237770295e-12	1.02205484961562e-11	UP
RIC3	-3.16160420320762	2.64860052440609e-33	2.38100572697384e-32	DOWN
RIIAD1	1.73231437076846	1.4404129801339e-09	3.45480870841206e-09	UP
RILP	-1.38898449927178	3.18908054541243e-32	2.71282166767302e-31	DOWN
RIMKLA	-1.56596905099985	5.22317728338189e-36	5.37219055141931e-35	DOWN
RIMKLB	-1.42494988336913	4.19007109855044e-17	1.58957969584006e-16	DOWN
RIMS1	-1.22273665294246	0.000124085101759011	0.000204301614686995	DOWN
RIMS3	-1.44404402619207	6.76494177496414e-16	2.38409089511133e-15	DOWN
RIMS4	-3.40323147356424	2.6535658566603e-19	1.14316037263795e-18	DOWN
RIN1	1.22242638135156	2.16884987538169e-31	1.75738403350495e-30	UP
RIOK3	-1.58211159882654	1.47724614953212e-63	5.61643825366875e-62	DOWN
RIPK2	1.51574336207909	3.05277560756623e-80	2.46101766919216e-78	UP
RIPOR2	-1.4892442282417	2.38535406130158e-18	9.71833651601415e-18	DOWN
RIPPLY1	1.89771026887637	1.06608506114825e-10	2.75999690302921e-10	UP
RIPPLY3	3.75018591687091	2.55176037384684e-44	3.87729523471161e-43	UP
RLN2	1.37089867008652	4.51728245543045e-10	1.12130348391177e-09	UP
RMDN2	-1.97749183594482	1.33547851534621e-76	9.45312495435917e-75	DOWN
RNASE1	-1.19945980784601	1.17807458173334e-16	4.34891089500162e-16	DOWN
RNASE7	1.18974174545774	2.90234364884942e-05	5.06600941834553e-05	UP
RNASEH2A	1.91439844777219	5.97092125159434e-21	2.80928537568258e-20	UP
RNASEL	-1.31126480783061	8.74310689021438e-47	1.51939840598107e-45	DOWN
RND2	-2.30140444961105	1.27999506211868e-30	9.91042864236218e-30	DOWN
RNF11	-1.03874850750404	4.11063044657019e-44	6.18120966892285e-43	DOWN
RNF112	-2.20820637520657	2.26485930080822e-31	1.83262058573297e-30	DOWN
RNF125	-1.52695262666754	2.28731424445624e-23	1.23004950397519e-22	DOWN
RNF150	-3.53200930578545	2.32809973253967e-58	6.94164394567009e-57	DOWN
RNF151	1.92709076427525	1.97850307869546e-08	4.38651312825619e-08	UP
RNF152	-3.06898551117852	9.28652220485508e-92	1.29364968922513e-89	DOWN
RNF165	-1.25270651895821	9.34245915428812e-07	1.84027422232601e-06	DOWN
RNF180	-1.90127361184419	1.67373972515304e-25	1.00847162055675e-24	DOWN
RNF182	3.51610524884871	5.29917719843528e-24	2.9537315158884e-23	UP
RNF183	3.36382957096054	3.85565995972053e-76	2.65369987662504e-74	UP
RNF208	1.19491553753948	6.9015054932989e-16	2.43025106480918e-15	UP
RNF224	2.16092458384353	4.91749785816088e-21	2.32370122670707e-20	UP
RNF225	3.32286894821451	5.66549227073952e-13	1.69303615772073e-12	UP
RNF32	1.34293339822471	6.97542139385872e-37	7.47464693730842e-36	UP
RNF39	1.33354592318051	5.07970288319787e-19	2.15370991733929e-18	UP
RNF43	2.46429400312209	5.52736059724827e-71	2.99837788410854e-69	UP
RNFT2	1.95881302847604	9.31163595954932e-49	1.77206029468451e-47	UP
ROBO2	1.87238003155359	7.60105985972307e-17	2.83359570407531e-16	UP
ROPN1	-3.41161546955586	1.12151230570798e-21	5.48564432002615e-21	DOWN

GeneSymbol	log2FoldChange	pvalue	padj	change
ROR1	-1.92438145751088	1.05703241971225e-21	5.17316063081773e-21	DOWN
ROR2	-1.27936660913451	5.78578764114385e-11	1.52348283978887e-10	DOWN
RORB	-1.92141480255057	9.8740494494139e-14	3.07744447937434e-13	DOWN
ROS1	-1.1706801299929	0.000328413647701306	0.000520968101250144	DOWN
RP1	5.02491792480808	4.02240768367966e-50	8.23057403007214e-49	UP
RP1L1	2.18599626282348	3.89831360229013e-18	1.57023675125325e-17	UP
RPA4	1.32301088300315	7.74807572481054e-10	1.89330960701833e-09	UP
RPEL1	1.20078769100643	5.04641595500137e-06	9.37714662516688e-06	UP
RPGRIP1L	1.19302862100919	3.6790840373624e-60	1.20420846508631e-58	UP
RPH3A	-2.67564629288964	3.44189020725402e-30	2.61718926545477e-29	DOWN
RPL10L	-2.03483825200829	3.48913245435561e-17	1.33120647299943e-16	DOWN
RPL22L1	1.88030112520574	4.71090171579069e-28	3.22321931540524e-27	UP
RPL26	1.28121959862689	1.79112201295223e-16	6.54814352541198e-16	UP
RPL36A	1.73251576520389	1.14187392155132e-48	2.16595322396221e-47	UP
RPL8	1.03309382111839	7.38243033611038e-18	2.9262522067537e-17	UP
RPN2	1.01555429780793	2.602877787787e-46	4.39611163130311e-45	UP
RPP21	1.49998384894231	3.70001928673837e-22	1.8594065177482e-21	UP
RPP40	1.34182304410979	2.61558495316083e-48	4.86074501487614e-47	UP
RPRM	-2.28550608051881	6.06511407866153e-15	2.03060625748382e-14	DOWN
RPRML	1.85977432770879	0.000271699712962397	0.000434156628145017	UP
RPS10	3.6782597519723	8.22470675059846e-57	2.33252147635457e-55	UP
RPS15	1.0394024169899	7.85122495294736e-18	3.10853524569514e-17	UP
RPS17	1.53474336019525	6.53164028534218e-41	8.5773342600802e-40	UP
RPS2	1.1547222135645	6.66533323585734e-26	4.12890244169277e-25	UP
RPS21	1.42711587278499	5.35691444944003e-21	2.52517464288303e-20	UP
RPS27AP5	1.88122487159386	2.9008376988326e-18	1.17388535556059e-17	UP
RPS6KA5	-1.23862658942664	7.30085757538121e-32	6.09151092286119e-31	DOWN
RPS6KA6	-1.27069036600923	1.1680788079429e-08	2.62753601378499e-08	DOWN
RPS6KL1	1.46562748974147	2.22261728499146e-44	3.40091694055856e-43	UP
RPTN	3.26733270103127	5.89949075097909e-05	9.98715073369618e-05	UP
RPUSD1	1.11936031259253	1.57334025447607e-25	9.51269925388605e-25	UP
RRM2	1.36384819526265	1.38770895135157e-45	2.26468378349436e-44	UP
RRP1	1.20725783858462	4.60920952257029e-48	8.46626217473802e-47	UP
RRP12	1.39113891805056	2.95126624853883e-75	1.95217951041377e-73	UP
RRP9	1.46800055216324	1.89912794477546e-63	7.17343056450652e-62	UP
RRS1	1.44004352496249	3.98066596613724e-51	8.54689722174449e-50	UP
RSAD2	-1.60961008310103	2.10604913681328e-15	7.21618134973037e-15	DOWN
RSPH14	1.60387989446702	1.74583810886851e-18	7.16480768082191e-18	UP
RSPO1	-2.09265642471635	3.36041177996204e-16	1.20574593703851e-15	DOWN
RSPO2	-4.16040781794282	7.7599053079924e-44	1.15786830443935e-42	DOWN
RSPO3	-1.37142079176069	2.65011256534642e-08	5.82951112940592e-08	DOWN
RSPO4	2.42876285530009	5.07611908162419e-09	1.17290952187264e-08	UP
RTEL1	1.38772835304021	6.15139832028572e-25	3.60169128954725e-24	UP
RTEL1-TNFRSF6B	2.07243157634967	4.52944466029326e-35	4.44846135756833e-34	UP
RTKN	1.23460221579708	1.42491983056469e-73	8.73666514423341e-72	UP
RTL1	1.23417990383771	0.0147139405586661	0.0197072415158874	UP
RTL10	1.10231419775219	2.58860331741519e-38	2.97330801887537e-37	UP
RTL3	-1.6542168910566	1.13665766103562e-09	2.74516225403789e-09	DOWN
RTL5	-1.74093867404999	1.07782188799938e-26	6.91529570218613e-26	DOWN
RTN1	-1.78524084753711	6.02286394804016e-23	3.16368416070055e-22	DOWN
RTN4R	1.5573830008918	1.23040238994519e-24	7.09202145518559e-24	UP
RTN4RL2	1.97307126697864	9.72390079167985e-23	5.0543965518066e-22	UP
RUBCNL	1.69055389808362	3.45257349974065e-18	1.39424077808404e-17	UP
RUNDC3B	-2.53796524763143	6.98767748858038e-47	1.221650884625e-45	DOWN
RUNX1	1.35186589771689	2.51759400832226e-50	5.22513283276704e-49	UP
RUNX1T1	-1.69294076493116	5.69814495213138e-24	3.1690130326242e-23	DOWN
RUVBL1	1.3683654970499	7.59669830293038e-95	1.27193564950891e-92	UP
RUVBL2	1.07295343917879	1.72161892154724e-24	9.85488174914599e-24	UP
RXFP3	2.80824330333109	6.80905181539306e-14	2.14095376058937e-13	UP

GeneSymbol	log2FoldChange	pvalue	padj	change
RXRG	-4.04302883627361	3.62808722487373e-73	2.18601670749918e-71	DOWN
RYR1	-1.08152056868631	4.00780858149534e-08	8.71043070763584e-08	DOWN
RYR3	-2.26950031039692	6.90606703411291e-63	2.54778273866543e-61	DOWN
S100A1	1.45925052146438	6.74731777485924e-15	2.25337637924097e-14	UP
S100A11	1.88893588628368	1.8358753462929e-79	1.45973047511408e-77	UP
S100A2	4.10360260560755	2.95971625078525e-75	1.95217951041377e-73	UP
S100A3	1.87725752006872	5.19520439709046e-20	2.32914763559568e-19	UP
S100A5	1.69922679602962	3.94761242080896e-16	1.41091492371811e-15	UP
S100A7	4.25289075538374	1.93972954139848e-15	6.66169351694448e-15	UP
S100A9	1.01214166384179	3.08016894739926e-06	5.82545692202273e-06	UP
S100B	-2.52815409086703	1.21542686614298e-47	2.18638719216402e-46	DOWN
S100G	1.83118668392956	0.000830063569882568	0.00127011396681592	UP
S100P	2.30642064757885	5.69168120872941e-39	6.75131095964613e-38	UP
S100Z	-1.06765955664362	1.98892712921251e-06	3.81927526477476e-06	DOWN
S1PR1	-1.14180349178867	2.43017115589609e-18	9.89398417994355e-18	DOWN
S1PR5	1.32818993306549	5.31426548231781e-21	2.50574884493907e-20	UP
SAA1	1.44572284350102	8.51464605255346e-07	1.68311422083225e-06	UP
SAA2	3.03175296445004	8.81681267149612e-24	4.85231223289387e-23	UP
SAA4	3.26775375258311	2.69423337937489e-10	6.77566231008881e-10	UP
SAC3D1	1.04053461730414	1.66752241066508e-14	5.41727010016996e-14	UP
SAG	-1.2750963219784	1.14091272277949e-08	2.56841800152026e-08	DOWN
SAGE1	3.56222184421977	8.50109375049078e-06	1.55118459056163e-05	UP
SALL2	-2.27850079839983	5.21751992633392e-38	5.89187253419278e-37	DOWN
SALL3	2.94730191467235	0.000840322261646759	0.0012846823127353	UP
SALL4	5.01618866480356	5.07618861147692e-146	3.04798869971199e-143	UP
SAMD10	1.56090334855637	1.20345180121744e-41	1.63716454801556e-40	UP
SAMD13	-1.10791391415068	9.24998522185776e-14	2.88707640559615e-13	DOWN
SAMD4A	-1.30920913836139	1.84812907602066e-23	1.00066764927698e-22	DOWN
SAMD9	-1.47670675481863	3.91651059958293e-18	1.57683697272919e-17	DOWN
SAPCD1	2.25245875749637	1.64736747335084e-31	1.34358828166081e-30	UP
SAPCD2	2.22399138233406	6.49448433269228e-72	3.64801469952164e-70	UP
SARNP	1.23197073560694	2.5881973036771e-07	5.30215054693286e-07	UP
SARS2	1.02286129461027	4.74392392821454e-19	2.01625451213082e-18	UP
SASH3	-1.20577118124059	9.10308466632426e-15	3.01582978110168e-14	DOWN
SBK1	1.87653834866101	1.65153015888051e-18	6.78897418710727e-18	UP
SBK2	1.30749377297337	0.00998364393438826	0.0136456194528652	UP
SBSN	3.51456036127339	1.76327867251982e-21	8.53599430764184e-21	UP
SBSPON	1.89614503638427	1.22457880619458e-12	3.57958548804201e-12	UP
SCAMP2	-1.00356369192613	5.09662943610691e-48	9.30268431561108e-47	DOWN
SCARA3	-1.59585220089033	1.5229449557334e-18	6.26927671730159e-18	DOWN
SCARA5	-4.71946802733981	6.07821570565979e-81	5.01611232619213e-79	DOWN
SCARB1	1.05981803484222	1.10536614766001e-22	5.72678986289908e-22	UP
SCARF2	1.2643718169747	4.4166535643398e-12	1.24829067547231e-11	UP
SCART1	1.15071539859451	2.62072144673951e-12	7.51805972851319e-12	UP
SCD	2.27764483679501	4.24617492481107e-62	1.52765793317635e-60	UP
SCG2	-2.36609436153798	3.54945418033272e-27	2.33761897284923e-26	DOWN
SCG3	-2.57693573110318	1.01968378225095e-15	3.55684168676599e-15	DOWN
SCGB2A1	-2.77386886020499	1.10096875317196e-22	5.70570502945934e-22	DOWN
SCGB3A2	1.01612350626896	0.026830415955028	0.034792823430511	UP
SCGN	-4.9727281483956	7.96528157341451e-71	4.26767532424206e-69	DOWN
SCHIP1	-1.17528919661338	5.5605485463395e-13	1.66224604012721e-12	DOWN
SCIMP	-1.28873702137736	3.86911871885825e-14	1.23393707420291e-13	DOWN
SCIN	-2.61598976536658	1.36211508147081e-40	1.76477008286096e-39	DOWN
SCLY	1.7611393028979	1.0948170077873e-60	3.65211658172419e-59	UP
SCML1	1.47839689167217	3.85164731189857e-36	3.98743963389357e-35	UP
SCN11A	-3.34996166105241	7.04493942746562e-57	2.00446944853691e-55	DOWN
SCN1B	-1.12529464761196	1.07556361198335e-14	3.54376332553756e-14	DOWN
SCN2B	-3.82747875138873	1.65823392257839e-44	2.55901488657433e-43	DOWN
SCN3A	-2.31305966940335	6.97581259871735e-35	6.78602373080812e-34	DOWN

GeneSymbol	log2FoldChange	pvalue	padj	change
SCN3B	-1.98770518152647	2.57715259737835e-18	1.04801396959713e-17	DOWN
SCN4A	-2.03784472090277	6.02449876689926e-17	2.26282564771391e-16	DOWN
SCN4B	-2.23879385283818	7.69480621841788e-46	1.27004417707403e-44	DOWN
SCN7A	-5.25418888608095	8.26949099212736e-56	2.23945018111841e-54	DOWN
SCN9A	-3.60370858403952	3.18740075762763e-62	1.1514981201778e-60	DOWN
SCNN1B	-3.94429812648998	2.41710054071895e-43	3.52208968330871e-42	DOWN
SCNN1G	-4.25603450237358	1.25510608844502e-31	1.0328526615356e-30	DOWN
SCO2	1.7544041831174	1.21107408719331e-11	3.33572177796538e-11	UP
SCP2	-1.16528064410759	4.61812737475926e-50	9.39432850194895e-49	DOWN
SCRG1	-3.39012597119165	1.40067324964224e-26	8.93076649433184e-26	DOWN
SCTR	-1.93014159592507	1.53790200117378e-16	5.64648568989098e-16	DOWN
SCUBE1	-1.90537065799219	4.25315370043372e-27	2.79051112982865e-26	DOWN
SCUBE2	-2.49199414626147	8.90464518545782e-43	1.2688755042093e-41	DOWN
SCX	1.34094572052657	3.37781618077289e-14	1.08002043987878e-13	UP
SDCBP2	-2.23693700681957	1.98351490235879e-42	2.77198595463672e-41	DOWN
SDHD	-1.10137150006435	4.58013236944353e-27	3.00278030681928e-26	DOWN
SDK1	-1.28687235395401	5.52569995530205e-12	1.55242035046264e-11	DOWN
SEC14L2	2.13862650499836	3.84238934683774e-72	2.17232226287291e-70	UP
SEC14L4	4.99712217728254	3.79784753409288e-31	3.02940536468893e-30	UP
SEC14L5	-2.42940812197535	8.81889362441975e-32	7.32999497288883e-31	DOWN
SEC14L6	-1.81512717434828	1.76722834865803e-18	7.24917484927734e-18	DOWN
SEC16B	1.11186275130219	6.88691877180213e-11	1.80252392264227e-10	UP
SECISBP2L	-1.16116784006108	2.66919922275194e-34	2.5055938579935e-33	DOWN
SECTM1	-2.90415300609692	1.44693786363101e-50	3.02467335166948e-49	DOWN
SELENBP1	-1.90134595813269	1.51315701412268e-22	7.76557709605604e-22	DOWN
SELENOH	1.4557335965454	4.57250761389676e-20	2.05526781313331e-19	UP
SELENOP	-2.98253905404547	1.84038979616189e-51	4.00583844007087e-50	DOWN
SELL	-1.13357701341889	8.20138440670817e-09	1.86827193451085e-08	DOWN
SEMA3D	-2.40431445337452	2.07339531579837e-16	7.55314490250982e-16	DOWN
SEMA3E	-3.765706666415	1.78785036850643e-24	1.02172098676739e-23	DOWN
SEMA3F	1.11428313454313	2.16894011581634e-18	8.86150967543638e-18	UP
SEMA3G	-1.96528982699313	1.14666874951682e-31	9.46300613049115e-31	DOWN
SEMA6A	-2.38923474178504	5.01680708502813e-42	6.88940550249171e-41	DOWN
SEMA6D	-3.05662152083331	1.69934615658182e-69	8.57701873175629e-68	DOWN
SEMG1	2.85057062727701	3.64092130687929e-11	9.70447921577975e-11	UP
SEMG2	3.95680287133338	2.58321154069843e-08	5.6859388899231e-08	UP
SENP3-EIF4A1	1.4637385891668	1.56503460277194e-09	3.74237126312383e-09	UP
SEPTIN5	1.17843791923645	5.23822603842415e-05	8.91973694573438e-05	UP
SEPTIN6	-1.27103236667162	2.49619214335748e-32	2.13488181690981e-31	DOWN
SERF1B	-1.0452483408823	2.01614937249309e-19	8.74401221001797e-19	DOWN
SERPINA10	2.42715154114803	1.35972436169508e-12	3.96531239494163e-12	UP
SERPINA4	6.88091953383165	1.21226190074966e-63	4.64958512725857e-62	UP
SERPINA7	3.32586934469621	1.21910102980038e-16	4.49654866170601e-16	UP
SERPINA9	-3.92099700195298	4.15889368247955e-13	1.25270395594216e-12	DOWN
SERPINB2	3.60696597230365	2.39843498063817e-18	9.76934463575495e-18	UP
SERPINB3	4.37092812431083	7.836509223805e-10	1.9143818057536e-09	UP
SERPINB4	4.61612013506413	5.44200076593785e-12	1.52989278878392e-11	UP
SERPINB5	3.08475917896657	1.70447394760921e-36	1.79443801993465e-35	UP
SERPINB7	4.29356906599757	1.94716457548805e-26	1.23519040994439e-25	UP
SERPIND1	3.61560183587275	7.01134743904877e-27	4.54536831556799e-26	UP
SERPINE1	1.9649156881201	4.22613293065163e-20	1.90252463085411e-19	UP
SERPINE2	1.36128866525129	5.69250176616655e-20	2.54816280859276e-19	UP
SERPINF2	1.59533807595307	1.09047470736213e-17	4.28536133588281e-17	UP
SERPINH1	1.19891651114162	1.54911011292823e-32	1.33736511633214e-31	UP
SERTAD4	-1.81324494115632	2.15359432560517e-21	1.03769901649471e-20	DOWN
SERTM1	-2.54378600329187	7.2327345138176e-06	1.32823883240989e-05	DOWN
SERTM2	-1.43279813147994	8.09319465461743e-06	1.47892537014223e-05	DOWN
SESN2	-1.12111849319471	4.53317406959345e-36	4.67631280058239e-35	DOWN
SETBP1	-2.30898679716767	5.54387591953142e-48	1.00557824361251e-46	DOWN

GeneSymbol	log2FoldChange	pvalue	padj	change
SETD6	1.0187928508877	5.81346807186975e-49	1.11856264680075e-47	UP
SETSIP	1.69760292722894	4.0786625659019e-17	1.54866444090819e-16	UP
SEZ6	-2.29282017381648	2.07680817993042e-14	6.70811738770698e-14	DOWN
SEZ6L	-2.49192058251821	4.08283100710277e-16	1.45685115423526e-15	DOWN
SEZ6L2	1.17367409553678	1.85135415790899e-17	7.17129287224871e-17	UP
SFMBT2	-1.12009085590894	2.1951850390494e-13	6.7285261547205e-13	DOWN
SFRP1	-4.21001610234965	6.67148860363459e-44	9.98029476418291e-43	DOWN
SFRP2	-1.14473720207643	0.000300990584874786	0.000479125062110308	DOWN
SFRP4	3.05417830201922	4.5492935122964e-29	3.26532761457614e-28	UP
SFRP5	-3.65616457251038	4.87992779687522e-20	2.18949195380027e-19	DOWN
SFTA2	7.03926087109042	1.68718538169246e-90	2.20894428957976e-88	UP
SFTPA1	-1.15332422493685	0.0228325516105042	0.02989572307645	DOWN
SFTPA2	-1.19224145997184	2.89411116402764e-05	5.05265266685516e-05	DOWN
SFTPB	1.50920297092468	9.92881151253227e-05	0.000164893080465164	UP
SFTPC	-1.60476048015443	0.0131165492924187	0.017690409116373	DOWN
SFTPD	-1.78554218784639	6.78991090539197e-16	2.39240628481567e-15	DOWN
SGCA	-2.37550108454402	5.20583239006886e-22	2.59071618771846e-21	DOWN
SGCD	-1.07926154916577	1.14363096166665e-08	2.57420449011135e-08	DOWN
SGCE	-1.86580099482255	7.05988705796489e-34	6.5044345682721e-33	DOWN
SGCG	-4.18184663750194	1.04991498195486e-39	1.29660777168652e-38	DOWN
SGCZ	-1.50463545289855	0.000107255265718691	0.000177599461958878	DOWN
SGK1	-2.83749984494092	2.97416496384634e-89	3.69922389396117e-87	DOWN
SGK2	-1.5649200215752	7.86672610951674e-21	3.68432764241568e-20	DOWN
SGO1	1.29435587966627	1.74943332569923e-38	2.02680522291421e-37	UP
SGPP1	-1.01504359311418	2.55689679187155e-22	1.29541006217222e-21	DOWN
SGSM1	-1.52398574817382	9.62643444456195e-18	3.7932813528662e-17	DOWN
SGSM3	1.07623263034198	1.9632697824026e-10	4.98707756688205e-10	UP
SH2D1B	-1.84260046672538	4.39547043073526e-21	2.08380960006515e-20	DOWN
SH2D2A	1.06548743802116	1.14941653423807e-21	5.62055324647219e-21	UP
SH2D4B	1.26201050709356	4.55351415981299e-06	8.49660759374449e-06	UP
SH2D5	3.22596409752232	1.25280088247745e-31	1.03144310953096e-30	UP
SH2D6	-2.29438036188106	1.50187464238709e-14	4.8937393615057e-14	DOWN
SH2D7	-3.63329308702813	6.99248607877056e-54	1.72220877071615e-52	DOWN
SH3BGR	-2.41010735046979	3.70409798385161e-35	3.64403718603435e-34	DOWN
SH3BGRL	-1.2314862814317	2.5729756715909e-26	1.62212981062318e-25	DOWN
SH3BGRL2	-1.07231544170413	5.24539967229343e-34	4.85324891039562e-33	DOWN
SH3GL2	-1.48850872498523	2.64353704021095e-05	4.62585775109972e-05	DOWN
SH3GL3	-2.31624721746422	3.61536884818221e-07	7.32794991891476e-07	DOWN
SH3TC2	3.23063455305188	5.59491278150412e-96	9.55139375140503e-94	UP
SHB	1.26368118684781	1.20220525510439e-40	1.56340553451327e-39	UP
SHC4	-1.1290359940104	6.24771624264505e-11	1.63966063199967e-10	DOWN
SHCBP1	1.34395632169037	7.65086312819272e-54	1.87640112184817e-52	UP
SHE	-1.86649836722061	1.84429499115075e-39	2.24108225268025e-38	DOWN
SHH	2.18575083178568	2.51617996543342e-50	5.22513283276704e-49	UP
SHISA2	3.08257671654902	7.42392192059628e-60	2.39393985932117e-58	UP
SHISA3	-2.40668894760843	4.12021478358146e-21	1.95704582723688e-20	DOWN
SHISA7	-1.42215914285207	4.11340229381703e-11	1.09236959192063e-10	DOWN
SHISAL1	-3.24568455893738	9.47144019739672e-51	1.9991053109972e-49	DOWN
SHLD1	1.0551827734156	2.08516151756374e-16	7.59441905570746e-16	UP
SHMT2	1.50584872186056	5.48238328610237e-56	1.49397089453679e-54	UP
SHOC1	1.1272974927939	0.000133151508193673	0.000218712122646583	UP
SHOX	-1.80584213399954	2.48695277922454e-13	7.59608993942061e-13	DOWN
SHOX2	1.80005442468461	2.99840910312367e-11	8.03745346562385e-11	UP
SHROOM3	-1.00785359457966	1.68998918641967e-31	1.37770513591413e-30	DOWN
SHROOM4	1.95976486183292	4.46273719299768e-45	7.0516917188447e-44	UP
SI	-3.12995751651609	1.06504720404309e-19	4.68324923333391e-19	DOWN
SIAE	-1.59241596069794	6.29676130011848e-51	1.33714029901174e-49	DOWN
SIAH3	-1.06563790960387	4.6801824627597e-05	8.00471635635347e-05	DOWN
SIDT1	-1.03237563157599	1.87837966547572e-11	5.09791538574326e-11	DOWN

GeneSymbol	log2FoldChange	pvalue	padj	change
SIGLEC1	-1.74401563661935	2.0994990425628e-18	8.58585646504133e-18	DOWN
SIGLEC11	-2.1182618522184	3.13078044680853e-29	2.27340616848528e-28	DOWN
SIGLEC15	2.45690678289084	6.64234318262999e-24	3.68119420239135e-23	UP
SIGLEC6	-2.26430410521852	2.72785596843177e-28	1.8834320372047e-27	DOWN
SIGLEC8	-2.33992825805385	1.21517061162819e-28	8.54249731945162e-28	DOWN
SIGMAR1	1.28697834336294	1.66582727229289e-38	1.93122838165354e-37	UP
SIM2	7.84964523173748	4.35751695861449e-254	1.51754885600708e-250	UP
SINHCAF	1.03544976993532	9.73100921371018e-26	5.95592490117172e-25	UP
SIPA1L2	-1.32269322470582	3.16360504581609e-30	2.40873872596395e-29	DOWN
SIX1	3.61807916482086	1.83753631211153e-41	2.47081233998441e-40	UP
SIX2	2.99530767565267	8.82178927086673e-20	3.89684973550488e-19	UP
SIX3	3.60471888274222	3.51213374083436e-14	1.12214284090181e-13	UP
SIX4	2.45930204163113	9.31286522507872e-28	6.30501252582798e-27	UP
SKA1	1.09165732737177	5.06921091679507e-22	2.52416842133693e-21	UP
SKA3	2.05189044659713	6.02308304004083e-81	4.99428309410624e-79	UP
SKAP1	1.32149631982518	1.47141894842139e-15	5.08672188780259e-15	UP
SKIDA1	-2.07307569617314	3.08186831372457e-35	3.04393493742972e-34	DOWN
SLA2	-1.09122557477335	1.47165800577061e-12	4.28384835414305e-12	DOWN
SLAMF1	-1.28742483678993	6.06315281575821e-17	2.276362224683e-16	DOWN
SLAMF7	-1.83104115524915	1.60934499811629e-20	7.40579398842467e-20	DOWN
SLAMF9	3.60189290077071	3.8157594002544e-37	4.14496683946537e-36	UP
SLC10A1	-1.29239943371683	1.7329204362987e-12	5.02419972648506e-12	DOWN
SLC10A2	-4.96132599221218	5.4832160451189e-13	1.63997322215142e-12	DOWN
SLC10A4	-1.77836852005334	1.6798661316487e-14	5.45635309651161e-14	DOWN
SLC10A5	-1.0392426276018	3.12608219861768e-13	9.47840315593411e-13	DOWN
SLC11A1	2.34022499493324	1.74294583789558e-34	1.65846534837573e-33	UP
SLC11A2	1.10798830898315	9.98476358789617e-28	6.74155441473579e-27	UP
SLC12A2	1.40400265149107	6.54746001504825e-33	5.78148687839935e-32	UP
SLC12A8	1.13511751331267	8.78370119571426e-55	2.2525859929451e-53	UP
SLC13A1	-3.79420477193425	1.12051966943272e-10	2.89704662269219e-10	DOWN
SLC13A2	-2.15055352879873	9.84532700609407e-12	2.72814575361419e-11	DOWN
SLC13A3	4.21716778057519	2.50698226029616e-45	4.02712934488348e-44	UP
SLC13A5	1.4662410754774	9.27360903343254e-07	1.82774594339741e-06	UP
SLC14A1	1.08702422131692	0.00150130174842092	0.00223992522879389	UP
SLC14A2	-2.53779442136363	4.90329806008892e-11	1.29719126588162e-10	DOWN
SLC15A1	-2.63518006649555	6.04709593613204e-18	2.40846481097592e-17	DOWN
SLC15A2	-1.15281180971487	2.06160879120776e-23	1.11004310084418e-22	DOWN
SLC16A12	-2.94518945912623	2.83549556837135e-52	6.44575513473243e-51	DOWN
SLC16A7	-1.08868791559043	3.93285523866119e-06	7.3796129602163e-06	DOWN
SLC16A8	1.23605878986897	1.00539881505549e-09	2.43524962672989e-09	UP
SLC16A9	-2.38361973667786	3.0395059575318e-34	2.84706386436263e-33	DOWN
SLC17A1	-1.91224915876761	5.63594299499326e-11	1.48604899109354e-10	DOWN
SLC17A4	-3.3736554654471	1.13965904838511e-53	2.77163170524162e-52	DOWN
SLC17A7	-3.0753327260895	2.42780850158101e-67	1.13035907588316e-65	DOWN
SLC17A8	-5.56036748814879	3.39196722406137e-53	7.98166557737577e-52	DOWN
SLC17A9	2.38820891369803	9.23225686012689e-42	1.25889811045724e-40	UP
SLC18A2	-1.27650292822549	1.84081262100433e-19	7.99951838521297e-19	DOWN
SLC18A3	-2.79725376561134	1.27956998658711e-09	3.07750720669078e-09	DOWN
SLC1A1	-1.88621196811898	2.6595959645832e-29	1.93691110544907e-28	DOWN
SLC1A2	-1.27751201775599	2.0327612574362e-10	5.15382524399192e-10	DOWN
SLC1A5	1.2252473689221	1.17841776331224e-32	1.02701644206987e-31	UP
SLC1A7	1.36489900144095	1.04868475384388e-06	2.05685375294926e-06	UP
SLC20A1	-1.51687417459926	1.37385859342432e-64	5.51221190951559e-63	DOWN
SLC22A11	4.68557384252966	1.03313950357689e-51	2.26290039946973e-50	UP
SLC22A12	2.80241679909657	1.38738795122869e-07	2.90193229966908e-07	UP
SLC22A15	1.14658118022062	4.69382613166788e-16	1.6700775322994e-15	UP
SLC22A17	-1.75983839105745	5.11072967816372e-30	3.86590512101933e-29	DOWN
SLC22A18AS	-2.02030320667238	3.04685730172856e-46	5.12607982560381e-45	DOWN
SLC22A2	1.16506899256899	1.80342075866751e-05	3.20242358460916e-05	UP

GeneSymbol	log2FoldChange	pvalue	padj	change
SLC22A23	-1.45263673534874	4.70054409230569e-42	6.46529022743436e-41	DOWN
SLC22A3	1.93870777047741	2.88257416076934e-43	4.17937251136358e-42	UP
SLC22A31	2.12806539752533	1.79842343178295e-10	4.5776856040983e-10	UP
SLC22A4	-1.54607591881447	1.37798363337923e-32	1.1937725874643e-31	DOWN
SLC22A5	-1.87491876947353	1.954708856467e-130	7.91566170178136e-128	DOWN
SLC23A1	-2.81903843452323	2.3146719911688e-41	3.09803100555129e-40	DOWN
SLC23A3	-1.86775284479395	1.18906535670045e-33	1.08745772354122e-32	DOWN
SLC24A2	1.40204504041647	2.22465576562953e-07	4.5784104534815e-07	UP
SLC24A4	-1.46785649735945	3.51795184960245e-22	1.76995364221692e-21	DOWN
SLC25A10	1.0363447684829	1.90977513915622e-13	5.87127727721172e-13	UP
SLC25A15	1.02774189822378	3.49022715981354e-34	3.26222896048487e-33	UP
SLC25A2	1.30281122680706	7.50640412578959e-06	1.3764639326282e-05	UP
SLC25A20	-1.50860482099172	3.67196217837896e-68	1.75659003879431e-66	DOWN
SLC25A22	1.11693674604663	1.87997092192135e-28	1.30734559358692e-27	UP
SLC25A23	-1.75333092560446	9.63648088003849e-85	9.81286792772574e-83	DOWN
SLC25A27	1.22508953382169	3.30337940132025e-15	1.12062625200057e-14	UP
SLC25A29	1.40142379908817	3.58229420316279e-30	2.72276250369592e-29	UP
SLC25A32	1.27615804485455	1.49885729496684e-10	3.8421319118589e-10	UP
SLC25A34	-3.86788195734878	2.82070551403928e-125	9.82338902319321e-123	DOWN
SLC25A42	-1.13820453154888	1.54276684496618e-34	1.47362584044959e-33	DOWN
SLC26A2	-4.12040219773319	1.02938728151616e-70	5.48156597340701e-69	DOWN
SLC26A3	-4.57461534390194	2.55564826109827e-48	4.75443409941285e-47	DOWN
SLC26A4	-1.01473644666926	5.14879360670744e-09	1.18907086304505e-08	DOWN
SLC26A7	-1.36698391906192	5.70806710719596e-10	1.40606270388461e-09	DOWN
SLC26A9	5.45058505374737	4.25209846914788e-34	3.95100270241579e-33	UP
SLC27A5	1.77329536602437	9.65410868593081e-22	4.73540829712995e-21	UP
SLC27A6	-2.90552910643796	3.53571679789118e-21	1.68631708029798e-20	DOWN
SLC28A2	-1.88070636709156	9.91297197105464e-08	2.09305906307717e-07	DOWN
SLC28A3	2.2300910842863	5.19390074743152e-20	2.32914763559568e-19	UP
SLC29A1	1.03220070994405	2.93640607585617e-24	1.66119684856671e-23	UP
SLC2A1	1.78311279256258	1.27514098641949e-28	8.95684953470048e-28	UP
SLC2A12	1.66640748757965	3.98138534900123e-16	1.42240178666718e-15	UP
SLC2A13	-1.79304108379557	1.3455603246846e-63	5.14950372169955e-62	DOWN
SLC2A2	1.23272357899617	0.0070048998603841	0.00974251767323229	UP
SLC2A4	-2.73895228418817	7.14531738658817e-60	2.3083749842794e-58	DOWN
SLC2A5	-1.58060982482406	6.14025644084778e-17	2.30381998285892e-16	DOWN
SLC2A7	-1.31356069197097	8.99237544220602e-08	1.90144788797976e-07	DOWN
SLC2A8	1.0298676060654	1.31815331461741e-15	4.57140084991695e-15	UP
SLC30A10	-5.65385881200043	2.91258019025248e-62	1.05440247095356e-60	DOWN
SLC30A2	2.3834969877052	1.92869514265366e-14	6.24360820208742e-14	UP
SLC30A4	-1.58531347334432	8.83083580087559e-59	2.67894327178827e-57	DOWN
SLC30A8	-2.88194335638688	8.9088703686961e-23	4.63905980054142e-22	DOWN
SLC34A1	1.26698257826075	8.37478147484434e-06	1.52861708408244e-05	UP
SLC34A2	1.00185933197226	0.00633830396550292	0.00885714524928195	UP
SLC34A3	2.78295773287677	1.39615351678923e-26	8.90520922631898e-26	UP
SLC35D1	-1.34890100103191	2.91820514687329e-50	6.02784178202901e-49	DOWN
SLC35D3	4.34549945281536	1.43141884714927e-48	2.69754289885392e-47	UP
SLC35E4	1.50200282115093	1.60162405712177e-28	1.11869553576661e-27	UP
SLC35F1	-2.27365500768534	1.56175795166547e-40	2.01592966733513e-39	DOWN
SLC35G1	-1.42521844586731	2.58093179573797e-53	6.13958543158269e-52	DOWN
SLC36A1	-2.01564021825383	1.79764075694882e-121	5.90609783033015e-119	DOWN
SLC37A2	-1.97663569986308	2.9035491698505e-20	1.31940244505759e-19	DOWN
SLC38A3	2.88589648967216	4.55936429934669e-18	1.83100116569474e-17	UP
SLC38A4	-1.80226263273622	1.50482452193634e-11	4.11099927839308e-11	DOWN
SLC38A5	2.08489906785762	1.62880073328151e-25	9.82414519176688e-25	UP
SLC38A8	2.08385381963945	2.0402494813993e-05	3.60569006592977e-05	UP
SLC39A1	1.10787395110178	1.12333901764764e-20	5.22175715811486e-20	UP
SLC39A10	1.84982310164347	1.34991604983786e-90	1.78076425574445e-88	UP
SLC39A2	1.30853025627573	0.00098672968078877	0.00149877215034672	UP

GeneSymbol	log2FoldChange	pvalue	padj	change
SLC39A5	-1.17943312166013	1.6218157351004e-10	4.14451795545806e-10	DOWN
SLC3A2	1.14937912834616	8.0898521343699e-47	1.41150897009803e-45	UP
SLC41A2	-1.66030439474566	2.20318640106896e-44	3.37712014100474e-43	DOWN
SLC43A1	1.17571906709976	1.01253233291104e-21	4.95955710632347e-21	UP
SLC44A4	-1.46384645711181	4.34881952624181e-33	3.85570236305746e-32	DOWN
SLC46A1	-1.02159777574728	3.67349965413666e-17	1.40001421487156e-16	DOWN
SLC46A3	-1.36458152906981	1.03520862771206e-29	7.70345633946588e-29	DOWN
SLC47A1	-1.25000212159973	1.65399519625434e-12	4.80096988704399e-12	DOWN
SLC47A2	-1.07793651181961	3.25451241162583e-13	9.85750993627422e-13	DOWN
SLC49A3	-1.08073138849887	2.04005104798785e-12	5.88915930016785e-12	DOWN
SLC4A1	-1.58828122723445	2.16229860725554e-06	4.1398686803893e-06	DOWN
SLC4A10	-3.07105725886142	3.52934892584756e-23	1.87768264117885e-22	DOWN
SLC4A11	4.10038924556349	1.04973649897646e-82	9.77489928164549e-81	UP
SLC4A4	-4.64078492065186	7.94737154839253e-65	3.26385803707923e-63	DOWN
SLC4A8	1.33095826667216	3.69080250530156e-16	1.32184171174036e-15	UP
SLC51A	-3.87196040013496	1.10356275961461e-125	3.92170170064678e-123	DOWN
SLC51B	-4.02405666089231	1.01439758208737e-83	1.00361960777769e-81	DOWN
SLC52A1	-2.55249352735537	3.9143538141241e-29	2.82706938885703e-28	DOWN
SLC52A2	1.32994912156845	2.12577111590394e-30	1.63210107765588e-29	UP
SLC5A10	-1.1905053914049	1.77138499707248e-13	5.45834842576944e-13	DOWN
SLC5A11	-3.53312887057958	9.48489127296585e-52	2.08799509148109e-50	DOWN
SLC5A2	1.67821830507019	3.63478576083902e-20	1.64396167411662e-19	UP
SLC5A6	1.8953138589677	7.09570869842229e-60	2.29660921125701e-58	UP
SLC5A7	-4.44694930352938	3.73605848672535e-31	2.98285128057536e-30	DOWN
SLC6A1	1.10854742860051	1.06287036492086e-12	3.12051284174119e-12	UP
SLC6A14	2.2078706945889	2.35484365404345e-15	8.04017500938403e-15	UP
SLC6A15	-1.22279699305848	0.00774133312520778	0.0107128533504922	DOWN
SLC6A16	-1.29912884556852	5.55715296257226e-14	1.75652032196897e-13	DOWN
SLC6A19	-5.15666994046005	2.15474379299685e-36	2.25891352603577e-35	DOWN
SLC6A2	-1.20758448232065	0.0161249697919944	0.0214946106551327	DOWN
SLC6A20	3.27428869575181	4.92819718077659e-41	6.50111344764112e-40	UP
SLC6A3	2.057272252636	2.69652756731685e-09	6.32726512999437e-09	UP
SLC6A6	2.85327992380285	1.59449532182345e-100	3.22848221382694e-98	UP
SLC6A9	-1.34500048551781	2.7185390634346e-23	1.45610337470276e-22	DOWN
SLC7A1	1.12943500916172	3.31050069891509e-31	2.65159837489459e-30	UP
SLC7A11	2.47922753817883	8.10006353460892e-52	1.78766040973568e-50	UP
SLC7A14	-3.90107896671848	4.18397102571199e-24	2.3494191380433e-23	DOWN
SLC7A3	-1.14221077025856	0.000156172722021997	0.00025489133082473	DOWN
SLC7A5	3.09916142406381	1.13638064674616e-117	3.24390101668703e-115	UP
SLC7A6	1.26314849724319	8.78481860404837e-47	1.52512508825817e-45	UP
SLC7A9	1.33635773496581	3.49918592411746e-05	6.05558780527304e-05	UP
SLC8A1	-1.78030568022458	5.15295214028493e-39	6.12480243131615e-38	DOWN
SLC8A2	-2.90383953136172	1.68534988869336e-25	1.01511579425173e-24	DOWN
SLC8A3	-2.23851687062789	7.86661751220573e-35	7.63553014158519e-34	DOWN
SLC9A1	-1.39807244098455	4.11821703601911e-41	5.45742109955866e-40	DOWN
SLC9A2	-2.23463219395524	1.54406891434867e-30	1.1907383527703e-29	DOWN
SLC9A3	-3.30210140963713	3.02194655153133e-39	3.64664970906549e-38	DOWN
SLC9A5	1.19477048318239	7.62382734882989e-19	3.18889516274741e-18	UP
SLC9A7	1.54857083663189	2.91124813551396e-21	1.39497974088345e-20	UP
SLC9A9	-2.2425569039848	6.50803036762171e-52	1.44546342846169e-50	DOWN
SLCO1A2	3.71474605184053	3.875202392309e-17	1.47333841173093e-16	UP
SLCO1B1	4.90372680655564	4.48022214534268e-21	2.1211013653304e-20	UP
SLCO1B3	6.51827000996719	1.63055132142938e-65	6.92507077074385e-64	UP
SLCO1C1	-1.66386217415323	2.22309438904828e-25	1.32798430862771e-24	DOWN
SLCO2A1	-2.10091161513668	1.41606718918116e-45	2.3087994349449e-44	DOWN
SLCO4A1	3.59028869107903	4.44610877269611e-157	3.68667105042654e-154	UP
SLCO4C1	-3.61610871026185	1.26110805619001e-42	1.78099550546932e-41	DOWN
SLCO5A1	1.75358749115802	2.4474721616078e-24	1.39001411448391e-23	UP
SLIT2	-2.05349413343556	8.22192089995943e-20	3.64018074322384e-19	DOWN

GeneSymbol	log2FoldChange	pvalue	padj	change
SLIT3	-2.52957136666491	3.41271134001708e-39	4.10114165381073e-38	DOWN
SLITRK2	-2.83338129655532	1.3658112167794e-21	6.64883162364541e-21	DOWN
SLITRK3	-3.66905695881827	4.84372210229108e-19	2.05566007719217e-18	DOWN
SLITRK4	-1.31647382537259	1.0227956941542e-05	1.8521153725361e-05	DOWN
SLITRK5	-1.48660971488352	7.96544606577427e-06	1.45634515270188e-05	DOWN
SLMAP	-1.37093780027291	4.86905661942126e-66	2.11962207284956e-64	DOWN
SLN	1.60349018551327	8.00362540353421e-07	1.58515842984237e-06	UP
SLURP1	2.32653020758583	3.70058961618697e-05	6.38825884670008e-05	UP
SMC1B	1.35033000851778	6.74949575491287e-08	1.4410123783754e-07	UP
SMCHD1	-1.19708113390631	1.89944296147145e-25	1.14248705658385e-24	DOWN
SMCO2	2.42940043841478	3.9065241798134e-31	3.11466600472027e-30	UP
SMIM10	-1.48008784303081	2.75252684021094e-18	1.11698321763209e-17	DOWN
SMIM10L2B	-1.47272015766021	1.84219281282586e-24	1.05243121554254e-23	DOWN
SMIM14	-1.86104778850607	7.45719127573951e-62	2.63926974968399e-60	DOWN
SMIM2	-1.72248065058316	2.61464719753063e-12	7.50187043180111e-12	DOWN
SMIM23	1.10991575082567	0.00188546627179386	0.00278753813811739	UP
SMIM28	-3.85605404455591	2.42110541208852e-24	1.37593696281649e-23	DOWN
SMIM31	-1.17473858072552	1.95113152241594e-10	4.95769052967003e-10	DOWN
SMIM32	-1.3966716107079	1.14638089901955e-07	2.41144365723936e-07	DOWN
SMIM33	-1.36468273385315	5.47102319085569e-08	1.17715219105857e-07	DOWN
SMIM36	3.24648660460874	6.97075494675848e-22	3.44932525967336e-21	UP
SMIM41	1.01548236666456	0.00052730410539988	0.000821356685511057	UP
SMIM6	-1.80102417573008	4.20702766556344e-38	4.77555232988632e-37	DOWN
SMKR1	3.97161887450115	8.79785696999405e-79	6.86982436854288e-77	UP
SMLR1	2.06334286232749	1.90261107053049e-12	5.50334992876204e-12	UP
SMOC1	1.71717532060762	1.909776004703e-08	4.23953717107259e-08	UP
SMOX	2.33669312155611	1.22667009095447e-80	1.00281719689156e-78	UP
SMPD1	-1.77751237578832	9.42208669201665e-62	3.30779829774367e-60	DOWN
SMPD3	-1.3208286831316	3.0896292276778e-23	1.64777071183931e-22	DOWN
SMPDL3A	-2.67113692950407	4.61233939043006e-98	8.45417534795354e-96	DOWN
SMPX	-2.50616579051775	7.31435566930965e-11	1.91123762409497e-10	DOWN
SMTN	-1.70428153230997	3.45665855492126e-46	5.79314681586563e-45	DOWN
SMTNL2	2.10073982361349	3.22438988382242e-16	1.15908961699009e-15	UP
SMYD1	-4.7951113694697	4.92855479804061e-40	6.20541754868265e-39	DOWN
SMYD5	1.09340375858664	1.20161579315141e-66	5.38640614439578e-65	UP
SNAI1	1.7407973772494	2.630624239985e-43	3.83002173000491e-42	UP
SNAP25	-2.57259556072432	9.56510845869161e-17	3.54603435365546e-16	DOWN
SNAP91	-3.26323294809452	1.87284053915908e-23	1.01278795988748e-22	DOWN
SNCA	-1.93562570456028	1.60911336121058e-19	7.0095893907414e-19	DOWN
SNCAIP	1.11616112739006	4.87435459201287e-07	9.7976609154704e-07	UP
SNCB	-2.12721118787242	1.02383107294451e-11	2.8334345952293e-11	DOWN
SNCG	-1.74227741968662	1.66002164161895e-16	6.08162357363997e-16	DOWN
SNRPB	1.35650232710045	2.72231181481898e-33	2.4397125903985e-32	UP
SNRPF	1.13808494314788	3.71480048350162e-36	3.85263971525989e-35	UP
SNTB1	2.26786936853109	1.19473283185136e-75	8.09489603152828e-74	UP
SNTG2	-2.533599151878	1.50111191221689e-21	7.30135802442255e-21	DOWN
SNX24	-1.41321779804589	9.96838662942277e-32	8.2538999704298e-31	DOWN
SOAT2	1.91431850398839	1.29232122641602e-06	2.52080088670127e-06	UP
SOBP	-1.34947470883597	4.73044954693601e-16	1.68242070998359e-15	DOWN
SOCS2	-1.23533345615366	1.47457751743739e-26	9.39166726815558e-26	DOWN
SOCS6	-1.11186817766499	1.97175199287444e-32	1.69383904548212e-31	DOWN
SOD3	1.03975931752188	1.50315083759056e-06	2.91766419963933e-06	UP
SOHLH1	2.84036897079871	7.92147377614118e-07	1.56978061754804e-06	UP
SOHLH2	-1.6425353897984	0.00231546344983938	0.00339129994550031	DOWN
SORBS1	-2.34797591090584	2.74182692905773e-44	4.15521604139967e-43	DOWN
SORBS2	-2.11990038729453	8.30669960504257e-39	9.76668198667159e-38	DOWN
SORCS1	-3.88992759295045	2.25625579014567e-42	3.14557102272271e-41	DOWN
SORCS3	-2.96562399775904	5.73076159222224e-14	1.80975247742774e-13	DOWN
SORD	1.42017610687288	2.86382523631879e-57	8.29746902496157e-56	UP

GeneSymbol	log2FoldChange	pvalue	padj	change
SOS2	-1.12388655607938	5.37621777157472e-48	9.79247699335049e-47	DOWN
SOSTDC1	-1.22045482696228	1.41335317289636e-06	2.74959744646562e-06	DOWN
SOWAHA	-1.17283799471591	2.5895713109329e-13	7.89982572482035e-13	DOWN
SOWAHB	-1.09394532409797	4.01976038058769e-30	3.05126798200408e-29	DOWN
SOX1	4.79646668491744	1.16312762178105e-27	7.83805777015223e-27	UP
SOX10	-3.89959743499442	1.41997875455581e-39	1.73760295524106e-38	DOWN
SOX12	1.23081673323069	1.15999601583153e-21	5.66751139833738e-21	UP
SOX14	7.71440460493761	8.47894103618781e-61	2.8338541317301e-59	UP
SOX15	-1.53611599332488	2.73292723948343e-22	1.38137770743469e-21	DOWN
SOX17	-1.06638524060598	1.14079943474956e-12	3.34161480120204e-12	DOWN
SOX2	1.16323966406345	0.00829439797906421	0.0114219337294935	UP
SOX21	2.51010488656879	1.53966992785719e-05	2.7494895347941e-05	UP
SOX4	1.67019312910079	3.82079336239121e-67	1.76476060528695e-65	UP
SOX5	-1.5562629210232	3.52682428019127e-19	1.50742737336698e-18	DOWN
SOX9	1.87630892343281	2.09754218306704e-74	1.31383100840814e-72	UP
SP140	-1.20763936521584	1.33166531961994e-10	3.42717827527965e-10	DOWN
SP5	3.70684819101673	7.98806141944215e-66	3.4429731063551e-64	UP
SP6	2.0141295846196	1.58918565579028e-32	1.37060375553621e-31	UP
SP7	1.33782225712973	0.00271408923329303	0.00394824025224157	UP
SP8	5.82880020040553	1.29079760589103e-41	1.74915631995178e-40	UP
SP9	4.98048624982424	1.16671191922006e-12	3.41674312973072e-12	UP
SPAAR	-1.07039762780375	3.3603479528913e-23	1.78886392246091e-22	DOWN
SPACA3	2.79495828616264	1.74232368831622e-18	7.15375675186284e-18	UP
SPAG17	2.51875350945022	5.06719663582326e-17	1.91399338437289e-16	UP
SPARC	1.34395459153204	3.47280758807435e-18	1.40176167202454e-17	UP
SPARCL1	-2.35919343069162	6.88033481033331e-39	8.11702371628278e-38	DOWN
SPART	-1.6801616235862	2.03357184500598e-25	1.21853360416687e-24	DOWN
SPATA12	3.0354172971541	1.91074163859715e-93	2.99745442818848e-91	UP
SPATA17	1.31904578474725	8.36946087798886e-17	3.11271726331525e-16	UP
SPATA21	1.78344974687883	2.07327931431473e-06	3.97599258812361e-06	UP
SPATA25	1.38755607768162	1.42764259989292e-16	5.25682820721832e-16	UP
SPATA33	1.2875564701944	1.56807833653145e-45	2.55186430598338e-44	UP
SPATA6L	1.20704439123174	6.19040806091472e-20	2.76606557774463e-19	UP
SPC24	1.28387861794151	4.58933855665489e-32	3.86432071020462e-31	UP
SPC25	1.14162005376331	6.26500905883608e-36	6.4134393146098e-35	UP
SPDYC	3.65739529649459	1.01251215628792e-39	1.25397398132585e-38	UP
SPDYE3	1.33973028956113	1.0499337214465e-08	2.37034822916477e-08	UP
SPECC1	-1.25276105841091	1.46815312444383e-31	1.20418984248424e-30	DOWN
SPECC1L	-1.05131194534417	1.167576269228e-69	5.96217172318687e-68	DOWN
SPEF1	1.38616806430521	7.99277987262371e-09	1.82218219327045e-08	UP
SPEG	-3.0041173089585	2.60829795291014e-34	2.45106811948323e-33	DOWN
SPHK1	1.90630460082721	1.9908541264138e-23	1.07294159403415e-22	UP
SPHKAP	-4.13453250700262	2.41418333339183e-30	1.85027175987464e-29	DOWN
SPIB	-4.70425852977787	1.37727150355171e-82	1.27566110060351e-80	DOWN
SPIN3	1.19521808915469	2.31666251539283e-14	7.46484907115755e-14	UP
SPIN4	1.00765584590428	1.91330081184891e-28	1.32999229687525e-27	UP
SPINDOC	1.43975077065397	5.11241373231523e-55	1.32869343762396e-53	UP
SPINK2	-3.10729203031 79	7.31160150739431e-31	5.74794207892809e-30	DOWN
SPINK4	1.20202104842035	0.00087514744667694	0.00133604615895016	UP
SPINK5	-1.97827895092634	3.76982942897018e-20	1.70238692548387e-19	DOWN
SPINK7	2.06667556370795	2.04506700293432e-05	3.61383719526033e-05	UP
SPNS1	1.40811385397174	2.3533812613337e-26	1.4869168324965e-25	UP
SPNS2	1.75954550842651	6.67818203825623e-30	5.02103557133661e-29	UP
SPNS3	1.74682130295141	3.00331253942678e-32	2.55729492660335e-31	UP
SPOCD1	2.22886072605862	7.60708611213735e-36	7.7690434293635e-35	UP
SPOCK3	-3.91182961411773	2.76086868005441e-19	1.18762367405602e-18	DOWN
SPON1	-1.51952077107584	1.93992957726076e-11	5.25676839851254e-11	DOWN
SPON2	1.29788522951809	8.12468291431643e-21	3.80309418244602e-20	UP
SPOUT1	2.25001576015243	4.65916936660457e-22	2.32531144112024e-21	UP

GeneSymbol	log2FoldChange	pvalue	padj	change
SPP1	3.88542572663837	4.83142607514087e-43	6.95286134268e-42	UP
SPPL2A	-1.65379094009943	1.4714685099197e-102	3.08707001966648e-100	DOWN
SPRED3	1.30423516840266	3.7130704334096e-16	1.32954339825131e-15	UP
SPRR1A	6.85082844890931	4.51822784091553e-31	3.58922907818714e-30	UP
SPRR1B	6.6715234373619	5.74687982679752e-31	4.54246111774967e-30	UP
SPRR2A	6.15169084079425	2.33113158620297e-29	1.70339883804248e-28	UP
SPRR2D	6.69868421317595	1.66099003776751e-35	1.66702129842338e-34	UP
SPRR2E	4.81649109850219	2.38860685460296e-10	6.02881738791149e-10	UP
SPRR2F	1.91972711973488	0.0181148886890309	0.0239783015387378	UP
SPRR3	5.25304731078602	1.82086137269835e-19	7.91479258182636e-19	UP
SPRY4	1.02094826367446	1.80075916279722e-32	1.55077246794204e-31	UP
SPSB3	1.35315868451759	4.3397045999614e-12	1.22694067542016e-11	UP
SPTBN2	3.12073111158661	2.8040254811196e-151	1.95305982810942e-148	UP
SPTBN4	-1.32098165507928	6.52228836118624e-09	1.4959510963295e-08	DOWN
SPTBN5	3.11172306574716	1.65355724172338e-51	3.60819451756005e-50	UP
SPTLC3	-1.16201692439379	2.99507250933525e-10	7.50729776954869e-10	DOWN
SPX	-2.37792514618577	1.14650035429636e-19	5.03378988133196e-19	DOWN
SPZ1	3.00911223138541	2.22552583967076e-12	6.41607308711705e-12	UP
SQLE	1.5129210180849	1.32694264904074e-33	1.21100903290076e-32	UP
SQOR	-1.36010680539959	2.99128732365966e-37	3.26361442148407e-36	DOWN
SRCIN1	1.85002445294019	1.97363389331443e-29	1.45007961959005e-28	UP
SRD5A2	2.12275365943637	1.54336098359423e-07	3.21350529801821e-07	UP
SRI	-1.45849367755742	1.99516657926232e-45	3.23179866462276e-44	DOWN
SRL	-1.63322976193372	4.08471423523301e-19	1.74117818795869e-18	DOWN
SRM	1.49223877655838	2.50008338260111e-38	2.87352818093948e-37	UP
SRMS	2.19967942051411	6.90239839628516e-31	5.43115514118904e-30	UP
SRPX	-2.68052963924544	6.91086377639889e-45	1.08121177842259e-43	DOWN
SRPX2	2.90346668491134	1.27503197795884e-49	2.53159998086628e-48	UP
SRRM4	-1.30415680734786	0.000283171998820942	0.000451793477686373	DOWN
SRRM5	1.08995793262096	4.72441153694806e-18	1.89553405743955e-17	UP
SRXN1	4.68172776851579	1.12328543098565e-30	8.73203982578267e-30	UP
SSBP2	-2.15311502841333	4.26698007127981e-54	1.06296028585401e-52	DOWN
SSBP4	1.11317071603125	1.42619194146096e-25	8.63801053101208e-25	UP
SSC4D	1.2880332890817	3.3528252275732e-31	2.6830305922671e-30	UP
SSC5D	-1.42805079727978	4.79639467247429e-11	1.26968106463659e-10	DOWN
SSPN	-1.80238281713368	1.35930878072655e-32	1.17876712145376e-31	DOWN
SST	-5.34753632434003	3.06145043668544e-46	5.14565988938258e-45	DOWN
SSTR1	-1.36557398403687	4.54030066875089e-13	1.3643942505357e-12	DOWN
SSTR2	-2.49530166716791	1.0358382593446e-53	2.52619770447725e-52	DOWN
SSTR3	-2.25307535113647	1.15782801752086e-21	5.65850667108919e-21	DOWN
SSTR5	4.29281798706957	1.82942446106766e-37	2.02002334436088e-36	UP
ST20	1.03528107675352	6.39079012112854e-13	1.90064608674998e-12	UP
ST3GAL4	-1.16549949834645	1.46345230669874e-08	3.27083750693686e-08	DOWN
ST6GALNAC1	-1.45714029454525	1.76770449822813e-12	5.1224893372685e-12	DOWN
ST6GALNAC3	-1.44373171394529	2.29783790904529e-22	1.16721853880413e-21	DOWN
ST8SIA1	-1.85708742241132	4.7964051223359e-32	4.03477306257174e-31	DOWN
ST8SIA3	-4.11986181701998	1.08842362899903e-33	9.97511613250528e-33	DOWN
ST8SIA4	-1.07837320708929	1.14317412634788e-13	3.55339005035624e-13	DOWN
ST8SIA6	-1.22122154212595	8.57326987829178e-08	1.81503159137623e-07	DOWN
STAB1	-1.42639985905228	5.48351805595982e-20	2.4565088733838e-19	DOWN
STAB2	-2.20921260711612	5.13972262557888e-40	6.45263086367737e-39	DOWN
STAC	-1.83127478401933	2.85076911953073e-13	8.66930539266304e-13	DOWN
STAC2	-1.29464976408469	1.2720716863715e-05	2.28639391771129e-05	DOWN
STAP1	-2.58085459134402	7.11802555618411e-32	5.944660863781e-31	DOWN
STAR	-1.21519642318111	2.38171597357484e-19	1.0285917720203e-18	DOWN
STARD9	-1.38560012646075	2.55558912835391e-22	1.29512437404036e-21	DOWN
STBD1	-1.47934996188872	6.46706535483233e-51	1.36996361342695e-49	DOWN
STC1	1.95153201788809	4.0978302397065e-33	3.63873115573734e-32	UP
STC2	3.56575258563992	5.72497895905995e-71	3.09593349733263e-69	UP

GeneSymbol	log2FoldChange	pvalue	padj	change
STEAP4	-1.18987121239092	4.21523360332197e-09	9.78534365213244e-09	DOWN
STIL	1.14993280473238	1.08661557771968e-36	1.15584832344733e-35	UP
STIM1	-1.06539859053866	7.95134702462682e-67	3.58696387927012e-65	DOWN
STK17B	-1.19609612953503	5.9770725931988e-34	5.52141989736715e-33	DOWN
STK31	3.71493703622744	6.39655875384674e-65	2.65198279954127e-63	UP
STK32A	-1.33903303118695	1.09283356373981e-08	2.46464329042888e-08	DOWN
STK32B	-1.62502349232561	4.15214027748397e-24	2.33229737586543e-23	DOWN
STK32C	1.01073662792973	4.6669312286719e-13	1.40088387320917e-12	UP
STMN2	-3.082620192583	5.2298634952483e-30	3.95430364927306e-29	DOWN
STMN3	1.35595396898067	9.58310411502429e-11	2.49097763778053e-10	UP
STMN4	-4.50281015536817	2.17303413418564e-48	4.06871434178221e-47	DOWN
STOM	-1.17663559160263	1.44573141908061e-20	6.67405121963167e-20	DOWN
STOML3	1.14890422936044	9.82934295615017e-06	1.78420044715358e-05	UP
STON1	-1.96606972101216	3.48417077612893e-29	2.52370489703548e-28	DOWN
STON1-GTF2A1L	-2.36165525461402	2.17423655986145e-10	5.5021045221432e-10	DOWN
STOX1	1.0605921172537	5.98721775015175e-10	1.47315843836926e-09	UP
STOX2	-2.61611751402469	2.6862964882313e-53	6.37281754081357e-52	DOWN
STPG1	-1.12373943976194	7.03802197336812e-50	1.40865605312941e-48	DOWN
STPG3	1.26653493948619	7.98857084952029e-08	1.69474883287886e-07	UP
STPG4	5.13203058208401	1.59355388412704e-76	1.1188932977542e-74	UP
STRA6	5.79739912291695	1.42288522948378e-111	3.64363242661781e-109	UP
STRIP2	2.34338236769173	4.87925538072014e-60	1.58512078254626e-58	UP
STRIT1	3.58936192555273	1.93713628828347e-07	4.00847940438265e-07	UP
STUM	-3.16774162677357	1.34155244124845e-25	8.1338623466084e-25	DOWN
STX12	-1.08977941589208	6.41753054309735e-52	1.42718338884999e-50	DOWN
STX16-NPEPL1	1.72167376325125	2.07047077910761e-19	8.9751326055765e-19	UP
STX1A	2.03172478561851	3.83184605962415e-42	5.28715811697586e-41	UP
STXBP1	1.11937846076222	3.88038593302844e-14	1.23730379512588e-13	UP
STXBP5L	-1.95265608465406	1.03916594223124e-08	2.34786512937233e-08	DOWN
STYK1	-1.75503276341475	1.45586353514273e-39	1.78026346470789e-38	DOWN
STYXL2	7.3824836273442	1.05728253298631e-71	5.88193634085962e-70	UP
SUCLG2	-1.36313342003264	1.36164893504717e-59	4.32671403393728e-58	DOWN
SUGCT	-2.04616736289311	6.23655576769621e-41	8.20220132801315e-40	DOWN
SULF1	1.65401605620007	2.41102329855971e-16	8.73738786635176e-16	UP
SULT1A1	-2.23309802541346	2.42302534477953e-47	4.30532044169857e-46	DOWN
SULT1A2	-3.79913336218275	4.02873019247347e-73	2.40247530279249e-71	DOWN
SULT1A3	1.35385206918096	1.21796683626613e-07	2.5580094704984e-07	UP
SULT1B1	-2.78176748043712	2.22068406924167e-45	3.58708457307098e-44	DOWN
SULT1C2	2.37109197642484	1.65281506602542e-27	1.10396888165326e-26	UP
SULT1C3	1.61528903580839	1.46204049198518e-05	2.61622763199445e-05	UP
SULT1E1	1.9326643531269	1.27262657683034e-06	2.48461112034384e-06	UP
SULT2B1	3.55438614261089	2.91968149292512e-91	3.94111735165156e-89	UP
SULT4A1	-1.16866787449669	0.00241072241786099	0.00352518554435787	DOWN
SUN3	1.38579941621562	7.62527830552458e-07	1.51332312667084e-06	UP
SUPT4H1	3.24095483018484	2.84342729337775e-35	2.81641635151233e-34	UP
SUSD5	-1.87016528983602	2.0682627708683e-17	7.99082751922114e-17	DOWN
SUSD6	-1.04782443077093	6.53365228621645e-38	7.34476999741039e-37	DOWN
SUV39H1	1.29577334102865	8.60317757126422e-39	1.01084433905819e-37	UP
SUV39H2	1.09167822545919	8.183532657463e-47	1.42642496661064e-45	UP
SV2B	-2.09984285419075	3.15014004539507e-19	1.3504034616067e-18	DOWN
SVEP1	-1.81247384844819	1.03376154947553e-25	6.30946016860057e-25	DOWN
SVIL	-2.13769392164014	1.74818614301368e-76	1.21764661233189e-74	DOWN
SVOP	-2.11736189022103	9.04928993371641e-13	2.66805427727402e-12	DOWN
SVOPL	-2.14311006135625	3.21659942649218e-09	7.51518124426517e-09	DOWN
SYCE3	1.31666651776082	8.55077321432804e-10	2.08273344497264e-09	UP
SYCP2	1.07727718956936	1.70795254697365e-06	3.29937627029645e-06	UP
SYCP2L	1.04497365470673	7.63809852351476e-05	0.000128120806849015	UP
SYN2	-1.77026547870549	5.41972242493986e-15	1.81802401436097e-14	DOWN
SYN3	1.74487875870916	1.42628275030486e-10	3.66387322226029e-10	UP

GeneSymbol	log2FoldChange	pvalue	padj	change
SYNC	-1.9017135969025	5.87850438127939e-23	3.08971919080042e-22	DOWN
SYNDIG1L	-2.28763201726735	1.44531028797732e-18	5.9581411090315e-18	DOWN
SYNE1	-1.62601589894832	3.99324979179063e-23	2.11866114029403e-22	DOWN
SYNE3	-1.7200197085989	3.70904426352346e-38	4.22957352722554e-37	DOWN
SYNE4	1.73346282028511	5.8652851896322e-15	1.96483668732331e-14	UP
SYNGR1	-1.98814074025483	2.29619542874668e-22	1.16672456961675e-21	DOWN
SYNGR4	1.50556136756978	1.04273395762651e-08	2.35531539812562e-08	UP
SYNM	-4.38974651779005	7.32565843207796e-59	2.23792439083813e-57	DOWN
SYNPO	-1.61437136314274	8.11493958323985e-43	1.15919149272318e-41	DOWN
SYNPO2	-3.99145971974977	1.31021871588476e-56	3.67388703698894e-55	DOWN
SYNPR	1.29994721805317	0.000787832208217942	0.00120782904047359	UP
SYP	-1.67441523241462	3.38092930845043e-20	1.53193135696194e-19	DOWN
SYPL2	-2.02248239930575	1.17803508416661e-25	7.16490566559315e-25	DOWN
SYT10	-3.79411278026535	1.62399847891731e-24	9.30526012286511e-24	DOWN
SYT11	-1.20649536913849	1.21590573312847e-16	4.48571324808603e-16	DOWN
SYT12	1.65973268608945	5.10824535960498e-13	1.53018882585243e-12	UP
SYT15	-1.66034475819364	2.61972994631336e-14	8.42270265050858e-14	DOWN
SYT2	-1.2076882777503	1.60015212513619e-12	4.64855671588196e-12	DOWN
SYT4	-3.20905948211031	1.9752296011036e-15	6.77861116358236e-15	DOWN
SYT5	-1.77277703321004	5.14184331091896e-13	1.53998826235005e-12	DOWN
SYT6	-1.92173749730876	5.219228515006e-10	1.28911242740141e-09	DOWN
SYT7	1.82414878883583	5.48307029718581e-32	4.60128689565766e-31	UP
SYT8	2.1814975053832	1.09098783276393e-20	5.07272927421052e-20	UP
SYT9	-3.11135709909894	2.7991387186448e-31	2.25237534693909e-30	DOWN
SYTL1	1.1765040266598	1.91479523473951e-11	5.19270042400234e-11	UP
SYTL2	-1.37109298996672	3.5771545436481e-28	2.45812912661974e-27	DOWN
SYTL4	-1.10102024260666	1.95318436900437e-25	1.17319073533884e-24	DOWN
TACC3	1.04897187331091	1.18024031964836e-30	9.16251657870569e-30	UP
TACR1	-2.52473857483662	6.94276977806536e-23	3.63373760581461e-22	DOWN
TACR2	-3.91387627948322	4.21165484919115e-54	1.05068117319435e-52	DOWN
TACSTD2	5.00816606177476	2.17736631942874e-69	1.08949654368427e-67	UP
TAF1A	1.23222962819896	3.2837221076193e-38	3.75686288173291e-37	UP
TAF1D	1.26040079396102	2.3598801098642e-46	3.99344920826679e-45	UP
TAF1L	1.45922114257956	1.56897866062475e-06	3.03967795031807e-06	UP
TAFA2	-1.43076969807953	3.82843689072508e-36	3.96576868400927e-35	DOWN
TAFA4	-2.98012323730046	5.5306112603137e-12	1.55354950598229e-11	DOWN
TAFA5	1.26270159430369	8.58880937849841e-15	2.85087567113597e-14	UP
TAGAP	-1.39385255456742	3.12232742678295e-17	1.19597640744768e-16	DOWN
TAGLN	-2.94411146619614	7.12288833729823e-47	1.24404066817828e-45	DOWN
TAGLN3	-3.60060368669648	5.87484265866063e-29	4.19256701701875e-28	DOWN
TAL1	-1.1488458666971	2.49819025665144e-15	8.52125111441166e-15	DOWN
TAL2	1.02258446252991	5.46103858164998e-05	9.28099402911098e-05	UP
TARBP1	1.23832303829214	3.02158599045168e-47	5.35247984249594e-46	UP
TAS1R1	-1.98493025745465	4.30914607812901e-13	1.29594405282315e-12	DOWN
TAS2R13	1.19873495002804	0.0137482615541273	0.0184849415830453	UP
TAS2R3	1.2814374040701	0.002749130222917	0.00399687772995355	UP
TAS2R30	1.28530791699973	0.0101496216654454	0.0138594229972083	UP
TAS2R38	2.85193285684402	1.57398807694185e-30	1.21327376643597e-29	UP
TAT	-4.0040485446502	2.40751029479426e-75	1.61238372166356e-73	DOWN
TAX1BP3	1.66899611403988	8.00150039667253e-10	1.95359122836874e-09	UP
TAZ	1.02526883428081	1.62471357208463e-31	1.32635430992544e-30	UP
TBC1D1	-1.14637514874587	3.1971663184658e-42	4.43250454645263e-41	DOWN
TBC1D16	1.1582501560301	1.34562607583055e-33	1.22741680767089e-32	UP
TBC1D30	1.17066923030329	6.73120793583252e-29	4.79192656527603e-28	UP
TBC1D9	-1.29959434286238	1.04400230693459e-21	5.11082714946638e-21	DOWN
TBPL2	-2.71064762828656	4.15935159105975e-17	1.57826954140605e-16	DOWN
TBX10	-1.67211053145522	1.44369254097954e-10	3.70563358137924e-10	DOWN
TBX15	3.06366161758825	3.29665980816192e-36	3.43124550146584e-35	UP
TBX18	1.424763133984	1.39935591137999e-06	2.72378543313881e-06	UP

GeneSymbol	log2FoldChange	pvalue	padj	change
TBX20	3.27775402276716	4.93885883422112e-17	1.86673212242875e-16	UP
TBX21	-1.02663456630997	1.20126394623029e-07	2.52353831532247e-07	DOWN
TBX4	2.51849456528074	1.20886166889435e-12	3.53482925952263e-12	UP
TBX5	4.00667632861714	1.50435850938091e-10	3.85566598820278e-10	UP
TBX6	1.02953058359849	1.1154178702592e-14	3.67090746074911e-14	UP
TBXT	1.9806073992265	0.00134638376447496	0.002018474428825	UP
TCAP	1.10398294732767	3.31871710041056e-08	7.25352339267592e-08	UP
TCEA3	-1.42345156619893	1.87493503159793e-22	9.5826955401276e-22	DOWN
TCEAL2	-3.92280885178961	2.08151950791623e-22	1.06074039190358e-21	DOWN
TCEAL3	-1.23285631143664	6.09792524793936e-19	2.56977667817928e-18	DOWN
TCEAL5	-3.4981523901062	3.20893344894742e-23	1.71087440742564e-22	DOWN
TCEAL6	-4.07664368550595	3.06647038311787e-31	2.46066584245306e-30	DOWN
TCEAL7	-2.25107250456067	2.08776435711709e-35	2.08333757882406e-34	DOWN
TCF21	-2.10036003701962	1.25853242507786e-42	1.77880074008773e-41	DOWN
TCF24	4.48913054921216	3.47758769401042e-36	3.61308081836536e-35	UP
TCF7	1.95690796626422	1.77520652212963e-64	7.10613130341223e-63	UP
TCF7L1	-1.57896331291061	2.41997628168025e-44	3.68670577365688e-43	DOWN
TCFL5	1.34262607549299	6.3453214517115e-35	6.18651077484055e-34	UP
TCL1A	-3.27933719696152	3.18004972065416e-17	1.21701551177474e-16	DOWN
TCN1	7.19960704782164	8.43282437567546e-82	7.2693450917642e-80	UP
TCN2	-1.74480986536062	1.16030446375574e-43	1.71805966219207e-42	DOWN
TCOF1	1.09936040437359	4.55289241888933e-35	4.46896931736865e-34	UP
TCP11	3.87274333826256	2.54632240152951e-18	1.03596056022975e-17	UP
TCP11L1	-1.20597330526631	3.57134907868338e-62	1.28753419269386e-60	DOWN
TCTE3	1.05711325891991	5.39955627871451e-19	2.28320722392559e-18	UP
TCTEX1D2	1.15595863099672	2.47376986750192e-11	6.66807348340727e-11	UP
TDGF1	3.03406935537456	1.19138184972768e-48	2.25494914666393e-47	UP
TDO2	1.7451307665794	1.4310456000656e-14	4.67344280456533e-14	UP
TDP2	-1.91593996006524	1.94369716144786e-74	1.22186276795277e-72	DOWN
TDRD10	-1.75044433039956	8.96057478070218e-32	7.43356306128476e-31	DOWN
TDRD12	-1.12488192804529	6.06834900006476e-08	1.30117179088939e-07	DOWN
TDRD5	1.19585595362022	1.15648467971933e-07	2.43199519884467e-07	UP
TEAD4	2.3335238084252	3.01537862387303e-118	8.75113132958352e-116	UP
TECTA	-1.0315668030568	4.24370480286822e-13	1.27736614921944e-12	DOWN
TEDC2	1.7653600762653	8.27051138151474e-58	2.44506646326513e-56	UP
TEF	-1.80260055099914	3.9956135240161e-83	3.86531212742736e-81	DOWN
TEK	-1.38796990416353	4.46195963076923e-24	2.50228995331995e-23	DOWN
TEKT1	1.61767699202321	0.000397375172239832	0.000625915321050402	UP
TEKT3	-1.0159322719714	1.80496284284481e-08	4.0114636863378e-08	DOWN
TEN1-CDK3	1.04991429329551	4.22009570316138e-12	1.1940937029436e-11	UP
TENM1	-1.82501741897437	2.89270611883378e-10	7.25907070863994e-10	DOWN
TENM2	-1.29968457868432	1.51317691836744e-06	2.93581612028215e-06	DOWN
TENT5A	-1.25358880334063	5.97839204033008e-23	3.14127159318852e-22	DOWN
TENT5B	-3.05623894144488	1.05140577100966e-52	2.4378333809043e-51	DOWN
TENT5C	-2.05452003255877	2.63237809285938e-40	3.36299337717978e-39	DOWN
TEPP	-1.735846595718	2.42296178727665e-15	8.2695087420322e-15	DOWN
TERB1	1.26215866084018	2.78023341932621e-06	5.27941161730941e-06	UP
TERT	1.60454107300883	1.44224985415352e-16	5.30949190494191e-16	UP
TESC	4.6418696165282	1.77970274093433e-118	5.25253624201516e-116	UP
TESK2	-1.31212786803632	4.24501091466996e-56	1.16223860152749e-54	DOWN
TESPA1	-1.62403302177752	1.51927918347883e-22	7.79469900468973e-22	DOWN
TEX11	-4.14540893604135	3.34406490289311e-117	9.39196808936738e-115	DOWN
TEX19	2.83427595772018	5.07439295945209e-15	1.70645818082154e-14	UP
TEX22	1.27573719354509	4.64027297873787e-15	1.5643963868105e-14	UP
TEX30	1.30198788522059	9.37561684801552e-28	6.34452663320402e-27	UP
TEX43	-1.17869352585612	1.25792734782151e-06	2.45673944679407e-06	DOWN
TEX45	2.52700035360955	2.26314770236972e-57	6.60103700860366e-56	UP
TEX46	1.85958162723764	3.837702187722e-08	8.3521945000379e-08	UP
TEX48	1.88774085408374	1.50006178881755e-06	2.91199285715495e-06	UP

GeneSymbol	log2FoldChange	pvalue	padj	change
TFAP2A	3.17646470162615	7.77206084700278e-36	7.9328778152907e-35	UP
TFAP2B	-3.3305272154632	8.89084007100907e-10	2.16284155010451e-09	DOWN
TFAP4	1.45973470798919	2.83150974474174e-85	2.95239995120886e-83	UP
TFCP2L1	-1.30211818761764	2.29394294230634e-16	8.33390954608394e-16	DOWN
TFF1	1.28799558806141	8.62146406880111e-06	1.57265403132237e-05	UP
TFF2	1.70604708151454	2.03250168131729e-07	4.19684000673284e-07	UP
TFR2	2.8229757116775	9.08985475415338e-58	2.68273967515378e-56	UP
TG	4.0864542334011	1.38618481078326e-68	6.74235645535446e-67	UP
TGDS	1.12386509851527	7.94936248501396e-30	5.95364511619562e-29	UP
TGFB1I1	-1.55476296754217	1.87313594253225e-32	1.61071190949699e-31	DOWN
TGFBI	3.30199481446642	1.44597216802273e-103	3.10848313108394e-101	UP
TGFBR3	-1.33224753183626	6.80885616711468e-18	2.70320593793817e-17	DOWN
TGFBR3L	-1.43374745831003	2.98799535728714e-08	6.55041711651026e-08	DOWN
TGIF1	1.0144884585066	5.85032261351312e-38	6.58937048312445e-37	UP
TGIF2	1.90246167266819	1.06106827161176e-71	5.88419802980114e-70	UP
TGM4	-1.29322300213043	1.76554938058237e-13	5.44229268261299e-13	DOWN
TGM6	2.68940225055793	0.000485176448287721	0.000758722720613748	UP
TH	3.4165145144666	5.59829323118639e-37	6.03610402691323e-36	UP
THBS2	2.24190128475406	9.27979750085572e-20	4.09397298916647e-19	UP
THBS4	-2.30253360610948	2.9303206873837e-13	8.89879213976499e-13	DOWN
THEG	1.14481991315261	0.00712362381130934	0.00990052369912439	UP
THEM6	1.2909004803787	1.05374287838073e-28	7.43168276275564e-28	UP
THEMIS	-1.593441111931	2.05534080680865e-17	7.94442829499646e-17	DOWN
THRB	-2.4565298015447	1.66587102993843e-34	1.58686062605678e-33	DOWN
THRSP	-2.08915438679375	1.46707354822942e-09	3.51584801752255e-09	DOWN
THSD7A	-1.32982728187898	2.65833749508727e-12	7.62220167988715e-12	DOWN
THY1	1.36162294337461	8.94478536526661e-21	4.18135698162114e-20	UP
TIAM1	-1.33743051297116	1.87961505486269e-13	5.7836608853727e-13	DOWN
TICRR	1.57370120017529	4.03826466067069e-49	7.81314472625097e-48	UP
TIFAB	-1.62862396462912	8.67617468389162e-16	3.03796963142177e-15	DOWN
TIGD1	1.46437560467367	1.5340086713089e-43	2.25225067398836e-42	UP
TIGD4	1.60576265658052	1.91326799971957e-34	1.81557142665487e-33	UP
TIMD4	-1.61551491326203	1.89717789542215e-09	4.50443941818734e-09	DOWN
TIMP1	2.01654683156558	5.52736011682021e-61	1.86166192870774e-59	UP
TINAG	-1.40705299117645	2.17640809312002e-12	6.27758723297977e-12	DOWN
TINCR	-2.85695599056572	2.39662421595496e-31	1.93384696350434e-30	DOWN
TJP3	-1.10703365006535	7.78298721695583e-23	4.06372283085013e-22	DOWN
TKT	1.17159898751908	2.47148128201473e-48	4.60769845436001e-47	UP
TLCD1	1.37107090606054	4.395439447196e-26	2.74034325435102e-25	UP
TLCD2	-1.51418911904047	9.81309933950517e-46	1.60899716383054e-44	DOWN
TLCD3A	1.25054255299023	1.08593599445501e-32	9.47839773004765e-32	UP
TLCD4	-1.63468468611268	2.12896851996019e-26	1.34708316998789e-25	DOWN
TLE4	-1.01890925495109	1.61649882801416e-19	7.03878321885737e-19	DOWN
TLL1	-2.49032548393593	1.22368047683716e-27	8.23336481575174e-27	DOWN
TLR1	-1.1287504622362	1.587390770041e-10	4.05951468331972e-10	DOWN
TLR10	-2.48465160179867	6.15587253099317e-29	4.39132357157657e-28	DOWN
TLR3	-2.09281791158461	1.99939626843746e-53	4.80213616859332e-52	DOWN
TLR6	-1.0396361404051	2.26275519565273e-10	5.71862935005819e-10	DOWN
TLR7	-1.90966736383208	1.98726335231371e-25	1.19242649048376e-24	DOWN
TLR8	-1.08340058962757	1.02323218199184e-06	2.00919508175733e-06	DOWN
TLX1	4.06653424596003	5.22358223664831e-75	3.38134711846681e-73	UP
TLX2	-1.29916824617173	9.0196417069567e-09	2.04717180713291e-08	DOWN
TLX3	3.88758441033313	3.77432909507253e-11	1.00508323187793e-10	UP
TM4SF19	2.69175243609517	2.27102813854106e-18	9.2720780718442e-18	UP
TM4SF4	1.40461736681227	2.06447815188141e-05	3.64702831071432e-05	UP
TM6SF2	-2.8225605621393	1.6061854096954e-38	1.86456710260173e-37	DOWN
TM7SF2	1.02076059450049	3.1669592974271e-17	1.21280541557286e-16	UP
TMCC3	-2.41996250206596	5.19288501454329e-82	4.5668538766789e-80	DOWN
TMCO2	1.19586824085177	0.00149320948074286	0.00222880650507247	UP

GeneSymbol	log2FoldChange	pvalue	padj	change
TMED7-TICAM2	1.69403933431361	1.84288539793789e-07	3.81798494161719e-07	UP
TMEFF2	-3.98896785148011	7.67057833869793e-33	6.74925622090687e-32	DOWN
TMEM100	-3.89478858141205	1.68471445258435e-73	1.02573191478501e-71	DOWN
TMEM108	-1.98674342813637	2.15108119171574e-39	2.60841064006589e-38	DOWN
TMEM130	-2.51147138648008	1.01736968126359e-29	7.57394538685029e-29	DOWN
TMEM131	-1.04732458138165	1.66246309253564e-62	6.05616523646926e-61	DOWN
TMEM132A	2.61518076115864	1.66576599629234e-87	1.90828837456832e-85	UP
TMEM132B	-1.76529608411307	2.20848328357958e-17	8.51745723520959e-17	DOWN
TMEM132D	-1.72266326419042	7.67798128230568e-11	2.00324674960727e-10	DOWN
TMEM140	-1.14561417412996	8.74811706756714e-40	1.0880783035539e-38	DOWN
TMEM145	1.91614855749189	2.71954509704941e-17	1.04330114066802e-16	UP
TMEM147	1.20061606080479	6.40447053922119e-31	5.04620115382165e-30	UP
TMEM151A	1.61115523053727	1.19630539785753e-08	2.68825214774722e-08	UP
TMEM151B	-3.80398284764512	2.6481717006123e-45	4.24219078406274e-44	DOWN
TMEM155	-2.38381709076743	2.06956309740025e-31	1.67849567838987e-30	DOWN
TMEM156	-1.72521209204218	1.84985821167601e-23	1.00129254087393e-22	DOWN
TMEM158	1.5645295093119	3.04892713089305e-23	1.62905701534951e-22	UP
TMEM160	1.8836907013006	6.83301271668234e-19	2.86771178927433e-18	UP
TMEM161A	1.35330687952778	9.68766527806386e-44	1.4381186316021e-42	UP
TMEM171	-2.29739585154989	6.98399439746867e-50	1.39984449481465e-48	DOWN
TMEM178B	1.30537835182688	4.1138482283162e-06	7.7051133913811e-06	UP
TMEM179	-2.62783438712742	1.08718563311107e-16	4.01850210769755e-16	DOWN
TMEM190	1.34209819605308	2.29263385099851e-06	4.38121523786623e-06	UP
TMEM198	1.45281192391816	1.71970673308278e-19	7.47883450129134e-19	UP
TMEM200B	-1.64602668941072	4.43467226465415e-21	2.10067867639888e-20	DOWN
TMEM201	1.00066143360601	5.65560533617417e-29	4.04107737869515e-28	UP
TMEM205	1.06720002609235	5.32436012381875e-19	2.25414740666316e-18	UP
TMEM211	3.94458851667586	9.67479929275794e-46	1.58781602341936e-44	UP
TMEM213	2.76795936006407	7.71521303271524e-12	2.15194541424978e-11	UP
TMEM215	1.50890719209896	5.8420157095001e-06	1.08093740887818e-05	UP
TMEM217	-1.05327988736082	5.54471241056092e-17	2.08892421473599e-16	DOWN
TMEM220	-3.01604092317971	7.26252596797704e-84	7.22642083887909e-82	DOWN
TMEM236	-3.65615824244588	8.3264778124896e-63	3.05883877951227e-61	DOWN
TMEM238L	-1.69449508707615	1.62100091938838e-21	7.86910761341226e-21	DOWN
TMEM249	4.23611531764028	1.31430202718932e-36	1.3929361655172e-35	UP
TMEM25	-1.50658704904984	1.15714153081068e-19	5.07923001663886e-19	DOWN
TMEM253	-2.73536366881317	3.84969775683768e-63	1.43851474334366e-61	DOWN
TMEM255A	-2.38485939718066	3.05507686943218e-31	2.45378475679993e-30	DOWN
TMEM255B	1.72133127687721	5.90797020710752e-25	3.46498771358583e-24	UP
TMEM270	2.49623632928634	1.61047339700886e-31	1.31596308128181e-30	UP
TMEM273	-1.27276925148988	4.36767662817168e-16	1.55657701855001e-15	DOWN
TMEM30B	-1.14193767919878	1.13162324793858e-42	1.60333243420296e-41	DOWN
TMEM31	1.11812906683852	0.00189635370053491	0.00280220697449206	UP
TMEM35A	-4.18045267426942	1.72174455240668e-68	8.30491354322922e-67	DOWN
TMEM37	-2.93642715271574	5.80773905300578e-74	3.61179143321392e-72	DOWN
TMEM40	3.88616550680635	2.15480344155889e-25	1.28895885702044e-24	UP
TMEM41A	1.01386210694123	7.83164520467799e-62	2.76057566698498e-60	UP
TMEM45B	-1.07441262146754	1.20082480360494e-20	5.57301767195439e-20	DOWN
TMEM47	-1.49668435294625	1.16698162591158e-18	4.82905205608324e-18	DOWN
TMEM52	1.49272199688658	3.18604947634236e-19	1.36512498847317e-18	UP
TMEM52B	1.6610133360823	2.88613379053753e-13	8.77020182425826e-13	UP
TMEM59	-1.10136529048994	9.3576634782796e-48	1.69030076916268e-46	DOWN
TMEM59L	-2.87821467064384	1.30096773734398e-31	1.06907745211754e-30	DOWN
TMEM61	-1.15817030573438	7.98226986360723e-07	1.58110869224198e-06	DOWN
TMEM63A	1.30334074543818	5.81226696420331e-39	6.88965314143446e-38	UP
TMEM63C	-1.20632061499278	7.25308195020208e-08	1.54417307737949e-07	DOWN
TMEM67	1.03647695610228	9.8324625362454e-21	4.58523487262028e-20	UP
TMEM72	-4.30688083386354	1.24444875580452e-65	5.33733649872515e-64	DOWN
TMEM74	-2.44932094348787	3.27352218008059e-35	3.23139692300132e-34	DOWN

GeneSymbol	log2FoldChange	pvalue	padj	change
TMEM74B	3.31317534132017	2.87460622518138e-49	5.60532118690743e-48	UP
TMEM82	-3.13933753359372	4.73764913377804e-43	6.82354709400141e-42	DOWN
TMEM92	1.05427369142825	1.20499487743925e-11	3.32002781659013e-11	UP
TMEM97	1.54772496504954	3.04681465005715e-56	8.43029436391838e-55	UP
TMIE	-1.27653585622934	3.20803721525428e-11	8.58615924218846e-11	DOWN
TMIGD1	-6.13550850800813	2.7668565528372e-48	5.13638306551749e-47	DOWN
TMIGD2	-1.41013321173906	1.56973433629959e-14	5.10625518363249e-14	DOWN
TMIGD3	-1.44318770776097	9.03094434318797e-16	3.15648000497656e-15	DOWN
TMOD1	-2.96492752542716	1.11109144112054e-43	1.64659023525378e-42	DOWN
TMOD2	-1.4018257657083	1.20575753442897e-20	5.59441938369617e-20	DOWN
TMPRSS11D	1.76895389064249	0.00146000279842587	0.00218092379934714	UP
TMPRSS11E	2.67178755234705	1.18479718539928e-07	2.48954668629874e-07	UP
TMPRSS13	3.3791432039809	2.88687116392074e-53	6.811529482026e-52	UP
TMPRSS15	1.54565163376143	0.0162065703366325	0.0215984240985597	UP
TMPRSS2	-1.39389005864109	2.59234875899674e-38	2.97564726040938e-37	DOWN
TMPRSS3	2.89629905613826	1.45131330082616e-43	2.13623994144429e-42	UP
TMPRSS5	3.32384252523181	2.9733662434999e-34	2.78811127614775e-33	UP
TMTC1	-1.91917717121076	2.52747871211667e-30	1.93539959604607e-29	DOWN
TNFAIP6	1.19975515763247	3.34004567877612e-08	7.29830786855673e-08	UP
TNFAIP8L2	-1.00403032642526	7.58110457434479e-13	2.24430081525103e-12	DOWN
TNFAIP8L3	-1.5078025974294	4.98834442095718e-18	1.99866639213363e-17	DOWN
TNFRSF10B	1.23032601978998	1.24063766658978e-37	1.38128028697749e-36	UP
TNFRSF10C	1.09598516948853	1.73189091873819e-06	3.34376500365761e-06	UP
TNFRSF11A	-1.08285214131897	4.21779580647473e-13	1.2702261912512e-12	DOWN
TNFRSF11B	1.93505843989678	7.9340769339502e-19	3.31389018111957e-18	UP
TNFRSF12A	2.23176216806079	1.67932936651084e-77	1.23384650882082e-75	UP
TNFRSF13B	-3.30741146757306	3.8392212407085e-39	4.60415698791027e-38	DOWN
TNFRSF13C	-1.80740357338336	2.05043600552186e-20	9.38597322927241e-20	DOWN
TNFRSF17	-3.30251438393265	1.46251770817595e-36	1.54625506086629e-35	DOWN
TNFRSF19	1.30484315520752	8.99417107904108e-07	1.77407681240759e-06	UP
TNFSF10	-1.52236987159325	8.6126379689368e-47	1.49822042910186e-45	DOWN
TNFSF11	1.33896224444336	5.71450761935655e-14	1.80494687422194e-13	UP
TNFSF12	-1.2193497837792	2.08636342649655e-23	1.12302461655593e-22	DOWN
TNFSF15	1.715673019449	1.4483108428556e-53	3.49299677377348e-52	UP
TNFSF4	1.1599199030758	1.14028141651362e-10	2.94595256761893e-10	UP
TNFSF8	-1.22528887930023	5.72255141028843e-12	1.60539371205659e-11	DOWN
TNFSF9	3.23668015515625	7.60652798669614e-42	1.03965833463375e-40	UP
TNNC2	1.52600816894654	3.94410040693753e-09	9.17059959754349e-09	UP
TNNI3	5.31167658433703	2.54638650943605e-54	6.40754744057946e-53	UP
TNNT1	4.1520882292342	1.68133907132454e-33	1.5280353470237e-32	UP
TNNT2	2.01455082600688	3.65711942327337e-16	1.31031729459792e-15	UP
TNNT3	-2.46324040097212	8.62208611405847e-15	2.86137574812465e-14	DOWN
TNR	-1.55949529595623	2.80690111926238e-11	7.5380273272233e-11	DOWN
TNS1	-2.89137540849342	3.92262906236367e-48	7.23567159565027e-47	DOWN
TNS2	-1.1970304523892	1.43731951003104e-26	9.1577184881707e-26	DOWN
TNS4	3.39578939405908	1.63355482125316e-78	1.25863230541953e-76	UP
TNXB	-4.03242510184503	2.46552088662914e-75	1.64490862830932e-73	DOWN
TOM1L2	-1.19140442486804	1.4771157013176e-43	2.17238308336515e-42	DOWN
TOMM34	1.81833658508387	1.32924204225868e-83	1.30768879558477e-81	UP
TOMM5	2.08399923622456	1.01392665357369e-24	5.86951622961389e-24	UP
TONSL	1.43699303444106	4.89425008415127e-40	6.16668427751998e-39	UP
TOP1MT	1.85893289226017	2.45852938656207e-64	9.72963004731941e-63	UP
TOP2A	1.3670917789238	9.90839455636693e-39	1.15950856458345e-37	UP
TOX	-1.5504161264003	6.27724626667443e-15	2.09880355686639e-14	DOWN
TP53I3	-1.01583201211148	4.11095265468782e-26	2.5666553809996e-25	DOWN
TP53INP1	-1.22430389815833	8.65901291558735e-30	6.47399707596061e-29	DOWN
TP53INP2	-2.58252688635337	2.11062248035967e-89	2.6440481475182e-87	DOWN
TP53RK	1.16811864978109	2.20060692465294e-29	1.60937288445954e-28	UP
TP73	2.227047158525	6.15785512538378e-33	5.44298128417806e-32	UP

GeneSymbol	log2FoldChange	pvalue	padj	change
TPGS1	1.49003082944447	4.21966747676216e-13	1.27057011538751e-12	UP
TPH1	-3.31075236495395	1.04536713227122e-32	9.1380411015255e-32	DOWN
TPM1	-1.76959337123934	1.885252323747e-60	6.22920279191774e-59	DOWN
TPM2	-2.71697718685146	2.74421998393843e-53	6.50137450072379e-52	DOWN
TPO	-1.95038673635005	9.15608493180215e-17	3.40018995345427e-16	DOWN
TPPP2	-1.57069952689688	2.23599951996559e-11	6.03931435414315e-11	DOWN
TPSAB1	-1.60228717035022	7.66699977705483e-14	2.40160941028703e-13	DOWN
TPSB2	-1.92190451864409	2.64041129471247e-18	1.07198605443759e-17	DOWN
TPSD1	-1.07231359005982	0.00183463947613932	0.00271585286049595	DOWN
TPSG1	-2.01610120960219	1.81602717178607e-13	5.59293971388589e-13	DOWN
TPX2	1.86616388003359	1.24661363163974e-85	1.31559291925714e-83	UP
TRABD2B	-1.05198308337911	9.42971385713639e-09	2.13634670042045e-08	DOWN
TRAF3IP3	-1.06729607264105	8.84859341656295e-12	2.45977900962022e-11	DOWN
TRAF5	1.45384316634389	4.29265470012114e-40	5.42832217089393e-39	UP
TRAIP	1.25192959920933	4.91228917638588e-46	8.16199345690909e-45	UP
TRAK2	-1.11003622221334	9.12801396070038e-69	4.47735512951199e-67	DOWN
TRANK1	-2.14107670765297	1.29365376521489e-70	6.86780274807528e-69	DOWN
TRAP1	1.43412201396074	1.57755187272451e-56	4.41638436651961e-55	UP
TRAPPC3L	-1.00778441605731	1.00949805472609e-05	1.82879625748495e-05	DOWN
TRARG1	-2.46057319636443	2.37949572976043e-05	4.17893687769222e-05	DOWN
TRAT1	-1.37167147501738	4.64711081670712e-11	1.23128637631347e-10	DOWN
TRDN	-2.42920237430145	1.77045886445649e-05	3.14613738205744e-05	DOWN
TREM1	2.44797197636963	3.54875411402596e-25	2.09970966318498e-24	UP
TREM2	1.12113717496033	8.39013134289698e-08	1.77755635811978e-07	UP
TREML4	1.50064896393083	0.000348701947403194	0.000552145767948696	UP
TREX2	1.45757352922792	4.14337694276748e-20	1.86720038054892e-19	UP
TRHDE	-2.37020979896671	2.18069947678292e-26	1.37931420229644e-25	DOWN
TRIB3	3.84565419042333	3.34355275199491e-167	3.63883025440547e-164	UP
TRIM29	4.0160732466054	1.03147332582679e-94	1.71057571644019e-92	UP
TRIM36	-1.5812455367868	1.77391056743806e-27	1.18349060194632e-26	DOWN
TRIM40	-3.09448411366193	5.72086493293964e-51	1.21781688358531e-49	DOWN
TRIM50	-1.68710368418495	9.89990364307945e-12	2.74195995127951e-11	DOWN
TRIM54	2.82878846653183	1.7754988565254e-16	6.49511798081446e-16	UP
TRIM55	-2.02621226424502	4.02695086738334e-14	1.28169064985827e-13	DOWN
TRIM58	-1.49550750923734	2.70102688349715e-07	5.52743931394241e-07	DOWN
TRIM59	1.25842033917493	4.16602600641318e-44	6.2591036108432e-43	UP
TRIM61	-1.20746692383497	1.83433463234973e-11	4.98148299331032e-11	DOWN
TRIM63	-1.10254683839611	0.000557793234158722	0.000866909459693488	DOWN
TRIM7	1.99003655244014	5.9724085571285e-13	1.78200051756817e-12	UP
TRIM71	5.76099715396467	2.54800653050748e-29	1.85797477871553e-28	UP
TRIM72	5.3697986080257	3.82646665910725e-41	5.08627968969729e-40	UP
TRIM9	-2.33608694205191	7.58013822320962e-26	4.68060095321806e-25	DOWN
TRIP13	2.21008688067245	7.06072656239215e-133	3.07371079077336e-130	UP
TRIP6	1.17832788798968	1.12842811088625e-14	3.71162045615078e-14	UP
TRMT1	1.44331478603881	4.22132701546429e-57	1.21296975776039e-55	UP
TRNP1	1.24778858357275	1.10471426026643e-07	2.32717026542698e-07	UP
TROAP	1.96624544903479	4.48691932669823e-83	4.29289704592287e-81	UP
TRPA1	-1.23653072002957	1.45874168740164e-08	3.260727728206e-08	DOWN
TRPC1	-1.30634485265257	7.45615382274753e-16	2.61814895171411e-15	DOWN
TRPC4	-1.17672791352378	8.02396984145338e-14	2.51071674481991e-13	DOWN
TRPC6	-1.34123099551643	5.97173832380292e-20	2.67041292841243e-19	DOWN
TRPC7	-2.28556911790592	1.84043678004371e-27	1.22646481633759e-26	DOWN
TRPM1	1.64155827493575	0.00175343945164828	0.00260029306519771	UP
TRPM2	1.59850873906848	2.00358606694059e-22	1.02222221458062e-21	UP
TRPM4	-1.01758851997844	1.0834986682405e-17	4.25890797067086e-17	DOWN
TRPM6	-4.15870951031263	3.78796607565665e-72	2.14852942265177e-70	DOWN
TRPM8	1.29216383689599	4.79377652022384e-05	8.19095579890665e-05	UP
TRPV3	-2.9575235598154	7.74482336239158e-45	1.20951219021816e-43	DOWN
TRPV4	1.67146312442331	4.3253645088993e-21	2.05224992352762e-20	UP

GeneSymbol	log2FoldChange	pvalue	padj	change
TRPV6	1.3583789564205	0.000390044793806723	0.000614591439196133	UP
TSACC	1.85127029801314	1.06516371756712e-42	1.51039868192152e-41	UP
TSC22D3	-1.33913773262126	1.55880855212697e-25	9.42811160756754e-25	DOWN
TSEN2	1.12193151683314	7.70095749799969e-33	6.77256428851862e-32	UP
TSHR	-1.19075413031913	1.70759159448477e-07	3.54443824469701e-07	DOWN
TSHZ1	-1.29277722989987	1.80052679902662e-44	2.77456399570359e-43	DOWN
TSHZ3	-1.45986140516359	7.82088326947437e-22	3.8568405656006e-21	DOWN
TSKS	-1.0095723275177	4.91058117061199e-05	8.37902498029069e-05	DOWN
TSLP	-1.13177814001856	4.62829563708851e-10	1.14836865102055e-09	DOWN
TSNARE1	1.01450044919295	1.32080978823753e-20	6.10545814775157e-20	UP
TSPAN1	-2.29341514480324	8.86375141650417e-46	1.45745517861744e-44	DOWN
TSPAN11	-1.64627687250528	2.54443826670877e-25	1.51526345889876e-24	DOWN
TSPAN18	-1.48923799590608	2.54901241292688e-18	1.03681273408773e-17	DOWN
TSPAN2	-1.97705962289939	1.94926888834259e-22	9.95092909783333e-22	DOWN
TSPAN5	1.2047318848906	2.53743411178243e-23	1.36203268151872e-22	UP
TSPAN7	-2.64236284247947	6.01927803981046e-55	1.5620519896754e-53	DOWN
TSPEAR	3.15278384608451	1.21129249949325e-29	8.96397632540413e-29	UP
TSPO2	1.31482716472956	3.04635219727826e-11	8.16219892463555e-11	UP
TSPOAP1	1.14811986517391	4.61034982573831e-07	9.28092734284175e-07	UP
TSPYL5	-1.79746793380172	7.2945038496652e-30	5.47732624123416e-29	DOWN
TST	-1.41432479901654	3.90655482073284e-38	4.44316388591907e-37	DOWN
TTC16	2.133171807819	1.97216607277571e-19	8.55752001625804e-19	UP
TTC22	-1.57149024235241	1.33032986195273e-50	2.79096793809432e-49	DOWN
TTC26	1.60539623269708	2.51607765182974e-82	2.28189896621413e-80	UP
TTC28	-1.47162368847884	2.92853244214002e-28	2.01958556098947e-27	DOWN
TTC29	4.09997749422964	6.76531874103628e-10	1.65898458298359e-09	UP
TTC9	1.51664143416734	3.15641001710082e-19	1.35275824828394e-18	UP
TTI1	1.2133936721565	1.48234045496697e-52	3.42334142471352e-51	UP
TTK	1.21746419847389	5.82433402621132e-32	4.88061253120393e-31	UP
TTLL4	1.13988578528081	4.79958321994225e-62	1.72319881667741e-60	UP
TTLL6	-2.29000358386226	9.78231432930956e-38	1.0954304785612e-36	DOWN
TTLL7	-1.73714238431517	2.2267727545983e-18	9.09565891996722e-18	DOWN
TTLL9	1.72037433861317	2.13683535972961e-16	7.77611580333787e-16	UP
TTYH3	1.61494023844985	2.79518560814059e-52	6.36242705811139e-51	UP
TUB	-2.003907140029	9.0728200615649e-26	5.5706987211576e-25	DOWN
TUBA1A	-1.27249979684653	3.03511416179087e-18	1.22765256444284e-17	DOWN
TUBA4B	1.74458947515876	9.6682740542738e-10	2.34508505512007e-09	UP
TUBAL3	-2.53506000089033	7.26596532849788e-37	7.77641390689205e-36	DOWN
TUBB2A	-1.1103069397873	3.16972567477261e-18	1.28120782671345e-17	DOWN
TUBB2B	-1.28183627903958	1.4312470821792e-07	2.99043741804492e-07	DOWN
TUBB3	4.15459674912357	3.90557701129795e-64	1.53863829180387e-62	UP
TUBB4A	-3.23555059771529	1.93345221036589e-43	2.82917675118498e-42	DOWN
TUBB6	-1.13353867248526	2.50320806262539e-12	7.19398613541771e-12	DOWN
TUNAR	-1.78527013739988	6.02524269991556e-08	1.29240639484639e-07	DOWN
TUSC3	-1.48096663457691	4.2281274925463e-21	2.00720785244571e-20	DOWN
TWIST1	1.81402432983931	1.80529761179413e-20	8.28125587833803e-20	UP
TWIST2	-1.39753085947453	6.11830443405215e-13	1.82240908501796e-12	DOWN
TWNK	1.52669996674171	9.49598020893087e-105	2.09308232124194e-102	UP
TXLNB	-1.69688192692935	1.80449313988335e-20	8.27974678387057e-20	DOWN
TXLNG	1.03088110325111	4.53329486355773e-31	3.59955601728822e-30	UP
TXNDC5	1.40058621234814	9.7965673987445e-09	2.21772787460138e-08	UP
TXNIP	-1.5844128576691	1.14325127821958e-34	1.09803830709529e-33	DOWN
TXNRD3	1.15106217965841	3.62310301232772e-51	7.8274308627373e-50	UP
TYR	3.83379150990794	1.12397447205332e-07	2.36574005582792e-07	UP
TYRO3	1.73497227888301	1.43506604023082e-33	1.30694586603239e-32	UP
TYRP1	-1.27426254847265	4.79114281689048e-08	1.03547436850582e-07	DOWN
UBD	4.63512358944197	7.12049174547683e-22	3.5204180228276e-21	UP
UBE2C	2.122240432554	8.28182598808207e-87	9.24432281605597e-85	UP
UBE2L5	-1.00387473893614	9.16591365333055e-12	2.54311750231748e-11	DOWN

GeneSymbol	log2FoldChange	pvalue	padj	change
UBE2QL1	-2.04104087163115	1.25101375800352e-22	6.45257777491569e-22	DOWN
UBE2S	1.88612478118739	7.70063921302961e-60	2.47858097257827e-58	UP
UBE2T	1.44196942632118	2.0988797271662e-48	3.93410039710926e-47	UP
UBE3D	1.17578963442876	5.79759347001954e-53	1.35145241088956e-51	UP
UBTD1	1.17290845142203	1.36310758501519e-21	6.63752583273755e-21	UP
UBXN11	1.18146731433517	3.12565805195159e-30	2.38192926295987e-29	UP
UCHL1	-2.08363276346956	3.04168846570269e-20	1.38073308793746e-19	DOWN
UCHL3	1.80722027311456	1.74491652454788e-30	1.34324630601026e-29	UP
UCKL1	1.38880012577767	1.93250436783712e-32	1.660942673107e-31	UP
UCN	2.38785721898521	4.38808858168667e-42	6.04029932592174e-41	UP
UCN2	4.62946247624842	1.17658512196746e-53	2.85744445311287e-52	UP
UCN3	-1.83209878704551	1.61943914646117e-08	3.61066502654653e-08	DOWN
UGDH	-1.85243639590745	1.03403245316861e-78	8.03821745849334e-77	DOWN
UGGT2	1.13508610619957	4.75203215305353e-30	3.60083271893477e-29	UP
UGP2	-2.16903814399484	1.32441655228218e-156	1.00269849673433e-153	DOWN
UGT1A1	-2.57698314972731	1.27350042649037e-26	8.13479931272075e-26	DOWN
UGT1A10	-3.57712614877472	1.60293575231677e-54	4.07473288395502e-53	DOWN
UGT1A6	1.5790105131571	7.08798629035553e-09	1.62078930103691e-08	UP
UGT1A7	-1.38158455343891	6.37140029452829e-08	1.36396844515148e-07	DOWN
UGT1A8	-4.23910278722543	3.91988777452903e-60	1.28061924611396e-58	DOWN
UGT1A9	-2.09042052053147	1.25122197864883e-11	3.44140393527279e-11	DOWN
UGT2A3	-3.21817955782772	2.22014097547774e-25	1.32697238027427e-24	DOWN
UGT2B11	-2.39546880289596	1.85925957860328e-09	4.41862795717470e-09	DOWN
UGT2B15	-3.16548217005921	1.13362538385339e-29	8.41133195798005e-29	DOWN
UGT2B17	-4.04352015131437	9.80523858575822e-26	5.99924875241771e-25	DOWN
UGT2B4	2.59041413335277	7.83799578240367e-07	1.55429928890781e-06	UP
UGT3A2	-1.38498400969524	4.48329987960906e-06	8.36828178836238e-06	DOWN
UHRF1	1.8861125997784	2.39778287094013e-57	6.97038282665783e-56	UP
ULBP1	3.83736066868349	1.70358141920493e-65	7.21763096170691e-64	UP
ULBP2	4.17570420620579	6.25015523948759e-67	2.83421753086452e-65	UP
ULBP3	2.03700988850544	5.25543215345389e-67	2.39562408607572e-65	UP
UMODL1	1.7247523909991	3.42904413597983e-11	9.15937191897788e-11	UP
UNC13C	-1.76675401114682	4.28640660226277e-07	8.64580078364435e-07	DOWN
UNC45B	-1.0845557714213	4.60571907220775e-07	9.27267732736196e-07	DOWN
UNC5A	2.08268158890954	7.73568257469381e-13	2.28772827230203e-12	UP
UNC5C	-2.39918412367875	3.39643998107412e-67	1.57293110080967e-65	DOWN
UNC5CL	2.15455578267693	6.62005231048772e-41	8.68688552242069e-40	UP
UNC5D	-3.12505486527301	7.21603000691732e-32	6.02362082983947e-31	DOWN
UNC79	-1.35647302826704	4.848897364242e-25	2.84960681078454e-24	DOWN
UNC80	-2.67960614118939	3.12450201532465e-21	1.49442065456641e-20	DOWN
UNC93A	3.87243598903065	2.99040832785093e-38	3.42578817189922e-37	UP
UPK1A	3.3010973261381	3.74218104384794e-17	1.42556548931359e-16	UP
UPK2	4.28773551489565	7.00356212878983e-24	3.8752153590282e-23	UP
UPK3A	1.38394093893374	1.9425311410894e-06	3.73471290270396e-06	UP
URAD	-1.25820529820348	0.000610181911924897	0.000945377491978666	DOWN
URB1	1.02583961871833	1.60353656093225e-39	1.95398055531934e-38	UP
URB2	1.22469460081135	2.06073425173397e-78	1.58077381169355e-76	UP
UROC1	2.78321568460187	2.04337710718154e-11	5.53020291690272e-11	UP
USP2	-3.86552451078696	7.20490069929106e-99	1.39398817640839e-96	DOWN
USP26	3.28543129093725	5.26636986401316e-15	1.76896794834223e-14	UP
USP41	2.05972989329918	7.62499481162278e-11	1.98972028555054e-10	UP
USP44	-1.29212522343453	5.64947437860518e-12	1.58540366405563e-11	DOWN
USP51	-1.4323869503027	1.33123735779183e-21	6.49141308071386e-21	DOWN
UST	-1.52799397946813	2.42754792714902e-12	6.98576963401185e-12	DOWN
UTP14A	1.37267798048735	2.04336989938873e-54	5.16417997939854e-53	UP
UTP4	1.14263972264235	2.29558630241851e-66	1.01454427116786e-64	UP
UTS2B	-2.17506217073489	1.92843664300133e-19	8.37403173680351e-19	DOWN
VAMP2	-1.31676599735483	5.23687588691959e-50	1.06281724730689e-48	DOWN
VARS1	1.12109306935148	1.0848613049478e-43	1.60908772598433e-42	UP

GeneSymbol	log2FoldChange	pvalue	padj	change
VARS2	1.44287712922386	9.65617446459873e-20	4.25570655408903e-19	UP
VASH2	1.19752653384708	7.05302825352571e-18	2.7975940997413e-17	UP
VAT1L	-1.98001967245254	9.26091478264326e-30	6.91807417890035e-29	DOWN
VAX1	3.63387479853378	7.91358494858332e-08	1.67965936993761e-07	UP
VCAN	1.28756823732683	4.17793323571376e-12	1.18235578471451e-11	UP
VCL	-1.16363426438513	1.00373400616883e-37	1.1225446531418e-36	DOWN
VDR	-1.10242735170835	3.60163391679052e-34	3.3645521133623e-33	DOWN
VEGFA	1.53110497560688	1.78846188619881e-53	4.30144845640605e-52	UP
VEGFD	-4.53115933386712	1.07522369952653e-79	8.62805082020989e-78	DOWN
VENTX	3.75152541722543	3.32278097311027e-31	2.66021080849513e-30	UP
VEPH1	-1.09268060823808	8.62960961169591e-07	1.70487170601839e-06	DOWN
VGF	2.25053033690469	2.89665258172118e-21	1.38836805410159e-20	UP
VGLL1	5.84930682237148	9.49046041273493e-26	5.81687388831233e-25	UP
VGLL3	-1.25378922821313	1.35204013918706e-08	3.0284377339419e-08	DOWN
VILL	-1.41389075467543	4.77099525198151e-25	2.80666690279574e-24	DOWN
VIP	-3.29591123077641	3.12838654161445e-30	2.3829656539428e-29	DOWN
VIPR1	-1.50206686846049	5.69431801017951e-25	3.34080726116091e-24	DOWN
VIPR2	-1.99178904841442	4.3162380406687e-22	2.15787117433718e-21	DOWN
VIT	-2.58583082221628	3.75550331325819e-22	1.88565683949725e-21	DOWN
VLDLR	-1.16307422003795	1.45837087992427e-11	3.98972696498372e-11	DOWN
VMA21	1.00155943870785	1.26086567197152e-34	1.20634362340879e-33	UP
VNN1	1.66391568335874	8.24774246571953e-09	1.87858652132863e-08	UP
VNN3	1.7121503430742	5.75131705159886e-11	1.51509355248852e-10	UP
VPREB3	-2.43263116046974	4.81163760579136e-24	2.68800274718142e-23	DOWN
VPS35L	-1.31177894810042	1.92770944394189e-22	9.84664257769435e-22	DOWN
VPS4B	-1.10282290346477	1.8568293815165e-42	2.60120434596515e-41	DOWN
VSIG1	1.44717277014885	4.70528337997549e-07	9.46546898053526e-07	UP
VSIG2	-2.83002129085962	1.77215511849691e-23	9.60427546790747e-23	DOWN
VSIG4	-1.59524161255039	7.73343179535146e-15	2.57726790148239e-14	DOWN
VSIG8	2.73151227816516	1.03159684986588e-10	2.67508502557178e-10	UP
VSIR	-1.60677891479029	5.47586489920777e-48	9.94277742334775e-47	DOWN
VSNL1	2.79650587325056	6.97584343329482e-64	2.68739738283103e-62	UP
VSTM2A	-5.21769619400742	5.65914964390537e-54	1.39975529473472e-52	DOWN
VSTM2B	1.60552842504425	0.00525606011495091	0.00740931086302151	UP
VSTM2L	1.32261216556212	1.10692347376329e-05	1.99884459697607e-05	UP
VSTM4	-1.27396867088965	5.76909743924505e-17	2.17111073502429e-16	DOWN
VTCN1	3.35042014287198	1.9141174792439e-16	6.98753200546626e-16	UP
VWA2	3.99741977374105	2.02789986034965e-126	7.35662922255591e-124	UP
VWA3B	-1.53689536684396	5.5736127907755e-16	1.97543902963106e-15	DOWN
VWA5A	-1.45642274790476	7.41935364100602e-28	5.05253050257481e-27	DOWN
VWA5B1	1.33521955245562	0.00279930129080542	0.00406506824925317	UP
VWC2	-2.37759711883314	7.80768066505914e-23	4.0753939874303e-22	DOWN
VWCE	1.35896359633954	1.36591786662576e-08	3.05873557247356e-08	UP
WAS	-1.07428621801638	1.81733743198431e-13	5.59598527022861e-13	DOWN
WASHC2C	-1.10146208749076	1.29072943484355e-43	1.90793477495168e-42	DOWN
WASL	-1.1148795851458	1.79605644215527e-54	4.55236256582965e-53	DOWN
WDFY4	-1.29343453455595	1.77028410052996e-13	5.45592160044748e-13	DOWN
WDR17	-2.78030771298795	6.40545533989079e-23	3.359584151612e-22	DOWN
WDR24	1.05583318058353	1.77369199666519e-14	5.75145227894432e-14	UP
WDR27	1.10088463108156	1.59881619961396e-25	9.64997798401311e-25	UP
WDR38	1.1096103189661	0.00142349508808175	0.0021287633088945	UP
WDR4	1.56220617823644	1.63857637320107e-55	4.36945335169221e-54	UP
WDR43	1.13191397078874	1.29078286807092e-61	4.50428899433244e-60	UP
WDR54	1.18688642392236	2.3540713984898e-22	1.19508586769396e-21	UP
WDR62	1.85898834751205	1.33974573831175e-59	4.26489808797486e-58	UP
WDR64	-2.03963448604648	6.12304195533364e-20	2.7366665700263e-19	DOWN
WDR7	-1.17210039098135	2.40817890331712e-45	3.87915071632386e-44	DOWN
WDR72	3.07007007689846	2.8280432229685e-30	2.16175226696885e-29	UP
WDR74	1.29565334823735	4.82927365091037e-52	1.08226695087905e-50	UP

GeneSymbol	log2FoldChange	pvalue	padj	change
WDR75	1.05123362601922	5.92469558049048e-57	1.69682112077435e-55	UP
WDR77	1.52786880989068	1.81479835624441e-57	5.31110651719057e-56	UP
WDR90	1.70194481565936	2.18967699668005e-41	2.93750736080044e-40	UP
WDR97	2.67139645027712	2.0983210984281e-34	1.98360832176594e-33	UP
WFDC1	-1.51488447010129	1.15878654428919e-23	6.34526732569424e-23	DOWN
WFDC10A	3.90892033014486	2.19009180899317e-22	1.11411243558276e-21	UP
WFDC10B	2.67500034672825	1.64083577815412e-23	8.91478109360303e-23	UP
WFDC13	1.53931606561052	2.41624559028805e-07	4.96039665924143e-07	UP
WFDC3	1.19369249417944	4.94371246662349e-09	1.14292173634247e-08	UP
WFDC8	2.38495092643885	0.0013149655149203	0.00197307147878563	UP
WFIKKN2	-1.68374419520439	8.6530384282744e-09	1.96755495105174e-08	DOWN
WFS1	1.0275900672505	1.04034090662895e-10	2.69735798200268e-10	UP
WIF1	3.35196983121141	2.33470305583778e-12	6.7263706669926e-12	UP
WNT1	-1.06178786813978	1.14346169969844e-05	2.06204417738701e-05	DOWN
WNT11	2.83177706027393	1.243906849303e-25	7.55762385447077e-25	UP
WNT16	-1.08048773773893	0.000156602540725378	0.000255473116137437	DOWN
WNT2	5.44923755606915	2.74759386348979e-206	4.34944108590433e-203	UP
WNT2B	-1.39682543595001	1.25210127478355e-32	1.08850921107368e-31	DOWN
WNT3	2.40344705287998	1.41719583949352e-63	5.41176121778522e-62	UP
WNT3A	1.46700492396985	5.80990537941865e-08	1.24775385263711e-07	UP
WNT5A	1.37951900465469	3.82350621682314e-21	1.82008512174799e-20	UP
WNT7A	2.65675206452411	4.56188026266778e-09	1.0567516431267e-08	UP
WNT7B	5.41379339467876	4.81680503645667e-60	1.5677574971929e-58	UP
WNT8B	2.36831002280647	1.02624664047376e-21	5.02531854627943e-21	UP
WNT9A	-1.69562361990631	3.10433381631523e-19	1.33109492104154e-18	DOWN
WSCD1	-2.99360155017751	1.01281627679131e-50	2.13254774217255e-49	DOWN
WSCD2	-2.70796833029008	6.0609148362928e-18	2.41341664862489e-17	DOWN
WT1	4.87260998495313	3.40193872820991e-50	7.00212282202355e-49	UP
WWTR1	-1.38002988597044	4.71566170343589e-18	1.89245948932771e-17	DOWN
XAGE2	4.08700643256486	2.0180257359725e-06	3.87387081253326e-06	UP
XCL2	-1.76505740994651	9.15329220241033e-15	3.03130994904091e-14	DOWN
XCR1	-1.26478173107945	4.60199193427942e-07	9.26624486026914e-07	DOWN
XDH	-2.37241922467835	1.41125699230604e-35	1.42211909762876e-34	DOWN
XIRP1	1.98528844078397	1.15147054075293e-13	3.57853944781915e-13	UP
XKR4	-4.48581530663131	4.73356967695483e-52	1.0621861956806e-50	DOWN
XKR5	1.2760623939847	6.00375437378284e-08	1.28811452575999e-07	UP
XKR9	1.11860608024277	3.1852787518376e-05	5.53766562557389e-05	UP
XKRX	2.48440584426798	4.92028142601897e-48	9.02812017610836e-47	UP
XPNPEP3	1.07491347417389	1.40858391764875e-50	2.94803747091559e-49	UP
XPO5	1.27761358885478	4.41870950505255e-83	4.25099384593812e-81	UP
XPOT	1.30347071163911	5.59026783431089e-61	1.87921493820184e-59	UP
XRCC2	2.05387237828259	1.6579658827573e-87	1.90828837456832e-85	UP
YDJC	1.50481775656006	3.87296362483213e-37	4.20448351615971e-36	UP
YPEL4	-1.44346871200965	5.54786532721394e-17	2.08965993819547e-16	DOWN
YTHDF1	1.02898930215123	3.00189190823073e-52	6.81511653168471e-51	UP
YY2	1.17675579158776	1.43224499375363e-25	8.67165579841166e-25	UP
ZAN	1.89596334109037	8.04628473350123e-06	1.47050751537003e-05	UP
ZAR1	2.21367201043661	3.93688667068053e-07	7.96202178821836e-07	UP
ZBED2	1.39280308315905	7.21123621326481e-08	1.53582749732852e-07	UP
ZBTB12	1.58208434623262	5.13992266971049e-39	6.11696791454862e-38	UP
ZBTB16	-3.4608126422546	1.47440158293684e-36	1.55787346866985e-35	DOWN
ZBTB7B	-1.40455677057836	1.61734557491217e-60	5.36435018970393e-59	DOWN
ZBTB7C	-2.15131883918894	5.38994637916962e-24	3.00240359246579e-23	DOWN
ZC3H12C	-1.49176865761563	4.1960333602206e-20	1.88995160117748e-19	DOWN
ZC3H8	1.0441836523838	9.56467665606261e-39	1.12079215755059e-37	UP
ZC3HAV1L	2.11860114219193	3.6026496817108e-82	3.2336566447232e-80	UP
ZCCHC12	-1.71171476306994	5.69924603486258e-21	2.68291352271052e-20	DOWN
ZCCHC24	-1.24272583934538	3.29978449827292e-16	1.18515650357009e-15	DOWN
ZCWPW2	-1.08240822404819	1.50963214428692e-19	6.58662604070864e-19	DOWN

GeneSymbol	log2FoldChange	pvalue	padj	change
ZDBF2	-1.27771548315082	5.40585952689777e-10	1.33331773288769e-09	DOWN
ZDHHC11	1.30308412117078	1.3385308519451e-08	2.99933570002832e-08	UP
ZDHHC15	-2.18324611452649	1.12226352125329e-31	9.27038647798078e-31	DOWN
ZDHHC22	-3.17493478602698	5.69534814269169e-13	1.70166604682036e-12	DOWN
ZDHHC9	1.21119956029546	9.60838919295089e-43	1.3646890784409e-41	UP
ZEB1	-1.99684098551194	6.81032328786091e-37	7.30222656474889e-36	DOWN
ZEB2	-1.60062271315052	8.19635859541777e-25	4.77494788297122e-24	DOWN
ZER1	-1.21749135972992	2.8631594786931e-70	1.48824465679053e-68	DOWN
ZFHX4	-1.40703660431852	4.84683190958914e-08	1.04686038255614e-07	DOWN
ZFP2	-1.06540184513182	7.52695043799334e-18	2.98150109137348e-17	DOWN
ZFP3	-1.08231623916935	1.53767926816537e-18	6.32843514454351e-18	DOWN
ZFP69B	1.24890350429527	2.29626261502604e-32	1.96872579593542e-31	UP
ZFP82	-1.01096912847706	9.79966118809385e-09	2.21813987089924e-08	DOWN
ZFPM1	1.37003906306046	2.42528269671235e-20	1.10756484652117e-19	UP
ZFPM2	-1.53781026905839	1.1553427517874e-16	4.26589977457041e-16	DOWN
ZFR2	-1.42930698105037	1.1183772972914e-07	2.35509782050251e-07	DOWN
ZFYVE28	-1.24913035592138	1.97734960754997e-28	1.37396603017828e-27	DOWN
ZG16	-4.42879526008034	5.56559033201381e-29	3.98002564481957e-28	DOWN
ZGRF1	1.03892983445451	5.03425750747961e-22	2.50819816817575e-21	UP
ZHX1-C8orf76	2.05794379676555	2.51977981533676e-20	1.14981462066192e-19	UP
ZIC2	6.0282698364754	8.63521233499143e-59	2.6241701987645e-57	UP
ZIC5	8.38627435207717	1.13744304082787e-56	3.21530773862593e-55	UP
ZIK1	-1.81871885056178	1.62256441745796e-39	1.97578420987381e-38	DOWN
ZMAT4	-3.15428199559933	1.21856407774087e-19	5.34076422997781e-19	DOWN
ZMYND15	1.00741780382821	9.36807756282802e-15	3.10008237555158e-14	UP
ZMYND19	1.44229876952732	1.50274622590504e-76	1.05940566929897e-74	UP
ZNF114	1.5503379626636	1.44150574619064e-09	3.45647749358546e-09	UP
ZNF121	1.55140751792659	8.3872364554302e-65	3.43361071250077e-63	UP
ZNF132	-1.1528197784193	6.53785119932677e-35	6.37065489277432e-34	DOWN
ZNF135	-1.67178617694565	1.32014923184815e-22	6.80312476299845e-22	DOWN
ZNF154	-1.25363145301219	1.60223944078351e-20	7.37504503895408e-20	DOWN
ZNF157	-1.38825325159264	1.25839375216608e-06	2.45737472316563e-06	DOWN
ZNF208	-1.39261658154452	9.13019314193058e-12	2.53441819194065e-11	DOWN
ZNF229	-2.17428631700125	5.22721759071438e-31	4.1392241885907e-30	DOWN
ZNF239	1.72027940207582	6.61935617033275e-46	1.09461395056034e-44	UP
ZNF25	-1.01589916373953	6.14381816711657e-26	3.81398594452766e-25	DOWN
ZNF251	1.07532648307595	5.61893285211068e-30	4.24479296111945e-29	UP
ZNF257	-1.27742565723134	5.75998097426791e-06	1.06655198537779e-05	DOWN
ZNF280A	5.19318649001676	3.8817222070763e-32	3.27641438641879e-31	UP
ZNF280B	1.03119075151515	1.10717636835334e-12	3.24621352115451e-12	UP
ZNF280C	1.2367454056596	6.94535475987741e-40	8.68194274470534e-39	UP
ZNF304	-1.39551001856352	8.93883754386195e-15	2.96315975436004e-14	DOWN
ZNF316	1.02991727683397	1.29545117936807e-26	8.27198070639394e-26	UP
ZNF354C	-1.17327325646741	1.11089597292557e-09	2.68480660326897e-09	DOWN
ZNF365	1.04971776168417	0.000195238294234638	0.000315838388843157	UP
ZNF382	-1.10396703925366	5.76939619423863e-13	1.72290337729853e-12	DOWN
ZNF385B	-2.37602165532015	1.89701383284636e-21	9.17320240804047e-21	DOWN
ZNF385D	-1.57060559666795	3.5740279916928e-11	9.53493939318933e-11	DOWN
ZNF396	-1.07373296653798	1.48471070057551e-22	7.62183591660418e-22	DOWN
ZNF415	-1.4350667090605	1.31688088728357e-12	3.8442325046553e-12	DOWN
ZNF418	-1.54256058243435	2.86800420724591e-18	1.16200196925865e-17	DOWN
ZNF439	-1.00592018730175	4.33820204992048e-09	1.0062756400062e-08	DOWN
ZNF454	-1.48522630963966	8.31704450045188e-19	3.47051751465058e-18	DOWN
ZNF469	1.91517860062883	2.02176914201688e-25	1.21229566356543e-24	UP
ZNF471	-1.79626191622359	1.7259625467053e-18	7.08825137400458e-18	DOWN
ZNF473	1.04120290573074	3.73544163896181e-63	1.39882247869337e-61	UP
ZNF483	-1.77498914874642	2.03770287757087e-36	2.13878964479455e-35	DOWN
ZNF485	1.10744714546939	1.48887077937339e-38	1.73184414704267e-37	UP
ZNF491	-1.1526592219533	1.54959171594465e-24	8.88769451572603e-24	DOWN

GeneSymbol	log2FoldChange	pvalue	padj	change
ZNF492	-1.12879112686353	0.00202962739031985	0.00298975566767952	DOWN
ZNF497	1.29287087987484	2.10113037378853e-17	8.11241312611522e-17	UP
ZNF511	1.32791287713862	3.51416841208847e-33	3.13324191293889e-32	UP
ZNF528	-1.09713452929361	5.98211001870349e-09	1.37495356066109e-08	DOWN
ZNF536	-3.0379391816951	1.1575054573776e-37	1.29037404156954e-36	DOWN
ZNF540	-1.53032826625827	7.55814771078566e-31	5.93908059963496e-30	DOWN
ZNF541	1.04260749670535	0.000154949626932174	0.000253013677210235	UP
ZNF549	-1.02590755124244	4.78923923715489e-08	1.03519144533985e-07	DOWN
ZNF556	3.2204264992755	6.66849632764642e-15	2.22747988784399e-14	UP
ZNF568	-1.39177164346273	4.54125300899812e-17	1.71906170968879e-16	DOWN
ZNF575	-1.88226397907526	4.11841057489497e-77	2.97567980666581e-75	DOWN
ZNF579	1.27894855335525	2.15631879756496e-22	1.09757320146152e-21	UP
ZNF582	-1.39348909364743	4.4998719162213e-17	1.70413809650199e-16	DOWN
ZNF598	1.04286503803154	1.39243015303566e-28	9.75709708443057e-28	UP
ZNF626	-1.77749292363512	1.15804266860906e-26	7.41632842533638e-26	DOWN
ZNF667	-1.59572550879884	4.70449769794956e-23	2.48315909106989e-22	DOWN
ZNF670-ZNF695	2.49483183056355	9.21170251192689e-32	7.63825599238967e-31	UP
ZNF671	-1.64844377823127	8.22031425183439e-36	8.38058150276301e-35	DOWN
ZNF676	-2.55355548458473	7.41751557080357e-19	3.1055830400193e-18	DOWN
ZNF677	-1.79121490372956	1.388194860255e-30	1.07290888156326e-29	DOWN
ZNF692	1.63098225010022	2.13039615882226e-61	7.31688132417593e-60	UP
ZNF695	2.20573657721977	6.56378618937218e-42	8.98547239902027e-41	UP
ZNF697	1.29220832137879	1.48471093585914e-32	1.28367783148536e-31	UP
ZNF703	1.0520129793612	3.7806502300033e-20	1.70638834772026e-19	UP
ZNF716	3.21328949913607	0.000983099128432522	0.00149351815768588	UP
ZNF724	1.23167523942695	2.46989524298245e-19	1.0661449148749e-18	UP
ZNF726	-1.34972685279676	1.26729073108374e-08	2.84262958912291e-08	DOWN
ZNF727	-1.78246274488424	3.47658053346493e-10	8.68421988656216e-10	DOWN
ZNF728	-2.99680541775623	3.08363437830011e-21	1.47595726853601e-20	DOWN
ZNF74	1.24703741725838	1.67211360816415e-46	2.86579864753566e-45	UP
ZNF750	2.70183760237546	1.16427478169866e-17	4.56405150241306e-17	UP
ZNF772	-1.19430642134341	1.50635585280696e-09	3.60601793578879e-09	DOWN
ZNF781	-1.86035405666398	5.74135817636731e-36	5.88430076074655e-35	DOWN
ZNF793	-1.0491165457871	3.83009733712935e-06	7.19299880623743e-06	DOWN
ZNF804A	-1.51226910042834	4.32298208262404e-11	1.14680205674486e-10	DOWN
ZNF829	-1.33127145562882	1.62692201853472e-13	5.02743444698227e-13	DOWN
ZNF831	-1.4755504122052	2.3069391273425e-13	7.06286765692395e-13	DOWN
ZNF835	-2.08001181280267	4.65308795204219e-30	3.52739314361823e-29	DOWN
ZNF850	1.07015023922789	9.19733837273518e-15	3.04484018359644e-14	UP
ZNF878	1.19069031208471	5.52661598661184e-17	2.08300788257299e-16	UP
ZNF98	-1.28044285828121	0.000134686516481064	0.000221150052945287	DOWN
ZNF99	-1.01872226544528	0.000111432330544789	0.000184253268614225	DOWN
ZNHIT2	1.12264663007694	5.10163443781601e-24	2.84635567015989e-23	UP
ZNRF3	1.58589370511408	1.03514074724627e-45	1.69407009697362e-44	UP
ZP3	1.87589563541378	1.82062204127308e-38	2.10507912381727e-37	UP
ZSCAN1	-1.56605457227769	1.30915606110394e-14	4.28663679804492e-14	DOWN
ZSCAN10	2.05172604553645	7.51959868317582e-06	1.37873825281816e-05	UP
ZSCAN18	-1.2973907259266	1.3576678887435e-12	3.95997838303024e-12	DOWN
ZSCAN23	-1.41452208785764	6.81697870340593e-07	1.35692787108376e-06	DOWN
ZSWIM4	1.26862424262891	5.40074595758554e-37	5.83032792060986e-36	UP
ZSWIM6	-1.12724744274167	7.46566755832769e-55	1.93164441594591e-53	DOWN
ZZEF1	-1.76505904813535	2.99036776210525e-84	3.00990022205426e-82	DOWN

Supplementary Table 3: Summary table of candidate genes.

symbol
A2M
ADCY2
ADCY3
ADCY5
ADCY9
ADCYAP1
ADCYAP1R1
ADGRG1
ADGRL3
ADORA3
ADRA1A
ADRA1B
ADRA1D
ADRA2A
ADRA2C
ADRB1
ADRB2
ADRB3
ADRM1
AGT
AGTR1
AKT3
ANK3
APC
APELA
APLN
APOC1
ARHGAP44
ARRB1
ASCL1
ASIC1
ASIP
ASPM
ASTN1
ATOH1
ATP1A2
ATP2A1
ATP2A3
ATP6V0A4
ATP6V0D2
ATP6V1C2
ATP6V1E2
ATP6V1F
ATP6V1G2
AURKA
AVPR1B
AXIN2
AXL
BCHE
BCL2
BCL2L1
BDKRB1
BDKRB2
BDNF
BECN2
BID
BUB1B
BYSL

symbol
CACNA1A
CACNA1C
CACNA1D
CACNG4
CACNG5
CACNG7
CACNG8
CADPS
CALB1
CALB2
CALCA
CALCB
CALML3
CALML5
CALML6
CALY
CAMK2A
CAMK2B
CAMK2D
CAMK4
CAPN2
CARTPT
CASP7
CAV1
CCKAR
CCL5
CCNB1
CCND1
CCR2
CCR7
CDH4
CDK1
CDK5R2
CDKN3
CELSR1
CEP85L
CGA
CHAT
CHL1
CHRDL1
CHRFAM7A
CHRM2
CHRM4
CHRM5
CHRNA1
CHRNA3
CHRNA4
CHRNA6
CHRNA7
CHRNB2
CHRNB4
CHRND
CHRNG
CLCN1
CLDN1
CLDN19
CLU
CNIH3
CNR1

symbol
CNR2
CNTFR
CNTN2
CNTN4
CNTN6
CNTNAP1
COL3A1
COL4A6
COMT
COX7A1
CPLX1
CPNE1
CPNE5
CPNE9
CREB3L3
CRLF1
CRTAC1
CSF1
CSNK1A1L
CSNK1E
CTSG
CX3CR1
CXCL12
CXCL13
CXCL8
CXCR5
CYP2C18
CYP2C8
CYP4X1
CYSLTR1
CYSLTR2
DCDC2
DCLK1
DCX
DDIT4
DKK1
DKK4
DLG2
DLGAP2
DNAAF4
DNAH10
DNAH12
DNAH14
DNAH2
DNAH5
DNER
DRD2
DRD4
DRD5
DSCAML1
DUSP1
ECEL1
EDN2
EDN3
EDNRA
EDNRB
EFNA5
EMX1
EPAS1

symbol
EPHA3
EPHA4
EPHB2
EPHB3
ERBB4
EVX1
F2
F2RL1
F2RL2
FABP7
FAS
FEZF1
FEZF2
FGF12
FGF13
FGFR1
FKBP1B
FLRT1
FLRT2
FOSB
FOXG1
FRRS1L
FSTL4
FZD10
FZD3
FZD9
GAB1
GABARAP
GABRA3
GABRA4
GABRB1
GABRB3
GABRD
GABRE
GABRG2
GABRP
GABRR1
GAD1
GAL
GALR1
GALR3
GAP43
GCG
GCGR
GCHFR
GDF15
GDNF
GFRA1
GFRA2
GFRA3
GHR
GHRHR
GLP2R
GLRA3
GLRB
GLS2
GNA11
GNA13
GNAI1

symbol
GNAL
GNAO1
GNAQ
GNAZ
GNG10
GNG11
GNG12
GNG13
GNG2
GNG4
GNG7
GNG8
GNGT1
GPC6
GPER1
GPIHBP1
GPM6A
GPM6B
GPR143
GPR156
GPR171
GPR84
GPR88
GPX2
GPX3
GRIA1
GRIA2
GRIA3
GRIA4
GRID2
GRIK1
GRIK2
GRIK3
GRIK5
GRIN2A
GRIN2B
GRIN2D
GRIN3A
GRIN3B
GRM2
GRM4
GRM8
GRP
GRPR
GSG1L
GUCY1A1
GZMA
HCN1
HCRT
HMGA2
HOMER1
HPCA
HSP90AB1
HTR1D
HTR2B
HTR2C
HTR3A
HTR3C
HTR3E

symbol
HTR4
HTR7
ICAM5
IGF1
IGSF10
IGSF9
IL1A
IL6R
IL6ST
INHBA
INSL5
IRAK1
IRAK2
ITGA4
ITGB3
ITPR1
KCNA1
KCNA2
KCNJ14
KCNJ5
KCNJ9
KCNMB2
KCNQ1
KCNQ5
KIF14
KIF1A
KIF5A
KIF5C
KIRREL3
KISS1
KISS1R
KLC3
KNG1
KRT4
KRT6B
L1CAM
LEP
LEPR
LGI1
LHB
LHFPL4
LHX1
LIFR
LIMK1
LPAR1
LRRC7
LRRK2
LTB4R
LTB4R2
LY6E
LY6G6D
LY6H
LYNX1
LYPD6B
MAD2L1
MAG
MAGEA3
MAGEA4
MAGEA6

symbol
MAGI2
MAOA
MAOB
MAP1B
MAP1LC3C
MAP2
MAP2K6
MAPK10
MAPT
MATK
MC1R
MC3R
MCM2
MDGA1
MEF2C
MGLL
MIF
MMP1
MMP11
MMP13
MMP7
MMP9
MTNR1A
MYPN
NCAM1
NCAM2
NDN
NDNF
NDUFA11
NDUFA13
NDUFA4L2
NDUFS7
NEDD4L
NEFH
NEFL
NEFM
NEO1
NEUROD1
NEXMIF
NEXN
NGF
NGFR
NKX2-1
NKX6-1
NLGN1
NLGN3
NLGN4X
NLGN4Y
NMB
NMU
NMUR2
NOS1
NOX4
NPBWR1
NPFFR1
NPSR1
NPTX1
NPTX2
NPW

symbol
NPY
NPY1R
NPY2R
NPY4R
NPY4R2
NPY5R
NR3C1
NRCAM
NRG3
NRP2
NRXN1
NRXN2
NSG1
NSMF
NTF4
NTN1
NTNG1
NTRK3
NTSR1
NXPH2
NXPH3
NXPH4
OLFM1
OLFM3
OPRD1
OPRM1
OXT
OXTR
P2RX1
P2RX2
P2RX4
P2RX7
P2RY1
P2RY10
P2RY13
P2RY14
P2RY2
P2RY4
P2RY8
PAH
PAK6
PALLD
PDPN
PENK
PHOX2B
PIK3CD
PIK3CG
PIK3R2
PIK3R5
PINK1
PLA2G4B
PLA2G4D
PLA2G4E
PLCB4
PLCG1
PLCG2
PLCL1
PLD1
PLXNA1

symbol
PLXNA2
PLXNA3
PNOC
POU4F1
PPP1R1A
PPP2R2B
PPP2R2C
PPP2R3A
PPP2R3B
PPY
PRIMA1
PRKACB
PRKCB
PRKCG
PRKG1
PRKG2
PRKN
PRL
PRNP
PROKR1
PROX1
PRPH
PRSS1
PRSS2
PSMA2
PSMA6
PSMA7
PTGDR
PTGDR2
PTGER3
PTGER4
PTGFR
PTGS1
PTH1R
PTK2B
PTN
PYY
RAB39B
RAB3B
RAP1A
RASAL1
RBFOX3
RELN
RGS2
RIMS1
RIMS3
RIMS4
RIPK2
RLN2
ROBO2
RPH3A
RPS6KA5
RPS6KA6
RRM2
RTN4R
RXFP3
RYR1
RYR3
S1PR1

symbol
S1PR5
SCG2
SCN1B
SCN2B
SCTR
SDHD
SEMA3D
SEMA3E
SEMA3F
SEMA3G
SEMA6A
SEMA6D
SEPTIN5
SH3GL2
SHC4
SHH
SHISA7
SIGMAR1
SLC17A7
SLC17A8
SLC18A2
SLC18A3
SLC1A1
SLC1A2
SLC1A7
SLC22A2
SLC22A3
SLC28A2
SLC29A1
SLC2A4
SLC38A3
SLC38A5
SLC44A4
SLC4A8
SLC5A7
SLC6A1
SLC6A15
SLC6A16
SLC6A2
SLC6A3
SLC6A6
SLC6A9
SLC7A11
SLIT2
SLIT3
SLITRK3
SLURP1
SNAP25
SNCA
SNCAIP
SNCG
SNTG2
SORCS1
SORCS3
SOS2
SOX1
SOX10
SOX14
SOX2

symbol
SOX5
SOX9
SPTBN2
SPX
SST
SSTR1
SSTR2
SSTR3
SSTR5
STX1A
STXBP1
SULF1
SV2B
SYN2
SYN3
SYP
SYT10
SYT11
SYT12
SYT2
SYT4
SYT5
SYT7
SYT8
SYT9
TACR1
TACR2
TBX20
TENM1
TH
THRB
TLX3
TMEM108
TMEM140
TMEM35A
TMOD2
TNR
TOP2A
TP73
TPH1
TRAK2
TRAP1
TRPC1
TSHR
TTK
TUBA1A
TUBAL3
TUBB2A
TUBB2B
TUBB3
TUBB4A
TUBB6
TWIST1
TXNIP
TYRO3
UCHL1
UCN
UCN2
UCN3

symbol
UNC13C
UNC5C
UNC5D
UTS2B
VAMP2
VAX1
VCL
VEGFA
VGF
VIP
VIPR1
VIPR2
VWC2
WDR62
WNT1
WNT11
WNT16
WNT2
WNT2B
WNT3
WNT3A
WNT5A
WNT7A
WNT7B
WNT8B
WNT9A

Supplementary Table 4: GO enrichment terms summary table.

	ONTOLOGY	ID	Description	GeneRatio	BgRatio	pvalue	p.adjust	qvalue	geneID	Count	richFactor
GO:0050804	BP	GO:0050804	modulation of chemical synaptic transmission	140/666	487/18614	1.02105805009711e-88	3.41741002382206e-85	1.95928824410165e-85	ADORA3/ADRA1A/ADRA2A/ADRB2/AGT/ARHGAP44/ASIC1/ATP1A2/BCHE/CACNA1A/CACNG4/CACNG5/CACNG7/CACNG8/CALB1/CALB2/CAMK2A/CAMK2B/CCR2/CHRDL1/CHRM2/CHRNA3/CHRNA6/CHRNA7/CHRNB2/CHRNB4/CNR1/CNR2/CNTN2/CNTN4/CPLX1/CX3CR1/DKK1/DLGAP2/DRD2/DRD5/EPHA4/EPHB2/FRRS1L/GABRR1/GPER1/GRIA1/GRIA2/GRIA3/GRIA4/GRID2/GRIK1/GRIK2/GRIK3/GRIK5/GRIN2A/GRIN2B/GRIN2D/GRIN3A/GRIN3B/GRM2/GRM4/GRM8/HCN1/HCRT/HOMER1/KISS1/LGI1/LRRK2/MAP1B/MAPT/MEF2C/NEFH/NEFL/NGFR/NLGN1/NLGN3/NLGN4X/NLGN4Y/NMU/NPTX1/NPTX2/NPY/NPY5R/NRG3/NRXN1/NRXN2/NSG1/NSMF/NTNG1/OXT/OXTR/P2RX1/P2RY1/P2RY4/PINK1/PLCG1/PLCL1/PRKCB/PRKCG/PRKN/PRNP/PTK2B/PTN/RAP1A/RELN/RIMS1/RIMS3/RIMS4/SCTR/SEPTIN5/SHISA7/SLC18A3/SLC1A1/SLC4A8/SLC6A1/SLC6A6/SLC6A9/SLC7A11/SNAP25/SNCA/SNCAIP/SNCG/SORCS3/STX1A/STXBP1/SV2B/SYN3/SYP/SYT11/SYT12/SYT4/SYT7/TACR1/TACR2/TMEM108/TNR/TUBB2B/UCN/UNC13C/VAMP2/VGF/WNT3A/WNT5A/WNT7A	140	2.87474332648871

	ONTOLOGY	ID	Description	GeneRatio	BgRatio	pvalue	p.adjust	qvalue	geneID	Count	richFactor
GO:0099177	BP	GO:0099177	regulation of trans-synaptic signaling	140/666	488/18614	1.39060428232841e-88	3.41741002382206e-85	1.95928824410165e-85	ADORA3/ADRA1A/ADRA2A/ADRB2/AGT/ARHGAP44/ASIC1/ATP1A2/BCHE/CACNA1A/CACNG4/CACNG5/CACNG7/CACNG8/CALB1/CALB2/CAMK2A/CAMK2B/CCR2/CHRDL1/CHRM2/CHRNA3/CHRNA6/CHRNA7/CHRNB2/CHRNB4/CNR1/CNR2/CNTN2/CNTN4/CPLX1/CX3CR1/DKK1/DLGAP2/DRD2/DRD5/EPHA4/EPHB2/FRRS1L/GABRR1/GPER1/GRIA1/GRIA2/GRIA3/GRIA4/GRID2/GRIK1/GRIK2/GRIK3/GRIK5/GRIN2A/GRIN2B/GRIN2D/GRIN3A/GRIN3B/GRM2/GRM4/GRM8/HCN1/HCRT/HOMER1/KISS1/LGI1/LRRK2/MAP1B/MAPT/MEF2C/NEFH/NEFL/NGFR/NLGN1/NLGN3/NLGN4X/NLGN4Y/NMU/NPTX1/NPTX2/NPY/NPY5R/NRG3/NRXN1/NRXN2/NSG1/NSMF/NTNG1/OXT/OXTR/P2RX1/P2RY1/P2RY4/PINK1/PLCG1/PLCL1/PRKCB/PRKCG/PRKN/PRNP/PTK2B/PTN/RAP1A/RELN/RIMS1/RIMS3/RIMS4/SCTR/SEPTIN5/SHISA7/SLC18A3/SLC1A1/SLC4A8/SLC6A1/SLC6A6/SLC6A9/SLC7A11/SNAP25/SNCA/SNCAIP/SNCG/SORCS3/STX1A/STXBP1/SV2B/SYN3/SYP/SYT11/SYT12/SYT4/SYT7/TACR1/TACR2/TMEM108/TNR/TUBB2B/UCN/UNC13C/VAMP2/VGF/WNT3A/WNT5A/WNT7A	140	2.86885245901639
GO:0001505	BP	GO:0001505	regulation of neurotransmitter levels	83/666	211/18614	1.31798356297351e-64	2.15929640400493e-61	1.23797964844915e-61	ASIC1/ATP1A2/BCHE/CADPS/CAMK2A/CHAT/CHRNA3/CHRNB4/COMT/CPLX1/DRD2/DRD4/GAD1/GCHFR/GDNF/GPER1/GPM6B/GRIK5/GRM4/HCRT/ITGB3/LRRK2/MAOA/MAOB/MEF2C/NLGN1/NOS1/NPY/NRXN1/NRXN2/P2RX1/P2RX7/P2RY1/P2RY4/PAH/PRIMA1/PRKCB/PRKCG/PRKN/RAB3B/RAP1A/RIMS1/RIMS3/RIMS4/RPH3A/SEPTIN5/SLC17A8/SLC18A2/SLC18A3/SLC1A2/SLC1A7/SLC22A2/SLC22A3/SLC29A1/SLC44A4/SLC4A8/SLC5A7/SLC6A1/SLC6A2/SLC6A3/SNAP25/SNCA/SNCAIP/SNCG/STX1A/STXBP1/SV2B/SYN2/SYN3/SYP/SYT11/SYT12/SYT2/SYT4/SYT5/SYT7/SYT8/SYT9/TACR2/TH/UNC13C/VAMP2/WNT7A	83	3.93364928909953

	ONTOLOGY	ID	Description	GeneRatio	BgRatio	pvalue	p.adjust	qvalue	geneID	Count	richFactor
GO:0006836	BP	GO:0006836	neurotransmitter transport	81/666	202/18614	7.60599034240209e-64	9.34586063322657e-61	5.35822003858168e-61	ASIC1/ATP1A2/CADPS/CAMK2A/CHAT/CHRNA3/CHRNB4/CPLX1/DRD2/DRD4/GDNF/GPER1/GPM6B/GRIK5/GRM4/HCRT/ITGB3/LRRK2/MEF2C/NLGN1/NOS1/NPY/NRXN1/NRXN2/P2RX1/P2RX7/P2RY1/P2RY4/PRKCB/PRKCG/PRKN/RAB3B/RAP1A/RIMS1/RIMS3/RIMS4/RPH3A/SEPTIN5/SLC17A7/SLC17A8/SLC18A2/SLC18A3/SLC1A1/SLC1A2/SLC1A7/SLC22A2/SLC22A3/SLC28A2/SLC29A1/SLC4A8/SLC5A7/SLC6A1/SLC6A15/SLC6A16/SLC6A2/SLC6A3/SLC6A6/SLC6A9/SNAP25/SNCA/SNCAIP/SNCG/STX1A/STXBP1/SV2B/SYN2/SYN3/SYP/SYT11/SYT12/SYT2/SYT4/SYT5/SYT7/SYT8/SYT9/TACR2/TH/UNC13C/VAMP2/WNT7A	81	4.00990099009901
GO:0023061	BP	GO:0023061	signal release	114/666	483/18614	1.59435231829381e-61	1.56724832888281e-58	8.98543401278426e-59	ADCY5/ADCYAP1/ADORA3/ADRA2A/ADRA2C/AGT/AGTR1/ASIC1/CADPS/CAMK2A/CARTPT/CCKAR/CCL5/CGA/CHRNA3/CHRNA4/CHRNA6/CHRNB2/CHRNB4/CNR1/CPLX1/CXCL12/DRD2/EDN3/EDNRB/EFNA5/F2/F2RL1/F2RL2/FKBP1B/GAL/GALR1/GCG/GDNF/GHRHR/GNA11/GNAZ/GPER1/GRIK5/GRM2/GRM4/GRP/HCRT/HTR2C/IL1A/INHBA/ITPR1/KCNA2/KCNQ1/KISS1/LEP/LRRK2/MAOB/MAP2K6/MEF2C/MIF/NEUROD1/NKX6-1/NLGN1/NMB/NPY/NRXN1/NRXN2/OPRM1/OXT/OXTR/P2RX1/P2RX4/P2RX7/P2RY1/P2RY4/PINK1/PRKCB/PRKCG/PRKN/RAP1A/RIMS1/RIMS3/RIMS4/RPH3A/SEPTIN5/SLC18A2/SLC18A3/SLC44A4/SLC4A8/SNAP25/SNCA/SNCAIP/SNCG/SSTR5/STX1A/STXBP1/SV2B/SYN2/SYN3/SYP/SYT10/SYT11/SYT12/SYT2/SYT4/SYT5/SYT7/SYT8/SYT9/TACR1/TACR2/UCN/UCN3/UNC13C/VAMP2/VGF/VIP/WNT7A	114	2.36024844720497

	ONTOLOGY	ID	Description	GeneRatio	BgRatio	pvalue	p.adjust	qvalue	geneID	Count	richFactor
GO:0042391	BP	GO:0042391	regulation of membrane potential	108/666	433/18614	7.85672268971146e-61	6.43596533665531e-58	3.68990291936098e-58	ADRA1A/ADRB1/ADRB2/ANK3/ASIC1/ATP1A2/BCL2/BCL2L1/BID/CACNA1C/CACNA1D/CAMK2D/CAV1/CHRFAM7A/CHRNA1/CHRNA3/CHRNA4/CHRNA6/CHRNA7/CHRNB2/CHRNB4/CHRND/CHRNG/CLCN1/CLDN19/CNR1/CNR2/CNTNAP1/DRD2/DRD4/FGF12/FGF13/FKBP1B/FZD9/GABRA3/GABRA4/GABRB1/GABRB3/GABRD/GABRE/GABRG2/GABRP/GABRR1/GLRA3/GLRB/GNA11/GNAQ/GPER1/GPR88/GRIA1/GRIA3/GRIA4/GRID2/GRIK1/GRIK2/GRIK3/GRIK5/GRIN2A/GRIN2B/GRIN2D/GRIN3A/GRIN3B/HCN1/HCRT/HTR3A/HTR3C/HTR3E/KCNA1/KCNA2/KCNJ5/KCNJ9/KCNMB2/KCNQ1/LRRK2/MAPT/MEF2C/NEDD4L/NLGN1/NLGN3/NLGN4X/NRCAM/NRXN1/NTSR1/OPRD1/OPRM1/P2RX1/P2RX2/P2RX4/P2RX7/PHOX2B/PINK1/PRKN/PTK2B/RELN/RIMS1/RIMS3/RIMS4/SCN1B/SCN2B/SLC1A7/SLC29A1/SLC4A8/SNCA/STX1A/TACR1/TMEM108/UCN3/WNT7A	108	2.49422632794457
GO:0001764	BP	GO:0001764	neuron migration	74/666	176/18614	3.75473547297967e-60	2.63636069281358e-57	1.51149276107768e-57	ADGRG1/ADGRL3/ASCL1/ASPM/ASTN1/ATOH1/AXL/CAMK2A/CAMK2B/CCKAR/CDK5R2/CELSR1/CEP85L/CHL1/CNTN2/COL3A1/CXCL12/DCDC2/DCLK1/DCX/DDIT4/DNAAF4/DNER/DRD2/EVX1/FEZF1/FEZF2/FGF13/FGFR1/FLRT2/FOXG1/FZD3/GFRA3/GPM6A/IGSF10/KIRREL3/LHX1/MAP1B/MDGA1/MEF2C/NDN/NDNF/NEO1/NEXMIF/NKX2-1/NKX6-1/NRCAM/NRG3/NRP2/NSMF/NTN1/NTNG1/NTRK3/PHOX2B/PLXNA1/PLXNA3/POU4F1/PRKG1/RELN/SEMA3E/SEMA6A/SOX1/SOX14/TBX20/TLX3/TUBA1A/TUBB2B/TWIST1/TYRO3/UNC5C/UNC5D/VAX1/VEGFA/WDR62	74	4.20454545454546

	ONTOLOGY	ID	Description	GeneRatio	BgRatio	pvalue	p.adjust	qvalue	geneID	Count	richFactor
GO:0061564	BP	GO:0061564	axon development	109/666	479/18614	5.13090392771298e-57	3.15229910058866e-54	1.80729339664311e-54	ANK3/ATOH1/BCL2/BDNF/CCKAR/CDH4/CHL1/CHRNB2/CNR1/CNTN2/CNTN4/CNTN6/CNTNAP1/CRTAC1/CXCL12/DCLK1/DRD2/DSCAML1/EDN2/EDN3/EDNRA/EFNA5/EPHA3/EPHA4/EPHB2/EPHB3/EVX1/FEZF1/FEZF2/FGF13/FKBP1B/FLRT2/FOXG1/FSTL4/FZD3/GAP43/GDNF/GFRA3/GPM6B/HSP90AB1/IGSF9/ITGA4/KCNA1/KIF5A/KIF5C/L1CAM/LGI1/LHX1/LIMK1/MAG/MAP1B/MAP2/MAPT/MYPN/NCAM1/NCAM2/NDN/NEFH/NEFL/NEO1/NEXN/NGF/NGFR/NKX2-1/NKX6-1/NLGN3/NPTX1/NRCAM/NRP2/NRXN1/NTN1/NTNG1/OLFM1/PALLD/PHOX2B/PLXNA1/PLXNA2/PLXNA3/POU4F1/PRKG1/PTN/RELN/ROBO2/RPS6KA5/RTN4R/SCN1B/SEMA3D/SEMA3E/SEMA3F/SEMA3G/SEMA6A/SEMA6D/SHH/SLIT2/SLIT3/SLITRK3/STXBP1/TNR/TUBB2B/TUBB3/UCHL1/UNC5C/VAX1/VCL/VEGFA/WNT3/WNT3A/WNT5A/WNT7A	109	2.27557411273486
GO:0007409	BP	GO:0007409	axonogenesis	102/666	427/18614	1.97637148077106e-55	1.0793184253322e-52	6.18800754856624e-53	ANK3/ATOH1/BCL2/BDNF/CCKAR/CDH4/CHL1/CHRNB2/CNTN2/CNTN4/CNTN6/CNTNAP1/CXCL12/DCLK1/DRD2/DSCAML1/EDN2/EDN3/EDNRA/EFNA5/EPHA3/EPHA4/EPHB2/EPHB3/EVX1/FEZF1/FEZF2/FGF13/FLRT2/FOXG1/FSTL4/FZD3/GAP43/GDNF/GFRA3/HSP90AB1/IGSF9/ITGA4/KIF5A/KIF5C/L1CAM/LGI1/LHX1/LIMK1/MAG/MAP1B/MAP2/MAPT/MYPN/NCAM1/NDN/NEFH/NEFL/NEO1/NEXN/NGF/NGFR/NKX2-1/NKX6-1/NLGN3/NPTX1/NRCAM/NRP2/NRXN1/NTN1/NTNG1/OLFM1/PALLD/PHOX2B/PLXNA1/PLXNA2/PLXNA3/POU4F1/PRKG1/RELN/ROBO2/RPS6KA5/RTN4R/SCN1B/SEMA3D/SEMA3E/SEMA3F/SEMA3G/SEMA6A/SEMA6D/SHH/SLIT2/SLIT3/SLITRK3/STXBP1/TNR/TUBB2B/TUBB3/UCHL1/UNC5C/VAX1/VCL/VEGFA/WNT3/WNT3A/WNT5A/WNT7A	102	2.38875878220141

	ONTOLOGY	ID	Description	GeneRatio	BgRatio	pvalue	p.adjust	qvalue	geneID	Count	richFactor
GO:0060078	BP	GO:0060078	regulation of postsynaptic membrane potential	64/666	148/18614	5.22425244411849e-53	2.56772007628424e-50	1.4721393466216e-50	ADRB1/ADRB2/CHRFAM7A/CHRNA1/CHRNA3/CHRNA4/CHRNA6/CHRNA7/CHRNB2/CHRNB4/CHRND/CHRNG/DRD2/DRD4/GABRA3/GABRA4/GABRB1/GABRB3/GABRD/GABRE/GABRG2/GABRP/GABRR1/GLRA3/GLRB/GRIA1/GRIA3/GRID2/GRIK1/GRIK2/GRIK3/GRIK5/GRIN2A/GRIN2B/GRIN2D/GRIN3A/HCN1/HCRT/HTR3A/HTR3C/HTR3E/KCNA1/KCNA2/LRRK2/MEF2C/NLGN1/NLGN3/NLGN4X/NRXN1/NTSR1/OPRM1/P2RX1/P2RX2/P2RX4/P2RX7/PTK2B/RELN/RIMS1/SLC1A7/SLC29A1/SNCA/STX1A/TMEM108/WNT7A	64	4.32432432432432
GO:0007188	BP	GO:0007188	adenylate cyclase-modulating G protein-coupled receptor signaling pathway	74/666	234/18614	1.35403916610024e-49	6.05009318307516e-47	3.46867258148358e-47	ADCY2/ADCY3/ADCY5/ADCY9/ADCYAP1/ADCYAP1R1/ADGRG1/ADGRL3/ADRA1A/ADRA1B/ADRA1D/ADRA2A/ADRA2C/ADRB1/ADRB2/ADRB3/CACNA1D/CALCA/CALCB/CHRM2/CHRM4/CHRM5/CNR1/DRD2/DRD4/DRD5/EDNRA/GALR1/GALR3/GCG/GCGR/GHRHR/GLP2R/GNA11/GNA13/GNAI1/GNAL/GNAO1/GNAQ/GNAZ/GNG2/GPER1/GPR171/GRIK3/GRM2/GRM4/GRM8/HTR1D/ITGB3/LPAR1/MC1R/MC3R/MTNR1A/NPY1R/NPY2R/OPRD1/OPRM1/P2RY1/PRKACB/PTGDR2/PTGER3/PTGER4/PTGFR/PTH1R/RGS2/S1PR1/SCTR/SSTR2/TSHR/UCN2/UCN3/VIP/VIPR1/VIPR2	74	3.16239316239316
GO:0007218	BP	GO:0007218	neuropeptide signaling pathway	53/666	109/18614	2.11858995076518e-47	8.67739134000904e-45	4.97496955982314e-45	ADCYAP1/CALCA/CARTPT/CYSLTR1/CYSLTR2/ECEL1/GAL/GALR1/GALR3/GLRB/GPR143/GPR171/GPR84/GRP/GRPR/HCRT/KISS1R/LTB4R/LTB4R2/NMB/NMU/NMUR2/NPBWR1/NPFFR1/NPSR1/NPW/NPY/NPY1R/NPY2R/NPY4R2/NPY5R/NTSR1/NXPH2/NXPH3/NXPH4/OPRD1/OPRM1/PENK/PNOC/PPY/PROKR1/PTGDR2/PYY/RXFP3/SORCS1/SORCS3/SSTR1/SSTR2/SSTR3/SSTR5/TENM1/TYRO3/UCN	53	4.86238532110092
GO:0015844	BP	GO:0015844	monoamine transport	47/666	86/18614	2.65246611464194e-45	1.00283622718963e-42	5.74951561853965e-43	ADORA3/ADRA2A/ADRA2C/AGT/CARTPT/CHRM5/CHRNA4/CHRNA6/CHRNB2/CNR1/CXCL12/DRD2/DRD4/GDNF/GPM6B/GRM2/ITGB3/KCNA2/MAOB/NOS1/OXT/OXTR/P2RX1/P2RY1/PINK1/PRKN/RAB3B/SDHD/SLC18A2/SLC18A3/SLC22A2/SLC22A3/SLC6A2/SLC6A3/SNCA/SNCG/STX1A/SYT10/SYT11/SYT12/SYT2/SYT4/SYT5/SYT7/SYT8/SYT9/VIP	47	5.46511627906977

	ONTOLOGY	ID	Description	GeneRatio	BgRatio	pvalue	p.adjust	qvalue	geneID	Count	richFactor
GO:0015837	BP	GO:0015837	amine transport	51/666	107/18614	4.505558432301 9e-45	1.581772835340 27e-42	9.068706709227 2e-43	ADORA3/ADRA2A /ADRA2C/AGT/AT P1A2/CARTPT/CH RNA3/CHRNA4/C HRNA6/CHRNB2/ CNR1/CXCL12/DR D2/DRD4/GDNF/ GRM2/KCNA2/LE P/NPY5R/NTSR1/ OXT/OXTR/P2RX7 /P2RY1/PINK1/PR KN/RAB3B/RGS2/ SDHD/SLC17A8/S LC18A2/SLC18A3 /SLC22A2/SLC38 A3/SLC6A1/SNCA /SNCG/STX1A/ST XBP1/SYT10/SYT 11/SYT12/SYT2/S YT4/SYT5/SYT7/S YT8/SYT9/TACR2/ TH/VIP	51	4.766355140186 92
GO:0099565	BP	GO:0099565	chemical synaptic transmission, postsynaptic	55/666	132/18614	1.481444844499 12e-44	4.854200940475 45e-42	2.783037086823 96e-42	ADRB2/CHRFAM7 A/CHRNA1/CHRN A3/CHRNA4/CHR NA6/CHRNA7/CH RNB2/CHRNB4/C HRND/CHRNG/DR D2/DRD4/GABRA 3/GABRA4/GABR B1/GABRB3/GAB RD/GABRE/GABR G2/GABRP/GABR R1/GLRA3/GLRB/ GRID2/GRIK2/GRI K5/GRIN2A/GRIN 2B/GRIN2D/HCRT /HTR3A/HTR3C/H TR3E/LRRK2/MEF 2C/NLGN1/NLGN 3/NLGN4X/NRXN 1/NTSR1/OPRM1/ P2RX1/P2RX2/P2 RX4/P2RX7/PTK2 B/RELN/RIMS1/SL C1A7/SLC29A1/S NCA/STX1A/TME M108/WNT7A	55	4.166666666666 67
GO:0060079	BP	GO:0060079	excitatory postsynaptic potential	53/666	124/18614	1.125560489614 59e-43	3.457581129034 82e-41	1.982319362301 49e-41	ADRB2/CHRFAM7 A/CHRNA1/CHRN A3/CHRNA4/CHR NA6/CHRNA7/CH RNB2/CHRNB4/C HRND/CHRNG/DR D2/GABRA3/GAB RA4/GABRB1/GA BRB3/GABRD/GA BRE/GABRG2/GA BRP/GABRR1/GL RA3/GLRB/GRID2 /GRIK2/GRIK5/GR IN2A/GRIN2B/GRI N2D/HCRT/HTR3 A/HTR3C/HTR3E/ LRRK2/MEF2C/NL GN1/NLGN3/NLG N4X/NRXN1/OPR M1/P2RX1/P2RX2 /P2RX4/P2RX7/PT K2B/RELN/RIMS1/ SLC1A7/SLC29A1 /SNCA/STX1A/TM EM108/WNT7A	53	4.274193548387 1
GO:0051952	BP	GO:0051952	regulation of amine transport	48/666	98/18614	3.341429050691 73e-43	9.660661049499 92e-41	5.538703138515 02e-41	ADORA3/ADRA2A /ADRA2C/AGT/AT P1A2/CARTPT/CH RNA3/CHRNA4/C HRNA6/CHRNB2/ CNR1/CXCL12/DR D2/DRD4/GDNF/ GRM2/KCNA2/LE P/NPY5R/NTSR1/ OXT/OXTR/P2RX7 /P2RY1/PINK1/PR KN/RAB3B/RGS2/ SDHD/SLC17A8/S LC18A3/SLC38A3 /SLC6A1/SNCA/S NCG/STX1A/STXB P1/SYT10/SYT11/ SYT12/SYT2/SYT4 /SYT5/SYT7/SYT8/ SYT9/TACR2/VIP	48	4.897959183673 47

	ONTOLOGY	ID	Description	GeneRatio	BgRatio	pvalue	p.adjust	qvalue	geneID	Count	richFactor
GO:0007411	BP	GO:0007411	axon guidance	67/666	225/18614	4.965499948210 29e-43	1.284496433971 24e-40	7.364345352553 43e-41	ATOH1/BDNF/CDH4/CHL1/CNTN2/CNTN4/CNTN6/CXCL12/DSCAML1/EDN3/EDNRA/EFNA5/EPHA3/EPHA4/EPHB2/EPHB3/EVX1/FEZF1/FEZF2/FLRT2/FOXG1/FZD3/GAP43/GDNF/GFRA3/IGSF9/KIF5A/KIF5C/L1CAM/LGI1/LHX1/MYPN/NCAM1/NEO1/NEXN/NGFR/NKX2-1/NRCAM/NRP2/NRXN1/NTN1/PALLD/PLXNA1/PLXNA2/PLXNA3/RELN/ROBO2/RPS6KA5/SCN1B/SEMA3D/SEMA3E/SEMA3F/SEMA3G/SEMA6A/SEMA6D/SHH/SLIT2/SLIT3/TNR/TUBB2B/TUBB3/UNC5C/VAX1/VEGFA/WNT3/WNT3A/WNT5A	67	2.977777777777 78
GO:0097485	BP	GO:0097485	neuron projection guidance	67/666	225/18614	4.965499948210 29e-43	1.284496433971 24e-40	7.364345352553 43e-41	ATOH1/BDNF/CDH4/CHL1/CNTN2/CNTN4/CNTN6/CXCL12/DSCAML1/EDN3/EDNRA/EFNA5/EPHA3/EPHA4/EPHB2/EPHB3/EVX1/FEZF1/FEZF2/FLRT2/FOXG1/FZD3/GAP43/GDNF/GFRA3/IGSF9/KIF5A/KIF5C/L1CAM/LGI1/LHX1/MYPN/NCAM1/NEO1/NEXN/NGFR/NKX2-1/NRCAM/NRP2/NRXN1/NTN1/PALLD/PLXNA1/PLXNA2/PLXNA3/RELN/ROBO2/RPS6KA5/SCN1B/SEMA3D/SEMA3E/SEMA3F/SEMA3G/SEMA6A/SEMA6D/SHH/SLIT2/SLIT3/TNR/TUBB2B/TUBB3/UNC5C/VAX1/VEGFA/WNT3/WNT3A/WNT5A	67	2.977777777777 78
GO:0060560	BP	GO:0060560	developmental growth involved in morphogenesis	68/666	234/18614	6.784402148121 36e-43	1.667266827900 82e-40	9.558865552905 73e-41	AURKA/BDNF/CACNG7/CDH4/CPNE1/CPNE5/CPNE9/CSF1/CXCL12/DCLK1/EDN2/EDN3/EDNRA/EFNA5/EMX1/FGF13/FLRT1/FSTL4/HSP90AB1/ITGA4/L1CAM/LHX1/LIMK1/MAG/MAGI2/MAP1B/MAP2/MAPT/NDN/NEDD4L/NGF/NKX6-1/NLGN3/NRCAM/NRP2/NTN1/OLFM1/PAK6/PLXNA1/PLXNA3/PRKG1/PRKN/RASAL1/RIMS1/RTN4R/S1PR1/SEMA3D/SEMA3E/SEMA3F/SEMA3G/SEMA6A/SEMA6D/SH3GL2/SHH/SLIT2/SLIT3/SOX9/SYT2/SYT4/TMEM108/TNR/VCL/VEGFA/WNT11/WNT3/WNT3A/WNT5A/WNT7B	68	2.905982905982 91
GO:0051937	BP	GO:0051937	catecholamine transport	41/666	73/18614	2.591468258030 75e-40	6.065269756295 78e-38	3.477373697618 21e-38	ADORA3/ADRA2A/ADRA2C/AGT/CARTPT/CHRM5/CHRNA4/CHRNA6/CHRNB2/CNR1/CXCL12/DRD2/DRD4/GDNF/GRM2/KCNA2/OXT/OXTR/P2RY1/PINK1/PRKN/RAB3B/SDHD/SLC18A2/SLC22A2/SLC22A3/SLC6A2/SLC6A3/SNCA/SNCG/STX1A/SYT10/SYT11/SYT12/SYT2/SYT4/SYT5/SYT7/SYT8/SYT9/VIP	41	5.616438356164 38

	ONTOLOGY	ID	Description	GeneRatio	BgRatio	pvalue	p.adjust	qvalue	geneID	Count	richFactor
GO:0007269	BP	GO:0007269	neurotransmitter secretion	53/666	141/18614	3.70775594012093e-40	7.92331323725841e-38	4.54263736920079e-38	ASIC1/CADPS/CAMK2A/CHRNA3/CHRNB4/CPLX1/GPER1/GRIK5/GRM4/HCRT/LRRK2/MEF2C/NLGN1/NPY/NRXN1/NRXN2/P2RX1/P2RX7/P2RY1/P2RY4/PRKCB/PRKCG/PRKN/RAP1A/RIMS1/RIMS3/RIMS4/RPH3A/SEPTIN5/SLC18A3/SLC4A8/SNAP25/SNCA/SNCAIP/SNCG/STX1A/STXBP1/SV2B/SYN2/SYN3/SYP/SYT11/SYT12/SYT2/SYT4/SYT5/SYT7/SYT8/SYT9/TACR2/UNC13C/VAMP2/WNT7A	53	3.75886524822695
GO:0099643	BP	GO:0099643	signal release from synapse	53/666	141/18614	3.70775594012093e-40	7.92331323725841e-38	4.54263736920079e-38	ASIC1/CADPS/CAMK2A/CHRNA3/CHRNB4/CPLX1/GPER1/GRIK5/GRM4/HCRT/LRRK2/MEF2C/NLGN1/NPY/NRXN1/NRXN2/P2RX1/P2RX7/P2RY1/P2RY4/PRKCB/PRKCG/PRKN/RAP1A/RIMS1/RIMS3/RIMS4/RPH3A/SEPTIN5/SLC18A3/SLC4A8/SNAP25/SNCA/SNCAIP/SNCG/STX1A/STXBP1/SV2B/SYN2/SYN3/SYP/SYT11/SYT12/SYT2/SYT4/SYT5/SYT7/SYT8/SYT9/TACR2/UNC13C/VAMP2/WNT7A	53	3.75886524822695
GO:0048588	BP	GO:0048588	developmental cell growth	65/666	231/18614	4.6584028015648e-40	9.54002073737124e-38	5.46953697359165e-38	ADRA1A/AGT/AURKA/BDNF/CACNG7/CDH4/CPNE1/CPNE5/CPNE9/CXCL12/DCLK1/EDN2/EDN3/EDNRA/EFNA5/EMX1/FGF13/FLRT1/FSTL4/HSP90AB1/IGF1/ITGA4/L1CAM/LIMK1/MAG/MAP1B/MAP2/MAPT/NDN/NEDD4L/NGF/NKX6-1/NLGN3/NRCAM/NRP2/NTN1/OLFM1/PAK6/PLXNA1/PLXNA3/PRKG1/PRKN/RASAL1/RGS2/RIMS1/RTN4R/SEMA3D/SEMA3E/SEMA3F/SEMA3G/SEMA6A/SEMA6D/SH3GL2/SLIT2/SLIT3/SOX9/SYT2/SYT4/TMEM108/TNR/VCL/VEGFA/WNT3/WNT3A/WNT5A	65	2.81385281385281

	ONTOLOGY	ID	Description	GeneRatio	BgRatio	pvalue	p.adjust	qvalue	geneID	Count	richFactor
GO:0050808	BP	GO:0050808	synapse organization	89/666	466/18614	7.62941236525471e-40	1.49994247100908e-37	8.59955237969973e-38	ADGRL3/ANK3/ARHGAP44/BDNF/CAMK2B/CHRDL1/CHRNA1/CHRNA7/CHRNB2/CNTN2/CNTNAP1/CX3CR1/DKK1/DNER/DRD2/EFNA5/EPHA4/EPHB2/EPHB3/ERBB4/FGF13/FLRT2/FRRS1L/FZD9/GABRB3/GABRG2/GAP43/GDNF/GLRB/GPM6A/GRID2/GRIN2B/HOMER1/ICAM5/IGSF9/ITGB3/KIF1A/KIRREL3/L1CAM/LHFPL4/LRRK2/MAP1B/MAPT/MDGA1/MEF2C/NEFH/NEFL/NLGN1/NLGN3/NLGN4X/NLGN4Y/NPTX1/NRCAM/NRG3/NRP2/NRXN1/NRXN2/NTN1/NTNG1/NTRK3/OXT/OXTR/P2RX2/POU4F1/PRNP/PTK2B/RAB39B/RELN/ROBO2/RPS6KA5/SEMA3E/SEMA3F/SHISA7/SLC18A3/SLC1A1/SLC6A1/SLC7A11/SLITRK3/SNCA/SNCG/SPTBN2/TMEM108/TNR/TUBA1A/UNC13C/WNT3A/WNT5A/WNT7A/WNT7B	89	1.90987124463519
GO:0099003	BP	GO:0099003	vesicle-mediated transport in synapse	62/666	220/18614	2.79868965326135e-38	5.29059986376136e-36	3.03323570922293e-36	ATP6V0A4/ATP6V1F/ATP6V1G2/CADPS/CALY/CPLX1/DRD4/GRIK5/GSG1L/HPCA/ITGB3/LPAR1/LRRK2/NLGN1/NLGN3/NLGN4X/NLGN4Y/NPY/NSG1/P2RX1/P2RX7/P2RY1/P2RY4/PLD1/PRKCB/PRKCG/PRKN/RAB3B/RAP1A/RIMS1/RIMS3/RIMS4/RPH3A/SEPTIN5/SH3GL2/SLC17A7/SLC17A8/SLC18A2/SLC2A4/SLC4A8/SNAP25/SNCA/SNCG/STX1A/STXBP1/SV2B/SYN2/SYN3/SYP/SYT11/SYT12/SYT2/SYT4/SYT5/SYT7/SYT8/SYT9/TH/UNC13C/VAMP2/WNT3A/WNT7A	62	2.81818181818182
GO:0051588	BP	GO:0051588	regulation of neurotransmitter transport	44/666	99/18614	2.49347786984538e-37	4.53905323344076e-35	2.60235487624799e-35	ASIC1/ATP1A2/CAMK2A/CHRNA3/CHRNB4/CPLX1/DRD2/DRD4/GDNF/GPER1/GPM6B/HCRT/ITGB3/LRRK2/MEF2C/NLGN1/NOS1/NPY/P2RX1/P2RY1/P2RY4/PRKCB/PRKCG/PRKN/RAB3B/RAP1A/RIMS1/RIMS3/RIMS4/SEPTIN5/SLC17A8/SLC18A3/SLC4A8/SNCA/SNCAIP/SNCG/STX1A/STXBP1/SV2B/SYP/SYT11/SYT4/TACR2/WNT7A	44	4.44444444444444

	ONTOLOGY	ID	Description	GeneRatio	BgRatio	pvalue	p.adjust	qvalue	geneID	Count	richFactor
GO:0035249	BP	GO:0035249	synaptic transmission, glutamatergic	46/666	112/18614	4.634407527179 69e-37	8.135040355745 77e-35	4.664025921150 38e-35	ATP1A2/CACNG4/CACNG5/CACNG7/CACNG8/CCR2/CNR1/DKK1/DRD2/FRRS1L/GRIA1/GRIA2/GRIA3/GRIA4/GRID2/GRIK1/GRIK2/GRIK3/GRIK5/GRIN2A/GRIN2B/GRIN2D/GRIN3A/GRIN3B/GRM2/GRM4/GRM8/HCN1/HOMER1/LRRK2/MEF2C/NLGN1/NLGN3/NR3C1/NRXN1/OXTR/P2RX1/PRKN/PTK2B/RELN/SLC17A7/SLC17A8/STXBP1/TNR/UCN/UNC13C	46	4.107142857142 86
GO:1902074	BP	GO:1902074	response to salt	78/666	383/18614	5.527203887238 97e-37	9.367657622682 6e-35	5.370716808035 83e-35	ASCL1/CACNG4/CAMK2D/CAV1/CCND1/CHRM2/CHRM4/CHRM5/CHRNA3/CHRNA7/CHRNB2/CHRND/CLDN1/CNR1/CPNE1/CPNE5/CPNE9/DLG2/DRD2/DRD4/DRD5/EDNRA/EDNRB/FOSB/GHR/GNA11/GNAQ/GRM2/HOMER1/HPCA/IL1A/ITPR1/KCNMB2/LEP/LY6G6D/LY6H/MEF2C/NEFL/NLGN1/NPTX1/OPRM1/OXT/OXTR/P2RX1/P2RX2/P2RX4/P2RX7/P2RY1/P2RY2/P2RY4/PENK/PLCG2/PRKCB/PRKCG/PTK2B/RASAL1/RYR1/RYR3/SHH/SLC1A1/SLC1A2/SLC6A1/SLC6A3/SYT10/SYT11/SYT12/SYT2/SYT4/SYT5/SYT7/SYT8/SYT9/TACR1/TH/TRPC1/TUBA1A/TXNIP/WNT5A	78	2.036553524804 18
GO:1990138	BP	GO:1990138	neuron projection extension	54/666	172/18614	3.759454648189 7e-36	6.159239865284 12e-34	3.531249155510 11e-34	AURKA/CACNG7/CDH4/CPNE1/CPNE5/CPNE9/CXCL12/DCLK1/EDN2/EDN3/EDNRA/EMX1/FLRT1/HSP90AB1/ITGA4/L1CAM/LIMK1/MAG/MAP1B/MAP2/MAPT/NDN/NEDD4L/NKX6-1/NLGN3/NRCAM/NRP2/NTN1/OLFM1/PAK6/PLXNA1/PLXNA3/PRKN/RASAL1/RIMS1/RTN4R/SEMA3D/SEMA3E/SEMA3F/SEMA3G/SEMA6A/SEMA6D/SH3GL2/SLIT2/SLIT3/SYT2/SYT4/TMEM108/TNR/VCL/VEGFA/WNT3/WNT3A/WNT5A	54	3.139534883720 93
GO:0099504	BP	GO:0099504	synaptic vesicle cycle	56/666	198/18614	9.776192287181 14e-35	1.549999519080 49e-32	8.886542191098 11e-33	ATP6V0A4/ATP6V1F/ATP6V1G2/CADPS/CPLX1/GRIK5/LPAR1/LRRK2/NLGN1/NLGN3/NLGN4X/NLGN4Y/NPY/P2RX1/P2RX7/P2RY1/P2RY4/PLD1/PRKCB/PRKCG/PRKN/RAB3B/RAP1A/RIMS1/RIMS3/RIMS4/RPH3A/SEPTIN5/SH3GL2/SLC17A7/SLC17A8/SLC18A2/SLC2A4/SLC4A8/SNAP25/SNCA/SNCG/STX1A/STXBP1/SV2B/SYN2/SYN3/SYP/SYT11/SYT12/SYT2/SYT4/SYT5/SYT7/SYT8/SYT9/TH/UNC13C/VAMP2/WNT3A/WNT7A	56	2.828282828282 83

	ONTOLOGY	ID	Description	GeneRatio	BgRatio	pvalue	p.adjust	qvalue	geneID	Count	richFactor
GO:0007200	BP	GO:0007200	phospholipase C-activating G protein-coupled receptor signaling pathway	44/666	113/18614	2.772083319613e-34	4.25774672371809e-32	2.44107468638289e-32	ADRA1A/ADRA1B/ADRA1D/ADRA2A/AGT/AGTR1/CALCA/CCKAR/CHRM2/CX3CR1/DRD2/DRD5/EDNRB/F2/F2RL1/F2RL2/GNA11/GNAQ/GNG13/GRP/GRPR/HCRT/HOMER1/HTR2B/HTR2C/ITPR1/KISS1/LPAR1/LTB4R/MC3R/NMUR2/OPRD1/OPRM1/P2RY1/P2RY10/P2RY2/P2RY4/P2RY8/PRKCB/PTGER3/PTH1R/S1PR1/TACR1/VIP	44	3.89380530973451
GO:0050432	BP	GO:0050432	catecholamine secretion	34/666	59/18614	3.80922455224896e-34	5.67343596191019e-32	3.25272539916123e-32	ADORA3/ADRA2A/ADRA2C/AGT/CARTPT/CHRNA4/CHRNA6/CHRNB2/CNR1/CXCL12/DRD2/GDNF/GRM2/KCNA2/OXT/OXTR/P2RY1/PINK1/PRKN/SDHD/SLC18A2/SNCA/SNCG/STX1A/SYT10/SYT11/SYT12/SYT2/SYT4/SYT5/SYT7/SYT8/SYT9/VIP	34	5.76271186440678
GO:0007204	BP	GO:0007204	positive regulation of cytosolic calcium ion concentration	53/666	179/18614	4.84657076340896e-34	7.00614567710443e-32	4.01680183704204e-32	ADCY5/ADCYAP1/ADCYAP1R1/ADRA1A/ADRA1B/ADRA1D/AGT/AGTR1/AVPR1B/BCL2/BDKRB1/BDKRB2/CACNA1A/CACNA1C/CALCA/CAV1/CXCL13/CYSLTR1/DRD2/EDN2/EDNRA/EDNRB/F2RL1/F2RL2/FKBP1B/GALR1/GNA13/GPER1/HCRT/HTR2C/KISS1/KNG1/LPAR1/NMB/NMUR2/NOS1/OXT/OXTR/P2RX2/P2RX4/P2RX7/P2RY1/P2RY10/P2RY4/P2RY8/PIK3CG/PTGDR/PTGER3/PTGER4/PTGFR/PTK2B/S1PR1/TACR1	53	2.9608938547486
GO:0090066	BP	GO:0090066	regulation of anatomical structure size	84/666	492/18614	8.04510064301655e-34	1.1297619902979e-31	6.47721335980611e-32	ADRA1A/ADRA1D/ADRA2A/ADRA2C/ADRB1/ADRB2/ADRB3/AGT/AGTR1/AKT3/ATP1A2/AVPR1B/BDKRB2/BDNF/CALCA/CAV1/CCR7/CDH4/CNTN2/CXCL12/DRD5/EDN2/EDN3/EDNRA/EDNRB/EFNA5/F2RL1/FGF13/FSTL4/GPER1/GUCY1A1/HTR1D/HTR2B/HTR7/KCNMB2/KNG1/L1CAM/LEP/LIMK1/LRRK2/MAG/MAP1B/MAP2/MAPT/NEFL/NGF/NKX6-1/NOS1/NRCAM/NTN1/OLFM1/OXTR/P2RX1/P2RX7/P2RY1/P2RY2/PIK3R2/PLXNA3/PRKG1/PTK2B/RAB3B/RGS2/RTN4R/SCTR/SEMA3D/SEMA3E/SEMA3F/SEMA3G/SEMA6A/SEMA6D/SLIT2/SPTBN2/TACR1/TENM1/TMOD2/TNR/UCN/UTS2B/VEGFA/WNT3/WNT3A/WNT5A/WNT7A/WNT7B	84	1.70731707317073

	ONTOLOGY	ID	Description	GeneRatio	BgRatio	pvalue	p.adjust	qvalue	geneID	Count	richFactor
GO:0050433	BP	GO:0050433	regulation of catecholamine secretion	33/666	57/18614	2.87087086898235e-33	3.91953620029118e-31	2.2471699755163e-31	ADORA3/ADRA2A/ADRA2C/AGT/CARTPT/CHRNA4/CHRNA6/CHRNB2/CNR1/CXCL12/DRD2/GDNF/GRM2/KCNA2/OXT/OXTR/P2RY1/PINK1/PRKN/SDHD/SNCA/SNCG/STX1A/SYT10/SYT11/SYT12/SYT2/SYT4/SYT5/SYT7/SYT8/SYT9/VIP	33	5.78947368421053
GO:0030900	BP	GO:0030900	forebrain development	75/666	397/18614	2.95596626916881e-33	3.92664167917965e-31	2.25124372761448e-31	ADGRG1/ASCL1/ASPM/ATOH1/ATP1A2/AXL/CCKAR/CDK5R2/CHRNB2/CNTN2/COL3A1/CXCL12/DCLK1/DKK1/DNAH5/DRD2/EMX1/EPHB2/EPHB3/ERBB4/FEZF1/FEZF2/FGF13/FOXG1/GHRHR/INHBA/KCNA1/KCNA2/KIF14/KIRREL3/LHX1/LPAR1/LRRK2/MDGA1/NDNF/NEFL/NEUROD1/NKX2-1/NPY/NRG3/NRP2/OXTR/PLXNA1/PLXNA3/POU4F1/PRKG1/PROX1/RELN/ROBO2/RTN4R/SEMA3E/SHH/SLC1A2/SLC6A3/SLC7A11/SLIT2/SOX1/SOX2/SSTR1/SSTR2/SSTR3/TH/TMEM108/TNR/TUBA1A/TUBB2A/TUBB2B/TYRO3/WDR62/WNT1/WNT2B/WNT3A/WNT5A/WNT7A/WNT7B	75	1.88916876574307
GO:0007611	BP	GO:0007611	learning or memory	62/666	276/18614	4.6661423161024e-32	6.03528670622191e-30	3.46018365102662e-30	ADCY3/AGT/ASIC1/ATP1A2/BCHE/BDNF/CALB1/CAMK4/CHRNA7/CHRNB2/CNR1/CNTN2/CX3CR1/DKK1/DNAAF4/DRD2/DRD5/EPHB2/FGF13/FZD9/GALR3/GPR88/GRIA1/GRIN2A/GRIN2B/GRPR/MAPT/MEF2C/NGF/NLGN3/NLGN4X/NLGN4Y/NPTX2/NRXN1/NRXN2/NTF4/NTSR1/OXT/OXTR/PAK6/PRKCG/PRKN/PRNP/PTN/RELN/SHISA7/SLC1A1/SLC2A4/SLC6A1/SLC7A11/SNAP25/SORCS3/SYT11/SYT4/TACR1/TACR2/TH/TMOD2/TNR/TUBA1A/UCN/VIP	62	2.2463768115942
GO:0007631	BP	GO:0007631	feeding behavior	41/666	109/18614	2.66313558677395e-31	3.35623369461384e-29	1.92421429576083e-29	ADRB3/AGT/APLN/ASIP/CALCA/CARTPT/CNR1/CNTFR/CNTN2/DRD2/GAL/GALR3/GCG/GDF15/GPR171/HCRT/HTR2C/INSL5/LEP/LEPR/MC1R/MC3R/NMU/NMUR2/NPSR1/NPW/NPY/NPY1R/NPY5R/OPRD1/OPRM1/OXT/OXTR/P2RY1/POU4F1/PPY/PYY/TACR1/TH/UCHL1/UCN	41	3.76146788990826

	ONTOLOGY	ID	Description	GeneRatio	BgRatio	pvalue	p.adjust	qvalue	geneID	Count	richFactor
GO:0016049	BP	GO:0016049	cell growth	81/666	495/18614	2.83421081758383e-31	3.48253654210613e-29	1.99662693649261e-29	ADRA1A/AGT/AGTR1/AURKA/BCL2/BDKRB1/BDNF/CACNG7/CAMK2D/CDH4/CPNE1/CPNE5/CPNE9/CXCL12/DCLK1/EDN2/EDN3/EDNRA/EFNA5/EMX1/F2/FGF13/FLRT1/FSTL4/GNG4/HSP90AB1/IGF1/INHBA/ITGA4/KIF14/L1CAM/LGI1/LIMK1/MAG/MAP1B/MAP2/MAPT/NDN/NDUFA13/NEDD4L/NGF/NKX6-1/NLGN3/NRCAM/NRG3/NRP2/NTN1/OLFM1/PAK6/PLXNA1/PLXNA3/PRKG1/PRKN/PRSS2/PTK2B/RASAL1/RGS2/RIMS1/RTN4R/SEMA3D/SEMA3E/SEMA3F/SEMA3G/SEMA6A/SEMA6D/SH3GL2/SLC44A4/SLIT2/SLIT3/SOX9/SYT2/SYT4/TMEM108/TNR/UCN/VCL/VEGFA/WNT11/WNT3/WNT3A/WNT5A	81	1.63636363636364
GO:0050806	BP	GO:0050806	positive regulation of synaptic transmission	49/666	170/18614	6.21922777232989e-31	7.45548890268327e-29	4.27442175777333e-29	ADRA1A/CACNG4/CACNG5/CACNG7/CACNG8/CALB1/CALB2/CCR2/CHRNA7/CX3CR1/DRD2/EPHA4/EPHB2/GPER1/GRIK2/GRIN2A/GRIN2B/GRIN2D/KISS1/LGI1/NLGN1/NLGN3/NMU/NRXN1/NSG1/OXT/OXTR/PINK1/PRKCG/PRNP/PTK2B/PTN/RELN/RIMS1/RIMS3/RIMS4/SHISA7/SLC18A3/SLC1A1/SLC4A8/SNAP25/SNCA/STX1A/STXBP1/SYT12/TACR1/TACR2/TNR/VAMP2	49	2.88235294117647
GO:0015872	BP	GO:0015872	dopamine transport	30/666	51/18614	1.20968055974694e-30	1.41561427408482e-28	8.11607733945506e-29	CHRM5/CHRNA4/CHRNA6/CHRNB2/CNR1/CXCL12/DRD2/DRD4/GDNF/GRM2/KCNA2/PINK1/PRKN/RAB3B/SLC18A2/SLC22A2/SLC22A3/SLC6A2/SLC6A3/SNCA/SNCG/SYT10/SYT11/SYT12/SYT2/SYT4/SYT5/SYT7/SYT8/SYT9	30	5.88235294117647
GO:0006936	BP	GO:0006936	muscle contraction	67/666	349/18614	3.3799017860243e-30	3.78000122952794e-28	2.16717102135167e-28	ADRA1A/ADRA1B/ADRA2A/ADRA2C/ADRB2/AGT/ATP1A2/ATP2A1/BDKRB2/CACNA1C/CACNA1D/CALCA/CAMK2D/CAV1/CHRM2/CHRNA1/CHRNA3/CHRNB2/CHRNB4/CHRND/CHRNG/CLCN1/DRD2/EDN2/EDN3/EDNRA/EDNRB/FGF12/FGF13/FKBP1B/GDNF/GNAO1/GPER1/GUCY1A1/HOMER1/HTR1D/HTR2B/HTR7/KCNA1/KCNJ5/KCNQ1/LTB4R/MAP2K6/NEDD4L/NMU/NMUR2/NOS1/OXT/OXTR/P2RX1/P2RX2/P2RX4/PIK3CG/PRKG1/PTGER3/RGS2/RYR1/SCN1B/SCN2B/SPX/SSTR2/SULF1/TACR1/TACR2/TBX20/TMOD2/UCN	67	1.91977077363897

	ONTOLOGY	ID	Description	GeneRatio	BgRatio	pvalue	p.adjust	qvalue	geneID	Count	richFactor
GO:0050890	BP	GO:0050890	cognition	64/666	317/18614	3.38392785552858e-30	3.78000122952794e-28	2.16717102135167e-28	ADCY3/AGT/ASIC1/ATP1A2/BCHE/BDNF/CALB1/CAMK4/CHL1/CHRNA4/CHRNA7/CHRNB2/CNR1/CNTN2/CX3CR1/DKK1/DNAAF4/DRD2/DRD5/EPHB2/FGF13/FZD9/GALR3/GPR88/GRIA1/GRIN2A/GRIN2B/GRPR/MAPT/MEF2C/NGF/NLGN3/NLGN4X/NLGN4Y/NPTX2/NRXN1/NRXN2/NTF4/NTSR1/OXT/OXTR/PAK6/PRKCG/PRKN/PRNP/PTN/RELN/SHISA7/SLC1A1/SLC2A4/SLC6A1/SLC7A11/SNAP25/SORCS3/SYT11/SYT4/TACR1/TACR2/TH/TMOD2/TNR/TUBA1A/UCN/VIP	64	2.01892744479495
GO:0046928	BP	GO:0046928	regulation of neurotransmitter secretion	35/666	82/18614	3.63560203688381e-29	3.97088533584087e-27	2.27660974333052e-27	ASIC1/CAMK2A/CHRNA3/CHRNB4/CPLX1/GPER1/HCRT/LRRK2/MEF2C/NLGN1/NPY/P2RX1/P2RY1/P2RY4/PRKCB/PRKCG/PRKN/RAP1A/RIMS1/RIMS3/RIMS4/SEPTIN5/SLC18A3/SLC4A8/SNCA/SNCAIP/SNCG/STX1A/STXBP1/SV2B/SYP/SYT11/SYT4/TACR2/WNT7A	35	4.26829268292683
GO:0003018	BP	GO:0003018	vascular process in circulatory system	57/666	265/18614	1.74307565217939e-28	1.86243844140472e-26	1.06778341438998e-26	ADRA1A/ADRA1D/ADRA2A/ADRA2C/ADRB1/ADRB2/ADRB3/AGT/AGTR1/ATP1A2/ATP2A3/AVPR1B/BDKRB2/CALCA/CAV1/DRD5/EDN2/EDN3/EDNRA/EDNRB/F2RL1/GPER1/GUCY1A1/HTR1D/HTR2B/HTR7/KCNMB2/KNG1/LEP/LEPR/NOS1/OXTR/P2RX1/P2RY1/P2RY2/PRKG1/RGS2/SH3GL2/SLC1A1/SLC1A2/SLC22A2/SLC22A3/SLC28A2/SLC29A1/SLC2A4/SLC38A3/SLC38A5/SLC4A8/SLC6A1/SLC6A6/SLC6A9/SLIT2/TACR1/TACR2/UCN/UTS2B/VEGFA	57	2.15094339622642
GO:0031644	BP	GO:0031644	regulation of nervous system process	44/666	150/18614	3.07932066707266e-28	3.22018320822599e-26	1.84621308527514e-26	ADRB2/AGT/CACNG4/CACNG5/CARTPT/CHRNA7/CHRNB4/DLGAP2/EDNRB/FGF12/GPR171/GRIN2A/GRIN2B/GRIN2D/HCRT/HOMER1/HTR2C/IL1A/LRRK2/MAG/MGLL/NLGN1/NLGN3/NLGN4X/NMUR2/NPTX1/NPTX2/NRXN1/NRXN2/NTSR1/OPRD1/OPRM1/OXT/PTK2B/RELN/RIMS1/SHISA7/SOX10/SPX/STX1A/TMEM108/TNR/VIP/WNT7A	44	2.93333333333333
GO:0006939	BP	GO:0006939	smooth muscle contraction	39/666	113/18614	3.62207967265637e-28	3.70885866481376e-26	2.12638317625024e-26	ADRA1A/ADRA2A/ADRA2C/ADRB2/AGT/ATP1A2/BDKRB2/CALCA/CAV1/CHRM2/CHRNA3/CHRNB2/CHRNB4/DRD2/EDN2/EDN3/EDNRA/EDNRB/FKBP1B/GDNF/GPER1/GUCY1A1/HTR1D/HTR2B/HTR7/NMU/NMUR2/OXT/OXTR/P2RX1/P2RX2/PRKG1/PTGER3/RGS2/SPX/SSTR2/SULF1/TACR1/TACR2	39	3.45132743362832

	ONTOLOGY	ID	Description	GeneRatio	BgRatio	pvalue	p.adjust	qvalue	geneID	Count	richFactor
GO:0019226	BP	GO:0019226	transmission of nerve impulse	31/666	65/18614	7.93383346665538e-28	7.95812071196147e-26	4.56259338136122e-26	AGT/ANK3/CACNG4/CACNG5/CACNG7/CACNG8/CARTPT/CHRM5/CHRNA1/CHRNB4/CLCN1/CLDN19/CNTNAP1/DRD5/FGF12/FKBP1B/GPER1/GPR88/GRIK2/HCN1/HCRT/KCNA1/KCNA2/KCNMB2/MAG/NRCAM/P2RX1/P2RX4/S1PR1/SCN1B/TNR	31	4.76923076923077
GO:0016079	BP	GO:0016079	synaptic vesicle exocytosis	36/666	97/18614	2.21197028555065e-27	2.15742699038638e-25	1.23690786598797e-25	CADPS/CPLX1/GRIK5/LRRK2/NLGN1/NPY/P2RX1/P2RX7/P2RY1/P2RY4/PRKCB/PRKCG/RAP1A/RIMS1/RIMS3/RIMS4/RPH3A/SEPTIN5/SLC4A8/SNAP25/SNCA/STX1A/STXBP1/SV2B/SYP/SYT11/SYT12/SYT2/SYT4/SYT5/SYT7/SYT8/SYT9/UNC13C/VAMP2/WNT7A	36	3.71134020618557
GO:0048167	BP	GO:0048167	regulation of synaptic plasticity	50/666	209/18614	2.23863227893602e-27	2.15742699038638e-25	1.23690786598797e-25	AGT/CALB1/CALB2/CAMK2A/CAMK2B/CHRDL1/CHRNA7/CNTN2/CNTN4/CX3CR1/DRD2/DRD5/EPHA4/EPHB2/GRIA1/GRID2/GRIK2/GRIN2A/GRIN2B/GRIN2D/GRM2/MAP1B/MAPT/MEF2C/NSG1/NSMF/PRKCG/PRNP/PTK2B/PTN/RELN/RIMS1/RIMS3/RIMS4/SCTR/SHISA7/SLC18A3/SLC1A1/SNAP25/SNCA/SORCS3/STXBP1/SYP/SYT12/SYT4/SYT7/TNR/UNC13C/VAMP2/VGF	50	2.39234449760766
GO:0051960	BP	GO:0051960	regulation of nervous system development	73/666	461/18614	2.68000174092374e-27	2.53311703012311e-25	1.45230053855321e-25	ASCL1/ASPM/ATOH1/BDNF/CAMK2B/CDH4/CX3CR1/CXCL12/DKK1/DRD2/EFNA5/EPHA4/EPHB2/EPHB3/F2/FEZF1/FEZF2/FGF13/FLRT2/FOXG1/FSTL4/FZD3/GPER1/GRID2/IL6ST/KIF14/L1CAM/LIMK1/MAG/MAP1B/MAP2/MAPT/NEFL/NGF/NKX6-1/NLGN1/NLGN3/NRXN1/NTN1/NTRK3/OPRM1/OXT/OXTR/PLXNA1/PLXNA2/PLXNA3/POU4F1/PROX1/PTN/RELN/ROBO2/RTN4R/SEMA3D/SEMA3E/SEMA3F/SEMA3G/SEMA6A/SEMA6D/SHH/SLIT2/SLITRK3/SOX10/SYT4/TNR/TP73/VAX1/VEGFA/WDR62/WNT2/WNT3/WNT3A/WNT5A/WNT7A	73	1.58351409978308

	ONTOLOGY	ID	Description	GeneRatio	BgRatio	pvalue	p.adjust	qvalue	geneID	Count	richFactor
GO:0003012	BP	GO:0003012	muscle system process	72/666	452/18614	4.28119479449456e-27	3.97020234244165e-25	2.2762181658117e-25	ADRA1A/ADRA1B/ADRA2A/ADRA2C/ADRB2/AGT/ATP1A2/ATP2A1/BDKRB2/CACNA1C/CACNA1D/CALCA/CAMK2B/CAMK2D/CAV1/CHRM2/CHRNA1/CHRNA3/CHRNB2/CHRNB4/CHRND/CHRNG/CLCN1/DRD2/EDN2/EDN3/EDNRA/EDNRB/FGF12/FGF13/FKBP1B/GDNF/GNAO1/GPER1/GUCY1A1/HOMER1/HTR1D/HTR2B/HTR7/IGF1/IL6ST/KCNA1/KCNJ5/KCNQ1/LEP/LTB4R/MAP2K6/NEDD4L/NMU/NMUR2/NOS1/OXT/OXTR/P2RX1/P2RX2/P2RX4/P2RY1/PIK3CG/PRKG1/PTGER3/RGS2/RYR1/SCN1B/SCN2B/SPX/SSTR2/SULF1/TACR1/TACR2/TBX20/TMOD2/UCN	72	1.5929203539823
GO:1902075	BP	GO:1902075	cellular response to salt	49/666	204/18614	6.19300979417382e-27	5.63678576636376e-25	3.23171290818778e-25	CAMK2D/CHRM2/CHRM4/CHRM5/CHRNA3/CHRNA7/CHRNB2/CHRND/CLDN1/CPNE1/CPNE5/CPNE9/DLG2/FOSB/GNA11/GNAQ/HPCA/ITPR1/LEP/LY6G6D/LY6H/MEF2C/NLGN1/NPTX1/OPRM1/P2RX4/P2RX7/P2RY1/P2RY2/P2RY4/PLCG2/PRKCB/RASAL1/RYR1/RYR3/SHH/SLC1A1/SLC1A2/SYT10/SYT11/SYT12/SYT2/SYT4/SYT5/SYT7/SYT8/SYT9/TUBA1A/WNT5A	49	2.40196078431373
GO:0006816	BP	GO:0006816	calcium ion transport	72/666	455/18614	6.51567444050067e-27	5.82264361364742e-25	3.33826994779336e-25	ADCYAP1R1/ADRA1A/ADRA2A/AGT/ASIC1/ATP1A2/ATP2A1/ATP2A3/BCL2/BDKRB1/CACNA1A/CACNA1C/CACNA1D/CACNG4/CACNG5/CACNG7/CACNG8/CAMK2A/CAMK2B/CAMK2D/CAV1/CCL5/CCR7/CHRNA4/CHRNA7/CHRNB2/CXCL12/CYSLTR1/DRD2/DRD4/EDNRA/EDNRB/F2/FKBP1B/GCG/GPER1/GPM6A/GRIN2A/GRIN2B/GRIN2D/GRIN3A/GRIN3B/HCRT/HOMER1/HPCA/HTR2B/HTR2C/ITGB3/ITPR1/NMUR2/NOS1/NPSR1/NTSR1/OPRD1/OPRM1/P2RX1/P2RX2/P2RX4/P2RX7/PIK3CG/PLCB4/PLCG1/PLCG2/PRKCB/PRNP/PTK2B/RYR1/RYR3/SNCA/TRPC1/UCN/WNT3A	72	1.58241758241758

	ONTOLOGY	ID	Description	GeneRatio	BgRatio	pvalue	p.adjust	qvalue	geneID	Count	richFactor
GO:0048638	BP	GO:0048638	regulation of developmental growth	61/666	328/18614	9.01405815448386e-27	7.91144568380146e-25	4.53583339841227e-25	ADRB1/ADRB2/ADRB3/BCL2/BDNF/CACNG7/CDH4/CDK1/CGA/CPNE5/CPNE9/CSF1/CXCL12/DRD2/EFNA5/ERBB4/FGF13/FSTL4/GDF15/GHR/GHRHR/IGF1/L1CAM/LEP/LIMK1/MAG/MAP1B/MAP2/MAPT/MEF2C/NEDD4L/NGF/NKX6-1/NPY1R/NRCAM/NTN1/OLFM1/PLXNA3/PRKN/PROX1/RASAL1/RGS2/RIMS1/RTN4R/SEMA3D/SEMA3E/SEMA3F/SEMA3G/SEMA6A/SEMA6D/SLC6A3/SYT2/SYT4/TBX20/TNR/TP73/VEGFA/WNT2/WNT3/WNT3A/WNT5A	61	1.85975609756098
GO:0007187	BP	GO:0007187	G protein-coupled receptor signaling pathway, coupled to cyclic nucleotide second messenger	28/666	56/18614	5.42379731727836e-26	4.67683575691634e-24	2.6813491077293e-24	ADRB3/AGT/CHRM2/CHRM4/CHRM5/CNR1/CNR2/DRD4/GALR3/HTR1D/HTR2B/HTR2C/HTR4/HTR7/MC1R/MC3R/MTNR1A/NPY/NPY1R/OPRD1/OPRM1/PTH1R/SSTR1/SSTR2/SSTR3/SSTR5/TSHR/VIPR1	28	5
GO:0015850	BP	GO:0015850	organic hydroxy compound transport	57/666	296/18614	7.11025799904562e-26	6.02533070091539e-24	3.45447561950002e-24	ADORA3/ADRA2A/ADRA2C/AGT/AGTR1/APOC1/CARTPT/CAV1/CHRM5/CHRNA4/CHRNA6/CHRNB2/CLU/CNR1/CXCL12/DRD2/DRD4/GAL/GALR1/GDNF/GPM6B/GRM2/ITGB3/KCNA2/KCNQ1/LEP/MAOB/NMB/NOS1/OXT/OXTR/P2RX1/P2RY1/PINK1/PRKN/RAB3B/SDHD/SHH/SLC18A2/SLC18A3/SLC22A2/SLC22A3/SLC6A2/SLC6A3/SNCA/SNCG/STX1A/SYT10/SYT11/SYT12/SYT2/SYT4/SYT5/SYT7/SYT8/SYT9/VIP	57	1.92567567567568
GO:0051966	BP	GO:0051966	regulation of synaptic transmission, glutamatergic	32/666	80/18614	1.09187755909021e-25	9.09589525920069e-24	5.21490852040054e-24	ATP1A2/CACNG4/CACNG5/CACNG7/CACNG8/CCR2/CNR1/DKK1/DRD2/FRRS1L/GRIK1/GRIK2/GRIK3/GRIN2A/GRIN2B/GRIN2D/GRM2/GRM4/GRM8/HCN1/HOMER1/LRRK2/MEF2C/NLGN1/NLGN3/NRXN1/OXTR/PTK2B/RELN/STXBP1/TNR/UCN	32	4
GO:0051047	BP	GO:0051047	positive regulation of secretion	58/666	317/18614	4.07860646855622e-25	3.3410584654923e-23	1.91551395023245e-23	ADCYAP1/AGT/AVPR1B/CADPS/CARTPT/CDK5R2/CHRNB2/CXCL12/DRD2/EDN3/EDNRB/F2/F2RL1/F2RL2/GAL/GALR1/GCG/GDNF/GHRHR/GNA11/GPER1/GRP/IGF1/IL1A/INHBA/ITPR1/KISS1/LEP/MAP2K6/MIF/NKX6-1/NLGN1/NMB/NTSR1/OXT/OXTR/P2RX4/P2RX7/P2RY1/P2RY2/PINK1/PRKCB/PRL/SLC18A3/SLC4A8/SLC6A1/SNCA/STX1A/STXBP1/SYT10/SYT4/SYT7/SYT9/TACR1/TACR2/UCN/UCN3/VIP	58	1.82965299684543

	ONTOLOGY	ID	Description	GeneRatio	BgRatio	pvalue	p.adjust	qvalue	geneID	Count	richFactor
GO:0030534	BP	GO:0030534	adult behavior	40/666	143/18614	6.68586832866499e-25	5.38705620252269e-23	3.08853658599416e-23	ASIP/ATP1A2/CARTPT/CHL1/CHRNA3/CHRNA4/CHRNB2/CHRNB4/CNTN2/CXCL12/DRD2/DRD4/EPHA4/FGF12/GABRG2/GDNF/GLRB/GRIN2D/GRM2/HOMER1/LEP/NLGN3/NLGN4X/NPY/NRXN1/NRXN2/NTF4/NTSR1/OPRD1/OPRM1/PRKN/SEPTIN5/SLC1A1/SLC1A2/SLC7A11/SNCA/SNCG/SPTBN2/TUBA1A/UCHL1	40	2.7972027972028
GO:0070997	BP	GO:0070997	neuron death	62/666	373/18614	1.91393437093298e-24	1.51725603760252e-22	8.69881546856978e-23	ASCL1/AXL/BCL2/BCL2L1/BDNF/BID/CAPN2/CASP7/CCL5/CCND1/CHL1/CHRNA7/CLU/CNTFR/CRLF1/CSF1/CX3CR1/DDIT4/DKK1/FAS/FZD9/GDNF/GHR/GRID2/GRIK2/GRIK5/GRIN2B/GRM4/IL6ST/KIF14/LRRK2/MAG/MAPT/MEF2C/NDNF/NEFL/NGF/NGFR/NPY/NR3C1/NSMF/NTF4/PINK1/POU4F1/PRKCG/PRKN/PRNP/PTK2B/SEMA3E/SIGMAR1/SLC1A1/SLC7A11/SNCA/SNCG/STXBP1/THRB/TUBA1A/TYRO3/UCN/WNT1/WNT3A/WNT5A	62	1.66219839142091
GO:1903522	BP	GO:1903522	regulation of blood circulation	52/666	261/18614	2.06850399695483e-24	1.61376145159254e-22	9.25210559707281e-23	ADORA3/ADRA1A/ADRA1B/ADRA1D/ADRA2A/ADRA2C/ADRB1/AGT/AGTR1/APELA/APLN/ATP1A2/ATP2A1/ATP2A3/AVPR1B/BDKRB2/CACNA1C/CACNA1D/CALCA/CAMK2D/CAV1/CHRM2/DRD2/EDN2/EDN3/EDNRA/EDNRB/EPAS1/FGF13/FKBP1B/HCN1/KCNJ5/KCNMB2/KCNQ1/LEP/MC3R/NOS1/OXT/OXTR/P2RX1/P2RX4/PIK3CG/RGS2/SCN1B/SCN2B/SLC1A1/SPX/TACR1/TH/THRB/TRPC1/UCN	52	1.99233716475096
GO:0014046	BP	GO:0014046	dopamine secretion	23/666	38/18614	3.48808013830743e-24	2.67873654371579e-22	1.53578791615937e-22	CHRNA4/CHRNA6/CHRNB2/CNR1/CXCL12/DRD2/GDNF/GRM2/KCNA2/PINK1/PRKN/SLC18A2/SNCA/SNCG/SYT10/SYT11/SYT12/SYT2/SYT4/SYT5/SYT7/SYT8/SYT9	23	6.05263157894737
GO:0010975	BP	GO:0010975	regulation of neuron projection development	68/666	452/18614	3.77538743461406e-24	2.85477372940432e-22	1.63671452023673e-22	AGT/ARHGAP44/BDNF/CAMK2B/CDH4/CHRNA3/CHRNB2/CNR1/CNTN2/CXCL12/DKK1/EFNA5/EPHA3/EPHA4/EPHB2/EPHB3/FEZF2/FGF13/FKBP1B/FSTL4/GRID2/KIF1A/L1CAM/LIMK1/LPAR1/LRRC7/LRRK2/MAG/MAGI2/MAP1B/MAP2/MAPT/NDNF/NEDD4L/NEFL/NGF/NLGN1/NRCAM/NSMF/NTN1/NTNG1/NTRK3/PLXNA1/PLXNA2/PLXNA3/PTK2B/PTN/RAP1A/RELN/RGS2/ROBO2/RTN4R/SCN1B/SEMA3D/SEMA3E/SEMA3F/SEMA3G/SEMA6A/SEMA6D/SLIT2/SNAP25/TNR/TUBB2B/VEGFA/WNT3/WNT3A/WNT5A/WNT7A	68	1.50442477876106

	ONTOLOGY	ID	Description	GeneRatio	BgRatio	pvalue	p.adjust	qvalue	geneID	Count	richFactor
GO:0099173	BP	GO:0099173	postsynapse organization	44/666	185/18614	4.16255843627704e-24	3.09984465368207e-22	1.77721992566406e-22	ARHGAP44/CAMK2B/CHRDL1/CHRNA7/CNTNAP1/EPHA4/EPHB2/EPHB3/FRRS1L/FZD9/GAP43/GDNF/GLRB/GRID2/GRIN2B/HOMER1/ITGB3/KIF1A/LHFPL4/LRRK2/NEFH/NEFL/NLGN1/NLGN3/NLGN4X/NLGN4Y/NPTX1/NRCAM/NRP2/NRXN1/NRXN2/NTRK3/PRNP/PTK2B/RELN/RPS6KA5/SEMA3F/SHISA7/SLC7A11/SLITRK3/SPTBN2/TMEM108/WNT5A/WNT7A	44	2.37837837837838
GO:0050767	BP	GO:0050767	regulation of neurogenesis	62/666	381/18614	6.17418387585697e-24	4.5292707089309e-22	2.5967463056825e-22	ASCL1/ASPM/ATOH1/BDNF/CAMK2B/CDH4/CX3CR1/CXCL12/DRD2/EFNA5/EPHA4/EPHB2/F2/FEZF1/FEZF2/FGF13/FOXG1/FSTL4/FZD3/GPER1/IL6ST/L1CAM/LIMK1/MAG/MAP1B/MAP2/MAPT/NEFL/NGF/NKX6-1/NTN1/NTRK3/OPRM1/PLXNA1/PLXNA2/PLXNA3/POU4F1/PROX1/PTN/RELN/ROBO2/RTN4R/SEMA3D/SEMA3E/SEMA3F/SEMA3G/SEMA6A/SEMA6D/SHH/SLIT2/SOX10/SYT4/TNR/TP73/VAX1/VEGFA/WDR62/WNT2/WNT3/WNT3A/WNT5A/WNT7A	62	1.62729658792651
GO:0007416	BP	GO:0007416	synapse assembly	45/666	197/18614	7.5730718666546e-24	5.47377179773638e-22	3.13825284629015e-22	ADGRL3/BDNF/CHRNB2/DKK1/DNER/DRD2/EFNA5/EPHB2/EPHB3/ERBB4/FGF13/FLRT2/GABRB3/GABRG2/GAP43/GPM6A/GRID2/ICAM5/KIRREL3/LHFPL4/MAP1B/MDGA1/MEF2C/NLGN1/NLGN3/NLGN4X/NLGN4Y/NPTX1/NRCAM/NRG3/NRXN1/NRXN2/NTN1/NTRK3/OXT/OXTR/POU4F1/PTK2B/ROBO2/SLITRK3/SNCA/SPTBN2/WNT3A/WNT5A/WNT7A	45	2.28426395939086
GO:0048675	BP	GO:0048675	axon extension	36/666	119/18614	7.87201979034473e-24	5.60738801007889e-22	3.21485842543903e-22	CDH4/CXCL12/DCLK1/EDN2/EDN3/EDNRA/HSP90AB1/L1CAM/LIMK1/MAG/MAP1B/MAP2/MAPT/NDN/NKX6-1/NLGN3/NRCAM/NRP2/NTN1/OLFM1/PLXNA3/RTN4R/SEMA3D/SEMA3E/SEMA3F/SEMA3G/SEMA6A/SEMA6D/SLIT2/SLIT3/TNR/VCL/VEGFA/WNT3/WNT3A/WNT5A	36	3.02521008403361
GO:0045055	BP	GO:0045055	regulated exocytosis	48/666	228/18614	1.04250863585824e-23	7.31989992177605e-22	4.1966851401391e-22	ADRA2A/CADPS/CCR2/CDK5R2/CPLX1/F2RL1/GRIK5/GRP/LRRK2/NLGN1/NPY/P2RX1/P2RX7/P2RY1/P2RY4/PIK3CD/PIK3CG/PRKCB/PRKCG/PTGDR/RAP1A/RIMS1/RIMS3/RIMS4/RPH3A/SEPTIN5/SLC18A2/SLC4A8/SNAP25/SNCA/STX1A/STXBP1/SV2B/SYN2/SYP/SYT10/SYT11/SYT12/SYT2/SYT4/SYT5/SYT7/SYT8/SYT9/TPH1/UNC13C/VAMP2/WNT7A	48	2.10526315789474

	ONTOLOGY	ID	Description	GeneRatio	BgRatio	pvalue	p.adjust	qvalue	geneID	Count	richFactor
GO:0021537	BP	GO:0021537	telencephalon development	52/666	270/18614	1.0862107021598e-23	7.51933183255694e-22	4.31102453622208e-22	ADGRG1/ASCL1/ASPM/ATOH1/ATP1A2/CDK5R2/CNTN2/COL3A1/CXCL12/DNAH5/DRD2/EMX1/EPHB2/EPHB3/ERBB4/FEZF1/FEZF2/FGF13/FOXG1/INHBA/KCNA1/KCNA2/KIF14/KIRREL3/LHX1/LPAR1/LRRK2/MDGA1/NEFL/NEUROD1/NKX2-1/NPY/NRG3/OXTR/PLXNA3/PROX1/RELN/ROBO2/RTN4R/SHH/SLC1A2/SLC7A11/SLIT2/TH/TMEM108/TNR/TUBA1A/TUBB2A/TUBB2B/WDR62/WNT3A/WNT5A	52	1.92592592592593
GO:0050803	BP	GO:0050803	regulation of synapse structure or activity	49/666	240/18614	1.48225880617586e-23	1.01184750449366e-21	5.80117956744558e-22	ARHGAP44/BDNF/CAMK2B/CHRNB2/CNTNAP1/DKK1/DRD2/EFNA5/EPHA4/EPHB2/EPHB3/FLRT2/GPM6A/GRID2/GRIN2B/HOMER1/ICAM5/IGSF9/KIF1A/LHFPL4/LRRK2/MDGA1/MEF2C/NEFL/NLGN1/NLGN3/NRCAM/NRP2/NRXN1/NTN1/NTRK3/OXT/OXTR/PRNP/PTK2B/RELN/ROBO2/RPS6KA5/SEMA3F/SLC17A7/SLC17A8/SLC18A3/SLC7A11/SLITRK3/SNCA/TUBA1A/WNT3A/WNT5A/WNT7A	49	2.04166666666667
GO:0007626	BP	GO:0007626	locomotory behavior	45/666	201/18614	1.84183411460671e-23	1.24008420182082e-21	7.10971870915956e-22	ADCY5/ASTN1/ATP1A2/CALB1/CHL1/CHRNA3/CHRNB2/CHRNB4/CNTN2/CXCL12/DRD2/DRD4/EPHA4/FEZF2/FGF12/GAD1/GDNF/GLRB/GNG7/GPR88/GRIN2D/HTR2C/LRRK2/MC3R/NKX2-1/NPY1R/NPY2R/NTF4/NTSR1/OPRD1/PAK6/PENK/PRKN/RELN/SLC18A2/SLC1A1/SLC6A3/SLURP1/SNAP25/SNCA/SNCG/TH/TNR/TUBA1A/UCHL1	45	2.23880597014925
GO:0007189	BP	GO:0007189	adenylate cyclase-activating G protein-coupled receptor signaling pathway	38/666	140/18614	3.38038392083582e-23	2.24521445552811e-21	1.28723865662553e-21	ADCY2/ADCY3/ADCY5/ADCY9/ADCYAP1/ADGRG1/ADGRL3/ADRA1A/ADRA1B/ADRA1D/ADRA2A/ADRA2C/ADRB1/ADRB2/ADRB3/CALCA/CALCB/DRD2/DRD5/GALR1/GCG/GCGR/GHRHR/GNA13/GNAQ/GNG2/GPER1/MC1R/MC3R/OPRM1/PTGER3/PTGER4/PTGFR/PTH1R/TSHR/UCN2/UCN3/VIP	38	2.71428571428571
GO:1903532	BP	GO:1903532	positive regulation of secretion by cell	53/666	289/18614	4.42304117355685e-23	2.89856631573759e-21	1.66182192584024e-21	ADCYAP1/AGT/CADPS/CARTPT/CDK5R2/CHRNB2/CXCL12/DRD2/EDN3/F2/F2RL1/F2RL2/GAL/GALR1/GCG/GDNF/GHRHR/GNA11/GPER1/GRP/IGF1/IL1A/INHBA/ITPR1/KISS1/LEP/MAP2K6/MIF/NKX6-1/NLGN1/NMB/NTSR1/OXT/OXTR/P2RX4/P2RX7/P2RY1/PINK1/PRKCB/SLC18A3/SLC4A8/SNCA/STX1A/STXBP1/SYT10/SYT4/SYT7/SYT9/TACR1/TACR2/UCN/UCN3/VIP	53	1.83391003460208

	ONTOLOGY	ID	Description	GeneRatio	BgRatio	pvalue	p.adjust	qvalue	geneID	Count	richFactor
GO:0008217	BP	GO:0008217	regulation of blood pressure	43/666	187/18614	5.84613697547225e-23	3.67554144466278e-21	2.107281564997e-21	ADRA1A/ADRA1B/ADRB1/ADRB2/ADRB3/AGT/AGTR1/APLN/ATP1A2/AVPR1B/BDKRB1/CALCA/CARTPT/CNR1/CTSG/DRD2/DRD5/EDN2/EDN3/EDNRA/EDNRB/F2RL1/GCGR/GNA11/GNA13/GUCY1A1/KCNQ1/LEP/MC3R/NOS1/NPY/NPY1R/NTSR1/OXT/OXTR/P2RX1/P2RX2/P2RX4/PTGS1/SPX/TACR1/UCN/UTS2B	43	2.29946524064171
GO:0001508	BP	GO:0001508	action potential	38/666	142/18614	5.90778787646713e-23	3.67554144466278e-21	2.107281564997e-21	ADRA1A/ANK3/ATP1A2/CACNA1C/CACNA1D/CAMK2D/CAV1/CHRNA1/CHRNA4/CHRNB2/CHRNB4/CLCN1/CLDN19/CNR1/CNR2/CNTNAP1/FGF12/FGF13/FKBP1B/GNA11/GNAQ/GPER1/GPR88/GRIK2/HCN1/KCNA1/KCNA2/KCNJ5/KCNMB2/KCNQ1/NEDD4L/NRCAM/NTSR1/P2RX1/P2RX4/SCN1B/SCN2B/TACR1	38	2.67605633802817
GO:0035296	BP	GO:0035296	regulation of tube diameter	38/666	142/18614	5.90778787646713e-23	3.67554144466278e-21	2.107281564997e-21	ADRA1A/ADRA1D/ADRA2A/ADRA2C/ADRB1/ADRB2/ADRB3/AGT/AGTR1/ATP1A2/AVPR1B/BDKRB2/CALCA/CAV1/DRD5/EDN2/EDN3/EDNRA/EDNRB/F2RL1/GPER1/GUCY1A1/HTR1D/HTR2B/HTR7/KCNMB2/KNG1/LEP/NOS1/OXTR/P2RX1/P2RY1/P2RY2/PRKG1/RGS2/TACR1/UCN/UTS2B	38	2.67605633802817
GO:0097746	BP	GO:0097746	blood vessel diameter maintenance	38/666	142/18614	5.90778787646713e-23	3.67554144466278e-21	2.107281564997e-21	ADRA1A/ADRA1D/ADRA2A/ADRA2C/ADRB1/ADRB2/ADRB3/AGT/AGTR1/ATP1A2/AVPR1B/BDKRB2/CALCA/CAV1/DRD5/EDN2/EDN3/EDNRA/EDNRB/F2RL1/GPER1/GUCY1A1/HTR1D/HTR2B/HTR7/KCNMB2/KNG1/LEP/NOS1/OXTR/P2RX1/P2RY1/P2RY2/PRKG1/RGS2/TACR1/UCN/UTS2B	38	2.67605633802817
GO:0014059	BP	GO:0014059	regulation of dopamine secretion	22/666	37/18614	6.0963092345578e-23	3.74541998598145e-21	2.14734471327779e-21	CHRNA4/CHRNA6/CHRNB2/CNR1/CXCL12/DRD2/GDNF/GRM2/KCNA2/PINK1/PRKN/SNCA/SNCG/SYT10/SYT11/SYT12/SYT2/SYT4/SYT5/SYT7/SYT8/SYT9	22	5.94594594594595
GO:0006874	BP	GO:0006874	intracellular calcium ion homeostasis	54/666	303/18614	6.98492884555727e-23	4.23838583653259e-21	2.42997459643363e-21	ATP1A2/ATP2A1/ATP2A3/BCL2/BDKRB1/CACNA1C/CALB1/CALB2/CALCA/CALCB/CAMK2D/CAV1/CCL5/CCR2/CCR7/CHRNA7/CNR1/CXCL12/DRD2/DRD4/DRD5/EDN2/EDN3/EDNRA/F2/FKBP1B/FZD9/GPER1/GRIA1/GRIK2/HTR2B/HTR2C/ITGB3/ITPR1/NOS1/NPSR1/NPY/NTSR1/P2RX1/P2RX7/P2RY1/P2RY4/PLCB4/PLCG1/PLCG2/PRKCB/PTH1R/PTK2B/RYR1/RYR3/SNCA/SV2B/TRPC1/WNT5A	54	1.78217821782178

	ONTOLOGY	ID	Description	GeneRatio	BgRatio	pvalue	p.adjust	qvalue	geneID	Count	richFactor
GO:0035150	BP	GO:0035150	regulation of tube size	38/666	143/18614	7.78004396981992e-23	4.66328245264206e-21	2.67357865304338e-21	ADRA1A/ADRA1D/ADRA2A/ADRA2C/ADRB1/ADRB2/ADRB3/AGT/AGTR1/ATP1A2/AVPR1B/BDKRB2/CALCA/CAV1/DRD5/EDN2/EDN3/EDNRA/EDNRB/F2RL1/GPER1/GUCY1A1/HTR1D/HTR2B/HTR7/KCNMB2/KNG1/LEP/NOS1/OXTR/P2RX1/P2RY1/P2RY2/PRKG1/RGS2/TACR1/UCN/UTS2B	38	2.65734265734266
GO:1901214	BP	GO:1901214	regulation of neuron death	56/666	327/18614	8.1558346937553e-23	4.82962982166353e-21	2.76894983832377e-21	ASCL1/AXL/BCL2/BCL2L1/BDNF/CAPN2/CCL5/CCND1/CHL1/CHRNA7/CLU/CNTFR/CRLF1/CSF1/CX3CR1/DDIT4/DKK1/FZD9/GDNF/GHR/GRID2/GRIK2/GRIK5/GRIN2B/GRM4/IL6ST/KIF14/LRRK2/MAG/MAPT/MEF2C/NDNF/NEFL/NGF/NPY/NR3C1/NSMF/NTF4/PINK1/POU4F1/PRKCG/PRKN/PRNP/PTK2B/SEMA3E/SIGMAR1/SLC1A1/SLC7A11/SNCA/SNCG/STXBP1/TYRO3/UCN/WNT1/WNT3A/WNT5A	56	1.71253822629969
GO:0050807	BP	GO:0050807	regulation of synapse organization	47/666	234/18614	2.54491486060088e-22	1.48907815950635e-20	8.53726451356963e-21	ARHGAP44/BDNF/CAMK2B/CHRNB2/CNTNAP1/DKK1/DRD2/EFNA5/EPHA4/EPHB2/EPHB3/FLRT2/GPM6A/GRID2/GRIN2B/HOMER1/ICAM5/IGSF9/KIF1A/LHFPL4/LRRK2/MDGA1/MEF2C/NEFL/NLGN1/NLGN3/NRCAM/NRP2/NRXN1/NTN1/NTRK3/OXT/OXTR/PRNP/PTK2B/RELN/ROBO2/RPS6KA5/SEMA3F/SLC18A3/SLC7A11/SLITRK3/SNCA/TUBA1A/WNT3A/WNT5A/WNT7A	47	2.00854700854701
GO:0019233	BP	GO:0019233	sensory perception of pain	32/666	103/18614	1.0162620912068e-21	5.87638609209581e-20	3.36908188007506e-20	BDKRB1/CALCA/CCR2/CHRNA4/CHRNB2/CNR1/CNR2/CXCL12/EDNRB/GPR171/GRIN2A/GRIN2D/IL1A/KCNA1/KCNA2/MC1R/MGLL/NDN/NMUR2/NPY1R/NTSR1/OPRD1/OPRM1/OXT/P2RX4/P2RX7/P2RY1/PENK/SPX/TACR1/UCHL1/VIP	32	3.10679611650485
GO:0070588	BP	GO:0070588	calcium ion transmembrane transport	57/666	360/18614	1.76923883548941e-21	1.0111405670268e-19	5.79712651481658e-20	ADCYAP1R1/ADRA1A/ADRA2A/ASIC1/ATP1A2/ATP2A1/ATP2A3/BCL2/BDKRB1/CACNA1A/CACNA1C/CACNA1D/CACNG4/CACNG5/CACNG7/CACNG8/CAMK2D/CAV1/CCR7/CHRNA7/DRD2/DRD4/EDNRA/EDNRB/F2/FKBP1B/GPER1/GPM6A/GRIN2A/GRIN2B/GRIN2D/GRIN3A/GRIN3B/HPCA/HTR2B/HTR2C/ITGB3/ITPR1/NOS1/NPSR1/NTSR1/OPRM1/P2RX1/P2RX2/P2RX4/P2RX7/PIK3CG/PLCB4/PLCG1/PLCG2/PRNP/PTK2B/RYR1/RYR3/SNCA/TRPC1/WNT3A	57	1.58333333333333

	ONTOLOGY	ID	Description	GeneRatio	BgRatio	pvalue	p.adjust	qvalue	geneID	Count	richFactor
GO:0007215	BP	GO:0007215	glutamate receptor signaling pathway	24/666	52/18614	2.14476031493522e-21	1.21166631585134e-19	6.94679172786642e-20	FRRS1L/GNAQ/GRIA1/GRIA2/GRIA3/GRIA4/GRID2/GRIK1/GRIK2/GRIK3/GRIK5/GRIN2A/GRIN2B/GRIN2D/GRIN3A/GRIN3B/GRM2/GRM4/GRM8/HOMER1/PRNP/PTK2B/SLC1A1/SSTR1	24	4.61538461538462
GO:0051962	BP	GO:0051962	positive regulation of nervous system development	50/666	280/18614	2.65639716885119e-21	1.48365819146632e-19	8.50619045575913e-20	ASCL1/ASPM/BDNF/CAMK2B/CDH4/CX3CR1/CXCL12/DRD2/EFNA5/EPHA4/EPHB2/EPHB3/FLRT2/FOXG1/FZD3/GPER1/GRID2/IL6ST/L1CAM/LIMK1/MAG/MAP1B/MAPT/NEFL/NGF/NKX6-1/NLGN1/NLGN3/NRXN1/NTN1/OPRM1/OXT/OXTR/PLXNA1/PLXNA2/PLXNA3/PTN/RELN/ROBO2/SHH/SLIT2/SLITRK3/SOX10/TP73/VEGFA/WDR62/WNT2/WNT3/WNT3A/WNT7A	50	1.78571428571429
GO:0055074	BP	GO:0055074	calcium ion homeostasis	54/666	327/18614	2.85302662094931e-21	1.57557593729954e-19	9.03317831375672e-20	ATP1A2/ATP2A1/ATP2A3/BCL2/BDKRB1/CACNA1C/CALB1/CALB2/CALCA/CALCB/CAMK2D/CAV1/CCL5/CCR2/CCR7/CHRNA7/CNR1/CXCL12/DRD2/DRD4/DRD5/EDN2/EDN3/EDNRA/F2/FKBP1B/FZD9/GPER1/GRIA1/GRIK2/HTR2B/HTR2C/ITGB3/ITPR1/NOS1/NPSR1/NPY/NTSR1/P2RX1/P2RX7/P2RY1/P2RY4/PLCB4/PLCG1/PLCG2/PRKCB/PTH1R/PTK2B/RYR1/RYR3/SNCA/SV2B/TRPC1/WNT5A	54	1.65137614678899
GO:0051954	BP	GO:0051954	positive regulation of amine transport	22/666	43/18614	5.59574112131767e-21	3.05589640125293e-19	1.75202327272133e-19	AGT/CARTPT/CHRNB2/CXCL12/DRD2/DRD4/GDNF/NTSR1/OXT/OXTR/P2RX7/PINK1/RAB3B/SLC17A8/SLC18A3/SLC38A3/SLC6A1/STX1A/STXBP1/SYT4/TACR2/VIP	22	5.11627906976744
GO:0007612	BP	GO:0007612	learning	37/666	152/18614	7.82483741552424e-21	4.22627207662655e-19	2.42302946921439e-19	ADCY3/AGT/ASIC1/ATP1A2/BCHE/CHRNB2/CNTN2/DKK1/DRD2/DRD5/EPHB2/FGF13/GPR88/GRIN2A/NLGN3/NLGN4X/NLGN4Y/NPTX2/NRXN1/NRXN2/NTSR1/PAK6/PRKN/PTN/RELN/SLC1A1/SLC6A1/SLC7A11/SNAP25/SORCS3/SYT11/TACR1/TACR2/TH/TNR/TUBA1A/UCN	37	2.43421052631579
GO:0007193	BP	GO:0007193	adenylate cyclase-inhibiting G protein-coupled receptor signaling pathway	28/666	80/18614	9.18790898204539e-21	4.90854050508186e-19	2.81419134381413e-19	ADCY2/ADCY5/ADRA2A/CHRM2/CHRM4/CHRM5/DRD2/DRD4/EDNRA/GNAI1/GNAZ/GRIK3/GRM2/GRM4/GRM8/HTR1D/ITGB3/LPAR1/MTNR1A/NPY1R/NPY2R/OPRD1/OPRM1/P2RY1/PTGDR2/RGS2/S1PR1/SSTR2	28	3.5

	ONTOLOGY	ID	Description	GeneRatio	BgRatio	pvalue	p.adjust	qvalue	geneID	Count	richFactor
GO:0034765	BP	GO:0034765	regulation of monoatomic ion transmembrane transport	65/666	478/18614	1.06322915575406e-20	5.61910892530235e-19	3.22157832479186e-19	ADCYAP1R1/ADRA2A/ADRB2/ANK3/ATP1A2/ATP2A1/BCL2/BDKRB1/CACNA1A/CACNA1C/CACNA1D/CACNG4/CAMK2D/CAV1/CCR2/CLCN1/DRD4/EDN3/EDNRA/EPHB2/F2/FGF12/FGF13/FKBP1B/GAL/GPER1/GRIN2A/GRIN2B/GRIN2D/GRP/HCN1/HOMER1/HPCA/ITGB3/KCNA1/KCNA2/KCNJ14/KCNJ5/KCNJ9/KCNQ1/KCNQ5/MMP9/NEDD4L/NLGN3/NOS1/NPSR1/NTSR1/P2RX1/P2RX2/P2RX4/P2RX7/PIK3CG/PINK1/PLCG1/PLCG2/PRNP/PTK2B/RELN/SCN1B/SCN2B/SLC6A9/SNCA/TRPC1/TWIST1/VAMP2	65	1.35983263598326
GO:0042886	BP	GO:0042886	amide transport	55/666	349/18614	1.162984985522e-20	6.08092681259642e-19	3.48635028694557e-19	ADCY5/ADCYAP1/ADRA2A/ATP1A2/CARTPT/CCL5/CNR1/CPLX1/DRD2/EDN3/EFNA5/F2/F2RL1/F2RL2/FKBP1B/GAL/GCG/GHRHR/GNA11/GNAZ/GPER1/GRM2/GRP/HTR2C/ITPR1/KISS1/LEP/NEUROD1/NKX6-1/NPY5R/NTSR1/P2RX7/PRKCB/PRKN/SLC17A7/SLC17A8/SLC1A1/SLC1A2/SLC1A7/SLC38A3/SLC38A5/SLC7A11/SNAP25/SNCA/SSTR5/STX1A/STXBP1/SYT4/SYT7/SYT9/TACR2/UCN/UCN3/VGF/VIP	55	1.57593123209169
GO:0050770	BP	GO:0050770	regulation of axonogenesis	37/666	155/18614	1.61317366664891e-20	8.34605112797829e-19	4.78500377353919e-19	BDNF/CDH4/CNTN2/CXCL12/EFNA5/EPHA4/EPHB2/EPHB3/FGF13/FSTL4/L1CAM/LIMK1/MAG/MAP1B/MAP2/MAPT/NEFL/NGF/NTN1/PLXNA1/PLXNA2/PLXNA3/ROBO2/RTN4R/SEMA3D/SEMA3E/SEMA3F/SEMA3G/SEMA6A/SEMA6D/SLIT2/TNR/VEGFA/WNT3/WNT3A/WNT5A/WNT7A	37	2.38709677419355
GO:0033674	BP	GO:0033674	positive regulation of kinase activity	61/666	430/18614	2.03918600173012e-20	1.03721265685296e-18	5.94660444909862e-19	ADCYAP1/ADRA1A/ADRA2A/ADRA2C/ADRB2/AGT/AXIN2/AXL/CALCA/CARTPT/CCL5/CCND1/CCR7/CDK5R2/CHRNA3/CLU/CSF1/DKK1/DRD2/DRD4/EDN3/EPHA4/ERBB4/F2/FGFR1/FZD10/GCG/GHR/HMGA2/HSP90AB1/HTR2B/IGF1/IL6R/IRAK1/ITGB3/KIF14/LEP/LRRK2/MAP2K6/MAPT/MEF2C/MIF/NOX4/NTRK3/P2RX7/PIK3CG/PIK3R5/PINK1/PRNP/PROX1/PTK2B/RAP1A/RELN/SLC1A1/SNCA/TENM1/TYRO3/UCN/VEGFA/WNT3A/WNT5A	61	1.41860465116279
GO:0001504	BP	GO:0001504	neurotransmitter uptake	22/666	45/18614	2.04699140823473e-20	1.03721265685296e-18	5.94660444909862e-19	ATP1A2/DRD2/DRD4/GDNF/GPM6B/ITGB3/NOS1/PRKN/RAB3B/SLC17A8/SLC18A2/SLC18A3/SLC1A2/SLC1A7/SLC22A2/SLC22A3/SLC29A1/SLC6A1/SLC6A2/SLC6A3/SNAP25/SNCA	22	4.88888888888889

	ONTOLOGY	ID	Description	GeneRatio	BgRatio	pvalue	p.adjust	qvalue	geneID	Count	richFactor
GO:0045860	BP	GO:0045860	positive regulation of protein kinase activity	56/666	366/18614	2.15555422072384e-20	1.0810764280467e-18	6.19808662607703e-19	ADCYAP1/ADRA1A/ADRA2A/ADRA2C/ADRB2/AGT/AXIN2/CALCA/CARTPT/CCL5/CCND1/CCR7/CDK5R2/CHRNA3/CLU/CSF1/DKK1/DRD2/DRD4/EDN3/EPHA4/FGFR1/FZD10/GCG/GHR/HMGA2/HSP90AB1/HTR2B/IGF1/IL6R/IRAK1/ITGB3/KIF14/LEP/LRRK2/MAP2K6/MEF2C/MIF/NOX4/NTRK3/P2RX7/PIK3CG/PIK3R5/PINK1/PRNP/PROX1/PTK2B/RAP1A/RELN/SLC1A1/SNCA/TENM1/UCN/VEGFA/WNT3A/WNT5A	56	1.53005464480874
GO:0033555	BP	GO:0033555	multicellular organismal response to stress	29/666	90/18614	2.64982208760176e-20	1.31554298591542e-18	7.54234314567773e-19	ADCYAP1R1/ADRA2A/ADRB1/ASIC1/ATP1A2/BCL2/CALCA/CAPN2/DRD4/EDNRB/EPHB2/GNG7/GRIK2/GRP/GRPR/HCN1/HTR2C/MEF2C/NMUR2/NOS1/P2RX2/P2RX4/PENK/PRKCG/RELN/SLC1A1/SLC6A2/TACR1/UCN	29	3.22222222222222
GO:0001558	BP	GO:0001558	regulation of cell growth	60/666	420/18614	2.98740032522239e-20	1.4683072598468e-18	8.41817965328456e-19	AGT/AGTR1/BCL2/BDKRB1/BDNF/CACNG7/CAMK2D/CDH4/CPNE5/CPNE9/CXCL12/EFNA5/F2/FGF13/FSTL4/GNG4/IGF1/INHBA/KIF14/L1CAM/LGI1/LIMK1/MAG/MAP1B/MAP2/MAPT/NDUFA13/NEDD4L/NGF/NKX6-1/NRCAM/NRG3/NTN1/OLFM1/PLXNA3/PRKN/PRSS2/PTK2B/RASAL1/RGS2/RIMS1/RTN4R/SEMA3D/SEMA3E/SEMA3F/SEMA3G/SEMA6A/SEMA6D/SLC44A4/SLIT2/SLIT3/SYT2/SYT4/TNR/UCN/VEGFA/WNT11/WNT3/WNT3A/WNT5A	60	1.42857142857143
GO:0021953	BP	GO:0021953	central nervous system neuron differentiation	39/666	177/18614	3.17228370994345e-20	1.54374004300713e-18	8.85065501982138e-19	ASCL1/CHRNB2/CNTN2/DCLK1/DRD2/EMX1/EPHA4/EPHB2/EPHB3/FEZF2/FOXG1/GABRB1/GRID2/HSP90AB1/INHBA/LEP/LHX1/MAP2/MAPT/MDGA1/NDNF/NKX2-1/NKX6-1/NPY/NRP2/PHOX2B/PLXNA1/PLXNA3/POU4F1/PROX1/SCN1B/SEMA3E/SHH/SLIT2/SOX1/TBX20/WNT1/WNT3A/WNT7A	39	2.20338983050847
GO:0043270	BP	GO:0043270	positive regulation of monoatomic ion transport	44/666	231/18614	5.13392437635083e-20	2.47384689311415e-18	1.41831945877102e-18	ADCYAP1R1/ADRA2A/ADRB2/ANK3/ATP2A1/BDKRB1/CACNA1D/CACNG4/CAMK2A/CAV1/CCL5/CCR2/CXCL12/DRD4/EDN3/EDNRA/EPHB2/F2/FGF12/GAL/GCG/GPER1/HCRT/HOMER1/KCNA1/KCNQ1/LEP/NLGN3/NOS1/NPSR1/NTSR1/P2RX1/P2RX2/P2RX4/P2RX7/P2RY1/PINK1/PLCG1/PLCG2/RELN/SCN1B/SNCA/TRPC1/UCN	44	1.9047619047619

	ONTOLOGY	ID	Description	GeneRatio	BgRatio	pvalue	p.adjust	qvalue	geneID	Count	richFactor
GO:0022898	BP	GO:0022898	regulation of transmembrane transporter activity	49/666	289/18614	6.71073874817677e-20	3.20226028614455e-18	1.83593741736017e-18	ADRA2A/ADRB2/ANK3/ATP1A2/ATP2A1/BCL2/CACNA1D/CACNG4/CACNG5/CACNG7/CACNG8/CAMK2D/CAV1/CCR2/CNIH3/DRD4/EDNRA/EPHB2/FGF12/FGF13/FKBP1B/GAL/GRP/GSG1L/HCN1/HOMER1/HPCA/KCNA1/KCNQ1/MEF2C/MMP9/NEDD4L/NLGN1/NLGN3/NOS1/NTSR1/OPRM1/PINK1/PLCG2/PRNP/PTK2B/RELN/SCN1B/SCN2B/SHISA7/SLC6A9/SNCA/TWIST1/VAMP2	49	1.69550173010381
GO:0046879	BP	GO:0046879	hormone secretion	50/666	305/18614	1.24425746366707e-19	5.88031291723429e-18	3.37133322898457e-18	ADCY5/ADCYAP1/ADRA2A/AGT/AGTR1/CARTPT/CCKAR/CCL5/CGA/CNR1/CPLX1/DRD2/EDN3/EFNA5/F2/F2RL1/F2RL2/FKBP1B/GAL/GALR1/GCG/GHRHR/GNA11/GNAZ/GPER1/GRP/HTR2C/INHBA/ITPR1/KCNQ1/KISS1/LEP/NEUROD1/NKX6-1/NMB/P2RY1/PRKCB/PRKN/SLC18A2/SNAP25/SSTR5/STX1A/SYT7/SYT9/TACR1/TACR2/UCN/UCN3/VGF/VIP	50	1.63934426229508
GO:0010038	BP	GO:0010038	response to metal ion	55/666	367/18614	1.28150993938155e-19	5.99868700196221e-18	3.43920010799439e-18	ANK3/ASCL1/BCL2/CAMK2D/CAV1/CCND1/CDK1/CLDN1/CPNE1/CPNE5/CPNE9/DLG2/DRD2/FOSB/HOMER1/HPCA/IL1A/KCNA1/KCNMB2/LRRK2/MAOB/MAPT/MEF2C/MMP9/NLGN1/NPTX1/P2RX4/P2RX7/PENK/PLCG2/PRKN/PRNP/PTK2B/RASAL1/RYR1/RYR3/SHH/SLC1A1/SLC6A1/SLC6A3/SNCA/SYT10/SYT11/SYT12/SYT2/SYT4/SYT5/SYT7/SYT8/SYT9/TH/TRPC1/TUBA1A/TXNIP/WNT5A	55	1.49863760217984
GO:1903305	BP	GO:1903305	regulation of regulated secretory pathway	33/666	130/18614	2.53039953423205e-19	1.17329374629722e-17	6.72679200907567e-18	ADRA2A/CCR2/CDK5R2/F2RL1/LRRK2/NLGN1/NPY/P2RX1/P2RY1/P2RY4/PRKCB/PRKCG/RAP1A/RIMS1/RIMS3/RIMS4/RPH3A/SEPTIN5/SLC4A8/STX1A/STXBP1/SV2B/SYP/SYT10/SYT11/SYT12/SYT2/SYT4/SYT5/SYT7/SYT8/SYT9/WNT7A	33	2.53846153846154
GO:0061387	BP	GO:0061387	regulation of extent of cell growth	30/666	105/18614	2.81410987114638e-19	1.29264953426957e-17	7.41108915401756e-18	BDNF/CDH4/CXCL12/EFNA5/FGF13/FSTL4/L1CAM/LIMK1/MAG/MAP1B/MAP2/MAPT/NGF/NKX6-1/NRCAM/NTN1/OLFM1/PLXNA3/RTN4R/SEMA3D/SEMA3E/SEMA3F/SEMA3G/SEMA6A/SEMA6D/TNR/VEGFA/WNT3/WNT3A/WNT5A	30	2.85714285714286

	ONTOLOGY	ID	Description	GeneRatio	BgRatio	pvalue	p.adjust	qvalue	geneID	Count	richFactor
GO:0043410	BP	GO:0043410	positive regulation of MAPK cascade	64/666	495/18614	3.05636706802292e-19	1.39093001290117e-17	7.97455618040678e-18	ADCYAP1/ADRA1A/ADRA1B/ADRA2A/ADRA2C/ADRB2/ADRB3/APELA/ARRB1/AVPR1B/CARTPT/CCL5/CCR7/CHRNA7/DKK1/DRD2/DRD4/EDN3/EPHA4/ERBB4/F2RL1/FGFR1/FZD10/GCG/GDF15/GHR/GPER1/GRM4/HTR2B/HTR2C/IGF1/IL1A/INHBA/IRAK1/ITGB3/KISS1/LEP/LPAR1/LRRK2/MAP2K6/MEF2C/MIF/NOX4/NPSR1/NPY/NPY5R/NTRK3/OPRM1/P2RX7/P2RY1/PIK3CG/PIK3R5/PLCG2/PTK2B/RAP1A/RIPK2/SOX2/TENM1/TP73/VEGFA/WNT16/WNT5A/WNT7A/WNT7B	64	1.29292929292929
GO:0006887	BP	GO:0006887	exocytosis	53/666	351/18614	4.31178065768226e-19	1.94425705802828e-17	1.11469211208261e-17	ADRA2A/ARHGAP44/CADPS/CCL5/CCR2/CDK5R2/CPLX1/F2RL1/GCGR/GRIK5/GRP/LRRK2/NLGN1/NPY/P2RX1/P2RX7/P2RY1/P2RY4/PIK3CD/PIK3CG/PRKCB/PRKCG/PRKN/PTGDR/RAB3B/RAP1A/RIMS1/RIMS3/RIMS4/RPH3A/SEPTIN5/SLC18A2/SLC4A8/SNAP25/SNCA/STX1A/STXBP1/SV2B/SYN2/SYP/SYT10/SYT11/SYT12/SYT2/SYT4/SYT5/SYT7/SYT8/SYT9/TPH1/UNC13C/VAMP2/WNT7A	53	1.50997150997151
GO:0034329	BP	GO:0034329	cell junction assembly	60/666	444/18614	4.85966957157986e-19	2.17138872221046e-17	1.24491248259515e-17	ADGRL3/AGT/APC/BCL2/BDNF/CAV1/CDH4/CHRNB2/CLDN1/CLDN19/CNTNAP1/DKK1/DNER/DRD2/EFNA5/EPHA3/EPHB2/EPHB3/ERBB4/FGF13/FLRT2/GABRB3/GABRG2/GAP43/GPM6A/GPM6B/GRID2/ICAM5/ITGB3/KIRREL3/LHFPL4/MAP1B/MDGA1/MEF2C/NLGN1/NLGN3/NLGN4X/NLGN4Y/NPTX1/NRCAM/NRG3/NRXN1/NRXN2/NTN1/NTRK3/OXT/OXTR/POU4F1/PTK2B/RAP1A/ROBO2/SLITRK3/SNCA/SPTBN2/VCL/VEGFA/WNT11/WNT3A/WNT5A/WNT7A	60	1.35135135135135
GO:0009914	BP	GO:0009914	hormone transport	50/666	315/18614	5.15791491559797e-19	2.28388755046523e-17	1.30941092736423e-17	ADCY5/ADCYAP1/ADRA2A/AGT/AGTR1/CARTPT/CCKAR/CCL5/CGA/CNR1/CPLX1/DRD2/EDN3/EFNA5/F2/F2RL1/F2RL2/FKBP1B/GAL/GALR1/GCG/GHRHR/GNA11/GNAZ/GPER1/GRP/HTR2C/INHBA/ITPR1/KCNQ1/KISS1/LEP/NEUROD1/NKX6-1/NMB/P2RY1/PRKCB/PRKN/SLC18A2/SNAP25/SSTR5/STX1A/SYT7/SYT9/TACR1/TACR2/UCN/UCN3/VGF/VIP	50	1.58730158730159

	ONTOLOGY	ID	Description	GeneRatio	BgRatio	pvalue	p.adjust	qvalue	geneID	Count	richFactor
GO:0099601	BP	GO:0099601	regulation of neurotransmitter receptor activity	24/666	63/18614	5.43860873511956e-19	2.38667517259934e-17	1.36834169021758e-17	ADRB2/CACNG4/CACNG5/CACNG7/CACNG8/CCR2/CNIH3/DLGAP2/EPHB2/GSG1L/HOMER1/LYNX1/MEF2C/NLGN1/NLGN3/NPTX1/NPTX2/NRXN2/OPRM1/PINK1/PTK2B/RELN/SHISA7/SLC6A9	24	3.80952380952381
GO:0098810	BP	GO:0098810	neurotransmitter reuptake	19/666	35/18614	6.102466662002e-19	2.60924654752539e-17	1.49594753070096e-17	ATP1A2/DRD2/DRD4/GDNF/GPM6B/ITGB3/NOS1/PRKN/RAB3B/SLC17A8/SLC18A2/SLC18A3/SLC1A2/SLC22A2/SLC22A3/SLC6A1/SLC6A2/SLC6A3/SNCA	19	5.42857142857143
GO:1990806	BP	GO:1990806	ligand-gated ion channel signaling pathway	19/666	35/18614	6.102466662002e-19	2.60924654752539e-17	1.49594753070096e-17	F2/F2RL2/GNA11/GRIA1/GRIA2/GRIA3/GRIA4/GRID2/GRIK1/GRIK2/GRIK3/GRIK5/GRIN2A/GRIN2B/GRIN2D/GRIN3A/GRIN3B/ITPR1/PTK2B	19	5.42857142857143
GO:0019932	BP	GO:0019932	second-messenger-mediated signaling	48/666	292/18614	6.10505295962197e-19	2.60924654752539e-17	1.49594753070096e-17	ADCY2/ADCYAP1/ADCYAP1R1/AGT/AGTR1/ATP1A2/CACNA1C/CAMK2D/CXCL8/EDN2/EDN3/EDNRB/FKBP1B/GAL/GHRHR/GNAI1/GPR143/GRIN2A/GRIN2B/GRIN2D/GUCY1A1/HPCA/HTR2B/HTR2C/IGF1/ITPR1/LPAR1/LRRK2/MAPT/NDNF/NEUROD1/NMUR2/NOS1/P2RX2/P2RX4/P2RX7/PLA2G4B/PLCG1/PLCG2/PRKG1/PRNP/PTGDR2/PTGFR/PTK2B/SCTR/SOX9/UCN/VEGFA	48	1.64383561643836
GO:0008016	BP	GO:0008016	regulation of heart contraction	41/666	213/18614	6.61080575299779e-19	2.80104398930898e-17	1.60590989117741e-17	ADORA3/ADRA1A/ADRA1B/ADRB1/AGT/APELA/APLN/ATP1A2/ATP2A1/ATP2A3/CACNA1C/CACNA1D/CALCA/CAMK2D/CAV1/CHRM2/DRD2/EDN2/EDN3/EDNRA/EDNRB/EPAS1/FGF13/FKBP1B/HCN1/KCNJ5/KCNQ1/MC3R/NOS1/OXT/P2RX4/PIK3CG/RGS2/SCN1B/SCN2B/SLC1A1/SPX/TH/THRB/TRPC1/UCN	41	1.92488262910798
GO:0046883	BP	GO:0046883	regulation of hormone secretion	44/666	247/18614	7.73981709654389e-19	3.22383059572146e-17	1.84830422546369e-17	ADCY5/ADCYAP1/ADRA2A/AGT/AGTR1/CARTPT/CCKAR/CCL5/CNR1/DRD2/EDN3/EFNA5/F2/F2RL1/F2RL2/FKBP1B/GAL/GALR1/GCG/GHRHR/GNA11/GNAZ/GPER1/GRP/HTR2C/INHBA/ITPR1/KISS1/LEP/NEUROD1/NKX6-1/NMB/P2RY1/PRKCB/PRKN/SNAP25/SSTR5/STX1A/SYT7/SYT9/TACR1/TACR2/UCN/UCN3	44	1.78137651821862

	ONTOLOGY	ID	Description	GeneRatio	BgRatio	pvalue	p.adjust	qvalue	geneID	Count	richFactor
GO:0060047	BP	GO:0060047	heart contraction	44/666	247/18614	7.73981709654389e-19	3.22383059572146e-17	1.84830422546369e-17	ADORA3/ADRA1A/ADRA1B/ADRB1/AGT/APELA/APLN/ATP1A2/ATP2A1/ATP2A3/CACNA1C/CACNA1D/CALCA/CAMK2D/CAV1/CHRM2/DRD2/EDN2/EDN3/EDNRA/EDNRB/EPAS1/FGF12/FGF13/FKBP1B/HCN1/KCNJ5/KCNQ1/MAP2K6/MC3R/NEDD4L/NOS1/OXT/P2RX4/PIK3CG/RGS2/SCN1B/SCN2B/SLC1A1/SPX/TH/THRB/TRPC1/UCN	44	1.78137651821862
GO:0003015	BP	GO:0003015	heart process	45/666	259/18614	8.50247490569802e-19	3.51173648416015e-17	2.0133680073024e-17	ADORA3/ADRA1A/ADRA1B/ADRB1/AGT/APELA/APLN/ATP1A2/ATP2A1/ATP2A3/CACNA1C/CACNA1D/CALCA/CAMK2D/CAV1/CHRM2/DRD2/EDN2/EDN3/EDNRA/EDNRB/EPAS1/FGF12/FGF13/FKBP1B/HCN1/KCNJ5/KCNQ1/MAP2K6/MC3R/NEDD4L/NOS1/NOX4/OXT/P2RX4/PIK3CG/RGS2/SCN1B/SCN2B/SLC1A1/SPX/TH/THRB/TRPC1/UCN	45	1.73745173745174
GO:0008038	BP	GO:0008038	neuron recognition	21/666	46/18614	9.30936544446437e-19	3.81296092996187e-17	2.18606765744133e-17	CNR1/CNTN2/CNTN4/CNTN6/CRTAC1/DSCAML1/EPHA3/EPHA4/EPHB2/EPHB3/FEZF2/FOXG1/GAP43/IGSF9/MYPN/NCAM2/NDN/NEXN/NRCAM/PALLD/ROBO2	21	4.56521739130435
GO:0017157	BP	GO:0017157	regulation of exocytosis	39/666	194/18614	9.82040226875485e-19	3.95633419269919e-17	2.26826720219644e-17	ADRA2A/CADPS/CCR2/CDK5R2/CPLX1/F2RL1/LRRK2/NLGN1/NPY/P2RX1/P2RY1/P2RY4/PRKCB/PRKCG/PRKN/RAB3B/RAP1A/RIMS1/RIMS3/RIMS4/RPH3A/SEPTIN5/SLC4A8/SNCA/STX1A/STXBP1/SV2B/SYP/SYT10/SYT11/SYT12/SYT2/SYT4/SYT5/SYT7/SYT8/SYT9/VAMP2/WNT7A	39	2.01030927835052
GO:0045664	BP	GO:0045664	regulation of neuron differentiation	39/666	194/18614	9.82040226875485e-19	3.95633419269919e-17	2.26826720219644e-17	ADRA2C/ASCL1/ASPM/ATOH1/BCL2/BDNF/CNTN2/CNTN4/CPNE1/CSNK1E/CXCL12/DKK1/EDNRB/FEZF1/FEZF2/FGFR1/FOXG1/LRRK2/MAG/MAP1B/MEF2C/NEUROD1/NGF/NKX6-1/NLGN1/NRCAM/NTF4/PHOX2B/PROX1/RELN/S1PR5/SHH/SOX2/SOX9/TLX3/TP73/VWC2/WDR62/WNT3A	39	2.01030927835052
GO:0048639	BP	GO:0048639	positive regulation of developmental growth	36/666	165/18614	1.29267415569868e-18	5.16544184980406e-17	2.96147943072773e-17	BCL2/BDNF/CACNG7/CDH4/CDK1/CPNE5/CPNE9/CSF1/CXCL12/DRD2/EFNA5/ERBB4/GHR/GHRHR/IGF1/L1CAM/LEP/LIMK1/MAP1B/MAPT/MEF2C/NEDD4L/NGF/NTN1/PRKN/PROX1/RASAL1/RIMS1/SLC6A3/SYT2/SYT4/TBX20/VEGFA/WNT2/WNT3/WNT3A	36	2.18181818181818

	ONTOLOGY	ID	Description	GeneRatio	BgRatio	pvalue	p.adjust	qvalue	geneID	Count	richFactor
GO:0032412	BP	GO:0032412	regulation of monoatomic ion transmembrane transporter activity	46/666	278/18614	2.577174046593 8e-18	1.021516970887 78e-16	5.856617082115 11e-17	ADRA2A/ADRB2/ANK3/ATP1A2/ATP2A1/CACNA1D/CACNG4/CACNG5/CACNG7/CACNG8/CAMK2D/CAV1/CCR2/CNIH3/DRD4/EDNRA/EPHB2/FGF12/FGF13/FKBP1B/GAL/GRP/GSG1L/HCN1/HOMER1/HPCA/KCNA1/KCNQ1/MEF2C/MMP9/NEDD4L/NLGN1/NLGN3/NOS1/NTSR1/OPRM1/PINK1/PLCG2/PRNP/PTK2B/RELN/SCN1B/SCN2B/SHISA7/SLC6A9/VAMP2	46	1.654676258992 81
GO:0007613	BP	GO:0007613	memory	31/666	124/18614	5.063736152609 38e-18	1.990141339900 04e-16	1.140998740035 85e-16	ASIC1/BDNF/CALB1/CAMK4/CHRNA7/CHRNB2/CNR1/CX3CR1/DRD2/FGF13/GRIA1/GRIN2A/MAPT/NGF/NTF4/OXT/OXTR/PAK6/PRNP/PTN/RELN/SHISA7/SLC1A1/SLC2A4/SLC6A1/SORCS3/SYT1 1/SYT4/TACR1/TH/TUBA1A	31	2.5
GO:0051402	BP	GO:0051402	neuron apoptotic process	44/666	259/18614	5.101888277261 55e-18	1.990141339900 04e-16	1.140998740035 85e-16	ASCL1/AXL/BCL2/BCL2L1/BDNF/BID/CASP7/CCND1/CHL1/CNTFR/CRLF1/CX3CR1/FAS/FZD9/GDNF/GRID2/GRIK2/GRIK5/GRM4/IL6ST/KIF14/MAG/MEF2C/NDNF/NEFL/NGF/NGFR/NR3C1/NSMF/NTF4/PINK1/POU4F1/PRKCG/PRKN/PRNP/PTK2B/SEMA3E/SIGMAR1/SLC1A1/SNCA/STXBP1/THRB/TUBA1A/TYRO3	44	1.698841698841 7
GO:0006937	BP	GO:0006937	regulation of muscle contraction	36/666	173/18614	6.671818469203 43e-18	2.582046281585 42e-16	1.480352925160 18e-16	ADRA1A/ADRA1B/ADRA2A/ADRA2C/ADRB2/ATP1A2/ATP2A1/CACNA1C/CALCA/CAMK2D/CAV1/CHRM2/CHRNA3/CHRNB4/EDN2/FGF13/FKBP1B/GPER1/GUCY1A1/KCNA1/KCNQ1/NMU/NMUR2/NOS1/OXT/OXTR/P2RX1/P2RX4/PIK3CG/PRKG1/RGS2/SPX/SSTR2/TACR1/TACR2/UCN	36	2.080924855491 33
GO:0032409	BP	GO:0032409	regulation of transporter activity	49/666	324/18614	9.230131892252 73e-18	3.544226425814 23e-16	2.031995318713 86e-16	ADRA2A/ADRB2/ANK3/ATP1A2/ATP2A1/BCL2/CACNA1D/CACNG4/CACNG5/CACNG7/CACNG8/CAMK2D/CAV1/CCR2/CNIH3/DRD4/EDNRA/EPHB2/FGF12/FGF13/FKBP1B/GAL/GRP/GSG1L/HCN1/HOMER1/HPCA/KCNA1/KCNQ1/MEF2C/MMP9/NEDD4L/NLGN1/NLGN3/NOS1/NTSR1/OPRM1/PINK1/PLCG2/PRNP/PTK2B/RELN/SCN1B/SCN2B/SHISA7/SLC6A9/SNCA/TWIST1/VAMP2	49	1.512345679012 35
GO:0035418	BP	GO:0035418	protein localization to synapse	25/666	78/18614	1.276850459120 54e-17	4.864899229904 99e-16	2.789170688752 08e-16	ARHGAP44/CACNG7/CPLX1/DLG2/ERBB4/GPC6/GRIN2A/HOMER1/KIF5A/KIF5C/LGI1/LHFPL4/LRRC7/MAPT/NLGN1/NPTX1/NRXN1/NRXN2/NSG1/RAP1A/RELN/SLITRK3/SNAP25/WNT5A/WNT7A	25	3.205128205128 21

	ONTOLOGY	ID	Description	GeneRatio	BgRatio	pvalue	p.adjust	qvalue	geneID	Count	richFactor
GO:0045761	BP	GO:0045761	regulation of adenylate cyclase activity	21/666	51/18614	1.29688988291064e-17	4.90324136500446e-16	2.81115321178282e-16	ADCYAP1/ADORA3/ADRB1/ADRB2/ADRB3/CACNA1C/CACNA1D/CALCA/CCR2/DRD5/EDNRA/EDNRB/GALR1/GALR3/GHRHR/GNG7/GRM2/HPCA/LTB4R2/P2RY13/VIPR2	21	4.11764705882353
GO:0006940	BP	GO:0006940	regulation of smooth muscle contraction	23/666	64/18614	1.37738077288268e-17	5.16780648757129e-16	2.96283513781193e-16	ADRA1A/ADRA2A/ADRA2C/ADRB2/ATP1A2/CALCA/CAV1/CHRM2/CHRNA3/CHRNB4/EDN2/GPER1/GUCY1A1/NMU/NMUR2/OXT/OXTR/P2RX1/PRKG1/RGS2/SPX/TACR1/TACR2	23	3.59375
GO:0010959	BP	GO:0010959	regulation of metal ion transport	56/666	423/18614	2.00446962377945e-17	7.46361227339091e-16	4.27907909318787e-16	ADCYAP1R1/ADRA2A/ADRB2/AGT/ANK3/ATP1A2/ATP2A1/BCL2/BDKRB1/CACNA1C/CACNA1D/CAMK2A/CAMK2B/CAMK2D/CAV1/CCL5/CXCL12/DRD2/DRD4/EDN3/F2/FGF12/FGF13/FKBP1B/GAL/GCG/GPER1/GRIN3B/GRP/HCRT/HOMER1/HPCA/ITGB3/KCNA1/KCNQ1/NEDD4L/NOS1/NPSR1/NTSR1/OPRD1/P2RX1/P2RX2/P2RX4/P2RX7/PIK3CG/PLCG1/PLCG2/PRNP/PTK2B/SCN1B/SCN2B/SNCA/TRPC1/UCN/VAMP2/VIP	56	1.32387706855792
GO:0051592	BP	GO:0051592	response to calcium ion	33/666	149/18614	2.22749126628866e-17	8.23166885248778e-16	4.71942550047862e-16	CAMK2D/CAV1/CCND1/CPNE1/CPNE5/CPNE9/FOSB/HOMER1/HPCA/KCNMB2/MEF2C/NLGN1/P2RX7/PENK/PLCG2/PTK2B/RASAL1/RYR1/RYR3/SLC6A1/SYT10/SYT11/SYT12/SYT2/SYT4/SYT5/SYT7/SYT8/SYT9/TRPC1/TUBA1A/TXNIP/WNT5A	33	2.21476510067114
GO:0050795	BP	GO:0050795	regulation of behavior	24/666	73/18614	2.93540813627098e-17	1.07241541625295e-15	6.14843083859112e-16	ADRB1/CHRNB2/CNR1/DRD2/EPHB2/GHRHR/GPR171/GRP/GRPR/HTR1D/HTR2B/INSL5/KCNA2/LEPR/MC1R/MC3R/MEF2C/NLGN1/NMU/NPSR1/NPY/PENK/RELN/UCN	24	3.28767123287671
GO:1904062	BP	GO:1904062	regulation of monoatomic cation transmembrane transport	50/666	346/18614	2.94559676895521e-17	1.07241541625295e-15	6.14843083859112e-16	ADCYAP1R1/ADRA2A/ANK3/ATP1A2/ATP2A1/BCL2/BDKRB1/CACNA1C/CACNA1D/CAMK2D/CAV1/DRD4/EDN3/EDNRA/F2/FGF12/FGF13/FKBP1B/GAL/GPER1/GRIN2A/GRIN2B/GRIN2D/GRP/HCN1/HOMER1/HPCA/ITGB3/KCNA1/KCNQ1/MMP9/NEDD4L/NOS1/NPSR1/NTSR1/P2RX1/P2RX2/P2RX4/P2RX7/PIK3CG/PLCG1/PLCG2/PRNP/PTK2B/SCN1B/SCN2B/SNCA/TRPC1/TWIST1/VAMP2	50	1.44508670520231
GO:0043279	BP	GO:0043279	response to alkaloid	27/666	98/18614	4.92405115093583e-17	1.77953760344482e-15	1.02025425162966e-15	BCHE/BCL2L1/CACNG4/CASP7/CHRNB2/CNR1/DRD2/DRD4/DRD5/FOSB/GHR/GRM2/HOMER1/OPRM1/OXT/OXTR/PENK/PRKCG/PTK2B/RYR1/RYR3/SLC1A1/SLC1A2/SLC6A1/SLC6A3/TACR1/TH	27	2.75510204081633

	ONTOLOGY	ID	Description	GeneRatio	BgRatio	pvalue	p.adjust	qvalue	geneID	Count	richFactor
GO:0097305	BP	GO:0097305	response to alcohol	42/666	252/18614	6.08226066562384e-17	2.18206650887162e-15	1.25103432976374e-15	A2M/ADCY2/ADCY3/ADCY5/ADCYAP1R1/BCL2L1/CCND1/CCR7/CDK1/CHRNB2/CLDN1/CNR1/DRD2/DRD4/EFNA5/FOSB/GNAI1/GNG2/GRIN2A/GRIN2B/GRIN3A/INHBA/LEP/MAOB/NEFL/NTRK3/OPRM1/OXT/P2RY4/PENK/PTGDR/PTGER4/PTGFR/PTK2B/RGS2/SLC2A4/SLC6A3/SLIT2/SLIT3/TACR1/TH/UCN3	42	1.66666666666667
GO:0031279	BP	GO:0031279	regulation of cyclase activity	22/666	61/18614	6.42255607617009e-17	2.28745384886783e-15	1.31145557710964e-15	ADCYAP1/ADORA3/ADRB1/ADRB2/ADRB3/CACNA1C/CACNA1D/CALCA/CCR2/DRD5/EDNRA/EDNRB/GALR1/GALR3/GHRHR/GNG7/GRM2/HPCA/LTB4R2/NOS1/P2RY13/VIPR2	22	3.60655737704918
GO:0050769	BP	GO:0050769	positive regulation of neurogenesis	40/666	232/18614	1.01307656136459e-16	3.58220956770286e-15	2.05377202178949e-15	ASCL1/ASPM/BDNF/CAMK2B/CDH4/CX3CR1/CXCL12/DRD2/EFNA5/EPHA4/EPHB2/FOXG1/FZD3/GPER1/IL6ST/L1CAM/LIMK1/MAG/MAP1B/MAPT/NEFL/NGF/NKX6-1/NTN1/OPRM1/PLXNA1/PLXNA2/PLXNA3/PTN/RELN/ROBO2/SHH/SLIT2/SOX10/TP73/VEGFA/WDR62/WNT2/WNT3/WNT3A	40	1.72413793103448
GO:0071248	BP	GO:0071248	cellular response to metal ion	37/666	200/18614	1.36601194157799e-16	4.79567763775417e-15	2.74948418616864e-15	ANK3/CAMK2D/CLDN1/CPNE1/CPNE5/CPNE9/DLG2/FOSB/HPCA/KCNA1/LRRK2/MEF2C/MMP9/NLGN1/NPTX1/P2RX4/PLCG2/PRKN/PRNP/RASAL1/RYR1/RYR3/SHH/SLC1A1/SNCA/SYT10/SYT11/SYT12/SYT2/SYT4/SYT5/SYT7/SYT8/SYT9/TH/TUBA1A/WNT5A	37	1.85
GO:0045927	BP	GO:0045927	positive regulation of growth	42/666	258/18614	1.46056897442476e-16	5.0912741200693e-15	2.91895718143717e-15	BCL2/BDNF/CACNG7/CDH4/CDK1/CPNE5/CPNE9/CSF1/CXCL12/DRD2/EFNA5/ERBB4/F2/GHR/GHRHR/IGF1/L1CAM/LEP/LGI1/LIMK1/MAP1B/MAPT/MEF2C/NEDD4L/NGF/NTN1/PRKN/PROX1/PRSS2/PTK2B/RASAL1/RIMS1/SLC44A4/SLC6A3/SYT2/SYT4/TBX20/UCN/VEGFA/WNT2/WNT3/WNT3A	42	1.62790697674419
GO:0097553	BP	GO:0097553	calcium ion transmembrane import into cytosol	37/666	201/18614	1.61877881814589e-16	5.60302668393455e-15	3.21235796603154e-15	ADCYAP1R1/ADRA1A/ATP1A2/BCL2/BDKRB1/CACNA1A/CACNA1C/CACNA1D/CAMK2D/CAV1/CCR7/DRD2/F2/FKBP1B/GPER1/GRIN2A/GRIN2B/GRIN2D/HTR2B/HTR2C/ITGB3/ITPR1/NOS1/NPSR1/NTSR1/P2RX1/P2RX2/P2RX4/P2RX7/PLCB4/PLCG1/PLCG2/PRNP/PTK2B/RYR1/SNCA/TRPC1	37	1.8407960199005

	ONTOLOGY	ID	Description	GeneRatio	BgRatio	pvalue	p.adjust	qvalue	geneID	Count	richFactor
GO:0021954	BP	GO:0021954	central nervous system neuron development	25/666	86/18614	1.756788271129 68e-16	6.038191854966 71e-15	3.461849246826 76e-15	ASCL1/CHRNB2/CNTN2/DCLK1/DRD2/EPHA4/EPHB2/EPHB3/FEZF2/FOXG1/GABRB1/HSP90AB1/LEP/MAP2/MAPT/NDNF/NPY/NRP2/PHOX2B/PLXNA1/PLXNA3/SCN1B/SEMA3E/SLIT2/SOX1	25	2.906976744186 05
GO:0017156	BP	GO:0017156	calcium-ion regulated exocytosis	22/666	65/18614	3.079507601773 5e-16	1.043846887083 91e-14	5.984640181450 2e-15	ADRA2A/CADPS/CDK5R2/RIMS1/RIMS3/RIMS4/RPH3A/SNAP25/STX1A/STXBP1/SYN2/SYT10/SYT11/SYT12/SYT2/SYT4/SYT5/SYT7/SYT8/SYT9/UNC13C/VAMP2	22	3.384615384615 39
GO:0051339	BP	GO:0051339	regulation of lyase activity	22/666	65/18614	3.079507601773 5e-16	1.043846887083 91e-14	5.984640181450 2e-15	ADCYAP1/ADORA3/ADRB1/ADRB2/ADRB3/CACNA1C/CACNA1D/CALCA/CCR2/DRD5/EDNRA/EDNRB/GALR1/GALR3/GHRHR/GNG7/GRM2/HPCA/LTB4R2/NOS1/P2RY13/VIPR2	22	3.384615384615 39
GO:0098657	BP	GO:0098657	import into cell	42/666	265/18614	3.922634570657 29e-16	1.320530747587 71e-14	7.570939254253 46e-15	AGT/ATP1A2/CACNA1A/CACNA1C/CACNA1D/DRD2/DRD4/GDNF/GPM6B/ITGA4/ITGB3/KCNJ14/KCNJ5/KCNJ9/KCNQ1/NOS1/NTSR1/P2RX1/PRKN/PRNP/RAB3B/RGS2/SLC17A8/SLC18A2/SLC18A3/SLC1A1/SLC1A2/SLC1A7/SLC22A2/SLC22A3/SLC29A1/SLC38A3/SLC38A5/SLC6A1/SLC6A2/SLC6A3/SLC6A6/SLC6A9/SLC7A11/SNAP25/SNCA/SPX	42	1.584905660377 36
GO:0043523	BP	GO:0043523	regulation of neuron apoptotic process	38/666	219/18614	4.839028536350 07e-16	1.607015220010 85e-14	9.213427732438 93e-15	ASCL1/AXL/BCL2/BCL2L1/BDNF/CCND1/CHL1/CNTFR/CRLF1/CX3CR1/FZD9/GDNF/GRID2/GRIK2/GRIK5/GRM4/IL6ST/KIF14/MAG/MEF2C/NDNF/NEFL/NGF/NR3C1/NSMF/NTF4/PINK1/POU4F1/PRKCG/PRKN/PRNP/PTK2B/SEMA3E/SIGMAR1/SLC1A1/SNCA/STXBP1/TYRO3	38	1.735159817351 6
GO:1901215	BP	GO:1901215	negative regulation of neuron death	38/666	219/18614	4.839028536350 07e-16	1.607015220010 85e-14	9.213427732438 93e-15	AXL/BCL2/BCL2L1/BDNF/CCND1/CHL1/CNTFR/CRLF1/CSF1/CX3CR1/FZD9/GDNF/GHR/GRIK2/IL6ST/KIF14/LRRK2/MAG/MEF2C/NDNF/NEFL/NGF/NPY/NTF4/PINK1/POU4F1/PRKCG/PRKN/PTK2B/SEMA3E/SLC1A1/SLC7A11/SNCA/STXBP1/TYRO3/UCN/WNT1/WNT3A	38	1.735159817351 6
GO:0071867	BP	GO:0071867	response to monoamine	27/666	107/18614	5.620217468201 09e-16	1.841557923747 23e-14	1.055812081570 13e-14	ADCY5/CALY/CHRM2/CHRM4/CHRM5/DRD2/DRD4/DRD5/GNA11/GNAO1/GNAQ/GNAZ/GNG2/HTR1D/HTR2B/HTR2C/HTR3A/HTR3C/HTR3E/HTR7/KCNQ1/LRRK2/NSG1/PENK/PRKN/SLC1A1/SNCA	27	2.523364485981 31

	ONTOLOGY	ID	Description	GeneRatio	BgRatio	pvalue	p.adjust	qvalue	geneID	Count	richFactor
GO:0071869	BP	GO:0071869	response to catecholamine	27/666	107/18614	5.62021746820109e-16	1.84155792374723e-14	1.05581208157013e-14	ADCY5/CALY/CHRM2/CHRM4/CHRM5/DRD2/DRD4/DRD5/GNA11/GNAO1/GNAQ/GNAZ/GNG2/HTR1D/HTR2B/HTR2C/HTR3A/HTR3C/HTR3E/HTR7/KCNQ1/LRRK2/NSG1/PENK/PRKN/SLC1A1/SNCA	27	2.52336448598131
GO:0030516	BP	GO:0030516	regulation of axon extension	25/666	90/18614	5.79382713232907e-16	1.88587154671506e-14	1.08121821075252e-14	CDH4/CXCL12/L1CAM/LIMK1/MAG/MAP1B/MAP2/MAPT/NKX6-1/NRCAM/NTN1/OLFM1/PLXNA3/RTN4R/SEMA3D/SEMA3E/SEMA3F/SEMA3G/SEMA6A/SEMA6D/TNR/VEGFA/WNT3/WNT3A/WNT5A	25	2.77777777777778
GO:0008361	BP	GO:0008361	regulation of cell size	34/666	178/18614	8.71453054435054e-16	2.81788931746598e-14	1.61556774703784e-14	AKT3/BDNF/CDH4/CXCL12/EFNA5/FGF13/FSTL4/L1CAM/LIMK1/MAG/MAP1B/MAP2/MAPT/NGF/NKX6-1/NRCAM/NTN1/OLFM1/P2RX7/PLXNA3/RTN4R/SCTR/SEMA3D/SEMA3E/SEMA3F/SEMA3G/SEMA6A/SEMA6D/TNR/UCN/VEGFA/WNT3/WNT3A/WNT5A	34	1.91011235955056
GO:0035235	BP	GO:0035235	ionotropic glutamate receptor signaling pathway	15/666	26/18614	9.26054534488681e-16	2.97487453399468e-14	1.70557137174145e-14	GRIA1/GRIA2/GRIA3/GRIA4/GRID2/GRIK1/GRIK2/GRIK3/GRIK5/GRIN2A/GRIN2B/GRIN2D/GRIN3A/GRIN3B/PTK2B	15	5.76923076923077
GO:0030072	BP	GO:0030072	peptide hormone secretion	39/666	238/18614	1.44898031660322e-15	4.62450536110704e-14	2.65134675840522e-14	ADCY5/ADCYAP1/ADRA2A/CARTPT/CCL5/CNR1/CPLX1/DRD2/EDN3/EFNA5/F2/F2RL1/F2RL2/FKBP1B/GAL/GCG/GHRHR/GNA11/GNAZ/GPER1/GRP/HTR2C/ITPR1/KISS1/LEP/NEUROD1/NKX6-1/PRKCB/PRKN/SNAP25/SSTR5/STX1A/SYT7/SYT9/TACR2/UCN/UCN3/VGF/VIP	39	1.63865546218487
GO:0051051	BP	GO:0051051	negative regulation of transport	57/666	483/18614	1.93255159238624e-15	6.12805875908281e-14	3.51337223281355e-14	ADRA2A/ADRA2C/ANK3/APOC1/ASIC1/ATP1A2/BCL2/CAMK2D/CARTPT/CAV1/CCR2/CNR1/DRD2/DRD4/EPHA3/EPHB2/F2RL1/FKBP1B/GABRE/GNAZ/GPM6B/GRP/HCRT/INHBA/ITGB3/LEP/LRRK2/MAOB/MAP1B/MMP9/NEDD4L/NMB/NOS1/NPSR1/NPY5R/NTSR1/OPRM1/OXT/OXTR/P2RY1/PRKCB/PRKG2/PRKN/PTGER3/PTK2B/RAP1A/RGS2/SHH/SLC6A9/SNCA/SPX/SYT11/SYT4/TACR2/TWIST1/UCN/VIP	57	1.18012422360248
GO:0051924	BP	GO:0051924	regulation of calcium ion transport	41/666	266/18614	2.40024570884469e-15	7.5623126019049e-14	4.33566650646237e-14	ADCYAP1R1/ADRA2A/AGT/ATP1A2/ATP2A1/BCL2/BDKRB1/CACNA1C/CACNA1D/CAMK2A/CAMK2B/CAMK2D/CAV1/CCL5/CXCL12/DRD4/F2/FKBP1B/GCG/GPER1/GRIN3B/HCRT/HOMER1/HPCA/ITGB3/NOS1/NPSR1/NTSR1/OPRD1/P2RX1/P2RX2/P2RX4/P2RX7/PIK3CG/PLCG1/PLCG2/PRNP/PTK2B/SNCA/TRPC1/UCN	41	1.54135338345865

	ONTOLOGY	ID	Description	GeneRatio	BgRatio	pvalue	p.adjust	qvalue	geneID	Count	richFactor
GO:0071868	BP	GO:0071868	cellular response to monoamine stimulus	26/666	104/18614	2.50210438545908e-15	7.77006551662261e-14	4.45477654612598e-14	ADCY5/CALY/CHRM2/CHRM4/CHRM5/DRD2/DRD4/DRD5/GNA11/GNAO1/GNAQ/GNAZ/GNG2/HTR1D/HTR2B/HTR2C/HTR3A/HTR3C/HTR3E/HTR7/KCNQ1/LRRK2/NSG1/PRKN/SLC1A1/SNCA	26	2.5
GO:0071870	BP	GO:0071870	cellular response to catecholamine stimulus	26/666	104/18614	2.50210438545908e-15	7.77006551662261e-14	4.45477654612598e-14	ADCY5/CALY/CHRM2/CHRM4/CHRM5/DRD2/DRD4/DRD5/GNA11/GNAO1/GNAQ/GNAZ/GNG2/HTR1D/HTR2B/HTR2C/HTR3A/HTR3C/HTR3E/HTR7/KCNQ1/LRRK2/NSG1/PRKN/SLC1A1/SNCA	26	2.5
GO:0090257	BP	GO:0090257	regulation of muscle system process	40/666	254/18614	2.51361224240691e-15	7.77006551662261e-14	4.45477654612598e-14	ADRA1A/ADRA1B/ADRA2A/ADRA2C/ADRB2/AGT/ATP1A2/ATP2A1/CACNA1C/CALCA/CAMK2B/CAMK2D/CAV1/CHRM2/CHRNA3/CHRNB4/EDN2/FGF13/FKBP1B/GPER1/GUCY1A1/IGF1/IL6ST/KCNA1/KCNQ1/NMU/NMUR2/NOS1/OXT/OXTR/P2RX1/P2RX4/PIK3CG/PRKG1/RGS2/SPX/SSTR2/TACR1/TACR2/UCN	40	1.5748031496063
GO:0042310	BP	GO:0042310	vasoconstriction	23/666	79/18614	2.75665279971536e-15	8.46809281912561e-14	4.85497338476185e-14	ADRA1A/ADRA1D/ADRA2A/ADRA2C/AGT/AGTR1/ATP1A2/AVPR1B/BDKRB2/CAV1/DRD5/EDN2/EDN3/EDNRA/EDNRB/HTR1D/HTR2B/HTR7/KCNMB2/LEP/OXTR/P2RX1/TACR1	23	2.91139240506329
GO:0002790	BP	GO:0002790	peptide secretion	39/666	243/18614	2.96031565058031e-15	8.98189057148531e-14	5.14954672803259e-14	ADCY5/ADCYAP1/ADRA2A/CARTPT/CCL5/CNR1/CPLX1/DRD2/EDN3/EFNA5/F2/F2RL1/F2RL2/FKBP1B/GAL/GCG/GHRHR/GNA11/GNAZ/GPER1/GRP/HTR2C/ITPR1/KISS1/LEP/NEUROD1/NKX6-1/PRKCB/PRKN/SNAP25/SSTR5/STX1A/SYT7/SYT9/TACR2/UCN/UCN3/VGF/VIP	39	1.60493827160494
GO:0071241	BP	GO:0071241	cellular response to inorganic substance	38/666	231/18614	2.96046037147634e-15	8.98189057148531e-14	5.14954672803259e-14	ANK3/CAMK2D/CLDN1/CPNE1/CPNE5/CPNE9/DLG2/FOSB/HPCA/KCNA1/LRRK2/MEF2C/MMP9/NLGN1/NPTX1/P2RX4/PHOX2B/PLCG2/PRKN/PRNP/RASAL1/RYR1/RYR3/SHH/SLC1A1/SNCA/SYT10/SYT11/SYT12/SYT2/SYT4/SYT5/SYT7/SYT8/SYT9/TH/TUBA1A/WNT5A	38	1.64502164502165
GO:0042063	BP	GO:0042063	gliogenesis	45/666	320/18614	3.37239360902165e-15	1.01689046554242e-13	5.83009214811169e-14	ADGRG1/ASCL1/ATOH1/CCR2/CDK1/CDK5R2/CLU/CNTN2/CNTNAP1/COL3A1/CSF1/CX3CR1/DNER/EMX1/EPHA4/F2/FOXG1/GAP43/GPM6B/IL6ST/LEPR/LPAR1/MAG/MAPT/NDN/NKX2-1/NKX6-1/NR3C1/NTN1/NTRK3/P2RX4/P2RY1/PENK/PHOX2B/PTK2B/PTN/RELN/SHH/SOX1/SOX10/SOX2/SOX9/TP73/TUBA1A/VAX1	45	1.40625

	ONTOLOGY	ID	Description	GeneRatio	BgRatio	pvalue	p.adjust	qvalue	geneID	Count	richFactor
GO:0010469	BP	GO:0010469	regulation of signaling receptor activity	33/666	177/18614	4.87369009698538e-15	1.46062114796849e-13	8.37411321542354e-14	ADRA2A/ADRA2C/ADRB2/CACNG4/CACNG5/CACNG7/CACNG8/CCR2/CGA/CNIH3/DKK1/DKK4/DLGAP2/EPHB2/GSG1L/HOMER1/LY6E/LY6G6D/LY6H/LYNX1/MEF2C/NLGN1/NLGN3/NPTX1/NPTX2/NRXN2/OPRM1/P2RY1/PINK1/PTK2B/RELN/SHISA7/SLC6A9	33	1.86440677966102
GO:0015833	BP	GO:0015833	peptide transport	40/666	259/18614	4.95029530487511e-15	1.47458796505825e-13	8.45418853661924e-14	ADCY5/ADCYAP1/ADRA2A/CARTPT/CCL5/CNR1/CPLX1/DRD2/EDN3/EFNA5/F2/F2RL1/F2RL2/FKBP1B/GAL/GCG/GHRHR/GNA11/GNAZ/GPER1/GRP/HTR2C/ITPR1/KISS1/LEP/NEUROD1/NKX6-1/PRKCB/PRKN/SLC7A11/SNAP25/SSTR5/STX1A/SYT7/SYT9/TACR2/UCN/UCN3/VGF/VIP	40	1.54440154440154
GO:1902414	BP	GO:1902414	protein localization to cell junction	26/666	107/18614	5.26081393250431e-15	1.55764460712402e-13	8.93037342886115e-14	ARHGAP44/CACNG7/CPLX1/DLG2/ERBB4/GPC6/GRIN2A/HOMER1/KIF5A/KIF5C/LGI1/LHFPL4/LRRC7/MAPT/NLGN1/NPTX1/NRXN1/NRXN2/NSG1/RAP1A/RELN/SLITRK3/SNAP25/VCL/WNT5A/WNT7A	26	2.42990654205607
GO:1903351	BP	GO:1903351	cellular response to dopamine	24/666	90/18614	6.14761408883475e-15	1.80931276925885e-13	1.03732511287807e-13	ADCY5/CALY/CHRM2/CHRM4/CHRM5/DRD2/DRD4/DRD5/GNA11/GNAO1/GNAQ/GNAZ/GNG2/HTR1D/HTR2B/HTR2C/HTR3A/HTR3C/HTR3E/HTR7/LRRK2/NSG1/PRKN/SLC1A1	24	2.66666666666667
GO:0032535	BP	GO:0032535	regulation of cellular component size	47/666	354/18614	7.52666887516582e-15	2.20199866199048e-13	1.26246194102875e-13	AKT3/BDNF/CCR7/CDH4/CNTN2/CXCL12/EFNA5/F2RL1/FGF13/FSTL4/L1CAM/LIMK1/MAG/MAP1B/MAP2/MAPT/NEFL/NGF/NKX6-1/NRCAM/NTN1/OLFM1/P2RX7/PIK3R2/PLXNA3/PTK2B/RAB3B/RTN4R/SCTR/SEMA3D/SEMA3E/SEMA3F/SEMA3G/SEMA6A/SEMA6D/SLIT2/SPTBN2/TENM1/TMOD2/TNR/UCN/VEGFA/WNT3/WNT3A/WNT5A/WNT7A/WNT7B	47	1.32768361581921
GO:1903350	BP	GO:1903350	response to dopamine	24/666	91/18614	8.07307643619229e-15	2.33884202372444e-13	1.34091772715325e-13	ADCY5/CALY/CHRM2/CHRM4/CHRM5/DRD2/DRD4/DRD5/GNA11/GNAO1/GNAQ/GNAZ/GNG2/HTR1D/HTR2B/HTR2C/HTR3A/HTR3C/HTR3E/HTR7/LRRK2/NSG1/PRKN/SLC1A1	24	2.63736263736264
GO:2000300	BP	GO:2000300	regulation of synaptic vesicle exocytosis	18/666	46/18614	8.1371716390006e-15	2.33884202372444e-13	1.34091772715325e-13	LRRK2/NLGN1/NPY/P2RX1/P2RY1/P2RY4/PRKCB/PRKCG/RAP1A/RIMS1/RIMS3/RIMS4/SEPTIN5/SLC4A8/SV2B/SYP/SYT4/WNT7A	18	3.91304347826087
GO:2001222	BP	GO:2001222	regulation of neuron migration	18/666	46/18614	8.1371716390006e-15	2.33884202372444e-13	1.34091772715325e-13	ADGRG1/CAMK2A/CAMK2B/COL3A1/DRD2/FLRT2/IGSF10/NEXMIF/NKX6-1/NRG3/NSMF/NTNG1/RELN/SEMA6A/SOX14/UNC5C/UNC5D/WDR62	18	3.91304347826087

	ONTOLOGY	ID	Description	GeneRatio	BgRatio	pvalue	p.adjust	qvalue	geneID	Count	richFactor
GO:0042755	BP	GO:0042755	eating behavior	17/666	40/18614	8.69081057421662e-15	2.48344964955085e-13	1.42382496371958e-13	ADRB3/CNTN2/GDF15/HCRT/LEP/NMUR2/NPSR1/NPY/NPY5R/OPRD1/OPRM1/OXT/OXTR/P2RY1/TACR1/TH/UCHL1	17	4.25
GO:0048511	BP	GO:0048511	rhythmic process	43/666	303/18614	1.01620212747042e-14	2.88707136214862e-13	1.65523157605009e-13	A2M/ADRB1/AXL/CARTPT/CDK1/CGA/CHRNB2/CSNK1E/DRD2/DRD4/GABRB1/GHRHR/GNA11/GNAQ/GRIN3A/HOMER1/HTR7/INHBA/KCNA2/KISS1/LEP/MAP2K6/MAPK10/MC3R/NGFR/NKX2-1/NLGN1/NPY5R/OPRM1/OXTR/PRKCG/PROKR1/PROX1/PTN/ROBO2/SLIT2/SLIT3/SOX14/TH/TOP2A/TPH1/TWIST1/TYRO3	43	1.41914191419142
GO:0050919	BP	GO:0050919	negative chemotaxis	18/666	47/18614	1.27530587381634e-14	3.60237262632605e-13	2.06533201706373e-13	EFNA5/FLRT2/ITGB3/NRG3/NRP2/NTN1/PLXNA3/ROBO2/SEMA3D/SEMA3E/SEMA3F/SEMA3G/SEMA6A/SEMA6D/SLIT2/SLIT3/UNC5C/WNT5A	18	3.82978723404255
GO:0051968	BP	GO:0051968	positive regulation of synaptic transmission, glutamatergic	16/666	35/18614	1.30253340358215e-14	3.65825810206072e-13	2.09737258429438e-13	CACNG4/CACNG5/CACNG7/CACNG8/CCR2/GRIN2A/GRIN2B/GRIN2D/NLGN1/NLGN3/NRXN1/OXTR/PTK2B/RELN/STXBP1/TNR	16	4.57142857142857
GO:0051480	BP	GO:0051480	regulation of cytosolic calcium ion concentration	20/666	61/18614	1.40662566716488e-14	3.90596901362452e-13	2.23939156170109e-13	CALB1/CALB2/CALCA/CALCB/CAV1/CNR1/F2/FKBP1B/FZD9/GPER1/GRIA1/ITPR1/NPY/P2RX1/P2RY1/P2RY4/RYR1/SV2B/TRPC1/WNT5A	20	3.27868852459016
GO:0097120	BP	GO:0097120	receptor localization to synapse	20/666	61/18614	1.40662566716488e-14	3.90596901362452e-13	2.23939156170109e-13	ARHGAP44/CACNG4/CACNG5/CACNG7/CACNG8/CPLX1/ERBB4/GPC6/ITGB3/KIF5A/KIF5C/LGI1/LHFPL4/LRRC7/NLGN1/NPTX1/NSG1/RAP1A/RELN/SNAP25	20	3.27868852459016
GO:0050920	BP	GO:0050920	regulation of chemotaxis	37/666	230/18614	1.43169186336887e-14	3.95323904969549e-13	2.26649267784651e-13	CCL5/CCR2/CCR7/CSF1/CX3CR1/CXCL12/CXCL13/CXCL8/DUSP1/EDN2/EDN3/F2RL1/FEZF2/FGFR1/IL6R/LPAR1/MIF/NTRK3/P2RX4/PLXNA3/PTK2B/PTN/ROBO2/S1PR1/SCG2/SEMA3D/SEMA3E/SEMA3F/SEMA3G/SEMA6A/SEMA6D/SLIT2/TUBB2B/VEGFA/WNT3/WNT3A/WNT5A	37	1.60869565217391
GO:0099505	BP	GO:0099505	regulation of presynaptic membrane potential	15/666	30/18614	1.6271824342629e-14	4.46793389072746e-13	2.56158034491137e-13	GABRA3/GABRB1/GABRR1/GRIA1/GRIA3/GRIA4/GRIK2/GRIK3/GRIK5/GRIN2B/GRIN2D/GRIN3B/KCNA1/KCNA2/KCNJ9	15	5
GO:0045165	BP	GO:0045165	cell fate commitment	41/666	281/18614	1.6497021303765e-14	4.50460331711138e-13	2.58260386141397e-13	APC/ASCL1/ATOH1/BCL2/DKK1/DSCAML1/EDNRA/EPAS1/ERBB4/FEZF2/FGF13/FOXG1/GAP43/IL6R/MEF2C/NEUROD1/NKX2-1/NTF4/NTRK3/PDPN/POU4F1/PROX1/SHH/SOX1/SOX2/SOX5/SOX9/TBX20/TLX3/WNT1/WNT11/WNT16/WNT2/WNT2B/WNT3/WNT3A/WNT5A/WNT7A/WNT7B/WNT8B/WNT9A	41	1.45907473309609

	ONTOLOGY	ID	Description	GeneRatio	BgRatio	pvalue	p.adjust	qvalue	geneID	Count	richFactor
GO:0035094	BP	GO:0035094	response to nicotine	18/666	48/18614	1.97322293162598e-14	5.358226911017 5e-13	3.07200801858839e-13	ATP1A2/BCL2/CHRNA3/CHRNA4/CHRNA7/CHRNB2/CHRNB4/CNR1/DRD2/GRM2/HOMER1/KCNQ1/NKX6-1/PENK/SLC6A3/SLC7A11/TACR1/TH	18	3.75
GO:0051590	BP	GO:0051590	positive regulation of neurotransmitter transport	13/666	21/18614	2.19449174091482e-14	5.92633346516282e-13	3.39771798174029e-13	DRD2/DRD4/GPER1/NLGN1/PRKN/RAB3B/SLC17A8/SLC18A3/SLC4A8/SNCA/STX1A/STXBP1/TACR2	13	6.19047619047619
GO:0051963	BP	GO:0051963	regulation of synapse assembly	26/666	114/18614	2.69296753339391e-14	7.23275159925194e-13	4.14672078624992e-13	BDNF/CHRNB2/DKK1/EFNA5/EPHB2/EPHB3/FLRT2/GRID2/ICAM5/LHFPL4/MDGA1/MEF2C/NLGN1/NLGN3/NRXN1/NTN1/NTRK3/OXT/OXTR/PTK2B/ROBO2/SLITRK3/SNCA/WNT3A/WNT5A/WNT7A	26	2.28070175438596
GO:0031346	BP	GO:0031346	positive regulation of cell projection organization	46/666	353/18614	2.96246173800005e-14	7.91331491427732e-13	4.53690507587308e-13	AGT/APC/BDNF/CAMK2B/CCR7/CDH4/CNR1/CXCL12/EFNA5/EPHA3/EPHA4/EPHB2/F2RL1/FKBP1B/GPM6A/L1CAM/LIMK1/LRRC7/MAGI2/MAP1B/MAPT/NDNF/NEFL/NGF/NLGN1/NTN1/NTRK3/P2RX7/PLXNA1/PLXNA2/PLXNA3/PTK2B/PTN/RAP1A/RELN/RGS2/ROBO2/SCN1B/SLIT2/TENM1/TUBB2B/VEGFA/WNT1/WNT3/WNT3A/WNT5A	46	1.30311614730878
GO:0043524	BP	GO:0043524	negative regulation of neuron apoptotic process	30/666	155/18614	3.04234836344866e-14	8.08277957100009e-13	4.6340634816228e-13	AXL/BCL2/BCL2L1/BDNF/CCND1/CHL1/CNTFR/CRLF1/CX3CR1/FZD9/GDNF/GRIK2/IL6ST/KIF14/MAG/MEF2C/NDNF/NEFL/NGF/NTF4/PINK1/POU4F1/PRKCG/PRKN/PTK2B/SEMA3E/SLC1A1/SNCA/STXBP1/TYRO3	30	1.93548387096774
GO:0071277	BP	GO:0071277	cellular response to calcium ion	23/666	88/18614	3.63866966405351e-14	9.52097472513848e-13	5.45861740947598e-13	CAMK2D/CPNE1/CPNE5/CPNE9/FOSB/HPCA/MEF2C/NLGN1/PLCG2/RASAL1/RYR1/RYR3/SYT10/SYT11/SYT12/SYT2/SYT4/SYT5/SYT7/SYT8/SYT9/TUBA1A/WNT5A	23	2.61363636363636
GO:0099072	BP	GO:0099072	regulation of postsynaptic membrane neurotransmitter receptor levels	23/666	88/18614	3.63866966405351e-14	9.52097472513848e-13	5.45861740947598e-13	ARHGAP44/CACNG4/CACNG5/CACNG7/CACNG8/CALY/CPLX1/DRD4/ERBB4/FRRS1L/GPC6/GSG1L/HPCA/ITGB3/LGI1/LHFPL4/LPAR1/LRRC7/NPTX1/NSG1/RAP1A/SNAP25/SST	23	2.61363636363636
GO:0051961	BP	GO:0051961	negative regulation of nervous system development	30/666	156/18614	3.64179704644158e-14	9.52097472513848e-13	5.45861740947598e-13	ATOH1/DKK1/EPHB2/F2/FGF13/FSTL4/MAG/MAP2/NKX6-1/NTN1/NTRK3/PLXNA3/PROX1/PTN/ROBO2/RTN4R/SEMA3D/SEMA3E/SEMA3F/SEMA3G/SEMA6A/SEMA6D/SOX10/SYT4/TNR/VAX1/WNT3/WNT3A/WNT5A/WNT7A	30	1.92307692307692
GO:0001941	BP	GO:0001941	postsynaptic membrane organization	16/666	37/18614	3.86396362698228e-14	1.00483498553534e-12	5.76097500943e-13	CHRDL1/EPHB2/FRRS1L/FZD9/GDNF/GLRB/LHFPL4/NLGN1/NLGN3/NLGN4X/NLGN4Y/NRXN1/NRXN2/RELN/SHISA7/SLC7A11	16	4.32432432432432

	ONTOLOGY	ID	Description	GeneRatio	BgRatio	pvalue	p.adjust	qvalue	geneID	Count	richFactor
GO:1901888	BP	GO:1901888	regulation of cell junction assembly	35/666	214/18614	4.49462230876472e-14	1.16268782355677e-12	6.66598555155854e-13	AGT/BDNF/CAV1/CHRNB2/CLDN1/CLDN19/DKK1/EFNA5/EPHA3/EPHB2/EPHB3/FLRT2/GPM6B/GRID2/ICAM5/LHFPL4/MDGA1/MEF2C/NLGN1/NLGN3/NRXN1/NTN1/NTRK3/OXT/OXTR/PTK2B/RAP1A/ROBO2/SLITRK3/SNCA/VCL/VEGFA/WNT3A/WNT5A/WNT7A	35	1.63551401869159
GO:0015874	BP	GO:0015874	norepinephrine transport	13/666	22/18614	5.18962202053277e-14	1.33544461941982e-12	7.65644428160167e-13	ADORA3/ADRA2A/ADRA2C/AGT/OXT/OXTR/P2RY1/SLC22A2/SLC22A3/SLC6A2/SLC6A3/SNCA/STX1A	13	5.90909090909091
GO:0035590	BP	GO:0035590	purinergic nucleotide receptor signaling pathway	15/666	32/18614	5.55185591987323e-14	1.42121728365505e-12	8.14820082099816e-13	ADCY5/ADORA3/CNTN2/CTSG/GPR171/P2RX1/P2RX2/P2RX4/P2RX7/P2RY1/P2RY13/P2RY14/P2RY2/P2RY4/P2RY8	15	4.6875
GO:0090276	BP	GO:0090276	regulation of peptide hormone secretion	33/666	192/18614	5.61056114481727e-14	1.42880352470347e-12	8.19169467394373e-13	ADCY5/ADCYAP1/ADRA2A/CARTPT/CCL5/CNR1/DRD2/EFNA5/F2/F2RL1/F2RL2/FKBP1B/GCG/GHRHR/GNA11/GNAZ/GPER1/GRP/HTR2C/ITPR1/KISS1/LEP/NEUROD1/NKX6-1/PRKCB/PRKN/SNAP25/SSTR5/STX1A/SYT7/SYT9/UCN/UCN3	33	1.71875
GO:0050878	BP	GO:0050878	regulation of body fluid levels	46/666	361/18614	6.81659752941389e-14	1.72698849778708e-12	9.90126510376613e-13	ADRA2A/ADRA2C/APLN/AXL/CAV1/CCND1/COL3A1/CTSG/DRD2/EDNRB/EPHB2/ERBB4/F2/F2RL1/F2RL2/GHRHR/GNA13/GNAQ/IL6ST/ITGB3/KNG1/OXT/OXTR/P2RX1/P2RY1/P2RY2/PDPN/PIK3CG/PLCG2/PRKACB/PRKG1/PRL/SHH/SLC22A2/SLC29A1/SLC6A3/SLC7A11/STXBP1/TACR1/TPH1/TYRO3/UCN/VCL/VEGFA/VIP/WNT3A	46	1.27423822714681
GO:0002791	BP	GO:0002791	regulation of peptide secretion	33/666	195/18614	8.87173651709624e-14	2.23613256315528e-12	1.28203177631669e-12	ADCY5/ADCYAP1/ADRA2A/CARTPT/CCL5/CNR1/DRD2/EFNA5/F2/F2RL1/F2RL2/FKBP1B/GCG/GHRHR/GNA11/GNAZ/GPER1/GRP/HTR2C/ITPR1/KISS1/LEP/NEUROD1/NKX6-1/PRKCB/PRKN/SNAP25/SSTR5/STX1A/SYT7/SYT9/UCN/UCN3	33	1.69230769230769
GO:1901654	BP	GO:1901654	response to ketone	34/666	208/18614	1.05894303888103e-13	2.65546175311238e-12	1.52244388565226e-12	A2M/ADCY2/ADCY3/ADCY5/AXIN2/BCL2L1/CAV1/CCND1/CCR7/CLDN1/DDIT4/EFNA5/FOSB/GABRB1/GNAI1/GNG2/HPCA/MAOB/NEFL/NR3C1/NTRK3/OXT/OXTR/P2RY4/PTGDR/PTGER4/PTGFR/SLIT2/SLIT3/SOX10/TACR1/TH/TXNIP/UCN3	34	1.63461538461538
GO:0018958	BP	GO:0018958	phenol-containing compound metabolic process	25/666	111/18614	1.15313778929593e-13	2.8769909819236e-12	1.64945223721357e-12	ASIP/BCL2/CGA/CHRNB2/COMT/DRD2/DRD4/EDNRA/EPAS1/GRIN2A/MAOA/MAOB/MC1R/NPY/PAH/PRKN/SLC1A1/SLC6A3/SLC7A11/SNCA/SNCAIP/TH/TPH1/TRPC1/WNT5A	25	2.25225225225225

	ONTOLOGY	ID	Description	GeneRatio	BgRatio	pvalue	p.adjust	qvalue	geneID	Count	richFactor
GO:0090087	BP	GO:0090087	regulation of peptide transport	33/666	197/18614	1.19787565558183e-13	2.97351456928521e-12	1.70479166932088e-12	ADCY5/ADCYAP1/ADRA2A/CARTPT/CCL5/CNR1/DRD2/EFNA5/F2/F2RL1/F2RL2/FKBP1B/GCG/GHRHR/GNA11/GNAZ/GPER1/GRP/HTR2C/ITPR1/KISS1/LEP/NEUROD1/NKX6-1/PRKCB/PRKN/SNAP25/SSTR5/STX1A/SYT7/SYT9/UCN/UCN3	33	1.6751269035533
GO:0015695	BP	GO:0015695	organic cation transport	19/666	60/18614	1.32766602954947e-13	3.27913494232946e-12	1.88001161655854e-12	ADORA3/ADRA2A/ADRA2C/AGT/CHRNA3/OXT/OXTR/P2RY1/SLC17A8/SLC18A3/SLC22A2/SLC22A3/SLC44A4/SLC5A7/SLC6A2/SLC6A3/SNCA/STX1A/TACR2	19	3.16666666666667
GO:0034767	BP	GO:0034767	positive regulation of monoatomic ion transmembrane transport	31/666	175/18614	1.41129991839341e-13	3.46826954945181e-12	1.98844730607325e-12	ADCYAP1R1/ADRB2/ANK3/ATP2A1/BDKRB1/CACNG4/CAV1/CCR2/DRD4/EDN3/EDNRA/EPHB2/F2/GAL/GPER1/KCNA1/KCNQ1/NLGN3/NOS1/NPSR1/NTSR1/P2RX1/P2RX2/P2RX4/P2RX7/PINK1/PLCG1/PLCG2/RELN/SNCA/TRPC1	31	1.77142857142857
GO:0030307	BP	GO:0030307	positive regulation of cell growth	30/666	164/18614	1.45624082389753e-13	3.56090728828674e-12	2.04155888220669e-12	BCL2/BDNF/CACNG7/CDH4/CPNE5/CPNE9/CXCL12/EFNA5/F2/IGF1/L1CAM/LGI1/LIMK1/MAP1B/MAPT/NEDD4L/NGF/NTN1/PRKN/PRSS2/PTK2B/RASAL1/RIMS1/SLC44A4/SYT2/SYT4/UCN/VEGFA/WNT3/WNT3A	30	1.82926829268293
GO:0035176	BP	GO:0035176	social behavior	18/666	53/18614	1.47516336164597e-13	3.58932075370789e-12	2.05784904592301e-12	CHRNB2/CX3CR1/DRD4/GAD1/GNG8/GRP/GRPR/KCNQ1/NLGN3/NLGN4X/NLGN4Y/NRXN1/NRXN2/OXT/OXTR/SEPTIN5/TH/UCN	18	3.39622641509434
GO:0099068	BP	GO:0099068	postsynapse assembly	16/666	40/18614	1.70636437572773e-13	4.13141916586294e-12	2.36864787857046e-12	EPHB2/GAP43/GRID2/NLGN1/NLGN3/NLGN4X/NLGN4Y/NPTX1/NRXN1/NRXN2/NTRK3/PTK2B/SLITRK3/SPTBN2/WNT5A/WNT7A	16	4
GO:0060291	BP	GO:0060291	long-term synaptic potentiation	24/666	104/18614	2.02234605513948e-13	4.87246610833851e-12	2.79350897296615e-12	CALB1/CALB2/CHRNA7/CX3CR1/DRD2/EPHA4/EPHB2/GRIN2A/GRIN2B/GRIN2D/NSG1/PRKCG/PRNP/PTK2B/PTN/RELN/SHISA7/SLC18A3/SLC1A1/SNAP25/SNCA/SYT12/TNR/VAMP2	24	2.30769230769231
GO:0007271	BP	GO:0007271	synaptic transmission, cholinergic	14/666	29/18614	2.32464138067196e-13	5.57346945658668e-12	3.19541205445897e-12	CHRFAM7A/CHRNA1/CHRNA3/CHRNA4/CHRNA6/CHRNA7/CHRNB2/CHRNB4/CHRNG/LYNX1/SLC18A3/SLC5A7/TACR1/TACR2	14	4.82758620689655
GO:0031623	BP	GO:0031623	receptor internalization	26/666	125/18614	2.73404915465159e-13	6.5232289296663e-12	3.73993336075744e-12	APELA/APLN/ARRB1/CACNG4/CACNG5/CACNG7/CACNG8/CALY/CAV1/CNTN2/CXCL8/DKK1/DRD2/DRD4/GRIA1/GSG1L/HPCA/HTR2B/ITGB3/LPAR1/MAGI2/PLCG2/SNAP25/SNCA/VEGFA/WNT3A	26	2.08

	ONTOLOGY	ID	Description	GeneRatio	BgRatio	pvalue	p.adjust	qvalue	geneID	Count	richFactor
GO:0050905	BP	GO:0050905	neuromuscular process	29/666	157/18614	2.87492270778128e-13	6.82620536654347e-12	3.9136374720216e-12	ADCY5/ASCL1/ATP2A1/CHRNA1/CHRND/CNTNAP1/DRD2/EDNRA/FGF12/GLRB/GPR88/GRID2/GRIN2A/GRIN2D/GRIN3A/HOMER1/ITPR1/KCNA1/NEFL/NRXN1/PENK/POU4F1/PRKN/SLC6A3/SLURP1/TNR/TUBA1A/UCHL1/UCN	29	1.84713375796178
GO:0051703	BP	GO:0051703	biological process involved in intraspecies interaction between organisms	18/666	55/18614	3.07600098122658e-13	7.26853116477339e-12	4.16723412284593e-12	CHRNB2/CX3CR1/DRD4/GAD1/GNG8/GRP/GRPR/KCNQ1/NLGN3/NLGN4X/NLGN4Y/NRXN1/NRXN2/OXT/OXTR/SEPTIN5/TH/UCN	18	3.27272727272727
GO:0002027	BP	GO:0002027	regulation of heart rate	24/666	106/18614	3.16803643184883e-13	7.45019093901291e-12	4.27138430020615e-12	ADRA1A/ADRA1B/ADRB1/AGT/CACNA1C/CACNA1D/CAMK2D/CAV1/DRD2/EDN2/EDN3/EDNRA/EDNRB/EPAS1/FKBP1B/HCN1/KCNJ5/KCNQ1/MC3R/OXT/SCN1B/SCN2B/SLC1A1/SPX	24	2.26415094339623
GO:1905144	BP	GO:1905144	response to acetylcholine	15/666	36/18614	4.782562618762e-13	1.11934739386739e-11	6.41750382477487e-12	CHRM2/CHRM4/CHRM5/CHRNA3/CHRNA7/CHRNB2/CHRND/EDNRA/GNA11/GNAQ/ITPR1/LY6G6D/LY6H/OPRM1/PRKCB	15	4.16666666666667
GO:0036465	BP	GO:0036465	synaptic vesicle recycling	21/666	81/18614	5.63257055368084e-13	1.31054410240713e-11	7.51368327278231e-12	LRRK2/NLGN1/NLGN3/NLGN4X/NLGN4Y/PRKN/SH3GL2/SLC17A7/SLC2A4/SNCA/SNCG/STX1A/SYP/SYT11/SYT2/SYT5/SYT7/SYT8/VAMP2/WNT3A/WNT7A	21	2.59259259259259
GO:0050768	BP	GO:0050768	negative regulation of neurogenesis	28/666	150/18614	5.65280467365841e-13	1.31054410240713e-11	7.51368327278231e-12	ATOH1/EPHB2/F2/FGF13/FSTL4/MAG/MAP2/NKX6-1/NTN1/NTRK3/PLXNA3/PROX1/PTN/RTN4R/SEMA3D/SEMA3E/SEMA3F/SEMA3G/SEMA6A/SEMA6D/SOX10/SYT4/TNR/VAX1/WNT3/WNT3A/WNT5A/WNT7A	28	1.86666666666667
GO:0051932	BP	GO:0051932	synaptic transmission, GABAergic	18/666	57/18614	6.19581725506426e-13	1.42969210369206e-11	8.19678912370003e-12	ADRA1A/CNR1/CNR2/DRD2/GABRA3/GABRA4/GABRB3/GABRE/GABRG2/NLGN1/NPY5R/OXTR/PLCL1/SLC6A1/SLITRK3/STXBP1/SYN3/TACR1	18	3.15789473684211
GO:0095500	BP	GO:0095500	acetylcholine receptor signaling pathway	14/666	31/18614	7.4374683407369e-13	1.70818490162252e-11	9.79345929569733e-12	CHRM2/CHRM4/CHRM5/CHRNA3/CHRNA7/CHRNB2/CHRND/GNA11/GNAQ/ITPR1/LY6G6D/LY6H/OPRM1/PRKCB	14	4.51612903225806
GO:0034764	BP	GO:0034764	positive regulation of transmembrane transport	35/666	235/18614	7.73754065002584e-13	1.76883778115707e-11	1.01411977087487e-11	ADCYAP1R1/ADRB2/AGT/ANK3/ATP2A1/BDKRB1/CACNG4/CAV1/CCR2/DRD4/EDN3/EDNRA/EPHB2/F2/GAL/GPER1/IGF1/KCNA1/KCNQ1/NLGN3/NOS1/NPSR1/NTSR1/P2RX1/P2RX2/P2RX4/P2RX7/PINK1/PLCG1/PLCG2/RELN/SLC17A8/SLC1A2/SNCA/TRPC1	35	1.48936170212766
GO:0007413	BP	GO:0007413	axonal fasciculation	12/666	21/18614	8.73450380542741e-13	1.96927000934292e-11	1.12903267441474e-11	CNR1/CNTN2/CNTN4/CRTAC1/EPHA3/EPHA4/EPHB2/EPHB3/FEZF2/NCAM2/NDN/NRCAM	12	5.71428571428571

	ONTOLOGY	ID	Description	GeneRatio	BgRatio	pvalue	p.adjust	qvalue	geneID	Count	richFactor
GO:0048791	BP	GO:0048791	calcium ion-regulated exocytosis of neurotransmitter	12/666	21/18614	8.73450380542741e-13	1.96927000934292e-11	1.12903267441474e-11	RIMS1/RIMS3/RIMS4/SNAP25/STXBP1/SYT11/SYT2/SYT4/SYT5/SYT7/SYT8/UNC13C	12	5.71428571428571
GO:0106030	BP	GO:0106030	neuron projection fasciculation	12/666	21/18614	8.73450380542741e-13	1.96927000934292e-11	1.12903267441474e-11	CNR1/CNTN2/CNTN4/CRTAC1/EPHA3/EPHA4/EPHB2/EPHB3/FEZF2/NCAM2/NDN/NRCAM	12	5.71428571428571
GO:0042596	BP	GO:0042596	fear response	16/666	44/18614	9.8991237510165e-13	2.22165265918932e-11	1.27373007841726e-11	ADRA2A/ADRB1/ASIC1/ATP1A2/BCL2/DRD4/EPHB2/GNG7/GRIK2/GRP/GRPR/HTR2C/MEF2C/PENK/SLC1A1/UCN	16	3.63636363636364
GO:0051899	BP	GO:0051899	membrane depolarization	20/666	75/18614	1.13180579281546e-12	2.52855703258544e-11	1.44968617577368e-11	ANK3/ATP1A2/BCL2/CACNA1C/CACNA1D/CAMK2D/CAV1/CHRNA4/CHRNA6/CHRNB2/FZD9/HCN1/LRRK2/NEDD4L/NTSR1/P2RX4/P2RX7/PHOX2B/SCN1B/SCN2B	20	2.66666666666667
GO:0048846	BP	GO:0048846	axon extension involved in axon guidance	15/666	38/18614	1.24328558471665e-12	2.75258948147853e-11	1.57812968719131e-11	CXCL12/NRP2/PLXNA3/SEMA3D/SEMA3E/SEMA3F/SEMA3G/SEMA6A/SEMA6D/SLIT2/SLIT3/VEGFA/WNT3/WNT3A/WNT5A	15	3.94736842105263
GO:1902284	BP	GO:1902284	neuron projection extension involved in neuron projection guidance	15/666	38/18614	1.24328558471665e-12	2.75258948147853e-11	1.57812968719131e-11	CXCL12/NRP2/PLXNA3/SEMA3D/SEMA3E/SEMA3F/SEMA3G/SEMA6A/SEMA6D/SLIT2/SLIT3/VEGFA/WNT3/WNT3A/WNT5A	15	3.94736842105263
GO:0098815	BP	GO:0098815	modulation of excitatory postsynaptic potential	16/666	45/18614	1.48564708339214e-12	3.27441946855264e-11	1.87730811528948e-11	ADRB2/CHRNA7/GRIN2A/GRIN2B/GRIN2D/LRRK2/NLGN1/NLGN3/NLGN4X/NRXN1/PTK2B/RELN/RIMS1/STX1A/TMEM108/WNT7A	16	3.55555555555556
GO:0019228	BP	GO:0019228	neuronal action potential	13/666	27/18614	1.77354853025759e-12	3.8742182338738e-11	2.22118803064307e-11	ANK3/CHRNA1/CHRNB4/FGF12/GPER1/GPR88/GRIK2/HCN1/KCNA1/KCNA2/KCNMB2/P2RX1/P2RX4	13	4.81481481481481
GO:0042133	BP	GO:0042133	neurotransmitter metabolic process	13/666	27/18614	1.77354853025759e-12	3.8742182338738e-11	2.22118803064307e-11	BCHE/CHAT/COMT/GAD1/GCHFR/MAOA/MAOB/NOS1/PAH/PRIMA1/SLC44A4/SLC5A7/TH	13	4.81481481481481
GO:0051048	BP	GO:0051048	negative regulation of secretion	30/666	180/18614	1.80970421761946e-12	3.93570629628303e-11	2.25644070357117e-11	ADRA2A/ADRA2C/ASIC1/CARTPT/CCR2/CNR1/DRD2/DRD4/F2RL1/FKBP1B/GNAZ/INHBA/LEP/MAOB/NMB/NPSR1/NPY5R/OPRM1/OXT/OXTR/P2RY1/PRKN/PTGER3/RAP1A/SNCA/SPX/SYT11/SYT4/TACR2/UCN	30	1.66666666666667
GO:0098926	BP	GO:0098926	postsynaptic signal transduction	15/666	39/18614	1.9541777084312e-12	4.2311821308103e-11	2.42584452838875e-11	CHRM2/CHRM4/CHRM5/CHRNA3/CHRNA7/CHRNB2/CHRND/GNA11/GNAQ/ITPR1/LY6G6D/LY6H/OPRM1/PRKCB/WNT3A	15	3.84615384615385
GO:1905145	BP	GO:1905145	cellular response to acetylcholine	14/666	33/18614	2.14894134502951e-12	4.63247662755264e-11	2.65591688857063e-11	CHRM2/CHRM4/CHRM5/CHRNA3/CHRNA7/CHRNB2/CHRND/GNA11/GNAQ/ITPR1/LY6G6D/LY6H/OPRM1/PRKCB	14	4.24242424242424
GO:0048640	BP	GO:0048640	negative regulation of developmental growth	24/666	116/18614	2.55897616596881e-12	5.49230037368415e-11	3.14887575099908e-11	ADRB1/ADRB2/ADRB3/CGA/FGF13/FSTL4/GDF15/MAG/MAP2/NTN1/PLXNA3/RGS2/RTN4R/SEMA3D/SEMA3E/SEMA3F/SEMA3G/SEMA6A/SEMA6D/TNR/TP73/WNT3/WNT3A/WNT5A	24	2.06896551724138

	ONTOLOGY	ID	Description	GeneRatio	BgRatio	pvalue	p.adjust	qvalue	geneID	Count	richFactor
GO:0048488	BP	GO:0048488	synaptic vesicle endocytosis	19/666	70/18614	2.9471320464027e-12	6.29789304698664e-11	3.61074255753777e-11	LRRK2/NLGN1/NLGN3/NLGN4X/NLGN4Y/PRKN/SH3GL2/SLC17A7/SLC2A4/SNCA/SNCG/STX1A/SYP/SYT11/SYT2/SYT5/SYT7/SYT8/VAMP2	19	2.71428571428571
GO:0042220	BP	GO:0042220	response to cocaine	16/666	47/18614	3.23073043708094e-12	6.87124787026519e-11	3.93946148711247e-11	CACNG4/CHRNB2/CNR1/DRD2/DRD4/DRD5/GRM2/HOMER1/OPRM1/OXT/OXTR/PTK2B/SLC1A1/SLC1A2/SLC6A1/SLC6A3	16	3.40425531914894
GO:0021543	BP	GO:0021543	pallium development	30/666	184/18614	3.24339675666637e-12	6.87124787026519e-11	3.93946148711247e-11	ADGRG1/ASCL1/ASPM/ATOH1/CDK5R2/COL3A1/EMX1/FEZF2/FGF13/FOXG1/KCNA1/KCNA2/KIF14/KIRREL3/MDGA1/NEFL/NEUROD1/NKX2-1/NPY/PLXNA3/PROX1/RELN/SLIT2/TH/TMEM108/TUBA1A/TUBB2A/TUBB2B/WDR62/WNT3A	30	1.6304347826087
GO:0009306	BP	GO:0009306	protein secretion	44/666	373/18614	3.55706714503222e-12	7.50342704628042e-11	4.30190591698724e-11	ADCY5/ADCYAP1/ADRA2A/CARTPT/CCL5/CNR1/CPLX1/DRD2/DRD4/EDNRB/EFNA5/F2/F2RL1/F2RL2/FKBP1B/GAL/GCG/GNA11/GNAZ/GPER1/IGF1/IL1A/ITPR1/LEP/NEUROD1/NKX6-1/OPRM1/P2RX7/PPY/PRKCB/PRKN/RAB3B/SCG2/SNAP25/SNCG/SSTR5/STX1A/SYT4/SYT7/SYT9/TACR2/UCN3/VGF/VIP	44	1.17962466487936
GO:0061351	BP	GO:0061351	neural precursor cell proliferation	27/666	150/18614	3.61859332185713e-12	7.60059238330247e-11	4.357613280527e-11	ADGRG1/ASCL1/ASPM/CX3CR1/DRD2/EMX1/FGF13/FOXG1/FZD3/FZD9/KCNA1/KIF14/LHX1/LRRK2/PROX1/PTN/SHH/SOX10/SOX5/VAX1/VEGFA/WDR62/WNT1/WNT2/WNT3A/WNT5A/WNT7A	27	1.8
GO:0030073	BP	GO:0030073	insulin secretion	31/666	197/18614	3.68687009067334e-12	7.71104957262105e-11	4.42094120167191e-11	ADCY5/ADCYAP1/ADRA2A/CARTPT/CCL5/CNR1/CPLX1/DRD2/EFNA5/F2/F2RL1/F2RL2/FKBP1B/GAL/GCG/GNA11/GNAZ/GPER1/ITPR1/LEP/NEUROD1/NKX6-1/PRKCB/PRKN/SNAP25/SSTR5/STX1A/SYT7/SYT9/UCN3/VGF	31	1.57360406091371
GO:0035592	BP	GO:0035592	establishment of protein localization to extracellular region	44/666	374/18614	3.89798298619e-12	8.11804507505247e-11	4.65428209368003e-11	ADCY5/ADCYAP1/ADRA2A/CARTPT/CCL5/CNR1/CPLX1/DRD2/DRD4/EDNRB/EFNA5/F2/F2RL1/F2RL2/FKBP1B/GAL/GCG/GNA11/GNAZ/GPER1/IGF1/IL1A/ITPR1/LEP/NEUROD1/NKX6-1/OPRM1/P2RX7/PPY/PRKCB/PRKN/RAB3B/SCG2/SNAP25/SNCG/SSTR5/STX1A/SYT4/SYT7/SYT9/TACR2/UCN3/VGF/VIP	44	1.17647058823529
GO:0006584	BP	GO:0006584	catecholamine metabolic process	17/666	55/18614	4.04708900447951e-12	8.32277927071832e-11	4.77166142479262e-11	CHRNB2/COMT/DRD2/DRD4/EDNRA/EPAS1/GRIN2A/MAOA/MAOB/NPY/PAH/PRKN/SLC1A1/SLC6A3/SNCA/SNCAIP/TH	17	3.09090909090909
GO:0009712	BP	GO:0009712	catechol-containing compound metabolic process	17/666	55/18614	4.04708900447951e-12	8.32277927071832e-11	4.77166142479262e-11	CHRNB2/COMT/DRD2/DRD4/EDNRA/EPAS1/GRIN2A/MAOA/MAOB/NPY/PAH/PRKN/SLC1A1/SLC6A3/SNCA/SNCAIP/TH	17	3.09090909090909

	ONTOLOGY	ID	Description	GeneRatio	BgRatio	pvalue	p.adjust	qvalue	geneID	Count	richFactor
GO:0050805	BP	GO:0050805	negative regulation of synaptic transmission	17/666	55/18614	4.04708900447951e-12	8.32277927071832e-11	4.77166142479262e-11	BCHE/CNR2/DRD2/DRD5/GRIA1/GRID2/GRIK2/GRIK3/GRM2/HCN1/MAPT/NPY5R/PTK2B/SLC6A1/SORCS3/STXBP1/TNR	17	3.09090909090909
GO:0032890	BP	GO:0032890	regulation of organic acid transport	20/666	80/18614	4.21183479708466e-12	8.625486678196 3e-11	4.94521129464721e-11	AGT/ATP1A2/AVPR1B/GRM2/IL1A/LEP/MAP2K6/MIF/NPY5R/NTSR1/OXT/P2RX4/P2RX7/RGS2/SLC17A8/SLC38A3/SLC6A1/SNCA/STXBP1/SYT4	20	2.5
GO:0003044	BP	GO:0003044	regulation of systemic arterial blood pressure mediated by a chemical signal	16/666	48/18614	4.68705380250514e-12	9.51936753690609e-11	5.45769596751033e-11	ADRA1A/ADRA1B/ADRB1/ADRB2/ADRB3/AGT/AGTR1/AVPR1B/CTSG/DRD5/EDN2/EDN3/EDNRB/F2RL1/OXTR/TACR1	16	3.33333333333333
GO:0042311	BP	GO:0042311	vasodilation	16/666	48/18614	4.68705380250514e-12	9.51936753690609e-11	5.45769596751033e-11	ADRA2A/ADRB1/ADRB2/ADRB3/AGT/BDKRB2/CALCA/EDNRB/F2RL1/GPER1/GUCY1A1/KNG1/NOS1/PRKG1/RGS2/UCN	16	3.33333333333333
GO:0032147	BP	GO:0032147	activation of protein kinase activity	23/666	109/18614	4.75468976582869e-12	9.61699596668642e-11	5.51366883392826e-11	ADRA2A/ADRA2C/ADRB2/AGT/CALCA/CCL5/CHRNA3/DRD2/GHR/IGF1/IL6R/IRAK1/ITGB3/KIF14/LEP/NTRK3/PINK1/PRNP/PTK2B/SLC1A1/UCN/VEGFA/WNT5A	23	2.11009174311927
GO:0021700	BP	GO:0021700	developmental maturation	40/666	319/18614	5.10970818194378e-12	1.02550242995684e-10	5.87946673447441e-11	ASCL1/ATP6V0A4/ATP6V1F/ATP6V1G2/AURKA/AXL/BCL2/CAMK2B/CDK5R2/CHRDL1/CNTN2/CNTNAP1/CX3CR1/EDNRA/EDNRB/EPAS1/GAL/GHRHR/IGF1/KIF14/LEP/LRRK2/MAP1B/NEFL/NKX6-1/NLGN1/NRCAM/NRXN1/PTH1R/PTK2B/RELN/RYR1/S1PR1/SOX10/STXBP1/SYP/UNC13C/VEGFA/WNT1/WNT5A	40	1.25391849529781
GO:0140238	BP	GO:0140238	presynaptic endocytosis	19/666	72/18614	5.11186358778079e-12	1.02550242995684e-10	5.87946673447441e-11	LRRK2/NLGN1/NLGN3/NLGN4X/NLGN4Y/PRKN/SH3GL2/SLC17A7/SLC2A4/SNCA/SNCG/STX1A/SYP/SYT11/SYT2/SYT5/SYT7/SYT8/VAMP2	19	2.63888888888889
GO:0021885	BP	GO:0021885	forebrain cell migration	18/666	64/18614	5.68794214808842e-12	1.13643234381523e-10	6.51545619616291e-11	ADGRG1/AXL/CDK5R2/CNTN2/COL3A1/CXCL12/DRD2/FEZF2/FGF13/FOXG1/LRRK2/MDGA1/NKX2-1/NRG3/RELN/SLIT2/TNR/TYRO3	18	2.8125
GO:0051580	BP	GO:0051580	regulation of neurotransmitter uptake	11/666	19/18614	6.602624280935 5e-12	1.31384203808899e-10	7.53259117837815e-11	ATP1A2/DRD2/DRD4/GDNF/GPM6B/ITGB3/NOS1/PRKN/RAB3B/SLC17A8/SNCA	11	5.78947368421053
GO:0007405	BP	GO:0007405	neuroblast proliferation	19/666	73/18614	6.68256958219827e-12	1.32438828614938e-10	7.59305550574906e-11	ASCL1/ASPM/CX3CR1/DRD2/FGF13/FOXG1/FZD3/FZD9/KCNA1/LRRK2/PROX1/PTN/SHH/SOX10/SOX5/VAX1/VEGFA/WDR62/WNT3A	19	2.6027397260274

	ONTOLOGY	ID	Description	GeneRatio	BgRatio	pvalue	p.adjust	qvalue	geneID	Count	richFactor
GO:0018105	BP	GO:0018105	peptidyl-serine phosphorylation	39/666	309/18614	7.73806861806377e-12	1.52741394609572e-10	8.75705334625099e-11	AKT3/AURKA/BCL2/BDKRB2/BDNF/CAMK2A/CAMK2B/CAMK2D/CAMK4/CAV1/CDK1/CSNK1A1L/CSNK1E/DCLK1/DDIT4/DKK1/GCG/HSP90AB1/LRRK2/MIF/NGF/NOS1/NTF4/NTRK3/OPRD1/PINK1/PRKACB/PRKCB/PRKCG/PRKG2/RIPK2/SLC1A1/SNCA/TENM1/TTK/UCN/VEGFA/WNT3A/WNT5A	39	1.2621359223301
GO:0071692	BP	GO:0071692	protein localization to extracellular region	44/666	382/18614	8.00433481245004e-12	1.57365222412768e-10	9.02214917597000e-11	ADCY5/ADCYAP1/ADRA2A/CARTPT/CCL5/CNR1/CPLX1/DRD2/DRD4/EDNRB/EFNA5/F2/F2RL1/F2RL2/FKBP1B/GAL/GCG/GNA11/GNAZ/GPER1/IGF1/IL1A/ITPR1/LEP/NEUROD1/NKX6-1/OPRM1/P2RX7/PPY/PRKCB/PRKN/RAB3B/SCG2/SNAP25/SNCG/SSTR5/STX1A/SYT4/SYT7/SYT9/TACR2/UCN3/VGF/VIP	44	1.15183246073298
GO:0031667	BP	GO:0031667	response to nutrient levels	50/666	477/18614	9.89473713405200e-12	1.93755510015401e-10	1.1108497088638e-10	A2M/ADRB1/ADRB2/ADRB3/ASCL1/BCHE/BCL2/BECN2/CARTPT/CCND1/CNR1/CNTN2/COMT/FAS/FKBP1B/GABARAP/GCGR/GDF15/GHR/HCRT/HTR2C/HTR4/IL1A/LEP/LRRK2/MAP1B/MAP1LC3C/NMUR2/NPY/OPRM1/OXT/PENK/PRKCG/PRL/PROX1/SCTR/SLC22A3/SLC38A3/SPX/SST/SSTR1/SSTR2/SSTR3/TH/UCN/UCN2/UCN3/VGF/WNT11/WNT2B	50	1.0482180293501
GO:0050771	BP	GO:0050771	negative regulation of axonogenesis	18/666	66/18614	1.01154758806913e-11	1.97291920450786e-10	1.13112485098625e-10	EPHB2/FGF13/FSTL4/MAG/MAP2/NTN1/PLXNA3/RTN4R/SEMA3D/SEMA3E/SEMA3F/SEMA3G/SEMA6A/SEMA6D/TNR/WNT3/WNT3A/WNT5A	18	2.72727272727273
GO:0001755	BP	GO:0001755	neural crest cell migration	17/666	58/18614	1.05966055407821e-11	2.05858957442466e-10	1.18024185698663e-10	EDN3/EDNRA/EDNRB/ERBB4/GDNF/HTR2B/NRP2/PHOX2B/SEMA3D/SEMA3E/SEMA3F/SEMA3G/SEMA6A/SEMA6D/SHH/SOX10/TWIST1	17	2.93103448275862
GO:0018209	BP	GO:0018209	peptidyl-serine modification	40/666	327/18614	1.12189965040959e-11	2.17091999282012e-10	1.24464374809219e-10	AKT3/AURKA/BCL2/BDKRB2/BDNF/CAMK2A/CAMK2B/CAMK2D/CAMK4/CAV1/CDK1/CSNK1A1L/CSNK1E/DCLK1/DDIT4/DKK1/GCG/HSP90AB1/LRRK2/MIF/NDNF/NGF/NOS1/NTF4/NTRK3/OPRD1/PINK1/PRKACB/PRKCB/PRKCG/PRKG2/RIPK2/SLC1A1/SNCA/TENM1/TTK/UCN/VEGFA/WNT3A/WNT5A	40	1.22324159021407
GO:0003073	BP	GO:0003073	regulation of systemic arterial blood pressure	21/666	94/18614	1.27042958489713e-11	2.44869074892917e-10	1.40389680031769e-10	ADRA1A/ADRA1B/ADRB1/ADRB2/ADRB3/AGT/AGTR1/AVPR1B/CALCA/CTSG/DRD2/DRD5/EDN2/EDN3/EDNRB/F2RL1/NTSR1/OXTR/P2RX2/SPX/TACR1	21	2.23404255319149
GO:0043113	BP	GO:0043113	receptor clustering	16/666	51/18614	1.34929349082532e-11	2.59053808883064e-10	1.48522149462968e-10	CHRDL1/FRRS1L/FZD9/GLRB/GRIK5/ITGA4/LHFPL4/MAGI2/NLGN1/NRXN1/NRXN2/PTN/RELN/SHISA7/SLC7A11/SLITRK3	16	3.13725490196078

	ONTOLOGY	ID	Description	GeneRatio	BgRatio	pvalue	p.adjust	qvalue	geneID	Count	richFactor
GO:2000311	BP	GO:2000311	regulation of AMPA receptor activity	12/666	25/18614	1.35458148254441e-11	2.59057120105283e-10	1.48524047871038e-10	ADRB2/CACNG4/CACNG5/CACNG7/CACNG8/CNIH3/GSG1L/MEF2C/NLGN1/NLGN3/RELN/SHISA7	12	4.8
GO:0050772	BP	GO:0050772	positive regulation of axonogenesis	19/666	76/18614	1.45189238636581e-11	2.76591127092557e-10	1.58576740852765e-10	BDNF/CDH4/CXCL12/EFNA5/L1CAM/LIMK1/MAP1B/MAPT/NEFL/NGF/NTN1/PLXNA1/PLXNA2/PLXNA3/ROBO2/SLIT2/VEGFA/WNT3/WNT3A	19	2.5
GO:0045762	BP	GO:0045762	positive regulation of adenylate cyclase activity	13/666	31/18614	1.5976930809573e-11	3.02025442034812e-10	1.73158881689177e-10	ADCYAP1/ADORA3/ADRB1/ADRB2/ADRB3/CACNA1C/CACNA1D/CALCA/DRD5/EDNRA/GHRHR/HPCA/VIPR2	13	4.19354838709677
GO:2000463	BP	GO:2000463	positive regulation of excitatory postsynaptic potential	13/666	31/18614	1.5976930809573e-11	3.02025442034812e-10	1.73158881689177e-10	ADRB2/CHRNA7/GRIN2A/GRIN2B/GRIN2D/NLGN1/NLGN3/NRXN1/PTK2B/RELN/RIMS1/STX1A/WNT7A	13	4.19354838709677
GO:0008277	BP	GO:0008277	regulation of G protein-coupled receptor signaling pathway	25/666	137/18614	1.67521316764152e-11	3.15466387699542e-10	1.80864918321288e-10	ADCYAP1/ADRB2/APELA/APLN/ARRB1/BECN2/CAMK2A/CCL5/CNTN2/CXCL8/DRD2/F2/GNG4/GNG7/GPER1/GRP/HTR2B/ITGB3/MGLL/OPRM1/PTGDR2/RGS2/SNCA/SYP/TMOD2	25	1.82481751824818
GO:0090497	BP	GO:0090497	mesenchymal cell migration	17/666	60/18614	1.94275733812428e-11	3.64452378506902e-10	2.08949835040526e-10	EDN3/EDNRA/EDNRB/ERBB4/GDNF/HTR2B/NRP2/PHOX2B/SEMA3D/SEMA3E/SEMA3F/SEMA3G/SEMA6A/SEMA6D/SHH/SOX10/TWIST1	17	2.83333333333333
GO:0007158	BP	GO:0007158	neuron cell-cell adhesion	10/666	16/18614	2.12122252458418e-11	3.96418582065827e-10	2.27276874056909e-10	ASTN1/CNTN4/NCAM2/NLGN1/NLGN3/NLGN4X/NLGN4Y/NRXN1/NRXN2/TNR	10	6.25
GO:0001662	BP	GO:0001662	behavioral fear response	14/666	38/18614	2.14971691585228e-11	4.00221918235376e-10	2.29457423593961e-10	ASIC1/ATP1A2/BCL2/DRD4/EPHB2/GNG7/GRIK2/GRP/GRPR/HTR2C/MEF2C/PENK/SLC1A1/UCN	14	3.68421052631579
GO:0043406	BP	GO:0043406	positive regulation of MAP kinase activity	23/666	117/18614	2.22519164200228e-11	4.12710072469479e-10	2.36617200621255e-10	ADRA2A/DKK1/DRD4/EDN3/EPHA4/FGFR1/FZD10/GHR/HTR2B/IRAK1/LRRK2/MAP2K6/MEF2C/MIF/NOX4/NTRK3/P2RX7/PIK3CG/PIK3R5/PTK2B/TENM1/VEGFA/WNT5A	23	1.96581196581197
GO:1903169	BP	GO:1903169	regulation of calcium ion transmembrane transport	29/666	186/18614	2.32339017082003e-11	4.29303108630843e-10	2.46130411052046e-10	ADCYAP1R1/ADRA2A/ATP1A2/ATP2A1/BCL2/BDKRB1/CACNA1C/CAMK2D/CAV1/DRD4/F2/FKBP1B/GPER1/HPCA/ITGB3/NOS1/NPSR1/NTSR1/P2RX1/P2RX2/P2RX4/P2RX7/PIK3CG/PLCG1/PLCG2/PRNP/PTK2B/SNCA/TRPC1	29	1.55913978494624
GO:0001975	BP	GO:0001975	response to amphetamine	13/666	32/18614	2.60333855003319e-11	4.77715315567863e-10	2.73886362858097e-10	ASIC1/CNR2/DRD2/DRD4/DRD5/EDNRA/GRIN2A/OXT/OXTR/RGS2/SLC18A2/SLC1A1/TH	13	4.0625
GO:0019229	BP	GO:0019229	regulation of vasoconstriction	17/666	61/18614	2.6048363086915e-11	4.77715315567863e-10	2.73886362858097e-10	ADRA1A/ADRA1D/ADRA2A/ADRA2C/AGT/AGTR1/ATP1A2/AVPR1B/BDKRB2/CAV1/EDN2/EDN3/KCNMB2/LEP/OXTR/P2RX1/TACR1	17	2.78688524590164

	ONTOLOGY	ID	Description	GeneRatio	BgRatio	pvalue	p.adjust	qvalue	geneID	Count	richFactor
GO:0019722	BP	GO:0019722	calcium-mediated signaling	29/666	187/18614	2.65864128706982e-11	4.85770331819635e-10	2.78504508921382e-10	AGTR1/ATP1A2/CACNA1C/CAMK2D/CXCL8/EDN2/EDNRB/FKBP1B/GPR143/GRIN2A/GRIN2B/GRIN2D/HPCA/HTR2B/IGF1/ITPR1/LRRK2/MAPT/NMUR2/P2RX2/P2RX4/P2RX7/PLA2G4B/PLCG1/PLCG2/PRNP/PTGDR2/PTGFR/PTK2B	29	1.55080213903743
GO:0050796	BP	GO:0050796	regulation of insulin secretion	27/666	163/18614	2.71870612896693e-11	4.94905208291572e-10	2.83741766364307e-10	ADCY5/ADRA2A/CARTPT/CCL5/CNR1/DRD2/EFNA5/F2/F2RL1/F2RL2/FKBP1B/GCG/GNA11/GNAZ/GPER1/ITPR1/LEP/NEUROD1/NKX6-1/PRKCB/PRKN/SNAP25/SSTR5/STX1A/SYT7/SYT9/UCN3	27	1.65644171779141
GO:0051928	BP	GO:0051928	positive regulation of calcium ion transport	24/666	129/18614	2.78522875374934e-11	5.04059448786581e-10	2.88990125695064e-10	ADCYAP1R1/ATP2A1/BDKRB1/CACNA1D/CAMK2A/CAV1/CCL5/CXCL12/F2/GCG/GPER1/HCRT/HOMER1/NPSR1/NTSR1/P2RX1/P2RX2/P2RX4/P2RX7/PLCG1/PLCG2/SNCA/TRPC1/UCN	24	1.86046511627907
GO:0021675	BP	GO:0021675	nerve development	20/666	88/18614	2.79976051920115e-11	5.04059448786581e-10	2.88990125695064e-10	BDNF/CHRNB2/EDNRA/EPHB2/ITGA4/KCNA1/KCNA2/LPAR1/NGF/NGFR/NPTX1/NRP2/NTF4/PHOX2B/PLXNA1/PLXNA3/POU4F1/PRKCG/SEMA3F/SULF1	20	2.27272727272727
GO:0048864	BP	GO:0048864	stem cell development	20/666	88/18614	2.79976051920115e-11	5.04059448786581e-10	2.88990125695064e-10	BCL2/EDN3/EDNRA/EDNRB/ERBB4/GDNF/HTR2B/NRP2/PHOX2B/SEMA3D/SEMA3E/SEMA3F/SEMA3G/SEMA6A/SEMA6D/SHH/SOX10/SOX9/TWIST1/WNT7A	20	2.27272727272727
GO:0006837	BP	GO:0006837	serotonin transport	11/666	21/18614	2.88584699283048e-11	5.17661969699336e-10	2.96788797533891e-10	CNR1/GPM6B/ITGB3/MAOB/NOS1/P2RX1/SLC18A2/SLC18A3/SLC22A2/SLC22A3/SNCA	11	5.23809523809524
GO:0030517	BP	GO:0030517	negative regulation of axon extension	15/666	46/18614	3.15101627221604e-11	5.63172544652431e-10	3.22881169788415e-10	MAG/MAP2/NTN1/PLXNA3/RTN4R/SEMA3D/SEMA3E/SEMA3F/SEMA3G/SEMA6A/SEMA6D/TNR/WNT3/WNT3A/WNT5A	15	3.26086956521739
GO:0002209	BP	GO:0002209	behavioral defense response	14/666	39/18614	3.24412443705743e-11	5.73556532666809e-10	3.28834574706655e-10	ASIC1/ATP1A2/BCL2/DRD4/EPHB2/GNG7/GRIK2/GRP/GRPR/HTR2C/MEF2C/PENK/SLC1A1/UCN	14	3.58974358974359
GO:0017158	BP	GO:0017158	regulation of calcium ion-dependent exocytosis	14/666	39/18614	3.24412443705743e-11	5.73556532666809e-10	3.28834574706655e-10	ADRA2A/CDK5R2/RPH3A/STX1A/STXBP1/SYT10/SYT11/SYT12/SYT2/SYT4/SYT5/SYT7/SYT8/SYT9	14	3.58974358974359
GO:0031281	BP	GO:0031281	positive regulation of cyclase activity	14/666	39/18614	3.24412443705743e-11	5.73556532666809e-10	3.28834574706655e-10	ADCYAP1/ADORA3/ADRB1/ADRB2/ADRB3/CACNA1C/CACNA1D/CALCA/DRD5/EDNRA/GHRHR/HPCA/NOS1/VIPR2	14	3.58974358974359
GO:0051209	BP	GO:0051209	release of sequestered calcium ion into cytosol	24/666	130/18614	3.30274146799862e-11	5.81827036387571e-10	3.33576265226648e-10	ATP1A2/BDKRB1/CACNA1C/CAMK2D/CCR7/DRD2/F2/FKBP1B/GPER1/HTR2B/HTR2C/ITGB3/ITPR1/NOS1/NPSR1/NTSR1/P2RX7/PLCB4/PLCG1/PLCG2/PTK2B/RYR1/SNCA/TRPC1	24	1.84615384615385

	ONTOLOGY	ID	Description	GeneRatio	BgRatio	pvalue	p.adjust	qvalue	geneID	Count	richFactor
GO:0030100	BP	GO:0030100	regulation of endocytosis	31/666	214/18614	3.33062061794485e-11	5.84642869185676e-10	3.35190653918735e-10	APELA/APLN/APOC1/ARRB1/AXL/CALY/CAV1/CLU/DKK1/DRD2/DRD4/EPHA3/F2RL1/GSG1L/HPCA/ITGB3/LPAR1/LRRK2/MAGI2/NEDD4L/NLGN1/PLCG2/PRKN/SLC17A7/SNCA/SYT11/SYT4/SYT7/VEGFA/WNT3A/WNT5A	31	1.44859813084112
GO:2000177	BP	GO:2000177	regulation of neural precursor cell proliferation	20/666	89/18614	3.49208159351114e-11	6.10803595448657e-10	3.50189264874671e-10	ADGRG1/ASCL1/ASPM/CX3CR1/DRD2/EMX1/FOXG1/FZD3/FZD9/LHX1/LRRK2/PROX1/PTN/SHH/SOX10/VAX1/VEGFA/WDR62/WNT3A/WNT5A	20	2.24719101123596
GO:0048762	BP	GO:0048762	mesenchymal cell differentiation	34/666	255/18614	3.83994554741648e-11	6.69267105161419e-10	3.83707884674652e-10	AXIN2/BCL2/EDN3/EDNRA/EDNRB/EPHA3/EPHA4/ERBB4/FGFR1/GDNF/HMGA2/HTR2B/IGF1/MEF2C/NKX2-1/NRP2/OLFM1/PDPN/PHOX2B/SEMA3D/SEMA3E/SEMA3F/SEMA3G/SEMA6A/SEMA6D/SHH/SOX10/SOX9/TBX20/TWIST1/WNT11/WNT16/WNT2/WNT5A	34	1.33333333333333
GO:0051283	BP	GO:0051283	negative regulation of sequestering of calcium ion	24/666	131/18614	3.9096568907803e-11	6.79009315130218e-10	3.89293341886512e-10	ATP1A2/BDKRB1/CACNA1C/CAMK2D/CCR7/DRD2/F2/FKBP1B/GPER1/HTR2B/HTR2C/ITGB3/ITPR1/NOS1/NPSR1/NTSR1/P2RX7/PLCB4/PLCG1/PLCG2/PTK2B/RYR1/SNCA/TRPC1	24	1.83206106870229
GO:0010720	BP	GO:0010720	positive regulation of cell development	46/666	432/18614	4.03693136571639e-11	6.98644988116058e-10	4.00550973536796e-10	ASCL1/ASPM/AURKA/AXL/BCL2/BDNF/CAMK2B/CDH4/CSF1/CX3CR1/CXCL12/DRD2/EFNA5/EPHA4/EPHB2/FOXG1/FZD3/GPER1/IL6ST/L1CAM/LIMK1/MAG/MAP1B/MAPT/NEFL/NGF/NKX6-1/NTN1/OPRM1/PLXNA1/PLXNA2/PLXNA3/POU4F1/PTN/RELN/RIPK2/ROBO2/SHH/SLIT2/SOX10/TP73/VEGFA/WDR62/WNT2/WNT3A	46	1.06481481481481
GO:0071875	BP	GO:0071875	adrenergic receptor signaling pathway	13/666	33/18614	4.15582742012786e-11	7.16697956839594e-10	4.10901200505347e-10	ADCY9/ADRA1A/ADRA1B/ADRA1D/ADRA2A/ADRA2C/ADRB1/ADRB2/ADRB3/DRD2/DRD5/KCNQ1/RGS2	13	3.93939393939394
GO:0001963	BP	GO:0001963	synaptic transmission, dopaminergic	12/666	27/18614	4.23963179109726e-11	7.26055409520663e-10	4.16266066560329e-10	CHRNB2/DRD2/DRD4/DRD5/GDNF/PINK1/PRKN/RAB3B/SLC6A2/SLC6A3/SNCA/TH	12	4.44444444444444
GO:0001964	BP	GO:0001964	startle response	12/666	27/18614	4.23963179109726e-11	7.26055409520663e-10	4.16266066560329e-10	DRD2/GLRB/GRID2/GRIN2A/GRIN2D/GRIN3A/KCNA1/PENK/PRKN/SLC6A3/TUBA1A/UCN	12	4.44444444444444
GO:0051349	BP	GO:0051349	positive regulation of lyase activity	14/666	40/18614	4.82811361626817e-11	8.23964528609654e-10	4.72399859311034e-10	ADCYAP1/ADORA3/ADRB1/ADRB2/ADRB3/CACNA1C/CACNA1D/CALCA/DRD5/EDNRA/GHRHR/HPCA/NOS1/VIPR2	14	3.5
GO:0008344	BP	GO:0008344	adult locomotory behavior	19/666	81/18614	4.86147125208514e-11	8.26786546851158e-10	4.7401779427543e-10	ATP1A2/CHL1/CNTN2/CXCL12/DRD2/DRD4/EPHA4/FGF12/GDNF/GLRB/GRIN2D/NTF4/NTSR1/OPRD1/PRKN/SNCA/SNCG/TUBA1A/UCHL1	19	2.34567901234568

	ONTOLOGY	ID	Description	GeneRatio	BgRatio	pvalue	p.adjust	qvalue	geneID	Count	richFactor
GO:0090494	BP	GO:0090494	dopamine uptake	10/666	17/18614	4.98663245798865e-11	8.45148225207387e-10	4.84545012342491e-10	DRD2/DRD4/GDNF/PRKN/RAB3B/SLC22A2/SLC22A3/SLC6A2/SLC6A3/SNCA	10	5.88235294117647
GO:0051282	BP	GO:0051282	regulation of sequestering of calcium ion	24/666	133/18614	5.45089796072699e-11	9.206585387276e-10	5.27836999127009e-10	ATP1A2/BDKRB1/CACNA1C/CAMK2D/CCR7/DRD2/F2/FKBP1B/GPER1/HTR2B/HTR2C/ITGB3/ITPR1/NOS1/NPSR1/NTSR1/P2RX7/PLCB4/PLCG1/PLCG2/PTK2B/RYR1/SNCA/TRPC1	24	1.80451127819549
GO:0033605	BP	GO:0033605	positive regulation of catecholamine secretion	9/666	13/18614	5.73057490773839e-11	9.64581358614185e-10	5.5301907094505e-10	CARTPT/CHRNB2/CXCL12/GDNF/OXT/OXTR/PINK1/STX1A/VIP	9	6.92307692307692
GO:0099171	BP	GO:0099171	presynaptic modulation of chemical synaptic transmission	13/666	34/18614	6.50971599366012e-11	1.09198819484094e-09	6.26064656548524e-10	ADORA3/ADRA1A/ADRA2A/CHRM2/CHRNA6/DRD2/GRIK2/GRIN3A/GRM2/NGFR/PRKCB/PRKCG/SYT12	13	3.82352941176471
GO:0045666	BP	GO:0045666	positive regulation of neuron differentiation	20/666	92/18614	6.64873486520884e-11	1.11151468920073e-09	6.37259693310565e-10	ADRA2C/ASCL1/ATOH1/CPNE1/CSNK1E/CXCL12/DKK1/FEZF1/FEZF2/FGFR1/FOXG1/MAP1B/MEF2C/NEUROD1/NGF/NKX6-1/NRCAM/PHOX2B/PROX1/VWC2	20	2.17391304347826
GO:0042417	BP	GO:0042417	dopamine metabolic process	14/666	41/18614	7.09239016930048e-11	1.18166432820718e-09	6.77478268803475e-10	CHRNB2/COMT/DRD2/DRD4/GRIN2A/MAOA/MAOB/NPY/PRKN/SLC1A1/SLC6A3/SNCA/SNCAIP/TH	14	3.41463414634146
GO:0050886	BP	GO:0050886	endocrine process	20/666	93/18614	8.1907636834981e-11	1.36005417244571e-09	7.79753711974552e-10	AGT/AGTR1/AVPR1B/CGA/CTSG/DRD5/EDN2/EDN3/EDNRB/F2RL1/GAL/GALR1/INHBA/KCNQ1/KISS1/LEP/OXTR/TACR1/TACR2/UCN	20	2.1505376344086
GO:0048168	BP	GO:0048168	regulation of neuronal synaptic plasticity	16/666	57/18614	8.9146494102281e-11	1.47526942260172e-09	8.458095506355e-10	AGT/CAMK2A/CAMK2B/CNTN2/DRD2/EPHB2/GRIK2/GRIN2A/GRIN2B/GRIN2D/NSMF/SHISA7/SNCA/SYP/SYT4/VGF	16	2.80701754385965
GO:0060485	BP	GO:0060485	mesenchyme development	38/666	320/18614	8.98480941991068e-11	1.48189054694164e-09	8.49605609929385e-10	APC/AXIN2/BCL2/DKK1/EDN3/EDNRA/EDNRB/EPHA3/EPHA4/ERBB4/FGFR1/GDNF/HMGA2/HTR2B/IGF1/MEF2C/NKX2-1/NRP2/OLFM1/PDPN/PHOX2B/ROBO2/SEMA3D/SEMA3E/SEMA3F/SEMA3G/SEMA6A/SEMA6D/SHH/SOX10/SOX9/TBX20/TWIST1/WNT11/WNT16/WNT2/WNT3A/WNT5A	38	1.1875
GO:0051208	BP	GO:0051208	sequestering of calcium ion	24/666	137/18614	1.03912117623516e-10	1.70812059571767e-09	9.79309061355935e-10	ATP1A2/BDKRB1/CACNA1C/CAMK2D/CCR7/DRD2/F2/FKBP1B/GPER1/HTR2B/HTR2C/ITGB3/ITPR1/NOS1/NPSR1/NTSR1/P2RX7/PLCB4/PLCG1/PLCG2/PTK2B/RYR1/SNCA/TRPC1	24	1.75182481751825
GO:0021987	BP	GO:0021987	cerebral cortex development	23/666	126/18614	1.07849263390653e-10	1.76693043188354e-09	1.01302623893607e-09	ADGRG1/ASCL1/ASPM/ATOH1/CDK5R2/COL3A1/EMX1/FGF13/FOXG1/KCNA1/KCNA2/KIF14/MDGA1/NEFL/NKX2-1/NPY/RELN/SLIT2/TH/TUBA1A/TUBB2A/TUBB2B/WDR62	23	1.82539682539683
GO:0090493	BP	GO:0090493	catecholamine uptake	10/666	18/18614	1.08608422328082e-10	1.77345646426088e-09	1.01676777958481e-09	DRD2/DRD4/GDNF/PRKN/RAB3B/SLC22A2/SLC22A3/SLC6A2/SLC6A3/SNCA	10	5.55555555555556

	ONTOLOGY	ID	Description	GeneRatio	BgRatio	pvalue	p.adjust	qvalue	geneID	Count	richFactor
GO:0043405	BP	GO:0043405	regulation of MAP kinase activity	28/666	186/18614	1.20067876163552e-10	1.95408480577436e-09	1.12032661028173e-09	ADRA2A/AGT/CAV1/DKK1/DRD4/DUSP1/EDN3/EPHA4/FGFR1/FZD10/GHR/HTR2B/IRAK1/LRRK2/MAP2K6/MEF2C/MIF/NOX4/NTRK3/P2RX7/PIK3CG/PIK3R5/PTK2B/RGS2/TENM1/UCHL1/VEGFA/WNT5A	28	1.50537634408602
GO:0045926	BP	GO:0045926	negative regulation of growth	33/666	252/18614	1.21256010336019e-10	1.96159091585699e-09	1.12463005445182e-09	ADRB1/ADRB2/ADRB3/AGT/BCL2/BDKRB1/CGA/FGF13/FSTL4/GDF15/GNG4/INHBA/MAG/MAP2/NDUFA13/NTN1/PLXNA3/RGS2/RTN4R/SEMA3D/SEMA3E/SEMA3F/SEMA3G/SEMA6A/SEMA6D/SLIT2/SLIT3/TNR/TP73/WNT11/WNT3/WNT3A/WNT5A	33	1.30952380952381
GO:0045776	BP	GO:0045776	negative regulation of blood pressure	15/666	50/18614	1.21327291642019e-10	1.96159091585699e-09	1.12463005445182e-09	ADRA1A/ADRB1/ADRB2/ADRB3/APLN/BDKRB1/CALCA/CNR1/DRD2/DRD5/NOS1/NPY/NTSR1/OXT/UCN	15	3
GO:0019098	BP	GO:0019098	reproductive behavior	14/666	43/18614	1.47621495281815e-10	2.37888409609876e-09	1.36387486753898e-09	CNR1/DRD5/EDNRB/MTNR1A/NR3C1/OXT/OXTR/P2RX1/P2RY1/TACR1/TH/THRB/VGF/VIP	14	3.25581395348837
GO:0006814	BP	GO:0006814	sodium ion transport	33/666	254/18614	1.4981048775802e-10	2.40626976251853e-09	1.37957576789894e-09	ADRB2/ANK3/ASIC1/ATP1A2/CAMK2D/DRD2/DRD4/EDNRA/EDNRB/FGF12/FGF13/GRP/HCN1/KCNQ1/NEDD4L/NOS1/P2RX4/P2RX7/SCN1B/SCN2B/SLC17A7/SLC17A8/SLC38A3/SLC38A5/SLC4A8/SLC5A7/SLC6A1/SLC6A15/SLC6A16/SLC6A2/SLC6A3/SLC6A6/SLC6A9	33	1.2992125984252
GO:0014821	BP	GO:0014821	phasic smooth muscle contraction	11/666	24/18614	1.85124435846335e-10	2.9638000071164e-09	1.69922206329723e-09	DRD2/EDN2/EDN3/EDNRB/GDNF/HTR1D/HTR2B/P2RX2/PTGER3/SSTR2/TACR2	11	4.58333333333333
GO:0051955	BP	GO:0051955	regulation of amino acid transport	14/666	44/18614	2.09450432155875e-10	3.33154975419458e-09	1.9100623637584e-09	AGT/ATP1A2/GRM2/LEP/NPY5R/NTSR1/P2RX7/RGS2/SLC17A8/SLC38A3/SLC6A1/SNCA/STXBP1/SYT4	14	3.18181818181818
GO:0099084	BP	GO:0099084	postsynaptic specialization organization	14/666	44/18614	2.09450432155875e-10	3.33154975419458e-09	1.9100623637584e-09	CNTNAP1/GAP43/GRID2/ITGB3/NLGN1/NPTX1/NRXN1/NRXN2/NTRK3/PTK2B/RELN/SLITRK3/SPTBN2/TMEM108	14	3.18181818181818
GO:0010721	BP	GO:0010721	negative regulation of cell development	35/666	286/18614	2.20110638378205e-10	3.48981866977057e-09	2.00080196583516e-09	ATOH1/CARTPT/EDNRB/EPHB2/F2/FGF13/FSTL4/IGF1/IL1A/INHBA/MAG/MAP2/NKX6-1/NTN1/NTRK3/PLXNA3/PROX1/PTN/RTN4R/SEMA3D/SEMA3E/SEMA3F/SEMA3G/SEMA6A/SEMA6D/SHH/SOX10/SYT4/TNR/VAX1/VEGFA/WNT3/WNT3A/WNT5A/WNT7A	35	1.22377622377622
GO:0051384	BP	GO:0051384	response to glucocorticoid	24/666	142/18614	2.24887432778888e-10	3.55408917076602e-09	2.03764988170277e-09	A2M/AXIN2/BCHE/BCL2/CCND1/CLDN1/DDIT4/FOSB/GHR/GHRHR/MAOB/NEFL/NR3C1/NTRK3/OXT/SLIT2/SLIT3/SSTR2/SSTR3/SSTR5/TH/UCN/UCN3/WNT7B	24	1.69014084507042

	ONTOLOGY	ID	Description	GeneRatio	BgRatio	pvalue	p.adjust	qvalue	geneID	Count	richFactor
GO:0007617	BP	GO:0007617	mating behavior	13/666	37/18614	2.263181786078 92e-10	3.565236691851 89e-09	2.044041039586 12e-09	CNR1/DRD5/EDN RB/MTNR1A/OXT/ OXTR/P2RX1/P2R Y1/TACR1/TH/TH RB/VGF/VIP	13	3.513513513513 51
GO:0001659	BP	GO:0001659	temperature homeostasis	27/666	179/18614	2.463164001735 87e-10	3.867875740744 98e-09	2.217551717722 19e-09	ADCYAP1/ADRB1/ ADRB2/ADRB3/A PC/CAV1/CCR2/C NR1/DRD2/EDN2/ EDNRB/EPAS1/H CRT/IL1A/LEP/LEP R/MAP2K6/MC3R/ NMU/NTSR1/OXT/ OXTR/PHOX2B/PL CL1/PTGER3/TSH R/VEGFA	27	1.508379888268 16
GO:0032355	BP	GO:0032355	response to estradiol	22/666	120/18614	2.515925245507 32e-10	3.938144134289 33e-09	2.257838378217 6e-09	ADCYAP1R1/AGT/ CCND1/GPER1/LE P/MAP1B/OXT/OX TR/PENK/POU4F1 /PTGFR/SLC6A1/S STR1/SSTR2/SST R3/STXBP1/TACR 1/TH/TXNIP/UCN/ WNT7A/WNT8B	22	1.833333333333 33
GO:0010977	BP	GO:0010977	negative regulation of neuron projection development	24/666	143/18614	2.612973336554 64e-10	4.077067920370 18e-09	2.337486924630 5e-09	DKK1/EPHA4/EPH B2/FGF13/FSTL4/ LPAR1/LRRK2/MA G/MAP2/NLGN1/N TN1/PLXNA3/RTN 4R/SEMA3D/SEM A3E/SEMA3F/SEM A3G/SEMA6A/SE MA6D/SLIT2/TNR/ WNT3/WNT3A/W NT5A	24	1.678321678321 68
GO:0015800	BP	GO:0015800	acidic amino acid transport	16/666	61/18614	2.732571067556 82e-10	4.236778169413 8e-09	2.429052879910 21e-09	ATP1A2/GRM2/N PY5R/NTSR1/P2R X7/SLC17A7/SLC 17A8/SLC1A1/SL C1A2/SLC1A7/SL C6A1/SLC6A6/SL C7A11/SNCA/STX BP1/SYT4	16	2.622950819672 13
GO:0022029	BP	GO:0022029	telencephalon cell migration	16/666	61/18614	2.732571067556 82e-10	4.236778169413 8e-09	2.429052879910 21e-09	ADGRG1/CDK5R2 /CNTN2/COL3A1/ CXCL12/DRD2/FE ZF2/FGF13/FOXG 1/LRRK2/MDGA1/ NKX2-1/NRG3/RE LN/SLIT2/TNR	16	2.622950819672 13
GO:0070482	BP	GO:0070482	response to oxygen levels	38/666	333/18614	2.888649837490 83e-10	4.464689921782 21e-09	2.559720494856 98e-09	BCL2/CAPN2/CAV 1/CHRNA4/CHRN A7/CHRNB2/CXC L12/DDIT4/DRD2/ EDNRA/EPAS1/EP HA4/FAS/GNGT1/ GUCY1A1/IL1A/IR AK1/ITPR1/LEP/LP AR1/NDNF/NOS1/ OPRD1/OXTR/P2 RX2/PDPN/PENK/ PINK1/PTK2B/RYR 1/SDHD/SLC1A1/ SLC29A1/SLC2A4 /TH/TWIST1/UCN 3/VEGFA	38	1.141141141141 14
GO:1903531	BP	GO:1903531	negative regulation of secretion by cell	25/666	156/18614	3.068343250242 75e-10	4.727557076784 68e-09	2.710428932816 32e-09	ADRA2A/ADRA2C /ASIC1/CARTPT/C CR2/CNR1/DRD2/ DRD4/F2RL1/FKB P1B/GNAZ/INHBA /LEP/MAOB/NMB/ NPY5R/OPRM1/P2 RY1/PRKN/RAP1A /SNCA/SYT11/SYT 4/TACR2/UCN	25	1.602564102564 1
GO:0071902	BP	GO:0071902	positive regulation of protein serine/threonine kinase activity	28/666	195/18614	3.693077117121 03e-10	5.672335634578 07e-09	3.252094553464 8e-09	ADRA2A/ADRB2/ CCND1/DKK1/DR D4/EDN3/EPHA4/ FGFR1/FZD10/GH R/HMGA2/HTR2B/ IRAK1/LRRK2/MA P2K6/MEF2C/MIF/ NOX4/NTRK3/P2R X7/PIK3CG/PIK3R 5/PROX1/PTK2B/ SNCA/TENM1/VE GFA/WNT5A	28	1.435897435897 44
GO:2000179	BP	GO:2000179	positive regulation of neural precursor cell proliferation	15/666	54/18614	4.084008080947 88e-10	6.253239787494 96e-09	3.585141706082 13e-09	ADGRG1/ASCL1/ ASPM/CX3CR1/D RD2/FOXG1/FZD 3/FZD9/LHX1/PR OX1/SHH/SOX10/ VEGFA/WDR62/W NT3A	15	2.777777777777 78
GO:0032098	BP	GO:0032098	regulation of appetite	10/666	20/18614	4.296967457068 56e-10	6.558880450773 9e-09	3.760373286228 35e-09	CARTPT/HTR2C/H TR4/LEP/NPY/OPR M1/SCTR/SLC22A 3/SPX/UCN	10	5

	ONTOLOGY	ID	Description	GeneRatio	BgRatio	pvalue	p.adjust	qvalue	geneID	Count	richFactor
GO:0006865	BP	GO:0006865	amino acid transport	24/666	147/18614	4.69710952587303e-10	7.14745923209472e-09	4.09782049886332e-09	AGT/ATP1A2/GRM2/LEP/NPY5R/NTSR1/P2RX7/RGS2/SLC17A7/SLC17A8/SLC1A1/SLC1A2/SLC1A7/SLC22A2/SLC38A3/SLC38A5/SLC6A1/SLC6A15/SLC6A6/SLC6A9/SLC7A11/SNCA/STXBP1/SYT4	24	1.63265306122449
GO:0007588	BP	GO:0007588	excretion	13/666	39/18614	4.83017033097059e-10	7.32724912861742e-09	4.200898627683e-09	ADRA1A/AGT/AGTR1/ATP6V0A4/CHRNA3/CHRNB2/CHRNB4/DRD2/EDNRB/NPSR1/OXT/SPX/TACR1	13	3.33333333333333
GO:0014032	BP	GO:0014032	neural crest cell development	18/666	82/18614	5.05902212616993e-10	7.65079807696161e-09	4.38639748396985e-09	EDN3/EDNRA/EDNRB/ERBB4/GDNF/HTR2B/NRP2/PHOX2B/SEMA3D/SEMA3E/SEMA3F/SEMA3G/SEMA6A/SEMA6D/SHH/SOX10/SOX9/TWIST1	18	2.19512195121951
GO:0140353	BP	GO:0140353	lipid export from cell	14/666	47/18614	5.63553455639966e-10	8.49651912414243e-09	4.87127090974552e-09	AGT/AGTR1/GAL/GALR1/IL1A/INHBA/KCNQ1/LEP/MAP2K6/MIF/NMB/OXT/P2RX4/P2RX7	14	2.97872340425532
GO:0045471	BP	GO:0045471	response to ethanol	22/666	126/18614	6.67481174144802e-10	1.00326298804945e-08	5.75196234728999e-09	ADCYAP1R1/CCND1/CDK1/CHRNB2/CLDN1/CNR1/DRD2/DRD4/GRIN2A/GRIN2B/GRIN3A/LEP/MAOB/NTRK3/OPRM1/PENK/PTK2B/RGS2/SLC2A4/SLC6A3/TACR1/TH	22	1.74603174603175
GO:0021761	BP	GO:0021761	limbic system development	21/666	115/18614	6.93901225281169e-10	1.0397940616637e-08	5.96140430063443e-09	ATP1A2/CDK5R2/DRD2/FEZF2/FGF13/KCNA1/KIF14/KIRREL3/NDNF/NEFL/NEUROD1/NKX2-1/NRP2/PLXNA1/PLXNA3/PROX1/RELN/SEMA3E/TMEM108/TUBA1A/WNT3A	21	1.82608695652174
GO:0046887	BP	GO:0046887	positive regulation of hormone secretion	23/666	138/18614	7.11124593353323e-10	1.06236394417373e-08	6.09080318799183e-09	ADCYAP1/DRD2/EDN3/F2/F2RL2/GAL/GALR1/GCG/GHRHR/GNA11/GPER1/GRP/INHBA/ITPR1/KISS1/LEP/NKX6-1/NMB/P2RY1/PRKCB/TACR1/UCN/UCN3	23	1.66666666666667
GO:0006898	BP	GO:0006898	receptor-mediated endocytosis	32/666	256/18614	7.68874492038457e-10	1.13943664059502e-08	6.53268059511242e-09	ADRB2/APELA/APLN/APOC1/ARRB1/CACNG4/CACNG5/CACNG7/CACNG8/CALY/CAV1/CLU/CNTN2/CXCL8/DKK1/DRD2/DRD4/GRIA1/GSG1L/HPCA/HTR2B/ITGA4/ITGB3/LPAR1/MAGI2/NLGN3/PLCG2/SNAP25/SNCA/TMEM108/VEGFA/WNT3A	32	1.25
GO:0014075	BP	GO:0014075	response to amine	14/666	48/18614	7.69670324878019e-10	1.13943664059502e-08	6.53268059511242e-09	ASIC1/CDK1/CNR2/DRD2/DRD4/DRD5/EDNRA/GRIN2A/OXT/OXTR/RGS2/SLC18A2/SLC1A1/TH	14	2.91666666666667
GO:0032309	BP	GO:0032309	icosanoid secretion	14/666	48/18614	7.69670324878019e-10	1.13943664059502e-08	6.53268059511242e-09	AVPR1B/BDKRB2/DRD2/DRD4/IL1A/LEP/MAP2K6/MIF/NMB/NMUR2/NTSR1/OXT/P2RX4/P2RX7	14	2.91666666666667
GO:0072578	BP	GO:0072578	neurotransmitter-gated ion channel clustering	9/666	16/18614	8.32336942771810e-10	1.22850933144848e-08	7.04335702797577e-09	CHRDL1/FRRS1L/GLRB/LHFPL4/NLGN1/RELN/SHISA7/SLC7A11/SLITRK3	9	5.625

	ONTOLOGY	ID	Description	GeneRatio	BgRatio	pvalue	p.adjust	qvalue	geneID	Count	richFactor
GO:0048017	BP	GO:0048017	inositol lipid-mediated signaling	26/666	176/18614	8.41649777047536e-10	1.23853552520618e-08	7.10083975151672e-09	AGT/CCL5/ERBB4/F2/F2RL1/FGFR1/GPER1/GPR143/HTR2B/IGF1/LEP/NTRK3/PIK3CD/PIK3CG/PIK3R2/PIK3R5/PLCB4/PLCG1/PLCG2/PLD1/RELN/SEMA3E/SOX9/TWIST1/TYRO3/WNT16	26	1.47727272727273
GO:0051953	BP	GO:0051953	negative regulation of amine transport	11/666	27/18614	8.76543023984673e-10	1.28220504847758e-08	7.35120825566093e-09	ADRA2A/ADRA2C/CNR1/DRD2/LEP/NPY5R/P2RY1/RGS2/SNCA/SYT11/SYT4	11	4.07407407407407
GO:0071880	BP	GO:0071880	adenylate cyclase-activating adrenergic receptor signaling pathway	11/666	27/18614	8.76543023984673e-10	1.28220504847758e-08	7.35120825566093e-09	ADCY9/ADRA1A/ADRA1B/ADRA1D/ADRA2A/ADRA2C/ADRB1/ADRB2/ADRB3/DRD2/DRD5	11	4.07407407407407
GO:0014033	BP	GO:0014033	neural crest cell differentiation	19/666	95/18614	9.04459972525684e-10	1.31911595399517e-08	7.56282788209044e-09	EDN3/EDNRA/EDNRB/ERBB4/GDNF/HTR2B/MEF2C/NRP2/PHOX2B/SEMA3D/SEMA3E/SEMA3F/SEMA3G/SEMA6A/SEMA6D/SHH/SOX10/SOX9/TWIST1	19	2
GO:1904064	BP	GO:1904064	positive regulation of cation transmembrane transport	24/666	152/18614	9.49273567662041e-10	1.38037857546122e-08	7.91406210100057e-09	ADCYAP1R1/ANK3/ATP2A1/BDKRB1/CAV1/DRD4/EDN3/EDNRA/F2/GAL/GPER1/KCNA1/KCNQ1/NOS1/NPSR1/NTSR1/P2RX1/P2RX2/P2RX4/P2RX7/PLCG1/PLCG2/SNCA/TRPC1	24	1.57894736842105
GO:0043271	BP	GO:0043271	negative regulation of monoatomic ion transport	23/666	140/18614	9.53305041547691e-10	1.3821517047808e-08	7.92422790319258e-09	ADRA2A/ANK3/ATP1A2/BCL2/CAMK2D/CAV1/CNR1/DRD4/EPHB2/FKBP1B/GABRE/GRP/HCRT/MAOB/MMP9/NEDD4L/NOS1/NTSR1/PRKG2/PTK2B/SLC6A9/TWIST1/VIP	23	1.64285714285714
GO:0032892	BP	GO:0032892	positive regulation of organic acid transport	14/666	49/18614	1.04241793508018e-09	1.5069071032115e-08	8.63948239074196e-09	AGT/AVPR1B/IL1A/MAP2K6/MIF/NTSR1/OXT/P2RX4/P2RX7/SLC17A8/SLC38A3/SLC6A1/STXBP1/SYT4	14	2.85714285714286
GO:0048841	BP	GO:0048841	regulation of axon extension involved in axon guidance	12/666	34/18614	1.06227359555119e-09	1.53110695663756e-08	8.77822631668631e-09	CXCL12/PLXNA3/SEMA3D/SEMA3E/SEMA3F/SEMA3G/SEMA6A/SEMA6D/VEGFA/WNT3/WNT3A/WNT5A	12	3.52941176470588
GO:0030308	BP	GO:0030308	negative regulation of cell growth	27/666	191/18614	1.08631681094234e-09	1.56118337011158e-08	8.95066205876467e-09	AGT/BCL2/BDKRB1/FGF13/FSTL4/GNG4/INHBA/MAG/MAP2/NDUFA13/NTN1/PLXNA3/RGS2/RTN4R/SEMA3D/SEMA3E/SEMA3F/SEMA3G/SEMA6A/SEMA6D/SLIT2/SLIT3/TNR/WNT11/WNT3/WNT3A/WNT5A	27	1.41361256544503
GO:0051583	BP	GO:0051583	dopamine uptake involved in synaptic transmission	8/666	12/18614	1.12344476950584e-09	1.60515437270965e-08	9.20275902070727e-09	DRD2/DRD4/GDNF/PRKN/RAB3B/SLC6A2/SLC6A3/SNCA	8	6.66666666666667
GO:0051934	BP	GO:0051934	catecholamine uptake involved in synaptic transmission	8/666	12/18614	1.12344476950584e-09	1.60515437270965e-08	9.20275902070727e-09	DRD2/DRD4/GDNF/PRKN/RAB3B/SLC6A2/SLC6A3/SNCA	8	6.66666666666667
GO:0008306	BP	GO:0008306	associative learning	18/666	86/18614	1.15397877243458e-09	1.6440016424684e-08	9.42548031672733e-09	ADCY3/AGT/ASIC1/ATP1A2/CHRNB2/DRD2/DRD5/GRIN2A/NPTX2/RELN/SLC1A1/SLC6A1/SLC7A11/SNAP25/TACR1/TNR/TUBA1A/UCN	18	2.09302325581395

	ONTOLOGY	ID	Description	GeneRatio	BgRatio	pvalue	p.adjust	qvalue	geneID	Count	richFactor
GO:0031960	BP	GO:0031960	response to corticosteroid	25/666	166/18614	1.178497632385 3e-09	1.674079729240 96e-08	9.597925652252 62e-09	A2M/AXIN2/BCHE /BCL2/CCND1/CL DN1/DDIT4/FOSB /GHR/GHRHR/GP ER1/MAOB/NEFL/ NR3C1/NTRK3/O XT/SLIT2/SLIT3/S STR2/SSTR3/SST R5/TH/UCN/UCN3 /WNT7B	25	1.506024096385 54
GO:0050708	BP	GO:0050708	regulation of protein secretion	33/666	275/18614	1.206474883420 41e-09	1.708883012107 01e-08	9.797461740987 25e-09	ADCY5/ADRA2A/ CARTPT/CCL5/CN R1/DRD2/DRD4/E FNA5/F2/F2RL1/F 2RL2/FKBP1B/GC G/GNA11/GNAZ/ GPER1/IGF1/IL1A/ ITPR1/LEP/NEUR OD1/NKX6-1/OPR M1/P2RX7/PRKCB /PRKN/SNAP25/S STR5/STX1A/SYT 4/SYT7/SYT9/UCN 3	33	1.2
GO:0051651	BP	GO:0051651	maintenance of location in cell	30/666	233/18614	1.283769694266 99e-09	1.813140243483 41e-08	1.039519501377 11e-08	ANK3/ASPM/ATP1 A2/ATP2A1/BDKR B1/CACNA1C/CA MK2D/CAV1/CCR 7/DRD2/F2/FKBP 1B/GPER1/GRIK5/ HTR2B/HTR2C/IT GB3/ITPR1/NOS1/ NPSR1/NTSR1/P2 RX7/PINK1/PLCB4 /PLCG1/PLCG2/PT K2B/RYR1/SNCA/ TRPC1	30	1.287553648068 67
GO:0099175	BP	GO:0099175	regulation of postsynapse organization	19/666	97/18614	1.311461028084 71e-09	1.846942966486 06e-08	1.058899463786 08e-08	ARHGAP44/CAMK 2B/EPHA4/EPHB2 /GRID2/GRIN2B/H OMER1/KIF1A/LR RK2/NLGN1/NRC AM/NRP2/PRNP/P TK2B/RELN/RPS6 KA5/SEMA3F/WN T5A/WNT7A	19	1.958762886597 94
GO:0046942	BP	GO:0046942	carboxylic acid transport	38/666	352/18614	1.417692144461 55e-09	1.990844825722 44e-08	1.141402066383 03e-08	AGT/ATP1A2/AVP R1B/BDKRB2/DR D2/DRD4/FABP7/ GRM2/IL1A/LEP/M AP2K6/MIF/NMB/ NMUR2/NPY5R/N TSR1/OXT/P2RX4 /P2RX7/RGS2/SL C17A7/SLC17A8/ SLC1A1/SLC1A2/ SLC1A7/SLC22A2 /SLC22A3/SLC38 A3/SLC38A5/SLC 6A1/SLC6A15/SL C6A6/SLC6A9/SL C7A11/SNCA/SPX /STXBP1/SYT4	38	1.079545454545 45
GO:0050921	BP	GO:0050921	positive regulation of chemotaxis	23/666	143/18614	1.464365679303 1e-09	2.050529149223 57e-08	1.175620609834 87e-08	CCL5/CCR2/CCR7 /CSF1/CX3CR1/C XCL12/CXCL13/C XCL8/EDN2/EDN3 /F2RL1/FGFR1/IL 6R/LPAR1/NTRK3 /P2RX4/PTK2B/PT N/S1PR1/SCG2/S LIT2/VEGFA/WNT 5A	23	1.608391608391 61
GO:0015849	BP	GO:0015849	organic acid transport	38/666	353/18614	1.536023828210 49e-09	2.144760544220 04e-08	1.229645869652 95e-08	AGT/ATP1A2/AVP R1B/BDKRB2/DR D2/DRD4/FABP7/ GRM2/IL1A/LEP/M AP2K6/MIF/NMB/ NMUR2/NPY5R/N TSR1/OXT/P2RX4 /P2RX7/RGS2/SL C17A7/SLC17A8/ SLC1A1/SLC1A2/ SLC1A7/SLC22A2 /SLC22A3/SLC38 A3/SLC38A5/SLC 6A1/SLC6A15/SL C6A6/SLC6A9/SL C7A11/SNCA/SPX /STXBP1/SYT4	38	1.076487252124 65
GO:0031128	BP	GO:0031128	developmental induction	12/666	35/18614	1.564226647963 61e-09	2.171148699764 59e-08	1.244774871611 03e-08	DKK1/GDNF/NKX 2-1/ROBO2/SOX9 /WNT1/WNT11/W NT2/WNT2B/WNT 3/WNT3A/WNT5A	12	3.428571428571 43
GO:0032228	BP	GO:0032228	regulation of synaptic transmission, GABAergic	12/666	35/18614	1.564226647963 61e-09	2.171148699764 59e-08	1.244774871611 03e-08	ADRA1A/CNR1/C NR2/DRD2/NLGN 1/NPY5R/OXTR/P LCL1/SLC6A1/ST XBP1/SYN3/TACR 1	12	3.428571428571 43

	ONTOLOGY	ID	Description	GeneRatio	BgRatio	pvalue	p.adjust	qvalue	geneID	Count	richFactor
GO:0051057	BP	GO:0051057	positive regulation of small GTPase mediated signal transduction	16/666	68/18614	1.56817454408226e-09	2.17114869976459e-08	1.24477487161103e-08	ADCYAP1R1/ADGRG1/ARRB1/COL3A1/CSF1/F2RL1/F2RL2/IGF1/LPAR1/NGF/P2RY10/P2RY8/PIK3CG/RELN/RTN4R/SOS2	16	2.35294117647059
GO:0010001	BP	GO:0010001	glial cell differentiation	30/666	235/18614	1.57795027956309e-09	2.17854652361028e-08	1.24901623252229e-08	ASCL1/CDK1/CLU/CNTN2/CNTNAP1/DNER/EMX1/EPHA4/F2/GAP43/GPM6B/IL6ST/LPAR1/MAG/MAPT/NKX2-1/NKX6-1/NR3C1/NTRK3/PHOX2B/PTN/RELN/SHH/SOX1/SOX10/SOX2/SOX9/TP73/TUBA1A/VAX1	30	1.27659574468085
GO:0031345	BP	GO:0031345	negative regulation of cell projection organization	27/666	195/18614	1.73177551759213e-09	2.38422315657292e-08	1.36693588695095e-08	ARHGAP44/DKK1/EPHA4/EPHB2/FGF13/FSTL4/GRIN2B/LPAR1/LRRK2/MAG/MAP2/NLGN1/NTN1/PLXNA3/PRNP/RTN4R/SEMA3D/SEMA3E/SEMA3F/SEMA3G/SEMA6A/SEMA6D/SLIT2/TNR/WNT3/WNT3A/WNT5A	27	1.38461538461538
GO:0030168	BP	GO:0030168	platelet activation	22/666	133/18614	1.9323306151816e-09	2.65290641721161e-08	1.52097884646902e-08	ADRA2A/ADRA2C/AXL/COL3A1/CTSG/F2/F2RL2/GNA13/GNAQ/IL6ST/ITGB3/P2RX1/P2RY1/PDPN/PIK3CG/PLCG2/PRKG1/SLC7A11/STXBP1/TYRO3/VCL/WNT3A	22	1.65413533834586
GO:0031503	BP	GO:0031503	protein-containing complex localization	26/666	183/18614	1.98177067017191e-09	2.71320413478968e-08	1.55554906437479e-08	ARHGAP44/CACNG4/CACNG5/CACNG7/CACNG8/CALY/CPLX1/DRD4/ERBB4/GPC6/GSG1L/HPCA/ITGB3/KIF5A/KIF5C/LGI1/LHFPL4/LPAR1/LRRC7/NLGN1/NPTX1/NSG1/RAP1A/RELN/SLC1A1/SNAP25	26	1.4207650273224
GO:0070371	BP	GO:0070371	ERK1 and ERK2 cascade	37/666	341/18614	2.0346808428704e-09	2.77790453964112e-08	1.59264345507721e-08	ADCYAP1/ADRA1A/AGT/APELA/ARRB1/CCL5/CCR7/CDK1/CHRNA7/DRD2/EPHA4/EPHB2/ERBB4/F2RL1/GCG/GPER1/HTR2B/HTR2C/IGF1/IL1A/INHBA/ITGB3/MIF/NOX4/NPSR1/NPY/NPY5R/OPRM1/OXTR/P2RY1/PTGER4/PTK2B/RAP1A/RIPK2/RPS6KA6/SEMA6A/SOX9	37	1.08504398826979
GO:0030431	BP	GO:0030431	sleep	11/666	29/18614	2.17873004486875e-09	2.96633190319388e-08	1.70067366383252e-08	ADRB1/CHRNB2/DRD2/GHRHR/GRIN2A/HCRT/KCNA2/OXT/OXTR/PTGDR/SLC29A1	11	3.79310344827586
GO:0051930	BP	GO:0051930	regulation of sensory perception of pain	12/666	36/18614	2.27047383622554e-09	3.07420906475167e-08	1.76252238932167e-08	EDNRB/GPR171/GRIN2D/IL1A/MGLL/NMUR2/NTSR1/OPRD1/OPRM1/OXT/SPX/VIP	12	3.33333333333333
GO:0051931	BP	GO:0051931	regulation of sensory perception	12/666	36/18614	2.27047383622554e-09	3.07420906475167e-08	1.76252238932167e-08	EDNRB/GPR171/GRIN2D/IL1A/MGLL/NMUR2/NTSR1/OPRD1/OPRM1/OXT/SPX/VIP	12	3.33333333333333
GO:0021872	BP	GO:0021872	forebrain generation of neurons	14/666	52/18614	2.47249671454379e-09	3.33854982197328e-08	1.91407568098141e-08	ASCL1/ASPM/FEZF2/FOXG1/INHBA/NDNF/NKX2-1/NRP2/PLXNA1/PLXNA3/PROX1/SEMA3E/SOX1/WNT3A	14	2.69230769230769

	ONTOLOGY	ID	Description	GeneRatio	BgRatio	pvalue	p.adjust	qvalue	geneID	Count	richFactor
GO:0048015	BP	GO:0048015	phosphatidylinositol-mediated signaling	25/666	172/18614	2.50874393532676e-09	3.37821272387151e-08	1.93681543327173e-08	AGT/CCL5/ERBB4/F2/F2RL1/FGFR1/GPER1/GPR143/HTR2B/IGF1/LEP/NTRK3/PIK3CD/PIK3CG/PIK3R2/PIK3R5/PLCB4/PLCG1/PLCG2/RELN/SEMA3E/SOX9/TWIST1/TYRO3/WNT16	25	1.45348837209302
GO:0009410	BP	GO:0009410	response to xenobiotic stimulus	43/666	440/18614	2.5628371322281e-09	3.44162418166697e-08	1.9731708377839e-08	ADCYAP1R1/ADRA1A/BCHE/BCL2/CCND1/CDK1/CYP2C18/CYP2C8/DRD2/EMX1/FOSB/FZD3/GAL/GRIN2A/GRM2/HTR2B/INHBA/ITGB3/KCNQ1/MAOB/MAP1B/MAP2K6/MEF2C/NEUROD1/NKX6-1/NOS1/OXTR/P2RX7/PRNP/PTK2B/SLC1A1/SLC1A2/SLC28A2/SLC29A1/SLC6A2/SLC6A3/SNCA/SOX10/SST/TH/TP73/TXNIP/UCHL1	43	0.977272727272727
GO:0071526	BP	GO:0071526	semaphorin-plexin signaling pathway	13/666	44/18614	2.61746501497539e-09	3.4958805838598e-08	2.00427741564334e-08	EDNRA/GDNF/NCAM1/NRP2/PLXNA1/PLXNA2/PLXNA3/SEMA3D/SEMA3E/SEMA3F/SEMA3G/SEMA6A/SEMA6D	13	2.95454545454545
GO:1903539	BP	GO:1903539	protein localization to postsynaptic membrane	13/666	44/18614	2.61746501497539e-09	3.4958805838598e-08	2.00427741564334e-08	ARHGAP44/CACNG7/CPLX1/ERBB4/GPC6/GRIN2A/LGI1/LHFPL4/LRRC7/NPTX1/NSG1/RAP1A/SNAP25	13	2.95454545454545
GO:0051610	BP	GO:0051610	serotonin uptake	8/666	13/18614	2.82926720219139e-09	3.75833737804613e-08	2.15475058329057e-08	GPM6B/ITGB3/NOS1/SLC18A2/SLC18A3/SLC22A2/SLC22A3/SNCA	8	6.15384615384615
GO:0097475	BP	GO:0097475	motor neuron migration	8/666	13/18614	2.82926720219139e-09	3.75833737804613e-08	2.15475058329057e-08	ASCL1/FZD3/LHX1/NTN1/PHOX2B/RELN/TBX20/VEGFA	8	6.15384615384615
GO:0099590	BP	GO:0099590	neurotransmitter receptor internalization	11/666	30/18614	3.32945586844744e-09	4.41085595509951e-08	2.52885611003938e-08	CACNG4/CACNG5/CACNG7/CACNG8/CALY/DRD4/GSG1L/HPCA/ITGB3/LPAR1/SNAP25	11	3.66666666666667
GO:0062237	BP	GO:0062237	protein localization to postsynapse	13/666	45/18614	3.56132130981402e-09	4.69273303960748e-08	2.69046342496744e-08	ARHGAP44/CACNG7/CPLX1/ERBB4/GPC6/GRIN2A/LGI1/LHFPL4/LRRC7/NPTX1/NSG1/RAP1A/SNAP25	13	2.88888888888889
GO:1902692	BP	GO:1902692	regulation of neuroblast proliferation	13/666	45/18614	3.56132130981402e-09	4.69273303960748e-08	2.69046342496744e-08	ASPM/CX3CR1/DRD2/FOXG1/FZD3/LRRK2/PROX1/PTN/SHH/SOX10/VAX1/VEGFA/WDR62	13	2.88888888888889
GO:0060048	BP	GO:0060048	cardiac muscle contraction	22/666	138/18614	3.94775135877355e-09	5.1880208364631e-08	2.97442453910408e-08	ADRA1A/ADRA1B/ATP1A2/ATP2A1/CACNA1C/CACNA1D/CAMK2D/CAV1/FGF12/FGF13/FKBP1B/KCNJ5/KCNQ1/MAP2K6/NEDD4L/NOS1/P2RX4/PIK3CG/RGS2/SCN1B/SCN2B/UCN	22	1.59420289855072
GO:0007190	BP	GO:0007190	activation of adenylate cyclase activity	10/666	24/18614	4.00547637536747e-09	5.23588201726891e-08	3.00186457358866e-08	ADCYAP1/ADORA3/ADRB1/ADRB2/ADRB3/CALCA/DRD5/EDNRA/GHRHR/VIPR2	10	4.16666666666667
GO:0021871	BP	GO:0021871	forebrain regionalization	10/666	24/18614	4.00547637536747e-09	5.23588201726891e-08	3.00186457358866e-08	ADGRG1/EMX1/FEZF1/FEZF2/LHX1/NKX2-1/SHH/WNT1/WNT2B/WNT7B	10	4.16666666666667
GO:0097106	BP	GO:0097106	postsynaptic density organization	12/666	38/18614	4.59882706650992e-09	5.99555305885843e-08	3.43740333855844e-08	CNTNAP1/GRID2/ITGB3/NLGN1/NPTX1/NRXN1/NRXN2/NTRK3/PTK2B/RELN/SLITRK3/TMEM108	12	3.15789473684211

	ONTOLOGY	ID	Description	GeneRatio	BgRatio	pvalue	p.adjust	qvalue	geneID	Count	richFactor
GO:0036293	BP	GO:0036293	response to decreased oxygen levels	34/666	306/18614	5.04212974514211e-09	6.55610256544272e-08	3.75878065378597e-08	BCL2/CAPN2/CAV1/CHRNA4/CHRNA7/CHRNB2/CXCL12/DDIT4/DRD2/EDNRA/EPAS1/EPHA4/GNGT1/IL1A/IRAK1/ITPR1/LEP/NDNF/NOS1/OPRD1/OXTR/P2RX2/PENK/PINK1/PTK2B/RYR1/SDHD/SLC1A1/SLC29A1/SLC2A4/TH/TWIST1/UCN3/VEGFA	34	1.11111111111111
GO:0070050	BP	GO:0070050	neuron cellular homeostasis	15/666	64/18614	5.39966702619965e-09	6.98404300888717e-08	4.00412981416522e-08	ATP6V0A4/ATP6V1F/ATP6V1G2/CALB1/CALB2/CHRNA1/CNR1/NPY/P2RX1/P2RY1/P2RY4/PRKN/SLC6A2/SV2B/TYRO3	15	2.34375
GO:0071300	BP	GO:0071300	cellular response to retinoic acid	15/666	64/18614	5.39966702619965e-09	6.98404300888717e-08	4.00412981416522e-08	EPHA3/FZD10/LEP/NDUFA13/NTRK3/PTK2B/SOX9/WNT11/WNT2/WNT3/WNT3A/WNT5A/WNT7B/WNT8B/WNT9A	15	2.34375
GO:0050678	BP	GO:0050678	regulation of epithelial cell proliferation	41/666	419/18614	5.84503875829202e-09	7.54025341128747e-08	4.32301941040136e-08	AGTR1/AKT3/APELA/APLN/BID/CAV1/CCND1/CLDN1/CXCL12/DRD2/EDNRB/FGFR1/HTR2B/IGF1/ITGA4/ITGB3/KRT4/LEP/MEF2C/NGFR/NRP2/PHOX2B/PIK3CD/PLCG1/PRL/PROX1/PTN/SCG2/SHH/SLURP1/SOX2/SOX9/SULF1/TACR1/TWIST1/VEGFA/VIP/WNT2/WNT3A/WNT5A/WNT7A	41	0.978520286396181
GO:0016358	BP	GO:0016358	dendrite development	29/666	234/18614	5.8737502617926e-09	7.55745616144258e-08	4.33288218540061e-08	ARHGAP44/CAMK2A/CAMK2B/CHRNA3/CHRNA7/CHRNB2/DCDC2/DCLK1/EPHA4/EPHB2/EPHB3/FEZF2/FLRT1/FSTL4/GRIN3A/IGSF9/KIF1A/LPAR1/LRRK2/MAP1B/MAP2/MEF2C/NEDD4L/NLGN1/NSMF/PRKG1/PTN/RELN/WNT7A	29	1.23931623931624
GO:2000027	BP	GO:2000027	regulation of animal organ morphogenesis	21/666	129/18614	6.05479515428576e-09	7.77005696692285e-08	4.45477164436526e-08	AGT/CELSR1/CSF1/DKK1/FZD3/GDNF/GPC6/LHX1/MAGI2/NGFR/ROBO2/SHH/SOX9/SULF1/VEGFA/WNT11/WNT2/WNT2B/WNT3A/WNT5A/WNT7A	21	1.62790697674419
GO:0014061	BP	GO:0014061	regulation of norepinephrine secretion	8/666	14/18614	6.39447368594492e-09	8.12184920148425e-08	4.65646309629455e-08	ADORA3/ADRA2A/ADRA2C/AGT/OXT/OXTR/P2RY1/STX1A	8	5.71428571428571
GO:0048243	BP	GO:0048243	norepinephrine secretion	8/666	14/18614	6.39447368594492e-09	8.12184920148425e-08	4.65646309629455e-08	ADORA3/ADRA2A/ADRA2C/AGT/OXT/OXTR/P2RY1/STX1A	8	5.71428571428571
GO:0070593	BP	GO:0070593	dendrite self-avoidance	8/666	14/18614	6.39447368594492e-09	8.12184920148425e-08	4.65646309629455e-08	CNTN2/CNTN4/CNTN6/DSCAML1/IGSF9/MYPN/NEXN/PALLD	8	5.71428571428571
GO:1900271	BP	GO:1900271	regulation of long-term synaptic potentiation	13/666	47/18614	6.4241536258436e-09	8.12184920148425e-08	4.65646309629455e-08	CALB1/CALB2/CHRNA7/CX3CR1/DRD2/EPHA4/EPHB2/NSG1/PRNP/PTN/RELN/SHISA7/SLC18A3	13	2.76595744680851
GO:0007210	BP	GO:0007210	serotonin receptor signaling pathway	12/666	39/18614	6.42807597024898e-09	8.12184920148425e-08	4.65646309629455e-08	CHRM2/CHRM4/CHRM5/DRD4/GNAZ/HTR1D/HTR2B/HTR2C/HTR3A/HTR3C/HTR3E/HTR7	12	3.07692307692308
GO:0045823	BP	GO:0045823	positive regulation of heart contraction	12/666	39/18614	6.42807597024898e-09	8.12184920148425e-08	4.65646309629455e-08	ADRA1A/ADRA1B/ADRB1/APELA/APLN/ATP1A2/EDN2/EDN3/KCNQ1/RGS2/SLC1A1/UCN	12	3.07692307692308

	ONTOLOGY	ID	Description	GeneRatio	BgRatio	pvalue	p.adjust	qvalue	geneID	Count	richFactor
GO:0050673	BP	GO:0050673	epithelial cell proliferation	45/666	488/18614	6.640639223106 44e-09	8.368908149120 04e-08	4.798108286168 95e-08	AGTR1/AKT3/APELA/APLN/BCL2L1/BID/CAV1/CCND1/CLDN1/CXCL12/DRD2/EDNRB/FABP7/FGFR1/HTR2B/IGF1/ITGA4/ITGB3/KRT4/LEP/MEF2C/NGFR/NKX6-1/NRP2/PHOX2B/PIK3CD/PLCG1/PRL/PROX1/PTN/SCG2/SHH/SLURP1/SOX2/SOX9/SULF1/TACR1/TWIST1/VEGFA/VIP/WNT16/WNT2/WNT3A/WNT5A/WNT7A	45	0.922131147540 984
GO:0071715	BP	GO:0071715	icosanoid transport	15/666	65/18614	6.790303508814 47e-09	8.535637275146 58e-08	4.893698342467 72e-08	AVPR1B/BDKRB2/DRD2/DRD4/IL1A/LEP/MAP2K6/MIF/NMB/NMUR2/NTSR1/OXT/P2RX4/P2RX7/SLC22A2	15	2.307692307692 31
GO:0042698	BP	GO:0042698	ovulation cycle	16/666	75/18614	7.226794975860 5e-09	9.061147272029 18e-08	5.194986613957 73e-08	A2M/AXL/CGA/GABRB1/INHBA/KISS1/LEP/MAP2K6/NPY5R/OPRM1/OXTR/PTN/ROBO2/SLIT2/SLIT3/TYRO3	16	2.133333333333 33
GO:0070374	BP	GO:0070374	positive regulation of ERK1 and ERK2 cascade	28/666	222/18614	7.268868207304 38e-09	9.090709221094 41e-08	5.211935232611 17e-08	ADCYAP1/ADRA1A/APELA/ARRB1/CCL5/CCR7/CHRNA7/DRD2/ERBB4/F2RL1/GCG/GPER1/HTR2B/HTR2C/IGF1/IL1A/INHBA/ITGB3/MIF/NOX4/NPSR1/NPY/NPY5R/OPRM1/P2RY1/PTK2B/RAP1A/RIPK2	28	1.261261261261 26
GO:0035725	BP	GO:0035725	sodium ion transmembrane transport	25/666	181/18614	7.297869077907 99e-09	9.103813837034 97e-08	5.219448442842 56e-08	ANK3/ASIC1/ATP1A2/CAMK2D/DRD4/FGF12/FGF13/GRP/HCN1/KCNQ1/NEDD4L/NOS1/SCN1B/SCN2B/SLC17A7/SLC17A8/SLC38A5/SLC4A8/SLC6A1/SLC6A15/SLC6A16/SLC6A2/SLC6A3/SLC6A6/SLC6A9	25	1.381215469613 26
GO:0051482	BP	GO:0051482	positive regulation of cytosolic calcium ion concentration involved in phospholipase C-activating G protein-coupled signaling pathway	11/666	32/18614	7.366291378097 52e-09	9.165904335025 14e-08	5.255046507439 59e-08	AGTR1/CALCA/DRD2/F2RL1/F2RL2/HTR2C/KISS1/LPAR1/P2RY10/P2RY8/S1PR1	11	3.4375
GO:0033135	BP	GO:0033135	regulation of peptidyl-serine phosphorylation	22/666	143/18614	7.795033467439 42e-09	9.674896336481 e-08	5.546864591264 04e-08	BCL2/BDKRB2/BDNF/CAV1/DDIT4/DKK1/GCG/HSP90AB1/MIF/NGF/NOS1/NTF4/NTRK3/OPRD1/PINK1/RIPK2/SNCA/TENM1/UCN/VEGFA/WNT3A/WNT5A	22	1.538461538461 54
GO:0097104	BP	GO:0097104	postsynaptic membrane assembly	7/666	10/18614	7.950209657119 74e-09	9.842639915552 52e-08	5.643036259342 31e-08	EPHB2/NLGN1/NLGN3/NLGN4X/NLGN4Y/NRXN1/NRXN2	7	7
GO:0006941	BP	GO:0006941	striated muscle contraction	25/666	182/18614	8.179941548395 8e-09	1.010161123376 01e-07	5.791511114799 14e-08	ADRA1A/ADRA1B/ATP1A2/ATP2A1/CACNA1C/CACNA1D/CAMK2D/CAV1/CHRNA1/CHRND/FGF12/FGF13/FKBP1B/HOMER1/KCNJ5/KCNQ1/MAP2K6/NEDD4L/NOS1/P2RX4/PIK3CG/RGS2/SCN1B/SCN2B/UCN	25	1.373626373626 37
GO:0010517	BP	GO:0010517	regulation of phospholipase activity	15/666	66/18614	8.500479486568 81e-09	1.047114202418 19e-07	6.003372532791 11e-08	ADCYAP1R1/ADRA1A/AGT/AGTR1/AVPR1B/CCL5/EDNRA/FGFR1/GNA13/GNAQ/HPCA/HTR2B/LPAR1/RGS2/SNCA	15	2.272727272727 27

	ONTOLOGY	ID	Description	GeneRatio	BgRatio	pvalue	p.adjust	qvalue	geneID	Count	richFactor
GO:0033138	BP	GO:0033138	positive regulation of peptidyl-serine phosphorylation	19/666	108/18614	8.57213857287189e-09	1.05330152714163e-07	6.03884604199422e-08	BCL2/BDNF/CAV1/GCG/HSP90AB1/MIF/NGF/NOS1/NTF4/NTRK3/OPRD1/PINK1/RIPK2/SNCA/TENM1/UCN/VEGFA/WNT3A/WNT5A	19	1.75925925925926
GO:0006576	BP	GO:0006576	biogenic amine metabolic process	18/666	97/18614	8.79350027118382e-09	1.07780682874984e-07	6.17934118019664e-08	CHRNB2/COMT/DRD2/DRD4/EDNRA/EPAS1/GRIN2A/MAOA/MAOB/NPY/PAH/PRKN/SLC1A1/SLC22A3/SLC6A3/SNCA/SNCAIP/TH	18	1.85567010309278
GO:0007212	BP	GO:0007212	dopamine receptor signaling pathway	12/666	40/18614	8.88625096458164e-09	1.08376981367044e-07	6.21352849214707e-08	ADCY5/CALY/DRD2/DRD4/DRD5/GNA11/GNAO1/GNAQ/GNG2/LRRK2/NSG1/SLC1A1	12	3
GO:1903524	BP	GO:1903524	positive regulation of blood circulation	12/666	40/18614	8.88625096458164e-09	1.08376981367044e-07	6.21352849214707e-08	ADRA1A/ADRA1B/ADRB1/APELA/APLN/ATP1A2/EDN2/EDN3/KCNQ1/RGS2/SLC1A1/UCN	12	3
GO:0002931	BP	GO:0002931	response to ischemia	14/666	57/18614	9.11391786909905e-09	1.1087848100649e-07	6.35694584043204e-08	BCL2/CAMK2A/CAV1/CSF1/CX3CR1/MAP2K6/MEF2C/NDNF/P2RX2/P2RX4/P2RX7/PINK1/PTK2B/UCHL1	14	2.45614035087719
GO:0050679	BP	GO:0050679	positive regulation of epithelial cell proliferation	28/666	225/18614	9.80697377367106e-09	1.19015496537267e-07	6.82346167436451e-08	AGTR1/AKT3/APELA/APLN/CCND1/CLDN1/CXCL12/FGFR1/HTR2B/IGF1/ITGA4/ITGB3/NRP2/PIK3CD/PLCG1/PROX1/PTN/SCG2/SHH/SOX9/TACR1/TWIST1/VEGFA/VIP/WNT2/WNT3A/WNT5A/WNT7A	28	1.24444444444444
GO:0010232	BP	GO:0010232	vascular transport	17/666	87/18614	1.00759637857187e-08	1.21380789232371e-07	6.95906993146771e-08	ATP1A2/ATP2A3/AVPR1B/LEPR/SLC1A1/SLC1A2/SLC22A2/SLC22A3/SLC28A2/SLC29A1/SLC2A4/SLC38A3/SLC38A5/SLC4A8/SLC6A1/SLC6A6/SLC6A9	17	1.95402298850575
GO:0048678	BP	GO:0048678	response to axon injury	17/666	87/18614	1.00759637857187e-08	1.21380789232371e-07	6.95906993146771e-08	AXL/BCL2/CDK1/DRD2/EPHA4/FKBP1B/GAP43/MAG/MAP1B/NEFL/NTRK3/P2RX4/PLCG2/PTN/RTN4R/SLC1A1/TNR	17	1.95402298850575
GO:0150104	BP	GO:0150104	transport across blood-brain barrier	17/666	87/18614	1.00759637857187e-08	1.21380789232371e-07	6.95906993146771e-08	ATP1A2/ATP2A3/AVPR1B/LEPR/SLC1A1/SLC1A2/SLC22A2/SLC22A3/SLC28A2/SLC29A1/SLC2A4/SLC38A3/SLC38A5/SLC4A8/SLC6A1/SLC6A6/SLC6A9	17	1.95402298850575
GO:0099637	BP	GO:0099637	neurotransmitter receptor transport	10/666	26/18614	1.0166126699823e-08	1.22167512786382e-07	7.00417479743309e-08	ARHGAP44/CACNG4/CACNG5/CACNG7/CACNG8/CPLX1/LRRC7/NSG1/SLC1A1/SNAP25	10	3.84615384615385
GO:0048880	BP	GO:0048880	sensory system development	39/666	395/18614	1.07976155630681e-08	1.29439708518243e-07	7.42110830868635e-08	BCL2/CACNA1C/CALB1/CHRDL1/DRD2/EPHB2/GNGT1/GPM6A/HCN1/HPCA/INHBA/LHX1/NEUROD1/OLFM3/PHOX2B/POU4F1/PROX1/SHH/SLC17A7/SLC17A8/SLC1A1/SLC44A4/SLC6A3/SLC7A11/SOX1/SOX2/SOX9/TH/THRB/TWIST1/VAX1/VEGFA/WNT16/WNT2/WNT2B/WNT5A/WNT7A/WNT7B/WNT9A	39	0.987341772151899

	ONTOLOGY	ID	Description	GeneRatio	BgRatio	pvalue	p.adjust	qvalue	geneID	Count	richFactor
GO:0001667	BP	GO:0001667	ameboidal-type cell migration	45/666	496/18614	1.09150586626068e-08	1.30529229505383e-07	7.48357332303712e-08	AGT/AKT3/APELA/CXCL13/EDN2/EDN3/EDNRA/EDNRB/ERBB4/FGFR1/GDNF/GNA13/HTR2B/ITGA4/ITGB3/LTB4R2/MEF2C/MMP9/NRP2/P2RX4/PHOX2B/PIK3CD/PIK3CG/PLCG1/PLCG2/PROX1/PTK2B/PTN/SCG2/SEMA3D/SEMA3E/SEMA3F/SEMA3G/SEMA6A/SEMA6D/SHH/SLIT2/SOX10/SOX9/TACR1/TWIST1/VEGFA/WNT11/WNT5A/WNT7A	45	0.907258064516129
GO:0045933	BP	GO:0045933	positive regulation of muscle contraction	13/666	49/18614	1.12262136274409e-08	1.33924368880757e-07	7.67822531442493e-08	ADRA1A/ATP2A1/EDN2/GPER1/KCNQ1/NMU/OXT/OXTR/RGS2/SPX/TACR1/TACR2/UCN	13	2.6530612244898
GO:0002687	BP	GO:0002687	positive regulation of leukocyte migration	22/666	147/18614	1.31305764918699e-08	1.56263398202278e-07	8.95897878647529e-08	BDKRB1/CCL5/CCR2/CCR7/CSF1/CX3CR1/CXCL12/CXCL13/CXCL8/EDN2/EDN3/F2RL1/IL1A/IL6R/ITGA4/ITGB3/P2RX4/PTK2B/PTN/TACR1/VEGFA/WNT5A	22	1.49659863945578
GO:0001956	BP	GO:0001956	positive regulation of neurotransmitter secretion	8/666	15/18614	1.3272638710838e-08	1.57192817503058e-07	9.01226476319934e-08	GPER1/NLGN1/SLC18A3/SLC4A8/SNCA/STX1A/STXBP1/TACR2	8	5.33333333333333
GO:1904936	BP	GO:1904936	interneuron migration	8/666	15/18614	1.3272638710838e-08	1.57192817503058e-07	9.01226476319934e-08	CNTN2/DRD2/EVX1/FEZF1/FEZF2/NKX2-1/RELN/SOX1	8	5.33333333333333
GO:0060191	BP	GO:0060191	regulation of lipase activity	17/666	89/18614	1.44411361656609e-08	1.70620635226499e-07	9.7821157680856e-08	ADCYAP1R1/ADRA1A/AGT/AGTR1/APOC1/AVPR1B/CCL5/EDNRA/FGFR1/GNA13/GNAQ/GPIHBP1/HPCA/HTR2B/LPAR1/RGS2/SNCA	17	1.91011235955056
GO:0009612	BP	GO:0009612	response to mechanical stimulus	27/666	215/18614	1.48056513927267e-08	1.7450785754257e-07	1.00049801283174e-07	AGT/ATP1A2/BDKRB1/CAPN2/CSF1/CXCL12/DRD2/FAS/FOSB/ITGB3/KCNA1/KCNQ1/MAG/MAP1B/NRXN1/NRXN2/P2RX7/P2RY1/PTGER4/PTK2B/PTN/SOX9/TACR1/TUBA1A/TXNIP/UCN/WNT11	27	1.25581395348837
GO:0015711	BP	GO:0015711	organic anion transport	41/666	433/18614	1.50076707327184e-08	1.76465793424188e-07	1.01172335813365e-07	AGT/ATP1A2/AVPR1B/BDKRB2/DRD2/DRD4/FABP7/GRM2/IL1A/LEP/MAP2K6/MIF/NMB/NMUR2/NPY5R/NTSR1/OXT/P2RX4/P2RX7/RGS2/SLC17A7/SLC17A8/SLC1A1/SLC1A2/SLC1A7/SLC22A2/SLC22A3/SLC2A4/SLC38A3/SLC38A5/SLC44A4/SLC4A8/SLC6A1/SLC6A15/SLC6A6/SLC6A9/SLC7A11/SNCA/SPX/STXBP1/SYT4	41	0.946882217090069
GO:0048265	BP	GO:0048265	response to pain	11/666	34/18614	1.53008807922273e-08	1.79056735937613e-07	1.02657789174919e-07	CALCA/CAPN2/EDNRB/NMUR2/P2RX2/P2RX4/PRKCG/RELN/SLC6A2/TACR1/UCN	11	3.23529411764706
GO:0110110	BP	GO:0110110	positive regulation of animal organ morphogenesis	11/666	34/18614	1.53008807922273e-08	1.79056735937613e-07	1.02657789174919e-07	CSF1/DKK1/GDNF/NGFR/ROBO2/SOX9/WNT11/WNT2/WNT2B/WNT3A/WNT5A	11	3.23529411764706
GO:0048520	BP	GO:0048520	positive regulation of behavior	10/666	27/18614	1.56305876856104e-08	1.82048195437855e-07	1.04372869130404e-07	GHRHR/GRP/GRPR/INSL5/MC1R/MEF2C/NLGN1/NPY/PENK/UCN	10	3.7037037037037
GO:0060259	BP	GO:0060259	regulation of feeding behavior	10/666	27/18614	1.56305876856104e-08	1.82048195437855e-07	1.04372869130404e-07	CNR1/GPR171/INSL5/LEPR/MC1R/MC3R/NMU/NPSR1/NPY/UCN	10	3.7037037037037

	ONTOLOGY	ID	Description	GeneRatio	BgRatio	pvalue	p.adjust	qvalue	geneID	Count	richFactor
GO:0030879	BP	GO:0030879	mammary gland development	21/666	136/18614	1.594718603835 23e-08	1.849482486654 98e-07	1.060355435407 27e-07	APLN/CAV1/CCND1/CSF1/ERBB4/GHRHR/NR3C1/NRG3/NTN1/OXTR/PRL/SLC29A1/SLC6A3/SOX9/TPH1/VEGFA/WNT2/WNT3/WNT3A/WNT5A/WNT7B	21	1.544117647058 82
GO:0007599	BP	GO:0007599	hemostasis	28/666	230/18614	1.595484383197 79e-08	1.849482486654 98e-07	1.060355435407 27e-07	ADRA2A/ADRA2C/AXL/CAV1/COL3A1/CTSG/EPHB2/F2/F2RL1/F2RL2/GNA13/GNAQ/IL6ST/ITGB3/KNG1/P2RX1/P2RY1/PDPN/PIK3CG/PLCG2/PRKG1/SHH/SLC7A11/STXBP1/TPH1/TYRO3/VCL/WNT3A	28	1.217391304347 83
GO:0071900	BP	GO:0071900	regulation of protein serine/threonine kinase activity	37/666	369/18614	1.719755748225 17e-08	1.988846941770 99e-07	1.140256628606 51e-07	ADRA2A/ADRB2/AGT/APC/CAV1/CCNB1/CCND1/CDK5R2/CDKN3/DKK1/DRD4/DUSP1/EDN3/EPHA4/FGFR1/FZD10/GHR/HMGA2/HTR2B/IRAK1/LRRK2/MAP2K6/MEF2C/MIF/NOX4/NTRK3/P2RX7/PIK3CG/PIK3R5/PROX1/PTK2B/RGS2/SNCA/TENM1/UCHL1/VEGFA/WNT5A	37	1.002710027100 27
GO:0001763	BP	GO:0001763	morphogenesis of a branching structure	26/666	203/18614	1.816157282025 53e-08	2.095402122336 97e-07	1.201347428708 27e-07	AGT/BCL2/CELSR1/CSF1/DRD2/EDNRA/FGF13/GDNF/GNA13/LHX1/LRRK2/NKX2-1/PLXNA1/PROX1/SEMA3E/SHH/SLIT2/SOX10/SOX9/SULF1/TBX20/VEGFA/WNT1/WNT2/WNT2B/WNT5A	26	1.280788177339 9
GO:0050922	BP	GO:0050922	negative regulation of chemotaxis	15/666	70/18614	2.001137525512 04e-08	2.303417081473 46e-07	1.320607705114 2e-07	CXCL13/DUSP1/MIF/PLXNA3/ROBO2/SEMA3D/SEMA3E/SEMA3F/SEMA3G/SEMA6A/SEMA6D/SLIT2/WNT3/WNT3A/WNT5A	15	2.142857142857 14
GO:0048863	BP	GO:0048863	stem cell differentiation	29/666	247/18614	2.007432907395 52e-08	2.305264658843 22e-07	1.321666968297 54e-07	A2M/BCL2/EDN3/EDNRA/EDNRB/ERBB4/GDNF/GPM6A/HMGA2/HTR2B/MEF2C/NRP2/PHOX2B/PTN/SEMA3D/SEMA3E/SEMA3F/SEMA3G/SEMA6A/SEMA6D/SHC4/SHH/SOX10/SOX5/SOX9/TWIST1/WNT3/WNT3A/WNT7A	29	1.174089068825 91
GO:0003007	BP	GO:0003007	heart morphogenesis	30/666	262/18614	2.040553855661 54e-08	2.337837342791 72e-07	1.340341717974 71e-07	APC/AXIN2/DKK1/EDNRA/FLRT2/HTR2B/MEF2C/NPY1R/NPY2R/NPY5R/NRP2/OLFM1/POU4F1/PROX1/ROBO2/RYR1/S1PR1/SHH/SLIT2/SLIT3/SOX9/TBX20/TH/TWIST1/VEGFA/WNT11/WNT16/WNT2/WNT3A/WNT5A	30	1.145038167938 93
GO:0043084	BP	GO:0043084	penile erection	7/666	11/18614	2.118648782262 7e-08	2.416046117127 88e-07	1.385180801103 24e-07	CNR1/EDNRB/OXT/OXTR/P2RY1/VGF/VIP	7	6.363636363636 36
GO:0070471	BP	GO:0070471	uterine smooth muscle contraction	7/666	11/18614	2.118648782262 7e-08	2.416046117127 88e-07	1.385180801103 24e-07	ADRA2A/AGT/GPER1/OXT/OXTR/TACR1/TACR2	7	6.363636363636 36
GO:0045777	BP	GO:0045777	positive regulation of blood pressure	11/666	35/18614	2.159684727501 47e-08	2.455454783927 13e-07	1.407774793933 27e-07	ADRA1A/ADRA1B/ADRB1/AGT/CARTPT/CNR1/DRD5/OXT/OXTR/SPX/TACR1	11	3.142857142857 14

	ONTOLOGY	ID	Description	GeneRatio	BgRatio	pvalue	p.adjust	qvalue	geneID	Count	richFactor
GO:0001666	BP	GO:0001666	response to hypoxia	32/666	293/18614	2.16319821249328e-08	2.45545478392713e-07	1.40777479393327e-07	BCL2/CAPN2/CAV1/CHRNA4/CHRNA7/CHRNB2/CXCL12/DDIT4/DRD2/EDNRA/EPAS1/EPHA4/GNGT1/IL1A/IRAK1/ITPR1/LEP/NDNF/NOS1/OPRD1/P2RX2/PENK/PINK1/PTK2B/RYR1/SDHD/SLC29A1/SLC2A4/TH/TWIST1/UCN3/VEGFA	32	1.09215017064846
GO:0021879	BP	GO:0021879	forebrain neuron differentiation	12/666	43/18614	2.21086091852876e-08	2.50377451948591e-07	1.43547772954196e-07	ASCL1/FEZF2/FOXG1/INHBA/NDNF/NKX2-1/NRP2/PLXNA1/PLXNA3/PROX1/SEMA3E/SOX1	12	2.7906976744186
GO:0007214	BP	GO:0007214	gamma-aminobutyric acid signaling pathway	10/666	28/18614	2.35379893590503e-08	2.65342242430579e-07	1.52127468648425e-07	GABRA3/GABRA4/GABRB1/GABRB3/GABRE/GABRG2/GABRR1/GPR156/PLCL1/SHISA7	10	3.57142857142857
GO:0048843	BP	GO:0048843	negative regulation of axon extension involved in axon guidance	10/666	28/18614	2.35379893590503e-08	2.65342242430579e-07	1.52127468648425e-07	PLXNA3/SEMA3D/SEMA3E/SEMA3F/SEMA3G/SEMA6A/SEMA6D/WNT3/WNT3A/WNT5A	10	3.57142857142857
GO:0089718	BP	GO:0089718	amino acid import across plasma membrane	13/666	52/18614	2.45906109406294e-08	2.76574033805935e-07	1.58566940835999e-07	AGT/ATP1A2/NTSR1/RGS2/SLC17A8/SLC1A1/SLC1A2/SLC22A2/SLC38A3/SLC38A5/SLC6A6/SLC6A9/SLC7A11	13	2.5
GO:2000241	BP	GO:2000241	regulation of reproductive process	26/666	206/18614	2.46600176682342e-08	2.76721431140117e-07	1.58651447483449e-07	APELA/ASPM/AURKA/BCL2L1/CNR1/DUSP1/EDNRB/IGF1/IL1A/INHBA/ITGB3/OXT/OXTR/P2RY1/PTGDR2/SHH/SOX9/STXBP1/SULF1/TACR1/TACR2/TTK/UNC5C/VEGFA/VIP/WNT5A	26	1.2621359223301
GO:0010919	BP	GO:0010919	regulation of inositol phosphate biosynthetic process	8/666	16/18614	2.57129404789041e-08	2.87879504450601e-07	1.65048655226055e-07	ADCYAP1R1/AVPR1B/GPER1/NTSR1/P2RY1/PTH1R/PTK2B/SNCA	8	5
GO:0007623	BP	GO:0007623	circadian rhythm	26/666	207/18614	2.72686695555211e-08	3.03912723050763e-07	1.74240907984557e-07	ADRB1/CARTPT/CDK1/CHRNB2/CSNK1E/DRD2/DRD4/GHRHR/GNA11/GNAQ/HOMER1/HTR7/KCNA2/LEP/MAPK10/MC3R/NGFR/NKX2-1/NLGN1/PRKCG/PROKR1/PROX1/SOX14/TH/TOP2A/TPH1	26	1.256038647343
GO:0044703	BP	GO:0044703	multi-organism reproductive process	26/666	207/18614	2.72686695555211e-08	3.03912723050763e-07	1.74240907984557e-07	ADCYAP1/ADRA2A/AGT/APELA/BCL2/CALCA/CAPN2/CNR1/FOSB/ITGB3/LEP/MMP9/OXT/OXTR/P2RX1/PLA2G4B/PNOC/PRL/PTGFR/PTN/RGS2/RLN2/SLC38A3/TACR1/UCN/VEGFA	26	1.256038647343
GO:0060326	BP	GO:0060326	cell chemotaxis	33/666	312/18614	2.8538721210305e-08	3.1734799716889e-07	1.81943692974486e-07	AGTR1/CALCA/CCL5/CCR2/CCR7/CNR2/CSF1/CTSG/CX3CR1/CXCL12/CXCL13/CXCL8/CXCR5/DUSP1/EDN2/EDN3/EDNRB/F2RL1/FGFR1/GAB1/IL6R/LPAR1/MIF/P2RX4/PIK3CD/PIK3CG/PTK2B/PTN/S1PR1/SCG2/SLIT2/VEGFA/WNT5A	33	1.05769230769231
GO:0007620	BP	GO:0007620	copulation	9/666	22/18614	2.98312804956776e-08	3.30226900081657e-07	1.89327496175745e-07	CNR1/EDNRB/OXT/OXTR/P2RX1/P2RY1/TACR1/VGF/VIP	9	4.09090909090909
GO:2000310	BP	GO:2000310	regulation of NMDA receptor activity	9/666	22/18614	2.98312804956776e-08	3.30226900081657e-07	1.89327496175745e-07	CCR2/EPHB2/MEF2C/NLGN1/OPRM1/PINK1/PTK2B/RELN/SLC6A9	9	4.09090909090909

	ONTOLOGY	ID	Description	GeneRatio	BgRatio	pvalue	p.adjust	qvalue	geneID	Count	richFactor
GO:0097484	BP	GO:0097484	dendrite extension	11/666	36/18614	3.010045158808 2e-08	3.324577967537 6e-07	1.906065260823 08e-07	CACNG7/CPNE5/CPNE9/NEDD4L/PRKN/RASAL1/RIMS1/SH3GL2/SYT2/SYT4/TMEM108	11	3.055555555555 56
GO:0050731	BP	GO:0050731	positive regulation of peptidyl-tyrosine phosphorylation	24/666	181/18614	3.262379096030 89e-08	3.595200281836 73e-07	2.061219929212 81e-07	ADRA1A/ADRA2A/AGT/CCL5/CRLF1/EFNA5/EPHA4/ERBB4/GFRA1/GHR/IGF1/IL6R/IL6ST/ITGB3/LEP/MIF/NOX4/PLCG2/PRNP/PTK2B/RELN/RIPK2/VEGFA/WNT3A	24	1.325966850828 73
GO:0009308	BP	GO:0009308	amine metabolic process	19/666	117/18614	3.329078593846 89e-08	3.660496932607 93e-07	2.098656162893 71e-07	CHRNB2/COMT/DRD2/DRD4/EDNRA/EPAS1/GRIN2A/MAOA/MAOB/NPY/PAH/PRKN/SLC1A1/SLC22A3/SLC6A3/SLC7A11/SNCA/SNCAIP/TH	19	1.623931623931 62
GO:0198738	BP	GO:0198738	cell-cell signaling by wnt	42/666	463/18614	3.422665432504 37e-08	3.755000134097 98e-07	2.152837256300 33e-07	APC/ASPM/ATP6V1C2/AXIN2/CAV1/CCND1/CELSR1/CSNK1A1L/CSNK1E/DCDC2/DKK1/DKK4/DRD2/EDNRA/EDNRB/FZD10/FZD3/FZD9/GNAQ/GPC6/LRRK2/MAGI2/OPRM1/PLCG2/PRKN/SHH/SOX10/SOX2/SOX9/SULF1/WNT1/WNT11/WNT16/WNT2/WNT2B/WNT3/WNT3A/WNT5A/WNT7A/WNT7B/WNT8B/WNT9A	42	0.907127429805 616
GO:0032102	BP	GO:0032102	negative regulation of response to external stimulus	41/666	446/18614	3.452633104530 9e-08	3.779441360527 7e-07	2.166850034188 07e-07	A2M/CARTPT/CLDN19/CNR2/CXCL13/DRD2/DUSP1/EPHA4/F2/F2RL1/GPER1/GRID2/GRIN3A/IGF1/KNG1/LEP/MIF/NPY/NPY5R/PLCG1/PLXNA3/PRKG1/PTGER4/ROBO2/RTN4R/SEMA3D/SEMA3E/SEMA3F/SEMA3G/SEMA6A/SEMA6D/SLC6A3/SLIT2/SPX/SYT11/TNR/TYRO3/UCN/WNT3/WNT3A/WNT5A	41	0.919282511210 762
GO:0003156	BP	GO:0003156	regulation of animal organ formation	10/666	29/18614	3.477970478756 69e-08	3.798716645130 92e-07	2.177901045995 71e-07	DKK1/GDNF/ROBO2/SHH/SULF1/WNT11/WNT2/WNT2B/WNT3A/WNT5A	10	3.448275862068 97
GO:0008037	BP	GO:0008037	cell recognition	22/666	155/18614	3.523121716352 54e-08	3.839499608840 96e-07	2.201282958262 51e-07	CCR7/CNR1/CNTN2/CNTN4/CNTN6/CRTAC1/DSCAML1/EPHA3/EPHA4/EPHB2/EPHB3/FEZF2/FOXG1/GAP43/IGSF9/MYPN/NCAM2/NDN/NEXN/NRCAM/PALLD/ROBO2	22	1.419354838709 68
GO:0007596	BP	GO:0007596	blood coagulation	27/666	224/18614	3.553367333338 08e-08	3.863893903397 49e-07	2.215268828911 51e-07	ADRA2A/ADRA2C/AXL/CAV1/COL3A1/CTSG/EPHB2/F2/F2RL1/F2RL2/GNA13/GNAQ/IL6ST/ITGB3/KNG1/P2RX1/P2RY1/PDPN/PIK3CG/PLCG2/PRKG1/SHH/SLC7A11/STXBP1/TYRO3/VCL/WNT3A	27	1.205357142857 14
GO:0002090	BP	GO:0002090	regulation of receptor internalization	14/666	63/18614	3.630973550840 02e-08	3.939566225690 67e-07	2.258653699453 64e-07	APELA/APLN/ARRB1/DKK1/DRD2/DRD4/GSG1L/HPCA/ITGB3/LPAR1/MAGI2/PLCG2/VEGFA/WNT3A	14	2.222222222222 22
GO:1901890	BP	GO:1901890	positive regulation of cell junction assembly	18/666	106/18614	3.741798352651 72e-08	4.050867599842 12e-07	2.322465613273 51e-07	AGT/BDNF/CAV1/CLDN1/CLDN19/EFNA5/EPHB2/EPHB3/FLRT2/GRID2/NLGN1/NLGN3/NRXN1/OXT/OXTR/SLITRK3/VEGFA/WNT7A	18	1.698113207547 17

	ONTOLOGY	ID	Description	GeneRatio	BgRatio	pvalue	p.adjust	qvalue	geneID	Count	richFactor
GO:0038179	BP	GO:0038179	neurotrophin signaling pathway	11/666	37/18614	4.14595521033974e-08	4.47854282611425e-07	2.56766271788999e-07	AGT/BDNF/DDIT4/GFRA1/MAGI2/NDN/NGF/NTF4/NTRK3/RAP1A/TMEM108	11	2.97297297297297
GO:0032526	BP	GO:0032526	response to retinoic acid	18/666	107/18614	4.35083499359428e-08	4.68955131436752e-07	2.6886392608153e-07	ASCL1/DKK1/EPHA3/FZD10/LEP/NDUFA13/NTRK3/OXT/PTK2B/SOX9/WNT11/WNT2/WNT3/WNT3A/WNT5A/WNT7B/WNT8B/WNT9A	18	1.68224299065421
GO:0030278	BP	GO:0030278	regulation of ossification	19/666	119/18614	4.41639084774315e-08	4.74281903562363e-07	2.7191790027016e-07	ADRB2/BCL2/CALCA/CSF1/DKK1/FZD9/GPM6B/MEF2C/OXT/P2RX7/PTGER4/PTK2B/PTN/S1PR1/SOX9/TACR1/TPH1/TWIST1/WNT5A	19	1.59663865546218
GO:0030595	BP	GO:0030595	leukocyte chemotaxis	28/666	241/18614	4.41955466595243e-08	4.74281903562363e-07	2.7191790027016e-07	CALCA/CCL5/CCR2/CCR7/CNR2/CSF1/CTSG/CX3CR1/CXCL12/CXCL13/CXCL8/CXCR5/DUSP1/EDN2/EDN3/EDNRB/F2RL1/IL6R/MIF/PIK3CD/PIK3CG/PTK2B/PTN/S1PR1/SCG2/SLIT2/VEGFA/WNT5A	28	1.16182572614108
GO:0014065	BP	GO:0014065	phosphatidylinositol 3-kinase signaling	21/666	144/18614	4.45461920218721e-08	4.76567323695548e-07	2.73228189866248e-07	AGT/CCL5/ERBB4/F2/F2RL1/FGFR1/GPER1/HTR2B/IGF1/LEP/NTRK3/PIK3CD/PIK3CG/PIK3R2/PIK3R5/RELN/SEMA3E/SOX9/TWIST1/TYRO3/WNT16	21	1.45833333333333
GO:0030902	BP	GO:0030902	hindbrain development	22/666	157/18614	4.46024351780167e-08	4.76567323695548e-07	2.73228189866248e-07	ASCL1/BCL2/CDK5R2/EPHB2/GRID2/KIF14/LHX1/LPAR1/NEUROD1/NLGN4X/PHOX2B/PLXNA2/POU4F1/PROX1/SHH/SPTBN2/SSTR1/SSTR2/SSTR3/TUBA1A/WNT1/WNT7A	22	1.40127388535032
GO:0045807	BP	GO:0045807	positive regulation of endocytosis	17/666	96/18614	4.70210361507904e-08	5.00506172517878e-07	2.86952941870827e-07	APELA/APLN/ARRB1/AXL/CALY/CLU/DRD2/F2RL1/LRRK2/MAGI2/NEDD4L/NLGN1/PLCG2/SNCA/VEGFA/WNT3A/WNT5A	17	1.77083333333333
GO:0099509	BP	GO:0099509	regulation of presynaptic cytosolic calcium ion concentration	8/666	17/18614	4.704656189283e-08	5.00506172517878e-07	2.86952941870827e-07	CALB1/CALB2/CNR1/NPY/P2RX1/P2RY1/P2RY4/SV2B	8	4.70588235294118
GO:1903861	BP	GO:1903861	positive regulation of dendrite extension	9/666	23/18614	4.74568916996773e-08	5.0378104255705e-07	2.88830508309733e-07	CACNG7/CPNE5/CPNE9/NEDD4L/PRKN/RASAL1/RIMS1/SYT2/SYT4	9	3.91304347826087
GO:0019935	BP	GO:0019935	cyclic-nucleotide-mediated signaling	16/666	85/18614	4.76830414613267e-08	5.05090837893148e-07	2.89581447350208e-07	ADCY2/ADCYAP1/ADCYAP1R1/EDNRB/GAL/GHRHR/GNAI1/GUCY1A1/HTR2B/HTR2C/LPAR1/PRKG1/PTK2B/SCTR/SOX9/UCN	16	1.88235294117647
GO:0001936	BP	GO:0001936	regulation of endothelial cell proliferation	24/666	185/18614	4.99620616653378e-08	5.28093619537926e-07	3.027695281904e-07	AGTR1/AKT3/APELA/APLN/CAV1/CXCL12/FGFR1/HTR2B/ITGA4/ITGB3/LEP/MEF2C/NGFR/NRP2/PIK3CD/PLCG1/PRL/PROX1/SCG2/SULF1/VEGFA/VIP/WNT2/WNT5A	24	1.2972972972973
GO:0010518	BP	GO:0010518	positive regulation of phospholipase activity	13/666	55/18614	5.09062403740728e-08	5.36918822829544e-07	3.07829242108409e-07	ADCYAP1R1/ADRA1A/AGT/AGTR1/AVPR1B/CCL5/EDNRA/FGFR1/GNA13/GNAQ/HPCA/HTR2B/LPAR1	13	2.36363636363636

	ONTOLOGY	ID	Description	GeneRatio	BgRatio	pvalue	p.adjust	qvalue	geneID	Count	richFactor
GO:0050817	BP	GO:0050817	coagulation	27/666	229/18614	5.65587905090849e-08	5.952600757005 4e-07	3.41277768945836e-07	ADRA2A/ADRA2C/AXL/CAV1/COL3A1/CTSG/EPHB2/F2/F2RL1/F2RL2/GNA13/GNAQ/IL6ST/ITGB3/KNG1/P2RX1/P2RY1/PDPN/PIK3CG/PLCG2/PRKG1/SHH/SLC7A11/STXBP1/TYRO3/VCL/WNT3A	27	1.17903930131004
GO:0018108	BP	GO:0018108	peptidyl-tyrosine phosphorylation	36/666	370/18614	5.80244643741037e-08	6.09380859826324e-07	3.49373574290318e-07	ADRA1A/ADRA2A/AGT/AXL/CAV1/CCL5/CRLF1/EFNA5/EPHA3/EPHA4/EPHB2/EPHB3/ERBB4/FGFR1/GFRA1/GHR/IGF1/IL6R/IL6ST/ITGB3/LEP/MAP2K6/MATK/MIF/NOX4/NTRK3/PLCG2/PRNP/PTK2B/RELN/RIPK2/RPS6KA5/TTK/TYRO3/VEGFA/WNT3A	36	0.972972972972973
GO:0022604	BP	GO:0022604	regulation of cell morphogenesis	28/666	245/18614	6.29597179734585e-08	6.59801735265562e-07	3.78281146930644e-07	CACNG7/CAMK2B/CNTN2/CPNE5/CPNE9/EFNA5/EPHA4/EPHB2/F2/GNA13/LPAR1/NEDD4L/NTNG1/P2RY1/PDPN/PLXNA1/PLXNA2/PLXNA3/PRKN/PTK2B/RASAL1/RELN/RIMS1/SEMA3E/SYT2/SYT4/VEGFA/WNT5A	28	1.14285714285714
GO:0042060	BP	GO:0042060	wound healing	40/666	439/18614	6.47208433025628e-08	6.76814776238503e-07	3.8803515682186e-07	ADRA2A/ADRA2C/AXL/CASP7/CAV1/CLDN1/CLDN19/COL3A1/CTSG/DRD5/EPHB2/F2/F2RL1/F2RL2/GNA13/GNAQ/IGF1/IL1A/IL6ST/ITGB3/KNG1/NDNF/OPRM1/P2RX1/P2RY1/PDPN/PIK3CG/PLCG2/PRKG1/SHH/SLC7A11/STXBP1/SYT11/SYT7/TYRO3/VCL/VEGFA/WNT3A/WNT5A/WNT7A	40	0.911161731207289
GO:0048545	BP	GO:0048545	response to steroid hormone	34/666	339/18614	6.52683148587952e-08	6.81027317716522e-07	3.9045031418903e-07	A2M/ATP1A2/AXIN2/BCHE/BCL2/CAV1/CCND1/CLDN1/DDIT4/DNAAF4/FOSB/GABRB1/GHR/GHRHR/GPER1/MAOB/NEFL/NR3C1/NTRK3/OXT/OXTR/SLIT2/SLIT3/SOX10/SST/SSTR2/SSTR3/SSTR5/TACR1/TH/TXNIP/UCN/UCN3/WNT7B	34	1.00294985250737
GO:0044706	BP	GO:0044706	multi-multicellular organism process	26/666	216/18614	6.54007922608745e-08	6.81027317716522e-07	3.9045031418903e-07	ADCYAP1/ADRA2A/AGT/APELA/BCL2/CALCA/CAPN2/CNR1/FOSB/ITGB3/LEP/MMP9/OXT/OXTR/P2RX1/PLA2G4B/PNOC/PRL/PTGFR/PTN/RGS2/RLN2/SLC38A3/TACR1/UCN/VEGFA	26	1.2037037037037
GO:0018212	BP	GO:0018212	peptidyl-tyrosine modification	36/666	372/18614	6.650220698517e-08	6.91032446790931e-07	3.96186509623456e-07	ADRA1A/ADRA2A/AGT/AXL/CAV1/CCL5/CRLF1/EFNA5/EPHA3/EPHA4/EPHB2/EPHB3/ERBB4/FGFR1/GFRA1/GHR/IGF1/IL6R/IL6ST/ITGB3/LEP/MAP2K6/MATK/MIF/NOX4/NTRK3/PLCG2/PRNP/PTK2B/RELN/RIPK2/RPS6KA5/TTK/TYRO3/VEGFA/WNT3A	36	0.967741935483871
GO:0001990	BP	GO:0001990	regulation of systemic arterial blood pressure by hormone	11/666	39/18614	7.61389164583838e-08	7.89499524035773e-07	4.52640194001984e-07	AGT/AGTR1/AVPR1B/CTSG/DRD5/EDN2/EDN3/EDNRB/F2RL1/OXTR/TACR1	11	2.82051282051282

	ONTOLOGY	ID	Description	GeneRatio	BgRatio	pvalue	p.adjust	qvalue	geneID	Count	richFactor
GO:0021536	BP	GO:0021536	diencephalon development	15/666	77/18614	7.757629449647 32e-08	8.027104998950 86e-07	4.602143830848 95e-07	CHRNB2/DRD2/GHRHR/NDNF/NKX2-1/NRP2/PLXNA1/PLXNA3/POU4F1/SEMA3E/SHH/SLC6A3/SOX2/WNT1/WNT5A	15	1.948051948051 95
GO:0001822	BP	GO:0001822	kidney development	32/666	310/18614	8.189448370361 43e-08	8.456121584102 19e-07	4.848109970689 42e-07	AGT/AGTR1/BCL2/CALB1/CRLF1/EDNRA/EDNRB/EPHA4/ERBB4/GDNF/GFRA1/IL6R/ITGB3/KIRREL3/LHX1/LRRK2/MAGI2/MEF2C/MMP9/PROX1/ROBO2/SHH/SLIT2/SOX9/SULF1/TP73/VEGFA/WNT1/WNT11/WNT2B/WNT5A/WNT7B	32	1.032258064516 13
GO:0060193	BP	GO:0060193	positive regulation of lipase activity	14/666	67/18614	8.326769643184 87e-08	8.579889475105 58e-07	4.919069256274 06e-07	ADCYAP1R1/ADRA1A/AGT/AGTR1/AVPR1B/CCL5/EDNRA/FGFR1/GNA13/GNAQ/GPIHBP1/HPCA/HTR2B/LPAR1	14	2.089552238805 97
GO:0016055	BP	GO:0016055	Wnt signaling pathway	41/666	461/18614	8.610383581736 72e-08	8.853563871179 07e-07	5.075973760913 72e-07	APC/ASPM/ATP6V1C2/AXIN2/CAV1/CCND1/CELSR1/CSNK1A1L/CSNK1E/DCDC2/DKK1/DKK4/DRD2/EDNRA/EDNRB/FZD10/FZD3/FZD9/GNAQ/GPC6/LRRK2/MAGI2/PLCG2/PRKN/SHH/SOX10/SOX2/SOX9/SULF1/WNT1/WNT11/WNT16/WNT2/WNT2B/WNT3/WNT3A/WNT5A/WNT7A/WNT7B/WNT8B/WNT9A	41	0.889370932754 881
GO:0055117	BP	GO:0055117	regulation of cardiac muscle contraction	15/666	78/18614	9.291456133690 53e-08	9.533926283317 11e-07	5.466042867792 45e-07	ADRA1A/ADRA1B/ATP1A2/ATP2A1/CACNA1C/CAMK2D/CAV1/FGF13/FKBP1B/KCNQ1/NOS1/P2RX4/PIK3CG/RGS2/UCN	15	1.923076923076 92
GO:0060070	BP	GO:0060070	canonical Wnt signaling pathway	32/666	313/18614	1.024009331707 86e-07	1.048542888613 36e-06	6.011563554784 95e-07	APC/ASPM/AXIN2/CAV1/CSNK1A1L/CSNK1E/DKK1/DKK4/EDNRA/EDNRB/FZD10/FZD3/FZD9/GNAQ/LRRK2/PRKN/SHH/SOX10/SOX2/SOX9/WNT1/WNT11/WNT16/WNT2/WNT2B/WNT3/WNT3A/WNT5A/WNT7A/WNT7B/WNT8B/WNT9A	32	1.022364217252 4
GO:0033604	BP	GO:0033604	negative regulation of catecholamine secretion	7/666	13/18614	1.034611424742 7e-07	1.053465671007 36e-06	6.039787120601 14e-07	ADRA2A/ADRA2C/CNR1/DRD2/P2RY1/SYT11/SYT4	7	5.384615384615 38
GO:0035589	BP	GO:0035589	G protein-coupled purinergic nucleotide receptor signaling pathway	7/666	13/18614	1.034611424742 7e-07	1.053465671007 36e-06	6.039787120601 14e-07	GPR171/P2RY1/P2RY13/P2RY14/P2RY2/P2RY4/P2RY8	7	5.384615384615 38
GO:0006821	BP	GO:0006821	chloride transport	18/666	113/18614	1.035247037836 32e-07	1.053465671007 36e-06	6.039787120601 14e-07	CLCN1/GABRA3/GABRA4/GABRB1/GABRB3/GABRE/GABRG2/GLRA3/GLRB/NMUR2/P2RY4/PRKG2/SLC17A7/SLC17A8/SLC1A1/SLC1A7/SLC4A8/SLC6A1	18	1.592920353982 3
GO:0099054	BP	GO:0099054	presynapse assembly	12/666	49/18614	1.092243097403 56e-07	1.109168352012 09e-06	6.359144784143 84e-07	DKK1/MDGA1/NLGN1/NLGN3/NLGN4X/NLGN4Y/NTRK3/SLITRK3/SNCA/WNT3A/WNT5A/WNT7A	12	2.448979591836 73
GO:0002688	BP	GO:0002688	regulation of leukocyte chemotaxis	19/666	126/18614	1.131582615299 7e-07	1.146748155504 74e-06	6.574599373103 17e-07	CCL5/CCR2/CCR7/CSF1/CX3CR1/CXCL12/CXCL13/CXCL8/DUSP1/EDN2/EDN3/F2RL1/IL6R/MIF/PTK2B/PTN/SLIT2/VEGFA/WNT5A	19	1.507936507936 51

	ONTOLOGY	ID	Description	GeneRatio	BgRatio	pvalue	p.adjust	qvalue	geneID	Count	richFactor
GO:0072006	BP	GO:0072006	nephron development	21/666	152/18614	1.15403921480404e-07	1.16709932937486e-06	6.69127783848907e-07	AGT/BCL2/CALB1/EDNRA/EDNRB/ERBB4/GDNF/IL6R/ITGB3/KIRREL3/LHX1/MAGI2/MEF2C/SHH/SOX9/SULF1/VEGFA/WNT1/WNT11/WNT2B/WNT7B	21	1.38157894736842
GO:0070372	BP	GO:0070372	regulation of ERK1 and ERK2 cascade	32/666	315/18614	1.18635550728229e-07	1.19731772449537e-06	6.86452759752444e-07	ADCYAP1/ADRA1A/APELA/ARRB1/CCL5/CCR7/CHRNA7/DRD2/EPHA4/EPHB2/ERBB4/F2RL1/GCG/GPER1/HTR2B/HTR2C/IGF1/IL1A/INHBA/ITGB3/MIF/NOX4/NPSR1/NPY/NPY5R/OPRM1/P2RY1/PTK2B/RAP1A/RIPK2/RPS6KA6/SEMA6A	32	1.01587301587302
GO:0007389	BP	GO:0007389	pattern specification process	41/666	467/18614	1.22433535908036e-07	1.23311645284426e-06	7.06977082885706e-07	ADGRG1/APC/ASCL1/AURKA/AXIN2/CHRDL1/DKK1/DNAAF4/DNAH5/DSCAML1/EMX1/ERBB4/FEZF1/FEZF2/FOXG1/GDNF/LHX1/MEF2C/NEUROD1/NKX2-1/NRG3/NRP2/NTF4/PLXNA2/PRKACB/RELN/ROBO2/SEMA3F/SHH/SOX1/TBX20/WNT1/WNT11/WNT2/WNT2B/WNT3/WNT3A/WNT5A/WNT7A/WNT7B/WNT8B	41	0.877944325481799
GO:0008347	BP	GO:0008347	glial cell migration	13/666	59/18614	1.24452704269321e-07	1.25088965538591e-06	7.17166912773592e-07	ADGRG1/CCR2/CDK5R2/COL3A1/CSF1/CX3CR1/EPHA4/FOXG1/NDN/NTN1/P2RX4/P2RY1/RELN	13	2.20338983050847
GO:0090596	BP	GO:0090596	sensory organ morphogenesis	29/666	269/18614	1.31314542132274e-07	1.31716525424515e-06	7.5516440233748e-07	ATOH1/BCL2/CALB1/EDNRA/EPHB2/FOXG1/FZD3/GNGT1/HCN1/KCNQ1/LHX1/NTN1/OLFM3/PROX1/SLC1A1/SLC44A4/SOX1/SOX9/TH/THRB/TWIST1/VEGFA/WNT1/WNT16/WNT2/WNT2B/WNT3A/WNT5A/WNT9A	29	1.07806691449814
GO:0001938	BP	GO:0001938	positive regulation of endothelial cell proliferation	18/666	115/18614	1.36338406667644e-07	1.36477244148976e-06	7.82458815841533e-07	AGTR1/AKT3/APELA/APLN/CXCL12/FGFR1/HTR2B/ITGA4/ITGB3/NRP2/PIK3CD/PLCG1/PROX1/SCG2/VEGFA/VIP/WNT2/WNT5A	18	1.56521739130435
GO:0048771	BP	GO:0048771	tissue remodeling	23/666	181/18614	1.39064756263723e-07	1.3892343029191e-06	7.96483424300353e-07	ADRB2/AGT/AXL/CALCA/CARTPT/CAV1/CCR2/EDNRA/EPAS1/IL1A/ITGB3/LEP/LEPR/MEF2C/NOX4/P2RX7/PTH1R/PTK2B/PTN/S1PR1/SYT7/TPH1/WNT16	23	1.2707182320442
GO:1904861	BP	GO:1904861	excitatory synapse assembly	10/666	33/18614	1.4117504569559e-07	1.40745507017003e-06	8.06929854440256e-07	GRID2/NLGN1/NPTX1/NRXN1/NRXN2/NTRK3/PTK2B/SLITRK3/WNT5A/WNT7A	10	3.03030303030303
GO:0002274	BP	GO:0002274	myeloid leukocyte activation	27/666	240/18614	1.4955821995541e-07	1.4880134637264e-06	8.53116034137298e-07	CAMK4/CCL5/CCR2/CLU/CNR1/CNR2/CSF1/CTSG/CX3CR1/CXCL8/EDN2/F2RL1/GRP/LRRK2/MAPT/MIF/NPY/PIK3CD/PIK3CG/PLCG2/PTGDR/SLC18A2/SNCA/STXBP1/SYT11/VAMP2/WNT5A	27	1.125
GO:0002028	BP	GO:0002028	regulation of sodium ion transport	16/666	92/18614	1.5140967891267e-07	1.50339105425409e-06	8.61932398616095e-07	ADRB2/ANK3/ATP1A2/CAMK2D/DRD2/DRD4/FGF12/FGF13/GRP/KCNQ1/NEDD4L/NOS1/P2RX4/P2RX7/SCN1B/SCN2B	16	1.73913043478261

	ONTOLOGY	ID	Description	GeneRatio	BgRatio	pvalue	p.adjust	qvalue	geneID	Count	richFactor
GO:0046579	BP	GO:0046579	positive regulation of Ras protein signal transduction	13/666	60/18614	1.53730126317438e-07	1.52335397348832e-06	8.7337764887899e-07	ADGRG1/ARRB1/COL3A1/CSF1/F2RL1/F2RL2/IGF1/LPAR1/NGF/P2RY10/P2RY8/PIK3CG/RTN4R	13	2.16666666666667
GO:0001654	BP	GO:0001654	eye development	36/666	385/18614	1.56993124837634e-07	1.55255776373636e-06	8.90120925956468e-07	BCL2/CACNA1C/CALB1/CHRDL1/DRD2/EPHB2/GNGT1/GPM6A/HCN1/HPCA/INHBA/LHX1/NEUROD1/OLFM3/PROX1/SHH/SLC17A7/SLC17A8/SLC1A1/SLC6A3/SLC7A11/SOX1/SOX2/SOX9/TH/THRB/TWIST1/VAX1/VEGFA/WNT16/WNT2/WNT2B/WNT5A/WNT7A/WNT7B/WNT9A	36	0.935064935064935
GO:0007274	BP	GO:0007274	neuromuscular synaptic transmission	9/666	26/18614	1.64767954407302e-07	1.62291482146671e-06	9.30458419888932e-07	CHAT/CHRNA1/CHRNA3/CHRNB2/CHRNB4/NLGN1/P2RX2/SLC5A7/STXBP1	9	3.46153846153846
GO:1903859	BP	GO:1903859	regulation of dendrite extension	9/666	26/18614	1.64767954407302e-07	1.62291482146671e-06	9.30458419888932e-07	CACNG7/CPNE5/CPNE9/NEDD4L/PRKN/RASAL1/RIMS1/SYT2/SYT4	9	3.46153846153846
GO:0046683	BP	GO:0046683	response to organophosphorus	19/666	129/18614	1.6576800191441e-07	1.62949945881865e-06	9.34233560262895e-07	FOSB/HCN1/KCNQ1/LPAR1/OXT/P2RX1/P2RX2/P2RX4/P2RX7/P2RY1/P2RY2/P2RY4/PENK/PIK3CG/PTK2B/RAP1A/RYR3/SLC6A3/VGF	19	1.47286821705426
GO:0007568	BP	GO:0007568	aging	22/666	169/18614	1.69049590196691e-07	1.65844059045257e-06	9.50826248464215e-07	ADRA1A/ADRB3/AGT/AVPR1B/CALCA/CLDN1/CNR1/EDNRA/EDNRB/FOXG1/GFRA1/GNA11/GNA13/INHBA/IRAK1/NPY/NPY5R/P2RY1/PENK/SLC1A2/SLC6A3/TH	22	1.30177514792899
GO:0072001	BP	GO:0072001	renal system development	32/666	320/18614	1.70330859514864e-07	1.66768162254095e-06	9.56124367626948e-07	AGT/AGTR1/BCL2/CALB1/CRLF1/EDNRA/EDNRB/EPHA4/ERBB4/GDNF/GFRA1/IL6R/ITGB3/KIRREL3/LHX1/LRRK2/MAGI2/MEF2C/MMP9/PROX1/ROBO2/SHH/SLIT2/SOX9/SULF1/TP73/VEGFA/WNT1/WNT11/WNT2B/WNT5A/WNT7B	32	1
GO:1904427	BP	GO:1904427	positive regulation of calcium ion transmembrane transport	16/666	93/18614	1.76895068380578e-07	1.72850747731718e-06	9.90997380045639e-07	ADCYAP1R1/ATP2A1/BDKRB1/CAV1/F2/GPER1/NPSR1/NTSR1/P2RX1/P2RX2/P2RX4/P2RX7/PLCG1/PLCG2/SNCA/TRPC1	16	1.72043010752688
GO:0002685	BP	GO:0002685	regulation of leukocyte migration	26/666	227/18614	1.77835119277613e-07	1.73424526041561e-06	9.94286997297764e-07	BDKRB1/CCL5/CCR2/CCR7/CSF1/CX3CR1/CXCL12/CXCL13/CXCL8/DUSP1/EDN2/EDN3/F2RL1/IL1A/IL6R/ITGA4/ITGB3/MIF/P2RX4/PTGER4/PTK2B/PTN/SLIT2/TACR1/VEGFA/WNT5A	26	1.14537444933921
GO:0051279	BP	GO:0051279	regulation of release of sequestered calcium ion into cytosol	15/666	82/18614	1.85698342941269e-07	1.80734129813136e-06	1.03619481824654e-06	ATP1A2/BDKRB1/CACNA1C/CAMK2D/F2/FKBP1B/GPER1/ITGB3/NOS1/NPSR1/NTSR1/PLCG1/PTK2B/SNCA/TRPC1	15	1.82926829268293
GO:0014074	BP	GO:0014074	response to purine-containing compound	20/666	143/18614	1.87997866331838e-07	1.82610575695846e-06	1.04695296062062e-06	FOSB/HCN1/KCNQ1/OXT/P2RX1/P2RX2/P2RX4/P2RX7/P2RY1/P2RY2/P2RY4/PENK/PIK3CG/PTK2B/RAP1A/RYR1/RYR3/SLC6A1/SLC6A3/VGF	20	1.3986013986014
GO:0042136	BP	GO:0042136	neurotransmitter biosynthetic process	7/666	14/18614	2.00527811262594e-07	1.93633436612112e-06	1.11014983093778e-06	CHAT/GAD1/NOS1/PAH/SLC44A4/SLC5A7/TH	7	5

	ONTOLOGY	ID	Description	GeneRatio	BgRatio	pvalue	p.adjust	qvalue	geneID	Count	richFactor
GO:0045956	BP	GO:0045956	positive regulation of calcium ion-dependent exocytosis	7/666	14/18614	2.00527811262594e-07	1.93633436612112e-06	1.11014983093778e-06	CDK5R2/STX1A/STXBP1/SYT10/SYT4/SYT7/SYT9	7	5
GO:0060732	BP	GO:0060732	positive regulation of inositol phosphate biosynthetic process	7/666	14/18614	2.00527811262594e-07	1.93633436612112e-06	1.11014983093778e-06	ADCYAP1R1/AVPR1B/GPER1/NTSR1/P2RY1/PTH1R/SNCA	7	5
GO:0010976	BP	GO:0010976	positive regulation of neuron projection development	21/666	157/18614	2.02124898400993e-07	1.9479291679233e-06	1.11679742625275e-06	AGT/BDNF/CAMK2B/CNR1/EPHA3/EPHB2/FKBP1B/LRRC7/MAGI2/NDNF/NLGN1/NTRK3/PTK2B/PTN/RAP1A/RELN/RGS2/SCN1B/TUBB2B/VEGFA/WNT5A	21	1.33757961783439
GO:0150063	BP	GO:0150063	visual system development	36/666	389/18614	2.0260563062166e-07	1.94874104599894e-06	1.11726289664061e-06	BCL2/CACNA1C/CALB1/CHRDL1/DRD2/EPHB2/GNGT1/GPM6A/HCN1/HPCA/INHBA/LHX1/NEUROD1/OLFM3/PROX1/SHH/SLC17A7/SLC17A8/SLC1A1/SLC6A3/SLC7A11/SOX1/SOX2/SOX9/TH/THRB/TWIST1/VAX1/VEGFA/WNT16/WNT2/WNT2B/WNT5A/WNT7A/WNT7B/WNT9A	36	0.925449871465296
GO:0048732	BP	GO:0048732	gland development	39/666	441/18614	2.06870668147496e-07	1.98587760536122e-06	1.1385542323825e-06	APLN/ASCL1/AURKA/BCL2/CAV1/CCND1/CGA/CLDN1/CSF1/DRD2/EDNRA/EPHB3/ERBB4/GHRHR/NKX2-1/NR3C1/NRG3/NTN1/OXTR/PLXNA1/PRL/PROX1/PTN/SHH/SLC29A1/SLC6A3/SOX10/SOX2/SOX9/SULF1/TPH1/VEGFA/WNT1/WNT11/WNT2/WNT3/WNT3A/WNT5A/WNT7B	39	0.884353741496599
GO:0045665	BP	GO:0045665	negative regulation of neuron differentiation	14/666	72/18614	2.15537138965064e-07	2.06503906045476e-06	1.18393951166405e-06	ASCL1/ASPM/CNTN2/CNTN4/FEZF2/FOXG1/MAG/PHOX2B/SHH/SOX2/SOX9/TLX3/TP73/WNT3A	14	1.94444444444444
GO:0001935	BP	GO:0001935	endothelial cell proliferation	24/666	201/18614	2.43153932741764e-07	2.32510034907738e-06	1.33303927493283e-06	AGTR1/AKT3/APELA/APLN/CAV1/CXCL12/FGFR1/HTR2B/ITGA4/ITGB3/LEP/MEF2C/NGFR/NRP2/PIK3CD/PLCG1/PRL/PROX1/SCG2/SULF1/VEGFA/VIP/WNT2/WNT5A	24	1.19402985074627
GO:1901653	BP	GO:1901653	cellular response to peptide	35/666	375/18614	2.44259302997815e-07	2.33113490142575e-06	1.33649903755779e-06	ADRB2/AGT/AGTR1/APC/CACNA1A/CAMK2A/CAV1/CYSLTR2/EDNRA/EPHA4/EPHB2/GAB1/GCGR/GDF15/GHR/GHRHR/GLP2R/GPER1/IGF1/INHBA/ITGA4/ITGB3/LEP/MAP1B/NGFR/NKX6-1/PIK3R2/PRKCB/PRNP/RIPK2/SLC2A4/SOS2/TSHR/VAMP2/WNT1	35	0.933333333333333
GO:0040013	BP	GO:0040013	negative regulation of locomotion	38/666	427/18614	2.53091371209675e-07	2.4107443594875e-06	1.38214116835638e-06	ADGRG1/ADORA3/BCL2/CLDN19/COL3A1/CX3CR1/CXCL12/CXCL13/DRD2/DUSP1/EPHA4/GNA13/MAGI2/MEF2C/MIF/NEXMIF/NGFR/NKX2-1/NRG3/PLXNA3/PRKG1/PTGER4/ROBO2/SEMA3D/SEMA3E/SEMA3F/SEMA3G/SEMA6A/SEMA6D/SHH/SLIT2/SLURP1/SULF1/VCL/WNT11/WNT3/WNT3A/WNT5A	38	0.889929742388759

	ONTOLOGY	ID	Description	GeneRatio	BgRatio	pvalue	p.adjust	qvalue	geneID	Count	richFactor
GO:0050900	BP	GO:0050900	leukocyte migration	36/666	393/18614	2.60378822967402e-07	2.47536153749474e-06	1.41918784298836e-06	BDKRB1/CALCA/CCL5/CCR2/CCR7/CNR2/CSF1/CTSG/CX3CR1/CXCL12/CXCL13/CXCL8/CXCR5/DUSP1/EDN2/EDN3/EDNRB/F2RL1/IL1A/IL6R/ITGA4/ITGB3/LEP/MIF/P2RX4/PIK3CD/PIK3CG/PTGER4/PTK2B/PTN/S1PR1/SCG2/SLIT2/TACR1/VEGFA/WNT5A	36	0.916030534351145
GO:0072073	BP	GO:0072073	kidney epithelium development	20/666	146/18614	2.65422581980916e-07	2.51844013597722e-06	1.44388590116422e-06	AGT/BCL2/CALB1/CRLF1/EDNRA/EDNRB/EPHA4/GDNF/LHX1/MAGI2/MEF2C/ROBO2/SHH/SLIT2/SOX9/VEGFA/WNT1/WNT11/WNT2B/WNT7B	20	1.36986301369863
GO:0045744	BP	GO:0045744	negative regulation of G protein-coupled receptor signaling pathway	12/666	53/18614	2.77019263488423e-07	2.61836476931846e-06	1.50117523959212e-06	ADRB2/APELA/APLN/ARRB1/BECN2/CCL5/CXCL8/DRD2/HTR2B/OPRM1/RGS2/SNCA	12	2.26415094339623
GO:0099172	BP	GO:0099172	presynapse organization	12/666	53/18614	2.77019263488423e-07	2.61836476931846e-06	1.50117523959212e-06	DKK1/MDGA1/NLGN1/NLGN3/NLGN4X/NLGN4Y/NTRK3/SLITRK3/SNCA/WNT3A/WNT5A/WNT7A	12	2.26415094339623
GO:0002690	BP	GO:0002690	positive regulation of leukocyte chemotaxis	16/666	96/18614	2.78449068076087e-07	2.61890479484093e-06	1.50148484998429e-06	CCL5/CCR2/CCR7/CSF1/CX3CR1/CXCL12/CXCL13/CXCL8/EDN2/EDN3/F2RL1/IL6R/PTK2B/PTN/VEGFA/WNT5A	16	1.66666666666667
GO:0061138	BP	GO:0061138	morphogenesis of a branching epithelium	23/666	188/18614	2.78485264020967e-07	2.61890479484093e-06	1.50148484998429e-06	AGT/BCL2/CELSR1/CSF1/EDNRA/GDNF/GNA13/LHX1/NKX2-1/PLXNA1/PROX1/SEMA3E/SHH/SLIT2/SOX10/SOX9/SULF1/TBX20/VEGFA/WNT1/WNT2/WNT2B/WNT5A	23	1.22340425531915
GO:0043266	BP	GO:0043266	regulation of potassium ion transport	17/666	108/18614	2.7867491509701e-07	2.61890479484093e-06	1.50148484998429e-06	ADRA2A/ANK3/CACNA1D/CAV1/DRD2/EDN3/GAL/GRP/HCRT/KCNA1/KCNQ1/NEDD4L/NOS1/PRNP/PTK2B/VAMP2/VIP	17	1.57407407407407
GO:0051965	BP	GO:0051965	positive regulation of synapse assembly	13/666	63/18614	2.82183472898484e-07	2.64681635361841e-06	1.51748725783295e-06	BDNF/EFNA5/EPHB2/EPHB3/FLRT2/GRID2/NLGN1/NLGN3/NRXN1/OXT/OXTR/SLITRK3/WNT7A	13	2.06349206349206
GO:0001503	BP	GO:0001503	ossification	38/666	429/18614	2.84856347796556e-07	2.66679799889538e-06	1.52894324421329e-06	ADRB2/ATP6V0A4/AXIN2/BCL2/CALCA/CHRDL1/CSF1/DKK1/FZD9/GPM6B/IGF1/IGSF10/IL6R/IL6ST/LEP/MAP2K6/MEF2C/MMP13/OXT/P2RX7/PENK/PTGER4/PTH1R/PTK2B/PTN/RYR1/S1PR1/SHH/SOX2/SOX9/TACR1/TPH1/TWIST1/WNT11/WNT3/WNT3A/WNT5A/WNT7B	38	0.885780885780886
GO:0006820	BP	GO:0006820	monoatomic anion transport	20/666	147/18614	2.97112803892738e-07	2.77625367135514e-06	1.59169697022385e-06	CLCN1/GABRA3/GABRA4/GABRB1/GABRB3/GABRE/GABRG2/GLRA3/GLRB/KCNQ1/NMUR2/P2RY4/PRKG2/PRNP/SLC17A7/SLC17A8/SLC1A1/SLC1A7/SLC4A8/SLC6A1	20	1.36054421768708

	ONTOLOGY	ID	Description	GeneRatio	BgRatio	pvalue	p.adjust	qvalue	geneID	Count	richFactor
GO:0009743	BP	GO:0009743	response to carbohydrate	26/666	234/18614	3.24171636744569e-07	3.02334647931604e-06	1.73336157308541e-06	ADCY5/ADRA2A/EFNA5/FKBP1B/GCG/GHRHR/GPER1/GRIK5/IL1A/LEP/MAP1B/MAP2K6/NEUROD1/NKX6-1/NOX4/NPTX1/OXT/P2RX2/PRKCB/PTK2B/SLC29A1/SLC6A1/TH/TXNIP/UCN3/VAMP2	26	1.11111111111111
GO:0014072	BP	GO:0014072	response to isoquinoline alkaloid	9/666	28/18614	3.41557050172459e-07	3.15555056691285e-06	1.80915754513588e-06	CNR1/DRD2/FOSB/GHR/OPRM1/PENK/PRKCG/SLC1A1/TACR1	9	3.21428571428571
GO:0032958	BP	GO:0032958	inositol phosphate biosynthetic process	9/666	28/18614	3.41557050172459e-07	3.15555056691285e-06	1.80915754513588e-06	ADCYAP1R1/AVPR1B/GPER1/NTSR1/P2RY1/PLCG2/PTH1R/PTK2B/SNCA	9	3.21428571428571
GO:0043278	BP	GO:0043278	response to morphine	9/666	28/18614	3.41557050172459e-07	3.15555056691285e-06	1.80915754513588e-06	CNR1/DRD2/FOSB/GHR/OPRM1/PENK/PRKCG/SLC1A1/TACR1	9	3.21428571428571
GO:0060402	BP	GO:0060402	calcium ion transport into cytosol	9/666	28/18614	3.41557050172459e-07	3.15555056691285e-06	1.80915754513588e-06	ADCYAP1R1/ADRA1A/BCL2/CACNA1C/CAV1/NOS1/P2RX2/P2RX4/P2RX7	9	3.21428571428571
GO:0098698	BP	GO:0098698	postsynaptic specialization assembly	9/666	28/18614	3.41557050172459e-07	3.15555056691285e-06	1.80915754513588e-06	GAP43/GRID2/NPTX1/NRXN1/NRXN2/NTRK3/PTK2B/SLITRK3/SPTBN2	9	3.21428571428571
GO:0007213	BP	GO:0007213	G protein-coupled acetylcholine receptor signaling pathway	8/666	21/18614	3.46751361254635e-07	3.17963235180323e-06	1.82296424603036e-06	CHRM2/CHRM4/CHRM5/GNA11/GNAQ/ITPR1/OPRM1/PRKCB	8	3.80952380952381
GO:0031280	BP	GO:0031280	negative regulation of cyclase activity	8/666	21/18614	3.46751361254635e-07	3.17963235180323e-06	1.82296424603036e-06	CCR2/EDNRB/GALR1/GALR3/GRM2/HPCA/LTB4R2/P2RY13	8	3.80952380952381
GO:0032305	BP	GO:0032305	positive regulation of icosanoid secretion	8/666	21/18614	3.46751361254635e-07	3.17963235180323e-06	1.82296424603036e-06	AVPR1B/IL1A/MAP2K6/MIF/NTSR1/OXT/P2RX4/P2RX7	8	3.80952380952381
GO:0098962	BP	GO:0098962	regulation of postsynaptic neurotransmitter receptor activity	8/666	21/18614	3.46751361254635e-07	3.17963235180323e-06	1.82296424603036e-06	CACNG4/CACNG5/DLGAP2/HOMER1/NPTX1/NPTX2/NRXN2/SHISA7	8	3.80952380952381
GO:0021766	BP	GO:0021766	hippocampus development	15/666	86/18614	3.55481801162057e-07	3.25361834769369e-06	1.86538230267731e-06	CDK5R2/FEZF2/FGF13/KCNA1/KIF14/KIRREL3/NEFL/NEUROD1/NKX2-1/PLXNA3/PROX1/RELN/TMEM108/TUBA1A/WNT3A	15	1.74418604651163
GO:0032095	BP	GO:0032095	regulation of response to food	7/666	15/18614	3.6437608724538e-07	3.3288261502064e-06	1.90850085219308e-06	CARTPT/LEP/NPY/OPRM1/PRKCG/SPX/UCN	7	4.66666666666667
GO:0120162	BP	GO:0120162	positive regulation of cold-induced thermogenesis	16/666	98/18614	3.72905412608979e-07	3.39412982032061e-06	1.94594111024218e-06	ADCYAP1/ADRB1/ADRB2/ADRB3/APC/CAV1/CCR2/EPAS1/HCRT/LEP/LEPR/OXT/OXTR/PHOX2B/TSHR/VEGFA	16	1.63265306122449
GO:1902476	BP	GO:1902476	chloride transmembrane transport	16/666	98/18614	3.72905412608979e-07	3.39412982032061e-06	1.94594111024218e-06	CLCN1/GABRA3/GABRA4/GABRB1/GABRB3/GABRE/GABRG2/GLRA3/GLRB/NMUR2/SLC17A7/SLC17A8/SLC1A1/SLC1A7/SLC4A8/SLC6A1	16	1.63265306122449
GO:0048483	BP	GO:0048483	autonomic nervous system development	11/666	45/18614	3.79185690499421e-07	3.44491251165371e-06	1.97505612115371e-06	ASCL1/EDNRA/EDNRB/FZD3/GDNF/GFRA3/NRP2/PHOX2B/PLXNA3/SEMA3F/SOX10	11	2.44444444444444
GO:0070472	BP	GO:0070472	regulation of uterine smooth muscle contraction	6/666	10/18614	3.81052814189086e-07	3.44912445992515e-06	1.97747093842044e-06	ADRA2A/GPER1/OXT/OXTR/TACR1/TACR2	6	6
GO:1903365	BP	GO:1903365	regulation of fear response	6/666	10/18614	3.81052814189086e-07	3.44912445992515e-06	1.97747093842044e-06	EPHB2/GRP/GRPR/MEF2C/PENK/UCN	6	6

	ONTOLOGY	ID	Description	GeneRatio	BgRatio	pvalue	p.adjust	qvalue	geneID	Count	richFactor
GO:0043583	BP	GO:0043583	ear development	25/666	221/18614	3.87929483114618e-07	3.50491435571387e-06	2.00945670723265e-06	ATOH1/BCL2/CALB1/EDNRA/EPHA4/EPHB2/FOXG1/FZD3/HPCA/KCNQ1/MCM2/NEUROD1/NTN1/NTRK3/PHOX2B/PROX1/SHH/SLC17A8/SLC44A4/SOX2/SOX9/TWIST1/WNT1/WNT3A/WNT5A	25	1.13122171945701
GO:2000243	BP	GO:2000243	positive regulation of reproductive process	15/666	87/18614	4.15547736070836e-07	3.74755435373974e-06	2.14856840069846e-06	APELA/AURKA/EDNRB/INHBA/OXT/OXTR/P2RY1/SHH/SOX9/TACR1/TACR2/UNC5C/VEGFA/VIP/WNT5A	15	1.72413793103448
GO:0051341	BP	GO:0051341	regulation of oxidoreductase activity	17/666	111/18614	4.17327351195244e-07	3.75671049656524e-06	2.15381785068376e-06	AGT/AGTR1/CAV1/CNR1/CNR2/DRD5/EDN2/EDN3/GDNF/GZMA/HTR2B/IL1A/LEP/LRRK2/PRKN/PTK2B/SNCA	17	1.53153153153153
GO:0010092	BP	GO:0010092	specification of animal organ identity	10/666	37/18614	4.66729345413742e-07	4.19373808539039e-06	2.40437690305511e-06	AXIN2/DKK1/GDNF/MEF2C/ROBO2/WNT11/WNT2/WNT2B/WNT3A/WNT5A	10	2.7027027027027
GO:0002052	BP	GO:0002052	positive regulation of neuroblast proliferation	9/666	29/18614	4.79606855595659e-07	4.30158338549756e-06	2.46620736156277e-06	ASPM/CX3CR1/DRD2/FOXG1/FZD3/SHH/SOX10/VEGFA/WDR62	9	3.10344827586207
GO:0003254	BP	GO:0003254	regulation of membrane depolarization	11/666	46/18614	4.82384406046869e-07	4.31076246494611e-06	2.4714699616985e-06	ANK3/BCL2/CAMK2D/FZD9/HCN1/LRRK2/NEDD4L/NTSR1/P2RX7/SCN1B/SCN2B	11	2.39130434782609
GO:1990090	BP	GO:1990090	cellular response to nerve growth factor stimulus	11/666	46/18614	4.82384406046869e-07	4.31076246494611e-06	2.4714699616985e-06	BDNF/CALCA/CSNK1E/MAGI2/MAPT/NGF/NTF4/NTRK3/RAP1A/SH3GL2/TMEM108	11	2.39130434782609
GO:0046849	BP	GO:0046849	bone remodeling	15/666	88/18614	4.84617803200814e-07	4.32286116648276e-06	2.47840645557089e-06	ADRB2/CALCA/CARTPT/ITGB3/LEP/LEPR/NOX4/P2RX7/PTH1R/PTK2B/PTN/S1PR1/SYT7/TPH1/WNT16	15	1.70454545454545
GO:0003002	BP	GO:0003002	regionalization	37/666	422/18614	5.25237216786048e-07	4.6767045661294e-06	2.68127389270834e-06	ADGRG1/ASCL1/AURKA/AXIN2/DKK1/DNAAF4/DNAH5/DSCAML1/EMX1/FEZF1/FEZF2/FOXG1/GDNF/LHX1/MEF2C/NEUROD1/NKX2-1/NRP2/NTF4/PLXNA2/PRKACB/RELN/ROBO2/SEMA3F/SHH/SOX1/TBX20/WNT1/WNT11/WNT2/WNT2B/WNT3/WNT3A/WNT5A/WNT7A/WNT7B/WNT8B	37	0.876777251184834
GO:0032303	BP	GO:0032303	regulation of icosanoid secretion	8/666	22/18614	5.27848550259757e-07	4.68298849192546e-06	2.68487662748502e-06	AVPR1B/IL1A/MAP2K6/MIF/NTSR1/OXT/P2RX4/P2RX7	8	3.63636363636364
GO:0048485	BP	GO:0048485	sympathetic nervous system development	8/666	22/18614	5.27848550259757e-07	4.68298849192546e-06	2.68487662748502e-06	ASCL1/EDNRA/FZD3/GDNF/GFRA3/NRP2/PHOX2B/SEMA3F	8	3.63636363636364
GO:0007589	BP	GO:0007589	body fluid secretion	15/666	89/18614	5.63868889034499e-07	4.99354160289111e-06	2.86292463906184e-06	APLN/CAV1/CCND1/ERBB4/GHRHR/OXTR/P2RY2/PRL/SLC22A2/SLC29A1/SLC6A3/TACR1/UCN/VEGFA/VIP	15	1.68539325842697
GO:0006813	BP	GO:0006813	potassium ion transport	26/666	241/18614	5.75748981096215e-07	5.08957957210054e-06	2.91798565390869e-06	ADRA2A/ANK3/ATP1A2/CACNA1D/CAV1/DRD2/EDN3/GAL/GRP/HCN1/HCRT/KCNA1/KCNA2/KCNJ14/KCNJ5/KCNJ9/KCNMB2/KCNQ1/KCNQ5/NEDD4L/NOS1/PRNP/PTK2B/SLC17A7/VAMP2/VIP	26	1.07883817427386

	ONTOLOGY	ID	Description	GeneRatio	BgRatio	pvalue	p.adjust	qvalue	geneID	Count	richFactor
GO:0048813	BP	GO:0048813	dendrite morphogenesis	19/666	140/18614	6.09557283640789e-07	5.37876849029529e-06	3.08378502939883e-06	ARHGAP44/CAMK2B/CHRNA3/CHRNA7/CHRNB2/DCDC2/DCLK1/EPHA4/EPHB2/EPHB3/KIF1A/LRRK2/MAP2/NEDD4L/NLGN1/NSMF/PTN/RELN/WNT7A	19	1.35714285714286
GO:0003014	BP	GO:0003014	renal system process	18/666	127/18614	6.26954657360882e-07	5.52236942818769e-06	3.16611510612164e-06	ADRA1A/AGT/AGTR1/ATP6V0A4/BCL2/CHRNA3/CHRNB2/CHRNB4/DRD2/EDNRA/EDNRB/F2RL1/KCNQ1/OXT/PRKACB/SPX/SULF1/TACR1	18	1.41732283464567
GO:2001257	BP	GO:2001257	regulation of cation channel activity	20/666	154/18614	6.35719488568746e-07	5.58955507390946e-06	3.20463434874029e-06	ANK3/CAMK2D/CAV1/DRD4/EDNRA/FGF12/FGF13/FKBP1B/GAL/GRP/HCN1/HOMER1/HPCA/KCNA1/KCNQ1/MMP9/NOS1/NTSR1/PLCG2/VAMP2	20	1.2987012987013
GO:0022037	BP	GO:0022037	metencephalon development	17/666	115/18614	6.99171984564593e-07	6.12957193913233e-06	3.51423977749258e-06	ASCL1/BCL2/CDK5R2/GRID2/KIF14/LHX1/LPAR1/NEUROD1/NLGN4X/PROX1/SPTBN2/SSTR1/SSTR2/SSTR3/TUBA1A/WNT1/WNT7A	17	1.47826086956522
GO:0043434	BP	GO:0043434	response to peptide hormone	37/666	427/18614	6.99631710651727e-07	6.12957193913233e-06	3.51423977749258e-06	AGT/AGTR1/APC/CAMK2A/CAV1/COL3A1/CXCL12/CYSLTR2/EDNRA/EDNRB/GAB1/GAL/GCGR/GDF15/GHR/GHRHR/GLP2R/GNAI1/GPER1/INHBA/ITGB3/KCNQ1/LEP/MAP1B/NEFL/NKX6-1/OXT/OXTR/PIK3R2/PRKCB/SLC2A4/SOS2/TH/TSHR/VAMP2/VGF/WNT1	37	0.866510538641686
GO:0086001	BP	GO:0086001	cardiac muscle cell action potential	14/666	79/18614	7.12256301616232e-07	6.21801016419854e-06	3.56494366537656e-06	ANK3/ATP1A2/CACNA1C/CACNA1D/CAMK2D/CAV1/FGF12/FGF13/HCN1/KCNJ5/KCNQ1/NEDD4L/SCN1B/SCN2B	14	1.77215189873418
GO:1902475	BP	GO:1902475	L-alpha-amino acid transmembrane transport	14/666	79/18614	7.12256301616232e-07	6.21801016419854e-06	3.56494366537656e-06	AGT/ATP1A2/NTSR1/RGS2/SLC17A7/SLC17A8/SLC1A1/SLC1A2/SLC1A7/SLC22A2/SLC38A3/SLC38A5/SLC6A9/SLC7A11	14	1.77215189873418
GO:0048645	BP	GO:0048645	animal organ formation	13/666	68/18614	7.17338553937794e-07	6.24021060637922e-06	3.57767174455794e-06	AXIN2/DKK1/GDNF/MEF2C/NTF4/ROBO2/SHH/SULF1/WNT11/WNT2/WNT2B/WNT3A/WNT5A	13	1.91176470588235
GO:0048663	BP	GO:0048663	neuron fate commitment	13/666	68/18614	7.17338553937794e-07	6.24021060637922e-06	3.57767174455794e-06	ASCL1/ATOH1/FEZF2/FOXG1/NKX2-1/NTRK3/POU4F1/PROX1/SHH/SOX1/SOX9/TLX3/WNT1	13	1.91176470588235
GO:0003333	BP	GO:0003333	amino acid transmembrane transport	16/666	103/18614	7.48362436262216e-07	6.49858900040422e-06	3.72580666147285e-06	AGT/ATP1A2/NTSR1/RGS2/SLC17A7/SLC17A8/SLC1A1/SLC1A2/SLC1A7/SLC22A2/SLC38A3/SLC38A5/SLC6A15/SLC6A6/SLC6A9/SLC7A11	16	1.55339805825243
GO:0060996	BP	GO:0060996	dendritic spine development	15/666	91/18614	7.58287217595535e-07	6.57315991972144e-06	3.76856006962452e-06	ARHGAP44/CAMK2A/CAMK2B/EPHA4/EPHB2/EPHB3/FSTL4/GRIN3A/KIF1A/LPAR1/LRRK2/MEF2C/NLGN1/RELN/WNT7A	15	1.64835164835165
GO:1990089	BP	GO:1990089	response to nerve growth factor	11/666	48/18614	7.65492475926398e-07	6.62393577320114e-06	3.79767116022047e-06	BDNF/CALCA/CSNK1E/MAGI2/MAPT/NGF/NTF4/NTRK3/RAP1A/SH3GL2/TMEM108	11	2.29166666666667
GO:0044062	BP	GO:0044062	regulation of excretion	8/666	23/18614	7.84057434825127e-07	6.77265780696924e-06	3.8829372916971e-06	AGT/AGTR1/DRD2/EDNRB/NPSR1/OXT/SPX/TACR1	8	3.47826086956522

	ONTOLOGY	ID	Description	GeneRatio	BgRatio	pvalue	p.adjust	qvalue	geneID	Count	richFactor
GO:0015908	BP	GO:0015908	fatty acid transport	17/666	116/18614	7.924294684323 12e-07	6.820999715139 78e-06	3.910652939429 07e-06	AVPR1B/BDKRB2/DRD2/DRD4/FABP7/IL1A/LEP/MAP2K6/MIF/NMB/NMUR2/NTSR1/OXT/P2RX4/P2RX7/SLC22A2/SPX	17	1.465517241379 31
GO:0042752	BP	GO:0042752	regulation of circadian rhythm	17/666	116/18614	7.924294684323 12e-07	6.820999715139 78e-06	3.910652939429 07e-06	ADRB1/CDK1/CHRNB2/CSNK1E/DRD2/DRD4/GHRHR/GNA11/GNAQ/KCNA2/MAPK10/NKX2-1/NLGN1/PRKCG/PROX1/SOX14/TOP2A	17	1.465517241379 31
GO:0044060	BP	GO:0044060	regulation of endocrine process	10/666	39/18614	7.984581383371 25e-07	6.860877185187 01e-06	3.933515709106 52e-06	AGT/AGTR1/F2RL1/GAL/GALR1/INHBA/KISS1/LEP/TACR2/UCN	10	2.564102564102 56
GO:0038003	BP	GO:0038003	G protein-coupled opioid receptor signaling pathway	6/666	11/18614	8.128924058288 33e-07	6.972715837083 27e-06	3.997635657947 62e-06	ITGB3/NPBWR1/OPRD1/OPRM1/SIGMAR1/SYP	6	5.454545454545 45
GO:0097061	BP	GO:0097061	dendritic spine organization	14/666	80/18614	8.353190266898 17e-07	7.152601073485 1e-06	4.100768447549 31e-06	ARHGAP44/CAMK2B/CHRNA7/EPHA4/EPHB2/EPHB3/GRIN2B/HOMER1/KIF1A/LRRK2/NLGN1/PRNP/RELN/WNT7A	14	1.75
GO:0043491	BP	GO:0043491	protein kinase B signaling	24/666	215/18614	8.423824801483 56e-07	7.200538939007 25e-06	4.128252447335 74e-06	AXL/CCL5/CCR7/CPNE1/CX3CR1/DRD2/FGFR1/GDF15/GPER1/GRM2/HSP90AB1/IGF1/LEP/MAGI2/MC1R/NOX4/NTRK3/P2RX4/PIK3CD/PIK3CG/PIK3R5/PINK1/SOX9/TYRO3	24	1.116279069767 44
GO:0048592	BP	GO:0048592	eye morphogenesis	20/666	157/18614	8.678905580864 33e-07	7.405698078116 e-06	4.245875409351 94e-06	BCL2/CALB1/EPHB2/GNGT1/HCN1/LHX1/OLFM3/PROX1/SLC1A1/SOX1/SOX9/TH/THRB/TWIST1/VEGFA/WNT16/WNT2/WNT2B/WNT5A/WNT9A	20	1.273885350318 47
GO:0007266	BP	GO:0007266	Rho protein signal transduction	18/666	130/18614	8.904747091827 92e-07	7.585239507163 65e-06	4.348811085437 08e-06	ADGRG1/ADRA1A/ADRA2A/AGTR1/ARRB1/CELSR1/COL3A1/F2RL1/F2RL2/GNA13/LIMK1/LPAR1/NGFR/NTN1/P2RY10/P2RY8/PDPN/RTN4R	18	1.384615384615 38
GO:0045987	BP	GO:0045987	positive regulation of smooth muscle contraction	9/666	31/18614	9.054160274908 87e-07	7.699169161103 3e-06	4.414129859029 51e-06	ADRA1A/EDN2/GPER1/NMU/OXT/OXTR/SPX/TACR1/TACR2	9	2.903225806451 61
GO:0032496	BP	GO:0032496	response to lipopolysaccharide	32/666	345/18614	9.177113037533 63e-07	7.790243623398 58e-06	4.466345168889 65e-06	AXL/BDKRB1/CAPN2/CCL5/CCR7/CLDN1/CNR1/CNR2/CSF1/CTSG/CX3CR1/CXCL13/CXCL8/EDNRB/EPHB2/IL1A/IRAK1/IRAK2/MAOB/MEF2C/MIF/NOS1/OPRM1/P2RX7/PENK/PLCG2/PTGER4/PTGFR/RIPK2/SNCA/TH/WNT5A	32	0.927536231884 058
GO:0071695	BP	GO:0071695	anatomical structure maturation	26/666	247/18614	9.238117498658 8e-07	7.828508190673 8e-06	4.488283220310 28e-06	ASCL1/AURKA/AXL/BCL2/CDK5R2/CNTN2/CX3CR1/EDNRA/EDNRB/EPAS1/GAL/GHRHR/IGF1/KIF14/LEP/LRRK2/NKX6-1/NRCAM/PTH1R/PTK2B/RYR1/S1PR1/SOX10/VEGFA/WNT1/WNT5A	26	1.052631578947 37
GO:0007565	BP	GO:0007565	female pregnancy	22/666	187/18614	9.888650171054 93e-07	8.365355523362 3e-06	4.796071475299 22e-06	ADCYAP1/ADRA2A/AGT/APELA/BCL2/CALCA/CAPN2/CNR1/FOSB/ITGB3/LEP/MMP9/OXT/OXTR/PNOC/PRL/PTN/RGS2/RLN2/SLC38A3/UCN/VEGFA	22	1.176470588235 29
GO:0031649	BP	GO:0031649	heat generation	7/666	17/18614	1.034294693494 51e-06	8.675014366084 49e-06	4.973606780105 62e-06	ADRB1/ADRB2/ADRB3/CNR1/EDNRB/IL1A/PTGER3	7	4.117647058823 53

	ONTOLOGY	ID	Description	GeneRatio	BgRatio	pvalue	p.adjust	qvalue	geneID	Count	richFactor
GO:0051412	BP	GO:0051412	response to corticosterone	7/666	17/18614	1.03429469349451e-06	8.67501436608449e-06	4.97360678010562e-06	CCND1/FOSB/MAOB/NEFL/NTRK3/TH/UCN3	7	4.11764705882353
GO:0061548	BP	GO:0061548	ganglion development	7/666	17/18614	1.03429469349451e-06	8.67501436608449e-06	4.97360678010562e-06	ASCL1/FZD3/NRP2/PHOX2B/SEMA3F/TUBB3/UNC5C	7	4.11764705882353
GO:0097154	BP	GO:0097154	GABAergic neuron differentiation	7/666	17/18614	1.03429469349451e-06	8.67501436608449e-06	4.97360678010562e-06	ASCL1/CNTN2/DRD2/FEZF2/INHBA/NKX2-1/TLX3	7	4.11764705882353
GO:1904948	BP	GO:1904948	midbrain dopaminergic neuron differentiation	7/666	17/18614	1.03429469349451e-06	8.67501436608449e-06	4.97360678010562e-06	CSNK1E/DKK1/WNT1/WNT2/WNT3/WNT3A/WNT5A	7	4.11764705882353
GO:0051235	BP	GO:0051235	maintenance of location	32/666	347/18614	1.04132985114677e-06	8.71914176897169e-06	4.9989061445708e-06	ANK3/ASPM/ATP1A2/ATP2A1/BDKRB1/CACNA1C/CAMK2D/CAV1/CCR7/DRD2/F2/FKBP1B/GPER1/GRIK5/HTR2B/HTR2C/ITGB3/ITPR1/LEP/NOS1/NPSR1/NTSR1/P2RX7/PINK1/PLCB4/PLCG1/PLCG2/PTK2B/RYR1/SLC18A2/SNCA/TRPC1	32	0.922190201729107
GO:0001759	BP	GO:0001759	organ induction	8/666	24/18614	1.13932228179056e-06	9.47507447546637e-06	5.43230162677592e-06	DKK1/GDNF/ROBO2/WNT11/WNT2/WNT2B/WNT3A/WNT5A	8	3.33333333333333
GO:0043576	BP	GO:0043576	regulation of respiratory gaseous exchange	8/666	24/18614	1.13932228179056e-06	9.47507447546637e-06	5.43230162677592e-06	ATP1A2/GRP/GRPR/NLGN3/NMB/NTSR1/PHOX2B/TLX3	8	3.33333333333333
GO:0045932	BP	GO:0045932	negative regulation of muscle contraction	8/666	24/18614	1.13932228179056e-06	9.47507447546637e-06	5.43230162677592e-06	ADRA2A/ADRB2/ATP1A2/CALCA/GUCY1A1/PIK3CG/PRKG1/RGS2	8	3.33333333333333
GO:0051350	BP	GO:0051350	negative regulation of lyase activity	8/666	24/18614	1.13932228179056e-06	9.47507447546637e-06	5.43230162677592e-06	CCR2/EDNRB/GALR1/GALR3/GRM2/HPCA/LTB4R2/P2RY13	8	3.33333333333333
GO:0007548	BP	GO:0007548	sex differentiation	28/666	283/18614	1.24012986992518e-06	1.02960106599363e-05	5.9029652592278e-06	A2M/ADCYAP1R1/ADGRG1/ASPM/AXL/BCL2/BCL2L1/CCND1/CGA/CNTFR/GFRA1/IL1A/INHBA/LEP/LHB/LHX1/NKX2-1/ROBO2/SHH/SLIT2/SLIT3/SOX9/TYRO3/VEGFA/VGF/WNT2B/WNT5A/WNT7A	28	0.989399293286219
GO:0007265	BP	GO:0007265	Ras protein signal transduction	31/666	333/18614	1.2554321516724e-06	1.04054789636928e-05	5.96572622708265e-06	ADGRG1/ADRA1A/ADRA2A/AGTR1/ARHGAP44/ARRB1/CELSR1/COL3A1/CSF1/EPHB2/F2RL1/F2RL2/GABARAP/GNA13/IGF1/KIF14/LIMK1/LPAR1/NGF/NGFR/NTN1/P2RY10/P2RY8/PDPN/PIK3CG/PLD1/RAB39B/RAP1A/RASAL1/RTN4R/SOS2	31	0.930930930930931
GO:0031099	BP	GO:0031099	regeneration	22/666	190/18614	1.29633755631463e-06	1.07264294432431e-05	6.14973531499959e-06	AURKA/AXL/BCL2/CCND1/CDK1/CLDN1/CXCL12/EPHA4/ERBB4/FKBP1B/FZD9/GAP43/IGF1/MAG/MAP1B/NEFL/PTN/RTN4R/SOX2/TNR/WNT1/WNT7A	22	1.15789473684211
GO:0014068	BP	GO:0014068	positive regulation of phosphatidylinositol 3-kinase signaling	14/666	83/18614	1.32684299788945e-06	1.09420022393064e-05	6.27332869189342e-06	AGT/CCL5/ERBB4/F2/F2RL1/FGFR1/GPER1/IGF1/LEP/NTRK3/RELN/SEMA3E/SOX9/WNT16	14	1.68674698795181
GO:0140029	BP	GO:0140029	exocytic process	14/666	83/18614	1.32684299788945e-06	1.09420022393064e-05	6.27332869189342e-06	CPLX1/GRIK5/NLGN1/P2RX1/RAB3B/RPH3A/SEPTIN5/SLC18A2/SNAP25/SNCA/STX1A/STXBP1/SYT9/UNC13C	14	1.68674698795181
GO:0098900	BP	GO:0098900	regulation of action potential	12/666	61/18614	1.39211091006154e-06	1.14610136062856e-05	6.57089113327118e-06	ADRA1A/CACNA1C/CAMK2D/CAV1/CHRNB2/CNR1/CNR2/FGF12/FGF13/HCN1/NTSR1/TACR1	12	1.9672131147541

	ONTOLOGY	ID	Description	GeneRatio	BgRatio	pvalue	p.adjust	qvalue	geneID	Count	richFactor
GO:0032970	BP	GO:0032970	regulation of actin filament-based process	34/666	387/18614	1.45193004331369e-06	1.19335052891083e-05	6.84178265437553e-06	ARHGAP44/ATP1A2/ATP2A1/CACNA1C/CAMK2D/CAV1/CCR7/CELSR1/CXCL12/EFNA5/EPHA3/F2RL1/FGF13/FZD10/GPM6B/IL1A/ITGB3/LIMK1/LPAR1/NTRK3/PDPN/PIK3R2/PRKN/PROX1/PTGER4/PTK2B/S1PR1/SEMA3E/SLIT2/SPTBN2/TACR1/TENM1/TMOD2/WNT11	34	0.878552971576227
GO:0060688	BP	GO:0060688	regulation of morphogenesis of a branching structure	11/666	51/18614	1.46339912673201e-06	1.20076906642535e-05	6.88431502022951e-06	AGT/GDNF/LHX1/LRRK2/SHH/SOX9/SULF1/VEGFA/WNT2/WNT2B/WNT5A	11	2.15686274509804
GO:0014831	BP	GO:0014831	gastro-intestinal system smooth muscle contraction	6/666	12/18614	1.57651006356067e-06	1.28713404691042e-05	7.37946745961169e-06	HTR1D/HTR2B/PTGER3/SPX/SULF1/TACR2	6	5
GO:0042756	BP	GO:0042756	drinking behavior	6/666	12/18614	1.57651006356067e-06	1.28713404691042e-05	7.37946745961169e-06	AGT/APLN/DRD2/OXT/TACR1/UCN	6	5
GO:1904953	BP	GO:1904953	Wnt signaling pathway involved in midbrain dopaminergic neuron differentiation	6/666	12/18614	1.57651006356067e-06	1.28713404691042e-05	7.37946745961169e-06	CSNK1E/WNT1/WNT2/WNT3/WNT3A/WNT5A	6	5
GO:0051957	BP	GO:0051957	positive regulation of amino acid transport	8/666	25/18614	1.62312009104544e-06	1.32080053766364e-05	7.57248602950271e-06	AGT/NTSR1/P2RX7/SLC17A8/SLC38A3/SLC6A1/STXBP1/SYT4	8	3.2
GO:2000193	BP	GO:2000193	positive regulation of fatty acid transport	8/666	25/18614	1.62312009104544e-06	1.32080053766364e-05	7.57248602950271e-06	AVPR1B/IL1A/MAP2K6/MIF/NTSR1/OXT/P2RX4/P2RX7	8	3.2
GO:0034766	BP	GO:0034766	negative regulation of monoatomic ion transmembrane transport	16/666	109/18614	1.62928862537089e-06	1.32362869317321e-05	7.58870056566835e-06	ADRA2A/ANK3/ATP1A2/BCL2/CAMK2D/CAV1/DRD4/EPHB2/FKBP1B/GRP/MMP9/NEDD4L/NOS1/NTSR1/SLC6A9/TWIST1	16	1.46788990825688
GO:0003128	BP	GO:0003128	heart field specification	7/666	18/18614	1.64028099085769e-06	1.3259837286292e-05	7.60220258401324e-06	AXIN2/DKK1/MEF2C/ROBO2/WNT11/WNT3A/WNT5A	7	3.88888888888889
GO:0010522	BP	GO:0010522	regulation of calcium ion transport into cytosol	7/666	18/18614	1.64028099085769e-06	1.3259837286292e-05	7.60220258401324e-06	ADCYAP1R1/BCL2/CAV1/NOS1/P2RX2/P2RX4/P2RX7	7	3.88888888888889
GO:0032310	BP	GO:0032310	prostaglandin secretion	7/666	18/18614	1.64028099085769e-06	1.3259837286292e-05	7.60220258401324e-06	IL1A/LEP/MAP2K6/MIF/OXT/P2RX4/P2RX7	7	3.88888888888889
GO:0007595	BP	GO:0007595	lactation	10/666	42/18614	1.67719491451473e-06	1.35137918112129e-05	7.74780118404818e-06	APLN/CAV1/CCND1/ERBB4/GHRHR/OXTR/PRL/SLC29A1/SLC6A3/VEGFA	10	2.38095238095238
GO:0071542	BP	GO:0071542	dopaminergic neuron differentiation	10/666	42/18614	1.67719491451473e-06	1.35137918112129e-05	7.74780118404818e-06	CSNK1E/DKK1/PHOX2B/SHH/VEGFA/WNT1/WNT2/WNT3/WNT3A/WNT5A	10	2.38095238095238
GO:0048839	BP	GO:0048839	inner ear development	22/666	193/18614	1.6889919470989e-06	1.35865718821458e-05	7.78952785318932e-06	ATOH1/CALB1/EPHA4/EPHB2/FOXG1/FZD3/HPCA/KCNQ1/MCM2/NEUROD1/NTN1/NTRK3/PHOX2B/PROX1/SHH/SLC17A8/SLC44A4/SOX2/SOX9/WNT1/WNT3A/WNT5A	22	1.13989637305699
GO:0043010	BP	GO:0043010	camera-type eye development	31/666	338/18614	1.72114172415518e-06	1.38225679317365e-05	7.92483040172586e-06	CACNA1C/CALB1/DRD2/EPHB2/GPM6A/HCN1/HPCA/INHBA/LHX1/NEUROD1/PROX1/SHH/SLC17A7/SLC17A8/SLC1A1/SLC6A3/SLC7A11/SOX1/SOX9/TH/THRB/TWIST1/VAX1/VEGFA/WNT16/WNT2/WNT2B/WNT5A/WNT7A/WNT7B/WNT9A	31	0.917159763313609

	ONTOLOGY	ID	Description	GeneRatio	BgRatio	pvalue	p.adjust	qvalue	geneID	Count	richFactor
GO:0006942	BP	GO:0006942	regulation of striated muscle contraction	15/666	97/18614	1.7546189853052e-06	1.40684377043638e-05	8.06579380726712e-06	ADRA1A/ADRA1B/ATP1A2/ATP2A1/CACNA1C/CAMK2D/CAV1/FGF13/FKBP1B/KCNQ1/NOS1/P2RX4/PIK3CG/RGS2/UCN	15	1.54639175257732
GO:0097529	BP	GO:0097529	myeloid leukocyte migration	25/666	241/18614	1.95069569100789e-06	1.56150966145012e-05	8.95253277014934e-06	CALCA/CCL5/CCR2/CCR7/CSF1/CTSG/CX3CR1/CXCL12/CXCL13/CXCL8/DUSP1/EDN2/EDN3/EDNRB/IL1A/IL6R/MIF/P2RX4/PIK3CD/PIK3CG/PTGER4/PTK2B/SCG2/SLIT2/VEGFA	25	1.03734439834025
GO:0015807	BP	GO:0015807	L-amino acid transport	14/666	86/18614	2.06238616004218e-06	1.64823219131826e-05	9.44973513125018e-06	AGT/ATP1A2/NTSR1/RGS2/SLC17A7/SLC17A8/SLC1A1/SLC1A2/SLC1A7/SLC22A2/SLC38A3/SLC38A5/SLC6A9/SLC7A11	14	1.62790697674419
GO:0002021	BP	GO:0002021	response to dietary excess	9/666	34/18614	2.1395822815476e-06	1.70438361650023e-05	9.7716655595034e-06	ADRB1/ADRB2/ADRB3/CNTN2/GDF15/LEP/NMUR2/SCTR/VGF	9	2.64705882352941
GO:0098664	BP	GO:0098664	G protein-coupled serotonin receptor signaling pathway	9/666	34/18614	2.1395822815476e-06	1.70438361650023e-05	9.7716655595034e-06	CHRM2/CHRM4/CHRM5/DRD4/GNAZ/HTR1D/HTR2B/HTR2C/HTR7	9	2.64705882352941
GO:0010259	BP	GO:0010259	multicellular organism aging	8/666	26/18614	2.27127659621794e-06	1.80636318291443e-05	1.03563403986977e-05	ADRA1A/AVPR1B/EDNRA/GNA11/GNA13/INHBA/SLC1A2/TH	8	3.07692307692308
GO:0098656	BP	GO:0098656	monoatomic anion transmembrane transport	17/666	125/18614	2.29665326329028e-06	1.82359463474503e-05	1.04551327027091e-05	CLCN1/GABRA3/GABRA4/GABRB1/GABRB3/GABRE/GABRG2/GLRA3/GLRB/NMUR2/PRNP/SLC17A7/SLC17A8/SLC1A1/SLC1A7/SLC4A8/SLC6A1	17	1.36
GO:0098739	BP	GO:0098739	import across plasma membrane	23/666	212/18614	2.32715774286012e-06	1.84483553325121e-05	1.05769121861401e-05	AGT/ATP1A2/CACNA1A/CACNA1C/CACNA1D/KCNJ14/KCNJ5/KCNJ9/KCNQ1/NTSR1/P2RX1/PRNP/RGS2/SLC17A8/SLC1A1/SLC1A2/SLC22A2/SLC38A3/SLC38A5/SLC6A1/SLC6A6/SLC6A9/SLC7A11	23	1.08490566037736
GO:0032368	BP	GO:0032368	regulation of lipid transport	19/666	153/18614	2.39096727269452e-06	1.88932542528836e-05	1.08319840734528e-05	AGT/AGTR1/APOC1/AVPR1B/CAV1/GAL/GALR1/IL1A/ITGB3/LEP/MAP2K6/MIF/NMB/NTSR1/OXT/P2RX4/P2RX7/PRKN/SHH	19	1.24183006535948
GO:0034763	BP	GO:0034763	negative regulation of transmembrane transport	19/666	153/18614	2.39096727269452e-06	1.88932542528836e-05	1.08319840734528e-05	ADRA2A/ANK3/ATP1A2/BCL2/CAMK2D/CAV1/DRD4/EPHB2/FKBP1B/GRP/LEP/MMP9/NEDD4L/NOS1/NTSR1/PRKCB/RGS2/SLC6A9/TWIST1	19	1.24183006535948
GO:1903829	BP	GO:1903829	positive regulation of protein localization	38/666	468/18614	2.40911709232633e-06	1.90061163864911e-05	1.08966908104378e-05	ANK3/APC/CDK1/EPHA3/EPHB2/ERBB4/F2/F2RL1/F2RL2/GCG/GNA11/GNAI1/GPER1/HPCA/HSP90AB1/IGF1/IL1A/ITPR1/LEP/MAPT/NGFR/NKX6-1/P2RX7/PIK3R2/PINK1/PRKCB/PRKG2/PRKN/PRNP/SHH/SYT11/TENM1/TMEM35A/UCN3/VAMP2/WNT3A/WNT5A/WNT7A	38	0.811965811965812
GO:0007194	BP	GO:0007194	negative regulation of adenylate cyclase activity	7/666	19/18614	2.51704827004167e-06	1.97940675956077e-05	1.13484433160447e-05	CCR2/EDNRB/GALR1/GALR3/GRM2/LTB4R2/P2RY13	7	3.68421052631579
GO:0014048	BP	GO:0014048	regulation of glutamate secretion	7/666	19/18614	2.51704827004167e-06	1.97940675956077e-05	1.13484433160447e-05	GRM2/NPY5R/NTSR1/P2RX7/SNCA/STXBP1/SYT4	7	3.68421052631579

	ONTOLOGY	ID	Description	GeneRatio	BgRatio	pvalue	p.adjust	qvalue	geneID	Count	richFactor
GO:0001709	BP	GO:0001709	cell fate determination	10/666	44/18614	2.65121798098109e-06	2.08158728059458e-05	1.19342702792776e-05	ASCL1/ATOH1/DSCAML1/FEZF2/FOXG1/MEF2C/NTF4/PROX1/WNT1/WNT7A	10	2.27272727272727
GO:0097479	BP	GO:0097479	synaptic vesicle localization	11/666	54/18614	2.66782871669517e-06	2.08795830295489e-05	1.197079697384e-05	KIF5A/KIF5C/LRRK2/MAP2/NLGN1/PINK1/PRKN/SLC2A4/SNCA/SYN2/SYN3	11	2.03703703703704
GO:1904645	BP	GO:1904645	response to amyloid-beta	11/666	54/18614	2.66782871669517e-06	2.08795830295489e-05	1.197079697384e-05	ADRB2/CACNA1A/CHRNA7/EPHA4/EPHB2/IGF1/ITGA4/MMP13/MMP9/NGFR/PRNP	11	2.03703703703704
GO:0086003	BP	GO:0086003	cardiac muscle cell contraction	13/666	76/18614	2.68526519651447e-06	2.09826366309517e-05	1.20298802293854e-05	ATP1A2/ATP2A1/CACNA1C/CACNA1D/CAMK2D/CAV1/FGF12/FGF13/KCNJ5/KCNQ1/NEDD4L/SCN1B/SCN2B	13	1.71052631578947
GO:0035025	BP	GO:0035025	positive regulation of Rho protein signal transduction	9/666	35/18614	2.78936475241723e-06	2.17614726319535e-05	1.24764067539197e-05	ADGRG1/ARRB1/COL3A1/F2RL1/F2RL2/LPAR1/P2RY10/P2RY8/RTN4R	9	2.57142857142857
GO:0046888	BP	GO:0046888	negative regulation of hormone secretion	12/666	65/18614	2.8240872990566e-06	2.19974470283093e-05	1.2611696888105e-05	ADRA2A/CARTPT/DRD2/F2RL1/FKBP1B/GNAZ/INHBA/LEP/NMB/PRKN/TACR2/UCN	12	1.84615384615385
GO:0048593	BP	GO:0048593	camera-type eye morphogenesis	17/666	127/18614	2.86807105614581e-06	2.23046981660707e-05	1.27878517943077e-05	CALB1/EPHB2/HCN1/LHX1/PROX1/SLC1A1/SOX1/SOX9/TH/THRB/TWIST1/VEGFA/WNT16/WNT2/WNT2B/WNT5A/WNT9A	17	1.33858267716535
GO:0048709	BP	GO:0048709	oligodendrocyte differentiation	15/666	101/18614	2.9556495037756e-06	2.29132765158629e-05	1.31367652691471e-05	ASCL1/CLU/CNTN2/CNTNAP1/GPM6B/LPAR1/MAG/NKX2-1/NKX6-1/PTN/SHH/SOX1/SOX10/SOX9/TP73	15	1.48514851485149
GO:0061337	BP	GO:0061337	cardiac conduction	15/666	101/18614	2.9556495037756e-06	2.29132765158629e-05	1.31367652691471e-05	AGT/ATP1A2/ATP2A1/ATP2A3/CACNA1C/CACNA1D/CAMK2D/CAV1/FKBP1B/HCN1/KCNJ5/KCNQ1/SCN1B/SCN2B/TRPC1	15	1.48514851485149
GO:0002526	BP	GO:0002526	acute inflammatory response	16/666	114/18614	2.98401277670945e-06	2.30967288150031e-05	1.32419431467073e-05	A2M/ADRA2A/CCR7/CNR1/CREB3L3/EDNRB/F2/IL1A/IL6R/IL6ST/NPY/NPY5R/OPRM1/PIK3CG/PTGER3/TACR1	16	1.40350877192982
GO:0002092	BP	GO:0002092	positive regulation of receptor internalization	8/666	27/18614	3.12683026293365e-06	2.40883553954842e-05	1.38104679324755e-05	APELA/APLN/ARRB1/DRD2/MAGI2/PLCG2/VEGFA/WNT3A	8	2.96296296296296
GO:0045745	BP	GO:0045745	positive regulation of G protein-coupled receptor signaling pathway	8/666	27/18614	3.12683026293365e-06	2.40883553954842e-05	1.38104679324755e-05	CNTN2/DRD2/F2/GPER1/GRP/ITGB3/PTGDR2/TMOD2	8	2.96296296296296
GO:0048668	BP	GO:0048668	collateral sprouting	8/666	27/18614	3.12683026293365e-06	2.40883553954842e-05	1.38104679324755e-05	BDNF/EFNA5/FGF13/FSTL4/NGF/PRKG1/WNT3/WNT3A	8	2.96296296296296
GO:0002237	BP	GO:0002237	response to molecule of bacterial origin	32/666	366/18614	3.27006523685756e-06	2.51523797169873e-05	1.44205001879049e-05	AXL/BDKRB1/CAPN2/CCL5/CCR7/CLDN1/CNR1/CNR2/CSF1/CTSG/CX3CR1/CXCL13/CXCL8/EDNRB/EPHB2/IL1A/IRAK1/IRAK2/MAOB/MEF2C/MIF/NOS1/OPRM1/P2RX7/PENK/PLCG2/PTGER4/PTGFR/RIPK2/SNCA/TH/WNT5A	32	0.87431693989071
GO:0042593	BP	GO:0042593	glucose homeostasis	25/666	248/18614	3.27580734349951e-06	2.51571767082814e-05	1.44232504252437e-05	ADCY5/ADRA2A/BECN2/CARTPT/CNR1/EFNA5/FKBP1B/GCG/GCGR/GHRHR/GPER1/GRIK5/LEP/LEPR/NEUROD1/NGFR/NMB/NOX4/NPTX1/PIK3R2/SLC29A1/SLC2A4/SSTR5/TH/VGF	25	1.00806451612903

	ONTOLOGY	ID	Description	GeneRatio	BgRatio	pvalue	p.adjust	qvalue	geneID	Count	richFactor
GO:1903307	BP	GO:1903307	positive regulation of regulated secretory pathway	10/666	45/18614	3.30037006397585e-06	2.53062696793156e-05	1.45087292245067e-05	CDK5R2/F2RL1/NLGN1/SLC4A8/STX1A/STXBP1/SYT10/SYT4/SYT7/SYT9	10	2.22222222222222
GO:0048259	BP	GO:0048259	regulation of receptor-mediated endocytosis	16/666	115/18614	3.35327997543994e-06	2.56719175689834e-05	1.4718364476558e-05	APELA/APLN/APOC1/ARRB1/CLU/DKK1/DRD2/DRD4/GSG1L/HPCA/ITGB3/LPAR1/MAGI2/PLCG2/VEGFA/WNT3A	16	1.39130434782609
GO:0051896	BP	GO:0051896	regulation of protein kinase B signaling	21/666	186/18614	3.37649250640573e-06	2.58094256127281e-05	1.47972013418157e-05	AXL/CCR7/CPNE1/CX3CR1/DRD2/FGFR1/GDF15/GPER1/GRM2/HSP90AB1/IGF1/LEP/MAGI2/MC1R/NOX4/NTRK3/P2RX4/PIK3CD/PIK3CG/PIK3R5/PINK1	21	1.12903225806452
GO:0033500	BP	GO:0033500	carbohydrate homeostasis	25/666	249/18614	3.52116706055985e-06	2.68539090417215e-05	1.53960303056569e-05	ADCY5/ADRA2A/BECN2/CARTPT/CNR1/EFNA5/FKBP1B/GCG/GCGR/GHRHR/GPER1/GRIK5/LEP/LEPR/NEUROD1/NGFR/NMB/NOX4/NPTX1/PIK3R2/SLC29A1/SLC2A4/SSTR5/TH/VGF	25	1.00401606425703
GO:0048754	BP	GO:0048754	branching morphogenesis of an epithelial tube	19/666	157/18614	3.52406334321676e-06	2.68539090417215e-05	1.53960303056569e-05	AGT/BCL2/CELSR1/CSF1/EDNRA/GDNF/GNA13/LHX1/NKX2-1/SEMA3E/SHH/SLIT2/SOX9/TBX20/VEGFA/WNT1/WNT2/WNT2B/WNT5A	19	1.21019108280255
GO:0106027	BP	GO:0106027	neuron projection organization	14/666	90/18614	3.60022562748126e-06	2.73918095341647e-05	1.57044223639683e-05	ARHGAP44/CAMK2B/CHRNA7/EPHA4/EPHB2/EPHB3/GRIN2B/HOMER1/KIF1A/LRRK2/NLGN1/PRNP/RELN/WNT7A	14	1.55555555555556
GO:0021516	BP	GO:0021516	dorsal spinal cord development	7/666	20/18614	3.75306399167667e-06	2.84226648984451e-05	1.6295438011059e-05	ASCL1/GDNF/LHX1/MDGA1/PROX1/WNT1/WNT3A	7	3.5
GO:0035813	BP	GO:0035813	regulation of renal sodium excretion	7/666	20/18614	3.75306399167667e-06	2.84226648984451e-05	1.6295438011059e-05	AGT/AGTR1/DRD2/EDNRB/OXT/SPX/TACR1	7	3.5
GO:0048670	BP	GO:0048670	regulation of collateral sprouting	7/666	20/18614	3.75306399167667e-06	2.84226648984451e-05	1.6295438011059e-05	BDNF/EFNA5/FGF13/FSTL4/NGF/WNT3/WNT3A	7	3.5
GO:0072009	BP	GO:0072009	nephron epithelium development	16/666	116/18614	3.76301605656117e-06	2.84541906430741e-05	1.63135125237478e-05	AGT/BCL2/CALB1/EDNRA/EDNRB/GDNF/LHX1/MAGI2/MEF2C/SHH/SOX9/VEGFA/WNT1/WNT11/WNT2B/WNT7B	16	1.37931034482759
GO:0060562	BP	GO:0060562	epithelial tube morphogenesis	30/666	334/18614	3.85526943620781e-06	2.91069881397256e-05	1.66877779622092e-05	AGT/BCL2/CELSR1/CSF1/EDNRA/EPHA4/FZD3/GDNF/GNA13/LHX1/MEF2C/NKX2-1/NR3C1/NTN1/PIK3CD/PRKACB/PROX1/SEMA3E/SHH/SLIT2/SOX9/TBX20/TWIST1/VEGFA/WNT1/WNT11/WNT2/WNT2B/WNT3A/WNT5A	30	0.898203592814371
GO:0055078	BP	GO:0055078	sodium ion homeostasis	11/666	56/18614	3.88874592374354e-06	2.92698104367527e-05	1.67811281338945e-05	AGT/AGTR1/ATP1A2/DRD2/EDNRA/EDNRB/IL1A/MC3R/OXT/SPX/TACR1	11	1.96428571428571
GO:0060986	BP	GO:0060986	endocrine hormone secretion	11/666	56/18614	3.88874592374354e-06	2.92698104367527e-05	1.67811281338945e-05	AGT/AGTR1/CGA/GAL/GALR1/INHBA/KCNQ1/KISS1/LEP/TACR2/UCN	11	1.96428571428571
GO:1902305	BP	GO:1902305	regulation of sodium ion transmembrane transport	12/666	67/18614	3.93873731827454e-06	2.96007552283171e-05	1.69708672155496e-05	ANK3/ATP1A2/CAMK2D/DRD4/FGF12/FGF13/GRP/KCNQ1/NEDD4L/NOS1/SCN1B/SCN2B	12	1.7910447761194
GO:0007622	BP	GO:0007622	rhythmic behavior	10/666	46/18614	4.08314385784502e-06	3.05924574105309e-05	1.75394353457175e-05	ADRB1/CHRNB2/CSNK1E/DRD2/GHRHR/KCNA2/KCNQ1/MC3R/NLGN1/TH	10	2.17391304347826

	ONTOLOGY	ID	Description	GeneRatio	BgRatio	pvalue	p.adjust	qvalue	geneID	Count	richFactor
GO:0046427	BP	GO:0046427	positive regulation of receptor signaling pathway via JAK-STAT	10/666	46/18614	4.08314385784502e-06	3.05924574105309e-05	1.75394353457175e-05	AGT/CAMK2A/CCL5/ERBB4/F2/GHR/IL6R/LEP/PRL/PTK2B	10	2.17391304347826
GO:0015697	BP	GO:0015697	quaternary ammonium group transport	8/666	28/18614	4.24093954803812e-06	3.16300726534254e-05	1.81343266034631e-05	CHRNA3/SLC17A8/SLC18A3/SLC22A2/SLC22A3/SLC44A4/SLC5A7/TACR2	8	2.85714285714286
GO:0032104	BP	GO:0032104	regulation of response to extracellular stimulus	8/666	28/18614	4.24093954803812e-06	3.16300726534254e-05	1.81343266034631e-05	BCL2/CARTPT/LEP/NPY/OPRM1/PRKCG/SPX/UCN	8	2.85714285714286
GO:0032107	BP	GO:0032107	regulation of response to nutrient levels	8/666	28/18614	4.24093954803812e-06	3.16300726534254e-05	1.81343266034631e-05	BCL2/CARTPT/LEP/NPY/OPRM1/PRKCG/SPX/UCN	8	2.85714285714286
GO:0007616	BP	GO:0007616	long-term memory	9/666	37/18614	4.6096190855283e-06	3.42757606737845e-05	1.96511669590879e-05	CALB1/CAMK4/DRD2/GRIA1/NTF4/PRNP/RELN/SLC2A4/TACR1	9	2.43243243243243
GO:0032094	BP	GO:0032094	response to food	9/666	37/18614	4.6096190855283e-06	3.42757606737845e-05	1.96511669590879e-05	CARTPT/GHR/LEP/NPY/OPRM1/OXT/PRKCG/SPX/UCN	9	2.43243243243243
GO:0051897	BP	GO:0051897	positive regulation of protein kinase B signaling	16/666	118/18614	4.71959023834287e-06	3.50404622680592e-05	2.00895898680933e-05	AXL/CCR7/CPNE1/CX3CR1/FGFR1/GDF15/HSP90AB1/IGF1/LEP/MC1R/NOX4/P2RX4/PIK3CD/PIK3CG/PIK3R5/PINK1	16	1.35593220338983
GO:0072593	BP	GO:0072593	reactive oxygen species metabolic process	24/666	237/18614	4.74179106716597e-06	3.51521916970147e-05	2.01536471966394e-05	AGT/AGTR1/BCL2/DDIT4/DRD5/F2/F2RL1/GLS2/GPX3/LEP/LRRK2/MAOB/MAPT/NDUFA13/NOX4/P2RX7/PINK1/PLCG2/PRKN/PTK2B/SLC18A2/SLC1A1/SNCA/TRAP1	24	1.0126582278481
GO:0001976	BP	GO:0001976	nervous system process involved in regulation of systemic arterial blood pressure	6/666	14/18614	4.81807013441384e-06	3.55567788448108e-05	2.03856073175689e-05	ADRA1A/AGT/CALCA/DRD2/P2RX2/TACR1	6	4.28571428571429
GO:0032306	BP	GO:0032306	regulation of prostaglandin secretion	6/666	14/18614	4.81807013441384e-06	3.55567788448108e-05	2.03856073175689e-05	IL1A/MAP2K6/MIF/OXT/P2RX4/P2RX7	6	4.28571428571429
GO:0032308	BP	GO:0032308	positive regulation of prostaglandin secretion	6/666	14/18614	4.81807013441384e-06	3.55567788448108e-05	2.03856073175689e-05	IL1A/MAP2K6/MIF/OXT/P2RX4/P2RX7	6	4.28571428571429
GO:0021549	BP	GO:0021549	cerebellum development	15/666	105/18614	4.84476354854215e-06	3.57001691770385e-05	2.04678166486978e-05	CDK5R2/GRID2/KIF14/LHX1/LPAR1/NEUROD1/NLGN4X/PROX1/SPTBN2/SSTR1/SSTR2/SSTR3/TUBA1A/WNT1/WNT7A	15	1.42857142857143
GO:1990845	BP	GO:1990845	adaptive thermogenesis	19/666	161/18614	5.12247056125793e-06	3.7690034144585e-05	2.16086569374212e-05	ADCYAP1/ADRB1/ADRB2/ADRB3/APC/CAV1/CCR2/EPAS1/HCRT/LEP/LEPR/MAP2K6/OXT/OXTR/PHOX2B/PLCL1/SCTR/TSHR/VEGFA	19	1.18012422360248
GO:0120161	BP	GO:0120161	regulation of cold-induced thermogenesis	18/666	147/18614	5.35984721106829e-06	3.93776517823627e-05	2.25762111305638e-05	ADCYAP1/ADRB1/ADRB2/ADRB3/APC/CAV1/CCR2/EPAS1/HCRT/LEP/LEPR/MAP2K6/OXT/OXTR/PHOX2B/PLCL1/TSHR/VEGFA	18	1.22448979591837
GO:0050730	BP	GO:0050730	regulation of peptidyl-tyrosine phosphorylation	25/666	255/18614	5.38140847311758e-06	3.94770487244372e-05	2.26331979301426e-05	ADRA1A/ADRA2A/AGT/CAV1/CCL5/CRLF1/EFNA5/EPHA4/ERBB4/GFRA1/GHR/IGF1/IL6R/IL6ST/ITGB3/LEP/MIF/NOX4/PLCG2/PRNP/PTK2B/RELN/RIPK2/VEGFA/WNT3A	25	0.980392156862745
GO:0031290	BP	GO:0031290	retinal ganglion cell axon guidance	7/666	21/18614	5.45622030688769e-06	3.99662038872623e-05	2.29136430489267e-05	EFNA5/EPHB2/EPHB3/NRCAM/ROBO2/SLIT2/VEGFA	7	3.33333333333333
GO:0015804	BP	GO:0015804	neutral amino acid transport	11/666	58/18614	5.57232629828793e-06	4.0695369622712e-05	2.3331692344595e-05	LEP/RGS2/SLC1A1/SLC1A2/SLC1A7/SLC38A3/SLC38A5/SLC6A15/SLC6A6/SLC6A9/SLC7A11	11	1.89655172413793

	ONTOLOGY	ID	Description	GeneRatio	BgRatio	pvalue	p.adjust	qvalue	geneID	Count	richFactor
GO:2000649	BP	GO:2000649	regulation of sodium ion transmembrane transporter activity	11/666	58/18614	5.57232629828793e-06	4.0695369622712e-05	2.3331692344595e-05	ANK3/ATP1A2/CAMK2D/DRD4/FGF12/FGF13/GRP/KCNQ1/NEDD4L/SCN1B/SCN2B	11	1.89655172413793
GO:0015732	BP	GO:0015732	prostaglandin transport	8/666	29/18614	5.67382819277816e-06	4.13138749148217e-05	2.36862971884088e-05	IL1A/LEP/MAP2K6/MIF/OXT/P2RX4/P2RX7/SLC22A2	8	2.75862068965517
GO:0015813	BP	GO:0015813	L-glutamate transmembrane transport	8/666	29/18614	5.67382819277816e-06	4.13138749148217e-05	2.36862971884088e-05	ATP1A2/NTSR1/SLC17A7/SLC17A8/SLC1A1/SLC1A2/SLC1A7/SLC7A11	8	2.75862068965517
GO:0021955	BP	GO:0021955	central nervous system neuron axonogenesis	9/666	38/18614	5.84987215576098e-06	4.25327243277592e-05	2.43850946137841e-05	CHRNB2/DCLK1/EPHA4/EPHB2/EPHB3/HSP90AB1/PHOX2B/SCN1B/SLIT2	9	2.36842105263158
GO:0106106	BP	GO:0106106	cold-induced thermogenesis	18/666	148/18614	5.90231399512288e-06	4.28506252378567e-05	2.4567355305829e-05	ADCYAP1/ADRB1/ADRB2/ADRB3/APC/CAV1/CCR2/EPAS1/HCRT/LEP/LEPR/MAP2K6/OXT/OXTR/PHOX2B/PLCL1/TSHR/VEGFA	18	1.21621621621622
GO:0006835	BP	GO:0006835	dicarboxylic acid transport	14/666	94/18614	6.08496969624101e-06	4.41115428569684e-05	2.52902715057246e-05	ATP1A2/GRM2/NPY5R/NTSR1/P2RX7/SLC17A7/SLC17A8/SLC1A1/SLC1A2/SLC1A7/SLC7A11/SNCA/STXBP1/SYT4	14	1.48936170212766
GO:0046189	BP	GO:0046189	phenol-containing compound biosynthetic process	10/666	48/18614	6.14205788460382e-06	4.43423406377932e-05	2.54225937543229e-05	ASIP/MC1R/PAH/SLC6A3/SLC7A11/SNCA/TH/TPH1/TRPC1/WNT5A	10	2.08333333333333
GO:0046717	BP	GO:0046717	acid secretion	10/666	48/18614	6.14205788460382e-06	4.43423406377932e-05	2.54225937543229e-05	CHRM5/DRD2/KCNQ1/NTSR1/OXT/OXTR/P2RX7/PTGER3/SLC6A1/UCN	10	2.08333333333333
GO:0090277	BP	GO:0090277	positive regulation of peptide hormone secretion	15/666	107/18614	6.14387262957012e-06	4.43423406377932e-05	2.54225937543229e-05	ADCYAP1/DRD2/F2/F2RL2/GCG/GHRHR/GNA11/GPER1/GRP/ITPR1/KISS1/NKX6-1/PRKCB/UCN/UCN3	15	1.4018691588785
GO:0003151	BP	GO:0003151	outflow tract morphogenesis	13/666	82/18614	6.4419044446934e-06	4.64251617971673e-05	2.66167281963949e-05	EDNRA/MEF2C/NPY1R/NPY2R/NPY5R/NRP2/ROBO2/RYR1/TBX20/TWIST1/VEGFA/WNT11/WNT5A	13	1.58536585365854
GO:0030282	BP	GO:0030282	bone mineralization	16/666	121/18614	6.56454188037305e-06	4.7239712067399e-05	2.70837306214975e-05	ADRB2/AXIN2/FZD9/GPM6B/IGF1/LEP/MEF2C/MMP13/P2RX7/PTH1R/PTK2B/PTN/S1PR1/SOX9/TWIST1/WNT11	16	1.32231404958678
GO:0009953	BP	GO:0009953	dorsal/ventral pattern formation	14/666	95/18614	6.90558625003413e-06	4.96212813142073e-05	2.84491449543573e-05	ASCL1/AXIN2/DSCAML1/FOXG1/LHX1/NKX2-1/PRKACB/SHH/SOX1/TBX20/WNT3/WNT3A/WNT7A/WNT8B	14	1.47368421052632
GO:0046660	BP	GO:0046660	female sex differentiation	16/666	122/18614	7.30932913560064e-06	5.2369318806818e-05	3.00246648703436e-05	A2M/ADCYAP1R1/AXL/BCL2/BCL2L1/CGA/INHBA/LEP/LHX1/ROBO2/SLIT2/SLIT3/TYRO3/VEGFA/VGF/WNT5A	16	1.31147540983607
GO:0098661	BP	GO:0098661	inorganic anion transmembrane transport	16/666	122/18614	7.30932913560064e-06	5.2369318806818e-05	3.00246648703436e-05	CLCN1/GABRA3/GABRA4/GABRB1/GABRB3/GABRE/GABRG2/GLRA3/GLRB/NMUR2/SLC17A7/SLC17A8/SLC1A1/SLC1A7/SLC4A8/SLC6A1	16	1.31147540983607
GO:0035265	BP	GO:0035265	organ growth	20/666	180/18614	7.3303518312207e-06	5.24434923587332e-05	3.00671904576386e-05	ADRA1A/AGT/BCL2/CDK1/CGA/ERBB4/IGF1/LEP/LEPR/MEF2C/MMP13/NLGN4X/PROX1/RGS2/S1PR1/SHH/SOX9/TBX20/TP73/WNT2	20	1.11111111111111

	ONTOLOGY	ID	Description	GeneRatio	BgRatio	pvalue	p.adjust	qvalue	geneID	Count	richFactor
GO:1990778	BP	GO:1990778	protein localization to cell periphery	30/666	345/18614	7.35414661801017e-06	5.25372538190697e-05	3.01209462919419e-05	ANK3/ARHGAP44/BCL2L1/CACNG7/CAV1/CPLX1/EPHA3/EPHB2/ERBB4/FGF13/GNAI1/GPC6/GPER1/GRIN2A/LGI1/LHFPL4/LRRC7/NPTX1/NSG1/P2RY1/PIK3R2/PRKG2/PRNP/RAB3B/RAP1A/SLC1A1/SNAP25/STXBP1/VAMP2/WNT3A	30	0.869565217391304
GO:0007202	BP	GO:0007202	activation of phospholipase C activity	8/666	30/18614	7.49576290868625e-06	5.331646121012e-05	3.05676857438542e-05	ADCYAP1R1/ADRA1A/AGT/AVPR1B/EDNRA/GNAQ/HTR2B/LPAR1	8	2.66666666666667
GO:0051938	BP	GO:0051938	L-glutamate import	8/666	30/18614	7.49576290868625e-06	5.331646121012e-05	3.05676857438542e-05	ATP1A2/NTSR1/SLC17A7/SLC17A8/SLC1A1/SLC1A2/SLC1A7/SLC7A11	8	2.66666666666667
GO:0097254	BP	GO:0097254	renal tubular secretion	8/666	30/18614	7.49576290868625e-06	5.331646121012e-05	3.05676857438542e-05	AGT/AGTR1/ATP6V0A4/DRD2/EDNRB/OXT/SPX/TACR1	8	2.66666666666667
GO:0001708	BP	GO:0001708	cell fate specification	15/666	109/18614	7.74460157553652e-06	5.48508846438443e-05	3.14474097963423e-05	APC/ASCL1/ATOH1/DKK1/NTRK3/POU4F1/SHH/SOX1/SOX2/SOX9/TBX20/TLX3/WNT11/WNT3A/WNT5A	15	1.37614678899083
GO:0002793	BP	GO:0002793	positive regulation of peptide secretion	15/666	109/18614	7.74460157553652e-06	5.48508846438443e-05	3.14474097963423e-05	ADCYAP1/DRD2/F2/F2RL2/GCG/GHRHR/GNA11/GPER1/GRP/ITPR1/KISS1/NKX6-1/PRKCB/UCN/UCN3	15	1.37614678899083
GO:0035812	BP	GO:0035812	renal sodium excretion	7/666	22/18614	7.75612712664737e-06	5.48508846438443e-05	3.14474097963423e-05	AGT/AGTR1/DRD2/EDNRB/OXT/SPX/TACR1	7	3.18181818181818
GO:0097107	BP	GO:0097107	postsynaptic density assembly	7/666	22/18614	7.75612712664737e-06	5.48508846438443e-05	3.14474097963423e-05	GRID2/NPTX1/NRXN1/NRXN2/NTRK3/PTK2B/SLITRK3	7	3.18181818181818
GO:0045986	BP	GO:0045986	negative regulation of smooth muscle contraction	6/666	15/18614	7.78719949919283e-06	5.49915022105356e-05	3.15280294303376e-05	ADRA2A/ADRB2/CALCA/GUCY1A1/PRKG1/RGS2	6	4
GO:0021545	BP	GO:0021545	cranial nerve development	11/666	60/18614	7.85952254135442e-06	5.54226015649311e-05	3.17751896748558e-05	CHRNB2/EPHB2/KCNA1/KCNA2/LPAR1/NRP2/PHOX2B/PLXNA1/PLXNA3/POU4F1/SEMA3F	11	1.83333333333333
GO:0001657	BP	GO:0001657	ureteric bud development	14/666	97/18614	8.84638211236998e-06	6.22031016914141e-05	3.56626231681567e-05	AGT/BCL2/CALB1/CRLF1/GDNF/LHX1/ROBO2/SHH/SLIT2/SOX9/VEGFA/WNT1/WNT11/WNT2B	14	1.44329896907216
GO:0060993	BP	GO:0060993	kidney morphogenesis	14/666	97/18614	8.84638211236998e-06	6.22031016914141e-05	3.56626231681567e-05	AGT/BCL2/CALB1/ERBB4/GDNF/LHX1/LRRK2/SHH/SOX9/VEGFA/WNT1/WNT11/WNT2B/WNT7B	14	1.44329896907216
GO:1904894	BP	GO:1904894	positive regulation of receptor signaling pathway via STAT	10/666	50/18614	9.04351017702247e-06	6.34983607429506e-05	3.64052281862995e-05	AGT/CAMK2A/CCL5/ERBB4/F2/GHR/IL6R/LEP/PRL/PTK2B	10	2
GO:0010863	BP	GO:0010863	positive regulation of phospholipase C activity	9/666	40/18614	9.2043645943249e-06	6.45355948375277e-05	3.69999009220028e-05	ADCYAP1R1/ADRA1A/AGT/AVPR1B/EDNRA/FGFR1/GNAQ/HTR2B/LPAR1	9	2.25
GO:0060292	BP	GO:0060292	long-term synaptic depression	8/666	31/18614	9.78805747605856e-06	6.85303454342277e-05	3.92901932274835e-05	DRD5/GRIA1/GRID2/GRM2/MAPT/PTK2B/SORCS3/STXBP1	8	2.58064516129032
GO:0007586	BP	GO:0007586	digestion	17/666	139/18614	9.8848681848767e-06	6.91097114205818e-05	3.96223585100171e-05	ADRA2A/CHRM5/KCNQ1/LEP/NEUROD1/NPSR1/OXT/OXTR/PRSS1/PRSS2/PTGER3/SOX9/SST/TACR1/UCN/UCN2/UCN3	17	1.22302158273381
GO:0072163	BP	GO:0072163	mesonephric epithelium development	14/666	98/18614	9.98672475485176e-06	6.96237619433992e-05	3.9917076773032e-05	AGT/BCL2/CALB1/CRLF1/GDNF/LHX1/ROBO2/SHH/SLIT2/SOX9/VEGFA/WNT1/WNT11/WNT2B	14	1.42857142857143

	ONTOLOGY	ID	Description	GeneRatio	BgRatio	pvalue	p.adjust	qvalue	geneID	Count	richFactor
GO:0072164	BP	GO:0072164	mesonephric tubule development	14/666	98/18614	9.98672475485176e-06	6.96237619433992e-05	3.991707677303 2e-05	AGT/BCL2/CALB1/CRLF1/GDNF/LHX1/ROBO2/SHH/SLIT2/SOX9/VEGFA/WNT1/WNT11/WNT2B	14	1.42857142857143
GO:0042745	BP	GO:0042745	circadian sleep/wake cycle	7/666	23/18614	1.08064076168196e-05	7.51251675200401e-05	4.30711727688917e-05	ADRB1/CHRNB2/DRD2/GHRHR/KCNA2/NLGN1/TH	7	3.04347826086957
GO:0048169	BP	GO:0048169	regulation of long-term neuronal synaptic plasticity	7/666	23/18614	1.08064076168196e-05	7.51251675200401e-05	4.30711727688917e-05	AGT/CAMK2B/DRD2/EPHB2/GRIK2/SNCA/SYP	7	3.04347826086957
GO:0014066	BP	GO:0014066	regulation of phosphatidylinositol 3-kinase signaling	15/666	112/18614	1.08432124426707e-05	7.52745609544162e-05	4.31568238314445e-05	AGT/CCL5/ERBB4/F2/F2RL1/FGFR1/GPER1/IGF1/LEP/NTRK3/RELN/SEMA3E/SOX9/TWIST1/WNT16	15	1.33928571428571
GO:2001243	BP	GO:2001243	negative regulation of intrinsic apoptotic signaling pathway	14/666	99/18614	1.12551767992515e-05	7.80242510131467e-05	4.47332912056955e-05	BCL2/BCL2L1/BDKRB2/BID/CLU/CXCL12/LRRK2/MAGEA3/MIF/MMP9/NDUFA13/PINK1/PRKN/TRAP1	14	1.41414141414141
GO:0007585	BP	GO:0007585	respiratory gaseous exchange by respiratory system	12/666	74/18614	1.14682046593862e-05	7.93890505646241e-05	4.55157655643837e-05	ATP1A2/CYSLTR1/ECEL1/EDNRA/GRP/GRPR/NDN/NLGN3/NMB/NTSR1/PHOX2B/TLX3	12	1.62162162162162
GO:0060537	BP	GO:0060537	muscle tissue development	34/666	426/18614	1.17383614503069e-05	8.11449318259612e-05	4.65224570323067e-05	ADRA1A/AGT/BCL2/CAV1/CDK1/CHRNA1/CHRND/CNTNAP1/COL3A1/DKK1/DNER/EDNRA/ERBB4/HOMER1/IGF1/MEF2C/NOX4/P2RX2/PHOX2B/POU4F1/PROX1/RGS2/RYR1/S1PR1/SHH/SOX9/TBX20/TP73/TWIST1/VAX1/VEGFA/WNT2/WNT3A/WNT5A	34	0.7981220657277
GO:0048608	BP	GO:0048608	reproductive structure development	27/666	301/18614	1.19462533641378e-05	8.24660607931704e-05	4.727989393228 4e-05	A2M/ADGRG1/ASPM/AXL/BCL2/BCL2L1/CCND1/CGA/GFRA1/IL1A/INHBA/LEP/LHB/LHX1/NKX2-1/ROBO2/SHH/SLIT2/SLIT3/SOX9/SULF1/TYRO3/VEGFA/VGF/WNT2B/WNT5A/WNT7A	27	0.897009966777409
GO:1902285	BP	GO:1902285	semaphorin-plexin signaling pathway involved in neuron projection guidance	6/666	16/18614	1.20828507445369e-05	8.32729182889851e-05	4.77424858937973e-05	EDNRA/NRP2/PLXNA1/PLXNA2/PLXNA3/SEMA3F	6	3.75
GO:0032414	BP	GO:0032414	positive regulation of ion transmembrane transporter activity	15/666	113/18614	1.20970221074945e-05	8.32729182889851e-05	4.77424858937973e-05	ADRB2/ANK3/ATP2A1/CACNG4/CCR2/DRD4/EDNRA/EPHB2/GAL/KCNA1/NLGN3/NTSR1/PINK1/PLCG2/RELN	15	1.32743362831858
GO:0003129	BP	GO:0003129	heart induction	5/666	10/18614	1.25352793843342e-05	8.46303546346187e-05	4.85207387389569e-05	DKK1/ROBO2/WNT11/WNT3A/WNT5A	5	5
GO:0035860	BP	GO:0035860	glial cell-derived neurotrophic factor receptor signaling pathway	5/666	10/18614	1.25352793843342e-05	8.46303546346187e-05	4.85207387389569e-05	GDF15/GFRA1/GFRA2/GFRA3/SULF1	5	5
GO:0042135	BP	GO:0042135	neurotransmitter catabolic process	5/666	10/18614	1.25352793843342e-05	8.46303546346187e-05	4.85207387389569e-05	BCHE/COMT/MAOA/MAOB/PRIMA1	5	5
GO:0048241	BP	GO:0048241	epinephrine transport	5/666	10/18614	1.25352793843342e-05	8.46303546346187e-05	4.85207387389569e-05	ADRA2A/ADRA2C/CARTPT/SLC22A3/VIP	5	5
GO:0048664	BP	GO:0048664	neuron fate determination	5/666	10/18614	1.25352793843342e-05	8.46303546346187e-05	4.85207387389569e-05	ATOH1/FEZF2/FOXG1/PROX1/WNT1	5	5
GO:0048672	BP	GO:0048672	positive regulation of collateral sprouting	5/666	10/18614	1.25352793843342e-05	8.46303546346187e-05	4.85207387389569e-05	BDNF/EFNA5/NGF/WNT3/WNT3A	5	5
GO:0090037	BP	GO:0090037	positive regulation of protein kinase C signaling	5/666	10/18614	1.25352793843342e-05	8.46303546346187e-05	4.85207387389569e-05	ADRA1A/MC1R/VEGFA/WNT11/WNT5A	5	5

	ONTOLOGY	ID	Description	GeneRatio	BgRatio	pvalue	p.adjust	qvalue	geneID	Count	richFactor
GO:0097090	BP	GO:0097090	presynaptic membrane organization	5/666	10/18614	1.25352793843342e-05	8.46303546346187e-05	4.85207387389569e-05	CNTN2/NLGN1/NLGN3/NLGN4X/NLGN4Y	5	5
GO:0097113	BP	GO:0097113	AMPA glutamate receptor clustering	5/666	10/18614	1.25352793843342e-05	8.46303546346187e-05	4.85207387389569e-05	CHRDL1/FRRS1L/NLGN1/SHISA7/SLC7A11	5	5
GO:0097688	BP	GO:0097688	glutamate receptor clustering	5/666	10/18614	1.25352793843342e-05	8.46303546346187e-05	4.85207387389569e-05	CHRDL1/FRRS1L/NLGN1/SHISA7/SLC7A11	5	5
GO:0098953	BP	GO:0098953	receptor diffusion trapping	5/666	10/18614	1.25352793843342e-05	8.46303546346187e-05	4.85207387389569e-05	CACNG4/CACNG5/CACNG7/CACNG8/ITGB3	5	5
GO:0098970	BP	GO:0098970	postsynaptic neurotransmitter receptor diffusion trapping	5/666	10/18614	1.25352793843342e-05	8.46303546346187e-05	4.85207387389569e-05	CACNG4/CACNG5/CACNG7/CACNG8/ITGB3	5	5
GO:0099628	BP	GO:0099628	neurotransmitter receptor diffusion trapping	5/666	10/18614	1.25352793843342e-05	8.46303546346187e-05	4.85207387389569e-05	CACNG4/CACNG5/CACNG7/CACNG8/ITGB3	5	5
GO:1990504	BP	GO:1990504	dense core granule exocytosis	5/666	10/18614	1.25352793843342e-05	8.46303546346187e-05	4.85207387389569e-05	CADPS/SNAP25/STXBP1/SYT4/UNC13C	5	5
GO:0033198	BP	GO:0033198	response to ATP	8/666	32/18614	1.26440970097825e-05	8.47827241515431e-05	4.86080960654668e-05	P2RX1/P2RX2/P2RX4/P2RX7/P2RY1/P2RY2/P2RY4/RYR3	8	2.5
GO:0034694	BP	GO:0034694	response to prostaglandin	8/666	32/18614	1.26440970097825e-05	8.47827241515431e-05	4.86080960654668e-05	A2M/CCR7/GNG2/OXT/P2RY4/PTGDR/PTGER4/PTGFR	8	2.5
GO:0050482	BP	GO:0050482	arachidonic acid secretion	8/666	32/18614	1.26440970097825e-05	8.47827241515431e-05	4.86080960654668e-05	AVPR1B/BDKRB2/DRD2/DRD4/MIF/NMB/NMUR2/NTSR1	8	2.5
GO:0140058	BP	GO:0140058	neuron projection arborization	8/666	32/18614	1.26440970097825e-05	8.47827241515431e-05	4.86080960654668e-05	CHRNA7/LRRK2/NLGN1/NTNG1/PAK6/PTN/TUBA1A/WNT5A	8	2.5
GO:1903963	BP	GO:1903963	arachidonate transport	8/666	32/18614	1.26440970097825e-05	8.47827241515431e-05	4.86080960654668e-05	AVPR1B/BDKRB2/DRD2/DRD4/MIF/NMB/NMUR2/NTSR1	8	2.5
GO:0019933	BP	GO:0019933	cAMP-mediated signaling	10/666	52/18614	1.30579627454489e-05	8.72009332797298e-05	4.99945169759248e-05	ADCY2/ADCYAP1/ADCYAP1R1/GAL/GHRHR/GNAI1/LPAR1/SCTR/SOX9/UCN	10	1.92307692307692
GO:0022602	BP	GO:0022602	ovulation cycle process	10/666	52/18614	1.30579627454489e-05	8.72009332797298e-05	4.99945169759248e-05	A2M/CGA/INHBA/KISS1/LEP/MAP2K6/NPY5R/ROBO2/SLIT2/SLIT3	10	1.92307692307692
GO:0031103	BP	GO:0031103	axon regeneration	10/666	52/18614	1.30579627454489e-05	8.72009332797298e-05	4.99945169759248e-05	BCL2/EPHA4/FKBP1B/GAP43/MAG/MAP1B/NEFL/PTN/RTN4R/TNR	10	1.92307692307692
GO:0086091	BP	GO:0086091	regulation of heart rate by cardiac conduction	9/666	42/18614	1.4079528292312e-05	9.3768132190669e-05	5.37596594473247e-05	CACNA1C/CACNA1D/CAMK2D/CAV1/HCN1/KCNJ5/KCNQ1/SCN1B/SCN2B	9	2.14285714285714
GO:1900274	BP	GO:1900274	regulation of phospholipase C activity	9/666	42/18614	1.4079528292312e-05	9.3768132190669e-05	5.37596594473247e-05	ADCYAP1R1/ADRA1A/AGT/AVPR1B/EDNRA/FGFR1/GNAQ/HTR2B/LPAR1	9	2.14285714285714
GO:0021884	BP	GO:0021884	forebrain neuron development	7/666	24/18614	1.47869721665374e-05	9.83463710399612e-05	5.63844804356109e-05	FOXG1/NDNF/NRP2/PLXNA1/PLXNA3/SEMA3E/SOX1	7	2.91666666666667
GO:0040014	BP	GO:0040014	regulation of multicellular organism growth	11/666	64/18614	1.49778136505248e-05	9.93467666563149e-05	5.69580327330845e-05	ADRB1/ADRB2/ADRB3/BCL2/CSF1/DRD2/GDF15/GHR/GHRHR/NPY1R/SLC6A3	11	1.71875
GO:0050918	BP	GO:0050918	positive chemotaxis	11/666	64/18614	1.49778136505248e-05	9.93467666563149e-05	5.69580327330845e-05	CCL5/CXCL12/CXCL8/F2RL1/GDNF/MIF/NTRK3/S1PR1/SCG2/VEGFA/WNT5A	11	1.71875
GO:0061458	BP	GO:0061458	reproductive system development	27/666	305/18614	1.51608589587881e-05	0.000100425366283616	5.75764213827148e-05	A2M/ADGRG1/ASPM/AXL/BCL2/BCL2L1/CCND1/CGA/GFRA1/IL1A/INHBA/LEP/LHB/LHX1/NKX2-1/ROBO2/SHH/SLIT2/SLIT3/SOX9/SULF1/TYRO3/VEGFA/VGF/WNT2B/WNT5A/WNT7A	27	0.885245901639344

	ONTOLOGY	ID	Description	GeneRatio	BgRatio	pvalue	p.adjust	qvalue	geneID	Count	richFactor
GO:0001823	BP	GO:0001823	mesonephros development	14/666	102/18614	1.59561016600751e-05	0.000105408924273211	6.043362216188 6e-05	AGT/BCL2/CALB1/CRLF1/GDNF/LHX1/ROBO2/SHH/SLIT2/SOX9/VEGFA/WNT1/WNT11/WNT2B	14	1.37254901960784
GO:1904063	BP	GO:1904063	negative regulation of cation transmembrane transport	14/666	102/18614	1.59561016600751e-05	0.000105408924273211	6.0433622161886e-05	ADRA2A/ANK3/ATP1A2/BCL2/CAMK2D/CAV1/DRD4/FKBP1B/GRP/MMP9/NEDD4L/NOS1/NTSR1/TWIST1	14	1.37254901960784
GO:2000191	BP	GO:2000191	regulation of fatty acid transport	8/666	33/18614	1.61703759534463e-05	0.000106681070887501	6.11629762308381e-05	AVPR1B/IL1A/MAP2K6/MIF/NTSR1/OXT/P2RX4/P2RX7	8	2.42424242424242
GO:1905475	BP	GO:1905475	regulation of protein localization to membrane	19/666	175/18614	1.71727446967084e-05	0.000113142145019198	6.48672746621821e-05	ANK3/ARHGAP44/BCL2L1/CPLX1/EPHA3/EPHB2/FRRS1L/FZD9/GPC6/GPER1/HPCA/PIK3R2/PRKN/PRNP/RAP1A/SHISA7/SLC1A1/SLC7A11/WNT3A	19	1.08571428571429
GO:0044058	BP	GO:0044058	regulation of digestive system process	9/666	43/18614	1.72463610674086e-05	0.000113475053074047	6.50581393327032e-05	KCNQ1/LEP/NEUROD1/NPSR1/OXT/OXTR/PTGER3/TACR1/UCN	9	2.09302325581395
GO:0045685	BP	GO:0045685	regulation of glial cell differentiation	12/666	77/18614	1.74156044929751e-05	0.000114379079905627	6.55764409503378e-05	CDK1/CNTN2/EPHA4/F2/IL6ST/MAG/NKX6-1/NTRK3/PTN/SHH/SOX1/TP73	12	1.55844155844156
GO:0098930	BP	GO:0098930	axonal transport	11/666	65/18614	1.74535727221202e-05	0.000114379079905627	6.55764409503378e-05	CALY/DLG2/KIF1A/KIF5A/KIF5C/MAP2/MAPT/NEFL/TMEM108/TRAK2/UCHL1	11	1.69230769230769
GO:1905330	BP	GO:1905330	regulation of morphogenesis of an epithelium	11/666	65/18614	1.74535727221202e-05	0.000114379079905627	6.55764409503378e-05	AGT/GDNF/LHX1/PIK3CD/SHH/SOX9/SULF1/VEGFA/WNT2/WNT2B/WNT5A	11	1.69230769230769
GO:0048144	BP	GO:0048144	fibroblast proliferation	14/666	103/18614	1.78688507324029e-05	0.000116944608987697	6.70473241441484e-05	AGT/CAV1/CCNB1/CDK1/COL3A1/GNG2/IGF1/ITGB3/MIF/NGFR/WNT1/WNT2/WNT5A/WNT7B	14	1.35922330097087
GO:0051969	BP	GO:0051969	regulation of transmission of nerve impulse	6/666	17/18614	1.81093358632873e-05	0.000117890577176235	6.75896718104151e-05	AGT/CARTPT/CHRNB4/FGF12/HCRT/TNR	6	3.52941176470588
GO:0060080	BP	GO:0060080	inhibitory postsynaptic potential	6/666	17/18614	1.81093358632873e-05	0.000117890577176235	6.75896718104151e-05	CHRNA4/DRD4/GRIK2/NLGN3/NTSR1/RIMS1	6	3.52941176470588
GO:0098712	BP	GO:0098712	L-glutamate import across plasma membrane	6/666	17/18614	1.81093358632873e-05	0.000117890577176235	6.75896718104151e-05	ATP1A2/NTSR1/SLC17A8/SLC1A1/SLC1A2/SLC7A11	6	3.52941176470588
GO:0098877	BP	GO:0098877	neurotransmitter receptor transport to plasma membrane	6/666	17/18614	1.81093358632873e-05	0.000117890577176235	6.75896718104151e-05	ARHGAP44/CPLX1/LRRC7/NSG1/SLC1A1/SNAP25	6	3.52941176470588
GO:0014910	BP	GO:0014910	regulation of smooth muscle cell migration	13/666	90/18614	1.82581866757127e-05	0.000118702364432709	6.80550901293274e-05	AGT/BCL2/CCL5/GNA13/IGF1/ITGB3/LPAR1/MEF2C/PLXNA1/PRKG1/SEMA6D/SLIT2/TACR1	13	1.44444444444444
GO:0014047	BP	GO:0014047	glutamate secretion	7/666	25/18614	1.99062479736733e-05	0.000128668277766277	7.37688021802912e-05	GRM2/NPY5R/NTSR1/P2RX7/SNCA/STXBP1/SYT4	7	2.8
GO:0050927	BP	GO:0050927	positive regulation of positive chemotaxis	7/666	25/18614	1.99062479736733e-05	0.000128668277766277	7.37688021802912e-05	CXCL12/CXCL8/F2RL1/NTRK3/S1PR1/SCG2/VEGFA	7	2.8
GO:0099633	BP	GO:0099633	protein localization to postsynaptic specialization membrane	7/666	25/18614	1.99062479736733e-05	0.000128668277766277	7.37688021802912e-05	CACNG7/ERBB4/GPC6/LGI1/LHFPL4/NPTX1/RAP1A	7	2.8
GO:0099645	BP	GO:0099645	neurotransmitter receptor localization to postsynaptic specialization membrane	7/666	25/18614	1.99062479736733e-05	0.000128668277766277	7.37688021802912e-05	CACNG7/ERBB4/GPC6/LGI1/LHFPL4/NPTX1/RAP1A	7	2.8
GO:0008088	BP	GO:0008088	axo-dendritic transport	12/666	78/18614	1.99219856317675e-05	0.000128668277766277	7.37688021802912e-05	CALY/DLG2/KIF1A/KIF5A/KIF5C/KLC3/MAP2/MAPT/NEFL/TMEM108/TRAK2/UCHL1	12	1.53846153846154

	ONTOLOGY	ID	Description	GeneRatio	BgRatio	pvalue	p.adjust	qvalue	geneID	Count	richFactor
GO:0070252	BP	GO:0070252	actin-mediated cell contraction	14/666	104/18614	1.99807829320671e-05	0.000128878672061824	7.38894265908877e-05	ATP1A2/ATP2A1/CACNA1C/CACNA1D/CAMK2D/CAV1/FGF12/FGF13/KCNJ5/KCNQ1/NEDD4L/PDPN/SCN1B/SCN2B	14	1.34615384615385
GO:0060074	BP	GO:0060074	synapse maturation	8/666	34/18614	2.04875432371073e-05	0.000131629117660631	7.54663271355162e-05	CAMK2B/CHRDL1/CNTNAP1/CX3CR1/NEFL/NLGN1/NRXN1/RELN	8	2.35294117647059
GO:0090075	BP	GO:0090075	relaxation of muscle	8/666	34/18614	2.04875432371073e-05	0.000131629117660631	7.54663271355162e-05	ATP1A2/ATP2A1/CAMK2D/GUCY1A1/P2RX4/P2RY1/PRKG1/RGS2	8	2.35294117647059
GO:0098801	BP	GO:0098801	regulation of renal system process	8/666	34/18614	2.04875432371073e-05	0.000131629117660631	7.54663271355162e-05	AGT/AGTR1/DRD2/EDNRB/F2RL1/OXT/SPX/TACR1	8	2.35294117647059
GO:0048512	BP	GO:0048512	circadian behavior	9/666	44/18614	2.09993978610613e-05	0.000134741567215556	7.72507737722428e-05	ADRB1/CHRNB2/CSNK1E/DRD2/GHRHR/KCNA2/MC3R/NLGN1/TH	9	2.04545454545455
GO:0021892	BP	GO:0021892	cerebral cortex GABAergic interneuron differentiation	5/666	11/18614	2.23039351997553e-05	0.000141450118073287	8.10969569164618e-05	ASCL1/CNTN2/DRD2/FEZF2/NKX2-1	5	4.54545454545454
GO:0034776	BP	GO:0034776	response to histamine	5/666	11/18614	2.23039351997553e-05	0.000141450118073287	8.10969569164618e-05	DRD2/DRD4/GABRB1/GABRB3/GABRG2	5	4.54545454545454
GO:0048484	BP	GO:0048484	enteric nervous system development	5/666	11/18614	2.23039351997553e-05	0.000141450118073287	8.10969569164618e-05	EDNRA/EDNRB/GDNF/PHOX2B/SOX10	5	4.54545454545454
GO:0060453	BP	GO:0060453	regulation of gastric acid secretion	5/666	11/18614	2.23039351997553e-05	0.000141450118073287	8.10969569164618e-05	KCNQ1/OXT/OXTR/PTGER3/UCN	5	4.54545454545454
GO:0071679	BP	GO:0071679	commissural neuron axon guidance	5/666	11/18614	2.23039351997553e-05	0.000141450118073287	8.10969569164618e-05	EPHB2/FZD3/GDNF/NCAM1/VEGFA	5	4.54545454545454
GO:0099105	BP	GO:0099105	ion channel modulating, G protein-coupled receptor signaling pathway	5/666	11/18614	2.23039351997553e-05	0.000141450118073287	8.10969569164618e-05	F2/F2RL1/F2RL2/GNA11/ITPR1	5	4.54545454545454
GO:0099566	BP	GO:0099566	regulation of postsynaptic cytosolic calcium ion concentration	5/666	11/18614	2.23039351997553e-05	0.000141450118073287	8.10969569164618e-05	CALB1/FZD9/GRIA1/ITPR1/WNT5A	5	4.54545454545454
GO:2000826	BP	GO:2000826	regulation of heart morphogenesis	5/666	11/18614	2.23039351997553e-05	0.000141450118073287	8.10969569164618e-05	DKK1/ROBO2/WNT11/WNT3A/WNT5A	5	4.54545454545454
GO:2001223	BP	GO:2001223	negative regulation of neuron migration	5/666	11/18614	2.23039351997553e-05	0.000141450118073287	8.10969569164618e-05	ADGRG1/COL3A1/DRD2/NEXMIF/NRG3	5	4.54545454545454
GO:0032946	BP	GO:0032946	positive regulation of mononuclear cell proliferation	17/666	148/18614	2.27281955913091e-05	0.000143955001715572	8.25330705343657e-05	BCL2/BCL2L1/CCL5/CCR2/CHRNB2/CSF1/EPHB2/IGF1/IL1A/IL6ST/LEP/MEF2C/MIF/RIPK2/SHH/TACR1/WNT3A	17	1.14864864864865
GO:1901216	BP	GO:1901216	positive regulation of neuron death	13/666	92/18614	2.32342322523128e-05	0.000146970722677114	8.42620602038088e-05	ASCL1/CAPN2/CLU/DDIT4/DKK1/GRIK5/GRIN2B/MAPT/NR3C1/POU4F1/PRNP/SNCA/WNT5A	13	1.41304347826087
GO:0015698	BP	GO:0015698	inorganic anion transport	19/666	179/18614	2.3643291546048e-05	0.000149366038494635	8.56353557959283e-05	CLCN1/GABRA3/GABRA4/GABRB1/GABRB3/GABRE/GABRG2/GLRA3/GLRB/KCNQ1/NMUR2/P2RY4/PRKG2/SLC17A7/SLC17A8/SLC1A1/SLC1A7/SLC4A8/SLC6A1	19	1.06145251396648
GO:0060541	BP	GO:0060541	respiratory system development	21/666	211/18614	2.38950886511463e-05	0.000150619374795395	8.63539254328367e-05	ASCL1/CELSR1/COL3A1/EDN2/EPAS1/GNG8/NKX2-1/PDPN/PHOX2B/PROX1/SHH/SLC7A11/SOX9/THRB/TP73/VEGFA/WNT11/WNT2/WNT2B/WNT5A/WNT7B	21	0.995260663507109

	ONTOLOGY	ID	Description	GeneRatio	BgRatio	pvalue	p.adjust	qvalue	geneID	Count	richFactor
GO:0098742	BP	GO:0098742	cell-cell adhesion via plasma-membrane adhesion molecules	25/666	278/18614	2.39029730092387e-05	0.000150619374795395	8.63539254328367e-05	ADGRL3/CDH4/CELSR1/CLDN1/CLDN19/CNTN2/CNTN4/CNTN6/DSCAML1/EFNA5/GRID2/IGSF9/KIRREL3/L1CAM/MAG/MDGA1/MYPN/NEXN/NLGN1/NTNG1/PALLD/ROBO2/SLITRK3/TENM1/UNC5D	25	0.899280575539568
GO:0048565	BP	GO:0048565	digestive tract development	16/666	134/18614	2.42680632992083e-05	0.000152724111543673	8.75606246404484e-05	ASCL1/BCL2/COL3A1/CXCL8/EDNRB/EPHB3/IL6ST/KCNQ1/NPY/OXTR/PYY/SHH/SOX10/SOX9/WNT11/WNT5A	16	1.19402985074627
GO:0014003	BP	GO:0014003	oligodendrocyte development	9/666	45/18614	2.54241548103327e-05	0.000159795039504841	9.16145678116311e-05	ASCL1/CLU/CNTN2/CNTNAP1/GPM6B/LPAR1/MAG/SHH/SOX10	9	2
GO:0021988	BP	GO:0021988	olfactory lobe development	8/666	35/18614	2.57314479733118e-05	0.000161108365336086	9.23675309749322e-05	ATP1A2/ERBB4/FEZF1/KIF14/LRRK2/ROBO2/SLIT2/WNT5A	8	2.28571428571429
GO:0045773	BP	GO:0045773	positive regulation of axon extension	8/666	35/18614	2.57314479733118e-05	0.000161108365336086	9.23675309749322e-05	CDH4/CXCL12/L1CAM/LIMK1/MAP1B/MAPT/NTN1/VEGFA	8	2.28571428571429
GO:0051354	BP	GO:0051354	negative regulation of oxidoreductase activity	8/666	35/18614	2.57314479733118e-05	0.000161108365336086	9.23675309749322e-05	CAV1/CNR1/CNR2/DRD5/GZMA/LRRK2/PRKN/SNCA	8	2.28571428571429
GO:0045921	BP	GO:0045921	positive regulation of exocytosis	12/666	80/18614	2.58918027524203e-05	0.000161906120264817	9.28249042027978e-05	CADPS/CDK5R2/F2RL1/NLGN1/SLC4A8/SNCA/STX1A/STXBP1/SYT10/SYT4/SYT7/SYT9	12	1.5
GO:0001696	BP	GO:0001696	gastric acid secretion	6/666	18/18614	2.63438632995022e-05	0.000163647451865854	9.38232539797378e-05	CHRM5/KCNQ1/OXT/OXTR/PTGER3/UCN	6	3.33333333333333
GO:0071318	BP	GO:0071318	cellular response to ATP	6/666	18/18614	2.63438632995022e-05	0.000163647451865854	9.38232539797378e-05	P2RX4/P2RX7/P2RY1/P2RY2/P2RY4/RYR3	6	3.33333333333333
GO:0090190	BP	GO:0090190	positive regulation of branching involved in ureteric bud morphogenesis	6/666	18/18614	2.63438632995022e-05	0.000163647451865854	9.38232539797378e-05	AGT/GDNF/LHX1/SOX9/VEGFA/WNT2B	6	3.33333333333333
GO:0002026	BP	GO:0002026	regulation of the force of heart contraction	7/666	26/18614	2.6403342691683e-05	0.000163647451865854	9.38232539797378e-05	ADRA1A/ADRB1/ATP1A2/CAMK2D/CAV1/EDN2/NOS1	7	2.69230769230769
GO:0010460	BP	GO:0010460	positive regulation of heart rate	7/666	26/18614	2.6403342691683e-05	0.000163647451865854	9.38232539797378e-05	ADRA1A/ADRA1B/ADRB1/EDN2/EDN3/KCNQ1/SLC1A1	7	2.69230769230769
GO:0050926	BP	GO:0050926	regulation of positive chemotaxis	7/666	26/18614	2.6403342691683e-05	0.000163647451865854	9.38232539797378e-05	CXCL12/CXCL8/F2RL1/NTRK3/S1PR1/SCG2/VEGFA	7	2.69230769230769
GO:1903421	BP	GO:1903421	regulation of synaptic vesicle recycling	7/666	26/18614	2.6403342691683e-05	0.000163647451865854	9.38232539797378e-05	LRRK2/NLGN1/PRKN/SLC17A7/SNCA/SYT11/SYT7	7	2.69230769230769
GO:1901655	BP	GO:1901655	cellular response to ketone	14/666	107/18614	2.76929499104315e-05	0.000171424242833464	9.82818863982832e-05	ADCY2/ADCY3/ADCY5/AXIN2/DDIT4/EFNA5/GNAI1/GNG2/NR3C1/P2RY4/PTGDR/PTGER4/PTGFR/SOX10	14	1.30841121495327
GO:0070665	BP	GO:0070665	positive regulation of leukocyte proliferation	18/666	166/18614	2.89127493917124e-05	0.000178749890893417	0.000102481867092505	BCL2/BCL2L1/CCL5/CCR2/CHRNB2/CSF1/EPHB2/IGF1/IL1A/IL6ST/LEP/MEF2C/MIF/NMB/RIPK2/SHH/TACR1/WNT3A	18	1.08433734939759
GO:1905952	BP	GO:1905952	regulation of lipid localization	19/666	182/18614	2.98438108057394e-05	0.000184274284058052	0.000105649142458297	AGT/AGTR1/APOC1/AVPR1B/CAV1/GAL/GALR1/IL1A/ITGB3/LEP/MAP2K6/MIF/NMB/NTSR1/OXT/P2RX4/P2RX7/PRKN/SHH	19	1.04395604395604
GO:0060997	BP	GO:0060997	dendritic spine morphogenesis	10/666	57/18614	3.0342353683593e-05	0.0001871175261667	0.000107279245606523	ARHGAP44/CAMK2B/EPHA4/EPHB2/EPHB3/KIF1A/LRRK2/NLGN1/RELN/WNT7A	10	1.75438596491228

	ONTOLOGY	ID	Description	GeneRatio	BgRatio	pvalue	p.adjust	qvalue	geneID	Count	richFactor
GO:2000273	BP	GO:2000273	positive regulation of signaling receptor activity	9/666	46/18614	3.06152115285055e-05	0.000188563614865419	0.000108108325104616	ADRA2A/ADRA2C/ADRB2/CACNG4/CCR2/EPHB2/NLGN3/PINK1/RELN	9	1.95652173913043
GO:0035270	BP	GO:0035270	endocrine system development	16/666	137/18614	3.19928251367612e-05	0.000196801921836272	0.000112831556407496	ASCL1/CGA/DRD2/EDNRA/GHRHR/IL6R/NEUROD1/NKX2-1/NKX6-1/NR3C1/SHH/SLC6A3/SOX2/SOX9/WNT11/WNT5A	16	1.16788321167883
GO:0010737	BP	GO:0010737	protein kinase A signaling	8/666	36/18614	3.20541790660456e-05	0.000196932862637018	0.000112906628105005	ADRB2/EDNRA/GAL/GCG/LRRK2/MC1R/MIF/PRKACB	8	2.22222222222222
GO:0009266	BP	GO:0009266	response to temperature stimulus	19/666	183/18614	3.22118165507981e-05	0.000197654280083861	0.000113320235109385	ADRB1/ADRB2/ADRB3/CALCA/CXCL12/GRIK2/HSP90AB1/HTR2B/IGF1/IL1A/IRAK1/MAPT/NGFR/NOS1/NTSR1/SCN2B/SST/TACR1/VGF	19	1.03825136612022
GO:0033002	BP	GO:0033002	muscle cell proliferation	23/666	249/18614	3.29594472642996e-05	0.000201989630054904	0.000115805801714832	AGT/APLN/CCL5/CDK1/ERBB4/GNA13/GPER1/IGF1/IL6R/IRAK1/ITGB3/MEF2C/MMP9/NPY5R/PRKG1/S1PR1/SHH/TACR1/TBX20/TP73/VIP/VIPR2/WNT2	23	0.923694779116466
GO:0048499	BP	GO:0048499	synaptic vesicle membrane organization	7/666	27/18614	3.45501220417454e-05	0.000210948881782831	0.000120942369017002	CPLX1/GRIK5/SNAP25/STX1A/STXBP1/SYP/SYT9	7	2.59259259259259
GO:0060384	BP	GO:0060384	innervation	7/666	27/18614	3.45501220417454e-05	0.000210948881782831	0.000120942369017002	EDNRA/ITGA4/NPTX1/NTF4/POU4F1/PRKCG/SULF1	7	2.59259259259259
GO:1902932	BP	GO:1902932	positive regulation of alcohol biosynthetic process	7/666	27/18614	3.45501220417454e-05	0.000210948881782831	0.000120942369017002	ADCYAP1R1/AVPR1B/GPER1/NTSR1/P2RY1/PTH1R/SNCA	7	2.59259259259259
GO:0046578	BP	GO:0046578	regulation of Ras protein signal transduction	19/666	184/18614	3.47459556389719e-05	0.000211881354795964	0.000121476979555345	ADGRG1/ADRA1A/ARHGAP44/ARRB1/COL3A1/CSF1/EPHB2/F2RL1/F2RL2/GABARAP/IGF1/KIF14/LPAR1/NGF/P2RY10/P2RY8/PIK3CG/RASAL1/RTN4R	19	1.03260869565217
GO:0045137	BP	GO:0045137	development of primary sexual characteristics	22/666	233/18614	3.48169986413378e-05	0.000212051484909758	0.00012157451948459	A2M/ADCYAP1R1/ADGRG1/ASPM/BCL2/BCL2L1/CCND1/CGA/GFRA1/IL1A/INHBA/LEP/LHB/NKX2-1/ROBO2/SLIT2/SLIT3/SOX9/VEGFA/VGF/WNT2B/WNT5A	22	0.944206008583691
GO:0001836	BP	GO:0001836	release of cytochrome c from mitochondria	10/666	58/18614	3.55027760555317e-05	0.000215960574644726	0.000123815700235355	BCL2/BCL2L1/BID/CLU/FZD9/GPER1/IGF1/MMP9/PINK1/PRKN	10	1.72413793103448
GO:0072080	BP	GO:0072080	nephron tubule development	13/666	96/18614	3.6849111398459e-05	0.000223793753421484	0.000128306661221676	AGT/BCL2/CALB1/GDNF/LHX1/MEF2C/SHH/SOX9/VEGFA/WNT1/WNT11/WNT2B/WNT7B	13	1.35416666666667
GO:0032411	BP	GO:0032411	positive regulation of transporter activity	15/666	124/18614	3.70973859918148e-05	0.000223793753421484	0.000128306661221676	ADRB2/ANK3/ATP2A1/CACNG4/CCR2/DRD4/EDNRA/EPHB2/GAL/KCNA1/NLGN3/NTSR1/PINK1/PLCG2/RELN	15	1.20967741935484
GO:0021527	BP	GO:0021527	spinal cord association neuron differentiation	5/666	12/18614	3.71092388684658e-05	0.000223793753421484	0.000128306661221676	ASCL1/LHX1/MDGA1/WNT1/WNT3A	5	4.16666666666667
GO:0032252	BP	GO:0032252	secretory granule localization	5/666	12/18614	3.71092388684658e-05	0.000223793753421484	0.000128306661221676	KIF1A/KIF5A/MAP2/PIK3CG/SYT4	5	4.16666666666667
GO:0038180	BP	GO:0038180	nerve growth factor signaling pathway	5/666	12/18614	3.71092388684658e-05	0.000223793753421484	0.000128306661221676	BDNF/MAGI2/NGF/NTF4/RAP1A	5	4.16666666666667
GO:0097048	BP	GO:0097048	dendritic cell apoptotic process	5/666	12/18614	3.71092388684658e-05	0.000223793753421484	0.000128306661221676	AXL/BCL2/BCL2L1/CCR7/CXCL12	5	4.16666666666667
GO:2000668	BP	GO:2000668	regulation of dendritic cell apoptotic process	5/666	12/18614	3.71092388684658e-05	0.000223793753421484	0.000128306661221676	AXL/BCL2/BCL2L1/CCR7/CXCL12	5	4.16666666666667

	ONTOLOGY	ID	Description	GeneRatio	BgRatio	pvalue	p.adjust	qvalue	geneID	Count	richFactor
GO:0016082	BP	GO:0016082	synaptic vesicle priming	6/666	19/18614	3.734085485156 08e-05	0.000224639292 038459	0.000128791430 055567	RPH3A/SNAP25/SNCA/STX1A/STXBP1/UNC13C	6	3.157894736842 11
GO:1900242	BP	GO:1900242	regulation of synaptic vesicle endocytosis	6/666	19/18614	3.734085485156 08e-05	0.000224639292 038459	0.000128791430 055567	LRRK2/NLGN1/PRKN/SLC17A7/SYT11/SYT7	6	3.157894736842 11
GO:0031646	BP	GO:0031646	positive regulation of nervous system process	8/666	37/18614	3.962509216411 93e-05	0.000237798935 27063	0.000136336188 835354	CARTPT/CHRNB4/HCRT/MAG/NTSR1/RIMS1/SOX10/TNR	8	2.162162162162 16
GO:0071312	BP	GO:0071312	cellular response to alkaloid	8/666	37/18614	3.962509216411 93e-05	0.000237798935 27063	0.000136336188 835354	BCL2L1/CASP7/OPRM1/RYR1/RYR3/SLC1A1/SLC1A2/TH	8	2.162162162162 16
GO:0030324	BP	GO:0030324	lung development	19/666	186/18614	4.035330782366 91e-05	0.000241873790 186992	0.000138672406 988398	ASCL1/CELSR1/COL3A1/EDN2/EPAS1/NKX2-1/PDPN/PROX1/SHH/SLC7A11/SOX9/THRB/TP73/VEGFA/WNT11/WNT2/WNT2B/WNT5A/WNT7B	19	1.021505376344 09
GO:0014909	BP	GO:0014909	smooth muscle cell migration	13/666	97/18614	4.118286712130 64e-05	0.000246245488 930926	0.000141178813 271529	AGT/BCL2/CCL5/GNA13/IGF1/ITGB3/LPAR1/MEF2C/PLXNA1/PRKG1/SEMA6D/SLIT2/TACR1	13	1.340206185567 01
GO:0034109	BP	GO:0034109	homotypic cell-cell adhesion	13/666	97/18614	4.118286712130 64e-05	0.000246245488 930926	0.000141178813 271529	ANK3/CCL5/CTSG/IL6ST/ITGB3/PDPN/PIK3CG/PRKG1/SLC7A11/STXBP1/TYRO3/VCL/WNT3A	13	1.340206185567 01
GO:0031102	BP	GO:0031102	neuron projection regeneration	10/666	59/18614	4.139508934717 47e-05	0.000247213686 684525	0.000141733905 713867	BCL2/EPHA4/FKBP1B/GAP43/MAG/MAP1B/NEFL/PTN/RTN4R/TNR	10	1.694915254237 29
GO:0008637	BP	GO:0008637	apoptotic mitochondrial changes	14/666	111/18614	4.197547949213 15e-05	0.000250375584 592022	0.000143546702 351094	ATP2A1/BCL2/BCL2L1/BID/CAMK2A/CLU/ERBB4/FZD9/GPER1/IGF1/MMP9/NPTX1/PINK1/PRKN	14	1.261261261261 26
GO:0002673	BP	GO:0002673	regulation of acute inflammatory response	9/666	48/18614	4.372324918515 92e-05	0.000260169212 766414	0.000149161638 930383	CCR7/CNR1/CREB3L3/EDNRB/IL6ST/NPY/NPY5R/PIK3CG/PTGER3	9	1.875
GO:0021795	BP	GO:0021795	cerebral cortex cell migration	9/666	48/18614	4.372324918515 92e-05	0.000260169212 766414	0.000149161638 930383	ADGRG1/CDK5R2/COL3A1/FGF13/FOXG1/MDGA1/NKX2-1/RELN/SLIT2	9	1.875
GO:0051091	BP	GO:0051091	positive regulation of DNA-binding transcription factor activity	24/666	271/18614	4.426883154522 48e-05	0.000263097106 462853	0.000150840274 990857	AGT/CAMK2A/CAV1/CLU/CX3CR1/IRAK1/IRAK2/NEUROD1/NKX6-1/NTSR1/OPRD1/PINK1/PLCG2/PRKCB/PSMA6/RELN/RIPK2/RPS6KA5/SHH/VEGFA/WNT1/WNT2/WNT3A/WNT5A	24	0.885608856088 561
GO:0002675	BP	GO:0002675	positive regulation of acute inflammatory response	7/666	28/18614	4.465318287298 11e-05	0.000265060862 102297	0.000151966146 136499	CCR7/CNR1/CREB3L3/IL6ST/NPY5R/PIK3CG/PTGER3	7	2.5
GO:0071677	BP	GO:0071677	positive regulation of mononuclear cell migration	11/666	72/18614	4.697640117878 97e-05	0.000278515092 634199	0.000159679799 321465	CCL5/CCR2/CCR7/CSF1/CX3CR1/CXCL12/CXCL13/ITGA4/ITGB3/PTK2B/WNT5A	11	1.527777777777 78
GO:0051385	BP	GO:0051385	response to mineralocorticoid	8/666	38/18614	4.863181749968 15e-05	0.000287982389 1698	0.000165107641 65713	CCND1/FOSB/GPER1/MAOB/NEFL/NTRK3/TH/UCN3	8	2.105263157894 74
GO:0071375	BP	GO:0071375	cellular response to peptide hormone stimulus	26/666	308/18614	4.884246489075 14e-05	0.000288881726 760582	0.000165623254 81353	AGT/AGTR1/APC/CAMK2A/CAV1/CYSLTR2/EDNRA/GAB1/GCGR/GDF15/GHR/GHRHR/GLP2R/GPER1/INHBA/ITGB3/LEP/MAP1B/NKX6-1/PIK3R2/PRKCB/SLC2A4/SOS2/TSHR/VAMP2/WNT1	26	0.844155844155 844
GO:0001704	BP	GO:0001704	formation of primary germ layer	15/666	127/18614	4.915685848783 58e-05	0.000290391778 206386	0.000166489005 784333	APELA/DKK1/DUSP1/HMGA2/INHBA/ITGA4/ITGB3/LHX1/MMP9/SOX2/TBX20/WNT11/WNT3/WNT3A/WNT5A	15	1.181102362204 72

	ONTOLOGY	ID	Description	GeneRatio	BgRatio	pvalue	p.adjust	qvalue	geneID	Count	richFactor
GO:0042053	BP	GO:0042053	regulation of dopamine metabolic process	6/666	20/18614	5.173562236294e-05	0.000304527645405808	0.000174593458639257	CHRNB2/DRD4/MAOB/NPY/PRKN/SLC6A3	6	3
GO:0042069	BP	GO:0042069	regulation of catecholamine metabolic process	6/666	20/18614	5.173562236294e-05	0.000304527645405808	0.000174593458639257	CHRNB2/DRD4/MAOB/NPY/PRKN/SLC6A3	6	3
GO:0042749	BP	GO:0042749	regulation of circadian sleep/wake cycle	6/666	20/18614	5.173562236294e-05	0.000304527645405808	0.000174593458639257	ADRB1/CHRNB2/DRD2/GHRHR/KCNA2/NLGN1	6	3
GO:0003170	BP	GO:0003170	heart valve development	11/666	73/18614	5.35496558855628e-05	0.000314077038994679	0.000180068369307438	APC/AXIN2/DKK1/MEF2C/OLFM1/ROBO2/SLIT2/SLIT3/SOX9/TBX20/TWIST1	11	1.50684931506849
GO:0035567	BP	GO:0035567	non-canonical Wnt signaling pathway	11/666	73/18614	5.35496558855628e-05	0.000314077038994679	0.000180068369307438	CELSR1/CSNK1E/DKK1/FZD10/FZD3/FZD9/GPC6/MAGI2/WNT11/WNT5A/WNT7A	11	1.50684931506849
GO:0070527	BP	GO:0070527	platelet aggregation	11/666	73/18614	5.35496558855628e-05	0.000314077038994679	0.000180068369307438	CTSG/IL6ST/ITGB3/PDPN/PIK3CG/PRKG1/SLC7A11/STXBP1/TYRO3/VCL/WNT3A	11	1.50684931506849
GO:0030323	BP	GO:0030323	respiratory tube development	19/666	190/18614	5.40381525422142e-05	0.000316564385870063	0.000181494428650031	ASCL1/CELSR1/COL3A1/EDN2/EPAS1/NKX2-1/PDPN/PROX1/SHH/SLC7A11/SOX9/THRB/TP73/VEGFA/WNT11/WNT2/WNT2B/WNT5A/WNT7B	19	1
GO:0086065	BP	GO:0086065	cell communication involved in cardiac conduction	10/666	61/18614	5.57139379256133e-05	0.000325992862981416	0.000186900014820635	ATP1A2/CACNA1C/CACNA1D/CAMK2D/CAV1/FKBP1B/HCN1/KCNJ5/KCNQ1/SCN1B	10	1.63934426229508
GO:0034284	BP	GO:0034284	response to monosaccharide	20/666	207/18614	5.65416968929462e-05	0.000330209922525149	0.000189317762510001	ADCY5/ADRA2A/EFNA5/FKBP1B/GCG/GHRHR/GPER1/GRIK5/IL1A/LEP/NEUROD1/NKX6-1/NOX4/NPTX1/PTK2B/SLC29A1/TH/TXNIP/UCN3/VAMP2	20	0.966183574879227
GO:0032410	BP	GO:0032410	negative regulation of transporter activity	14/666	114/18614	5.65690243674823e-05	0.000330209922525149	0.000189317762510001	ADRA2A/ANK3/ATP1A2/CAMK2D/CAV1/DRD4/EPHB2/FKBP1B/GRP/MMP9/NEDD4L/SLC6A9/SNCA/TWIST1	14	1.2280701754386
GO:0061326	BP	GO:0061326	renal tubule development	13/666	100/18614	5.69573625769833e-05	0.000331476110134742	0.000190043700129722	AGT/BCL2/CALB1/GDNF/LHX1/MEF2C/SHH/SOX9/VEGFA/WNT11/WNT2B/WNT7B	13	1.3
GO:0010996	BP	GO:0010996	response to auditory stimulus	7/666	29/18614	5.70557048166819e-05	0.000331476110134742	0.000190043700129722	ATP1A2/DRD2/NRXN1/NRXN2/PTN/TACR1/UCN	7	2.41379310344828
GO:0021952	BP	GO:0021952	central nervous system projection neuron axonogenesis	7/666	29/18614	5.70557048166819e-05	0.000331476110134742	0.000190043700129722	CHRNB2/DCLK1/EPHA4/EPHB2/EPHB3/SCN1B/SLIT2	7	2.41379310344828
GO:0099170	BP	GO:0099170	postsynaptic modulation of chemical synaptic transmission	7/666	29/18614	5.70557048166819e-05	0.000331476110134742	0.000190043700129722	DRD2/NEFH/NEFL/PTK2B/SLC1A1/SORCS3/WNT5A	7	2.41379310344828
GO:0010524	BP	GO:0010524	positive regulation of calcium ion transport into cytosol	5/666	13/18614	5.85280610209494e-05	0.000338828527582999	0.000194259028396357	ADCYAP1R1/CAV1/P2RX2/P2RX4/P2RX7	5	3.84615384615385
GO:0021979	BP	GO:0021979	hypothalamus cell differentiation	5/666	13/18614	5.85280610209494e-05	0.000338828527582999	0.000194259028396357	NDNF/NRP2/PLXNA1/PLXNA3/SEMA3E	5	3.84615384615385
GO:0035815	BP	GO:0035815	positive regulation of renal sodium excretion	5/666	13/18614	5.85280610209494e-05	0.000338828527582999	0.000194259028396357	AGT/DRD2/EDNRB/OXT/TACR1	5	3.84615384615385
GO:0032370	BP	GO:0032370	positive regulation of lipid transport	12/666	87/18614	6.06932034264885e-05	0.000350537126722904	0.000200971866624664	AVPR1B/CAV1/GAL/GALR1/IL1A/MAP2K6/MIF/NMB/NTSR1/OXT/P2RX4/P2RX7	12	1.37931034482759

	ONTOLOGY	ID	Description	GeneRatio	BgRatio	pvalue	p.adjust	qvalue	geneID	Count	richFactor
GO:0048708	BP	GO:0048708	astrocyte differentiation	12/666	87/18614	6.06932034264885e-05	0.000350537126722904	0.000200971866624664	CNTN2/EPHA4/F2/GAP43/IL6ST/MAG/MAPT/NR3C1/NTRK3/SHH/SOX9/VAX1	12	1.37931034482759
GO:0009636	BP	GO:0009636	response to toxic substance	22/666	242/18614	6.17172741997996e-05	0.000356033336492975	0.000204122983732226	BCL2/CCL5/CDK1/CLDN1/DRD2/GABRB1/GPX2/GPX3/MAOB/MAP1B/NEFL/OPRD1/PENK/PINK1/PRKCG/PRKN/PTGS1/SCN2B/SLC18A2/SLC6A1/SLC7A11/TH	22	0.909090909090909
GO:0010634	BP	GO:0010634	positive regulation of epithelial cell migration	18/666	176/18614	6.29041140741578e-05	0.000362454537719209	0.000207804422010885	AGT/AKT3/FGFR1/ITGB3/MMP9/NRP2/P2RX4/PIK3CD/PIK3CG/PLCG1/PLCG2/PROX1/PTK2B/SOX9/TACR1/VEGFA/WNT5A/WNT7A	18	1.02272727272727
GO:0043255	BP	GO:0043255	regulation of carbohydrate biosynthetic process	13/666	101/18614	6.32699196884374e-05	0.00036413542771507	0.000208768119075492	ADCYAP1R1/AVPR1B/GCG/GPER1/IGF1/LEP/LEPR/NR3C1/NTSR1/P2RY1/PTH1R/PTK2B/SNCA	13	1.28712871287129
GO:0046173	BP	GO:0046173	polyol biosynthetic process	10/666	62/18614	6.43273704787118e-05	0.00036978833438932	0.000212009074510941	ADCYAP1R1/AVPR1B/GPER1/LEP/NTSR1/P2RY1/PLCG2/PTH1R/PTK2B/SNCA	10	1.61290322580645
GO:0051656	BP	GO:0051656	establishment of organelle localization	33/666	445/18614	6.77046978712425e-05	0.000388503740157021	0.000222739093516163	ASIP/CCNB1/GPR143/GRP/ITGA4/KIF14/KIF1A/KIF5A/KIF5C/LRRK2/MAD2L1/MAP1B/MAP2/MAPT/NEFH/NEFL/NLGN1/NTN1/PIK3CD/PIK3CG/PINK1/PRKN/PTGDR/SEPTIN5/SLC18A2/SLC2A4/SNCA/STXBP1/SYT11/SYT4/TRAK2/TUBA1A/UCHL1	33	0.741573033707865
GO:0015718	BP	GO:0015718	monocarboxylic acid transport	18/666	177/18614	6.77411404505731e-05	0.000388503740157021	0.000222739093516163	AVPR1B/BDKRB2/DRD2/DRD4/FABP7/IL1A/LEP/MAP2K6/MIF/NMB/NMUR2/NTSR1/OXT/P2RX4/P2RX7/SLC22A2/SLC22A3/SPX	18	1.01694915254237
GO:0032413	BP	GO:0032413	negative regulation of ion transmembrane transporter activity	12/666	88/18614	6.80275709882984e-05	0.000389691738237164	0.000223420203086339	ADRA2A/ANK3/ATP1A2/CAMK2D/CAV1/DRD4/EPHB2/FKBP1B/GRP/MMP9/NEDD4L/SLC6A9	12	1.36363636363636
GO:0055123	BP	GO:0055123	digestive system development	16/666	146/18614	6.97285093641271e-05	0.00039897045811954	0.000228739929621675	ASCL1/BCL2/COL3A1/CXCL8/EDNRB/EPHB3/IL6ST/KCNQ1/NPY/OXTR/PYY/SHH/SOX10/SOX9/WNT11/WNT5A	16	1.0958904109589
GO:0002029	BP	GO:0002029	desensitization of G protein-coupled receptor signaling pathway	6/666	21/18614	7.02473831853607e-05	0.000399151316018553	0.000228843620063536	ADRB2/APELA/APLN/ARRB1/DRD2/HTR2B	6	2.85714285714286
GO:0008090	BP	GO:0008090	retrograde axonal transport	6/666	21/18614	7.02473831853607e-05	0.000399151316018553	0.000228843620063536	CALY/DLG2/KIF1A/KIF5A/NEFL/TMEM108	6	2.85714285714286
GO:0022410	BP	GO:0022410	circadian sleep/wake cycle process	6/666	21/18614	7.02473831853607e-05	0.000399151316018553	0.000228843620063536	ADRB1/CHRNB2/DRD2/GHRHR/KCNA2/NLGN1	6	2.85714285714286
GO:0060004	BP	GO:0060004	reflex	6/666	21/18614	7.02473831853607e-05	0.000399151316018553	0.000228843620063536	ADRA1A/ASCL1/GLRB/NMB/NPSR1/SLC1A1	6	2.85714285714286
GO:0090189	BP	GO:0090189	regulation of branching involved in ureteric bud morphogenesis	6/666	21/18614	7.02473831853607e-05	0.000399151316018553	0.000228843620063536	AGT/GDNF/LHX1/SOX9/VEGFA/WNT2B	6	2.85714285714286
GO:0097049	BP	GO:0097049	motor neuron apoptotic process	6/666	21/18614	7.02473831853607e-05	0.000399151316018553	0.000228843620063536	BCL2/CNTFR/CRLF1/FAS/NEFL/SLC1A1	6	2.85714285714286

	ONTOLOGY	ID	Description	GeneRatio	BgRatio	pvalue	p.adjust	qvalue	geneID	Count	richFactor
GO:0051146	BP	GO:0051146	striated muscle cell differentiation	25/666	297/18614	7.10432862148394e-05	0.000403207565526485	0.000231169171261851	ADRA1A/AGT/BCL2/BDNF/CAPN2/CDK1/CNTNAP1/DKK1/DNER/GDF15/HOMER1/IGF1/MEF2C/MYPN/NOS1/NOX4/P2RX2/PROX1/RGS2/RYR1/SHH/TMOD2/VEGFA/WNT1/WNT3A	25	0.841750841750842
GO:0032570	BP	GO:0032570	response to progesterone	8/666	40/18614	7.18004617385023e-05	0.000406098123641817	0.000232826401882346	CAV1/FOSB/GABRB1/OXT/OXTR/SOX10/TACR1/TXNIP	8	2
GO:0043267	BP	GO:0043267	negative regulation of potassium ion transport	8/666	40/18614	7.18004617385023e-05	0.000406098123641817	0.000232826401882346	ANK3/CAV1/GRP/HCRT/NEDD4L/NOS1/PTK2B/VIP	8	2
GO:0048489	BP	GO:0048489	synaptic vesicle transport	8/666	40/18614	7.18004617385023e-05	0.000406098123641817	0.000232826401882346	KIF5A/KIF5C/LRRK2/MAP2/PINK1/PRKN/SLC2A4/SNCA	8	2
GO:0045778	BP	GO:0045778	positive regulation of ossification	9/666	51/18614	7.20808535883402e-05	0.000407076920698169	0.000233387571175028	ADRB2/FZD9/GPM6B/MEF2C/OXT/P2RX7/PTN/TACR1/WNT5A	9	1.76470588235294
GO:0021602	BP	GO:0021602	cranial nerve morphogenesis	7/666	30/18614	7.21391653973765e-05	0.000407076920698169	0.000233387571175028	CHRNB2/EPHB2/KCNA2/NRP2/PLXNA1/PLXNA3/SEMA3F	7	2.33333333333333
GO:0008406	BP	GO:0008406	gonad development	21/666	228/18614	7.46670441072069e-05	0.000420858396544635	0.00024128884243722	A2M/ADGRG1/ASPM/BCL2/BCL2L1/CCND1/CGA/GFRA1/IL1A/INHBA/LEP/LHB/NKX2-1/ROBO2/SLIT2/SLIT3/SOX9/VEGFA/VGF/WNT2B/WNT5A	21	0.921052631578947
GO:0071887	BP	GO:0071887	leukocyte apoptotic process	14/666	117/18614	7.54098275657191e-05	0.000424558192996002	0.000243410030015591	AXL/BCL2/BCL2L1/CASP7/CCL5/CCR7/CXCL12/FAS/MEF2C/MIF/P2RX7/PIK3CD/SLC7A11/WNT5A	14	1.1965811965812
GO:0001894	BP	GO:0001894	tissue homeostasis	23/666	263/18614	7.6921916783066e-05	0.000432081395415736	0.000247723273658066	ADRB2/AKT3/BCL2/CALCA/CARTPT/CLDN1/COL3A1/CSF1/ITGB3/NEUROD1/NOX4/P2RX4/P2RX7/PTH1R/PTK2B/S1PR1/SH3GL2/SLC1A1/SLC28A2/SOX9/TUBA1A/VCL/VEGFA	23	0.874524714828897
GO:0060249	BP	GO:0060249	anatomical structure homeostasis	23/666	263/18614	7.6921916783066e-05	0.000432081395415736	0.000247723273658066	ADRB2/AKT3/BCL2/CALCA/CARTPT/CLDN1/COL3A1/CSF1/ITGB3/NEUROD1/NOX4/P2RX4/P2RX7/PTH1R/PTK2B/S1PR1/SH3GL2/SLC1A1/SLC28A2/SOX9/TUBA1A/VCL/VEGFA	23	0.874524714828897
GO:0002065	BP	GO:0002065	columnar/cuboidal epithelial cell differentiation	14/666	118/18614	8.28013314830805e-05	0.00046457596374354	0.000266353237659465	ASCL1/CAV1/EMX1/IL6ST/MAP1B/NEUROD1/NKX6-1/NPY/PROX1/PYY/SOX9/VAX1/WNT11/WNT5A	14	1.1864406779661
GO:2000106	BP	GO:2000106	regulation of leukocyte apoptotic process	12/666	90/18614	8.50146652417028e-05	0.000476450489923568	0.000273161206087785	AXL/BCL2/BCL2L1/CCL5/CCR7/CXCL12/MEF2C/MIF/P2RX7/PIK3CD/SLC7A11/WNT5A	12	1.33333333333333
GO:0051281	BP	GO:0051281	positive regulation of release of sequestered calcium ion into cytosol	8/666	41/18614	8.64376923598021e-05	0.00048332338788217	0.000277101613612586	BDKRB1/F2/GPER1/NPSR1/NTSR1/PLCG1/SNCA/TRPC1	8	1.95121951219512
GO:2000403	BP	GO:2000403	positive regulation of lymphocyte migration	8/666	41/18614	8.64376923598021e-05	0.00048332338788217	0.000277101613612586	CCL5/CCR2/CXCL12/CXCL13/ITGA4/ITGB3/PTK2B/WNT5A	8	1.95121951219512
GO:0007216	BP	GO:0007216	G protein-coupled glutamate receptor signaling pathway	5/666	14/18614	8.83669448468027e-05	0.000491872631848285	0.000282003113017692	GRIK3/GRM2/GRM4/GRM8/HOMER1	5	3.57142857142857
GO:1902287	BP	GO:1902287	semaphorin-plexin signaling pathway involved in axon guidance	5/666	14/18614	8.83669448468027e-05	0.000491872631848285	0.000282003113017692	EDNRA/PLXNA1/PLXNA2/PLXNA3/SEMA3F	5	3.57142857142857

	ONTOLOGY	ID	Description	GeneRatio	BgRatio	pvalue	p.adjust	qvalue	geneID	Count	richFactor
GO:1904321	BP	GO:1904321	response to forskolin	5/666	14/18614	8.83669448468027e-05	0.000491872631848285	0.000282003113017692	ADCY2/ADCY3/ADCY5/EFNA5/GNAI1	5	3.57142857142857
GO:1904322	BP	GO:1904322	cellular response to forskolin	5/666	14/18614	8.83669448468027e-05	0.000491872631848285	0.000282003113017692	ADCY2/ADCY3/ADCY5/EFNA5/GNAI1	5	3.57142857142857
GO:0032148	BP	GO:0032148	activation of protein kinase B activity	7/666	31/18614	9.03249052077609e-05	0.000502202385855368	0.000287925424197638	ADRA2A/ADRA2C/IGF1/NTRK3/PINK1/SLC1A1/WNT5A	7	2.25806451612903
GO:0003085	BP	GO:0003085	negative regulation of systemic arterial blood pressure	6/666	22/18614	9.36816042073783e-05	0.000517935978266889	0.000296945893627555	ADRA1A/ADRB1/ADRB2/ADRB3/CALCA/NTSR1	6	2.72727272727273
GO:0022401	BP	GO:0022401	negative adaptation of signaling pathway	6/666	22/18614	9.36816042073783e-05	0.000517935978266889	0.000296945893627555	ADRB2/APELA/APLN/ARRB1/DRD2/HTR2B	6	2.72727272727273
GO:0031629	BP	GO:0031629	synaptic vesicle fusion to presynaptic active zone membrane	6/666	22/18614	9.36816042073783e-05	0.000517935978266889	0.000296945893627555	CPLX1/GRIK5/SNAP25/STX1A/STXBP1/SYT9	6	2.72727272727273
GO:0086012	BP	GO:0086012	membrane depolarization during cardiac muscle cell action potential	6/666	22/18614	9.36816042073783e-05	0.000517935978266889	0.000296945893627555	ANK3/ATP1A2/CACNA1C/CACNA1D/SCN1B/SCN2B	6	2.72727272727273
GO:1902473	BP	GO:1902473	regulation of protein localization to synapse	6/666	22/18614	9.36816042073783e-05	0.000517935978266889	0.000296945893627555	ARHGAP44/GPC6/MAPT/RAP1A/WNT5A/WNT7A	6	2.72727272727273
GO:0019221	BP	GO:0019221	cytokine-mediated signaling pathway	35/666	492/18614	9.45015220790805e-05	0.000521882001144585	0.000299208249090122	AXL/CAV1/CCL5/CCR2/CCR7/CNTFR/CPNE1/CRLF1/CSF1/CTSG/CX3CR1/CXCL12/CXCL13/CXCL8/CXCR5/EDN2/F2RL1/FAS/GHR/IL1A/IL6R/IL6ST/IRAK1/IRAK2/LEP/LEPR/LIFR/PRKN/PTK2B/RIPK2/RPS6KA5/SLC1A1/SLIT2/SLIT3/WNT5A	35	0.711382113821138
GO:0046425	BP	GO:0046425	regulation of receptor signaling pathway via JAK-STAT	13/666	105/18614	9.498608596951e-05	0.000523969262110148	0.000300404928986211	AGT/CAMK2A/CAV1/CCL5/EPHB2/ERBB4/F2/GHR/IL6R/LEP/NEUROD1/PRL/PTK2B	13	1.23809523809524
GO:0051648	BP	GO:0051648	vesicle localization	20/666	215/18614	9.59220911307036e-05	0.000528539325008305	0.000303025062493384	ASIP/GPR143/ITGA4/KIF1A/KIF5A/KIF5C/LRRK2/MAP2/NLGN1/PIK3CG/PINK1/PRKN/SEPTIN5/SLC2A4/SNCA/SYN2/SYN3/SYT11/SYT4/TRAK2	20	0.930232558139535
GO:0048146	BP	GO:0048146	positive regulation of fibroblast proliferation	9/666	53/18614	9.85174681369838e-05	0.00054162567773297	0.000310527801957737	AGT/CCNB1/IGF1/ITGB3/MIF/NGFR/WNT1/WNT2/WNT5A	9	1.69811320754717
GO:0048260	BP	GO:0048260	positive regulation of receptor-mediated endocytosis	9/666	53/18614	9.85174681369838e-05	0.00054162567773297	0.000310527801957737	APELA/APLN/ARRB1/CLU/DRD2/MAGI2/PLCG2/VEGFA/WNT3A	9	1.69811320754717
GO:2001234	BP	GO:2001234	negative regulation of apoptotic signaling pathway	21/666	233/18614	0.000101797757031007	0.000559034609840672	0.00032050878632403	BCL2/BCL2L1/BDKRB2/BDNF/BID/CLU/CX3CR1/CXCL12/FZD9/GDNF/IGF1/IL1A/LRRK2/MAGEA3/MIF/MMP9/NDUFA13/PINK1/PRKN/SCG2/TRAP1	21	0.901287553648069
GO:0071805	BP	GO:0071805	potassium ion transmembrane transport	20/666	217/18614	0.000108947497580673	0.000597630525233269	0.000342636808063278	ANK3/ATP1A2/CACNA1D/CAV1/EDN3/GAL/GRP/HCN1/KCNA1/KCNA2/KCNJ14/KCNJ5/KCNJ9/KCNMB2/KCNQ1/KCNQ5/NEDD4L/PRNP/SLC17A7/VAMP2	20	0.921658986175115
GO:0060675	BP	GO:0060675	ureteric bud morphogenesis	10/666	66/18614	0.00011101248081598	0.000608279089420894	0.000348741901243183	AGT/BCL2/GDNF/LHX1/SHH/SOX9/VEGFA/WNT1/WNT11/WNT2B	10	1.51515151515152
GO:0061333	BP	GO:0061333	renal tubule morphogenesis	11/666	79/18614	0.000112013105221179	0.000612737750098114	0.000351298165018504	AGT/BCL2/GDNF/LHX1/MEF2C/SHH/SOX9/VEGFA/WNT1/WNT11/WNT2B	11	1.39240506329114

	ONTOLOGY	ID	Description	GeneRatio	BgRatio	pvalue	p.adjust	qvalue	geneID	Count	richFactor
GO:0010644	BP	GO:0010644	cell communication by electrical coupling	7/666	32/18614	0.000112075531503195	0.000612737750098114	0.000351298165018504	ANK3/ATP1A2/CACNA1C/CAMK2D/CAV1/FKBP1B/KCNA1	7	2.1875
GO:0010675	BP	GO:0010675	regulation of cellular carbohydrate metabolic process	16/666	152/18614	0.000112838196343701	0.000615537996702877	0.000352903617748804	ADCYAP1R1/AVPR1B/DDIT4/GCG/GCGR/GPER1/IGF1/LEP/LEPR/NR3C1/NTSR1/P2RY1/PRKN/PTH1R/PTK2B/SNCA	16	1.05263157894737
GO:0071322	BP	GO:0071322	cellular response to carbohydrate stimulus	16/666	152/18614	0.000112838196343701	0.000615537996702877	0.000352903617748804	ADCY5/ADRA2A/EFNA5/FKBP1B/GCG/GHRHR/GPER1/GRIK5/LEP/MAP2K6/NEUROD1/NOX4/NPTX1/PRKCB/SLC29A1/TH	16	1.05263157894737
GO:0060998	BP	GO:0060998	regulation of dendritic spine development	9/666	54/18614	0.000114516982808768	0.000623312259695563	0.000357360800814911	CAMK2B/EPHB2/FSTL4/GRIN3A/KIF1A/LPAR1/MEF2C/NLGN1/RELN	9	1.66666666666667
GO:0086002	BP	GO:0086002	cardiac muscle cell action potential involved in contraction	9/666	54/18614	0.000114516982808768	0.000623312259695563	0.000357360800814911	CACNA1C/CACNA1D/CAV1/FGF12/KCNJ5/KCNQ1/NEDD4L/SCN1B/SCN2B	9	1.66666666666667
GO:0007498	BP	GO:0007498	mesoderm development	15/666	137/18614	0.000117695644633624	0.000639904970546749	0.000366873824737088	APELA/DKK1/HMGA2/INHBA/ITGB3/LHX1/POU4F1/RPS6KA6/SHH/TBX20/VEGFA/WNT11/WNT3/WNT3A/WNT5A	15	1.09489051094891
GO:0098884	BP	GO:0098884	postsynaptic neurotransmitter receptor internalization	6/666	23/18614	0.000122931673592281	0.000664522219940736	0.000380987521075408	CALY/DRD4/GSG1L/HPCA/ITGB3/LPAR1	6	2.60869565217391
GO:0099500	BP	GO:0099500	vesicle fusion to plasma membrane	6/666	23/18614	0.000122931673592281	0.000664522219940736	0.000380987521075408	CPLX1/GRIK5/SNAP25/STX1A/STXBP1/SYT9	6	2.60869565217391
GO:0140239	BP	GO:0140239	postsynaptic endocytosis	6/666	23/18614	0.000122931673592281	0.000664522219940736	0.000380987521075408	CALY/DRD4/GSG1L/HPCA/ITGB3/LPAR1	6	2.60869565217391
GO:0071674	BP	GO:0071674	mononuclear cell migration	19/666	202/18614	0.000122954268015503	0.000664522219940736	0.000380987521075408	CALCA/CCL5/CCR2/CCR7/CSF1/CTSG/CX3CR1/CXCL12/CXCL13/DUSP1/IL6R/ITGA4/ITGB3/PIK3CD/PIK3CG/PTK2B/S1PR1/SLIT2/WNT5A	19	0.940594059405941
GO:0060428	BP	GO:0060428	lung epithelium development	8/666	43/18614	0.000123169835679758	0.000664522219940736	0.000380987521075408	ASCL1/NKX2-1/SHH/SOX9/THRB/TP73/WNT2/WNT7B	8	1.86046511627907
GO:0061001	BP	GO:0061001	regulation of dendritic spine morphogenesis	8/666	43/18614	0.000123169835679758	0.000664522219940736	0.000380987521075408	ARHGAP44/CAMK2B/EPHA4/EPHB2/KIF1A/LRRK2/NLGN1/RELN	8	1.86046511627907
GO:0090278	BP	GO:0090278	negative regulation of peptide hormone secretion	8/666	43/18614	0.000123169835679758	0.000664522219940736	0.000380987521075408	ADRA2A/CARTPT/DRD2/F2RL1/FKBP1B/GNAZ/LEP/PRKN	8	1.86046511627907
GO:0072028	BP	GO:0072028	nephron morphogenesis	11/666	80/18614	0.000125742588223445	0.000677658795085782	0.00038851905433306	AGT/BCL2/ERBB4/GDNF/LHX1/SHH/SOX9/VEGFA/WNT1/WNT11/WNT2B	11	1.375
GO:0072171	BP	GO:0072171	mesonephric tubule morphogenesis	10/666	67/18614	0.000126368129082209	0.000680284068388889	0.000390024190411106	AGT/BCL2/GDNF/LHX1/SHH/SOX9/VEGFA/WNT1/WNT11/WNT2B	10	1.49253731343284
GO:0002031	BP	GO:0002031	G protein-coupled receptor internalization	5/666	15/18614	0.000128656817520607	0.000688082979449168	0.00039449550484241	APELA/APLN/ARRB1/DRD2/HTR2B	5	3.33333333333333
GO:0035641	BP	GO:0035641	locomotory exploration behavior	5/666	15/18614	0.000128656817520607	0.000688082979449168	0.00039449550484241	ATP1A2/GAD1/LRRK2/PENK/TNR	5	3.33333333333333
GO:0042976	BP	GO:0042976	activation of Janus kinase activity	5/666	15/18614	0.000128656817520607	0.000688082979449168	0.00039449550484241	AGT/CCL5/GHR/IL6R/PTK2B	5	3.33333333333333
GO:0045117	BP	GO:0045117	azole transmembrane transport	5/666	15/18614	0.000128656817520607	0.000688082979449168	0.00039449550484241	SLC22A2/SLC28A2/SLC38A3/SLC38A5/SLC44A4	5	3.33333333333333
GO:0045989	BP	GO:0045989	positive regulation of striated muscle contraction	5/666	15/18614	0.000128656817520607	0.000688082979449168	0.00039449550484241	ADRA1A/ATP2A1/KCNQ1/RGS2/UCN	5	3.33333333333333

	ONTOLOGY	ID	Description	GeneRatio	BgRatio	pvalue	p.adjust	qvalue	geneID	Count	richFactor
GO:0060572	BP	GO:0060572	morphogenesis of an epithelial bud	5/666	15/18614	0.000128656817520607	0.000688082979449168	0.00039449550484241	SHH/SULF1/WNT2/WNT2B/WNT5A	5	3.33333333333333
GO:0051403	BP	GO:0051403	stress-activated MAPK cascade	21/666	237/18614	0.000129458463373787	0.000691617769002349	0.000396522089761586	AGT/CCR7/DUSP1/F2RL1/FAS/IL1A/IRAK1/LEP/LRRK2/MAP2K6/MAPK10/PINK1/PRKN/PTGER4/PTK2B/RIPK2/VEGFA/WNT16/WNT5A/WNT7A/WNT7B	21	0.886075949367089
GO:0022612	BP	GO:0022612	gland morphogenesis	14/666	123/18614	0.000129996082987383	0.000692983457573741	0.000397305073818045	BCL2/CAV1/CSF1/NR3C1/NRG3/NTN1/PLXNA1/PROX1/PTN/SHH/SOX9/SULF1/WNT3A/WNT5A	14	1.13821138211382
GO:0042471	BP	GO:0042471	ear morphogenesis	14/666	123/18614	0.000129996082987383	0.000692983457573741	0.000397305073818045	ATOH1/EDNRA/EPHB2/FOXG1/FZD3/KCNQ1/NTN1/PROX1/SLC44A4/SOX9/TWIST1/WNT1/WNT3A/WNT5A	14	1.13821138211382
GO:0071496	BP	GO:0071496	cellular response to external stimulus	26/666	327/18614	0.00013070361537905	0.000696000292078041	0.000399034701909925	AGT/ATP1A2/AXL/BCL2/BECN2/CARTPT/COMT/FAS/GABARAP/GCGR/ITGA4/ITGB3/LEP/LRRK2/MAG/MAP1LC3C/P2RX7/PENK/PTGER4/SLC1A2/SLC38A3/SOX9/UCN2/UCN3/WNT11/WNT2B	26	0.795107033639144
GO:0043647	BP	GO:0043647	inositol phosphate metabolic process	9/666	55/18614	0.000132634161879143	0.000705516131640677	0.000404490375199891	ADCYAP1R1/AVPR1B/GPER1/NTSR1/P2RY1/PLCG2/PTH1R/PTK2B/SNCA	9	1.63636363636364
GO:0032956	BP	GO:0032956	regulation of actin cytoskeleton organization	27/666	346/18614	0.000133268637473283	0.000708124706141825	0.00040598593742928	ARHGAP44/CCR7/CELSR1/CXCL12/EFNA5/EPHA3/F2RL1/FZD10/GPM6B/IL1A/ITGB3/LIMK1/LPAR1/NTRK3/PIK3R2/PRKN/PROX1/PTGER4/PTK2B/S1PR1/SEMA3E/SLIT2/SPTBN2/TACR1/TENM1/TMOD2/WNT11	27	0.780346820809249
GO:0045862	BP	GO:0045862	positive regulation of proteolysis	28/666	365/18614	0.000134584576645765	0.000714344702174877	0.000409552019643871	ADRA2A/ATP2A3/AURKA/AXIN2/BID/CAV1/CLU/CNTN2/CSNK1E/EPHA4/FAS/GABARAP/GPER1/GRIN2A/GRIN2B/LRRK2/MAPT/NDUFA13/NGFR/P2RX1/PINK1/PRKN/PTK2B/RIPK2/SLC1A1/SLC22A2/SNCA/WNT3A	28	0.767123287671233
GO:0021772	BP	GO:0021772	olfactory bulb development	7/666	33/18614	0.00013789615030505	0.000729558211786137	0.00041827431235241	ERBB4/FEZF1/KIF14/LRRK2/ROBO2/SLIT2/WNT5A	7	2.12121212121212
GO:0048665	BP	GO:0048665	neuron fate specification	7/666	33/18614	0.00013789615030505	0.000729558211786137	0.00041827431235241	ASCL1/ATOH1/NTRK3/POU4F1/SOX1/SOX9/TLX3	7	2.12121212121212
GO:0048710	BP	GO:0048710	regulation of astrocyte differentiation	7/666	33/18614	0.00013789615030505	0.000729558211786137	0.00041827431235241	CNTN2/EPHA4/F2/IL6ST/MAG/NTRK3/SHH	7	2.12121212121212
GO:0007264	BP	GO:0007264	small GTPase mediated signal transduction	34/666	483/18614	0.000144857395889718	0.000765563549245124	0.000438917089753001	ADCYAP1R1/ADGRG1/ADRA1A/ADRA2A/AGTR1/ARHGAP44/ARRB1/CELSR1/COL3A1/CSF1/EPHB2/F2RL1/F2RL2/GABARAP/GNA13/IGF1/KIF14/LIMK1/LPAR1/NGF/NGFR/NTN1/P2RY10/P2RY8/PDPN/PIK3CG/PLD1/RAB39B/RAP1A/RASAL1/RELN/RTN4R/SLIT2/SOS2	34	0.703933747412008
GO:0002792	BP	GO:0002792	negative regulation of peptide secretion	8/666	44/18614	0.000145874377417246	0.000767636579235293	0.000440105610668283	ADRA2A/CARTPT/DRD2/F2RL1/FKBP1B/GNAZ/LEP/PRKN	8	1.81818181818182
GO:0021587	BP	GO:0021587	cerebellum morphogenesis	8/666	44/18614	0.000145874377417246	0.000767636579235293	0.000440105610668283	GRID2/KIF14/LHX1/PROX1/SPTBN2/TUBA1A/WNT1/WNT7A	8	1.81818181818182

	ONTOLOGY	ID	Description	GeneRatio	BgRatio	pvalue	p.adjust	qvalue	geneID	Count	richFactor
GO:0051602	BP	GO:0051602	response to electrical stimulus	8/666	44/18614	0.000145874377417246	0.000767636579235293	0.000440105610668283	FZD3/HPCA/NSMF/OXT/P2RX7/TACR1/TACR2/TH	8	1.81818181818182
GO:1904646	BP	GO:1904646	cellular response to amyloid-beta	8/666	44/18614	0.000145874377417246	0.000767636579235293	0.000440105610668283	ADRB2/CACNA1A/EPHA4/EPHB2/IGF1/ITGA4/NGFR/PRNP	8	1.81818181818182
GO:0070661	BP	GO:0070661	leukocyte proliferation	27/666	348/18614	0.000146508057475771	0.000770146633682796	0.000441544688840573	BCL2/BCL2L1/BID/CCL5/CCR2/CHRNB2/CLU/CSF1/EPHB2/F2RL1/FKBP1B/GAL/IGF1/IL1A/IL6ST/LEP/MEF2C/MIF/NMB/P2RX7/PIK3CG/PRNP/RIPK2/SHH/SOS2/TACR1/WNT3A	27	0.775862068965517
GO:0001837	BP	GO:0001837	epithelial to mesenchymal transition	17/666	172/18614	0.000151719717155452	0.000795840351994715	0.000456275552238551	AXIN2/EDNRA/EPHA3/EPHA4/FGFR1/HMGA2/IGF1/NKX2-1/OLFM1/PDPN/SOX9/TBX20/TWIST1/WNT11/WNT16/WNT2/WNT5A	17	0.988372093023256
GO:0071453	BP	GO:0071453	cellular response to oxygen levels	17/666	172/18614	0.000151719717155452	0.000795840351994715	0.000456275552238551	BCL2/CAV1/EPAS1/EPHA4/FAS/GNGT1/IRAK1/LPAR1/NDNF/OPRD1/PINK1/SDHD/SLC29A1/SLC2A4/TWIST1/UCN3/VEGFA	17	0.988372093023256
GO:0014812	BP	GO:0014812	muscle cell migration	13/666	110/18614	0.000153236943849656	0.000802941981898782	0.0004603470976159	AGT/BCL2/CCL5/GNA13/IGF1/ITGB3/LPAR1/MEF2C/PLXNA1/PRKG1/SEMA6D/SLIT2/TACR1	13	1.18181818181818
GO:0051090	BP	GO:0051090	regulation of DNA-binding transcription factor activity	33/666	465/18614	0.000155456858624909	0.000813706560321009	0.000466518704712607	ADORA3/AGT/ARRB1/CAMK2A/CAV1/CLU/CX3CR1/IRAK1/IRAK2/LRRK2/MAPK10/NDN/NEUROD1/NKX6-1/NTSR1/OPRD1/PINK1/PLCG2/POU4F1/PRKCB/PRNP/PROX1/PSMA6/RELN/RIPK2/RPS6KA5/SHH/TWIST1/VEGFA/WNT1/WNT2/WNT3A/WNT5A	33	0.709677419354839
GO:0062012	BP	GO:0062012	regulation of small molecule metabolic process	26/666	331/18614	0.000158813824717718	0.000826863751190727	0.000474062057490513	ADCYAP1R1/APOC1/AVPR1B/CAV1/CNR1/DDIT4/GCG/GPER1/HPCA/IGF1/LEP/LEPR/NOS1/NR3C1/NTSR1/P2RX7/P2RY1/PINK1/PRKN/PROX1/PTH1R/PTK2B/SLC7A11/SNCA/SOX9/TWIST1	26	0.785498489425982
GO:0008045	BP	GO:0008045	motor neuron axon guidance	6/666	24/18614	0.000158979907400862	0.000826863751190727	0.000474062057490513	EPHA4/KIF5C/LHX1/PLXNA3/SEMA3F/SLIT2	6	2.5
GO:0023058	BP	GO:0023058	adaptation of signaling pathway	6/666	24/18614	0.000158979907400862	0.000826863751190727	0.000474062057490513	ADRB2/APELA/APLN/ARRB1/DRD2/HTR2B	6	2.5
GO:0031338	BP	GO:0031338	regulation of vesicle fusion	6/666	24/18614	0.000158979907400862	0.000826863751190727	0.000474062057490513	CPLX1/GRIK5/SNCA/STXBP1/SYT4/SYT9	6	2.5
GO:0034695	BP	GO:0034695	response to prostaglandin E	6/666	24/18614	0.000158979907400862	0.000826863751190727	0.000474062057490513	A2M/CCR7/GNG2/OXT/P2RY4/PTGER4	6	2.5
GO:1900273	BP	GO:1900273	positive regulation of long-term synaptic potentiation	6/666	24/18614	0.000158979907400862	0.000826863751190727	0.000474062057490513	CHRNA7/DRD2/EPHB2/RELN/SHISA7/SLC18A3	6	2.5
GO:0032835	BP	GO:0032835	glomerulus development	10/666	69/18614	0.000162502757745163	0.000844292869257373	0.000484054614981418	BCL2/EDNRA/EDNRB/IL6R/ITGB3/KIRREL3/LHX1/MAGI2/MEF2C/SULF1	10	1.44927536231884
GO:0007259	BP	GO:0007259	receptor signaling pathway via JAK-STAT	17/666	173/18614	0.000162785597192703	0.000844869282156425	0.000484385087183755	AGT/CAMK2A/CAV1/CCL5/CCR2/CRLF1/EPHB2/ERBB4/F2/GHR/IGF1/IL6R/IL6ST/LEP/NEUROD1/PRL/PTK2B	17	0.982658959537572

	ONTOLOGY	ID	Description	GeneRatio	BgRatio	pvalue	p.adjust	qvalue	geneID	Count	richFactor
GO:0001678	BP	GO:0001678	intracellular glucose homeostasis	16/666	157/18614	0.000165002900972398	0.000855473901138542	0.000490464985457595	ADCY5/ADRA2A/CARTPT/EFNA5/FKBP1B/GCG/GHRHR/GPER1/GRIK5/NEUROD1/NGFR/NOX4/NPTX1/PIK3R2/SLC29A1/TH	16	1.01910828025478
GO:0003206	BP	GO:0003206	cardiac chamber morphogenesis	14/666	126/18614	0.000168281977838063	0.000867262666061195	0.00049722378477182	EDNRA/MEF2C/NPY2R/NPY5R/NRP2/POU4F1/PROX1/ROBO2/SLIT2/SLIT3/TBX20/WNT11/WNT2/WNT5A	14	1.11111111111111
GO:0033238	BP	GO:0033238	regulation of amine metabolic process	7/666	34/18614	0.000168335418804146	0.000867262666061195	0.00049722378477182	CHRNB2/DRD4/MAOB/NPY/PRKN/SLC6A3/SLC7A11	7	2.05882352941176
GO:0060914	BP	GO:0060914	heart formation	7/666	34/18614	0.000168335418804146	0.000867262666061195	0.00049722378477182	AXIN2/DKK1/MEF2C/ROBO2/WNT11/WNT3A/WNT5A	7	2.05882352941176
GO:0099174	BP	GO:0099174	regulation of presynapse organization	7/666	34/18614	0.000168335418804146	0.000867262666061195	0.00049722378477182	DKK1/MDGA1/NTRK3/SLITRK3/SNCA/WNT3A/WNT7A	7	2.05882352941176
GO:1905606	BP	GO:1905606	regulation of presynapse assembly	7/666	34/18614	0.000168335418804146	0.000867262666061195	0.00049722378477182	DKK1/MDGA1/NTRK3/SLITRK3/SNCA/WNT3A/WNT7A	7	2.05882352941176
GO:2000406	BP	GO:2000406	positive regulation of T cell migration	7/666	34/18614	0.000168335418804146	0.000867262666061195	0.00049722378477182	CCL5/CCR2/CXCL12/CXCL13/ITGA4/ITGB3/WNT5A	7	2.05882352941176
GO:0061098	BP	GO:0061098	positive regulation of protein tyrosine kinase activity	8/666	45/18614	0.000171917668791714	0.000884790934147929	0.000507273187495638	ADRA1A/ADRA2A/AGT/EPHA4/NOX4/PRNP/RELN/WNT3A	8	1.77777777777778
GO:0007492	BP	GO:0007492	endoderm development	11/666	83/18614	0.000175837753869001	0.00090158895621385	0.000516903921568663	APELA/DKK1/DUSP1/HMGA2/INHBA/ITGA4/LHX1/MMP9/NKX2-1/SOX2/TBX20	11	1.32530120481928
GO:2001056	BP	GO:2001056	positive regulation of cysteine-type endopeptidase activity	15/666	142/18614	0.000176006330988367	0.00090158895621385	0.000516903921568663	ATP2A3/BID/FAS/GPER1/GRIN2A/GRIN2B/MAPT/NDUFA13/NGFR/P2RX1/RIPK2/SLC1A1/SLC22A2/SNCA/WNT3A	15	1.05633802816901
GO:0001706	BP	GO:0001706	endoderm formation	9/666	57/18614	0.000176098758487344	0.00090158895621385	0.000516903921568663	DKK1/DUSP1/HMGA2/INHBA/ITGA4/LHX1/MMP9/SOX2/TBX20	9	1.57894736842105
GO:0010676	BP	GO:0010676	positive regulation of cellular carbohydrate metabolic process	9/666	57/18614	0.000176098758487344	0.00090158895621385	0.000516903921568663	ADCYAP1R1/AVPR1B/GCG/GPER1/IGF1/NTSR1/P2RY1/PTH1R/SNCA	9	1.57894736842105
GO:0032768	BP	GO:0032768	regulation of monooxygenase activity	9/666	57/18614	0.000176098758487344	0.00090158895621385	0.000516903921568663	CAV1/CNR1/CNR2/GDNF/HTR2B/IL1A/LEP/PTK2B/SNCA	9	1.57894736842105
GO:0042692	BP	GO:0042692	muscle cell differentiation	30/666	410/18614	0.000181040533533157	0.000925165904170849	0.000530421186585718	ADRA1A/AGT/BCL2/BDNF/CAPN2/CDK1/CNTNAP1/DKK1/DNER/EDNRA/EDNRB/GDF15/GPER1/HOMER1/IGF1/MEF2C/MYPN/NOS1/NOX4/P2RX2/PROX1/RGS2/RYR1/SHH/SOX9/TMOD2/UCHL1/VEGFA/WNT1/WNT3A	30	0.731707317073171
GO:0045187	BP	GO:0045187	regulation of circadian sleep/wake cycle, sleep	5/666	16/18614	0.000181644984237003	0.000925165904170849	0.000530421186585718	ADRB1/CHRNB2/DRD2/GHRHR/KCNA2	5	3.125
GO:0048532	BP	GO:0048532	anatomical structure arrangement	5/666	16/18614	0.000181644984237003	0.000925165904170849	0.000530421186585718	KCNA2/KIF14/NRP2/PLXNA3/SEMA3F	5	3.125
GO:0098969	BP	GO:0098969	neurotransmitter receptor transport to postsynaptic membrane	5/666	16/18614	0.000181644984237003	0.000925165904170849	0.000530421186585718	ARHGAP44/CPLX1/LRRC7/NSG1/SNAP25	5	3.125
GO:0099149	BP	GO:0099149	regulation of postsynaptic neurotransmitter receptor internalization	5/666	16/18614	0.000181644984237003	0.000925165904170849	0.000530421186585718	DRD4/GSG1L/HPCA/ITGB3/LPAR1	5	3.125
GO:0014902	BP	GO:0014902	myotube differentiation	14/666	127/18614	0.00018304084218234	0.000931293782852292	0.000533934455575432	BCL2/BDNF/CAPN2/CNTNAP1/DNER/GDF15/HOMER1/IGF1/MEF2C/NOS1/P2RX2/RYR1/SHH/WNT1	14	1.10236220472441

	ONTOLOGY	ID	Description	GeneRatio	BgRatio	pvalue	p.adjust	qvalue	geneID	Count	richFactor
GO:0032944	BP	GO:0032944	regulation of mononuclear cell proliferation	21/666	243/18614	0.000183429263344695	0.000931293782852292	0.000533934455575432	BCL2/BCL2L1/BID/CCL5/CCR2/CHRNB2/CSF1/EPHB2/GAL/IGF1/IL1A/IL6ST/LEP/MEF2C/MIF/PRNP/RIPK2/SHH/SOS2/TACR1/WNT3A	21	0.864197530864197
GO:0034394	BP	GO:0034394	protein localization to cell surface	10/666	70/18614	0.000183606037758672	0.000931293782852292	0.000533934455575432	BDNF/EPHB2/ERBB4/GPIHBP1/GPM6B/HSP90AB1/NEDD4L/TMEM35A/VCL/WNT11	10	1.42857142857143
GO:0051881	BP	GO:0051881	regulation of mitochondrial membrane potential	10/666	70/18614	0.000183606037758672	0.000931293782852292	0.000533934455575432	BCL2/BCL2L1/BID/FZD9/LRRK2/MAPT/OPRD1/P2RX7/PINK1/PRKN	10	1.42857142857143
GO:0009755	BP	GO:0009755	hormone-mediated signaling pathway	18/666	192/18614	0.000191538516863996	0.000970527639573755	0.000556428225333607	ASIP/CGA/DNAAF4/GCGR/GHR/GHRHR/GPER1/LHB/NR3C1/SST/SSTR1/SSTR2/SSTR3/SSTR5/THRB/TSHR/UCN2/UCN3	18	0.9375
GO:0051056	BP	GO:0051056	regulation of small GTPase mediated signal transduction	24/666	298/18614	0.000193314647115316	0.000978518527880304	0.000561009605211881	ADCYAP1R1/ADGRG1/ADRA1A/ARHGAP44/ARRB1/COL3A1/CSF1/EPHB2/F2RL1/F2RL2/GABARAP/GNA13/IGF1/KIF14/LPAR1/NGF/P2RY10/P2RY8/PIK3CG/RASAL1/RELN/RTN4R/SLIT2/SOS2	24	0.805369127516778
GO:0048660	BP	GO:0048660	regulation of smooth muscle cell proliferation	17/666	176/18614	0.000200323362654036	0.00101295198296768	0.000580751182396421	AGT/APLN/CCL5/GNA13/GPER1/IGF1/IL6R/IRAK1/ITGB3/MEF2C/MMP9/NPY5R/PRKG1/S1PR1/TACR1/VIP/VIPR2	17	0.965909090909091
GO:0003081	BP	GO:0003081	regulation of systemic arterial blood pressure by renin-angiotensin	6/666	25/18614	0.000202897905114711	0.00102071975807452	0.000585204645792256	AGT/AGTR1/CTSG/EDNRB/F2RL1/TACR1	6	2.4
GO:0006582	BP	GO:0006582	melanin metabolic process	6/666	25/18614	0.000202897905114711	0.00102071975807452	0.000585204645792256	ASIP/BCL2/MC1R/SLC7A11/TRPC1/WNT5A	6	2.4
GO:0022038	BP	GO:0022038	corpus callosum development	6/666	25/18614	0.000202897905114711	0.00102071975807452	0.000585204645792256	EPHB2/EPHB3/KCNA1/KCNA2/LPAR1/RTN4R	6	2.4
GO:0030318	BP	GO:0030318	melanocyte differentiation	6/666	25/18614	0.000202897905114711	0.00102071975807452	0.000585204645792256	BCL2/EDN3/EDNRB/GNA11/MEF2C/SOX10	6	2.4
GO:0086010	BP	GO:0086010	membrane depolarization during action potential	6/666	25/18614	0.000202897905114711	0.00102071975807452	0.000585204645792256	ANK3/ATP1A2/CACNA1C/CACNA1D/SCN1B/SCN2B	6	2.4
GO:0070528	BP	GO:0070528	protein kinase C signaling	7/666	35/18614	0.000203986411511855	0.00102514643413167	0.000587742571969903	ADGRG1/ADRA1A/HTR2B/MC1R/VEGFA/WNT11/WNT5A	7	2
GO:0031098	BP	GO:0031098	stress-activated protein kinase signaling cascade	21/666	245/18614	0.000205382479500193	0.00103052961181566	0.000590828884902396	AGT/CCR7/DUSP1/F2RL1/FAS/IL1A/IRAK1/LEP/LRRK2/MAP2K6/MAPK10/PINK1/PRKN/PTGER4/PTK2B/RIPK2/VEGFA/WNT16/WNT5A/WNT7A/WNT7B	21	0.857142857142857
GO:1905039	BP	GO:1905039	carboxylic acid transmembrane transport	16/666	160/18614	0.000205476911409836	0.00103052961181566	0.000590828884902396	AGT/ATP1A2/NTSR1/RGS2/SLC17A7/SLC17A8/SLC1A1/SLC1A2/SLC1A7/SLC22A2/SLC38A3/SLC38A5/SLC6A15/SLC6A6/SLC6A9/SLC7A11	16	1
GO:0072678	BP	GO:0072678	T cell migration	10/666	71/18614	0.000206966236543056	0.00103694093028452	0.000594504657144439	CCL5/CCR2/CXCL12/CXCL13/ITGA4/ITGB3/PIK3CD/PIK3CG/S1PR1/WNT5A	10	1.40845070422535

	ONTOLOGY	ID	Description	GeneRatio	BgRatio	pvalue	p.adjust	qvalue	geneID	Count	richFactor
GO:0030336	BP	GO:0030336	negative regulation of cell migration	28/666	375/18614	0.000211159769552239	0.00105687399933733	0.000605932793537725	ADGRG1/ADORA3/BCL2/CLDN19/COL3A1/CX3CR1/CXCL12/CXCL13/DRD2/DUSP1/EPHA4/GNA13/MAGI2/MEF2C/MIF/NEXMIF/NGFR/NKX2-1/NRG3/PRKG1/PTGER4/SEMA6D/SHH/SLIT2/SLURP1/SULF1/VCL/WNT11	28	0.746666666666667
GO:0048568	BP	GO:0048568	embryonic organ development	32/666	453/18614	0.000211589132007668	0.00105794566003834	0.000606547203924106	A2M/APELA/ATOH1/CXCL8/DSCAML1/EDNRA/EPAS1/EPHB2/FOXG1/FZD3/GDNF/KCNQ1/LHX1/MEF2C/NEUROD1/NTN1/PROX1/SHH/SLC44A4/SOX9/TBX20/TH/TUBB2B/TWIST1/VEGFA/WNT1/WNT11/WNT16/WNT2/WNT3A/WNT5A/WNT7B	32	0.706401766004415
GO:1990868	BP	GO:1990868	response to chemokine	12/666	99/18614	0.000214214549852349	0.00106889798225817	0.000612826449323793	CCL5/CCR2/CCR7/CX3CR1/CXCL12/CXCL13/CXCL8/CXCR5/DUSP1/PTK2B/SLIT2/SLIT3	12	1.21212121212121
GO:1990869	BP	GO:1990869	cellular response to chemokine	12/666	99/18614	0.000214214549852349	0.00106889798225817	0.000612826449323793	CCL5/CCR2/CCR7/CX3CR1/CXCL12/CXCL13/CXCL8/CXCR5/DUSP1/PTK2B/SLIT2/SLIT3	12	1.21212121212121
GO:0009749	BP	GO:0009749	response to glucose	18/666	194/18614	0.000217930543102807	0.00108633734214026	0.00062282487870846	ADCY5/ADRA2A/EFNA5/FKBP1B/GCG/GHRHR/GPER1/GRIK5/NEUROD1/NKX6-1/NOX4/NPTX1/PTK2B/SLC29A1/TH/TXNIP/UCN3/VAMP2	18	0.927835051546392
GO:1903825	BP	GO:1903825	organic acid transmembrane transport	16/666	161/18614	0.000220759878389524	0.00109932604081511	0.000630271630617773	AGT/ATP1A2/NTSR1/RGS2/SLC17A7/SLC17A8/SLC1A1/SLC1A2/SLC1A7/SLC22A2/SLC38A3/SLC38A5/SLC6A15/SLC6A6/SLC6A9/SLC7A11	16	0.993788819875776
GO:0050671	BP	GO:0050671	positive regulation of lymphocyte proliferation	15/666	145/18614	0.000221856101757635	0.00110366674103115	0.000632760264655007	BCL2/CCL5/CCR2/CHRNB2/EPHB2/IGF1/IL1A/IL6ST/LEP/MEF2C/MIF/RIPK2/SHH/TACR1/WNT3A	15	1.03448275862069
GO:0021575	BP	GO:0021575	hindbrain morphogenesis	8/666	47/18614	0.000235509406575445	0.00116878160269286	0.000670092274007344	GRID2/KIF14/LHX1/PROX1/SPTBN2/TUBA1A/WNT1/WNT7A	8	1.70212765957447
GO:0090199	BP	GO:0090199	regulation of release of cytochrome c from mitochondria	8/666	47/18614	0.000235509406575445	0.00116878160269286	0.000670092274007344	BCL2L1/BID/CLU/GPER1/IGF1/MMP9/PINK1/PRKN	8	1.70212765957447
GO:0060419	BP	GO:0060419	heart growth	12/666	100/18614	0.000235658711753535	0.00116878160269286	0.000670092274007344	ADRA1A/AGT/CDK1/ERBB4/IGF1/MEF2C/PROX1/RGS2/S1PR1/TBX20/TP73/WNT2	12	1.2
GO:0021696	BP	GO:0021696	cerebellar cortex morphogenesis	7/666	36/18614	0.000245487295205672	0.00121507558503109	0.000696633793677408	GRID2/KIF14/LHX1/PROX1/SPTBN2/TUBA1A/WNT7A	7	1.94444444444444
GO:1905332	BP	GO:1905332	positive regulation of morphogenesis of an epithelium	7/666	36/18614	0.000245487295205672	0.00121507558503109	0.000696633793677408	AGT/GDNF/LHX1/PIK3CD/SOX9/VEGFA/WNT2B	7	1.94444444444444
GO:0021542	BP	GO:0021542	dentate gyrus development	5/666	17/18614	0.000249784837726052	0.00123015278298953	0.000705277935442086	FEZF2/NEUROD1/PROX1/TMEM108/TUBA1A	5	2.94117647058824
GO:0032274	BP	GO:0032274	gonadotropin secretion	5/666	17/18614	0.000249784837726052	0.00123015278298953	0.000705277935442086	CGA/INHBA/KISS1/LEP/TACR2	5	2.94117647058824
GO:0034112	BP	GO:0034112	positive regulation of homotypic cell-cell adhesion	5/666	17/18614	0.000249784837726052	0.00123015278298953	0.000705277935442086	ANK3/CCL5/CTSG/IL6ST/PDPN	5	2.94117647058824
GO:0060457	BP	GO:0060457	negative regulation of digestive system process	5/666	17/18614	0.000249784837726052	0.00123015278298953	0.000705277935442086	NPSR1/OXT/OXTR/PTGER3/UCN	5	2.94117647058824

	ONTOLOGY	ID	Description	GeneRatio	BgRatio	pvalue	p.adjust	qvalue	geneID	Count	richFactor
GO:1903540	BP	GO:1903540	establishment of protein localization to postsynaptic membrane	5/666	17/18614	0.000249784837726052	0.00123015278298953	0.000705277935442086	ARHGAP44/CPLX1/LRRC7/NSG1/SNAP25	5	2.94117647058824
GO:0010595	BP	GO:0010595	positive regulation of endothelial cell migration	14/666	131/18614	0.000253795726211093	0.00124740599432752	0.000715169641123258	AGT/AKT3/FGFR1/ITGB3/NRP2/P2RX4/PIK3CD/PIK3CG/PLCG1/PROX1/PTK2B/VEGFA/WNT5A/WNT7A	14	1.06870229007634
GO:0030048	BP	GO:0030048	actin filament-based movement	14/666	131/18614	0.000253795726211093	0.00124740599432752	0.000715169641123258	ATP1A2/ATP2A1/CACNA1C/CACNA1D/CAMK2D/CAV1/FGF12/FGF13/KCNJ5/KCNQ1/NEDD4L/PDPN/SCN1B/SCN2B	14	1.06870229007634
GO:0002438	BP	GO:0002438	acute inflammatory response to antigenic stimulus	6/666	26/18614	0.00025584627384162	0.00125122829445926	0.000717361063183049	A2M/CCR7/CNR1/NPY/NPY5R/OPRM1	6	2.30769230769231
GO:0014829	BP	GO:0014829	vascular associated smooth muscle contraction	6/666	26/18614	0.00025584627384162	0.00125122829445926	0.000717361063183049	AGT/EDN2/EDN3/EDNRA/EDNRB/P2RX1	6	2.30769230769231
GO:0021854	BP	GO:0021854	hypothalamus development	6/666	26/18614	0.00025584627384162	0.00125122829445926	0.000717361063183049	NDNF/NKX2-1/NRP2/PLXNA1/PLXNA3/SEMA3E	6	2.30769230769231
GO:0022010	BP	GO:0022010	central nervous system myelination	6/666	26/18614	0.00025584627384162	0.00125122829445926	0.000717361063183049	CLU/CNTN2/CNTNAP1/GPM6B/MAG/SOX10	6	2.30769230769231
GO:0032291	BP	GO:0032291	axon ensheathment in central nervous system	6/666	26/18614	0.00025584627384162	0.00125122829445926	0.000717361063183049	CLU/CNTN2/CNTNAP1/GPM6B/MAG/SOX10	6	2.30769230769231
GO:1904892	BP	GO:1904892	regulation of receptor signaling pathway via STAT	13/666	116/18614	0.000261519268381711	0.00127770099810746	0.000732538538723281	AGT/CAMK2A/CAV1/CCL5/EPHB2/ERBB4/F2/GHR/IL6R/LEP/NEUROD1/PRL/PTK2B	13	1.12068965517241
GO:0048659	BP	GO:0048659	smooth muscle cell proliferation	17/666	180/18614	0.000261973157325321	0.00127864753550541	0.000733081212731807	AGT/APLN/CCL5/GNA13/GPER1/IGF1/IL6R/IRAK1/ITGB3/MEF2C/MMP9/NPY5R/PRKG1/S1PR1/TACR1/VIP/VIPR2	17	0.944444444444444
GO:0001658	BP	GO:0001658	branching involved in ureteric bud morphogenesis	9/666	60/18614	0.000263061312634231	0.00128014490257153	0.000733939691424531	AGT/BCL2/GDNF/LHX1/SHH/SOX9/VEGFA/WNT1/WNT2B	9	1.5
GO:0003179	BP	GO:0003179	heart valve morphogenesis	9/666	60/18614	0.000263061312634231	0.00128014490257153	0.000733939691424531	AXIN2/MEF2C/OLFM1/ROBO2/SLIT2/SLIT3/SOX9/TBX20/TWIST1	9	1.5
GO:0071709	BP	GO:0071709	membrane assembly	9/666	60/18614	0.000263061312634231	0.00128014490257153	0.000733939691424531	ANK3/CAV1/EPHB2/NLGN1/NLGN3/NLGN4X/NLGN4Y/NRXN1/NRXN2	9	1.5
GO:0070663	BP	GO:0070663	regulation of leukocyte proliferation	22/666	268/18614	0.000270081512994725	0.00131300755328296	0.0007527806864354	BCL2/BCL2L1/BID/CCL5/CCR2/CHRNB2/CSF1/EPHB2/GAL/IGF1/IL1A/IL6ST/LEP/MEF2C/MIF/NMB/PRNP/RIPK2/SHH/SOS2/TACR1/WNT3A	22	0.82089552238806
GO:2001233	BP	GO:2001233	regulation of apoptotic signaling pathway	28/666	381/18614	0.000273848668746797	0.00133000613329101	0.000762526405486972	AGT/BCL2/BCL2L1/BDKRB2/BDNF/BID/CAV1/CLU/CX3CR1/CXCL12/FAS/FGFR1/FZD9/GDNF/GPER1/IGF1/IL1A/INHBA/LRRK2/MAGEA3/MIF/MMP9/NDUFA13/NGFR/PINK1/PRKN/SCG2/TRAP1	28	0.73490813648294
GO:0021510	BP	GO:0021510	spinal cord development	12/666	102/18614	0.000284064973401573	0.00137114203558217	0.000786110666435397	ASCL1/GDNF/LHX1/MDGA1/NEFL/PROX1/RELN/SHH/SOX1/TBX20/WNT1/WNT3A	12	1.17647058823529
GO:0042472	BP	GO:0042472	inner ear morphogenesis	12/666	102/18614	0.000284064973401573	0.00137114203558217	0.000786110666435397	ATOH1/EPHB2/FOXG1/FZD3/KCNQ1/NTN1/PROX1/SLC44A4/SOX9/WNT1/WNT3A/WNT5A	12	1.17647058823529
GO:0001820	BP	GO:0001820	serotonin secretion	4/666	10/18614	0.000287061069097468	0.00137114203558217	0.000786110666435397	CNR1/MAOB/P2RX1/SLC18A2	4	4

	ONTOLOGY	ID	Description	GeneRatio	BgRatio	pvalue	p.adjust	qvalue	geneID	Count	richFactor
GO:0006171	BP	GO:0006171	cAMP biosynthetic process	4/666	10/18614	0.000287061069097468	0.00137114203558217	0.000786110666435397	ADCY2/ADCY3/ADCY5/ADCY9	4	4
GO:0008343	BP	GO:0008343	adult feeding behavior	4/666	10/18614	0.000287061069097468	0.00137114203558217	0.000786110666435397	ASIP/CARTPT/LEP/NPY	4	4
GO:0021604	BP	GO:0021604	cranial nerve structural organization	4/666	10/18614	0.000287061069097468	0.00137114203558217	0.000786110666435397	KCNA2/NRP2/PLXNA3/SEMA3F	4	4
GO:0032096	BP	GO:0032096	negative regulation of response to food	4/666	10/18614	0.000287061069097468	0.00137114203558217	0.000786110666435397	CARTPT/LEP/SPX/UCN	4	4
GO:0032099	BP	GO:0032099	negative regulation of appetite	4/666	10/18614	0.000287061069097468	0.00137114203558217	0.000786110666435397	CARTPT/LEP/SPX/UCN	4	4
GO:0032105	BP	GO:0032105	negative regulation of response to extracellular stimulus	4/666	10/18614	0.000287061069097468	0.00137114203558217	0.000786110666435397	CARTPT/LEP/SPX/UCN	4	4
GO:0032108	BP	GO:0032108	negative regulation of response to nutrient levels	4/666	10/18614	0.000287061069097468	0.00137114203558217	0.000786110666435397	CARTPT/LEP/SPX/UCN	4	4
GO:0032275	BP	GO:0032275	luteinizing hormone secretion	4/666	10/18614	0.000287061069097468	0.00137114203558217	0.000786110666435397	CGA/KISS1/LEP/TACR2	4	4
GO:0051967	BP	GO:0051967	negative regulation of synaptic transmission, glutamatergic	4/666	10/18614	0.000287061069097468	0.00137114203558217	0.000786110666435397	DRD2/GRIK2/GRIK3/HCN1	4	4
GO:0060068	BP	GO:0060068	vagina development	4/666	10/18614	0.000287061069097468	0.00137114203558217	0.000786110666435397	AXL/LHX1/TYRO3/WNT5A	4	4
GO:0060452	BP	GO:0060452	positive regulation of cardiac muscle contraction	4/666	10/18614	0.000287061069097468	0.00137114203558217	0.000786110666435397	ADRA1A/KCNQ1/RGS2/UCN	4	4
GO:0098917	BP	GO:0098917	retrograde trans-synaptic signaling	4/666	10/18614	0.000287061069097468	0.00137114203558217	0.000786110666435397	CNR1/GUCY1A1/NOS1/SYT4	4	4
GO:0098976	BP	GO:0098976	excitatory chemical synaptic transmission	4/666	10/18614	0.000287061069097468	0.00137114203558217	0.000786110666435397	CHRDL1/GRIN2A/GRIN2B/GRIN2D	4	4
GO:1901725	BP	GO:1901725	regulation of histone deacetylase activity	4/666	10/18614	0.000287061069097468	0.00137114203558217	0.000786110666435397	CAMK2D/LRRK2/PINK1/VEGFA	4	4
GO:0009746	BP	GO:0009746	response to hexose	18/666	199/18614	0.000298238109360016	0.001423145929616	0.000815925823972164	ADCY5/ADRA2A/EFNA5/FKBP1B/GCG/GHRHR/GPER1/GRIK5/NEUROD1/NKX6-1/NOX4/NPTX1/PTK2B/SLC29A1/TH/TXNIP/UCN3/VAMP2	18	0.904522613065327
GO:1905517	BP	GO:1905517	macrophage migration	9/666	61/18614	0.000298948358256877	0.00142515148480364	0.000817075659863862	CCL5/CCR2/CSF1/CX3CR1/EDN2/EDNRB/MIF/P2RX4/PTK2B	9	1.47540983606557
GO:0007528	BP	GO:0007528	neuromuscular junction development	8/666	49/18614	0.000317181144853021	0.00150914358853107	0.000865230473094756	ANK3/CHRNA1/CNTNAP1/FZD9/LRRK2/P2RX2/SLC18A3/UNC13C	8	1.63265306122449
GO:0046850	BP	GO:0046850	regulation of bone remodeling	8/666	49/18614	0.000317181144853021	0.00150914358853107	0.000865230473094756	CALCA/CARTPT/ITGB3/LEP/LEPR/P2RX7/S1PR1/SYT7	8	1.63265306122449
GO:0006109	BP	GO:0006109	regulation of carbohydrate metabolic process	17/666	183/18614	0.000318445513955177	0.00151083570378664	0.000866200605886777	ADCYAP1R1/AVPR1B/DDIT4/GCG/GCGR/GPER1/IGF1/LEP/LEPR/NR3C1/NTSR1/P2RX7/P2RY1/PRKN/PTH1R/PTK2B/SNCA	17	0.92896174863388
GO:0010818	BP	GO:0010818	T cell chemotaxis	6/666	27/18614	0.000319073745784442	0.00151083570378664	0.000866200605886777	CCL5/CCR2/CXCL13/PIK3CD/PIK3CG/WNT5A	6	2.22222222222222
GO:0016188	BP	GO:0016188	synaptic vesicle maturation	6/666	27/18614	0.000319073745784442	0.00151083570378664	0.000866200605886777	ATP6V0A4/ATP6V1F/ATP6V1G2/STXBP1/SYP/UNC13C	6	2.22222222222222
GO:0021895	BP	GO:0021895	cerebral cortex neuron differentiation	6/666	27/18614	0.000319073745784442	0.00151083570378664	0.000866200605886777	ASCL1/CNTN2/DRD2/EMX1/FEZF2/NKX2-1	6	2.22222222222222
GO:0035929	BP	GO:0035929	steroid hormone secretion	6/666	27/18614	0.000319073745784442	0.00151083570378664	0.000866200605886777	AGT/AGTR1/GAL/GALR1/INHBA/KCNQ1	6	2.22222222222222

	ONTOLOGY	ID	Description	GeneRatio	BgRatio	pvalue	p.adjust	qvalue	geneID	Count	richFactor
GO:0001707	BP	GO:0001707	mesoderm formation	10/666	75/18614	0.000326892454284347	0.00154488116616112	0.000885719736962749	APELA/DKK1/HMGA2/INHBA/ITGB3/TBX20/WNT11/WNT3/WNT3A/WNT5A	10	1.33333333333333
GO:0072078	BP	GO:0072078	nephron tubule morphogenesis	10/666	75/18614	0.000326892454284347	0.00154488116616112	0.000885719736962749	AGT/BCL2/GDNF/LHX1/SHH/SOX9/VEGFA/WNT1/WNT11/WNT2B	10	1.33333333333333
GO:0021554	BP	GO:0021554	optic nerve development	5/666	18/18614	0.000335723674043955	0.00158205355505852	0.000907031614690081	CHRNB2/EPHB2/KCNA1/KCNA2/LPAR1	5	2.77777777777778
GO:0048486	BP	GO:0048486	parasympathetic nervous system development	5/666	18/18614	0.000335723674043955	0.00158205355505852	0.000907031614690081	GDNF/NRP2/PHOX2B/PLXNA3/SEMA3F	5	2.77777777777778
GO:0050802	BP	GO:0050802	circadian sleep/wake cycle, sleep	5/666	18/18614	0.000335723674043955	0.00158205355505852	0.000907031614690081	ADRB1/CHRNB2/DRD2/GHRHR/KCNA2	5	2.77777777777778
GO:0022617	BP	GO:0022617	extracellular matrix disassembly	9/666	62/18614	0.000338789658068263	0.00159497238448804	0.000914438308780742	CTSG/MMP1/MMP11/MMP13/MMP7/MMP9/PDPN/PRSS1/PRSS2	9	1.45161290322581
GO:0048469	BP	GO:0048469	cell maturation	17/666	184/18614	0.000339478127726153	0.00159515774165778	0.000914544578769158	ASCL1/AURKA/AXL/BCL2/CNTN2/EDNRA/EDNRB/EPAS1/GHRHR/KIF14/LRRK2/NKX6-1/NRCAM/PTH1R/PTK2B/SOX10/VEGFA	17	0.923913043478261
GO:0097696	BP	GO:0097696	receptor signaling pathway via STAT	17/666	184/18614	0.000339478127726153	0.00159515774165778	0.000914544578769158	AGT/CAMK2A/CAV1/CCL5/CCR2/CRLF1/EPHB2/ERBB4/F2/GHR/IGF1/IL6R/IL6ST/LEP/NEUROD1/PRL/PTK2B	17	0.923913043478261
GO:0003180	BP	GO:0003180	aortic valve morphogenesis	7/666	38/18614	0.00034881954932106	0.0016374862320086	0.000938812580839971	AXIN2/ROBO2/SLIT2/SLIT3/SOX9/TBX20/TWIST1	7	1.84210526315789
GO:0010952	BP	GO:0010952	positive regulation of peptidase activity	17/666	185/18614	0.000361702660257533	0.0016898940828572	0.000968859337111684	ATP2A3/BID/EPHA4/FAS/GPER1/GRIN2A/GRIN2B/MAPT/NDUFA13/NGFR/P2RX1/PINK1/RIPK2/SLC1A1/SLC22A2/SNCA/WNT3A	17	0.918918918918919
GO:0032640	BP	GO:0032640	tumor necrosis factor production	17/666	185/18614	0.000361702660257533	0.0016898940828572	0.000968859337111684	AXL/CCR2/CHRNA7/CLU/CX3CR1/EPHB2/IGF1/IL1A/LEP/LRRK2/MC1R/MIF/PLCG2/RIPK2/SYT11/TWIST1/WNT5A	17	0.918918918918919
GO:0032680	BP	GO:0032680	regulation of tumor necrosis factor production	17/666	185/18614	0.000361702660257533	0.0016898940828572	0.000968859337111684	AXL/CCR2/CHRNA7/CLU/CX3CR1/EPHB2/IGF1/IL1A/LEP/LRRK2/MC1R/MIF/PLCG2/RIPK2/SYT11/TWIST1/WNT5A	17	0.918918918918919
GO:0045766	BP	GO:0045766	positive regulation of angiogenesis	17/666	185/18614	0.000361702660257533	0.0016898940828572	0.000968859337111684	AKT3/APELA/CHRNA7/CX3CR1/CXCL8/GAB1/HMGA2/IL1A/ITGB3/PIK3CD/PLCG1/PRKCB/PTK2B/RLN2/TWIST1/VEGFA/WNT5A	17	0.918918918918919
GO:1904018	BP	GO:1904018	positive regulation of vasculature development	17/666	185/18614	0.000361702660257533	0.0016898940828572	0.000968859337111684	AKT3/APELA/CHRNA7/CX3CR1/CXCL8/GAB1/HMGA2/IL1A/ITGB3/PIK3CD/PLCG1/PRKCB/PTK2B/RLN2/TWIST1/VEGFA/WNT5A	17	0.918918918918919
GO:0006869	BP	GO:0006869	lipid transport	31/666	447/18614	0.000363026414967091	0.00169446802427659	0.000971481694273908	AGT/AGTR1/APOC1/AVPR1B/BDKRB2/CAV1/CLU/DRD2/DRD4/FABP7/GAL/GALR1/IL1A/INHBA/ITGB3/KCNQ1/LEP/MAP2K6/MIF/NMB/NMUR2/NTSR1/OXT/P2RX4/P2RX7/PRKN/SHH/SIGMAR1/SLC22A2/SPX/SYT7	31	0.693512304250559
GO:0007422	BP	GO:0007422	peripheral nervous system development	10/666	76/18614	0.000364570873149377	0.00169845103462482	0.000973765255595791	ASCL1/CDK1/CLDN1/CNTNAP1/EDNRB/GDNF/GFRA3/NEFH/POU4F1/SOX10	10	1.31578947368421

	ONTOLOGY	ID	Description	GeneRatio	BgRatio	pvalue	p.adjust	qvalue	geneID	Count	richFactor
GO:0150076	BP	GO:0150076	neuroinflammatory response	10/666	76/18614	0.000364570873149377	0.00169845103462482	0.000973765255595791	CLU/CX3CR1/IGF1/LRRK2/MAPT/MMP9/NR3C1/PLCG2/SNCA/SYT11	10	1.31578947368421
GO:0008089	BP	GO:0008089	anterograde axonal transport	8/666	50/18614	0.000365923672124885	0.00170152776584088	0.000975529223998722	CALY/DLG2/KIF1A/KIF5A/KIF5C/MAP2/NEFL/TRAK2	8	1.6
GO:0090102	BP	GO:0090102	cochlea development	8/666	50/18614	0.000365923672124885	0.00170152776584088	0.000975529223998722	CALB1/EPHA4/KCNQ1/MCM2/NTRK3/SLC17A8/SOX9/WNT5A	8	1.6
GO:0051098	BP	GO:0051098	regulation of binding	27/666	369/18614	0.0003746349256292	0.00174038814694472	0.000997808870668957	ADRB2/ATP2A3/AURKA/BDNF/CAV1/CPNE1/CSNK1E/DKK1/EPHA4/GCG/GZMA/HMGA2/IGF1/ITGA4/LRRK2/MAP2/MMP9/NEUROD1/NGF/P2RY1/PLCL1/POU4F1/PRKN/RIPK2/TWIST1/WNT3A/WNT5A	27	0.731707317073171
GO:0051216	BP	GO:0051216	cartilage development	18/666	203/18614	0.000379920811491838	0.00176327742066325	0.00101093187452279	AXIN2/COL3A1/GHR/HMGA2/LEP/MEF2C/MMP13/PRKG2/PTH1R/SOX5/SOX9/SULF1/WNT11/WNT2B/WNT5A/WNT7A/WNT7B/WNT9A	18	0.886699507389163
GO:0003401	BP	GO:0003401	axis elongation	6/666	28/18614	0.000393915717487566	0.00182650542589754	0.00104718210100716	MAGI2/SHH/SOX9/WNT11/WNT3A/WNT5A	6	2.14285714285714
GO:0048562	BP	GO:0048562	embryonic organ morphogenesis	23/666	294/18614	0.000394708365598696	0.00182845581236342	0.00104830030726495	ATOH1/DSCAML1/EDNRA/EPHB2/FOXG1/FZD3/KCNQ1/LHX1/MEF2C/NEUROD1/NTN1/PROX1/SHH/SLC4A4/SOX9/TBX20/TH/TWIST1/WNT1/WNT11/WNT16/WNT3A/WNT5A	23	0.782312925170068
GO:0060021	BP	GO:0060021	roof of mouth development	11/666	91/18614	0.000398359095427983	0.0018411507705388	0.00105557865026125	EPHB2/EPHB3/GABRB3/INHBA/SHH/TWIST1/VAX1/WNT11/WNT3A/WNT5A/WNT7A	11	1.20879120879121
GO:0070098	BP	GO:0070098	chemokine-mediated signaling pathway	11/666	91/18614	0.000398359095427983	0.0018411507705388	0.00105557865026125	CCL5/CCR2/CCR7/CX3CR1/CXCL12/CXCL13/CXCL8/CXCR5/PTK2B/SLIT2/SLIT3	11	1.20879120879121
GO:2000146	BP	GO:2000146	negative regulation of cell motility	28/666	390/18614	0.000398896932177451	0.0018411507705388	0.00105557865026125	ADGRG1/ADORA3/BCL2/CLDN19/COL3A1/CX3CR1/CXCL12/CXCL13/DRD2/DUSP1/EPHA4/GNA13/MAGI2/MEF2C/MIF/NEXMIF/NGFR/NKX2-1/NRG3/PRKG1/PTGER4/SEMA6D/SHH/SLIT2/SLURP1/SULF1/VCL/WNT11	28	0.717948717948718
GO:0032943	BP	GO:0032943	mononuclear cell proliferation	24/666	313/18614	0.000398947216810543	0.0018411507705388	0.00105557865026125	BCL2/BCL2L1/BID/CCL5/CCR2/CHRNB2/CSF1/EPHB2/FKBP1B/GAL/IGF1/IL1A/IL6ST/LEP/MEF2C/MIF/P2RX7/PIK3CG/PRNP/RIPK2/SHH/SOS2/TACR1/WNT3A	24	0.766773162939297
GO:0003407	BP	GO:0003407	neural retina development	10/666	77/18614	0.00040579815212856	0.0018675074135879	0.00107068958530274	CALB1/GPM6A/HCN1/LHX1/NEUROD1/SLC17A7/SLC17A8/SLC1A1/SOX9/THRB	10	1.2987012987013
GO:0048332	BP	GO:0048332	mesoderm morphogenesis	10/666	77/18614	0.00040579815212856	0.0018675074135879	0.00107068958530274	APELA/DKK1/HMGA2/INHBA/ITGB3/TBX20/WNT11/WNT3/WNT3A/WNT5A	10	1.2987012987013
GO:0072088	BP	GO:0072088	nephron epithelium morphogenesis	10/666	77/18614	0.00040579815212856	0.0018675074135879	0.00107068958530274	AGT/BCL2/GDNF/LHX1/SHH/SOX9/VEGFA/WNT1/WNT11/WNT2B	10	1.2987012987013
GO:0046545	BP	GO:0046545	development of primary female sexual characteristics	12/666	106/18614	0.000406515637241979	0.00186905926758122	0.00107157930273918	A2M/ADCYAP1R1/BCL2/BCL2L1/CGA/INHBA/LEP/ROBO2/SLIT2/SLIT3/VEGFA/VGF	12	1.13207547169811

	ONTOLOGY	ID	Description	GeneRatio	BgRatio	pvalue	p.adjust	qvalue	geneID	Count	richFactor
GO:0030279	BP	GO:0030279	negative regulation of ossification	7/666	39/18614	0.000412156053979801	0.0018932215002904	0.00108543212641803	BCL2/CALCA/DKK1/MEF2C/PTK2B/SOX9/TPH1	7	1.79487179487179
GO:0021515	BP	GO:0021515	cell differentiation in spinal cord	8/666	51/18614	0.000420592103480521	0.00193016824333031	0.00110661463562569	ASCL1/LHX1/MDGA1/SHH/SOX1/TBX20/WNT1/WNT3A	8	1.56862745098039
GO:0050729	BP	GO:0050729	positive regulation of inflammatory response	15/666	154/18614	0.000426404639321647	0.00195501753942714	0.00112086137025142	AGT/AGTR1/CCR2/CCR7/CNR1/CREB3L3/IL6ST/LRRK2/NPY5R/PIK3CG/PLCG2/PTGER3/PTGER4/SNCA/WNT5A	15	0.974025974025974
GO:0071675	BP	GO:0071675	regulation of mononuclear cell migration	13/666	122/18614	0.000429415364677031	0.00196698650269115	0.0011277234818663	CCL5/CCR2/CCR7/CSF1/CX3CR1/CXCL12/CXCL13/DUSP1/ITGA4/ITGB3/PTK2B/SLIT2/WNT5A	13	1.0655737704918
GO:0044091	BP	GO:0044091	membrane biogenesis	9/666	64/18614	0.000431665173259754	0.00197361332704344	0.00113152280912251	ANK3/CAV1/EPHB2/NLGN1/NLGN3/NLGN4X/NLGN4Y/NRXN1/NRXN2	9	1.40625
GO:0045453	BP	GO:0045453	bone resorption	9/666	64/18614	0.000431665173259754	0.00197361332704344	0.00113152280912251	ADRB2/CALCA/CARTPT/ITGB3/NOX4/P2RX7/PTH1R/PTK2B/S1PR1	9	1.40625
GO:0014706	BP	GO:0014706	striated muscle tissue development	21/666	259/18614	0.000434971848422093	0.0019766004370547	0.00113323539540514	ADRA1A/AGT/CDK1/DKK1/EDNRA/ERBB4/IGF1/MEF2C/NOX4/POU4F1/PROX1/RGS2/S1PR1/SHH/TBX20/TP73/TWIST1/VEGFA/WNT2/WNT3A/WNT5A	21	0.810810810810811
GO:0010950	BP	GO:0010950	positive regulation of endopeptidase activity	16/666	171/18614	0.000436478265500063	0.0019766004370547	0.00113323539540514	ATP2A3/BID/EPHA4/FAS/GPER1/GRIN2A/GRIN2B/MAPT/NDUFA13/NGFR/P2RX1/RIPK2/SLC1A1/SLC22A2/SNCA/WNT3A	16	0.935672514619883
GO:0055017	BP	GO:0055017	cardiac muscle tissue growth	11/666	92/18614	0.00043812583561289	0.0019766004370547	0.00113323539540514	ADRA1A/AGT/CDK1/ERBB4/IGF1/MEF2C/RGS2/S1PR1/TBX20/TP73/WNT2	11	1.19565217391304
GO:0001660	BP	GO:0001660	fever generation	4/666	11/18614	0.00043835085989616	0.0019766004370547	0.00113323539540514	CNR1/EDNRB/IL1A/PTGER3	4	3.63636363636364
GO:0003139	BP	GO:0003139	secondary heart field specification	4/666	11/18614	0.00043835085989616	0.0019766004370547	0.00113323539540514	AXIN2/MEF2C/WNT11/WNT5A	4	3.63636363636364
GO:0014049	BP	GO:0014049	positive regulation of glutamate secretion	4/666	11/18614	0.00043835085989616	0.0019766004370547	0.00113323539540514	NTSR1/P2RX7/STXBP1/SYT4	4	3.63636363636364
GO:0021889	BP	GO:0021889	olfactory bulb interneuron differentiation	4/666	11/18614	0.00043835085989616	0.0019766004370547	0.00113323539540514	ERBB4/ROBO2/SLIT2/WNT5A	4	3.63636363636364
GO:0032276	BP	GO:0032276	regulation of gonadotropin secretion	4/666	11/18614	0.00043835085989616	0.0019766004370547	0.00113323539540514	INHBA/KISS1/LEP/TACR2	4	3.63636363636364
GO:0060174	BP	GO:0060174	limb bud formation	4/666	11/18614	0.00043835085989616	0.0019766004370547	0.00113323539540514	PLXNA2/SHH/SOX9/WNT3	4	3.63636363636364
GO:0060601	BP	GO:0060601	lateral sprouting from an epithelium	4/666	11/18614	0.00043835085989616	0.0019766004370547	0.00113323539540514	CELSR1/SHH/SULF1/WNT5A	4	3.63636363636364
GO:0060900	BP	GO:0060900	embryonic camera-type eye formation	4/666	11/18614	0.00043835085989616	0.0019766004370547	0.00113323539540514	PROX1/TWIST1/WNT16/WNT5A	4	3.63636363636364
GO:0086100	BP	GO:0086100	endothelin receptor signaling pathway	4/666	11/18614	0.00043835085989616	0.0019766004370547	0.00113323539540514	EDNRA/EDNRB/GNA11/PTK2B	4	3.63636363636364
GO:1905150	BP	GO:1905150	regulation of voltage-gated sodium channel activity	4/666	11/18614	0.00043835085989616	0.0019766004370547	0.00113323539540514	FGF12/FGF13/GRP/KCNQ1	4	3.63636363636364
GO:1905383	BP	GO:1905383	protein localization to presynapse	4/666	11/18614	0.00043835085989616	0.0019766004370547	0.00113323539540514	DLG2/KIF5A/KIF5C/WNT7A	4	3.63636363636364
GO:2000672	BP	GO:2000672	negative regulation of motor neuron apoptotic process	4/666	11/18614	0.00043835085989616	0.0019766004370547	0.00113323539540514	BCL2/CNTFR/CRLF1/NEFL	4	3.63636363636364
GO:0007625	BP	GO:0007625	grooming behavior	5/666	19/18614	0.000442288966612667	0.00198163196982795	0.0011361201013502	DRD2/NMU/NMUR2/OXT/SLC1A1	5	2.63157894736842
GO:0032026	BP	GO:0032026	response to magnesium ion	5/666	19/18614	0.000442288966612667	0.00198163196982795	0.0011361201013502	ANK3/CCND1/KCNA1/RYR3/SNCA	5	2.63157894736842

	ONTOLOGY	ID	Description	GeneRatio	BgRatio	pvalue	p.adjust	qvalue	geneID	Count	richFactor
GO:0035930	BP	GO:0035930	corticosteroid hormone secretion	5/666	19/18614	0.000442288966612667	0.00198163196982795	0.0011361201013502	AGT/AGTR1/GAL/GALR1/KCNQ1	5	2.63157894736842
GO:0061744	BP	GO:0061744	motor behavior	5/666	19/18614	0.000442288966612667	0.00198163196982795	0.0011361201013502	GRP/GRPR/NR3C1/SLC1A1/TUBA1A	5	2.63157894736842
GO:0071625	BP	GO:0071625	vocalization behavior	5/666	19/18614	0.000442288966612667	0.00198163196982795	0.0011361201013502	NLGN3/NLGN4X/NLGN4Y/NRXN1/NRXN2	5	2.63157894736842
GO:0090036	BP	GO:0090036	regulation of protein kinase C signaling	5/666	19/18614	0.000442288966612667	0.00198163196982795	0.0011361201013502	ADRA1A/MC1R/VEGFA/WNT11/WNT5A	5	2.63157894736842
GO:1904862	BP	GO:1904862	inhibitory synapse assembly	5/666	19/18614	0.000442288966612667	0.00198163196982795	0.0011361201013502	FGF13/GABRB3/GABRG2/LHFPL4/WNT5A	5	2.63157894736842
GO:0022600	BP	GO:0022600	digestive system process	12/666	107/18614	0.000443281988452546	0.0019842722889292	0.0011376338635677	ADRA2A/CHRM5/KCNQ1/LEP/NEUROD1/NPSR1/OXT/OXTR/PTGER3/SOX9/TACR1/UCN	12	1.1214953271028
GO:1901617	BP	GO:1901617	organic hydroxy compound biosynthetic process	20/666	242/18614	0.00046276279408098	0.00206958974786899	0.00118654853671259	ADCYAP1R1/ASIP/AVPR1B/GPER1/LEP/MC1R/NTSR1/P2RY1/PAH/PLCG2/PROX1/PTH1R/PTK2B/SLC6A3/SLC7A11/SNCA/TH/TPH1/TRPC1/WNT5A	20	0.826446280991736
GO:0006979	BP	GO:0006979	response to oxidative stress	30/666	434/18614	0.000474011387653655	0.0021179690639252	0.0012142856313386	AXL/BCL2/CAPN2/CDK1/CHRNA4/EDNRA/EPAS1/FKBP1B/GPX2/GPX3/IL1A/LRRK2/MAPT/MMP9/PENK/PINK1/PRKN/PRNP/PTGS1/PTK2B/SIGMAR1/SLC1A1/SLC7A11/SNCA/TACR1/TRAP1/TXNIP/UCN/WNT1/WNT16	30	0.691244239631337
GO:0086009	BP	GO:0086009	membrane repolarization	8/666	52/18614	0.000481711671039834	0.00214299445433981	0.0012286333253237	ATP1A2/CACNA1D/CAV1/KCNJ5/KCNQ1/NEDD4L/SCN1B/SCN2B	8	1.53846153846154
GO:0007263	BP	GO:0007263	nitric oxide mediated signal transduction	6/666	29/18614	0.000481792242532144	0.00214299445433981	0.0012286333253237	AGT/GUCY1A1/NDNF/NEUROD1/NOS1/VEGFA	6	2.06896551724138
GO:0035640	BP	GO:0035640	exploration behavior	6/666	29/18614	0.000481792242532144	0.00214299445433981	0.0012286333253237	ATP1A2/CHL1/GAD1/LRRK2/PENK/TNR	6	2.06896551724138
GO:0060441	BP	GO:0060441	epithelial tube branching involved in lung morphogenesis	6/666	29/18614	0.000481792242532144	0.00214299445433981	0.0012286333253237	CELSR1/NKX2-1/SHH/SOX9/WNT2/WNT2B	6	2.06896551724138
GO:0090025	BP	GO:0090025	regulation of monocyte chemotaxis	6/666	29/18614	0.000481792242532144	0.00214299445433981	0.0012286333253237	CCL5/CCR2/CX3CR1/CXCL12/DUSP1/SLIT2	6	2.06896551724138
GO:1903115	BP	GO:1903115	regulation of actin filament-based movement	7/666	40/18614	0.00048435269475826	0.00215243534786333	0.00123404603013978	ATP1A2/ATP2A1/CACNA1C/CAMK2D/CAV1/FGF13/PDPN	7	1.75
GO:0071706	BP	GO:0071706	tumor necrosis factor superfamily cytokine production	17/666	190/18614	0.000492711977832552	0.00218563120130595	0.00125307805876662	AXL/CCR2/CHRNA7/CLU/CX3CR1/EPHB2/IGF1/IL1A/LEP/LRRK2/MC1R/MIF/PLCG2/RIPK2/SYT11/TWIST1/WNT5A	17	0.894736842105263
GO:1903555	BP	GO:1903555	regulation of tumor necrosis factor superfamily cytokine production	17/666	190/18614	0.000492711977832552	0.00218563120130595	0.00125307805876662	AXL/CCR2/CHRNA7/CLU/CX3CR1/EPHB2/IGF1/IL1A/LEP/LRRK2/MC1R/MIF/PLCG2/RIPK2/SYT11/TWIST1/WNT5A	17	0.894736842105263
GO:0031214	BP	GO:0031214	biomineral tissue development	16/666	173/18614	0.000496552925058819	0.0022006831620055	0.00126170773136772	ADRB2/AXIN2/FZD9/GPM6B/IGF1/LEP/MEF2C/MMP13/P2RX7/PTH1R/PTK2B/PTN/S1PR1/SOX9/TWIST1/WNT11	16	0.92485549132948
GO:0045913	BP	GO:0045913	positive regulation of carbohydrate metabolic process	10/666	79/18614	0.000499933787305017	0.00221367077892267	0.00126915386307779	ADCYAP1R1/AVPR1B/GCG/GPER1/IGF1/NTSR1/P2RX7/P2RY1/PTH1R/SNCA	10	1.26582278481013

	ONTOLOGY	ID	Description	GeneRatio	BgRatio	pvalue	p.adjust	qvalue	geneID	Count	richFactor
GO:0071326	BP	GO:0071326	cellular response to monosaccharide stimulus	14/666	140/18614	0.000502995934336117	0.00222522503803962	0.00127577821423827	ADCY5/ADRA2A/EFNA5/FKBP1B/GCG/GHRHR/GPER1/GRIK5/LEP/NEUROD1/NOX4/NPTX1/SLC29A1/TH	14	1
GO:1901379	BP	GO:1901379	regulation of potassium ion transmembrane transport	11/666	94/18614	0.000527682870859398	0.00233233930780031	0.00133718955442125	ANK3/CACNA1D/CAV1/EDN3/GAL/GRP/KCNA1/KCNQ1/NEDD4L/PRNP/VAMP2	11	1.17021276595745
GO:0046777	BP	GO:0046777	protein autophosphorylation	19/666	227/18614	0.000544098187697927	0.00240273368601555	0.00137754844513865	AURKA/CAMK2A/CAMK2B/CAMK2D/CAMK4/CAV1/EPHA4/EPHB3/ERBB4/FGFR1/IRAK1/LRRK2/PINK1/PRKCG/PRKG2/PTK2B/TTK/TYRO3/VEGFA	19	0.837004405286344
GO:0060071	BP	GO:0060071	Wnt signaling pathway, planar cell polarity pathway	8/666	53/18614	0.00054983508488923	0.00242371250424266	0.00138957613617981	CELSR1/DKK1/FZD3/GPC6/MAGI2/WNT11/WNT5A/WNT7A	8	1.50943396226415
GO:1902930	BP	GO:1902930	regulation of alcohol biosynthetic process	8/666	53/18614	0.00054983508488923	0.00242371250424266	0.00138957613617981	ADCYAP1R1/AVPR1B/GPER1/NTSR1/P2RY1/PTH1R/PTK2B/SNCA	8	1.50943396226415
GO:0030500	BP	GO:0030500	regulation of bone mineralization	10/666	80/18614	0.000553389672888101	0.00243719555756722	0.00139730631420623	ADRB2/FZD9/GPM6B/MEF2C/P2RX7/PTK2B/PTN/S1PR1/SOX9/TWIST1	10	1.25
GO:0055001	BP	GO:0055001	muscle cell development	17/666	192/18614	0.00055555084924123	0.00244452320861293	0.00140150744326323	ADRA1A/AGT/BCL2/CDK1/CNTNAP1/DNER/EDNRA/HOMER1/IGF1/MYPN/P2RX2/PROX1/RGS2/RYR1/TMOD2/UCHL1/VEGFA	17	0.885416666666667
GO:0014904	BP	GO:0014904	myotube cell development	7/666	41/18614	0.000566276371722648	0.00248726395622593	0.00142601180292698	BCL2/CNTNAP1/DNER/HOMER1/IGF1/P2RX2/RYR1	7	1.70731707317073
GO:0060421	BP	GO:0060421	positive regulation of heart growth	7/666	41/18614	0.000566276371722648	0.00248726395622593	0.00142601180292698	CDK1/ERBB4/IGF1/MEF2C/PROX1/TBX20/WNT2	7	1.70731707317073
GO:0002064	BP	GO:0002064	epithelial cell development	18/666	210/18614	0.000569970703884403	0.00249885153928099	0.00143265525955029	CLDN1/EDNRA/EDNRB/EPAS1/F2RL1/IL1A/IL6ST/MAGI2/NKX6-1/PALLD/PROX1/RAP1A/SOX9/VCL/VEGFA/WNT5A/WNT7A/WNT7B	18	0.857142857142857
GO:0002444	BP	GO:0002444	myeloid leukocyte mediated immunity	12/666	110/18614	0.000570826779931089	0.00249885153928099	0.00143265525955029	CCR2/CTSG/CX3CR1/F2/F2RL1/GRP/PIK3CD/PIK3CG/PTGDR/SLC18A2/STXBP1/VAMP2	12	1.09090909090909
GO:0035902	BP	GO:0035902	response to immobilization stress	5/666	20/18614	0.000572473414695909	0.00249885153928099	0.00143265525955029	GAL/PTK2B/TH/TPH1/UCN3	5	2.5
GO:0071379	BP	GO:0071379	cellular response to prostaglandin stimulus	5/666	20/18614	0.000572473414695909	0.00249885153928099	0.00143265525955029	GNG2/P2RY4/PTGDR/PTGER4/PTGFR	5	2.5
GO:0090128	BP	GO:0090128	regulation of synapse maturation	5/666	20/18614	0.000572473414695909	0.00249885153928099	0.00143265525955029	CAMK2B/CNTNAP1/NEFL/NRXN1/RELN	5	2.5
GO:0140131	BP	GO:0140131	positive regulation of lymphocyte chemotaxis	5/666	20/18614	0.000572473414695909	0.00249885153928099	0.00143265525955029	CCL5/CCR2/CXCL13/PTK2B/WNT5A	5	2.5
GO:1903975	BP	GO:1903975	regulation of glial cell migration	5/666	20/18614	0.000572473414695909	0.00249885153928099	0.00143265525955029	CCR2/CSF1/CX3CR1/NTN1/P2RX4	5	2.5
GO:0045907	BP	GO:0045907	positive regulation of vasoconstriction	6/666	30/18614	0.000584205511815837	0.00254554086043869	0.001459423437039	ADRA1A/ADRA1D/AVPR1B/CAV1/OXTR/TACR1	6	2
GO:1902230	BP	GO:1902230	negative regulation of intrinsic apoptotic signaling pathway in response to DNA damage	6/666	30/18614	0.000584205511815837	0.00254554086043869	0.001459423437039	BCL2/BCL2L1/BID/CLU/CXCL12/MIF	6	2
GO:0051099	BP	GO:0051099	positive regulation of binding	16/666	176/18614	0.000599913432084679	0.00261166919282214	0.00149733649497989	BDNF/CAV1/EPHA4/GCG/IGF1/LRRK2/MMP9/NEUROD1/NGF/PLCL1/POU4F1/PRKN/RIPK2/TWIST1/WNT3A/WNT5A	16	0.909090909090909

	ONTOLOGY	ID	Description	GeneRatio	BgRatio	pvalue	p.adjust	qvalue	geneID	Count	richFactor
GO:0014009	BP	GO:0014009	glial cell proliferation	8/666	54/18614	0.000625542580903225	0.00272083343817642	0.00155992313840515	CLU/CSF1/LEPR/NTN1/PENK/PTK2B/SHH/SOX10	8	1.48148148148148
GO:0001973	BP	GO:0001973	G protein-coupled adenosine receptor signaling pathway	4/666	12/18614	0.000638971670410009	0.00273566703838432	0.00156842761937245	ADCY5/ADORA3/CNTN2/P2RY1	4	3.33333333333333
GO:0002087	BP	GO:0002087	regulation of respiratory gaseous exchange by nervous system process	4/666	12/18614	0.000638971670410009	0.00273566703838432	0.00156842761937245	ATP1A2/NLGN3/PHOX2B/TLX3	4	3.33333333333333
GO:0003306	BP	GO:0003306	Wnt signaling pathway involved in heart development	4/666	12/18614	0.000638971670410009	0.00273566703838432	0.00156842761937245	DKK1/WNT11/WNT3A/WNT5A	4	3.33333333333333
GO:0019336	BP	GO:0019336	phenol-containing compound catabolic process	4/666	12/18614	0.000638971670410009	0.00273566703838432	0.00156842761937245	COMT/MAOA/MAOB/SLC6A3	4	3.33333333333333
GO:0030432	BP	GO:0030432	peristalsis	4/666	12/18614	0.000638971670410009	0.00273566703838432	0.00156842761937245	DRD2/GDNF/P2RX2/SSTR2	4	3.33333333333333
GO:0031630	BP	GO:0031630	regulation of synaptic vesicle fusion to presynaptic active zone membrane	4/666	12/18614	0.000638971670410009	0.00273566703838432	0.00156842761937245	CPLX1/GRIK5/STXBP1/SYT9	4	3.33333333333333
GO:0032957	BP	GO:0032957	inositol trisphosphate metabolic process	4/666	12/18614	0.000638971670410009	0.00273566703838432	0.00156842761937245	GPER1/P2RY1/PLCG2/PTK2B	4	3.33333333333333
GO:0035588	BP	GO:0035588	G protein-coupled purinergic receptor signaling pathway	4/666	12/18614	0.000638971670410009	0.00273566703838432	0.00156842761937245	ADCY5/ADORA3/CNTN2/P2RY1	4	3.33333333333333
GO:0040015	BP	GO:0040015	negative regulation of multicellular organism growth	4/666	12/18614	0.000638971670410009	0.00273566703838432	0.00156842761937245	ADRB1/ADRB2/ADRB3/GDF15	4	3.33333333333333
GO:0060123	BP	GO:0060123	regulation of growth hormone secretion	4/666	12/18614	0.000638971670410009	0.00273566703838432	0.00156842761937245	ADCYAP1/DRD2/GHRHR/KISS1	4	3.33333333333333
GO:0060134	BP	GO:0060134	prepulse inhibition	4/666	12/18614	0.000638971670410009	0.00273566703838432	0.00156842761937245	DRD2/GRID2/GRIN3A/SLC6A3	4	3.33333333333333
GO:0099550	BP	GO:0099550	trans-synaptic signaling, modulating synaptic transmission	4/666	12/18614	0.000638971670410009	0.00273566703838432	0.00156842761937245	CNR1/EPHB2/GUCY1A1/SYT4	4	3.33333333333333
GO:0150065	BP	GO:0150065	regulation of deacetylase activity	4/666	12/18614	0.000638971670410009	0.00273566703838432	0.00156842761937245	CAMK2D/LRRK2/PINK1/VEGFA	4	3.33333333333333
GO:1900272	BP	GO:1900272	negative regulation of long-term synaptic potentiation	4/666	12/18614	0.000638971670410009	0.00273566703838432	0.00156842761937245	CX3CR1/EPHA4/PRNP/PTN	4	3.33333333333333
GO:1901632	BP	GO:1901632	regulation of synaptic vesicle membrane organization	4/666	12/18614	0.000638971670410009	0.00273566703838432	0.00156842761937245	CPLX1/GRIK5/STXBP1/SYT9	4	3.33333333333333
GO:1902667	BP	GO:1902667	regulation of axon guidance	4/666	12/18614	0.000638971670410009	0.00273566703838432	0.00156842761937245	FEZF2/SLIT2/TUBB2B/VEGFA	4	3.33333333333333
GO:1903977	BP	GO:1903977	positive regulation of glial cell migration	4/666	12/18614	0.000638971670410009	0.00273566703838432	0.00156842761937245	CCR2/CSF1/CX3CR1/P2RX4	4	3.33333333333333
GO:1904338	BP	GO:1904338	regulation of dopaminergic neuron differentiation	4/666	12/18614	0.000638971670410009	0.00273566703838432	0.00156842761937245	CSNK1E/DKK1/SHH/WNT3A	4	3.33333333333333
GO:0050999	BP	GO:0050999	regulation of nitric-oxide synthase activity	7/666	42/18614	0.000658839017521452	0.00281582067053734	0.00161438173904341	CAV1/CNR1/CNR2/HTR2B/IL1A/LEP/PTK2B	7	1.66666666666667
GO:2000008	BP	GO:2000008	regulation of protein localization to cell surface	7/666	42/18614	0.000658839017521452	0.00281582067053734	0.00161438173904341	BDNF/EPHB2/ERBB4/GPM6B/HSP90AB1/NEDD4L/TMEM35A	7	1.66666666666667
GO:1905954	BP	GO:1905954	positive regulation of lipid localization	12/666	112/18614	0.000671938075086243	0.00286930985147601	0.00164504844940864	AVPR1B/CAV1/GAL/GALR1/IL1A/MAP2K6/MIF/NMB/NTSR1/OXT/P2RX4/P2RX7	12	1.07142857142857
GO:0050848	BP	GO:0050848	regulation of calcium-mediated signaling	10/666	82/18614	0.000674538806556306	0.00287541911034193	0.00164855104318367	GPR143/IGF1/ITPR1/LRRK2/MAPT/P2RX2/P2RX4/PLCG2/PRNP/PTK2B	10	1.21951219512195

	ONTOLOGY	ID	Description	GeneRatio	BgRatio	pvalue	p.adjust	qvalue	geneID	Count	richFactor
GO:0061097	BP	GO:0061097	regulation of protein tyrosine kinase activity	10/666	82/18614	0.000674538806556306	0.00287541911034193	0.00164855104318367	ADRA1A/ADRA2A/AGT/CAV1/EPHA4/ITGB3/NOX4/PRNP/RELN/WNT3A	10	1.21951219512195
GO:0060538	BP	GO:0060538	skeletal muscle organ development	16/666	178/18614	0.000678606773002901	0.00289025328363021	0.00165705585271255	BCL2/CAV1/CHRNA1/CHRND/CNTFR/CNTNAP1/DNER/HOMER1/MEF2C/P2RX2/PHOX2B/RYR1/SHH/TWIST1/VAX1/WNT3A	16	0.898876404494382
GO:0015909	BP	GO:0015909	long-chain fatty acid transport	9/666	68/18614	0.000680483470934914	0.00289323205851652	0.00165876366025566	AVPR1B/BDKRB2/DRD2/DRD4/MIF/NMB/NMUR2/NTSR1/SPX	9	1.32352941176471
GO:0061180	BP	GO:0061180	mammary gland epithelium development	9/666	68/18614	0.000680483470934914	0.00289323205851652	0.00165876366025566	CCND1/CSF1/ERBB4/NR3C1/NTN1/WNT2/WNT3/WNT5A/WNT7B	9	1.32352941176471
GO:0035051	BP	GO:0035051	cardiocyte differentiation	15/666	161/18614	0.000681645222785337	0.00289566661191869	0.00166015945175485	ADRA1A/AGT/CDK1/DKK1/EDNRA/GPER1/IGF1/INHBA/MEF2C/NOX4/PROX1/RGS2/TWIST1/VEGFA/WNT3A	15	0.93167701863354
GO:0043087	BP	GO:0043087	regulation of GTPase activity	26/666	364/18614	0.000684216373972238	0.00290407899660928	0.00166498248625005	ADCYAP1/ADRB1/ARHGAP44/CCL5/CCR7/CXCL13/EFNA5/EPHA3/EPHA4/EPHB3/F2RL1/FZD10/LRRK2/NTRK3/PLXNA1/PLXNA2/PLXNA3/PRKG1/PTK2B/RAP1A/RASAL1/RTN4R/S1PR1/SOS2/WNT11/WNT5A	26	0.714285714285714
GO:0007369	BP	GO:0007369	gastrulation	17/666	196/18614	0.000702115164588804	0.00297497989158461	0.00170563177593232	APELA/APLN/DKK1/DUSP1/HMGA2/INHBA/ITGA4/ITGB3/LHX1/MMP9/SOX2/TBX20/WNT11/WNT3/WNT3A/WNT5A/WNT8B	17	0.86734693877551
GO:0040018	BP	GO:0040018	positive regulation of multicellular organism growth	6/666	31/18614	0.000702736857401776	0.00297497989158461	0.00170563177593232	BCL2/CSF1/DRD2/GHR/GHRHR/SLC6A3	6	1.93548387096774
GO:0048873	BP	GO:0048873	homeostasis of number of cells within a tissue	6/666	31/18614	0.000702736857401776	0.00297497989158461	0.00170563177593232	AKT3/BCL2/CSF1/P2RX7/SOX9/TUBA1A	6	1.93548387096774
GO:0035264	BP	GO:0035264	multicellular organism growth	14/666	145/18614	0.000715284045825633	0.00302549146749827	0.00173459134946573	ADRB1/ADRB2/ADRB3/BCL2/COL3A1/CSF1/DRD2/GDF15/GHR/GHRHR/NPY1R/SLC1A2/SLC6A3/SPTBN2	14	0.96551724137931
GO:0002062	BP	GO:0002062	chondrocyte differentiation	12/666	113/18614	0.00072786627233536	0.00307342158808273	0.00176207090888204	AXIN2/COL3A1/HMGA2/MEF2C/PRKG2/PTH1R/SOX5/SOX9/SULF1/WNT2B/WNT7A/WNT9A	12	1.06194690265487
GO:0044344	BP	GO:0044344	cellular response to fibroblast growth factor stimulus	12/666	113/18614	0.00072786627233536	0.00307342158808273	0.00176207090888204	APLN/CCL5/CXCL13/CXCL8/FGFR1/FLRT1/FLRT2/LHX1/NDNF/NGFR/SULF1/WNT5A	12	1.06194690265487
GO:0009190	BP	GO:0009190	cyclic nucleotide biosynthetic process	5/666	21/18614	0.000729419049542955	0.00307469522170122	0.00176280111548839	ADCY2/ADCY3/ADCY5/ADCY9/GUCY1A1	5	2.38095238095238
GO:1903429	BP	GO:1903429	regulation of cell maturation	5/666	21/18614	0.000729419049542955	0.00307469522170122	0.00176280111548839	AURKA/BCL2/EDNRB/KIF14/LRRK2	5	2.38095238095238
GO:0035023	BP	GO:0035023	regulation of Rho protein signal transduction	10/666	83/18614	0.000742854019253196	0.00312596532930604	0.00179219557456814	ADGRG1/ADRA1A/ARRB1/COL3A1/F2RL1/F2RL2/LPAR1/P2RY10/P2RY8/RTN4R	10	1.20481927710843
GO:0046209	BP	GO:0046209	nitric oxide metabolic process	10/666	83/18614	0.000742854019253196	0.00312596532930604	0.00179219557456814	AGT/CAV1/CLU/CX3CR1/GCHFR/HSP90AB1/NOS1/NPY/P2RX4/PTK2B	10	1.20481927710843
GO:0051650	BP	GO:0051650	establishment of vesicle localization	17/666	197/18614	0.000743543212747309	0.00312618895693159	0.00179232378598401	ASIP/GPR143/ITGA4/KIF1A/KIF5A/KIF5C/LRRK2/MAP2/NLGN1/PINK1/PRKN/SEPTIN5/SLC2A4/SNCA/SYT11/SYT4/TRAK2	17	0.862944162436548

	ONTOLOGY	ID	Description	GeneRatio	BgRatio	pvalue	p.adjust	qvalue	geneID	Count	richFactor
GO:0002275	BP	GO:0002275	myeloid cell activation involved in immune response	11/666	98/18614	0.000753028673822445	0.00316066262325988	0.00181208841729757	CCR2/CX3CR1/F2RL1/GRP/PIK3CD/PIK3CG/PLCG2/PTGDR/SLC18A2/STXBP1/VAMP2	11	1.12244897959184
GO:0097306	BP	GO:0097306	cellular response to alcohol	11/666	98/18614	0.000753028673822445	0.00316066262325988	0.00181208841729757	ADCY2/ADCY3/ADCY5/EFNA5/GNAI1/GNG2/INHBA/P2RY4/PTGDR/PTGER4/PTGFR	11	1.12244897959184
GO:0003176	BP	GO:0003176	aortic valve development	7/666	43/18614	0.00076299691399067	0.00319976948145405	0.00183450937556406	AXIN2/ROBO2/SLIT2/SLIT3/SOX9/TBX20/TWIST1	7	1.62790697674419
GO:0051222	BP	GO:0051222	positive regulation of protein transport	23/666	309/18614	0.000786010734126071	0.00329347208715229	0.00188823146700363	ANK3/CDK1/F2/F2RL1/F2RL2/GCG/GNA11/GPER1/HPCA/IGF1/IL1A/ITPR1/LEP/NKX6-1/P2RX7/PIK3R2/PINK1/PRKCB/PRNP/SHH/TENM1/UCN3/VAMP2	23	0.744336569579288
GO:2000116	BP	GO:2000116	regulation of cysteine-type endopeptidase activity	19/666	234/18614	0.000787173570053717	0.0032955350058041	0.00188941419083099	ATP2A3/BID/FAS/GPER1/GRIN2A/GRIN2B/MAGEA3/MAPT/MMP9/NDUFA13/NGFR/P2RX1/RIPK2/SLC1A1/SLC22A2/SNCA/SOX2/VEGFA/WNT3A	19	0.811965811965812
GO:2001057	BP	GO:2001057	reactive nitrogen species metabolic process	10/666	84/18614	0.000816765344933484	0.0034165120598707	0.00195877341848774	AGT/CAV1/CLU/CX3CR1/GCHFR/HSP90AB1/NOS1/NPY/P2RX4/PTK2B	10	1.19047619047619
GO:0045765	BP	GO:0045765	regulation of angiogenesis	25/666	349/18614	0.00082623284743721	0.00345317554860024	0.00197979353078179	AGT/AKT3/APELA/CCR2/CHRNA7/CX3CR1/CXCL13/CXCL8/GAB1/HMGA2/IL1A/ITGB3/LEP/PIK3CD/PLCG1/PRKCB/PRL/PTK2B/RLN2/SEMA3E/SEMA6A/SULF1/TWIST1/VEGFA/WNT5A	25	0.716332378223496
GO:0043254	BP	GO:0043254	regulation of protein-containing complex assembly	28/666	409/18614	0.000838453360867536	0.00350127295553436	0.0020073689997249	BID/CALY/CCR7/CLU/CXCL13/DKK1/KIF14/MAP1B/MAP2/MAPT/MMP1/OPRD1/PIK3R2/PINK1/PLCG2/PTGER4/PTK2B/SLIT2/SNCA/SOX9/SPTBN2/STX1A/STXBP1/TBX20/TENM1/TMOD2/TUBB4A/VEGFA	28	0.684596577017115
GO:0009880	BP	GO:0009880	embryonic pattern specification	9/666	70/18614	0.000842953870723088	0.00351409522867174	0.00201472033563297	CHRDL1/ERBB4/LHX1/NRP2/SEMA3F/SHH/WNT1/WNT5A/WNT7A	9	1.28571428571429
GO:2000379	BP	GO:2000379	positive regulation of reactive oxygen species metabolic process	9/666	70/18614	0.000842953870723088	0.00351409522867174	0.00201472033563297	AGT/AGTR1/F2/F2RL1/LEP/MAPT/PLCG2/PTK2B/SNCA	9	1.28571428571429
GO:0031333	BP	GO:0031333	negative regulation of protein-containing complex assembly	14/666	148/18614	0.00087581367475969	0.00364798661986769	0.00209148368183023	CLU/DKK1/KIF14/MAP2/OPRD1/PTGER4/SLIT2/SNCA/SOX9/SPTBN2/STXBP1/TBX20/TMOD2/TUBB4A	14	0.945945945945946
GO:0007519	BP	GO:0007519	skeletal muscle tissue development	15/666	165/18614	0.000878817414761643	0.00365739847040938	0.00209687973556479	BCL2/CAV1/CHRNA1/CHRND/CNTNAP1/DNER/HOMER1/MEF2C/P2RX2/PHOX2B/RYR1/SHH/TWIST1/VAX1/WNT3A	15	0.909090909090909
GO:0007517	BP	GO:0007517	muscle organ development	25/666	351/18614	0.000896182063020587	0.00371085233830673	0.00212752620006363	BCL2/CAV1/CHRNA1/CHRND/CNTFR/CNTNAP1/COL3A1/DNER/EDNRA/HOMER1/IGF1/MEF2C/P2RX2/PHOX2B/POU4F1/PROX1/RYR1/S1PR1/SHH/TBX20/TWIST1/VAX1/WNT2/WNT3A/WNT5A	25	0.712250712250712
GO:0002024	BP	GO:0002024	diet induced thermogenesis	4/666	13/18614	0.000896946607916255	0.00371085233830673	0.00212752620006363	ADRB1/ADRB2/ADRB3/SCTR	4	3.07692307692308
GO:0007320	BP	GO:0007320	insemination	4/666	13/18614	0.000896946607916255	0.00371085233830673	0.00212752620006363	OXT/OXTR/P2RX1/TACR1	4	3.07692307692308

	ONTOLOGY	ID	Description	GeneRatio	BgRatio	pvalue	p.adjust	qvalue	geneID	Count	richFactor
GO:0021681	BP	GO:0021681	cerebellar granular layer development	4/666	13/18614	0.000896946607916255	0.00371085233830673	0.00212752620006363	GRID2/KIF14/PROX1/WNT7A	4	3.07692307692308
GO:0032536	BP	GO:0032536	regulation of cell projection size	4/666	13/18614	0.000896946607916255	0.00371085233830673	0.00212752620006363	CNTN2/NEFL/WNT7A/WNT7B	4	3.07692307692308
GO:0042415	BP	GO:0042415	norepinephrine metabolic process	4/666	13/18614	0.000896946607916255	0.00371085233830673	0.00212752620006363	EDNRA/EPAS1/PRKN/TH	4	3.07692307692308
GO:0051481	BP	GO:0051481	negative regulation of cytosolic calcium ion concentration	4/666	13/18614	0.000896946607916255	0.00371085233830673	0.00212752620006363	ATP1A2/BCL2/DRD2/NOS1	4	3.07692307692308
GO:0072132	BP	GO:0072132	mesenchyme morphogenesis	8/666	57/18614	0.00090438350552178	0.00373533187364668	0.00214156094142575	APC/ROBO2/SOX9/TBX20/TWIST1/WNT11/WNT3A/WNT5A	8	1.40350877192982
GO:0090175	BP	GO:0090175	regulation of establishment of planar polarity	8/666	57/18614	0.00090438350552178	0.00373533187364668	0.00214156094142575	CELSR1/DKK1/FZD3/GPC6/MAGI2/WNT11/WNT5A/WNT7A	8	1.40350877192982
GO:1902683	BP	GO:1902683	regulation of receptor localization to synapse	5/666	22/18614	0.000916400651112245	0.00378178774157572	0.00216819527422995	ARHGAP44/CPLX1/GPC6/ITGB3/RAP1A	5	2.27272727272727
GO:0043393	BP	GO:0043393	regulation of protein binding	17/666	201/18614	0.000930832112787408	0.0038381206664011	0.00220049237542555	ADRB2/ATP2A3/AURKA/BDNF/CAV1/CSNK1E/DKK1/GCG/ITGA4/LRRK2/MAP2/MMP9/PLCL1/PRKN/RIPK2/WNT3A/WNT5A	17	0.845771144278607
GO:0002548	BP	GO:0002548	monocyte chemotaxis	9/666	71/18614	0.000935274656329867	0.00385153216679086	0.00220818153033124	CALCA/CCL5/CCR2/CTSG/CX3CR1/CXCL12/DUSP1/IL6R/SLIT2	9	1.26760563380282
GO:0071456	BP	GO:0071456	cellular response to hypoxia	14/666	149/18614	0.000935651964831799	0.00385153216679086	0.00220818153033124	BCL2/EPAS1/EPHA4/GNGT1/IRAK1/NDNF/OPRD1/PINK1/SDHD/SLC29A1/SLC2A4/TWIST1/UCN3/VEGFA	14	0.939597315436242
GO:0061448	BP	GO:0061448	connective tissue development	21/666	275/18614	0.00094633608000937	0.00389225258012222	0.00223152758087213	AXIN2/COL3A1/CSF1/GHR/HMGA2/IL6R/ITGB3/LEP/MEF2C/MMP13/PRKG2/PTH1R/SOX5/SOX9/SULF1/WNT11/WNT2B/WNT5A/WNT7A/WNT7B/WNT9A	21	0.763636363636364
GO:0061053	BP	GO:0061053	somite development	10/666	86/18614	0.000982759646337684	0.00403868199142953	0.00231547929347472	AXIN2/DKK1/LHX1/PLXNA2/PPP2R3A/SHH/WNT1/WNT11/WNT3A/WNT5A	10	1.16279069767442
GO:0010632	BP	GO:0010632	regulation of epithelial cell migration	22/666	295/18614	0.000987718391109241	0.004055669082959	0.00232521842588879	AGT/AKT3/CXCL13/FGFR1/ITGB3/MEF2C/MMP9/NRP2/P2RX4/PIK3CD/PIK3CG/PLCG1/PLCG2/PROX1/PTK2B/PTN/SLIT2/SOX9/TACR1/VEGFA/WNT5A/WNT7A	22	0.745762711864407
GO:0090183	BP	GO:0090183	regulation of kidney development	6/666	33/18614	0.000994853798762187	0.00407815381227368	0.00233810949412789	AGT/GDNF/LHX1/SOX9/VEGFA/WNT2B	6	1.81818181818182
GO:1990776	BP	GO:1990776	response to angiotensin	6/666	33/18614	0.000994853798762187	0.00407815381227368	0.00233810949412789	AGT/AGTR1/CAMK2A/CAV1/COL3A1/INHBA	6	1.81818181818182
GO:2000377	BP	GO:2000377	regulation of reactive oxygen species metabolic process	14/666	150/18614	0.000998895230986017	0.00409130838358023	0.00234565134504348	AGT/AGTR1/BCL2/F2/F2RL1/LEP/MAPT/PINK1/PLCG2/PRKN/PTK2B/SLC18A2/SNCA/TRAP1	14	0.933333333333333
GO:0050670	BP	GO:0050670	regulation of lymphocyte proliferation	19/666	239/18614	0.0010133641457869	0.00414711471818702	0.00237764653864896	BCL2/BID/CCL5/CCR2/CHRNB2/EPHB2/GAL/IGF1/IL1A/IL6ST/LEP/MEF2C/MIF/PRNP/RIPK2/SHH/SOS2/TACR1/WNT3A	19	0.794979079497908
GO:0090132	BP	GO:0090132	epithelium migration	26/666	374/18614	0.00101630059698032	0.00415416480152348	0.00238168853106567	AGT/AKT3/APELA/CXCL13/EDN2/FGFR1/ITGB3/LTB4R2/MEF2C/MMP9/NRP2/P2RX4/PIK3CD/PIK3CG/PLCG1/PLCG2/PROX1/PTK2B/PTN/SCG2/SLIT2/SOX9/TACR1/VEGFA/WNT5A/WNT7A	26	0.695187165775401

	ONTOLOGY	ID	Description	GeneRatio	BgRatio	pvalue	p.adjust	qvalue	geneID	Count	richFactor
GO:0061005	BP	GO:0061005	cell differentiation involved in kidney development	8/666	58/18614	0.00101677726474725	0.00415416480152348	0.00238168853106567	EDNRA/EDNRB/GDNF/LHX1/MAGI2/MEF2C/MMP9/SHH	8	1.37931034482759
GO:1901342	BP	GO:1901342	regulation of vasculature development	25/666	355/18614	0.00105164144468503	0.00429303795733134	0.00246130804985298	AGT/AKT3/APELA/CCR2/CHRNA7/CX3CR1/CXCL13/CXCL8/GAB1/HMGA2/IL1A/ITGB3/LEP/PIK3CD/PLCG1/PRKCB/PRL/PTK2B/RLN2/SEMA3E/SEMA6A/SULF1/TWIST1/VEGFA/WNT5A	25	0.704225352112676
GO:0008585	BP	GO:0008585	female gonad development	11/666	102/18614	0.00105303562430442	0.00429516190328319	0.00246252576218645	A2M/BCL2/BCL2L1/CGA/INHBA/LEP/ROBO2/SLIT2/SLIT3/VEGFA/VGF	11	1.07843137254902
GO:0010507	BP	GO:0010507	negative regulation of autophagy	10/666	87/18614	0.00107556616805657	0.00437616532781294	0.00250896708948016	ADRA1A/BCL2/HTR2B/LEP/LEPR/LRRK2/MAGEA3/MAGEA6/PINK1/SNCA	10	1.14942528735632
GO:0032092	BP	GO:0032092	positive regulation of protein binding	10/666	87/18614	0.00107556616805657	0.00437616532781294	0.00250896708948016	BDNF/CAV1/GCG/LRRK2/MMP9/PLCL1/PRKN/RIPK2/WNT3A/WNT5A	10	1.14942528735632
GO:0048145	BP	GO:0048145	regulation of fibroblast proliferation	10/666	87/18614	0.00107556616805657	0.00437616532781294	0.00250896708948016	AGT/CAV1/CCNB1/IGF1/ITGB3/MIF/NGFR/WNT1/WNT2/WNT5A	10	1.14942528735632
GO:1902903	BP	GO:1902903	regulation of supramolecular fiber organization	26/666	376/18614	0.00109735635752725	0.00446113027067528	0.00255767965617556	APC/CCR7/CHRNA7/CLU/CXCL12/F2RL1/FGF13/LIMK1/LPAR1/MAP1B/MAP2/MAPT/PIK3R2/PRKN/PROX1/PTGER4/PTK2B/S1PR1/SLIT2/SNCA/SPTBN2/TACR1/TENM1/TMOD2/TUBB4A/WNT11	26	0.691489361702128
GO:0009895	BP	GO:0009895	negative regulation of catabolic process	24/666	337/18614	0.00112751368599717	0.00457994195593066	0.00262579742271807	ADRA1A/ADRA2A/APOC1/AXIN2/BCL2/CNR1/DDIT4/GDNF/GRIN2A/HSP90AB1/HTR2B/LEP/LEPR/LRRK2/MAD2L1/MAGEA3/MAGEA6/PIK3CG/PINK1/PRKCG/SHH/SNCA/VIP/WNT1	24	0.712166172106825
GO:0021533	BP	GO:0021533	cell differentiation in hindbrain	5/666	23/18614	0.00113680870042335	0.00461388502277518	0.00264525784782763	GRID2/LHX1/PHOX2B/PROX1/WNT7A	5	2.17391304347826
GO:0014911	BP	GO:0014911	positive regulation of smooth muscle cell migration	7/666	46/18614	0.00115525075142817	0.00468486587728505	0.00268595298034846	AGT/BCL2/CCL5/IGF1/ITGB3/LPAR1/SEMA6D	7	1.52173913043478
GO:0042462	BP	GO:0042462	eye photoreceptor cell development	6/666	34/18614	0.00117196488018176	0.00474091142888343	0.0027180853231506	GNGT1/HCN1/OLFM3/TH/THRB/VEGFA	6	1.76470588235294
GO:0070633	BP	GO:0070633	transepithelial transport	6/666	34/18614	0.00117196488018176	0.00474091142888343	0.0027180853231506	CLDN19/EDNRB/ITPR1/P2RY4/SLC1A1/SLC1A2	6	1.76470588235294
GO:0086004	BP	GO:0086004	regulation of cardiac muscle cell contraction	6/666	34/18614	0.00117196488018176	0.00474091142888343	0.0027180853231506	ATP1A2/ATP2A1/CACNA1C/CAMK2D/CAV1/FGF13	6	1.76470588235294
GO:0007254	BP	GO:0007254	JNK cascade	15/666	170/18614	0.0011913118594663	0.00481521199776056	0.0027606837325063	CCR7/F2RL1/IL1A/IRAK1/LRRK2/MAPK10/PINK1/PRKN/PTGER4/PTK2B/RIPK2/WNT16/WNT5A/WNT7A/WNT7B	15	0.882352941176471
GO:0001101	BP	GO:0001101	response to acid chemical	13/666	136/18614	0.00120054391101986	0.00484455937821233	0.00277750933350632	BCL2/BCL2L1/CAPN2/COL3A1/COL4A6/GLRA3/HPCA/NSMF/SLC1A2/SST/TH/TUBA1A/VEGFA	13	0.955882352941176
GO:0071333	BP	GO:0071333	cellular response to glucose stimulus	13/666	136/18614	0.00120054391101986	0.00484455937821233	0.00277750933350632	ADCY5/ADRA2A/EFNA5/FKBP1B/GCG/GHRHR/GPER1/GRIK5/NEUROD1/NOX4/NPTX1/SLC29A1/TH	13	0.955882352941176
GO:0021819	BP	GO:0021819	layer formation in cerebral cortex	4/666	14/18614	0.00122037847314404	0.0049004576760645	0.00280955725198365	ADGRG1/CDK5R2/COL3A1/RELN	4	2.85714285714286
GO:0030252	BP	GO:0030252	growth hormone secretion	4/666	14/18614	0.00122037847314404	0.0049004576760645	0.00280955725198365	ADCYAP1/DRD2/GHRHR/KISS1	4	2.85714285714286

	ONTOLOGY	ID	Description	GeneRatio	BgRatio	pvalue	p.adjust	qvalue	geneID	Count	richFactor
GO:0050930	BP	GO:0050930	induction of positive chemotaxis	4/666	14/18614	0.00122037847314404	0.0049004576760645	0.00280955725198365	CXCL12/CXCL8/SCG2/VEGFA	4	2.85714285714286
GO:0055057	BP	GO:0055057	neuroblast division	4/666	14/18614	0.00122037847314404	0.0049004576760645	0.00280955725198365	ASPM/FGF13/SOX5/WNT3A	4	2.85714285714286
GO:0099150	BP	GO:0099150	regulation of postsynaptic specialization assembly	4/666	14/18614	0.00122037847314404	0.0049004576760645	0.00280955725198365	GAP43/GRID2/PTK2B/SPTBN2	4	2.85714285714286
GO:1903909	BP	GO:1903909	regulation of receptor clustering	4/666	14/18614	0.00122037847314404	0.0049004576760645	0.00280955725198365	FRRS1L/FZD9/SHISA7/SLC7A11	4	2.85714285714286
GO:0090130	BP	GO:0090130	tissue migration	26/666	379/18614	0.00122943089176211	0.00493277782286592	0.00282808721567962	AGT/AKT3/APELA/CXCL13/EDN2/FGFR1/ITGB3/LTB4R2/MEF2C/MMP9/NRP2/P2RX4/PIK3CD/PIK3CG/PLCG1/PLCG2/PROX1/PTK2B/PTN/SCG2/SLIT2/SOX9/TACR1/VEGFA/WNT5A/WNT7A	26	0.686015831134565
GO:0048738	BP	GO:0048738	cardiac muscle tissue development	19/666	243/18614	0.00123235579204008	0.00494048019402692	0.00283250318132678	ADRA1A/AGT/CDK1/DKK1/EDNRA/ERBB4/IGF1/MEF2C/NOX4/POU4F1/PROX1/RGS2/S1PR1/TBX20/TP73/VEGFA/WNT2/WNT3A/WNT5A	19	0.781893004115226
GO:0071774	BP	GO:0071774	response to fibroblast growth factor	12/666	120/18614	0.00123879849199315	0.00496226127803285	0.00284499083178111	APLN/CCL5/CXCL13/CXCL8/FGFR1/FLRT1/FLRT2/LHX1/NDNF/NGFR/SULF1/WNT5A	12	1
GO:0045785	BP	GO:0045785	positive regulation of cell adhesion	31/666	482/18614	0.00125645717246274	0.0050288982106306	0.00288319548318427	ADGRG1/ANK3/CAV1/CCL5/CCR2/CCR7/CSF1/CTSG/CXCL12/CXCL13/EPHA4/IGF1/IL1A/IL6ST/IRAK1/ITGA4/ITGB3/LEP/NDNF/NPY/PDPN/PIK3R2/PRSS2/PTK2B/RIPK2/SHH/SOX2/VEGFA/VWC2/WNT3A/WNT5A	31	0.643153526970954
GO:0001736	BP	GO:0001736	establishment of planar polarity	9/666	74/18614	0.00126250551738652	0.00504079172863913	0.00289001434011179	CELSR1/DKK1/ERBB4/FZD3/GPC6/MAGI2/WNT11/WNT5A/WNT7A	9	1.21621621621622
GO:0007164	BP	GO:0007164	establishment of tissue polarity	9/666	74/18614	0.00126250551738652	0.00504079172863913	0.00289001434011179	CELSR1/DKK1/ERBB4/FZD3/GPC6/MAGI2/WNT11/WNT5A/WNT7A	9	1.21621621621622
GO:0060420	BP	GO:0060420	regulation of heart growth	9/666	74/18614	0.00126250551738652	0.00504079172863913	0.00289001434011179	CDK1/ERBB4/IGF1/MEF2C/PROX1/RGS2/TBX20/TP73/WNT2	9	1.21621621621622
GO:0051591	BP	GO:0051591	response to cAMP	10/666	89/18614	0.00128270247207722	0.00511727487845743	0.00293386407873439	FOSB/HCN1/KCNQ1/OXT/PENK/PIK3CG/PTK2B/RAP1A/SLC6A3/VGF	10	1.12359550561798
GO:0001754	BP	GO:0001754	eye photoreceptor cell differentiation	7/666	47/18614	0.00131620548677741	0.00524242298825847	0.00300561467892443	GNGT1/HCN1/OLFM3/SOX9/TH/THRB/VEGFA	7	1.48936170212766
GO:1905314	BP	GO:1905314	semi-lunar valve development	7/666	47/18614	0.00131620548677741	0.00524242298825847	0.00300561467892443	AXIN2/ROBO2/SLIT2/SLIT3/SOX9/TBX20/TWIST1	7	1.48936170212766
GO:0032760	BP	GO:0032760	positive regulation of tumor necrosis factor production	11/666	105/18614	0.00133759448902779	0.00532330114459238	0.00305198418676957	CCR2/CLU/EPHB2/IL1A/LEP/LRRK2/MIF/PLCG2/RIPK2/TWIST1/WNT5A	11	1.04761904761905
GO:0010970	BP	GO:0010970	transport along microtubule	15/666	172/18614	0.00134015389893677	0.00532917185539987	0.00305535001486437	CALY/DLG2/KIF1A/KIF5A/KIF5C/KLC3/MAP1B/MAP2/MAPT/NEFH/NEFL/TMEM108/TRAK2/TUBA1A/UCHL1	15	0.872093023255814
GO:0071331	BP	GO:0071331	cellular response to hexose stimulus	13/666	138/18614	0.001372462764736	0.00545323725842962	0.00312647987167449	ADCY5/ADRA2A/EFNA5/FKBP1B/GCG/GHRHR/GPER1/GRIK5/NEUROD1/NOX4/NPTX1/SLC29A1/TH	13	0.942028985507246

	ONTOLOGY	ID	Description	GeneRatio	BgRatio	pvalue	p.adjust	qvalue	geneID	Count	richFactor
GO:0010639	BP	GO:0010639	negative regulation of organelle organization	25/666	362/18614	0.0013802366853672	0.00547969572583181	0.00314164918521213	APC/BCL2L1/BUB1B/CCNB1/CLU/DUSP1/FGF13/FZD9/IGF1/LRRK2/MAD2L1/MAP1B/MAP2/MAPT/MCM2/PINK1/PRKN/S1PR1/SLIT2/SNCA/SPTBN2/TMOD2/TOP2A/TTK/TUBB4A	25	0.69060773480663
GO:0042438	BP	GO:0042438	melanin biosynthetic process	5/666	24/18614	0.00139413206700821	0.00552593476560108	0.00316815920490744	ASIP/MC1R/SLC7A11/TRPC1/WNT5A	5	2.08333333333333
GO:0060571	BP	GO:0060571	morphogenesis of an epithelial fold	5/666	24/18614	0.00139413206700821	0.00552593476560108	0.00316815920490744	SHH/SULF1/WNT2/WNT2B/WNT5A	5	2.08333333333333
GO:0097191	BP	GO:0097191	extrinsic apoptotic signaling pathway	18/666	227/18614	0.00139940451181234	0.00554236355806418	0.00317757825024101	AGT/BCL2/BCL2L1/BID/CAV1/FAS/FGFR1/GABARAP/GDNF/GPER1/IGF1/IL1A/IL6R/INHBA/NDUFA13/NGF/P2RX7/SCG2	18	0.79295154185022
GO:2001242	BP	GO:2001242	regulation of intrinsic apoptotic signaling pathway	15/666	173/18614	0.00142024259898848	0.0056203642302966	0.00322229802313091	BCL2/BCL2L1/BDKRB2/BID/CAV1/CLU/CXCL12/LRRK2/MAGEA3/MIF/MMP9/NDUFA13/PINK1/PRKN/TRAP1	15	0.867052023121387
GO:0072676	BP	GO:0072676	lymphocyte migration	12/666	122/18614	0.00142996023041327	0.00565426752412003	0.00324173564535404	CCL5/CCR2/CCR7/CXCL12/CXCL13/ITGA4/ITGB3/PIK3CD/PIK3CG/PTK2B/S1PR1/WNT5A	12	0.983606557377049
GO:0090090	BP	GO:0090090	negative regulation of canonical Wnt signaling pathway	13/666	139/18614	0.00146580941123561	0.00579136113844296	0.00332033490766436	APC/AXIN2/CAV1/CSNK1A1L/DKK1/DKK4/PRKN/SHH/SOX10/SOX2/SOX9/WNT11/WNT5A	13	0.935251798561151
GO:0006953	BP	GO:0006953	acute-phase response	7/666	48/18614	0.00149416577802294	0.00588447499918492	0.00337371945661895	A2M/CNR1/EDNRB/F2/IL1A/IL6R/PTGER3	7	1.45833333333333
GO:0035850	BP	GO:0035850	epithelial cell differentiation involved in kidney development	7/666	48/18614	0.00149416577802294	0.00588447499918492	0.00337371945661895	EDNRA/EDNRB/GDNF/LHX1/MAGI2/MEF2C/MMP9	7	1.45833333333333
GO:0048066	BP	GO:0048066	developmental pigmentation	7/666	48/18614	0.00149416577802294	0.00588447499918492	0.00337371945661895	BCL2/EDN3/EDNRB/GNA11/GPR143/MEF2C/SOX10	7	1.45833333333333
GO:2000404	BP	GO:2000404	regulation of T cell migration	7/666	48/18614	0.00149416577802294	0.00588447499918492	0.00337371945661895	CCL5/CCR2/CXCL12/CXCL13/ITGA4/ITGB3/WNT5A	7	1.45833333333333
GO:0001942	BP	GO:0001942	hair follicle development	10/666	91/18614	0.00152122392537472	0.00597667113766325	0.00342657785201575	BCL2/DKK1/DKK4/FZD3/GAL/INHBA/NGFR/SHH/SOX9/WNT5A	10	1.0989010989011
GO:0030901	BP	GO:0030901	midbrain development	10/666	91/18614	0.00152122392537472	0.00597667113766325	0.00342657785201575	CSNK1E/DKK1/FZD3/MAOB/SHH/WNT1/WNT2/WNT3/WNT3A/WNT5A	10	1.0989010989011
GO:0072384	BP	GO:0072384	organelle transport along microtubule	10/666	91/18614	0.00152122392537472	0.00597667113766325	0.00342657785201575	KIF1A/KIF5A/MAP1B/MAP2/MAPT/NEFH/NEFL/TRAK2/TUBA1A/UCHL1	10	1.0989010989011
GO:1904951	BP	GO:1904951	positive regulation of establishment of protein localization	23/666	325/18614	0.00153801278785326	0.00603780579257091	0.00346162790741818	ANK3/CDK1/F2/F2RL1/F2RL2/GCG/GNA11/GPER1/HPCA/IGF1/IL1A/ITPR1/LEP/NKX6-1/P2RX7/PIK3R2/PINK1/PRKCB/PRNP/SHH/TENM1/UCN3/VAMP2	23	0.707692307692308
GO:0036294	BP	GO:0036294	cellular response to decreased oxygen levels	14/666	157/18614	0.0015501896180853	0.00608075177405366	0.00348624993288893	BCL2/EPAS1/EPHA4/GNGT1/IRAK1/NDNF/OPRD1/PINK1/SDHD/SLC29A1/SLC2A4/TWIST1/UCN3/VEGFA	14	0.89171974522293
GO:1904019	BP	GO:1904019	epithelial cell apoptotic process	13/666	140/18614	0.00156436512146942	0.00613146297609427	0.0035153239571675	ATOH1/BCL2/BCL2L1/BID/EDNRA/GPER1/ITGA4/NDNF/NEUROD1/PIK3CG/PLCG1/SCG2/WNT11	13	0.928571428571429

	ONTOLOGY	ID	Description	GeneRatio	BgRatio	pvalue	p.adjust	qvalue	geneID	Count	richFactor
GO:0046651	BP	GO:0046651	lymphocyte proliferation	22/666	306/18614	0.00157997229308554	0.00618770025539079	0.00354756622234431	BCL2/BID/CCL5/CCR2/CHRNB2/EPHB2/FKBP1B/GAL/IGF1/IL1A/IL6ST/LEP/MEF2C/MIF/P2RX7/PIK3CG/PRNP/RIPK2/SHH/SOS2/TACR1/WNT3A	22	0.718954248366013
GO:0001569	BP	GO:0001569	branching involved in blood vessel morphogenesis	6/666	36/18614	0.0015975860859502	0.00623185366067084	0.00357288049464386	EDNRA/GNA13/SEMA3E/SHH/TBX20/VEGFA	6	1.66666666666667
GO:0019934	BP	GO:0019934	cGMP-mediated signaling	6/666	36/18614	0.0015975860859502	0.00623185366067084	0.00357288049464386	EDNRB/GUCY1A1/HTR2B/HTR2C/PRKG1/PTK2B	6	1.66666666666667
GO:0021799	BP	GO:0021799	cerebral cortex radially oriented cell migration	6/666	36/18614	0.0015975860859502	0.00623185366067084	0.00357288049464386	ADGRG1/CDK5R2/COL3A1/FOXG1/MDGA1/RELN	6	1.66666666666667
GO:0050931	BP	GO:0050931	pigment cell differentiation	6/666	36/18614	0.0015975860859502	0.00623185366067084	0.00357288049464386	BCL2/EDN3/EDNRB/GNA11/MEF2C/SOX10	6	1.66666666666667
GO:0086005	BP	GO:0086005	ventricular cardiac muscle cell action potential	6/666	36/18614	0.0015975860859502	0.00623185366067084	0.00357288049464386	CACNA1C/CAV1/KCNJ5/KCNQ1/NEDD4L/SCN2B	6	1.66666666666667
GO:0002864	BP	GO:0002864	regulation of acute inflammatory response to antigenic stimulus	4/666	15/18614	0.00161736816609801	0.00627416301213238	0.00359713752390178	CCR7/CNR1/NPY/NPY5R	4	2.66666666666667
GO:0010820	BP	GO:0010820	positive regulation of T cell chemotaxis	4/666	15/18614	0.00161736816609801	0.00627416301213238	0.00359713752390178	CCL5/CCR2/CXCL13/WNT5A	4	2.66666666666667
GO:0032230	BP	GO:0032230	positive regulation of synaptic transmission, GABAergic	4/666	15/18614	0.00161736816609801	0.00627416301213238	0.00359713752390178	ADRA1A/NLGN1/OXTR/TACR1	4	2.66666666666667
GO:0044065	BP	GO:0044065	regulation of respiratory system process	4/666	15/18614	0.00161736816609801	0.00627416301213238	0.00359713752390178	ATP1A2/NLGN3/PHOX2B/TLX3	4	2.66666666666667
GO:0070207	BP	GO:0070207	protein homotrimerization	4/666	15/18614	0.00161736816609801	0.00627416301213238	0.00359713752390178	ASIC1/MIF/SIGMAR1/SLC1A2	4	2.66666666666667
GO:0072531	BP	GO:0072531	pyrimidine-containing compound transmembrane transport	4/666	15/18614	0.00161736816609801	0.00627416301213238	0.00359713752390178	SLC22A2/SLC28A2/SLC29A1/SLC44A4	4	2.66666666666667
GO:2000671	BP	GO:2000671	regulation of motor neuron apoptotic process	4/666	15/18614	0.00161736816609801	0.00627416301213238	0.00359713752390178	BCL2/CNTFR/CRLF1/NEFL	4	2.66666666666667
GO:0043280	BP	GO:0043280	positive regulation of cysteine-type endopeptidase activity involved in apoptotic process	12/666	124/18614	0.00164483821396145	0.00637569386563131	0.00365534774927344	ATP2A3/BID/FAS/GPER1/MAPT/NDUFA13/NGFR/P2RX1/SLC1A1/SLC22A2/SNCA/WNT3A	12	0.967741935483871
GO:0006809	BP	GO:0006809	nitric oxide biosynthetic process	9/666	77/18614	0.00167635153891033	0.00649272483352582	0.00372244458517934	AGT/CAV1/CLU/CX3CR1/GCHFR/HSP90AB1/NOS1/P2RX4/PTK2B	9	1.16883116883117
GO:0014013	BP	GO:0014013	regulation of gliogenesis	11/666	108/18614	0.00168241136834247	0.00651106446882144	0.00373295916539808	ATOH1/F2/IL6ST/MAG/NKX6-1/NTN1/NTRK3/PTN/SHH/SOX10/TP73	11	1.01851851851852
GO:0043114	BP	GO:0043114	regulation of vascular permeability	7/666	49/18614	0.00169032988355126	0.00652738468270956	0.00374231595986796	BDKRB2/SH3GL2/SLIT2/TACR1/TACR2/UCN/VEGFA	7	1.42857142857143
GO:0061028	BP	GO:0061028	establishment of endothelial barrier	7/666	49/18614	0.00169032988355126	0.00652738468270956	0.00374231595986796	CLDN1/EDNRA/EDNRB/F2RL1/RAP1A/VCL/VEGFA	7	1.42857142857143
GO:0016048	BP	GO:0016048	detection of temperature stimulus	5/666	25/18614	0.00169194060748158	0.00652738468270956	0.00374231595986796	CALCA/CXCL12/GRIK2/NGFR/NTSR1	5	2
GO:0086064	BP	GO:0086064	cell communication by electrical coupling involved in cardiac conduction	5/666	25/18614	0.00169194060748158	0.00652738468270956	0.00374231595986796	ATP1A2/CACNA1C/CAMK2D/CAV1/FKBP1B	5	2
GO:0051092	BP	GO:0051092	positive regulation of NF-kappaB transcription factor activity	14/666	159/18614	0.00174771205201666	0.00673725861620539	0.00386264203364177	AGT/CAMK2A/CAV1/CLU/CX3CR1/IRAK1/IRAK2/NTSR1/PLCG2/PRKCB/PSMA6/RIPK2/RPS6KA5/WNT5A	14	0.880503144654088

	ONTOLOGY	ID	Description	GeneRatio	BgRatio	pvalue	p.adjust	qvalue	geneID	Count	richFactor
GO:0051353	BP	GO:0051353	positive regulation of oxidoreductase activity	8/666	63/18614	0.00175737242327775	0.00676388837933447	0.00387790955538435	AGT/AGTR1/EDN2/EDN3/GDNF/HTR2B/PTK2B/SNCA	8	1.26984126984127
GO:0070059	BP	GO:0070059	intrinsic apoptotic signaling pathway in response to endoplasmic reticulum stress	8/666	63/18614	0.00175737242327775	0.00676388837933447	0.00387790955538435	ATP2A1/ATP2A3/BCL2/BCL2L1/ITPR1/LRRK2/MAGEA3/PRKN	8	1.26984126984127
GO:0001952	BP	GO:0001952	regulation of cell-matrix adhesion	12/666	125/18614	0.00176184455714992	0.00677579499091694	0.00388473591919145	BCL2/CCR7/CSF1/EFNA5/EPHA3/GPM6B/ITGB3/NEXMIF/PTK2B/SEMA3E/VCL/VEGFA	12	0.96
GO:0010594	BP	GO:0010594	regulation of endothelial cell migration	18/666	232/18614	0.00178411080598543	0.0068560630269104	0.0039307556294992	AGT/AKT3/CXCL13/FGFR1/ITGB3/MEF2C/NRP2/P2RX4/PIK3CD/PIK3CG/PLCG1/PROX1/PTK2B/PTN/SLIT2/VEGFA/WNT5A/WNT7A	18	0.775862068965517
GO:0031330	BP	GO:0031330	negative regulation of cellular catabolic process	19/666	251/18614	0.00179312194728872	0.00688530810228442	0.00394752257639137	ADRA1A/APOC1/AXIN2/BCL2/CNR1/GDNF/HSP90AB1/HTR2B/LEP/LEPR/LRRK2/MAGEA3/MAGEA6/PIK3CG/PINK1/SHH/SNCA/VIP/WNT1	19	0.756972111553785
GO:0051781	BP	GO:0051781	positive regulation of cell division	10/666	93/18614	0.00179453130687843	0.00688534065051327	0.00394754123712031	CXCR5/DRD2/HTR2B/IL1A/KIF14/PTN/RXFP3/SHH/SSTR5/VEGFA	10	1.0752688172043
GO:1903557	BP	GO:1903557	positive regulation of tumor necrosis factor superfamily cytokine production	11/666	109/18614	0.00181229441197332	0.00694807101002251	0.00398350615063024	CCR2/CLU/EPHB2/IL1A/LEP/LRRK2/MIF/PLCG2/RIPK2/TWIST1/WNT5A	11	1.00917431192661
GO:0050709	BP	GO:0050709	negative regulation of protein secretion	9/666	78/18614	0.00183620218700773	0.00703424298452298	0.00403291073931959	ADRA2A/DRD2/DRD4/F2RL1/FKBP1B/GNAZ/OPRM1/PRKN/SYT4	9	1.15384615384615
GO:0010934	BP	GO:0010934	macrophage cytokine production	6/666	37/18614	0.0018499856233763	0.00704858863480195	0.00404113546615941	AXL/P2RX7/PLCG2/RIPK2/TWIST1/WNT5A	6	1.62162162162162
GO:0010935	BP	GO:0010935	regulation of macrophage cytokine production	6/666	37/18614	0.0018499856233763	0.00704858863480195	0.00404113546615941	AXL/P2RX7/PLCG2/RIPK2/TWIST1/WNT5A	6	1.62162162162162
GO:0034110	BP	GO:0034110	regulation of homotypic cell-cell adhesion	6/666	37/18614	0.0018499856233763	0.00704858863480195	0.00404113546615941	ANK3/CCL5/CTSG/IL6ST/PDPN/PRKG1	6	1.62162162162162
GO:0045920	BP	GO:0045920	negative regulation of exocytosis	6/666	37/18614	0.0018499856233763	0.00704858863480195	0.00404113546615941	ADRA2A/CCR2/PRKN/RAP1A/SNCA/SYT4	6	1.62162162162162
GO:0048741	BP	GO:0048741	skeletal muscle fiber development	6/666	37/18614	0.0018499856233763	0.00704858863480195	0.00404113546615941	BCL2/CNTNAP1/DNER/HOMER1/P2RX2/RYR1	6	1.62162162162162
GO:0055023	BP	GO:0055023	positive regulation of cardiac muscle tissue growth	6/666	37/18614	0.0018499856233763	0.00704858863480195	0.00404113546615941	CDK1/ERBB4/IGF1/MEF2C/TBX20/WNT2	6	1.62162162162162
GO:0086019	BP	GO:0086019	cell-cell signaling involved in cardiac conduction	6/666	37/18614	0.0018499856233763	0.00704858863480195	0.00404113546615941	CACNA1C/CACNA1D/HCN1/KCNJ5/KCNQ1/SCN1B	6	1.62162162162162
GO:0003231	BP	GO:0003231	cardiac ventricle development	12/666	126/18614	0.00188560283558847	0.00717872806887478	0.0041157477197361	EDNRA/MEF2C/NPY2R/NPY5R/POU4F1/PROX1/ROBO2/SLIT2/SLIT3/TBX20/WNT11/WNT5A	12	0.952380952380952
GO:0010631	BP	GO:0010631	epithelial cell migration	25/666	371/18614	0.00192976798273879	0.00734118392814332	0.00420888780331738	AGT/AKT3/CXCL13/EDN2/FGFR1/ITGB3/LTB4R2/MEF2C/MMP9/NRP2/P2RX4/PIK3CD/PIK3CG/PLCG1/PLCG2/PROX1/PTK2B/PTN/SCG2/SLIT2/SOX9/TACR1/VEGFA/WNT5A/WNT7A	25	0.673854447439353
GO:0022404	BP	GO:0022404	molting cycle process	10/666	94/18614	0.0019453465968807	0.00738900967826015	0.00423630752448518	BCL2/DKK1/DKK4/FZD3/GAL/INHBA/NGFR/SHH/SOX9/WNT5A	10	1.06382978723404

	ONTOLOGY	ID	Description	GeneRatio	BgRatio	pvalue	p.adjust	qvalue	geneID	Count	richFactor
GO:0022405	BP	GO:0022405	hair cycle process	10/666	94/18614	0.0019453465968807	0.00738900967826015	0.00423630752448518	BCL2/DKK1/DKK4/FZD3/GAL/INHBA/NGFR/SHH/SOX9/WNT5A	10	1.06382978723404
GO:0009408	BP	GO:0009408	response to heat	11/666	110/18614	0.00195023525997801	0.00740185814887408	0.00424367388007409	CALCA/CXCL12/HSP90AB1/IGF1/IL1A/IRAK1/MAPT/NOS1/SCN2B/SST/TACR1	11	1
GO:0008584	BP	GO:0008584	male gonad development	13/666	144/18614	0.00201516976703208	0.0076411850812471	0.00438088610858242	ADGRG1/ASPM/BCL2/BCL2L1/CCND1/GFRA1/IL1A/INHBA/LHB/NKX2-1/SOX9/WNT2B/WNT5A	13	0.902777777777778
GO:0090288	BP	GO:0090288	negative regulation of cellular response to growth factor stimulus	12/666	127/18614	0.00201640224829654	0.0076411850812471	0.00438088610858242	AGT/APLN/CHRDL1/CXCL13/DKK1/NGFR/SEMA6A/SLIT2/SULF1/VWC2/WNT1/WNT5A	12	0.94488188976378
GO:0010876	BP	GO:0010876	lipid localization	31/666	497/18614	0.00202603979487172	0.00767179167318528	0.004398433647613	AGT/AGTR1/APOC1/AVPR1B/BDKRB2/CAV1/CLU/DRD2/DRD4/FABP7/GAL/GALR1/IL1A/INHBA/ITGB3/KCNQ1/LEP/MAP2K6/MIF/NMB/NMUR2/NTSR1/OXT/P2RX4/P2RX7/PRKN/SHH/SIGMAR1/SLC22A2/SPX/SYT7	31	0.62374245472837
GO:0044550	BP	GO:0044550	secondary metabolite biosynthetic process	5/666	26/18614	0.00203386782977681	0.00767188057049349	0.00439848461470495	ASIP/MC1R/SLC7A11/TRPC1/WNT5A	5	1.92307692307692
GO:0045822	BP	GO:0045822	negative regulation of heart contraction	5/666	26/18614	0.00203386782977681	0.00767188057049349	0.00439848461470495	ADRA1A/ATP1A2/FKBP1B/PIK3CG/SPX	5	1.92307692307692
GO:0048596	BP	GO:0048596	embryonic camera-type eye morphogenesis	5/666	26/18614	0.00203386782977681	0.00767188057049349	0.00439848461470495	PROX1/TH/TWIST1/WNT16/WNT5A	5	1.92307692307692
GO:1901623	BP	GO:1901623	regulation of lymphocyte chemotaxis	5/666	26/18614	0.00203386782977681	0.00767188057049349	0.00439848461470495	CCL5/CCR2/CXCL13/PTK2B/WNT5A	5	1.92307692307692
GO:2000773	BP	GO:2000773	negative regulation of cellular senescence	5/666	26/18614	0.00203386782977681	0.00767188057049349	0.00439848461470495	AKT3/HMGA2/MIF/TWIST1/WNT1	5	1.92307692307692
GO:0050727	BP	GO:0050727	regulation of inflammatory response	27/666	414/18614	0.00207316439483692	0.00781411273053948	0.00448002993621121	AGT/AGTR1/CCL5/CCR2/CCR7/CNR1/CNR2/CREB3L3/EDNRB/GPER1/IGF1/IL6ST/LRRK2/MGLL/MMP9/NPY/NPY5R/PIK3CG/PLCG1/PLCG2/PSMA6/PTGER3/PTGER4/SNCA/SYT11/TYRO3/WNT5A	27	0.652173913043478
GO:0003188	BP	GO:0003188	heart valve formation	4/666	16/18614	0.00209594256005169	0.00785778618051414	0.00450506903790466	OLFM1/SOX9/TBX20/TWIST1	4	2.5
GO:0010819	BP	GO:0010819	regulation of T cell chemotaxis	4/666	16/18614	0.00209594256005169	0.00785778618051414	0.00450506903790466	CCL5/CCR2/CXCL13/WNT5A	4	2.5
GO:0021535	BP	GO:0021535	cell migration in hindbrain	4/666	16/18614	0.00209594256005169	0.00785778618051414	0.00450506903790466	EPHB2/PHOX2B/PLXNA2/POU4F1	4	2.5
GO:0021984	BP	GO:0021984	adenohypophysis development	4/666	16/18614	0.00209594256005169	0.00785778618051414	0.00450506903790466	DRD2/GHRHR/SLC6A3/SOX2	4	2.5
GO:0048172	BP	GO:0048172	regulation of short-term neuronal synaptic plasticity	4/666	16/18614	0.00209594256005169	0.00785778618051414	0.00450506903790466	GRIK2/SHISA7/SYP/SYT4	4	2.5
GO:2000650	BP	GO:2000650	negative regulation of sodium ion transmembrane transporter activity	4/666	16/18614	0.00209594256005169	0.00785778618051414	0.00450506903790466	ATP1A2/CAMK2D/GRP/NEDD4L	4	2.5
GO:2000846	BP	GO:2000846	regulation of corticosteroid hormone secretion	4/666	16/18614	0.00209594256005169	0.00785778618051414	0.00450506903790466	AGT/AGTR1/GAL/GALR1	4	2.5
GO:0046676	BP	GO:0046676	negative regulation of insulin secretion	6/666	38/18614	0.00213145426226165	0.00797874919955524	0.00457442021892368	ADRA2A/DRD2/F2RL1/FKBP1B/GNAZ/PRKN	6	1.57894736842105
GO:1902229	BP	GO:1902229	regulation of intrinsic apoptotic signaling pathway in response to DNA damage	6/666	38/18614	0.00213145426226165	0.00797874919955524	0.00457442021892368	BCL2/BCL2L1/BID/CLU/CXCL12/MIF	6	1.57894736842105

	ONTOLOGY	ID	Description	GeneRatio	BgRatio	pvalue	p.adjust	qvalue	geneID	Count	richFactor
GO:0050885	BP	GO:0050885	neuromuscular process controlling balance	7/666	51/18614	0.00214223479331898	0.0079922405366922	0.00458215514627082	ADCY5/CNTNAP1/GPR88/NEFL/NRXN1/POU4F1/TNR	7	1.37254901960784
GO:0051489	BP	GO:0051489	regulation of filopodium assembly	7/666	51/18614	0.00214223479331898	0.0079922405366922	0.00458215514627082	ARHGAP44/CCR7/EPHB2/GAP43/GPM6A/NLGN1/TENM1	7	1.37254901960784
GO:0051646	BP	GO:0051646	mitochondrion localization	7/666	51/18614	0.00214223479331898	0.0079922405366922	0.00458215514627082	ATP2A1/LRRK2/MAP1B/MAPT/NEFL/TRAK2/UCHL1	7	1.37254901960784
GO:0061082	BP	GO:0061082	myeloid leukocyte cytokine production	7/666	51/18614	0.00214223479331898	0.0079922405366922	0.00458215514627082	AXL/MIF/P2RX7/PLCG2/RIPK2/TWIST1/WNT5A	7	1.37254901960784
GO:0046546	BP	GO:0046546	development of primary male sexual characteristics	13/666	145/18614	0.00214318881533272	0.0079922405366922	0.00458215514627082	ADGRG1/ASPM/BCL2/BCL2L1/CCND1/GFRA1/IL1A/INHBA/LHB/NKX2-1/SOX9/WNT2B/WNT5A	13	0.896551724137931
GO:0048857	BP	GO:0048857	neural nucleus development	8/666	65/18614	0.00215186107405767	0.00801242210529808	0.00459372575378979	ASCL1/BCL2/CDK5R2/CHRNB2/KIRREL3/MAOB/NKX2-1/PHOX2B	8	1.23076923076923
GO:0080164	BP	GO:0080164	regulation of nitric oxide metabolic process	8/666	65/18614	0.00215186107405767	0.00801242210529808	0.00459372575378979	AGT/CAV1/CLU/CX3CR1/HSP90AB1/NPY/P2RX4/PTK2B	8	1.23076923076923
GO:1904375	BP	GO:1904375	regulation of protein localization to cell periphery	12/666	128/18614	0.00215453908103198	0.008016320653499	0.00459596089081048	ARHGAP44/BCL2L1/CPLX1/EPHA3/EPHB2/GNAI1/GPC6/GPER1/PIK3R2/PRNP/RAP1A/WNT3A	12	0.9375
GO:0030198	BP	GO:0030198	extracellular matrix organization	22/666	314/18614	0.00218153087386669	0.00811060835480695	0.00465001843247164	AGT/AXIN2/CAV1/COL3A1/COL4A6/CTSG/FLRT2/GPM6B/ITGB3/MMP1/MMP11/MMP13/MMP7/MMP9/NDNF/PDPN/PRSS1/PRSS2/SOX9/SULF1/TNR/WNT3A	22	0.700636942675159
GO:0043473	BP	GO:0043473	pigmentation	11/666	112/18614	0.00225176022089265	0.0083653828312074	0.00479608713158263	ASIP/BCL2/DRD2/EDN3/EDNRB/GNA11/GPR143/MC1R/MEF2C/SOX10/TH	11	0.982142857142857
GO:0043062	BP	GO:0043062	extracellular structure organization	22/666	315/18614	0.00226886305790096	0.00842255432747978	0.00482886500715605	AGT/AXIN2/CAV1/COL3A1/COL4A6/CTSG/FLRT2/GPM6B/ITGB3/MMP1/MMP11/MMP13/MMP7/MMP9/NDNF/PDPN/PRSS1/PRSS2/SOX9/SULF1/TNR/WNT3A	22	0.698412698412698
GO:0050714	BP	GO:0050714	positive regulation of protein secretion	13/666	146/18614	0.00227782375445485	0.00844306467054721	0.00484062410945117	F2/F2RL1/F2RL2/GCG/GNA11/GPER1/IGF1/IL1A/ITPR1/NKX6-1/P2RX7/PRKCB/UCN3	13	0.89041095890411
GO:0062013	BP	GO:0062013	positive regulation of small molecule metabolic process	13/666	146/18614	0.00227782375445485	0.00844306467054721	0.00484062410945117	ADCYAP1R1/AVPR1B/GCG/GPER1/IGF1/NOS1/NTSR1/P2RX7/P2RY1/PINK1/PTH1R/SNCA/TWIST1	13	0.89041095890411
GO:0001655	BP	GO:0001655	urogenital system development	8/666	66/18614	0.00237356284526804	0.0087780747814089	0.00503269394224589	EPHB2/EPHB3/LHX1/SHH/SOX9/SULF1/WNT11/WNT5A	8	1.21212121212121
GO:0048814	BP	GO:0048814	regulation of dendrite morphogenesis	8/666	66/18614	0.00237356284526804	0.0087780747814089	0.00503269394224589	CAMK2B/CHRNA3/CHRNB2/EPHA4/EPHB2/NEDD4L/NSMF/RELN	8	1.21212121212121
GO:2000272	BP	GO:2000272	negative regulation of signaling receptor activity	8/666	66/18614	0.00237356284526804	0.0087780747814089	0.00503269394224589	DKK1/DKK4/EPHB2/LY6E/LY6G6D/LY6H/LYNX1/SLC6A9	8	1.21212121212121
GO:0043299	BP	GO:0043299	leukocyte degranulation	9/666	81/18614	0.00239000970316713	0.00883225390305747	0.00506375621320016	CCR2/F2RL1/GRP/PIK3CD/PIK3CG/PTGDR/SLC18A2/STXBP1/VAMP2	9	1.11111111111111
GO:0021695	BP	GO:0021695	cerebellar cortex development	7/666	52/18614	0.00240053839011796	0.00884456235939263	0.00507081296484319	GRID2/KIF14/LHX1/PROX1/SPTBN2/TUBA1A/WNT7A	7	1.34615384615385
GO:0046622	BP	GO:0046622	positive regulation of organ growth	7/666	52/18614	0.00240053839011796	0.00884456235939263	0.00507081296484319	CDK1/ERBB4/IGF1/MEF2C/PROX1/TBX20/WNT2	7	1.34615384615385

	ONTOLOGY	ID	Description	GeneRatio	BgRatio	pvalue	p.adjust	qvalue	geneID	Count	richFactor
GO:0048013	BP	GO:0048013	ephrin receptor signaling pathway	7/666	52/18614	0.00240053839011796	0.00884456235939263	0.00507081296484319	EFNA5/EPHA3/EPHA4/EPHB2/EPHB3/MMP9/NTRK3	7	1.34615384615385
GO:0060425	BP	GO:0060425	lung morphogenesis	7/666	52/18614	0.00240053839011796	0.00884456235939263	0.00507081296484319	CELSR1/NKX2-1/SHH/SOX9/WNT2/WNT2B/WNT7B	7	1.34615384615385
GO:0009651	BP	GO:0009651	response to salt stress	5/666	27/18614	0.0024235937579569	0.00886306794669505	0.00508142268957598	BDKRB2/CLDN1/OXT/PTK2B/TH	5	1.85185185185185
GO:0021544	BP	GO:0021544	subpallium development	5/666	27/18614	0.0024235937579569	0.00886306794669505	0.00508142268957598	ASCL1/DRD2/INHBA/LRRK2/SLC7A11	5	1.85185185185185
GO:0021801	BP	GO:0021801	cerebral cortex radial glia-guided migration	5/666	27/18614	0.0024235937579569	0.00886306794669505	0.00508142268957598	ADGRG1/CDK5R2/COL3A1/FOXG1/RELN	5	1.85185185185185
GO:0022030	BP	GO:0022030	telencephalon glial cell migration	5/666	27/18614	0.0024235937579569	0.00886306794669505	0.00508142268957598	ADGRG1/CDK5R2/COL3A1/FOXG1/RELN	5	1.85185185185185
GO:0034643	BP	GO:0034643	establishment of mitochondrion localization, microtubule-mediated	5/666	27/18614	0.0024235937579569	0.00886306794669505	0.00508142268957598	MAP1B/MAPT/NEFL/TRAK2/UCHL1	5	1.85185185185185
GO:0047497	BP	GO:0047497	mitochondrion transport along microtubule	5/666	27/18614	0.0024235937579569	0.00886306794669505	0.00508142268957598	MAP1B/MAPT/NEFL/TRAK2/UCHL1	5	1.85185185185185
GO:0048641	BP	GO:0048641	regulation of skeletal muscle tissue development	5/666	27/18614	0.0024235937579569	0.00886306794669505	0.00508142268957598	BCL2/MEF2C/SHH/TWIST1/WNT3A	5	1.85185185185185
GO:1903523	BP	GO:1903523	negative regulation of blood circulation	5/666	27/18614	0.0024235937579569	0.00886306794669505	0.00508142268957598	ADRA1A/ATP1A2/FKBP1B/PIK3CG/SPX	5	1.85185185185185
GO:1905564	BP	GO:1905564	positive regulation of vascular endothelial cell proliferation	5/666	27/18614	0.0024235937579569	0.00886306794669505	0.00508142268957598	AKT3/APLN/FGFR1/ITGA4/PLCG1	5	1.85185185185185
GO:2000108	BP	GO:2000108	positive regulation of leukocyte apoptotic process	5/666	27/18614	0.0024235937579569	0.00886306794669505	0.00508142268957598	CCL5/MEF2C/P2RX7/PIK3CD/WNT5A	5	1.85185185185185
GO:0003016	BP	GO:0003016	respiratory system process	6/666	39/18614	0.00244405393649472	0.00892461002813636	0.0051167063329916	ATP1A2/ECEL1/NDN/NLGN3/PHOX2B/TLX3	6	1.53846153846154
GO:0031111	BP	GO:0031111	negative regulation of microtubule polymerization or depolymerization	6/666	39/18614	0.00244405393649472	0.00892461002813636	0.0051167063329916	APC/FGF13/MAP1B/MAP2/SNCA/TUBB4A	6	1.53846153846154
GO:0045229	BP	GO:0045229	external encapsulating structure organization	22/666	317/18614	0.0024524404008831	0.00894858542712726	0.00513045204013914	AGT/AXIN2/CAV1/COL3A1/COL4A6/CTSG/FLRT2/GPM6B/ITGB3/MMP1/MMP11/MMP13/MMP7/MMP9/NDNF/PDPN/PRSS1/PRSS2/SOX9/SULF1/TNR/WNT3A	22	0.694006309148265
GO:0043409	BP	GO:0043409	negative regulation of MAPK cascade	15/666	183/18614	0.00246620084743019	0.0089921195586939	0.00515541126703937	AGT/CAV1/DUSP1/EPHA4/EPHB2/F2RL1/GPER1/P2RX7/PIK3R2/PINK1/PRKN/RGS2/RPS6KA6/SEMA6A/UCHL1	15	0.819672131147541
GO:0030111	BP	GO:0030111	regulation of Wnt signaling pathway	23/666	338/18614	0.00254009301417432	0.00925467543711399	0.00530594124220253	APC/ASPM/ATP6V1C2/AXIN2/CAV1/CSNK1A1L/CSNK1E/DCDC2/DKK1/DKK4/FZD9/GNAQ/LRRK2/PRKN/SHH/SOX10/SOX2/SOX9/SULF1/WNT11/WNT3/WNT3A/WNT5A	23	0.680473372781065
GO:0031349	BP	GO:0031349	positive regulation of defense response	28/666	441/18614	0.00254315126990685	0.00925895443821641	0.00530839450256579	AGT/AGTR1/CAV1/CCL5/CCR2/CCR7/CNR1/CREB3L3/F2RL1/GRP/GRPR/IL6ST/IRAK1/IRAK2/LRRK2/MAP2K6/MEF2C/NPY5R/PENK/PIK3CG/PLCG2/PTGER3/PTGER4/RIPK2/SNCA/TYRO3/UCN/WNT5A	28	0.634920634920635
GO:0042509	BP	GO:0042509	regulation of tyrosine phosphorylation of STAT protein	9/666	82/18614	0.00260150611507806	0.00946439863479545	0.00542618089529312	CAV1/CCL5/CRLF1/ERBB4/GHR/IGF1/IL6R/IL6ST/LEP	9	1.09756097560976

	ONTOLOGY	ID	Description	GeneRatio	BgRatio	pvalue	p.adjust	qvalue	geneID	Count	richFactor
GO:0031589	BP	GO:0031589	cell-substrate adhesion	24/666	359/18614	0.00260756631185484	0.00947942930677997	0.00543479836253145	AXL/BCL2/CCR7/COL3A1/CSF1/EFNA5/EPHA3/EPHB3/GPM6B/ITGA4/ITGB3/KIF14/L1CAM/NDNF/NEXMIF/NPY/PDPN/PTK2B/SEMA3E/TYRO3/VCL/VEGFA/VWC2/WNT1	24	0.668523676880223
GO:0003205	BP	GO:0003205	cardiac chamber development	14/666	166/18614	0.00261092507129372	0.00948431685589154	0.00543760051897449	EDNRA/MEF2C/NPY2R/NPY5R/NRP2/POU4F1/PROX1/ROBO2/SLIT2/SLIT3/TBX20/WNT11/WNT2/WNT5A	14	0.843373493975904
GO:0042531	BP	GO:0042531	positive regulation of tyrosine phosphorylation of STAT protein	8/666	67/18614	0.00261277009621102	0.00948431685589154	0.00543760051897449	CCL5/CRLF1/ERBB4/GHR/IGF1/IL6R/IL6ST/LEP	8	1.19402985074627
GO:0055119	BP	GO:0055119	relaxation of cardiac muscle	4/666	17/18614	0.00266399108999804	0.00964886971801058	0.00553194286772272	ATP1A2/CAMK2D/P2RX4/RGS2	4	2.35294117647059
GO:0070206	BP	GO:0070206	protein trimerization	4/666	17/18614	0.00266399108999804	0.00964886971801058	0.00553194286772272	ASIC1/MIF/SIGMAR1/SLC1A2	4	2.35294117647059
GO:1902306	BP	GO:1902306	negative regulation of sodium ion transmembrane transport	4/666	17/18614	0.00266399108999804	0.00964886971801058	0.00553194286772272	ATP1A2/CAMK2D/GRP/NEDD4L	4	2.35294117647059
GO:0060828	BP	GO:0060828	regulation of canonical Wnt signaling pathway	19/666	260/18614	0.00266829086216126	0.00965732664766024	0.00553679144097798	APC/ASPM/AXIN2/CAV1/CSNK1A1L/CSNK1E/DKK1/DKK4/FZD9/GNAQ/LRRK2/PRKN/SHH/SOX10/SOX2/SOX9/WNT11/WNT3A/WNT5A	19	0.730769230769231
GO:0010823	BP	GO:0010823	negative regulation of mitochondrion organization	7/666	53/18614	0.00268216715395711	0.00970040585849829	0.00556148985023289	BCL2L1/CLU/FZD9/IGF1/MAPT/PINK1/PRKN	7	1.32075471698113
GO:0010810	BP	GO:0010810	regulation of cell-substrate adhesion	17/666	222/18614	0.00270657739218515	0.00978149109013972	0.00560797807962821	BCL2/CCR7/CSF1/EFNA5/EPHA3/GPM6B/ITGB3/NDNF/NEXMIF/NPY/PDPN/PTK2B/SEMA3E/VCL/VEGFA/VWC2/WNT1	17	0.765765765765766
GO:0031668	BP	GO:0031668	cellular response to extracellular stimulus	19/666	261/18614	0.00278454570440863	0.0100558722536138	0.00576528779202745	AXL/BCL2/BECN2/CARTPT/COMT/FAS/GABARAP/GCGR/ITGA4/LEP/LRRK2/MAP1LC3C/P2RX7/PENK/SLC1A2/SLC38A3/UCN2/UCN3/WNT2B	19	0.727969348659004
GO:0043281	BP	GO:0043281	regulation of cysteine-type endopeptidase activity involved in apoptotic process	16/666	204/18614	0.00280940372926157	0.0101381933401767	0.00581248456853947	ATP2A3/BID/FAS/GPER1/MAGEA3/MAPT/MMP9/NDUFA13/NGFR/P2RX1/SLC1A1/SLC22A2/SNCA/SOX2/VEGFA/WNT3A	16	0.784313725490196
GO:0032024	BP	GO:0032024	positive regulation of insulin secretion	9/666	83/18614	0.00282755839700269	0.0101962212188322	0.00584575342995421	F2/F2RL2/GCG/GNA11/GPER1/ITPR1/NKX6-1/PRKCB/UCN3	9	1.08433734939759
GO:0097193	BP	GO:0097193	intrinsic apoptotic signaling pathway	21/666	301/18614	0.00285806833774681	0.0102778322504846	0.00589254311389354	ATP2A1/ATP2A3/BCL2/BCL2L1/BDKRB2/BID/CAV1/CLU/CXCL12/DDIT4/ITPR1/LRRK2/MAGEA3/MIF/MMP9/NDUFA13/PINK1/POU4F1/PRKN/TP73/TRAP1	21	0.697674418604651
GO:0003171	BP	GO:0003171	atrioventricular valve development	5/666	28/18614	0.00286482811458067	0.0102778322504846	0.00589254311389354	AXIN2/OLFM1/SLIT3/TBX20/TWIST1	5	1.78571428571429
GO:0043949	BP	GO:0043949	regulation of cAMP-mediated signaling	5/666	28/18614	0.00286482811458067	0.0102778322504846	0.00589254311389354	ADCYAP1R1/GNAI1/LPAR1/SCTR/UCN	5	1.78571428571429
GO:0048679	BP	GO:0048679	regulation of axon regeneration	5/666	28/18614	0.00286482811458067	0.0102778322504846	0.00589254311389354	EPHA4/FKBP1B/PTN/RTN4R/TNR	5	1.78571428571429
GO:0050951	BP	GO:0050951	sensory perception of temperature stimulus	5/666	28/18614	0.00286482811458067	0.0102778322504846	0.00589254311389354	ADRA2A/CALCA/CXCL12/GRIK2/NTSR1	5	1.78571428571429
GO:0060479	BP	GO:0060479	lung cell differentiation	5/666	28/18614	0.00286482811458067	0.0102778322504846	0.00589254311389354	ASCL1/NKX2-1/SOX9/THRB/TP73	5	1.78571428571429

	ONTOLOGY	ID	Description	GeneRatio	BgRatio	pvalue	p.adjust	qvalue	geneID	Count	richFactor
GO:0060487	BP	GO:0060487	lung epithelial cell differentiation	5/666	28/18614	0.00286482811458067	0.0102778322504846	0.00589254311389354	ASCL1/NKX2-1/SOX9/THRB/TP73	5	1.78571428571429
GO:0055021	BP	GO:0055021	regulation of cardiac muscle tissue growth	8/666	68/18614	0.0028704083129145	0.0102828402754918	0.00589541434223732	CDK1/ERBB4/IGF1/MEF2C/RGS2/TBX20/TP73/WNT2	8	1.17647058823529
GO:2000401	BP	GO:2000401	regulation of lymphocyte migration	8/666	68/18614	0.0028704083129145	0.0102828402754918	0.00589541434223732	CCL5/CCR2/CXCL12/CXCL13/ITGA4/ITGB3/PTK2B/WNT5A	8	1.17647058823529
GO:0046661	BP	GO:0046661	male sex differentiation	14/666	168/18614	0.00291360186191959	0.0104299731619336	0.00597976937505942	ADGRG1/ASPM/BCL2/BCL2L1/CCND1/GFRA1/IL1A/INHBA/LHB/NKX2-1/SHH/SOX9/WNT2B/WNT5A	14	0.833333333333333
GO:0022411	BP	GO:0022411	cellular component disassembly	30/666	488/18614	0.00294074199938553	0.0105194664679621	0.0060310781677431	ADRB2/APC/AURKA/CDK1/CTSG/CX3CR1/DDIT4/DKK1/EPHA4/F2RL1/FGF13/GABARAP/GPER1/MAP1B/MAP1LC3C/MMP1/MMP11/MMP13/MMP7/MMP9/PDPN/PINK1/PRKCB/PRKN/PRSS1/PRSS2/SH3GL2/SPTBN2/TMOD2/TOP2A	30	0.614754098360656
GO:0071222	BP	GO:0071222	cellular response to lipopolysaccharide	17/666	224/18614	0.00296944103371471	0.0106144019496057	0.00608550709837649	AXL/CAPN2/CCL5/CTSG/CX3CR1/CXCL13/CXCL8/EDNRB/EPHB2/IL1A/IRAK1/IRAK2/MEF2C/MIF/PLCG2/RIPK2/WNT5A	17	0.758928571428571
GO:0007632	BP	GO:0007632	visual behavior	7/666	54/18614	0.00298847294143322	0.0106591759848652	0.00611117719365727	ATP1A2/CHRNB2/DRD2/GRIN2A/SLC1A2/SLC7A11/TUBA1A	7	1.2962962962963
GO:0009395	BP	GO:0009395	phospholipid catabolic process	7/666	54/18614	0.00298847294143322	0.0106591759848652	0.00611117719365727	APOC1/PLA2G4B/PLA2G4D/PLA2G4E/PLCG1/PLCG2/PLD1	7	1.2962962962963
GO:1903170	BP	GO:1903170	negative regulation of calcium ion transmembrane transport	7/666	54/18614	0.00298847294143322	0.0106591759848652	0.00611117719365727	ADRA2A/ATP1A2/BCL2/DRD4/FKBP1B/NOS1/NTSR1	7	1.2962962962963
GO:0001819	BP	GO:0001819	positive regulation of cytokine production	30/666	489/18614	0.00302992230770662	0.0107991792185482	0.00619144461488539	ADCYAP1/ADRA2A/AGT/CCR2/CCR7/CLU/DRD2/EPHB2/F2RL1/HTR2B/IL1A/IL6R/IL6ST/IRAK1/LEP/LRRK2/MIF/NMB/P2RX7/PIK3CD/PIK3CG/PLCG2/PTGER4/RIPK2/SULF1/TWIST1/UCN/WNT11/WNT3A/WNT5A	30	0.613496932515337
GO:0043542	BP	GO:0043542	endothelial cell migration	20/666	283/18614	0.00307391266626355	0.0109480295323807	0.00627678429259156	AGT/AKT3/CXCL13/EDN2/FGFR1/ITGB3/MEF2C/NRP2/P2RX4/PIK3CD/PIK3CG/PLCG1/PROX1/PTK2B/PTN/SCG2/SLIT2/VEGFA/WNT5A/WNT7A	20	0.706713780918728
GO:0032755	BP	GO:0032755	positive regulation of interleukin-6 production	10/666	100/18614	0.00307871254411815	0.0109571847605653	0.00628203321818993	F2RL1/IL1A/IL6R/LEP/P2RX7/PLCG2/RIPK2/TWIST1/UCN/WNT5A	10	1
GO:0014015	BP	GO:0014015	positive regulation of gliogenesis	8/666	69/18614	0.00314742178635727	0.0111774408092095	0.00640831162312017	IL6ST/MAG/NKX6-1/NTN1/PTN/SHH/SOX10/TP73	8	1.15942028985507
GO:0045576	BP	GO:0045576	mast cell activation	8/666	69/18614	0.00314742178635727	0.0111774408092095	0.00640831162312017	CNR1/CNR2/GRP/PIK3CD/PIK3CG/PTGDR/SLC18A2/STXBP1	8	1.15942028985507
GO:1901016	BP	GO:1901016	regulation of potassium ion transmembrane transporter activity	8/666	69/18614	0.00314742178635727	0.0111774408092095	0.00640831162312017	ANK3/CACNA1D/CAV1/GAL/GRP/KCNA1/NEDD4L/VAMP2	8	1.15942028985507
GO:0019216	BP	GO:0019216	regulation of lipid metabolic process	23/666	344/18614	0.00316297327594458	0.011224558592973	0.00643532544913825	ADRA2A/APOC1/CAPN2/CAV1/CCR7/CGA/CHRM5/CNR1/F2/GAL/GPER1/HTR2B/HTR2C/IL1A/LEP/NR3C1/PIK3CG/PLCG2/PROX1/PTK2B/SNCA/SOX9/TWIST1	23	0.668604651162791

	ONTOLOGY	ID	Description	GeneRatio	BgRatio	pvalue	p.adjust	qvalue	geneID	Count	richFactor
GO:0048246	BP	GO:0048246	macrophage chemotaxis	6/666	41/18614	0.00317107337236084	0.0112447520226398	0.00644690285690565	CCL5/CSF1/EDN2/EDNRB/MIF/PTK2B	6	1.46341463414634
GO:0019751	BP	GO:0019751	polyol metabolic process	11/666	117/18614	0.00317323927882022	0.0112447520226398	0.00644690285690565	ADCYAP1R1/AVPR1B/GPER1/LEP/NTSR1/P2RY1/PLCG2/PRKG2/PTH1R/PTK2B/SNCA	11	0.94017094017094
GO:0048872	BP	GO:0048872	homeostasis of number of cells	21/666	304/18614	0.00321058436649279	0.0113688920470548	0.00651807549605733	ADGRG1/AKT3/AXL/BCL2/CCR7/CSF1/CX3CR1/EMX1/EPAS1/FAS/INHBA/KCNQ1/MEF2C/MIF/P2RX7/PIK3CD/SLC7A11/SOS2/SOX9/TUBA1A/VEGFA	21	0.690789473684211
GO:0016331	BP	GO:0016331	morphogenesis of embryonic epithelium	13/666	152/18614	0.00323918610161379	0.011461914823205	0.0065714078238946	CELSR1/FZD3/GDNF/PRKACB/SHH/SOX9/SULF1/TWIST1/WNT16/WNT2/WNT2B/WNT5A/WNT7B	13	0.855263157894737
GO:1903034	BP	GO:1903034	regulation of response to wounding	14/666	170/18614	0.00324448417056722	0.0114709481437471	0.00657658685673521	ADRA2A/CAV1/CLDN1/CLDN19/EPHA4/EPHB2/F2/F2RL1/FKBP1B/KNG1/PRKG1/PTN/RTN4R/TNR	14	0.823529411764706
GO:0042594	BP	GO:0042594	response to starvation	16/666	207/18614	0.00324640668727409	0.0114709481437471	0.00657658685673521	BCL2/BECN2/CARTPT/COMT/FAS/GABARAP/GCGR/HCRT/LRRK2/MAP1LC3C/SLC38A3/SSTR1/SSTR2/SSTR3/UCN3/WNT2B	16	0.772946859903382
GO:0070167	BP	GO:0070167	regulation of biomineral tissue development	10/666	101/18614	0.00331042739326803	0.0116712331516416	0.00669141535513083	ADRB2/FZD9/GPM6B/MEF2C/P2RX7/PTK2B/PTN/S1PR1/SOX9/TWIST1	10	0.99009900990099
GO:0010663	BP	GO:0010663	positive regulation of striated muscle cell apoptotic process	4/666	18/18614	0.0033292103516992	0.0116712331516416	0.00669141535513083	AGT/CAMK2A/CAMK2D/CAPN2	4	2.22222222222222
GO:0010666	BP	GO:0010666	positive regulation of cardiac muscle cell apoptotic process	4/666	18/18614	0.0033292103516992	0.0116712331516416	0.00669141535513083	AGT/CAMK2A/CAMK2D/CAPN2	4	2.22222222222222
GO:0010958	BP	GO:0010958	regulation of amino acid import across plasma membrane	4/666	18/18614	0.0033292103516992	0.0116712331516416	0.00669141535513083	AGT/ATP1A2/RGS2/SLC17A8	4	2.22222222222222
GO:0015812	BP	GO:0015812	gamma-aminobutyric acid transport	4/666	18/18614	0.0033292103516992	0.0116712331516416	0.00669141535513083	NTSR1/P2RX7/SLC6A1/SLC6A6	4	2.22222222222222
GO:0019896	BP	GO:0019896	axonal transport of mitochondrion	4/666	18/18614	0.0033292103516992	0.0116712331516416	0.00669141535513083	MAPT/NEFL/TRAK2/UCHL1	4	2.22222222222222
GO:0032769	BP	GO:0032769	negative regulation of monooxygenase activity	4/666	18/18614	0.0033292103516992	0.0116712331516416	0.00669141535513083	CAV1/CNR1/CNR2/SNCA	4	2.22222222222222
GO:0042402	BP	GO:0042402	cellular biogenic amine catabolic process	4/666	18/18614	0.0033292103516992	0.0116712331516416	0.00669141535513083	COMT/MAOA/MAOB/SLC6A3	4	2.22222222222222
GO:0043117	BP	GO:0043117	positive regulation of vascular permeability	4/666	18/18614	0.0033292103516992	0.0116712331516416	0.00669141535513083	TACR1/TACR2/UCN/VEGFA	4	2.22222222222222
GO:1903789	BP	GO:1903789	regulation of amino acid transmembrane transport	4/666	18/18614	0.0033292103516992	0.0116712331516416	0.00669141535513083	AGT/ATP1A2/RGS2/SLC17A8	4	2.22222222222222
GO:2001224	BP	GO:2001224	positive regulation of neuron migration	4/666	18/18614	0.0033292103516992	0.0116712331516416	0.00669141535513083	NSMF/RELN/SEMA6A/WDR62	4	2.22222222222222
GO:1904385	BP	GO:1904385	cellular response to angiotensin	5/666	29/18614	0.00336129392125744	0.0117753097811691	0.00675108513876742	AGT/AGTR1/CAMK2A/CAV1/INHBA	5	1.72413793103448
GO:0014823	BP	GO:0014823	response to activity	8/666	70/18614	0.00344477248514878	0.0120591572396768	0.0069138221193157	AGT/CDK1/GCG/ITGB3/LEP/OXT/PHOX2B/TH	8	1.14285714285714
GO:0046620	BP	GO:0046620	regulation of organ growth	10/666	102/18614	0.00355579606380757	0.012438959184067	0.00713157225159233	CDK1/CGA/ERBB4/IGF1/MEF2C/PROX1/RGS2/TBX20/TP73/WNT2	10	0.980392156862745
GO:0021983	BP	GO:0021983	pituitary gland development	6/666	42/18614	0.00358977909816741	0.0125399888184028	0.00718949511524643	DRD2/GHRHR/NKX2-1/SLC6A3/SOX2/WNT5A	6	1.42857142857143

	ONTOLOGY	ID	Description	GeneRatio	BgRatio	pvalue	p.adjust	qvalue	geneID	Count	richFactor
GO:0070296	BP	GO:0070296	sarcoplasmic reticulum calcium ion transport	6/666	42/18614	0.00358977909816741	0.0125399888184028	0.00718949511524643	ATP1A2/ATP2A1/CACNA1C/CAMK2D/FKBP1B/RYR1	6	1.42857142857143
GO:0007260	BP	GO:0007260	tyrosine phosphorylation of STAT protein	9/666	86/18614	0.00359998831362451	0.0125578016759861	0.00719970768038012	CAV1/CCL5/CRLF1/ERBB4/GHR/IGF1/IL6R/IL6ST/LEP	9	1.04651162790698
GO:0034103	BP	GO:0034103	regulation of tissue remodeling	9/666	86/18614	0.00359998831362451	0.0125578016759861	0.00719970768038012	AGT/CALCA/CARTPT/ITGB3/LEP/LEPR/P2RX7/S1PR1/SYT7	9	1.04651162790698
GO:2000107	BP	GO:2000107	negative regulation of leukocyte apoptotic process	7/666	56/18614	0.00368064071334211	0.0128300348270046	0.00735578588250604	AXL/BCL2/BCL2L1/CCL5/CCR7/CXCL12/MIF	7	1.25
GO:1903828	BP	GO:1903828	negative regulation of protein localization	16/666	210/18614	0.00373804059135531	0.0130208855467834	0.00746520546313415	ADRA2A/BCL2L1/DCLK1/DRD2/DRD4/F2RL1/FKBP1B/FZD9/GNAZ/GPM6B/LRRK2/MAPT/NEDD4L/OPRM1/PRKN/SYT4	16	0.761904761904762
GO:0001776	BP	GO:0001776	leukocyte homeostasis	10/666	103/18614	0.00381537363748118	0.0132808508698442	0.00761425020690109	AXL/BCL2/CSF1/FAS/MEF2C/MIF/P2RX7/PIK3CD/SLC7A11/SOS2	10	0.970873786407767
GO:0006906	BP	GO:0006906	vesicle fusion	11/666	120/18614	0.00385753447320421	0.0134086152304093	0.00768750077031764	CPLX1/GRIK5/SNAP25/SNCA/STX1A/STXBP1/SYT11/SYT4/SYT7/SYT9/VAMP2	11	0.916666666666667
GO:0043200	BP	GO:0043200	response to amino acid	11/666	120/18614	0.00385753447320421	0.0134086152304093	0.00768750077031764	BCL2/BCL2L1/CAPN2/COL3A1/COL4A6/GLRA3/HPCA/NSMF/SLC1A2/SST/TUBA1A	11	0.916666666666667
GO:0060045	BP	GO:0060045	positive regulation of cardiac muscle cell proliferation	5/666	30/18614	0.00391671160314576	0.0135950830010321	0.00779440749451472	CDK1/ERBB4/MEF2C/TBX20/WNT2	5	1.66666666666667
GO:0090140	BP	GO:0090140	regulation of mitochondrial fission	5/666	30/18614	0.00391671160314576	0.0135950830010321	0.00779440749451472	AURKA/LRRK2/MAPT/PINK1/PRKN	5	1.66666666666667
GO:0030178	BP	GO:0030178	negative regulation of Wnt signaling pathway	14/666	174/18614	0.00399858521991388	0.0138694751982193	0.00795172353282282	APC/AXIN2/CAV1/CSNK1A1L/CSNK1E/DKK1/DKK4/PRKN/SHH/SOX10/SOX2/SOX9/WNT11/WNT5A	14	0.804597701149425
GO:0048284	BP	GO:0048284	organelle fusion	13/666	156/18614	0.0040466663685701	0.01399211397173	0.00802203546657842	CDK1/CPLX1/GRIK5/PRKN/SNAP25/SNCA/STX1A/STXBP1/SYT11/SYT4/SYT7/SYT9/VAMP2	13	0.833333333333333
GO:0097530	BP	GO:0097530	granulocyte migration	13/666	156/18614	0.0040466663685701	0.01399211397173	0.00802203546657842	CCL5/CCR7/CSF1/CXCL13/CXCL8/EDN2/EDN3/IL1A/PIK3CD/PIK3CG/PTGER4/SCG2/SLIT2	13	0.833333333333333
GO:0030501	BP	GO:0030501	positive regulation of bone mineralization	6/666	43/18614	0.00404817620911497	0.01399211397173	0.00802203546657842	ADRB2/FZD9/GPM6B/MEF2C/P2RX7/PTN	6	1.3953488372093
GO:0045687	BP	GO:0045687	positive regulation of glial cell differentiation	6/666	43/18614	0.00404817620911497	0.01399211397173	0.00802203546657842	IL6ST/MAG/NKX6-1/PTN/SHH/TP73	6	1.3953488372093
GO:0060412	BP	GO:0060412	ventricular septum morphogenesis	6/666	43/18614	0.00404817620911497	0.01399211397173	0.00802203546657842	PROX1/ROBO2/SLIT2/SLIT3/WNT11/WNT5A	6	1.3953488372093
GO:0043388	BP	GO:0043388	positive regulation of DNA binding	7/666	57/18614	0.00406931672243695	0.0140355731163352	0.00804695170154291	IGF1/MMP9/NEUROD1/NGF/POU4F1/PRKN/TWIST1	7	1.2280701754386
GO:0046148	BP	GO:0046148	pigment biosynthetic process	7/666	57/18614	0.00406931672243695	0.0140355731163352	0.00804695170154291	ASIP/GPR143/MC1R/SLC6A9/SLC7A11/TRPC1/WNT5A	7	1.2280701754386
GO:0048016	BP	GO:0048016	inositol phosphate-mediated signaling	7/666	57/18614	0.00406931672243695	0.0140355731163352	0.00804695170154291	EDN2/EDN3/IGF1/ITPR1/NMUR2/PLCG2/PRNP	7	1.2280701754386
GO:0032872	BP	GO:0032872	regulation of stress-activated MAPK cascade	15/666	193/18614	0.00408227946051172	0.0140481881840743	0.00805418424131645	CCR7/DUSP1/F2RL1/FAS/IL1A/LEP/PINK1/PRKN/PTK2B/RIPK2/VEGFA/WNT16/WNT5A/WNT7A/WNT7B	15	0.77720207253886

	ONTOLOGY	ID	Description	GeneRatio	BgRatio	pvalue	p.adjust	qvalue	geneID	Count	richFactor
GO:0030705	BP	GO:0030705	cytoskeleton-dependent intracellular transport	16/666	212/18614	0.00409859279558697	0.0140481881840743	0.00805418424131645	CALY/DLG2/KIF1A/KIF5A/KIF5C/KLC3/MAP1B/MAP2/MAPT/NEFH/NEFL/SYT4/TMEM108/TRAK2/TUBA1A/UCHL1	16	0.754716981132075
GO:0099111	BP	GO:0099111	microtubule-based transport	16/666	212/18614	0.00409859279558697	0.0140481881840743	0.00805418424131645	CALY/DLG2/DNAH5/KIF1A/KIF5A/KIF5C/KLC3/MAP1B/MAP2/MAPT/NEFH/NEFL/TMEM108/TRAK2/TUBA1A/UCHL1	16	0.754716981132075
GO:0010738	BP	GO:0010738	regulation of protein kinase A signaling	4/666	19/18614	0.00409905605438894	0.0140481881840743	0.00805418424131645	ADRB2/LRRK2/MC1R/MIF	4	2.10526315789474
GO:0010766	BP	GO:0010766	negative regulation of sodium ion transport	4/666	19/18614	0.00409905605438894	0.0140481881840743	0.00805418424131645	ATP1A2/CAMK2D/GRP/NEDD4L	4	2.10526315789474
GO:0030540	BP	GO:0030540	female genitalia development	4/666	19/18614	0.00409905605438894	0.0140481881840743	0.00805418424131645	AXL/LHX1/TYRO3/WNT5A	4	2.10526315789474
GO:0048266	BP	GO:0048266	behavioral response to pain	4/666	19/18614	0.00409905605438894	0.0140481881840743	0.00805418424131645	CAPN2/P2RX2/P2RX4/TACR1	4	2.10526315789474
GO:0051797	BP	GO:0051797	regulation of hair follicle development	4/666	19/18614	0.00409905605438894	0.0140481881840743	0.00805418424131645	DKK4/GAL/NGFR/WNT5A	4	2.10526315789474
GO:0051900	BP	GO:0051900	regulation of mitochondrial depolarization	4/666	19/18614	0.00409905605438894	0.0140481881840743	0.00805418424131645	BCL2/FZD9/LRRK2/P2RX7	4	2.10526315789474
GO:0043954	BP	GO:0043954	cellular component maintenance	8/666	72/18614	0.00410441469630329	0.0140481881840743	0.00805418424131645	CLDN1/CNTNAP1/F2RL1/GRIN2B/HOMER1/ITGB3/NLGN1/PRNP	8	1.11111111111111
GO:0060411	BP	GO:0060411	cardiac septum morphogenesis	8/666	72/18614	0.00410441469630329	0.0140481881840743	0.00805418424131645	NRP2/PROX1/ROBO2/SLIT2/SLIT3/TBX20/WNT11/WNT5A	8	1.11111111111111
GO:0072089	BP	GO:0072089	stem cell proliferation	11/666	121/18614	0.00411013332239218	0.0140481956046993	0.00805418849574985	AXIN2/BYSL/HMGA2/SHH/SOX9/WNT1/WNT11/WNT2B/WNT3/WNT5A/WNT7B	11	0.909090909090909
GO:0098773	BP	GO:0098773	skin epidermis development	11/666	121/18614	0.00411013332239218	0.0140481956046993	0.00805418849574985	BCL2/CLDN1/DKK1/DKK4/FZD3/GAL/INHBA/NGFR/SHH/SOX9/WNT5A	11	0.909090909090909
GO:0090174	BP	GO:0090174	organelle membrane fusion	11/666	122/18614	0.00437573606123694	0.0149456169152047	0.00856870299984001	CPLX1/GRIK5/SNAP25/SNCA/STX1A/STXBP1/SYT11/SYT4/SYT7/SYT9/VAMP2	11	0.901639344262295
GO:0060415	BP	GO:0060415	muscle tissue morphogenesis	8/666	73/18614	0.00446870768014899	0.0152314134867769	0.00873255745657263	COL3A1/EDNRA/POU4F1/PROX1/S1PR1/TBX20/WNT2/WNT5A	8	1.0958904109589
GO:0061035	BP	GO:0061035	regulation of cartilage development	8/666	73/18614	0.00446870768014899	0.0152314134867769	0.00873255745657263	AXIN2/LEP/PRKG2/SOX5/SOX9/WNT11/WNT5A/WNT9A	8	1.0958904109589
GO:0071260	BP	GO:0071260	cellular response to mechanical stimulus	8/666	73/18614	0.00446870768014899	0.0152314134867769	0.00873255745657263	AGT/ATP1A2/FAS/ITGB3/MAG/PTGER4/SOX9/WNT11	8	1.0958904109589
GO:0120032	BP	GO:0120032	regulation of plasma membrane bounded cell projection assembly	15/666	195/18614	0.0044914365968664	0.0152451858661121	0.00874045351257314	APC/ARHGAP44/CAV1/CCR7/DCDC2/EPHB2/F2RL1/GAP43/GPM6A/NLGN1/P2RX7/PLD1/SLIT2/TENM1/WNT1	15	0.769230769230769
GO:0006868	BP	GO:0006868	glutamine transport	3/666	10/18614	0.00453230280064933	0.0152451858661121	0.00874045351257314	LEP/SLC38A3/SLC38A5	3	3
GO:0014824	BP	GO:0014824	artery smooth muscle contraction	3/666	10/18614	0.00453230280064933	0.0152451858661121	0.00874045351257314	AGT/EDN2/EDNRA	3	3
GO:0021561	BP	GO:0021561	facial nerve development	3/666	10/18614	0.00453230280064933	0.0152451858661121	0.00874045351257314	NRP2/PLXNA3/SEMA3F	3	3
GO:0021610	BP	GO:0021610	facial nerve morphogenesis	3/666	10/18614	0.00453230280064933	0.0152451858661121	0.00874045351257314	NRP2/PLXNA3/SEMA3F	3	3
GO:0033197	BP	GO:0033197	response to vitamin E	3/666	10/18614	0.00453230280064933	0.0152451858661121	0.00874045351257314	CCND1/FKBP1B/LEP	3	3
GO:0035747	BP	GO:0035747	natural killer cell chemotaxis	3/666	10/18614	0.00453230280064933	0.0152451858661121	0.00874045351257314	CCL5/PIK3CD/PIK3CG	3	3
GO:0044557	BP	GO:0044557	relaxation of smooth muscle	3/666	10/18614	0.00453230280064933	0.0152451858661121	0.00874045351257314	GUCY1A1/PRKG1/RGS2	3	3
GO:0048340	BP	GO:0048340	paraxial mesoderm morphogenesis	3/666	10/18614	0.00453230280064933	0.0152451858661121	0.00874045351257314	WNT11/WNT3A/WNT5A	3	3

	ONTOLOGY	ID	Description	GeneRatio	BgRatio	pvalue	p.adjust	qvalue	geneID	Count	richFactor
GO:0051933	BP	GO:0051933	amino acid neurotransmitter reuptake	3/666	10/18614	0.00453230280064933	0.0152451858661121	0.00874045351257314	ATP1A2/SLC17A8/SLC6A1	3	3
GO:0070099	BP	GO:0070099	regulation of chemokine-mediated signaling pathway	3/666	10/18614	0.00453230280064933	0.0152451858661121	0.00874045351257314	CCL5/SLIT2/SLIT3	3	3
GO:0070294	BP	GO:0070294	renal sodium ion absorption	3/666	10/18614	0.00453230280064933	0.0152451858661121	0.00874045351257314	EDNRA/EDNRB/KCNQ1	3	3
GO:0071073	BP	GO:0071073	positive regulation of phospholipid biosynthetic process	3/666	10/18614	0.00453230280064933	0.0152451858661121	0.00874045351257314	CAPN2/HTR2B/HTR2C	3	3
GO:0071377	BP	GO:0071377	cellular response to glucagon stimulus	3/666	10/18614	0.00453230280064933	0.0152451858661121	0.00874045351257314	CYSLTR2/GCGR/GLP2R	3	3
GO:1905918	BP	GO:1905918	regulation of CoA-transferase activity	3/666	10/18614	0.00453230280064933	0.0152451858661121	0.00874045351257314	AGT/AGTR1/CHRNA7	3	3
GO:0010039	BP	GO:0010039	response to iron ion	5/666	31/18614	0.00453478366963498	0.0152451858661121	0.00874045351257314	BCL2/CCND1/DRD2/SLC6A3/SNCA	5	1.61290322580645
GO:0010464	BP	GO:0010464	regulation of mesenchymal cell proliferation	5/666	31/18614	0.00453478366963498	0.0152451858661121	0.00874045351257314	SHH/SOX9/WNT11/WNT2/WNT5A	5	1.61290322580645
GO:0051654	BP	GO:0051654	establishment of mitochondrion localization	5/666	31/18614	0.00453478366963498	0.0152451858661121	0.00874045351257314	MAP1B/MAPT/NEFL/TRAK2/UCHL1	5	1.61290322580645
GO:0070570	BP	GO:0070570	regulation of neuron projection regeneration	5/666	31/18614	0.00453478366963498	0.0152451858661121	0.00874045351257314	EPHA4/FKBP1B/PTN/RTN4R/TNR	5	1.61290322580645
GO:0098901	BP	GO:0098901	regulation of cardiac muscle cell action potential	5/666	31/18614	0.00453478366963498	0.0152451858661121	0.00874045351257314	CACNA1C/CAMK2D/CAV1/FGF13/HCN1	5	1.61290322580645
GO:1904407	BP	GO:1904407	positive regulation of nitric oxide metabolic process	6/666	44/18614	0.0045484540590307	0.0152702538935354	0.00875482565144175	AGT/CLU/HSP90AB1/NPY/P2RX4/PTK2B	6	1.36363636363636
GO:1905521	BP	GO:1905521	regulation of macrophage migration	6/666	44/18614	0.0045484540590307	0.0152702538935354	0.00875482565144175	CCL5/CSF1/CX3CR1/MIF/P2RX4/PTK2B	6	1.36363636363636
GO:0070302	BP	GO:0070302	regulation of stress-activated protein kinase signaling cascade	15/666	196/18614	0.00470818591348312	0.0157957227063273	0.00905609031104316	CCR7/DUSP1/F2RL1/FAS/IL1A/LEP/PINK1/PRKN/PTK2B/RIPK2/VEGFA/WNT16/WNT5A/WNT7A/WNT7B	15	0.76530612244898
GO:0045123	BP	GO:0045123	cellular extravasation	8/666	74/18614	0.00485733824533232	0.01628500509946	0.00933660837420451	CCR2/CX3CR1/CXCL12/ITGA4/LEP/PIK3CD/PIK3CG/PTGER4	8	1.08108108108108
GO:0032635	BP	GO:0032635	interleukin-6 production	14/666	178/18614	0.00488933917649641	0.0163699605262124	0.00938531548507162	ARRB1/CAPN2/F2RL1/IL1A/IL6R/LEP/NMB/P2RX7/PLCG2/RIPK2/SYT11/TWIST1/UCN/WNT5A	14	0.786516853932584
GO:0032675	BP	GO:0032675	regulation of interleukin-6 production	14/666	178/18614	0.00488933917649641	0.0163699605262124	0.00938531548507162	ARRB1/CAPN2/F2RL1/IL1A/IL6R/LEP/NMB/P2RX7/PLCG2/RIPK2/SYT11/TWIST1/UCN/WNT5A	14	0.786516853932584
GO:0052548	BP	GO:0052548	regulation of endopeptidase activity	22/666	336/18614	0.00491322902145435	0.0164387478832186	0.0094247530295821	A2M/ATP2A3/BID/EPHA4/FAS/GPER1/GRIN2A/GRIN2B/KNG1/MAGEA3/MAPT/MMP9/NDUFA13/NGFR/P2RX1/RIPK2/SLC1A1/SLC22A2/SNCA/SOX2/VEGFA/WNT3A	22	0.654761904761905
GO:0032388	BP	GO:0032388	positive regulation of intracellular transport	15/666	197/18614	0.0049333577416972	0.0164836528215104	0.00945049817490673	ANK3/ATP2A1/CALY/CDK1/HPCA/LEP/MAP2/PIK3R2/PINK1/PLA2G4E/PRKN/PRNP/SHH/TENM1/VAMP2	15	0.761421319796954
GO:0060491	BP	GO:0060491	regulation of cell projection assembly	15/666	197/18614	0.0049333577416972	0.0164836528215104	0.00945049817490673	APC/ARHGAP44/CAV1/CCR7/DCDC2/EPHB2/F2RL1/GAP43/GPM6A/NLGN1/P2RX7/PLD1/SLIT2/TENM1/WNT1	15	0.761421319796954
GO:0001780	BP	GO:0001780	neutrophil homeostasis	4/666	20/18614	0.0049807017143122	0.0165294726035412	0.00947676782345766	AXL/CSF1/PIK3CD/SLC7A11	4	2

	ONTOLOGY	ID	Description	GeneRatio	BgRatio	pvalue	p.adjust	qvalue	geneID	Count	richFactor
GO:0009310	BP	GO:0009310	amine catabolic process	4/666	20/18614	0.0049807017143122	0.0165294726035412	0.00947676782345766	COMT/MAOA/MAOB/SLC6A3	4	2
GO:0021511	BP	GO:0021511	spinal cord patterning	4/666	20/18614	0.0049807017143122	0.0165294726035412	0.00947676782345766	ASCL1/RELN/SHH/SOX1	4	2
GO:0032793	BP	GO:0032793	positive regulation of CREB transcription factor activity	4/666	20/18614	0.0049807017143122	0.0165294726035412	0.00947676782345766	OPRD1/RELN/RPS6KA5/VEGFA	4	2
GO:0048643	BP	GO:0048643	positive regulation of skeletal muscle tissue development	4/666	20/18614	0.0049807017143122	0.0165294726035412	0.00947676782345766	BCL2/MEF2C/SHH/WNT3A	4	2
GO:0060749	BP	GO:0060749	mammary gland alveolus development	4/666	20/18614	0.0049807017143122	0.0165294726035412	0.00947676782345766	CCND1/ERBB4/TPH1/VEGFA	4	2
GO:0061377	BP	GO:0061377	mammary gland lobule development	4/666	20/18614	0.0049807017143122	0.0165294726035412	0.00947676782345766	CCND1/ERBB4/TPH1/VEGFA	4	2
GO:0071371	BP	GO:0071371	cellular response to gonadotropin stimulus	4/666	20/18614	0.0049807017143122	0.0165294726035412	0.00947676782345766	EDNRA/EFNA5/INHBA/NSMF	4	2
GO:1902004	BP	GO:1902004	positive regulation of amyloid-beta formation	4/666	20/18614	0.0049807017143122	0.0165294726035412	0.00947676782345766	CHRNA7/CLU/CSNK1E/EPHA4	4	2
GO:1902236	BP	GO:1902236	negative regulation of endoplasmic reticulum stress-induced intrinsic apoptotic signaling pathway	4/666	20/18614	0.0049807017143122	0.0165294726035412	0.00947676782345766	BCL2L1/LRRK2/MAGEA3/PRKN	4	2
GO:0009582	BP	GO:0009582	detection of abiotic stimulus	12/666	142/18614	0.00504102184886921	0.0167183686823159	0.00958506675859285	CALCA/CXCL12/GNA11/GNAQ/GNGT1/GPR88/GRIK2/KCNA1/KCNQ1/NGFR/NTSR1/TACR1	12	0.845070422535211
GO:0030574	BP	GO:0030574	collagen catabolic process	6/666	45/18614	0.0050928100741745	0.0168560010199109	0.009663974884682	MMP1/MMP11/MMP13/MMP7/MMP9/PRSS2	6	1.33333333333333
GO:0030890	BP	GO:0030890	positive regulation of B cell proliferation	6/666	45/18614	0.0050928100741745	0.0168560010199109	0.009663974884682	BCL2/CHRNB2/EPHB2/MEF2C/MIF/WNT3A	6	1.33333333333333
GO:0060443	BP	GO:0060443	mammary gland morphogenesis	6/666	45/18614	0.0050928100741745	0.0168560010199109	0.009663974884682	CAV1/CSF1/NR3C1/NRG3/NTN1/WNT5A	6	1.33333333333333
GO:0050954	BP	GO:0050954	sensory perception of mechanical stimulus	14/666	179/18614	0.00513536632235264	0.0169854141819403	0.00973817074799038	ATP6V0A4/CACNA1D/CHRNB2/CXCL12/DCDC2/KCNA1/KCNQ1/LHFPL4/P2RX2/P2RX4/SLC17A8/TH/THRB/UCN	14	0.782122905027933
GO:0003338	BP	GO:0003338	metanephros morphogenesis	5/666	32/18614	0.00521918003119248	0.0172047416856546	0.00986391679445252	CALB1/GDNF/LHX1/SOX9/WNT7B	5	1.5625
GO:0007628	BP	GO:0007628	adult walking behavior	5/666	32/18614	0.00521918003119248	0.0172047416856546	0.00986391679445252	CNTN2/DRD2/EPHA4/GLRB/UCHL1	5	1.5625
GO:0010765	BP	GO:0010765	positive regulation of sodium ion transport	5/666	32/18614	0.00521918003119248	0.0172047416856546	0.00986391679445252	ANK3/DRD4/FGF12/NOS1/SCN1B	5	1.5625
GO:0033032	BP	GO:0033032	regulation of myeloid cell apoptotic process	5/666	32/18614	0.00521918003119248	0.0172047416856546	0.00986391679445252	BCL2/MEF2C/MIF/PIK3CD/SLC7A11	5	1.5625
GO:0035115	BP	GO:0035115	embryonic forelimb morphogenesis	5/666	32/18614	0.00521918003119248	0.0172047416856546	0.00986391679445252	CACNA1C/SHH/TWIST1/WNT3/WNT7A	5	1.5625
GO:0071219	BP	GO:0071219	cellular response to molecule of bacterial origin	17/666	237/18614	0.00524373702998249	0.0172741069050697	0.00990368564220624	AXL/CAPN2/CCL5/CTSG/CX3CR1/CXCL13/CXCL8/EDNRB/EPHB2/IL1A/IRAK1/IRAK2/MEF2C/MIF/PLCG2/RIPK2/WNT5A	17	0.717299578059072
GO:2000242	BP	GO:2000242	negative regulation of reproductive process	8/666	75/18614	0.00527133814126448	0.0173534005119323	0.00994914668746433	BCL2L1/DUSP1/IGF1/IL1A/PTGDR2/SULF1/TTK/WNT5A	8	1.06666666666667
GO:1902600	BP	GO:1902600	proton transmembrane transport	12/666	143/18614	0.00532934281025841	0.017532610383146	0.010051892272995	ATP1A2/ATP6V0A4/ATP6V0D2/ATP6V1C2/ATP6V1E2/ATP6V1F/ATP6V1G2/COX7A1/DRD4/NDUFS7/SLC18A3/TWIST1	12	0.839160839160839

	ONTOLOGY	ID	Description	GeneRatio	BgRatio	pvalue	p.adjust	qvalue	geneID	Count	richFactor
GO:0032963	BP	GO:0032963	collagen metabolic process	10/666	108/18614	0.00534645954735533	0.017577156304516	0.0100774315847704	F2/INHBA/MMP1/MMP11/MMP13/MMP7/MMP9/P2RX7/PRSS2/UCN	10	0.925925925925926
GO:0019748	BP	GO:0019748	secondary metabolic process	7/666	60/18614	0.00542294376573341	0.0178047886496858	0.0102079390084508	ASIP/BCL2/MC1R/SLC7A11/TH/TRPC1/WNT5A	7	1.16666666666667
GO:0055008	BP	GO:0055008	cardiac muscle tissue morphogenesis	7/666	60/18614	0.00542294376573341	0.0178047886496858	0.0102079390084508	EDNRA/POU4F1/PROX1/S1PR1/TBX20/WNT2/WNT5A	7	1.16666666666667
GO:0009798	BP	GO:0009798	axis specification	9/666	92/18614	0.00563461573814937	0.0184874074452631	0.0105993017574491	AURKA/AXIN2/CHRDL1/LHX1/SHH/WNT1/WNT3/WNT5A/WNT7A	9	0.978260869565217
GO:0007094	BP	GO:0007094	mitotic spindle assembly checkpoint signaling	6/666	46/18614	0.00568344397258644	0.0185855802563289	0.0106555867315291	APC/BUB1B/CCNB1/DUSP1/MAD2L1/TTK	6	1.30434782608696
GO:0042551	BP	GO:0042551	neuron maturation	6/666	46/18614	0.00568344397258644	0.0185855802563289	0.0106555867315291	BCL2/CNTN2/EDNRA/EDNRB/LRRK2/NRCAM	6	1.30434782608696
GO:0071173	BP	GO:0071173	spindle assembly checkpoint signaling	6/666	46/18614	0.00568344397258644	0.0185855802563289	0.0106555867315291	APC/BUB1B/CCNB1/DUSP1/MAD2L1/TTK	6	1.30434782608696
GO:0071174	BP	GO:0071174	mitotic spindle checkpoint signaling	6/666	46/18614	0.00568344397258644	0.0185855802563289	0.0106555867315291	APC/BUB1B/CCNB1/DUSP1/MAD2L1/TTK	6	1.30434782608696
GO:2001239	BP	GO:2001239	regulation of extrinsic apoptotic signaling pathway in absence of ligand	6/666	46/18614	0.00568344397258644	0.0185855802563289	0.0106555867315291	BCL2/BCL2L1/FGFR1/GDNF/IL1A/INHBA	6	1.30434782608696
GO:0018107	BP	GO:0018107	peptidyl-threonine phosphorylation	10/666	109/18614	0.00570344592466666	0.018613835803278	0.0106717863565616	BCL2/CAMK2A/CAMK2D/CDK1/DDIT4/GCG/LRRK2/RIPK2/TTK/WNT5A	10	0.917431192660551
GO:0042116	BP	GO:0042116	macrophage activation	10/666	109/18614	0.00570344592466666	0.018613835803278	0.0106717863565616	CLU/CX3CR1/EDN2/LRRK2/MAPT/MIF/PLCG2/SNCA/SYT11/WNT5A	10	0.917431192660551
GO:0060840	BP	GO:0060840	artery development	10/666	109/18614	0.00570344592466666	0.018613835803278	0.0106717863565616	AKT3/COL3A1/EDNRA/LEP/NGFR/PROX1/ROBO2/SHH/VEGFA/WNT11	10	0.917431192660551
GO:0006919	BP	GO:0006919	activation of cysteine-type endopeptidase activity involved in apoptotic process	8/666	76/18614	0.00571174906389597	0.0186285644651949	0.0106802306737328	ATP2A3/BID/MAPT/NGFR/P2RX1/SLC1A1/SLC22A2/SNCA	8	1.05263157894737
GO:0051100	BP	GO:0051100	negative regulation of binding	13/666	163/18614	0.00584590426648576	0.0190534611868551	0.0109238347908575	ADRB2/ATP2A3/AURKA/CAV1/CPNE1/CSNK1E/DKK1/GZMA/HMGA2/ITGA4/LRRK2/MAP2/P2RY1	13	0.797546012269939
GO:0055007	BP	GO:0055007	cardiac muscle cell differentiation	11/666	127/18614	0.00591523526628143	0.0192666542967351	0.0110460638330267	ADRA1A/AGT/CDK1/DKK1/IGF1/MEF2C/NOX4/PROX1/RGS2/VEGFA/WNT3A	11	0.866141732283465
GO:0060042	BP	GO:0060042	retina morphogenesis in camera-type eye	7/666	61/18614	0.00594154371504763	0.0193395280526219	0.0110878442141391	CALB1/HCN1/LHX1/PROX1/SLC1A1/SOX9/THRB	7	1.14754098360656
GO:0022407	BP	GO:0022407	regulation of cell-cell adhesion	29/666	491/18614	0.0059523982253215	0.0193523253964907	0.0110951812574622	ANK3/CAV1/CCL5/CCR2/CCR7/CTSG/CXCL12/CXCL13/EFNA5/EPHB3/IGF1/IL1A/IL6ST/IRAK1/ITGA4/LEP/MDGA1/NEXMIF/PDPN/PRKG1/PRNP/RIPK2/SHH/SOX2/TNR/VEGFA/WNT1/WNT3A/WNT5A	29	0.590631364562118
GO:0007350	BP	GO:0007350	blastoderm segmentation	4/666	21/18614	0.00598100351778731	0.0193523253964907	0.0110951812574622	NRP2/SEMA3F/SHH/WNT5A	4	1.9047619047619
GO:0021756	BP	GO:0021756	striatum development	4/666	21/18614	0.00598100351778731	0.0193523253964907	0.0110951812574622	DRD2/INHBA/LRRK2/SLC7A11	4	1.9047619047619
GO:0051882	BP	GO:0051882	mitochondrial depolarization	4/666	21/18614	0.00598100351778731	0.0193523253964907	0.0110951812574622	BCL2/FZD9/LRRK2/P2RX7	4	1.9047619047619
GO:0090026	BP	GO:0090026	positive regulation of monocyte chemotaxis	4/666	21/18614	0.00598100351778731	0.0193523253964907	0.0110951812574622	CCL5/CCR2/CX3CR1/CXCL12	4	1.9047619047619

	ONTOLOGY	ID	Description	GeneRatio	BgRatio	pvalue	p.adjust	qvalue	geneID	Count	richFactor
GO:0090201	BP	GO:0090201	negative regulation of release of cytochrome c from mitochondria	4/666	21/18614	0.00598100351778731	0.0193523253964907	0.0110951812574622	BCL2L1/CLU/IGF1/PRKN	4	1.9047619047619
GO:1903204	BP	GO:1903204	negative regulation of oxidative stress-induced neuron death	4/666	21/18614	0.00598100351778731	0.0193523253964907	0.0110951812574622	PINK1/PRKN/SLC7A11/WNT1	4	1.9047619047619
GO:2000010	BP	GO:2000010	positive regulation of protein localization to cell surface	4/666	21/18614	0.00598100351778731	0.0193523253964907	0.0110951812574622	EPHB2/ERBB4/HSP90AB1/TMEM35A	4	1.9047619047619
GO:2000047	BP	GO:2000047	regulation of cell-cell adhesion mediated by cadherin	4/666	21/18614	0.00598100351778731	0.0193523253964907	0.0110951812574622	NEXMIF/VEGFA/WNT3A/WNT5A	4	1.9047619047619
GO:0003357	BP	GO:0003357	noradrenergic neuron differentiation	3/666	11/18614	0.00606753477843177	0.0193523253964907	0.0110951812574622	ASCL1/EDNRA/PHOX2B	3	2.72727272727273
GO:0007406	BP	GO:0007406	negative regulation of neuroblast proliferation	3/666	11/18614	0.00606753477843177	0.0193523253964907	0.0110951812574622	PROX1/PTN/VAX1	3	2.72727272727273
GO:0007614	BP	GO:0007614	short-term memory	3/666	11/18614	0.00606753477843177	0.0193523253964907	0.0110951812574622	CALB1/CHRNA7/SLC2A4	3	2.72727272727273
GO:0010739	BP	GO:0010739	positive regulation of protein kinase A signaling	3/666	11/18614	0.00606753477843177	0.0193523253964907	0.0110951812574622	ADRB2/MC1R/MIF	3	2.72727272727273
GO:0010960	BP	GO:0010960	magnesium ion homeostasis	3/666	11/18614	0.00606753477843177	0.0193523253964907	0.0110951812574622	ANK3/EDN3/KCNA1	3	2.72727272727273
GO:0014820	BP	GO:0014820	tonic smooth muscle contraction	3/666	11/18614	0.00606753477843177	0.0193523253964907	0.0110951812574622	AGT/EDN2/EDNRA	3	2.72727272727273
GO:0016081	BP	GO:0016081	synaptic vesicle docking	3/666	11/18614	0.00606753477843177	0.0193523253964907	0.0110951812574622	SNAP25/STX1A/UNC13C	3	2.72727272727273
GO:0021548	BP	GO:0021548	pons development	3/666	11/18614	0.00606753477843177	0.0193523253964907	0.0110951812574622	ASCL1/BCL2/CDK5R2	3	2.72727272727273
GO:0021559	BP	GO:0021559	trigeminal nerve development	3/666	11/18614	0.00606753477843177	0.0193523253964907	0.0110951812574622	PLXNA3/POU4F1/SEMA3F	3	2.72727272727273
GO:0021781	BP	GO:0021781	glial cell fate commitment	3/666	11/18614	0.00606753477843177	0.0193523253964907	0.0110951812574622	ASCL1/SOX2/SOX9	3	2.72727272727273
GO:0031272	BP	GO:0031272	regulation of pseudopodium assembly	3/666	11/18614	0.00606753477843177	0.0193523253964907	0.0110951812574622	APC/CCR7/F2RL1	3	2.72727272727273
GO:0031274	BP	GO:0031274	positive regulation of pseudopodium assembly	3/666	11/18614	0.00606753477843177	0.0193523253964907	0.0110951812574622	APC/CCR7/F2RL1	3	2.72727272727273
GO:0033860	BP	GO:0033860	regulation of NAD(P)H oxidase activity	3/666	11/18614	0.00606753477843177	0.0193523253964907	0.0110951812574622	AGT/AGTR1/DRD5	3	2.72727272727273
GO:0042711	BP	GO:0042711	maternal behavior	3/666	11/18614	0.00606753477843177	0.0193523253964907	0.0110951812574622	NR3C1/OXT/OXTR	3	2.72727272727273
GO:0048755	BP	GO:0048755	branching morphogenesis of a nerve	3/666	11/18614	0.00606753477843177	0.0193523253964907	0.0110951812574622	DRD2/FGF13/LRRK2	3	2.72727272727273
GO:0060746	BP	GO:0060746	parental behavior	3/666	11/18614	0.00606753477843177	0.0193523253964907	0.0110951812574622	NR3C1/OXT/OXTR	3	2.72727272727273
GO:0070944	BP	GO:0070944	neutrophil-mediated killing of bacterium	3/666	11/18614	0.00606753477843177	0.0193523253964907	0.0110951812574622	CTSG/F2/F2RL1	3	2.72727272727273
GO:0072205	BP	GO:0072205	metanephric collecting duct development	3/666	11/18614	0.00606753477843177	0.0193523253964907	0.0110951812574622	CALB1/SHH/WNT7B	3	2.72727272727273
GO:0098887	BP	GO:0098887	neurotransmitter receptor transport, endosome to postsynaptic membrane	3/666	11/18614	0.00606753477843177	0.0193523253964907	0.0110951812574622	ARHGAP44/LRRC7/NSG1	3	2.72727272727273
GO:0099624	BP	GO:0099624	atrial cardiac muscle cell membrane repolarization	3/666	11/18614	0.00606753477843177	0.0193523253964907	0.0110951812574622	CACNA1D/KCNJ5/KCNQ1	3	2.72727272727273
GO:0099639	BP	GO:0099639	neurotransmitter receptor transport, endosome to plasma membrane	3/666	11/18614	0.00606753477843177	0.0193523253964907	0.0110951812574622	ARHGAP44/LRRC7/NSG1	3	2.72727272727273
GO:2000253	BP	GO:2000253	positive regulation of feeding behavior	3/666	11/18614	0.00606753477843177	0.0193523253964907	0.0110951812574622	INSL5/MC1R/NPY	3	2.72727272727273

	ONTOLOGY	ID	Description	GeneRatio	BgRatio	pvalue	p.adjust	qvalue	geneID	Count	richFactor
GO:0030177	BP	GO:0030177	positive regulation of Wnt signaling pathway	12/666	146/18614	0.00627371734050701	0.0199969654530428	0.0114647698276587	ASPM/ATP6V1C2/CAV1/CSNK1E/DKK1/FZD9/LRRK2/SHH/SULF1/WNT3/WNT3A/WNT5A	12	0.821917808219178
GO:0001774	BP	GO:0001774	microglial cell activation	6/666	47/18614	0.00632255204368046	0.0201134908056243	0.0115315767814223	CLU/CX3CR1/LRRK2/MAPT/SNCA/SYT11	6	1.27659574468085
GO:0021517	BP	GO:0021517	ventral spinal cord development	6/666	47/18614	0.00632255204368046	0.0201134908056243	0.0115315767814223	ASCL1/LHX1/RELN/SHH/SOX1/TBX20	6	1.27659574468085
GO:0031577	BP	GO:0031577	spindle checkpoint signaling	6/666	47/18614	0.00632255204368046	0.0201134908056243	0.0115315767814223	APC/BUB1B/CCNB1/DUSP1/MAD2L1/TTK	6	1.27659574468085
GO:0001959	BP	GO:0001959	regulation of cytokine-mediated signaling pathway	13/666	165/18614	0.00646281952721881	0.020546415249858	0.0117797833964491	AXL/CAV1/CCL5/CPNE1/CSF1/F2RL1/IRAK1/IRAK2/PRKN/RIPK2/SLIT2/SLIT3/WNT5A	13	0.787878787878788
GO:0001738	BP	GO:0001738	morphogenesis of a polarized epithelium	9/666	94/18614	0.00648024583614016	0.020588499214369	0.0118039112056253	CELSR1/DKK1/ERBB4/FZD3/GPC6/MAGI2/WNT11/WNT5A/WNT7A	9	0.957446808510638
GO:0045428	BP	GO:0045428	regulation of nitric oxide biosynthetic process	7/666	62/18614	0.00649628808579356	0.0206128185549873	0.0118178541032716	AGT/CAV1/CLU/CX3CR1/HSP90AB1/P2RX4/PTK2B	7	1.12903225806452
GO:0046530	BP	GO:0046530	photoreceptor cell differentiation	7/666	62/18614	0.00649628808579356	0.0206128185549873	0.0118178541032716	GNGT1/HCN1/OLFM3/SOX9/TH/THRB/VEGFA	7	1.12903225806452
GO:0032874	BP	GO:0032874	positive regulation of stress-activated MAPK cascade	11/666	129/18614	0.00663874882153009	0.0210376856594587	0.0120614412401073	CCR7/F2RL1/IL1A/LEP/PTK2B/RIPK2/VEGFA/WNT16/WNT5A/WNT7A/WNT7B	11	0.852713178294574
GO:0042542	BP	GO:0042542	response to hydrogen peroxide	11/666	129/18614	0.00663874882153009	0.0210376856594587	0.0120614412401073	AXL/BCL2/CAPN2/CDK1/FKBP1B/LRRK2/PINK1/PTK2B/SIGMAR1/TRAP1/TXNIP	11	0.852713178294574
GO:0090287	BP	GO:0090287	regulation of cellular response to growth factor stimulus	22/666	345/18614	0.00665159063719354	0.0210647989573494	0.0120769859846494	AGT/APLN/CAV1/CHRDL1/CXCL13/DKK1/FGFR1/FSTL4/HSP90AB1/ITGB3/NGFR/NKX2-1/PRKCB/SEMA6A/SLIT2/SULF1/TBX20/TMEM108/VEGFA/VWC2/WNT1/WNT5A	22	0.63768115942029
GO:0002437	BP	GO:0002437	inflammatory response to antigenic stimulus	8/666	78/18614	0.00667601198969996	0.0211285247452513	0.0121135216026209	A2M/CCR7/CNR1/CYSLTR1/NPY/NPY5R/OPRM1/PLCG1	8	1.02564102564103
GO:0009314	BP	GO:0009314	response to radiation	27/666	452/18614	0.00674258052259049	0.0213254718587724	0.0122264364011209	ATP1A2/BCL2/BCL2L1/CASP7/CCND1/CHRNB2/COL3A1/CXCL12/DRD2/GNA11/GNAQ/GNGT1/GPR88/GRIN2A/IL1A/ITGB3/MAPK10/MC1R/MMP1/MMP9/NPY/OPRM1/PENK/SLC1A2/SLC7A11/TH/TUBA1A	27	0.597345132743363
GO:0030856	BP	GO:0030856	regulation of epithelial cell differentiation	13/666	166/18614	0.00679010467706865	0.0214424489376844	0.0122935023411	ASCL1/ATOH1/CAV1/CCND1/GDNF/IL1A/LHX1/MMP9/NKX6-1/SOX9/TP73/VCL/VEGFA	13	0.783132530120482
GO:0036475	BP	GO:0036475	neuron death in response to oxidative stress	5/666	34/18614	0.00680137902214648	0.0214424489376844	0.0122935023411	PINK1/PRKN/SLC1A1/SLC7A11/WNT1	5	1.47058823529412
GO:0060563	BP	GO:0060563	neuroepithelial cell differentiation	5/666	34/18614	0.00680137902214648	0.0214424489376844	0.0122935023411	ASCL1/EMX1/MAP1B/VAX1/WNT11	5	1.47058823529412
GO:0090659	BP	GO:0090659	walking behavior	5/666	34/18614	0.00680137902214648	0.0214424489376844	0.0122935023411	CNTN2/DRD2/EPHA4/GLRB/UCHL1	5	1.47058823529412
GO:1901797	BP	GO:1901797	negative regulation of signal transduction by p53 class mediator	5/666	34/18614	0.00680137902214648	0.0214424489376844	0.0122935023411	BCL2/BDKRB2/MIF/PRKN/TWIST1	5	1.47058823529412
GO:0046330	BP	GO:0046330	positive regulation of JNK cascade	9/666	95/18614	0.00693799242436536	0.0218591235677922	0.0125323925236343	CCR7/F2RL1/IL1A/PTK2B/RIPK2/WNT16/WNT5A/WNT7A/WNT7B	9	0.947368421052632

	ONTOLOGY	ID	Description	GeneRatio	BgRatio	pvalue	p.adjust	qvalue	geneID	Count	richFactor
GO:0071216	BP	GO:0071216	cellular response to biotic stimulus	18/666	264/18614	0.00694803780034019	0.0218767493841589	0.0125424978532727	AXL/CAPN2/CCL5/CTSG/CX3CR1/CXCL13/CXCL8/EDNRB/EPHB2/IL1A/IRAK1/IRAK2/MEF2C/MIF/PLCG2/RIPK2/TXNIP/WNT5A	18	0.681818181818182
GO:0008542	BP	GO:0008542	visual learning	6/666	48/18614	0.00701232151020294	0.0219386124905458	0.0125779655484695	ATP1A2/CHRNB2/DRD2/GRIN2A/SLC7A11/TUBA1A	6	1.25
GO:0033046	BP	GO:0033046	negative regulation of sister chromatid segregation	6/666	48/18614	0.00701232151020294	0.0219386124905458	0.0125779655484695	APC/BUB1B/CCNB1/DUSP1/MAD2L1/TTK	6	1.25
GO:0033048	BP	GO:0033048	negative regulation of mitotic sister chromatid segregation	6/666	48/18614	0.00701232151020294	0.0219386124905458	0.0125779655484695	APC/BUB1B/CCNB1/DUSP1/MAD2L1/TTK	6	1.25
GO:0035987	BP	GO:0035987	endodermal cell differentiation	6/666	48/18614	0.00701232151020294	0.0219386124905458	0.0125779655484695	DKK1/HMGA2/INHBA/ITGA4/MMP9/SOX2	6	1.25
GO:0036230	BP	GO:0036230	granulocyte activation	6/666	48/18614	0.00701232151020294	0.0219386124905458	0.0125779655484695	CCL5/CCR2/CTSG/CXCL8/F2RL1/VAMP2	6	1.25
GO:0042461	BP	GO:0042461	photoreceptor cell development	6/666	48/18614	0.00701232151020294	0.0219386124905458	0.0125779655484695	GNGT1/HCN1/OLFM3/TH/THRB/VEGFA	6	1.25
GO:0045841	BP	GO:0045841	negative regulation of mitotic metaphase/anaphase transition	6/666	48/18614	0.00701232151020294	0.0219386124905458	0.0125779655484695	APC/BUB1B/CCNB1/DUSP1/MAD2L1/TTK	6	1.25
GO:0070509	BP	GO:0070509	calcium ion import	6/666	48/18614	0.00701232151020294	0.0219386124905458	0.0125779655484695	ATP2A1/CACNA1D/CXCL12/GCG/HOMER1/UCN	6	1.25
GO:1902003	BP	GO:1902003	regulation of amyloid-beta formation	6/666	48/18614	0.00701232151020294	0.0219386124905458	0.0125779655484695	CHRNA7/CLU/CSNK1E/EPHA4/IGF1/PRNP	6	1.25
GO:2000816	BP	GO:2000816	negative regulation of mitotic sister chromatid separation	6/666	48/18614	0.00701232151020294	0.0219386124905458	0.0125779655484695	APC/BUB1B/CCNB1/DUSP1/MAD2L1/TTK	6	1.25
GO:0000302	BP	GO:0000302	response to reactive oxygen species	15/666	205/18614	0.00706684254629393	0.0220925389548624	0.0126662155125913	AXL/BCL2/CAPN2/CDK1/FKBP1B/IL1A/LRRK2/MAPT/MMP9/PINK1/PTK2B/SIGMAR1/TACR1/TRAP1/TXNIP	15	0.731707317073171
GO:0016050	BP	GO:0016050	vesicle organization	23/666	368/18614	0.00708509791636621	0.0220925389548624	0.0126662155125913	ASIP/ATP6V0A4/ATP6V1F/ATP6V1G2/BCL2/CAV1/CPLX1/GPR143/GRIK5/P2RX7/PRKN/SLC2A4/SNAP25/SNCA/STX1A/STXBP1/SYP/SYT11/SYT4/SYT7/SYT9/UNC13C/VAMP2	23	0.625
GO:0001885	BP	GO:0001885	endothelial cell development	7/666	63/18614	0.00708865254360804	0.0220925389548624	0.0126662155125913	CLDN1/EDNRA/EDNRB/F2RL1/RAP1A/VCL/VEGFA	7	1.11111111111111
GO:0009713	BP	GO:0009713	catechol-containing compound biosynthetic process	4/666	22/18614	0.00710647082149289	0.0220925389548624	0.0126662155125913	PAH/SLC6A3/SNCA/TH	4	1.81818181818182
GO:0010881	BP	GO:0010881	regulation of cardiac muscle contraction by regulation of the release of sequestered calcium ion	4/666	22/18614	0.00710647082149289	0.0220925389548624	0.0126662155125913	ATP1A2/CACNA1C/CAMK2D/FKBP1B	4	1.81818181818182
GO:0034104	BP	GO:0034104	negative regulation of tissue remodeling	4/666	22/18614	0.00710647082149289	0.0220925389548624	0.0126662155125913	AGT/CALCA/CARTPT/P2RX7	4	1.81818181818182
GO:0035584	BP	GO:0035584	calcium-mediated signaling using intracellular calcium source	4/666	22/18614	0.00710647082149289	0.0220925389548624	0.0126662155125913	FKBP1B/GPR143/PRNP/PTGFR	4	1.81818181818182
GO:0042423	BP	GO:0042423	catecholamine biosynthetic process	4/666	22/18614	0.00710647082149289	0.0220925389548624	0.0126662155125913	PAH/SLC6A3/SNCA/TH	4	1.81818181818182
GO:0050961	BP	GO:0050961	detection of temperature stimulus involved in sensory perception	4/666	22/18614	0.00710647082149289	0.0220925389548624	0.0126662155125913	CALCA/CXCL12/GRIK2/NTSR1	4	1.81818181818182

	ONTOLOGY	ID	Description	GeneRatio	BgRatio	pvalue	p.adjust	qvalue	geneID	Count	richFactor
GO:2000831	BP	GO:2000831	regulation of steroid hormone secretion	4/666	22/18614	0.00710647082149289	0.0220925389548624	0.0126662155125913	AGT/AGTR1/GAL/GALR1	4	1.81818181818182
GO:0010506	BP	GO:0010506	regulation of autophagy	22/666	348/18614	0.00733258066281339	0.0227810581275144	0.0130609610981113	ADRA1A/ADRB2/ATP6V0D2/ATP6V1C2/ATP6V1E2/ATP6V1G2/BCL2/HTR2B/ITPR1/LEP/LEPR/LRRK2/MAGEA3/MAGEA6/MAPT/PIK3R2/PINK1/PRKN/RAB39B/RIPK2/SNCA/UCHL1	22	0.632183908045977
GO:0032091	BP	GO:0032091	negative regulation of protein binding	9/666	96/18614	0.0074200978532684	0.0230383960510513	0.0132084994867836	ADRB2/ATP2A3/AURKA/CAV1/CSNK1E/DKK1/ITGA4/LRRK2/MAP2	9	0.9375
GO:0070304	BP	GO:0070304	positive regulation of stress-activated protein kinase signaling cascade	11/666	131/18614	0.00742977690436111	0.0230393397381292	0.013209040526631	CCR7/F2RL1/IL1A/LEP/PTK2B/RIPK2/VEGFA/WNT16/WNT5A/WNT7A/WNT7B	11	0.83969465648855
GO:0071621	BP	GO:0071621	granulocyte chemotaxis	11/666	131/18614	0.00742977690436111	0.0230393397381292	0.013209040526631	CCL5/CCR7/CSF1/CXCL13/CXCL8/EDN2/EDN3/PIK3CD/PIK3CG/SCG2/SLIT2	11	0.83969465648855
GO:0051302	BP	GO:0051302	regulation of cell division	14/666	187/18614	0.00748343495845606	0.023178459788895	0.0132888015965887	ASPM/AURKA/BCL2L1/CXCR5/DRD2/HTR2B/IL1A/KIF14/PTN/RXFP3/SHH/SSTR5/TXNIP/VEGFA	14	0.748663101604278
GO:0007156	BP	GO:0007156	homophilic cell adhesion via plasma membrane adhesion molecules	13/666	168/18614	0.00748407236723831	0.023178459788895	0.0132888015965887	CDH4/CELSR1/CNTN2/CNTN4/CNTN6/DSCAML1/IGSF9/KIRREL3/L1CAM/MYPN/NEXN/PALLD/ROBO2	13	0.773809523809524
GO:0045444	BP	GO:0045444	fat cell differentiation	17/666	246/18614	0.00752937543507157	0.0233040807703884	0.013360823306169	ADRB1/ADRB2/ADRB3/AXIN2/CCND1/CNTN2/GPER1/HMGA2/HTR2C/LEP/MMP11/RGS2/SLC2A4/TPH1/WNT1/WNT3A/WNT5A	17	0.691056910569106
GO:0062197	BP	GO:0062197	cellular response to chemical stress	22/666	349/18614	0.00757186364267224	0.0234208368808899	0.013427762559328	AXL/BCL2/BDKRB2/CAV1/CDK1/EDNRA/EPAS1/FAS/LRRK2/MAPT/MMP9/PENK/PINK1/PRKN/SIGMAR1/SLC1A1/SLC2A4/SLC7A11/SNCA/TRAP1/WNT1/WNT16	22	0.630372492836676
GO:0014808	BP	GO:0014808	release of sequestered calcium ion into cytosol by sarcoplasmic reticulum	5/666	35/18614	0.00770623614458876	0.023744903659845	0.0136135583010987	ATP1A2/CACNA1C/CAMK2D/FKBP1B/RYR1	5	1.42857142857143
GO:0035633	BP	GO:0035633	maintenance of blood-brain barrier	5/666	35/18614	0.00770623614458876	0.023744903659845	0.0136135583010987	CLDN1/SH3GL2/SLC1A1/VCL/VEGFA	5	1.42857142857143
GO:0048048	BP	GO:0048048	embryonic eye morphogenesis	5/666	35/18614	0.00770623614458876	0.023744903659845	0.0136135583010987	PROX1/TH/TWIST1/WNT16/WNT5A	5	1.42857142857143
GO:0048333	BP	GO:0048333	mesodermal cell differentiation	5/666	35/18614	0.00770623614458876	0.023744903659845	0.0136135583010987	DKK1/HMGA2/INHBA/ITGB3/WNT3A	5	1.42857142857143
GO:0060999	BP	GO:0060999	positive regulation of dendritic spine development	5/666	35/18614	0.00770623614458876	0.023744903659845	0.0136135583010987	CAMK2B/EPHB2/LPAR1/NLGN1/RELN	5	1.42857142857143
GO:0061081	BP	GO:0061081	positive regulation of myeloid leukocyte cytokine production involved in immune response	5/666	35/18614	0.00770623614458876	0.023744903659845	0.0136135583010987	MIF/P2RX7/PLCG2/RIPK2/WNT5A	5	1.42857142857143
GO:0046503	BP	GO:0046503	glycerolipid catabolic process	7/666	64/18614	0.00772011313294654	0.023744903659845	0.0136135583010987	APOC1/GPIHBP1/MGLL/PIK3CG/PLA2G4B/PLA2G4D/PLA2G4E	7	1.09375
GO:0048247	BP	GO:0048247	lymphocyte chemotaxis	7/666	64/18614	0.00772011313294654	0.023744903659845	0.0136135583010987	CCL5/CCR2/CXCL13/PIK3CD/PIK3CG/PTK2B/WNT5A	7	1.09375
GO:0071384	BP	GO:0071384	cellular response to corticosteroid stimulus	7/666	64/18614	0.00772011313294654	0.023744903659845	0.0136135583010987	AXIN2/DDIT4/GPER1/NR3C1/SSTR2/SSTR3/SSTR5	7	1.09375

	ONTOLOGY	ID	Description	GeneRatio	BgRatio	pvalue	p.adjust	qvalue	geneID	Count	richFactor
GO:0060043	BP	GO:0060043	regulation of cardiac muscle cell proliferation	6/666	49/18614	0.00775492499299156	0.0238185785269035	0.0136557979796585	CDK1/ERBB4/MEF2C/TBX20/TP73/WNT2	6	1.22448979591837
GO:0042440	BP	GO:0042440	pigment metabolic process	8/666	80/18614	0.00775860513155085	0.0238185785269035	0.0136557979796585	ASIP/BCL2/GPR143/MC1R/SLC6A9/SLC7A11/TRPC1/WNT5A	8	1
GO:0048644	BP	GO:0048644	muscle organ morphogenesis	8/666	80/18614	0.00775860513155085	0.0238185785269035	0.0136557979796585	COL3A1/EDNRA/POU4F1/PROX1/S1PR1/TBX20/WNT2/WNT5A	8	1
GO:0001967	BP	GO:0001967	suckling behavior	3/666	12/18614	0.00787705581443902	0.0238544234922783	0.0136763488116569	CNTFR/OXTR/POU4F1	3	2.5
GO:0006995	BP	GO:0006995	cellular response to nitrogen starvation	3/666	12/18614	0.00787705581443902	0.0238544234922783	0.0136763488116569	BECN2/GABARAP/MAP1LC3C	3	2.5
GO:0010459	BP	GO:0010459	negative regulation of heart rate	3/666	12/18614	0.00787705581443902	0.0238544234922783	0.0136763488116569	ADRA1A/FKBP1B/SPX	3	2.5
GO:0014051	BP	GO:0014051	gamma-aminobutyric acid secretion	3/666	12/18614	0.00787705581443902	0.0238544234922783	0.0136763488116569	NTSR1/P2RX7/SLC6A1	3	2.5
GO:0031223	BP	GO:0031223	auditory behavior	3/666	12/18614	0.00787705581443902	0.0238544234922783	0.0136763488116569	DRD2/NRXN1/NRXN2	3	2.5
GO:0031650	BP	GO:0031650	regulation of heat generation	3/666	12/18614	0.00787705581443902	0.0238544234922783	0.0136763488116569	CNR1/EDNRB/PTGER3	3	2.5
GO:0033083	BP	GO:0033083	regulation of immature T cell proliferation	3/666	12/18614	0.00787705581443902	0.0238544234922783	0.0136763488116569	IL1A/RIPK2/SHH	3	2.5
GO:0033084	BP	GO:0033084	regulation of immature T cell proliferation in thymus	3/666	12/18614	0.00787705581443902	0.0238544234922783	0.0136763488116569	IL1A/RIPK2/SHH	3	2.5
GO:0035933	BP	GO:0035933	glucocorticoid secretion	3/666	12/18614	0.00787705581443902	0.0238544234922783	0.0136763488116569	GAL/GALR1/KCNQ1	3	2.5
GO:0042416	BP	GO:0042416	dopamine biosynthetic process	3/666	12/18614	0.00787705581443902	0.0238544234922783	0.0136763488116569	SLC6A3/SNCA/TH	3	2.5
GO:0043562	BP	GO:0043562	cellular response to nitrogen levels	3/666	12/18614	0.00787705581443902	0.0238544234922783	0.0136763488116569	BECN2/GABARAP/MAP1LC3C	3	2.5
GO:0048521	BP	GO:0048521	negative regulation of behavior	3/666	12/18614	0.00787705581443902	0.0238544234922783	0.0136763488116569	DRD2/NPSR1/UCN	3	2.5
GO:0048681	BP	GO:0048681	negative regulation of axon regeneration	3/666	12/18614	0.00787705581443902	0.0238544234922783	0.0136763488116569	EPHA4/RTN4R/TNR	3	2.5
GO:0051001	BP	GO:0051001	negative regulation of nitric-oxide synthase activity	3/666	12/18614	0.00787705581443902	0.0238544234922783	0.0136763488116569	CAV1/CNR1/CNR2	3	2.5
GO:0060788	BP	GO:0060788	ectodermal placode formation	3/666	12/18614	0.00787705581443902	0.0238544234922783	0.0136763488116569	DKK4/NRG3/PROX1	3	2.5
GO:0070943	BP	GO:0070943	neutrophil-mediated killing of symbiont cell	3/666	12/18614	0.00787705581443902	0.0238544234922783	0.0136763488116569	CTSG/F2/F2RL1	3	2.5
GO:0071372	BP	GO:0071372	cellular response to follicle-stimulating hormone stimulus	3/666	12/18614	0.00787705581443902	0.0238544234922783	0.0136763488116569	EDNRA/EFNA5/INHBA	3	2.5
GO:0071697	BP	GO:0071697	ectodermal placode morphogenesis	3/666	12/18614	0.00787705581443902	0.0238544234922783	0.0136763488116569	DKK4/NRG3/PROX1	3	2.5
GO:0072497	BP	GO:0072497	mesenchymal stem cell differentiation	3/666	12/18614	0.00787705581443902	0.0238544234922783	0.0136763488116569	SOX5/SOX9/WNT3	3	2.5
GO:1901731	BP	GO:1901731	positive regulation of platelet aggregation	3/666	12/18614	0.00787705581443902	0.0238544234922783	0.0136763488116569	CTSG/IL6ST/PDPN	3	2.5
GO:1902950	BP	GO:1902950	regulation of dendritic spine maintenance	3/666	12/18614	0.00787705581443902	0.0238544234922783	0.0136763488116569	GRIN2B/HOMER1/PRNP	3	2.5
GO:1905809	BP	GO:1905809	negative regulation of synapse organization	3/666	12/18614	0.00787705581443902	0.0238544234922783	0.0136763488116569	DKK1/ROBO2/WNT5A	3	2.5
GO:0051153	BP	GO:0051153	regulation of striated muscle cell differentiation	9/666	97/18614	0.007927371468069	0.0239920140182014	0.0137552329660492	BCL2/BDNF/DKK1/IGF1/MEF2C/PROX1/RGS2/SHH/WNT3A	9	0.927835051546392

	ONTOLOGY	ID	Description	GeneRatio	BgRatio	pvalue	p.adjust	qvalue	geneID	Count	richFactor
GO:0051259	BP	GO:0051259	protein complex oligomerization	17/666	248/18614	0.00813283222906961	0.02459597414626	0.0141014986966939	ASIC1/CLDN1/GLRA3/GRIN2B/HCN1/HOMER1/KCNA1/KCNA2/MIF/PRNP/RRM2/RYR1/RYR3/SIGMAR1/SLC1A2/SNCA/TP73	17	0.685483870967742
GO:0010821	BP	GO:0010821	regulation of mitochondrion organization	12/666	151/18614	0.008136938751133	0.02459597414626	0.0141014986966939	AURKA/BCL2L1/BID/CLU/FZD9/GPER1/IGF1/LRRK2/MAPT/MMP9/PINK1/PRKN	12	0.794701986754967
GO:0042303	BP	GO:0042303	molting cycle	10/666	115/18614	0.00825222257311944	0.0249138046356769	0.0142837190147684	BCL2/DKK1/DKK4/FZD3/GAL/INHBA/NGFR/SHH/SOX9/WNT5A	10	0.869565217391304
GO:0042633	BP	GO:0042633	hair cycle	10/666	115/18614	0.00825222257311944	0.0249138046356769	0.0142837190147684	BCL2/DKK1/DKK4/FZD3/GAL/INHBA/NGFR/SHH/SOX9/WNT5A	10	0.869565217391304
GO:0035493	BP	GO:0035493	SNARE complex assembly	4/666	23/18614	0.00836324179434963	0.0251562628024654	0.0144227264597526	SNCA/STX1A/STXBP1/VAMP2	4	1.73913043478261
GO:0046058	BP	GO:0046058	cAMP metabolic process	4/666	23/18614	0.00836324179434963	0.0251562628024654	0.0144227264597526	ADCY2/ADCY3/ADCY5/ADCY9	4	1.73913043478261
GO:0097164	BP	GO:0097164	ammonium ion metabolic process	4/666	23/18614	0.00836324179434963	0.0251562628024654	0.0144227264597526	BCHE/GRIN2A/SLC22A3/TPH1	4	1.73913043478261
GO:1900120	BP	GO:1900120	regulation of receptor binding	4/666	23/18614	0.00836324179434963	0.0251562628024654	0.0144227264597526	ATP2A3/BDNF/MMP9/PLCL1	4	1.73913043478261
GO:1902254	BP	GO:1902254	negative regulation of intrinsic apoptotic signaling pathway by p53 class mediator	4/666	23/18614	0.00836324179434963	0.0251562628024654	0.0144227264597526	BCL2/BDKRB2/MIF/PRKN	4	1.73913043478261
GO:2000178	BP	GO:2000178	negative regulation of neural precursor cell proliferation	4/666	23/18614	0.00836324179434963	0.0251562628024654	0.0144227264597526	PROX1/PTN/VAX1/WNT5A	4	1.73913043478261
GO:0009952	BP	GO:0009952	anterior/posterior pattern specification	15/666	209/18614	0.00838108094669341	0.025194503274005	0.014444650696474	AURKA/AXIN2/DKK1/FEZF1/FEZF2/LHX1/NEUROD1/PLXNA2/SHH/WNT1/WNT2/WNT2B/WNT3/WNT3A/WNT5A	15	0.717703349282297
GO:0007018	BP	GO:0007018	microtubule-based movement	25/666	417/18614	0.008535936652285	0.0255994287308869	0.0146768047786227	ADCY3/CALY/DLG2/DNAAF4/DNAH10/DNAH12/DNAH14/DNAH2/DNAH5/KIF14/KIF1A/KIF5A/KIF5C/KLC3/MAP1B/MAP2/MAPT/NEFH/NEFL/TACR1/TACR2/TMEM108/TRAK2/TUBA1A/UCHL1	25	0.599520383693046
GO:0001974	BP	GO:0001974	blood vessel remodeling	6/666	50/18614	0.00855251509730218	0.0255994287308869	0.0146768047786227	AGT/AXL/CCR2/EDNRA/EPAS1/MEF2C	6	1.2
GO:0002269	BP	GO:0002269	leukocyte activation involved in inflammatory response	6/666	50/18614	0.00855251509730218	0.0255994287308869	0.0146768047786227	CLU/CX3CR1/LRRK2/MAPT/SNCA/SYT11	6	1.2
GO:0048546	BP	GO:0048546	digestive tract morphogenesis	6/666	50/18614	0.00855251509730218	0.0255994287308869	0.0146768047786227	BCL2/EPHB3/SHH/SOX10/WNT11/WNT5A	6	1.2
GO:0051985	BP	GO:0051985	negative regulation of chromosome segregation	6/666	50/18614	0.00855251509730218	0.0255994287308869	0.0146768047786227	APC/BUB1B/CCNB1/DUSP1/MAD2L1/TTK	6	1.2
GO:1902100	BP	GO:1902100	negative regulation of metaphase/anaphase transition of cell cycle	6/666	50/18614	0.00855251509730218	0.0255994287308869	0.0146768047786227	APC/BUB1B/CCNB1/DUSP1/MAD2L1/TTK	6	1.2
GO:1905819	BP	GO:1905819	negative regulation of chromosome separation	6/666	50/18614	0.00855251509730218	0.0255994287308869	0.0146768047786227	APC/BUB1B/CCNB1/DUSP1/MAD2L1/TTK	6	1.2
GO:0051495	BP	GO:0051495	positive regulation of cytoskeleton organization	14/666	190/18614	0.00855744891248162	0.0255994287308869	0.0146768047786227	CCR7/F2RL1/LIMK1/LPAR1/MAP1B/MAPT/NTRK3/P2RX7/PLXNA3/PROX1/PTK2B/TACR1/TENM1/WNT11	14	0.736842105263158
GO:0010613	BP	GO:0010613	positive regulation of cardiac muscle hypertrophy	5/666	36/18614	0.00869150228307885	0.025953058153908	0.0148795495375085	ADRA1A/AGT/CAMK2D/IGF1/IL6ST	5	1.38888888888889

	ONTOLOGY	ID	Description	GeneRatio	BgRatio	pvalue	p.adjust	qvalue	geneID	Count	richFactor
GO:1903514	BP	GO:1903514	release of sequestered calcium ion into cytosol by endoplasmic reticulum	5/666	36/18614	0.00869150228307885	0.025953058153908	0.0148795495375085	ATP1A2/CACNA1C/CAMK2D/FKBP1B/RYR1	5	1.38888888888889
GO:1903725	BP	GO:1903725	regulation of phospholipid metabolic process	5/666	36/18614	0.00869150228307885	0.025953058153908	0.0148795495375085	APOC1/CAPN2/CHRM5/HTR2B/HTR2C	5	1.38888888888889
GO:1903050	BP	GO:1903050	regulation of proteolysis involved in protein catabolic process	16/666	230/18614	0.00880279282900714	0.0262694151515301	0.0150609250651916	AURKA/AXIN2/CAV1/CLU/CSNK1E/DNAAF4/EPHA4/GABARAP/HSP90AB1/LRRK2/PINK1/PRKCG/PRKN/PTK2B/SHH/WNT1	16	0.695652173913043
GO:2001021	BP	GO:2001021	negative regulation of response to DNA damage stimulus	8/666	82/18614	0.00896807281511986	0.0267464064844139	0.0153343963503295	BCL2/BCL2L1/BID/CLU/CXCL12/HMGA2/MIF/TWIST1	8	0.975609756097561
GO:0007292	BP	GO:0007292	female gamete generation	12/666	153/18614	0.00899327294364787	0.0268052980703634	0.0153681603971436	ASPM/AURKA/BCL2/EDNRA/IGF1/INHBA/LEP/PTK2B/PTN/RGS2/TOP2A/TTK	12	0.784313725490196
GO:0003300	BP	GO:0003300	cardiac muscle hypertrophy	9/666	99/18614	0.00902067834718759	0.0268381562205975	0.0153869988119161	ADRA1A/AGT/CAMK2D/HTR2B/IGF1/IL6ST/LEP/P2RX4/RGS2	9	0.909090909090909
GO:0036473	BP	GO:0036473	cell death in response to oxidative stress	9/666	99/18614	0.00902067834718759	0.0268381562205975	0.0153869988119161	BCL2/LRRK2/PINK1/PRKN/SIGMAR1/SLC1A1/SLC7A11/TRAP1/WNT1	9	0.909090909090909
GO:0048661	BP	GO:0048661	positive regulation of smooth muscle cell proliferation	9/666	99/18614	0.00902067834718759	0.0268381562205975	0.0153869988119161	AGT/CCL5/IGF1/IL6R/IRAK1/ITGB3/MMP9/NPY5R/S1PR1	9	0.909090909090909
GO:0031100	BP	GO:0031100	animal organ regeneration	7/666	66/18614	0.00910621245147492	0.0270762457344218	0.0155235015968404	AURKA/AXL/CCND1/CDK1/CLDN1/CXCL12/WNT1	7	1.06060606060606
GO:0044403	BP	GO:0044403	biological process involved in symbiotic interaction	20/666	313/18614	0.00918820494353199	0.0273035231544497	0.0156538055328932	AGTR1/AVPR1B/AXL/BCL2L1/CAV1/CCL5/CDK1/CLDN1/CTSG/CX3CR1/CXCL8/F2/F2RL1/HMGA2/HSP90AB1/ITGB3/LY6E/NCAM1/PRKN/TYRO3	20	0.638977635782748
GO:0007088	BP	GO:0007088	regulation of mitotic nuclear division	10/666	117/18614	0.0092719776858525	0.0275358128857795	0.0157869831547318	APC/AURKA/BUB1B/CCNB1/DUSP1/EDN3/IGF1/IL1A/MAD2L1/TTK	10	0.854700854700855
GO:0032886	BP	GO:0032886	regulation of microtubule-based process	17/666	252/18614	0.00945610155723637	0.0280656637402275	0.0160907601504715	APC/AURKA/CALY/EFNA5/EPHA3/FGF13/GNAI1/MAP1B/MAP2/MAPT/NEFH/SNCA/TACR1/TACR2/TUBB4A/WDR62/WNT3A	17	0.674603174603175
GO:0035282	BP	GO:0035282	segmentation	9/666	100/18614	0.00960834529474391	0.0284774740371225	0.0163268614868291	AXIN2/DKK1/LHX1/NRP2/PLXNA2/SEMA3F/SHH/WNT3A/WNT5A	9	0.9
GO:1901019	BP	GO:1901019	regulation of calcium ion transmembrane transporter activity	9/666	100/18614	0.00960834529474391	0.0284774740371225	0.0163268614868291	ADRA2A/ATP1A2/ATP2A1/CAMK2D/DRD4/FKBP1B/HPCA/NOS1/PLCG2	9	0.9
GO:0002683	BP	GO:0002683	negative regulation of immune system process	28/666	487/18614	0.00961223386115692	0.0284774740371225	0.0163268614868291	A2M/AXL/CARTPT/CCR2/CNR1/CNR2/COL3A1/CTSG/CX3CR1/CXCL12/DRD2/DUSP1/F2RL1/GAL/GPER1/INHBA/MIF/NPY/NPY5R/PLCG1/PRNP/PTGER4/SHH/SLIT2/SOX9/SYT11/TWIST1/TYRO3	28	0.574948665297741
GO:0001570	BP	GO:0001570	vasculogenesis	8/666	83/18614	0.00962306101971902	0.0284923764529632	0.0163354054215522	APELA/CAV1/RAP1A/SHH/TBX20/VEGFA/WNT7A/WNT7B	8	0.963855421686747
GO:0098659	BP	GO:0098659	inorganic cation import across plasma membrane	11/666	136/18614	0.00972963595012835	0.0286918688508304	0.016449779496423	ATP1A2/CACNA1A/CACNA1C/CACNA1D/KCNJ14/KCNJ5/KCNJ9/KCNQ1/P2RX1/PRNP/SLC6A1	11	0.808823529411765

	ONTOLOGY	ID	Description	GeneRatio	BgRatio	pvalue	p.adjust	qvalue	geneID	Count	richFactor
GO:0099587	BP	GO:0099587	inorganic ion import across plasma membrane	11/666	136/18614	0.00972963595012835	0.0286918688508304	0.016449779496423	ATP1A2/CACNA1A/CACNA1C/CACNA1D/KCNJ14/KCNJ5/KCNJ9/KCNQ1/P2RX1/PRNP/SLC6A1	11	0.808823529411765
GO:0002053	BP	GO:0002053	positive regulation of mesenchymal cell proliferation	4/666	24/18614	0.00975706373981791	0.0286918688508304	0.016449779496423	SHH/SOX9/WNT2/WNT5A	4	1.66666666666667
GO:0040037	BP	GO:0040037	negative regulation of fibroblast growth factor receptor signaling pathway	4/666	24/18614	0.00975706373981791	0.0286918688508304	0.016449779496423	APLN/NGFR/SULF1/WNT5A	4	1.66666666666667
GO:0048011	BP	GO:0048011	neurotrophin TRK receptor signaling pathway	4/666	24/18614	0.00975706373981791	0.0286918688508304	0.016449779496423	AGT/DDIT4/NDN/TMEM108	4	1.66666666666667
GO:0060907	BP	GO:0060907	positive regulation of macrophage cytokine production	4/666	24/18614	0.00975706373981791	0.0286918688508304	0.016449779496423	P2RX7/PLCG2/RIPK2/WNT5A	4	1.66666666666667
GO:1901863	BP	GO:1901863	positive regulation of muscle tissue development	4/666	24/18614	0.00975706373981791	0.0286918688508304	0.016449779496423	BCL2/MEF2C/SHH/WNT3A	4	1.66666666666667
GO:2001026	BP	GO:2001026	regulation of endothelial cell chemotaxis	4/666	24/18614	0.00975706373981791	0.0286918688508304	0.016449779496423	CXCL13/FGFR1/P2RX4/VEGFA	4	1.66666666666667
GO:0003230	BP	GO:0003230	cardiac atrium development	5/666	37/18614	0.00976048926115737	0.0286918688508304	0.016449779496423	PROX1/TBX20/WNT11/WNT2/WNT5A	5	1.35135135135135
GO:0014742	BP	GO:0014742	positive regulation of muscle hypertrophy	5/666	37/18614	0.00976048926115737	0.0286918688508304	0.016449779496423	ADRA1A/AGT/CAMK2D/IGF1/IL6ST	5	1.35135135135135
GO:0031076	BP	GO:0031076	embryonic camera-type eye development	5/666	37/18614	0.00976048926115737	0.0286918688508304	0.016449779496423	PROX1/TH/TWIST1/WNT16/WNT5A	5	1.35135135135135
GO:0048854	BP	GO:0048854	brain morphogenesis	5/666	37/18614	0.00976048926115737	0.0286918688508304	0.016449779496423	AKT3/EMX1/FZD3/TUBA1A/WNT5A	5	1.35135135135135
GO:0038034	BP	GO:0038034	signal transduction in absence of ligand	7/666	67/18614	0.00986378072625571	0.0289608615708165	0.0166039998768701	BCL2/BCL2L1/FAS/FGFR1/GDNF/IL1A/INHBA	7	1.04477611940298
GO:0097192	BP	GO:0097192	extrinsic apoptotic signaling pathway in absence of ligand	7/666	67/18614	0.00986378072625571	0.0289608615708165	0.0166039998768701	BCL2/BCL2L1/FAS/FGFR1/GDNF/IL1A/INHBA	7	1.04477611940298
GO:0060041	BP	GO:0060041	retina development in camera-type eye	12/666	155/18614	0.00991822190634393	0.0289987576053169	0.0166257266390606	CALB1/GPM6A/HCN1/HPCA/LHX1/NEUROD1/PROX1/SLC17A7/SLC17A8/SLC1A1/SOX9/THRB	12	0.774193548387097
GO:0000101	BP	GO:0000101	sulfur amino acid transport	3/666	13/18614	0.00997108857639586	0.0289987576053169	0.0166257266390606	SLC1A1/SLC1A2/SLC7A11	3	2.30769230769231
GO:0003096	BP	GO:0003096	renal sodium ion transport	3/666	13/18614	0.00997108857639586	0.0289987576053169	0.0166257266390606	EDNRA/EDNRB/KCNQ1	3	2.30769230769231
GO:0021978	BP	GO:0021978	telencephalon regionalization	3/666	13/18614	0.00997108857639586	0.0289987576053169	0.0166257266390606	ADGRG1/EMX1/SHH	3	2.30769230769231
GO:0033079	BP	GO:0033079	immature T cell proliferation	3/666	13/18614	0.00997108857639586	0.0289987576053169	0.0166257266390606	IL1A/RIPK2/SHH	3	2.30769230769231
GO:0033080	BP	GO:0033080	immature T cell proliferation in thymus	3/666	13/18614	0.00997108857639586	0.0289987576053169	0.0166257266390606	IL1A/RIPK2/SHH	3	2.30769230769231
GO:0043568	BP	GO:0043568	positive regulation of insulin-like growth factor receptor signaling pathway	3/666	13/18614	0.00997108857639586	0.0289987576053169	0.0166257266390606	GHRHR/IGF1/WNT1	3	2.30769230769231
GO:0061000	BP	GO:0061000	negative regulation of dendritic spine development	3/666	13/18614	0.00997108857639586	0.0289987576053169	0.0166257266390606	FSTL4/GRIN3A/NLGN1	3	2.30769230769231
GO:0070234	BP	GO:0070234	positive regulation of T cell apoptotic process	3/666	13/18614	0.00997108857639586	0.0289987576053169	0.0166257266390606	CCL5/P2RX7/WNT5A	3	2.30769230769231
GO:0070493	BP	GO:0070493	thrombin-activated receptor signaling pathway	3/666	13/18614	0.00997108857639586	0.0289987576053169	0.0166257266390606	F2RL1/F2RL2/SNCA	3	2.30769230769231

	ONTOLOGY	ID	Description	GeneRatio	BgRatio	pvalue	p.adjust	qvalue	geneID	Count	richFactor
GO:0070571	BP	GO:0070571	negative regulation of neuron projection regeneration	3/666	13/18614	0.00997108857639586	0.0289987576053169	0.0166257266390606	EPHA4/RTN4R/TNR	3	2.30769230769231
GO:0071287	BP	GO:0071287	cellular response to manganese ion	3/666	13/18614	0.00997108857639586	0.0289987576053169	0.0166257266390606	LRRK2/PRKN/TH	3	2.30769230769231
GO:0071696	BP	GO:0071696	ectodermal placode development	3/666	13/18614	0.00997108857639586	0.0289987576053169	0.0166257266390606	DKK4/NRG3/PROX1	3	2.30769230769231
GO:0098693	BP	GO:0098693	regulation of synaptic vesicle cycle	3/666	13/18614	0.00997108857639586	0.0289987576053169	0.0166257266390606	LPAR1/PLD1/RAB3B	3	2.30769230769231
GO:0098935	BP	GO:0098935	dendritic transport	3/666	13/18614	0.00997108857639586	0.0289987576053169	0.0166257266390606	KIF5A/KIF5C/TRAK2	3	2.30769230769231
GO:1900452	BP	GO:1900452	regulation of long-term synaptic depression	3/666	13/18614	0.00997108857639586	0.0289987576053169	0.0166257266390606	GRID2/MAPT/SORCS3	3	2.30769230769231
GO:0003197	BP	GO:0003197	endocardial cushion development	6/666	52/18614	0.0103211340179701	0.0299459112740985	0.0171687539713576	APC/DKK1/ROBO2/SOX9/TBX20/TWIST1	6	1.15384615384615
GO:0010543	BP	GO:0010543	regulation of platelet activation	6/666	52/18614	0.0103211340179701	0.0299459112740985	0.0171687539713576	CTSG/F2/GNAQ/IL6ST/PDPN/PRKG1	6	1.15384615384615
GO:0101023	BP	GO:0101023	vascular endothelial cell proliferation	6/666	52/18614	0.0103211340179701	0.0299459112740985	0.0171687539713576	AKT3/APLN/FGFR1/ITGA4/MEF2C/PLCG1	6	1.15384615384615
GO:1905562	BP	GO:1905562	regulation of vascular endothelial cell proliferation	6/666	52/18614	0.0103211340179701	0.0299459112740985	0.0171687539713576	AKT3/APLN/FGFR1/ITGA4/MEF2C/PLCG1	6	1.15384615384615
GO:0018210	BP	GO:0018210	peptidyl-threonine modification	10/666	119/18614	0.0103853792757584	0.0300790448676207	0.0172450828528395	BCL2/CAMK2A/CAMK2D/CDK1/DDIT4/GCG/LRRK2/RIPK2/TTK/WNT5A	10	0.840336134453782
GO:0021782	BP	GO:0021782	glial cell development	10/666	119/18614	0.0103853792757584	0.0300790448676207	0.0172450828528395	ASCL1/CLU/CNTN2/CNTNAP1/GPM6B/LPAR1/MAG/MAPT/SHH/SOX10	10	0.840336134453782
GO:0045446	BP	GO:0045446	endothelial cell differentiation	10/666	119/18614	0.0103853792757584	0.0300790448676207	0.0172450828528395	CLDN1/EDNRA/EDNRB/F2RL1/PDPN/PROX1/RAP1A/S1PR1/VCL/VEGFA	10	0.840336134453782
GO:0007584	BP	GO:0007584	response to nutrient	12/666	156/18614	0.0104075182011584	0.0301076821416678	0.0172615013317438	A2M/ASCL1/BCHE/CCND1/CNR1/FKBP1B/GCGR/IL1A/LEP/MAP1B/PENK/SST	12	0.769230769230769
GO:2001236	BP	GO:2001236	regulation of extrinsic apoptotic signaling pathway	12/666	156/18614	0.0104075182011584	0.0301076821416678	0.0172615013317438	AGT/BCL2/BCL2L1/BID/CAV1/FGFR1/GDNF/GPER1/IGF1/IL1A/INHBA/SCG2	12	0.769230769230769
GO:0031334	BP	GO:0031334	positive regulation of protein-containing complex assembly	14/666	195/18614	0.0106146047912886	0.0306886956171668	0.0175946111617831	BID/CALY/CCR7/CLU/CXCL13/MAP1B/MAPT/MMP1/PINK1/PLCG2/PTK2B/SNCA/TENM1/VEGFA	14	0.717948717948718
GO:0052547	BP	GO:0052547	regulation of peptidase activity	25/666	425/18614	0.0106810030156467	0.0308625101833648	0.017694263481473	A2M/ATP2A3/BID/CAV1/EPHA4/FAS/GPER1/GRIN2A/GRIN2B/KNG1/MAGEA3/MAPT/MMP9/NDUFA13/NGF/NGFR/P2RX1/PINK1/RIPK2/SLC1A1/SLC22A2/SNCA/SOX2/VEGFA/WNT3A	25	0.588235294117647
GO:0071214	BP	GO:0071214	cellular response to abiotic stimulus	21/666	339/18614	0.0107569204587999	0.031045369380506	0.0177991013185446	AGT/ASCL1/ASIC1/ATP1A2/BCL2L1/BDKRB2/FAS/GNA11/GPR88/HPCA/ITGB3/MAG/MC1R/MMP1/MMP9/NSMF/PTGER4/SLC2A4/SLC38A3/SOX9/WNT11	21	0.619469026548673
GO:0104004	BP	GO:0104004	cellular response to environmental stimulus	21/666	339/18614	0.0107569204587999	0.031045369380506	0.0177991013185446	AGT/ASCL1/ASIC1/ATP1A2/BCL2L1/BDKRB2/FAS/GNA11/GPR88/HPCA/ITGB3/MAG/MC1R/MMP1/MMP9/NSMF/PTGER4/SLC2A4/SLC38A3/SOX9/WNT11	21	0.619469026548673

	ONTOLOGY	ID	Description	GeneRatio	BgRatio	pvalue	p.adjust	qvalue	geneID	Count	richFactor
GO:0008360	BP	GO:0008360	regulation of cell shape	11/666	138/18614	0.010789450383528	0.0311027264721644	0.0178319856006819	F2/GNA13/LPAR1/P2RY1/PDPN/PLXNA1/PLXNA2/PLXNA3/PTK2B/SEMA3E/VEGFA	11	0.797101449275362
GO:1904950	BP	GO:1904950	negative regulation of establishment of protein localization	11/666	138/18614	0.010789450383528	0.0311027264721644	0.0178319856006819	ADRA2A/DRD2/DRD4/F2RL1/FKBP1B/GNAZ/LRRK2/MAPT/OPRM1/PRKN/SYT4	11	0.797101449275362
GO:0014897	BP	GO:0014897	striated muscle hypertrophy	9/666	102/18614	0.0108697991746843	0.0313159806234311	0.0179542496394336	ADRA1A/AGT/CAMK2D/HTR2B/IGF1/IL6ST/LEP/P2RX4/RGS2	9	0.882352941176471
GO:0000578	BP	GO:0000578	embryonic axis specification	5/666	38/18614	0.0109164036549295	0.0313950403534104	0.0179995765971151	CHRDL1/LHX1/WNT1/WNT5A/WNT7A	5	1.31578947368421
GO:0033028	BP	GO:0033028	myeloid cell apoptotic process	5/666	38/18614	0.0109164036549295	0.0313950403534104	0.0179995765971151	BCL2/MEF2C/MIF/PIK3CD/SLC7A11	5	1.31578947368421
GO:0060306	BP	GO:0060306	regulation of membrane repolarization	5/666	38/18614	0.0109164036549295	0.0313950403534104	0.0179995765971151	CACNA1D/CAV1/KCNQ1/NEDD4L/SCN1B	5	1.31578947368421
GO:0042176	BP	GO:0042176	regulation of protein catabolic process	22/666	361/18614	0.0109734917479395	0.0315376381567627	0.0180813315512456	ADRA2A/APC/AURKA/AXIN2/CAV1/CLU/CSNK1E/DNAAF4/GABARAP/GRIN2A/HSP90AB1/LRRK2/MAD2L1/NDUFA13/PINK1/PRKCG/PRKN/SHH/SNCA/SOX9/VIP/WNT5A	22	0.609418282548476
GO:0051101	BP	GO:0051101	regulation of DNA binding	10/666	120/18614	0.0109788197123542	0.0315376381567627	0.0180813315512456	CPNE1/GZMA/HMGA2/IGF1/MMP9/NEUROD1/NGF/POU4F1/PRKN/TWIST1	10	0.833333333333333
GO:0003071	BP	GO:0003071	renal system process involved in regulation of systemic arterial blood pressure	4/666	25/18614	0.0112932776697867	0.0322236907007929	0.0184746629557258	AGT/AGTR1/EDNRB/F2RL1	4	1.6
GO:0003181	BP	GO:0003181	atrioventricular valve morphogenesis	4/666	25/18614	0.0112932776697867	0.0322236907007929	0.0184746629557258	AXIN2/OLFM1/SLIT3/TWIST1	4	1.6
GO:0010623	BP	GO:0010623	programmed cell death involved in cell development	4/666	25/18614	0.0112932776697867	0.0322236907007929	0.0184746629557258	BCL2/BCL2L1/IL1A/UNC5C	4	1.6
GO:0042659	BP	GO:0042659	regulation of cell fate specification	4/666	25/18614	0.0112932776697867	0.0322236907007929	0.0184746629557258	DKK1/WNT11/WNT3A/WNT5A	4	1.6
GO:0048714	BP	GO:0048714	positive regulation of oligodendrocyte differentiation	4/666	25/18614	0.0112932776697867	0.0322236907007929	0.0184746629557258	NKX6-1/PTN/SHH/TP73	4	1.6
GO:0070841	BP	GO:0070841	inclusion body assembly	4/666	25/18614	0.0112932776697867	0.0322236907007929	0.0184746629557258	CLU/MAPT/PRKN/SNCAIP	4	1.6
GO:1902993	BP	GO:1902993	positive regulation of amyloid precursor protein catabolic process	4/666	25/18614	0.0112932776697867	0.0322236907007929	0.0184746629557258	CHRNA7/CLU/CSNK1E/EPHA4	4	1.6
GO:2000679	BP	GO:2000679	positive regulation of transcription regulatory region DNA binding	4/666	25/18614	0.0112932776697867	0.0322236907007929	0.0184746629557258	IGF1/NEUROD1/POU4F1/TWIST1	4	1.6
GO:0033047	BP	GO:0033047	regulation of mitotic sister chromatid segregation	6/666	53/18614	0.0112963212772057	0.0322236907007929	0.0184746629557258	APC/BUB1B/CCNB1/DUSP1/MAD2L1/TTK	6	1.13207547169811
GO:0050873	BP	GO:0050873	brown fat cell differentiation	6/666	53/18614	0.0112963212772057	0.0322236907007929	0.0184746629557258	ADRB1/ADRB2/ADRB3/LEP/RGS2/SLC2A4	6	1.13207547169811
GO:0051155	BP	GO:0051155	positive regulation of striated muscle cell differentiation	6/666	53/18614	0.0112963212772057	0.0322236907007929	0.0184746629557258	BCL2/IGF1/MEF2C/PROX1/SHH/WNT3A	6	1.13207547169811
GO:0070169	BP	GO:0070169	positive regulation of biomineral tissue development	6/666	53/18614	0.0112963212772057	0.0322236907007929	0.0184746629557258	ADRB2/FZD9/GPM6B/MEF2C/P2RX7/PTN	6	1.13207547169811
GO:0050680	BP	GO:0050680	negative regulation of epithelial cell proliferation	13/666	177/18614	0.0113280732479891	0.0322768000080384	0.0185051118748233	CAV1/DRD2/KRT4/MEF2C/NGFR/PHOX2B/PRL/SCG2/SLURP1/SOX2/SOX9/SULF1/WNT5A	13	0.734463276836158

	ONTOLOGY	ID	Description	GeneRatio	BgRatio	pvalue	p.adjust	qvalue	geneID	Count	richFactor
GO:0060759	BP	GO:0060759	regulation of response to cytokine stimulus	13/666	177/18614	0.0113280732479891	0.0322768000080384	0.0185051118748233	AXL/CAV1/CCL5/CPNE1/CSF1/F2RL1/IRAK1/IRAK2/PRKN/RIPK2/SLIT2/SLIT3/WNT5A	13	0.734463276836158
GO:0009581	BP	GO:0009581	detection of external stimulus	11/666	139/18614	0.0113514401692506	0.032305922658869	0.0185218086326053	CALCA/CXCL12/GNA11/GNAQ/GNGT1/GPR88/GRIK2/KCNA1/KCNQ1/NGFR/NTSR1	11	0.79136690647482
GO:0046328	BP	GO:0046328	regulation of JNK cascade	11/666	139/18614	0.0113514401692506	0.032305922658869	0.0185218086326053	CCR7/F2RL1/IL1A/PINK1/PRKN/PTK2B/RIPK2/WNT16/WNT5A/WNT7A/WNT7B	11	0.79136690647482
GO:0010611	BP	GO:0010611	regulation of cardiac muscle hypertrophy	7/666	69/18614	0.0115152043494658	0.0327530262601992	0.018778144519688	ADRA1A/AGT/CAMK2D/IGF1/IL6ST/P2RX4/RGS2	7	1.01449275362319
GO:0033077	BP	GO:0033077	T cell differentiation in thymus	8/666	86/18614	0.011801851800838	0.0335489309433885	0.0192344569546396	BCL2/CAMK4/CCR7/IL1A/RIPK2/SHH/SOS2/WNT1	8	0.930232558139535
GO:0010769	BP	GO:0010769	regulation of cell morphogenesis involved in differentiation	5/666	39/18614	0.0121623374370522	0.0345337310820979	0.0197990679673987	CAMK2B/CNTN2/EPHA4/EPHB2/RELN	5	1.28205128205128
GO:0052652	BP	GO:0052652	cyclic purine nucleotide metabolic process	5/666	39/18614	0.0121623374370522	0.0345337310820979	0.0197990679673987	ADCY2/ADCY3/ADCY5/ADCY9/GUCY1A1	5	1.28205128205128
GO:0014896	BP	GO:0014896	muscle hypertrophy	9/666	104/18614	0.0122515332597327	0.034588741165629	0.0198306066499735	ADRA1A/AGT/CAMK2D/HTR2B/IGF1/IL6ST/LEP/P2RX4/RGS2	9	0.865384615384615
GO:0050773	BP	GO:0050773	regulation of dendrite development	9/666	104/18614	0.0122515332597327	0.034588741165629	0.0198306066499735	CAMK2B/CHRNA3/CHRNB2/EPHA4/EPHB2/NEDD4L/NSMF/PTN/RELN	9	0.865384615384615
GO:0030857	BP	GO:0030857	negative regulation of epithelial cell differentiation	6/666	54/18614	0.0123348023191773	0.034588741165629	0.0198306066499735	CAV1/CCND1/IL1A/MMP9/SOX9/VEGFA	6	1.11111111111111
GO:0043303	BP	GO:0043303	mast cell degranulation	6/666	54/18614	0.0123348023191773	0.034588741165629	0.0198306066499735	GRP/PIK3CD/PIK3CG/PTGDR/SLC18A2/STXBP1	6	1.11111111111111
GO:0071385	BP	GO:0071385	cellular response to glucocorticoid stimulus	6/666	54/18614	0.0123348023191773	0.034588741165629	0.0198306066499735	AXIN2/DDIT4/NR3C1/SSTR2/SSTR3/SSTR5	6	1.11111111111111
GO:0003207	BP	GO:0003207	cardiac chamber formation	3/666	14/18614	0.0123576458772827	0.034588741165629	0.0198306066499735	EDNRA/MEF2C/TBX20	3	2.14285714285714
GO:0007638	BP	GO:0007638	mechanosensory behavior	3/666	14/18614	0.0123576458772827	0.034588741165629	0.0198306066499735	DRD2/NRXN1/NRXN2	3	2.14285714285714
GO:0021859	BP	GO:0021859	pyramidal neuron differentiation	3/666	14/18614	0.0123576458772827	0.034588741165629	0.0198306066499735	FOXG1/PLXNA3/UNC5D	3	2.14285714285714
GO:0031115	BP	GO:0031115	negative regulation of microtubule polymerization	3/666	14/18614	0.0123576458772827	0.034588741165629	0.0198306066499735	MAP2/SNCA/TUBB4A	3	2.14285714285714
GO:0031269	BP	GO:0031269	pseudopodium assembly	3/666	14/18614	0.0123576458772827	0.034588741165629	0.0198306066499735	APC/CCR7/F2RL1	3	2.14285714285714
GO:0043950	BP	GO:0043950	positive regulation of cAMP-mediated signaling	3/666	14/18614	0.0123576458772827	0.034588741165629	0.0198306066499735	ADCYAP1R1/SCTR/UCN	3	2.14285714285714
GO:0048148	BP	GO:0048148	behavioral response to cocaine	3/666	14/18614	0.0123576458772827	0.034588741165629	0.0198306066499735	DRD2/DRD4/HOMER1	3	2.14285714285714
GO:0048711	BP	GO:0048711	positive regulation of astrocyte differentiation	3/666	14/18614	0.0123576458772827	0.034588741165629	0.0198306066499735	IL6ST/MAG/SHH	3	2.14285714285714
GO:0051608	BP	GO:0051608	histamine transport	3/666	14/18614	0.0123576458772827	0.034588741165629	0.0198306066499735	SLC18A2/SLC22A2/SLC22A3	3	2.14285714285714
GO:0055012	BP	GO:0055012	ventricular cardiac muscle cell differentiation	3/666	14/18614	0.0123576458772827	0.034588741165629	0.0198306066499735	CDK1/MEF2C/PROX1	3	2.14285714285714
GO:0070141	BP	GO:0070141	response to UV-A	3/666	14/18614	0.0123576458772827	0.034588741165629	0.0198306066499735	CCND1/MMP1/MMP9	3	2.14285714285714
GO:0070307	BP	GO:0070307	lens fiber cell development	3/666	14/18614	0.0123576458772827	0.034588741165629	0.0198306066499735	PROX1/WNT7A/WNT7B	3	2.14285714285714
GO:0070942	BP	GO:0070942	neutrophil mediated cytotoxicity	3/666	14/18614	0.0123576458772827	0.034588741165629	0.0198306066499735	CTSG/F2/F2RL1	3	2.14285714285714
GO:0071415	BP	GO:0071415	cellular response to purine-containing compound	3/666	14/18614	0.0123576458772827	0.034588741165629	0.0198306066499735	P2RY1/RYR1/RYR3	3	2.14285714285714

	ONTOLOGY	ID	Description	GeneRatio	BgRatio	pvalue	p.adjust	qvalue	geneID	Count	richFactor
GO:0071871	BP	GO:0071871	response to epinephrine	3/666	14/18614	0.0123576458772827	0.034588741165629	0.0198306066499735	KCNQ1/PENK/SNCA	3	2.14285714285714
GO:0098814	BP	GO:0098814	spontaneous synaptic transmission	3/666	14/18614	0.0123576458772827	0.034588741165629	0.0198306066499735	NSG1/PRKN/RPH3A	3	2.14285714285714
GO:0098840	BP	GO:0098840	protein transport along microtubule	3/666	14/18614	0.0123576458772827	0.034588741165629	0.0198306066499735	DLG2/KIF5A/KIF5C	3	2.14285714285714
GO:0099118	BP	GO:0099118	microtubule-based protein transport	3/666	14/18614	0.0123576458772827	0.034588741165629	0.0198306066499735	DLG2/KIF5A/KIF5C	3	2.14285714285714
GO:1902947	BP	GO:1902947	regulation of tau-protein kinase activity	3/666	14/18614	0.0123576458772827	0.034588741165629	0.0198306066499735	CLU/DKK1/HSP90AB1	3	2.14285714285714
GO:1903727	BP	GO:1903727	positive regulation of phospholipid metabolic process	3/666	14/18614	0.0123576458772827	0.034588741165629	0.0198306066499735	CAPN2/HTR2B/HTR2C	3	2.14285714285714
GO:0032722	BP	GO:0032722	positive regulation of chemokine production	7/666	70/18614	0.0124119190010566	0.0347011273550586	0.019895040516034	ADCYAP1/F2RL1/IL6R/MIF/RIPK2/TWIST1/WNT5A	7	1
GO:0046847	BP	GO:0046847	filopodium assembly	7/666	70/18614	0.0124119190010566	0.0347011273550586	0.019895040516034	ARHGAP44/CCR7/EPHB2/GAP43/GPM6A/NLGN1/TENM1	7	1
GO:0002088	BP	GO:0002088	lens development in camera-type eye	8/666	87/18614	0.0126029235992055	0.0352151048835104	0.0201897169295192	PROX1/SLC7A11/SOX1/WNT2/WNT2B/WNT5A/WNT7A/WNT7B	8	0.919540229885057
GO:0009416	BP	GO:0009416	response to light stimulus	20/666	323/18614	0.0126598594775376	0.035354096211419	0.0202694041993829	ATP1A2/BCL2/CASP7/CCND1/CHRNB2/DRD2/GNA11/GNAQ/GNGT1/GPR88/GRIN2A/MAPK10/MC1R/MMP1/MMP9/NPY/SLC1A2/SLC7A11/TH/TUBA1A	20	0.619195046439629
GO:0002407	BP	GO:0002407	dendritic cell chemotaxis	4/666	26/18614	0.0129768067316067	0.0359937951951733	0.0206361599266432	CCL5/CCR2/CCR7/PIK3CG	4	1.53846153846154
GO:0010882	BP	GO:0010882	regulation of cardiac muscle contraction by calcium ion signaling	4/666	26/18614	0.0129768067316067	0.0359937951951733	0.0206361599266432	ATP1A2/CACNA1C/CAMK2D/FKBP1B	4	1.53846153846154
GO:0021697	BP	GO:0021697	cerebellar cortex formation	4/666	26/18614	0.0129768067316067	0.0359937951951733	0.0206361599266432	GRID2/LHX1/PROX1/WNT7A	4	1.53846153846154
GO:0032469	BP	GO:0032469	endoplasmic reticulum calcium ion homeostasis	4/666	26/18614	0.0129768067316067	0.0359937951951733	0.0206361599266432	ATP2A1/BCL2/CAMK2D/ITPR1	4	1.53846153846154
GO:0033622	BP	GO:0033622	integrin activation	4/666	26/18614	0.0129768067316067	0.0359937951951733	0.0206361599266432	CXCL12/CXCL13/KIF14/PTGER4	4	1.53846153846154
GO:0048745	BP	GO:0048745	smooth muscle tissue development	4/666	26/18614	0.0129768067316067	0.0359937951951733	0.0206361599266432	COL3A1/PROX1/SHH/SOX9	4	1.53846153846154
GO:0050995	BP	GO:0050995	negative regulation of lipid catabolic process	4/666	26/18614	0.0129768067316067	0.0359937951951733	0.0206361599266432	ADRA2A/APOC1/CNR1/PIK3CG	4	1.53846153846154
GO:0060575	BP	GO:0060575	intestinal epithelial cell differentiation	4/666	26/18614	0.0129768067316067	0.0359937951951733	0.0206361599266432	IL6ST/NPY/PYY/SOX9	4	1.53846153846154
GO:0060740	BP	GO:0060740	prostate gland epithelium morphogenesis	4/666	26/18614	0.0129768067316067	0.0359937951951733	0.0206361599266432	SHH/SOX9/SULF1/WNT5A	4	1.53846153846154
GO:0062009	BP	GO:0062009	secondary palate development	4/666	26/18614	0.0129768067316067	0.0359937951951733	0.0206361599266432	WNT11/WNT3A/WNT5A/WNT7A	4	1.53846153846154
GO:1903306	BP	GO:1903306	negative regulation of regulated secretory pathway	4/666	26/18614	0.0129768067316067	0.0359937951951733	0.0206361599266432	ADRA2A/CCR2/RAP1A/SYT4	4	1.53846153846154
GO:1905523	BP	GO:1905523	positive regulation of macrophage migration	4/666	26/18614	0.0129768067316067	0.0359937951951733	0.0206361599266432	CCL5/CSF1/CX3CR1/P2RX4	4	1.53846153846154
GO:0003208	BP	GO:0003208	cardiac ventricle morphogenesis	7/666	71/18614	0.0133578465147226	0.0370297888436895	0.0212301214830127	EDNRA/MEF2C/NPY2R/NPY5R/POU4F1/PROX1/TBX20	7	0.985915492957746
GO:0061900	BP	GO:0061900	glial cell activation	6/666	55/18614	0.0134385538312834	0.037161461081154	0.0213056125318304	CLU/CX3CR1/LRRK2/MAPT/SNCA/SYT11	6	1.09090909090909
GO:0072348	BP	GO:0072348	sulfur compound transport	6/666	55/18614	0.0134385538312834	0.037161461081154	0.0213056125318304	SLC1A1/SLC1A2/SLC22A2/SLC44A4/SLC6A6/SLC7A11	6	1.09090909090909

	ONTOLOGY	ID	Description	GeneRatio	BgRatio	pvalue	p.adjust	qvalue	geneID	Count	richFactor
GO:1902991	BP	GO:1902991	regulation of amyloid precursor protein catabolic process	6/666	55/18614	0.0134385538312834	0.037161461081154	0.0213056125318304	CHRNA7/CLU/CSNK1E/EPHA4/IGF1/PRNP	6	1.09090909090909
GO:1903202	BP	GO:1903202	negative regulation of oxidative stress-induced cell death	6/666	55/18614	0.0134385538312834	0.037161461081154	0.0213056125318304	LRRK2/PINK1/PRKN/SLC7A11/TRAP1/WNT1	6	1.09090909090909
GO:0033273	BP	GO:0033273	response to vitamin	8/666	88/18614	0.0134431490950746	0.037161461081154	0.0213056125318304	ASCL1/BCHE/CCND1/FKBP1B/IL1A/LEP/MAP1B/PENK	8	0.909090909090909
GO:0000266	BP	GO:0000266	mitochondrial fission	5/666	40/18614	0.0135012594184326	0.0372174369274236	0.0213377049107915	AURKA/LRRK2/MAPT/PINK1/PRKN	5	1.25
GO:0009187	BP	GO:0009187	cyclic nucleotide metabolic process	5/666	40/18614	0.0135012594184326	0.0372174369274236	0.0213377049107915	ADCY2/ADCY3/ADCY5/ADCY9/GUCY1A1	5	1.25
GO:0035136	BP	GO:0035136	forelimb morphogenesis	5/666	40/18614	0.0135012594184326	0.0372174369274236	0.0213377049107915	CACNA1C/SHH/TWIST1/WNT3/WNT7A	5	1.25
GO:0071392	BP	GO:0071392	cellular response to estradiol stimulus	5/666	40/18614	0.0135012594184326	0.0372174369274236	0.0213377049107915	GPER1/POU4F1/SSTR1/SSTR2/SSTR3	5	1.25
GO:0099622	BP	GO:0099622	cardiac muscle cell membrane repolarization	5/666	40/18614	0.0135012594184326	0.0372174369274236	0.0213377049107915	CACNA1D/KCNJ5/KCNQ1/SCN1B/SCN2B	5	1.25
GO:0090068	BP	GO:0090068	positive regulation of cell cycle process	17/666	262/18614	0.01352232307474	0.0372546064531093	0.0213590151469666	AURKA/CCNB1/CCND1/CDK1/CXCR5/DRD2/EDN3/IGF1/IL1A/KIF14/MAD2L1/PHOX2B/PLCG2/RRM2/RXFP3/SSTR5/WNT5A	17	0.648854961832061
GO:0014743	BP	GO:0014743	regulation of muscle hypertrophy	7/666	72/18614	0.0143543699413216	0.0394805418363714	0.0226352006202208	ADRA1A/AGT/CAMK2D/IGF1/IL6ST/P2RX4/RGS2	7	0.972222222222222
GO:0051926	BP	GO:0051926	negative regulation of calcium ion transport	7/666	72/18614	0.0143543699413216	0.0394805418363714	0.0226352006202208	ADRA2A/ATP1A2/BCL2/DRD4/FKBP1B/NOS1/NTSR1	7	0.972222222222222
GO:1900046	BP	GO:1900046	regulation of hemostasis	7/666	72/18614	0.0143543699413216	0.0394805418363714	0.0226352006202208	CAV1/EPHB2/F2/F2RL1/KNG1/PRKG1/TPH1	7	0.972222222222222
GO:0072659	BP	GO:0072659	protein localization to plasma membrane	18/666	285/18614	0.0145127716435074	0.0398938884943172	0.0228721827915162	ANK3/ARHGAP44/BCL2L1/EPHA3/EPHB2/FGF13/GPER1/LRRC7/NSG1/P2RY1/PIK3R2/PRKG2/PRNP/RAB3B/RAP1A/STXBP1/VAMP2/WNT3A	18	0.631578947368421
GO:0030593	BP	GO:0030593	neutrophil chemotaxis	9/666	107/18614	0.0145638985229712	0.0400120521187274	0.0229399290082633	CCL5/CCR7/CXCL13/CXCL8/EDN2/EDN3/PIK3CD/PIK3CG/SLIT2	9	0.841121495327103
GO:0002279	BP	GO:0002279	mast cell activation involved in immune response	6/666	56/18614	0.0146095034054363	0.040070150244263	0.0229732381440043	GRP/PIK3CD/PIK3CG/PTGDR/SLC18A2/STXBP1	6	1.07142857142857
GO:0034205	BP	GO:0034205	amyloid-beta formation	6/666	56/18614	0.0146095034054363	0.040070150244263	0.0229732381440043	CHRNA7/CLU/CSNK1E/EPHA4/IGF1/PRNP	6	1.07142857142857
GO:0045839	BP	GO:0045839	negative regulation of mitotic nuclear division	6/666	56/18614	0.0146095034054363	0.040070150244263	0.0229732381440043	APC/BUB1B/CCNB1/DUSP1/MAD2L1/TTK	6	1.07142857142857
GO:0007205	BP	GO:0007205	protein kinase C-activating G protein-coupled receptor signaling pathway	4/666	27/18614	0.0148121481183284	0.0405354721612384	0.0232400190556589	BDKRB1/GAP43/HCRT/HTR2B	4	1.48148148148148
GO:0062149	BP	GO:0062149	detection of stimulus involved in sensory perception of pain	4/666	27/18614	0.0148121481183284	0.0405354721612384	0.0232400190556589	CALCA/CXCL12/KCNA1/NTSR1	4	1.48148148148148
GO:0090200	BP	GO:0090200	positive regulation of release of cytochrome c from mitochondria	4/666	27/18614	0.0148121481183284	0.0405354721612384	0.0232400190556589	BID/GPER1/MMP9/PINK1	4	1.48148148148148
GO:0099560	BP	GO:0099560	synaptic membrane adhesion	4/666	27/18614	0.0148121481183284	0.0405354721612384	0.0232400190556589	EFNA5/MDGA1/NTNG1/SLITRK3	4	1.48148148148148
GO:0003203	BP	GO:0003203	endocardial cushion morphogenesis	5/666	41/18614	0.014936007480387	0.0405567447748013	0.0232522151870521	APC/ROBO2/SOX9/TBX20/TWIST1	5	1.21951219512195

	ONTOLOGY	ID	Description	GeneRatio	BgRatio	pvalue	p.adjust	qvalue	geneID	Count	richFactor
GO:0150077	BP	GO:0150077	regulation of neuroinflammatory response	5/666	41/18614	0.014936007480387	0.0405567447748013	0.0232522151870521	IGF1/LRRK2/MMP9/PLCG2/SYT11	5	1.21951219512195
GO:1990000	BP	GO:1990000	amyloid fibril formation	5/666	41/18614	0.014936007480387	0.0405567447748013	0.0232522151870521	CHRNA7/CLU/MAPT/PRKN/SNCA	5	1.21951219512195
GO:0003214	BP	GO:0003214	cardiac left ventricle morphogenesis	3/666	15/18614	0.0150427153050789	0.0405567447748013	0.0232522151870521	EDNRA/NPY2R/NPY5R	3	2
GO:0003376	BP	GO:0003376	sphingosine-1-phosphate receptor signaling pathway	3/666	15/18614	0.0150427153050789	0.0405567447748013	0.0232522151870521	PIK3CG/S1PR1/S1PR5	3	2
GO:0007567	BP	GO:0007567	parturition	3/666	15/18614	0.0150427153050789	0.0405567447748013	0.0232522151870521	OXTR/PLA2G4B/PTGFR	3	2
GO:0010649	BP	GO:0010649	regulation of cell communication by electrical coupling	3/666	15/18614	0.0150427153050789	0.0405567447748013	0.0232522151870521	ANK3/CAMK2D/CAV1	3	2
GO:0015816	BP	GO:0015816	glycine transport	3/666	15/18614	0.0150427153050789	0.0405567447748013	0.0232522151870521	RGS2/SLC38A5/SLC6A9	3	2
GO:0031268	BP	GO:0031268	pseudopodium organization	3/666	15/18614	0.0150427153050789	0.0405567447748013	0.0232522151870521	APC/CCR7/F2RL1	3	2
GO:0038166	BP	GO:0038166	angiotensin-activated signaling pathway	3/666	15/18614	0.0150427153050789	0.0405567447748013	0.0232522151870521	AGTR1/CAMK2A/CAV1	3	2
GO:0042074	BP	GO:0042074	cell migration involved in gastrulation	3/666	15/18614	0.0150427153050789	0.0405567447748013	0.0232522151870521	APELA/WNT11/WNT5A	3	2
GO:0043455	BP	GO:0043455	regulation of secondary metabolic process	3/666	15/18614	0.0150427153050789	0.0405567447748013	0.0232522151870521	ASIP/SLC7A11/WNT5A	3	2
GO:0043584	BP	GO:0043584	nose development	3/666	15/18614	0.0150427153050789	0.0405567447748013	0.0232522151870521	ASCL1/GNG8/PROX1	3	2
GO:0045838	BP	GO:0045838	positive regulation of membrane potential	3/666	15/18614	0.0150427153050789	0.0405567447748013	0.0232522151870521	ANK3/BID/UCN3	3	2
GO:0048021	BP	GO:0048021	regulation of melanin biosynthetic process	3/666	15/18614	0.0150427153050789	0.0405567447748013	0.0232522151870521	ASIP/SLC7A11/WNT5A	3	2
GO:0048934	BP	GO:0048934	peripheral nervous system neuron differentiation	3/666	15/18614	0.0150427153050789	0.0405567447748013	0.0232522151870521	ASCL1/NEFH/POU4F1	3	2
GO:0048935	BP	GO:0048935	peripheral nervous system neuron development	3/666	15/18614	0.0150427153050789	0.0405567447748013	0.0232522151870521	ASCL1/NEFH/POU4F1	3	2
GO:0051956	BP	GO:0051956	negative regulation of amino acid transport	3/666	15/18614	0.0150427153050789	0.0405567447748013	0.0232522151870521	LEP/NPY5R/RGS2	3	2
GO:0071380	BP	GO:0071380	cellular response to prostaglandin E stimulus	3/666	15/18614	0.0150427153050789	0.0405567447748013	0.0232522151870521	GNG2/P2RY4/PTGER4	3	2
GO:0072148	BP	GO:0072148	epithelial cell fate commitment	3/666	15/18614	0.0150427153050789	0.0405567447748013	0.0232522151870521	NEUROD1/PDPN/PROX1	3	2
GO:0090280	BP	GO:0090280	positive regulation of calcium ion import	3/666	15/18614	0.0150427153050789	0.0405567447748013	0.0232522151870521	CXCL12/GCG/UCN	3	2
GO:0097152	BP	GO:0097152	mesenchymal cell apoptotic process	3/666	15/18614	0.0150427153050789	0.0405567447748013	0.0232522151870521	EDNRA/SHH/SOX9	3	2
GO:0098883	BP	GO:0098883	synapse pruning	3/666	15/18614	0.0150427153050789	0.0405567447748013	0.0232522151870521	CX3CR1/DKK1/EPHA4	3	2
GO:1900376	BP	GO:1900376	regulation of secondary metabolite biosynthetic process	3/666	15/18614	0.0150427153050789	0.0405567447748013	0.0232522151870521	ASIP/SLC7A11/WNT5A	3	2
GO:1904889	BP	GO:1904889	regulation of excitatory synapse assembly	3/666	15/18614	0.0150427153050789	0.0405567447748013	0.0232522151870521	GRID2/PTK2B/WNT7A	3	2
GO:2000052	BP	GO:2000052	positive regulation of non-canonical Wnt signaling pathway	3/666	15/18614	0.0150427153050789	0.0405567447748013	0.0232522151870521	CSNK1E/DKK1/WNT5A	3	2
GO:2001028	BP	GO:2001028	positive regulation of endothelial cell chemotaxis	3/666	15/18614	0.0150427153050789	0.0405567447748013	0.0232522151870521	FGFR1/P2RX4/VEGFA	3	2

	ONTOLOGY	ID	Description	GeneRatio	BgRatio	pvalue	p.adjust	qvalue	geneID	Count	richFactor
GO:0051783	BP	GO:0051783	regulation of nuclear division	11/666	145/18614	0.0152059998889039	0.0409745007971287	0.0234917253593004	APC/AURKA/BUB1B/CCNB1/DUSP1/EDN3/IGF1/IL1A/MAD2L1/TTK/WNT5A	11	0.758620689655172
GO:0010657	BP	GO:0010657	muscle cell apoptotic process	8/666	90/18614	0.0152451964479525	0.0410351262550308	0.023526483479085	AGT/CAMK2A/CAMK2D/CAPN2/GNGT1/GRIA4/IGF1/PTK2B	8	0.888888888888889
GO:0051262	BP	GO:0051262	protein tetramerization	8/666	90/18614	0.0152451964479525	0.0410351262550308	0.023526483479085	GRIN2B/HCN1/HOMER1/RRM2/RYR1/RYR3/SNCA/TP73	8	0.888888888888889
GO:0043588	BP	GO:0043588	skin development	19/666	308/18614	0.0153784282854507	0.0413710864931528	0.0237190980440478	BCL2/CLDN1/COL3A1/DKK1/DKK4/FZD3/GAL/IL1A/INHBA/KRT4/KRT6B/NGFR/PALLD/RYR1/SHH/SOX9/TXNIP/WNT16/WNT5A	19	0.616883116883117
GO:0042246	BP	GO:0042246	tissue regeneration	7/666	73/18614	0.015402849272052	0.0414141160679079	0.0237437679956716	ERBB4/FZD9/GAP43/IGF1/PTN/SOX2/WNT7A	7	0.958904109589041
GO:0045936	BP	GO:0045936	negative regulation of phosphate metabolic process	25/666	439/18614	0.0154722733460415	0.0415780336226319	0.0238377461065022	AGT/APC/APOC1/BDKRB1/BDKRB2/CAV1/DDIT4/DKK1/DRD2/DUSP1/EPHB2/FKBP1B/GNAQ/HPCA/INHBA/LRRK2/MAPT/NTRK3/PRKN/PRNP/RGS2/SH3GL2/SLIT2/SNCA/UCHL1	25	0.569476082004556
GO:0001541	BP	GO:0001541	ovarian follicle development	6/666	57/18614	0.0158495253681421	0.0425220617818877	0.0243789815045486	BCL2/BCL2L1/CGA/INHBA/VEGFA/VGF	6	1.05263157894737
GO:0002448	BP	GO:0002448	mast cell mediated immunity	6/666	57/18614	0.0158495253681421	0.0425220617818877	0.0243789815045486	GRP/PIK3CD/PIK3CG/PTGDR/SLC18A2/STXBP1	6	1.05263157894737
GO:0050881	BP	GO:0050881	musculoskeletal movement	6/666	57/18614	0.0158495253681421	0.0425220617818877	0.0243789815045486	ASCL1/ATP2A1/CHRNA1/CHRND/HOMER1/ITPR1	6	1.05263157894737
GO:0010563	BP	GO:0010563	negative regulation of phosphorus metabolic process	25/666	440/18614	0.0158711328672649	0.0425568019872377	0.0243988989494749	AGT/APC/APOC1/BDKRB1/BDKRB2/CAV1/DDIT4/DKK1/DRD2/DUSP1/EPHB2/FKBP1B/GNAQ/HPCA/INHBA/LRRK2/MAPT/NTRK3/PRKN/PRNP/RGS2/SH3GL2/SLIT2/SNCA/UCHL1	25	0.568181818181818
GO:0030038	BP	GO:0030038	contractile actin filament bundle assembly	9/666	109/18614	0.0162747036644023	0.0435676299077002	0.0249784323527147	ARRB1/LIMK1/LPAR1/PIK3R2/PTGER4/PTK2B/S1PR1/TACR1/WNT11	9	0.825688073394495
GO:0043149	BP	GO:0043149	stress fiber assembly	9/666	109/18614	0.0162747036644023	0.0435676299077002	0.0249784323527147	ARRB1/LIMK1/LPAR1/PIK3R2/PTGER4/PTK2B/S1PR1/TACR1/WNT11	9	0.825688073394495
GO:1904035	BP	GO:1904035	regulation of epithelial cell apoptotic process	9/666	109/18614	0.0162747036644023	0.0435676299077002	0.0249784323527147	ATOH1/BCL2/GPER1/ITGA4/NDNF/NEUROD1/PLCG1/SCG2/WNT11	9	0.825688073394495
GO:0008544	BP	GO:0008544	epidermis development	22/666	375/18614	0.0163915676566223	0.0438565895657587	0.0251441002875271	ATOH1/BCL2/CALML5/CLDN1/DKK1/DKK4/FZD3/GAL/IL1A/INHBA/KCNQ1/KRT4/KRT6B/NGFR/NTF4/PALLD/SHH/SLC44A4/SOX9/TXNIP/WNT16/WNT5A	22	0.586666666666667
GO:0014912	BP	GO:0014912	negative regulation of smooth muscle cell migration	5/666	42/18614	0.0164692815860923	0.0439926733671978	0.0252221205983806	GNA13/MEF2C/PRKG1/SEMA6D/SLIT2	5	1.19047619047619
GO:0045124	BP	GO:0045124	regulation of bone resorption	5/666	42/18614	0.0164692815860923	0.0439926733671978	0.0252221205983806	CALCA/CARTPT/ITGB3/P2RX7/S1PR1	5	1.19047619047619
GO:0045429	BP	GO:0045429	positive regulation of nitric oxide biosynthetic process	5/666	42/18614	0.0164692815860923	0.0439926733671978	0.0252221205983806	AGT/CLU/HSP90AB1/P2RX4/PTK2B	5	1.19047619047619
GO:0002067	BP	GO:0002067	glandular epithelial cell differentiation	7/666	74/18614	0.0165046190808138	0.0440391980359391	0.0252487943764435	ASCL1/CAV1/NEUROD1/NKX6-1/PROX1/SOX9/WNT5A	7	0.945945945945946
GO:0051145	BP	GO:0051145	smooth muscle cell differentiation	7/666	74/18614	0.0165046190808138	0.0440391980359391	0.0252487943764435	EDNRA/EDNRB/GPER1/MEF2C/SHH/SOX9/VEGFA	7	0.945945945945946

	ONTOLOGY	ID	Description	GeneRatio	BgRatio	pvalue	p.adjust	qvalue	geneID	Count	richFactor
GO:0032386	BP	GO:0032386	regulation of intracellular transport	20/666	332/18614	0.0166089955201169	0.0442936586985213	0.0253946831580964	ANK3/ARHGAP44/ATP2A1/CALY/CDK1/HPCA/LEP/LRRK2/MAP1B/MAP2/NEFH/PIK3R2/PINK1/PLA2G4E/PRKN/PRNP/SHH/SLC1A1/TENM1/VAMP2	20	0.602409638554217
GO:1990266	BP	GO:1990266	neutrophil migration	10/666	128/18614	0.0166937769631659	0.044495614844881	0.0255104697627556	CCL5/CCR7/CXCL13/CXCL8/EDN2/EDN3/IL1A/PIK3CD/PIK3CG/SLIT2	10	0.78125
GO:0045787	BP	GO:0045787	positive regulation of cell cycle	21/666	354/18614	0.016769603172063	0.0446183437626441	0.025580833378508	ASCL1/AURKA/CCNB1/CCND1/CDK1/CXCR5/DRD2/EDN3/FOXG1/IGF1/IL1A/KIF14/MAD2L1/MAGEA4/PHOX2B/PLCG2/PROX1/RRM2/RXFP3/SSTR5/WNT5A	21	0.593220338983051
GO:0010758	BP	GO:0010758	regulation of macrophage chemotaxis	4/666	28/18614	0.0168033681189531	0.0446183437626441	0.025580833378508	CCL5/CSF1/MIF/PTK2B	4	1.42857142857143
GO:0010971	BP	GO:0010971	positive regulation of G2/M transition of mitotic cell cycle	4/666	28/18614	0.0168033681189531	0.0446183437626441	0.025580833378508	CCNB1/CCND1/CDK1/PHOX2B	4	1.42857142857143
GO:0035116	BP	GO:0035116	embryonic hindlimb morphogenesis	4/666	28/18614	0.0168033681189531	0.0446183437626441	0.025580833378508	SHH/TWIST1/WNT3/WNT7A	4	1.42857142857143
GO:0042634	BP	GO:0042634	regulation of hair cycle	4/666	28/18614	0.0168033681189531	0.0446183437626441	0.025580833378508	DKK4/GAL/NGFR/WNT5A	4	1.42857142857143
GO:0060512	BP	GO:0060512	prostate gland morphogenesis	4/666	28/18614	0.0168033681189531	0.0446183437626441	0.025580833378508	SHH/SOX9/SULF1/WNT5A	4	1.42857142857143
GO:1903779	BP	GO:1903779	regulation of cardiac conduction	4/666	28/18614	0.0168033681189531	0.0446183437626441	0.025580833378508	AGT/ATP2A1/ATP2A3/TRPC1	4	1.42857142857143
GO:0034599	BP	GO:0034599	cellular response to oxidative stress	18/666	290/18614	0.0170286043301789	0.0451676148315324	0.0258957444780237	AXL/BCL2/CDK1/EDNRA/EPAS1/LRRK2/MAPT/MMP9/PENK/PINK1/PRKN/SIGMAR1/SLC1A1/SLC7A11/SNCA/TRAP1/WNT1/WNT16	18	0.620689655172414
GO:0044262	BP	GO:0044262	cellular carbohydrate metabolic process	18/666	290/18614	0.0170286043301789	0.0451676148315324	0.0258957444780237	ADCYAP1R1/AVPR1B/DDIT4/GCG/GCGR/GPER1/IGF1/IL6ST/LEP/LEPR/NR3C1/NTSR1/P2RY1/PPP1R1A/PRKN/PTH1R/PTK2B/SNCA	18	0.620689655172414
GO:1902904	BP	GO:1902904	negative regulation of supramolecular fiber organization	12/666	167/18614	0.0171060958319498	0.0453486844735886	0.0259995563175663	APC/CLU/FGF13/MAP1B/MAP2/PRKN/S1PR1/SLIT2/SNCA/SPTBN2/TMOD2/TUBB4A	12	0.718562874251497
GO:0010965	BP	GO:0010965	regulation of mitotic sister chromatid separation	6/666	58/18614	0.0171604368268008	0.0453948046306382	0.0260259981787693	APC/BUB1B/CCNB1/DUSP1/MAD2L1/TTK	6	1.03448275862069
GO:0048010	BP	GO:0048010	vascular endothelial growth factor receptor signaling pathway	6/666	58/18614	0.0171604368268008	0.0453948046306382	0.0260259981787693	AXL/ITGB3/PRKCB/PTK2B/SULF1/VEGFA	6	1.03448275862069
GO:0050879	BP	GO:0050879	multicellular organismal movement	6/666	58/18614	0.0171604368268008	0.0453948046306382	0.0260259981787693	ASCL1/ATP2A1/CHRNA1/CHRND/HOMER1/ITPR1	6	1.03448275862069
GO:0090311	BP	GO:0090311	regulation of protein deacetylation	6/666	58/18614	0.0171604368268008	0.0453948046306382	0.0260259981787693	CAMK2D/LRRK2/MAPT/NDN/PINK1/VEGFA	6	1.03448275862069
GO:0001656	BP	GO:0001656	metanephros development	8/666	92/18614	0.0172161469750144	0.0454687600119267	0.0260683986832848	BCL2/CALB1/GDNF/LHX1/ROBO2/SHH/SOX9/WNT7B	8	0.869565217391304
GO:0009791	BP	GO:0009791	post-embryonic development	8/666	92/18614	0.0172161469750144	0.0454687600119267	0.0260683986832848	BCL2/EMX1/GABRG2/ITPR1/LHX1/NDN/SLC18A2/VEGFA	8	0.869565217391304
GO:0055006	BP	GO:0055006	cardiac cell development	8/666	92/18614	0.0172161469750144	0.0454687600119267	0.0260683986832848	ADRA1A/AGT/CDK1/IGF1/INHBA/PROX1/RGS2/VEGFA	8	0.869565217391304

	ONTOLOGY	ID	Description	GeneRatio	BgRatio	pvalue	p.adjust	qvalue	geneID	Count	richFactor
GO:0002696	BP	GO:0002696	positive regulation of leukocyte activation	22/666	377/18614	0.0173131555408363	0.0457004078857199	0.0262012083118428	AXL/BCL2/CAV1/CCL5/CCR2/CCR7/CHRNB2/EPHB2/F2RL1/IGF1/IL1A/IL6ST/LEP/LRRK2/MEF2C/MIF/RIPK2/SHH/STXBP1/TACR1/WNT3A/WNT5A	22	0.583554376657825
GO:0007162	BP	GO:0007162	negative regulation of cell adhesion	19/666	312/18614	0.0173737338292804	0.0458356960659759	0.0262787724727992	CTSG/CXCL12/DUSP1/EFNA5/EPHA4/EPHB2/KNG1/NEXMIF/PLXNA1/PLXNA2/PLXNA3/PRKG1/PRNP/SEMA3E/SEMA6A/SHH/TNR/VEGFA/WNT1	19	0.608974358974359
GO:0048662	BP	GO:0048662	negative regulation of smooth muscle cell proliferation	7/666	75/18614	0.0176609862363556	0.0465685339869571	0.0266989271260018	APLN/GNA13/GPER1/MEF2C/PRKG1/VIP/VIPR2	7	0.933333333333333
GO:0006750	BP	GO:0006750	glutathione biosynthetic process	3/666	16/18614	0.0180304320923929	0.0472134116857277	0.0270686519425375	SLC1A1/SLC1A2/SLC7A11	3	1.875
GO:0021783	BP	GO:0021783	preganglionic parasympathetic fiber development	3/666	16/18614	0.0180304320923929	0.0472134116857277	0.0270686519425375	NRP2/PLXNA3/SEMA3F	3	1.875
GO:0034134	BP	GO:0034134	toll-like receptor 2 signaling pathway	3/666	16/18614	0.0180304320923929	0.0472134116857277	0.0270686519425375	F2RL1/IRAK1/RIPK2	3	1.875
GO:0044849	BP	GO:0044849	estrous cycle	3/666	16/18614	0.0180304320923929	0.0472134116857277	0.0270686519425375	OPRM1/OXTR/PTN	3	1.875
GO:0045779	BP	GO:0045779	negative regulation of bone resorption	3/666	16/18614	0.0180304320923929	0.0472134116857277	0.0270686519425375	CALCA/CARTPT/P2RX7	3	1.875
GO:0060033	BP	GO:0060033	anatomical structure regression	3/666	16/18614	0.0180304320923929	0.0472134116857277	0.0270686519425375	DRD2/SLC6A3/TH	3	1.875
GO:0060841	BP	GO:0060841	venous blood vessel development	3/666	16/18614	0.0180304320923929	0.0472134116857277	0.0270686519425375	PROX1/TBX20/VEGFA	3	1.875
GO:0070230	BP	GO:0070230	positive regulation of lymphocyte apoptotic process	3/666	16/18614	0.0180304320923929	0.0472134116857277	0.0270686519425375	CCL5/P2RX7/WNT5A	3	1.875
GO:0072044	BP	GO:0072044	collecting duct development	3/666	16/18614	0.0180304320923929	0.0472134116857277	0.0270686519425375	CALB1/SHH/WNT7B	3	1.875
GO:0072176	BP	GO:0072176	nephric duct development	3/666	16/18614	0.0180304320923929	0.0472134116857277	0.0270686519425375	EPHA4/LHX1/WNT11	3	1.875
GO:0097091	BP	GO:0097091	synaptic vesicle clustering	3/666	16/18614	0.0180304320923929	0.0472134116857277	0.0270686519425375	NLGN1/SYN2/SYN3	3	1.875
GO:0097401	BP	GO:0097401	synaptic vesicle lumen acidification	3/666	16/18614	0.0180304320923929	0.0472134116857277	0.0270686519425375	ATP6V0A4/ATP6V1F/ATP6V1G2	3	1.875
GO:2000696	BP	GO:2000696	regulation of epithelial cell differentiation involved in kidney development	3/666	16/18614	0.0180304320923929	0.0472134116857277	0.0270686519425375	GDNF/LHX1/MMP9	3	1.875
GO:0006904	BP	GO:0006904	vesicle docking involved in exocytosis	5/666	43/18614	0.0181036375567473	0.0473042948386034	0.0271207575698327	RAB3B/SNAP25/STX1A/STXBP1/UNC13C	5	1.16279069767442
GO:0010463	BP	GO:0010463	mesenchymal cell proliferation	5/666	43/18614	0.0181036375567473	0.0473042948386034	0.0271207575698327	SHH/SOX9/WNT11/WNT2/WNT5A	5	1.16279069767442
GO:0043507	BP	GO:0043507	positive regulation of JUN kinase activity	5/666	43/18614	0.0181036375567473	0.0473042948386034	0.0271207575698327	DKK1/EPHA4/FZD10/PTK2B/WNT5A	5	1.16279069767442
GO:0048713	BP	GO:0048713	regulation of oligodendrocyte differentiation	5/666	43/18614	0.0181036375567473	0.0473042948386034	0.0271207575698327	NKX6-1/PTN/SHH/SOX1/TP73	5	1.16279069767442
GO:2000060	BP	GO:2000060	positive regulation of ubiquitin-dependent protein catabolic process	9/666	111/18614	0.0181282711332381	0.0473434923591208	0.0271432305071192	AURKA/AXIN2/CAV1/CLU/CSNK1E/GABARAP/LRRK2/PRKN/PTK2B	9	0.810810810810811
GO:0001649	BP	GO:0001649	osteoblast differentiation	16/666	250/18614	0.0182587952077007	0.0476310431180821	0.0273080906841796	AXIN2/IGF1/IL6R/IL6ST/MAP2K6/MEF2C/PENK/PTH1R/SHH/SOX2/SOX9/TWIST1/WNT11/WNT3/WNT3A/WNT7B	16	0.64

	ONTOLOGY	ID	Description	GeneRatio	BgRatio	pvalue	p.adjust	qvalue	geneID	Count	richFactor
GO:0032436	BP	GO:0032436	positive regulation of proteasomal ubiquitin-dependent protein catabolic process	8/666	93/18614	0.018267449903883	0.0476310431180821	0.0273080906841796	AURKA/AXIN2/CAV1/CLU/CSNK1E/GABARAP/LRRK2/PRKN	8	0.860215053763441
GO:2001251	BP	GO:2001251	negative regulation of chromosome organization	8/666	93/18614	0.018267449903883	0.0476310431180821	0.0273080906841796	APC/BUB1B/CCNB1/DUSP1/MAD2L1/MCM2/TOP2A/TTK	8	0.860215053763441
GO:0045732	BP	GO:0045732	positive regulation of protein catabolic process	14/666	209/18614	0.0184630957815208	0.0481156499290428	0.0275859281169455	ADRA2A/APC/AURKA/AXIN2/CAV1/CLU/CSNK1E/GABARAP/LRRK2/NDUFA13/PRKN/SOX9/VIP/WNT5A	14	0.669856459330144
GO:0001961	BP	GO:0001961	positive regulation of cytokine-mediated signaling pathway	6/666	59/18614	0.0185439939378822	0.0482752808287559	0.0276774485792321	AXL/CPNE1/CSF1/PRKN/RIPK2/WNT5A	6	1.01694915254237
GO:2001258	BP	GO:2001258	negative regulation of cation channel activity	6/666	59/18614	0.0185439939378822	0.0482752808287559	0.0276774485792321	ANK3/CAV1/DRD4/FKBP1B/GRP/MMP9	6	1.01694915254237
GO:0071466	BP	GO:0071466	cellular response to xenobiotic stimulus	13/666	189/18614	0.0186474132607939	0.0485188121634738	0.0278170712987352	BCHE/CYP2C18/CYP2C8/ITGB3/KCNQ1/MEF2C/NOS1/PRNP/SLC28A2/SLC29A1/SOX10/TH/UCHL1	13	0.687830687830688
GO:0031669	BP	GO:0031669	cellular response to nutrient levels	15/666	230/18614	0.0187659855093546	0.0488014914171841	0.0279791385177067	BCL2/BECN2/CARTPT/COMT/FAS/GABARAP/GCGR/LEP/LRRK2/MAP1LC3C/PENK/SLC38A3/UCN2/UCN3/WNT2B	15	0.652173913043478
GO:0003209	BP	GO:0003209	cardiac atrium morphogenesis	4/666	29/18614	0.0189540999905844	0.0491347054080813	0.0281701785891596	PROX1/TBX20/WNT2/WNT5A	4	1.37931034482759
GO:0030878	BP	GO:0030878	thyroid gland development	4/666	29/18614	0.0189540999905844	0.0491347054080813	0.0281701785891596	CGA/EDNRA/NKX2-1/SHH	4	1.37931034482759
GO:0072530	BP	GO:0072530	purine-containing compound transmembrane transport	4/666	29/18614	0.0189540999905844	0.0491347054080813	0.0281701785891596	SLC22A2/SLC22A3/SLC28A2/SLC29A1	4	1.37931034482759
GO:0099625	BP	GO:0099625	ventricular cardiac muscle cell membrane repolarization	4/666	29/18614	0.0189540999905844	0.0491347054080813	0.0281701785891596	KCNJ5/KCNQ1/SCN1B/SCN2B	4	1.37931034482759
GO:1901017	BP	GO:1901017	negative regulation of potassium ion transmembrane transporter activity	4/666	29/18614	0.0189540999905844	0.0491347054080813	0.0281701785891596	ANK3/CAV1/GRP/NEDD4L	4	1.37931034482759
GO:1902430	BP	GO:1902430	negative regulation of amyloid-beta formation	4/666	29/18614	0.0189540999905844	0.0491347054080813	0.0281701785891596	CHRNA7/CLU/IGF1/PRNP	4	1.37931034482759
GO:0038127	BP	GO:0038127	ERBB signaling pathway	10/666	131/18614	0.0193226905795046	0.0500637976796338	0.0287028508622112	ADRA2A/AGT/ERBB4/GAB1/GPER1/MMP9/NRG3/PLCG1/PTK2B/SOX9	10	0.763358778625954
GO:1903573	BP	GO:1903573	negative regulation of response to endoplasmic reticulum stress	5/666	44/18614	0.0198414815949161	0.0513808651417348	0.0294579591978206	BCL2L1/CLU/LRRK2/MAGEA3/PRKN	5	1.13636363636364
GO:0060350	BP	GO:0060350	endochondral bone morphogenesis	6/666	60/18614	0.0200018884014543	0.0517689739300411	0.0296804718553772	AXIN2/COL3A1/GHR/MEF2C/MMP13/SOX9	6	1
GO:0032271	BP	GO:0032271	regulation of protein polymerization	13/666	191/18614	0.0201514783265806	0.0521286926184967	0.0298867077452944	CCR7/MAP1B/MAP2/MAPT/PIK3R2/PINK1/PTK2B/SLIT2/SNCA/SPTBN2/TENM1/TMOD2/TUBB4A	13	0.680628272251309
GO:1902905	BP	GO:1902905	positive regulation of supramolecular fiber organization	12/666	171/18614	0.0202111218600341	0.0522554781389098	0.0299593971147104	CCR7/CLU/F2RL1/LIMK1/LPAR1/MAP1B/MAPT/PROX1/PTK2B/TACR1/TENM1/WNT11	12	0.701754385964912
GO:0007041	BP	GO:0007041	lysosomal transport	10/666	132/18614	0.0202629208668258	0.0523618591274704	0.030020388046097	ADRB2/BECN2/CACNG4/CACNG5/CACNG7/CACNG8/CLU/LRRK2/PINK1/PRKN	10	0.757575757575758

	ONTOLOGY	ID	Description	GeneRatio	BgRatio	pvalue	p.adjust	qvalue	geneID	Count	richFactor
GO:0042326	BP	GO:0042326	negative regulation of phosphorylation	22/666	383/18614	0.0203237821939078	0.0524915341476913	0.0300947340393789	AGT/APC/BDKRB1/BDKRB2/CAV1/DDIT4/DKK1/DRD2/DUSP1/EPHB2/GNAQ/INHBA/LRRK2/MAPT/NTRK3/PRKN/PRNP/RGS2/SH3GL2/SLIT2/SNCA/UCHL1	22	0.574412532637076
GO:1904705	BP	GO:1904705	regulation of vascular associated smooth muscle cell proliferation	8/666	95/18614	0.0205065493208025	0.0529357615082692	0.0303494209043501	AGT/APLN/GNA13/GPER1/IGF1/MEF2C/MMP9/PRKG1	8	0.842105263157895
GO:0007173	BP	GO:0007173	epidermal growth factor receptor signaling pathway	9/666	114/18614	0.0211897737313607	0.0545852754783431	0.0312951292938961	ADRA2A/AGT/ERBB4/GAB1/GPER1/MMP9/PLCG1/PTK2B/SOX9	9	0.789473684210526
GO:0034698	BP	GO:0034698	response to gonadotropin	4/666	30/18614	0.0212675443578826	0.0545852754783431	0.0312951292938961	EDNRA/EFNA5/INHBA/NSMF	4	1.33333333333333
GO:0060603	BP	GO:0060603	mammary gland duct morphogenesis	4/666	30/18614	0.0212675443578826	0.0545852754783431	0.0312951292938961	CSF1/NR3C1/NTN1/WNT5A	4	1.33333333333333
GO:0090312	BP	GO:0090312	positive regulation of protein deacetylation	4/666	30/18614	0.0212675443578826	0.0545852754783431	0.0312951292938961	LRRK2/NDN/PINK1/VEGFA	4	1.33333333333333
GO:0090330	BP	GO:0090330	regulation of platelet aggregation	4/666	30/18614	0.0212675443578826	0.0545852754783431	0.0312951292938961	CTSG/IL6ST/PDPN/PRKG1	4	1.33333333333333
GO:0001991	BP	GO:0001991	regulation of systemic arterial blood pressure by circulatory renin-angiotensin	3/666	17/18614	0.0213232408786203	0.0545852754783431	0.0312951292938961	CTSG/EDNRB/F2RL1	3	1.76470588235294
GO:0019184	BP	GO:0019184	nonribosomal peptide biosynthetic process	3/666	17/18614	0.0213232408786203	0.0545852754783431	0.0312951292938961	SLC1A1/SLC1A2/SLC7A11	3	1.76470588235294
GO:0033623	BP	GO:0033623	regulation of integrin activation	3/666	17/18614	0.0213232408786203	0.0545852754783431	0.0312951292938961	CXCL13/KIF14/PTGER4	3	1.76470588235294
GO:0033631	BP	GO:0033631	cell-cell adhesion mediated by integrin	3/666	17/18614	0.0213232408786203	0.0545852754783431	0.0312951292938961	CCL5/CXCL13/ITGA4	3	1.76470588235294
GO:0042481	BP	GO:0042481	regulation of odontogenesis	3/666	17/18614	0.0213232408786203	0.0545852754783431	0.0312951292938961	CSF1/NGFR/SHH	3	1.76470588235294
GO:0048311	BP	GO:0048311	mitochondrion distribution	3/666	17/18614	0.0213232408786203	0.0545852754783431	0.0312951292938961	LRRK2/MAPT/TRAK2	3	1.76470588235294
GO:0061003	BP	GO:0061003	positive regulation of dendritic spine morphogenesis	3/666	17/18614	0.0213232408786203	0.0545852754783431	0.0312951292938961	CAMK2B/EPHB2/RELN	3	1.76470588235294
GO:0072189	BP	GO:0072189	ureter development	3/666	17/18614	0.0213232408786203	0.0545852754783431	0.0312951292938961	LHX1/SHH/SOX9	3	1.76470588235294
GO:0098915	BP	GO:0098915	membrane repolarization during ventricular cardiac muscle cell action potential	3/666	17/18614	0.0213232408786203	0.0545852754783431	0.0312951292938961	KCNJ5/KCNQ1/SCN2B	3	1.76470588235294
GO:0099638	BP	GO:0099638	endosome to plasma membrane protein transport	3/666	17/18614	0.0213232408786203	0.0545852754783431	0.0312951292938961	ARHGAP44/LRRC7/NSG1	3	1.76470588235294
GO:1901163	BP	GO:1901163	regulation of trophoblast cell migration	3/666	17/18614	0.0213232408786203	0.0545852754783431	0.0312951292938961	APELA/ITGB3/VEGFA	3	1.76470588235294
GO:0042098	BP	GO:0042098	T cell proliferation	14/666	213/18614	0.0213605501838501	0.0546523186640413	0.0313335668605533	BID/CCL5/CCR2/FKBP1B/IGF1/IL1A/IL6ST/LEP/P2RX7/PIK3CG/PRNP/RIPK2/SHH/SOS2	14	0.657276995305164
GO:0051258	BP	GO:0051258	protein polymerization	17/666	276/18614	0.0213799135933549	0.0546734002660453	0.0313456534801528	CCR7/FGF13/MAP1B/MAP2/MAPT/NEFL/PIK3R2/PINK1/PRKN/PTK2B/SLIT2/SNCA/SPTBN2/TENM1/TMOD2/TUBA1A/TUBB4A	17	0.615942028985507
GO:0001933	BP	GO:0001933	negative regulation of protein phosphorylation	20/666	341/18614	0.0214635829458919	0.0548474122052082	0.0314454189587926	AGT/APC/BDKRB1/BDKRB2/CAV1/DDIT4/DKK1/DRD2/DUSP1/EPHB2/GNAQ/LRRK2/NTRK3/PRKN/PRNP/RGS2/SH3GL2/SLIT2/SNCA/UCHL1	20	0.586510263929619

	ONTOLOGY	ID	Description	GeneRatio	BgRatio	pvalue	p.adjust	qvalue	geneID	Count	richFactor
GO:1903533	BP	GO:1903533	regulation of protein targeting	7/666	78/18614	0.0214702789588648	0.0548474122052082	0.0314454189587926	ANK3/HPCA/LRRK2/PINK1/PRKN/PRNP/SLC1A1	7	0.897435897435897
GO:0051306	BP	GO:0051306	mitotic sister chromatid separation	6/666	61/18614	0.0215357441854259	0.0549289998294595	0.0314921952226724	APC/BUB1B/CCNB1/DUSP1/MAD2L1/TTK	6	0.983606557377049
GO:0060038	BP	GO:0060038	cardiac muscle cell proliferation	6/666	61/18614	0.0215357441854259	0.0549289998294595	0.0314921952226724	CDK1/ERBB4/MEF2C/TBX20/TP73/WNT2	6	0.983606557377049
GO:1903409	BP	GO:1903409	reactive oxygen species biosynthetic process	6/666	61/18614	0.0215357441854259	0.0549289998294595	0.0314921952226724	MAOB/NOX4/PLCG2/SLC18A2/SNCA/TRAP1	6	0.983606557377049
GO:0014014	BP	GO:0014014	negative regulation of gliogenesis	5/666	45/18614	0.0216850655350088	0.0552236946467738	0.0316611512704211	ATOH1/F2/NKX6-1/NTRK3/SOX10	5	1.11111111111111
GO:1904706	BP	GO:1904706	negative regulation of vascular associated smooth muscle cell proliferation	5/666	45/18614	0.0216850655350088	0.0552236946467738	0.0316611512704211	APLN/GNA13/GPER1/MEF2C/PRKG1	5	1.11111111111111
GO:0007044	BP	GO:0007044	cell-substrate junction assembly	8/666	96/18614	0.0216962267269421	0.0552236946467738	0.0316611512704211	BCL2/EFNA5/EPHA3/GPM6B/ITGB3/PTK2B/VCL/VEGFA	8	0.833333333333333
GO:0043502	BP	GO:0043502	regulation of muscle adaptation	8/666	96/18614	0.0216962267269421	0.0552236946467738	0.0316611512704211	ADRA1A/AGT/CAMK2B/CAMK2D/IGF1/IL6ST/P2RX4/RGS2	8	0.833333333333333
GO:2000058	BP	GO:2000058	regulation of ubiquitin-dependent protein catabolic process	12/666	173/18614	0.0219123593141669	0.0557449513608334	0.0319600010264927	AURKA/AXIN2/CAV1/CLU/CSNK1E/GABARAP/HSP90AB1/LRRK2/PRKN/PTK2B/SHH/WNT1	12	0.69364161849711
GO:0045216	BP	GO:0045216	cell-cell junction organization	14/666	214/18614	0.0221355346744367	0.0562835762673857	0.0322688084098713	AGT/APC/CAV1/CDH4/CLDN1/CLDN19/CNTNAP1/EPHA4/EPHB2/F2RL1/NLGN4X/VCL/VEGFA/WNT11	14	0.654205607476635
GO:0051224	BP	GO:0051224	negative regulation of protein transport	10/666	134/18614	0.022242931320607	0.0565274081906844	0.0324086034644669	ADRA2A/DRD2/DRD4/F2RL1/FKBP1B/GNAZ/LRRK2/OPRM1/PRKN/SYT4	10	0.746268656716418
GO:0060348	BP	GO:0060348	bone development	15/666	235/18614	0.0222847304548284	0.0566043670209208	0.0324527259227938	AXIN2/COL3A1/GHR/IGF1/LEP/LEPR/MAP2K6/MEF2C/MMP13/PTGER4/RYR1/SOX9/SULF1/TWIST1/WNT1	15	0.638297872340425
GO:0061136	BP	GO:0061136	regulation of proteasomal protein catabolic process	13/666	194/18614	0.0225756886279729	0.0573137962843425	0.0328594597961524	AURKA/AXIN2/CAV1/CLU/CSNK1E/DNAAF4/GABARAP/HSP90AB1/LRRK2/PINK1/PRKCG/PRKN/SHH	13	0.670103092783505
GO:0022409	BP	GO:0022409	positive regulation of cell-cell adhesion	19/666	321/18614	0.0225977666212788	0.057340228675057	0.0328746141592606	ANK3/CAV1/CCL5/CCR2/CCR7/CTSG/CXCL13/IGF1/IL1A/IL6ST/IRAK1/ITGA4/LEP/PDPN/RIPK2/SHH/SOX2/WNT3A/WNT5A	19	0.59190031152648
GO:0035148	BP	GO:0035148	tube formation	11/666	154/18614	0.0227288254430216	0.057643022214887	0.0330482134109873	CELSR1/FZD3/GDNF/PIK3CD/PRKACB/PROX1/SHH/SOX9/TWIST1/VEGFA/WNT5A	11	0.714285714285714
GO:1990874	BP	GO:1990874	vascular associated smooth muscle cell proliferation	8/666	97/18614	0.022933876637852	0.0581330601727914	0.0333291646586845	AGT/APLN/GNA13/GPER1/IGF1/MEF2C/MMP9/PRKG1	8	0.824742268041237
GO:0043467	BP	GO:0043467	regulation of generation of precursor metabolites and energy	10/666	135/18614	0.0232839056315493	0.0589898949376623	0.0338204098619954	CCNB1/CDK1/COX7A1/DDIT4/GCGR/IGF1/P2RX7/PINK1/SNCA/TRAP1	10	0.740740740740741
GO:0032467	BP	GO:0032467	positive regulation of cytokinesis	5/666	46/18614	0.0236364827987188	0.0597002093235533	0.0342276511986191	CXCR5/DRD2/KIF14/RXFP3/SSTR5	5	1.08695652173913
GO:0034331	BP	GO:0034331	cell junction maintenance	5/666	46/18614	0.0236364827987188	0.0597002093235533	0.0342276511986191	CLDN1/CNTNAP1/F2RL1/ITGB3/NLGN1	5	1.08695652173913
GO:1901861	BP	GO:1901861	regulation of muscle tissue development	5/666	46/18614	0.0236364827987188	0.0597002093235533	0.0342276511986191	BCL2/MEF2C/SHH/TWIST1/WNT3A	5	1.08695652173913

	ONTOLOGY	ID	Description	GeneRatio	BgRatio	pvalue	p.adjust	qvalue	geneID	Count	richFactor
GO:0070507	BP	GO:0070507	regulation of microtubule cytoskeleton organization	11/666	155/18614	0.0237074132131845	0.0597002093235533	0.0342276511986191	APC/EFNA5/EPHA3/FGF13/GNAI1/MAP1B/MAP2/MAPT/SNCA/TUBB4A/WNT3A	11	0.709677419354839
GO:0003298	BP	GO:0003298	physiological muscle hypertrophy	4/666	31/18614	0.0237464718672526	0.0597002093235533	0.0342276511986191	ADRA1A/AGT/IGF1/RGS2	4	1.29032258064516
GO:0003301	BP	GO:0003301	physiological cardiac muscle hypertrophy	4/666	31/18614	0.0237464718672526	0.0597002093235533	0.0342276511986191	ADRA1A/AGT/IGF1/RGS2	4	1.29032258064516
GO:0010880	BP	GO:0010880	regulation of release of sequestered calcium ion into cytosol by sarcoplasmic reticulum	4/666	31/18614	0.0237464718672526	0.0597002093235533	0.0342276511986191	ATP1A2/CACNA1C/CAMK2D/FKBP1B	4	1.29032258064516
GO:0033137	BP	GO:0033137	negative regulation of peptidyl-serine phosphorylation	4/666	31/18614	0.0237464718672526	0.0597002093235533	0.0342276511986191	BDKRB2/CAV1/DDIT4/DKK1	4	1.29032258064516
GO:0035767	BP	GO:0035767	endothelial cell chemotaxis	4/666	31/18614	0.0237464718672526	0.0597002093235533	0.0342276511986191	CXCL13/FGFR1/P2RX4/VEGFA	4	1.29032258064516
GO:0051491	BP	GO:0051491	positive regulation of filopodium assembly	4/666	31/18614	0.0237464718672526	0.0597002093235533	0.0342276511986191	CCR7/GPM6A/NLGN1/TENM1	4	1.29032258064516
GO:0061049	BP	GO:0061049	cell growth involved in cardiac muscle cell development	4/666	31/18614	0.0237464718672526	0.0597002093235533	0.0342276511986191	ADRA1A/AGT/IGF1/RGS2	4	1.29032258064516
GO:0061311	BP	GO:0061311	cell surface receptor signaling pathway involved in heart development	4/666	31/18614	0.0237464718672526	0.0597002093235533	0.0342276511986191	DKK1/WNT11/WNT3A/WNT5A	4	1.29032258064516
GO:0086011	BP	GO:0086011	membrane repolarization during action potential	4/666	31/18614	0.0237464718672526	0.0597002093235533	0.0342276511986191	CAV1/KCNJ5/KCNQ1/SCN2B	4	1.29032258064516
GO:1902751	BP	GO:1902751	positive regulation of cell cycle G2/M phase transition	4/666	31/18614	0.0237464718672526	0.0597002093235533	0.0342276511986191	CCNB1/CCND1/CDK1/PHOX2B	4	1.29032258064516
GO:1903203	BP	GO:1903203	regulation of oxidative stress-induced neuron death	4/666	31/18614	0.0237464718672526	0.0597002093235533	0.0342276511986191	PINK1/PRKN/SLC7A11/WNT1	4	1.29032258064516
GO:0070227	BP	GO:0070227	lymphocyte apoptotic process	7/666	80/18614	0.0243053110445637	0.0610739283149442	0.0350152392994818	BCL2/CASP7/CCL5/FAS/MIF/P2RX7/WNT5A	7	0.875
GO:0007605	BP	GO:0007605	sensory perception of sound	11/666	156/18614	0.024716370765821	0.0619584526688832	0.0355223596497511	ATP6V0A4/CACNA1D/CHRNB2/DCDC2/KCNQ1/LHFPL4/P2RX2/SLC17A8/TH/THRB/UCN	11	0.705128205128205
GO:0010573	BP	GO:0010573	vascular endothelial growth factor production	6/666	63/18614	0.0248374788963632	0.0619584526688832	0.0355223596497511	ADGRG1/CCR2/IL1A/IL6R/IL6ST/SULF1	6	0.952380952380952
GO:0035904	BP	GO:0035904	aorta development	6/666	63/18614	0.0248374788963632	0.0619584526688832	0.0355223596497511	COL3A1/EDNRA/LEP/NGFR/PROX1/ROBO2	6	0.952380952380952
GO:0051784	BP	GO:0051784	negative regulation of nuclear division	6/666	63/18614	0.0248374788963632	0.0619584526688832	0.0355223596497511	APC/BUB1B/CCNB1/DUSP1/MAD2L1/TTK	6	0.952380952380952
GO:0008356	BP	GO:0008356	asymmetric cell division	3/666	18/18614	0.0249220469840045	0.0619584526688832	0.0355223596497511	ASPM/FGF13/SOX5	3	1.66666666666667
GO:0021513	BP	GO:0021513	spinal cord dorsal/ventral patterning	3/666	18/18614	0.0249220469840045	0.0619584526688832	0.0355223596497511	ASCL1/SHH/SOX1	3	1.66666666666667
GO:0032354	BP	GO:0032354	response to follicle-stimulating hormone	3/666	18/18614	0.0249220469840045	0.0619584526688832	0.0355223596497511	EDNRA/EFNA5/INHBA	3	1.66666666666667
GO:0035234	BP	GO:0035234	ectopic germ cell programmed cell death	3/666	18/18614	0.0249220469840045	0.0619584526688832	0.0355223596497511	BCL2L1/IL1A/UNC5C	3	1.66666666666667
GO:0042753	BP	GO:0042753	positive regulation of circadian rhythm	3/666	18/18614	0.0249220469840045	0.0619584526688832	0.0355223596497511	GHRHR/NKX2-1/NLGN1	3	1.66666666666667
GO:0043116	BP	GO:0043116	negative regulation of vascular permeability	3/666	18/18614	0.0249220469840045	0.0619584526688832	0.0355223596497511	SH3GL2/SLIT2/VEGFA	3	1.66666666666667
GO:0046851	BP	GO:0046851	negative regulation of bone remodeling	3/666	18/18614	0.0249220469840045	0.0619584526688832	0.0355223596497511	CALCA/CARTPT/P2RX7	3	1.66666666666667

	ONTOLOGY	ID	Description	GeneRatio	BgRatio	pvalue	p.adjust	qvalue	geneID	Count	richFactor
GO:0048339	BP	GO:0048339	paraxial mesoderm development	3/666	18/18614	0.0249220469840045	0.0619584526688832	0.0355223596497511	WNT11/WNT3A/WNT5A	3	1.66666666666667
GO:0050884	BP	GO:0050884	neuromuscular process controlling posture	3/666	18/18614	0.0249220469840045	0.0619584526688832	0.0355223596497511	CNTNAP1/POU4F1/SLURP1	3	1.66666666666667
GO:0050965	BP	GO:0050965	detection of temperature stimulus involved in sensory perception of pain	3/666	18/18614	0.0249220469840045	0.0619584526688832	0.0355223596497511	CALCA/CXCL12/NTSR1	3	1.66666666666667
GO:0060602	BP	GO:0060602	branch elongation of an epithelium	3/666	18/18614	0.0249220469840045	0.0619584526688832	0.0355223596497511	SHH/SOX9/WNT5A	3	1.66666666666667
GO:0061450	BP	GO:0061450	trophoblast cell migration	3/666	18/18614	0.0249220469840045	0.0619584526688832	0.0355223596497511	APELA/ITGB3/VEGFA	3	1.66666666666667
GO:0071071	BP	GO:0071071	regulation of phospholipid biosynthetic process	3/666	18/18614	0.0249220469840045	0.0619584526688832	0.0355223596497511	CAPN2/HTR2B/HTR2C	3	1.66666666666667
GO:0072077	BP	GO:0072077	renal vesicle morphogenesis	3/666	18/18614	0.0249220469840045	0.0619584526688832	0.0355223596497511	GDNF/LHX1/SOX9	3	1.66666666666667
GO:0086103	BP	GO:0086103	G protein-coupled receptor signaling pathway involved in heart process	3/666	18/18614	0.0249220469840045	0.0619584526688832	0.0355223596497511	CAV1/EDNRA/RGS2	3	1.66666666666667
GO:0090520	BP	GO:0090520	sphingolipid mediated signaling pathway	3/666	18/18614	0.0249220469840045	0.0619584526688832	0.0355223596497511	PIK3CG/S1PR1/S1PR5	3	1.66666666666667
GO:1905209	BP	GO:1905209	positive regulation of cardiocyte differentiation	3/666	18/18614	0.0249220469840045	0.0619584526688832	0.0355223596497511	GPER1/MEF2C/WNT3A	3	1.66666666666667
GO:0007160	BP	GO:0007160	cell-matrix adhesion	15/666	239/18614	0.0254404298326206	0.0632152237751921	0.0362428985482014	BCL2/CCR7/COL3A1/CSF1/EFNA5/EPHA3/GPM6B/ITGA4/ITGB3/L1CAM/NEXMIF/PTK2B/SEMA3E/VCL/VEGFA	15	0.627615062761506
GO:0003158	BP	GO:0003158	endothelium development	10/666	137/18614	0.0254707134178298	0.0632584923944585	0.0362677055501345	CLDN1/EDNRA/EDNRB/F2RL1/PDPN/PROX1/RAP1A/S1PR1/VCL/VEGFA	10	0.72992700729927
GO:0002861	BP	GO:0002861	regulation of inflammatory response to antigenic stimulus	5/666	47/18614	0.0256976650314615	0.0636934057638091	0.0365170524666096	CCR7/CNR1/NPY/NPY5R/PLCG1	5	1.06382978723404
GO:0031018	BP	GO:0031018	endocrine pancreas development	5/666	47/18614	0.0256976650314615	0.0636934057638091	0.0365170524666096	IL6R/NEUROD1/NKX6-1/SOX9/WNT5A	5	1.06382978723404
GO:0048806	BP	GO:0048806	genitalia development	5/666	47/18614	0.0256976650314615	0.0636934057638091	0.0365170524666096	AXL/LHX1/SHH/TYRO3/WNT5A	5	1.06382978723404
GO:1990573	BP	GO:1990573	potassium ion import across plasma membrane	5/666	47/18614	0.0256976650314615	0.0636934057638091	0.0365170524666096	ATP1A2/KCNJ14/KCNJ5/KCNJ9/KCNQ1	5	1.06382978723404
GO:0014855	BP	GO:0014855	striated muscle cell proliferation	7/666	81/18614	0.0258148862718055	0.0639194791062589	0.0366466660743064	CDK1/ERBB4/MEF2C/SHH/TBX20/TP73/WNT2	7	0.864197530864197
GO:0031016	BP	GO:0031016	pancreas development	7/666	81/18614	0.0258148862718055	0.0639194791062589	0.0366466660743064	IL6R/NEUROD1/NKX6-1/PROX1/SHH/SOX9/WNT5A	7	0.864197530864197
GO:0010743	BP	GO:0010743	regulation of macrophage derived foam cell differentiation	4/666	32/18614	0.0263932278438326	0.0651545529143332	0.0373547653588199	AGT/AGTR1/CSF1/ITGB3	4	1.25
GO:0051642	BP	GO:0051642	centrosome localization	4/666	32/18614	0.0263932278438326	0.0651545529143332	0.0373547653588199	ASPM/AURKA/MAD2L1/PLXNA2	4	1.25
GO:0061037	BP	GO:0061037	negative regulation of cartilage development	4/666	32/18614	0.0263932278438326	0.0651545529143332	0.0373547653588199	LEP/SOX9/WNT11/WNT9A	4	1.25
GO:0061842	BP	GO:0061842	microtubule organizing center localization	4/666	32/18614	0.0263932278438326	0.0651545529143332	0.0373547653588199	ASPM/AURKA/MAD2L1/PLXNA2	4	1.25
GO:0098868	BP	GO:0098868	bone growth	4/666	32/18614	0.0263932278438326	0.0651545529143332	0.0373547653588199	LEP/LEPR/MMP13/SOX9	4	1.25

	ONTOLOGY	ID	Description	GeneRatio	BgRatio	pvalue	p.adjust	qvalue	geneID	Count	richFactor
GO:1902235	BP	GO:1902235	regulation of endoplasmic reticulum stress-induced intrinsic apoptotic signaling pathway	4/666	32/18614	0.0263932278438326	0.0651545529143332	0.0373547653588199	BCL2L1/LRRK2/MAGEA3/PRKN	4	1.25
GO:0050867	BP	GO:0050867	positive regulation of cell activation	22/666	394/18614	0.0268843399661083	0.0663335998661758	0.0380307430190615	AXL/BCL2/CAV1/CCL5/CCR2/CCR7/CHRNB2/EPHB2/F2RL1/IGF1/IL1A/IL6ST/LEP/LRRK2/MEF2C/MIF/RIPK2/SHH/STXBP1/TACR1/WNT3A/WNT5A	22	0.558375634517767
GO:0034446	BP	GO:0034446	substrate adhesion-dependent cell spreading	8/666	100/18614	0.0269434329822574	0.0664460477209208	0.0380952122394186	AXL/EFNA5/EPHB3/ITGA4/ITGB3/KIF14/PDPN/TYRO3	8	0.8
GO:0002718	BP	GO:0002718	regulation of cytokine production involved in immune response	9/666	119/18614	0.0270911340319836	0.0667767922603808	0.0382848365114396	AXL/CCR2/F2RL1/MIF/P2RX7/PLCG2/RIPK2/TWIST1/WNT5A	9	0.756302521008403
GO:0051149	BP	GO:0051149	positive regulation of muscle cell differentiation	7/666	82/18614	0.0273872555277628	0.0674728626160172	0.0386839113825728	BCL2/GPER1/IGF1/MEF2C/PROX1/SHH/WNT3A	7	0.853658536585366
GO:0043268	BP	GO:0043268	positive regulation of potassium ion transport	5/666	48/18614	0.0278703793943985	0.0685600173791135	0.0393072049095437	ADRA2A/EDN3/GAL/KCNA1/KCNQ1	5	1.04166666666667
GO:0044331	BP	GO:0044331	cell-cell adhesion mediated by cadherin	5/666	48/18614	0.0278703793943985	0.0685600173791135	0.0393072049095437	CDH4/NEXMIF/VEGFA/WNT3A/WNT5A	5	1.04166666666667
GO:0045747	BP	GO:0045747	positive regulation of Notch signaling pathway	5/666	48/18614	0.0278703793943985	0.0685600173791135	0.0393072049095437	ASCL1/CNTN6/IL6ST/ROBO2/WNT1	5	1.04166666666667
GO:0030888	BP	GO:0030888	regulation of B cell proliferation	6/666	65/18614	0.0284607253449103	0.0699072788956693	0.0400796242659328	BCL2/CHRNB2/EPHB2/MEF2C/MIF/WNT3A	6	0.923076923076923
GO:0031663	BP	GO:0031663	lipopolysaccharide-mediated signaling pathway	6/666	65/18614	0.0284607253449103	0.0699072788956693	0.0400796242659328	CCL5/IRAK1/IRAK2/MIF/PLCG2/RIPK2	6	0.923076923076923
GO:0050435	BP	GO:0050435	amyloid-beta metabolic process	6/666	65/18614	0.0284607253449103	0.0699072788956693	0.0400796242659328	CHRNA7/CLU/CSNK1E/EPHA4/IGF1/PRNP	6	0.923076923076923
GO:0003184	BP	GO:0003184	pulmonary valve morphogenesis	3/666	19/18614	0.0288263577833074	0.0702088942046363	0.0402525480078838	ROBO2/SLIT2/TBX20	3	1.57894736842105
GO:0010744	BP	GO:0010744	positive regulation of macrophage derived foam cell differentiation	3/666	19/18614	0.0288263577833074	0.0702088942046363	0.0402525480078838	AGT/AGTR1/CSF1	3	1.57894736842105
GO:0021692	BP	GO:0021692	cerebellar Purkinje cell layer morphogenesis	3/666	19/18614	0.0288263577833074	0.0702088942046363	0.0402525480078838	KIF14/LHX1/SPTBN2	3	1.57894736842105
GO:0030903	BP	GO:0030903	notochord development	3/666	19/18614	0.0288263577833074	0.0702088942046363	0.0402525480078838	SOX9/WNT11/WNT5A	3	1.57894736842105
GO:0032328	BP	GO:0032328	alanine transport	3/666	19/18614	0.0288263577833074	0.0702088942046363	0.0402525480078838	SLC38A3/SLC38A5/SLC6A6	3	1.57894736842105
GO:0032930	BP	GO:0032930	positive regulation of superoxide anion generation	3/666	19/18614	0.0288263577833074	0.0702088942046363	0.0402525480078838	AGT/F2RL1/MAPT	3	1.57894736842105
GO:0036342	BP	GO:0036342	post-anal tail morphogenesis	3/666	19/18614	0.0288263577833074	0.0702088942046363	0.0402525480078838	FZD3/WNT3A/WNT5A	3	1.57894736842105
GO:0044070	BP	GO:0044070	regulation of monoatomic anion transport	3/666	19/18614	0.0288263577833074	0.0702088942046363	0.0402525480078838	GABRE/PRKG2/PRNP	3	1.57894736842105
GO:0045091	BP	GO:0045091	regulation of single stranded viral RNA replication via double stranded DNA intermediate	3/666	19/18614	0.0288263577833074	0.0702088942046363	0.0402525480078838	CXCL8/HMGA2/TOP2A	3	1.57894736842105
GO:0060438	BP	GO:0060438	trachea development	3/666	19/18614	0.0288263577833074	0.0702088942046363	0.0402525480078838	SHH/SOX9/WNT7B	3	1.57894736842105
GO:0061577	BP	GO:0061577	calcium ion transmembrane transport via high voltage-gated calcium channel	3/666	19/18614	0.0288263577833074	0.0702088942046363	0.0402525480078838	CACNA1C/CAMK2D/NOS1	3	1.57894736842105

	ONTOLOGY	ID	Description	GeneRatio	BgRatio	pvalue	p.adjust	qvalue	geneID	Count	richFactor
GO:0072087	BP	GO:0072087	renal vesicle development	3/666	19/18614	0.0288263577833074	0.0702088942046363	0.0402525480078838	GDNF/LHX1/SOX9	3	1.57894736842105
GO:0086014	BP	GO:0086014	atrial cardiac muscle cell action potential	3/666	19/18614	0.0288263577833074	0.0702088942046363	0.0402525480078838	CACNA1C/KCNJ5/KCNQ1	3	1.57894736842105
GO:0086026	BP	GO:0086026	atrial cardiac muscle cell to AV node cell signaling	3/666	19/18614	0.0288263577833074	0.0702088942046363	0.0402525480078838	CACNA1C/KCNJ5/KCNQ1	3	1.57894736842105
GO:0086066	BP	GO:0086066	atrial cardiac muscle cell to AV node cell communication	3/666	19/18614	0.0288263577833074	0.0702088942046363	0.0402525480078838	CACNA1C/KCNJ5/KCNQ1	3	1.57894736842105
GO:1902176	BP	GO:1902176	negative regulation of oxidative stress-induced intrinsic apoptotic signaling pathway	3/666	19/18614	0.0288263577833074	0.0702088942046363	0.0402525480078838	PINK1/PRKN/TRAP1	3	1.57894736842105
GO:1902307	BP	GO:1902307	positive regulation of sodium ion transmembrane transport	3/666	19/18614	0.0288263577833074	0.0702088942046363	0.0402525480078838	ANK3/DRD4/NOS1	3	1.57894736842105
GO:0046165	BP	GO:0046165	alcohol biosynthetic process	10/666	140/18614	0.0290215471602769	0.0706188935766661	0.0404876110948728	ADCYAP1R1/AVPR1B/GPER1/LEP/NTSR1/P2RY1/PLCG2/PTH1R/PTK2B/SNCA	10	0.714285714285714
GO:0072332	BP	GO:0072332	intrinsic apoptotic signaling pathway by p53 class mediator	7/666	83/18614	0.0290234313377142	0.0706188935766661	0.0404876110948728	BCL2/BDKRB2/DDIT4/MIF/POU4F1/PRKN/TP73	7	0.843373493975904
GO:0044409	BP	GO:0044409	entry into host	11/666	160/18614	0.0290654081643168	0.070686037173487	0.0405261062297852	AGTR1/AVPR1B/AXL/CAV1/CDK1/CLDN1/CXCL8/ITGB3/LY6E/NCAM1/TYRO3	11	0.6875
GO:0036336	BP	GO:0036336	dendritic cell migration	4/666	33/18614	0.029209738718669	0.0707221013804227	0.0405467827585568	CCL5/CCR2/CCR7/PIK3CG	4	1.21212121212121
GO:0036474	BP	GO:0036474	cell death in response to hydrogen peroxide	4/666	33/18614	0.029209738718669	0.0707221013804227	0.0405467827585568	LRRK2/PINK1/SIGMAR1/TRAP1	4	1.21212121212121
GO:0050901	BP	GO:0050901	leukocyte tethering or rolling	4/666	33/18614	0.029209738718669	0.0707221013804227	0.0405467827585568	CX3CR1/CXCL12/ITGA4/LEP	4	1.21212121212121
GO:0061036	BP	GO:0061036	positive regulation of cartilage development	4/666	33/18614	0.029209738718669	0.0707221013804227	0.0405467827585568	PRKG2/SOX5/SOX9/WNT5A	4	1.21212121212121
GO:0071711	BP	GO:0071711	basement membrane organization	4/666	33/18614	0.029209738718669	0.0707221013804227	0.0405467827585568	CAV1/COL3A1/FLRT2/MMP11	4	1.21212121212121
GO:0090279	BP	GO:0090279	regulation of calcium ion import	4/666	33/18614	0.029209738718669	0.0707221013804227	0.0405467827585568	CXCL12/GCG/HOMER1/UCN	4	1.21212121212121
GO:1901380	BP	GO:1901380	negative regulation of potassium ion transmembrane transport	4/666	33/18614	0.029209738718669	0.0707221013804227	0.0405467827585568	ANK3/CAV1/GRP/NEDD4L	4	1.21212121212121
GO:1902992	BP	GO:1902992	negative regulation of amyloid precursor protein catabolic process	4/666	33/18614	0.029209738718669	0.0707221013804227	0.0405467827585568	CHRNA7/CLU/IGF1/PRNP	4	1.21212121212121
GO:1905207	BP	GO:1905207	regulation of cardiocyte differentiation	4/666	33/18614	0.029209738718669	0.0707221013804227	0.0405467827585568	DKK1/GPER1/MEF2C/WNT3A	4	1.21212121212121
GO:0002367	BP	GO:0002367	cytokine production involved in immune response	9/666	121/18614	0.0297475312749284	0.0719887327505037	0.0412729747974725	AXL/CCR2/F2RL1/MIF/P2RX7/PLCG2/RIPK2/TWIST1/WNT5A	9	0.743801652892562
GO:0042773	BP	GO:0042773	ATP synthesis coupled electron transport	8/666	102/18614	0.0298719936049751	0.0721833080474201	0.0413845297730778	BID/CCNB1/CDK1/COX7A1/NDUFS7/PINK1/SDHD/SNCA	8	0.784313725490196
GO:0042775	BP	GO:0042775	mitochondrial ATP synthesis coupled electron transport	8/666	102/18614	0.0298719936049751	0.0721833080474201	0.0413845297730778	BID/CCNB1/CDK1/COX7A1/NDUFS7/PINK1/SDHD/SNCA	8	0.784313725490196
GO:1905477	BP	GO:1905477	positive regulation of protein localization to membrane	8/666	102/18614	0.0298719936049751	0.0721833080474201	0.0413845297730778	ANK3/EPHA3/EPHB2/GPER1/HPCA/PRKN/PRNP/WNT3A	8	0.784313725490196

	ONTOLOGY	ID	Description	GeneRatio	BgRatio	pvalue	p.adjust	qvalue	geneID	Count	richFactor
GO:0033628	BP	GO:0033628	regulation of cell adhesion mediated by integrin	5/666	49/18614	0.0301562264854675	0.072834325885048	0.0417577748876744	CCL5/CXCL13/ITGB3/NEXMIF/PIK3CG	5	1.02040816326531
GO:0032434	BP	GO:0032434	regulation of proteasomal ubiquitin-dependent protein catabolic process	10/666	141/18614	0.0302795095484415	0.0730961637674802	0.0419078932174428	AURKA/AXIN2/CAV1/CLU/CSNK1E/GABARAP/HSP90AB1/LRRK2/PRKN/SHH	10	0.709219858156028
GO:0001756	BP	GO:0001756	somitogenesis	6/666	66/18614	0.0303961766342539	0.0732698421566247	0.042007467463358	AXIN2/DKK1/LHX1/PLXNA2/WNT3A/WNT5A	6	0.909090909090909
GO:0060760	BP	GO:0060760	positive regulation of response to cytokine stimulus	6/666	66/18614	0.0303961766342539	0.0732698421566247	0.042007467463358	AXL/CPNE1/CSF1/PRKN/RIPK2/WNT5A	6	0.909090909090909
GO:2001259	BP	GO:2001259	positive regulation of cation channel activity	6/666	66/18614	0.0303961766342539	0.0732698421566247	0.042007467463358	ANK3/EDNRA/GAL/KCNA1/NTSR1/PLCG2	6	0.909090909090909
GO:0033157	BP	GO:0033157	regulation of intracellular protein transport	14/666	224/18614	0.0310853382458622	0.0748943320972611	0.0429388289392018	ANK3/ARHGAP44/CDK1/HPCA/LEP/LRRK2/PIK3R2/PINK1/PRKN/PRNP/SHH/SLC1A1/TENM1/VAMP2	14	0.625
GO:0008630	BP	GO:0008630	intrinsic apoptotic signaling pathway in response to DNA damage	8/666	103/18614	0.0314153006120187	0.0755781705864277	0.0433308909696133	BCL2/BCL2L1/BID/CLU/CXCL12/DDIT4/MIF/TP73	8	0.776699029126213
GO:0150115	BP	GO:0150115	cell-substrate junction organization	8/666	103/18614	0.0314153006120187	0.0755781705864277	0.0433308909696133	BCL2/EFNA5/EPHA3/GPM6B/ITGB3/PTK2B/VCL/VEGFA	8	0.776699029126213
GO:1903076	BP	GO:1903076	regulation of protein localization to plasma membrane	8/666	103/18614	0.0314153006120187	0.0755781705864277	0.0433308909696133	ARHGAP44/BCL2L1/EPHA3/EPHB2/GPER1/PIK3R2/PRNP/WNT3A	8	0.776699029126213
GO:0061025	BP	GO:0061025	membrane fusion	12/666	183/18614	0.0320396484785076	0.07704250111148	0.0441704289715547	CPLX1/GRIK5/RIMS1/SNAP25/SNCA/STX1A/STXBP1/SYT11/SYT4/SYT7/SYT9/VAMP2	12	0.655737704918033
GO:0050866	BP	GO:0050866	negative regulation of cell activation	14/666	225/18614	0.0321069916278929	0.0770451854219632	0.0441719679551524	AXL/CCR2/CNR1/CNR2/CTSG/F2/GAL/GPER1/INHBA/PRKG1/PRNP/SHH/SYT11/TYRO3	14	0.622222222222222
GO:0110053	BP	GO:0110053	regulation of actin filament organization	16/666	268/18614	0.0321425447313592	0.0770451854219632	0.0441719679551524	CCR7/CXCL12/F2RL1/LIMK1/LPAR1/PIK3R2/PRKN/PTGER4/PTK2B/S1PR1/SLIT2/SPTBN2/TACR1/TENM1/TMOD2/WNT11	16	0.597014925373134
GO:0001782	BP	GO:0001782	B cell homeostasis	4/666	34/18614	0.0321975200115386	0.0770451854219632	0.0441719679551524	BCL2/MEF2C/MIF/SOS2	4	1.17647058823529
GO:0010661	BP	GO:0010661	positive regulation of muscle cell apoptotic process	4/666	34/18614	0.0321975200115386	0.0770451854219632	0.0441719679551524	AGT/CAMK2A/CAMK2D/CAPN2	4	1.17647058823529
GO:0032743	BP	GO:0032743	positive regulation of interleukin-2 production	4/666	34/18614	0.0321975200115386	0.0770451854219632	0.0441719679551524	CCR2/IL1A/PLCG2/RIPK2	4	1.17647058823529
GO:0034405	BP	GO:0034405	response to fluid shear stress	4/666	34/18614	0.0321975200115386	0.0770451854219632	0.0441719679551524	MEF2C/P2RX4/P2RX7/PTK2B	4	1.17647058823529
GO:0046475	BP	GO:0046475	glycerophospholipid catabolic process	4/666	34/18614	0.0321975200115386	0.0770451854219632	0.0441719679551524	APOC1/PLA2G4B/PLA2G4D/PLA2G4E	4	1.17647058823529
GO:0060317	BP	GO:0060317	cardiac epithelial to mesenchymal transition	4/666	34/18614	0.0321975200115386	0.0770451854219632	0.0441719679551524	OLFM1/TWIST1/WNT16/WNT2	4	1.17647058823529
GO:0060351	BP	GO:0060351	cartilage development involved in endochondral bone morphogenesis	4/666	34/18614	0.0321975200115386	0.0770451854219632	0.0441719679551524	AXIN2/GHR/MMP13/SOX9	4	1.17647058823529
GO:0090313	BP	GO:0090313	regulation of protein targeting to membrane	4/666	34/18614	0.0321975200115386	0.0770451854219632	0.0441719679551524	ANK3/HPCA/PRNP/SLC1A1	4	1.17647058823529
GO:0010660	BP	GO:0010660	regulation of muscle cell apoptotic process	7/666	85/18614	0.0324910285685276	0.0776341300020967	0.0445096248895675	AGT/CAMK2A/CAMK2D/CAPN2/GRIA4/IGF1/PTK2B	7	0.823529411764706

	ONTOLOGY	ID	Description	GeneRatio	BgRatio	pvalue	p.adjust	qvalue	geneID	Count	richFactor
GO:0035050	BP	GO:0035050	embryonic heart tube development	7/666	85/18614	0.0324910285685276	0.0776341300020967	0.0445096248895675	APELA/EDNRA/MEF2C/SHH/TBX20/WNT3A/WNT5A	7	0.823529411764706
GO:0055013	BP	GO:0055013	cardiac muscle cell development	7/666	85/18614	0.0324910285685276	0.0776341300020967	0.0445096248895675	ADRA1A/AGT/CDK1/IGF1/PROX1/RGS2/VEGFA	7	0.823529411764706
GO:0038084	BP	GO:0038084	vascular endothelial growth factor signaling pathway	5/666	50/18614	0.0325566388619304	0.0777153375455987	0.044556183243469	GAB1/NRP2/PIK3CD/SEMA6A/VEGFA	5	1
GO:2000378	BP	GO:2000378	negative regulation of reactive oxygen species metabolic process	5/666	50/18614	0.0325566388619304	0.0777153375455987	0.044556183243469	BCL2/PINK1/PRKN/SLC18A2/TRAP1	5	1
GO:0110020	BP	GO:0110020	regulation of actomyosin structure organization	8/666	104/18614	0.0330122812247091	0.0785126443088403	0.0450132995266403	LIMK1/LPAR1/PIK3R2/PROX1/PTGER4/S1PR1/TACR1/WNT11	8	0.769230769230769
GO:0001502	BP	GO:0001502	cartilage condensation	3/666	20/18614	0.0330344147366596	0.0785126443088403	0.0450132995266403	SOX5/SOX9/WNT7A	3	1.5
GO:0008340	BP	GO:0008340	determination of adult lifespan	3/666	20/18614	0.0330344147366596	0.0785126443088403	0.0450132995266403	GHRHR/LEP/LRRK2	3	1.5
GO:0015810	BP	GO:0015810	aspartate transmembrane transport	3/666	20/18614	0.0330344147366596	0.0785126443088403	0.0450132995266403	NTSR1/SLC1A1/SLC1A2	3	1.5
GO:0039692	BP	GO:0039692	single stranded viral RNA replication via double stranded DNA intermediate	3/666	20/18614	0.0330344147366596	0.0785126443088403	0.0450132995266403	CXCL8/HMGA2/TOP2A	3	1.5
GO:0042693	BP	GO:0042693	muscle cell fate commitment	3/666	20/18614	0.0330344147366596	0.0785126443088403	0.0450132995266403	DKK1/MEF2C/WNT3A	3	1.5
GO:0055093	BP	GO:0055093	response to hyperoxia	3/666	20/18614	0.0330344147366596	0.0785126443088403	0.0450132995266403	CAV1/FAS/PDPN	3	1.5
GO:0072234	BP	GO:0072234	metanephric nephron tubule development	3/666	20/18614	0.0330344147366596	0.0785126443088403	0.0450132995266403	CALB1/SOX9/WNT7B	3	1.5
GO:0097284	BP	GO:0097284	hepatocyte apoptotic process	3/666	20/18614	0.0330344147366596	0.0785126443088403	0.0450132995266403	BCL2L1/BID/PIK3CG	3	1.5
GO:0051494	BP	GO:0051494	negative regulation of cytoskeleton organization	11/666	164/18614	0.033937486339452	0.0806199832568423	0.0462214906416591	APC/FGF13/MAP1B/MAP2/PRKN/S1PR1/SLIT2/SNCA/SPTBN2/TMOD2/TUBB4A	11	0.670731707317073
GO:0006970	BP	GO:0006970	response to osmotic stress	7/666	86/18614	0.034324246763133	0.081420691525482	0.0466805570945474	BDKRB2/CLDN1/OXT/PTK2B/SLC2A4/SST/TH	7	0.813953488372093
GO:0008543	BP	GO:0008543	fibroblast growth factor receptor signaling pathway	7/666	86/18614	0.034324246763133	0.081420691525482	0.0466805570945474	APLN/FGFR1/FLRT1/FLRT2/NGFR/SULF1/WNT5A	7	0.813953488372093
GO:0048041	BP	GO:0048041	focal adhesion assembly	7/666	86/18614	0.034324246763133	0.081420691525482	0.0466805570945474	BCL2/EFNA5/EPHA3/GPM6B/PTK2B/VCL/VEGFA	7	0.813953488372093
GO:0051701	BP	GO:0051701	biological process involved in interaction with host	13/666	206/18614	0.0344719792493358	0.0817316825906828	0.0468588561964465	AGTR1/AVPR1B/AXL/BCL2L1/CAV1/CDK1/CLDN1/CXCL8/HSP90AB1/ITGB3/LY6E/NCAM1/TYRO3	13	0.631067961165048
GO:0042987	BP	GO:0042987	amyloid precursor protein catabolic process	6/666	68/18614	0.0345205427874612	0.0817679362893357	0.046879641365648	CHRNA7/CLU/CSNK1E/EPHA4/IGF1/PRNP	6	0.882352941176471
GO:1904377	BP	GO:1904377	positive regulation of protein localization to cell periphery	6/666	68/18614	0.0345205427874612	0.0817679362893357	0.046879641365648	EPHA3/EPHB2/GNAI1/GPER1/PRNP/WNT3A	6	0.882352941176471
GO:0042102	BP	GO:0042102	positive regulation of T cell proliferation	8/666	105/18614	0.034663641025411	0.0820673389402192	0.04705129653434	CCL5/CCR2/IGF1/IL1A/IL6ST/LEP/RIPK2/SHH	8	0.761904761904762
GO:0015980	BP	GO:0015980	energy derivation by oxidation of organic compounds	19/666	337/18614	0.0347473969441134	0.0822260259895605	0.0471422758631587	ADRB3/BID/CCNB1/CDK1/COX7A1/GCGR/IGF1/IL6ST/LEP/LEPR/NDUFA11/NDUFA13/NDUFS7/PINK1/PPP1R1A/SDHD/SNCA/TRAP1/UCN	19	0.56379821958457

	ONTOLOGY	ID	Description	GeneRatio	BgRatio	pvalue	p.adjust	qvalue	geneID	Count	richFactor
GO:1903131	BP	GO:1903131	mononuclear cell differentiation	25/666	474/18614	0.0350010538316745	0.082786419433436	0.0474635637036587	AXL/BCL2/CAMK4/CCR2/CCR7/CSF1/F2RL1/FZD9/IL1A/IL6R/INHBA/ITGA4/LEP/LEPR/PIK3CD/PIK3R2/PLCG2/PTGER4/PTK2B/RIPK2/SHH/SOS2/TYRO3/VEGFA/WNT1	25	0.527426160337553
GO:0061178	BP	GO:0061178	regulation of insulin secretion involved in cellular response to glucose stimulus	5/666	51/18614	0.0350728801362887	0.0828765412835861	0.0475152328567029	ADCY5/ADRA2A/EFNA5/FKBP1B/GCG	5	0.980392156862745
GO:2001238	BP	GO:2001238	positive regulation of extrinsic apoptotic signaling pathway	5/666	51/18614	0.0350728801362887	0.0828765412835861	0.0475152328567029	AGT/BID/CAV1/GPER1/INHBA	5	0.980392156862745
GO:0007249	BP	GO:0007249	I-kappaB kinase/NF-kappaB signaling	17/666	293/18614	0.0351202225579398	0.0829485314138751	0.0475565066327448	CCR7/CPNE1/CX3CR1/EDNRA/EDNRB/F2RL1/HTR2B/IL1A/IRAK1/IRAK2/LPAR1/PINK1/PLCG2/PRKCB/PRKN/RIPK2/WNT5A	17	0.580204778156997
GO:0007034	BP	GO:0007034	vacuolar transport	11/666	165/18614	0.0352404951168244	0.0831926193559999	0.0476964484694569	ADRB2/ATP6V0D2/BECN2/CACNG4/CACNG5/CACNG7/CACNG8/CLU/LRRK2/PINK1/PRKN	11	0.666666666666667
GO:0035137	BP	GO:0035137	hindlimb morphogenesis	4/666	35/18614	0.0353576856717902	0.0833092162401002	0.0477632964340629	SHH/TWIST1/WNT3/WNT7A	4	1.14285714285714
GO:1901099	BP	GO:1901099	negative regulation of signal transduction in absence of ligand	4/666	35/18614	0.0353576856717902	0.0833092162401002	0.0477632964340629	BCL2/BCL2L1/GDNF/IL1A	4	1.14285714285714
GO:1902253	BP	GO:1902253	regulation of intrinsic apoptotic signaling pathway by p53 class mediator	4/666	35/18614	0.0353576856717902	0.0833092162401002	0.0477632964340629	BCL2/BDKRB2/MIF/PRKN	4	1.14285714285714
GO:2001240	BP	GO:2001240	negative regulation of extrinsic apoptotic signaling pathway in absence of ligand	4/666	35/18614	0.0353576856717902	0.0833092162401002	0.0477632964340629	BCL2/BCL2L1/GDNF/IL1A	4	1.14285714285714
GO:0046785	BP	GO:0046785	microtubule polymerization	7/666	87/18614	0.0362248625399766	0.0853115473809224	0.0489112838975701	FGF13/MAP1B/MAP2/MAPT/SNCA/TUBA1A/TUBB4A	7	0.804597701149425
GO:0030858	BP	GO:0030858	positive regulation of epithelial cell differentiation	6/666	69/18614	0.0367115274006036	0.0864162630143518	0.0495446455189634	ATOH1/GDNF/LHX1/NKX6-1/SOX9/TP73	6	0.869565217391304
GO:0035107	BP	GO:0035107	appendage morphogenesis	10/666	146/18614	0.0371500616306753	0.0873648578539565	0.0500884991112152	CACNA1C/COL3A1/DKK1/PLXNA2/SHH/SOX9/TWIST1/WNT3/WNT5A/WNT7A	10	0.684931506849315
GO:0035108	BP	GO:0035108	limb morphogenesis	10/666	146/18614	0.0371500616306753	0.0873648578539565	0.0500884991112152	CACNA1C/COL3A1/DKK1/PLXNA2/SHH/SOX9/TWIST1/WNT3/WNT5A/WNT7A	10	0.684931506849315
GO:0007398	BP	GO:0007398	ectoderm development	3/666	21/18614	0.0375433166064716	0.0875357690326413	0.0501864868448639	KRT6B/LHX1/SHH	3	1.42857142857143
GO:0010288	BP	GO:0010288	response to lead ion	3/666	21/18614	0.0375433166064716	0.0875357690326413	0.0501864868448639	CLDN1/MAPT/SLC6A1	3	1.42857142857143
GO:0030728	BP	GO:0030728	ovulation	3/666	21/18614	0.0375433166064716	0.0875357690326413	0.0501864868448639	INHBA/LEP/RGS2	3	1.42857142857143
GO:0032332	BP	GO:0032332	positive regulation of chondrocyte differentiation	3/666	21/18614	0.0375433166064716	0.0875357690326413	0.0501864868448639	PRKG2/SOX5/SOX9	3	1.42857142857143
GO:0033762	BP	GO:0033762	response to glucagon	3/666	21/18614	0.0375433166064716	0.0875357690326413	0.0501864868448639	CYSLTR2/GCGR/GLP2R	3	1.42857142857143
GO:0035020	BP	GO:0035020	regulation of Rac protein signal transduction	3/666	21/18614	0.0375433166064716	0.0875357690326413	0.0501864868448639	ARHGAP44/GABARAP/PIK3CG	3	1.42857142857143
GO:0035809	BP	GO:0035809	regulation of urine volume	3/666	21/18614	0.0375433166064716	0.0875357690326413	0.0501864868448639	DRD2/EDNRB/OXT	3	1.42857142857143
GO:0038083	BP	GO:0038083	peptidyl-tyrosine autophosphorylation	3/666	21/18614	0.0375433166064716	0.0875357690326413	0.0501864868448639	CAV1/PTK2B/VEGFA	3	1.42857142857143

	ONTOLOGY	ID	Description	GeneRatio	BgRatio	pvalue	p.adjust	qvalue	geneID	Count	richFactor
GO:0044321	BP	GO:0044321	response to leptin	3/666	21/18614	0.0375433166064716	0.0875357690326413	0.0501864868448639	CCND1/LEP/LEPR	3	1.42857142857143
GO:0090141	BP	GO:0090141	positive regulation of mitochondrial fission	3/666	21/18614	0.0375433166064716	0.0875357690326413	0.0501864868448639	AURKA/PINK1/PRKN	3	1.42857142857143
GO:0090231	BP	GO:0090231	regulation of spindle checkpoint	3/666	21/18614	0.0375433166064716	0.0875357690326413	0.0501864868448639	CCNB1/DUSP1/MAD2L1	3	1.42857142857143
GO:0090266	BP	GO:0090266	regulation of mitotic cell cycle spindle assembly checkpoint	3/666	21/18614	0.0375433166064716	0.0875357690326413	0.0501864868448639	CCNB1/DUSP1/MAD2L1	3	1.42857142857143
GO:0097062	BP	GO:0097062	dendritic spine maintenance	3/666	21/18614	0.0375433166064716	0.0875357690326413	0.0501864868448639	GRIN2B/HOMER1/PRNP	3	1.42857142857143
GO:1900118	BP	GO:1900118	negative regulation of execution phase of apoptosis	3/666	21/18614	0.0375433166064716	0.0875357690326413	0.0501864868448639	BCL2L1/FZD3/GCG	3	1.42857142857143
GO:1903206	BP	GO:1903206	negative regulation of hydrogen peroxide-induced cell death	3/666	21/18614	0.0375433166064716	0.0875357690326413	0.0501864868448639	LRRK2/PINK1/TRAP1	3	1.42857142857143
GO:1903504	BP	GO:1903504	regulation of mitotic spindle checkpoint	3/666	21/18614	0.0375433166064716	0.0875357690326413	0.0501864868448639	CCNB1/DUSP1/MAD2L1	3	1.42857142857143
GO:1903817	BP	GO:1903817	negative regulation of voltage-gated potassium channel activity	3/666	21/18614	0.0375433166064716	0.0875357690326413	0.0501864868448639	ANK3/CAV1/GRP	3	1.42857142857143
GO:2000738	BP	GO:2000738	positive regulation of stem cell differentiation	3/666	21/18614	0.0375433166064716	0.0875357690326413	0.0501864868448639	PTN/SOX5/SOX9	3	1.42857142857143
GO:0048599	BP	GO:0048599	oocyte development	5/666	52/18614	0.0377060446169609	0.0878734989532303	0.0503801160138775	AURKA/BCL2/EDNRA/IGF1/PTK2B	5	0.961538461538462
GO:0003279	BP	GO:0003279	cardiac septum development	8/666	107/18614	0.0381321691946654	0.088824460470038	0.050925326482474	NRP2/PROX1/ROBO2/SLIT2/SLIT3/TBX20/WNT11/WNT5A	8	0.747663551401869
GO:0071560	BP	GO:0071560	cellular response to transforming growth factor beta stimulus	16/666	274/18614	0.0381602922585971	0.0888478618905753	0.0509387431131488	CAV1/CLDN1/COL3A1/CX3CR1/GDF15/HSP90AB1/MEF2C/NKX2-1/NR3C1/PENK/SOX5/SOX9/WNT1/WNT2/WNT5A/WNT7A	16	0.583941605839416
GO:0031110	BP	GO:0031110	regulation of microtubule polymerization or depolymerization	7/666	88/18614	0.0381936525907706	0.0888834292062677	0.0509591347615096	APC/FGF13/MAP1B/MAP2/MAPT/SNCA/TUBB4A	7	0.795454545454545
GO:0050775	BP	GO:0050775	positive regulation of dendrite morphogenesis	4/666	36/18614	0.0386909585953792	0.089998136060714	0.0515982246044935	CAMK2B/EPHA4/EPHB2/RELN	4	1.11111111111111
GO:0030193	BP	GO:0030193	regulation of blood coagulation	6/666	70/18614	0.0389896156880612	0.0906499342984014	0.0519719171423292	CAV1/EPHB2/F2/F2RL1/KNG1/PRKG1	6	0.857142857142857
GO:0019058	BP	GO:0019058	viral life cycle	18/666	320/18614	0.0398518812891022	0.092610873066637	0.0530961733039326	AGTR1/AVPR1B/AXL/BCL2/CAV1/CCL5/CDK1/CLDN1/CXCL8/HMGA2/HSP90AB1/ITGB3/LY6E/NCAM1/PRKN/PROX1/TOP2A/TYRO3	18	0.5625
GO:0032231	BP	GO:0032231	regulation of actin filament bundle assembly	8/666	108/18614	0.0399505949285782	0.0927525621511393	0.0531774072663918	LIMK1/LPAR1/PIK3R2/PRKN/PTGER4/S1PR1/TACR1/WNT11	8	0.740740740740741
GO:0090398	BP	GO:0090398	cellular senescence	8/666	108/18614	0.0399505949285782	0.0927525621511393	0.0531774072663918	AKT3/HMGA2/MAP2K6/MAPK10/MIF/TWIST1/WNT1/WNT16	8	0.740740740740741
GO:0006119	BP	GO:0006119	oxidative phosphorylation	10/666	148/18614	0.0401781984790119	0.0932369431181981	0.0534551152170941	BID/CCNB1/CDK1/COX7A1/NDUFA11/NDUFA13/NDUFS7/PINK1/SDHD/SNCA	10	0.675675675675676
GO:0006112	BP	GO:0006112	energy reserve metabolic process	7/666	89/18614	0.040231343009841	0.0933162108982389	0.0535005614551771	ADRB3/GCGR/IGF1/IL6ST/LEP/LEPR/PPP1R1A	7	0.786516853932584
GO:0042113	BP	GO:0042113	B cell activation	16/666	276/18614	0.0403363806257713	0.0935157126300312	0.053614940881425	BCL2/CHRNA4/CHRNB2/CXCR5/EPHB2/FZD9/INHBA/ITGA4/MEF2C/MIF/PIK3CD/PIK3R2/PLCG2/PRKCB/PTK2B/WNT3A	16	0.579710144927536

	ONTOLOGY	ID	Description	GeneRatio	BgRatio	pvalue	p.adjust	qvalue	geneID	Count	richFactor
GO:0009948	BP	GO:0009948	anterior/posterior axis specification	5/666	53/18614	0.0404570574648521	0.0936629474516005	0.053699354356253	AURKA/LHX1/SHH/WNT3/WNT5A	5	0.943396226415094
GO:0010659	BP	GO:0010659	cardiac muscle cell apoptotic process	5/666	53/18614	0.0404570574648521	0.0936629474516005	0.053699354356253	AGT/CAMK2A/CAMK2D/CAPN2/GNGT1	5	0.943396226415094
GO:0071622	BP	GO:0071622	regulation of granulocyte chemotaxis	5/666	53/18614	0.0404570574648521	0.0936629474516005	0.053699354356253	CCL5/CCR7/CSF1/CXCL8/SLIT2	5	0.943396226415094
GO:0120034	BP	GO:0120034	positive regulation of plasma membrane bounded cell projection assembly	8/666	109/18614	0.0418259130485129	0.0967864230854243	0.0554901225249624	APC/CCR7/F2RL1/GPM6A/NLGN1/P2RX7/TENM1/WNT1	8	0.73394495412844
GO:0048706	BP	GO:0048706	embryonic skeletal system development	9/666	129/18614	0.0421881405053135	0.0974010273229777	0.0558424907947981	DSCAML1/EDNRA/LHX1/SHH/SULF1/TWIST1/WNT11/WNT5A/WNT9A	9	0.697674418604651
GO:0070306	BP	GO:0070306	lens fiber cell differentiation	4/666	37/18614	0.0421976821510424	0.0974010273229777	0.0558424907947981	PROX1/SLC7A11/WNT7A/WNT7B	4	1.08108108108108
GO:0009267	BP	GO:0009267	cellular response to starvation	11/666	170/18614	0.0422855488410442	0.0974010273229777	0.0558424907947981	BCL2/BECN2/CARTPT/COMT/FAS/GABARAP/GCGR/LRRK2/MAP1LC3C/SLC38A3/WNT2B	11	0.647058823529412
GO:0003228	BP	GO:0003228	atrial cardiac muscle tissue development	3/666	22/18614	0.0423491343619946	0.0974010273229777	0.0558424907947981	EDNRA/PROX1/WNT2	3	1.36363636363636
GO:0010042	BP	GO:0010042	response to manganese ion	3/666	22/18614	0.0423491343619946	0.0974010273229777	0.0558424907947981	LRRK2/PRKN/TH	3	1.36363636363636
GO:0032928	BP	GO:0032928	regulation of superoxide anion generation	3/666	22/18614	0.0423491343619946	0.0974010273229777	0.0558424907947981	AGT/F2RL1/MAPT	3	1.36363636363636
GO:0033630	BP	GO:0033630	positive regulation of cell adhesion mediated by integrin	3/666	22/18614	0.0423491343619946	0.0974010273229777	0.0558424907947981	CCL5/CXCL13/ITGB3	3	1.36363636363636
GO:0051444	BP	GO:0051444	negative regulation of ubiquitin-protein transferase activity	3/666	22/18614	0.0423491343619946	0.0974010273229777	0.0558424907947981	BUB1B/LIMK1/MAD2L1	3	1.36363636363636
GO:0061318	BP	GO:0061318	renal filtration cell differentiation	3/666	22/18614	0.0423491343619946	0.0974010273229777	0.0558424907947981	EDNRA/EDNRB/MAGI2	3	1.36363636363636
GO:0072111	BP	GO:0072111	cell proliferation involved in kidney development	3/666	22/18614	0.0423491343619946	0.0974010273229777	0.0558424907947981	IL6R/ITGB3/SHH	3	1.36363636363636
GO:0072112	BP	GO:0072112	podocyte differentiation	3/666	22/18614	0.0423491343619946	0.0974010273229777	0.0558424907947981	EDNRA/EDNRB/MAGI2	3	1.36363636363636
GO:1903589	BP	GO:1903589	positive regulation of blood vessel endothelial cell proliferation involved in sprouting angiogenesis	3/666	22/18614	0.0423491343619946	0.0974010273229777	0.0558424907947981	AGTR1/APELA/VEGFA	3	1.36363636363636
GO:2000725	BP	GO:2000725	regulation of cardiac muscle cell differentiation	3/666	22/18614	0.0423491343619946	0.0974010273229777	0.0558424907947981	DKK1/MEF2C/WNT3A	3	1.36363636363636
GO:0031113	BP	GO:0031113	regulation of microtubule polymerization	5/666	54/18614	0.0433266753368597	0.0994167176847177	0.0569981374400577	MAP1B/MAP2/MAPT/SNCA/TUBB4A	5	0.925925925925926
GO:0032330	BP	GO:0032330	regulation of chondrocyte differentiation	5/666	54/18614	0.0433266753368597	0.0994167176847177	0.0569981374400577	AXIN2/PRKG2/SOX5/SOX9/WNT9A	5	0.925925925925926
GO:0043550	BP	GO:0043550	regulation of lipid kinase activity	5/666	54/18614	0.0433266753368597	0.0994167176847177	0.0569981374400577	CCR7/F2/PIK3R2/PIK3R5/PTK2B	5	0.925925925925926
GO:0071320	BP	GO:0071320	cellular response to cAMP	5/666	54/18614	0.0433266753368597	0.0994167176847177	0.0569981374400577	HCN1/KCNQ1/PENK/PIK3CG/RAP1A	5	0.925925925925926
GO:0098703	BP	GO:0098703	calcium ion import across plasma membrane	5/666	54/18614	0.0433266753368597	0.0994167176847177	0.0569981374400577	CACNA1A/CACNA1C/CACNA1D/P2RX1/PRNP	5	0.925925925925926
GO:0010717	BP	GO:0010717	regulation of epithelial to mesenchymal transition	8/666	110/18614	0.0437586707404064	0.100361113713998	0.0575395837473625	AXIN2/EPHA3/EPHA4/NKX2-1/OLFM1/PDPN/TBX20/TWIST1	8	0.727272727272727
GO:0002260	BP	GO:0002260	lymphocyte homeostasis	6/666	72/18614	0.0438102847146604	0.100432625640185	0.0575805833568077	BCL2/FAS/MEF2C/MIF/P2RX7/SOS2	6	0.833333333333333

	ONTOLOGY	ID	Description	GeneRatio	BgRatio	pvalue	p.adjust	qvalue	geneID	Count	richFactor
GO:0016051	BP	GO:0016051	carbohydrate biosynthetic process	13/666	214/18614	0.0445612841364031	0.102106625422108	0.0585403300861986	ADCYAP1R1/AVPR1B/GCG/GPER1/IGF1/LEP/LEPR/NR3C1/NTSR1/P2RY1/PTH1R/PTK2B/SNCA	13	0.607476635514019
GO:0071559	BP	GO:0071559	response to transforming growth factor beta	16/666	280/18614	0.0449537213392401	0.102957847335678	0.0590283572988404	CAV1/CLDN1/COL3A1/CX3CR1/GDF15/HSP90AB1/MEF2C/NKX2-1/NR3C1/PENK/SOX5/SOX9/WNT1/WNT2/WNT5A/WNT7A	16	0.571428571428571
GO:0007340	BP	GO:0007340	acrosome reaction	4/666	38/18614	0.0458778325623498	0.104732720410566	0.0600459372573938	ADCY3/GLRB/RIMS1/STXBP1	4	1.05263157894737
GO:0010742	BP	GO:0010742	macrophage derived foam cell differentiation	4/666	38/18614	0.0458778325623498	0.104732720410566	0.0600459372573938	AGT/AGTR1/CSF1/ITGB3	4	1.05263157894737
GO:0010770	BP	GO:0010770	positive regulation of cell morphogenesis involved in differentiation	4/666	38/18614	0.0458778325623498	0.104732720410566	0.0600459372573938	CAMK2B/EPHA4/EPHB2/RELN	4	1.05263157894737
GO:0030808	BP	GO:0030808	regulation of nucleotide biosynthetic process	4/666	38/18614	0.0458778325623498	0.104732720410566	0.0600459372573938	HPCA/NOS1/PINK1/SNCA	4	1.05263157894737
GO:0040036	BP	GO:0040036	regulation of fibroblast growth factor receptor signaling pathway	4/666	38/18614	0.0458778325623498	0.104732720410566	0.0600459372573938	APLN/NGFR/SULF1/WNT5A	4	1.05263157894737
GO:0045742	BP	GO:0045742	positive regulation of epidermal growth factor receptor signaling pathway	4/666	38/18614	0.0458778325623498	0.104732720410566	0.0600459372573938	ADRA2A/AGT/GPER1/MMP9	4	1.05263157894737
GO:1900371	BP	GO:1900371	regulation of purine nucleotide biosynthetic process	4/666	38/18614	0.0458778325623498	0.104732720410566	0.0600459372573938	HPCA/NOS1/PINK1/SNCA	4	1.05263157894737
GO:0002686	BP	GO:0002686	negative regulation of leukocyte migration	5/666	55/18614	0.0463154874874174	0.10536125360255	0.0604062913517218	CXCL12/DUSP1/MIF/PTGER4/SLIT2	5	0.909090909090909
GO:0010658	BP	GO:0010658	striated muscle cell apoptotic process	5/666	55/18614	0.0463154874874174	0.10536125360255	0.0604062913517218	AGT/CAMK2A/CAMK2D/CAPN2/GNGT1	5	0.909090909090909
GO:0043525	BP	GO:0043525	positive regulation of neuron apoptotic process	5/666	55/18614	0.0463154874874174	0.10536125360255	0.0604062913517218	ASCL1/GRIK5/NR3C1/POU4F1/PRNP	5	0.909090909090909
GO:0060632	BP	GO:0060632	regulation of microtubule-based movement	5/666	55/18614	0.0463154874874174	0.10536125360255	0.0604062913517218	CALY/MAP2/NEFH/TACR1/TACR2	5	0.909090909090909
GO:0061462	BP	GO:0061462	protein localization to lysosome	5/666	55/18614	0.0463154874874174	0.10536125360255	0.0604062913517218	CACNG4/CACNG5/CACNG7/CACNG8/CLU	5	0.909090909090909
GO:2000772	BP	GO:2000772	regulation of cellular senescence	5/666	55/18614	0.0463154874874174	0.10536125360255	0.0604062913517218	AKT3/HMGA2/MIF/TWIST1/WNT1	5	0.909090909090909
GO:0045109	BP	GO:0045109	intermediate filament organization	6/666	73/18614	0.046354234157282	0.10536125360255	0.0604062913517218	KRT4/KRT6B/NEFH/NEFL/NEFM/SHH	6	0.821917808219178
GO:1905818	BP	GO:1905818	regulation of chromosome separation	6/666	73/18614	0.046354234157282	0.10536125360255	0.0604062913517218	APC/BUB1B/CCNB1/DUSP1/MAD2L1/TTK	6	0.821917808219178
GO:0072331	BP	GO:0072331	signal transduction by p53 class mediator	11/666	173/18614	0.0469497731583606	0.10536125360255	0.0604062913517218	AURKA/BCL2/BDKRB2/DDIT4/MAP2K6/MIF/POU4F1/PRKN/RPS6KA6/TP73/TWIST1	11	0.635838150289017
GO:0051251	BP	GO:0051251	positive regulation of lymphocyte activation	18/666	327/18614	0.0474311035923297	0.10536125360255	0.0604062913517218	AXL/BCL2/CAV1/CCL5/CCR2/CCR7/CHRNB2/EPHB2/IGF1/IL1A/IL6ST/LEP/MEF2C/MIF/RIPK2/SHH/TACR1/WNT3A	18	0.55045871559633
GO:0002689	BP	GO:0002689	negative regulation of leukocyte chemotaxis	3/666	23/18614	0.0474470182471412	0.10536125360255	0.0604062913517218	DUSP1/MIF/SLIT2	3	1.30434782608696
GO:0003177	BP	GO:0003177	pulmonary valve development	3/666	23/18614	0.0474470182471412	0.10536125360255	0.0604062913517218	ROBO2/SLIT2/TBX20	3	1.30434782608696
GO:0003283	BP	GO:0003283	atrial septum development	3/666	23/18614	0.0474470182471412	0.10536125360255	0.0604062913517218	TBX20/WNT11/WNT5A	3	1.30434782608696

	ONTOLOGY	ID	Description	GeneRatio	BgRatio	pvalue	p.adjust	qvalue	geneID	Count	richFactor
GO:0006883	BP	GO:0006883	intracellular sodium ion homeostasis	3/666	23/18614	0.0474470182471412	0.10536125360255	0.0604062913517218	AGT/ATP1A2/IL1A	3	1.30434782608696
GO:0006925	BP	GO:0006925	inflammatory cell apoptotic process	3/666	23/18614	0.0474470182471412	0.10536125360255	0.0604062913517218	MEF2C/PIK3CD/SLC7A11	3	1.30434782608696
GO:0021846	BP	GO:0021846	cell proliferation in forebrain	3/666	23/18614	0.0474470182471412	0.10536125360255	0.0604062913517218	KIF14/WNT3A/WNT7A	3	1.30434782608696
GO:0021904	BP	GO:0021904	dorsal/ventral neural tube patterning	3/666	23/18614	0.0474470182471412	0.10536125360255	0.0604062913517218	PRKACB/SHH/WNT3A	3	1.30434782608696
GO:0031065	BP	GO:0031065	positive regulation of histone deacetylation	3/666	23/18614	0.0474470182471412	0.10536125360255	0.0604062913517218	LRRK2/PINK1/VEGFA	3	1.30434782608696
GO:0032891	BP	GO:0032891	negative regulation of organic acid transport	3/666	23/18614	0.0474470182471412	0.10536125360255	0.0604062913517218	LEP/NPY5R/RGS2	3	1.30434782608696
GO:0036303	BP	GO:0036303	lymph vessel morphogenesis	3/666	23/18614	0.0474470182471412	0.10536125360255	0.0604062913517218	PDPN/PROX1/VEGFA	3	1.30434782608696
GO:0038095	BP	GO:0038095	Fc-epsilon receptor signaling pathway	3/666	23/18614	0.0474470182471412	0.10536125360255	0.0604062913517218	MAPK10/PLCG1/PLCG2	3	1.30434782608696
GO:0040019	BP	GO:0040019	positive regulation of embryonic development	3/666	23/18614	0.0474470182471412	0.10536125360255	0.0604062913517218	LHX1/WNT1/WNT3A	3	1.30434782608696
GO:0051131	BP	GO:0051131	chaperone-mediated protein complex assembly	3/666	23/18614	0.0474470182471412	0.10536125360255	0.0604062913517218	CLU/HSP90AB1/TMEM35A	3	1.30434782608696
GO:0060065	BP	GO:0060065	uterus development	3/666	23/18614	0.0474470182471412	0.10536125360255	0.0604062913517218	LHX1/WNT5A/WNT7A	3	1.30434782608696
GO:0072170	BP	GO:0072170	metanephric tubule development	3/666	23/18614	0.0474470182471412	0.10536125360255	0.0604062913517218	CALB1/SOX9/WNT7B	3	1.30434782608696
GO:0072243	BP	GO:0072243	metanephric nephron epithelium development	3/666	23/18614	0.0474470182471412	0.10536125360255	0.0604062913517218	CALB1/SOX9/WNT7B	3	1.30434782608696
GO:0072311	BP	GO:0072311	glomerular epithelial cell differentiation	3/666	23/18614	0.0474470182471412	0.10536125360255	0.0604062913517218	EDNRA/EDNRB/MAGI2	3	1.30434782608696
GO:0072574	BP	GO:0072574	hepatocyte proliferation	3/666	23/18614	0.0474470182471412	0.10536125360255	0.0604062913517218	PROX1/PTN/WNT3A	3	1.30434782608696
GO:0072575	BP	GO:0072575	epithelial cell proliferation involved in liver morphogenesis	3/666	23/18614	0.0474470182471412	0.10536125360255	0.0604062913517218	PROX1/PTN/WNT3A	3	1.30434782608696
GO:0097150	BP	GO:0097150	neuronal stem cell population maintenance	3/666	23/18614	0.0474470182471412	0.10536125360255	0.0604062913517218	ASPM/PROX1/SOX2	3	1.30434782608696
GO:1901032	BP	GO:1901032	negative regulation of response to reactive oxygen species	3/666	23/18614	0.0474470182471412	0.10536125360255	0.0604062913517218	LRRK2/PINK1/TRAP1	3	1.30434782608696
GO:2000353	BP	GO:2000353	positive regulation of endothelial cell apoptotic process	3/666	23/18614	0.0474470182471412	0.10536125360255	0.0604062913517218	GPER1/ITGA4/PLCG1	3	1.30434782608696
GO:0003253	BP	GO:0003253	cardiac neural crest cell migration involved in outflow tract morphogenesis	2/666	10/18614	0.0475465433347826	0.10536125360255	0.0604062913517218	EDNRA/TWIST1	2	2
GO:0003310	BP	GO:0003310	pancreatic A cell differentiation	2/666	10/18614	0.0475465433347826	0.10536125360255	0.0604062913517218	NEUROD1/NKX6-1	2	2
GO:0009629	BP	GO:0009629	response to gravity	2/666	10/18614	0.0475465433347826	0.10536125360255	0.0604062913517218	GHR/STX1A	2	2
GO:0014012	BP	GO:0014012	peripheral nervous system axon regeneration	2/666	10/18614	0.0475465433347826	0.10536125360255	0.0604062913517218	MAP1B/NEFL	2	2
GO:0014848	BP	GO:0014848	urinary tract smooth muscle contraction	2/666	10/18614	0.0475465433347826	0.10536125360255	0.0604062913517218	P2RX2/TACR1	2	2
GO:0033034	BP	GO:0033034	positive regulation of myeloid cell apoptotic process	2/666	10/18614	0.0475465433347826	0.10536125360255	0.0604062913517218	MEF2C/PIK3CD	2	2

	ONTOLOGY	ID	Description	GeneRatio	BgRatio	pvalue	p.adjust	qvalue	geneID	Count	richFactor
GO:0033210	BP	GO:0033210	leptin-mediated signaling pathway	2/666	10/18614	0.0475465433347826	0.10536125360255	0.0604062913517218	LEP/LEPR	2	2
GO:0035768	BP	GO:0035768	endothelial cell chemotaxis to fibroblast growth factor	2/666	10/18614	0.0475465433347826	0.10536125360255	0.0604062913517218	CXCL13/FGFR1	2	2
GO:0036093	BP	GO:0036093	germ cell proliferation	2/666	10/18614	0.0475465433347826	0.10536125360255	0.0604062913517218	PTGDR2/UCHL1	2	2
GO:0038130	BP	GO:0038130	ERBB4 signaling pathway	2/666	10/18614	0.0475465433347826	0.10536125360255	0.0604062913517218	ERBB4/NRG3	2	2
GO:0043653	BP	GO:0043653	mitochondrial fragmentation involved in apoptotic process	2/666	10/18614	0.0475465433347826	0.10536125360255	0.0604062913517218	ERBB4/NPTX1	2	2
GO:0044341	BP	GO:0044341	sodium-dependent phosphate transport	2/666	10/18614	0.0475465433347826	0.10536125360255	0.0604062913517218	SLC17A7/SLC17A8	2	2
GO:0048630	BP	GO:0048630	skeletal muscle tissue growth	2/666	10/18614	0.0475465433347826	0.10536125360255	0.0604062913517218	CHRNA1/CHRND	2	2
GO:0048671	BP	GO:0048671	negative regulation of collateral sprouting	2/666	10/18614	0.0475465433347826	0.10536125360255	0.0604062913517218	FGF13/FSTL4	2	2
GO:0048865	BP	GO:0048865	stem cell fate commitment	2/666	10/18614	0.0475465433347826	0.10536125360255	0.0604062913517218	EDNRA/SOX9	2	2
GO:0055015	BP	GO:0055015	ventricular cardiac muscle cell development	2/666	10/18614	0.0475465433347826	0.10536125360255	0.0604062913517218	CDK1/PROX1	2	2
GO:0060054	BP	GO:0060054	positive regulation of epithelial cell proliferation involved in wound healing	2/666	10/18614	0.0475465433347826	0.10536125360255	0.0604062913517218	CLDN1/WNT7A	2	2
GO:0060502	BP	GO:0060502	epithelial cell proliferation involved in lung morphogenesis	2/666	10/18614	0.0475465433347826	0.10536125360255	0.0604062913517218	SOX9/WNT2	2	2
GO:0060947	BP	GO:0060947	cardiac vascular smooth muscle cell differentiation	2/666	10/18614	0.0475465433347826	0.10536125360255	0.0604062913517218	GPER1/VEGFA	2	2
GO:0071374	BP	GO:0071374	cellular response to parathyroid hormone stimulus	2/666	10/18614	0.0475465433347826	0.10536125360255	0.0604062913517218	CSF1/MEF2C	2	2
GO:0071600	BP	GO:0071600	otic vesicle morphogenesis	2/666	10/18614	0.0475465433347826	0.10536125360255	0.0604062913517218	PROX1/SOX9	2	2
GO:0090394	BP	GO:0090394	negative regulation of excitatory postsynaptic potential	2/666	10/18614	0.0475465433347826	0.10536125360255	0.0604062913517218	LRRK2/NLGN4X	2	2
GO:0098779	BP	GO:0098779	positive regulation of mitophagy in response to mitochondrial depolarization	2/666	10/18614	0.0475465433347826	0.10536125360255	0.0604062913517218	PINK1/PRKN	2	2
GO:0140059	BP	GO:0140059	dendrite arborization	2/666	10/18614	0.0475465433347826	0.10536125360255	0.0604062913517218	CHRNA7/PTN	2	2
GO:0150172	BP	GO:0150172	regulation of phosphatidylcholine metabolic process	2/666	10/18614	0.0475465433347826	0.10536125360255	0.0604062913517218	APOC1/CAPN2	2	2
GO:1902513	BP	GO:1902513	regulation of organelle transport along microtubule	2/666	10/18614	0.0475465433347826	0.10536125360255	0.0604062913517218	MAP2/NEFH	2	2
GO:1903596	BP	GO:1903596	regulation of gap junction assembly	2/666	10/18614	0.0475465433347826	0.10536125360255	0.0604062913517218	AGT/CAV1	2	2
GO:1904181	BP	GO:1904181	positive regulation of membrane depolarization	2/666	10/18614	0.0475465433347826	0.10536125360255	0.0604062913517218	ANK3/P2RX7	2	2
GO:2000048	BP	GO:2000048	negative regulation of cell-cell adhesion mediated by cadherin	2/666	10/18614	0.0475465433347826	0.10536125360255	0.0604062913517218	NEXMIF/VEGFA	2	2
GO:2000347	BP	GO:2000347	positive regulation of hepatocyte proliferation	2/666	10/18614	0.0475465433347826	0.10536125360255	0.0604062913517218	PTN/WNT3A	2	2

	ONTOLOGY	ID	Description	GeneRatio	BgRatio	pvalue	p.adjust	qvalue	geneID	Count	richFactor
GO:2000544	BP	GO:2000544	regulation of endothelial cell chemotaxis to fibroblast growth factor	2/666	10/18614	0.0475465433347826	0.10536125360255	0.0604062913517218	CXCL13/FGFR1	2	2
GO:2000849	BP	GO:2000849	regulation of glucocorticoid secretion	2/666	10/18614	0.0475465433347826	0.10536125360255	0.0604062913517218	GAL/GALR1	2	2
GO:2001054	BP	GO:2001054	negative regulation of mesenchymal cell apoptotic process	2/666	10/18614	0.0475465433347826	0.10536125360255	0.0604062913517218	SHH/SOX9	2	2
GO:0046718	BP	GO:0046718	viral entry into host cell	10/666	153/18614	0.0484783617329901	0.107377714248601	0.0615623796206041	AGTR1/AVPR1B/AXL/CAV1/CDK1/CLDN1/ITGB3/LY6E/NCAM1/TYRO3	10	0.65359477124183
GO:0003281	BP	GO:0003281	ventricular septum development	6/666	74/18614	0.0489880237228527	0.108408886356516	0.062153576864891	PROX1/ROBO2/SLIT2/SLIT3/WNT11/WNT5A	6	0.810810810810811
GO:0008333	BP	GO:0008333	endosome to lysosome transport	6/666	74/18614	0.0489880237228527	0.108408886356516	0.062153576864891	ADRB2/BECN2/CACNG4/CACNG5/CACNG7/CACNG8	6	0.810810810810811
GO:0032465	BP	GO:0032465	regulation of cytokinesis	7/666	93/18614	0.0490838110626033	0.108425587133796	0.0621631518460504	AURKA/BCL2L1/CXCR5/DRD2/KIF14/RXFP3/SSTR5	7	0.752688172043011
GO:0045995	BP	GO:0045995	regulation of embryonic development	7/666	93/18614	0.0490838110626033	0.108425587133796	0.0621631518460504	CDK1/LHX1/RPS6KA6/WNT1/WNT2/WNT2B/WNT3A	7	0.752688172043011
GO:1900542	BP	GO:1900542	regulation of purine nucleotide metabolic process	7/666	93/18614	0.0490838110626033	0.108425587133796	0.0621631518460504	DDIT4/HPCA/IGF1/NOS1/P2RX7/PINK1/SNCA	7	0.752688172043011
GO:1903035	BP	GO:1903035	negative regulation of response to wounding	7/666	93/18614	0.0490838110626033	0.108425587133796	0.0621631518460504	CLDN19/EPHA4/F2/KNG1/PRKG1/RTN4R/TNR	7	0.752688172043011
GO:0001954	BP	GO:0001954	positive regulation of cell-matrix adhesion	5/666	56/18614	0.0494239172993763	0.108834477386395	0.0623975790466093	CCR7/CSF1/ITGB3/PTK2B/VEGFA	5	0.892857142857143
GO:0009994	BP	GO:0009994	oocyte differentiation	5/666	56/18614	0.0494239172993763	0.108834477386395	0.0623975790466093	AURKA/BCL2/EDNRA/IGF1/PTK2B	5	0.892857142857143
GO:0019369	BP	GO:0019369	arachidonic acid metabolic process	5/666	56/18614	0.0494239172993763	0.108834477386395	0.0623975790466093	CYP2C18/CYP2C8/MGLL/PLA2G4B/PTGS1	5	0.892857142857143
GO:0042908	BP	GO:0042908	xenobiotic transport	5/666	56/18614	0.0494239172993763	0.108834477386395	0.0623975790466093	CLDN1/SLC22A2/SLC22A3/SLC28A2/SLC29A1	5	0.892857142857143
GO:0045806	BP	GO:0045806	negative regulation of endocytosis	5/666	56/18614	0.0494239172993763	0.108834477386395	0.0623975790466093	APOC1/CAV1/EPHA3/ITGB3/SYT11	5	0.892857142857143
GO:0051058	BP	GO:0051058	negative regulation of small GTPase mediated signal transduction	5/666	56/18614	0.0494239172993763	0.108834477386395	0.0623975790466093	ADRA1A/ARHGAP44/EPHB2/RASAL1/SLIT2	5	0.892857142857143
GO:0070231	BP	GO:0070231	T cell apoptotic process	5/666	56/18614	0.0494239172993763	0.108834477386395	0.0623975790466093	BCL2/CCL5/FAS/P2RX7/WNT5A	5	0.892857142857143
GO:0021532	BP	GO:0021532	neural tube patterning	4/666	39/18614	0.0497310320052409	0.109265991196137	0.0626449769089381	PRKACB/SHH/WNT1/WNT3A	4	1.02564102564103
GO:0042307	BP	GO:0042307	positive regulation of protein import into nucleus	4/666	39/18614	0.0497310320052409	0.109265991196137	0.0626449769089381	CDK1/LEP/PIK3R2/SHH	4	1.02564102564103
GO:0042401	BP	GO:0042401	biogenic amine biosynthetic process	4/666	39/18614	0.0497310320052409	0.109265991196137	0.0626449769089381	PAH/SLC6A3/SNCA/TH	4	1.02564102564103
GO:0070232	BP	GO:0070232	regulation of T cell apoptotic process	4/666	39/18614	0.0497310320052409	0.109265991196137	0.0626449769089381	BCL2/CCL5/P2RX7/WNT5A	4	1.02564102564103
GO:0090077	BP	GO:0090077	foam cell differentiation	4/666	39/18614	0.0497310320052409	0.109265991196137	0.0626449769089381	AGT/AGTR1/CSF1/ITGB3	4	1.02564102564103
GO:0001701	BP	GO:0001701	in utero embryonic development	21/666	398/18614	0.0499499758142006	0.109698003184449	0.0628926603897348	ADCY9/BCL2L1/BYSL/CAPN2/CCNB1/COL3A1/EDNRA/EMX1/EPAS1/GNA13/IGF1/PLCG1/PTH1R/RRM2/SLC5A7/SOX10/TWIST1/VEGFA/WNT2/WNT3A/WNT7B	21	0.527638190954774

	ONTOLOGY	ID	Description	GeneRatio	BgRatio	pvalue	p.adjust	qvalue	geneID	Count	richFactor
GO:0097060	CC	GO:0097060	synaptic membrane	110/673	390/19518	1.01864866166818e-69	4.54317303104007e-67	2.8522162526709e-67	ADORA3/ADRA1A/ADRA2A/ANK3/CACNA1C/CACNG4/CACNG5/CACNG7/CACNG8/CHRM2/CHRM4/CHRM5/CHRNA1/CHRNA3/CHRNA4/CHRNA6/CHRNA7/CHRNB2/CHRNB4/CHRND/CHRNG/CNIH3/CNR1/CNTN2/CNTN6/DLG2/DRD2/EPHA4/EPHB2/ERBB4/GABRA3/GABRA4/GABRB1/GABRB3/GABRD/GABRE/GABRG2/GABRP/GABRR1/GLRA3/GLRB/GPER1/GPM6A/GRIA1/GRIA2/GRIA3/GRIA4/GRID2/GRIK1/GRIK2/GRIK3/GRIK5/GRIN2A/GRIN2B/GRIN2D/GRIN3A/GRIN3B/GRM2/GSG1L/HCN1/HTR3A/ITGB3/KCNA1/KCNA2/KCNJ9/LHFPL4/LPAR1/LRRC7/NDUFS7/NLGN1/NLGN3/NLGN4X/NLGN4Y/NRCAM/NRP2/NRXN1/NSG1/NTNG1/NTRK3/OPRD1/OPRM1/P2RX1/P2RY1/P2RY4/PRKCG/RIMS1/RIMS3/RIMS4/RPH3A/SHC4/SHISA7/SIGMAR1/SLC1A2/SLC1A7/SLC4A8/SLC6A1/SLC6A2/SLC6A3/SLC6A6/SLC6A9/SLITRK3/SNAP25/SNCAIP/SORCS3/STX1A/STXBP1/SYP/SYT11/SYT7/UNC13C	110	2.82051282051282
GO:0045211	CC	GO:0045211	postsynaptic membrane	84/673	277/19518	7.19037477608288e-56	1.60345357506648e-53	1.0066524686516e-53	ADRA1A/ADRA2A/ANK3/CACNA1C/CACNG4/CACNG5/CACNG7/CACNG8/CHRM2/CHRM4/CHRM5/CHRNA1/CHRNA3/CHRNA4/CHRNA6/CHRNA7/CHRNB2/CHRNB4/CHRND/CHRNG/CNIH3/CNTN2/DLG2/DRD2/EPHA4/EPHB2/ERBB4/GABRA3/GABRA4/GABRB1/GABRB3/GABRD/GABRE/GABRG2/GABRP/GABRR1/GLRA3/GLRB/GRIA1/GRIA2/GRIA3/GRIA4/GRID2/GRIK1/GRIK2/GRIK3/GRIK5/GRIN2A/GRIN2B/GRIN2D/GRIN3A/GRIN3B/GSG1L/HCN1/HTR3A/ITGB3/KCNA1/KCNA2/LHFPL4/LPAR1/LRRC7/NLGN1/NLGN3/NLGN4X/NLGN4Y/NRCAM/NRP2/NSG1/NTRK3/OPRD1/OPRM1/P2RX1/P2RY1/RPH3A/SHC4/SHISA7/SIGMAR1/SLC6A1/SLC6A3/SLC6A6/SLC6A9/SLITRK3/SORCS3/STX1A	84	3.03249097472924

	ONTOLOGY	ID	Description	GeneRatio	BgRatio	pvalue	p.adjust	qvalue	geneID	Count	richFactor
GO:0043025	CC	GO:0043025	neuronal cell body	106/673	500/19518	1.13748573333571e-53	1.69106212355908e-51	1.06165335111333e-51	ADCYAP1/ADRA2A/ASCL1/ASTN1/ATP1A2/AURKA/CACNA1A/CACNA1C/CACNG7/CALB1/CALCA/CAPN2/CCR2/CHRM2/CHRNA3/CHRNA4/CNR2/CNTN2/CPLX1/CPNE5/CSNK1E/CX3CR1/DLG2/DNER/DRD2/EPHA4/EPHB2/EVX1/FLRT1/FZD3/GABRD/GAL/GAP43/GFRA1/GHR/GLRA3/GPM6A/GRIA1/GRIA2/GRIA4/GRIK2/GRIK3/GRIK5/GRIN3A/GRIN3B/HOMER1/HPCA/HSP90AB1/IL6ST/ITGA4/KCNA1/KCNA2/KCNJ14/KCNQ1/KIF5A/KIF5C/KISS1/L1CAM/LPAR1/LRRK2/MAP1B/MAP2/MAPT/NDN/NDUFS7/NGFR/NPY/NRXN1/NSMF/NTSR1/OLFM1/OPRM1/P2RX2/P2RX4/P2RX7/PENK/PNOC/PRPH/PTK2B/RTN4R/SCN1B/SLC17A8/SLC1A1/SLC22A3/SLC4A8/SLC5A7/SLC6A1/SLC6A2/SLC6A3/SLC6A6/SNCA/SNCAIP/SNCG/SPTBN2/SST/SYT11/SYT4/SYT5/TH/TUBB3/TUBB4A/UCHL1/UCN/UNC5C/VGF/VIP	106	2.12
GO:0098978	CC	GO:0098978	glutamatergic synapse	86/673	410/19518	4.91654593702255e-43	5.48194871978015e-41	3.44158215591579e-41	ADGRL3/ADRA1A/ADRA2A/ARHGAP44/AURKA/CACNG4/CACNG5/CACNG7/CADPS/CALB1/CALY/CAMK4/CHRM2/CNR1/CNTNAP1/CPLX1/DLGAP2/DRD2/DRD4/EPHA4/EPHB2/ERBB4/FZD9/GABRG2/GABRR1/GPM6A/GRIA1/GRID2/GRIK2/GRIK3/GRIK5/GRIN2A/GRIN2D/GRIN3A/GRM2/GSG1L/GUCY1A1/HCN1/HOMER1/HPCA/ICAM5/ITGB3/KCNA1/KCNA2/LGI1/LPAR1/LRRK2/NLGN1/NLGN3/NLGN4X/NLGN4Y/NPTX1/NPTX2/NRCAM/NRG3/NRP2/NRXN2/NSG1/NTNG1/NTRK3/P2RX1/P2RY1/P2RY4/PLCG1/PTK2B/RAP1A/RTN4R/SEMA3F/SH3GL2/SHISA7/SLC1A2/SLC4A8/SNAP25/SORCS3/SPTBN2/STX1A/STXBP1/SYN2/SYN3/SYT10/SYT4/TNR/VGF/WNT3A/WNT5A/WNT7A	86	2.09756097560976

	ONTOLOGY	ID	Description	GeneRatio	BgRatio	pvalue	p.adjust	qvalue	geneID	Count	richFactor
GO:0042734	CC	GO:0042734	presynaptic membrane	53/673	164/19518	5.18006617510225e-37	4.62061902819121e-35	2.90083705805726e-35	ADORA3/ADRA1A/ADRA2A/CHRM2/CHRNA6/CHRNB2/CNR1/CNTN6/DRD2/EPHA4/EPHB2/ERBB4/GABRA3/GABRB1/GABRR1/GPER1/GPM6A/GRIK1/GRIK2/GRIK3/GRIK5/GRIN2A/GRIN2D/GRM2/HCN1/KCNA1/KCNA2/KCNJ9/LPAR1/NRXN1/NTNG1/OPRD1/OPRM1/P2RX1/P2RY1/P2RY4/RIMS1/RIMS3/RIMS4/SLC1A2/SLC4A8/SLC6A1/SLC6A2/SLC6A3/SLC6A9/SNAP25/SNCAIP/STX1A/STXBP1/SYP/SYT11/SYT7/UNC13C	53	3.23170731707317
GO:0098984	CC	GO:0098984	neuron to neuron synapse	76/673	382/19518	2.35784072271094e-36	1.7526616038818e-34	1.10032567059844e-34	ADRA2A/ARHGAP44/AURKA/CACNA1C/CACNG4/CACNG5/CACNG7/CACNG8/CALB1/CALCA/CAMK2A/CHRM2/CHRNA3/DCLK1/DLG2/DLGAP2/DRD2/EPHA4/EPHB2/ERBB4/GAP43/GPER1/GRIA1/GRIA2/GRIA3/GRIA4/GRID2/GRIK1/GRIK2/GRIK3/GRIK5/GRIN2A/GRIN2B/GRIN2D/GRIN3A/GRIN3B/GSG1L/HOMER1/ITPR1/LRRC7/MAGI2/MAP1B/NEFH/NGFR/NLGN1/NLGN3/NLGN4X/NLGN4Y/NOS1/NRCAM/NSMF/NTSR1/OPRD1/P2RX4/P2RY1/PAK6/PENK/PRKCG/PRKN/PRNP/PTK2B/SHISA7/SIGMAR1/SLC1A1/SLC4A8/SLC6A9/SLITRK3/SORCS3/STX1A/SYN2/SYN3/SYT11/SYT12/SYT7/SYT9/TMEM108	76	1.98952879581152
GO:0099572	CC	GO:0099572	postsynaptic specialization	74/673	364/19518	4.5200147441926e-36	2.87989510844272e-34	1.80800589767704e-34	ADRA2A/ARHGAP44/AURKA/CACNA1C/CACNG4/CACNG5/CACNG7/CACNG8/CAMK2A/CHRNA1/CHRNA3/CHRNB2/CHRND/DCLK1/DLG2/DLGAP2/DRD2/EPHA4/ERBB4/GABRA3/GABRA4/GABRB1/GABRG2/GAP43/GLRB/GPER1/GRIA1/GRIA2/GRIA3/GRIA4/GRID2/GRIK1/GRIK2/GRIK3/GRIK5/GRIN2A/GRIN2B/GRIN2D/GRIN3A/GRIN3B/GSG1L/HOMER1/ITPR1/LHFPL4/LRRC7/MAGI2/MAP1B/NEFH/NGFR/NLGN1/NLGN3/NLGN4X/NLGN4Y/NOS1/NRCAM/NSMF/OPRD1/P2RX4/P2RY1/PAK6/PRKCG/PRKN/PRNP/PTK2B/SHISA7/SIGMAR1/SLC6A9/SLITRK3/SORCS3/STX1A/SYN2/SYN3/SYT11/TMEM108	74	2.03296703296703

	ONTOLOGY	ID	Description	GeneRatio	BgRatio	pvalue	p.adjust	qvalue	geneID	Count	richFactor
GO:0034702	CC	GO:0034702	ion channel complex	67/673	299/19518	1.82506209890734e-35	1.01747212014084e-33	6.38771734617568e-34	ATP2A1/CACNA1A/CACNA1C/CACNA1D/CACNG4/CACNG5/CACNG7/CACNG8/CHRFAM7A/CHRNA1/CHRNA3/CHRNA4/CHRNA6/CHRNA7/CHRNB2/CHRNB4/CHRND/CLCN1/CNIH3/FKBP1B/GABRA3/GABRA4/GABRB1/GABRB3/GABRD/GABRE/GABRG2/GABRP/GABRR1/GLRA3/GLRB/GRIA1/GRIA2/GRIA3/GRIA4/GRID2/GRIK1/GRIK2/GRIK3/GRIK5/GRIN2A/GRIN2B/GRIN2D/GRIN3A/GRIN3B/HCN1/HTR3A/HTR3C/HTR3E/KCNA1/KCNA2/KCNJ14/KCNJ5/KCNMB2/KCNQ1/KCNQ5/OLFM3/PTK2B/RYR1/RYR3/SCN1B/SCN2B/SHISA7/SLC17A7/SLC17A8/TRPC1/VWC2	67	2.24080267558528
GO:0150034	CC	GO:0150034	distal axon	64/673	277/19518	9.49343521399053e-35	4.70452456159975e-33	2.95351317768594e-33	ADRA2A/CALB1/CALB2/CALCA/CDK5R2/CHRM2/CNR1/CPLX1/CSNK1E/DRD2/EPHA4/FGF13/GAD1/GAP43/GPER1/GPM6A/GRIK3/GRIK5/HSP90AB1/ITGA4/KCNA1/KCNA2/KIF5C/L1CAM/LRRK2/MAP1B/MAP2/MAPT/NEFL/NEO1/NGFR/NMU/NPY/NTSR1/OLFM1/OPRD1/OXT/P2RX4/PALLD/PENK/PINK1/PNOC/PRKCB/PRKCG/PRNP/PTK2B/RTN4R/SIGMAR1/SLC17A8/SLC18A2/SLC18A3/SLC1A1/SLC4A8/SLC6A3/SNAP25/SNCA/SNCG/SYP/TH/TUBB3/UCN/UCN3/UNC13C/UNC5C	64	2.31046931407942
GO:1902495	CC	GO:1902495	transmembrane transporter complex	72/673	392/19518	4.22912071988491e-32	1.88618784106867e-30	1.18415380156778e-30	ATP1A2/ATP2A1/ATP6V1F/CACNA1A/CACNA1C/CACNA1D/CACNG4/CACNG5/CACNG7/CACNG8/CHRFAM7A/CHRNA1/CHRNA3/CHRNA4/CHRNA6/CHRNA7/CHRNB2/CHRNB4/CHRND/CLCN1/CNIH3/FKBP1B/GABRA3/GABRA4/GABRB1/GABRB3/GABRD/GABRE/GABRG2/GABRP/GABRR1/GLRA3/GLRB/GRIA1/GRIA2/GRIA3/GRIA4/GRID2/GRIK1/GRIK2/GRIK3/GRIK5/GRIN2A/GRIN2B/GRIN2D/GRIN3A/GRIN3B/HCN1/HTR3A/HTR3C/HTR3E/KCNA1/KCNA2/KCNJ14/KCNJ5/KCNMB2/KCNQ1/KCNQ5/NDUFA11/NDUFA13/NDUFS7/OLFM3/PTK2B/RYR1/RYR3/SCN1B/SCN2B/SHISA7/SLC17A7/SLC17A8/TRPC1/VWC2	72	1.83673469387755

	ONTOLOGY	ID	Description	GeneRatio	BgRatio	pvalue	p.adjust	qvalue	geneID	Count	richFactor
GO:0032279	CC	GO:0032279	asymmetric synapse	68/673	353/19518	1.14850594847487e-31	4.65666957290718e-30	2.92346968702693e-30	ADRA2A/ARHGAP44/AURKA/CACNA1C/CACNG4/CACNG5/CACNG7/CACNG8/CAMK2A/CHRM2/CHRNA3/DCLK1/DLG2/DLGAP2/DRD2/EPHA4/ERBB4/GAP43/GPER1/GRIA1/GRIA2/GRIA3/GRIA4/GRID2/GRIK1/GRIK2/GRIK3/GRIK5/GRIN2A/GRIN2B/GRIN2D/GRIN3A/GRIN3B/GSG1L/HOMER1/ITPR1/LRRC7/MAGI2/MAP1B/NEFH/NGFR/NLGN1/NLGN3/NLGN4X/NLGN4Y/NOS1/NRCAM/NSMF/OPRD1/P2RX4/P2RY1/PAK6/PRKCG/PRKN/PRNP/PTK2B/SHISA7/SIGMAR1/SLC1A1/SLC4A8/SLC6A9/SLITRK3/SORCS3/STX1A/SYN2/SYN3/SYT11/TMEM108	68	1.92634560906516
GO:0044306	CC	GO:0044306	neuron projection terminus	43/673	129/19518	7.42055721279039e-31	2.7579737640871e-29	1.73146334965109e-29	ADRA2A/CALB1/CALB2/CALCA/CHRM2/CPLX1/DRD2/EPHA4/FLRT1/GAD1/GPER1/GRIK3/GRIK5/KCNA1/KCNA2/LRRK2/NMU/NPY/NTSR1/OPRD1/OXT/P2RX4/PENK/PNOC/PRKCB/PRKCG/PRNP/SLC17A8/SLC18A2/SLC18A3/SLC1A1/SLC1A2/SLC4A8/SLC6A3/SNCA/SNCG/SYP/TH/UCHL1/UCN/UCN3/UNC13C/VAMP2	43	3.33333333333333
GO:1990351	CC	GO:1990351	transporter complex	72/673	418/19518	3.01752696474809e-30	1.03524386636742e-28	6.49928884714973e-29	ATP1A2/ATP2A1/ATP6V1F/CACNA1A/CACNA1C/CACNA1D/CACNG4/CACNG5/CACNG7/CACNG8/CHRFAM7A/CHRNA1/CHRNA3/CHRNA4/CHRNA6/CHRNA7/CHRNB2/CHRNB4/CHRND/CLCN1/CNIH3/FKBP1B/GABRA3/GABRA4/GABRB1/GABRB3/GABRD/GABRE/GABRG2/GABRP/GABRR1/GLRA3/GLRB/GRIA1/GRIA2/GRIA3/GRIA4/GRID2/GRIK1/GRIK2/GRIK3/GRIK5/GRIN2A/GRIN2B/GRIN2D/GRIN3A/GRIN3B/HCN1/HTR3A/HTR3C/HTR3E/KCNA1/KCNA2/KCNJ14/KCNJ5/KCNMB2/KCNQ1/KCNQ5/NDUFA11/NDUFA13/NDUFS7/OLFM3/PTK2B/RYR1/RYR3/SCN1B/SCN2B/SHISA7/SLC17A7/SLC17A8/TRPC1/VWC2	72	1.72248803827751
GO:0099634	CC	GO:0099634	postsynaptic specialization membrane	42/673	129/19518	1.0931631663825e-29	3.48250551576139e-28	2.186326332765e-28	ADRA2A/CACNA1C/CACNG4/CACNG5/CACNG7/CACNG8/CHRNA1/CHRNB2/CHRND/DLG2/EPHA4/ERBB4/GABRA3/GABRA4/GABRB1/GABRG2/GRIA1/GRIA2/GRIA3/GRIA4/GRID2/GRIK1/GRIK2/GRIK3/GRIK5/GRIN2A/GRIN2B/GRIN2D/GRIN3A/GRIN3B/GSG1L/NLGN1/NLGN3/NLGN4X/NLGN4Y/NRCAM/OPRD1/SHISA7/SIGMAR1/SLITRK3/SORCS3/STX1A	42	3.25581395348837

	ONTOLOGY	ID	Description	GeneRatio	BgRatio	pvalue	p.adjust	qvalue	geneID	Count	richFactor
GO:0014069	CC	GO:0014069	postsynaptic density	64/673	339/19518	2.58902431911037e-29	7.6980323088215e-28	4.83284539567269e-28	ADRA2A/ARHGAP44/AURKA/CACNA1C/CACNG4/CACNG5/CACNG7/CACNG8/CAMK2A/CHRNA3/DCLK1/DLG2/DLGAP2/DRD2/EPHA4/ERBB4/GAP43/GPER1/GRIA1/GRIA2/GRIA3/GRIA4/GRID2/GRIK1/GRIK2/GRIK3/GRIK5/GRIN2A/GRIN2B/GRIN2D/GRIN3A/GRIN3B/GSG1L/HOMER1/ITPR1/LRRC7/MAGI2/MAP1B/NEFH/NGFR/NLGN1/NLGN4X/NLGN4Y/NOS1/NRCAM/NSMF/OPRD1/P2RX4/P2RY1/PAK6/PRKCG/PRKN/PRNP/PTK2B/SHISA7/SIGMAR1/SLC6A9/SLITRK3/SORCS3/STX1A/SYN2/SYN3/SYT11/TMEM108	64	1.88790560471976
GO:0043679	CC	GO:0043679	axon terminus	39/673	114/19518	1.39396203190115e-28	3.88566916392445e-27	2.43943355582701e-27	ADRA2A/CALB1/CALB2/CALCA/CHRM2/CPLX1/DRD2/EPHA4/GAD1/GPER1/GRIK3/GRIK5/KCNA1/KCNA2/LRRK2/NMU/NPY/NTSR1/OPRD1/OXT/P2RX4/PENK/PNOC/PRKCB/PRKCG/PRNP/SLC17A8/SLC18A2/SLC18A3/SLC1A1/SLC4A8/SLC6A3/SNCA/SNCG/SYP/TH/UCN/UCN3/UNC13C	39	3.42105263157895
GO:0098878	CC	GO:0098878	neurotransmitter receptor complex	26/673	46/19518	1.73193371110866e-26	4.54377903032037e-25	2.85259670064956e-25	CACNG4/CACNG5/CACNG7/CACNG8/CNIH3/GRIA1/GRIA2/GRIA3/GRIA4/GRID2/GRIK1/GRIK2/GRIK3/GRIK5/GRIN2A/GRIN2B/GRIN2D/GRIN3A/GRIN3B/HTR3A/HTR3C/HTR3E/OLFM3/PTK2B/SHISA7/VWC2	26	5.65217391304348
GO:0098839	CC	GO:0098839	postsynaptic density membrane	33/673	102/19518	1.75761859059491e-23	4.23823662032583e-22	2.66077635356779e-22	ADRA2A/CACNA1C/CACNG4/CACNG5/CACNG7/CACNG8/DLG2/EPHA4/ERBB4/GRIA1/GRIA2/GRIA3/GRIA4/GRID2/GRIK1/GRIK2/GRIK3/GRIK5/GRIN2A/GRIN2B/GRIN2D/GRIN3A/GRIN3B/GSG1L/NLGN4X/NLGN4Y/NRCAM/OPRD1/SHISA7/SIGMAR1/SLITRK3/SORCS3/STX1A	33	3.23529411764706
GO:0008328	CC	GO:0008328	ionotropic glutamate receptor complex	23/673	41/19518	1.80552681134957e-23	4.23823662032583e-22	2.66077635356779e-22	CACNG4/CACNG5/CACNG7/CACNG8/CNIH3/GRIA1/GRIA2/GRIA3/GRIA4/GRID2/GRIK1/GRIK2/GRIK3/GRIK5/GRIN2A/GRIN2B/GRIN2D/GRIN3A/GRIN3B/OLFM3/PTK2B/SHISA7/VWC2	23	5.60975609756098
GO:0098982	CC	GO:0098982	GABA-ergic synapse	30/673	84/19518	6.67188335681182e-23	1.48782998856904e-21	9.34063669953654e-22	ADRA1A/ADRA2A/CALB1/CNR1/DRD2/EFNA5/ERBB4/GABRA3/GABRA4/GABRB1/GABRD/GABRG2/GABRR1/GAD1/GAP43/GLRB/GUCY1A1/LHFPL4/LPAR1/MDGA1/NLGN1/NLGN3/NLGN4X/NLGN4Y/NPY/NPY5R/SLC6A1/SLC6A6/SLITRK3/SST	30	3.57142857142857

	ONTOLOGY	ID	Description	GeneRatio	BgRatio	pvalue	p.adjust	qvalue	geneID	Count	richFactor
GO:0070382	CC	GO:0070382	exocytic vesicle	45/673	225/19518	5.82701317067834e-22	1.23754660672502e-20	7.76935089423778e-21	ATP6V0A4/ATP6V1F/ATP6V1G2/BCL2L1/BDNF/DRD2/GRIA1/GRIN2A/HCRT/IGF1/KIRREL3/LRRK2/NGF/NTF4/OPRD1/PENK/RAB3B/RPH3A/SEPTIN5/SLC17A7/SLC17A8/SLC18A2/SLC18A3/SLC4A8/SLC6A9/SNAP25/SNCA/SNCAIP/STX1A/SV2B/SYN2/SYN3/SYP/SYT10/SYT11/SYT12/SYT2/SYT4/SYT5/SYT7/SYT8/SYT9/TH/UNC13C/VAMP2	45	2
GO:0043204	CC	GO:0043204	perikaryon	38/673	156/19518	6.43889728587929e-22	1.30534008613735e-20	8.19496018202818e-21	ADCYAP1/ASTN1/CACNA1C/CCR2/CNR2/CPLX1/CPNE5/DLG2/DRD2/EPHA4/GAP43/GLRA3/GRIK3/GRIK5/HPCA/KCNA1/KCNA2/KIF5A/LRRK2/MAP1B/NDN/NGFR/NPY/NSMF/NTSR1/OLFM1/OPRM1/PENK/PRPH/RTN4R/SCN1B/SLC17A8/SLC1A1/SLC5A7/SYT11/TH/UCN/VIP	38	2.43589743589744
GO:0008021	CC	GO:0008021	synaptic vesicle	43/673	210/19518	1.86548535100801e-21	3.61741941978074e-20	2.2710256447054e-20	ATP6V0A4/ATP6V1F/ATP6V1G2/BCL2L1/BDNF/DRD2/GRIA1/GRIN2A/HCRT/KIRREL3/LRRK2/NGF/NTF4/OPRD1/PENK/RAB3B/RPH3A/SEPTIN5/SLC17A7/SLC17A8/SLC18A2/SLC18A3/SLC4A8/SLC6A9/SNAP25/SNCA/SNCAIP/STX1A/SV2B/SYN2/SYN3/SYP/SYT11/SYT12/SYT2/SYT4/SYT5/SYT7/SYT8/SYT9/TH/UNC13C/VAMP2	43	2.04761904761905
GO:0031045	CC	GO:0031045	dense core granule	20/673	41/19518	5.84009732464407e-19	1.08528475282969e-17	6.81344687875142e-18	ADRB1/ADRB2/CADPS/CALCA/GRP/KIF1A/NPY/OPRD1/P2RX2/PENK/SCG2/SLC6A9/SPX/SST/SYT2/SYT4/SYT5/SYT7/SYT8/SYT9	20	4.8780487804878
GO:0030672	CC	GO:0030672	synaptic vesicle membrane	31/673	125/19518	2.33819502924256e-18	4.01090378093146e-17	2.51805618533814e-17	ATP6V0A4/ATP6V1F/ATP6V1G2/BCL2L1/DRD2/GRIA1/LRRK2/OPRD1/RAB3B/RPH3A/SLC17A7/SLC17A8/SLC18A2/SLC18A3/SLC4A8/SLC6A9/SNCA/STX1A/SV2B/SYN2/SYN3/SYP/SYT12/SYT2/SYT4/SYT5/SYT7/SYT8/SYT9/UNC13C/VAMP2	31	2.48
GO:0099501	CC	GO:0099501	exocytic vesicle membrane	31/673	125/19518	2.33819502924256e-18	4.01090378093146e-17	2.51805618533814e-17	ATP6V0A4/ATP6V1F/ATP6V1G2/BCL2L1/DRD2/GRIA1/LRRK2/OPRD1/RAB3B/RPH3A/SLC17A7/SLC17A8/SLC18A2/SLC18A3/SLC4A8/SLC6A9/SNCA/STX1A/SV2B/SYN2/SYN3/SYP/SYT12/SYT2/SYT4/SYT5/SYT7/SYT8/SYT9/UNC13C/VAMP2	31	2.48
GO:0060076	CC	GO:0060076	excitatory synapse	23/673	64/19518	6.21193251649737e-18	1.02611922309549e-16	6.44200409118246e-17	CALB2/CNTN6/GPM6A/GRIA1/GRIA2/GRIA3/GRID2/HOMER1/KCNJ9/NLGN1/NLGN3/NLGN4X/NLGN4Y/PALLD/SHISA7/SLC17A7/SLC17A8/SLC6A9/SPTBN2/STXBP1/SYP/SYT11/UNC13C	23	3.59375

	ONTOLOGY	ID	Description	GeneRatio	BgRatio	pvalue	p.adjust	qvalue	geneID	Count	richFactor
GO:0048786	CC	GO:0048786	presynaptic active zone	24/673	82/19518	2.64811000597205e-16	4.21806093808405e-15	2.64811000597205e-15	ADRA2A/ARHGAP44/CNTNAP1/FZD3/GABRA3/GABRB1/GAD1/GPER1/GPM6A/GRIN2D/HCN1/NTNG1/P2RX1/P2RY1/P2RY4/RIMS1/RIMS3/RIMS4/SLC17A7/STX1A/STXBP1/SYP/SYT11/UNC13C	24	2.92682926829268
GO:0043195	CC	GO:0043195	terminal bouton	19/673	47/19518	3.6000049200213e-16	5.53655929079137e-15	3.47586681933091e-15	CALB1/CALB2/CALCA/CPLX1/GRIK3/GRIK5/LRRK2/NMU/NPY/NTSR1/OXT/P2RX4/PRNP/SLC18A2/SLC18A3/SLC4A8/SYP/TH/UNC13C	19	4.04255319148936
GO:0030133	CC	GO:0030133	transport vesicle	52/673	425/19518	1.87994335921749e-15	2.79484912737001e-14	1.75461380193633e-14	ATP6V0A4/ATP6V1F/ATP6V1G2/BCL2L1/BDNF/CNIH3/DRD2/GRIA1/GRIN2A/HCRT/IGF1/ITPR1/KCNQ1/KIRREL3/LRRK2/NGF/NPTX1/NTF4/OPRD1/PENK/RAB3B/RPH3A/SEPTIN5/SLC17A7/SLC17A8/SLC18A2/SLC18A3/SLC2A4/SLC4A8/SLC6A9/SNAP25/SNCA/SNCAIP/SPX/STX1A/SV2B/SYN2/SYN3/SYP/SYT10/SYT11/SYT12/SYT2/SYT4/SYT5/SYT7/SYT8/SYT9/TH/UNC13C/VAMP2/VGF	52	1.22352941176471
GO:0043197	CC	GO:0043197	dendritic spine	32/673	175/19518	8.27035486228131e-15	1.18986395760563e-13	7.46999794012505e-14	ARHGAP44/ATP1A2/CALB1/CAMK2A/DRD2/EPHA4/EPHB2/GPER1/GPM6A/GRIA1/GRIA2/GRIA3/GRIA4/GRID2/GRIN2A/HOMER1/HPCA/LPAR1/MAP1B/MAPT/NGFR/NLGN1/NOS1/NTSR1/OPRD1/OPRM1/P2RX4/PTK2B/RPH3A/SHISA7/SLC1A1/SYT11	32	1.82857142857143
GO:0044309	CC	GO:0044309	neuron spine	32/673	178/19518	1.36464356377693e-14	1.90197196701409e-13	1.19406311830481e-13	ARHGAP44/ATP1A2/CALB1/CAMK2A/DRD2/EPHA4/EPHB2/GPER1/GPM6A/GRIA1/GRIA2/GRIA3/GRIA4/GRID2/GRIN2A/HOMER1/HPCA/LPAR1/MAP1B/MAPT/NGFR/NLGN1/NOS1/NTSR1/OPRD1/OPRM1/P2RX4/PTK2B/RPH3A/SHISA7/SLC1A1/SYT11	32	1.79775280898876
GO:0098992	CC	GO:0098992	neuronal dense core vesicle	14/673	28/19518	7.544781033698e-14	1.0196885881907e-12	6.4016323922286e-13	ADRB1/ADRB2/CADPS/CALCA/GRP/KIF1A/NPY/OPRD1/P2RX2/PENK/SCG2/SST/SYT4/SYT5	14	5
GO:0044304	CC	GO:0044304	main axon	19/673	64/19518	2.59801883202388e-13	3.40798940906662e-12	2.13954492049025e-12	ANK3/CNTN2/CNTNAP1/DLG2/KCNA1/KCNA2/LGI1/LRRC7/MAG/MAP1B/MAP2/MAPT/NRCAM/ROBO2/SCN1B/SLC1A2/TUBB4A/UCN/UCN3	19	2.96875
GO:0098802	CC	GO:0098802	plasma membrane signaling receptor complex	41/673	319/19518	3.81268357887974e-13	4.85844821765818e-12	3.05014686310379e-12	CACNG4/CACNG5/CACNG7/CACNG8/CHRFAM7A/CHRNA1/CHRNA3/CHRNA4/CHRNA6/CHRNA7/CHRNB2/CHRNB4/CHRND/CNIH3/CNTFR/GPR156/GRIA1/GRIA2/GRIA3/GRIA4/GRID2/GRIK1/GRIK2/GRIK3/GRIK5/GRIN2A/GRIN2B/GRIN2D/GRIN3A/GRIN3B/HTR3A/HTR3C/HTR3E/IL6R/IL6ST/ITGA4/ITGB3/OLFM3/PTK2B/SHISA7/VWC2	41	1.28526645768025

	ONTOLOGY	ID	Description	GeneRatio	BgRatio	pvalue	p.adjust	qvalue	geneID	Count	richFactor
GO:0030658	CC	GO:0030658	transport vesicle membrane	34/673	234/19518	1.23788394109569e-12	1.53360066035744e-11	9.62798620852203e-12	ATP6V0A4/ATP6V1F/ATP6V1G2/BCL2L1/CNIH3/DRD2/GRIA1/ITPR1/LRRK2/OPRD1/RAB3B/RPH3A/SLC17A7/SLC17A8/SLC18A2/SLC18A3/SLC4A8/SLC6A9/SNCA/STX1A/SV2B/SYN2/SYN3/SYP/SYT10/SYT12/SYT2/SYT4/SYT5/SYT7/SYT8/SYT9/UNC13C/VAMP2	34	1.45299145299145
GO:0030426	CC	GO:0030426	growth cone	28/673	167/19518	3.64896949081605e-12	4.39848754838907e-11	2.76138231737431e-11	CDK5R2/CNR1/CSNK1E/EPHA4/FGF13/GAP43/GPM6A/HSP90AB1/ITGA4/KIF5C/L1CAM/LRRK2/MAP1B/MAP2/MAPT/NEFL/NEO1/NGFR/OLFM1/PALLD/PINK1/PTK2B/RTN4R/SIGMAR1/SNAP25/SNCA/TUBB3/UNC5C	28	1.67664670658683
GO:0098685	CC	GO:0098685	Schaffer collateral - CA1 synapse	21/673	94/19518	6.41280586000308e-12	7.52660898305625e-11	4.72522537052859e-11	ADORA3/ADRB1/CPLX1/EPHA4/GABRB1/GSG1L/ITPR1/NEFH/NEFL/NTNG1/PLCG1/PTK2B/PTN/SLC1A1/STX1A/SYN2/SYP/TNR/TUBB2B/WNT5A/WNT7A	21	2.23404255319149
GO:0030427	CC	GO:0030427	site of polarized growth	28/673	172/19518	7.62881607831884e-12	8.72423582289795e-11	5.47709872289557e-11	CDK5R2/CNR1/CSNK1E/EPHA4/FGF13/GAP43/GPM6A/HSP90AB1/ITGA4/KIF5C/L1CAM/LRRK2/MAP1B/MAP2/MAPT/NEFL/NEO1/NGFR/OLFM1/PALLD/PINK1/PTK2B/RTN4R/SIGMAR1/SNAP25/SNCA/TUBB3/UNC5C	28	1.62790697674419
GO:0045121	CC	GO:0045121	membrane raft	39/673	323/19518	1.03062356797144e-11	1.14914527828815e-10	7.21436497580006e-11	ADCY2/ADCYAP1R1/ADGRG1/ADRA1A/ADRA1B/ATP1A2/CAPN2/CAV1/CHRNA3/CHRNA7/CHRNB2/CNR1/EDNRB/EFNA5/FAS/GPM6B/IL6ST/KCNQ1/LRRK2/MAG/MAPT/NOS1/NTSR1/OPRD1/OPRM1/P2RX1/PDPN/PRNP/PTK2B/RTN4R/S1PR1/SHH/SLC1A1/SLC1A2/SLC2A4/SLC6A2/SLC6A3/SULF1/TNR	39	1.20743034055728
GO:0098857	CC	GO:0098857	membrane microdomain	39/673	324/19518	1.13422623496167e-11	1.2338168312022e-10	7.74593526315285e-11	ADCY2/ADCYAP1R1/ADGRG1/ADRA1A/ADRA1B/ATP1A2/CAPN2/CAV1/CHRNA3/CHRNA7/CHRNB2/CNR1/EDNRB/EFNA5/FAS/GPM6B/IL6ST/KCNQ1/LRRK2/MAG/MAPT/NOS1/NTSR1/OPRD1/OPRM1/P2RX1/PDPN/PRNP/PTK2B/RTN4R/S1PR1/SHH/SLC1A1/SLC1A2/SLC2A4/SLC6A2/SLC6A3/SULF1/TNR	39	1.2037037037037
GO:0032281	CC	GO:0032281	AMPA glutamate receptor complex	12/673	26/19518	1.59010914952164e-11	1.68854447782536e-10	1.06007276634776e-10	CACNG4/CACNG5/CACNG7/CACNG8/CNIH3/GRIA1/GRIA2/GRIA3/GRIA4/OLFM3/SHISA7/VWC2	12	4.61538461538462
GO:0048787	CC	GO:0048787	presynaptic active zone membrane	13/673	34/19518	4.13398098796439e-11	4.28780353635376e-10	2.69189459681402e-10	ADRA2A/GABRA3/GABRB1/GPM6A/GRIN2D/HCN1/NTNG1/P2RX1/P2RY1/P2RY4/STX1A/STXBP1/SYT11	13	3.82352941176471

	ONTOLOGY	ID	Description	GeneRatio	BgRatio	pvalue	p.adjust	qvalue	geneID	Count	richFactor
GO:0034703	CC	GO:0034703	cation channel complex	28/673	192/19518	1.11864186389856e-10	1.13389607113354e-09	7.11863004299082e-10	ATP2A1/CACNA1A/CACNA1C/CACNA1D/CACNG4/CACNG7/CACNG8/FKBP1B/GRIK1/GRIK2/GRIK3/GRIK5/HCN1/HTR3A/HTR3C/HTR3E/KCNA1/KCNA2/KCNJ14/KCNJ5/KCNMB2/KCNQ1/KCNQ5/RYR1/RYR3/SCN1B/SCN2B/TRPC1	28	1.45833333333333
GO:0098688	CC	GO:0098688	parallel fiber to Purkinje cell synapse	10/673	19/19518	1.55107653586016e-10	1.53728918887474e-09	9.65114288979655e-10	CALB2/CNTN6/GPM6A/GRIA3/GRID2/KCNJ9/SLC6A9/SPTBN2/STXBP1/UNC13C	10	5.26315789473684
GO:0098691	CC	GO:0098691	dopaminergic synapse	9/673	15/19518	2.71695202073693e-10	2.63426217662755e-09	1.6537968821877e-09	ADRA1A/ADRA2A/CHRNA6/DRD2/PRKN/RAB3B/SLC18A2/SLC6A3/SYT11	9	6
GO:0005892	CC	GO:0005892	acetylcholine-gated channel complex	9/673	16/19518	6.02026165692381e-10	5.71284404040004e-09	3.58653885944397e-09	CHRFAM7A/CHRNA1/CHRNA3/CHRNA4/CHRNA6/CHRNA7/CHRNB2/CHRNB4/CHRND	9	5.625
GO:0042383	CC	GO:0042383	sarcolemma	22/673	136/19518	1.52455389115507e-09	1.41656465719825e-08	8.89323103173792e-09	ADRA1A/ANK3/ATP1A2/CACNA1C/CACNA1D/CACNG4/CACNG7/CACNG8/CAMK2D/CAV1/CLCN1/FGF13/GHRHR/NOS1/OPRM1/RYR1/RYR3/SCN1B/SCN2B/SLC2A4/SNTG2/VCL	22	1.61764705882353
GO:0034707	CC	GO:0034707	chloride channel complex	14/673	55/19518	3.45553596728447e-09	3.14524294165077e-08	1.97459198130541e-08	CLCN1/GABRA3/GABRA4/GABRB1/GABRB3/GABRD/GABRE/GABRG2/GABRP/GABRR1/GLRA3/GLRB/SLC17A7/SLC17A8	14	2.54545454545454
GO:0030666	CC	GO:0030666	endocytic vesicle membrane	26/673	196/19518	4.10711216028322e-09	3.66354404697263e-08	2.2999828097586e-08	ADRB2/ATP6V0A4/ATP6V0D2/CACNG4/CACNG8/CAMK2A/CAMK2B/CAMK2D/CAV1/CHRM2/GRIA1/GRIA2/GRIA3/GRIA4/SH3GL2/SLC18A3/SYT2/SYT7/SYT9/VAMP2/WNT1/WNT3/WNT3A/WNT5A/WNT7A/WNT7B	26	1.3265306122449
GO:1902711	CC	GO:1902711	GABA-A receptor complex	9/673	19/19518	4.42910112871163e-09	3.87329235961841e-08	2.43166336478286e-08	GABRA3/GABRA4/GABRB1/GABRB3/GABRD/GABRE/GABRG2/GABRP/GABRR1	9	4.73684210526316
GO:0060077	CC	GO:0060077	inhibitory synapse	9/673	20/19518	7.80692471843376e-09	6.69593927773357e-08	4.20372869454126e-08	GABRG2/GAD1/IGSF9/LHFPL4/NLGN1/NLGN3/NLGN4X/NLGN4Y/SYT11	9	4.5
GO:1902710	CC	GO:1902710	GABA receptor complex	9/673	21/19518	1.3244912331593e-08	1.11457186790387e-07	6.99731217518122e-08	GABRA3/GABRA4/GABRB1/GABRB3/GABRD/GABRE/GABRG2/GABRP/GABRR1	9	4.28571428571429
GO:0032280	CC	GO:0032280	symmetric synapse	7/673	11/19518	1.64362821369963e-08	1.35751515427784e-07	8.52251666362769e-08	CHRM2/NLGN3/NLGN4X/NLGN4Y/NTSR1/PENK/SLC4A8	7	6.36363636363636
GO:0019898	CC	GO:0019898	extrinsic component of membrane	30/673	288/19518	7.77917554537111e-08	6.30820416951912e-07	3.96030755037075e-07	APC/BECN2/CDH4/CNR2/CNTFR/GFRA1/GFRA2/GFRA3/GNA11/GNA13/GNAI1/GNAL/GNAO1/GNAQ/GNAZ/GNG13/GNG2/HPCA/KIF1A/MGLL/PIK3CD/PIK3CG/PIK3R2/PIK3R5/PRNP/RPH3A/SNAP25/STXBP1/SYN3/WNT2	30	1.04166666666667
GO:0098686	CC	GO:0098686	hippocampal mossy fiber to CA3 synapse	11/673	41/19518	9.28804751338496e-08	7.39726641244588e-07	4.64402375669248e-07	CALB1/CALCA/EPHB2/GPER1/GRIK2/GRIK5/GRIN2D/SLC6A9/SYT12/SYT7/SYT9	11	2.68292682926829

	ONTOLOGY	ID	Description	GeneRatio	BgRatio	pvalue	p.adjust	qvalue	geneID	Count	richFactor
GO:0032589	CC	GO:0032589	neuron projection membrane	13/673	60/19518	1.00733676474989e-07	7.88196836979739e-07	4.94832094964858e-07	GABRA3/GABRA4/GABRE/GABRG2/GPER1/GRIA1/HPCA/MAPT/OPRD1/OPRM1/ROBO2/SHISA7/SLC1A2	13	2.16666666666667
GO:0005834	CC	GO:0005834	heterotrimeric G-protein complex	9/673	26/19518	1.20589124135728e-07	9.27288782147151e-07	5.82154392379377e-07	GNA11/GNA13/GNAI1/GNAL/GNAO1/GNAQ/GNAZ/GNG13/GNG2	9	3.46153846153846
GO:0031594	CC	GO:0031594	neuromuscular junction	14/673	73/19518	1.65378047396842e-07	1.25014591761002e-06	7.84844970696876e-07	ANK3/CHRNA1/CHRND/DLG2/EPHA4/ERBB4/NEFL/NGFR/NLGN1/P2RX7/SLC5A7/SYP/TUBA1A/UNC13C	14	1.91780821917808
GO:0043194	CC	GO:0043194	axon initial segment	8/673	21/19518	2.62022938368746e-07	1.94770384187434e-06	1.22277371238748e-06	ANK3/CNTN2/KCNA1/KCNA2/LGI1/LRRC7/MAP2/NRCAM	8	3.80952380952381
GO:0097440	CC	GO:0097440	apical dendrite	7/673	15/19518	2.83984226657992e-07	2.07634368999122e-06	1.30353415515144e-06	CLU/MAP1B/MAP2/NSMF/PTK2B/SLC17A8/SLC1A1	7	4.66666666666667
GO:0120111	CC	GO:0120111	neuron projection cytoplasm	15/673	89/19518	3.54829594887862e-07	2.55248385999978e-06	1.60245623497744e-06	CALY/DLG2/GRIK3/HPCA/KIF1A/KIF5A/KIF5C/LRRK2/MAP2/MAPT/NEFL/OPRM1/TMEM108/TRAK2/UCHL1	15	1.68539325842697
GO:0043198	CC	GO:0043198	dendritic shaft	10/673	38/19518	4.38336636694867e-07	3.05465843696735e-06	1.91772278554004e-06	CNIH3/EPHA4/GPER1/HOMER1/KIRREL3/LPAR1/MAP2/NTSR1/RTN4R/SLC1A1	10	2.63157894736842
GO:0097386	CC	GO:0097386	glial cell projection	10/673	38/19518	4.38336636694867e-07	3.05465843696735e-06	1.91772278554004e-06	ADGRG1/GRM2/MAPT/PINK1/SLC17A8/SLC1A1/SLC1A2/SLC4A8/SLC7A11/SYT4	10	2.63157894736842
GO:0030139	CC	GO:0030139	endocytic vesicle	32/673	347/19518	4.73421934493709e-07	3.24840281206452e-06	2.03935602551136e-06	ADRB2/ATP6V0A4/ATP6V0D2/CACNG4/CACNG8/CAMK2A/CAMK2B/CAMK2D/CAV1/CHRM2/DRD2/GRIA1/GRIA2/GRIA3/GRIA4/LPAR1/NLGN3/PLD1/SH3GL2/SLC18A3/STXBP1/SYT11/SYT2/SYT7/SYT9/VAMP2/WNT1/WNT3/WNT3A/WNT5A/WNT7A/WNT7B	32	0.922190201729107
GO:0099568	CC	GO:0099568	cytoplasmic region	28/673	283/19518	6.06028600065696e-07	4.09528417620152e-06	2.57103042452113e-06	CALY/DCDC2/DLG2/DNAH10/DNAH12/DNAH2/DNAH5/GABARAP/GNAI1/GRIK3/HPCA/KIF1A/KIF5A/KIF5C/LRRK2/MAP2/MAPT/NEFL/OPRM1/RIMS1/RIMS3/RIMS4/STXBP1/TMEM108/TRAK2/TUBB4A/UCHL1/UNC13C	28	0.989399293286219
GO:0044295	CC	GO:0044295	axonal growth cone	8/673	24/19518	8.63934779208034e-07	5.75096882875796e-06	3.61047370415298e-06	EPHA4/GPM6A/HSP90AB1/KIF5C/L1CAM/NEO1/OLFM1/RTN4R	8	3.33333333333333
GO:1905360	CC	GO:1905360	GTPase complex	9/673	32/19518	8.99143793218565e-07	5.89732546728647e-06	3.70235679560585e-06	GNA11/GNA13/GNAI1/GNAL/GNAO1/GNAQ/GNAZ/GNG13/GNG2	9	2.8125
GO:0032590	CC	GO:0032590	dendrite membrane	10/673	41/19518	9.4674136853854e-07	6.11951667200274e-06	3.8418490317506e-06	GABRA3/GABRA4/GABRE/GABRG2/GPER1/GRIA1/HPCA/OPRD1/OPRM1/SHISA7	10	2.4390243902439
GO:0030315	CC	GO:0030315	T-tubule	11/673	52/19518	1.25733288831653e-06	8.01100668841678e-06	5.02933155326614e-06	ADRA1A/ANK3/ATP1A2/CACNA1C/CACNA1D/CACNG4/CACNG7/CACNG8/SCN1B/SCN2B/SLC2A4	11	2.11538461538462
GO:0044305	CC	GO:0044305	calyx of Held	7/673	19/19518	1.97073273573543e-06	1.22075944463611e-05	7.66396063897112e-06	CALB1/CPLX1/KCNA1/KCNA2/PRKCB/PRKCG/UNC13C	7	3.68421052631579
GO:0097449	CC	GO:0097449	astrocyte projection	7/673	19/19518	1.97073273573543e-06	1.22075944463611e-05	7.66396063897112e-06	ADGRG1/GRM2/PINK1/SLC17A8/SLC1A2/SLC7A11/SYT4	7	3.68421052631579

	ONTOLOGY	ID	Description	GeneRatio	BgRatio	pvalue	p.adjust	qvalue	geneID	Count	richFactor
GO:0045177	CC	GO:0045177	apical part of cell	36/673	446/19518	2.23701305469736e-06	1.36672304437674e-05	8.58032404541454e-06	ADRB2/ASPM/ATP6V0A4/ATP6V0D2/BYSL/CHL1/CLDN1/CNTFR/F2RL2/FZD3/GPIHBP1/GPR143/HOMER1/IL6R/ITGB3/KCNA1/KCNQ1/KISS1/OXTR/P2RX2/P2RY1/P2RY4/PDPN/PLD1/PRKG2/PTH1R/SLC1A1/SLC22A2/SLC29A1/SLC38A3/SLC44A4/SLC4A8/SLC6A6/SLC6A9/SLC7A11/SPTBN2	36	0.807174887892377
GO:0032839	CC	GO:0032839	dendrite cytoplasm	8/673	28/19518	3.23078639660182e-06	1.94720369308704e-05	1.22245971763312e-05	GRIK3/HPCA/KIF5A/KIF5C/LRRK2/MAP2/OPRM1/TRAK2	8	2.85714285714286
GO:0016324	CC	GO:0016324	apical plasma membrane	32/673	381/19518	3.5775341017556e-06	2.12744027917733e-05	1.33561273132209e-05	ADRB2/ASPM/ATP6V0A4/ATP6V0D2/CLDN1/CNTFR/F2RL2/FZD3/GPIHBP1/GPR143/IL6R/ITGB3/KCNA1/KCNQ1/KISS1/OXTR/P2RX2/P2RY1/P2RY4/PDPN/PLD1/PRKG2/PTH1R/SLC1A1/SLC22A2/SLC29A1/SLC38A3/SLC44A4/SLC4A8/SLC6A6/SLC6A9/SPTBN2	32	0.83989501312336
GO:0031256	CC	GO:0031256	leading edge membrane	20/673	180/19518	4.26735193672385e-06	2.50426179444584e-05	1.57218229247721e-05	APC/ARHGAP44/GABRA3/GABRA4/GABRE/GABRG2/GPER1/GRIA1/HPCA/ITGB3/KCNA2/MAPT/OPRD1/OPRM1/PDPN/PLCG1/PLCG2/ROBO2/SHISA7/SLC1A2	20	1.11111111111111
GO:0031253	CC	GO:0031253	cell projection membrane	30/673	349/19518	4.53241083114396e-06	2.6252665333639e-05	1.64814939314326e-05	ADCY3/APC/ATP6V0A4/DRD2/DRD5/FZD9/GABRA3/GABRA4/GABRE/GABRG2/GAP43/GNA13/GPER1/GRIA1/HPCA/ITGB3/KCNA2/MAPT/OPRD1/OPRM1/PDPN/PLCG1/PLCG2/ROBO2/SHISA7/SLC1A2/SLC28A2/SLC6A6/SLC7A11/SSTR3	30	0.859598853868195
GO:0098981	CC	GO:0098981	cholinergic synapse	5/673	11/19518	1.86651253334248e-05	0.000106726229470609	6.70030140174224e-05	CHRM2/CHRNB2/CHRNB4/NEFL/PLD1	5	4.54545454545454
GO:0016323	CC	GO:0016323	basolateral plasma membrane	22/673	234/19518	2.14306077052437e-05	0.000120987987804287	7.59565842717497e-05	ADRA2A/ANK3/ATP6V0A4/AURKA/CLDN1/CLDN19/ERBB4/GPIHBP1/KCNQ1/LEPR/LRRK2/P2RY1/P2RY4/PDPN/PTH1R/SLC22A2/SLC29A1/SLC38A3/SLC4A8/SLC6A6/SLC6A9/TSHR	22	0.94017094017094
GO:0034706	CC	GO:0034706	sodium channel complex	6/673	19/19518	3.03599289435356e-05	0.00016705635807916	0.000104878431081087	GRIK1/GRIK2/GRIK3/GRIK5/SCN1B/SCN2B	6	3.15789473684211
GO:0099522	CC	GO:0099522	cytosolic region	6/673	19/19518	3.03599289435356e-05	0.00016705635807916	0.000104878431081087	CALB1/HOMER1/LRRK2/PRKCB/PRKCG/STXBP1	6	3.15789473684211
GO:0060198	CC	GO:0060198	clathrin-sculpted vesicle	5/673	12/19518	3.10889634990364e-05	0.00016705635807916	0.000104878431081087	GAD1/SLC17A7/SLC18A2/SLC18A3/VAMP2	5	4.16666666666667
GO:1990454	CC	GO:1990454	L-type voltage-gated calcium channel complex	5/673	12/19518	3.10889634990364e-05	0.00016705635807916	0.000104878431081087	CACNA1C/CACNA1D/CACNG4/CACNG7/CACNG8	5	4.16666666666667
GO:0009897	CC	GO:0009897	external side of plasma membrane	32/673	426/19518	3.41908178348847e-05	0.000181536961361412	0.000113969392782949	ASTN1/CAPN2/CHRNA4/CHRNB2/CNTFR/CRLF1/CXCL12/EFNA5/F2/FAS/GFRA1/GFRA2/GFRA3/GHR/GPIHBP1/GRIA2/IL6R/IL6ST/ITGA4/ITGB3/LEPR/LIFR/LY6G6D/NCAM1/NRCAM/P2RX1/P2RX7/PRNP/RTN4R/S1PR1/SLC2A4/WNT2	32	0.751173708920188

	ONTOLOGY	ID	Description	GeneRatio	BgRatio	pvalue	p.adjust	qvalue	geneID	Count	richFactor
GO:0016529	CC	GO:0016529	sarcoplasmic reticulum	11/673	73/19518	3.841398797383 83e-05	0.000201560454 545081	0.000126540195 678526	ANK3/ATP2A1/ATP2A3/CAMK2B/CAMK2D/FKBP1B/ITPR1/NOS1/RYR1/RYR3/SLC2A4	11	1.506849315068 49
GO:0032838	CC	GO:0032838	plasma membrane bounded cell projection cytoplasm	22/673	244/19518	4.071621484553 05e-05	0.000211156183 966356	0.000132564420 427309	CALY/DCDC2/DLG2/DNAH10/DNAH12/DNAH2/DNAH5/GABARAP/GRIK3/HPCA/KIF1A/KIF5A/KIF5C/LRRK2/MAP2/MAPT/NEFL/OPRM1/TMEM108/TRAK2/TUBB4A/UCHL1	22	0.901639344262 295
GO:0032809	CC	GO:0032809	neuronal cell body membrane	7/673	29/19518	4.518334163966 47e-05	0.000231629544 497591	0.000145417651 254093	CX3CR1/FLRT1/HPCA/KCNA2/SLC4A8/SLC6A2/SLC6A3	7	2.413793103448 28
GO:0099523	CC	GO:0099523	presynaptic cytosol	5/673	13/19518	4.908652297025 99e-05	0.000248779423 235636	0.000156184391 269009	CALB1/LRRK2/PRKCB/PRKCG/STXBP1	5	3.846153846153 85
GO:0034705	CC	GO:0034705	potassium channel complex	12/673	89/19518	5.359610600184 44e-05	0.000268582733 447445	0.000168616962 702432	GRIK1/GRIK2/GRIK3/GRIK5/HCN1/KCNA1/KCNA2/KCNJ14/KCNJ5/KCNMB2/KCNQ1/KCNQ5	12	1.348314606741 57
GO:1904115	CC	GO:1904115	axon cytoplasm	10/673	63/19518	5.449999112056 11e-05	0.000270077733 775225	0.000169555527 930635	CALY/DLG2/KIF1A/KIF5A/KIF5C/MAPT/NEFL/TMEM108/TRAK2/UCHL1	10	1.587301587301 59
GO:0016528	CC	GO:0016528	sarcoplasm	12/673	90/19518	5.993398493024 85e-05	0.000293742387 680119	0.000184412261 323842	ANK3/ATP2A1/ATP2A3/CAMK2B/CAMK2D/FKBP1B/ITPR1/MEF2C/NOS1/RYR1/RYR3/SLC2A4	12	1.333333333333 33
GO:0030175	CC	GO:0030175	filopodium	13/673	108/19518	8.812650380145 18e-05	0.000427084893 399866	0.000268125045 183772	EPHA4/FGF13/FZD3/FZD9/GAP43/GPM6A/ITGB3/LY6G6D/MAP2/NLGN1/PDPN/TUBB3/UNC5C	13	1.203703703703 7
GO:0044298	CC	GO:0044298	cell body membrane	7/673	32/19518	8.905581857889 57e-05	0.000427084893 399866	0.000268125045 183772	CX3CR1/FLRT1/HPCA/KCNA2/SLC4A8/SLC6A2/SLC6A3	7	2.1875
GO:0031234	CC	GO:0031234	extrinsic component of cytoplasmic side of plasma membrane	11/673	80/19518	9.094188337223 14e-05	0.000431490212 595906	0.000270890716 427923	CNR2/GNA11/GNA13/GNAI1/GNAL/GNAO1/GNAQ/GNAZ/GNG13/GNG2/SNAP25	11	1.375
GO:0009925	CC	GO:0009925	basal plasma membrane	22/673	266/19518	0.000145700818 244147	0.000684026999 335681	0.000429433990 614329	ADRA2A/ANK3/ATP6V0A4/AURKA/CLDN1/CLDN19/ERBB4/GPIHBP1/KCNQ1/LEPR/LRRK2/P2RY1/P2RY4/PDPN/PTH1R/SLC22A2/SLC29A1/SLC38A3/SLC4A8/SLC6A6/SLC6A9/TSHR	22	0.827067669172 932
GO:0098985	CC	GO:0098985	asymmetric, glutamatergic, excitatory synapse	5/673	16/19518	0.000152840417 831623	0.000710071107 842747	0.000445784552 0089	NLGN1/NLGN3/NLGN4X/NLGN4Y/SHISA7	5	3.125
GO:0019897	CC	GO:0019897	extrinsic component of plasma membrane	15/673	147/19518	0.000173883017 816374	0.000799503360 269101	0.000501930360 707059	APC/CDH4/CNR2/GNA11/GNA13/GNAI1/GNAL/GNAO1/GNAQ/GNAZ/GNG13/GNG2/SNAP25/STXBP1/WNT2	15	1.020408163265 31
GO:0016234	CC	GO:0016234	inclusion body	10/673	74/19518	0.000218056078 269008	0.000992377662 3263	0.000623017366 482879	CLU/CPNE1/LRRK2/MAPT/NEFH/NEFM/PINK1/PRKN/PRNP/SNCA	10	1.351351351351 35
GO:0044224	CC	GO:0044224	juxtaparanode region of axon	4/673	10/19518	0.000249194093 627499	0.001111405657 57865	0.000697743462 156998	CNTN2/DLG2/KCNA1/KCNA2	4	4
GO:0099571	CC	GO:0099571	postsynaptic cytoskeleton	4/673	10/19518	0.000249194093 627499	0.001111405657 57865	0.000697743462 156998	NEFH/NEFL/NEFM/SPTBN2	4	4
GO:0030665	CC	GO:0030665	clathrin-coated vesicle membrane	14/673	136/19518	0.000258522145 252416	0.001141592839 43146	0.000716695056 145312	ADRB2/ATP6V1F/ATP6V1G2/CHRM2/GAD1/SH3GL2/SLC17A7/SLC18A2/SLC18A3/SYT11/SYT2/SYT9/VAMP2/WNT5A	14	1.029411764705 88
GO:0098831	CC	GO:0098831	presynaptic active zone cytoplasmic component	5/673	18/19518	0.000283098036 248274	0.001237860040 8503	0.000777131864 210948	RIMS1/RIMS3/RIMS4/STXBP1/UNC13C	5	2.777777777777 78

	ONTOLOGY	ID	Description	GeneRatio	BgRatio	pvalue	p.adjust	qvalue	geneID	Count	richFactor
GO:0005796	CC	GO:0005796	Golgi lumen	12/673	106/19518	0.000291989332874919	0.00126434215982732	0.000793757409757061	CGA/F2/GPC6/LHB/MMP11/NGF/WNT1/WNT3/WNT3A/WNT5A/WNT7A/WNT7B	12	1.13207547169811
GO:0005875	CC	GO:0005875	microtubule associated complex	15/673	156/19518	0.000333374827182385	0.00142966512426292	0.000897547611644883	AURKA/DCX/DNAH10/DNAH12/DNAH14/DNAH2/DNAH5/GABARAP/KIF14/KIF1A/KIF5A/KIF5C/KLC3/MAP1B/MAP2	15	0.961538461538462
GO:0034704	CC	GO:0034704	calcium channel complex	10/673	79/19518	0.000374819802090802	0.00158586777920852	0.000995612058695931	ATP2A1/CACNA1A/CACNA1C/CACNA1D/CACNG4/CACNG7/CACNG8/FKBP1B/RYR1/RYR3	10	1.26582278481013
GO:0002116	CC	GO:0002116	semaphorin receptor complex	4/673	11/19518	0.000380924932652685	0.00158586777920852	0.000995612058695931	NRP2/PLXNA1/PLXNA2/PLXNA3	4	3.63636363636364
GO:0099569	CC	GO:0099569	presynaptic cytoskeleton	4/673	11/19518	0.000380924932652685	0.00158586777920852	0.000995612058695931	NEFL/RIMS1/RIMS3/RIMS4	4	3.63636363636364
GO:0045178	CC	GO:0045178	basal part of cell	22/673	285/19518	0.00038402179406843	0.00158586777920852	0.000995612058695931	ADRA2A/ANK3/ATP6V0A4/AURKA/CLDN1/CLDN19/ERBB4/GPIHBP1/KCNQ1/LEPR/LRRK2/P2RY1/P2RY4/PDPN/PTH1R/SLC22A2/SLC29A1/SLC38A3/SLC4A8/SLC6A6/SLC6A9/TSHR	22	0.771929824561404
GO:0031965	CC	GO:0031965	nuclear membrane	23/673	308/19518	0.000456628925023067	0.00186840826202099	0.00117299173400421	ADRA1A/ADRA1B/ATP2A3/BCL2/BCL2L1/CCND1/CPNE1/EDNRB/EPHA3/GCHFR/GHRHR/GNAQ/ITPR1/NRXN1/NSMF/P2RX1/P2RX2/P2RX4/P2RX7/PRKG2/PRNP/SIGMAR1/STX1A	23	0.746753246753247
GO:0033017	CC	GO:0033017	sarcoplasmic reticulum membrane	7/673	42/19518	0.000529419558137531	0.00214655566299399	0.00134761342071372	ATP2A1/ATP2A3/CAMK2B/CAMK2D/FKBP1B/RYR1/RYR3	7	1.66666666666667
GO:0005874	CC	GO:0005874	microtubule	30/673	461/19518	0.000701586589940621	0.00281898755958123	0.00176976797462499	APC/ASPM/AURKA/CDK1/DCDC2/DCX/DNAH10/DNAH12/DNAH14/DNAH2/DNAH5/FGF13/GABARAP/KIF14/KIF1A/KIF5A/KIF5C/KLC3/MAP1B/MAP1LC3C/MAP2/MAPT/SCTR/TUBA1A/TUBAL3/TUBB2A/TUBB2B/TUBB3/TUBB4A/TUBB6	30	0.650759219088937
GO:1990909	CC	GO:1990909	Wnt signalosome	4/673	13/19518	0.000781067981344731	0.00311032428285491	0.00195266995336183	APC/LRRK2/WNT2/WNT3	4	3.07692307692308
GO:0009898	CC	GO:0009898	cytoplasmic side of plasma membrane	14/673	159/19518	0.00124003096851317	0.00489428152174226	0.00307264310782025	CNR2/GNA11/GNA13/GNAI1/GNAL/GNAO1/GNAQ/GNAZ/GNG13/GNG2/NTSR1/RGS2/SNAP25/TH	14	0.880503144654088
GO:0016471	CC	GO:0016471	vacuolar proton-transporting V-type ATPase complex	5/673	25/19518	0.00143747976956931	0.00562382436164835	0.00353065206560883	ATP6V0A4/ATP6V0D2/ATP6V1C2/ATP6V1F/ATP6V1G2	5	2
GO:0016328	CC	GO:0016328	lateral plasma membrane	8/673	65/19518	0.00170965472802938	0.00663048703218349	0.00416263759868022	ANK3/APC/CLDN1/DRD2/FGF13/FZD3/NSG1/SLC6A9	8	1.23076923076923
GO:0030662	CC	GO:0030662	coated vesicle membrane	16/673	202/19518	0.00176465986899448	0.00678481294458223	0.00425952382171081	ADRB2/ATP6V1F/ATP6V1G2/CHRM2/CNIH3/GAD1/GRIA1/SH3GL2/SLC17A7/SLC18A2/SLC18A3/SYT11/SYT2/SYT9/VAMP2/WNT5A	16	0.792079207920792
GO:0005635	CC	GO:0005635	nuclear envelope	30/673	490/19518	0.00181875678300404	0.00693303867709232	0.00435258033539428	ADRA1A/ADRA1B/ATP2A3/BCHE/BCL2/BCL2L1/CCND1/CPNE1/EDNRB/EPHA3/GABRB1/GCHFR/GHRHR/GNAQ/GNAZ/GPER1/ITPR1/MAD2L1/NRXN1/NSMF/P2RX1/P2RX2/P2RX4/P2RX7/PRKG2/PRNP/PTGER3/SIGMAR1/STX1A/TYRO3	30	0.612244897959184

	ONTOLOGY	ID	Description	GeneRatio	BgRatio	pvalue	p.adjust	qvalue	geneID	Count	richFactor
GO:0044853	CC	GO:0044853	plasma membrane raft	11/673	114/19518	0.00194791766562486	0.0073624684649889	0.00462217751165222	ADCYAP1R1/ADRA1A/ADRA1B/ATP1A2/CAV1/CHRNA3/CHRNA7/CHRNB2/EFNA5/LRRK2/SLC6A3	11	0.964912280701754
GO:0099738	CC	GO:0099738	cell cortex region	6/673	39/19518	0.00203134519699807	0.00761327695681629	0.00477963575764251	GNAI1/RIMS1/RIMS3/RIMS4/STXBP1/UNC13C	6	1.53846153846154
GO:0033176	CC	GO:0033176	proton-transporting V-type ATPase complex	5/673	27/19518	0.00206347844913598	0.00766926156928872	0.00481478304798395	ATP6V0A4/ATP6V0D2/ATP6V1C2/ATP6V1F/ATP6V1G2	5	1.85185185185185
GO:0098858	CC	GO:0098858	actin-based cell projection	17/673	225/19518	0.00213343705993202	0.00786374321264199	0.00493687914695013	CALB1/EPHA4/FGF13/FZD3/FZD9/GAP43/GPM6A/ITGB3/LRRK2/LY6G6D/MAP2/NLGN1/OXTR/PDPN/SLC6A6/TUBB3/UNC5C	17	0.755555555555556
GO:0000159	CC	GO:0000159	protein phosphatase type 2A complex	4/673	17/19518	0.00232943298577593	0.00851579599718086	0.00534623963948574	PPP2R2B/PPP2R2C/PPP2R3A/PPP2R3B	4	2.35294117647059
GO:0005891	CC	GO:0005891	voltage-gated calcium channel complex	7/673	55/19518	0.0027070889256343	0.00981594846205609	0.00616247885510247	CACNA1A/CACNA1C/CACNA1D/CACNG4/CACNG7/CACNG8/RYR1	7	1.27272727272727
GO:0005942	CC	GO:0005942	phosphatidylinositol 3-kinase complex	5/673	29/19518	0.00286791455532393	0.0103149892931645	0.00647577803158311	BECN2/PIK3CD/PIK3CG/PIK3R2/PIK3R5	5	1.72413793103448
GO:0098562	CC	GO:0098562	cytoplasmic side of membrane	15/673	193/19518	0.00290411067766179	0.0103149892931645	0.00647577803158311	CNR2/GNA11/GNA13/GNAI1/GNAL/GNAO1/GNAQ/GNAZ/GNG13/GNG2/LRRK2/NTSR1/RGS2/SNAP25/TH	15	0.77720207253886
GO:0031527	CC	GO:0031527	filopodium membrane	4/673	18/19518	0.0029141001142124	0.0103149892931645	0.00647577803158311	FZD9/GAP43/ITGB3/PDPN	4	2.22222222222222
GO:0030136	CC	GO:0030136	clathrin-coated vesicle	16/673	215/19518	0.00330142301686541	0.0115939737442675	0.00727872791119933	ADRB2/ASTN1/ATP6V1F/ATP6V1G2/CHRM2/GAD1/SH3GL2/SLC17A7/SLC18A2/SLC18A3/SLC2A4/SYT11/SYT2/SYT9/VAMP2/WNT5A	16	0.744186046511628
GO:0030669	CC	GO:0030669	clathrin-coated endocytic vesicle membrane	8/673	73/19518	0.00358197893791948	0.0124175747759028	0.00779578685482687	ADRB2/CHRM2/SH3GL2/SLC18A3/SYT2/SYT9/VAMP2/WNT5A	8	1.0958904109589
GO:0033178	CC	GO:0033178	proton-transporting two-sector ATPase complex, catalytic domain	4/673	19/19518	0.00359163037240238	0.0124175747759028	0.00779578685482687	ATP6V1C2/ATP6V1E2/ATP6V1F/ATP6V1G2	4	2.10526315789474
GO:0097730	CC	GO:0097730	non-motile cilium	13/673	166/19518	0.00503649718333196	0.0172790595674312	0.0108478400871765	DCDC2/DNAAF4/DRD2/DRD5/GALR3/GNA11/GNAQ/GNGT1/MAGI2/MAP1B/NPY2R/PTGS1/SSTR3	13	0.783132530120482
GO:0030018	CC	GO:0030018	Z disc	11/673	129/19518	0.0050780350833046	0.0172885774591897	0.0108538154452312	ADRA1A/ANK3/CACNA1C/CACNA1D/FKBP1B/HOMER1/MYPN/NEXN/PALLD/RYR1/RYR3	11	0.852713178294574
GO:0043083	CC	GO:0043083	synaptic cleft	4/673	21/19518	0.00525130433461637	0.017743043433628	0.011139130406762	LGI1/NLGN1/NPTX1/SLC1A1	4	1.9047619047619
GO:0008076	CC	GO:0008076	voltage-gated potassium channel complex	8/673	78/19518	0.00538074105316006	0.0178193244818364	0.0111870198540677	HCN1/KCNA1/KCNA2/KCNJ14/KCNJ5/KCNMB2/KCNQ1/KCNQ5	8	1.02564102564103
GO:0000221	CC	GO:0000221	vacuolar proton-transporting V-type ATPase, V1 domain	3/673	11/19518	0.00547364900002597	0.0178193244818364	0.0111870198540677	ATP6V1C2/ATP6V1F/ATP6V1G2	3	2.72727272727273
GO:0031094	CC	GO:0031094	platelet dense tubular network	3/673	11/19518	0.00547364900002597	0.0178193244818364	0.0111870198540677	ATP2A1/ATP2A3/ITPR1	3	2.72727272727273
GO:0033180	CC	GO:0033180	proton-transporting V-type ATPase, V1 domain	3/673	11/19518	0.00547364900002597	0.0178193244818364	0.0111870198540677	ATP6V1C2/ATP6V1F/ATP6V1G2	3	2.72727272727273
GO:0033270	CC	GO:0033270	paranode region of axon	3/673	11/19518	0.00547364900002597	0.0178193244818364	0.0111870198540677	CNTNAP1/KCNA1/MAG	3	2.72727272727273
GO:0005788	CC	GO:0005788	endoplasmic reticulum lumen	20/673	312/19518	0.00604928464731926	0.0195505866137999	0.0122739108786188	BCHE/BDNF/CHRDL1/CLU/COL3A1/COL4A6/CSF1/F2/GCG/KNG1/PENK/SCG2/SHH/VGF/WNT1/WNT3/WNT3A/WNT5A/WNT7A/WNT7B	20	0.641025641025641

	ONTOLOGY	ID	Description	GeneRatio	BgRatio	pvalue	p.adjust	qvalue	geneID	Count	richFactor
GO:0031252	CC	GO:0031252	cell leading edge	25/673	422/19518	0.00634364841881227	0.0202457449486177	0.0127103331515985	APC/ARHGAP44/GABRA3/GABRA4/GABRE/GABRG2/GPER1/GRIA1/HPCA/ITGB3/KCNA2/LIMK1/MAPT/OPRD1/OPRM1/PALLD/PDPN/PLCG1/PLCG2/PTK2B/ROBO2/SHISA7/SLC1A2/TUBB3/UNC5C	25	0.592417061611374
GO:0005637	CC	GO:0005637	nuclear inner membrane	7/673	64/19518	0.00635516657579927	0.0202457449486177	0.0127103331515985	GHRHR/ITPR1/P2RX1/P2RX2/P2RX4/P2RX7/SIGMAR1	7	1.09375
GO:0099524	CC	GO:0099524	postsynaptic cytosol	3/673	12/19518	0.00711291769429148	0.0224990162528652	0.0141249429390185	CALB1/HOMER1/PRKCG	3	2.5
GO:0016469	CC	GO:0016469	proton-transporting two-sector ATPase complex	6/673	50/19518	0.00719265262796547	0.022591007549807	0.0141826953227488	ATP6V0A4/ATP6V0D2/ATP6V1C2/ATP6V1E2/ATP6V1F/ATP6V1G2	6	1.2
GO:0030667	CC	GO:0030667	secretory granule membrane	20/673	319/19518	0.00765930554984188	0.0238884634631432	0.014997241636054	CADPS/CAV1/CHRNB4/CPNE1/GPR84/ITGB3/ITPR1/KIF1A/P2RX1/PLD1/RAP1A/SLC18A2/SNAP25/SNCA/SYT2/SYT4/SYT5/SYT9/VAMP2/VGF	20	0.626959247648903
GO:0005901	CC	GO:0005901	caveola	8/673	83/19518	0.00779840242360952	0.024153385284235	0.0151635602681296	ADCYAP1R1/ADRA1A/ADRA1B/ATP1A2/CAV1/EFNA5/LRRK2/SLC6A3	8	0.963855421686747
GO:0035253	CC	GO:0035253	ciliary rootlet	3/673	13/19518	0.00901247260817549	0.027531251940043	0.0172841940430763	KIF5A/KIF5C/KLC3	3	2.30769230769231
GO:0042583	CC	GO:0042583	chromaffin granule	3/673	13/19518	0.00901247260817549	0.027531251940043	0.0172841940430763	CLU/PENK/SYT2	3	2.30769230769231
GO:0031674	CC	GO:0031674	I band	11/673	141/19518	0.00972252500419156	0.029498273141969	0.0185190952460792	ADRA1A/ANK3/CACNA1C/CACNA1D/FKBP1B/HOMER1/MYPN/NEXN/PALLD/RYR1/RYR3	11	0.780141843971631
GO:0030673	CC	GO:0030673	axolemma	3/673	14/19518	0.0111802874735437	0.0336919473864897	0.0211518952202178	MAPT/ROBO2/SLC1A2	3	2.14285714285714
GO:0030135	CC	GO:0030135	coated vesicle	19/673	312/19518	0.0122560178982997	0.0366857985412192	0.0230314430303618	ADRB2/ASTN1/ATP6V1F/ATP6V1G2/CHRM2/CNIH3/GAD1/GRIA1/SH3GL2/SLC17A7/SLC18A2/SLC18A3/SLC28A2/SLC2A4/SYT11/SYT2/SYT9/VAMP2/WNT5A	19	0.608974358974359
GO:0031091	CC	GO:0031091	platelet alpha granule	8/673	91/19518	0.0132495780883475	0.0393954121826867	0.0247325457649154	A2M/CLU/IGF1/ITGB3/KNG1/SNCA/STXBP1/VEGFA	8	0.879120879120879
GO:0032591	CC	GO:0032591	dendritic spine membrane	3/673	15/19518	0.0136225247600867	0.040236066509925	0.0252603108134059	GPER1/GRIA1/SHISA7	3	2
GO:0045334	CC	GO:0045334	clathrin-coated endocytic vesicle	8/673	92/19518	0.0140878918310983	0.0413368405044069	0.0259513796888653	ADRB2/CHRM2/SH3GL2/SLC18A3/SYT2/SYT9/VAMP2/WNT5A	8	0.869565217391304
GO:0033268	CC	GO:0033268	node of Ranvier	3/673	16/19518	0.016343683328441	0.0476423710097038	0.0299100087056436	ANK3/CNTN2/SCN1B	3	1.875
GO:0062023	CC	GO:0062023	collagen-containing extracellular matrix	23/673	415/19518	0.0178179821975957	0.0516027276631668	0.0323963312683558	A2M/AGT/CLU/COL3A1/COL4A6/CTSG/CXCL12/EFNA5/F2/GDF15/GPC6/KNG1/L1CAM/MMP9/NCAM1/PRSS1/SHH/SULF1/TNR/VWC2/WNT2/WNT2B/WNT5A	23	0.55421686746988
GO:0005871	CC	GO:0005871	kinesin complex	5/673	47/19518	0.0223379536669411	0.0638888347969698	0.0401095823837479	KIF14/KIF1A/KIF5A/KIF5C/KLC3	5	1.06382978723404
GO:0072686	CC	GO:0072686	mitotic spindle	12/673	180/19518	0.0224115953895797	0.0638888347969698	0.0401095823837479	ASPM/AURKA/CDK1/DCDC2/MAD2L1/NEXMIF/TUBB2A/TUBB2B/TUBB3/TUBB4A/TUBB6/WDR62	12	0.666666666666667
GO:0031143	CC	GO:0031143	pseudopodium	3/673	18/19518	0.0226332643451149	0.0638888347969698	0.0401095823837479	ARRB1/CAPN2/F2RL1	3	1.66666666666667
GO:0043034	CC	GO:0043034	costamere	3/673	18/19518	0.0226332643451149	0.0638888347969698	0.0401095823837479	ANK3/HOMER1/VCL	3	1.66666666666667
GO:0005790	CC	GO:0005790	smooth endoplasmic reticulum	4/673	32/19518	0.0234294507627192	0.0657203461646085	0.0412594101481847	GABARAP/RYR1/RYR3/TH	4	1.25
GO:0031201	CC	GO:0031201	SNARE complex	5/673	48/19518	0.0242511083885508	0.0675999646330853	0.0424394396799639	CPLX1/SNAP25/STX1A/SYN2/VAMP2	5	1.04166666666667

	ONTOLOGY	ID	Description	GeneRatio	BgRatio	pvalue	p.adjust	qvalue	geneID	Count	richFactor
GO:0097038	CC	GO:0097038	perinuclear endoplasmic reticulum	3/673	19/19518	0.0262035707149984	0.0723141655382804	0.045399027692194	CAPN2/CLU/NOX4	3	1.57894736842105
GO:0043209	CC	GO:0043209	myelin sheath	5/673	49/19518	0.0262665803076265	0.0723141655382804	0.045399027692194	BCL2/CNTN2/GPM6B/MAG/TUBB4A	5	1.02040816326531
GO:0030863	CC	GO:0030863	cortical cytoskeleton	8/673	104/19518	0.0273599625275177	0.0748622287562754	0.0469987086362267	CALB1/CAPN2/NSMF/PRKCB/RIMS1/RIMS3/RIMS4/SPTBN2	8	0.769230769230769
GO:0005771	CC	GO:0005771	multivesicular body	6/673	67/19518	0.0277493748181127	0.0754647632248675	0.0473769813967778	GFRA1/LRRK2/NEDD4L/NSG1/SLC17A8/SLC2A4	6	0.895522388059701
GO:0014704	CC	GO:0014704	intercalated disc	5/673	50/19518	0.0283859056053666	0.0767279630302636	0.0481700216333494	ANK3/ATP1A2/FGF13/SCN1B/VCL	5	1
GO:0005839	CC	GO:0005839	proteasome core complex	3/673	20/19518	0.0300568020243936	0.0807550223065033	0.0506982202821097	PSMA2/PSMA6/PSMA7	3	1.5
GO:0030016	CC	GO:0030016	myofibril	14/673	233/19518	0.0317435553825157	0.0847762018000121	0.0532227275874515	ADRA1A/ANK3/CACNA1C/CACNA1D/FKBP1B/HOMER1/MYPN/NEXN/PALLD/PSMA6/RYR1/RYR3/TMOD2/VCL	14	0.600858369098712
GO:0008287	CC	GO:0008287	protein serine/threonine phosphatase complex	5/673	52/19518	0.0329415415711164	0.0874519496471303	0.0549025692851939	ITPR1/PPP2R2B/PPP2R2C/PPP2R3A/PPP2R3B	5	0.961538461538462
GO:0005938	CC	GO:0005938	cell cortex	17/673	302/19518	0.0333800015370922	0.0880916016896042	0.0553041445584959	CALB1/CAPN2/CAV1/GAD1/GNAI1/NSMF/PRKCB/PTK2B/RIMS1/RIMS3/RIMS4/RYR1/SEPTIN5/SNCA/SPTBN2/STXBP1/UNC13C	17	0.562913907284768
GO:0030017	CC	GO:0030017	sarcomere	13/673	213/19518	0.0337491361085298	0.0885418512023782	0.0555868124140491	ADRA1A/ANK3/CACNA1C/CACNA1D/FKBP1B/HOMER1/MYPN/NEXN/PALLD/PSMA6/RYR1/RYR3/TMOD2	13	0.610328638497653
GO:0031968	CC	GO:0031968	organelle outer membrane	14/673	236/19518	0.0348071203610848	0.090783483514876	0.0569941152111329	BCL2/BCL2L1/BID/CNR1/EPHA4/GHRHR/LRRK2/MAOA/MAOB/PINK1/PPP2R2B/PRKN/SIGMAR1/SOX10	14	0.593220338983051
GO:0019867	CC	GO:0019867	outer membrane	14/673	238/19518	0.0369645791896321	0.0958500134800925	0.060174896355215	BCL2/BCL2L1/BID/CNR1/EPHA4/GHRHR/LRRK2/MAOA/MAOB/PINK1/PPP2R2B/PRKN/SIGMAR1/SOX10	14	0.588235294117647
GO:0005769	CC	GO:0005769	early endosome	22/673	423/19518	0.0372561104558096	0.0960475448745148	0.0602989070961079	ADRB1/ADRB2/ATP6V0D2/CACNG7/CAV1/DKK1/DNER/EPHA3/EPHA4/F2RL1/GPER1/GRIA1/KCNQ1/NSG1/PLA2G4B/PLA2G4E/RAP1A/SH3GL2/SLC1A1/TMEM108/TRAK2/WNT3A	22	0.520094562647754
GO:0030286	CC	GO:0030286	dynein complex	5/673	54/19518	0.0379274154672214	0.0966607274193185	0.0606838647475542	DNAH10/DNAH12/DNAH14/DNAH2/DNAH5	5	0.925925925925926
GO:1903293	CC	GO:1903293	phosphatase complex	5/673	54/19518	0.0379274154672214	0.0966607274193185	0.0606838647475542	ITPR1/PPP2R2B/PPP2R2C/PPP2R3A/PPP2R3B	5	0.925925925925926
GO:0005858	CC	GO:0005858	axonemal dynein complex	3/673	22/19518	0.0386034602083374	0.0972719957792006	0.0610676206685564	DNAH12/DNAH2/DNAH5	3	1.36363636363636
GO:0031258	CC	GO:0031258	lamellipodium membrane	3/673	22/19518	0.0386034602083374	0.0972719957792006	0.0610676206685564	ITGB3/KCNA2/PDPN	3	1.36363636363636
GO:0043292	CC	GO:0043292	contractile fiber	14/673	243/19518	0.0427748197008627	0.107177357228004	0.0672862332373121	ADRA1A/ANK3/CACNA1C/CACNA1D/FKBP1B/HOMER1/MYPN/NEXN/PALLD/PSMA6/RYR1/RYR3/TMOD2/VCL	14	0.576131687242798
GO:0097136	CC	GO:0097136	Bcl-2 family protein complex	2/673	10/19518	0.0444649685734351	0.110789809965095	0.0695541407852616	BCL2/BCL2L1	2	2
GO:0031526	CC	GO:0031526	brush border membrane	5/673	57/19518	0.0462277584896154	0.114542112702047	0.0719098465394018	ATP6V0A4/DRD5/GNA13/SLC28A2/SLC7A11	5	0.87719298245614

	ONTOLOGY	ID	Description	GeneRatio	BgRatio	pvalue	p.adjust	qvalue	geneID	Count	richFactor
GO:0030594	MF	GO:0030594	neurotransmitter receptor activity	51/665	98/18369	2.28681451599945e-47	1.59619653216762e-44	1.04471315783554e-44	ADRB1/CHRFAM7A/CHRM2/CHRM4/CHRM5/CHRNA1/CHRNA3/CHRNA4/CHRNA6/CHRNA7/CHRNB2/CHRNB4/CHRND/CHRNG/DRD2/DRD4/DRD5/GABRA3/GABRA4/GABRB1/GABRB3/GABRD/GABRE/GABRG2/GABRP/GABRR1/GLRA3/GLRB/GRIA1/GRIA2/GRIA3/GRIA4/GRID2/GRIK1/GRIK2/GRIK3/GRIK5/GRIN2A/GRIN2B/GRIN2D/GRIN3A/GRIN3B/HTR1D/HTR2B/HTR2C/HTR3A/HTR3C/HTR3E/HTR4/HTR7/PTK2B	51	5.20408163265306
GO:0008528	MF	GO:0008528	G protein-coupled peptide receptor activity	57/665	142/18369	9.09424200484705e-45	3.17389045969162e-42	2.07731633163348e-42	ADCYAP1R1/AGTR1/AVPR1B/BDKRB1/BDKRB2/CCKAR/CCR2/CCR7/CX3CR1/CXCR5/CYSLTR1/CYSLTR2/EDNRA/EDNRB/F2RL1/F2RL2/GAL/GALR1/GALR3/GCGR/GHRHR/GLP2R/GPR143/GPR171/GPR84/GRPR/KISS1R/LTB4R/LTB4R2/MC1R/MC3R/NMUR2/NPBWR1/NPFFR1/NPSR1/NPY1R/NPY2R/NPY4R2/NPY5R/NTSR1/OPRD1/OPRM1/OXTR/PROKR1/PTH1R/RXFP3/SCTR/SORCS1/SORCS3/SSTR1/SSTR2/SSTR3/SSTR5/TACR1/TACR2/VIPR1/VIPR2	57	4.01408450704225
GO:0001653	MF	GO:0001653	peptide receptor activity	57/665	148/18369	1.47168346617487e-43	3.42411686463354e-41	2.24108990989437e-41	ADCYAP1R1/AGTR1/AVPR1B/BDKRB1/BDKRB2/CCKAR/CCR2/CCR7/CX3CR1/CXCR5/CYSLTR1/CYSLTR2/EDNRA/EDNRB/F2RL1/F2RL2/GAL/GALR1/GALR3/GCGR/GHRHR/GLP2R/GPR143/GPR171/GPR84/GRPR/KISS1R/LTB4R/LTB4R2/MC1R/MC3R/NMUR2/NPBWR1/NPFFR1/NPSR1/NPY1R/NPY2R/NPY4R2/NPY5R/NTSR1/OPRD1/OPRM1/OXTR/PROKR1/PTH1R/RXFP3/SCTR/SORCS1/SORCS3/SSTR1/SSTR2/SSTR3/SSTR5/TACR1/TACR2/VIPR1/VIPR2	57	3.85135135135135
GO:0005230	MF	GO:0005230	extracellular ligand-gated monoatomic ion channel activity	41/665	67/18369	2.69725178182005e-42	4.706704359276e-40	3.08054545607869e-40	CHRFAM7A/CHRNA1/CHRNA3/CHRNA4/CHRNA6/CHRNA7/CHRNB2/CHRNB4/CHRND/CHRNG/GABRA3/GABRA4/GABRB1/GABRB3/GABRE/GABRG2/GLRA3/GLRB/GRIA1/GRIA2/GRIA3/GRIA4/GRID2/GRIK1/GRIK2/GRIK3/GRIK5/GRIN2A/GRIN2B/GRIN2D/GRIN3A/GRIN3B/HTR3A/HTR3C/HTR3E/P2RX1/P2RX2/P2RX4/P2RX7/PTK2B/SLC1A7	41	6.11940298507463

	ONTOLOGY	ID	Description	GeneRatio	BgRatio	pvalue	p.adjust	qvalue	geneID	Count	richFactor
GO:0015276	MF	GO:0015276	ligand-gated monoatomic ion channel activity	51/665	140/18369	1.25180257827764e-37	1.45626366606298e-35	9.5312687539034e-36	ASIC1/CHRFAM7A/CHRNA1/CHRNA3/CHRNA4/CHRNA6/CHRNA7/CHRNB2/CHRNB4/CHRND/CHRNG/FKBP1B/GABRA3/GABRA4/GABRB1/GABRB3/GABRE/GABRG2/GABRR1/GLRA3/GLRB/GRIA1/GRIA2/GRIA3/GRIA4/GRID2/GRIK1/GRIK2/GRIK3/GRIK5/GRIN2A/GRIN2B/GRIN2D/GRIN3A/GRIN3B/HCN1/HTR3A/HTR3C/HTR3E/ITPR1/KCNJ14/KCNJ5/KCNJ9/P2RX1/P2RX2/P2RX4/P2RX7/PTK2B/RYR1/RYR3/SLC1A7	51	3.64285714285714
GO:0022834	MF	GO:0022834	ligand-gated channel activity	51/665	140/18369	1.25180257827764e-37	1.45626366606298e-35	9.5312687539034e-36	ASIC1/CHRFAM7A/CHRNA1/CHRNA3/CHRNA4/CHRNA6/CHRNA7/CHRNB2/CHRNB4/CHRND/CHRNG/FKBP1B/GABRA3/GABRA4/GABRB1/GABRB3/GABRE/GABRG2/GABRR1/GLRA3/GLRB/GRIA1/GRIA2/GRIA3/GRIA4/GRID2/GRIK1/GRIK2/GRIK3/GRIK5/GRIN2A/GRIN2B/GRIN2D/GRIN3A/GRIN3B/HCN1/HTR3A/HTR3C/HTR3E/ITPR1/KCNJ14/KCNJ5/KCNJ9/P2RX1/P2RX2/P2RX4/P2RX7/PTK2B/RYR1/RYR3/SLC1A7	51	3.64285714285714
GO:0022824	MF	GO:0022824	transmitter-gated monoatomic ion channel activity	35/665	56/18369	9.85553930103972e-37	8.59895804015716e-35	5.62803165348847e-35	CHRFAM7A/CHRNA1/CHRNA3/CHRNA4/CHRNA6/CHRNA7/CHRNB2/CHRNB4/CHRND/CHRNG/GABRA3/GABRA4/GABRB1/GABRB3/GABRE/GABRG2/GLRA3/GRIA1/GRIA2/GRIA3/GRIA4/GRID2/GRIK1/GRIK2/GRIK3/GRIK5/GRIN2A/GRIN2B/GRIN2D/GRIN3A/GRIN3B/HTR3A/HTR3C/HTR3E/PTK2B	35	6.25
GO:0022835	MF	GO:0022835	transmitter-gated channel activity	35/665	56/18369	9.85553930103972e-37	8.59895804015716e-35	5.62803165348847e-35	CHRFAM7A/CHRNA1/CHRNA3/CHRNA4/CHRNA6/CHRNA7/CHRNB2/CHRNB4/CHRND/CHRNG/GABRA3/GABRA4/GABRB1/GABRB3/GABRE/GABRG2/GLRA3/GRIA1/GRIA2/GRIA3/GRIA4/GRID2/GRIK1/GRIK2/GRIK3/GRIK5/GRIN2A/GRIN2B/GRIN2D/GRIN3A/GRIN3B/HTR3A/HTR3C/HTR3E/PTK2B	35	6.25

	ONTOLOGY	ID	Description	GeneRatio	BgRatio	pvalue	p.adjust	qvalue	geneID	Count	richFactor
GO:0001664	MF	GO:0001664	G protein-coupled receptor binding	67/665	289/18369	3.37537281030983e-35	2.61778913510696e-33	1.71334713412218e-33	ADRA2A/ADRA2C/ADRB1/ADRB3/AGT/AGTR1/APELA/APLN/ARRB1/ASIP/BDKRB2/CALCA/CALCB/CCL5/CCR2/CX3CR1/CXCL12/CXCL13/CXCL8/EDN2/EDN3/EDNRB/GAL/GCG/GNA11/GNA13/GNAI1/GNAL/GNAO1/GNAQ/GNAZ/HCRT/HOMER1/INSL5/KISS1/MAGI2/NMB/NMU/NPW/NPY/OXT/P2RY1/P2RY2/PENK/PNOC/PPY/PRKN/PRNP/PYY/S1PR1/UCHL1/UCN/UCN2/UCN3/UTS2B/WNT1/WNT11/WNT16/WNT2/WNT2B/WNT3/WNT3A/WNT5A/WNT7A/WNT7B/WNT8B/WNT9A	67	2.31833910034602
GO:0022836	MF	GO:0022836	gated channel activity	67/665	308/18369	2.23756579900621e-33	1.41983720700576e-31	9.29285700257126e-32	ASIC1/CACNA1A/CACNA1C/CACNA1D/CACNG4/CACNG5/CACNG7/CACNG8/CHRFAM7A/CHRNA1/CHRNA3/CHRNA4/CHRNA6/CHRNA7/CHRNB2/CHRNB4/CHRND/CHRNG/CLCN1/FKBP1B/GABRA3/GABRA4/GABRB1/GABRB3/GABRE/GABRG2/GABRR1/GLRA3/GLRB/GRIA1/GRIA2/GRIA3/GRIA4/GRID2/GRIK1/GRIK2/GRIK3/GRIK5/GRIN2A/GRIN2B/GRIN2D/GRIN3A/GRIN3B/HCN1/HTR3A/HTR3C/HTR3E/ITPR1/KCNA1/KCNA2/KCNJ14/KCNJ5/KCNJ9/KCNMB2/KCNQ1/KCNQ5/OPRM1/P2RX1/P2RX2/P2RX4/P2RX7/PTK2B/RYR1/RYR3/SCN1B/SCN2B/SLC1A7	67	2.17532467532468
GO:0022839	MF	GO:0022839	monoatomic ion gated channel activity	67/665	308/18369	2.23756579900621e-33	1.41983720700576e-31	9.29285700257126e-32	ASIC1/CACNA1A/CACNA1C/CACNA1D/CACNG4/CACNG5/CACNG7/CACNG8/CHRFAM7A/CHRNA1/CHRNA3/CHRNA4/CHRNA6/CHRNA7/CHRNB2/CHRNB4/CHRND/CHRNG/CLCN1/FKBP1B/GABRA3/GABRA4/GABRB1/GABRB3/GABRE/GABRG2/GABRR1/GLRA3/GLRB/GRIA1/GRIA2/GRIA3/GRIA4/GRID2/GRIK1/GRIK2/GRIK3/GRIK5/GRIN2A/GRIN2B/GRIN2D/GRIN3A/GRIN3B/HCN1/HTR3A/HTR3C/HTR3E/ITPR1/KCNA1/KCNA2/KCNJ14/KCNJ5/KCNJ9/KCNMB2/KCNQ1/KCNQ5/OPRM1/P2RX1/P2RX2/P2RX4/P2RX7/PTK2B/RYR1/RYR3/SCN1B/SCN2B/SLC1A7	67	2.17532467532468
GO:0098960	MF	GO:0098960	postsynaptic neurotransmitter receptor activity	31/665	52/18369	9.9065242667489e-32	5.76229494849228e-30	3.77143116821844e-30	ADRB1/CHRFAM7A/CHRM2/CHRM4/CHRM5/CHRNA1/CHRNA3/CHRNA4/CHRNA6/CHRNA7/CHRNB2/CHRNB4/CHRND/CHRNG/DRD2/DRD4/DRD5/GRIA1/GRIA2/GRIA3/GRIA4/GRID2/GRIK1/GRIK2/GRIK3/GRIK5/GRIN2A/GRIN2B/GRIN2D/GRIN3A/GRIN3B	31	5.96153846153846

	ONTOLOGY	ID	Description	GeneRatio	BgRatio	pvalue	p.adjust	qvalue	geneID	Count	richFactor
GO:0048018	MF	GO:0048018	receptor ligand activity	82/665	497/18369	1.42226603024484e-31	7.63647453162229e-30	4.99808467308713e-30	ADCYAP1/AGT/APELA/APLN/ASIP/BDNF/CALCA/CALCB/CARTPT/CCL5/CGA/CRLF1/CSF1/CTSG/CXCL12/CXCL13/CXCL8/DKK1/EDN2/EDN3/EFNA5/F2/F2RL2/FGF12/FGF13/FLRT2/GAL/GCG/GDF15/GDNF/GRP/HCRT/IGF1/IL1A/INHBA/INSL5/LEP/LHB/MIF/NGF/NMB/NPY/NRG3/NTF4/OXT/PENK/PNOC/PPY/PRL/PTN/PYY/RLN2/SCG2/SEMA3D/SEMA3E/SEMA3F/SEMA3G/SEMA6A/SEMA6D/SHH/SLURP1/SPX/SST/UCN/UCN2/UCN3/UTS2B/VEGFA/VGF/VIP/WNT1/WNT11/WNT16/WNT2/WNT2B/WNT3/WNT3A/WNT5A/WNT7A/WNT7B/WNT8B/WNT9A	82	1.64989939637827
GO:0005216	MF	GO:0005216	monoatomic ion channel activity	73/665	432/18369	7.98889188998916e-29	3.98303324229459e-27	2.60690156410172e-27	ASIC1/CACNA1A/CACNA1C/CACNA1D/CACNG4/CACNG5/CACNG7/CACNG8/CHRFAM7A/CHRNA1/CHRNA3/CHRNA4/CHRNA6/CHRNA7/CHRNB2/CHRNB4/CHRND/CHRNG/CLCN1/FKBP1B/GABRA3/GABRA4/GABRB1/GABRB3/GABRE/GABRG2/GABRR1/GLRA3/GLRB/GPM6A/GRIA1/GRIA2/GRIA3/GRIA4/GRID2/GRIK1/GRIK2/GRIK3/GRIK5/GRIN2A/GRIN2B/GRIN2D/GRIN3A/GRIN3B/HCN1/HTR3A/HTR3C/HTR3E/ITPR1/KCNA1/KCNA2/KCNJ14/KCNJ5/KCNJ9/KCNMB2/KCNQ1/KCNQ5/NMUR2/OPRM1/P2RX1/P2RX2/P2RX4/P2RX7/PTK2B/RYR1/RYR3/SCN1B/SCN2B/SLC17A7/SLC17A8/SLC1A1/SLC1A7/TRPC1	73	1.68981481481481
GO:0099529	MF	GO:0099529	neurotransmitter receptor activity involved in regulation of postsynaptic membrane potential	25/665	37/18369	7.36289447127362e-28	3.42620022729932e-26	2.24245347405807e-26	ADRB1/CHRFAM7A/CHRNA1/CHRNA3/CHRNA4/CHRNA6/CHRNA7/CHRNB2/CHRNB4/CHRND/CHRNG/GRIA1/GRIA2/GRIA3/GRIA4/GRID2/GRIK1/GRIK2/GRIK3/GRIK5/GRIN2A/GRIN2B/GRIN2D/GRIN3A/GRIN3B	25	6.75675675675676
GO:1904315	MF	GO:1904315	transmitter-gated monoatomic ion channel activity involved in regulation of postsynaptic membrane potential	24/665	35/18369	4.91093099051997e-27	2.14239364461434e-25	1.40220003281952e-25	CHRFAM7A/CHRNA1/CHRNA3/CHRNA4/CHRNA6/CHRNA7/CHRNB2/CHRNB4/CHRND/CHRNG/GRIA1/GRIA2/GRIA3/GRIA4/GRID2/GRIK1/GRIK2/GRIK3/GRIK5/GRIN2A/GRIN2B/GRIN2D/GRIN3A/GRIN3B	24	6.85714285714286

	ONTOLOGY	ID	Description	GeneRatio	BgRatio	pvalue	p.adjust	qvalue	geneID	Count	richFactor
GO:0015267	MF	GO:0015267	channel activity	74/665	478/18369	1.01450933552171e-26	4.16545597761268e-25	2.72629753322863e-25	ASIC1/BCL2/CACNA1A/CACNA1C/CACNA1D/CACNG4/CACNG5/CACNG7/CACNG8/CHRFAM7A/CHRNA1/CHRNA3/CHRNA4/CHRNA6/CHRNA7/CHRNB2/CHRNB4/CHRND/CHRNG/CLCN1/FKBP1B/GABRA3/GABRA4/GABRB1/GABRB3/GABRE/GABRG2/GABRR1/GLRA3/GLRB/GPM6A/GRIA1/GRIA2/GRIA3/GRIA4/GRID2/GRIK1/GRIK2/GRIK3/GRIK5/GRIN2A/GRIN2B/GRIN2D/GRIN3A/GRIN3B/HCN1/HTR3A/HTR3C/HTR3E/ITPR1/KCNA1/KCNA2/KCNJ14/KCNJ5/KCNJ9/KCNMB2/KCNQ1/KCNQ5/NMUR2/OPRM1/P2RX1/P2RX2/P2RX4/P2RX7/PTK2B/RYR1/RYR3/SCN1B/SCN2B/SLC17A7/SLC17A8/SLC1A1/SLC1A7/TRPC1	74	1.54811715481172
GO:0022803	MF	GO:0022803	passive transmembrane transporter activity	74/665	479/18369	1.16089780468934e-26	4.50170370929531e-25	2.94637220605363e-25	ASIC1/BCL2/CACNA1A/CACNA1C/CACNA1D/CACNG4/CACNG5/CACNG7/CACNG8/CHRFAM7A/CHRNA1/CHRNA3/CHRNA4/CHRNA6/CHRNA7/CHRNB2/CHRNB4/CHRND/CHRNG/CLCN1/FKBP1B/GABRA3/GABRA4/GABRB1/GABRB3/GABRE/GABRG2/GABRR1/GLRA3/GLRB/GPM6A/GRIA1/GRIA2/GRIA3/GRIA4/GRID2/GRIK1/GRIK2/GRIK3/GRIK5/GRIN2A/GRIN2B/GRIN2D/GRIN3A/GRIN3B/HCN1/HTR3A/HTR3C/HTR3E/ITPR1/KCNA1/KCNA2/KCNJ14/KCNJ5/KCNJ9/KCNMB2/KCNQ1/KCNQ5/NMUR2/OPRM1/P2RX1/P2RX2/P2RX4/P2RX7/PTK2B/RYR1/RYR3/SCN1B/SCN2B/SLC17A7/SLC17A8/SLC1A1/SLC1A7/TRPC1	74	1.54488517745303
GO:0008188	MF	GO:0008188	neuropeptide receptor activity	26/665	47/18369	1.28029104733441e-25	4.70338500547061e-24	3.07837293375697e-24	GAL/GALR1/GALR3/GPR143/GPR171/GRPR/KISS1R/NMUR2/NPBWR1/NPFFR1/NPSR1/NPY1R/NPY2R/NPY4R2/NPY5R/NTSR1/OPRM1/PROKR1/SORCS1/SORCS3/SSTR1/SSTR2/SSTR3/SSTR5/TACR1/TACR2	26	5.53191489361702
GO:0005179	MF	GO:0005179	hormone activity	37/665	122/18369	2.50058326259053e-24	8.72703558644096e-23	5.71185861033838e-23	ADCYAP1/AGT/APELA/APLN/ASIP/CALCA/CALCB/CARTPT/CGA/EDN2/EDN3/GAL/GCG/GRP/HCRT/IGF1/INHBA/INSL5/LEP/LHB/NMB/NPY/OXT/PENK/PNOC/PPY/PRL/PYY/RLN2/SPX/SST/UCN/UCN2/UCN3/UTS2B/VGF/VIP	37	3.0327868852459

	ONTOLOGY	ID	Description	GeneRatio	BgRatio	pvalue	p.adjust	qvalue	geneID	Count	richFactor
GO:0099094	MF	GO:0099094	ligand-gated monoatomic cation channel activity	33/665	108/18369	6.02281366221758e-22	2.00186854106089e-20	1.31022613002628e-20	ASIC1/CHRFAM7A/CHRNA1/CHRNA3/CHRNA4/CHRNA6/CHRNA7/CHRNB2/CHRNB4/CHRND/CHRNG/FKBP1B/GRIK1/GRIK2/GRIK3/GRIK5/GRIN2A/GRIN2B/GRIN2D/HCN1/HTR3A/HTR3C/HTR3E/ITPR1/KCNJ14/KCNJ5/KCNJ9/P2RX1/P2RX2/P2RX4/P2RX7/RYR1/RYR3	33	3.05555555555556
GO:0008066	MF	GO:0008066	glutamate receptor activity	18/665	27/18369	3.14904736703329e-20	9.99106846449653e-19	6.53917013058588e-19	GRIA1/GRIA2/GRIA3/GRIA4/GRID2/GRIK1/GRIK2/GRIK3/GRIK5/GRIN2A/GRIN2B/GRIN2D/GRIN3A/GRIN3B/GRM2/GRM4/GRM8/PTK2B	18	6.66666666666667
GO:0005261	MF	GO:0005261	monoatomic cation channel activity	52/665	324/18369	9.83285443835932e-20	2.98405756433687e-18	1.95307040102881e-18	ASIC1/CACNA1A/CACNA1C/CACNA1D/CACNG4/CACNG5/CACNG7/CACNG8/CHRFAM7A/CHRNA1/CHRNA3/CHRNA4/CHRNA6/CHRNA7/CHRNB2/CHRNB4/CHRND/CHRNG/FKBP1B/GPM6A/GRIK1/GRIK2/GRIK3/GRIK5/GRIN2A/GRIN2B/GRIN2D/GRIN3A/GRIN3B/HCN1/HTR3A/HTR3C/HTR3E/ITPR1/KCNA1/KCNA2/KCNJ14/KCNJ5/KCNJ9/KCNMB2/KCNQ1/KCNQ5/OPRM1/P2RX1/P2RX2/P2RX4/P2RX7/RYR1/RYR3/SCN1B/SCN2B/TRPC1	52	1.60493827160494
GO:0042277	MF	GO:0042277	peptide binding	51/665	317/18369	2.01701747810696e-19	5.86615916549442e-18	3.83941046271238e-18	ADCYAP1R1/ADRB2/AVPR1B/BCHE/BDKRB1/CACNA1A/CCKAR/CHRNA7/CLU/EDNRB/EPHA4/EPHB2/GALR1/GALR3/GCGR/GHR/GHRHR/GLP2R/GPR171/GRIA1/GRIA2/GRIA3/GRIA4/GRIN2A/GRIN2B/GRPR/HSP90AB1/INHBA/MC3R/NGFR/NLGN1/NMUR2/NPBWR1/NPFFR1/NPY1R/NPY4R2/NPY5R/OPRD1/OPRM1/OXTR/PRNP/PTGDR2/PTH1R/SCTR/SSTR1/SSTR2/SSTR3/SSTR5/TUBB3/VIPR1/VIPR2	51	1.60883280757098
GO:0005184	MF	GO:0005184	neuropeptide hormone activity	18/665	30/18369	5.24857942125745e-19	1.4200626084009e-17	9.2943322582716e-18	ADCYAP1/ASIP/CALCB/CARTPT/GAL/GRP/HCRT/NMB/NPY/OXT/PENK/PNOC/PPY/PYY/SPX/UCN/VGF/VIP	18	6
GO:0033218	MF	GO:0033218	amide binding	57/665	400/18369	5.2896314926108e-19	1.4200626084009e-17	9.2943322582716e-18	ADCYAP1R1/ADRB2/AVPR1B/BCHE/BDKRB1/CACNA1A/CCKAR/CHRNA7/CLU/EDNRB/EPHA4/EPHB2/FKBP1B/GALR1/GALR3/GCGR/GHR/GHRHR/GLP2R/GPR171/GRIA1/GRIA2/GRIA3/GRIA4/GRIN2A/GRIN2B/GRIN2D/GRPR/HSP90AB1/INHBA/MAG/MC3R/NGFR/NLGN1/NMUR2/NPBWR1/NPFFR1/NPY1R/NPY4R2/NPY5R/OPRD1/OPRM1/OXTR/P2RX1/PRNP/PTGDR2/PTH1R/RTN4R/SCTR/SLC1A1/SSTR1/SSTR2/SSTR3/SSTR5/TUBB3/VIPR1/VIPR2	57	1.425

	ONTOLOGY	ID	Description	GeneRatio	BgRatio	pvalue	p.adjust	qvalue	geneID	Count	richFactor
GO:0004970	MF	GO:0004970	ionotropic glutamate receptor activity	15/665	19/18369	6.98528400837284e-19	1.80582527327565e-17	1.1819155008319e-17	GRIA1/GRIA2/GRIA3/GRIA4/GRID2/GRIK1/GRIK2/GRIK3/GRIK5/GRIN2A/GRIN2B/GRIN2D/GRIN3A/GRIN3B/PTK2B	15	7.89473684210526
GO:0005231	MF	GO:0005231	excitatory extracellular ligand-gated monoatomic ion channel activity	18/665	31/18369	1.20980290352235e-18	3.01586580949499e-17	1.97388894785225e-17	CHRFAM7A/CHRNA1/CHRNA3/CHRNA4/CHRNA6/CHRNA7/CHRNB2/CHRNB4/CHRND/CHRNG/GLRA3/GLRB/GRIK2/P2RX1/P2RX2/P2RX4/P2RX7/SLC1A7	18	5.80645161290323
GO:0046873	MF	GO:0046873	metal ion transmembrane transporter activity	57/665	435/18369	2.7634752850521e-17	6.65139913436679e-16	4.35335126574451e-16	ASIC1/ATP1A2/ATP2A1/ATP2A3/CACNA1A/CACNA1C/CACNA1D/CACNG4/CACNG5/CACNG7/CACNG8/CHRNA7/FKBP1B/GPM6A/GRIK1/GRIK2/GRIK3/GRIK5/GRIN2A/GRIN2B/GRIN2D/GRIN3A/GRIN3B/HCN1/ITPR1/KCNA1/KCNA2/KCNJ14/KCNJ5/KCNJ9/KCNMB2/KCNQ1/KCNQ5/OPRM1/P2RX4/RYR1/RYR3/SCN1B/SCN2B/SLC17A7/SLC17A8/SLC18A2/SLC1A1/SLC1A2/SLC1A7/SLC28A2/SLC38A3/SLC38A5/SLC4A8/SLC5A7/SLC6A1/SLC6A15/SLC6A2/SLC6A3/SLC6A6/SLC6A9/TRPC1	57	1.31034482758621
GO:0016247	MF	GO:0016247	channel regulator activity	33/665	152/18369	5.92033092311476e-17	1.3774636614447e-15	9.01552147590107e-16	ADRA2A/ADRB2/BCL2/CACNG4/CACNG5/CACNG7/CACNG8/CAMK2D/CAV1/CHRNA7/DRD4/F2/F2RL1/F2RL2/FGF12/FGF13/FKBP1B/GNA11/GRM2/ITPR1/KCNMB2/LYNX1/NEDD4L/NOS1/NPY/NPY2R/NRXN1/NRXN2/PRKCB/PRKG1/SCN1B/SCN2B/STX1A	33	2.17105263157895
GO:0042923	MF	GO:0042923	neuropeptide binding	17/665	32/18369	8.78850609171066e-17	1.97883137161743e-15	1.29514826614683e-15	ADCYAP1R1/GALR1/GPR171/GPRP/MC3R/NMUR2/NPBWR1/NPY1R/NPY4R2/NPY5R/OPRD1/OPRM1/PTGDR2/SSTR1/SSTR2/SSTR3/SSTR5	17	5.3125
GO:0071855	MF	GO:0071855	neuropeptide receptor binding	17/665	33/18369	1.75223460496748e-16	3.82206173208532e-15	2.50154545577594e-15	APELA/APLN/ASIP/EDN2/EDN3/GAL/GNAO1/HCRT/KISS1/NMB/NMU/NPY/PPY/PYY/UCN/UCN2/UCN3	17	5.15151515151515
GO:0099106	MF	GO:0099106	ion channel regulator activity	31/665	147/18369	1.25118704201136e-15	2.64645016764828e-14	1.73210582530441e-14	ADRA2A/ADRB2/CACNG4/CACNG7/CACNG8/CAMK2D/CAV1/CHRNA7/DRD4/F2/F2RL1/F2RL2/FGF12/FGF13/FKBP1B/GNA11/GRM2/ITPR1/KCNMB2/LYNX1/NEDD4L/NOS1/NPY/NPY2R/NRXN1/NRXN2/PRKCB/PRKG1/SCN1B/SCN2B/STX1A	31	2.10884353741497
GO:0008227	MF	GO:0008227	G protein-coupled amine receptor activity	17/665	39/18369	6.25264095669316e-15	1.28363040816818e-13	8.40138134738338e-14	ADRA1A/ADRA1B/ADRA1D/ADRA2A/ADRA2C/ADRB1/ADRB2/ADRB3/CHRM2/CHRM4/CHRM5/DRD4/HTR1D/HTR2B/HTR2C/HTR4/HTR7	17	4.35897435897436

	ONTOLOGY	ID	Description	GeneRatio	BgRatio	pvalue	p.adjust	qvalue	geneID	Count	richFactor
GO:0042562	MF	GO:0042562	hormone binding	23/665	84/18369	1.54462945811377e-14	3.08043246218118e-13	2.01614792427482e-13	ADCYAP1R1/ATP1A2/CCKAR/EDNRB/GALR1/GALR3/GCGR/GHR/GHRHR/GLP2R/GPER1/INHBA/MC1R/MC3R/MTNR1A/NR3C1/OXTR/PTH1R/SCTR/THRB/UCN2/VIPR1/VIPR2	23	2.73809523809524
GO:0099507	MF	GO:0099507	ligand-gated monoatomic ion channel activity involved in regulation of presynaptic membrane potential	12/665	17/18369	2.41053999744126e-14	4.67376921726111e-13	3.05898935347809e-13	GABRA3/GABRB1/GABRR1/GRIA1/GRIA3/GRIA4/GRIK2/GRIK3/GRIK5/GRIN2B/GRIN2D/GRIN3B	12	7.05882352941177
GO:0005326	MF	GO:0005326	neurotransmitter transmembrane transporter activity	12/665	19/18369	1.83618479886144e-13	3.46393780974402e-12	2.26715278152451e-12	CPLX1/SLC17A7/SLC17A8/SLC18A3/SLC22A2/SLC22A3/SLC28A2/SLC29A1/SLC6A15/SLC6A16/SLC6A2/SLC6A3	12	6.31578947368421
GO:1901338	MF	GO:1901338	catecholamine binding	10/665	13/18369	9.382403174495e-13	1.72339931994671e-11	1.12796758385896e-11	ADRA2A/ADRA2C/ADRB2/ADRB3/DRD2/DRD4/DRD5/GPR143/SLC6A3/TH	10	7.69230769230769
GO:0015464	MF	GO:0015464	acetylcholine receptor activity	12/665	21/18369	1.00199989056762e-12	1.79332288106717e-11	1.17373266533427e-11	CHRFAM7A/CHRM2/CHRM4/CHRM5/CHRNA1/CHRNA3/CHRNA4/CHRNA6/CHRNA7/CHRNB2/CHRNB4/CHRNG	12	5.71428571428571
GO:0005246	MF	GO:0005246	calcium channel regulator activity	17/665	54/18369	3.47757116169305e-12	6.06836167715437e-11	3.97175232677575e-11	CACNG4/CACNG7/CACNG8/F2/F2RL1/F2RL2/FKBP1B/GNA11/GRM2/ITPR1/NPY/NPY2R/NRXN1/NRXN2/PRKCB/PRKG1/STX1A	17	3.14814814814815
GO:0017046	MF	GO:0017046	peptide hormone binding	16/665	48/18369	5.58318677204855e-12	9.50503504119485e-11	6.22106048541482e-11	ADCYAP1R1/CCKAR/EDNRB/GALR1/GALR3/GCGR/GHR/GHRHR/GLP2R/INHBA/MC3R/OXTR/PTH1R/SCTR/VIPR1/VIPR2	16	3.33333333333333
GO:0001614	MF	GO:0001614	purinergic nucleotide receptor activity	11/665	20/18369	1.60998559574457e-11	2.61341847867374e-10	1.71048653256583e-10	GPR171/P2RX1/P2RX2/P2RX4/P2RX7/P2RY1/P2RY13/P2RY14/P2RY2/P2RY4/P2RY8	11	5.5
GO:0016502	MF	GO:0016502	nucleotide receptor activity	11/665	20/18369	1.60998559574457e-11	2.61341847867374e-10	1.71048653256583e-10	GPR171/P2RX1/P2RX2/P2RX4/P2RX7/P2RY1/P2RY13/P2RY14/P2RY2/P2RY4/P2RY8	11	5.5
GO:0005544	MF	GO:0005544	calcium-dependent phospholipid binding	16/665	52/18369	2.24186377612408e-11	3.5564111721241e-10	2.32767674363122e-10	CPNE1/CPNE5/CPNE9/PLA2G4B/PLA2G4D/PLA2G4E/RPH3A/SYT10/SYT11/SYT12/SYT2/SYT4/SYT5/SYT7/SYT8/SYT9	16	3.07692307692308
GO:0099589	MF	GO:0099589	serotonin receptor activity	12/665	27/18369	4.85210418944902e-11	7.52615272052314e-10	4.92587887303128e-10	CHRM2/CHRM4/CHRM5/DRD4/HTR1D/HTR2B/HTR2C/HTR3A/HTR3C/HTR3E/HTR4/HTR7	12	4.44444444444444
GO:0022848	MF	GO:0022848	acetylcholine-gated monoatomic cation-selective channel activity	10/665	17/18369	5.59250474602393e-11	8.30546449515894e-10	5.43593966354845e-10	CHRFAM7A/CHRNA1/CHRNA3/CHRNA4/CHRNA6/CHRNA7/CHRNB2/CHRNB4/CHRND/CHRNG	10	5.88235294117647
GO:0042165	MF	GO:0042165	neurotransmitter binding	10/665	17/18369	5.59250474602393e-11	8.30546449515894e-10	5.43593966354845e-10	CHRFAM7A/CHRNA1/CHRNA3/CHRNA4/CHRNA7/CHRNB2/CHRND/GRIN2D/GRIN3A/GRIN3B	10	5.88235294117647

	ONTOLOGY	ID	Description	GeneRatio	BgRatio	pvalue	p.adjust	qvalue	geneID	Count	richFactor
GO:0015081	MF	GO:0015081	sodium ion transmembrane transporter activity	26/665	155/18369	6.11195811889144e-11	8.88780576455464e-10	5.81708294648878e-10	ASIC1/ATP1A2/GRIK1/GRIK2/GRIK3/GRIK5/HCN1/SCN1B/SCN2B/SLC17A7/SLC17A8/SLC18A2/SLC1A1/SLC1A2/SLC1A7/SLC28A2/SLC38A3/SLC38A5/SLC4A8/SLC5A7/SLC6A1/SLC6A15/SLC6A2/SLC6A3/SLC6A6/SLC6A9	26	1.67741935483871
GO:0031681	MF	GO:0031681	G-protein beta-subunit binding	11/665	22/18369	6.32828778914984e-11	9.01458138127874e-10	5.9000577883803e-10	F2RL1/GNG10/GNG11/GNG12/GNG13/GNG2/GNG4/GNG7/GNG8/GNGT1/OPRM1	11	5
GO:0004953	MF	GO:0004953	icosanoid receptor activity	9/665	14/18369	1.72343721199082e-10	2.40591834793919e-09	1.57467736842951e-09	CYSLTR1/CYSLTR2/LTB4R/LTB4R2/PTGDR/PTGDR2/PTGER3/PTGER4/PTGFR	9	6.42857142857143
GO:0015108	MF	GO:0015108	chloride transmembrane transporter activity	20/665	107/18369	1.40601212406917e-09	1.92430678941232e-08	1.25946184075546e-08	CLCN1/GABRA3/GABRA4/GABRB1/GABRB3/GABRE/GABRG2/GLRA3/GLRB/NMUR2/SLC17A7/SLC17A8/SLC18A2/SLC1A1/SLC1A7/SLC4A8/SLC6A1/SLC6A2/SLC6A3/SLC6A6	20	1.86915887850467
GO:0016917	MF	GO:0016917	GABA receptor activity	10/665	22/18369	1.5774488403436e-09	2.0893533858484e-08	1.36748509946947e-08	GABRA3/GABRA4/GABRB1/GABRB3/GABRD/GABRE/GABRG2/GABRP/GABRR1/GPR156	10	4.54545454545454
GO:0015085	MF	GO:0015085	calcium ion transmembrane transporter activity	23/665	142/18369	1.58647176862414e-09	2.0893533858484e-08	1.36748509946947e-08	ATP2A1/ATP2A3/CACNA1A/CACNA1C/CACNA1D/CACNG4/CACNG5/CACNG7/CACNG8/CHRNA7/FKBP1B/GPM6A/GRIN2A/GRIN2B/GRIN2D/GRIN3A/GRIN3B/ITPR1/OPRM1/P2RX4/RYR1/RYR3/TRPC1	23	1.61971830985915
GO:0005109	MF	GO:0005109	frizzled binding	12/665	37/18369	3.70681273286283e-09	4.7913986806264e-08	3.13597802351359e-08	WNT1/WNT11/WNT16/WNT2/WNT2B/WNT3/WNT3A/WNT5A/WNT7A/WNT7B/WNT8B/WNT9A	12	3.24324324324324
GO:0005262	MF	GO:0005262	calcium channel activity	21/665	126/18369	4.7970591660629e-09	6.08790417802164e-08	3.98454292453837e-08	CACNA1A/CACNA1C/CACNA1D/CACNG4/CACNG5/CACNG7/CACNG8/CHRNA7/FKBP1B/GPM6A/GRIN2A/GRIN2B/GRIN2D/GRIN3A/GRIN3B/ITPR1/OPRM1/P2RX4/RYR1/RYR3/TRPC1	21	1.66666666666667
GO:0005244	MF	GO:0005244	voltage-gated monoatomic ion channel activity	26/665	189/18369	5.06287485157900e-09	6.19980113403884e-08	4.05777965943727e-08	CACNA1A/CACNA1C/CACNA1D/CACNG4/CACNG5/CACNG7/CACNG8/CLCN1/GRIN2A/GRIN2B/GRIN2D/GRIN3A/GRIN3B/HCN1/KCNA1/KCNA2/KCNJ14/KCNJ5/KCNJ9/KCNQ1/KCNQ5/OPRM1/PTK2B/RYR1/SCN1B/SCN2B	26	1.37566137566138
GO:0022832	MF	GO:0022832	voltage-gated channel activity	26/665	189/18369	5.06287485157900e-09	6.19980113403884e-08	4.05777965943727e-08	CACNA1A/CACNA1C/CACNA1D/CACNG4/CACNG5/CACNG7/CACNG8/CLCN1/GRIN2A/GRIN2B/GRIN2D/GRIN3A/GRIN3B/HCN1/KCNA1/KCNA2/KCNJ14/KCNJ5/KCNJ9/KCNQ1/KCNQ5/OPRM1/PTK2B/RYR1/SCN1B/SCN2B	26	1.37566137566138
GO:0004890	MF	GO:0004890	GABA-A receptor activity	9/665	19/18369	6.75572833063552e-09	7.99237012675186e-08	5.23101890364999e-08	GABRA3/GABRA4/GABRB1/GABRB3/GABRD/GABRE/GABRG2/GABRP/GABRR1	9	4.73684210526316

	ONTOLOGY	ID	Description	GeneRatio	BgRatio	pvalue	p.adjust	qvalue	geneID	Count	richFactor
GO:0099095	MF	GO:0099095	ligand-gated monoatomic anion channel activity	9/665	19/18369	6.75572833063552e-09	7.99237012675186e-08	5.23101890364999e-08	GABRA3/GABRA4/GABRB1/GABRB3/GABRE/GABRG2/GLRA3/GLRB/SLC1A7	9	4.73684210526316
GO:0008509	MF	GO:0008509	monoatomic anion transmembrane transporter activity	20/665	123/18369	1.72231015464786e-08	2.00362081324034e-07	1.3113729949424e-07	CLCN1/GABRA3/GABRA4/GABRB1/GABRB3/GABRE/GABRG2/GLRA3/GLRB/NMUR2/SLC17A7/SLC17A8/SLC18A2/SLC1A1/SLC1A7/SLC4A8/SLC6A1/SLC6A2/SLC6A3/SLC6A6	20	1.6260162601626
GO:0015370	MF	GO:0015370	solute:sodium symporter activity	16/665	79/18369	1.87745966791597e-08	2.14830630853336e-07	1.40606988071705e-07	SLC17A7/SLC17A8/SLC18A2/SLC1A1/SLC1A2/SLC1A7/SLC28A2/SLC38A3/SLC4A8/SLC5A7/SLC6A1/SLC6A15/SLC6A2/SLC6A3/SLC6A6/SLC6A9	16	2.0253164556962
GO:0042166	MF	GO:0042166	acetylcholine binding	7/665	11/18369	2.29668286212295e-08	2.5856203834868e-07	1.69229263524849e-07	CHRFAM7A/CHRNA1/CHRNA3/CHRNA4/CHRNA7/CHRNB2/CHRND	7	6.36363636363636
GO:0022853	MF	GO:0022853	active monoatomic ion transmembrane transporter activity	28/665	233/18369	2.71148976420603e-08	3.00415850066002e-07	1.96622649568157e-07	ATP1A2/ATP2A1/ATP2A3/ATP6V0A4/ATP6V0D2/ATP6V1C2/ATP6V1E2/ATP6V1F/ATP6V1G2/COX7A1/SLC17A7/SLC17A8/SLC18A2/SLC18A3/SLC1A1/SLC1A2/SLC1A7/SLC28A2/SLC38A3/SLC38A5/SLC4A8/SLC5A7/SLC6A1/SLC6A15/SLC6A2/SLC6A3/SLC6A6/SLC6A9	28	1.20171673819742
GO:0030548	MF	GO:0030548	acetylcholine receptor regulator activity	8/665	16/18369	2.81590465078289e-08	3.0238483788407e-07	1.97911355212919e-07	GPIHBP1/LY6E/LY6G6D/LY6H/LYNX1/LYPD6B/SLURP1/TMEM35A	8	5
GO:0099602	MF	GO:0099602	neurotransmitter receptor regulator activity	8/665	16/18369	2.81590465078289e-08	3.0238483788407e-07	1.97911355212919e-07	GPIHBP1/LY6E/LY6G6D/LY6H/LYNX1/LYPD6B/SLURP1/TMEM35A	8	5
GO:0004993	MF	GO:0004993	G protein-coupled serotonin receptor activity	9/665	22/18369	3.29900006786439e-08	3.48894249601416e-07	2.28351838828253e-07	CHRM2/CHRM4/CHRM5/DRD4/HTR1D/HTR2B/HTR2C/HTR4/HTR7	9	4.09090909090909
GO:0000149	MF	GO:0000149	SNARE binding	18/665	107/18369	5.18586554482814e-08	5.40258828401499e-07	3.53600258673278e-07	CACNA1A/CPLX1/LRRK2/SNAP25/SNCA/STX1A/STXBP1/SYT10/SYT11/SYT12/SYT2/SYT4/SYT5/SYT7/SYT8/SYT9/UNC13C/VAMP2	18	1.68224299065421
GO:0001540	MF	GO:0001540	amyloid-beta binding	16/665	85/18369	5.59702008077013e-08	5.74517649467287e-07	3.76022711308705e-07	ADRB2/BCHE/CACNA1A/CHRNA7/CLU/EPHA4/EPHB2/GRIA1/GRIA2/GRIA3/GRIA4/GRIN2A/GRIN2B/NGFR/NLGN1/PRNP	16	1.88235294117647
GO:0004435	MF	GO:0004435	phosphatidylinositol phospholipase C activity	9/665	24/18369	8.12508483850698e-08	8.21928872069257e-07	5.37953748270332e-07	BDKRB2/CCL5/CHRM5/EDNRA/F2RL2/PLCB4/PLCG1/PLCG2/PLCL1	9	3.75
GO:0045028	MF	GO:0045028	G protein-coupled purinergic nucleotide receptor activity	7/665	13/18369	1.12070878032835e-07	1.1175067552417e-06	7.31409940845873e-07	GPR171/P2RY1/P2RY13/P2RY14/P2RY2/P2RY4/P2RY8	7	5.38461538461538
GO:0016248	MF	GO:0016248	channel inhibitor activity	11/665	41/18369	1.5121064357619e-07	1.48654970727015e-06	9.72949137317519e-07	BCL2/CAMK2D/CAV1/F2RL1/FKBP1B/ITPR1/KCNMB2/LYNX1/NEDD4L/SCN1B/STX1A	11	2.68292682926829
GO:0015103	MF	GO:0015103	inorganic anion transmembrane transporter activity	20/665	141/18369	1.78927910980723e-07	1.73460669256312e-06	1.13530282698295e-06	CLCN1/GABRA3/GABRA4/GABRB1/GABRB3/GABRE/GABRG2/GLRA3/GLRB/NMUR2/SLC17A7/SLC17A8/SLC18A2/SLC1A1/SLC1A7/SLC4A8/SLC6A1/SLC6A2/SLC6A3/SLC6A6	20	1.41843971631206

	ONTOLOGY	ID	Description	GeneRatio	BgRatio	pvalue	p.adjust	qvalue	geneID	Count	richFactor
GO:0004629	MF	GO:0004629	phospholipase C activity	9/665	27/18369	2.64171574087849e-07	2.52591450292217e-06	1.65321504187637e-06	BDKRB2/CCL5/CHRM5/EDNRA/F2RL2/PLCB4/PLCG1/PLCG2/PLCL1	9	3.33333333333333
GO:0045499	MF	GO:0045499	chemorepellent activity	9/665	28/18369	3.7685456912834e-07	3.5546552601565e-06	2.32652749646799e-06	EFNA5/FLRT2/NRG3/SEMA3D/SEMA3E/SEMA3F/SEMA3G/SEMA6A/SEMA6D	9	3.21428571428571
GO:0097110	MF	GO:0097110	scaffold protein binding	13/665	66/18369	5.69699153111663e-07	5.22817234115603e-06	3.4218470759489e-06	ADCY5/GRID2/GRM2/HOMER1/IL6ST/KCNQ1/NLGN1/NLGN3/NLGN4X/NLGN4Y/NOS1/P2RY1/PLCG2	13	1.96969696969697
GO:0005283	MF	GO:0005283	amino acid:sodium symporter activity	8/665	22/18369	5.76746805543e-07	5.22817234115603e-06	3.4218470759489e-06	SLC1A1/SLC1A2/SLC1A7/SLC38A3/SLC6A1/SLC6A15/SLC6A6/SLC6A9	8	3.63636363636364
GO:0031683	MF	GO:0031683	G-protein beta/gamma-subunit complex binding	8/665	22/18369	5.76746805543e-07	5.22817234115603e-06	3.4218470759489e-06	GNA11/GNA13/GNAI1/GNAL/GNAO1/GNAQ/GNAZ/PIK3R5	8	3.63636363636364
GO:0008504	MF	GO:0008504	monoamine transmembrane transporter activity	6/665	11/18369	8.70623987165587e-07	7.79096850053308e-06	5.09920122037605e-06	SLC18A2/SLC18A3/SLC22A2/SLC22A3/SLC6A2/SLC6A3	6	5.45454545454545
GO:0008200	MF	GO:0008200	ion channel inhibitor activity	10/665	40/18369	1.14570977588083e-06	1.01002039041367e-05	6.61059944864325e-06	CAMK2D/CAV1/F2RL1/FKBP1B/ITPR1/KCNMB2/LYNX1/NEDD4L/SCN1B/STX1A	10	2.5
GO:0030276	MF	GO:0030276	clathrin binding	13/665	70/18369	1.1576164933108e-06	1.01002039041367e-05	6.61059944864325e-06	CALY/DNER/LRRK2/NSG1/SYT10/SYT11/SYT12/SYT2/SYT4/SYT5/SYT7/SYT8/SYT9	13	1.85714285714286
GO:0005125	MF	GO:0005125	cytokine activity	25/665	235/18369	1.51279621205319e-06	1.30361945186806e-05	8.53220995491989e-06	CCL5/CRLF1/CSF1/CXCL12/CXCL13/CXCL8/GDF15/IL1A/INHBA/MIF/SCG2/SLURP1/VEGFA/WNT1/WNT11/WNT16/WNT2/WNT2B/WNT3/WNT3A/WNT5A/WNT7A/WNT7B/WNT8B/WNT9A	25	1.06382978723404
GO:0001786	MF	GO:0001786	phosphatidylserine binding	12/665	62/18369	1.88601773238503e-06	1.60541509415213e-05	1.05074672125173e-05	AXL/CPNE1/GAP43/SYT10/SYT11/SYT12/SYT2/SYT4/SYT5/SYT7/SYT8/SYT9	12	1.93548387096774
GO:0072341	MF	GO:0072341	modified amino acid binding	14/665	85/18369	2.04497396123174e-06	1.71974918667441e-05	1.12557856585235e-05	AXL/CPNE1/GAP43/GPR143/NOX4/SYT10/SYT11/SYT12/SYT2/SYT4/SYT5/SYT7/SYT8/SYT9	14	1.64705882352941
GO:0005254	MF	GO:0005254	chloride channel activity	13/665	74/18369	2.23458736818163e-06	1.85683569403664e-05	1.21530190199352e-05	CLCN1/GABRA3/GABRA4/GABRB1/GABRB3/GABRE/GABRG2/GLRA3/GLRB/NMUR2/SLC17A7/SLC17A8/SLC1A7	13	1.75675675675676
GO:0099604	MF	GO:0099604	ligand-gated calcium channel activity	8/665	26/18369	2.47792648730848e-06	2.03481492722508e-05	1.33178959194041e-05	FKBP1B/GRIN2A/GRIN2B/GRIN2D/ITPR1/P2RX4/RYR1/RYR3	8	3.07692307692308
GO:0051428	MF	GO:0051428	peptide hormone receptor binding	7/665	19/18369	2.72039113429394e-06	2.16744062704111e-05	1.41859332247902e-05	ADCYAP1/GNAO1/LEP/UCN/UCN2/UCN3/VIP	7	3.68421052631579
GO:0005253	MF	GO:0005253	monoatomic anion channel activity	14/665	87/18369	2.72319651017994e-06	2.16744062704111e-05	1.41859332247902e-05	CLCN1/GABRA3/GABRA4/GABRB1/GABRB3/GABRE/GABRG2/GLRA3/GLRB/NMUR2/SLC17A7/SLC17A8/SLC1A1/SLC1A7	14	1.60919540229885
GO:0015294	MF	GO:0015294	solute:monoatomic cation symporter activity	16/665	112/18369	2.73258990228678e-06	2.16744062704111e-05	1.41859332247902e-05	SLC17A7/SLC17A8/SLC18A2/SLC1A1/SLC1A2/SLC1A7/SLC28A2/SLC38A3/SLC4A8/SLC5A7/SLC6A1/SLC6A15/SLC6A2/SLC6A3/SLC6A6/SLC6A9	16	1.42857142857143
GO:0022851	MF	GO:0022851	GABA-gated chloride ion channel activity	6/665	13/18369	3.03852276623232e-06	2.38302122565186e-05	1.55969116563551e-05	GABRA3/GABRA4/GABRB1/GABRB3/GABRE/GABRG2	6	4.61538461538462

	ONTOLOGY	ID	Description	GeneRatio	BgRatio	pvalue	p.adjust	qvalue	geneID	Count	richFactor
GO:0022843	MF	GO:0022843	voltage-gated monoatomic cation channel activity	18/665	140/18369	3.125143236200 35e-06	2.423722198742 05e-05	1.586330016971 87e-05	CACNA1A/CACNA1C/CACNA1D/CACNG4/CACNG5/CACNG7/CACNG8/HCN1/KCNA1/KCNA2/KCNJ14/KCNJ5/KCNJ9/KCNQ1/KCNQ5/OPRM1/RYR1/SCN2B	18	1.285714285714 29
GO:0044325	MF	GO:0044325	transmembrane transporter binding	17/665	129/18369	4.161532648122 08e-06	3.192032734493 64e-05	2.089190479219 18e-05	ANK3/ATP2A3/CAMK2D/CAV1/FGF12/FGF13/FKBP1B/HOMER1/KCNQ1/LRRK2/NEDD4L/NOS1/PRNP/RIMS1/RIMS3/RIMS4/STX1A	17	1.317829457364 34
GO:0005416	MF	GO:0005416	amino acid:monoatomic cation symporter activity	8/665	28/18369	4.623305519215 82e-06	3.507681796100 7e-05	2.295783289862 32e-05	SLC1A1/SLC1A2/SLC1A7/SLC38A3/SLC6A1/SLC6A15/SLC6A6/SLC6A9	8	2.857142857142 86
GO:0005313	MF	GO:0005313	L-glutamate transmembrane transporter activity	6/665	14/18369	5.154564621707 36e-06	3.868694737582 51e-05	2.532066831715 9e-05	SLC17A7/SLC17A8/SLC1A1/SLC1A2/SLC1A7/SLC7A11	6	4.285714285714 29
GO:0019905	MF	GO:0019905	syntaxin binding	12/665	68/18369	5.213571965180 64e-06	3.871354501804 35e-05	2.533807651610 75e-05	CACNA1A/CPLX1/LRRK2/SNAP25/STXBP1/SYT10/SYT2/SYT5/SYT7/SYT8/UNC13C/VAMP2	12	1.764705882352 94
GO:0005237	MF	GO:0005237	inhibitory extracellular ligand-gated monoatomic ion channel activity	6/665	15/18369	8.328006151068 45e-06	6.027638488704 2e-05	3.945098935451 09e-05	GABRA3/GABRA4/GABRE/GABRG2/GLRA3/GLRB	6	4
GO:0099103	MF	GO:0099103	channel activator activity	6/665	15/18369	8.328006151068 45e-06	6.027638488704 2e-05	3.945098935451 09e-05	ADRA2A/F2/F2RL1/F2RL2/GNA11/ITPR1	6	4
GO:0015171	MF	GO:0015171	amino acid transmembrane transporter activity	13/665	83/18369	8.376517670548 81e-06	6.027638488704 2e-05	3.945098935451 09e-05	SLC17A7/SLC17A8/SLC1A1/SLC1A2/SLC1A7/SLC22A2/SLC38A3/SLC38A5/SLC6A1/SLC6A15/SLC6A6/SLC6A9/SLC7A11	13	1.566265060240 96
GO:0002020	MF	GO:0002020	protease binding	17/665	138/18369	1.043396718839 36e-05	7.431539895406 87e-05	4.863954629175 96e-05	A2M/ADRM1/BCL2/BDKRB2/CHL1/COL3A1/CTSG/F2RL1/ITGB3/MAGEA3/MIF/NDUFS7/PINK1/PRKN/PRNP/RIPK2/SLC6A3	17	1.231884057971 01
GO:0098632	MF	GO:0098632	cell-cell adhesion mediator activity	10/665	51/18369	1.205644309232 81e-05	8.500401291358 6e-05	5.563526105338 01e-05	CNTN2/CNTN4/CNTN6/DSCAML1/IGSF9/MYPN/NEXN/NRCAM/NTNG1/PALLD	10	1.960784313725 49
GO:0004954	MF	GO:0004954	prostanoid receptor activity	5/665	10/18369	1.326953642441 89e-05	9.170432103212 29e-05	6.002062332670 99e-05	PTGDR/PTGDR2/PTGER3/PTGER4/PTGFR	5	5
GO:0004955	MF	GO:0004955	prostaglandin receptor activity	5/665	10/18369	1.326953642441 89e-05	9.170432103212 29e-05	6.002062332670 99e-05	PTGDR/PTGDR2/PTGER3/PTGER4/PTGFR	5	5
GO:0022804	MF	GO:0022804	active transmembrane transporter activity	34/665	428/18369	1.645541840965 51e-05	0.000112606686 764111	7.370125479659 78e-05	ATP1A2/ATP2A1/ATP2A3/ATP6V0A4/ATP6V0D2/ATP6V1C2/ATP6V1E2/ATP6V1F/ATP6V1G2/COX7A1/NDUFS7/SLC17A7/SLC17A8/SLC18A2/SLC18A3/SLC1A1/SLC1A2/SLC1A7/SLC22A2/SLC22A3/SLC28A2/SLC2A4/SLC38A3/SLC38A5/SLC4A8/SLC5A7/SLC6A1/SLC6A15/SLC6A16/SLC6A2/SLC6A3/SLC6A6/SLC6A9/SLC7A11	34	0.794392523364 486
GO:0050997	MF	GO:0050997	quaternary ammonium group binding	10/665	53/18369	1.723674855250 92e-05	0.000116808257 181082	7.645118928757 28e-05	APOC1/BCHE/CHRFAM7A/CHRNA1/CHRNA3/CHRNA4/CHRNA7/CHRNB2/CHRND/SLC5A7	10	1.886792452830 19

	ONTOLOGY	ID	Description	GeneRatio	BgRatio	pvalue	p.adjust	qvalue	geneID	Count	richFactor
GO:0015293	MF	GO:0015293	symporter activity	17/665	144/18369	1.83895326709366e-05	0.000123422055810709	8.07799309634261e-05	SLC17A7/SLC17A8/SLC18A2/SLC1A1/SLC1A2/SLC1A7/SLC28A2/SLC38A3/SLC4A8/SLC5A7/SLC6A1/SLC6A15/SLC6A16/SLC6A2/SLC6A3/SLC6A6/SLC6A9	17	1.18055555555556
GO:0008083	MF	GO:0008083	growth factor activity	18/665	160/18369	2.04563039945346e-05	0.000135985716077954	8.90028665025364e-05	AGT/BDNF/CSF1/CXCL12/DKK1/F2/FGF12/FGF13/GDF15/GDNF/IGF1/INHBA/NGF/NRG3/NTF4/PTN/VEGFA/VGF	18	1.125
GO:0004620	MF	GO:0004620	phospholipase activity	14/665	104/18369	2.27281709998472e-05	0.000149662861866918	9.79545800787854e-05	BDKRB2/CCL5/CHRM5/EDNRA/F2RL2/MGLL/PLA2G4B/PLA2G4D/PLA2G4E/PLCB4/PLCG1/PLCG2/PLCL1/PLD1	14	1.34615384615385
GO:0008046	MF	GO:0008046	axon guidance receptor activity	5/665	11/18369	2.36019634554634e-05	0.000151139178824894	9.89208318654865e-05	EPHB2/EPHB3/GFRA3/L1CAM/ROBO2	5	4.54545454545454
GO:0043176	MF	GO:0043176	amine binding	5/665	11/18369	2.36019634554634e-05	0.000151139178824894	9.89208318654865e-05	DRD4/HTR2B/HTR2C/HTR3A/SLC6A3	5	4.54545454545454
GO:0099528	MF	GO:0099528	G protein-coupled neurotransmitter receptor activity	5/665	11/18369	2.36019634554634e-05	0.000151139178824894	9.89208318654865e-05	ADRB1/CHRM2/CHRM4/CHRM5/GABRB1	5	4.54545454545454
GO:0016597	MF	GO:0016597	amino acid binding	10/665	55/18369	2.42235844561347e-05	0.000153709654094382	0.000100603211999641	GCHFR/GLRB/GPR143/GRIN2B/GRIN2D/GRIN3A/GRIN3B/NOS1/SLC1A1/TH	10	1.81818181818182
GO:0004896	MF	GO:0004896	cytokine receptor activity	13/665	92/18369	2.62376650825751e-05	0.000164990002050788	0.000107986217599219	CCR2/CCR7/CNTFR/CRLF1/CX3CR1/CXCR5/GFRA1/GFRA2/GHR/IL6R/IL6ST/LEPR/LIFR	13	1.41304347826087
GO:0015172	MF	GO:0015172	acidic amino acid transmembrane transporter activity	6/665	18/18369	2.81425283107875e-05	0.000172311269832716	0.00011277799895551	SLC17A7/SLC17A8/SLC1A1/SLC1A2/SLC1A7/SLC7A11	6	3.33333333333333
GO:0031690	MF	GO:0031690	adrenergic receptor binding	6/665	18/18369	2.81425283107875e-05	0.000172311269832716	0.00011277799895551	ADRA2A/ADRA2C/ADRB1/ADRB3/MAGI2/UCHL1	6	3.33333333333333
GO:0050811	MF	GO:0050811	GABA receptor binding	6/665	18/18369	2.81425283107875e-05	0.000172311269832716	0.00011277799895551	ERBB4/GABARAP/GABRB1/LHFPL4/SHISA7/TRAK2	6	3.33333333333333
GO:0015175	MF	GO:0015175	neutral L-amino acid transmembrane transporter activity	9/665	46/18369	3.35485631697201e-05	0.000203625192108388	0.000133273010669643	SLC1A1/SLC1A2/SLC1A7/SLC38A3/SLC38A5/SLC6A15/SLC6A6/SLC6A9/SLC7A11	9	1.95652173913043
GO:0005343	MF	GO:0005343	organic acid:sodium symporter activity	8/665	36/18369	3.48393143427284e-05	0.000209636563889865	0.000137207463019457	SLC1A1/SLC1A2/SLC1A7/SLC38A3/SLC6A1/SLC6A15/SLC6A6/SLC6A9	8	2.22222222222222
GO:0004713	MF	GO:0004713	protein tyrosine kinase activity	16/665	137/18369	3.67934968498081e-05	0.00021950308377065	0.000143665115904784	AXL/EPHA3/EPHA4/EPHB2/EPHB3/ERBB4/FGFR1/MAP2K6/MATK/NRP2/NTRK3/PTK2B/RIPK2/RPS6KA5/TTK/TYRO3	16	1.16788321167883
GO:0005165	MF	GO:0005165	neurotrophin receptor binding	5/665	12/18369	3.92548997350899e-05	0.000232202711992311	0.000151977042685361	BDNF/EFNA5/NGF/NTF4/PLCG1	5	4.16666666666667
GO:0030165	MF	GO:0030165	PDZ domain binding	12/665	83/18369	4.22702344670288e-05	0.000247938013932656	0.00016227582272172	ADRB1/FZD3/GNG12/GRIA1/GRID2/GRIK5/KIF14/LPAR1/NLGN1/PRKN/SNTG2/SSTR2	12	1.44578313253012
GO:0004714	MF	GO:0004714	transmembrane receptor protein tyrosine kinase activity	10/665	60/18369	5.30609827475472e-05	0.000308638049648233	0.000202004092214346	AXL/EPHA3/EPHA4/EPHB2/EPHB3/ERBB4/FGFR1/NRP2/NTRK3/TYRO3	10	1.66666666666667
GO:0046982	MF	GO:0046982	protein heterodimerization activity	28/665	343/18369	5.63481763639835e-05	0.00032504981076083	0.000212745615850099	ADRA1A/ADRA1B/ADRA2A/ADRA2C/ADRB1/AGTR1/ATP1A2/AURKA/BCL2/BCL2L1/BDKRB2/CAV1/CREB3L3/EPAS1/FZD9/IRAK1/IRAK2/MEF2C/NEUROD1/P2RY1/PIK3R2/SOS2/SYT10/SYT5/TENM1/TOP2A/TUBA1A/TUBB2B	28	0.816326530612245

	ONTOLOGY	ID	Description	GeneRatio	BgRatio	pvalue	p.adjust	qvalue	geneID	Count	richFactor
GO:0039706	MF	GO:0039706	co-receptor binding	5/665	13/18369	6.189013534062 58e-05	0.000354092741 53899	0.000231754260 033059	DKK1/DKK4/LRRK 2/NEO1/WNT3A	5	3.846153846153 85
GO:0098631	MF	GO:0098631	cell adhesion mediator activity	10/665	62/18369	7.090496317504 11e-05	0.000402371254 440477	0.000263352623 174735	CNTN2/CNTN4/C NTN6/DSCAML1/I GSF9/MYPN/NEX N/NRCAM/NTNG1 /PALLD	10	1.612903225806 45
GO:0019955	MF	GO:0019955	cytokine binding	15/665	130/18369	7.353531167531 97e-05	0.000413932641 527203	0.000270919569 330125	A2M/CCR7/CHRD L1/CNTFR/CRLF1/ CX3CR1/GHR/IL6 R/IL6ST/ITGA4/IT GB3/LEPR/LIFR/N RP2/PDPN	15	1.153846153846 15
GO:0019838	MF	GO:0019838	growth factor binding	15/665	132/18369	8.765567966567 44e-05	0.000489469315 253126	0.000320358441 893917	A2M/CHRDL1/CO L3A1/CXCL13/FG FR1/FSTL4/GHR/ GHRHR/IL6R/IL6S T/ITGB3/LIFR/NG FR/NRP2/NTRK3	15	1.136363636363 64
GO:0005245	MF	GO:0005245	voltage-gated calcium channel activity	9/665	52/18369	9.231549560193 44e-05	0.000511355146 876465	0.000334682753 346985	CACNA1A/CACNA 1C/CACNA1D/CA CNG4/CACNG5/C ACNG7/CACNG8/ OPRM1/RYR1	9	1.730769230769 23
GO:1901474	MF	GO:1901474	azole transmembrane transporter activity	5/665	14/18369	9.340989685113 62e-05	0.000511355146 876465	0.000334682753 346985	SLC22A2/SLC28A 2/SLC38A3/SLC3 8A5/SLC44A4	5	3.571428571428 57
GO:0005272	MF	GO:0005272	sodium channel activity	8/665	41/18369	9.377286361058 39e-05	0.000511355146 876465	0.000334682753 346985	ASIC1/GRIK1/GRI K2/GRIK3/GRIK5/ HCN1/SCN1B/SC N2B	8	1.951219512195 12
GO:0051427	MF	GO:0051427	hormone receptor binding	7/665	31/18369	9.718851827244 54e-05	0.000525872757 784239	0.000344184552 674348	ADCYAP1/GNAO1 /LEP/UCN/UCN2/ UCN3/VIP	7	2.258064516129 03
GO:0019956	MF	GO:0019956	chemokine binding	6/665	22/18369	9.993240295306 4e-05	0.000536560132 778759	0.000351179456 531415	A2M/CCR7/CX3C R1/ITGA4/ITGB3/ PDPN	6	2.727272727272 73
GO:0015179	MF	GO:0015179	L-amino acid transmembrane transporter activity	10/665	65/18369	0.000107088323 756349	0.000569359192 474362	0.000372646493 038566	SLC17A7/SLC17A 8/SLC1A1/SLC1A 2/SLC1A7/SLC22 A2/SLC38A3/SLC 38A5/SLC6A9/SL C7A11	10	1.538461538461 54
GO:0019829	MF	GO:0019829	ATPase-coupled monoatomic cation transmembrane transporter activity	9/665	53/18369	0.000107672512 043862	0.000569359192 474362	0.000372646493 038566	ATP1A2/ATP2A1/ ATP2A3/ATP6V0A 4/ATP6V0D2/ATP 6V1C2/ATP6V1E2 /ATP6V1F/ATP6V 1G2	9	1.698113207547 17
GO:0015101	MF	GO:0015101	organic cation transmembrane transporter activity	7/665	32/18369	0.000120547563 793655	0.000632648116 751665	0.000414069194 194274	SLC18A3/SLC22A 2/SLC22A3/SLC4 4A4/SLC5A7/SLC 6A2/SLC6A3	7	2.1875
GO:0005267	MF	GO:0005267	potassium channel activity	14/665	121/18369	0.000123046234 866135	0.000640942327 884791	0.000419497768 514552	GRIK1/GRIK2/GRI K3/GRIK5/HCN1/ KCNA1/KCNA2/K CNJ14/KCNJ5/KCN J9/KCNMB2/KCNQ 1/KCNQ5/SCN2B	14	1.157024793388 43
GO:0005516	MF	GO:0005516	calmodulin binding	19/665	200/18369	0.000125335624 167771	0.000648031597 548917	0.000424137706 73538	ADCY3/ASPM/CA CNA1C/CAMK2A/ CAMK2B/CAMK2 D/CAMK4/FAS/GA P43/KCNQ1/MAP 2/NGFR/NOS1/RG S2/RYR1/RYR3/SY T7/UNC13C/VAM P2	19	0.95
GO:0030215	MF	GO:0030215	semaphorin receptor binding	6/665	23/18369	0.000131086652 517049	0.000672782966 594856	0.000440337516 969035	SEMA3D/SEMA3E /SEMA3F/SEMA3 G/SEMA6A/SEMA 6D	6	2.608695652173 91
GO:0005126	MF	GO:0005126	cytokine receptor binding	23/665	270/18369	0.000135695650 70182	0.000687635419 249395	0.000450058470 749868	BDNF/BID/CCL5/C CR2/CRLF1/CSF1/ CX3CR1/CXCL12/ CXCL13/CXCL8/E FNA5/IL1A/IL6R/I L6ST/ITGB3/LIFR/ MIF/NGF/NTF4/PL CG1/PRL/TRAP1/ VEGFA	23	0.851851851851 852
GO:0015378	MF	GO:0015378	sodium:chloride symporter activity	5/665	15/18369	0.000135950842 20117	0.000687635419 249395	0.000450058470 749868	SLC18A2/SLC6A1 /SLC6A2/SLC6A3/ SLC6A6	5	3.333333333333 33

	ONTOLOGY	ID	Description	GeneRatio	BgRatio	pvalue	p.adjust	qvalue	geneID	Count	richFactor
GO:0106310	MF	GO:0106310	protein serine kinase activity	28/665	363/18369	0.000148868991620614	0.000747557957922222	0.00048927786719687	AKT3/AURKA/BUB1B/CAMK2A/CAMK2B/CAMK2D/CAMK4/CDK1/CSNK1A1L/CSNK1E/DCLK1/IRAK1/LIMK1/LRRK2/MAP2K6/MAPK10/PAK6/PIK3CG/PINK1/PRKACB/PRKCB/PRKCG/PRKG1/PRKG2/RIPK2/RPS6KA5/RPS6KA6/TTK	28	0.771349862258953
GO:0042625	MF	GO:0042625	ATPase-coupled ion transmembrane transporter activity	6/665	24/18369	0.000169464924125995	0.000833003641126368	0.000545202202154794	ATP6V0A4/ATP6V0D2/ATP6V1C2/ATP6V1E2/ATP6V1F/ATP6V1G2	6	2.5
GO:0044769	MF	GO:0044769	ATPase activity, coupled to transmembrane movement of ions, rotational mechanism	6/665	24/18369	0.000169464924125995	0.000833003641126368	0.000545202202154794	ATP6V0A4/ATP6V0D2/ATP6V1C2/ATP6V1E2/ATP6V1F/ATP6V1G2	6	2.5
GO:0046961	MF	GO:0046961	proton-transporting ATPase activity, rotational mechanism	6/665	24/18369	0.000169464924125995	0.000833003641126368	0.000545202202154794	ATP6V0A4/ATP6V0D2/ATP6V1C2/ATP6V1E2/ATP6V1F/ATP6V1G2	6	2.5
GO:0035254	MF	GO:0035254	glutamate receptor binding	8/665	45/18369	0.000186229609012059	0.000906588793517301	0.000593363800914656	CACNG4/CAMK2A/DRD2/HOMER1/NSG1/PLCG1/PRNP/SHISA7	8	1.77777777777778
GO:0015079	MF	GO:0015079	potassium ion transmembrane transporter activity	16/665	157/18369	0.000188295618362661	0.000906588793517301	0.000593363800914656	ATP1A2/GRIK1/GRIK2/GRIK3/GRIK5/HCN1/KCNA1/KCNA2/KCNJ14/KCNJ5/KCNJ9/KCNMB2/KCNQ1/KCNQ5/SCN2B/SLC17A7	16	1.01910828025478
GO:0005200	MF	GO:0005200	structural constituent of cytoskeleton	13/665	111/18369	0.000188331482893995	0.000906588793517301	0.000593363800914656	ANK3/KRT6B/NEFH/NEFL/NEFM/SPTBN2/TUBA1A/TUBAL3/TUBB2A/TUBB2B/TUBB3/TUBB4A/TUBB6	13	1.17117117117117
GO:0016298	MF	GO:0016298	lipase activity	14/665	126/18369	0.000189802137929608	0.00090741022106073	0.000593901426542538	BDKRB2/CCL5/CHRM5/EDNRA/F2RL2/MGLL/PLA2G4B/PLA2G4D/PLA2G4E/PLCB4/PLCG1/PLCG2/PLCL1/PLD1	14	1.11111111111111
GO:0015373	MF	GO:0015373	monoatomic anion:sodium symporter activity	5/665	16/18369	0.000191875200059019	0.000911080881912891	0.000596303879882664	SLC18A2/SLC6A1/SLC6A2/SLC6A3/SLC6A6	5	3.125
GO:0042887	MF	GO:0042887	amide transmembrane transporter activity	8/665	46/18369	0.00021837298708413	0.00102989422286975	0.000674067399676474	SLC17A7/SLC17A8/SLC1A1/SLC1A2/SLC1A7/SLC38A3/SLC38A5/SLC7A11	8	1.73913043478261
GO:0008569	MF	GO:0008569	minus-end-directed microtubule motor activity	5/665	17/18369	0.000263759517869289	0.0012355982783407	0.000808701029708736	DNAH10/DNAH12/DNAH14/DNAH2/DNAH5	5	2.94117647058824
GO:0043121	MF	GO:0043121	neurotrophin binding	4/665	10/18369	0.000300248398546878	0.00138790319328292	0.000908384837708922	A2M/FSTL4/NGFR/NTRK3	4	4
GO:0051378	MF	GO:0051378	serotonin binding	4/665	10/18369	0.000300248398546878	0.00138790319328292	0.000908384837708922	DRD4/HTR2B/HTR2C/HTR3A	4	4
GO:0017080	MF	GO:0017080	sodium channel regulator activity	7/665	37/18369	0.000315131551094343	0.00144711725436744	0.000947140534452526	CAMK2D/FGF12/FGF13/NEDD4L/NOS1/SCN1B/SCN2B	7	1.89189189189189
GO:0009678	MF	GO:0009678	pyrophosphate hydrolysis-driven proton transmembrane transporter activity	6/665	27/18369	0.00033974947402999	0.00154996818871198	0.00101445663384256	ATP6V0A4/ATP6V0D2/ATP6V1C2/ATP6V1E2/ATP6V1F/ATP6V1G2	6	2.22222222222222
GO:0004674	MF	GO:0004674	protein serine/threonine kinase activity	30/665	421/18369	0.000346256622213954	0.00156939689808662	0.00102717275489307	AKT3/AURKA/BUB1B/CAMK2A/CAMK2B/CAMK2D/CAMK4/CDK1/CSNK1A1L/CSNK1E/DCLK1/IRAK1/IRAK2/LIMK1/LRRK2/MAP2K6/MAPK10/PAK6/PIK3CG/PINK1/PRKACB/PRKCB/PRKCG/PRKG1/PRKG2/PTK2B/RIPK2/RPS6KA5/RPS6KA6/TTK	30	0.712589073634204

	ONTOLOGY	ID	Description	GeneRatio	BgRatio	pvalue	p.adjust	qvalue	geneID	Count	richFactor
GO:0005003	MF	GO:0005003	ephrin receptor activity	5/665	18/18369	0.000354381436930251	0.0015856297626751	0.0010377971904705	EPHA3/EPHA4/EPHB2/EPHB3/NTRK3	5	2.77777777777778
GO:0015651	MF	GO:0015651	quaternary ammonium group transmembrane transporter activity	5/665	18/18369	0.000354381436930251	0.0015856297626751	0.0010377971904705	SLC18A3/SLC22A2/SLC22A3/SLC44A4/SLC5A7	5	2.77777777777778
GO:0015291	MF	GO:0015291	secondary active transmembrane transporter activity	21/665	255/18369	0.000412003915048312	0.00183171167327211	0.00119885819061996	SLC17A7/SLC17A8/SLC18A2/SLC18A3/SLC1A1/SLC1A2/SLC1A7/SLC28A2/SLC2A4/SLC38A3/SLC38A5/SLC4A8/SLC5A7/SLC6A1/SLC6A15/SLC6A16/SLC6A2/SLC6A3/SLC6A6/SLC6A9/SLC7A11	21	0.823529411764706
GO:0019199	MF	GO:0019199	transmembrane receptor protein kinase activity	10/665	77/18369	0.000444770537751416	0.001964872375636	0.00128601208117332	AXL/EPHA3/EPHA4/EPHB2/EPHB3/ERBB4/FGFR1/NRP2/NTRK3/TYRO3	10	1.2987012987013
GO:0004016	MF	GO:0004016	adenylate cyclase activity	4/665	11/18369	0.000458332075741971	0.00198705458924159	0.00130053037510308	ADCY2/ADCY3/ADCY5/ADCY9	4	3.63636363636364
GO:0008503	MF	GO:0008503	benzodiazepine receptor activity	4/665	11/18369	0.000458332075741971	0.00198705458924159	0.00130053037510308	GABRA3/GABRA4/GABRE/GABRG2	4	3.63636363636364
GO:0016594	MF	GO:0016594	glycine binding	4/665	11/18369	0.000458332075741971	0.00198705458924159	0.00130053037510308	GLRB/GRIN2B/GRIN3A/GRIN3B	4	3.63636363636364
GO:0005543	MF	GO:0005543	phospholipid binding	32/665	475/18369	0.000591587587199186	0.00254893911027797	0.00166828468384956	APOC1/ARHGAP44/AXL/CPNE1/CPNE5/CPNE9/GAP43/ITPR1/KCNQ1/LPAR1/MAP1B/MAPT/PLA2G4B/PLA2G4D/PLA2G4E/PLD1/RASAL1/RPH3A/SNCA/SPTBN2/SYT10/SYT11/SYT12/SYT2/SYT4/SYT5/SYT7/SYT8/SYT9/UNC13C/VAMP2/WNT5A	32	0.673684210526316
GO:0017075	MF	GO:0017075	syntaxin-1 binding	5/665	20/18369	0.000603863923635784	0.00258587128035446	0.00169245684764566	LRRK2/SNAP25/STXBP1/UNC13C/VAMP2	5	2.5
GO:0008179	MF	GO:0008179	adenylate cyclase binding	4/665	12/18369	0.000667870663745413	0.00282529529269272	0.00184916024284216	ADCY2/ADCY5/ADCYAP1R1/ADRB2	4	3.33333333333333
GO:0017154	MF	GO:0017154	semaphorin receptor activity	4/665	12/18369	0.000667870663745413	0.00282529529269272	0.00184916024284216	NRP2/PLXNA1/PLXNA2/PLXNA3	4	3.33333333333333
GO:0030547	MF	GO:0030547	signaling receptor inhibitor activity	7/665	42/18369	0.000706057061419745	0.00296884234259628	0.00194311201430672	CCL5/DKK1/DKK4/GPIHBP1/LY6E/LY6G6D/LY6H	7	1.66666666666667
GO:0015377	MF	GO:0015377	chloride:monoatomic cation symporter activity	5/665	21/18369	0.000769145852316525	0.0032147533228559	0.00210406114027968	SLC18A2/SLC6A1/SLC6A2/SLC6A3/SLC6A6	5	2.38095238095238
GO:0010854	MF	GO:0010854	adenylate cyclase regulator activity	4/665	13/18369	0.00093719534303643	0.00389382350856802	0.00254851365211661	ADRA2A/F2RL1/GNAZ/RGS2	4	3.07692307692308
GO:0009975	MF	GO:0009975	cyclase activity	5/665	22/18369	0.000965973337423425	0.00398964135811569	0.00261122658637039	ADCY2/ADCY3/ADCY5/ADCY9/GUCY1A1	5	2.27272727272727
GO:0008081	MF	GO:0008081	phosphoric diester hydrolase activity	10/665	86/18369	0.00107353234896254	0.00440779752691677	0.00288491046098911	BDKRB2/CCL5/CHRM5/EDNRA/F2RL2/PLCB4/PLCG1/PLCG2/PLCL1/PLD1	10	1.16279069767442
GO:0008201	MF	GO:0008201	heparin binding	15/665	168/18369	0.00118667874717807	0.00483309041159639	0.00316326532745112	ADGRG1/CTSG/CXCL13/CXCL8/F2/FGFR1/KNG1/NDNF/NRP2/PTN/RTN4R/SLIT2/SLIT3/TENM1/VEGFA	15	0.892857142857143
GO:0015296	MF	GO:0015296	monoatomic anion:monoatomic cation symporter activity	5/665	23/18369	0.00119788630545297	0.00483309041159639	0.00316326532745112	SLC18A2/SLC6A1/SLC6A2/SLC6A3/SLC6A6	5	2.17391304347826
GO:0016849	MF	GO:0016849	phosphorus-oxygen lyase activity	5/665	23/18369	0.00119788630545297	0.00483309041159639	0.00316326532745112	ADCY2/ADCY3/ADCY5/ADCY9/GUCY1A1	5	2.17391304347826
GO:0003777	MF	GO:0003777	microtubule motor activity	9/665	73/18369	0.00124162076861575	0.00498075457755055	0.00325991175789012	DNAH10/DNAH12/DNAH14/DNAH2/DNAH5/KIF14/KIF1A/KIF5A/KIF5C	9	1.23287671232877
GO:0019855	MF	GO:0019855	calcium channel inhibitor activity	4/665	14/18369	0.00127470952151055	0.00505538207962705	0.00330875557616972	F2RL1/FKBP1B/ITPR1/STX1A	4	2.85714285714286
GO:0042043	MF	GO:0042043	neurexin family protein binding	4/665	14/18369	0.00127470952151055	0.00505538207962705	0.00330875557616972	NLGN1/NLGN3/NLGN4X/NLGN4Y	4	2.85714285714286

	ONTOLOGY	ID	Description	GeneRatio	BgRatio	pvalue	p.adjust	qvalue	geneID	Count	richFactor
GO:0010857	MF	GO:0010857	calcium-dependent protein kinase activity	5/665	24/18369	0.00146852351106534	0.00579112661425766	0.00379030153911601	CAMK2B/CAMK4/PINK1/PRKCB/PRKCG	5	2.08333333333333
GO:0005005	MF	GO:0005005	transmembrane-ephrin receptor activity	4/665	15/18369	0.00168880358671396	0.00662238709846261	0.00433436284230549	EPHA3/EPHA4/EPHB2/EPHB3	4	2.66666666666667
GO:0060229	MF	GO:0060229	lipase activator activity	5/665	25/18369	0.00178160406584896	0.00694726054727695	0.00454699302898235	CCL5/F2/F2RL2/GNA11/GPIHBP1	5	2
GO:0140375	MF	GO:0140375	immune receptor activity	13/665	141/18369	0.00184915621741571	0.00717061688753424	0.00469318010735916	CCR2/CCR7/CNTFR/CRLF1/CX3CR1/CXCR5/GFRA1/GFRA2/GHR/IL6R/IL6ST/LEPR/LIFR	13	0.921985815602837
GO:0045505	MF	GO:0045505	dynein intermediate chain binding	6/665	37/18369	0.00196284535930955	0.00756942575026557	0.00495420113951931	DNAH10/DNAH12/DNAH14/DNAH2/DNAH5/FRRS1L	6	1.62162162162162
GO:0005539	MF	GO:0005539	glycosaminoglycan binding	18/665	232/18369	0.0020252304472365	0.00776709259434657	0.00508357440197016	ADGRG1/CTSG/CXCL13/CXCL8/F2/FGFR1/KNG1/NDNF/NRP2/PRNP/PTN/RTN4R/SHH/SLIT2/SLIT3/SULF1/TENM1/VEGFA	18	0.775862068965517
GO:0001965	MF	GO:0001965	G-protein alpha-subunit binding	5/665	26/18369	0.00214090907610232	0.00816587177660886	0.00534457600821632	F2RL1/HTR2B/LPAR1/OPRM1/RGS2	5	1.92307692307692
GO:0004683	MF	GO:0004683	calmodulin-dependent protein kinase activity	5/665	27/18369	0.00255026353111876	0.00962207537686969	0.00629766357044404	CAMK2A/CAMK2B/CAMK2D/CAMK4/PTK2B	5	1.85185185185185
GO:0051959	MF	GO:0051959	dynein light intermediate chain binding	5/665	27/18369	0.00255026353111876	0.00962207537686969	0.00629766357044404	DNAH10/DNAH12/DNAH14/DNAH2/DNAH5	5	1.85185185185185
GO:0042056	MF	GO:0042056	chemoattractant activity	6/665	39/18369	0.00259132361243589	0.00972442947032393	0.00636465448668464	CCL5/GDNF/MIF/SCG2/VEGFA/WNT5A	6	1.53846153846154
GO:0015278	MF	GO:0015278	calcium-release channel activity	4/665	17/18369	0.00277978593028499	0.0103758854510103	0.00679103345760588	FKBP1B/ITPR1/RYR1/RYR3	4	2.35294117647059
GO:0015459	MF	GO:0015459	potassium channel regulator activity	7/665	53/18369	0.0028630253162065	0.0106297429293199	0.00695718357913561	ADRA2A/ADRB2/CAV1/DRD4/F2RL1/KCNMB2/NEDD4L	7	1.32075471698113
GO:0044389	MF	GO:0044389	ubiquitin-like protein ligase binding	22/665	320/18369	0.00316389761757033	0.011684658926265	0.00764762776956571	APC/ARRB1/AURKA/AXIN2/BCL2/BID/CCNB1/CLU/GABARAP/HSP90AB1/MAP1LC3C/MC1R/NGFR/PINK1/PRKACB/PRKN/PTK2B/SNCAIP/SYT11/TXNIP/UCHL1/VCL	22	0.6875
GO:0031625	MF	GO:0031625	ubiquitin protein ligase binding	21/665	301/18369	0.00327238436299215	0.0120217067650975	0.00786822611378721	APC/ARRB1/AURKA/AXIN2/BCL2/BID/CLU/GABARAP/HSP90AB1/MAP1LC3C/MC1R/NGFR/PINK1/PRKACB/PRKN/PTK2B/SNCAIP/SYT11/TXNIP/UCHL1/VCL	21	0.697674418604651
GO:0005217	MF	GO:0005217	intracellular ligand-gated monoatomic ion channel activity	5/665	29/18369	0.00353453419112077	0.012916779400012	0.00845405257066087	FKBP1B/HCN1/ITPR1/RYR1/RYR3	5	1.72413793103448
GO:0003924	MF	GO:0003924	GTPase activity	22/665	327/18369	0.00409427434278237	0.0148843931836567	0.00974185890771683	GNA11/GNA13/GNAI1/GNAL/GNAO1/GNAQ/GNAZ/GNG10/GNG11/GNG8/GNGT1/LRRK2/RAB39B/RAB3B/RAP1A/RGS2/SEPTIN5/TUBB2A/TUBB2B/TUBB3/TUBB4A/TUBB6	22	0.672782874617737
GO:1901681	MF	GO:1901681	sulfur compound binding	19/665	269/18369	0.00438939927566302	0.0158746149969574	0.0103899606525102	ADGRG1/CTSG/CXCL13/CXCL8/F2/FGFR1/KNG1/NDNF/NRP2/P2RX1/PRNP/PTN/RRM2/RTN4R/SLIT2/SLIT3/TENM1/TH/VEGFA	19	0.706319702602231
GO:0005249	MF	GO:0005249	voltage-gated potassium channel activity	9/665	88/18369	0.00453209511580448	0.0163061978908842	0.0106724323399845	HCN1/KCNA1/KCNA2/KCNJ14/KCNJ5/KCNJ9/KCNQ1/KCNQ5/SCN2B	9	1.02272727272727
GO:0016595	MF	GO:0016595	glutamate binding	3/665	10/18369	0.00468432427411294	0.0167674786837479	0.0109743413493388	GRIN2B/GRIN2D/SLC1A1	3	3

	ONTOLOGY	ID	Description	GeneRatio	BgRatio	pvalue	p.adjust	qvalue	geneID	Count	richFactor
GO:0015078	MF	GO:0015078	proton transmembrane transporter activity	12/665	140/18369	0.00493380234873465	0.0175703777521264	0.0114998400609605	ATP6V0A4/ATP6V0D2/ATP6V1C2/ATP6V1E2/ATP6V1F/ATP6V1G2/COX7A1/SLC17A7/SLC18A2/SLC18A3/SLC38A3/SLC38A5	12	0.857142857142857
GO:0005123	MF	GO:0005123	death receptor binding	4/665	20/18369	0.00519200024642386	0.0183960211776845	0.0120402249903711	BDNF/BID/NGF/NTF4	4	2
GO:0019894	MF	GO:0019894	kinesin binding	6/665	45/18369	0.00538833219584546	0.0189952316803037	0.0124324092131682	DCDC2/KCNA2/KIF5A/KLC3/NEFH/SNCA	6	1.33333333333333
GO:0046943	MF	GO:0046943	carboxylic acid transmembrane transporter activity	13/665	162/18369	0.00610975427237372	0.0214301933774716	0.0140260954996572	SLC17A7/SLC17A8/SLC1A1/SLC1A2/SLC1A7/SLC22A2/SLC38A3/SLC38A5/SLC6A1/SLC6A15/SLC6A6/SLC6A9/SLC7A11	13	0.802469135802469
GO:0035173	MF	GO:0035173	histone kinase activity	4/665	21/18369	0.00623267186856676	0.0216438057923363	0.0141659051634353	AURKA/CDK1/PRKCB/RPS6KA5	4	1.9047619047619
GO:0099186	MF	GO:0099186	structural constituent of postsynapse	4/665	21/18369	0.00623267186856676	0.0216438057923363	0.0141659051634353	HOMER1/NEFH/NEFL/SPTBN2	4	1.9047619047619
GO:0005342	MF	GO:0005342	organic acid transmembrane transporter activity	13/665	163/18369	0.00642666030717993	0.0222069747248098	0.0145345001215012	SLC17A7/SLC17A8/SLC1A1/SLC1A2/SLC1A7/SLC22A2/SLC38A3/SLC38A5/SLC6A1/SLC6A15/SLC6A6/SLC6A9/SLC7A11	13	0.797546012269939
GO:0010851	MF	GO:0010851	cyclase regulator activity	4/665	22/18369	0.00740304931993194	0.0253300413005514	0.0165785521406113	ADRA2A/F2RL1/GNAZ/RGS2	4	1.81818181818182
GO:0016004	MF	GO:0016004	phospholipase activator activity	4/665	22/18369	0.00740304931993194	0.0253300413005514	0.0165785521406113	CCL5/F2/F2RL2/GNA11	4	1.81818181818182
GO:0000099	MF	GO:0000099	sulfur amino acid transmembrane transporter activity	3/665	12/18369	0.00813613824342093	0.0274349009367527	0.0179561861054904	SLC1A1/SLC1A2/SLC7A11	3	2.5
GO:0033130	MF	GO:0033130	acetylcholine receptor binding	3/665	12/18369	0.00813613824342093	0.0274349009367527	0.0179561861054904	LY6H/LYNX1/NRXN1	3	2.5
GO:0070679	MF	GO:0070679	inositol 1,4,5 trisphosphate binding	3/665	12/18369	0.00813613824342093	0.0274349009367527	0.0179561861054904	ITPR1/RPH3A/TRPC1	3	2.5
GO:0009931	MF	GO:0009931	calcium-dependent protein serine/threonine kinase activity	4/665	23/18369	0.00870939942824331	0.029226734619778	0.0191289440883482	CAMK2B/CAMK4/PRKCB/PRKCG	4	1.73913043478261
GO:0003774	MF	GO:0003774	cytoskeletal motor activity	10/665	115/18369	0.00891923474666557	0.0297876835080027	0.0194960860239378	DNAH10/DNAH12/DNAH14/DNAH2/DNAH5/GPR88/KIF14/KIF1A/KIF5A/KIF5C	10	0.869565217391304
GO:0042626	MF	GO:0042626	ATPase-coupled transmembrane transporter activity	9/665	99/18369	0.00969397461230273	0.0321469320099095	0.0210402179042387	ATP1A2/ATP2A1/ATP2A3/ATP6V0A4/ATP6V0D2/ATP6V1C2/ATP6V1E2/ATP6V1F/ATP6V1G2	9	0.909090909090909
GO:0015631	MF	GO:0015631	tubulin binding	23/665	374/18369	0.00971776884540243	0.0321469320099095	0.0210402179042387	APC/DCX/FGF13/GABARAP/KIF14/KIF1A/KIF5A/KIF5C/KLC3/LRRK2/MAP1B/MAP2/MAPT/NDN/NEFH/NEFM/PRKN/PRNP/RGS2/SLC6A2/SNCA/SYT11/UNC5C	23	0.614973262032086
GO:0008514	MF	GO:0008514	organic anion transmembrane transporter activity	16/665	230/18369	0.00977153781394203	0.0321723273308091	0.0210568391819803	SLC17A7/SLC17A8/SLC1A1/SLC1A2/SLC1A7/SLC22A2/SLC22A3/SLC38A3/SLC38A5/SLC44A4/SLC4A8/SLC6A1/SLC6A15/SLC6A6/SLC6A9/SLC7A11	16	0.695652173913043
GO:0019207	MF	GO:0019207	kinase regulator activity	15/665	211/18369	0.0100649808092787	0.0329828948585752	0.0215873569124138	APC/CCL5/CCNB1/CDK5R2/EFNA5/ERBB4/F2/F2RL2/HSP90AB1/MAP2K6/NRG3/PIK3R2/PIK3R5/SNCA/WNT11	15	0.710900473933649
GO:1902282	MF	GO:1902282	voltage-gated potassium channel activity involved in ventricular cardiac muscle cell action potential repolarization	3/665	13/18369	0.0102958211135363	0.0335816969030296	0.0219792737986953	KCNJ5/KCNQ1/SCN2B	3	2.30769230769231

	ONTOLOGY	ID	Description	GeneRatio	BgRatio	pvalue	p.adjust	qvalue	geneID	Count	richFactor
GO:0043177	MF	GO:0043177	organic acid binding	15/665	212/18369	0.010482601882355	0.0340318889017851	0.022273925174747	APOC1/FABP7/GCHFR/GLRB/GPR143/GRIN2B/GRIN2D/GRIN3A/GRIN3B/MAG/NOS1/P2RX1/RTN4R/SLC1A1/TH	15	0.707547169811321
GO:0001637	MF	GO:0001637	G protein-coupled chemoattractant receptor activity	4/665	25/18369	0.0117529975727894	0.0376311573660871	0.024629652083972	CCR2/CCR7/CX3CR1/CXCR5	4	1.6
GO:0004950	MF	GO:0004950	chemokine receptor activity	4/665	25/18369	0.0117529975727894	0.0376311573660871	0.024629652083972	CCR2/CCR7/CX3CR1/CXCR5	4	1.6
GO:0035255	MF	GO:0035255	ionotropic glutamate receptor binding	4/665	25/18369	0.0117529975727894	0.0376311573660871	0.024629652083972	CACNG4/DRD2/NSG1/SHISA7	4	1.6
GO:0089720	MF	GO:0089720	caspase binding	3/665	14/18369	0.0127561242749978	0.0406565056801301	0.0266097473460662	CTSG/MAGEA3/RIPK2	3	2.14285714285714
GO:0008574	MF	GO:0008574	plus-end-directed microtubule motor activity	4/665	26/18369	0.0135006500267325	0.0426400620753813	0.0279079881476632	KIF14/KIF1A/KIF5A/KIF5C	4	1.53846153846154
GO:0098918	MF	GO:0098918	structural constituent of synapse	4/665	26/18369	0.0135006500267325	0.0426400620753813	0.0279079881476632	HOMER1/NEFH/NEFL/SPTBN2	4	1.53846153846154
GO:0048487	MF	GO:0048487	beta-tubulin binding	5/665	40/18369	0.0141441546035573	0.044471260870644	0.0291065106588139	FGF13/GABARAP/RGS2/SLC6A2/SYT11	5	1.25
GO:0031406	MF	GO:0031406	carboxylic acid binding	14/665	200/18369	0.0143052722545295	0.0447761436487068	0.0293060569198292	APOC1/FABP7/GCHFR/GLRB/GPR143/GRIN2B/GRIN2D/GRIN3A/GRIN3B/MAG/NOS1/RTN4R/SLC1A1/TH	14	0.7
GO:0019887	MF	GO:0019887	protein kinase regulator activity	13/665	184/18369	0.0166456069063067	0.0518071437237194	0.0339078576023137	APC/CCL5/CCNB1/CDK5R2/EFNA5/ERBB4/F2/F2RL2/HSP90AB1/MAP2K6/NRG3/SNCA/WNT11	13	0.706521739130435
GO:1901682	MF	GO:1901682	sulfur compound transmembrane transporter activity	6/665	57/18369	0.0167000105126603	0.0518071437237194	0.0339078576023137	SLC1A1/SLC1A2/SLC22A2/SLC44A4/SLC6A6/SLC7A11	6	1.05263157894737
GO:0051393	MF	GO:0051393	alpha-actinin binding	4/665	28/18369	0.017470322820322	0.0537193186281267	0.0351593791051229	CACNA1C/CACNA1D/MYPN/PALLD	4	1.42857142857143
GO:0051721	MF	GO:0051721	protein phosphatase 2A binding	4/665	28/18369	0.017470322820322	0.0537193186281267	0.0351593791051229	BCL2/GRIN3A/MAPT/SLC6A3	4	1.42857142857143
GO:0022858	MF	GO:0022858	alanine transmembrane transporter activity	3/665	16/18369	0.0186003024620234	0.0562035113354646	0.0367852871657241	SLC38A3/SLC38A5/SLC6A6	3	1.875
GO:0042813	MF	GO:0042813	Wnt receptor activity	3/665	16/18369	0.0186003024620234	0.0562035113354646	0.0367852871657241	FZD10/FZD3/FZD9	3	1.875
GO:0045125	MF	GO:0045125	bioactive lipid receptor activity	3/665	16/18369	0.0186003024620234	0.0562035113354646	0.0367852871657241	LPAR1/S1PR1/S1PR5	3	1.875
GO:0086008	MF	GO:0086008	voltage-gated potassium channel activity involved in cardiac muscle cell action potential repolarization	3/665	16/18369	0.0186003024620234	0.0562035113354646	0.0367852871657241	KCNJ5/KCNQ1/SCN2B	3	1.875
GO:0047498	MF	GO:0047498	calcium-dependent phospholipase A2 activity	3/665	17/18369	0.0219904131473959	0.0661608119693205	0.0433023561976853	PLA2G4B/PLA2G4D/PLA2G4E	3	1.76470588235294
GO:0019001	MF	GO:0019001	guanyl nucleotide binding	23/665	404/18369	0.0222136977729892	0.0662613719895147	0.0433681728901363	GNA11/GNA13/GNAI1/GNAL/GNAO1/GNAQ/GNAZ/GUCY1A1/LRRK2/NMUR2/PRKG1/PRKG2/RAB39B/RAB3B/RAP1A/SEPTIN5/TUBA1A/TUBAL3/TUBB2A/TUBB2B/TUBB3/TUBB4A/TUBB6	23	0.569306930693069
GO:0032561	MF	GO:0032561	guanyl ribonucleotide binding	23/665	404/18369	0.0222136977729892	0.0662613719895147	0.0433681728901363	GNA11/GNA13/GNAI1/GNAL/GNAO1/GNAQ/GNAZ/GUCY1A1/LRRK2/NMUR2/PRKG1/PRKG2/RAB39B/RAB3B/RAP1A/SEPTIN5/TUBA1A/TUBAL3/TUBB2A/TUBB2B/TUBB3/TUBB4A/TUBB6	23	0.569306930693069
GO:0004712	MF	GO:0004712	protein serine/threonine/tyrosine kinase activity	5/665	45/18369	0.0226798499842546	0.067363979953233	0.0440898315483383	AURKA/MAP2K6/MAPK10/PRKCG/TTK	5	1.11111111111111

	ONTOLOGY	ID	Description	GeneRatio	BgRatio	pvalue	p.adjust	qvalue	geneID	Count	richFactor
GO:0048306	MF	GO:0048306	calcium-dependent protein binding	6/665	62/18369	0.0243479339266319	0.0720121096643606	0.0471320398044526	A2M/NOS1/NSMF/SNAP25/STX1A/VAMP2	6	0.967741935483871
GO:0030295	MF	GO:0030295	protein kinase activator activity	10/665	135/18369	0.0249874967162272	0.0735918679659348	0.0481659941143353	CCL5/CCNB1/CDK5R2/EFNA5/ERBB4/F2/F2RL2/MAP2K6/NRG3/WNT11	10	0.740740740740741
GO:0008970	MF	GO:0008970	phospholipase A1 activity	3/665	18/18369	0.0256939440483407	0.0750392173462	0.0491132865755554	PLA2G4B/PLA2G4D/PLA2G4E	3	1.66666666666667
GO:0045236	MF	GO:0045236	CXCR chemokine receptor binding	3/665	18/18369	0.0256939440483407	0.0750392173462	0.0491132865755554	CXCL12/CXCL13/CXCL8	3	1.66666666666667
GO:0005546	MF	GO:0005546	phosphatidylinositol-4,5-bisphosphate binding	7/665	82/18369	0.028939697449564	0.0841662867491486	0.0550869679522402	KCNQ1/PLA2G4E/RPH3A/SYT10/SYT5/SYT7/SYT9	7	0.853658536585366
GO:0032813	MF	GO:0032813	tumor necrosis factor receptor superfamily binding	5/665	48/18369	0.0291201047106394	0.084339556381852	0.0552003732012121	BDNF/BID/NGF/NTF4/TRAP1	5	1.04166666666667
GO:0005248	MF	GO:0005248	voltage-gated sodium channel activity	3/665	19/18369	0.0297101136233046	0.0853401617657063	0.0558552710093749	HCN1/SCN1B/SCN2B	3	1.57894736842105
GO:0033612	MF	GO:0033612	receptor serine/threonine kinase binding	3/665	19/18369	0.0297101136233046	0.0853401617657063	0.0558552710093749	INHBA/MAGI2/OPRD1	3	1.57894736842105
GO:0030551	MF	GO:0030551	cyclic nucleotide binding	4/665	33/18369	0.030320379080679	0.0867361663865325	0.0567689582442393	FKBP1B/HCN1/PRKG2	4	1.21212121212121
GO:0019209	MF	GO:0019209	kinase activator activity	10/665	141/18369	0.0324268928768336	0.092383556032775	0.060465183710186	CCL5/CCNB1/CDK5R2/EFNA5/ERBB4/F2/F2RL2/MAP2K6/NRG3/WNT11	10	0.709219858156028
GO:0030296	MF	GO:0030296	protein tyrosine kinase activator activity	4/665	34/18369	0.0334111368450839	0.0948007053571893	0.0620472117705024	CCL5/EFNA5/ERBB4/NRG3	4	1.17647058823529
GO:0015174	MF	GO:0015174	basic amino acid transmembrane transporter activity	3/665	20/18369	0.0340368395133425	0.0957972337915848	0.0626994412087887	SLC22A2/SLC38A3/SLC38A5	3	1.5
GO:0015662	MF	GO:0015662	P-type ion transporter activity	3/665	20/18369	0.0340368395133425	0.0957972337915848	0.0626994412087887	ATP1A2/ATP2A1/ATP2A3	3	1.5
GO:0008017	MF	GO:0008017	microtubule binding	16/665	268/18369	0.0351901278168476	0.0986454185387936	0.0645635826358564	APC/DCX/FGF13/GABARAP/KIF14/KIF1A/KIF5A/KIF5C/KLC3/LRRK2/MAP1B/MAP2/MAPT/NEFH/NEFM/PRNP	16	0.597014925373134
GO:0005525	MF	GO:0005525	GTP binding	21/665	382/18369	0.0385792978748545	0.107713399666594	0.0704985906428919	GNA11/GNA13/GNAI1/GNAL/GNAO1/GNAQ/GNAZ/GUCY1A1/LRRK2/NMUR2/RAB39B/RAB3B/RAP1A/SEPTIN5/TUBA1A/TUBAL3/TUBB2A/TUBB2B/TUBB3/TUBB4A/TUBB6	21	0.549738219895288
GO:0042805	MF	GO:0042805	actinin binding	4/665	36/18369	0.0401239342618653	0.111579705636582	0.0730290940224347	CACNA1C/CACNA1D/MYPN/PALLD	4	1.11111111111111
GO:1902936	MF	GO:1902936	phosphatidylinositol bisphosphate binding	8/665	107/18369	0.0404038638725507	0.11191228961524	0.0732467707631036	KCNQ1/MAPT/PLA2G4E/RPH3A/SYT10/SYT5/SYT7/SYT9	8	0.747663551401869
GO:0015399	MF	GO:0015399	primary active transmembrane transporter activity	11/665	170/18369	0.0453328726380483	0.125068557712876	0.0818575690655834	ATP1A2/ATP2A1/ATP2A3/ATP6V0A4/ATP6V0D2/ATP6V1C2/ATP6V1E2/ATP6V1F/ATP6V1G2/COX7A1/NDUFS7	11	0.647058823529412
GO:0043621	MF	GO:0043621	protein self-association	6/665	72/18369	0.0459299654443257	0.126216991654092	0.0826092208986214	CCL5/CNTN2/PTH1R/PTK2B/SYP/VAMP2	6	0.833333333333333
GO:0140828	MF	GO:0140828	metal cation:monoatomic cation antiporter activity	4/665	38/18369	0.047547100910291	0.129137046525056	0.0845204014354911	SLC17A7/SLC38A3/SLC38A5/SLC4A8	4	1.05263157894737
GO:0050840	MF	GO:0050840	extracellular matrix binding	5/665	55/18369	0.0482823402157843	0.129137046525056	0.0845204014354911	ADGRG1/ITGB3/SHH/SLIT2/VEGFA	5	0.909090909090909
GO:0008401	MF	GO:0008401	retinoic acid 4-hydroxylase activity	2/665	10/18369	0.0485672376055001	0.129137046525056	0.0845204014354911	CYP2C18/CYP2C8	2	2
GO:0015187	MF	GO:0015187	glycine transmembrane transporter activity	2/665	10/18369	0.0485672376055001	0.129137046525056	0.0845204014354911	SLC38A5/SLC6A9	2	2

	ONTOLOGY	ID	Description	GeneRatio	BgRatio	pvalue	p.adjust	qvalue	geneID	Count	richFactor
GO:0016303	MF	GO:0016303	1-phosphatidylinositol-3-kinase activity	2/665	10/18369	0.0485672376055001	0.129137046525056	0.0845204014354911	PIK3CD/PIK3CG	2	2
GO:0035374	MF	GO:0035374	chondroitin sulfate binding	2/665	10/18369	0.0485672376055001	0.129137046525056	0.0845204014354911	PTN/RTN4R	2	2
GO:0043208	MF	GO:0043208	glycosphingolipid binding	2/665	10/18369	0.0485672376055001	0.129137046525056	0.0845204014354911	MAG/RTN4R	2	2
GO:0052813	MF	GO:0052813	phosphatidylinositol bisphosphate kinase activity	2/665	10/18369	0.0485672376055001	0.129137046525056	0.0845204014354911	PIK3CD/PIK3CG	2	2
GO:0017134	MF	GO:0017134	fibroblast growth factor binding	3/665	23/18369	0.0488426651613391	0.129137046525056	0.0845204014354911	CXCL13/FGFR1/ITGB3	3	1.30434782608696
GO:0043274	MF	GO:0043274	phospholipase binding	3/665	23/18369	0.0488426651613391	0.129137046525056	0.0845204014354911	NEFL/PRKN/STXBP1	3	1.30434782608696

Supplementary Table 5: KEGG enrichment terms summary table.

	ID	Description	GeneRatio	BgRatio	pvalue	p.adjust	qvalue	geneID	Count	richFactor
1	hsa04080	Neuroactive ligand-receptor interaction	177/528	370/9434	1.446090287903e-129	3.94782648597518e-127	1.94841638791141e-127	ADCYAP1/ADCYAP1R1/ADORA3/ADRA1A/ADRA1B/ADRA1D/ADRA2A/ADRA2C/ADRB1/ADRB2/ADRB3/AGT/AGTR1/APELA/APLN/AVPR1B/BDKRB1/BDKRB2/CALCA/CALCB/CCKAR/CGA/CHRFAM7A/CHRM2/CHRM4/CHRM5/CHRNA1/CHRNA3/CHRNA4/CHRNA6/CHRNA7/CHRNB2/CHRNB4/CHRND/CHRNG/CNR1/CNR2/CTSG/CYSLTR1/CYSLTR2/DRD2/DRD4/DRD5/EDN2/EDN3/EDNRA/EDNRB/F2/F2RL1/F2RL2/GABRA3/GABRA4/GABRB1/GABRB3/GABRD/GABRE/GABRG2/GABRP/GABRR1/GAL/GALR1/GALR3/GCG/GCGR/GHR/GHRHR/GLP2R/GLRA3/GLRB/GPR156/GRIA1/GRIA2/GRIA3/GRIA4/GRID2/GRIK1/GRIK2/GRIK3/GRIK5/GRIN2A/GRIN2B/GRIN2D/GRIN3A/GRIN3B/GRM2/GRM4/GRM8/GRP/GRPR/GZMA/HCRT/HTR1D/HTR2B/HTR2C/HTR4/HTR7/INSL5/KISS1/KISS1R/KNG1/LEP/LEPR/LHB/LPAR1/LTB4R/LTB4R2/LYNX1/LYPD6B/MC1R/MC3R/MTNR1A/NMB/NMU/NMUR2/NPBWR1/NPFFR1/NPSR1/NPW/NPY/NPY1R/NPY2R/NPY4R/NPY4R2/NPY5R/NR3C1/NTSR1/OPRD1/OPRM1/OXT/OXTR/P2RX1/P2RX2/P2RX4/P2RX7/P2RY1/P2RY10/P2RY13/P2RY14/P2RY2/P2RY4/P2RY8/PENK/PNOC/PPY/PRL/PRSS1/PRSS2/PTGDR/PTGER3/PTGER4/PTGFR/PTH1R/PYY/RLN2/RXFP3/S1PR1/S1PR5/SCTR/SLURP1/SPX/SST/SSTR1/SSTR2/SSTR3/SSTR5/TACR1/TACR2/THRB/TSHR/UCN/UCN2/UCN3/UTS2B/VGF/VIP/VIPR1/VIPR2	177	0.478378378378378

	ID	Description	GeneRatio	BgRatio	pvalue	p.adjust	qvalue	geneID	Count	richFactor
4	hsa05022	Pathways of neurodegeneration - multiple diseases	110/528	483/9434	8.15903096620836e-40	5.56853863443721e-38	2.74830516756492e-38	ADRM1/APC/ATP2A1/ATP2A3/AXIN2/BCL2/BCL2L1/BDNF/BECN2/BID/CACNA1C/CACNA1D/CALML3/CALML5/CALML6/CAMK2A/CAMK2B/CAMK2D/CAPN2/CASP7/CHRFAM7A/CHRM5/CHRNA7/COX7A1/CSF1/CSNK1A1L/CSNK1E/DKK1/DKK4/DNAH10/DNAH12/DNAH14/DNAH2/DNAH5/FAS/FZD10/FZD3/FZD9/GNAQ/GPX2/GPX3/GRIA1/GRIA2/GRIA3/GRIA4/GRIN2A/GRIN2B/GRIN2D/IL1A/ITPR1/KIF5A/KIF5C/KLC3/LRRK2/MAP1LC3C/MAP2K6/MAPK10/MAPT/NDUFA11/NDUFA13/NDUFA4L2/NDUFS7/NEFH/NEFL/NEFM/NOS1/NOX4/PINK1/PLCB4/PLCG1/PRKCB/PRKCG/PRKN/PRNP/PRPH/PSMA2/PSMA6/PSMA7/RAB39B/RYR1/RYR3/SDHD/SEPTIN5/SIGMAR1/SLC6A3/SNCA/SNCAIP/SPTBN2/STX1A/TRAP1/TUBA1A/TUBAL3/TUBB2A/TUBB2B/TUBB3/TUBB4A/TUBB6/UCHL1/WNT1/WNT11/WNT16/WNT2/WNT2B/WNT3/WNT3A/WNT5A/WNT7A/WNT7B/WNT8B/WNT9A	110	0.227743271221532
2	hsa04082	Neuroactive ligand signaling	106/528	199/9434	1.7142861357163e-81	2.34000057525275e-79	1.15488750195624e-79	ADCY2/ADCY3/ADCY5/ADCY9/ADORA3/ADRA1A/ADRA1B/ADRA1D/ADRA2A/ADRA2C/ADRB1/ADRB2/CHAT/CHRFAM7A/CHRM2/CHRM4/CHRM5/CHRNA3/CHRNA4/CHRNA6/CHRNA7/CHRNB2/CHRNB4/DRD2/DRD4/DRD5/GABRA3/GABRA4/GABRB1/GABRB3/GABRD/GABRE/GABRG2/GABRP/GABRR1/GLRA3/GLRB/GNA11/GNAI1/GNAL/GNAO1/GNAQ/GNG10/GNG11/GNG12/GNG13/GNG2/GNG4/GNG7/GNG8/GNGT1/GRIA1/GRIA2/GRIA3/GRIA4/GRIK1/GRIK2/GRIK3/GRIK5/GRIN2A/GRIN2B/GRIN2D/GRIN3A/GRIN3B/GRM2/GRM4/GRM8/HTR1D/HTR2B/HTR2C/HTR3A/HTR3C/HTR3E/HTR4/HTR7/MC3R/OPRD1/OPRM1/P2RX1/P2RX2/P2RX4/P2RY1/P2RY13/P2RY14/P2RY2/P2RY4/PENK/PLCB4/PNOC/PRKACB/PRKCB/PRKCG/SLC17A7/SLC17A8/SLC18A2/SLC18A3/SLC1A1/SLC1A2/SLC1A7/SLC5A7/SLC6A1/SLC6A2/SLC6A3/SLC6A9/TACR1/TACR2	106	0.532663316582915

	ID	Description	GeneRatio	BgRatio	pvalue	p.adjust	qvalue	geneID	Count	richFactor
3	hsa04081	Hormone signaling	77/528	219/9434	3.89230946262432e-42	3.54200161098813e-40	1.74812495163478e-40	ADCY2/ADCY3/ADCY5/ADCY9/ADRA1A/ADRA1B/ADRA1D/ADRA2A/ADRA2C/ADRB1/ADRB2/ADRB3/AGT/AGTR1/AVPR1B/CALCA/CALCB/CCKAR/CGA/DRD2/DRD4/DRD5/GCG/GCGR/GHR/GHRHR/GNA11/GNA13/GNAI1/GNAO1/GNAQ/GNG10/GNG11/GNG12/GNG13/GNG2/GNG4/GNG7/GNG8/GNGT1/GPER1/HCRT/IGF1/ITGB3/LEP/LEPR/LHB/MC1R/MC3R/NOS1/OPRD1/OPRM1/OXT/OXTR/PENK/PIK3CD/PIK3R2/PLCB4/PNOC/PRKACB/PRKG1/PRKG2/PRL/PTH1R/SST/SSTR1/SSTR2/SSTR3/SSTR5/THRB/TSHR/UCN/UCN2/UCN3/VIP/VIPR1/VIPR2	77	0.351598173515982
6	hsa04020	Calcium signaling pathway	76/528	254/9434	5.6111861707956e-36	2.553089707712e-34	1.26005584186287e-34	ADCY2/ADCY3/ADCY9/ADRA1A/ADRA1B/ADRA1D/ADRB1/ADRB2/ADRB3/AGTR1/ATP2A1/ATP2A3/AVPR1B/BDKRB1/BDKRB2/CACNA1A/CACNA1C/CACNA1D/CALML3/CALML5/CALML6/CAMK2A/CAMK2B/CAMK2D/CAMK4/CCKAR/CHRFAM7A/CHRM2/CHRM5/CHRNA7/CYSLTR1/CYSLTR2/DRD5/EDNRA/EDNRB/ERBB4/FGFR1/GDNF/GNA11/GNAL/GNAQ/GRIN2A/GRIN2B/GRIN2D/GRIN3A/GRIN3B/GRPR/HTR2B/HTR2C/HTR4/HTR7/ITPR1/LTB4R2/NGF/NOS1/NTRK3/NTSR1/OXTR/P2RX1/P2RX2/P2RX4/P2RX7/PLCB4/PLCG1/PLCG2/PRKACB/PRKCB/PRKCG/PTGER3/PTGFR/PTK2B/RYR1/RYR3/TACR1/TACR2/VEGFA	76	0.299212598425197
15	hsa05010	Alzheimer disease	73/528	391/9434	1.04942512226332e-20	1.90995372251924e-19	9.42641513331261e-20	ADRM1/AKT3/APC/ATP2A1/ATP2A3/AXIN2/BECN2/BID/CACNA1C/CACNA1D/CALML3/CALML5/CALML6/CAPN2/CASP7/CHRFAM7A/CHRM5/CHRNA7/COX7A1/CSF1/CSNK1A1L/CSNK1E/DKK1/DKK4/FAS/FZD10/FZD3/FZD9/GNAQ/GRIN2A/GRIN2B/GRIN2D/IL1A/ITPR1/KIF5A/KIF5C/KLC3/MAPK10/MAPT/NDUFA11/NDUFA13/NDUFA4L2/NDUFS7/NOS1/NOX4/PIK3CD/PIK3R2/PLCB4/PSMA2/PSMA6/PSMA7/RYR3/SDHD/SNCA/TUBA1A/TUBAL3/TUBB2A/TUBB2B/TUBB3/TUBB4A/TUBB6/WNT1/WNT11/WNT16/WNT2/WNT2B/WNT3/WNT3A/WNT5A/WNT7A/WNT7B/WNT8B/WNT9A	73	0.186700767263427

	ID	Description	GeneRatio	BgRatio	pvalue	p.adjust	qvalue	geneID	Count	richFactor
11	hsa04024	cAMP signaling pathway	63/528	226/9434	6.59500434980493e-28	1.63676017045159e-26	8.07809145239265e-27	ADCY2/ADCY3/ADCY5/ADCY9/ADCYAP1/ADCYAP1R1/ADRB1/ADRB2/AKT3/ATP1A2/ATP2A1/ATP2A3/BDNF/CACNA1C/CACNA1D/CALML3/CALML5/CALML6/CAMK2A/CAMK2B/CAMK2D/CAMK4/CGA/CHRM2/CREB3L3/DRD2/DRD5/EDN2/EDN3/EDNRA/GCG/GNAI1/GRIA1/GRIA2/GRIA3/GRIA4/GRIN2A/GRIN2B/GRIN2D/GRIN3A/GRIN3B/HTR1D/HTR4/LHB/MAPK10/NPY/NPY1R/OXT/OXTR/PIK3CD/PIK3R2/PLD1/PRKACB/PTGER3/RAP1A/SOX9/SST/SSTR1/SSTR2/SSTR5/TSHR/VIP/VIPR2	63	0.278761061946903
7	hsa04728	Dopaminergic synapse	56/528	132/9434	7.65143205909854e-36	2.98405850304843e-34	1.47275684746558e-34	ADCY5/AKT3/ARRB1/CACNA1A/CACNA1C/CACNA1D/CALML3/CALML5/CALML6/CALY/CAMK2A/CAMK2B/CAMK2D/COMT/CREB3L3/DRD2/DRD4/DRD5/GNAI1/GNAL/GNAO1/GNAQ/GNG10/GNG11/GNG12/GNG13/GNG2/GNG4/GNG7/GNG8/GNGT1/GRIA1/GRIA2/GRIA3/GRIA4/GRIN2A/GRIN2B/ITPR1/KCNJ5/KCNJ9/KIF5A/KIF5C/MAOA/MAOB/MAPK10/PLCB4/PPP2R2B/PPP2R2C/PPP2R3A/PPP2R3B/PRKACB/PRKCB/PRKCG/SLC18A2/SLC6A3/TH	56	0.424242424242424
5	hsa04724	Glutamatergic synapse	53/528	116/9434	5.43518745168754e-36	2.553089707712e-34	1.26005584186287e-34	ADCY2/ADCY3/ADCY5/ADCY9/CACNA1A/CACNA1C/CACNA1D/GLS2/GNAI1/GNAO1/GNAQ/GNG10/GNG11/GNG12/GNG13/GNG2/GNG4/GNG7/GNG8/GNGT1/GRIA1/GRIA2/GRIA3/GRIA4/GRIK1/GRIK2/GRIK3/GRIK5/GRIN2A/GRIN2B/GRIN2D/GRIN3A/GRIN3B/GRM2/GRM4/GRM8/HOMER1/ITPR1/PLA2G4B/PLA2G4D/PLA2G4E/PLCB4/PLD1/PRKACB/PRKCB/PRKCG/SLC17A7/SLC17A8/SLC1A1/SLC1A2/SLC1A7/SLC38A3/TRPC1	53	0.456896551724138
8	hsa04725	Cholinergic synapse	52/528	116/9434	8.38267511515918e-35	2.86058788304807e-33	1.41181896676365e-33	ADCY2/ADCY3/ADCY5/ADCY9/AKT3/BCL2/CACNA1A/CACNA1C/CACNA1D/CAMK2A/CAMK2B/CAMK2D/CAMK4/CHAT/CHRFAM7A/CHRM2/CHRM4/CHRM5/CHRNA3/CHRNA4/CHRNA6/CHRNA7/CHRNB2/CHRNB4/CREB3L3/GNA11/GNAI1/GNAO1/GNAQ/GNG10/GNG11/GNG12/GNG13/GNG2/GNG4/GNG7/GNG8/GNGT1/ITPR1/KCNJ14/KCNQ1/KCNQ5/PIK3CD/PIK3CG/PIK3R2/PIK3R5/PLCB4/PRKACB/PRKCB/PRKCG/SLC18A3/SLC5A7	52	0.448275862068966

	ID	Description	GeneRatio	BgRatio	pvalue	p.adjust	qvalue	geneID	Count	richFactor
27	hsa05012	Parkinson disease	51/528	271/9434	7.85607925900115e-15	7.94336902854561e-14	3.92038263217211e-14	ADCY5/ADRM1/BCL2L1/CALML3/CALML5/CALML6/CAMK2A/CAMK2B/CAMK2D/COX7A1/DRD2/DUSP1/GNAI1/GNAL/ITPR1/KIF5A/KIF5C/KLC3/LRRK2/MAOA/MAOB/MAPK10/MAPT/NDUFA11/NDUFA13/NDUFA4L2/NDUFS7/PINK1/PLCG1/PRKACB/PRKN/PSMA2/PSMA6/PSMA7/RYR3/SDHD/SEPTIN5/SLC18A2/SLC6A3/SNCA/SNCAIP/TH/TRAP1/TUBA1A/TUBAL3/TUBB2A/TUBB2B/TUBB3/TUBB4A/TUBB6/UCHL1	51	0.188191881918819
52	hsa05014	Amyotrophic lateral sclerosis	50/528	371/9434	4.58539386610041e-09	2.40733177970272e-08	1.18811824870618e-08	ADRM1/BCL2/BCL2L1/BECN2/BID/COX7A1/DNAH10/DNAH12/DNAH14/DNAH2/DNAH5/ERBB4/GPX2/GPX3/GRIA1/GRIA2/GRIN2A/GRIN2B/GRIN2D/KIF5A/KIF5C/KLC3/MAP1LC3C/MAP2K6/NDUFA11/NDUFA13/NDUFA4L2/NDUFS7/NEFH/NEFL/NEFM/NOS1/NRG3/PINK1/PRKN/PRPH/PSMA2/PSMA6/PSMA7/RAB39B/SDHD/SIGMAR1/SLC1A2/TUBA1A/TUBAL3/TUBB2A/TUBB2B/TUBB3/TUBB4A/TUBB6	50	0.134770889487871
12	hsa04723	Retrograde endocannabinoid signaling	49/528	149/9434	1.96690069968502e-25	4.47469909178341e-24	2.20844990841827e-24	ADCY2/ADCY3/ADCY5/ADCY9/CACNA1A/CACNA1C/CACNA1D/CNR1/GABRA3/GABRA4/GABRB1/GABRB3/GABRD/GABRE/GABRG2/GABRP/GABRR1/GNAI1/GNAO1/GNAQ/GNG10/GNG11/GNG12/GNG13/GNG2/GNG4/GNG7/GNG8/GNGT1/GRIA1/GRIA2/GRIA3/GRIA4/ITPR1/KCNJ5/KCNJ9/MAPK10/MGLL/NDUFA11/NDUFA13/NDUFA4L2/NDUFS7/PLCB4/PRKACB/PRKCB/PRKCG/RIMS1/SLC17A7/SLC17A8	49	0.328859060402685
9	hsa04713	Circadian entrainment	48/528	97/9434	1.01230360849688e-34	3.07065427910719e-33	1.51549546067368e-33	ADCY2/ADCY3/ADCY5/ADCY9/ADCYAP1/ADCYAP1R1/CACNA1C/CACNA1D/CALML3/CALML5/CALML6/CAMK2A/CAMK2B/CAMK2D/GNAI1/GNAO1/GNAQ/GNG10/GNG11/GNG12/GNG13/GNG2/GNG4/GNG7/GNG8/GNGT1/GRIA1/GRIA2/GRIA3/GRIA4/GRIN2A/GRIN2B/GRIN2D/GUCY1A1/ITPR1/KCNJ5/KCNJ9/MTNR1A/NOS1/PLCB4/PRKACB/PRKCB/PRKCG/PRKG1/PRKG2/RPS6KA5/RYR1/RYR3	48	0.494845360824742
61	hsa04151	PI3K-Akt signaling pathway	47/528	362/9434	4.46461598411353e-08	1.99809862895573e-07	9.86144686741211e-08	AKT3/BCL2/BCL2L1/BDNF/CCND1/CHRM2/COL4A6/CREB3L3/CSF1/DDIT4/EFNA5/ERBB4/FGFR1/GDNF/GHR/GNG10/GNG11/GNG12/GNG13/GNG2/GNG4/GNG7/GNG8/GNGT1/HSP90AB1/IGF1/IL6R/ITGA4/ITGB3/LPAR1/MAGI2/NGF/NGFR/NTF4/PIK3CD/PIK3CG/PIK3R2/PIK3R5/PPP2R2B/PPP2R2C/PPP2R3A/PPP2R3B/PRL/RELN/SOS2/TNR/VEGFA	47	0.129834254143646

	ID	Description	GeneRatio	BgRatio	pvalue	p.adjust	qvalue	geneID	Count	richFactor
10	hsa04726	Serotonergic synapse	46/528	115/9434	3.30398085901982e-28	9.01986774512412e-27	4.45167947320566e-27	ADCY5/CACNA1A/CACNA1C/CACNA1D/CYP2C18/CYP2C8/CYP4X1/DUSP1/GABRB1/GABRB3/GNAI1/GNAO1/GNAQ/GNG10/GNG11/GNG12/GNG13/GNG2/GNG4/GNG7/GNG8/GNGT1/HTR1D/HTR2B/HTR2C/HTR3A/HTR3C/HTR3E/HTR4/HTR7/ITPR1/KCNJ5/KCNJ9/MAOA/MAOB/PLA2G4B/PLA2G4D/PLA2G4E/PLCB4/PRKACB/PRKCB/PRKCG/PTGS1/SLC18A2/TPH1/TRPC1	46	0.4
33	hsa05163	Human cytomegalovirus infection	44/528	226/9434	1.76152576164616e-13	1.45726222099819e-12	7.19219449731128e-13	ADCY2/ADCY3/ADCY5/ADCY9/AKT3/BID/CALML3/CALML5/CALML6/CCL5/CCND1/CREB3L3/CXCL12/CXCL8/FAS/GNA11/GNA13/GNAI1/GNAO1/GNAQ/GNG10/GNG11/GNG12/GNG13/GNG2/GNG4/GNG7/GNG8/GNGT1/IL6R/ITGB3/ITPR1/MAP2K6/PIK3CD/PIK3R2/PLCB4/PRKACB/PRKCB/PRKCG/PTGER3/PTGER4/PTK2B/SOS2/VEGFA	44	0.194690265486726
36	hsa04014	Ras signaling pathway	44/528	238/9434	1.16503053695167e-12	8.83481490521686e-12	4.3603482084741e-12	AKT3/BCL2L1/BDNF/CALML3/CALML5/CALML6/CSF1/EFNA5/FGFR1/GAB1/GNG10/GNG11/GNG12/GNG13/GNG2/GNG4/GNG7/GNG8/GNGT1/GRIN2A/GRIN2B/HTR7/IGF1/MAPK10/NGF/NGFR/NTF4/PAK6/PIK3CD/PIK3R2/PLA2G4B/PLA2G4D/PLA2G4E/PLCG1/PLCG2/PLD1/PRKACB/PRKCB/PRKCG/RAP1A/RASAL1/SHC4/SOS2/VEGFA	44	0.184873949579832
63	hsa05165	Human papillomavirus infection	44/528	333/9434	7.34500363063272e-08	3.18283490660751e-07	1.57086126102086e-07	AKT3/APC/ATP6V0A4/ATP6V0D2/ATP6V1C2/ATP6V1E2/ATP6V1F/ATP6V1G2/AXIN2/CCND1/COL4A6/CREB3L3/CSNK1A1L/DLG2/FAS/FZD10/FZD3/FZD9/ITGA4/ITGB3/PIK3CD/PIK3R2/PPP2R2B/PPP2R2C/PPP2R3A/PPP2R3B/PRKACB/PTGER4/RELN/SOS2/TNR/VEGFA/WNT1/WNT11/WNT16/WNT2/WNT2B/WNT3/WNT3A/WNT5A/WNT7A/WNT7B/WNT8B/WNT9A	44	0.132132132132132
19	hsa04261	Adrenergic signaling in cardiomyocytes	43/528	154/9434	2.482771048013e-19	3.56734997951342e-18	1.76063542462972e-18	ADCY2/ADCY3/ADCY5/ADCY9/ADRA1A/ADRA1B/ADRA1D/ADRB1/ADRB2/AGT/AGTR1/AKT3/ATP1A2/ATP2A1/ATP2A3/BCL2/CACNA1C/CACNA1D/CACNG4/CACNG5/CACNG7/CACNG8/CALML3/CALML5/CALML6/CAMK2A/CAMK2B/CAMK2D/CREB3L3/GNAI1/GNAQ/KCNQ1/PIK3CG/PIK3R5/PLCB4/PPP1R1A/PPP2R2B/PPP2R2C/PPP2R3A/PPP2R3B/PRKACB/RPS6KA5/SCN1B	43	0.279220779220779

	ID	Description	GeneRatio	BgRatio	pvalue	p.adjust	qvalue	geneID	Count	richFactor
20	hsa04022	cGMP-PKG signaling pathway	43/528	166/9434	5.61027741681375e-18	7.51173229825094e-17	3.70735197291737e-17	ADCY2/ADCY3/ADCY5/ADCY9/ADORA3/ADRA1A/ADRA1B/ADRA1D/ADRA2A/ADRA2C/ADRB1/ADRB2/ADRB3/AGTR1/AKT3/ATP1A2/ATP2A1/ATP2A3/BDKRB2/CACNA1C/CACNA1D/CALML3/CALML5/CALML6/CREB3L3/EDNRA/EDNRB/GNA11/GNA13/GNAI1/GNAQ/GUCY1A1/ITPR1/KCNMB2/KNG1/MEF2C/OPRD1/PIK3CG/PIK3R5/PLCB4/PRKG1/PRKG2/RGS2	43	0.259036144578313
22	hsa04921	Oxytocin signaling pathway	41/528	155/9434	1.51095365550385e-17	1.87495612705705e-16	9.25368745954509e-17	ADCY2/ADCY3/ADCY5/ADCY9/CACNA1C/CACNA1D/CACNG4/CACNG5/CACNG7/CACNG8/CALML3/CALML5/CALML6/CAMK2A/CAMK2B/CAMK2D/CAMK4/CCND1/GNAI1/GNAO1/GNAQ/GUCY1A1/ITPR1/KCNJ14/KCNJ5/KCNJ9/MEF2C/OXT/OXTR/PIK3CG/PIK3R5/PLA2G4B/PLA2G4D/PLA2G4E/PLCB4/PRKACB/PRKCB/PRKCG/RGS2/RYR1/RYR3	41	0.264516129032258
34	hsa05205	Proteoglycans in cancer	41/528	204/9434	4.06592846805763e-13	3.26026026414275e-12	1.60907389169182e-12	AKT3/ANK3/CAMK2A/CAMK2B/CAMK2D/CAV1/CCND1/ERBB4/FAS/FGFR1/FZD10/FZD3/FZD9/GAB1/IGF1/ITGB3/ITPR1/MMP9/PIK3CD/PIK3R2/PLCG2/PRKACB/PRKCB/PRKCG/SHH/SOS2/TWIST1/VEGFA/WNT1/WNT11/WNT16/WNT2/WNT2B/WNT3/WNT3A/WNT5A/WNT7A/WNT7B/WNT8B/WNT9A	41	0.200980392156863
56	hsa05020	Prion disease	41/528	278/9434	9.02092667154507e-09	4.39770175237822e-08	2.17044852247701e-08	ADRM1/CACNA1C/CACNA1D/CAV1/CCL5/COX7A1/CREB3L3/GRIN2A/GRIN2B/GRIN2D/GRIN3A/GRIN3B/IL1A/ITPR1/KIF5A/KIF5C/KLC3/MAPK10/NCAM1/NCAM2/NDUFA11/NDUFA13/NDUFA4L2/NDUFS7/PIK3CD/PIK3R2/PRKACB/PRNP/PSMA2/PSMA6/PSMA7/RYR1/RYR3/SDHD/TUBA1A/TUBAL3/TUBB2A/TUBB2B/TUBB3/TUBB4A/TUBB6	41	0.147482014388489
68	hsa05016	Huntington disease	41/528	311/9434	2.22085776556408e-07	8.91609073527931e-07	4.40046120730963e-07	ADRM1/BDNF/BECN2/COX7A1/CREB3L3/DNAH10/DNAH12/DNAH14/DNAH2/DNAH5/GNAQ/GPX2/GPX3/GRIA1/GRIA2/GRIA3/GRIA4/GRIN2B/ITPR1/KIF5A/KIF5C/KLC3/MAPK10/NDUFA11/NDUFA13/NDUFA4L2/NDUFS7/PLCB4/PSMA2/PSMA6/PSMA7/SDHD/SLC1A2/STX1A/TUBA1A/TUBAL3/TUBB2A/TUBB2B/TUBB3/TUBB4A/TUBB6	41	0.131832797427653
71	hsa04010	MAPK signaling pathway	39/528	300/9434	6.37398617027287e-07	2.45084256969647e-06	1.20959263127491e-06	AKT3/ARRB1/BDNF/CACNA1A/CACNA1C/CACNA1D/CACNG4/CACNG5/CACNG7/CACNG8/CSF1/DUSP1/EFNA5/ERBB4/FAS/FGFR1/GDNF/GNG12/IGF1/IL1A/IRAK1/MAP2K6/MAPK10/MAPT/MEF2C/NGF/NGFR/NTF4/PLA2G4B/PLA2G4D/PLA2G4E/PRKACB/PRKCB/PRKCG/RAP1A/RPS6KA5/RPS6KA6/SOS2/VEGFA	39	0.13

	ID	Description	GeneRatio	BgRatio	pvalue	p.adjust	qvalue	geneID	Count	richFactor
13	hsa04727	GABAergic synapse	38/528	89/9434	1.03065205865707e-24	2.16436932317984e-23	1.06820618225186e-23	ADCY2/ADCY3/ADCY5/ADCY9/CACNA1A/CACNA1C/CACNA1D/GABARAP/GABRA3/GABRA4/GABRB1/GABRB3/GABRD/GABRE/GABRG2/GABRP/GABRR1/GAD1/GLS2/GNAI1/GNAO1/GNG10/GNG11/GNG12/GNG13/GNG2/GNG4/GNG7/GNG8/GNGT1/PLCL1/PRKACB/PRKCB/PRKCG/SLC38A3/SLC38A5/SLC6A1/TRAK2	38	0.426966292134831
37	hsa04360	Axon guidance	38/528	184/9434	1.2556684653623e-12	9.26479705524072e-12	4.57256226362372e-12	CAMK2A/CAMK2B/CAMK2D/CXCL12/EFNA5/EPHA3/EPHA4/EPHB2/EPHB3/FZD3/GNAI1/L1CAM/LIMK1/NEO1/NTN1/NTNG1/PAK6/PIK3CD/PIK3R2/PLCG1/PLCG2/PLXNA1/PLXNA2/PLXNA3/ROBO2/SEMA3D/SEMA3E/SEMA3F/SEMA3G/SEMA6A/SEMA6D/SHH/SLIT2/SLIT3/TRPC1/UNC5C/UNC5D/WNT5A	38	0.206521739130435
43	hsa04062	Chemokine signaling pathway	38/528	193/9434	5.89845210668696e-12	3.74483122122219e-11	1.84822979107939e-11	ADCY2/ADCY3/ADCY5/ADCY9/AKT3/ARRB1/CCL5/CCR2/CCR7/CX3CR1/CXCL12/CXCL13/CXCL8/CXCR5/GNAI1/GNAQ/GNG10/GNG11/GNG12/GNG13/GNG2/GNG4/GNG7/GNG8/GNGT1/PIK3CD/PIK3CG/PIK3R2/PIK3R5/PLCB4/PLCG1/PLCG2/PRKACB/PRKCB/PTK2B/RAP1A/SHC4/SOS2	38	0.196891191709845
28	hsa04934	Cushing syndrome	37/528	155/9434	2.13080810452428e-14	2.07753790191117e-13	1.02535126834251e-13	ADCY2/ADCY3/ADCY5/ADCY9/AGT/AGTR1/APC/AXIN2/CACNA1C/CACNA1D/CAMK2A/CAMK2B/CAMK2D/CCND1/CREB3L3/FZD10/FZD3/FZD9/GNA11/GNAI1/GNAQ/ITPR1/PLCB4/PRKACB/RAP1A/WNT1/WNT11/WNT16/WNT2/WNT2B/WNT3/WNT3A/WNT5A/WNT7A/WNT7B/WNT8B/WNT9A	37	0.238709677419355
16	hsa04750	Inflammatory mediator regulation of TRP channels	36/528	99/9434	1.24053409177031e-20	2.1166612940831e-19	1.04466028780658e-19	ADCY2/ADCY3/ADCY5/ADCY9/ASIC1/BDKRB1/BDKRB2/CALML3/CALML5/CALML6/CAMK2A/CAMK2B/CAMK2D/F2RL1/GNAQ/HTR2B/HTR2C/IGF1/ITPR1/KNG1/MAP2K6/MAPK10/NGF/P2RY2/PIK3CD/PIK3R2/PLA2G4B/PLA2G4D/PLA2G4E/PLCB4/PLCG1/PLCG2/PRKACB/PRKCB/PRKCG/PTGER4	36	0.363636363636364
17	hsa04916	Melanogenesis	36/528	101/9434	2.71174025490692e-20	4.35473582111523e-19	2.14924305032871e-19	ADCY2/ADCY3/ADCY5/ADCY9/ASIP/CALML3/CALML5/CALML6/CAMK2A/CAMK2B/CAMK2D/CREB3L3/EDNRB/FZD10/FZD3/FZD9/GNAI1/GNAO1/GNAQ/MC1R/PLCB4/PRKACB/PRKCB/PRKCG/WNT1/WNT11/WNT16/WNT2/WNT2B/WNT3/WNT3A/WNT5A/WNT7A/WNT7B/WNT8B/WNT9A	36	0.356435643564356

	ID	Description	GeneRatio	BgRatio	pvalue	p.adjust	qvalue	geneID	Count	richFactor
23	hsa04926	Relaxin signaling pathway	36/528	130/9434	3.07582480727358e-16	3.65087031472038e-15	1.80185618000466e-15	ADCY2/ADCY3/ADCY5/ADCY9/AKT3/ARRB1/COL3A1/COL4A6/CREB3L3/EDNRB/GNAI1/GNAO1/GNG10/GNG11/GNG12/GNG13/GNG2/GNG4/GNG7/GNG8/GNGT1/INSL5/MAPK10/MMP1/MMP13/MMP9/NOS1/PIK3CD/PIK3R2/PLCB4/PRKACB/RLN2/RXFP3/SHC4/SOS2/VEGFA	36	0.276923076923077
25	hsa04371	Apelin signaling pathway	36/528	140/9434	3.99556903763037e-15	4.36316138909237e-14	2.15340141817553e-14	ADCY2/ADCY3/ADCY5/ADCY9/AGTR1/AKT3/APELA/APLN/BECN2/CALML3/CALML5/CALML6/CAMK4/CCND1/GNA13/GNAI1/GNAQ/GNG10/GNG11/GNG12/GNG13/GNG2/GNG4/GNG7/GNG8/GNGT1/ITPR1/MAP1LC3C/MEF2C/NOS1/PIK3CG/PIK3R5/PLCB4/PRKACB/RYR1/RYR3	36	0.257142857142857
51	hsa05170	Human immunodeficiency virus 1 infection	36/528	213/9434	1.87945058674857e-09	1.00605884349482e-08	4.9653183715958e-09	AKT3/BCL2/BCL2L1/BID/CALML3/CALML5/CALML6/CCNB1/CDK1/FAS/GNA11/GNAI1/GNAO1/GNAQ/GNG10/GNG11/GNG12/GNG13/GNG2/GNG4/GNG7/GNG8/GNGT1/IRAK1/ITPR1/LIMK1/MAP2K6/MAPK10/PAK6/PIK3CD/PIK3R2/PLCG1/PLCG2/PRKCB/PRKCG/PTK2B	36	0.169014084507042
49	hsa05034	Alcoholism	35/528	191/9434	3.33152376059642e-10	1.85613466661801e-09	9.1607957326819e-10	ADCY5/BDNF/CALML3/CALML5/CALML6/CAMK4/CREB3L3/DRD2/FOSB/GNAI1/GNAO1/GNG10/GNG11/GNG12/GNG13/GNG2/GNG4/GNG7/GNG8/GNGT1/GRIN2A/GRIN2B/GRIN2D/GRIN3A/GRIN3B/MAOA/MAOB/NPY/PRKACB/SHC4/SLC18A2/SLC29A1/SLC6A3/SOS2/TH	35	0.183246073298429
29	hsa04270	Vascular smooth muscle contraction	34/528	134/9434	3.64343554733405e-14	3.42985484283516e-13	1.69277586228225e-13	ADCY2/ADCY3/ADCY5/ADCY9/ADRA1A/ADRA1B/ADRA1D/AGT/AGTR1/AVPR1B/CACNA1C/CACNA1D/CALCA/CALCB/CALML3/CALML5/CALML6/EDN2/EDN3/EDNRA/GNA11/GNA13/GNAQ/GUCY1A1/ITPR1/KCNMB2/PLA2G4B/PLA2G4D/PLA2G4E/PLCB4/PRKACB/PRKCB/PRKCG/PRKG1	34	0.253731343283582
58	hsa04015	Rap1 signaling pathway	34/528	212/9434	2.11509694158834e-08	9.95554250092442e-08	4.9134738388985e-08	ADCY2/ADCY3/ADCY5/ADCY9/AKT3/CALML3/CALML5/CALML6/CNR1/CSF1/DRD2/EFNA5/FGFR1/GNAI1/GNAO1/GNAQ/GRIN2A/GRIN2B/IGF1/ITGB3/LPAR1/MAGI2/MAP2K6/NGF/NGFR/P2RY1/PIK3CD/PIK3R2/PLCB4/PLCG1/PRKCB/PRKCG/RAP1A/VEGFA	34	0.160377358490566
21	hsa05032	Morphine addiction	32/528	91/9434	5.77825561403919e-18	7.51173229825094e-17	3.70735197291737e-17	ADCY2/ADCY3/ADCY5/ADCY9/ARRB1/CACNA1A/GABRA3/GABRA4/GABRB1/GABRB3/GABRD/GABRE/GABRG2/GABRP/GABRR1/GNAI1/GNAO1/GNG10/GNG11/GNG12/GNG13/GNG2/GNG4/GNG7/GNG8/GNGT1/KCNJ5/KCNJ9/OPRM1/PRKACB/PRKCB/PRKCG	32	0.351648351648352

	ID	Description	GeneRatio	BgRatio	pvalue	p.adjust	qvalue	geneID	Count	richFactor
31	hsa04935	Growth hormone synthesis, secretion and action	32/528	122/9434	7.69071802262086e-14	6.77279361346934e-13	3.34265503190313e-13	ADCY2/ADCY3/ADCY5/ADCY9/AKT3/CACNA1C/CACNA1D/CREB3L3/GHR/GHRHR/GNA11/GNAI1/GNAQ/IGF1/ITPR1/MAP2K6/MAPK10/PIK3CD/PIK3R2/PLCB4/PLCG1/PLCG2/PRKACB/PRKCB/PRKCG/SHC4/SOS2/SST/SSTR1/SSTR2/SSTR3/SSTR5	32	0.262295081967213
69	hsa05207	Chemical carcinogenesis - receptor activation	32/528	217/9434	4.04764965797587e-07	1.60146138641654e-06	7.90387728788576e-07	ADCY2/ADCY3/ADCY5/ADCY9/ADRB1/ADRB2/ADRB3/AKT3/ARRB1/BCL2/CACNA1A/CACNA1C/CACNA1D/CCND1/CHRFAM7A/CHRNA3/CHRNA4/CHRNA7/CHRNB2/CHRNB4/CREB3L3/GNAI1/HSP90AB1/PIK3CD/PIK3R2/PRKACB/PRKCB/PRKCG/RPS6KA6/SOS2/VEGFA/WNT1	32	0.147465437788018
48	hsa04072	Phospholipase D signaling pathway	31/528	149/9434	1.29456117149165e-10	7.36281666285878e-10	3.63385591997657e-10	ADCY2/ADCY3/ADCY5/ADCY9/AGT/AGTR1/AKT3/AVPR1B/CXCL8/F2/GAB1/GNA13/GRM2/GRM4/GRM8/LPAR1/PIK3CD/PIK3CG/PIK3R2/PIK3R5/PLA2G4B/PLA2G4D/PLA2G4E/PLCB4/PLCG1/PLCG2/PLD1/PTGFR/PTK2B/SHC4/SOS2	31	0.208053691275168
55	hsa04310	Wnt signaling pathway	31/528	174/9434	7.08799105612394e-09	3.51822101513061e-08	1.73638823958634e-08	APC/AXIN2/CAMK2A/CAMK2B/CAMK2D/CCND1/CSNK1A1L/CSNK1E/DKK1/DKK4/FZD10/FZD3/FZD9/MAPK10/MMP7/PLCB4/PRKACB/PRKCB/PRKCG/WNT1/WNT11/WNT16/WNT2/WNT2B/WNT3/WNT3A/WNT5A/WNT7A/WNT7B/WNT8B/WNT9A	31	0.17816091954023
18	hsa05031	Amphetamine addiction	30/528	69/9434	5.03250534300074e-20	7.63263310355113e-19	3.7670215432988e-19	ADCY5/CACNA1C/CACNA1D/CALML3/CALML5/CALML6/CAMK2A/CAMK2B/CAMK2D/CAMK4/CREB3L3/FOSB/GRIA1/GRIA2/GRIA3/GRIA4/GRIN2A/GRIN2B/GRIN2D/GRIN3A/GRIN3B/MAOA/MAOB/PRKACB/PRKCB/PRKCG/SLC18A2/SLC6A3/STX1A/TH	30	0.434782608695652
38	hsa04722	Neurotrophin signaling pathway	30/528	120/9434	1.8335643305809e-12	1.31727121644365e-11	6.50128072892952e-12	AKT3/BCL2/BDNF/CALML3/CALML5/CALML6/CAMK2A/CAMK2B/CAMK2D/CAMK4/GAB1/IRAK1/IRAK2/MAPK10/MATK/NGF/NGFR/NTF4/NTRK3/PIK3CD/PIK3R2/PLCG1/PLCG2/RAP1A/RIPK2/RPS6KA5/RPS6KA6/SHC4/SOS2/TP73	30	0.25
57	hsa05225	Hepatocellular carcinoma	30/528	170/9434	1.56581921800532e-08	7.49944993886757e-08	3.70129011827665e-08	AKT3/APC/AXIN2/BCL2L1/CCND1/CSNK1A1L/FZD10/FZD3/FZD9/GAB1/PIK3CD/PIK3R2/PLCG1/PLCG2/PRKCB/PRKCG/SHC4/SOS2/WNT1/WNT11/WNT16/WNT2/WNT2B/WNT3/WNT3A/WNT5A/WNT7A/WNT7B/WNT8B/WNT9A	30	0.176470588235294
70	hsa05167	Kaposi sarcoma-associated herpesvirus infection	30/528	196/9434	4.14932376841624e-07	1.61823626968233e-06	7.9866683061245e-07	AKT3/BECN2/BID/CALML3/CALML5/CALML6/CCND1/CXCL8/FAS/GNG10/GNG11/GNG12/GNG13/GNG2/GNG4/GNG7/GNG8/GNGT1/IL6ST/ITPR1/MAP1LC3C/MAP2K6/MAPK10/PIK3CD/PIK3CG/PIK3R2/PIK3R5/PLCG1/PLCG2/VEGFA	30	0.153061224489796

	ID	Description	GeneRatio	BgRatio	pvalue	p.adjust	qvalue	geneID	Count	richFactor
26	hsa04540	Gap junction	29/528	92/9434	5.9910010895417e-15	6.29055114401879e-14	3.10464833790015e-14	ADCY2/ADCY3/ADCY5/ADCY9/ADRB1/CDK1/DRD2/GNA11/GNAI1/GNAQ/GUCY1A1/HTR2B/HTR2C/ITPR1/LPAR1/PLCB4/PRKACB/PRKCB/PRKCG/PRKG1/PRKG2/SOS2/TUBA1A/TUBAL3/TUBB2A/TUBB2B/TUBB3/TUBB4A/TUBB6	29	0.315217391304348
46	hsa04611	Platelet activation	29/528	126/9434	3.91255813625012e-11	2.32201819825279e-10	1.14601245180781e-10	ADCY2/ADCY3/ADCY5/ADCY9/AKT3/COL3A1/F2/GNA13/GNAI1/GNAQ/GUCY1A1/ITGB3/ITPR1/P2RX1/P2RY1/PIK3CD/PIK3CG/PIK3R2/PIK3R5/PLA2G4B/PLA2G4D/PLA2G4E/PLCB4/PLCG2/PRKACB/PRKG1/PRKG2/PTGS1/RAP1A	29	0.23015873015873
30	hsa04912	GnRH signaling pathway	28/528	93/9434	6.56218016144785e-14	5.97158394691755e-13	2.9472247742643e-13	ADCY2/ADCY3/ADCY5/ADCY9/CACNA1C/CACNA1D/CALML3/CALML5/CALML6/CAMK2A/CAMK2B/CAMK2D/CGA/GNA11/GNAQ/ITPR1/LHB/MAP2K6/MAPK10/PLA2G4B/PLA2G4D/PLA2G4E/PLCB4/PLD1/PRKACB/PRKCB/PTK2B/SOS2	28	0.301075268817204
53	hsa05017	Spinocerebellar ataxia	28/528	144/9434	5.17750332017426e-09	2.66690265359919e-08	1.31622725915056e-08	ADRM1/AKT3/ATP2A1/ATP2A3/BECN2/CACNA1A/GNAQ/GRIA1/GRIA2/GRIA3/GRIN2A/GRIN2B/GRIN2D/GRIN3A/GRIN3B/ITPR1/MAPK10/PIK3CD/PIK3R2/PLCB4/PRKCB/PRKCG/PSMA2/PSMA6/PSMA7/RELN/RYR1/SPTBN2	28	0.194444444444444
60	hsa04150	mTOR signaling pathway	28/528	158/9434	4.33000874519259e-08	1.97015397906263e-07	9.72352841025705e-08	AKT3/ATP6V1C2/ATP6V1E2/ATP6V1F/ATP6V1G2/DDIT4/FZD10/FZD3/FZD9/IGF1/PIK3CD/PIK3R2/PRKCB/PRKCG/RPS6KA6/SOS2/WNT1/WNT11/WNT16/WNT2/WNT2B/WNT3/WNT3A/WNT5A/WNT7A/WNT7B/WNT8B/WNT9A	28	0.177215189873418
62	hsa05226	Gastric cancer	27/528	150/9434	5.39946669551564e-08	2.37750710947705e-07	1.1733985348489e-07	AKT3/APC/AXIN2/BCL2/CCND1/CSNK1A1L/FZD10/FZD3/FZD9/GAB1/PIK3CD/PIK3R2/SHC4/SHH/SOS2/WNT1/WNT11/WNT16/WNT2/WNT2B/WNT3/WNT3A/WNT5A/WNT7A/WNT7B/WNT8B/WNT9A	27	0.18
83	hsa05417	Lipid and atherosclerosis	27/528	216/9434	6.9391537089088e-05	0.000228239634040012	0.000112645741882096	AKT3/BCL2/BCL2L1/BID/CALML3/CALML5/CALML6/CAMK2A/CAMK2B/CAMK2D/CASP7/CCL5/CXCL8/CYP2C8/FAS/HSP90AB1/IRAK1/ITPR1/MAP2K6/MAPK10/MMP1/MMP9/PIK3CD/PIK3R2/PLCB4/PLCG1/RAP1A	27	0.125
14	hsa05033	Nicotine addiction	26/528	41/9434	4.44880391306299e-23	8.67516763047282e-22	4.2815556456546e-22	CACNA1A/CHRFAM7A/CHRNA4/CHRNA6/CHRNA7/CHRNB2/GABRA3/GABRA4/GABRB1/GABRB3/GABRD/GABRE/GABRG2/GABRP/GABRR1/GRIA1/GRIA2/GRIA3/GRIA4/GRIN2A/GRIN2B/GRIN2D/GRIN3A/GRIN3B/SLC17A7/SLC17A8	26	0.634146341463415

	ID	Description	GeneRatio	BgRatio	pvalue	p.adjust	qvalue	geneID	Count	richFactor
44	hsa04970	Salivary secretion	26/528	97/9434	1.02273435546109e-11	6.34560179638355e-11	3.1318181219861e-11	ADCY2/ADCY3/ADCY5/ADCY9/ADRA1A/ADRA1B/ADRA1D/ADRB1/ADRB2/ADRB3/ATP1A2/CALML3/CALML5/CALML6/GNAQ/GUCY1A1/ITPR1/NOS1/PLCB4/PRKACB/PRKCB/PRKCG/PRKG1/PRKG2/RYR3/VAMP2	26	0.268041237113402
59	hsa04915	Estrogen signaling pathway	26/528	139/9434	4.24128948038541e-08	1.962494962958e-07	9.68572798375259e-08	ADCY2/ADCY3/ADCY5/ADCY9/AKT3/BCL2/CALML3/CALML5/CALML6/CREB3L3/GNAI1/GNAO1/GNAQ/GPER1/HSP90AB1/ITPR1/KCNJ5/KCNJ9/MMP9/OPRM1/PIK3CD/PIK3R2/PLCB4/PRKACB/SHC4/SOS2	26	0.18705035971223
64	hsa04550	Signaling pathways regulating pluripotency of stem cells	26/528	144/9434	8.95499045250903e-08	3.81986311489838e-07	1.88526114789664e-07	AKT3/APC/AXIN2/FGFR1/FZD10/FZD3/FZD9/IGF1/IL6ST/INHBA/LIFR/PIK3CD/PIK3R2/SOX2/WNT1/WNT11/WNT16/WNT2/WNT2B/WNT3/WNT3A/WNT5A/WNT7A/WNT7B/WNT8B/WNT9A	26	0.180555555555556
66	hsa05224	Breast cancer	26/528	148/9434	1.58412642606055e-07	6.55252294415955e-07	3.23394230519538e-07	AKT3/APC/AXIN2/CCND1/CSNK1A1L/FGFR1/FZD10/FZD3/FZD9/IGF1/PIK3CD/PIK3R2/SHC4/SOS2/WNT1/WNT11/WNT16/WNT2/WNT2B/WNT3/WNT3A/WNT5A/WNT7A/WNT7B/WNT8B/WNT9A	26	0.175675675675676
35	hsa04721	Synaptic vesicle cycle	25/528	79/9434	4.17982085146507e-13	3.26026026414275e-12	1.60907389169182e-12	ATP6V0A4/ATP6V0D2/ATP6V1C2/ATP6V1E2/ATP6V1F/ATP6V1G2/CACNA1A/CPLX1/RIMS1/SLC17A7/SLC17A8/SLC18A2/SLC18A3/SLC1A1/SLC1A2/SLC1A7/SLC6A1/SLC6A2/SLC6A3/SLC6A9/SNAP25/STX1A/STXBP1/UNC13C/VAMP2	25	0.316455696202532
40	hsa04911	Insulin secretion	25/528	86/9434	3.55710353749154e-12	2.42772316433797e-11	1.19818224420768e-11	ADCY2/ADCY3/ADCY5/ADCY9/ADCYAP1/ADCYAP1R1/ATP1A2/CACNA1C/CACNA1D/CAMK2A/CAMK2B/CAMK2D/CCKAR/CREB3L3/GCG/GNA11/GNAQ/KCNMB2/PLCB4/PRKACB/PRKCB/PRKCG/SNAP25/STX1A/VAMP2	25	0.290697674418605
47	hsa04925	Aldosterone synthesis and secretion	25/528	98/9434	8.3396470434987e-11	4.84409285718116e-10	2.39076107853938e-10	ADCY2/ADCY3/ADCY5/ADCY9/AGT/AGTR1/ATP1A2/CACNA1C/CACNA1D/CALML3/CALML5/CALML6/CAMK2A/CAMK2B/CAMK2D/CAMK4/CREB3L3/GNA11/GNAQ/ITPR1/KCNJ5/PLCB4/PRKACB/PRKCB/PRKCG	25	0.255102040816327
24	hsa04730	Long-term depression	24/528	60/9434	2.91234789789756e-15	3.31279573385847e-14	1.63500232864424e-14	CACNA1A/GNA11/GNA13/GNAI1/GNAO1/GNAQ/GNAZ/GRIA1/GRIA2/GRIA3/GRID2/GUCY1A1/IGF1/ITPR1/NOS1/PLA2G4B/PLA2G4D/PLA2G4E/PLCB4/PRKCB/PRKCG/PRKG1/PRKG2/RYR1	24	0.4
32	hsa04924	Renin secretion	24/528	69/9434	1.15800859030205e-13	9.87926078601434e-13	4.87582564337704e-13	ADCY5/ADCYAP1/ADCYAP1R1/ADRB1/ADRB2/ADRB3/AGT/AGTR1/CACNA1C/CACNA1D/CALML3/CALML5/CALML6/EDN2/EDN3/EDNRA/GNAI1/GNAQ/GUCY1A1/ITPR1/PLCB4/PRKACB/PRKG2/PTGER4	24	0.347826086956522

	ID	Description	GeneRatio	BgRatio	pvalue	p.adjust	qvalue	geneID	Count	richFactor
77	hsa04390	Hippo signaling pathway	24/528	157/9434	6.15017970692816e-06	2.18051825972907e-05	1.07617635336542e-05	APC/AXIN2/CCND1/CSNK1E/DLG2/FZD10/FZD3/FZD9/PPP2R2B/PPP2R2C/SOX2/TP73/WNT1/WNT11/WNT16/WNT2/WNT2B/WNT3/WNT3A/WNT5A/WNT7A/WNT7B/WNT8B/WNT9A	24	0.152866242038217
67	hsa04071	Sphingolipid signaling pathway	23/528	122/9434	2.2191183544177e-07	8.91609073527931e-07	4.40046120730963e-07	ADORA3/AKT3/BCL2/BDKRB2/BID/GNA13/GNAI1/GNAQ/KNG1/MAPK10/OPRD1/PIK3CD/PIK3R2/PLCB4/PLD1/PPP2R2B/PPP2R2C/PPP2R3A/PPP2R3B/PRKCB/PRKCG/S1PR1/S1PR5	23	0.188524590163934
94	hsa05415	Diabetic cardiomyopathy	23/528	205/9434	0.00110002843468014	0.00319476343263488	0.00157674848419998	AGT/AGTR1/AKT3/ATP2A1/ATP2A3/CAMK2A/CAMK2B/CAMK2D/COL3A1/COX7A1/MAPK10/MMP9/NDUFA11/NDUFA13/NDUFA4L2/NDUFS7/PIK3CD/PIK3R2/PLCB4/PRKCB/PRKCG/SDHD/SLC2A4	23	0.11219512195122
42	hsa04720	Long-term potentiation	22/528	67/9434	4.72559994997726e-12	3.07163996748522e-11	1.51598193883982e-11	CACNA1C/CALML3/CALML5/CALML6/CAMK2A/CAMK2B/CAMK2D/CAMK4/GNAQ/GRIA1/GRIA2/GRIN2A/GRIN2B/GRIN2D/ITPR1/PLCB4/PPP1R1A/PRKACB/PRKCB/PRKCG/RAP1A/RPS6KA6	22	0.328358208955224
102	hsa04510	Focal adhesion	22/528	203/9434	0.00218394744983928	0.00584527111157463	0.00288488414426654	AKT3/BCL2/CAPN2/CAV1/CCND1/COL4A6/IGF1/ITGA4/ITGB3/MAPK10/PAK6/PIK3CD/PIK3R2/PRKCB/PRKCG/RAP1A/RELN/SHC4/SOS2/TNR/VCL/VEGFA	22	0.108374384236453
119	hsa04810	Regulation of actin cytoskeleton	22/528	232/9434	0.0106200507326365	0.0243636457984013	0.0120244714177573	AKT3/APC/BDKRB1/BDKRB2/CHRM2/CHRM4/CHRM5/CXCL12/F2/FGFR1/GNA13/GNG12/ITGA4/ITGB3/KNG1/LIMK1/LPAR1/PAK6/PIK3CD/PIK3R2/SOS2/VCL	22	0.0948275862068965
39	hsa04923	Regulation of lipolysis in adipocytes	21/528	59/9434	2.43073598425981e-12	1.70151518898187e-11	8.39768437207168e-12	ADCY2/ADCY3/ADCY5/ADCY9/ADRB1/ADRB2/ADRB3/AKT3/CGA/GNAI1/MGLL/NPY/NPY1R/PIK3CD/PIK3R2/PRKACB/PRKG1/PRKG2/PTGER3/PTGS1/TSHR	21	0.355932203389831
45	hsa04929	GnRH secretion	21/528	65/9434	2.07278673965897e-11	1.25749062205977e-10	6.20623865909585e-11	AKT3/ARRB1/CACNA1C/CACNA1D/CGA/GNA11/GNAQ/GPER1/HCN1/ITPR1/KCNJ5/KCNJ9/KISS1/KISS1R/LHB/PIK3CD/PIK3R2/PLCB4/PRKCB/PRKCG/TRPC1	21	0.323076923076923
50	hsa04971	Gastric acid secretion	21/528	76/9434	5.55561675516679e-10	3.03336674832107e-09	1.49709251507652e-09	ADCY2/ADCY3/ADCY5/ADCY9/ATP1A2/CALML3/CALML5/CALML6/CAMK2A/CAMK2B/CAMK2D/GNAI1/GNAQ/ITPR1/KCNQ1/PLCB4/PRKACB/PRKCB/PRKCG/SST/SSTR2	21	0.276315789473684
72	hsa04928	Parathyroid hormone synthesis, secretion and action	21/528	115/9434	1.29146594542019e-06	4.89680837638487e-06	2.4167783773945e-06	ADCY2/ADCY3/ADCY5/ADCY9/ARRB1/BCL2/CREB3L3/FGFR1/GNA11/GNA13/GNAI1/GNAQ/ITPR1/MEF2C/MMP13/PLCB4/PLD1/PRKACB/PRKCB/PRKCG/PTH1R	21	0.182608695652174
127	hsa04714	Thermogenesis	21/528	235/9434	0.0226719825559853	0.0487358365179841	0.0240531601091265	ADCY2/ADCY3/ADCY5/ADCY9/ADRB3/CNR1/COX7A1/CREB3L3/FGFR1/GCG/MGLL/NDUFA11/NDUFA13/NDUFA4L2/NDUFS7/PRKACB/PRKG1/PRKG2/RPS6KA6/SDHD/SOS2	21	0.0893617021276596

	ID	Description	GeneRatio	BgRatio	pvalue	p.adjust	qvalue	geneID	Count	richFactor
139	hsa05132	Salmonella infection	21/528	251/9434	0.0421752889363238	0.0828334811483194	0.040881764360844	AKT3/BCL2/CASP7/CXCL8/HSP90AB1/IRAK1/KIF5A/KIF5C/KLC3/MAP2K6/MAPK10/PIK3CD/PIK3CG/RIPK2/TUBA1A/TUBAL3/TUBB2A/TUBB2B/TUBB3/TUBB4A/TUBB6	21	0.0836653386454183
90	hsa04514	Cell adhesion molecules	20/528	160/9434	0.000592634534573924	0.00179765808820757	0.00088721895234588	CDH4/CLDN1/CLDN19/CNTN2/CNTNAP1/ITGA4/L1CAM/MAG/NCAM1/NCAM2/NEO1/NLGN1/NLGN3/NLGN4X/NLGN4Y/NRCAM/NRXN1/NRXN2/NTNG1/SLITRK3	20	0.125
41	hsa05030	Cocaine addiction	19/528	49/9434	4.62594673514175e-12	3.07163996748522e-11	1.51598193883982e-11	ADCY5/BDNF/CREB3L3/DRD2/FOSB/GNAI1/GRIA2/GRIN2A/GRIN2B/GRIN2D/GRIN3A/GRIN3B/GRM2/MAOA/MAOB/PRKACB/SLC18A2/SLC6A3/TH	19	0.387755102040816
75	hsa05414	Dilated cardiomyopathy	19/528	105/9434	4.74930591792174e-06	1.72874735412351e-05	8.53208642096818e-06	ADCY2/ADCY3/ADCY5/ADCY9/ADRB1/AGT/ATP2A1/ATP2A3/CACNA1C/CACNA1D/CACNG4/CACNG5/CACNG7/CACNG8/IGF1/ITGA4/ITGB3/NOS1/PRKACB	19	0.180952380952381
76	hsa04972	Pancreatic secretion	19/528	106/9434	5.49112853100102e-06	1.97247116968852e-05	9.73496470869987e-06	ADCY2/ADCY3/ADCY5/ADCY9/ATP1A2/ATP2A1/ATP2A3/CCKAR/GNAQ/ITPR1/KCNQ1/PLCB4/PRKCB/PRKCG/PRSS1/PRSS2/RAP1A/SCTR/TRPC1	19	0.179245283018868
54	hsa05217	Basal cell carcinoma	18/528	63/9434	5.34246563044457e-09	2.70091317983587e-08	1.33301286685557e-08	APC/AXIN2/FZD10/FZD3/FZD9/SHH/WNT1/WNT11/WNT16/WNT2/WNT2B/WNT3/WNT3A/WNT5A/WNT7A/WNT7B/WNT8B/WNT9A	18	0.285714285714286
65	hsa05214	Glioma	18/528	76/9434	1.30706566552236e-07	5.48967579519391e-07	2.70938307994918e-07	AKT3/CALML3/CALML5/CALML6/CAMK2A/CAMK2B/CAMK2D/CAMK4/CCND1/IGF1/PIK3CD/PIK3R2/PLCG1/PLCG2/PRKCB/PRKCG/SHC4/SOS2	18	0.236842105263158
79	hsa04933	AGE-RAGE signaling pathway in diabetic complications	18/528	101/9434	1.05133748666779e-05	3.63310296025705e-05	1.79308725241142e-05	AGT/AGTR1/AKT3/BCL2/CCND1/COL3A1/COL4A6/CXCL8/IL1A/MAPK10/NOX4/PIK3CD/PIK3R2/PLCB4/PLCG1/PLCG2/PRKCB/VEGFA	18	0.178217821782178
81	hsa05142	Chagas disease	18/528	103/9434	1.39341529952737e-05	4.69632563914781e-05	2.3178318172775e-05	AKT3/BDKRB2/CCL5/CXCL8/FAS/GNA11/GNAI1/GNAL/GNAO1/GNAQ/IRAK1/KNG1/MAPK10/PIK3CD/PIK3R2/PLCB4/PPP2R2B/PPP2R2C	18	0.174757281553398
92	hsa04114	Oocyte meiosis	18/528	139/9434	0.000721539639593536	0.00214109045227212	0.00105671709231 09	ADCY2/ADCY3/ADCY5/ADCY9/AURKA/CALML3/CALML5/CALML6/CAMK2A/CAMK2B/CAMK2D/CCNB1/CDK1/IGF1/ITPR1/MAD2L1/PRKACB/RPS6KA6	18	0.129496402877698
106	hsa04932	Non-alcoholic fatty liver disease	18/528	157/9434	0.00291476189113305	0.00750688675735 21	0.00370496049717011	AKT3/BID/CASP7/COX7A1/CXCL8/FAS/IL1A/IL6R/LEP/LEPR/MAPK10/NDUFA11/NDUFA13/NDUFA4L2/NDUFS7/PIK3CD/PIK3R2/SDHD	18	0.114649681528662
74	hsa04012	ErbB signaling pathway	17/528	86/9434	4.33082373472097e-06	1.59772281024166e-05	7.88542586122738e-06	AKT3/CAMK2A/CAMK2B/CAMK2D/ERBB4/GAB1/MAPK10/NRG3/PAK6/PIK3CD/PIK3R2/PLCG1/PLCG2/PRKCB/PRKCG/SHC4/SOS2	17	0.197674418604651

	ID	Description	GeneRatio	BgRatio	pvalue	p.adjust	qvalue	geneID	Count	richFactor
88	hsa04914	Progesterone-mediated oocyte maturation	17/528	111/9434	0.000134774387904765	0.000418106907931827	0.000206353129806338	ADCY2/ADCY3/ADCY5/ADCY9/AKT3/AURKA/CCNB1/CDK1/GNAI1/HSP90AB1/IGF1/MAD2L1/MAPK10/PIK3CD/PIK3R2/PRKACB/RPS6KA6	17	0.153153153153153
105	hsa05418	Fluid shear stress and atherosclerosis	17/528	142/9434	0.00238734383240262	0.0062070939642468	0.00306345875235624	AKT3/BCL2/CALML3/CALML5/CALML6/CAV1/DUSP1/HSP90AB1/IL1A/ITGB3/MAP2K6/MAPK10/MEF2C/MMP9/PIK3CD/PIK3R2/VEGFA	17	0.119718309859155
145	hsa04814	Motor proteins	17/528	197/9434	0.049768824956035	0.0937026842275693	0.0462461676542467	DNAH10/DNAH12/DNAH14/DNAH2/DNAH5/KIF14/KIF1A/KIF5A/KIF5C/KLC3/TUBA1A/TUBAL3/TUBB2A/TUBB2B/TUBB3/TUBB4A/TUBB6	17	0.0862944162436548
73	hsa04918	Thyroid hormone synthesis	16/528	75/9434	2.90709013965982e-06	1.08717206592758e-05	5.36564582374127e-06	ADCY2/ADCY3/ADCY5/ADCY9/ATP1A2/CGA/CREB3L3/GNAQ/GPX2/GPX3/ITPR1/PLCB4/PRKACB/PRKCB/PRKCG/TSHR	16	0.213333333333333
78	hsa01521	EGFR tyrosine kinase inhibitor resistance	16/528	80/9434	7.06360256016941e-06	2.4722608960593e-05	1.22016346518446e-05	AKT3/AXL/BCL2/BCL2L1/GAB1/IGF1/IL6R/PIK3CD/PIK3R2/PLCG1/PLCG2/PRKCB/PRKCG/SHC4/SOS2/VEGFA	16	0.2
85	hsa01522	Endocrine resistance	16/528	99/9434	0.000110306453598409	0.000350158858515879	0.000172817944438144	ADCY2/ADCY3/ADCY5/ADCY9/AKT3/BCL2/CCND1/GPER1/IGF1/MAPK10/MMP9/PIK3CD/PIK3R2/PRKACB/SHC4/SOS2	16	0.161616161616162
86	hsa05231	Choline metabolism in cancer	16/528	99/9434	0.000110306453598409	0.000350158858515879	0.000172817944438144	AKT3/MAPK10/PIK3CD/PIK3R2/PLA2G4B/PLA2G4D/PLA2G4E/PLCG1/PLD1/PRKCB/PRKCG/SLC22A2/SLC22A3/SLC44A4/SLC5A7/SOS2	16	0.161616161616162
124	hsa05161	Hepatitis B	16/528	163/9434	0.0201112138016927	0.0442771078053396	0.0218525922463215	AKT3/BCL2/BID/CREB3L3/CXCL8/FAS/IRAK1/MAP2K6/MAPK10/MMP9/PIK3CD/PIK3R2/PRKCB/PRKCG/PTK2B/SOS2	16	0.098159509202454
143	hsa05152	Tuberculosis	16/528	182/9434	0.0486120383168301	0.0928048004230392	0.0458030246930751	AKT3/ATP6V0A4/ATP6V0D2/BCL2/BID/CALML3/CALML5/CALML6/CAMK2A/CAMK2B/CAMK2D/IL1A/IRAK1/IRAK2/MAPK10/RIPK2	16	0.0879120879120879
84	hsa04742	Taste transduction	15/528	86/9434	7.36619629383761e-05	0.000239401379549722	0.000118154527018949	CACNA1A/CACNA1C/GABRA3/GABRA4/GNG13/GRM4/HTR1D/HTR3A/HTR3C/HTR3E/P2RX2/P2RY1/P2RY4/PLCB4/PRKACB	15	0.174418604651163
93	hsa04922	Glucagon signaling pathway	15/528	107/9434	0.000861968819223969	0.00253029556610907	0.00124880598597247	ADCY2/AKT3/CALML3/CALML5/CALML6/CAMK2A/CAMK2B/CAMK2D/CREB3L3/GCG/GCGR/GNAQ/ITPR1/PLCB4/PRKACB	15	0.14018691588785
95	hsa04066	HIF-1 signaling pathway	15/528	110/9434	0.00115168503952178	0.00330957911357312	0.00163341479289516	AKT3/BCL2/CAMK2A/CAMK2B/CAMK2D/EPAS1/IGF1/IL6R/PIK3CD/PIK3R2/PLCG1/PLCG2/PRKCB/PRKCG/VEGFA	15	0.136363636363636
101	hsa04670	Leukocyte transendothelial migration	15/528	116/9434	0.00198079054989819	0.00535401802101194	0.00264243033232901	CLDN1/CLDN19/CXCL12/GNAI1/ITGA4/MMP9/PIK3CD/PIK3R2/PLCG1/PLCG2/PRKCB/PRKCG/PTK2B/RAP1A/VCL	15	0.129310344827586
107	hsa04919	Thyroid hormone signaling pathway	15/528	122/9434	0.00325638658127231	0.00830835081016206	0.00410051630499612	AKT3/ATP1A2/ATP2A1/ATP2A3/CCND1/ITGB3/PIK3CD/PIK3R2/PLCB4/PLCG1/PLCG2/PRKACB/PRKCB/PRKCG/THRB	15	0.122950819672131
134	hsa04218	Cellular senescence	15/528	157/9434	0.0296163441099223	0.0603377756866327	0.0297791991050279	AKT3/CACNA1D/CALML3/CALML5/CALML6/CAPN2/CCNB1/CCND1/CDK1/CXCL8/IL1A/ITPR1/MAP2K6/PIK3CD/PIK3R2	15	0.0955414012738854

	ID	Description	GeneRatio	BgRatio	pvalue	p.adjust	qvalue	geneID	Count	richFactor
135	hsa04145	Phagosome	15/528	159/9434	0.0326589351843781	0.0660436244839646	0.0325952725426932	ATP6V0A4/ATP6V0D2/ATP6V1C2/ATP6V1E2/ATP6V1F/ATP6V1G2/ITGB3/NOS1/TUBA1A/TUBAL3/TUBB2A/TUBB2B/TUBB3/TUBB4A/TUBB6	15	0.0943396226415094
144	hsa04630	JAK-STAT signaling pathway	15/528	168/9434	0.0492111369846914	0.0932961138668109	0.0460455082897698	AKT3/BCL2/BCL2L1/CCND1/CNTFR/GHR/IL6R/IL6ST/LEP/LEPR/LIFR/PIK3CD/PIK3R2/PRL/SOS2	15	0.0892857142857143
80	hsa04927	Cortisol synthesis and secretion	14/528	65/9434	1.07737403853608e-05	3.67653890650438e-05	1.81452469648182e-05	ADCY2/ADCY3/ADCY5/ADCY9/AGT/AGTR1/CACNA1C/CACNA1D/CREB3L3/GNA11/GNAQ/ITPR1/PLCB4/PRKACB	14	0.215384615384615
98	hsa05146	Amoebiasis	14/528	103/9434	0.00171701164476842	0.00478310386756916	0.00236066047830674	COL3A1/COL4A6/CTSG/CXCL8/GNA11/GNAL/GNAQ/PIK3CD/PIK3R2/PLCB4/PRKACB/PRKCB/PRKCG/VCL	14	0.135922330097087
114	hsa04152	AMPK signaling pathway	14/528	122/9434	0.00809362475872962	0.0193821013959051	0.00956587229101931	ADRA1A/AKT3/CCND1/CREB3L3/IGF1/LEP/LEPR/PIK3CD/PIK3R2/PPP2R2B/PPP2R2C/PPP2R3A/PPP2R3B/SLC2A4	14	0.114754098360656
123	hsa04068	FoxO signaling pathway	14/528	133/9434	0.0165816978337391	0.0368032805578112	0.0181639479907454	AKT3/CCNB1/CCND1/CSNK1E/FOXG1/GABARAP/HOMER1/IGF1/MAPK10/PIK3CD/PIK3R2/S1PR1/SLC2A4/SOS2	14	0.105263157894737
126	hsa04210	Apoptosis	14/528	137/9434	0.0209557337176334	0.045404089721539	0.0224088046437517	AKT3/BCL2/BCL2L1/BID/CAPN2/CASP7/FAS/ITPR1/MAPK10/NGF/PIK3CD/PIK3R2/TUBA1A/TUBAL3	14	0.102189781021898
132	hsa04380	Osteoclast differentiation	14/528	143/9434	0.02907308744725	0.0601284308568126	0.0296758787340351	AKT3/ATP2A1/ATP2A3/CAMK4/CSF1/FOSB/IL1A/ITGB3/ITPR1/MAP2K6/MAPK10/PIK3CD/PIK3R2/PLCG2	14	0.0979020979020979
104	hsa05323	Rheumatoid arthritis	13/528	95/9434	0.00233189733289065	0.00612123049883795	0.00302108156487857	ATP6V0A4/ATP6V0D2/ATP6V1C2/ATP6V1E2/ATP6V1F/ATP6V1G2/CCL5/CSF1/CXCL12/CXCL8/IL1A/MMP1/VEGFA	13	0.136842105263158
109	hsa05410	Hypertrophic cardiomyopathy	13/528	99/9434	0.00337242243283827	0.00844652590976924	0.00416871145729887	AGT/ATP2A1/ATP2A3/CACNA1C/CACNA1D/CACNG4/CACNG5/CACNG7/CACNG8/IGF1/ITGA4/ITGB3/NOS1	13	0.131313131313131
140	hsa05135	Yersinia infection	13/528	138/9434	0.0451030079021804	0.0879508654092517	0.0434074061013465	AKT3/CXCL8/GNAQ/IRAK1/ITGA4/LIMK1/MAP2K6/MAPK10/PIK3CD/PIK3R2/PLCG1/PTK2B/RPS6KA6	13	0.0942028985507246
82	hsa05110	Vibrio cholerae infection	12/528	51/9434	1.77808089462912e-05	5.91970834431401e-05	2.92162200914669e-05	ADCY3/ADCY9/ATP6V0A4/ATP6V0D2/ATP6V1C2/ATP6V1E2/ATP6V1F/ATP6V1G2/KCNQ1/PLCG1/PLCG2/PRKACB	12	0.235294117647059
117	hsa04666	Fc gamma R-mediated phagocytosis	12/528	99/9434	0.00901330819335857	0.02103105245117	0.0103796981443985	AKT3/LIMK1/PIK3CD/PIK3R2/PLA2G4B/PLA2G4D/PLA2G4E/PLCG1/PLCG2/PLD1/PRKCB/PRKCG	12	0.121212121212121
136	hsa04668	TNF signaling pathway	12/528	119/9434	0.0340181259980303	0.0682863852754579	0.0337021681714232	AKT3/CASP7/CCL5/CREB3L3/CSF1/FAS/MAP2K6/MAPK10/MMP9/PIK3CD/PIK3R2/RPS6KA5	12	0.100840336134454
87	hsa04913	Ovarian steroidogenesis	11/528	52/9434	0.000112825666393059	0.00035403916006097	0.00017473303446233	ADCY2/ADCY3/ADCY5/ADCY9/CGA/IGF1/LHB/PLA2G4B/PLA2G4D/PLA2G4E/PRKACB	11	0.211538461538462
89	hsa04370	VEGF signaling pathway	11/528	60/9434	0.00042634264097072	0.0013077701234 2505	0.000645438888754218	AKT3/PIK3CD/PIK3R2/PLA2G4B/PLA2G4D/PLA2G4E/PLCG1/PLCG2/PRKCB/PRKCG/VEGFA	11	0.183333333333333

	ID	Description	GeneRatio	BgRatio	pvalue	p.adjust	qvalue	geneID	Count	richFactor
96	hsa04664	Fc epsilon RI signaling pathway	11/528	69/9434	0.00143889227110538	0.00409184989595594	0.00201949792435844	AKT3/MAP2K6/MAPK10/PIK3CD/PIK3R2/PLA2G4B/PLA2G4D/PLA2G4E/PLCG1/PLCG2/SOS2	11	0.159420289855072
99	hsa04917	Prolactin signaling pathway	11/528	71/9434	0.00182618029198149	0.00498547219710946	0.00246053765656453	AKT3/CCND1/CGA/LHB/MAPK10/PIK3CD/PIK3R2/PRL/SHC4/SOS2/TH	11	0.154929577464789
100	hsa05120	Epithelial cell signaling in Helicobacter pylori infection	11/528	71/9434	0.00182618029198149	0.00498547219710946	0.00246053765656453	ATP6V0A4/ATP6V0D2/ATP6V1C2/ATP6V1E2/ATP6V1F/ATP6V1G2/CCL5/CXCL8/MAPK10/PLCG1/PLCG2	11	0.154929577464789
103	hsa05223	Non-small cell lung cancer	11/528	73/9434	0.00229447844367349	0.00608148170022197	0.00300146388135112	AKT3/CCND1/KIF5A/KIF5C/PIK3CD/PIK3R2/PLCG1/PLCG2/PRKCB/PRKCG/SOS2	11	0.150684931506849
115	hsa05412	Arrhythmogenic right ventricular cardiomyopathy	11/528	86/9434	0.00821165781977798	0.0194937616069512	0.009620981244225	ATP2A1/ATP2A3/CACNA1C/CACNA1D/CACNG4/CACNG5/CACNG7/CACNG8/ITGA4/ITGB3/NOS1	11	0.127906976744186
120	hsa04211	Longevity regulating pathway	11/528	90/9434	0.0114346454830291	0.0260138184738912	0.0128389001914713	ADCY2/ADCY3/ADCY5/ADCY9/AKT3/CAMK4/CREB3L3/IGF1/PIK3CD/PIK3R2/PRKACB	11	0.122222222222222
125	hsa04070	Phosphatidylinositol signaling system	11/528	98/9434	0.020676627230233	0.0451577538708289	0.0222872276671143	CALML3/CALML5/CALML6/ITPR1/PIK3CD/PIK3R2/PLCB4/PLCG1/PLCG2/PRKCB/PRKCG	11	0.112244897959184
129	hsa04061	Viral protein interaction with cytokine and cytokine receptor	11/528	100/9434	0.023666451677473	0.0500848163406987	0.0247189376965854	CCL5/CCR2/CCR7/CSF1/CX3CR1/CXCL12/CXCL13/CXCL8/CXCR5/IL6R/IL6ST	11	0.11
137	hsa05215	Prostate cancer	11/528	106/9434	0.0345173418452264	0.0687827322901226	0.0339471360444793	AKT3/BCL2/CCND1/CREB3L3/FGFR1/HSP90AB1/IGF1/MMP9/PIK3CD/PIK3R2/SOS2	11	0.10377358490566
91	hsa04961	Endocrine and other factor-regulated calcium reabsorption	10/528	53/9434	0.000609598935364448	0.00182879680609334	0.000902587203315782	ADCY9/ATP1A2/BDKRB2/CALB1/GNAQ/PLCB4/PRKACB/PRKCB/PRKCG/PTH1R	10	0.188679245283019
116	hsa04115	p53 signaling pathway	10/528	75/9434	0.00862617531109064	0.0203012574131702	0.0100195139729546	BCL2/BCL2L1/BID/CCNB1/CCND1/CDK1/FAS/IGF1/RRM2/TP73	10	0.133333333333333
128	hsa04260	Cardiac muscle contraction	10/528	87/9434	0.0229994608902792	0.0490535376800485	0.0242099588318728	ATP1A2/ATP2A1/ATP2A3/CACNA1C/CACNA1D/CACNG4/CACNG5/CACNG7/CACNG8/COX7A1	10	0.114942528735632
113	hsa04213	Longevity regulating pathway - multiple species	9/528	62/9434	0.00718725948573704	0.0173639100850107	0.00856981103096732	ADCY2/ADCY3/ADCY5/ADCY9/AKT3/IGF1/PIK3CD/PIK3R2/PRKACB	9	0.145161290322581
122	hsa05211	Renal cell carcinoma	9/528	70/9434	0.0155507272766431	0.034797938905931	0.0171742285712711	AKT3/EPAS1/GAB1/PAK6/PIK3CD/PIK3R2/RAP1A/SOS2/VEGFA	9	0.128571428571429
133	hsa05133	Pertussis	9/528	78/9434	0.0295072239443353	0.0603377756866327	0.0297791991050279	CALML3/CALML5/CALML6/CASP7/CXCL8/GNAI1/IL1A/IRAK1/MAPK10	9	0.115384615384615
97	hsa04215	Apoptosis - multiple species	7/528	32/9434	0.00163392436936225	0.00459857064779273	0.00226958566769797	BCL2/BCL2L1/BECN2/BID/CASP7/MAPK10/NGFR	7	0.21875
111	hsa05143	African trypanosomiasis	7/528	37/9434	0.00392267132851006	0.00964765110525448	0.00476151664342616	F2RL1/FAS/GNAQ/KNG1/PLCB4/PRKCB/PRKCG	7	0.189189189189189
112	hsa04960	Aldosterone-regulated sodium reabsorption	7/528	38/9434	0.00458169382075635	0.0111678786880936	0.00551181211519561	ATP1A2/IGF1/NEDD4L/PIK3CD/PIK3R2/PRKCB/PRKCG	7	0.184210526315789
121	hsa04930	Type II diabetes mellitus	7/528	47/9434	0.0148160720493988	0.0334279972684784	0.0164981054573558	CACNA1A/CACNA1C/CACNA1D/MAPK10/PIK3CD/PIK3R2/SLC2A4	7	0.148936170212766
130	hsa04973	Carbohydrate digestion and absorption	7/528	52/9434	0.0248317444615346	0.0521466633692226	0.0257365448670156	AKT3/ATP1A2/CACNA1D/PIK3CD/PIK3R2/PLCB4/PRKCB	7	0.134615384615385
138	hsa04340	Hedgehog signaling pathway	7/528	56/9434	0.0356073645653182	0.0704406559879121	0.0347653902697234	ARRB1/BCL2/CCND1/CSNK1A1L/CSNK1E/PRKACB/SHH	7	0.125
141	hsa05213	Endometrial cancer	7/528	59/9434	0.0454590065768048	0.0880163744359411	0.0434397375276671	AKT3/APC/AXIN2/CCND1/PIK3CD/PIK3R2/SOS2	7	0.11864406779661

	ID	Description	GeneRatio	BgRatio	pvalue	p.adjust	qvalue	geneID	Count	richFactor
110	hsa04966	Collecting duct acid secretion	6/528	28/9434	0.00391299716366633	0.00964765110525448	0.00476151664342616	ATP6V0A4/ATP6V0D2/ATP6V1C2/ATP6V1E2/ATP6V1F/ATP6V1G2	6	0.214285714285714
131	hsa05219	Bladder cancer	6/528	41/9434	0.0253187413147365	0.052763483808573	0.0260409713803638	CCND1/CXCL8/MMP1/MMP9/RPS6KA5/VEGFA	6	0.146341463414634
142	hsa00350	Tyrosine metabolism	5/528	36/9434	0.0484327283635992	0.0928048004230392	0.0458030246930751	COMT/MAOA/MAOB/MIF/TH	5	0.138888888888889
108	hsa03265	Virion - Ebolavirus, Lyssavirus and Morbillivirus	4/528	12/9434	0.00334788385281839	0.00844652590976924	0.00416871145729887	CHRNA1/NCAM1/NCAM2/NGFR	4	0.333333333333333
118	hsa00360	Phenylalanine metabolism	4/528	16/9434	0.0102974697042237	0.0238238070275684	0.0117580385561163	MAOA/MAOB/MIF/PAH	4	0.25

Supplementary Table 6: Genes selected by LASSO regression with their regression coefficients at lambda.min.

variable	coefficient
POU4F1	0.147364739283179
ATP2A1	0.178077614233134
KLC3	0.175640690796946
CALB2	0.0769326700081523

Supplementary Table 7: GSEA enrichment pathway table.

	ID	Description	setSize	enrichmentScore	NES	pvalue	p.adjust	qvalue	rank	leading_edge	core_enrichment
KEGG_OXIDATIVE_PHOSPHORYLATION	KEGG_OXIDATIVE_PHOSPHORYLATION	KEGG_OXIDATIVE_PHOSPHORYLATION	116	0.569541463894845	2.59165583478799	3.83884725645068e-13	6.76441159635193e-11	4.88808121045061e-11	3153	tags=46%, list=17%, signal=38%	ATP12A/NDUFA11/COX8A/CYC1/ATP5MF/UQCR11/NDUFB7/NDUFS7/COX4I2/NDUFS8/ATP6V1F/NDUFB8/NDUFA9/ATP6V0B/COX6A1/COX7A1/NDUFB10/SDHA/COX5B/ATP5ME/ATP5F1D/ATP5MG/NDUFA2/NDUFA3/COX7B/NDUFS3/NDUFV1/COX4I1/ATP5MC1/COX5A/NDUFB2/ATP5F1E/UQCRC1/UQCRQ/LHPP/UQCRFS1/NDUFS6/ATP6V0E2/COX7A2/NDUFAB1/ATP5MC3/ATP6V0D1/UQCR10/COX6B1/NDUFB1/NDUFA8/ATP5PO/NDUFB9/NDUFB4/NDUFA10/ATP5F1B/NDUFA1/UQCRHL
KEGG_CYTOKINE_CYTOKINE_RECEPTOR_INTERACTION	KEGG_CYTOKINE_CYTOKINE_RECEPTOR_INTERACTION	KEGG_CYTOKINE_CYTOKINE_RECEPTOR_INTERACTION	261	-0.562428626716005	-1.97760521710663	7.31287740146154e-13	6.76441159635193e-11	4.88808121045061e-11	3553	tags=45%, list=19%, signal=37%	IFNGR1/PDGFRA/CXCL16/CCL28/IFNA1/CSF1R/IL23A/CCL18/VEGFC/GDF5/IL1RAP/LTA/IL10/TNFRSF13B/IL19/CRLF2/CSF1/IL1R2/FAS/IL1B/TNFRSF8/CXCL3/IFNK/IL15/CTF1/IL22/CCL21/IL6/IL6ST/CXCR1/IL12RB2/OSMR/LEPR/TNFSF14/IL12RB1/INHBC/KITLG/CCR4/IL17A/CXCR2/IL1R1/LIFR/IL10RA/CD70/CCL22/IL18RAP/CCL23/INHBE/XCL2/TPO/CSF2RB/CCL4L2/BMPR1B/IL7/CCL2/CX3CL1/IL22RA2/HGF/TNFSF4/TGFB3/PRL/CSF2/CCL17/TNFSF9/IL26/CCR2/CCR1/CSF3R/IL12B/PDGFC/CXCL6/IL2RB/NGFR/IL9R/XCR1/CNTFR/CXCL1/INHBA/CXCR6/IL21R/TGFB2/CSF3/TNFRSF9/IL1A/CCL4/FASLG/CXCR4/IL2RA/CCR7/CCL13/CCR5/IL7R/CCR8/XCL1/CCL19/CXCL9/CCL26/TNFSF8/CCL5/TNFSF18/TNFSF13B/EDA2R/CCL8/CXCR5/CXCL8/IL21/TNFRSF11B/CCL7/IFNG/CXCL10/CXCL13/CXCL11/CXCL5/LEP/IFNE/PPBP/CCL25
KEGG_RIBOSOME	KEGG_RIBOSOME	KEGG_RIBOSOME	87	0.574768198803246	2.4018959298061	2.19288746197955e-10	1.35228060155406e-08	9.77181430425977e-09	4074	tags=54%, list=22%, signal=43%	RPS21/RPS5/RPS10/RPL10L/RPL8/RPLP2/RPS18/RPS28/RPL19/FAU/RPL27/RPS4Y1/RPS3/RPS16/RPLP1/RPS15/RPL13/RPL18A/RPL18/RPS23/RPS2/RPL23A/RPS9/RPL28/RPL35/RPL17/UBA52/RPS19/RPS13/RPL30/RPLP0/RPS11/RPL27A/RPS26/RPS17/RPS8/RPS15A/RPL7A/RPL10/RPL26/RPL23/RPL3L/RPL36/RPL15/RPL26L1/RPSA/RPL13A

	ID	Description	setSize	enrichmentScore	NES	pvalue	p.adjust	qvalue	rank	leading_edge	core_enrichment
KEGG_PARKINSONS_DISEASE	KEGG_PARKINSONS_DISEASE	KEGG_PARKINSONS_DISEASE	113	0.5137436297338	2.32153498776902	3.29647413621769e-10	1.52461928800068e-08	1.1017163560517e-08	3153	tags=42%, list=17%, signal=36%	TH/COX8A/CYC1/SLC25A31/UQCR11/NDUFB7/SEPTIN5/NDUFS7/COX4I2/NDUFS8/NDUFB8/NDUFA9/COX6A1/COX7A1/SLC25A6/NDUFB10/SLC25A5/SDHA/COX5B/ATP5F1D/NDUFA2/NDUFA3/COX7B/NDUFS3/NDUFV1/COX4I1/ATP5MC1/COX5A/NDUFB2/ATP5F1E/UQCRC1/UQCRQ/UQCRFS1/NDUFS6/COX7A2/NDUFAB1/ATP5MC3/UQCR10/COX6B1/NDUFB1/NDUFA8/ATP5PO/NDUFB9/NDUFB4/NDUFA10/ATP5F1B/NDUFA1/UQCRHL
KEGG_CELL_ADHESION_MOLECULES_CAMS	KEGG_CELL_ADHESION_MOLECULES_CAMS	KEGG_CELL_ADHESION_MOLECULES_CAMS	130	-0.612775656016554	-2.04372562689057	5.39241222601125e-10	1.99519252362416e-08	1.44176074253354e-08	4175	tags=58%, list=22%, signal=46%	CLDN1/HLA-E/CNTNAP2/MADCAM1/PTPRM/SDC2/CD28/CLDN19/L1CAM/CD6/CD226/CD4/SPN/ITGB7/HLA-F/ITGB1/ICAM3/SELP/ITGAL/SELPLG/JAM3/NCAM1/NLGN3/ITGA4/CNTNAP1/ICAM1/JAM2/NRCAM/CD80/ICOSLG/PDCD1/ALCAM/HLA-DPB1/HLA-DMB/HLA-DQB1/ITGAV/MAG/HLA-DMA/HLA-DRB1/VCAM1/HLA-DPA1/ITGB8/SIGLEC1/HLA-DOB/NEGR1/HLA-DQA2/ITGB2/CD2/ICOS/ITGAM/CTLA4/NLGN4X/CDH2/SELE/HLA-DRA/NCAM2/VCAN/CLDN8/PTPRC/CD274/HLA-DOA/SELL/HLA-DRB5/CD86/CD8A/CLDN16/PDCD1LG2/HLA-DQA1/CLDN11/CNTN2/CADM3/NLGN1/NRXN1/CD22/CNTN1/CLDN18

	ID	Description	setSize	enrichmentScore	NES	pvalue	p.adjust	qvalue	rank	leading_edge	core_enrichment
KEGG_NEUROACTIVE_LIGAND_RECEPTOR_INTERACTION	KEGG_NEUROACTIVE_LIGAND_RECEPTOR_INTERACTION	KEGG_NEUROACTIVE_LIGAND_RECEPTOR_INTERACTION	272	-0.501264513767884	-1.76804014031932	2.27276941868696e-08	7.00770570761811e-07	5.06388975742532e-07	3707	tags=41%, list=20%, signal=34%	DRD2/C5AR1/AVPR2/GNRHR/MTNR1B/BRS3/GRM7/GABRG1/GRIA1/CALCRL/F2RL2/CGA/P2RY14/FPR1/CHRNG/HRH4/MAS1/F2R/NPFFR2/MC2R/NR3C1/NPY1R/CYSLTR1/GABRB1/CHRM1/P2RY1/LPAR6/GLRA1/GRM1/GLRA3/LEPR/GRIN1/ADORA3/GRM6/HRH2/DRD3/TSHR/SSTR4/CYSLTR2/C3AR1/PTGFR/GABRA5/CTSG/P2RY13/AGTR2/FPR3/P2RY10/AVPR1A/MCHR2/SSTR1/PRL/HTR1B/FPR2/GRIK1/MC4R/GABRB3/PTGER2/GHRHR/GLP1R/LPAR4/TACR2/CCKAR/P2RX7/GRIA4/PTGDR/MTNR1A/PTAFR/GRIA3/DRD5/CHRM4/CHRNA7/HTR2A/CHRNB4/ADRA1A/GRIN2A/RXFP1/GRIK5/CHRNB2/GLRB/CHRNA9/GZMA/TRHR/TAAR1/LPAR3/ADCYAP1R1/CHRNA3/MCHR1/CNR2/OPRM1/TACR1/GRIK2/GABRA4/OPRD1/HTR2B/PRSS1/GRID2/NTSR2/PLG/GABRP/PRSS2/GABRG2/GABRG3/PRLHR/ADRB1/AGTR1/ADRB3/NTSR1/CCKBR/LEP/NMUR2/CHRM2/HTR2C
KEGG_HUNTINGTONS_DISEASE	KEGG_HUNTINGTONS_DISEASE	KEGG_HUNTINGTONS_DISEASE	172	0.411521989880024	1.95350913028577	5.44098063054784e-08	1.43797345235907e-06	1.03910457154823e-06	4083	tags=41%, list=22%, signal=32%	POLR2I/COX8A/CYC1/POLR2L/SLC25A31/UQCR11/NDUFB7/NDUFS7/COX4I2/NDUFS8/POLR2G/NDUFB8/NDUFA9/GPX1/COX6A1/COX7A1/SLC25A6/NDUFB10/SLC25A5/SDHA/COX5B/ATP5F1D/NDUFA2/POLR2F/NDUFA3/COX7B/NDUFS3/NDUFV1/COX4I1/ATP5MC1/COX5A/NDUFB2/ATP5F1E/UQCRC1/UQCRQ/AP2S1/UQCRFS1/POLR2J/NDUFS6/COX7A2/POLR2E/NDUFAB1/ATP5MC3/UQCR10/POLR2H/COX6B1/NDUFB1/NDUFA8/ATP5PO/NDUFB9/BAX/NDUFB4/NDUFA10/ATP5F1B/NDUFA1/CLTA/UQCRHL/AP2A2/POLR2C/TAF4/NDUFC2/PPARG/CREB3/NDUFA6/DCTN2/SDHB/PPARGC1A/AP2A1/ATP5MC2/AP2M1
KEGG_LEISHMANIA_INFECTION	KEGG_LEISHMANIA_INFECTION	KEGG_LEISHMANIA_INFECTION	69	-0.651370898720265	-2.04743824301977	1.77224497944971e-07	3.65373515586504e-06	2.64025243126521e-06	3303	tags=54%, list=17%, signal=44%	TLR2/IL10/ITGB1/IL1B/ITGA4/NCF1/FCGR3B/PRKCB/HLA-DPB1/HLA-DMB/HLA-DQB1/HLA-DMA/HLA-DRB1/HLA-DPA1/TGFB3/HLA-DOB/HLA-DQA2/ITGB2/IL12B/ITGAM/FCGR2A/C3/JAK2/STAT1/TGFB2/HLA-DRA/IL1A/NCF2/CR1/HLA-DOA/FCGR1A/HLA-DRB5/HLA-DQA1/FCGR3A/NOS2/PTGS2/IFNG

	ID	Description	setSize	enrichmentScore	NES	pvalue	p.adjust	qvalue	rank	leading_edge	core_enrichment
KEGG_CHEMOKINE_SIGNALING_PATHWAY	KEGG_CHEMOKINE_SIGNALING_PATHWAY	KEGG_CHEMOKINE_SIGNALING_PATHWAY	186	-0.52607108070753	-1.80918855551477	1.9010275552104e-07	3.65373515586504e-06	2.64025243126521e-06	4079	tags=39%, list=22%, signal=31%	CXCL2/PIK3CA/GNG7/GNAI1/CCL16/RAC2/ELMO1/RAP1A/CXCL16/CCL28/CCL18/HCK/FGR/LYN/WAS/GNG8/GNGT2/CXCL3/PIK3R5/CCL21/CXCR1/NCF1/TIAM2/ROCK1/PIK3CG/CCR4/TIAM1/CXCR2/PRKCB/ADCY2/CCL22/CCL23/XCL2/GNB4/CCL4L2/GNG2/DOCK2/CCL2/CX3CL1/CCL17/CCR2/CCR1/CXCL6/AKT3/SHC4/XCR1/CXCL1/JAK2/CXCR6/STAT1/CCL4/CXCR4/CCR7/CCL13/CCR5/CCR8/XCL1/CCL19/ITK/CXCL9/CCL26/CCL5/CCL8/CXCR5/CXCL8/CCL7/ADCY8/CXCL10/CXCL13/CXCL11/CXCL5/PPBP/CCL25
KEGG_HEMATOPOIETIC_CELL_LINEAGE	KEGG_HEMATOPOIETIC_CELL_LINEAGE	KEGG_HEMATOPOIETIC_CELL_LINEAGE	84	-0.624892383861581	-2.01158664651285	1.97499197614327e-07	3.65373515586504e-06	2.64025243126521e-06	3902	tags=58%, list=21%, signal=47%	CD37/IL5RA/CD1C/CD3E/ITGB3/CSF1R/CD4/ITGA2/GP1BA/CSF1/CD3D/IL1R2/DNTT/ITGA5/IL1B/CD38/IL6/ITGA4/ITGA1/KITLG/IL1R1/THPO/TPO/HLA-DRB1/IL7/CD3G/CD1E/CSF2/CD2/CSF3R/CD55/ITGAM/IL9R/CD19/CSF3/HLA-DRA/IL1A/IL2RA/FCER2/CR1/FCGR1A/HLA-DRB5/ANPEP/CD8A/IL7R/CD1B/CD22/MS4A1/CR2
KEGG_PYRUVATE_METABOLISM	KEGG_PYRUVATE_METABOLISM	KEGG_PYRUVATE_METABOLISM	40	0.66316517931247	2.43675233650277	2.27625647766493e-07	3.8282495306183e-06	2.76635954701863e-06	4291	tags=57%, list=23%, signal=45%	PKLR/ME3/PC/HAGHL/ACOT12/ACSS2/PDHA2/ACAT2/ACACA/PCK1/HAGH/PDHA1/MDH2/LDHD/PCK2/GRHPR/ACSS1/LDHB/LDHAL6A/ALDH2/ACAT1/LDHA/ALDH1B1
KEGG_GRAFT_VERSUS_HOST_DISEASE	KEGG_GRAFT_VERSUS_HOST_DISEASE	KEGG_GRAFT_VERSUS_HOST_DISEASE	37	-0.742008925255434	-2.13183057074956	2.95662492879171e-07	4.55813009855388e-06	3.29378391189954e-06	3030	tags=76%, list=16%, signal=64%	HLA-F/FAS/IL1B/IL6/CD80/HLA-DPB1/HLA-DMB/HLA-DQB1/HLA-DMA/HLA-DRB1/HLA-DPA1/HLA-DOB/HLA-DQA2/HLA-DRA/IL1A/FASLG/HLA-DOA/HLA-DRB5/CD86/HLA-DQA1/PRF1/KIR2DL3/KLRD1/KLRC1/KIR2DL1/IFNG/KIR3DL2/KIR3DL1
KEGG_ECM_RECEPTOR_INTERACTION	KEGG_ECM_RECEPTOR_INTERACTION	KEGG_ECM_RECEPTOR_INTERACTION	83	-0.616920645053948	-1.98050779673094	3.44656949105749e-07	4.90473350650489e-06	3.54424554950851e-06	4658	tags=61%, list=25%, signal=47%	LAMA4/COL6A1/ITGA2B/HMMR/COL4A1/VWF/LAMC1/COL1A1/ITGB6/SDC2/ITGA11/HSPG2/ITGB3/SV2A/LAMB3/ITGA2/GP1BA/COL5A1/ITGB7/ITGB1/ITGA5/VTN/COL4A4/COL11A2/ITGA4/COL1A2/ITGA1/TNN/TNXB/LAMC2/COL3A1/LAMA3/ITGAV/ITGA10/ITGB8/LAMA2/COL6A3/SPP1/IBSP/COL5A2/THBS2/TNC/COL6A6/COL2A1/TNR/SV2B/FN1/RELN/COMP/COL11A1/THBS4

	ID	Description	setSize	enrichmentScore	NES	pvalue	p.adjust	qvalue	rank	leading_edge	core_enrichment
KEGG_ANTIGEN_PROCESSING_AND_PRESENTATION	KEGG_ANTIGEN_PROCESSING_AND_PRESENTATION	KEGG_ANTIGEN_PROCESSING_AND_PRESENTATION	80	-0.616807769060345	-1.97839059966924	8.608402182084 25e-07	1.137538859775 42e-05	8.220053211463 91e-06	3898	tags=48%, list=21%, signal=38%	CANX/HSPA1B/TAP2/B2M/IFNA1/CD4/LTA/HLA-F/TAP1/CTSL/HLA-DPB1/HLA-DMB/HLA-DQB1/HLA-DMA/HLA-DRB1/HLA-DPA1/HSPA2/HLA-DOB/HLA-DQA2/CD74/HSPA6/KLRC3/HLA-DRA/CIITA/HLA-DOA/HLA-DRB5/KLRC4/CD8A/HLA-DQA1/KIR2DL3/KIR3DL3/KLRD1/KLRC1/KIR2DL1/KIR2DL4/KIR3DL2/KLRC2/KIR3DL1
KEGG_TYPE_I_DIABETES_MELLITUS	KEGG_TYPE_I_DIABETES_MELLITUS	KEGG_TYPE_I_DIABETES_MELLITUS	41	-0.691740047717705	-2.0314735868126	5.915422263016 65e-06	7.201346543901 29e-05	5.203817986204 63e-05	3234	tags=63%, list=17%, signal=53%	LTA/HLA-F/FAS/IL1B/CD80/PTPRN/HLA-DPB1/HLA-DMB/HLA-DQB1/HLA-DMA/HLA-DRB1/HLA-DPA1/HLA-DOB/HLA-DQA2/IL12B/HLA-DRA/IL1A/FASLG/HLA-DOA/HLA-DRB5/CD86/HLA-DQA1/PRF1/GAD2/IFNG/GAD1
KEGG_ALZHEIMERS_DISEASE	KEGG_ALZHEIMERS_DISEASE	KEGG_ALZHEIMERS_DISEASE	157	0.379558674548167	1.79911407306333	6.228191605536 25e-06	7.201346543901 29e-05	5.203817986204 63e-05	3153	tags=34%, list=17%, signal=29%	CHP2/COX8A/CYC1/UQCR11/NDUFB7/BID/NDUFS7/COX4I2/NDUFS8/NDUFB8/NDUFA9/BAD/COX6A1/COX7A1/HSD17B10/FADD/NDUFB10/SDHA/COX5B/ATP5F1D/NDUFA2/NDUFA3/COX7B/NDUFS3/NDUFV1/COX4I1/ATP5MC1/COX5A/NDUFB2/ATP5F1E/UQCRC1/UQCRQ/LPL/UQCRFS1/NDUFS6/COX7A2/CAPN1/NDUFAB1/CALML6/ATP5MC3/UQCR10/COX6B1/NDUFB1/NDUFA8/ATP5PO/NDUFB9/NDUFB4/GRIN2C/NDUFA10/ATP5F1B/NDUFA1/GAPDH/MAPK3/UQCRHL
KEGG_INTESTINAL_IMMUNE_NETWORK_FOR_IGA_PRODUCTION	KEGG_INTESTINAL_IMMUNE_NETWORK_FOR_IGA_PRODUCTION	KEGG_INTESTINAL_IMMUNE_NETWORK_FOR_IGA_PRODUCTION	45	-0.679607506546698	-2.01812511497404	7.599960291860 4e-06	7.949527697180 38e-05	5.744466671646 7e-05	4182	tags=67%, list=22%, signal=52%	IL4/MADCAM1/CD28/CCL28/IL10/TNFRSF13B/ITGB7/IL15/IL6/ITGA4/CD80/ICOSLG/HLA-DPB1/HLA-DMB/HLA-DQB1/HLA-DMA/HLA-DRB1/HLA-DPA1/HLA-DOB/HLA-DQA2/ICOS/HLA-DRA/CXCR4/HLA-DOA/HLA-DRB5/CD86/HLA-DQA1/TNFSF13B/AICDA/CCL25
KEGG_CALCIUM_SIGNALING_PATHWAY	KEGG_CALCIUM_SIGNALING_PATHWAY	KEGG_CALCIUM_SIGNALING_PATHWAY	177	-0.501414045391432	-1.71812184577414	7.734675597256 58e-06	7.949527697180 38e-05	5.744466671646 7e-05	2953	tags=31%, list=16%, signal=26%	SLC8A3/F2R/PDE1A/CYSLTR1/CHRM1/CD38/GRM1/GRIN1/CALML3/HRH2/MYLK3/CACNA1I/PLCG2/PRKCB/ADCY2/PLCD4/CYSLTR2/ATP2B2/SLC8A1/ITPR1/PTGFR/AVPR1A/PRKCG/ATP2B4/CACNA1C/TACR2/CCKAR/P2RX7/CACNA1S/PTAFR/DRD5/MYLK/CHRNA7/HTR2A/ADRA1A/GRIN2A/TRHR/CAMK2B/TACR1/ATP2B3/PLN/HTR2B/NOS2/ADCY8/ERBB4/CACNA1E/ADRB1/AGTR1/ADRB3/NOS1/NTSR1/CCKBR/CHRM2/HTR2C

	ID	Description	setSize	enrichmentScore	NES	pvalue	p.adjust	qvalue	rank	leading_edge	core_enrichment
KEGG_CITRATE_CYCLE_TCA_CYCLE	KEGG_CITRATE_CYCLE_TCA_CYCLE	KEGG_CITRATE_CYCLE_TCA_CYCLE	30	0.63066722787235	2.20424096830515	5.29968113723388e-05	0.000516021584414878	0.000372886152038063	5852	tags=77%, list=31%, signal=53%	OGDHL/PC/PDHA2/SDHA/IDH3G/PCK1/PDHA1/MDH2/PCK2/IDH2/SUCLG1/IDH3B/SDHB/DLST/CS/IDH3A/FH/IDH1/PDHB/OGDH/ACO2/ACLY/ACO1
KEGG_FOCAL_ADHESION	KEGG_FOCAL_ADHESION	KEGG_FOCAL_ADHESION	198	-0.470004519826405	-1.62278273196157	6.63199350403668e-05	0.000613459399123393	0.000443296407901399	4201	tags=38%, list=22%, signal=30%	COL4A1/PIK3CD/PIK3CA/VWF/LAMC1/COL1A1/ITGB6/RAC2/ITGA11/BIRC2/PDGFRA/RAP1A/ITGB3/VEGFC/LAMB3/ITGA2/ACTN3/PRKCA/FLNA/COL5A1/PPP1R12A/ITGB7/ITGB1/CAPN2/ITGA5/VTN/COL4A4/PIK3R5/COL11A2/ITGA4/ROCK1/MYLK3/COL1A2/ITGA1/PIK3CG/TNN/PRKCB/TNXB/LAMC2/FYN/COL3A1/LAMA3/ITGAV/ARHGAP5/ITGA10/ITGB8/LAMA2/COL6A3/HGF/SPP1/PRKCG/CAV1/IBSP/PDGFC/AKT3/SHC4/COL5A2/THBS2/TNC/COL6A6/MYLK/COL2A1/TNR/PAK3/FN1/CAV2/RELN/COMP/BIRC3/IGF1/FLNC/COL11A1/PAK5/CAV3/THBS4
KEGG_VIRAL_MYOCARDITIS	KEGG_VIRAL_MYOCARDITIS	KEGG_VIRAL_MYOCARDITIS	68	-0.589683423327888	-1.85055323124665	8.57208914858923e-05	0.000755160234518575	0.000545691890662071	3857	tags=53%, list=20%, signal=42%	SGCB/MYH7/CD28/RAC2/MYH3/HLA-F/ITGAL/MYH13/MYH8/ICAM1/SGCA/CD80/HLA-DPB1/SGCD/HLA-DMB/HLA-DQB1/FYN/HLA-DMA/HLA-DRB1/HLA-DPA1/MYH10/LAMA2/HLA-DOB/HLA-DQA2/ITGB2/CAV1/CD55/HLA-DRA/HLA-DOA/HLA-DRB5/CD86/HLA-DQA1/MYH1/PRF1/MYH11/MYH2
KEGG_GLYCOLYSIS_GLUCONEOGENESIS	KEGG_GLYCOLYSIS_GLUCONEOGENESIS	KEGG_GLYCOLYSIS_GLUCONEOGENESIS	60	0.481883027536922	1.87268368376837	0.000126206556423925	0.0010612824062921	0.000766901084489879	3977	tags=52%, list=21%, signal=41%	ALDOC/ALDOA/PKLR/ACSS2/ADH4/PDHA2/GCK/ADH1C/PCK1/ADH1A/PDHA1/FBP1/PGK2/PCK2/TPI1/ADH6/PFKL/ACSS1/PGK1/GPI/ENO3/ALDH3A1/LDHB/GAPDH/LDHAL6A/AKR1A1/ALDH2/PFKM/PGM1/PGAM4/LDHA
KEGG_COMPLEMENT_AND_COAGULATION_CASCADES	KEGG_COMPLEMENT_AND_COAGULATION_CASCADES	KEGG_COMPLEMENT_AND_COAGULATION_CASCADES	69	-0.565115724035913	-1.77631445831576	0.000190641308021464	0.00153341921669438	0.00110807533724146	4125	tags=48%, list=22%, signal=38%	SERPINA1/FGB/C8G/VWF/CFI/C1R/C5AR1/F2R/C1QA/C9/SERPINA5/KLKB1/PROS1/C1S/SERPING1/C3AR1/C4A/C8B/F13A1/CFH/CD55/TFPI/F3/C3/C4B/CR1/MASP1/C6/PLG/F5/C7/CR2/FGG
KEGG_RNA_POLYMERASE	KEGG_RNA_POLYMERASE	KEGG_RNA_POLYMERASE	29	0.583702592625751	2.02756647957553	0.000544892851669559	0.0041811564471141	0.0030213762092944	3436	tags=45%, list=18%, signal=37%	POLR2I/POLR2L/POLR2G/POLR3K/POLR1H/POLR2F/POLR1C/POLR2J/POLR2E/POLR2H/POLR1D/POLR2C/POLR3H

	ID	Description	setSize	enrichmentScore	NES	pvalue	p.adjust	qvalue	rank	leading_edge	core_enrichment
KEGG_NATURAL_KILLER_CELL_MEDIATED_CYTOTOXICITY	KEGG_NATURAL_KILLER_CELL_MEDIATED_CYTOTOXICITY	KEGG_NATURAL_KILLER_CELL_MEDIATED_CYTOTOXICITY	131	-0.494886123177444	-1.65149195190476	0.000565021141501905	0.0041811564471141	0.0030213762092944	4245	tags=40%, list=22%, signal=31%	TNFSF10/PIK3CD/HLA-E/PPP3R2/PIK3CA/CD247/RAC2/KLRK1/IFNGR1/IFNA1/PPP3CA/PRKCA/FAS/ITGAL/PIK3R5/NFATC1/FCGR3B/ICAM1/FCER1G/PIK3CG/NCR2/PLCG2/NCR3/PRKCB/FYN/LCP2/SH2D1B/ULBP1/MICB/PRKCG/CSF2/ITGB2/ULBP2/SHC4/CD244/LCK/KLRC3/SH2D1A/FASLG/PRF1/NCR1/KIR2DL3/FCGR3A/RAET1E/KLRD1/KLRC1/KIR2DL1/KIR2DL4/IFNG/KIR3DL2/KLRC2/KIR3DL1
KEGG_ARRHYTHMOGENIC_RIGHT_VENTRICULAR_CARDIOMYOPATHY_ARVC	KEGG_ARRHYTHMOGENIC_RIGHT_VENTRICULAR_CARDIOMYOPATHY_ARVC	KEGG_ARRHYTHMOGENIC_RIGHT_VENTRICULAR_CARDIOMYOPATHY_ARVC	74	-0.546795808458479	-1.73986881525164	0.000622523339575352	0.00442949299313231	0.00320082850712833	3992	tags=46%, list=21%, signal=36%	TCF7L2/ITGB6/SGCB/CACNA2D3/ITGA11/ITGB3/DSC2/ITGA2/ACTN3/ITGB7/ITGB1/ITGA5/CACNA2D2/GJA1/ITGA4/CACNG1/SGCA/ITGA1/SGCD/ITGAV/ITGA10/SLC8A1/ITGB8/LAMA2/CACNB4/CACNA1C/CDH2/CACNA1S/CACNG5/CACNA2D1/CACNG6/CTNNA3/DES/CACNG7
KEGG_ASTHMA	KEGG_ASTHMA	KEGG_ASTHMA	28	-0.677217263716619	-1.83137943684755	0.000748047032402322	0.00512550744423813	0.00370378062826881	4182	tags=68%, list=22%, signal=53%	IL4/IL9/MS4A2/IL10/FCER1A/FCER1G/HLA-DPB1/HLA-DMB/HLA-DQB1/HLA-DMA/HLA-DRB1/HLA-DPA1/HLA-DOB/HLA-DQA2/PRG2/HLA-DRA/HLA-DOA/HLA-DRB5/HLA-DQA1
KEGG_STEROID_BIOSYNTHESIS	KEGG_STEROID_BIOSYNTHESIS	KEGG_STEROID_BIOSYNTHESIS	17	0.673240922344135	2.0128556596513	0.000790649245743335	0.00522393251651846	0.0037749042935866	1777	tags=47%, list=9%, signal=43%	TM7SF2/CYP51A1/DHCR7/CEL/EBP/LSS/NSDHL/DHCR24
KEGG_ALLOGRAFT_REJECTION	KEGG_ALLOGRAFT_REJECTION	KEGG_ALLOGRAFT_REJECTION	35	-0.636049527103064	-1.81445484706781	0.00101288061455498	0.0064614797825059	0.0046691774246273	3179	tags=69%, list=17%, signal=57%	IL4/HLA-E/CD28/IL10/HLA-F/FAS/CD80/HLA-DPB1/HLA-DMB/HLA-DQB1/HLA-DMA/HLA-DRB1/HLA-DPA1/HLA-DOB/HLA-DQA2/IL12B/HLA-DRA/FASLG/HLA-DOA/HLA-DRB5/CD86/HLA-DQA1/PRF1/IFNG
KEGG_GLUTATHIONE_METABOLISM	KEGG_GLUTATHIONE_METABOLISM	KEGG_GLUTATHIONE_METABOLISM	48	0.470788156480829	1.8311601976017	0.00127172041895445	0.00784227591688577	0.00566696467393737	3469	tags=40%, list=18%, signal=32%	GPX7/GSS/SRM/GPX4/GPX1/GSTP1/GSTM3/GGT7/TXNDC12/G6PD/GSTA2/GSTM1/GSTZ1/OPLAH/PGD/GPX2/IDH2/ODC1/MGST3
KEGG_REGULATION_OF_ACTIN_CYTOSKELETON	KEGG_REGULATION_OF_ACTIN_CYTOSKELETON	KEGG_REGULATION_OF_ACTIN_CYTOSKELETON	211	-0.425253977341017	-1.48301648642339	0.00139480600528444	0.00832384228960067	0.00601495289205852	4461	tags=35%, list=24%, signal=27%	KRAS/IQGAP1/ACTN2/ABI2/PIKFYVE/ITGA2B/PFN3/PIK3CD/PIP4K2A/FGFR1/APC/PIK3CA/ENAH/MRAS/WASF1/ITGB6/GNA13/FGF11/RAC2/ITGA11/PDGFRA/ITGB3/ITGA2/ACTN3/FGF5/PPP1R12A/ITGB7/ITGB1/F2R/WAS/ITGA5/ITGAL/MSN/ITGAX/CHRM1/PIK3R5/ARHGEF6/ITGA4/NCKAP1L/TIAM2/ROCK1/MYLK3/ITGA1/PIK3CG/TIAM1/RDX/ITGAV/ITGA10/FGF9/FGF1/ITGB8/MYH10/FGF13/ITGB2/CFL2/PDGFC/ITGAD/ITGAM/FGF10/FGF19/FGF7/MYLK/ARHGEF4/CHRM4/PAK3/FN1/INSRR/FGF23/FGF2/FGF14/FGF20/PAK5/CHRM2

	ID	Description	setSize	enrichmentScore	NES	pvalue	p.adjust	qvalue	rank	leading_edge	core_enrichment
KEGG_VASCULAR_SMOOTH_MUSCLE_CONTRACTION	KEGG_VASCULAR_SMOOTH_MUSCLE_CONTRACTION	KEGG_VASCULAR_SMOOTH_MUSCLE_CONTRACTION	115	-0.468369057193766	-1.54264400356358	0.00231471412010249	0.0133454955509339	0.00964368668545438	3739	tags=34%, list=20%, signal=27%	PRKCH/GNA13/CALCRL/IRAG1/PRKCA/PRKG1/PPP1R12A/GUCY1B1/PRKCQ/CALML3/ROCK1/MYLK3/KCNMB2/PLA2G5/PLA2G2D/PRKCB/ADCY2/PLA2G2C/PLA2G2E/ITPR1/AVPR1A/PRKCG/ACTG2/PLA2G2A/CACNA1C/PPP1R12B/PLA2G3/KCNMB1/KCNMA1/CALD1/GUCY1A1/CACNA1S/MYLK/ADRA1A/GUCY1A2/RAMP1/ADCY8/MYH11/AGTR1
KEGG_LONG_TERM_DEPRESSION	KEGG_LONG_TERM_DEPRESSION	KEGG_LONG_TERM_DEPRESSION	70	-0.518243373022141	-1.63438136043197	0.00238054785503145	0.0133454955509339	0.00964368668545438	4573	tags=46%, list=24%, signal=35%	PLA2G2F/GNAQ/KRAS/PLA2G4A/GRM5/PRKG2/GNAI1/GNA13/GRIA1/RYR1/PRKCA/PRKG1/PPP1R17/LYN/GUCY1B1/GRM1/PLA2G5/PLA2G2D/PRKCB/PLA2G2C/PLA2G2E/GNAO1/ITPR1/PRKCG/PLA2G2A/PLA2G3/GUCY1A1/GRIA3/GUCY1A2/IGF1/GRID2/NOS1
KEGG_PYRIMIDINE_METABOLISM	KEGG_PYRIMIDINE_METABOLISM	KEGG_PYRIMIDINE_METABOLISM	97	0.334428591193246	1.45842986788788	0.0033646652137567	0.0183077371924997	0.0132294886113654	3207	tags=30%, list=17%, signal=25%	NME2/ENTPD8/POLR2I/POLR2L/NME3/POLR2G/NME1/POLR3K/POLE4/UCKL1/POLR1H/POLR2F/NME4/POLR1C/POLR2J/ENTPD6/DTYMK/POLR2E/POLD4/POLD2/NT5C/PNP/POLR2H/UCK1/POLD1/ENTPD5/TXNRD2/POLR1D/POLR2C
KEGG_TERPENOID_BACKBONE_BIOSYNTHESIS	KEGG_TERPENOID_BACKBONE_BIOSYNTHESIS	KEGG_TERPENOID_BACKBONE_BIOSYNTHESIS	15	0.65906562341608	1.88466507873118	0.00360245548654267	0.0190415504288684	0.0137597547907043	1281	tags=33%, list=7%, signal=31%	HMGCS2/MVD/MVK/FDPS/ACAT2
KEGG_LEUKOCYTE_TRANSENDOTHELIAL_MIGRATION	KEGG_LEUKOCYTE_TRANSENDOTHELIAL_MIGRATION	KEGG_LEUKOCYTE_TRANSENDOTHELIAL_MIGRATION	114	-0.465249165228782	-1.52964400211897	0.00415365952664928	0.0213451947897255	0.0154244081837561	4188	tags=36%, list=22%, signal=28%	PIK3CD/CLDN1/PIK3CA/GNAI1/RAC2/CLDN19/RAP1A/MMP2/RHOH/NOX3/ACTN3/PRKCA/ITGB1/ITGAL/JAM3/MSN/PIK3R5/ITGA4/NCF1/ICAM1/JAM2/ROCK1/PIK3CG/TXK/PLCG2/PRKCB/ARHGAP5/VCAM1/PRKCG/RASSF5/ITGB2/ITGAM/CYBB/CLDN8/CXCR4/NCF2/CLDN16/CLDN11/ITK/CTNNA3/CLDN18
KEGG_PANTOTHENATE_AND_COA_BIOSYNTHESIS	KEGG_PANTOTHENATE_AND_COA_BIOSYNTHESIS	KEGG_PANTOTHENATE_AND_COA_BIOSYNTHESIS	16	-0.704016468150244	-1.7292501458041	0.00532430046106025	0.0266215023053012	0.0192371595605875	1024	tags=31%, list=5%, signal=30%	BCAT1/DPYD/DPYS/VNN2/VNN1
KEGG_ABC_TRANSPORTERS	KEGG_ABC_TRANSPORTERS	KEGG_ABC_TRANSPORTERS	44	-0.568079010304268	-1.68868201696033	0.0054785988424905	0.0266721259437037	0.0192737410802297	3783	tags=36%, list=20%, signal=29%	ABCB4/TAP2/ABCG8/TAP1/ABCA3/ABCB11/ABCA1/ABCG2/ABCC9/ABCA6/ABCC8/ABCA4/ABCD2/ABCA9/ABCB5/ABCA12
KEGG_PORPHYRIN_AND_CHLOROPHYLL_METABOLISM	KEGG_PORPHYRIN_AND_CHLOROPHYLL_METABOLISM	KEGG_PORPHYRIN_AND_CHLOROPHYLL_METABOLISM	41	0.44680787383399	1.67798907674098	0.00638595807996883	0.0302923652511342	0.0218897888301226	2020	tags=34%, list=11%, signal=31%	UGT2B10/ALAS2/FTH1/UGT1A6/UGT2A1/UGT2A3/HMBS/MMAB/UGT2B11/UGT1A10/UGT1A8/HMOX2/UROD/FTMT

	ID	Description	setSize	enrichmentScore	NES	pvalue	p.adjust	qvalue	rank	leading_edge	core_enrichment
KEGG_JAK_STAT_SIGNALING_PATHWAY	KEGG_JAK_STAT_SIGNALING_PATHWAY	KEGG_JAK_STAT_SIGNALING_PATHWAY	155	-0.418086957072948	-1.41816175785409	0.00839880560346025	0.0388444759160037	0.0280696924115645	4971	tags=41%, list=26%, signal=31%	SOS1/EPOR/STAM2/IL20/IL2RG/PRLR/SOCS4/STAT2/SOCS3/SPRY1/EP300/SPRY2/PIK3CD/IL4/LIF/GHR/PIK3CA/IL5RA/IL9/IFNGR1/IFNA1/IL23A/IL10/IL19/CRLF2/IFNK/IL15/CTF1/PIK3R5/IL22/IL6/IL6ST/IL12RB2/OSMR/LEPR/IL12RB1/SPRED1/PIK3CG/LIFR/STAT4/IL10RA/TPO/CSF2RB/IL7/IL22RA2/PRL/CSF2/IL26/CSF3R/IL12B/IL2RB/AKT3/IL9R/CNTFR/JAK2/STAT1/IL21R/CSF3/IL2RA/IL7R/IL21/IFNG/LEP/IFNE
KEGG_FRUCTOSE_AND_MANNOSE_METABOLISM	KEGG_FRUCTOSE_AND_MANNOSE_METABOLISM	KEGG_FRUCTOSE_AND_MANNOSE_METABOLISM	34	0.482050818780086	1.7253166207951	0.00896560302353619	0.0401633663372	0.0290227455182043	5144	tags=50%, list=27%, signal=36%	ALDOC/ALDOA/PHPT1/KHK/AKR1B10/FBP1/PFKFB1/TPI1/PFKL/GMPPB/PMM1/MPI/PFKM/FCSK/GFUS/SORD/GMDS
KEGG_AUTOIMMUNE_THYROID_DISEASE	KEGG_AUTOIMMUNE_THYROID_DISEASE	KEGG_AUTOIMMUNE_THYROID_DISEASE	50	-0.527280198470896	-1.5851490584005	0.00911816965493189	0.0401633663372	0.0290227455182043	4182	tags=54%, list=22%, signal=42%	IL4/HLA-E/CD28/IFNA1/CGA/IL10/HLA-F/FAS/TSHR/CD80/HLA-DPB1/HLA-DMB/HLA-DQB1/TPO/HLA-DMA/HLA-DRB1/HLA-DPA1/HLA-DOB/HLA-DQA2/CTLA4/HLA-DRA/FASLG/HLA-DOA/HLA-DRB5/CD86/HLA-DQA1/PRF1
KEGG_TAURINE_AND_HYPOTAURINE_METABOLISM	KEGG_TAURINE_AND_HYPOTAURINE_METABOLISM	KEGG_TAURINE_AND_HYPOTAURINE_METABOLISM	10	-0.750292114560897	-1.62123223105101	0.00974087717161361	0.0419084250406632	0.0302837552214181	1087	tags=40%, list=6%, signal=38%	BAAT/GAD2/CDO1/GAD1
KEGG_DILATED_CARDIOMYOPATHY	KEGG_DILATED_CARDIOMYOPATHY	KEGG_DILATED_CARDIOMYOPATHY	90	-0.475318918433055	-1.54179307266964	0.0101555167869112	0.0426993319449676	0.0308552782760221	3893	tags=43%, list=21%, signal=35%	TTN/ITGB6/SGCB/MYH7/CACNA2D3/ITGA11/ITGB3/ITGA2/ITGB7/ITGB1/ITGA5/TPM2/CACNA2D2/ITGA4/CACNG1/SGCA/ITGA1/ADCY2/SGCD/ITGAV/ITGA10/SLC8A1/ITGB8/LAMA2/CACNB4/TGFB3/CACNA1C/TNNT2/TGFB2/CACNA1S/CACNG5/CACNA2D1/IGF1/CACNG6/PLN/DES/CACNG7/ADCY8/ADRB1
KEGG_SYSTEMIC_LUPUS_ERYTHEMATOSUS	KEGG_SYSTEMIC_LUPUS_ERYTHEMATOSUS	KEGG_SYSTEMIC_LUPUS_ERYTHEMATOSUS	131	-0.441926547407716	-1.47475975218487	0.0105030201836965	0.0431790829774188	0.031201954697765	3515	tags=38%, list=19%, signal=31%	H4C8/H3-4/H2BC14/H3C3/H2AC19/ACTN3/H4C12/IL10/H2BC18/C1QA/C9/FCGR3B/H3C11/RO60/H2BC21/H4C11/C1S/CD80/HLA-DPB1/H3C1/HLA-DMB/HLA-DQB1/CTSG/C4A/C8B/FCGR2B/HLA-DMA/HLA-DRB1/HLA-DPA1/HLA-DOB/HLA-DQA2/H2BW1/FCGR2A/C3/C4B/HLA-DRA/HLA-DOA/FCGR1A/HLA-DRB5/CD86/GRIN2A/HLA-DQA1/H2AC18/C6/FCGR3A/IFNG/H3C14/C7/H4C6/H2BC3
KEGG_TYPE_II_DIABETES_MELLITUS	KEGG_TYPE_II_DIABETES_MELLITUS	KEGG_TYPE_II_DIABETES_MELLITUS	47	-0.515317045587296	-1.53956512771932	0.0110854195288453	0.0445826654964429	0.0322162077840583	2834	tags=21%, list=15%, signal=18%	IRS1/PIK3R5/HK3/PIK3CG/MAFA/CACNA1C/ABCC8/SLC2A2/CACNA1E/ADIPOQ
KEGG_PPAR_SIGNALING_PATHWAY	KEGG_PPAR_SIGNALING_PATHWAY	KEGG_PPAR_SIGNALING_PATHWAY	69	-0.478294368617288	-1.50341101153275	0.01359290447333	0.0535039856928949	0.0386629085803565	1873	tags=20%, list=10%, signal=18%	SORBS1/CYP8B1/FABP7/ACSL6/APOC3/MMP1/UCP1/OLR1/APOA2/PLIN1/APOA1/CYP7A1/FABP4/ADIPOQ
KEGG_PENTOSE_AND_GLUCURONATE_INTERCONVERSIONS	KEGG_PENTOSE_AND_GLUCURONATE_INTERCONVERSIONS	KEGG_PENTOSE_AND_GLUCURONATE_INTERCONVERSIONS	28	0.487046291095841	1.68859676495416	0.0153005200216729	0.0579543863210541	0.0418788453073904	1811	tags=36%, list=10%, signal=32%	UGT2B10/RPEL1/UGT1A6/UGT2A1/DCXR/UGT2A3/UGT2B11/UGT1A10/UGT1A8/DHDH

	ID	Description	setSize	enrichmentScore	NES	pvalue	p.adjust	qvalue	rank	leading_edge	core_enrichment
KEGG_BASE_EXCISION_REPAIR	KEGG_BASE_EXCISION_REPAIR	KEGG_BASE_EXCISION_REPAIR	33	0.441079612960862	1.59373654682639	0.0153500807012522	0.0579543863210541	0.0418788453073904	3352	tags=30%, list=18%, signal=25%	MPG/POLE4/NTHL1/POLD4/POLD2/OGG1/APEX2/POLD1/APEX1/SMUG1
KEGG_HYPERTROPHIC_CARDIOMYOPATHY_HCM	KEGG_HYPERTROPHIC_CARDIOMYOPATHY_HCM	KEGG_HYPERTROPHIC_CARDIOMYOPATHY_HCM	83	-0.450598450380012	-1.44656164666763	0.0161850754498021	0.0598847791642677	0.0432737806763129	3893	tags=45%, list=21%, signal=36%	TTN/ITGB6/SGCB/MYH7/CACNA2D3/ITGA11/ITGB3/ITGA2/ITGB7/ITGB1/PRKAA1/ITGA5/TPM2/CACNA2D2/IL6/ITGA4/CACNG1/SGCA/ITGA1/SGCD/ITGAV/ITGA10/SLC8A1/ITGB8/LAMA2/CACNB4/TGFB3/CACNA1C/TNNT2/TGFB2/CACNA1S/CACNG5/CACNA2D1/IGF1/CACNG6/DES/CACNG7
KEGG_VALINE_LEUCINE_AND_ISOLEUCINE_DEGRADATION	KEGG_VALINE_LEUCINE_AND_ISOLEUCINE_DEGRADATION	KEGG_VALINE_LEUCINE_AND_ISOLEUCINE_DEGRADATION	44	0.402677610062203	1.5336024319043	0.021297201844241	0.0772545557095016	0.0558254826464108	4291	tags=45%, list=23%, signal=35%	HMGCS2/BCKDHA/HSD17B10/ACAT2/ACAA1/OXCT2/BCAT2/ACAA2/HMGCL/ABAT/HADH/PCCA/ALDH2/ACADS/PCCB/ACAT1/MCCC1/IVD/AUH/ALDH1B1
KEGG_PROPANOATE_METABOLISM	KEGG_PROPANOATE_METABOLISM	KEGG_PROPANOATE_METABOLISM	32	0.435033779053643	1.56268662501116	0.0268431139232799	0.0954995399193612	0.0690096248634929	4291	tags=47%, list=23%, signal=36%	ACSS2/ACAT2/ACACA/ABAT/ACSS1/LDHB/MLYCD/PCCA/LDHAL6A/ALDH2/SUCLG1/PCCB/ACAT1/LDHA/ALDH1B1
KEGG_GAP_JUNCTION	KEGG_GAP_JUNCTION	KEGG_GAP_JUNCTION	89	-0.440114867334858	-1.42418244495631	0.0288568257491676	0.100726655916906	0.0727868295957157	5189	tags=39%, list=27%, signal=29%	MAP3K2/TUBA8/PDGFRB/SOS1/EGFR/ITPR2/GNAQ/KRAS/LPAR1/CDK1/GRM5/PRKG2/GNAI1/DRD2/TUBB4A/PDGFRA/PRKCA/PRKG1/GUCY1B1/GJA1/GRM1/PRKCB/ADCY2/ITPR1/TUBB2B/PRKCG/PDGFC/GJD2/GUCY1A1/HTR2A/GUCY1A2/HTR2B/ADCY8/ADRB1/HTR2C
KEGG_PURINE_METABOLISM	KEGG_PURINE_METABOLISM	KEGG_PURINE_METABOLISM	158	0.268687489971819	1.27225941449071	0.031975530225609	0.109545797995142	0.0791596947105721	2315	tags=20%, list=12%, signal=18%	NME2/ENTPD8/POLR2I/URAD/PDE6A/POLR2L/PKLR/PRPS1L1/IMPDH1/NME3/POLR2G/NME1/ENTPD2/APRT/POLR3K/POLE4/POLR1H/GUK1/POLR2F/NME4/AK1/ENPP3/POLR1C/POLR2J/ENTPD6/POLR2E/PDE6H/POLD4/POLD2/NT5C/PNP/POLR2H
KEGG_BETA_ALANINE_METABOLISM	KEGG_BETA_ALANINE_METABOLISM	KEGG_BETA_ALANINE_METABOLISM	22	-0.590634059419413	-1.53692036568607	0.0333333333333333	0.112121212121212	0.0810207336523126	1323	tags=32%, list=7%, signal=30%	AOC2/CNDP1/AOC3/DPYD/DPYS/GAD2/GAD1
KEGG_PRION_DISEASES	KEGG_PRION_DISEASES	KEGG_PRION_DISEASES	35	-0.536190695961836	-1.52958813077298	0.0366161616161616	0.120964105339105	0.087410761752867	2878	tags=37%, list=15%, signal=32%	C1QA/IL1B/C9/IL6/NCAM1/EGR1/FYN/C8B/NCAM2/IL1A/CCL5/C6/C7
KEGG_RETINOL_METABOLISM	KEGG_RETINOL_METABOLISM	KEGG_RETINOL_METABOLISM	64	0.327693740381464	1.2930799355284	0.0395626181222307	0.128404988642328	0.092787673158325	2305	tags=33%, list=12%, signal=29%	UGT2B10/CYP26C1/CYP3A43/UGT1A6/UGT2A1/UGT2A3/RDH8/CYP2B6/ADH4/UGT2B11/UGT1A10/PNPLA4/CYP26A1/DGAT2/ADH1C/UGT1A8/ADH1A/DGAT1/CYP2A7/RDH12/RDH5
KEGG_PROTEASOME	KEGG_PROTEASOME	KEGG_PROTEASOME	44	0.376069214241306	1.43226404228364	0.046960416183749	0.149787534379199	0.108239071784685	4479	tags=36%, list=24%, signal=28%	PSMD3/PSMB3/PSMA6/PSMA7/PSMB5/PSMC3/PSMD8/PSMD13/PSMC4/PSMB4/PSMD7/PSMB1/PSME3/PSMB7/POMP/PSMD2

Supplementary Table 8: Correlation value table between differential immune cells.

	CD56bright natural killer cell	Central memory CD4 T cell	Memory B cell	Natural killer T cell	Type 17 T helper cell
CD56bright natural killer cell	1	0.031	-0.08	-0.016	0.112
Central memory CD4 T cell	0.031	1	0.043	0.746	0.138
Memory B cell	-0.08	0.043	1	0.067	-0.208
Natural killer T cell	-0.016	0.746	0.067	1	0.1
Type 17 T helper cell	0.112	0.138	-0.208	0.1	1

Supplementary Table 9: P-value table between differential immune cells.

	CD56bright natural killer cell	Central memory CD4 T cell	Memory B cell	Natural killer T cell	Type 17 T helper cell
CD56bright natural killer cell	0	0.380012391846664	0.137556322139967	0.942761820250793	0.0417017602663112
Central memory CD4 T cell	0.380012391846664	0	0.59062552977248	1.11623353410016e-72	0.0205512990927736
Memory B cell	0.137556322139967	0.59062552977248	0	0.247942172068973	2.80723811343525e-05
Natural killer T cell	0.942761820250793	1.11623353410016e-72	0.247942172068973	0	0.104597610870467
Type 17 T helper cell	0.0417017602663112	0.0205512990927736	2.80723811343525e-05	0.104597610870467	0

Supplementary Table 10: Correlation value and P-value table between differential immune cells and prognostic genes.

	gene	cell	Correlation	Pvalue
1	POU4F1	CD56bright natural killer cell	-0.0220948531205217	0.673109645807229
2	ATP2A1	CD56bright natural killer cell	-0.1225657472054	0.018830256557294
3	KLC3	CD56bright natural killer cell	-0.0404039055389958	0.440290843862656
4	CALB2	CD56bright natural killer cell	-0.106436916891995	0.0415625036590083
5	POU4F1	Central memory CD4 T cell	-0.112840390263829	0.0306765263417651
6	ATP2A1	Central memory CD4 T cell	-0.129186562797146	0.0132565053483719
7	KLC3	Central memory CD4 T cell	0.0371480007439406	0.478032528025331
8	CALB2	Central memory CD4 T cell	0.461759230072867	8.80820891109959e-21
9	POU4F1	Memory B cell	0.0441588257732626	0.398956097370409
10	ATP2A1	Memory B cell	-0.172255911971406	0.000921385181877808
11	KLC3	Memory B cell	-0.129768738893668	0.0128442146573558
12	CALB2	Memory B cell	-0.0324079300316011	0.535987976301308
13	POU4F1	Natural killer T cell	-0.0632654465410897	0.226636491818941
14	ATP2A1	Natural killer T cell	-0.144909619100118	0.00541427526423698
15	KLC3	Natural killer T cell	0.0747594486976459	0.152917988897425
16	CALB2	Natural killer T cell	0.506757417818927	2.44792380653011e-25
17	POU4F1	Type 17 T helper cell	-0.157060976972653	0.00255004268530701
18	ATP2A1	Type 17 T helper cell	-0.0338692740626865	0.517752250062642
19	KLC3	Type 17 T helper cell	-0.0482576242186687	0.356598188198475
20	CALB2	Type 17 T helper cell	-0.159415339525006	0.0021901852705052
